# An imprinted non-coding genomic cluster at 14q32 defines clinically relevant molecular subtypes in osteosarcoma across multiple independent datasets

**DOI:** 10.1186/s13045-017-0465-4

**Published:** 2017-05-15

**Authors:** Katherine E. Hill, Andrew D. Kelly, Marieke L. Kuijjer, William Barry, Ahmed Rattani, Cassandra C. Garbutt, Haydn Kissick, Katherine Janeway, Antonio Perez-Atayde, Jeffrey Goldsmith, Mark C. Gebhardt, Mohamed S. Arredouani, Greg Cote, Francis Hornicek, Edwin Choy, Zhenfeng Duan, John Quackenbush, Benjamin Haibe-Kains, Dimitrios Spentzos

**Affiliations:** 1 Center for Sarcoma and Connective Tissue Oncology, Department of Orthopedics, Massachusetts General Hospital, Harvard Medical School, Boston, MA USA; 2 Hematology-Oncology, Cancer Center, Beth Israel Deaconess Medical Center, Harvard Medical School, Boston, MA USA; 3 Department of Biostatistics and Computational Biology, Dana-Farber Cancer Institute, Boston, MA USA; 4 Department of Molecular Biology, Princeton University, Princeton, NJ USA; 5 Fels Institute for Cancer Research and Molecular Biology, Lewis Katz School of Medicine, Temple University, Philadelphia, PA USA; 6 Cancer Center, Division of Hematology and Oncology, Massachusetts General Hospital, Harvard Medical School, Boston, MA USA; 7 Princess Margaret Cancer Centre, University Health Network, Toronto, Canada; 8 Department of Medical Biophysics, University of Toronto, Toronto, Canada; 9 Department of Computer Science, University of Toronto, Toronto, Canada; 10 Ontario Institute of Cancer Research, Toronto, Canada; 11 Department of Pediatric Oncology, Boston Children’s Hospital, Harvard Medical School, Boston, MA USA; 12 Department of Pathology, Boston Children’s Hospital, Harvard Medical School, Boston, MA USA; 13 Orthopedics, Cancer Center, Beth Israel Deaconess Medical Center, Harvard Medical School, Boston, MA USA; 14 Surgery, Cancer Center, Beth Israel Deaconess Medical Center, Harvard Medical School, Boston, MA USA; 15 Department of Medicine, Mount Auburn Hospital, Cambridge, MA USA; 16 Department of Urology, Medical School, Emory University, Atlanta, GA USA

**Keywords:** Osteosarcoma prognosis, Molecular subtypes, MicroRNA expression, Methylation, Loss of imprinting

## Abstract

**Background:**

A microRNA (miRNA) collection on the imprinted 14q32 *MEG3* region has been associated with outcome in osteosarcoma. We assessed the clinical utility of this miRNA set and their association with methylation status.

**Methods:**

We integrated coding and non-coding RNA data from three independent annotated clinical osteosarcoma cohorts (*n* = 65, *n* = 27, and *n* = 25) and miRNA and methylation data from one in vitro (19 cell lines) and one clinical (NCI Therapeutically Applicable Research to Generate Effective Treatments (TARGET) osteosarcoma dataset, *n* = 80) dataset. We used time-dependent receiver operating characteristic (tdROC) analysis to evaluate the clinical value of candidate miRNA profiles and machine learning approaches to compare the coding and non-coding transcriptional programs of high- and low-risk osteosarcoma tumors and high- versus low-aggressiveness cell lines. In the cell line and TARGET datasets, we also studied the methylation patterns of the *MEG3* imprinting control region on 14q32 and their association with miRNA expression and tumor aggressiveness.

**Results:**

In the tdROC analysis, miRNA sets on 14q32 showed strong discriminatory power for recurrence and survival in the three clinical datasets. High- or low-risk tumor classification was robust to using different microRNA sets or classification methods. Machine learning approaches showed that genome-wide miRNA profiles and miRNA regulatory networks were quite different between the two outcome groups and mRNA profiles categorized the samples in a manner concordant with the miRNAs, suggesting potential molecular subtypes. Further, miRNA expression patterns were reproducible in comparing high-aggressiveness versus low-aggressiveness cell lines. Methylation patterns in the *MEG3* differentially methylated region (DMR) also distinguished high-aggressiveness from low-aggressiveness cell lines and were associated with expression of several 14q32 miRNAs in both the cell lines and the large TARGET clinical dataset. Within the limits of available CpG array coverage, we observed a potential methylation-sensitive regulation of the non-coding RNA cluster by CTCF, a known enhancer-blocking factor.

**Conclusions:**

Loss of imprinting/methylation changes in the 14q32 non-coding region defines reproducible previously unrecognized osteosarcoma subtypes with distinct transcriptional programs and biologic and clinical behavior. Future studies will define the precise relationship between 14q32 imprinting, non-coding RNA expression, genomic enhancer binding, and tumor aggressiveness, with possible therapeutic implications for both early- and advanced-stage patients.

**Electronic supplementary material:**

The online version of this article (doi:10.1186/s13045-017-0465-4) contains supplementary material, which is available to authorized users.

## Background

Osteosarcoma is a bone malignancy primarily affecting adolescents and young adults, which is characterized by substantial clinical heterogeneity. Although patients with optimal neoadjuvant chemotherapy response (>90% necrosis) have good prognosis, those with lower levels of tumor necrosis have more heterogeneous outcomes [1, 2]. Stratification of patients using pathologic necrosis as the only prognostic stratification factor has not led to improved outcomes in adjuvant clinical trials [3–5]. MicroRNAs (miRNAs) have emerged as novel candidate biomarkers as well as potential modulators of tumor behavior. We recently described miRNA expression models for recurrence and overall survival from formalin-fixed-paraffin-embedded (FFPE) biopsy specimens that confer strong prognostic discrimination independent of chemotherapy response [6]. The majority of these prognostic miRNAs are located on the 14q32 locus, one of the few genomic regions that are imprinted in normal cells, which is thought to be critical in tissue development via a tightly controlled, allele-specific DNA methylation effect on gene expression. In addition, this locus contains a large cluster of non-coding elements, both miRNAs and other small nucleolar RNAs (snoRNAs) and long non-coding RNAs [7, 8]. In this study, we provide evidence for the clinical utility of 14q32 miRNAs as individualized prognostic biomarkers in osteosarcoma. Additionally, we show that there are substantial global transcriptional (miRNA and messenger RNA (mRNA)) changes across clinical risk groups, and we find in vitro and clinical evidence that differential methylation in the 14q32 non-coding cluster region may be underlying the miRNA expression changes and different tumor aggressiveness phenotypes. Our findings suggest that the non-coding 14q32 cluster contains a large number of useful clinically relevant biomarkers and is a locus of substantial genomic and epigenetic alterations that give rise to novel subtypes of osteosarcoma with distinct clinical, molecular, and biological context and therapeutic implications.

## Results

### 14q32 miRNAs accurately predict individual patient outcome

**Fig. 1:**
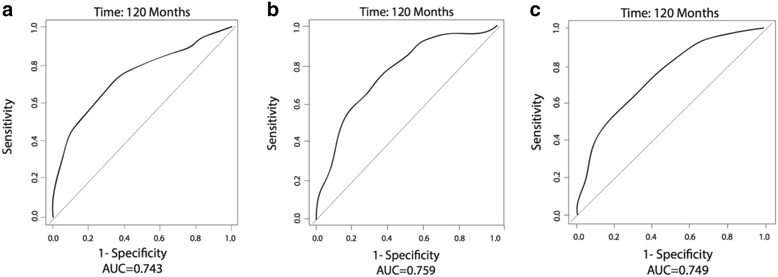
Time-dependent ROC analyses using various sets of 14q32 miRNA markers in the Boston cohort. **a** Signed averaged expression of top 5 miRNA markers (miR-495, miR-329, miR-487b, miR-410, miR-656). **b** Signed averaged expression of 18 miRNA markers from a previously published 18-miRNA signature. **c** Signed averaged expression of all miRNAs on 14q32

**Fig. 2:**
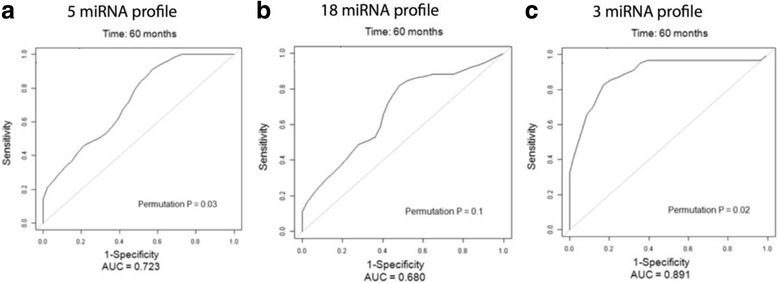
Time-dependent ROC analyses using various sets of 14q32 miRNAs in the Utah cohort. **a** Signed averaged expression of top 5 miRNA markers. **b** Signed averaged expression of 14 miRNAs mapped to the Agilent platform from the previously published 18 miRNA signature. **c** Penalized Cox regression model using three of the top 3 miRNA markers

While miRNA profiles were prognostic of outcome in recent studies, the precise clinical utility of miRNAs located in the 14q32 region for individualized patient outcome prediction has not yet been determined. We used two previously published genomic datasets with outcome annotation (called “Boston” and “Utah” datasets) and studied the clinical prognostic utility of the 14q32 miRNAs via the time-dependent receiver operator characteristic (tdROC) curve method. A summary table for the two datasets, the details of which have been previously reported [6, 9], is provided in Additional file 1. In order to minimize overfitting that is frequently associated with the construction of complex multivariate models, we used the simple, yet robust “signed average” approach. First, we generated a tdROC curve based on the signed average of the top 5 prognostic miRNAs residing at the 14q32 locus, which were previously identified in the Boston dataset [6] (and were able to be mapped on the Agilent platform used in the Utah dataset). These were miR-495, miR-329, miR-487b, miR-410, and miR-656, and the resulting tdROC curve was highly discriminatory for recurrence at 120 months (area under the curve (AUC) = 0.743; Fig. 1a). Then, we mapped this five-miRNA profile on the Agilent array and assessed its performance in the Utah cohort again using the signed average method. In this analysis, the prognostic model was fully frozen (selected miRNA features and signs were predefined in the Boston cohort) and applied to the Utah cohort, and we found that the 5-miRNA profile maintained a very strong discriminatory power for overall survival at 60 months (AUC = 0.723, permutation *p* = 0.03; Fig. 2a). The 60-month endpoint was chosen due to the much shorter follow-up in the Utah cohort.

Given the rich miRNA content of the 14q32 chromosomal region, we were interested to test if the power for prognostic discrimination extended beyond the top 5 miRNA prognostic markers. Thus, we extended this analysis to a group of 18 miRNAs on the same locus that were part of a larger prognostic profile (that included the top 5) in the previous study and found that that they still accurately predicted an individual patient’s risk to recur in both the Boston and Utah cohorts (Figs. 1b and 2b). Then, we tested all 62 miRNAs that are located on 14q32, and we found that the collection of these miRNAs offered good discriminatory capacity as well in the Boston cohort (Fig. 1c). We could not perform this analysis in the Utah cohort due to significant differences in the probe content between miRNA DASL (cDNA-mediated annealing, selection, extension, and ligation) and Agilent miRNA arrays, such that a significant subset of the total group of 14q32 miRNAs could not be mapped on the arrays from the Utah cohort. These results demonstrate that significant prognostic power resides on the entire 14q32 non-coding region, and miRNA subsets from this locus can be used for very accurate prognostic discrimination, using a simple method that minimizes model overfitting.

#### Model streamlining and additional validation

**Fig. 3:**
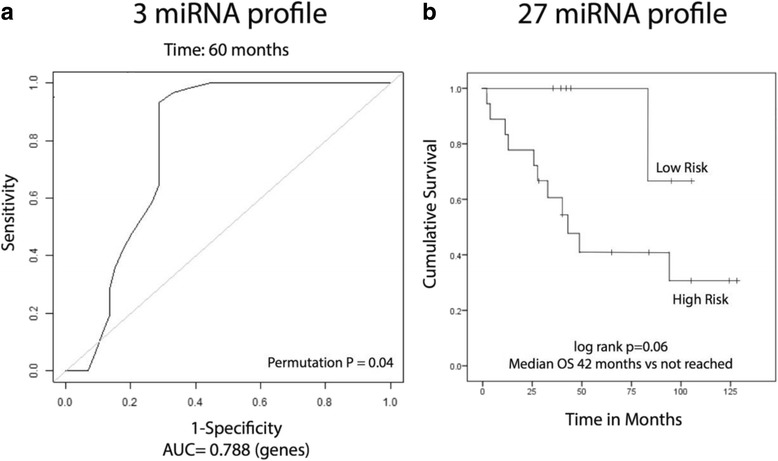
Prognostic analysis using 14q32 miRNAs in the Texas dataset. **a** Time-dependent ROC analysis using the candidate top 3 miRNA markers in a penalized Cox regression model in the Texas dataset. **b** Kaplan-Meier analysis using two patient clusters generated by unsupervised hierarchical clustering using 23 of all previously defined 27 prognostic 14q32 miRNAs that were available on the TaqMan qRTPCR assay

As a prelude for future clinical optimization, we also performed multivariate modeling using penalized Cox regression and found a possible modest gain in accuracy with potential use of only three mRNA markers (AUC = 0.891, permutation *p* < 0.01, and AUC = 0.806, *p* = 0.02, for the two datasets, respectively; Fig. 2c). We then tested the 3-miRNA profile on another, third dataset, which became recently available (called the “Texas” dataset [10]) and had not been used before in any of our analyses (Additional file 1). After mapping the profile to the Taqman qRTPCR assay used in the Texas dataset, we performed penalized Cox regression, which strongly supported the reproducibility of the 3-miRNA model (AUC = 0.788, permutation *p* = 0.04; Fig. 3a). In order to further control for any residual amount of overfitting for the 3-miRNA model, we also performed the same analysis using 20 randomly generated lists of 3 miRNAs from the global Taqman assay, and none of these randomly generated models performed as well as the candidate 3-miRNA model. We then extended our analysis to the entire group of prognostic miRNAs previously identified in the Boston dataset. Using 23 of those 27 miRNAs that were available on the Taqman platform, we performed unsupervised hierarchal clustering of the samples in the Texas dataset. This resulted in two groups with substantially different survival (median survival 42 months versus not yet reached, log-rank *p* value 0.06; Fig. 3b). Given the sample size limitations and the technical differences between the three different expression platforms (DASL, Agilent, Taqman) involved in this analysis, these results provide strong independent evidence for the prognostic role of the 14q32 miRNA cluster in osteosarcoma.

### 14q32 prognostic profiles and outcome following chemotherapy regimen selection

**Fig. 4:**
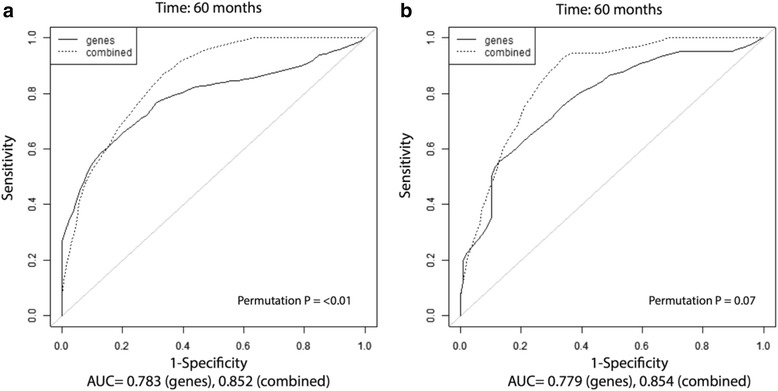
Multivariate prognostic models including 14q32 miRNA profiles and clinicopathologic covariates. Multivariate models included signed averaged expression of **a** 5-miRNA and **b** 18-miRNA profiles (only 14 of 18 were included in this platform), together with pathologic necrosis and use of alternate postoperative chemotherapy regimen

For the patients who experience suboptimal response to standard preoperative methotrexate, doxorubicin, and cisplatin (MAP) chemotherapy (defined as <90% necrosis in the operative specimen), there remains uncertainly as to whether adding alternate chemotherapy regimens such as ifosfamide/etoposide (IE) offer any benefit, with studies to date, including a recent large randomized trial, failing to show survival benefit. We were interested to assess if the miRNA profiles may have prognostic interaction with chemotherapy choice. In the Boston dataset (the only one for which details of postoperative alternative chemotherapy regimens were available), we constructed multivariate models using the signed averaged expression values for the 5-miRNA and 18-miRNA profiles, together with two clinicopathologic covariates, namely chemotherapy-induced necrosis and use of postoperative alternate chemotherapy regimen in addition to conventional MAP chemotherapy. tdROC analysis showed improved prognostic power with the combined models (AUC = 0.852, permutation *p* < 0.01, and AUC = 0.854, permutation *p* = 0.07, respectively) at the 60-month follow-up time (Fig. 4) while use of chemotherapy as the sole prognostic variable was (as expected) not prognostic, and analysis combining the profiles with use or not of alternate chemotherapy did not show any improvement in prognostic power. (Analysis at a follow-up time of 120 months produced similar, only slightly lower, AUC values compared to the 60-month time point analysis (Additional file 2). This observation raises the possibility that these miRNA profiles may affect outcomes in the context of pathologic necrosis and use of alternate adjuvant chemotherapy, although this would need to be proven in a larger prospectively designed study, where predictive interaction with chemotherapy can be statistically assessed.

### Robust osteosarcoma molecular subtype discrimination with subsets of 14q32 miRNAs

**Fig. 5:**
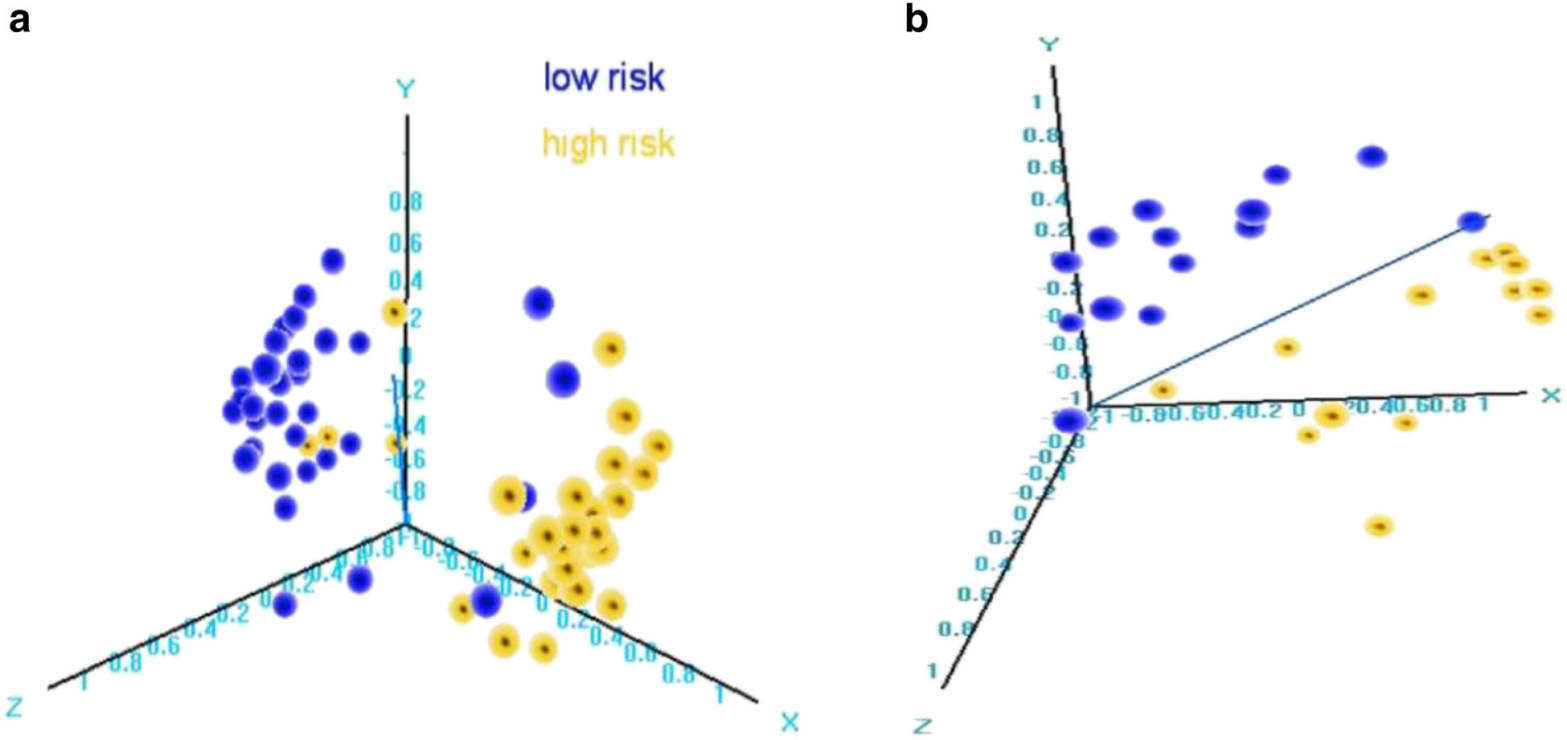
Display (multidimensional scaling (MDS)) of sample risk stratification based on 14q32 prognostic miRNAs. **a** MDS of the Boston cohort using 18 miRNAs. **b** MDS of the Utah cohort using 14 miRNAs that were mapped on the Agilent array. *Yellow/blue color* represents sample risk assignments according to the 5-miRNA prognostic model. Classification was highly concordant between the 5 and the 18 miRNA set

We then considered the hypothesis that 14q32 miRNAs are not simply markers of prognosis but signify previously unrecognized distinct molecular subtypes of osteosarcoma. To address this, we first ascertained that sample assignments as “high” or “low” risk were not sensitive to the precise classification algorithm that was used. For example, class assignments were highly similar whether the 5-miRNA profile was used in the signed averaged expression model (described in the previous paragraph) or in a clustering-based grouping (Fisher’s exact *p* < 0.001) or in the supervised multivariate 5-miRNA model previously published (Fisher’s *p* < 0.001). This observation also held true in the Utah dataset (Fisher’s *p* < 0.001, for the signed average-based versus clustering-based grouping). Further, the classification was stable when we used a larger set of 18 miRNAs from this locus to classify samples using any of these methods in both datasets (Fig. 5a, b). Finally, similar concordance was observed in the Texas data, where the 3-miRNA model classification was highly associated with the groups generated by hierarchical clustering using 3, 5, and 23 of the prognostic miRNAs mapped on the Taqman platform (Fisher’s *p* = 0.04, 0.07, and 0.03, respectively). Confirmation of strong similarity in terms of risk group assignments for the samples independent of the specific number of miRNAs and grouping method suggests that the miRNA markers track potential underlying molecular phenotype.

### Substantial global miRNA changes across osteosarcoma subtypes

**Fig. 6:**
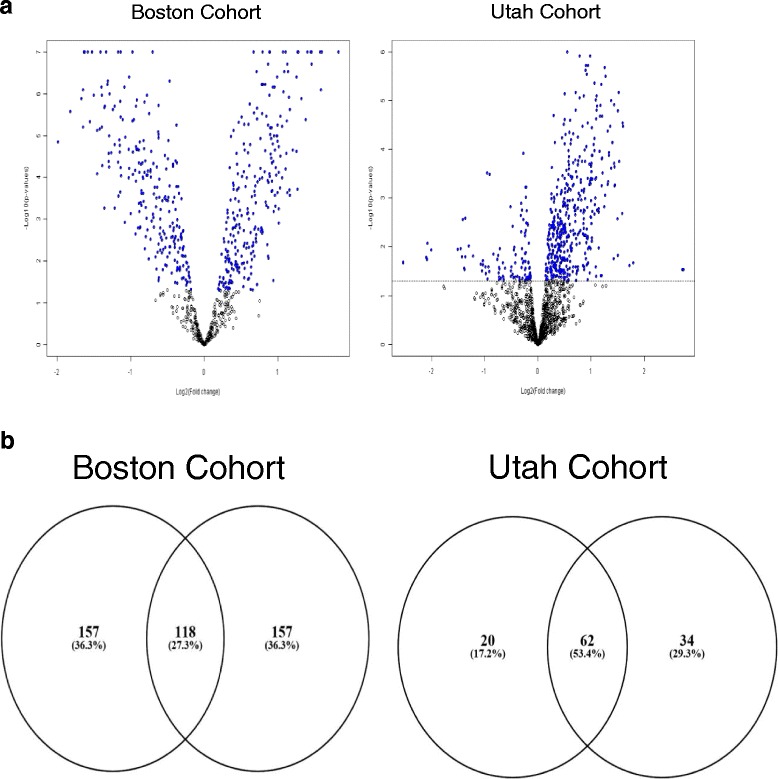
Global miRNA differential expression and overlap in the Boston and Utah datasets. **a** Global miRNA differential expression between high- and low-risk subtypes in the Boston dataset (*left panel*) and the Utah dataset (*right panel*). *Blue dots* represent probes with statistically significant differential expression. **b** Overlap between the Boston and Utah datasets in terms of miRNAs shown in **a**. *Left panel* shows the total overlap of all differentially expressed miRNAs. *Right panel* shows the overlap in miRNAs upregulated in high-risk subtypes (hypergeometric test *p* < 0.0001 for the overlap in both comparisons). Overlap in miRNAs downregulated in high-risk subtypes was smaller but still statistically significant (*p* < 0.01)

**Fig. 7:**
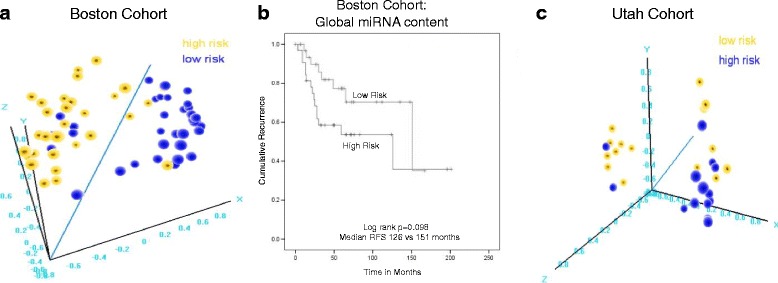
Global miRNA content distinguishes patients with different recurrence and survival probabilities. **a** MDS of the Boston cohort. **b** Kaplan-Meier analysis (recurrence-free survival) in the Boston cohort groups based on unsupervised hierarchical clustering of samples using the global miRNA content. **c** MDS of the Utah cohort. *Yellow/blue color* represents sample risk assignments according to the 5-miRNA prognostic model. Classification was highly concordant for the 5-miRNA set and clustering using the global miRNA content

If the two prognostic risk groups represent molecular subtypes, one might expect that they display large-scale molecular differences in addition to the marker 14q32 miRNAs. Thus, we performed global miRNA differential expression analysis between the high- and low-risk groups in the Boston dataset (which were defined in our previous report) and found that 492 miRNA probes (64%) were differentially expressed across the risk subtypes (*t* test *p* < 0.05; Benjamini-Hochberg false discovery rate (FDR) <0.07; Fig. 6a, Additional file 3). Since the proposed subtypes are not yet established, and in order to ascertain that these subtype-related miRNA expression differences are not due to a statistical artifact, we generated 100 random splits of the samples and tested them for differential miRNA expression. None of these randomly generated groups yielded a similar level of global miRNA differential expression. Multidimensional scaling (principal component analysis) was then performed with the global miRNA content (all miRNAs only filtered by low variance), and the three-dimensional sample groups were highly associated with the 5-miRNA model risk predictions (Fisher’s *p* < 0.001; Fig. 7a). Of interest, hierarchical clustering the samples using the global miRNA content provided a trend for prognostic discrimination (median RFS 126 versus 151 months; log-rank *p* = 0.092; Fig. 7b), though it did not reach the level of statistical significance achieved when using the 14q32 miRNA markers. We then assessed the correlation of the miRNAs with DICER, a key gene involved in the miRNA biogenesis and processing machinery. We found that only a small fraction of the miRNAs that were differentially expressed between the subtypes (5%) were moderately or strongly correlated with *DICER1* (Spearman rank correlation coefficient = 0.44–0.51; *p* < 0.01; Additional file 4), suggesting that DICER may account for (only) a small part of the miRNA deregulation in the subtypes.

These findings were reproduced in the Utah dataset, where 546 miRNA probes (36%) were differentially expressed across survival risk groups (*p* < 0.05; FDR <0.139; Fig. 6a, Additional file 3), but with no similar differences observed in any of the 100 random sample splits. Multidimensional scaling/principal component analysis using the global miRNA content also demonstrated that the risk groups were significantly associated with the 5-miRNA signed averaged expression-based predictions (Fisher’s *p* = 0.05; Fig. 7c) and classification was highly concordant regardless of the number of miRNAs used in clustering. Finally, in the Texas dataset, hierarchical clustering using different numbers of miRNAs (3, 5, 23) produced highly similar groups, which were also similar to clustering using the 14q32 miRNAs or even the global miRNA content (Fisher’s *p* < 0.05 for all comparisons). These results from the three datasets support the notion that these risk groups represent true molecular osteosarcoma subtypes with substantial underlying molecular differences beyond the set of 14q32 miRNAs.

We then explored this hypothesis in a publicly available miRNA dataset from 19 osteosarcoma cell lines [11]. In this dataset, experimental data were available, grouping the cell lines according to their proliferative capacity. We compared highly aggressive and less aggressive cell lines (by virtue of high proliferation versus low proliferation, as previously published [12]) and again found a substantial amount of differential miRNA expression. Importantly, we also found a moderate to strong amount of overlap in subtype-specific differential expression among the two clinical and the cell line datasets. Figure 6 shows the scale of differential miRNA expression between high- and low-risk subtypes and the degree of overlap between the two clinical datasets, which was highly significant using a hypergeometric distribution test. Generally, there was a much stronger concordance in miRNAs upregulated in the aggressive phenotype than in miRNAs downregulated in the aggressive phenotype among the datasets. Details of the cell line miRNA differential expression analysis are provided in Additional file 3. The observation of reproducible large-scale miRNA overexpression in aggressive samples in two clinical and one in vitro datasets further supports the hypothesis of distinct molecular osteosarcoma subtypes with different biologic and clinical behavior.

### Global mRNA expression changes across osteosarcoma subtypes

We also studied global mRNA transcript changes (other than microRNA), which were available for a subset of 37 samples from the Boston dataset, with respect to the risk subtypes, and found that 1362 mRNA probes (13%) were differentially expressed across risk subtypes (*p* < 0.05; FDR <0.275) in the Boston dataset. Although these changes were statistically more modest compared to the respective miRNA changes in supervised univariate analysis (partly due to the smaller sample size of the mRNA dataset), they still are consistent with the notion of largely different transcriptional programs between the two subtypes. Also, unsupervised genome-wide gene expression (mRNA) clustering generated subgroups (Additional file 5) that showed a strong trend for association with both the 5- and 18-miRNA previously defined model risk predictions (Fisher’s exact *p* = 0.05–0.08 for different association tests). These observations further speak to the possible existence of distinct molecular phenotypes. Interestingly, the list of differentially expressed genes was enriched for all the transcripts on the 14q32 genomic locus, taken collectively as a single gene set or potential functional unit (KS/LS enrichment *p* = 0.06). This collection includes 124 coding genes and 95 non-coding RNAs (long non-coding RNAs and small nucleolar RNAs (snoRNAs), which were included in the DASL whole genome array), raising the possibility of a regional mechanism of coordinated regulation affecting both coding and non-coding RNA elements on the 14q32 locus.

### 14q32 miRNAs are highly correlated with other 14q32 non-coding genes

In a hypothesis-based approach, we tested the 14q32 miRNAs and found that several of them were also highly correlated with other non-coding genes located on 14q32. For example, *MEG3*, a long non-coding RNA, was also highly correlated with several of the miRNAs, as were numerous snoRNAs located near the 14q32 miRNA cluster (*p* < 0.05, uncorrected because of the small number of variables). These correlations (Table 1) suggest a potentially highly coordinated mechanism of expression regulation including coding as well as non-coding elements within the larger imprinted 14q32 chromosomal region. A list of all significant correlations of the 14q32 genes with prognostic miRNAs is shown in Additional file 6.

**Table 1:** Association between long (or short) non-coding genes with prognostic miRNAs on the 14q32 locus

Gene name	Number of miRNAs	Average correlation	Correlation range
SNORD112	10	0.391	0.488 to 0.326
SNORD113-2	17	0.461	0.569 to 0.360
SNORD113-3	16	0.442	0.561 to 0.342
SNORD113-5	2	0.357	0.368 to 0.345
SNORD113-6	4	0.365	0.392 to 0.328
SNORD113-8	17	0.571	0.723 to 0.437
SNORD113-9	18	0.409	0.508 to 0.350
SNORD114-1	3	0.363	0.409 to 0.328
SNORD114-13	1	0.450	0.450
SNORD114-17	2	−0.337	−0.326 to −0.348
SNORD114-24	2	0.367	0.407 to 0.327
MEG3	13	0.449	0.570 to 0.327

Analysis in the Boston dataset (Spearman *p* < 0.05; total number of miRNAs tested, 28)

### 14q32 prognostic miRNAs correlate with aggressive osteosarcoma behavior in vitro

We used the 19-osteosarcoma cell line dataset (introduced above) to test the hypothesis that 14q32 miRNAs define distinct osteosarcoma subtypes in vitro, correlated with tumor aggressiveness as predicted by our genomic analysis of the clinical osteosarcoma cohorts. In this study, five variables were used as metrics of cancer cells’ aggressiveness: tumorigenicity, colony-forming ability, invasion, migration, and proliferation [13]. We selected all miRNAs on the 14q32 locus, which were associated with recurrence in univariate analysis, in our previous study of the clinical Boston cohort (Cox regression *p* < 0.05), resulting in a matrix of 27 miRNAs (a subset of which is the 5-miRNA profile shown above).

**Fig. 8:**
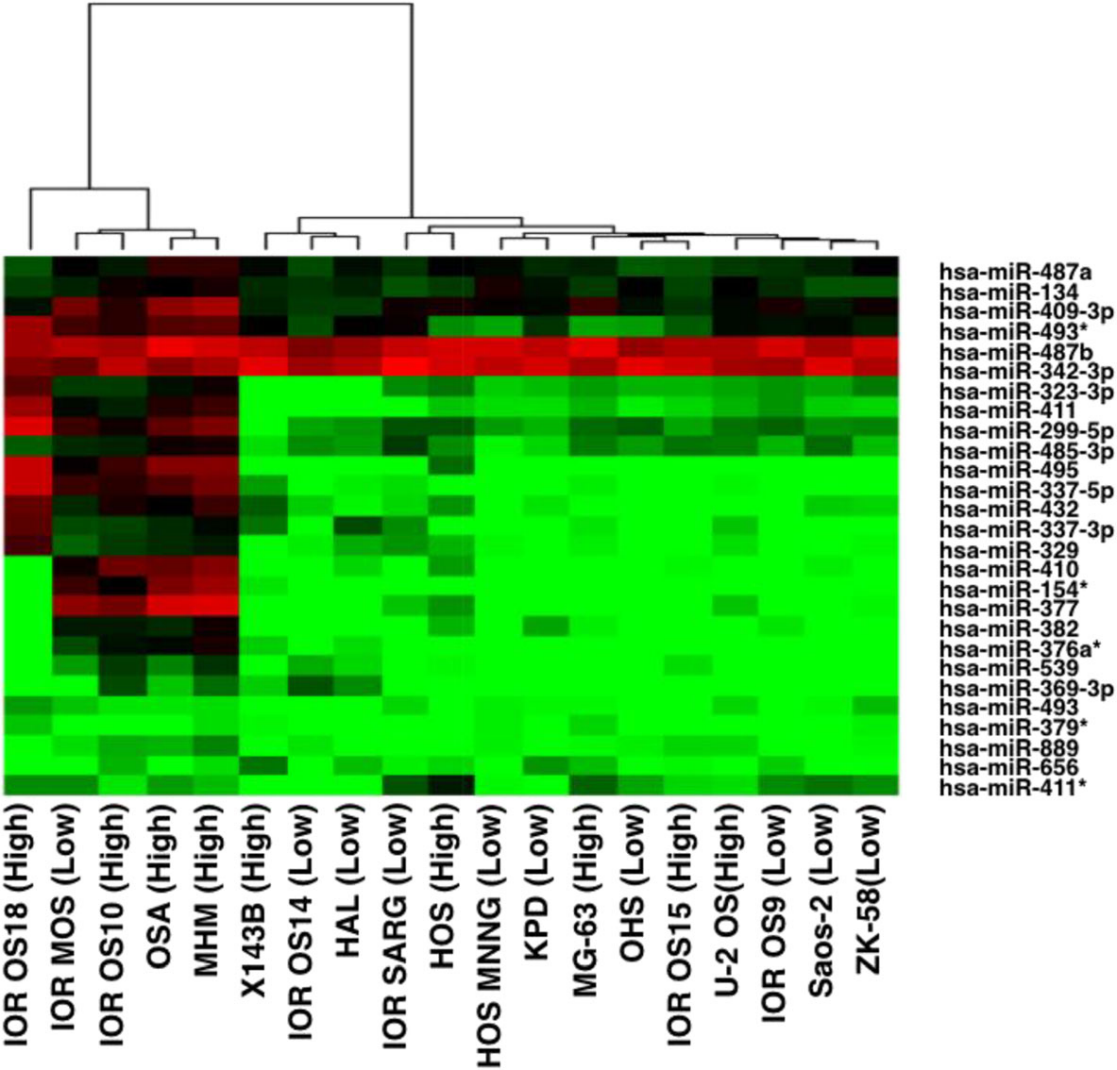
Hierarchical clustering of aggressive and non-aggressive cell lines. Separation by hierarchical clustering of aggressive and non-aggressive cell lines based on 27 miRNAs from 14q32 that are significantly associated with recurrence in the clinical Boston cohort (clusters were the same whether the top 5 or all 27 miRNAs were used). Aggressive cell lines tend to segregate preferentially together. (Mann-Whitney *p* = 0.02 for supervised median expression difference between the two groups). *Red*: relative overexpression. *Green*: relative underexpression

We studied the association between the 14q32 prognostic miRNAs with cell line “aggressiveness” metrics, as reported in the public dataset. We found that the median expression levels of the 27 prognostic miRNAs (in aggregate) were higher in the aggressive cell lines (defined by proliferative capacity, Mann-Whitney *p* = 0.02). In addition, unsupervised hierarchical clustering of the cell lines using the expression patterns of these miRNAs distinguished a cluster of mainly aggressive cell lines based on a composite metric of migration/invasion/colony-forming capacity, which can be viewed as an in vitro surrogate for metastatic potential (Fig. 8, Fisher’s *p* = 0.05 for association between the composite metric and miRNA-based cluster groups).

Further, we found a number of significant or strongly trending (*p* < 0.1) associations testing each individual miRNA in relation to the different aggressiveness metrics of the cell lines (Additional files 7 and 8). In a two-group differential expression analysis (high versus low proliferative cell lines), 13 miRNAs were upregulated in the more proliferative cell lines. Eight miRNAs were upregulated in highly invasive cell lines, while one was downregulated. Three miRNAs were upregulated in cell lines with increased migratory ability, while two were downregulated. Two miRNAs were upregulated in cell lines with higher colony-forming ability, while two miRNAs were upregulated in cell lines with higher tumorigenicity. Generally, the large majority of the miRNAs appear upregulated in the more aggressive phenotype, with the exception of hsa-miR-493 and hsa-miR-411*, which were consistently downregulated in the aggressive cell lines.

To circumvent possible pitfalls of binarization in cell line assays, we also analyzed proliferation as a continuous variable. In this analysis more (17) of the 27 prognostic miRNAs were highly or moderately correlated either positively (Spearman coefficient = 0.394 to 0.646) or negatively (Spearman coefficient = −0.403 to −0.582) with greater levels of proliferation at 72 h, which was the end time point of the proliferation experiments in the public data (all at *p* < 0.1). Two miRNAs were highly or moderately correlated with colony-forming ability (Spearman = 0.618, 0.653; *p* < 0.1) while three miRNAs were highly or moderately correlated with invasiveness (Spearman = 0.405, −0.418, −0.54; *p* < 0.1). Again, the majority of these correlations were positive, while hsa-miR-493 expression was negatively correlated with migration capacity at a significant or trending level (Spearman = −0.453; *p* < 0.1). This miRNA was the only one consistently negatively associated with the other more aggressive phenotypes in all types of statistical analysis. Proliferation appears to be the single individual attribute better capturing the extent of association between 14q32 miRNAs and cell line aggressiveness. However, the composite invasion/migration/colony-forming capacity metric also showed a clear association with aggressiveness, as a possible surrogate for clinical metastatic potential.

### Genomic map and context for methylation regulation in the imprinted 14q32 locus

**Fig. 9:**
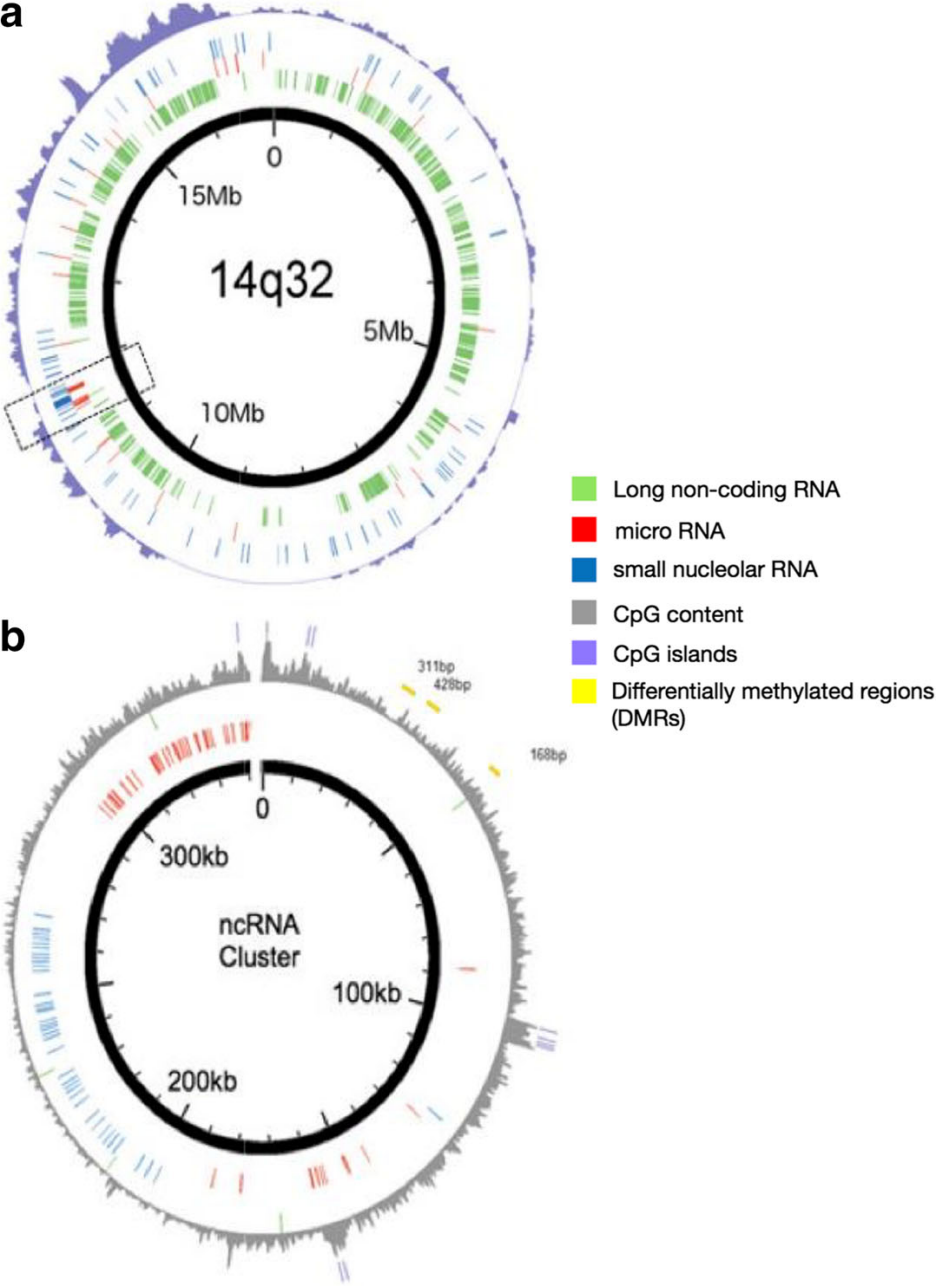
Map of the 14q32 locus and its non-coding cluster. **a** Map of the 14q32 locus. **b** Map of the non-coding RNA cluster on the 14q32 locus. Long non-coding RNAs are in *green*, miRNAs in *red*, snoRNAs in *blue*, differentially methylated regions (*DMRs*) in *yellow*, CpG content in *gray*, and CpG islands in *violet*

The hallmark of 14q32 is allele-specific methylation (imprinting). In order to explore the genomic context as it relates to imprinting control on miRNAs, we first considered a map of the locus containing the genes, non-coding RNAs (miRNAs or others), and CpG densities localized on 14q32 (Fig. 9a). Most of the prognostic miRNAs (previously defined in the clinical cohorts) are generally clustered in a 350-kb region of 14q32, which also contains the majority of all other non-coding RNAs on this locus, including 41 out of 47 snoRNAs. The non-coding RNA cluster also includes the *DLK1-DIO3* differentially methylated region (DMR), which includes both the intergenic DMR (IG-DMR) and the *MEG3* DMR and controls imprinting of this locus (Fig. 9b). Upon further exploration, we noted that there are several CpG islands (CGIs) within the 14q32 miRNA/non-coding cluster. CGIs are unmethylated in “normal tissue”; however, variable degrees of CGI methylation have been associated with various disease states including cancer [14].

### Methylation at the imprinted *MEG3* locus on 14q32 correlates with deregulation of the non-coding RNA cluster

Based on these observations, we were interested to study the association between miRNA expression and DNA methylation. The non-coding RNA cluster lies in the *DLK1-DIO3* imprinted region, which also includes the long non-coding RNAs, *MEG3*, *MEG8*, and *MEG9* (Fig. 8), and is regulated by at least two DMRs, the intergenic DMR and the *MEG3* DMR [15–17], with involvement of CCCTC zinc finger-binding factor (CTCF). Recent data suggest a process of loss of imprinting (LOI) in this region, which is involved in cancer development [18], and a correlation between methylation patterns in the *MEG3* promoter and expression of several miRNAs in the 14q32 ncRNA cluster has been suggested. The direction of the correlation may be different for methylation of CTCF binding sites (positive) versus conventional promoter methylation (negative) [16]. This is consistent with the known role of CTCF as a transcription enhancer blocker. We hypothesized that this regulatory pattern may hold true in osteosarcoma, and we tested this hypothesis using publicly available miRNA expression (Agilent array) and methylation data (Illumina 27K array) from the previously mentioned 19-osteosarcoma cell line dataset.

We focused on seven CpG sites interrogated by the 27K array, which are located upstream of the miRNAs and proximal to the *MEG3* promoter (no intergenic probes are interrogated by this array). One of these CpG sites, corresponding to the probe cg09280976, is located within a known CTCF binding site in the *MEG3* promoter, and another one, corresponding to the probe cg04291079, is located in the *MEG3* gene body, in a region where there is no known CTCF binding site. The other five probes are located close to but not entirely within CTCF binding sites. In our analysis, we found that 7 of the 27 14q32 prognostic miRNAs showed moderately or highly positive correlation with the methylation probe cg09280976 at a significant or strongly trending level (Spearman = 0.596–0.404; *p* < 0.1; Table 2). Eight of these 27 miRNAs were moderately or highly negatively correlated with the methylation probe cg04291079 at a significant or strongly trending level (Spearman = −0.393 to −0.552; *p* < 0.1; Table 2), while two were moderately positively correlated (Spearman = 0.554–0.400; *p* < 0.1; Table 2). Methylation probes located near, but not entirely within, CTCF binding sites showed variable, positive and negative, correlations with miRNAs (Table 2).

**Table 2:** Correlation between *MEG3* methylation probes and prognostic miRNAs

Methylation probe	Distance from *MEG3* TSS	Correlation with 14q32 prognostic miRNAs	Number of miRNAs	Overlap with CTCF binding site
cg1656	1326	None	None	Near site (in promoter)
cg0928	775	0.466 (0.596 to 0.404)	7	In site (in promoter)
cg2583	156	0.459 (0.528 to 0.396)	3	Near site (in promoter)
cg05711	−218	−0.561	1	Near site (within gene body)
cg1537	−628	−0.443 (−0.408 to −0.631)	13	Near site (within gene body)
cg1510	−1264	0.546 (0.621 to 0.428)	2	Near site (within gene body)
cg0429	−1968	−0.283 (−0.554 to −0.552)	10	Not in site (within gene body)

Locations of *MEG3* promoter and gene body methylation probes with respect to MEG3 transcription start sites (TSSs), CTCF binding sites, and correlation with the 14q32 prognostic miRNAs associated with proliferation (*p* < 0.1)

We also found that expression of 23 of the 27 prognostic miRNAs was also positively correlated with *MEG3* expression (Spearman = 0.808–0.409; *p* < 0.1) while only one miRNA was negatively correlated (Spearman = −0.582; *p* < 0.1). Finally, four of the methylation probes in the non-coding RNA cluster were positively correlated with *MEG3* expression (Spearman = 0.528–0.41; *p* < 0.1). These results, taken together, suggested a possible methylation-based regulation of both short and long non-coding RNAs in this imprinted genomic region.

**Fig. 10:**
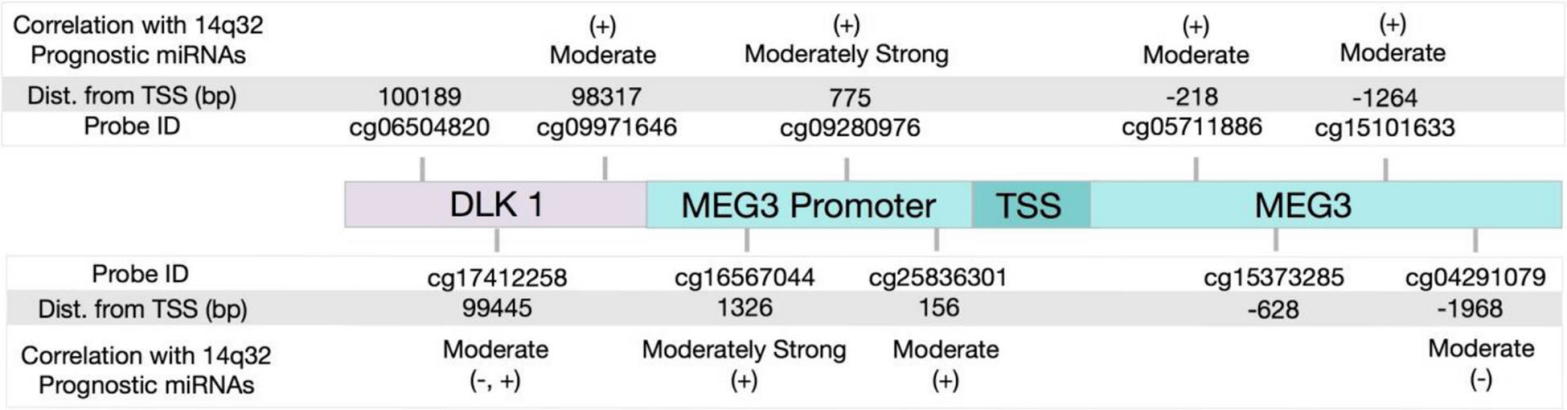
Association between *MEG3* methylation patterns and 14q32 miRNA expression in the clinical NCI TARGET data. Analysis of 10 probes for CpG sites within the *DLK1/MEG3* imprinted region included on the Illumina 450K methylation array. Correlation coefficients for each probe, averaged over the prognostic 14q32 miRNAs, are shown. Coefficients between 0.3 and 0.5 were considered moderately strong, and those between 0.15 and 0.3 were considered moderate. A + or − sign symbolizes a positive or negative correlation coefficient, respectively (Spearman *p* < 0.1). Upstream or downstream distances of the CpG sites from the *MEG3* TSS are also shown

We then sought to validate these observations using a genomic resource that recently became available by the NIH. The NCI Therapeutically Applicable Research to Generate Effective Treatments (TARGET) osteosarcoma project generated several genomic profiles for a large number of patient samples and includes DNA methylation data (on the more advanced Illumina 450K methylation array) as well as miRNA TaqMan qRTPCR expression data. For most of the *MEG3* CpG sites targeted by methylation probes, we found remarkable similarity between the moderate or moderately strong methylation/miRNA correlations observed in the cell lines (Table 2) and the correlations seen in the TARGET data (Fig. 10). This was particularly striking, for the two methylation sites were previously identified as clearly within (targeted by probe cg09280976) or clearly not within a CTCF binding site (targeted by probe cg04291079), both showing the same positive or negative association with miRNA expression seen in the cell line analysis. The rest of the *MEG3 *methylation sites showed generally similar variable associations seen in the cell line data with the exception of two probes within the gene body. These differences could be possibly related to the small sample size and inherently heterogeneous nature of the cell lines, as well as the fact that the effect of gene body methylation is much less clear than that of promoter methylation. Also, one promoter site (probe cg1656), which did not show significant associations in the cell lines, did show moderately strong associations in the TARGET data, which probably further supports the original hypothesis as it is located very near a CTCF binding site and it should be expected to show a clear positive correlation with expression. Due to publication restrictions currently in place by the NCI on the osteosarcoma TARGET data (see “Methods”), we are only allowed to provide numerical details of the correlation coefficients for a small subset of the miRNAs. Therefore, as an example, we show the average correlation coefficients for the following four miRNAs: miR-495, miR-329, miR-656, and miR-411*, which were some our top miRNA prognostic markers in the three previously analyzed clinical datasets (Boston, Utah, Texas). For these four miRNAs, the average correlation coefficients were as follows: cg09971646, 0.2105; cg16567044, 0.408; cg09280976, 0.323; cg25836301, 0.219; cg05711886, 0.185; and cg15101633, 0.199. These correlation coefficients were largely unchanged when the larger group of prognostic 14q32 miRNAs was considered.

Of interest, additional three CpG sites targeted by probes in the arrays and located in the *DLK1* gene locus showed moderate, variable (positive/negative), or no association with the miRNAs, potentially to be expected given their localization ~100 kb upstream of the *MEG3* transcription start site (TSS), further underscoring the potential specificity of the *MEG3* methylation effect on the non-coding RNA cluster.

### Differential methylation at the imprinted 14q32 locus correlates with aggressive osteosarcoma behavior in vitro

**Fig. 11:**
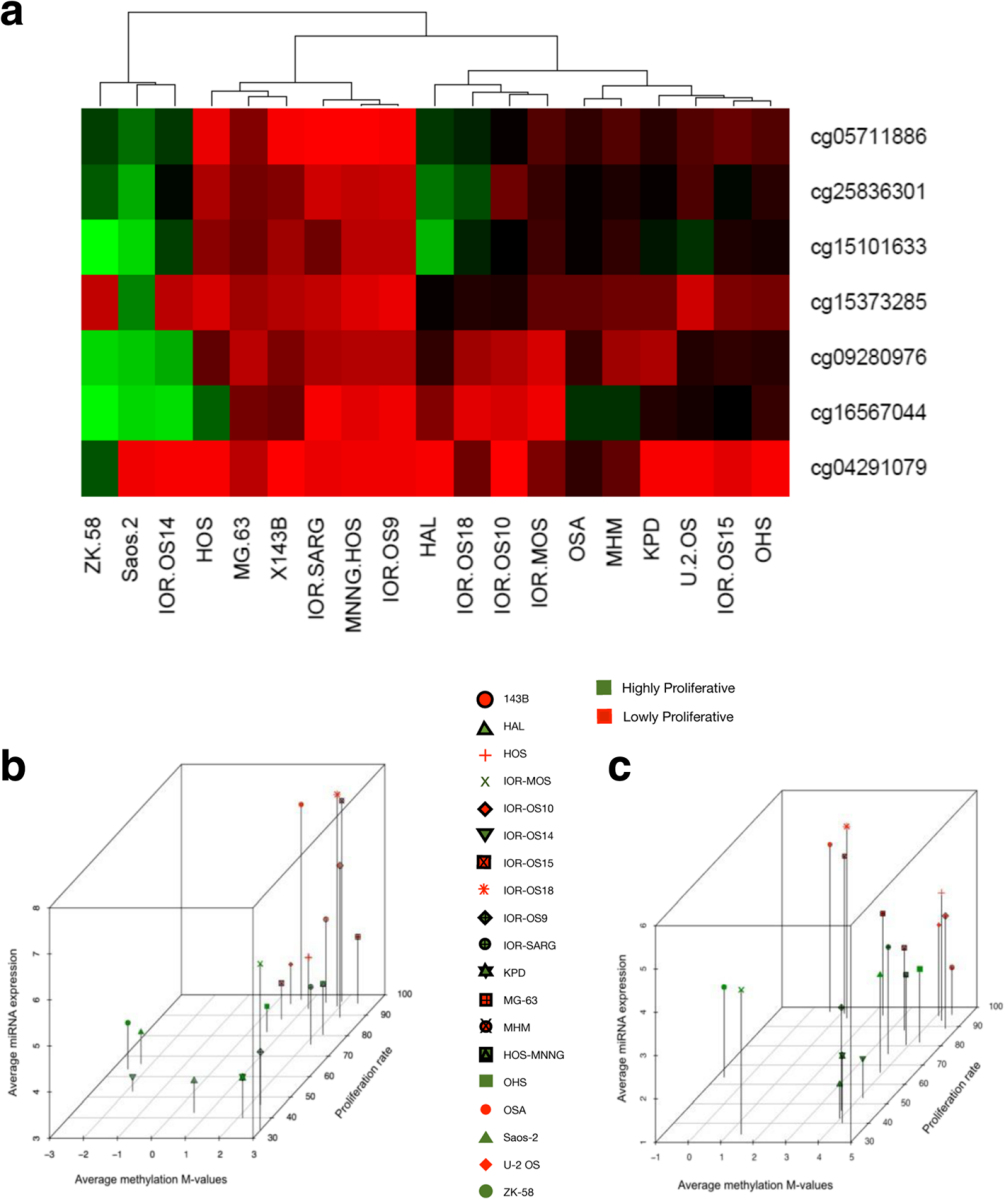
Association between methylation patterns and cell line proliferation. **a** Unsupervised clustering of the *MEG3* methylation probes shows an association between methylation patterns and proliferative capacity (Fisher’s *p* < 0.05). **b** Three-way plot of methylation intensity of the cg09280976 probe versus average prognostic miRNA expression and cell line proliferative capacity. **c** Three-way plot of methylation intensity of the cg04291079 probe versus average prognostic miRNA expression and proliferative capacity

Next, we used the same cell line data to determine if methylation patterns in the non-coding RNA cluster correlated with aggressiveness phenotypes. First, we performed hierarchical clustering using the methylation patterns of the seven *MEG3* methylation probes analyzed in the previous section and examined the resulting clusters for the 19 cell lines. We observed that the cell line clusters were significantly associated with aggressiveness (measured by proliferation, Fisher’s *p* < 0.05 when using either two or three proliferation groups, high/low or high/intermediate/low, as previously published) (Fig. 11a). Similar results were obtained when we repeated the clustering including additional four probes from the 27K array located in the *DLK1* promoter and gene body (recognizing that methylation effects often represent an aggregate of changes in a broader genomic region, especially when CTCF binding is involved).

In a two-group differential methylation analysis between two proliferation phenotypes (high/low), 7 of 11 CpG sites had higher methylation in cell lines with increased colony-forming ability (*p* < 0.1) (high and low categories as previously published). Four CpG sites had higher methylation in more invasive cell lines, cell lines with greater migratory capability (*p* < 0.1) as well as cell lines with higher tumorigenicity (*p* < 0.1), compared to their respective less aggressive counterparts. In a continuous variable correlation analysis, methylation at five CpG sites was positively correlated with colony-forming ability (Spearman = 0.634–0.392; *p* < 0.1). Methylation intensity at one CpG site near *DLK1* was also positively correlated with invasive ability (Spearman = 0.599); trending *p* < 0.1) while another CpG site was positively correlated with migration (Spearman = 0.5; *p* < 0.05). Detailed results of the differential methylation analyses and the continuous variable correlation analyses are included in Additional file 9.

We then hypothesized that there may be a three-way association between prognostic miRNA expression, methylation, and cell line aggressiveness. As an example, we focused on two CpG sites represented by Illumina 27K array probes cg09280976 and cg04291079, because, as explained above, their corresponding locations are known to be clearly within or clearly outside known CTCF binding sites and it was therefore conceptually easier to predict biologic associations. For each probe, we plotted CpG methylation versus average expression of miRNAs that were associated with that probe and with proliferative capacity at a Spearman *p* < 0.1 versus proliferative capacity as a continuous variable. The resulting 3-D plots (Fig. 11b, c) recapitulate possible biologic interactions in this imprinted region. For the probe that is located within a CTCF binding site, we observe two cell line clusters in the “corners” of the plot displaying the patterns “high methylation/high expression/high aggressiveness” and “low methylation/low expression/low aggressiveness,” respectively, consistent with the known transcription enhancer-blocking role of CTCF in the imprinted locus (Fig. 11b). For the probe that is located outside CTCF binding sites, we also observe two cell line clusters in diagonally opposed corners as compared to panel b. These clusters display patterns of “high methylation/low expression/low aggressiveness” and “low methylation/high expression/high aggressiveness,” respectively, consistent with a conventional negative regulatory effect of promoter methylation on expression. Associations between individual probe methylation and aggressiveness showed a strong trend for significance in panel b, while in panel c they did not, possibly related to the possible heterogeneity in the cell line group and the presence of some cell lines that did not necessarily follow these clear biologic patterns. Also, it is unlikely that one methylation probe, in isolation, can fully account for overall tumor behavior especially when its regulatory effect would be contrary to the broader methylation effect in this same region.

### Differential miRNA network targeting between high- and low-risk tumors reveals possible therapeutic targets

**Fig. 12:**
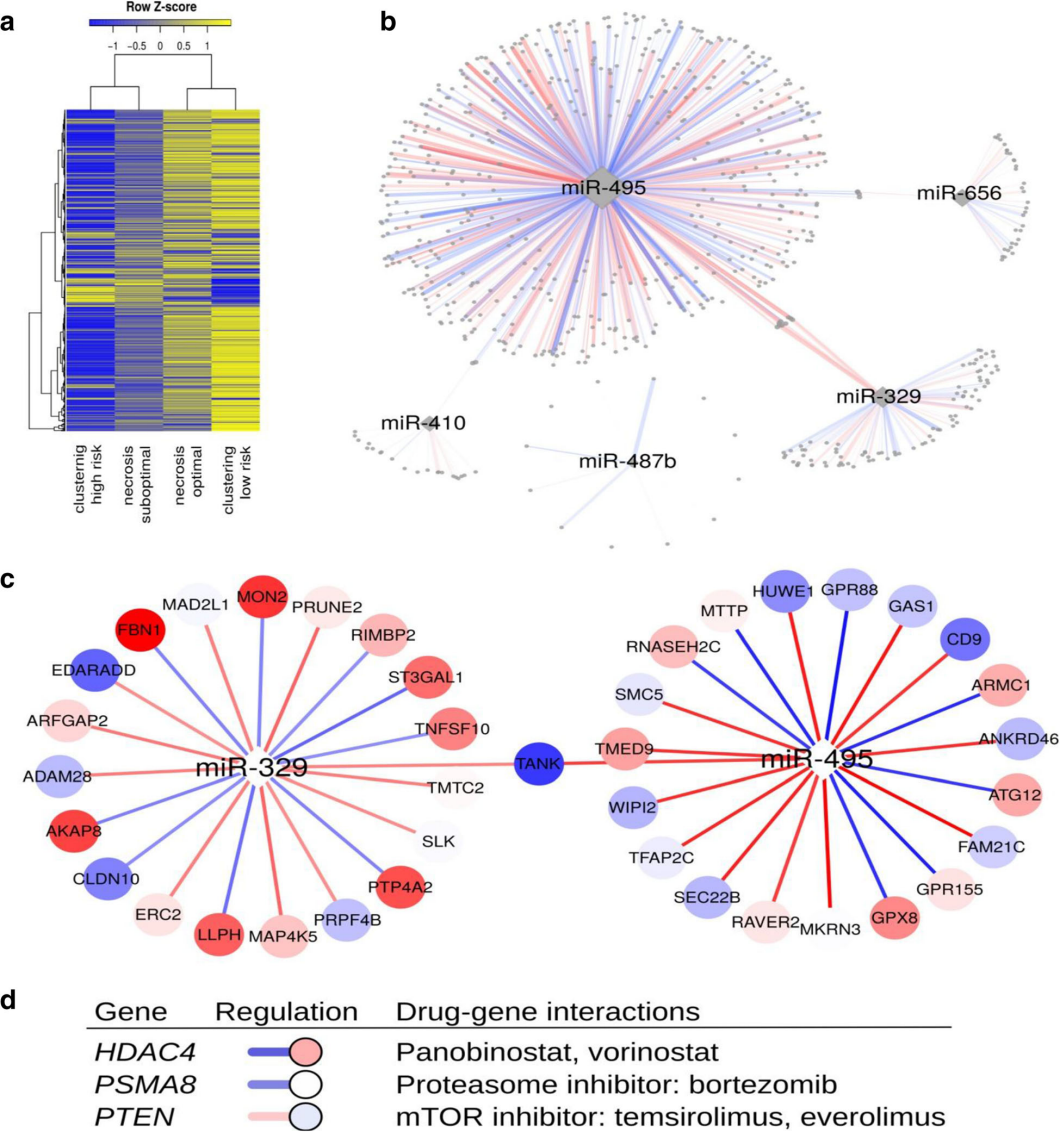
Differential miRNA networks between high- and low-risk patient groups in the Boston cohort. **a** Differential regulation (“targeting”) by all miRNAs present in the Boston dataset across the two recurrence risk groups (clustering high/low). For comparison, samples with high versus low chemotherapy-induced necrosis do not demonstrate major differences in miRNA targeting. **b** Network module derived from the 5-miRNA profile. Node size corresponds to the number of edges (connections). Edge thickness is proportional to statistical significance. **c** The two miRNAs with the highest number of significant edges from the network module shown in **b**. For both miRNAs, the 20 most differential edges are shown. **d** Selected candidate drug-gene interaction from the network module. In all networks, *red* and *blue edges* indicate edges with higher targeting in the high-risk and low-risk groups, respectively. In **c** and **d**, genes upregulated and downregulated in the high-risk group are shown in *red* and *blue*, respectively

Expression levels of miRNAs do not fully reflect their biologic activity. We previously reported on miRNA gene targets with gene set enrichment and found modest evidence that some miRNAs appear to exert regulatory influence [6]. However, this approach assumes that the regulators affect each gene equally and does not take into account multiple sources of information on gene regulation. Inference on biologic activity can be drawn by analyzing miRNA regulation of target gene expression in the context of a gene regulatory network. Our group recently described a network inference method for network that models the regulatory effects of transcription factors. This method (called “PANDA”) integrates different “omics” data types to infer network edges that accurately estimate gene expression regulation [19, 20]. Here, we implemented a modification of PANDA (called “PUMA,” PANDA Using MicroRNA Associations), which estimates regulatory effects of miRNAs on gene expression. We implemented PUMA to reconstruct networks for the high- and low-risk osteosarcoma subtypes using mRNA expression data from the Boston clinical cohort and “prior” data from the STRING database, JASPAR, and TargetScan. The gene regulatory networks showed substantial differences in gene targeting between the two risk groups. This is interesting in light of our earlier finding of a large number of differentially expressed miRNAs between the high- and low-risk patient groups, especially because our network construction model did not incorporate miRNA expression levels. In order to place this finding in a wider context, we analyzed chemotherapy response phenotypes in a similar manner and found much less differential miRNA targeting between groups of patients with optimal versus suboptimal chemotherapy-induced necrosis (Fig. 12a). We then focused on a network module derived by the 5-miRNA prognostic profile to determine what differences in gene regulation may be driven by these miRNAs (Fig. 12b, c). We identified significant differences in multiple edges of these networks using a permutation test on sample labels (Additional file 10). Patients with poor prognosis showed a particularly active module targeted by miR-495. The top differential edges of this network included at least two tumor suppressor genes (*GAS1* and *CD9*). Although miR-495 showed the strongest overall statistical signal for perturbed regulation, the other four miRNAs also contributed a number of statistically significant differential edges. These included multiple genes of known or potential significance including, for example, another known tumor suppressor gene in osteosarcoma (*RASF5*), which was differentially regulated by two of five miRNAs in this network module. Thus, increased targeting of tumor suppressor genes may be a mechanism by which these prognostic miRNAs individually or synergistically could affect tumor behavior and prognosis. Furthermore, Gene Ontology analysis of target genes in the network modules suggested that these miRNAs may differentially regulate pathways of cellular senescence, insulin signaling, osteoblast differentiation, and processes related to cellular proliferation and cell cycle and growth signaling (Additional file 11).

We then curated the list of all significant edges from the 5-miRNA network (in all 652 edges/637 genes) and screened it against the Drug Gene Interaction Database, a previously published interactive database of gene drug interaction supported by clinical or preclinical evidence. Figure 12d shows a selected subset of these interactions, suggesting possible hypotheses for therapeutic development including histone deacetylase (HDAC) inhibitors, proteasome inhibitors, or mTOR inhibitors, some of which are already in clinical or preclinical development in sarcoma or osteosarcoma. A detailed list of possible gene-drug interactions from the networks is provided in Additional file 12.

## Discussion

We, and others, previously reported that a collection of miRNAs on the 14q32 locus is associated with prognosis in osteosarcoma [6, 21]. Prognostic association via a Kaplan-Meier analysis does not guarantee the value of a test for predicting individual patient course. Thus, our tdROC analysis complements prior findings and demonstrates an excellent discriminatory power for miRNAs in this locus, which was replicated in three separate miRNA datasets. This replication is quite significant when one considers the very different characteristics of the three datasets (different array platforms, frozen versus paraffin tissue material, somewhat different clinical characteristics with respect to age distribution and the mix of metastatic/nonmetastatic cases, and a relatively small number of recurrence/death events in two of the datasets), which may also explain any minor differences in the findings among the three datasets. New studies will be required, in larger multi-institutional cohorts, in order to select the optimal subset of miRNAs among the 50–60 present at 14q32, for a clinically useful test to be developed. While potentially different subsets of these miRNAs could also be appropriate markers, we believe that the 5-miRNA or 3-miRNA profiles described in this study would be excellent candidates.

Evidence to date has been disappointing with respect to the use of alternate chemotherapy regimens, such as ifosfamide/etoposide, for patients who do not achieve an optimal pathologic response to standard preoperative chemotherapy with the MAP regimen. This was further underscored by the recent publication of the results from the large international randomized EURAMOS-1 study. This may signify true lack of clinical benefit from these regimens, but it could also suggest lack of an optimal stratification approach for selection of the right patient subset. Our findings suggest that these miRNAs may also be candidates for further study as possible markers for selecting patients for such alternate regimens, in stratification schemes that may include miRNA expression and conventional pathologic necrosis in the operative specimen. Proof of this concept will require analysis in a large cohort with prospectively randomized chemotherapy regimen allocation, such that a formal test of interaction can be performed. It was inherently not possible to perform such analysis in our data, but our findings would perhaps justify such a study in the future.

We suggest that, in addition to being prognostic markers, these miRNAs may track previously unidentified osteosarcoma molecular subtypes, potentially related to imprinting defects at 14q32. We found that the subgroups of patients defined by the miRNA risk profile harbor substantially different global (genome-wide) miRNA expression patterns, as well as different mRNA expression patterns. The miRNA patterns were more strongly associated with patients’ outcome compared to mRNA patterns. It is quite possible that miRNAs are better surrogates for tumor behavior, given their capacity to regulate large numbers of coding genes. In addition, miRNA detection may be more degradation proof than mRNA detection in banked tissue, though our results are possibly confounded by the smaller sample size of the mRNA dataset (compared to the miRNA dataset). That having been said, the characterization of patient samples as “high risk” versus “low risk” was consistent regardless of which particular method (supervised or unsupervised) or subset of miRNAs was used for sample risk prediction, and clustering-based groups were associated whether using either the global miRNA or the global mRNA data. Furthermore, the large-scale miRNA differences between high- and low-risk samples were also reproducible in two additional clinical and one in vitro datasets. Taken together, these observations support the notion of robust molecular subtypes in osteosarcoma with very different transcriptional programs, coding and non-coding.

Using the largest (published to date) osteosarcoma cell line dataset with genome-wide molecular information, we found that the expression patterns of the 14q32 miRNAs as a function of cell line aggressiveness were largely concordant with the findings in the clinical datasets. Cell line proliferation seemed to be the single best correlate of individual miRNA expression, although a composite metric of invasion/migration/colony formation that was used as a surrogate for metastatic potential also showed a clear association with collective 14q32 miRNA patterns. These findings notwithstanding, it should be acknowledged that attributes such as invasion and migration and overall clinical metastatic potential may also be more heavily dependent on tumor-stroma interactions and other elements, which are generally lost in cell line systems and which our current bioinformatics analysis in the cell lines may not adequately capture. Some of the individual miRNA associations presented are only strongly trending as opposed to nominally significant in the cell lines; however, the cell line datasets are often imperfect correlates of clinical observations, and the cell line dataset was much smaller compared to the clinical datasets, where the associations have been shown to be more robust. Also, 14q32 miRNAs can probably be considered functionally interrelated, and when we analyzed them in aggregate via clustering, their association with cell line aggressiveness was robust. Previous reports have demonstrated both tumor-promoting and tumor-suppressing effects of 14q32 miRNAs in different settings, but the outcome-related findings in our study are consistent with previously reported effects of these miRNAs in other malignancies such as leukemia, lung cancer, and liver cancer as well as their growth-promoting effect in mouse pluripotent stem cells [16, 22–24].

Network analysis using inferred miRNA-mRNA regulatory events by integrating target prediction with mRNA data provided insights into potential 14q32 miRNA-driven mechanisms in tumors with different prognosis and suggested that these may include perturbation of tsumor suppressor genes [25–29]. Furthermore, this analysis recapitulated the strong correlation between the miRNAs and tumor aggressiveness showing that their target networks include a large number of proliferation and cell cycle-related pathways and further supporting the notion of distinct regulatory programs affecting cellular behavior in the proposed osteosarcoma subtypes. To place these results in context, we observed a much wider network perturbation related to tumor aggressiveness and recurrence, compared to chemotherapy response, an endpoint that is more proximal in the natural history of the tumor. This leads us to speculate that overcoming short-term chemotherapy resistance may prove easier than achieving long-term remission or cure in osteosarcoma. This network analysis is limited by the fact that it provides in silico evidence, requiring future functional elucidation. Due to the multidimensional and multi-interactive nature of the networks, complex experimental designs will be needed in order to provide optimal in vitro systems for functional exploration of these networks. However, this is one of the first large-scale attempts to generate such networks in human specimen cohorts in this rare tumor.

The 14q32 chromosomal band uniquely contains a very large cluster (>100) of non-coding RNAs, including snoRNAs, microRNAs, and long non-coding RNAs, which is the largest known miRNA cluster in the genome (54 miRNAs). Genetic defects at 14q32 have been associated with severe developmental abnormalities, suggesting a very tight regulatory role in early tissue growth and differentiation. In our study, we noted a high degree of coordinated expression between miRNAs, snoRNAs, and long non-coding RNAs, suggesting perhaps an integrated mechanism of expression regulation in this region. Furthermore, 14q32 is an imprinted genomic region [7, 30, 31]. Imprinting is defined as allele-specific expression and is found in genomic regions critical for tissue growth and embryonic development. Typically, non-coding RNAs are expressed on the maternal allele while coding RNAs are expressed on the paternal allele, controlled by allele-specific methylation in genomic areas called imprinting control regions, or differentially methylated regions (DMRs). Disruption of this mechanism, called “loss of imprinting” (LOI), has been described not only in developmental abnormalities but also in cancer [18]. An enhancer-blocking factor, CCTC zinc finger-binding factor (CTCF), is also involved in gene and miRNA expression control in imprinted regions such as the *H19-IGF2* and 14q32 loci, in a methylation-sensitive manner. Specifically, it binds to unmethylated insulator sequences on DNA, preventing active enhancer-promoter interactions, thereby reducing transcription [15, 17, 32].

Our data support a three-way interaction between methylation, miRNA expression, and the phenotype (tumor aggressiveness), possibly contingent upon CTCF binding activity. Specifically, expression patterns of a large subset of the 14q32 miRNAs were associated with methylation patterns in the *ME*G3 DMR region, and both were associated with tumor aggressiveness. The mechanism behind this interaction is likely complex. We found that hypermethylation of *MEG3* DMR sites within a CTCF binding domain is associated with increased miRNA expression (potentially by inhibiting CTCF binding and its enhancer-blocking effect as described above) and higher tumor aggressiveness. In contrast, hypermethylation of *MEG3* DMR sites outside a CTCF binding domain is associated with decreased miRNA expression and decreased tumor aggressiveness. A very similar mechanism of methylation-sensitive CTCF binding and miRNA expression control at 14q32 was recently described in acute promyelocytic leukemia, and other reports have shown regulation of this imprinted domain by allele-specific enhancer activity in human embryonic stem cells [15–17, 32]. This genomic region has been reported to contain a large number of enhancer elements [33], and further studies will be required in order to identify which of them may be involved in the regulation of non-coding 14q32 genes in osteosarcoma.

The basic methylation/expression associations discovered in the cell lines were also reproducible in the large clinical osteosarcoma TARGET dataset. Perhaps more importantly, findings related to methylation sites within or outside known CTCF binding domains were highly similar between the in vitro and the clinical TARGET data. Future functional characterization and validation of the CTCF-related methylation/expression loop will require elaborate targeted designs including perhaps CRISPR approaches, but our initial observations herein, both in an in vitro and in a large clinical dataset, provide first evidence in support of this hypothesis.

Both coding and non-coding 14q32 genes, taken collectively as a single gene set, were enriched in the overall transcription program differences characterizing the subtypes. We also found evidence of coordinated expression regulation of coding and non-coding genes on 14q32, most strikingly a strong positive correlation between snoRNAs and miRNAs. Whether all these changes or changes in genes like *DICER* (a known miRNA processing gene) are functional elements of a wider pathogenetic mechanism controlled by imprinting and related to the biology of the osteosarcoma subtypes remains to be clarified. While very little is known on the function of snoRNAs in general, it is interesting to note that overexpression of 14q32 snoRNAs has been reported to promote tumor growth in acute leukemia [34, 35].

The methylation platform employed in the cell line dataset was not comprehensive enough to allow assessment of all relevant *MEG3* DMR CpG sites as well as the intergenic DMR sites. In addition, recent reports have suggested that the effect of methylation on gene expression in osteosarcoma may be different for different genomic “compartments” such as promoter CGIs, CGI shores, enhancers, or intergenic regions. Pilot analysis in a small clinical cohort showed either hypermethylation or hypomethylation in osteosarcoma tumors with high recurrence potential, and one other recent study also provided initial evidence of and insight into the effect of 14q32 methylation patterns in osteosarcoma [36, 37]. Our findings do not contradict but rather complement these reports and indicate that both IG-DMR and *MEG3* DMR methylation in conjunction with loss of imprinting, possible gene enhancer function, and expression of the entire 14q32 non-coding RNA cluster should be thoroughly studied in relation to osteosarcoma biology and outcome.

It is unclear what fraction of osteosarcoma tumors harbors the methylator phenotype proposed here, and studies in small clinical cohorts may underestimate its incidence. Though methylation data on the clinical cohorts were not available, the miRNA-defined high-risk patient group appears enriched for this phenotype, and the cell line data suggest that it could affect about 20–25% of the cases. This would be consistent with data in other tumor settings where the CpG island methylator phenotype (CIMP) is a relatively rare epigenetic phenomenon [14]. In addition, methylation changes in different imprinting control regions or genomic compartments may lead to different phenotypes. Integrated studies utilizing methyl sequencing and global non-coding and coding gene profiling in large national cohorts, such as the NCI TARGET osteosarcoma initiative, will hopefully provide answers to these questions. In addition, these comprehensive studies can also fully address the possible influence of chromosomal amplification or copy number variation on miRNA expression. This was not examined in our study, though prior literature suggests that genomic copy number variation may not be a big factor in the regulation of gene expression at 14q32 [38–43].

Ultimately, this line of research will allow the development of a set of mechanistically relevant clinical biomarkers based on loss of imprinting and/or non-coding RNA expression, with important therapeutic implications. Randomized trials have tested the addition of interferon or ifosfamide and etoposide to the standard neoadjuvant chemotherapy consisting of cisplatin/doxorubicin/methotrexate in localized osteosarcoma patients, using chemotherapy-induced pathologic necrosis as a risk stratification marker [3–5, 44]. While results to date point toward a lack of additional, or uncertain, benefit with either intervention, it is conceivable that rather than absolute lack of antitumor activity, these results reflect an imperfect marker for treatment stratification. Our prior work has shown that 14q32 miRNAs confer prognostic value independent of chemotherapy response, with the added benefit that they can be obtained early, at the time of diagnosis, as opposed to after 10 weeks of preoperative treatment [6]. As suggested above, a strategy that combines pathologic necrosis with miRNA biomarkers may allow for better treatment stratification in the future. Furthermore, development of methylation 14q32 biomarkers may enhance the discriminatory power of the miRNA assays, or even perhaps surpass them, as previous reports have suggested that methylation markers may be more stable and less susceptible to random variations over time in human cancer specimens [45]. Testing all these hypotheses in archived clinical trial material, such as that of the NCI TARGET initiative, could revolutionize an approach to adjuvant treatment as well as treatment with demethylating agents, such as decitabine, currently in clinical trials in metastatic osteosarcoma. In addition, new studies employing RNA and DNA methylation sequencing approaches will provide further depth in our understanding of the clinical and biologic effects addressed in this study. Finally, our drug-gene interaction screen, though computational in nature, was based on multiple sources of public experimental and clinical data, providing additional testable hypotheses for therapeutic development. Some of the drugs from our screen are already in clinical development for osteosarcoma (such as HDAC inhibitors), and an altered mTOR/PI3K/PTEN pathway was identified as a therapeutic target in 25% of tumors in a recently reported osteosarcoma genomic study [46]. One might envision a possible combinatorial and stratified application of these drugs, based on the molecular subtypes presented here.

## Conclusions

In conclusion, our findings support a set of clinically applicable biomarkers of osteosarcoma outcome localized on the 14q32 chromosome and suggest that this genomic region defines previously unrecognized molecular subtypes with distinct transcriptional programs and epigenetic regulation. An unmet medical challenge in osteosarcoma is the propensity of this tumor for early metastasis despite effective chemotherapy in a significant subset of patients. Modulation of the non-coding 14q32 region may ultimately address the highly proliferative and migratory potential of the aggressive subtypes, thus providing valuable new therapeutic avenues in this disease.

## Methods

### Human and cell line miRNA, mRNA, and methylation array data

We used three previously published clinically annotated human osteosarcoma datasets. One consisted of DASL miRNA expression data from 65 and mRNA data from 37 diagnostic biopsy specimens from Beth Israel Deaconess Medical Center and Boston Children’s Hospital (called “Boston dataset,” GEO accession GSE39040), and another consisted of Agilent miRNA expression data from 27 frozen tissue specimens from the University of Utah (called “Utah dataset,” Array Express accession E-MTAB-1136). The third dataset consisted of ABI TaqMan human microRNA qRTPCR data from 25 frozen diagnostic biopsy samples from the University of Texas Health Science Center (called “Texas dataset,” GEO accession GSE79181, details of which were published before [10]). Cell line miRNA data (Agilent arrays) and methylation data (Illumina 27K array) were derived from 19 osteosarcoma cell lines (published by the Institute of Cancer Research, Oslo University, GEO accession GSE28425, GSE36004). Transcription and methylation array details as well as clinical cohort and cell line annotations have been previously reported [6, 9, 11, 47].

We also used new data provided by the NCI, which launched the TARGET initiative, producing a repository of large-scale genomic data from a number of rare pediatric cancers. Within the osteosarcoma TARGET project, methylation profiles for 86 osteosarcoma patients (Illumina 450K array) and miRNA expression profiles for 89 osteosarcoma patients (MegaPlex TaqMan) became recently publicly available (http://target.nci.nih.gov/dataMatrix/TARGET_DataMatrix.html, retrieved November 10, 2016). The NCI has currently placed a limitation on publishing findings from analyzing osteosarcoma TARGET data. (https://ocg.cancer.gov/programs/target/target-publication-guidelines). This limitation allows investigators to only publish data from a focused analysis of a handful of genes, until the primary osteosarcoma project TARGET investigator team publishes their first “global” analysis of the genomic data. In complying with this limitation, any TARGET-derived data we present here are only related to methylation and expression analysis of a very small number of genes and miRNAs, all focused on the 14q32 locus. Therefore, our analysis is in no way similar in scope to the (currently unpublished) global genomic investigation of osteosarcoma undertaken by the TARGET initiative.

For any global expression analysis in the Boston, Utah, and Texas datasets, variance filtering was performed excluding 33% of probes with the lowest variance for the miRNA arrays and 66% of probes with the lowest variance for mRNA arrays, before performing any genome-wide (global) analyses. MiRNA expression values were quantile normalized before being subjected to further statistical analysis. For analyses involving methylation intensity, we utilized the *M* value (a transformation of the conventional beta value) as it has been shown to possess better statistical properties for differential analysis [48]. Further methodological details related to processing and analyzing these data are provided in Additional file 13.

### Recurrence and survival prediction and tdROC curves

In order to avoid overfitting, we used the signed average method and leave-one-out cross-validation in all survival analyses. In this approach, we averaged the expression levels of individual miRNA features in each candidate profile, weighted only by the sign of their hazard ratio (positive or negative) in univariate Cox regression analysis, and the resulting signed averaged metric was used as the prognostic index [49–51]. Kaplan-Meier analysis with log-rank test and Cox regression were used to analyze or model recurrence and survival as necessary. In the Utah dataset, time-censored recurrence data were not available, so we used overall survival as the time-censored endpoint. Time-dependent receiver operating characteristic (tdROC) and area under the curve (AUC) analyses were performed as previously described. For the Boston dataset, the tdROC endpoint was 120 months, while for the Utah dataset, it was set to 60 months due to the shorter follow-up available in that study. Penalized multivariate Cox regression analysis was performed as previously described [52].

### Class comparison, gene set enrichment, and clustering

Two-class comparison of continuous variables was done using the *t* test, and *p* values were corrected with the Benjamini-Hochberg FDR test while the Mann-Whitney test was used for non-parametric two-class continuous variable comparisons. In the case of testing a small number of variables based on prespecified hypotheses, we present unadjusted univariate *p* values. Univariate two-sided *p* values <0.05 were considered significant, except in some exploratory analyses in cell lines involving a limited number of preselected methylation and expression probes, where we also report associations at a *p* < 0.1 as suggestive of biologic inference. When examining the proposed molecular tumor subtypes, not previously established in the literature, we generated 100 random splits of the samples and considered that the expression differences between the proposed subtypes are significant if less than 5% of the random splits showed the same degree of differential expression as the proposed subtypes. We performed gene set analysis using the KS/LS statistic to determine if the expression profiles of the high- and low-risk groups were enriched for gene sets or miRNA targets of interest, according to the functional class scoring method [53]. Unsupervised hierarchical clustering was performed with the average linkage method as previously described [54]. We also used multidimensional scaling to construct 3-D representation of sample locations in the multivariate expression space.

### Standard biostatistical tests

We explored categorical associations using 2 × 2 tables and Fisher’s exact test. Spearman rank correlation was used to determine the association between continuous variables. The hypergeometric distribution test was used to assess significance of the overlap between two miRNA lists. All reported *p* values are two-sided.

### Chromosomal coordinates for methylation site localization and CTCF and enhancer binding site identification

Data plots showing gene coordinates and CpG island frequencies were plotted using Circos [55]. Chr14q32 was defined as the region between the co-ordinates 89,800,000 and 109,000,000 on chromosome 14. The location of genes, non-coding RNA, and miRNAs within these co-ordinates were derived from UCSC hg19 gene annotation tables. UCSC bed file annotations of CpG island locations in hg19 were used to determine CpG island location. CPG frequency was determined by counting the number of CpG islands within 100,000 bp upstream a given location. CTCF binding sites in *MEG3* were determined in previous studies [16, 17]. These sites were cross-referenced with the University of Tennessee CTCF binding site database, which includes data from the UCSC genome browser and the Broad Institute [56]. Search for enhancer sites was performed using the VISTA Enhancer Browser (Lawrence Berkeley National Laboratory) as previously described [33].

### MiRNA regulatory network analysis and drug-gene interaction screen

We used a method called PUMA (PANDA using MicroRNA Associations, Kuijjer et al., in preparation). This is a network reconstruction algorithm that models gene regulation by miRNAs and transcription factors and is an extension of our previously published network reconstruction method PANDA [19], which uses message passing between regulatory, protein-protein interaction, and gene expression data to model information flow between regulators and their target genes. PUMA extension works similar to PANDA, but in the message-passing steps does not allow microRNAs to form edges in the protein-protein interaction network, thereby indirectly influencing microRNA-target gene edges. We reconstructed networks for both subtypes and generated a set of “background” permuted networks to estimate edge significance (defined as activity score outside the four standard deviation range of the background distribution. Details are provided in Additional file 14, and implementation of the PUMA algorithm is available at https://github.com/mararie/PUMA. For drug target identification, we utilized the Drug Gene Interaction Database (DGIdb) as previously described [57].

### Bioinformatics/biostatistics analysis software

We used the NCI BRB-ArrayTools software (developed by Dr Richard Simon and the BRB-ArrayTools Development Team) and the SPSS software, version 18 (IBM Corporation, NY).

## Additional files

Additional file 1: Clinical characteristics of the Boston, Utah, and Texas datasets. These published datasets were used to assess the prognostic value of a subset of miRNA subsets located on 14q32 via the tdROC curve method. Available follow-up was different between the two datasets such that we needed to choose a primary endpoint of 120 months for the Boston dataset and 60 months for the Utah and Texas datasets. (PDF 141 kb)

Additional file 2: Multivariate prognostic models including 14q32 miRNA profiles and clinicopathologic covariates, analyzed at the follow-up time of 120 months. (PDF 447 kb)

Additional file 3: Differentially expressed miRNAs between the two subtypes in the three datasets. The Boston, Utah, and cell line data were analyzed to determine the differential expression of their miRNA population. (PDF 154 kb)

Additional file 4: Correlations between DICER and differentially expressed miRNAs in the Boston dataset. DICER1 is a gene that encodes for an endoribonuclease essential for the formation of microRNA, and it is also located on 14q32. We analyzed the correlation between miRNA expression on the 14q32 locus and DICER1 expression and found that a small fraction (5%) of miRNAs was significantly correlated with DICER1. (PDF 174 kb)

Additional file 5: Boston dataset hierarchical clustering using the messenger RNA (whole-genome) profiles. (Based on data available for a subset of 37 patients only). (PDF 20 kb)

Additional file 6: Correlations between prognostic miRNAs and other 14q32 transcripts in the Boston dataset. Eighteen prognostic miRNAs that we found on 14q32 were compared with the remaining coding and non-coding transcripts on that same locus to investigate whether a larger pattern of co-regulation may exist. (PDF 424 kb)

Additional file 7: Association between prognostic 14q32 miRNAs and cell line aggressiveness (binary analysis). An analysis between 14q32 miRNA expression and the following characteristics associated with cell line aggressiveness was performed: proliferation, invasiveness, migration, colony forming, and tumorigenicity. These attributes were analyzed as binary (categorical) variables. (PDF 538 kb)

Additional file 8: Association between prognostic 14q32 miRNAs and cell line aggressiveness (continuous variable analysis). Spearman correlation coefficients were assigned between 14q32 miRNAs and continuous variables representing cell line aggressiveness (colony forming, invasiveness, migration, and proliferation). (PDF 462 kb)

Additional file 9: Association between *DLK1/MEG3* methylation and cell line aggressiveness. The relationship between methylation and cell line aggressiveness was analyzed for methylation site neighboring or on *DLK1 * and *MEG3*, both located on 14q32. This analysis was limited by the number of available methylation probes for this region on the Illumina methylation array. Cell line aggressiveness was assessed via colony forming, invasiveness, migration, and tumorigenicity. (PDF 447 kb)

Additional file 10: Network edge differences between the two subtypes. (PDF 557 kb)

Additional file 11: Significant network GO categories. (PDF 412 kb)

Additional file 12: Network gene-drug screen interactions. This includes a detailed list of possible gene-drug interactions as a result from the screen applied between the significant edges from the 5-miRNA network and the Drug Gene Interaction Database. (PDF 488 kb)

Additional file 13: Supplementary methods. Additional details are provided on certain aspects of our analytical procedures. (PDF 461 kb)

Additional file 14: Supplementary PUMA methods. This file is the detailed protocol followed for the implementation of PUMA in order to reconstruct networks for high- and low-risk osteosarcoma subtypes. (PDF 998 kb)

## Abbreviations

AUC: Area under the curve
CGI: CpG island
CIMP: CpG island methylator phenotype
CTCF: CCCTC zinc finger-binding factor
DASL: cDNA-mediated annealing, selection, extension, and ligation
DGIdb: Drug Gene Interaction Database
DMR: Differentially methylated region
HDAC: Histone deacetylase
IG-DMR: Intergenic DMR
KS: Kolmogorov-Smirnov
LS: least squares
LOI: Loss of imprinting
PANDA: Passing Attributes between Networ for Data Assimilation
PUMA: PANDA Using MicroRNA Associations
snoRNAs: Small nucleolar RNAs
tdROC: Time-dependent receiver operating characteristic
TSS: Transcription start site
