# Supplementary material for: An imprinted non-coding genomic cluster at 14q32 defines clinically relevant molecular subtypes in osteosarcoma across multiple independent datasets
Source: J Hematol Oncol. 2017 May 15;10:107. doi: 10.1186/s13045-017-0465-4 (PMC5433149; doi:10.1186/s13045-017-0465-4)
Supplement: Supplementary file 1 — Clinical characteristics of the Boston, Utah, and Texas datasets. These published datasets were used to assess the prognostic value of a subset of miRNA subsets located on 14q32 via the tdROC curve method. Available follow-up was different between the two datasets such that we needed to choose a primary endpoint of 120 months for the Boston dataset and 60 months for the Utah and Texas datasets. (PDF 141 kb) [file 13045_2017_465_MOESM1_ESM.pdf]

| **Characteristics – Boston Dataset** | **Number (n= 65)** |
|---|---|
| *Age (years)* | |
| Median | 12 |
| Range | 3 to 76 |
| *Gender* | |
| Male | 30 (46%) |
| Female | 35 (54%) |
| *Tumor location* | |
| Axial | 3 |
| Appendicular | 62 |
| *Events* | |
| Recurrence | 23 |
| Death | 14 |
| *Metastases at diagnosis* | |
| No | 54 |
| Yes | 11 |
| *Survival follow-up time (months)* | |
| Median | 63 |
| Range | 0 to 201 |
| **Characteristics – Utah Dataset** | **Number (n= 29)** |
| *Age (years)* | |
| Median | 16 |
| Range | 3 to 62 |
| *Gender* | |
| Male | 17 (59%) |
| Female | 12 (41%) |
| *Tumor location* | |
| Axial | 1 |
| Appendicular | 28 |
| *Events* | |
| Recurrence | 10 |
| Death | 7 |
| *Metastases at diagnosis* | |
| No | 19 |
| Yes | 10 |
| *Survival follow-up time (months)* | |
| Median | 34 |
| Range | 4 to 107 |
| **Characteristics – Texas Dataset** | **Number (n= 25)** |
| *Age (years)* | |
| Median | 16 |
| Range | 7 to 28 |
| *Gender* | |
| Male | 14 (56%) |
| Female | 11 (44%) |
| | |

| | |
|---|---|
| *Tumor location* | |
| Axial | 1 |
| Appendicular | 24 |
| *Events* | |
| Recurrence | 14 |
| Death | 12 |
| *Metastases at diagnosis* | |
| No | 25 |
| Yes | 0 |
| *Survival follow-up time (months)* | |
| Median | 43 |
| Range | 2 to 128 |
