# Supplementary material for: An imprinted non-coding genomic cluster at 14q32 defines clinically relevant molecular subtypes in osteosarcoma across multiple independent datasets
Source: J Hematol Oncol. 2017 May 15;10:107. doi: 10.1186/s13045-017-0465-4 (PMC5433149; doi:10.1186/s13045-017-0465-4)
Supplement: Supplementary file 2 — Multivariate prognostic models including 14q32 miRNA profiles and clinicopathologic covariates, analyzed at the follow-up time of 120 months. (PDF 447 kb) [file 13045_2017_465_MOESM2_ESM.pdf]

Network gene -drug interaction screen

Interaction
MTTP and LOMITAPIDE
MTTP and UNII-Q70OH404HR
MTTP and HESPERETIN
GPX8 and GLUTATHIONE
CCL2 and 300816-15-3
CCL2 and MIMOSINE
CCL2 and DANAZOL
HGF and ABT-510
HGF and O2-SULFO-GLUCURONIC ACID
ABCC4 and ZIDOVUDINE
ABCC4 and 6-MERCAPTOPURINE
ABCC4 and LAMIVUDINE
DYRK1A and N-(5-{[(2S)-4-AMINO-2-(3-CHLOROPHENYL)BUTANOYL]AMINO}-1H-INDAZOL-3-YL)BENZAM
DAPP1 and INOSITOL 1,3,4,5-TETRAKISPHOSPHATE
SLK and DB07853
CDH2 and EXHERIN
NCOA1 and GENISTEIN
NCOA1 and [5-HYDROXY-2-(4-HYDROXYPHENYL)-1-BENZOFURAN-7-YL]ACETONITRILE
NCOA1 and 2-(5-HYDROXY-NAPHTHALEN-1-YL)-1,3-BENZOOXAZOL-6-OL
NCOA1 and 1-CHLORO-6-(4-HYDROXYPHENYL)-2-NAPHTHOL
NCOA1 and 5-HYDROXY-2-(4-HYDROXYPHENYL)-1-BENZOFURAN-7-CARBONITRILE
NCOA1 and 3-(6-HYDROXY-NAPHTHALEN-2-YL)-BENZO[D]ISOOXAZOL-6-OL
NCOA1 and 3-(3-FLUORO-4-HYDROXYPHENYL)-7-HYDROXY-1-NAPHTHONITRILE
NCOA1 and 4-(4-HYDROXYPHENYL)-1-NAPHTHALDEHYDE OXIME
NCOA1 and ALEGLITAZAR
NCOA1 and (2S)-3-(1-{[2-(2-CHLOROPHENYL)-5-METHYL-1,3-OXAZOL-4-YL]METHYL}-1H-INDOL-5-YL)-2-E
NCOA1 and 3-BROMO-6-HYDROXY-2-(4-HYDROXYPHENYL)-1H-INDEN-1-ONE
NCOA1 and 1,3-CYCLOHEXANEDIOL, 4-METHYLENE-5-[(2E)-[(1S,3AS,7AS)-OCTAHYDRO-1-(5-HYDROXY-5
CCR1 and UCB35625
VDAC2 and OLESOXIME
VDAC2 and DIHYDROXYALUMINIUM
GAD2 and L-GLUTAMIC ACID
GAD2 and PYRIDOXAL PHOSPHATE
THNSL1 and PYRIDOXAL PHOSPHATE
THNSL1 and L-THREONINE
HDAC4 and BELINOSTAT
HDAC4 and VALPROIC ACID
HDAC4 and PANOBINOSTAT
HDAC4 and VORINOSTAT
HDAC4 and PIVANEX

HDAC4 and PCI-24781
HDAC4 and DACINOSTAT
HDAC4 and GIVINOSTAT
HDAC4 and ROMIDEPSIN
HDAC4 and SCRIPTAID
HDAC4 and TRICHOSTATIN A
HDAC4 and CUDC-101
HDAC4 and PRACINOSTAT
HDAC4 and RESMINOSTAT
HDAC4 and 2,2,2-TRIFLUORO-1-{5-[(3-PHENYL-5,6-DIHYDROIMIDAZO[1,2-A]PYRAZIN-7(8H)-YL)CARBON[illegible]
HDAC4 and N-HYDROXY-5-[(3-PHENYL-5,6-DIHYDROIMIDAZO[1,2-A]PYRAZIN-7(8H)-YL)CARBONYL]THIO[illegible]
PRKAA2 and METFORMIN HYDROCHLORIDE
SCNN1A and AMILORIDE
SCNN1A and TRIAMTERENE
PDE4D and DYPHYLLINE
PDE4D and ROLIPRAM
PDE4D and RS-25344
PDE4D and RESVERATROL
PDE4D and ETAZOLATE
PDE4D and OXTRIPHYLLINE
PDE4D and (4R)-4-(3-BUTOXY-4-METHOXYBENZYL)IMIDAZOLIDIN-2-ONE
PDE4D and ROFLUMILAST
PDE4D and 4-[8-(3-NITROPHENYL)-1,7-NAPHTHYRIDIN-6-YL]BENZOIC ACID
PDE4D and IBUDILAST
PDE4D and KETOTIFEN
PDE4D and 3-ISOBUTYL-1-METHYLXANTHINE
PDE4D and PICLAMILAST
PDE4D and ILOPROST
CDK6 and FLAVOPIRIDOL
CDK6 and LEE011
CDK6 and LY2835219
CDK6 and PALBOCICLIB
CDK6 and CDK/CRK INHIBITOR
CDK6 and RGB-286638
CDK6 and APREMILAST
CTSS and N-[(1S)-1-{1-[(1R,3E)-1-ACETYLPENT-3-EN-1-YL]-1H-1,2,3-TRIAZOL-4-YL}-1,2-DIMETHYLPROPY[illegible]
CTSS and N-[(1S)-2-{[(1R)-2-(BENZYLOXY)-1-CYANO-1-METHYLETHYL]AMINO}-1-(CYCLOHEXYLMETHYL)-[illegible]
CTSS and N-[1-(AMINOMETHYL)CYCLOPROPYL]-3-(MORPHOLIN-4-YLSULFONYL)-N~2~-[(1S)-2,2,2-TRIFL[illegible]
CTSS and N-(1-CYANOCYCLOPROPYL)-3-({[(2S)-5-OXOPYRROLIDIN-2-YL]METHYL}SULFONYL)-N~2~-[(1S)-[illegible]
CTSS and N-[1-(AMINOMETHYL)CYCLOPROPYL]-3-(BENZYLSULFONYL)-N~2~-[(1S)-2,2,2-TRIFLUORO-1-(4[illegible]
CTSS and (1R)-2-[(CYANOMETHYL)AMINO]-1-({[2-(DIFLUOROMETHOXY)BENZYL]SULFONYL}METHYL)-2-[illegible]

CTSS and MORPHOLINE-4-CARBOXYLIC ACID [1-(2-BENZYLSULFANYL-1-FORMYL-ETHYLCARBAMOYL)-2-P
CTSS and DB08611
RORB and MELATONIN
ADAM28 and FUROYL-LEUCINE
ADAM28 and BATIMASTAT
CAMK2G and BOSUTINIB
UAP1 and URIDINE-DIPHOSPHATE-N-ACETYLGLUCOSAMINE
RICTOR and OSI-027
RICTOR and AZD8055
MAT1A and 3-OXIRAN-2YLALANINE
MAT1A and L-2-AMINO-4-METHOXY-CIS-BUT-3-ENOIC ACID
MAT1A and S-ADENOSYLMETHIONINE
CLCN6 and DIDS
CLCN6 and NITROUS OXIDE
CLCN6 and BUSULFAN
IRS1 and [4-({5-(AMINOCARBONYL)-4-[(3-METHYLPHENYL)AMINO]PYRIMIDIN-2-YL}AMINO)PHENYL]ACE
EDN1 and MURAGLITAZAR
GABRA1 and HALAZEPAM
GABRA1 and ALPRAZOLAM
GABRA1 and ESTAZOLAM
GABRA1 and BUTETHAL
GABRA1 and MIDAZOLAM
GABRA1 and ZOLPIDEM
GABRA1 and CLONAZEPAM
GABRA1 and TRIAZOLAM
GABRA1 and BUTABARBITAL
GABRA1 and FLUDIAZEPAM
GABRA1 and ADINAZOLAM
GABRA1 and FLUMAZENIL
GABRA1 and LORAZEPAM
GABRA1 and METHYPRYLON
GABRA1 and DIAZEPAM
GABRA1 and MEPROBAMATE
GABRA1 and FLURAZEPAM
GABRA1 and PRAZEPAM
GABRA1 and METHOHEXITAL
GABRA1 and ETOMIDATE
GABRA1 and THIAMYLAL
GABRA1 and OXAZEPAM
GABRA1 and CINOLAZEPAM
GABRA1 and DESFLURANE

GABRA1 and HEXOBARBITAL
GABRA1 and THIOPENTAL
GABRA1 and TEMAZEPAM
GABRA1 and BROMAZEPAM
GABRA1 and METHARBITAL
GABRA1 and SECOBARBITAL
GABRA1 and CLORAZEPATE
GABRA1 and CLOBAZAM
GABRA1 and BUTALBITAL
GABRA1 and NITRAZEPAM
GABRA1 and PICROTOXIN
GABRA1 and CLOTIAZEPAM
GABRA1 and TOPIRAMATE
GABRA1 and PROGABIDE
GABRA1 and PENTOBARBITAL
GABRA1 and TALBUTAL
GABRA1 and CHLORDIAZEPOXIDE
GABRA1 and QUAZEPAM
GABRA1 and SEVOFLURANE
GABRA1 and ZOPICLONE
GABRA1 and ISOFLURANE
GABRA1 and ENFLURANE
GABRA1 and PHENOBARBITAL
GABRA1 and ZALEPLON
GABRA1 and PRIMIDONE
GABRA1 and MUSCIMOL
GABRA1 and BRETAZENIL
GABRA1 and ISOGUVACINE
GABRA1 and [18F]FLUOROETHYLFLUMAZENIL
GABRA1 and INDIPLON
GABRA1 and GABAZINE
GABRA1 and GABOXADOL
GABRA1 and DMCM
GABRA1 and FLUNITRAZEPAM
GABRA1 and TETRAHYDRODEOXYCORTICOSTERONE
GABRA1 and TBPS
GABRA1 and ISONIPECOTIC ACID
GABRA1 and OCINAPLON
GABRA1 and BICUCULLINE
GABRA1 and METHOXYFLURANE
GABRA1 and MEPHOBARBITAL

GABRA1 and AMOBARBITAL
GABRA1 and ETHCHLORVYNOL
GABRA1 and ETHANOL
GABRA1 and ESZOPICLONE
GABRA1 and KETAZOLAM
GABRA1 and GLUTETHIMIDE
GABRA1 and AMOXAPINE
GABRA1 and DEHYDROEPIANDROSTERONE
GABRA1 and ACAMPROSATE
GABRA1 and HALOTHANE
GABRA1 and GANAXOLONE
GABRA1 and HEPTABARBITAL
GABRA1 and APROBARBITAL
GABRA1 and QUINIDINE BARBITURATE
GABRA1 and BARBITURIC ACID DERIVATIVE
GABRA1 and BARBITAL
GABRA1 and PROPOFOL
GABRA1 and OLANZAPINE
GABRA1 and ERGOLOID MESYLATE
PRKCB and ENZASTAURIN
PRKCB and QUERCETIN
PRKCB and PKCBETA INHIBITOR
PRKCB and BISINDOLYLMALEIMIDE IV
PRKCB and RUBOXISTAURIN
PRKCB and INGENOL MEBUTATE
PRKCB and BRYOSTATIN
PRKCB and BRYOSTATIN-1
PRKCB and SOTRASTAURIN ACETATE
PRKCB and VITAMIN E
PRKCB and ELLAGIC ACID
DCK and FLUDARABINE
DCK and 2'-DEOXYCYTIDINE
GABRP and BROMAZEPAM
GABRP and CLONAZEPAM
GABRP and CLORAZEPATE
GABRP and OXAZEPAM
GABRP and QUAZEPAM
GABRP and CINOLAZEPAM
GABRP and ADINAZOLAM
GABRP and FLUDIAZEPAM
GABRP and DIAZEPAM

GABRP and NITRAZEPAM
GABRP and MIDAZOLAM
GABRP and CHLORDIAZEPOXIDE
GABRP and CLOTIAZEPAM
GABRP and ALPRAZOLAM
GABRP and LORAZEPAM
GABRP and TEMAZEPAM
GABRP and TRIAZOLAM
GABRP and FLURAZEPAM
GABRP and HALAZEPAM
GABRP and PRAZEPAM
GABRP and CLOBAZAM
GABRP and ESTAZOLAM
GABRP and PICROTOXIN
GABRP and TBPS
GABRP and GABOXADOL
GABRP and OCINAPLON
GABRP and ETHANOL
RFK and RIBOFLAVIN MONOPHOSPHATE
RFK and RIBOFLAVIN
FASN and ORLISTAT
FASN and CERULENIN
IL25 and VIDOFLUDIMUS
CACNA1B and AMLODIPINE
CACNA1B and GABAPENTIN
CACNA1B and CLEVIDIPINE
CACNA1B and LEVETIRACETAM
CACNA1B and ZICONOTIDE
CACNA1B and SAFINAMIDE
CACNA1B and RALFINAMIDE
CACNA1B and CILNIDIPINE
CACNA1B and AGMATINE
CACNA1B and VERAPAMIL
PTGR2 and INDOMETHACIN
PTGR2 and DB07177
P4HA1 and L-PROLINE
P4HA1 and SUCCINIC ACID
P4HA1 and HYDRALAZINE
OPRM1 and HYDROCODONE
OPRM1 and NALOXONE
OPRM1 and ANILERIDINE

OPRM1 and FENTANYL
OPRM1 and METHADYL ACETATE
OPRM1 and BUPRENORPHINE
OPRM1 and OXYCODONE
OPRM1 and DIPHENOXYLATE
OPRM1 and OXYMORPHONE
OPRM1 and DEZOCINE
OPRM1 and PENTAZOCINE
OPRM1 and LEVALLORPHAN
OPRM1 and ALVIMOPAN
OPRM1 and TAPENTADOL
OPRM1 and BUTORPHANOL
OPRM1 and SUFENTANIL
OPRM1 and NALTREXONE
OPRM1 and LOPERAMIDE
OPRM1 and NALBUPHINE
OPRM1 and LEVORPHANOL
OPRM1 and MORPHINE
OPRM1 and ALFENTANIL
OPRM1 and REMIFENTANIL
OPRM1 and METHYLNALTREXONE BROMIDE
OPRM1 and CODEINE
OPRM1 and TRAMADOL
OPRM1 and METHADONE
OPRM1 and HYDROMORPHONE
OPRM1 and NALOXEGOL OXALATE
OPRM1 and FENTANYL CITRATE
OPRM1 and LEVALLORPHAN TARTRATE
OPRM1 and TAPENTADOL HYDROCHLORIDE
OPRM1 and SUFENTANIL CITRATE
OPRM1 and OXYCODONE HYDROCHLORIDE
OPRM1 and FENTANYL HYDROCHLORIDE
OPRM1 and DIPHENOXYLATE HYDROCHLORIDE
OPRM1 and LOPERAMIDE HYDROCHLORIDE
OPRM1 and BUPRENORPHINE HYDROCHLORIDE
OPRM1 and DIHYDROCODEINE BITARTRATE
OPRM1 and LEVORPHANOL TARTRATE
OPRM1 and METHADONE HYDROCHLORIDE
OPRM1 and TRAMADOL HYDROCHLORIDE
OPRM1 and OXYCODONE TEREPHTHALATE
OPRM1 and ANILERIDINE PHOSPHATE

OPRM1 and ALFENTANIL HYDROCHLORIDE
OPRM1 and PROPOXYPHENE NAPSYLATE
OPRM1 and BUTORPHANOL TARTRATE
OPRM1 and DSLET
OPRM1 and NALTRINDOLE
OPRM1 and NALTRIBEN
OPRM1 and CTAP
OPRM1 and CTOP
OPRM1 and DIPRENORPHINE
OPRM1 and NALORPHINE
OPRM1 and ETORPHINE
OPRM1 and (-)-CYCLAZOCINE
OPRM1 and NALMEFENE
OPRM1 and NALOXONE BENZOYLHYDRAZONE
OPRM1 and ELUXADOLINE
OPRM1 and DADLE
OPRM1 and (-)-PENTAZOCINE
OPRM1 and DYNORPHIN B
OPRM1 and ETONITAZENE
OPRM1 and MEPERIDINE
OPRM1 and DAMGO
OPRM1 and DIHYDROMORPHINE
OPRM1 and ETHYLKETOCYCLAZOCINE
OPRM1 and BNTX
OPRM1 and METHYLNALTREXONE
OPRM1 and QUADAZOCINE
OPRM1 and NORMORPHINE
OPRM1 and NICOTINE
OPRM1 and ETHANOL
OPRM1 and LEVACETYLMETHADOL
OPRM1 and DEXTROMETHORPHAN
OPRM1 and ONDANSETRON
OPRM1 and CARFENTANIL
OPRM1 and DIMETHYLTHIAMBUTENE
OPRM1 and KETAMINE
OPRM1 and DEXTROPROPOXYPHENE
OPRM1 and AMITRIPTYLINE
OPRM1 and NALOXEGOL
OPRM1 and ETHYLMORPHINE
OPRM1 and KETOBEMIDONE
NMNAT1 and NICOTINAMIDE MONONUCLEOTIDE

LEP and RISPERIDONE
LEP and OLANZAPINE
DAPK1 and 5,6-DIHYDRO-BENZO[H]CINNOLIN-3-YLAMINE
DAPK1 and DB04395
DAPK1 and 6-(3-AMINOPROPYL)-4,9-DIMETHYLPYRROLO[3,4-C]CARBAZOLE-1,3(2H,6H)-DIONE
HTR1E and RAUWOLSCINE
HTR1E and SERTINDOLE
HTR1E and METHYSERGIDE
HTR1E and METHIOTHEPIN
HTR1E and ZOLMITRIPTAN
HTR1E and TRYPTAMINE
HTR1E and SUMATRIPTAN
HTR1E and QUETIAPINE
HTR1E and ELETRIPTAN
HTR1E and METERGOLINE
HTR1E and METHYLERGONOVINE
HTR1E and 5-FLUOROTRYPTAMINE
HTR1E and CLOZAPINE
HTR1E and DIHYDROERGOTAMINE
HTR1E and DONITRIPTAN
HTR1E and EMDT
HTR1E and LYSERGOL
HTR1E and FLUSPIRILENE
HTR1E and RIZATRIPTAN
HTR1E and XANOMELINE
HTR1E and ZIPRASIDONE
HTR1E and ZOTEPINE
HTR1E and RISPERIDONE
HTR1E and YOHIMBINE
HTR1E and 8-OH-DPAT
HTR1E and ASENAPINE
HTR1E and ERGOTAMINE
HTR1E and LY344864
HTR1E and OLANZAPINE
HTR1E and [3H]5-HT
HTR1E and NARATRIPTAN
HTR1E and LOXAPINE
HTR1E and ARIPIPRAZOLE
CDH5 and LENALIDOMIDE
PREP and DB03382
PREP and DB07148

PREP and 1-HYDROXY-1-THIO-GLYCEROL
PREP and DOUBLE OXIDIZED CYSTEINE
PREP and Z-PRO-PROLINAL
GLUD2 and L-GLUTAMIC ACID
PTDSS2 and PHOSPHATIDYLSERINE
MTAP and ADENINE
MTAP and DB02158
PTK2 and MASITINIB
PTK2 and ENMD-2076
GCSH and DIHYDROLIPOIC ACID
GCSH and 6-(HYDROXYETHYLDITHIO)-8-(AMINOMETHYLTHIO)OCTANOIC ACID
GCSH and GLYCINE
UNG and 1-(2-DEOXY-5-O-PHOSPHONO-BETA-D-ERYTHRO-PENTOFURANOSYL)-4-METHYL-1H-INDOLE
UNG and 4-[(1E,7E)-8-(2,6-DIOXO-1,2,3,6-TETRAHYDROPYRIMIDIN-4-YL)-3,6-DIOXA-2,7-DIAZAOCTA-1,7 [illegible]
UNG and 3-[(1E,7E)-8-(2,6-DIOXO-1,2,3,6-TETRAHYDROPYRIMIDIN-4-YL)-3,6-DIOXA-2,7-DIAZAOCTA-1,7 [illegible]
GABRG1 and TEMAZEPAM
GABRG1 and DIAZEPAM
GABRG1 and TRIAZOLAM
GABRG1 and ESTAZOLAM
GABRG1 and CLONAZEPAM
GABRG1 and ADINAZOLAM
GABRG1 and HALAZEPAM
GABRG1 and ALPRAZOLAM
GABRG1 and LORAZEPAM
GABRG1 and BROMAZEPAM
GABRG1 and NITRAZEPAM
GABRG1 and CLOBAZAM
GABRG1 and MIDAZOLAM
GABRG1 and CINOLAZEPAM
GABRG1 and QUAZEPAM
GABRG1 and OXAZEPAM
GABRG1 and FLURAZEPAM
GABRG1 and PRAZEPAM
GABRG1 and CLORAZEPATE
GABRG1 and CHLORDIAZEPOXIDE
GABRG1 and CLOTIAZEPAM
GABRG1 and FLUDIAZEPAM
GABRG1 and PICROTOXIN
GABRG1 and TBPS
GABRG1 and GABOXADOL
GABRG1 and OCINAPLON

GABRG1 and ETAZOLATE
GABRG1 and KETAZOLAM
GABRG1 and ETHANOL
ACSL4 and ROSIGLITAZONE
ACSL4 and TROGLITAZONE
DAGLB and ORLISTAT
MDM2 and PEMETREXED
MDM2 and CISPLATINUM
MDM2 and CIS-[4,5-BIS-(4-BROMOPHENYL)-2-(2-ETHOXY-4-METHOXYPHENYL)-4,5-DIHYDROIMIDAZOL-
PSMA8 and CARFILZOMIB
PSMA8 and BORTEZOMIB
LIPF and ORLISTAT
LIPF and DB02457
NTRK3 and LESTAURTINIB
ALK and CRIZOTINIB
ALK and PEMETREXED
ALK and CERITINIB
ALK and PF-06463922
ALK and ASP3026
ALK and X-396
ALK and AP26113
ALK and AZD3463
ALK and ALECTINIB
ALK and CRENOLANIB
ALK and GANETESPIB
SLC7A11 and RILUZOLE
SLC7A11 and SULFASALAZINE
SLC7A11 and L-GLUTAMIC ACID
RARB and ALITRETINOIN
RARB and ADAPALENE
RARB and TAZAROTENE
RARB and TAMIBAROTENE
RARB and ISOTRETINOIN
RARB and BMS641
RARB and TTNPB
RARB and FENRETINIDE
RARB and ETRETINATE
RARB and ACITRETIN
GCLC and L-GLUTAMIC ACID
GCLC and L-CYSTEINE
PTEN and TEMSIROLIMUS

PTEN and PACLITAXEL
PTEN and EVEROLIMUS
PTEN and EGFR INHIBITOR
PTEN and CARBOPLATIN
PTEN and ERLOTINIB
PTEN and GSK2126458
PTEN and VANDETANIB
PTEN and ZSTK474
PTEN and GEFITINIB
PTEN and XL765
PTEN and PF-04691502
PTEN and GSK2636771
HPGDS and HQL-79
HPGDS and GLUTATHIONE
HPGDS and 4-{[4-(4-FLUORO-3-METHYLPHENYL)-1,3-THIAZOL-2-YL]AMINO}-2-HYDROXYBENZOIC ACID
HPGDS and 1-PHENYL-1H-PYRAZOLE-4-CARBOXYLIC ACID
HPGDS and 3-PHENYL-5-(1H-PYRAZOL-3-YL)ISOXAZOLE
HPGDS and NOCODAZOLE
HPGDS and 3-(4-NITROPHENYL)-1H-PYRAZOLE
HPGDS and GLYCEROL
GRIA3 and NBQX
GRIA3 and ATPO
GRIA3 and CYCLOTHIAZIDE
GRIA3 and TEZAMPANEL
GRIA3 and ANIRACETAM
GRIA3 and AMPA
GRIA3 and PIRACETAM
GRIA3 and PERAMPANEL
GRIA3 and TALAMPANEL
GRIA3 and L-GLUTAMIC ACID
GRIA3 and LITHIUM
GRIA3 and ETHANOL
CLK1 and DEBROMOHYMENIALDISINE
FAH and ACETOACETIC ACID
FAH and FUMARATE
ADRB1 and TIMOLOL
ADRB1 and BISOPROLOL
ADRB1 and ESMOLOL
ADRB1 and DESIPRAMINE
ADRB1 and ARBUTAMINE
ADRB1 and METOPROLOL

ADRB1 and ATENOLOL
ADRB1 and DOBUTAMINE
ADRB1 and PRACTOLOL
ADRB1 and OXPRENOLOL
ADRB1 and PINDOLOL
ADRB1 and DRONEDARONE
ADRB1 and NADOLOL
ADRB1 and NEBIVOLOL
ADRB1 and BEVANTOLOL
ADRB1 and METIPRANOLOL
ADRB1 and BETAXOLOL
ADRB1 and PENBUTOLOL
ADRB1 and AMIODARONE
ADRB1 and ALPRENOLOL
ADRB1 and ISOPROTERENOL
ADRB1 and ISOETHARINE
ADRB1 and ZIPRASIDONE
ADRB1 and CARVEDILOL
ADRB1 and LEVOBUNOLOL
ADRB1 and PROPRANOLOL
ADRB1 and LABETALOL
ADRB1 and EPINEPHRINE
ADRB1 and CARTEOLOL
ADRB1 and ACEBUTOLOL
ADRB1 and ACEBUTOLOL HYDROCHLORIDE
ADRB1 and ESMOLOL HYDROCHLORIDE
ADRB1 and OXPRENOLOL HYDROCHLORIDE
ADRB1 and DOPAMINE HYDROCHLORIDE
ADRB1 and PROPRANOLOL HYDROCHLORIDE
ADRB1 and PENBUTOLOL SULFATE
ADRB1 and BETAXOLOL HYDROCHLORIDE
ADRB1 and NEBIVOLOL HYDROCHLORIDE
ADRB1 and METIPRANOLOL HYDROCHLORIDE
ADRB1 and DOBUTAMINE HYDROCHLORIDE
ADRB1 and SOTALOL HYDROCHLORIDE
ADRB1 and METOPROLOL FUMARATE
ADRB1 and METOPROLOL SUCCINATE
ADRB1 and PROPAFENONE HYDROCHLORIDE
ADRB1 and TIMOLOL MALEATE
ADRB1 and BISOPROLOL FUMARATE
ADRB1 and INDACATEROL

ADRB1 and SOTALOL
ADRB1 and CICLOPROLOL
ADRB1 and SR59230A
ADRB1 and DENOPAMINE
ADRB1 and BUPRANOLOL
ADRB1 and ARFORMOTEROL
ADRB1 and XAMOTEROL
ADRB1 and NIHP
ADRB1 and PROPAFENONE
ADRB1 and MIRABEGRON
ADRB1 and PRENALTEROL
ADRB1 and BUCINDOLOL
ADRB1 and MURAGLITAZAR
ADRB1 and NOREPINEPHRINE
ADRB1 and LEVOBETAXOLOL
ADRB1 and TRIMIPRAMINE
ADRB1 and AMITRIPTYLINE
ADRB1 and AMPHETAMINE
ADRB1 and NORTRIPTYLINE
ADRB1 and OLANZAPINE
ADRB1 and BUFURALOL
ADRB1 and MEPHENTERMINE
ADRB1 and FENOTEROL
ADRB1 and DB08347
ADRB1 and BOPINDOLOL
ADRB1 and CLENBUTEROL
ADRB1 and PIRBUTEROL
ADRB1 and LOXAPINE
ADRB1 and CABERGOLINE
ADRB1 and PSEUDOEPHEDRINE
ADRB1 and PHENYLPROPANOLAMINE
ADRB1 and MIRTAZAPINE
ADRB1 and DROXIDOPA
ADRB1 and ASENAPINE
ADRB1 and SALBUTAMOL
PRKACB and PHOSPHONOTHREONINE
SLCO1B3 and CYCLOSPORIN A
SLCO1B3 and GLYCYRRHIZIN
SLCO1B3 and SILDENAFIL
SLCO1B3 and GEMFIBROZIL
SLCO1B3 and RIFAMPICIN

SLCO1B3 and DOCETAXEL
SLCO1B3 and MYCOPHENOLATE MOFETIL
PTPRE and ALENDRONATE
MAPK10 and JNK INHIBITOR IX
MAPK10 and JNK INHIBITOR VIII
MAPK10 and JNK INHIBITOR V
MAPK10 and N-BENZYL-4-[4-(3-CHLOROPHENYL)-1H-PYRAZOL-3-YL]-1H-PYRROLE-2-CARBOXAMIDE
MAPK10 and DB04395
MAPK10 and 9-(4-HYDROXYPHENYL)-2,7-PHENANTHROLINE
KAT2B and ANACARDIC ACID
KAT2B and GARCINOL
KAT2B and LYS-COA
KAT2B and PLUMBAGIN
KAT2B and COENZYME A
KAT2B and (3E)-4-(1-METHYL-1H-INDOL-3-YL)BUT-3-EN-2-ONE
KAT2B and N-(3-AMINOPROPYL)-2-NITROBENZENAMINE
SMARCA5 and BETA-D-GLUCOSE
SMARCA5 and 4-DEOXY-ALPHA-D-GLUCOSE
GADL1 and PYRIDOXAL PHOSPHATE
KCNN3 and LEI-DAB7
KCNN3 and TUBOCURARINE
KCNN3 and DEQUALINIUM
KCNN3 and APAMIN
KCNN3 and CYPPA
KCNN3 and MICONAZOLE
EN2 and N-CYCLOHEXYLTAURINE
CCND1 and TAMOXIFEN
CCND1 and LEE011
CCND1 and LY2835219
PARS2 and L-PROLINE
P4HA2 and SUCCINIC ACID
P4HA2 and L-PROLINE
DLAT and DIHYDROLIPOIC ACID
MERTK and AT9283
PI4KB and PIK-93
PI4KB and WORTMANNIN
SLC36A1 and 5-HYDROXY-L-TRYPTOPHAN
SLC36A1 and L-TRYPTOPHAN
SLC36A1 and L-ALANINE
SLC36A1 and GLYCINE
PRKD1 and QUERCETIN

PRKD1 and BRYOSTATIN
PRKD1 and BRYOSTATIN-1

IIDE

ETHOXYPROPANOIC ACID

-METHYL-1,3-HEXADIYNYL)-7A-METHYL-4H-INDEN-4-YLIDENE]ETHYLIDENE]-, (1R,3S,5Z)

YL]THIOPHEN-2-YL}ETHANE-1,1-DIOL
PHENE-2-CARBOXAMIDE

.]BENZAMIDE
2-OXOETHYL]MORPHOLINE-4-CARBOXAMIDE
JORO-1-(4-FLUOROPHENYL)ETHYL]-L-ALANINAMIDE
2,2,2-TRIFLUORO-1-(4-FLUOROPHENYL)ETHYL]-L-ALANINAMIDE
-HYDROXYPHENYL)ETHYL]-L-ALANINAMIDE
OXOETHYL MORPHOLINE-4-CARBOXYLATE

'HENYL-ETHYL]-AMIDE

ETIC ACID

-DIEN-1-YL]BENZOIC ACID
-DIEN-1-YL]BENZOIC ACID

1-YL]-[4-(2-HYDROXYETHYL)PIPERAZIN-1-YL]METHANONE
