# Supplementary material for: An imprinted non-coding genomic cluster at 14q32 defines clinically relevant molecular subtypes in osteosarcoma across multiple independent datasets
Source: J Hematol Oncol. 2017 May 15;10:107. doi: 10.1186/s13045-017-0465-4 (PMC5433149; doi:10.1186/s13045-017-0465-4)
Supplement: Supplementary file 3 — Differentially expressed miRNAs between the two subtypes in the three datasets. The Boston, Utah, and cell line data were analyzed to determine the differential expression of their miRNA population. (PDF 154 kb) [file 13045_2017_465_MOESM3_ESM.pdf]

Supplementary methods (PUMA)

Reconstruction of condition-specific osteosarcoma regulatory networks

We used PUMA (Panda Using MicroRNA Associations, github.com/mararie/PUMA) to reconstruct gene regulatory networks. PUMA is an extension of PANDA[1], a message passing algorithm that integrates a regulatory "prior" with protein-protein interaction data and gene expression data to model condition-specific gene regulatory networks. PUMA integrates a regulatory prior that consists of two different types of regulators: transcription factors (TFs) and microRNAs (miRNAs). We prepared the PUMA input data as follows:

Regulatory prior: We built a gene regulatory prior using both TF binding and miRNA target prediction data. We downloaded 116 core human TF binding site motifs from JASPAR[2,3] (developmental server, http://jaspar.binf.ku.dk/, version: 2014, accession date: July 2013). We mapped position weight matrices (PWM) to promoter regions ([-750, +250] kb from transcription start sites) using Bioconductor packages *BioStrings*[4] (version 2.28.0) and *GenomicFeatures*[5] (version 1.12.4). A minimum score of 90% of the highest possible match score was considered a match. We assessed the significance of these matches by comparing them to matches of the same set motifs to a background of 10,000 random promoter sequences using the one-sided *pnorm* function in R, and corrected for multiple testing using Benjamini and Hochberg's FDR[6]. Entries with FDR<0.05 were considered significant. We converted promoters to gene names and selected motif-gene pairs as an edge in the regulatory prior if the PWM had a significant match to at least one promoter of the target gene. We downloaded miRNA target prediction data from TargetScan[7] (http://www.targetscan.org/, file: "Summary_Counts.txt", accession date: November 2013) and selected Homo Sapiens miRNAs-target gene pairs with context+ scores[7] < -0.1 as edges in the regulatory prior. For genes with multiple transcripts, the minimum context+ score was used.

Protein-protein interaction data: We downloaded protein-protein interactions (PPIs) and their annotations from StringDb[8] (files: "protein.links.detailed.v9.05.txt.gz" and "protein.aliases.v9.05.txt.gz", accession date: October 2013) and selected all Homo Sapiens interactions. We converted StringDb identifiers to gene symbols using the annotation file and Bioconductor package *org.Hs.eg.db*[9] (version 2.10.1). Finally, we converted gene symbols to JASPAR motif identifiers. For JASPAR PWMs that represented a TF complex, we converted all genes in the complex to the corresponding JASPAR motif identifier. We selected the maximum StringDb score for duplicate interactions. We divided the StringDb PPI scores by 1,000 to have scores ranging from 0-1.

Expression data: Normalized high-grade osteosarcoma expression data of 37 pre-treatment biopsies[10] were downloaded from the Gene Expression Omnibus (GEO accession: GSE39055, accessed: June 2014). To make the data compatible with our motif prior, we converted Illumina transcript identifiers to gene symbols and collapsed transcripts encoding for the same genes by selecting those transcripts with maximum variance across all samples. We used mRNA expression data from samples belonging to the high or low risk subtypes to reconstruct subtype-specific networks. As a control, we also reconstructed networks on samples from patients who had good and poor response to chemotherapy (high and low necrosis).

We pruned the motif prior and gene expression data to contain only those genes that were present in both data types (n=17,924) and removed two TFs that did not have any motif hit in the promoters of this set of genes. Finally, we only used miRNA regulators for which we had miRNA expression data available, which reduced the number of regulators to 750 (114 TFs, 636 miRNAs).

Reconstruction of background networks

We reconstructed 25 background regulatory networks by randomly selecting *N* samples from the expression data using the “-j” option in PUMA, with *N* being either 15, 18, 19, or 22 (the same sample sizes as the data on which the condition-specific networks were built). We then applied PUMA to these 25 permuted expression datasets, using the same regulatory and PPI priors as described above.

Identification of subtype-specific differences in osteosarcoma

We calculated miRNA out-degrees by summing up all edge z-scores connected to a miRNA (PANDA/PUMA networks are complete, weighted networks). Out-degree differences were visualized on a z-score scale using the function *heatmap.2* from R package *gplots* (version 2.17.0). For each of the 5 miRNAs of interest, we calculated the edge weight differences between the high and low risk subtype networks, and selected those edges that had a z-score > 0.8 in either one or both of the subtype-specific networks (3,450 edges). We compared the z-score differences between the high and low risk networks to the distribution of edges in the background networks using the *pnorm* function in R to obtain a p-value for each edge, which we subsequently corrected for multiple testing using Benjamini & Hochberg's FDR[6]. We selected edges with FDR<0.25, which resulted in a network module consisting of 5 miRNAs, 637 genes, and 652 edges. We performed GO-term enrichment analysis on the 637 genes from this subnetwork using the *classical* test statistic from *topGO*[11] (version 2.22.0). We visualized the network module using Cytoscape[12] (version 3.3.0), using an edge weighted spring-embedded layout on edge betweenness. Edges were colored based on edge difference, and edge thickness corresponded to the negative log FDRs. The top 20 edges for the 2 most connected miRNAs from this network module were visualized using the circular y-files layout in Cytoscape. Edges were colored with the same color scale settings as we used for visualizing the complete network module.

References

1. Glass, K., Huttenhower, C., Quackenbush, J. & Yuan, G.-C. Passing messages between biological networks to refine predicted interactions. *PloS One* **8,** e64832 (2013).
2. Sandelin, A., Alkema, W., Engström, P., Wasserman, W. W. & Lenhard, B. JASPAR: an open-access database for eukaryotic transcription factor binding profiles. *Nucleic Acids Res.* **32,** D91–94 (2004).
3. Mathelier, A. *et al.* JASPAR 2014: an extensively expanded and updated open-access database of transcription factor binding profiles. *Nucleic Acids Res.* (2013). doi:10.1093/nar/gkt997
4. Pages H, Aboyoun P, Gentleman R & DebRoy S. Biostrings: String objects representing biological sequences, and matching algorithms.
5. Lawrence, M. *et al.* Software for computing and annotating genomic ranges. *PLoS Comput. Biol.* **9,** e1003118 (2013).
6. Benjamini, Y. & Hochberg, Y. Controlling the False Discovery Rate: A Practical and Powerful Approach to Multiple Testing. *J R Stat Soc Ser B* 289–300 (1995).
7. Garcia, D. M. *et al.* Weak seed-pairing stability and high target-site abundance decrease the proficiency of lsy-6 and other microRNAs. *Nat. Struct. Mol. Biol.* **18,** 1139–1146 (2011).
8. Jensen, L. J. *et al.* STRING 8--a global view on proteins and their functional interactions in 630 organisms. *Nucleic Acids Res.* **37,** D412–416 (2009).
9. Carlson, M. *org.Hs.eg.db: Genome wide annotation for Human*.
10. Kelly, A. D. *et al.* MicroRNA paraffin-based studies in osteosarcoma reveal reproducible independent prognostic profiles at 14q32. *Genome Med.* **5,** 2 (2013).
11. Alexa, A., Rahnenführer, J. & Lengauer, T. Improved scoring of functional groups from gene expression data by decorrelating GO graph structure. *Bioinforma. Oxf. Engl.* **22,** 1600–1607 (2006).
12. Shannon, P. *et al.* Cytoscape: a software environment for integrated models of biomolecular interaction networks. *Genome Res.* **13,** 2498–2504 (2003).
