# Supplementary figures and images for: An imprinted non-coding genomic cluster at 14q32 defines clinically relevant molecular subtypes in osteosarcoma across multiple independent datasets
Source: J Hematol Oncol. 2017 May 15;10:107. doi: 10.1186/s13045-017-0465-4 (PMC5433149; doi:10.1186/s13045-017-0465-4)

a. 5 miRNA profile

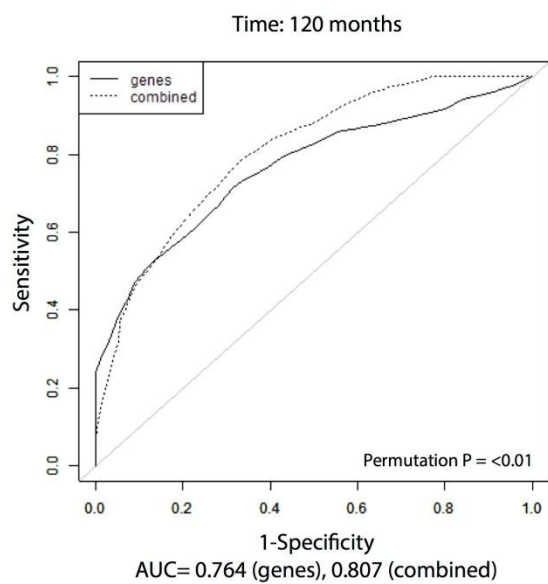

b. 18 miRNA profile

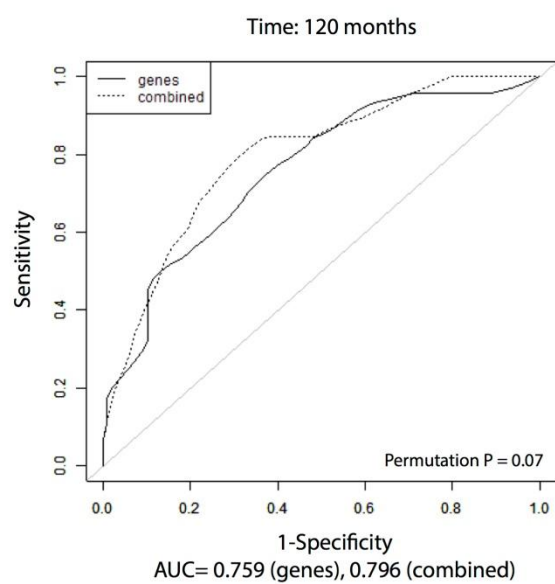

Supplement: Supplementary file 4 — Correlations between DICER and differentially expressed miRNAs in the Boston dataset. DICER1 is a gene that encodes for an endoribonuclease essential for the formation of microRNA, and it is also located on 14q32. We analyzed the correlation between miRNA expression on the 14q32 locus and DICER1 expression and found that a small fraction (5%) of miRNAs was significantly correlated with DICER1. (PDF 174 kb) [file 13045_2017_465_MOESM4_ESM.pdf]

Boston Dataset Whole Genome Hierarchical Clustering

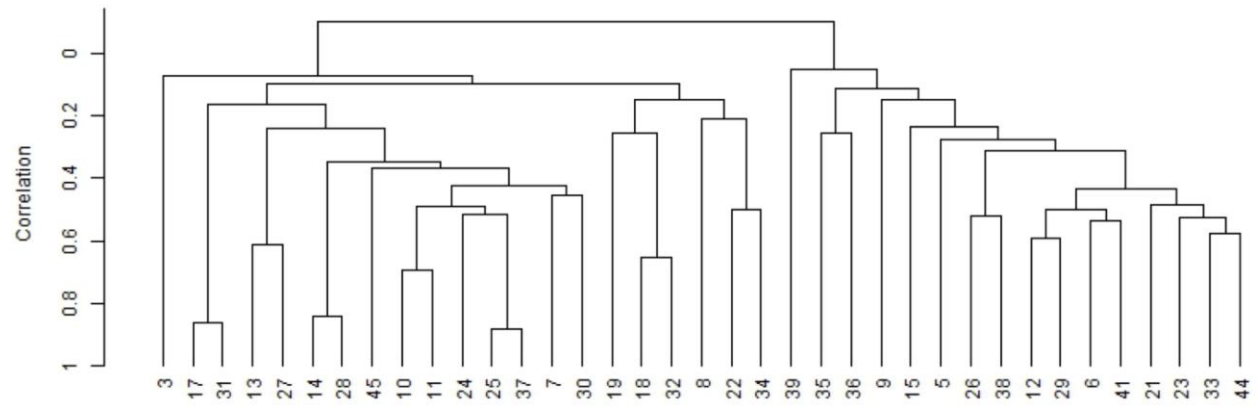

Supplement: Supplementary file 5 — Boston dataset hierarchical clustering using the messenger RNA (whole-genome) profiles. (Based on data available for a subset of 37 patients only). (PDF 20 kb) [file 13045_2017_465_MOESM5_ESM.pdf]
