# Supplementary material for: An imprinted non-coding genomic cluster at 14q32 defines clinically relevant molecular subtypes in osteosarcoma across multiple independent datasets
Source: J Hematol Oncol. 2017 May 15;10:107. doi: 10.1186/s13045-017-0465-4 (PMC5433149; doi:10.1186/s13045-017-0465-4)
Supplement: Supplementary file 6 — Correlations between prognostic miRNAs and other 14q32 transcripts in the Boston dataset. Eighteen prognostic miRNAs that we found on 14q32 were compared with the remaining coding and non-coding transcripts on that same locus to investigate whether a larger pattern of co-regulation may exist. (PDF 424 kb) [file 13045_2017_465_MOESM6_ESM.pdf]

ADDITIONAL METHODS DESCRIPTION

Taqman RT-PCR data pre processing: MicroRNA profiling using qRT PCR yields delta Ct (cycle threshold) values that represent gene expression. A higher delta Ct value means lower gene expression. We devised an approach to invert the relationship between Ct values and gene expression in order to produce more intuitive analysis results. All of delta Ct values were multiplied by -1, making the largest values the smallest. Then the sum of the largest and the smallest delta Ct values within the original data matrix were added to the new inverted delta Ct matrix. This approach preserved the relative relationship amongst the values, while effectively inverting the relationship between the delta Ct value and gene expression to a more intuitive positive correlation. This approach was applied to both the TARGET and Texas TaqMan miRNA PCR datasets.

Composite aggressiveness index including migration/invasion/colony forming capacity in the osteosarcoma cell line dataset.

Data on cell line aggressiveness including metrics for proliferation, migration, invasion, and colony formation were previously generated by Lauvrak et al, and we obtained them from the publicly available website related to their publication (*Br J Cancer* 2013, 109(8):2228-2236.). In that analysis, the cell lines were grouped in categories 1 to 4, with respect to all these in vitro aggressiveness attributes. We then used these pre-defined categories to generate a “composite index” including migration/invasion/colony formation, and considered this a possible suggorate for metastatic potential. In combining these categories, we considered categories 3 and 4 to

represent “high aggressiveness” and categories 1 and 2 to represent “low aggressiveness” in keeping with the ranking by Lauvrak et al. In the case where a cell line displayed heterogeneous rankings for the three different in vitro attributes, we assigned this cell line to the category suggested by two of the three rankings. For example, if the cell line was ranked as “high aggressiveness” for invasion/migration but “low aggressiveness” for colony formation, we considered it to be more likely “highly aggressive” as this categorization was supported by two of the three attributes.
