# Supplementary material for: An imprinted non-coding genomic cluster at 14q32 defines clinically relevant molecular subtypes in osteosarcoma across multiple independent datasets
Source: J Hematol Oncol. 2017 May 15;10:107. doi: 10.1186/s13045-017-0465-4 (PMC5433149; doi:10.1186/s13045-017-0465-4)
Supplement: Supplementary file 7 — Association between prognostic 14q32 miRNAs and cell line aggressiveness (binary analysis). An analysis between 14q32 miRNA expression and the following characteristics associated with cell line aggressiveness was performed: proliferation, invasiveness, migration, colony forming, and tumorigenicity. These attributes were analyzed as binary (categorical) variables. (PDF 538 kb) [file 13045_2017_465_MOESM7_ESM.pdf]

Differentially expressed miRNAs between the two subtypes

Parametric p-value	FDR	Fold-change	miRNA ID
1.40E-05	9.50E-05	0.25	hsa-miR-938
2.70E-06	3.05E-05	0.28	HS_175
1.30E-06	1.81E-05	0.31	hsa-miR-638
< 1e-07	< 1e-07	0.32	hsa-miR-1268
< 1e-07	< 1e-07	0.32	HS_167.1
8.00E-07	1.33E-05	0.32	HS_111
6.20E-06	5.28E-05	0.32	hsa-miR-202*:9.1
< 1e-07	< 1e-07	0.33	solexa-539-2056
4.60E-06	4.30E-05	0.34	hsa-miR-1238
1.00E-07	3.07E-06	0.35	solexa-9081-91
1.10E-06	1.62E-05	0.35	HS_228.1
7.50E-06	5.99E-05	0.36	hsa-miR-550*
8.29E-05	0.000352	0.36	HS_12
7.10E-06	5.79E-05	0.37	HS_126
1.00E-07	3.07E-06	0.38	hsa-miR-324-5p
7.00E-07	1.22E-05	0.38	hsa-miR-623
1.10E-06	1.62E-05	0.38	hsa-miR-149*
6.30E-06	5.31E-05	0.38	HS_3
5.18E-05	0.000251	0.38	HS_170
2.00E-06	2.43E-05	0.39	HS_200
2.10E-06	2.52E-05	0.39	HS_242
0.0005417	0.0016	0.39	HS_76
< 1e-07	< 1e-07	0.4	hsa-miR-1183
5.00E-07	1.07E-05	0.4	HS_217
6.00E-07	1.15E-05	0.4	HS_142.1
1.00E-06	1.60E-05	0.41	hsa-miR-566
1.02E-05	7.67E-05	0.41	hsa-miR-7-1*
2.65E-05	0.000147	0.41	hsa-miR-885-3p
4.12E-05	0.000215	0.41	HS_240
5.70E-05	0.00027	0.41	HS_64

Boston Dataset	
Downregulated	235
Upregulated	257

2.40E-06	2.75E-05	0.42	HS_147
2.59E-05	0.000146	0.42	hsa-miR-1225-5p
2.00E-06	2.43E-05	0.43	hsa-miR-1197
1.68E-05	0.000109	0.43	HS_48.1
0.0005446	0.0016	0.43	HS_72
< 1e-07	< 1e-07	0.44	solexa-9655-85
4.36E-05	0.000224	0.44	HS_196.1
0.0002813	0.000926	0.44	hsa-miR-380
1.00E-07	3.07E-06	0.45	hsa-miR-612
1.50E-06	2.02E-05	0.45	HS_284.1
3.60E-06	3.68E-05	0.45	HS_283_a
1.29E-05	9.06E-05	0.45	hsa-miR-302d
1.73E-05	0.000112	0.45	HS_56
2.49E-05	0.000143	0.45	hsa-miR-622
5.95E-05	0.000277	0.45	HS_135
0.0002207	0.000759	0.45	HS_112
4.20E-06	4.08E-05	0.46	HS_182.1
9.25E-05	0.000381	0.46	hsa-miR-1237
7.00E-07	1.22E-05	0.47	hsa-miR-1304
1.10E-05	8.04E-05	0.47	hsa-miR-375
0.0011956	0.00316	0.47	hsa-miR-122
4.00E-07	9.02E-06	0.49	hsa-miR-944
2.18E-05	0.000133	0.49	hsa-miR-1224-3p
6.16E-05	0.000283	0.49	HS_188
0.0001024	0.000403	0.49	HS_31.1
0.0007353	0.00205	0.5	hsa-miR-129-3p
1.00E-07	3.07E-06	0.51	HS_276.1
4.50E-06	4.26E-05	0.51	hsa-miR-632
2.48E-05	0.000143	0.51	HS_55
8.35E-05	0.000352	0.51	HS_17
2.00E-06	2.43E-05	0.52	hsa-miR-187*

4.10E-06	4.03E-05	0.52	hsa-miR-518f
1.83E-05	0.000116	0.52	hsa-miR-654-5p
6.75E-05	0.000303	0.52	hsa-miR-193b*
0.0022844	0.00534	0.52	hsa-miR-576-3p
1.40E-06	1.92E-05	0.53	hsa-miR-1182
2.13E-05	0.000132	0.53	hsa-miR-520a-5p
2.13E-05	0.000132	0.53	HS_232
0.0001223	0.000467	0.53	hsa-miR-1236
1.44E-05	9.69E-05	0.54	HS_269
2.26E-05	0.000134	0.54	HS_53
4.49E-05	0.000229	0.54	HS_74
7.49E-05	0.000326	0.54	hsa-miR-521
0.000422	0.00128	0.54	HS_2
0.0006574	0.00188	0.54	HS_257
0.0016291	0.00406	0.54	hsa-miR-572
0.0016295	0.00406	0.54	hsa-miR-668
4.77E-05	0.000236	0.55	hsa-miR-1181
0.0002036	0.000713	0.55	hsa-miR-1307
0.0002377	0.000803	0.55	HS_221
0.0004026	0.00123	0.55	HS_19
0.0004647	0.0014	0.55	hsa-miR-326
0.0015612	0.00399	0.55	HS_116
4.53E-05	0.000229	0.56	hsa-miR-1229
9.87E-05	0.000396	0.56	hsa-miR-129*
0.0001064	0.000416	0.56	hsa-miR-1224-5p
0.0002444	0.000822	0.56	HS_55.1
0.0003721	0.00116	0.56	HS_198
1.60E-06	2.12E-05	0.57	HS_65
1.32E-05	9.12E-05	0.57	hsa-miR-423-5p
2.08E-05	0.000131	0.57	hsa-miR-488*
4.05E-05	0.000213	0.57	HS_166.1

7.63E-05	0.00033	0.57	HS_105
0.0050508	0.0104	0.57	hsa-miR-654-3p
1.10E-06	1.62E-05	0.58	HS_155
8.87E-05	0.00037	0.58	HS_149
0.0005265	0.00157	0.58	hsa-miR-877*
0.0009447	0.00257	0.58	hsa-miR-518e
0.0017808	0.00436	0.58	HS_163
0.0025943	0.00597	0.58	hsa-miR-218-2*
3.00E-06	3.24E-05	0.59	solexa-8926-93
7.06E-05	0.000315	0.59	hsa-miR-92b*
8.95E-05	0.000371	0.59	hsa-miR-520e
0.0087981	0.0169	0.59	hsa-miR-504
6.49E-05	0.000293	0.6	hsa-miR-569
7.40E-05	0.000325	0.6	hsa-miR-1249
0.0029835	0.00667	0.6	HS_78
1.00E-07	3.07E-06	0.61	hsa-miR-302b*
2.41E-05	0.00014	0.61	hsa-miR-1184
9.73E-05	0.000393	0.61	HS_18
0.0011194	0.00298	0.61	HS_287
4.66E-05	0.000232	0.62	hsa-miR-1281
0.0002519	0.000844	0.62	hsa-miR-1825
0.0007747	0.00215	0.62	hsa-miR-124a:9.1
0.0009255	0.00253	0.62	HS_6
0.0015518	0.00399	0.62	HS_29
0.0001717	0.000621	0.63	hsa-miR-595
0.0005131	0.00153	0.63	hsa-miR-18b*
0.0079586	0.0154	0.63	HS_176
5.54E-05	0.000264	0.64	hsa-miR-512-5p
0.0001707	0.000621	0.64	HS_32
0.0003093	0.000993	0.64	HS_120
0.0062251	0.0125	0.64	HS_250

0.0154746	0.0274	0.64	hsa-miR-205
1.90E-06	2.43E-05	0.65	hsa-miR-551a
8.20E-06	6.48E-05	0.65	hsa-miR-637
9.40E-06	7.28E-05	0.65	hsa-miR-544
1.15E-05	8.24E-05	0.65	hsa-miR-1179
0.0003053	0.000984	0.65	HS_22.1
0.0039435	0.0085	0.65	hsa-miR-346
0.0251101	0.0426	0.65	hsa-miR-647
0.0290518	0.0482	0.65	HS_254
0.0001085	0.000422	0.66	hsa-miR-636
0.0005818	0.00169	0.66	solexa-1460-671
0.0009928	0.00268	0.66	HS_50
0.0016056	0.00406	0.66	HS_38.1
0.0022215	0.00523	0.66	hsa-miR-1295
0.0026759	0.00609	0.66	hsa-miR-518e*,hsa-miR-519a*,hsa-miR-519b-5p,hsa-miR-519c-5p,hsa-miR-522*,hsa-miR-523*
0.0055789	0.0114	0.66	HS_202.1
0.000357	0.00112	0.67	HS_243.1
0.0031461	0.00699	0.67	hsa-miR-296-3p
0.0064319	0.0128	0.67	hsa-miR-663
0.0019134	0.00462	0.68	HS_128
0.0053729	0.011	0.68	HS_132.1
0.0161156	0.0282	0.68	hsa-miR-936
2.99E-05	0.000163	0.69	HS_256
0.000286	0.000933	0.69	solexa-6676-127
0.0003177	0.00101	0.69	HS_280_b
0.0022054	0.0052	0.69	hsa-miR-1225-3p
0.0046434	0.00976	0.69	HS_5.1
0.0124884	0.0229	0.69	hsa-miR-1275
6.34E-05	0.000288	0.7	hsa-miR-1228*
0.0003511	0.0011	0.7	hsa-miR-1293
0.0027969	0.00633	0.7	HS_11.1

0.0129801	0.0236	0.7	hsa-miR-509-3p
1.30E-05	9.06E-05	0.71	HS_278
3.63E-05	0.000193	0.71	solexa-9578-86
0.0003029	0.00098	0.71	hsa-miR-1180
0.0003627	0.00113	0.71	HS_150
0.0079515	0.0154	0.71	HS_169
5.00E-07	1.07E-05	0.72	HS_261.1
0.0001625	6.00E-04	0.72	hsa-miR-1233
0.0006605	0.00188	0.72	HS_255
0.0015097	0.00391	0.72	solexa-7534-111
0.0044989	0.00953	0.72	hsa-miR-1207-5p
0.00455	0.00961	0.72	solexa-3044-295
0.0063665	0.0127	0.72	hsa-miR-509-5p
0.002668	0.00609	0.73	hsa-miR-940
0.0195853	0.0337	0.73	hsa-miR-665
4.89E-05	0.000239	0.74	HS_117
0.0003832	0.00118	0.74	HS_101
0.0012352	0.00326	0.74	hsa-miR-639
0.0026544	0.00608	0.74	hsa-miR-1207-3p
5.45E-05	0.000261	0.75	hsa-miR-631
0.0003014	0.00098	0.75	hsa-miR-498
0.0016259	0.00406	0.75	HS_157
0.0018791	0.00456	0.75	HS_194
0.0034129	0.00752	0.75	HS_280_a
4.02E-05	0.000213	0.76	HS_136
0.0005848	0.00169	0.76	hsa-miR-520a-3p
0.0025707	0.00594	0.76	hsa-miR-646
0.0028994	0.00652	0.76	HS_14.1
0.0039988	0.00859	0.76	hsa-miR-220a
0.0047118	0.00985	0.76	hsa-miR-337:9.1
0.0096627	0.0183	0.76	HS_77

0.0326623	0.0533	0.76	HS_140
5.70E-06	4.97E-05	0.77	HS_190
4.56E-05	0.000229	0.77	HS_8
0.0001673	0.000614	0.77	HS_7
0.0014614	0.0038	0.77	hsa-miR-518b
0.0169216	0.0294	0.77	HS_139
0.0001628	6.00E-04	0.78	HS_154
0.0010147	0.00272	0.78	HS_244
0.0031736	0.00704	0.78	solexa-7297-115
0.0062432	0.0125	0.78	HS_110
0.0108402	0.0203	0.78	hsa-miR-518c
0.0145534	0.0261	0.78	hsa-miR-554
0.0147639	0.0264	0.78	hsa-miR-548n
0.0334095	0.0541	0.78	HS_54
0.0002363	0.000802	0.79	hsa-miR-583
0.0015571	0.00399	0.79	HS_159
0.0048773	0.0101	0.79	hsa-miR-650
0.0137997	0.025	0.79	hsa-miR-564
0.0151314	0.0269	0.79	hsa-miR-1468
0.0158182	0.028	0.79	solexa-8048-104
0.0165591	0.0289	0.79	HS_58
0.0026014	0.00597	0.8	hsa-miR-219-1-3p
0.0003184	0.00101	0.81	solexa-9029-92
0.0022613	0.0053	0.81	hsa-miR-330-5p
0.0026987	0.00612	0.81	hsa-miR-923
0.0030553	0.00681	0.81	HS_62
0.0075123	0.0148	0.81	solexa-7509-112
0.026458	0.0444	0.81	HS_86
0.0018881	0.00457	0.82	hsa-miR-620
0.0019558	0.00469	0.82	HS_52
0.0045872	0.00967	0.82	hsa-miR-183*

0.0049038	0.0102	0.82	hsa-miR-380*
0.0053392	0.0109	0.82	HS_195
0.0078054	0.0152	0.82	hsa-miR-448
0.0127801	0.0233	0.82	HS_10
0.0252499	0.0427	0.82	HS_25
0.0077466	0.0152	0.84	HS_51
0.010913	0.0204	0.84	hsa-miR-490-5p
0.0285989	0.0477	0.84	hsa-miR-657
0.038647	0.0619	0.84	HS_174.1
0.0112023	0.0209	0.85	hsa-miR-1178
0.0159676	0.0282	0.85	hsa-miR-588
0.0315107	0.0519	0.85	hsa-miR-1282
0.0326716	0.0533	0.85	hsa-miR-616
0.0137875	0.025	0.86	HS_89
0.0186147	0.0321	0.86	hsa-miR-1254
0.0227243	0.0388	0.87	hsa-miR-300
0.0248085	0.0422	0.87	hsa-miR-371-5p
0.0317123	0.0521	0.87	HS_75.1
0.048073	0.0754	0.88	HS_263.1
0.0459416	0.0725	1.14	hsa-miR-330-3p
0.0363113	0.0583	1.15	hsa-miR-629*
0.0485001	0.0758	1.15	hsa-miR-483-5p
0.0247724	0.0422	1.16	hsa-miR-23b
0.0314056	0.0518	1.16	hsa-miR-643
0.0489658	0.0763	1.16	hsa-miR-34b
0.0069502	0.0138	1.18	HS_199
0.0324621	0.0532	1.18	hsa-let-7e
0.002962	0.00664	1.2	hsa-let-7i
0.0252715	0.0427	1.2	hsa-miR-136*
0.0265833	0.0445	1.2	hsa-miR-190b
0.0347743	0.056	1.2	hsa-miR-126*

0.0398177	0.0636	1.2	hsa-miR-579
0.0034844	0.00764	1.21	hsa-miR-23b*
0.0082134	0.0159	1.22	hsa-miR-616*
0.0102194	0.0194	1.22	hsa-miR-93*
0.0440583	0.07	1.22	hsa-miR-664*
0.0011775	0.00313	1.23	hsa-miR-30d
0.0017177	0.00424	1.23	hsa-miR-372
0.0052613	0.0108	1.23	hsa-miR-548d-5p
0.0160375	0.0282	1.23	hsa-miR-374a
0.0330901	0.0538	1.23	hsa-miR-30e
0.0007214	0.00203	1.24	hsa-miR-23a
0.0059967	0.0121	1.24	hsa-miR-130a
0.008884	0.017	1.24	hsa-let-7f
0.0183406	0.0318	1.24	hsa-miR-20a
0.0417367	0.0664	1.24	hsa-miR-17
0.0006142	0.00176	1.25	hsa-miR-195*
0.0009109	0.0025	1.25	hsa-miR-27b*
0.0034559	0.0076	1.25	hsa-miR-30c
0.0059758	0.0121	1.25	hsa-miR-582-5p
0.0073172	0.0145	1.25	hsa-miR-214
0.0181677	0.0315	1.25	hsa-miR-766
0.0480948	0.0754	1.25	hsa-miR-137
0.010346	0.0195	1.26	hsa-miR-191
0.034172	0.0552	1.26	hsa-miR-20a*
0.0444499	0.0704	1.26	hsa-miR-26a-2*
5.83E-05	0.000273	1.27	hsa-miR-337-5p
0.0087946	0.0169	1.27	hsa-miR-221
0.0091439	0.0174	1.27	hsa-miR-424
0.0292598	0.0485	1.27	hsa-miR-204
0.0449793	0.0711	1.27	hsa-miR-628-3p
0.0003467	0.00109	1.28	hsa-miR-199a-3p,hsa-miR-199b-3p

0.0023327	0.00544	1.28	hsa-miR-30e*
0.0226481	0.0388	1.28	hsa-miR-190
1.05E-05	7.74E-05	1.29	hsa-miR-411*
0.0112665	0.0209	1.29	hsa-miR-138-1*
0.0306504	0.0507	1.29	hsa-miR-193a-5p
0.0330645	0.0538	1.29	hsa-miR-188-5p
0.003694	0.00805	1.3	hsa-miR-196b
0.012166	0.0224	1.3	hsa-miR-34c-5p
0.0151261	0.0269	1.3	HS_80
0.0333429	0.0541	1.3	hsa-miR-15b
0.0001384	0.00052	1.31	HS_303_a
0.0007179	0.00202	1.31	hsa-miR-126
0.0016085	0.00406	1.31	hsa-let-7c
0.0016686	0.00414	1.31	hsa-miR-26b*
7.50E-06	5.99E-05	1.32	hsa-miR-545*
0.0001549	0.00058	1.32	hsa-miR-625*
0.0001922	0.000686	1.32	hsa-miR-345:9.1
0.0009614	0.00261	1.32	hsa-miR-148a
0.0024974	0.00579	1.32	hsa-let-7g
0.016098	0.0282	1.32	hsa-miR-551b
0.0010183	0.00272	1.33	HS_186
0.0003258	0.00103	1.34	hsa-miR-27a
0.0015727	0.00401	1.34	hsa-miR-27a*
0.0032009	0.00708	1.34	hsa-miR-511
0.0047925	0.00999	1.34	hsa-miR-16-1*
0.0103261	0.0195	1.34	hsa-miR-135b
0.0122652	0.0225	1.34	hsa-miR-93
0.0068428	0.0136	1.35	hsa-miR-128a:9.1
0.0108517	0.0203	1.35	hsa-miR-181c*
2.66E-05	0.000147	1.36	hsa-miR-376a*
4.79E-05	0.000236	1.36	hsa-miR-34a*

0.0002022	0.000711	1.36	hsa-miR-935
0.001366	0.00356	1.36	hsa-miR-339-3p
0.0141723	0.0256	1.36	hsa-miR-377
0.0144224	0.026	1.36	hsa-miR-187
0.0167276	0.0292	1.36	hsa-miR-744
8.09E-05	0.000347	1.37	hsa-miR-491-5p
0.0008235	0.00227	1.37	hsa-miR-374a*
0.0021149	0.00502	1.37	hsa-miR-342-5p
0.0056993	0.0116	1.37	hsa-miR-424*
0.0122382	0.0225	1.37	hsa-miR-499-5p
4.90E-06	4.53E-05	1.38	hsa-miR-502-5p
0.0001765	0.000635	1.38	hsa-miR-24-2*
0.0003813	0.00118	1.38	hsa-miR-769-5p
0.0013206	0.00346	1.39	hsa-miR-548c-5p
0.0144823	0.026	1.39	hsa-miR-885-5p
0.0184641	0.0319	1.39	hsa-miR-29c*
0.0006048	0.00174	1.4	hsa-miR-598
0.0016212	0.00406	1.4	hsa-miR-221*
3.60E-06	3.68E-05	1.42	hsa-miR-24
0.0008307	0.00228	1.42	hsa-miR-502-3p,hsa-miR-500*
0.0077964	0.0152	1.42	hsa-miR-130b*
9.44E-05	0.000385	1.43	hsa-miR-539
0.0116069	0.0215	1.43	hsa-miR-197
0.0409155	0.0652	1.44	hsa-miR-1248
1.61E-05	0.000106	1.45	hsa-miR-340*
7.42E-05	0.000325	1.45	hsa-miR-199a*:9.1
0.0018476	0.0045	1.45	HS_209.1
0.0044304	0.00941	1.45	hsa-miR-181a-2*
0.0113282	0.021	1.45	hsa-miR-101
5.40E-06	4.87E-05	1.46	hsa-miR-212
2.26E-05	0.000134	1.46	hsa-miR-125a-5p

0.0038192	0.00825	1.46	hsa-miR-484
0.0152273	0.027	1.46	hsa-miR-15b*
0.0255208	0.043	1.46	hsa-miR-363
0.0001684	0.000615	1.48	hsa-miR-143
0.0049568	0.0102	1.48	hsa-miR-425
0.0001009	0.000403	1.49	hsa-miR-382
0.0005659	0.00166	1.5	hsa-let-7i*
0.0007284	0.00204	1.5	hsa-miR-487a
0.0038114	0.00825	1.5	hsa-let-7d
0.0107542	0.0202	1.5	hsa-miR-454
2.54E-05	0.000144	1.51	hsa-miR-145
0.0007157	0.00202	1.51	hsa-miR-30b
0.0269308	0.045	1.51	hsa-miR-379
5.30E-06	4.84E-05	1.52	hsa-miR-299-5p
0.0010044	0.0027	1.52	hsa-miR-615-5p
0.0046723	0.00979	1.52	hsa-miR-501-5p
0.0257768	0.0434	1.52	hsa-miR-1273
1.80E-06	2.34E-05	1.53	hsa-miR-27b
5.30E-05	0.000256	1.53	hsa-miR-125b-2*
1.66E-05	0.000109	1.54	hsa-miR-29b-1*
0.0020632	0.00491	1.55	hsa-miR-136
0.0139163	0.0252	1.55	hsa-miR-1285
0.0203519	0.0349	1.55	hsa-miR-376c
0.0470562	0.0741	1.55	hsa-miR-142-5p
0.000285	0.000933	1.56	hsa-miR-610
0.0065186	0.013	1.56	hsa-miR-107
0.0090994	0.0174	1.56	hsa-miR-130b
0.0043479	0.00926	1.57	hsa-miR-18b
0.0059986	0.0121	1.58	hsa-miR-30a*
0.0484739	0.0758	1.58	hsa-miR-182
< 1e-07	< 1e-07	1.59	hsa-miR-493*

1.00E-07	3.07E-06	1.59	hsa-miR-381
0.0007456	0.00207	1.59	hsa-miR-199a-5p
0.0037071	0.00805	1.59	hsa-miR-362-5p
0.0042832	0.00915	1.59	hsa-miR-155
2.90E-06	3.22E-05	1.6	hsa-miR-656
0.0002159	0.000746	1.6	hsa-miR-22
9.59E-05	0.000389	1.61	hsa-miR-501-3p
0.0002724	0.000901	1.61	hsa-miR-24-1*
0.0017201	0.00424	1.61	hsa-miR-32
0.0077833	0.0152	1.61	hsa-miR-542-5p
0.0001593	0.000593	1.62	hsa-miR-421
0.000401	0.00123	1.62	hsa-miR-152
0.0015298	0.00395	1.62	hsa-miR-106b*
0.0028842	0.00651	1.62	hsa-miR-181c
0.0023701	0.00551	1.63	hsa-miR-576-5p
0.0035308	0.00772	1.63	hsa-miR-218
3.00E-07	7.42E-06	1.64	hsa-miR-485-3p
2.20E-06	2.60E-05	1.64	hsa-miR-125b
4.00E-06	4.03E-05	1.64	hsa-miR-29a
0.0359736	0.0578	1.64	hsa-miR-19a
0.0001269	0.000482	1.65	hsa-miR-324-3p
0.0017477	0.0043	1.67	hsa-miR-768-3p:11.0
0.0001108	0.000429	1.69	hsa-miR-493
1.16E-05	8.24E-05	1.7	hsa-miR-28-3p
6.16E-05	0.000283	1.7	hsa-miR-452*:9.1
0.0016228	0.00406	1.71	solexa-51-13984
0.0019575	0.00469	1.71	hsa-miR-16-2*
6.00E-07	1.15E-05	1.72	hsa-let-7f-1*
0.0001018	0.000403	1.72	hsa-miR-660
< 1e-07	< 1e-07	1.73	hsa-miR-379*
3.00E-06	3.24E-05	1.73	hsa-miR-323-3p

0.0018176	0.00444	1.73	hsa-miR-17*
0.001991	0.00476	1.73	hsa-miR-886-3p
0.0021539	0.0051	1.73	hsa-miR-106a
6.00E-07	1.15E-05	1.74	hsa-miR-362-3p
7.39E-05	0.000325	1.76	hsa-miR-151:9.1
6.00E-07	1.15E-05	1.77	hsa-miR-132
2.27E-05	0.000134	1.78	hsa-miR-655
0.0003969	0.00122	1.78	hsa-miR-100
3.40E-06	3.62E-05	1.79	hsa-miR-195
0.0004744	0.00143	1.8	hsa-miR-186
0.00012	0.000463	1.81	hsa-miR-103
0.0041272	0.00884	1.81	hsa-miR-18a
2.00E-07	5.48E-06	1.82	hsa-miR-28-5p
5.70E-06	4.97E-05	1.82	hsa-miR-423-3p
0.0005303	0.00157	1.82	hsa-miR-106b
0.0001306	0.000493	1.83	hsa-miR-532-3p
0.0077482	0.0152	1.83	hsa-miR-210
0.0085871	0.0165	1.83	solexa-3277-272
1.10E-06	1.62E-05	1.84	hsa-miR-361-5p
8.32E-05	0.000352	1.84	hsa-miR-193a-3p
< 1e-07	< 1e-07	1.85	hsa-miR-889
1.00E-07	3.07E-06	1.85	hsa-miR-361-3p
6.40E-06	5.34E-05	1.86	hsa-miR-146b-5p
9.40E-06	7.28E-05	1.86	hsa-miR-29c
9.41E-05	0.000385	1.86	hsa-miR-338-3p
1.16E-05	8.24E-05	1.87	hsa-miR-139-5p
2.82E-05	0.000154	1.87	hsa-miR-410
3.49E-05	0.000187	1.87	hsa-miR-450a
6.50E-06	5.36E-05	1.88	hsa-miR-21*
0.000212	0.000736	1.88	hsa-miR-193b
4.56E-05	0.000229	1.89	hsa-miR-532-5p

0.0001213	0.000465	1.89	hsa-miR-151-3p
0.0002083	0.000726	1.9	hsa-miR-652
7.00E-07	1.22E-05	1.91	hsa-miR-340
1.61E-05	0.000106	1.91	hsa-miR-335*
4.19E-05	0.000217	1.91	hsa-miR-542-3p
0.0006596	0.00188	1.92	hsa-miR-140-5p
0.0289108	0.0481	1.92	hsa-miR-133b
1.00E-05	7.59E-05	1.93	hsa-let-7b*
7.00E-07	1.22E-05	1.94	hsa-miR-10b
0.0002686	0.000896	1.94	hsa-miR-503
2.26E-05	0.000134	1.95	hsa-miR-339-5p
0.0004953	0.00148	1.95	hsa-miR-140-3p
5.78E-05	0.000272	1.96	hsa-miR-29a*
8.72E-05	0.000365	1.97	hsa-miR-450b-5p
1.03E-05	7.67E-05	1.98	hsa-miR-151-5p
4.00E-07	9.02E-06	1.99	hsa-miR-222
5.70E-06	4.97E-05	1.99	hsa-miR-154
1.00E-05	7.59E-05	1.99	hsa-miR-505
0.0002298	0.000783	1.99	hsa-miR-127-3p
1.35E-05	9.25E-05	2	hsa-miR-590-3p
1.82E-05	0.000116	2.02	hsa-let-7a*
7.67E-05	0.00033	2.02	hsa-miR-34a
0.000569	0.00166	2.02	hsa-miR-378
0.0012568	0.0033	2.02	solexa-8211-102
0.0001772	0.000635	2.04	hsa-miR-149
3.50E-06	3.68E-05	2.05	hsa-miR-128
3.11E-05	0.000168	2.05	hsa-miR-199b-5p
6.10E-06	5.26E-05	2.08	hsa-miR-365
0.0001025	0.000403	2.08	hsa-miR-369-3p
< 1e-07	< 1e-07	2.1	hsa-miR-495
9.00E-07	1.47E-05	2.11	hsa-miR-664

3.00E-07	7.42E-06	2.13	hsa-miR-99a*
< 1e-07	< 1e-07	2.15	hsa-miR-342-3p
6.25E-05	0.000285	2.15	hsa-miR-374b
4.50E-06	4.26E-05	2.17	hsa-miR-331-3p
2.67E-05	0.000147	2.17	hsa-miR-98
2.00E-07	5.48E-06	2.18	hsa-miR-99b
0.0002291	0.000783	2.19	hsa-miR-497
3.00E-07	7.42E-06	2.2	hsa-miR-145*
1.20E-06	1.74E-05	2.23	hsa-miR-432
0.0001995	0.000705	2.23	hsa-miR-1201
2.40E-06	2.75E-05	2.24	hsa-miR-134
0.000272	0.000901	2.24	HS_192.1
2.19E-05	0.000133	2.31	hsa-miR-500
4.00E-07	9.02E-06	2.39	hsa-miR-574-3p
2.40E-05	0.00014	2.39	hsa-miR-214*
< 1e-07	< 1e-07	2.41	hsa-miR-411
0.0001959	0.000696	2.41	hsa-miR-224
< 1e-07	< 1e-07	2.43	hsa-miR-22*
1.30E-06	1.81E-05	2.5	hsa-miR-154*
4.10E-06	4.03E-05	2.6	hsa-miR-708
< 1e-07	< 1e-07	2.64	hsa-miR-329
1.00E-07	3.07E-06	2.73	hsa-miR-455-3p
1.00E-07	3.07E-06	2.74	hsa-miR-409-3p
2.00E-07	5.48E-06	2.75	hsa-miR-148b
< 1e-07	< 1e-07	2.97	hsa-miR-337-3p
8.00E-07	1.33E-05	3.01	hsa-miR-455-5p
< 1e-07	< 1e-07	3.03	hsa-miR-487b
< 1e-07	< 1e-07	3.54	hsa-miR-99a

Parametric p-value	FDR	Fold-change	miRNA ID
0.0207158	0.079	0.17	hsa-mir-23b-A
0.0210736	0.0793	0.17	hsa-mir-23b-A
0.0161757	0.0664	0.23	hsa-mir-140-P
0.0084105	0.0421	0.24	hsa-mir-136-P
0.0178319	0.0707	0.24	hsa-mir-140-P
0.0114017	0.0509	0.25	hsa-mir-136-P
0.0113161	0.0509	0.35	hsa-mir-370-A
0.0109645	0.0497	0.36	hsa-mir-95-A
0.0157745	0.0652	0.37	hsa-mir-370-A
0.0027612	0.0201	0.38	hsa-mir-320-A
0.027254	0.0935	0.38	mmu-mir-137-A
0.0289121	0.097	0.38	mmu-mir-137-A
0.0025546	0.0192	0.39	hsa-mir-320-A
0.0161043	0.0662	0.39	hsa-mir-95-A
0.0095209	0.0456	0.41	hsa-let-7a3-A
0.0151619	0.0634	0.43	hsa-let-7a3-A
0.0229329	0.0838	0.48	hsa-mir-146a-A
0.0314015	0.101	0.48	hsa-mir-130b-A
0.0194936	0.0757	0.49	hsa-let-7a-A
0.0234987	0.0856	0.49	hsa-mir-130b-A
0.0253472	0.0906	0.5	hsa-let-7a-A
0.0003088	0.0054	0.51	hsa_mir_320_Hcd306 right
0.0358167	0.111	0.51	hsa-mir-488-P
0.0256506	0.0908	0.52	hsa-mir-590-P
0.0455429	0.13	0.52	hsa-mir-488-P
0.0003226	0.00552	0.53	hsa_mir_320_Hcd306 right
0.0318115	0.102	0.53	hsa-mir-590-P
0.0141956	0.0604	0.56	hsa-mir-563-P
0.0146991	0.0621	0.59	hsa-mir-425-5p-A

Utah Dataset

Downregulated	102
Upregulated	444

0.0183338	0.0723	0.59	hsa-mir-16b-chr3-A
0.0265761	0.0926	0.6	hsa-mir-563-P
0.0339024	0.106	0.6	hsa-mir-590-A
0.0420619	0.123	0.6	hsa-mir-590-A
0.0464508	0.132	0.6	hsa-mir-106a-A
0.0109493	0.0497	0.61	hsa-mir-137-A
0.0279922	0.0949	0.61	hsa-mir-101-1/2-A
0.0438465	0.126	0.61	hsa-mir-222-A
0.0212211	0.0793	0.63	hsa-mir-425-5p-A
0.0402553	0.12	0.63	hsa-mir-101-1/2-A
0.0414082	0.122	0.64	hsa-mir-184-A
0.0475215	0.133	0.64	hsa-mir-633-A
0.0273459	0.0935	0.65	hsa-mir-137-A
0.0014753	0.0137	0.7	hsa-mir-367-P
0.0312615	0.101	0.7	hsa-mir-15a-P
0.0405392	0.121	0.7	hsa-mir-29b-1-P
0.0255073	0.0907	0.71	hsa-mir-15a-P
0.0038345	0.025	0.72	hsa-mir-144-A
0.0369718	0.114	0.72	hsa-miR-302c*-5p-A
0.0113785	0.0509	0.73	hsa-mir-193b-P
0.0428638	0.124	0.73	hsa-mir-577-A
0.0101023	0.0473	0.74	hsa-mir-367-P
0.0408605	0.121	0.74	hsa-mir-577-A
0.0145177	0.0615	0.75	hsa-mir-193b-P
0.0211874	0.0793	0.75	hsa-mir-125b2-P
0.0407213	0.121	0.77	hsa-mir-490-P
0.0428548	0.124	0.79	hsa-mir-141-P
0.0474338	0.133	0.79	hsa-mir-194-1-A
0.0012898	0.0125	0.8	hsa-mir-376a*-5p-A
0.0022374	0.0175	0.8	hsa-mir-302c*-5p-A
0.004866	0.029	0.8	hsa-mir-144-A

0.0017648	0.0152	0.81	hsa-mir-101-2-A
0.0107664	0.0492	0.81	hsa-mir-101-2-A
0.0172824	0.0691	0.81	hsa-mir-493-5p-A
0.0260975	0.0913	0.81	hsa-mir-571-P
0.0210485	0.0793	0.82	hsa-mir-24-5p/hsa-mir-189-A
0.0434804	0.125	0.82	hsa-mir-509-A
0.0001203	0.00316	0.83	hsa-mir-140-A
0.0027601	0.0201	0.83	hsa-mir-140-A
0.0201194	0.0771	0.83	hsa-mir-99b-P
0.0051673	0.03	0.84	hsa-mir-376a*-5p-A
0.0137397	0.0591	0.84	hsa-mir-34c-A
0.0376408	0.115	0.84	hsa-mir-583-P
0.0478389	0.134	0.84	hsa-mir-628-A
0.0006012	0.00744	0.85	hsa-mir-98-A
0.0009574	0.0103	0.85	hsa-mir-98-A
0.0046143	0.028	0.85	hsa-mir-106a-P
0.0059359	0.0323	0.85	hsa-mir-302c*-5p-A
0.0131626	0.0569	0.85	hsa-mir-376b-A
0.01983	0.0766	0.85	hsa-mir-365-2-A
0.0270183	0.0933	0.85	hsa-mir-601-P
0.0294076	0.0978	0.85	hsa-mir-208-A
0.0005967	0.00744	0.86	hsa-miR-302c-3p
0.0018234	0.0154	0.86	hsa-miR-302c-3p
0.0032439	0.0228	0.86	hsa-mir-302c-3p-A
0.0444823	0.127	0.86	hsa-mir-99b-P
0.0033444	0.0231	0.87	hsa-mir-376b-A
0.0433066	0.125	0.87	hsa-mir-337-P
0.0035483	0.0237	0.88	hsa-mir-588-A
0.0202339	0.0774	0.88	hsa-mir-365-2-A
0.0209811	0.0793	0.88	hsa-mir-644-A
0.0409291	0.121	0.88	hsa-mir-802-A

0.0467433	0.132	0.88	hsa-mir-495-A
0.0084828	0.0422	0.89	hsa-mir-302c-3p-A
0.0266977	0.0926	0.9	hsa-mir-802-A
0.0273004	0.0935	0.9	hsa-mir-545-P
0.0338086	0.106	0.9	hsa-mir-493-5p-A
0.0360894	0.112	0.9	hsa-mir-652-A
0.0368838	0.114	0.9	hsa-mir-510-A
0.0418394	0.123	0.9	hsa-mir-503-A
0.0455549	0.13	0.9	hsa-mir-412-P
0.0394427	0.119	0.91	hsa-mir-569-P
0.042748	0.124	0.91	hsa-mir-369-3p-A
0.0387052	0.117	1.1	hsa-mir-202-3p-A
0.0227825	0.0838	1.11	hsa-mir-378-5p-A
0.0307457	0.0998	1.11	hsa-mir-16-1-P
0.0456488	0.13	1.11	hsa-mir-124a1-A
0.0272782	0.0935	1.12	hsa-mir-18a*-3p-A
0.0303529	0.0994	1.12	hsa-mir-588-P
0.047381	0.133	1.12	hsa-mir-551a-P
0.0069564	0.0359	1.13	hsa-mir-588-P
0.0085598	0.0424	1.13	hsa-mir-518b-P
0.016593	0.0677	1.13	hsa-mir-616-P
0.0277021	0.0944	1.13	mmu-mir-433-3p-A
0.0419858	0.123	1.13	hsa-mir-9*-A
0.0472165	0.133	1.13	hsa-mir-498-P
0.0331505	0.105	1.14	hsa-mir-455-P
0.0049952	0.0295	1.15	hsa-mir-433-A
0.0056257	0.0314	1.15	hsa-mir-656-P
0.0058961	0.0322	1.15	hsa-mir-124a3-A
0.0072691	0.0373	1.15	hsa-mir-378-5p-A
0.0091498	0.0445	1.15	hsa-mir-621-A
0.0101025	0.0473	1.15	hsa-mir-448-P

0.0147906	0.0624	1.15	hsa-mir-30d-P
0.0172993	0.0691	1.15	hsa-mir-130a-P
0.0214371	0.0794	1.15	hsa-mir-495-P
0.0035444	0.0237	1.16	hsa-mir-124a3-A
0.0053359	0.0303	1.16	hsa-mir-518a2-5p/mir527-A
0.0137862	0.0591	1.16	hsa-mir-524-3p-A
0.0042955	0.0268	1.17	hsa-mir-621-A
0.0116888	0.0519	1.17	hsa-mir-187-P
0.0284014	0.0958	1.17	hsa-mir-1-1-P
0.0016821	0.0149	1.18	hsa-mir-525-5p-A
0.0069664	0.0359	1.18	hsa-mir-183-A
0.0260182	0.0912	1.18	hsa-mir-372-A
0.0320751	0.102	1.18	hsa-mir-558-A
0.0008071	0.00903	1.19	hsa-mir-607-A
0.0010325	0.0107	1.19	hsa-mir-148a-P
0.0019499	0.0159	1.19	hsa-mir-410-A
0.0036492	0.0243	1.19	hsa-mir-30d-P
0.0038696	0.0252	1.19	hsa-mir-584-A
0.0165724	0.0677	1.19	hsa-mir-30c-1-P
0.0211405	0.0793	1.19	hsa-mir-561-P
0.0283775	0.0958	1.19	hsa-miR-769-5p-A
0.0299366	0.0986	1.19	hsa-mir-135a1-A
0.032083	0.102	1.19	hsa-mir-9*-A
0.0342667	0.107	1.19	hsa-mir-26a-2-P
0.038613	0.117	1.19	hsa-mir-32-P
0.0002322	0.00442	1.2	hsa-mir-518a2-5p/mir527-A
0.0019321	0.0158	1.2	hsa-mir-365-1-P
0.0037459	0.0246	1.2	hsa-mir-377-P
0.0089299	0.0437	1.2	hsa-mir-372-A
0.0093384	0.0452	1.2	hsa-mir-651-A
0.0096438	0.0459	1.2	mmu-mir-383-P

0.0141666	0.0604	1.2	mmu-mir-383-P
0.0200342	0.077	1.2	hsa-mir-337-A
0.0307199	0.0998	1.2	hsa-mir-217-P
0.0435007	0.125	1.2	hsa-mir-500-P
0.0031649	0.0224	1.21	hsa-mir-493-3p-A
0.0053	0.0303	1.21	hsa-mir-671-P
0.0120875	0.0529	1.21	hsa-mir-493-3p-A
0.0140376	0.06	1.21	hsa-mir-671-P
0.003096	0.0221	1.22	hsa-mir-659-A
0.0034826	0.0237	1.22	hsa-mir-544-A
0.0040557	0.0259	1.22	hsa-mir-611-A
0.0056004	0.0314	1.22	hsa-mir-558-A
0.0061685	0.0332	1.22	hsa-mir-200c-P
0.0118119	0.0521	1.22	hsa-miR-769-5p-A
0.0212509	0.0793	1.22	hsa-mir-181b-1-P
0.0246269	0.0888	1.22	hsa-mir-217-P
0.0380859	0.116	1.22	hsa-mir-126-3p-A
0.0424245	0.124	1.22	hsa-mir-548c-P
1.99E-05	0.00112	1.23	hsa-mir-649-A
0.0009443	0.0102	1.23	hsa-mir-622-A
0.0012069	0.0119	1.23	hsa-mir-584-A
0.0026101	0.0195	1.23	hsa-mir-650-A
0.0036777	0.0243	1.23	hsa-mir-452-5p-A
0.0041385	0.0261	1.23	hsa-mir-26a-2-P
0.0044502	0.0273	1.23	hsa-mir-659-A
0.0387903	0.117	1.23	hsa-mir-10a-P
7.14E-05	0.00231	1.24	hsa-mir-607-A
0.0010249	0.0107	1.24	hsa-mir-622-A
0.0024054	0.0185	1.24	hsa-mir-135a1-A
0.0024735	0.0189	1.24	hsa-mir-651-A
0.0029591	0.0214	1.24	hsa-mir-609-A

0.0171131	0.0691	1.24	hsa-mir-337-A
0.0213251	0.0794	1.24	hsa-mir-7-1-A
0.0229702	0.0838	1.24	hsa-mir-105-2-P
0.0248649	0.0893	1.24	hsa-mir-105-2-P
0.0262166	0.0915	1.24	hsa-mir-502-P
0.0267128	0.0926	1.24	hsa-mir-224-A
0.0040304	0.0259	1.25	hsa-mir-346-P
0.0098019	0.0463	1.25	hsa-mir-340-P
0.0151632	0.0634	1.25	hsa-mir-661-P
0.0017586	0.0152	1.26	hsa-mir-365-1-P
0.007147	0.0367	1.26	hsa-mir-346-P
0.0094231	0.0452	1.26	hsa-mir-340-P
0.0153156	0.0637	1.26	hsa-mir-635-A
0.0277108	0.0944	1.26	hsa-miR-769-3p-A
0.0296258	0.098	1.26	hsa-mir-576-A
0.0400261	0.12	1.26	hsa-mir-644-P
0.0243667	0.0882	1.27	hsa-mir-631-P
0.0279723	0.0949	1.27	hsa-mir-548c-P
0.0384677	0.117	1.27	hsa-mir-373-3p-A
0.0005848	0.00736	1.28	hsa-mir-452-5p-A
0.0008346	0.0092	1.28	hsa-mir-148a-P
0.0047635	0.0285	1.28	hsa-mir-370-P
0.0051879	0.03	1.28	hsa-mir-550-2-A
0.0131671	0.0569	1.28	hsa-mir-613-A
0.0158547	0.0654	1.28	hsa-mir-629-P
0.0196995	0.0763	1.28	hsa-mir-548d1-A
0.0314843	0.101	1.28	hsa-mir-96-A
0.0383147	0.117	1.28	hsa-mir-644-P
0.0021967	0.0173	1.29	hsa-mir-650-A
0.0053234	0.0303	1.29	hsa-mir-561-P
0.0063864	0.0339	1.29	hsa-mir-500-P

0.0169747	0.0687	1.29	hsa-mir-31-A
0.0004165	0.00613	1.3	hsa-mir-495-P
0.0025091	0.019	1.3	hsa-mir-181b-1-P
0.0031783	0.0224	1.3	hsa-mir-502-P
0.0041141	0.0261	1.3	hsa-mir-587-P
0.0085988	0.0425	1.3	hsa-mir-548d1-A
0.0111286	0.0503	1.3	hsa-mir-185-A
0.0113726	0.0509	1.3	hsa-mir-615-P
0.0185626	0.0729	1.3	hsa-mir-645-A
0.0035387	0.0237	1.31	hsa-mir-1-2-P
0.0103803	0.0482	1.31	hsa-mir-215-P
0.0308342	0.0999	1.31	hsa-mir-609-P
0.00907	0.0442	1.32	hsa-miR-769-3p-A
0.0097734	0.0463	1.32	hsa-mir-661-P
0.0115462	0.0514	1.32	hsa-mir-654-A
0.0409838	0.121	1.32	mmu-mir-128a-P
0.0018265	0.0154	1.33	hsa-mir-370-P
0.0035347	0.0237	1.33	hsa-mir-224-A
0.0050694	0.0298	1.33	hsa-mir-661-A
0.0057933	0.0319	1.33	hsa-mir-576-A
0.0063033	0.0337	1.33	hsa-mir-1-2-P
0.0152705	0.0637	1.33	hsa-mir-96-A
0.0246925	0.0888	1.33	hsa-mir-181b-2-P
0.0292485	0.0976	1.33	hsa-mir-659-P
0.0314944	0.101	1.33	hsa-mir-27a-A
0.0342561	0.107	1.33	hsa-mir-200b-P
0.001876	0.0156	1.34	hsa-mir-7-1-A
0.0026865	0.0198	1.34	hsa-mir-424-P
0.0031162	0.0222	1.34	hsa-mir-572-P
0.0066696	0.0349	1.34	hsa-mir-629-P
0.0066953	0.0349	1.34	hsa-mir-29a-P

0.0124583	0.0543	1.34	hsa-mir-654-P
0.0214868	0.0794	1.34	hsa-mir-30c-2-P
0.0008277	0.0092	1.35	hsa-mir-424-P
0.0059065	0.0322	1.35	hsa-mir-30a-3p-A
0.0080193	0.0405	1.35	hsa-mir-615-P
0.0094184	0.0452	1.35	hsa-mir-544-A
0.0171963	0.0691	1.35	hsa-mir-148b-P
0.0210357	0.0793	1.35	hsa-mir-129-1-P
0.029556	0.098	1.35	hsa-mir-487a-A
0.0317888	0.102	1.35	hsa-mir-619-A
0.0006931	0.00824	1.36	hsa-mir-516-3-5p-A
0.0007356	0.00849	1.36	hsa-mir-635-A
0.0007363	0.00849	1.36	hsa-mir-661-A
0.0030036	0.0217	1.36	hsa-mir-654-A
0.0044938	0.0275	1.36	hsa-miR-373*-5p-A
0.0347336	0.108	1.36	hsa-mir-34b-P
0.0408014	0.121	1.36	hsa-mir-770-P
5.79E-05	0.002	1.37	hsa-mir-611-A
0.0003485	0.00589	1.37	hsa-mir-134-P
0.0033155	0.0231	1.37	hsa-mir-200b-P
0.0051616	0.03	1.37	hsa-mir-29a-P
0.0172026	0.0691	1.37	hsa-mir-339-A
0.0191182	0.0744	1.37	hsa-mir-185-P
0.0044374	0.0273	1.38	hsa-mir-634-P
0.0304297	0.0994	1.38	hsa-mir-34c-P
0.0011902	0.0118	1.39	hsa-mir-562-A
0.001389	0.0133	1.39	hsa-mir-192-P
0.0014675	0.0137	1.39	hsa-mir-572-P
0.0037031	0.0244	1.39	hsa-mir-663-A
0.0040632	0.0259	1.39	hsa-mir-499-A
0.0096094	0.0458	1.39	hsa-mir-574-P

0.0303951	0.0994	1.39	hsa-mir-621-P
3.07E-05	0.00137	1.4	hsa-mir-516-3-5p-A
0.0052213	0.0301	1.4	hsa-mir-654-P
0.0078586	0.0399	1.4	hsa-mir-148b-P
0.0167334	0.0681	1.4	hsa-mir-560-P
0.0298585	0.0986	1.4	hsa-mir-27a-A
0.0325755	0.103	1.4	hsa-mir-770-P
0.0350759	0.109	1.4	hsa-mir-136-A
0.0425189	0.124	1.4	hsa-mir-766-A
0.0003919	0.00609	1.41	hsa-mir-633-P
0.001019	0.0107	1.41	hsa-mir-613-A
0.0040011	0.0258	1.41	hsa-mir-499-A
0.0044323	0.0273	1.41	hsa-mir-487a-A
0.0087137	0.0429	1.41	hsa-mir-609-P
0.0128796	0.056	1.41	hsa-mir-129-1-P
0.0167753	0.0681	1.41	hsa-mir-339-A
0.0187239	0.0733	1.41	hsa-mir-560-P
0.0467736	0.132	1.41	hsa-mir-138-2-A
7.30E-06	0.000751	1.42	hsa-mir-519e*-5p-A
2.42E-05	0.00123	1.42	hsa-mir-134-P
7.80E-05	0.00237	1.42	hsa-mir-331-A
0.0006236	0.00765	1.42	hsa-mir-31-A
0.0007587	0.00862	1.42	hsa-mir-564-A
0.0012695	0.0124	1.42	hsa-mir-215-P
0.0034002	0.0233	1.42	hsa-mir-634-P
0.0049384	0.0294	1.42	hsa-mir-632-P
0.0337054	0.106	1.42	hsa-mir-660-P
0.0014626	0.0137	1.43	hsa-mir-520e-P
0.0039877	0.0258	1.43	hsa-mir-181b-2-P
0.0043928	0.0273	1.43	hsa-mir-632-P
0.0188252	0.0735	1.43	hsa-mir-766-A

0.0431925	0.125	1.43	hsa-mir-192-A
4.97E-05	0.0018	1.44	hsa-mir-548d2-A
0.0022044	0.0173	1.44	hsa-mir-663-A
0.0049983	0.0295	1.44	hsa-mir-135a-1-A
0.0002709	0.00497	1.45	hsa-mir-562-A
0.0005025	0.00695	1.45	hsa-mir-192-P
0.0005122	0.00698	1.45	hsa-mir-631-A
0.0020263	0.0163	1.45	hsa-mir-520e-P
0.0063427	0.0338	1.45	hsa-mir-616-A
0.0249865	0.0895	1.45	hsa-mir-657-A
1.00E-06	0.000522	1.46	hsa-mir-331-A
0.0076173	0.0389	1.46	hsa-mir-519e*-5p-A
0.0178773	0.0707	1.46	hsa-mir-339-P
0.0185789	0.0729	1.46	hsa-mir-29c-A
0.0396719	0.119	1.46	hsa-mir-548c-A
0.0005401	0.00703	1.47	hsa-mir-587-P
0.0011405	0.0113	1.47	hsa-mir-498-A
0.001412	0.0134	1.47	hsa-miR-373*-5p-A
0.0016392	0.0146	1.47	hsa-mir-658-A
0.0060429	0.0327	1.47	hsa-mir-616-A
0.0118153	0.0521	1.47	hsa-mir-621-P
0.0294354	0.0978	1.47	hsa-mir-34c-P
0.0368575	0.114	1.47	hsa-mir-213-P
0.0007201	0.00843	1.48	hsa-mir-645-A
0.0025704	0.0193	1.48	hsa-mir-135a-1-A
0.0499898	0.139	1.48	hsa-mir-497-A
0.0001046	0.00291	1.49	hsa-mir-30a-3p-A
0.0001393	0.00337	1.49	hsa-mir-564-A
0.000179	0.00384	1.49	hsa-mir-612-P
0.0003202	0.00552	1.49	hsa-mir-152-P
0.0058007	0.0319	1.49	hsa-mir-574-P

0.0061964	0.0332	1.49	hsa-mir-657-P
0.0464815	0.132	1.49	hsa-mir-199b-A
0.0002042	0.00408	1.5	hsa-mir-658-A
0.0002506	0.00465	1.5	hsa-mir-152-P
0.0006373	0.00776	1.5	hsa-mir-612-P
0.0023527	0.0183	1.5	hsa-mir-9-5p-A
4.43E-05	0.00169	1.51	hsa-mir-602-P
0.0034103	0.0233	1.51	hsa-mir-765-P
0.0155626	0.0645	1.51	hsa-mir-215-A
0.0372914	0.114	1.51	hsa-mir-595-A
2.51E-05	0.00123	1.52	hsa-mir-548d2-A
0.0011395	0.0113	1.52	hsa-mir-9-5p-A
0.0200272	0.077	1.52	hsa-mir-581-P
0.0270212	0.0933	1.52	hsa-mir-596-A
7.66E-05	0.00237	1.53	hsa-mir-383-P
0.0003578	0.00598	1.53	hsa-mir-425-3p-A
0.0006839	0.0082	1.53	hsa-mir-498-A
0.0047024	0.0283	1.53	hsa-mir-668-P
0.0093973	0.0452	1.53	hsa-mir-136-A
0.0103709	0.0482	1.53	hsa-mir-29c-A
0.0105612	0.0487	1.53	hsa-mir-324-3p-A
6.35E-05	0.0021	1.54	hsa-mir-602-P
0.0119822	0.0527	1.54	hsa-mir-581-P
0.0120666	0.0529	1.54	hsa-mir-92b-P
0.0107702	0.0492	1.55	hsa-mir-92b-P
0.0003726	0.00609	1.56	hsa-mir-637-P
0.0003981	0.00609	1.56	hsa-mir-425-3p-A
0.008371	0.042	1.56	hsa-mir-324-3p-A
0.0010764	0.011	1.57	hsa-mir-765-P
0.0321749	0.102	1.57	hsa-mir-92b-A
4.76E-05	0.00177	1.58	hsa-mir-631-A

0.0001866	0.00394	1.58	hsa-mir-551a-A
0.0005605	0.00716	1.58	hsa-mir-345-P
0.0046468	0.0281	1.58	hsa-mir-632-A
0.0258826	0.0912	1.58	hsa-mir-92b-A
0.0005181	0.00698	1.59	hsa-mir-637-P
0.0005272	0.00698	1.59	hsa-mir-345-P
0.0055136	0.0311	1.59	hsa-mir-632-A
0.0015351	0.014	1.6	hsa-mir-639-P
0.0015023	0.0138	1.61	hsa-mir-639-P
0.0014219	0.0134	1.62	hsa-mir-212-A
0.0017928	0.0153	1.62	hsa-mir-212-A
0.0022071	0.0173	1.62	hsa-mir-338-A
0.0054452	0.0308	1.62	hsa-mir-550-2-P
6.80E-06	0.000751	1.63	hsa-mir-383-P
0.0001641	0.00378	1.63	hsa-mir-603-A
0.001757	0.0152	1.63	hsa-mir-668-P
0.0030243	0.0217	1.63	hsa-mir-550-2-P
1.55E-05	0.00098	1.64	hsa-mir-487b-P
0.0002886	0.00517	1.64	hsa-mir-603-A
0.0067714	0.0352	1.64	hsa-mir-564-P
0.0255793	0.0907	1.64	hsa-mir-30a-5p-A
0.0484122	0.135	1.64	hsa-mir-614-A
0.0088321	0.0434	1.65	hsa-mir-331-P
0.0352266	0.109	1.65	hsa-mir-220-A
0.0003636	0.00602	1.66	hsa-mir-181c-P
0.0307069	0.0998	1.66	hsa-mir-220-A
5.64E-05	0.002	1.68	hsa-mir-662-A
0.0015757	0.0142	1.68	hsa-mir-591-P
0.0056211	0.0314	1.68	hsa-mir-596-A
0.0254295	0.0906	1.68	hsa-mir-30a-5p-A
0.0301767	0.0992	1.68	hsa-mir-10b-P

0.000173	0.00384	1.69	hsa-mir-662-A
0.0002064	0.00408	1.69	hsa-mir-181c-P
0.00107	0.011	1.69	hsa-mir-598-P
0.000791	0.00892	1.7	hsa-mir-548a2-A
0.0051035	0.0299	1.71	hsa-mir-7-3-A
1.20E-06	0.000522	1.72	hsa-mir-487b-P
8.80E-06	0.000788	1.72	hsa-mir-551a-A
0.0001391	0.00337	1.72	hsa-mir-604-P
0.0084687	0.0422	1.72	hsa-mir-7-3-A
0.0008955	0.00974	1.73	hsa-mir-564-P
0.0018283	0.0154	1.74	hsa-mir-205-A
0.0045946	0.028	1.74	hsa-mir-331-P
0.000419	0.00613	1.75	hsa-mir-338-A
0.0004681	0.00666	1.75	hsa-mir-591-P
0.0011049	0.0112	1.76	hsa-mir-598-P
0.0007039	0.0083	1.77	hsa-mir-330-A
0.0001762	0.00384	1.78	hsa-mir-330-A
3.31E-05	0.00138	1.79	hsa-mir-604-P
0.0019739	0.016	1.8	hsa-mir-589-P
0.0052939	0.0303	1.8	hsa-mir-129-2-A
0.0244025	0.0882	1.8	hsa-mir-633-P
0.0017372	0.0152	1.81	hsa-mir-205-A
0.0033453	0.0231	1.81	hsa-miR-126*-5p-A
0.0041522	0.0261	1.81	hsa-mir-550-1-P
7.90E-06	0.000751	1.82	hsa-mir-646-P
0.0007528	0.00861	1.82	hsa-mir-326-P
0.0026722	0.0197	1.82	hsa-mir-129-2-A
0.0006568	0.00793	1.83	hsa-mir-326-P
0.0056862	0.0316	1.83	hsa-mir-550-1-P
0.0260156	0.0912	1.83	hsa-mir-539-P
0.0292044	0.0976	1.83	hsa-mir-589-A

0.0001025	0.00291	1.84	hsa-mir-548a2-A
0.001838	0.0154	1.84	hsa-miR-126*-5p-A
0.0001789	0.00384	1.85	hsa-mir-487b-A
0.0010211	0.0107	1.85	hsa-mir-650-P
0.0057314	0.0317	1.85	hsa-mir-218-2-P
0.0135093	0.0582	1.85	hsa-mir-566-A
2.40E-06	0.000522	1.86	hsa-mir-615-A
1.90E-06	0.000522	1.87	hsa-mir-615-A
0.0175298	0.0698	1.87	hsa-mir-663-P
8.80E-05	0.00258	1.88	hsa-mir-487b-A
0.0081467	0.0411	1.88	hsa-mir-202*-5p-A
2.90E-06	0.000541	1.89	hsa-mir-422a-P
0.0042604	0.0267	1.89	hsa-mir-218-2-P
0.0064174	0.0339	1.89	hsa-mir-27b-A
0.0229439	0.0838	1.89	hsa-mir-219-2-P
0.001548	0.014	1.9	hsa-mir-627-A
2.90E-05	0.00134	1.91	hsa-mir-770-A
0.0003874	0.00609	1.91	hsa-mir-412-A
0.0060979	0.0329	1.91	hsa-mir-202*-5p-A
0.0066783	0.0349	1.91	hsa-mir-612-A
0.0221683	0.0817	1.91	hsa-mir-219-2-P
0.047031	0.133	1.91	hsa-mir-216-P
1.90E-06	0.000522	1.92	hsa-mir-646-P
0.0209801	0.0793	1.92	hsa-mir-194-1-P
0.0109307	0.0497	1.93	hsa-mir-663-P
0.010285	0.048	1.94	hsa-mir-566-A
0.0001982	0.00402	1.95	hsa-mir-627-A
0.0005232	0.00698	1.95	hsa-mir-650-P
0.0005023	0.00695	1.96	hsa-miR-324-3p-A
0.0100924	0.0473	1.96	hsa-mir-126-5p-A
1.20E-06	0.000522	1.97	hsa-mir-422a-P

0.0004432	0.00642	1.97	hsa-mir-589-P
0.0338864	0.106	1.97	hsa-mir-216-P
0.0004005	0.00609	1.98	hsa-mir-184-P
0.0106966	0.0492	1.98	hsa-mir-539-P
0.0004082	0.00609	1.99	hsa-mir-184-P
0.0004995	0.00695	1.99	hsa-mir-326-A
0.0023992	0.0185	1.99	hsa-mir-129-2-P
0.0002175	0.00424	2	hsa-mir-320-P
0.0003916	0.00609	2	hsa-miR-324-5p-A
0.0011377	0.0113	2	hsa-mir-593-A
0.0025149	0.019	2	hsa-mir-593-A
0.0001305	0.00335	2.01	hsa-miR-324-5p-A
0.0001915	0.00399	2.01	hsa-mir-323-P
0.0002845	0.00515	2.01	hsa-mir-320-P
0.0033773	0.0233	2.01	hsa-mir-612-A
3.63E-05	0.00145	2.02	hsa-mir-296-A
0.0065733	0.0346	2.02	hsa-mir-222-P
0.0076948	0.0392	2.02	hsa-mir-126-5p-A
0.0001336	0.00335	2.03	hsa-mir-412-A
0.0004045	0.00609	2.03	hsa-miR-324-3p-A
0.0018932	0.0157	2.03	hsa-mir-129-2-P
8.08E-05	0.00241	2.06	hsa-mir-634-A
4.40E-06	0.000636	2.08	hsa-mir-770-A
6.00E-05	0.00203	2.08	hsa-mir-658-P
0.0001052	0.00291	2.08	hsa-mir-634-A
0.0021122	0.0168	2.09	hsa-mir-222-P
0.0013516	0.013	2.1	hsa-mir-202*-5p-A
0.0004023	0.00609	2.11	hsa-mir-326-A
0.000982	0.0105	2.11	hsa-mir-194-2-A
9.80E-06	0.000809	2.12	hsa-mir-629-A
0.0258355	0.0912	2.13	hsa-mir-594-P

0.0399292	0.12	2.13	hsa-mir-594-P
2.37E-05	0.00123	2.14	hsa-mir-658-P
0.0003778	0.00609	2.14	hsa-mir-194-2-A
7.90E-06	0.000751	2.15	hsa-mir-296-A
1.57E-05	0.00098	2.15	hsa-mir-629-A
1.31E-05	0.000906	2.16	hsa-mir-133b-P
0.0017648	0.0152	2.16	mmu-mir-152-A
0.0104116	0.0482	2.2	hsa-mir-194-1-P
0.0016311	0.0146	2.21	mmu-mir-152-A
0.0019235	0.0158	2.21	hsa-mir-556-P
0.0001087	0.00295	2.23	hsa-mir-346-A
2.06E-05	0.00112	2.25	hsa-mir-323-P
1.25E-05	0.000906	2.26	hsa-mir-150-A
0.038694	0.117	2.27	hsa-mir-130b-P
4.60E-06	0.000636	2.28	hsa-mir-133b-P
0.0005524	0.00713	2.29	hsa-mir-202*-5p-A
0.0392041	0.118	2.29	hsa-mir-193a-A
2.06E-05	0.00112	2.3	hsa-mir-150-A
0.0001567	0.00367	2.3	hsa-mir-449b-P
0.000151	0.00359	2.31	hsa-mir-449b-P
0.0003057	0.0054	2.32	hsa-mir-346-A
0.0015098	0.0138	2.37	hsa-mir-335-P
2.10E-06	0.000522	2.38	hsa-mir-7-2-A
0.0008757	0.00959	2.39	hsa-mir-556-P
3.20E-06	0.000541	2.42	hsa-mir-7-2-A
0.0005207	0.00698	2.48	hsa-mir-146b-P
4.21E-05	0.00164	2.49	hsa-mir-324-5p-A
0.0004664	0.00666	2.49	hsa-mir-335-P
0.0001344	0.00335	2.52	hsa-mir-602-A
0.0001179	0.00315	2.53	hsa-mir-196b-P
3.32E-05	0.00138	2.57	hsa-mir-429-P

1.01E-05	0.000809	2.59	hsa-mir-429-P
0.0002433	0.00457	2.6	hsa-mir-602-A
7.27E-05	0.00231	2.63	hsa-mir-196b-P
0.0176859	0.0703	2.67	hsa-mir-218-2-A
0.0001942	0.00399	2.69	hsa-mir-146b-P
1.21E-05	0.000906	2.7	hsa-mir-324-5p-A
0.0005643	0.00716	2.71	hsa-mir-578-P
0.0002258	0.00435	2.72	hsa-mir-636-A
0.0005372	0.00703	2.73	hsa-mir-578-P
0.0026421	0.0196	2.81	hsa-mir-371-A
1.61E-05	0.00098	2.83	mmu-mir-138-A
0.0150053	0.0631	2.83	hsa-mir-218-2-A
6.90E-06	0.000751	2.86	mmu-mir-138-A
0.0001791	0.00384	2.88	hsa-mir-636-A
0.0020319	0.0163	2.99	hsa-mir-371-A
2.84E-05	0.00134	3.02	hsa-mir-429-A
3.35E-05	0.00138	3.04	hsa-mir-429-A
0.0242193	0.088	3.3	hsa-mir-151-A
0.0214791	0.0794	3.45	hsa-mir-151-A
0.028739	0.0968	6.54	hsa-mir-187-A
0.0289251	0.097	6.66	hsa-mir-187-A

Parametric p-value	FDR	Fold-change	miRNA ID
0.0413666	0.865	0.29	hsa-miR-214*
0.0007395	0.441	0.3	hsa-miR-181a*
0.001372	0.441	0.3	hsa-miR-181a*
0.0017084	0.441	0.31	hsa-miR-181a
0.0024692	0.476	0.33	hsa-miR-181a
0.001298	0.441	0.34	hsa-miR-181b
0.001459	0.441	0.35	hsa-miR-181b
0.0124021	0.865	0.39	hsa-miR-27a*
0.0478382	0.865	0.42	hsa-miR-25*
0.0329642	0.865	0.45	hsa-let-7c*
0.0095276	0.865	0.5	hsa-miR-92b
0.0340366	0.865	0.5	hsa-miR-601
0.0020816	0.441	0.52	hsa-miR-541*
0.0157942	0.865	0.53	hsa-miR-345
0.0066471	0.83	0.54	hsa-miR-604
0.0415109	0.865	0.54	hsa-miR-23a*
0.0442117	0.865	0.54	hsa-miR-7-1*
0.0475225	0.865	0.54	hsa-miR-624*
0.0330585	0.865	0.55	hsa-miR-618
0.043304	0.865	0.55	hsa-miR-99b*
0.0002792	0.441	0.56	hsa-miR-489
0.0137023	0.865	0.56	hsa-miR-186*
0.0315515	0.865	0.56	hsa-miR-513c
0.0366332	0.865	0.56	hsa-miR-431*
0.0011309	0.441	0.57	hsa-miR-19b-1*
0.0318338	0.865	0.57	hsa-miR-593
0.0272831	0.865	0.58	hsa-miR-454*
0.0478018	0.865	0.58	hsa-miR-7-1*
0.0139654	0.865	0.59	hsa-miR-936

Cell Line Dataset

Downregulated	71
Upregulated	40

0.0355989	0.865	0.59	hsa-miR-513a-5p
0.0408042	0.865	0.59	hsa-let-7c*
0.0032041	0.566	0.6	hsa-miR-801
0.0045275	0.738	0.6	hsa-miR-125b-1*
0.0070458	0.83	0.6	hsa-miR-99b*
0.04933	0.865	0.6	hsa-miR-431*
0.0421827	0.865	0.61	hsa-miR-186
0.0160993	0.865	0.62	hsa-miR-491-5p
0.021372	0.865	0.62	hsa-miR-371-3p
0.0464366	0.865	0.62	hsa-miR-513a-5p
0.0144711	0.865	0.63	hsa-miR-801
0.0395691	0.865	0.63	hsa-miR-185*
0.0413134	0.865	0.63	hsa-miR-200a*
0.0017363	0.441	0.64	hsa-miR-491-3p
0.0101205	0.865	0.64	hsa-miR-648
0.0409127	0.865	0.64	hsa-let-7e*
0.0068107	0.83	0.65	hsa-miR-518d-3p
0.0206805	0.865	0.65	hsa-miR-888*
0.0317645	0.865	0.65	hsa-miR-202
0.047068	0.865	0.65	hsa-miR-431*
0.0369221	0.865	0.66	hsa-miR-552
0.0430273	0.865	0.66	hsa-miR-412
0.048702	0.865	0.66	hsa-miR-500
0.0097486	0.865	0.67	hsa-miR-491-3p
0.0401414	0.865	0.67	hsa-miR-618
0.0478112	0.865	0.68	hsa-miR-501-5p
0.0288293	0.865	0.69	hsa-miR-421
0.0345715	0.865	0.69	hsa-miR-575
0.0435123	0.865	0.7	hsa-miR-500
0.0454971	0.865	0.7	hsa-miR-125a-3p
0.0057618	0.83	0.71	hsa-miR-939

0.020549	0.865	0.71	hsa-miR-564
0.0466663	0.865	0.72	hsa-miR-338-5p
0.0074757	0.834	0.73	hsa-miR-939
0.0202308	0.865	0.73	hsa-miR-768-3p
0.0449114	0.865	0.73	hsa-miR-564
0.045279	0.865	0.73	hsa-miR-1226*
0.0321557	0.865	0.76	hsa-miR-191*
0.0438345	0.865	0.77	hsa-miR-768-3p
0.0464971	0.865	0.78	hsa-miR-107
0.0271578	0.865	0.83	hsa-miR-193b*
0.0179891	0.865	1.23	hsa-miR-597
0.0255068	0.865	1.24	hsa-miR-363*
0.021013	0.865	1.3	hsa-miR-561
0.026498	0.865	1.61	hsa-miR-587
0.0466056	0.865	1.64	hsa-miR-29c
0.0288576	0.865	1.68	hsa-miR-29c
0.0429729	0.865	1.75	hsa-miR-151-3p
0.0252651	0.865	1.82	hsa-miR-122*
0.0248672	0.865	1.88	hsa-miR-142-5p
0.0152789	0.865	1.9	hsa-miR-339-5p
0.0335683	0.865	1.93	hsa-miR-379*
0.0321512	0.865	1.99	hsa-miR-411*
0.0020336	0.441	2	hsa-miR-379*
0.0245298	0.865	2	hsa-miR-151-5p
0.026201	0.865	2.02	hsa-miR-151-5p
0.0439253	0.865	2.06	hsa-miR-549
0.0129595	0.865	2.14	hsa-miR-889
0.0352917	0.865	2.14	hsa-miR-584
0.0446692	0.865	2.18	hsa-miR-183*
0.0433384	0.865	2.22	hsa-miR-126
0.0376391	0.865	2.39	hsa-miR-139-5p

0.0244401	0.865	2.4	hsa-miR-488
0.0172945	0.865	2.74	hsa-miR-935
0.0401966	0.865	2.76	hsa-miR-613
0.0436318	0.865	2.76	hsa-miR-323-3p
0.0207626	0.865	2.8	hsa-miR-708
0.0195821	0.865	2.86	hsa-miR-126
0.0491625	0.865	2.97	hsa-miR-329
0.0371807	0.865	3.19	hsa-miR-337-3p
0.0452773	0.865	3.4	hsa-miR-136*
0.0460414	0.865	3.62	hsa-miR-432
0.0122016	0.865	4.44	hsa-miR-130a
0.0354964	0.865	4.52	hsa-miR-495
0.0114378	0.865	4.66	hsa-miR-130a
0.0148754	0.865	5.51	hsa-miR-411
0.0350592	0.865	7.8	hsa-miR-495
0.0444765	0.865	8.43	hsa-miR-135b
0.0222012	0.865	8.56	hsa-miR-135b
0.0092825	0.865	9.4	hsa-miR-146a
0.0062871	0.83	20.03	hsa-miR-146a
0.037349	0.865	22.56	hsa-miR-155
