# Supplementary material for: An imprinted non-coding genomic cluster at 14q32 defines clinically relevant molecular subtypes in osteosarcoma across multiple independent datasets
Source: J Hematol Oncol. 2017 May 15;10:107. doi: 10.1186/s13045-017-0465-4 (PMC5433149; doi:10.1186/s13045-017-0465-4)
Supplement: Supplementary file 8 — Association between prognostic 14q32 miRNAs and cell line aggressiveness (continuous variable analysis). Spearman correlation coefficients were assigned between 14q32 miRNAs and continuous variables representing cell line aggressiveness (colony forming, invasiveness, migration, and proliferation). (PDF 462 kb) [file 13045_2017_465_MOESM8_ESM.pdf]

Correlation between DICER and differentially expressed miRNAs (Boston dataset)

Correlation coefficient	Parametric p-value	Symbol
0.551	0.0004051	hsa-miR-199a-3p,hsa-miR-199b-3p
0.518	0.0010124	hsa-miR-886-3p
0.512	0.0011861	hsa-miR-199b-5p
0.494	0.0018876	hsa-miR-369-3p
0.484	0.0023956	hsa-miR-337-3p
0.473	0.0030802	hsa-miR-455-3p
0.473	0.0031087	hsa-miR-487b
0.464	0.0038646	hsa-miR-23a
0.457	0.0044276	hsa-miR-27a
0.45	0.0051929	hsa-miR-145
0.448	0.0054356	hsa-miR-411
0.443	0.0060839	hsa-miR-214
0.442	0.0062262	hsa-miR-27b
0.441	0.0063246	hsa-miR-493*
0.44	0.0063736	hsa-miR-99a
0.44	0.0064736	hsa-miR-99a*
0.432	0.0076164	hsa-miR-365
0.432	0.0076571	hsa-miR-455-5p
0.428	0.0083143	hsa-miR-10b
0.427	0.0083825	hsa-miR-495
0.426	0.0086051	hsa-miR-493
0.424	0.0088434	hsa-miR-221
0.421	0.0094831	hsa-miR-432
0.421	0.0095369	hsa-miR-410
0.42	0.0095938	hsa-miR-34a
0.42	0.0096406	HS_192.1
0.418	0.0100955	hsa-miR-199a*:9.1
0.414	0.0108214	hsa-miR-539
0.411	0.0114983	hsa-miR-1274a

0.411	0.0115771	hsa-miR-125b-2*
0.409	0.011977	hsa-miR-199a-5p
0.4	0.0142903	hsa-miR-24
0.395	0.0155882	hsa-miR-154
0.394	0.0157773	hsa-miR-335*
0.391	0.0166191	hsa-miR-34a*
0.388	0.0175863	hsa-miR-22*
0.388	0.0176629	hsa-miR-299-5p
0.387	0.0179592	hsa-let-7f-1*
0.386	0.0182378	hsa-miR-132
0.385	0.0186977	hsa-miR-329
0.382	0.0196313	hsa-miR-99b
0.376	0.0217474	hsa-miR-134
0.375	0.0222936	hsa-miR-212
0.372	0.0234751	hsa-miR-224
0.367	0.0254946	hsa-miR-655
0.366	0.0259018	hsa-miR-222
0.364	0.0265785	hsa-miR-379*
0.36	0.0285325	solexa-8211-102
0.356	0.0307466	hsa-miR-21*
0.356	0.0308499	hsa-miR-143
0.355	0.0308642	hsa-miR-23b
0.355	0.0313021	hsa-miR-214*
0.352	0.0325272	hsa-miR-193a-3p
0.352	0.0325825	hsa-miR-411*
0.351	0.0330262	hsa-miR-376c
0.348	0.0350761	HS_186
0.345	0.0362506	hsa-miR-27b*
0.344	0.03704	hsa-miR-105
0.343	0.037498	hsa-miR-24-2*
0.341	0.038862	hsa-miR-154*

0.341	0.0389552	hsa-miR-379
0.341	0.0391352	hsa-miR-361-5p
0.34	0.0392531	hsa-miR-520h,hsa-miR-520g
0.34	0.0398216	hsa-miR-487a
0.337	0.041103	hsa-miR-23b*
0.337	0.0413742	hsa-miR-152
0.337	0.0413872	hsa-miR-574-3p
0.337	0.0415033	hsa-miR-452*:9.1
0.336	0.0416849	hsa-let-7a*
0.336	0.0417065	hsa-miR-195
0.332	0.0444695	hsa-miR-889
0.332	0.044773	hsa-miR-377
0.332	0.044842	hsa-miR-148b
0.331	0.0456615	hsa-miR-27a*
0.327	0.0481273	hsa-miR-610
0.326	0.0486623	hsa-miR-219-5p
0.326	0.0488594	hsa-miR-1259
-0.325	0.0494985	hsa-miR-193b*
-0.325	0.0498513	hsa-miR-1295
-0.335	0.0424802	HS_54
-0.335	0.0425407	hsa-miR-608
-0.338	0.0405939	hsa-miR-184
-0.339	0.0403152	HS_200
-0.339	0.0403496	HS_166.1
-0.342	0.0379609	hsa-miR-629
-0.344	0.03705	hsa-miR-663b
-0.346	0.0362006	hsa-miR-1268
-0.348	0.0349617	hsa-miR-371-5p
-0.352	0.0327536	hsa-miR-1275
-0.357	0.0301289	hsa-miR-202*
-0.358	0.0295074	HS_284.1

-0.358	0.0297487	hsa-miR-106a:9.1
-0.359	0.0292267	hsa-miR-639
-0.36	0.0288575	hsa-miR-671:9.1
-0.361	0.0282317	HS_176
-0.364	0.0266506	hsa-miR-425*
-0.367	0.0253132	hsa-miR-544
-0.37	0.0241446	HS_147
-0.372	0.0234365	HS_149
-0.372	0.0235737	HS_51
-0.373	0.0230504	HS_76
-0.376	0.0217227	HS_65
-0.376	0.0218529	hsa-miR-15a*
-0.377	0.0212869	HS_94
-0.378	0.0208912	HS_256
-0.379	0.0206463	HS_38.1
-0.379	0.0207778	HS_56
-0.388	0.0175815	HS_287
-0.39	0.0169545	hsa-miR-885-3p
-0.397	0.0149636	hsa-miR-324-5p
-0.4	0.0140629	HS_221
-0.403	0.0133387	solexa-9081-91
-0.405	0.0129172	HS_135
-0.406	0.0125573	HS_18
-0.406	0.0126887	solexa-539-2056
-0.409	0.0119462	hsa-miR-149*
-0.409	0.0119798	hsa-miR-647
-0.412	0.0113536	hsa-miR-659
-0.414	0.0108612	hsa-miR-198
-0.416	0.0104447	HS_159
-0.418	0.0100161	HS_170
-0.419	0.0097719	HS_52

-0.421	0.0094182	hsa-miR-1207-5p
-0.424	0.0089444	HS_168
-0.425	0.0088003	hsa-miR-1307
-0.428	0.0082662	HS_50
-0.43	0.007843	HS_142.1
-0.434	0.0072314	HS_155
-0.435	0.0070915	HS_17
-0.436	0.0070486	hsa-miR-636
-0.437	0.0068387	hsa-miR-583
-0.438	0.00664	HS_154
-0.439	0.0065777	HS_254
-0.441	0.0062568	HS_188
-0.453	0.0048616	hsa-miR-576-3p
-0.453	0.0048879	HS_196.1
-0.475	0.0029435	hsa-miR-183*
-0.48	0.0026235	hsa-miR-218-2*
-0.482	0.0024972	hsa-miR-650
-0.484	0.0024	hsa-miR-92b*
-0.499	0.0016649	HS_78
-0.503	0.0014935	HS_195
-0.505	0.0014208	HS_12
-0.511	0.0012291	hsa-miR-1224-5p
-0.512	0.0012057	hsa-miR-520a-3p
-0.519	0.0009875	hsa-miR-122
-0.53	0.0007468	hsa-miR-625
-0.543	0.0005128	hsa-miR-558
-0.556	0.0003554	HS_25
-0.577	0.0001836	hsa-miR-498
