# Supplementary material for: An imprinted non-coding genomic cluster at 14q32 defines clinically relevant molecular subtypes in osteosarcoma across multiple independent datasets
Source: J Hematol Oncol. 2017 May 15;10:107. doi: 10.1186/s13045-017-0465-4 (PMC5433149; doi:10.1186/s13045-017-0465-4)
Supplement: Supplementary file 9 — Association between DLK1/MEG3 methylation and cell line aggressiveness. The relationship between methylation and cell line aggressiveness was analyzed for methylation site neighboring or on DLK1 and MEG3, both located on 14q32. This analysis was limited by the number of available methylation probes for this region on the Illumina methylation array. Cell line aggressiveness was assessed via colony forming, invasiveness, migration, and tumorigenicity. (PDF 447 kb) [file 13045_2017_465_MOESM9_ESM.pdf]

Correlation prognostic miRNAs and other transcripts (Boston dataset)

miRNA	Correlation	p-value	Gene Name
hsa-miR-323-3p	0.693	2.00E-06	SNORD113-8
	0.579	0.0001753	SNORD114-13
	0.555	0.0003633	SNORD113-3
	0.536	0.0006223	SNORD113-9
	0.437	0.0068449	SNORD113-2
	0.421	0.0094436	C14ORF79
	0.385	0.0185031	MEG3
	0.384	0.0189887	SNORD114-1
	0.382	0.0197998	JAG2
	0.381	0.0199426	TRAF3
	0.375	0.0220378	SCARNA13
	0.364	0.0270083	EVL
	0.354	0.0315423	EML1
	-0.354	0.0317575	SNHG10
	-0.341	0.0390169	LGMN
	0.326	0.0486735	SNORD112

miRNA	Correlation	p-value	Gene Name
hsa-miR-487b	0.533	0.0006756	SNORD113-3
	0.515	0.0011147	SNORD113-9
	0.494	0.0018979	MEG3
	0.486	0.0023221	SNORD113-8
	0.478	0.0027725	EVL
	0.473	0.0031087	DICER1
	0.457	0.0044855	SNORD113-2
	0.422	0.009257	SNORD114-13
	0.417	0.01033	SNORD112
	0.406	0.0126551	SNORD114-1
	0.401	0.0137914	C14ORF79
	0.396	0.0151538	EML1
	-0.392	0.0164407	LGMN
	0.383	0.0191895	PSMC1
	-0.378	0.0209991	SNHG10
	0.373	0.0230511	CCNK
	0.358	0.0294499	SNORD113-6
	-0.356	0.0306338	SLC25A29
	-0.354	0.031421	ASPG
	-0.35	0.0334383	PRIMA1
	-0.349	0.0344163	AK7
	-0.343	0.0375604	MIR342
	0.34	0.0396491	HSP90AA1
	0.34	0.0397558	ANKRD9
	0.337	0.0416714	HSP90AA1
	-0.334	0.0434224	MIR410
	0.331	0.045108	BTBD7
	-0.328	0.0474125	OTUB2
	0.326	0.049201	PSMC1
	0.325	0.0496291	INF2

miRNA	Correlation	p-value	Gene Name
hsa-miR-329	0.537	0.0006151	SNORD113-8
	0.508	0.0013412	SNORD113-3
	0.506	0.0013956	SNORD113-9
	0.49	0.0020922	SNORD113-2
	0.451	0.0050755	SNORD114-13
	0.435	0.0071487	C14ORF79
	0.426	0.0084941	MEG3
	0.412	0.0111836	SNORD112
	0.409	0.0119495	INF2
	-0.39	0.0170369	SNHG10
	0.385	0.0186977	DICER1
	-0.385	0.0187435	LGMN
	0.379	0.0207123	EVL
	0.364	0.0266679	EML1
	0.357	0.0300959	CDCA4
	0.346	0.0357472	SERPINA3
	0.344	0.0368084	HSP90AA1
	0.342	0.037975	CCNK
	0.328	0.0475161	EML1

miRNA	Correlation	p-value	Gene Name
hsa-miR-337-3p	0.521	0.0009493	SNORD113-3
	0.511	0.0012182	SNORD113-8
	0.5	0.0016382	SNORD113-9
	0.484	0.0023956	DICER1
	0.459	0.0042397	SNORD113-2
	-0.448	0.0053901	LGMN
	-0.439	0.0065781	SNHG10
	-0.431	0.0077528	PRIMA1
	0.425	0.0087851	MEG3
	-0.423	0.0090745	MIR342
	-0.413	0.0110765	ASPG
	0.409	0.0119981	SNORD114-13
	-0.395	0.015482	C14ORF177
	0.392	0.0165335	CCNK
	-0.385	0.0184908	AK7
	-0.385	0.0187135	OTUB2
	0.384	0.0190453	C14ORF79
	0.38	0.0203728	EVL
	0.375	0.0221269	AKT1
	0.372	0.0234147	PSMC1
	-0.369	0.0244508	SMEK1
	0.367	0.0253585	PSMC1
	0.363	0.0273051	HSP90AA1
	-0.362	0.0278871	TDP1
	-0.361	0.0280067	MIR541
	0.358	0.029841	PAPOLA
	-0.357	0.0302175	MIR376A2
	0.344	0.0372959	INF2
	0.34	0.0394008	EML1
	-0.34	0.0395773	SNORD114-31
	0.337	0.0413137	SNORD112
	-0.337	0.0413449	SLC25A29
	0.335	0.0429616	ANKRD9
	0.331	0.0452889	PPP1R13B
	0.331	0.0454561	EML1

miRNA	Correlation	p-value	Gene Name
hsa-miR-376a*	0.723	4.00E-07	SNORD113-8
	0.657	9.90E-06	SNORD114-13
	0.59	0.0001209	SNORD113-3
	0.513	0.0011789	MEG3
	0.498	0.001707	SNORD113-9
	0.497	0.0017707	SNORD114-1
	0.348	0.0349416	SNORD113-2
	0.342	0.0383881	JAG2
	0.331	0.0455883	TRAF3

**miRNA**	**Correlation**	**p-value**	**Gene Name**
hsa-miR-379*	0.675	4.70E-06	SNORD114-13
	0.599	9.09E-05	SNORD113-8
	0.522	0.0009254	C14ORF79
	0.506	0.0013881	SNORD113-3
	0.486	0.0022661	MEG3
	0.477	0.0028296	SNORD113-9
	0.433	0.0073705	SNORD113-2
	0.429	0.008043	EML1
	0.418	0.0100604	INF2
	0.393	0.016038	SCARNA13
	0.385	0.01869	SNORD112
	-0.383	0.0194189	SNHG10
	0.379	0.0206948	CDCA4
	-0.375	0.0221269	LGMN
	0.367	0.025632	JAG2
	0.364	0.0265785	DICER1
	0.352	0.032577	BTBD7
	0.35	0.0334934	CCNK
	0.346	0.0358201	SNORD114-1

miRNA	Correlation	p-value	Gene Name
hsa-miR-382	0.574	0.0002044	SNORD113-2
	0.531	0.0007282	SNORD113-9
	0.493	0.0019144	SNORD112
	0.482	0.0025448	SNORD113-8
	0.468	0.0034909	SNORD113-3
	0.452	0.0049602	SNORD114-13
	0.427	0.0083962	C14ORF79
	0.424	0.0088495	JAG2
	0.381	0.020146	SNORD113-5
	0.377	0.0212962	MEG3
	-0.371	0.0238953	MOAP1

miRNA	Correlation	p-value	Gene Name
hsa-miR-410	0.679	3.80E-06	SNORD113-8
	0.614	5.39E-05	SNORD113-3
	0.594	0.0001078	SNORD113-9
	0.566	0.0002622	MEG3
	0.536	0.0006275	SNORD114-1
	0.481	0.0026071	SNORD112
	0.458	0.0043746	SNORD113-2
	0.424	0.0088811	TRAF3
	0.421	0.0095369	DICER1
	0.416	0.0104882	SNORD114-13
	0.384	0.0189902	CCNK
	0.364	0.0268405	BTBD7
	0.35	0.0335462	SNORD114-4
	0.343	0.0374421	C14ORF79
	-0.34	0.0393322	SNHG10
	0.328	0.0474464	HSP90AA1

miRNA	Correlation	p-value	Gene Name
hsa-miR-411*	0.631	2.80E-05	SNORD114-13
	0.6	8.57E-05	SNORD113-8
	0.566	0.0002628	SNORD113-9
	0.556	0.0003538	C14ORF79
	0.467	0.0035609	SNORD113-3
	0.465	0.0037058	JAG2
	0.434	0.0073429	BTBD7
	0.403	0.0132993	INF2
	0.397	0.0150115	MEG3
	0.395	0.0155883	EML1
	0.393	0.016015	SNORD112
	-0.377	0.0214286	LGMN
	-0.364	0.0269842	MIR342
	-0.353	0.0322207	MIR376A2
	0.352	0.0325825	DICER1
	0.348	0.0347913	SCARNA13
	0.348	0.0350254	C14ORF102
	0.344	0.0368407	AKT1
	0.343	0.0377019	SNORD113-2
	-0.337	0.0415382	SNHG10
	0.335	0.042724	SNORD114-1
	0.335	0.0427413	CCNK
	-0.334	0.0432588	C14ORF177
	-0.328	0.0471357	CCDC88C
	-0.328	0.0478552	TDP1
	-0.325	0.0499044	ASPG

miRNA	Correlation	p-value	Gene Name
hsa-miR-432	0.679	3.80E-06	SNORD113-9
	0.655	1.08E-05	SNORD112
	0.593	0.0001109	SNORD113-2
	0.526	0.0008231	SNORD113-8
	0.504	0.0014843	SNORD113-3
	0.494	0.0019129	MEG3
	0.431	0.0076969	C14ORF79
	0.431	0.007735	JAG2
	0.427	0.0083209	EIF5
	0.421	0.0094831	DICER1
	0.414	0.0107923	MIR494
	0.411	0.0114186	EML1
	0.394	0.0157982	TNFAIP2
	0.393	0.0161404	CCNK
	0.388	0.0176882	EIF5
	0.375	0.0222573	SERPINA3
	0.352	0.0328499	BTBD7
	0.347	0.0351333	HSP90AA1
	-0.342	0.0380081	PRIMA1
	0.342	0.038149	INF2
	0.332	0.0450095	EVL

miRNA	Correlation	p-value	Gene Name
hsa-miR-485-3p	0.541	0.0005465	SNORD114-13
	0.494	0.0018866	SNORD113-9
	0.494	0.0018872	SNORD113-8
	0.461	0.0040978	SNORD113-2
	0.435	0.0071428	SNORD113-3
	0.427	0.0083488	JAG2
	0.401	0.0138669	C14ORF79
	0.391	0.0166082	INF2
	0.366	0.0259731	EML1
	-0.333	0.0440437	LGMN

miRNA	Correlation	p-value	Gene Name
hsa-miR-487a	0.71	9.00E-07	SNORD113-8
	0.611	5.98E-05	SNORD114-13
	0.591	0.0001174	SNORD113-9
	0.567	0.0002552	SNORD113-3
	0.5	0.0016121	MEG3
	0.455	0.0046736	SNORD113-2
	0.451	0.0050674	SNORD114-1
	0.436	0.0069089	BTBD7
	0.434	0.0072278	C14ORF79
	0.42	0.0096417	SNORD112
	0.396	0.0153847	TRAF3
	0.382	0.0195747	CCNK
	0.379	0.0207831	JAG2
	0.378	0.0212432	SCARNA13
	0.361	0.0281357	SNORD114-4
	0.34	0.0398216	DICER1
	0.329	0.0466853	EML1

miRNA	Correlation	p-value	Gene Name
hsa-miR-493*	0.56	0.0003113	C14ORF79
	0.53	0.0007403	EVL
	0.517	0.0010459	AKT1
	-0.497	0.0017354	LGMN
	0.474	0.0030716	INF2
	0.468	0.0034912	SNORD113-9
	0.468	0.0034924	ANKRD9
	0.468	0.0035276	HSP90AA1
	0.458	0.0043701	PAPOLA
	-0.454	0.0047951	TDP1
	0.449	0.005345	JAG2
	0.445	0.0058167	IFI27
	0.444	0.0059324	CDCA4
	-0.443	0.006042	MIR381
	0.443	0.006057	CRIP2
	0.441	0.0063246	DICER1
	-0.43	0.007934	SLC25A29
	0.42	0.0096938	CCNK
	-0.419	0.0098269	CLMN
	-0.418	0.0100715	OTUB2
	-0.412	0.011205	MIR376A2
	0.412	0.0112656	WDR20
	0.412	0.0112701	PSMC1
	0.41	0.0118087	WARS
	-0.405	0.0129999	JAG2
	-0.402	0.0135206	MIR342
	0.397	0.0150197	EML1
	-0.397	0.015092	AK7
	0.392	0.0165528	C14ORF102
	-0.387	0.0180619	AMN
	-0.385	0.0186201	MIR541
	0.373	0.0228063	SETD3
	-0.371	0.023933	MIR655
	-0.368	0.0250945	UBR7
	0.365	0.0264623	C14ORF159
	-0.363	0.0273747	PRIMA1
	-0.359	0.0290981	MIR376A1
	-0.358	0.029719	MIR410
	-0.358	0.0298529	C14ORF177
	0.356	0.0303229	BAG5
	-0.354	0.0314951	CLMN

-0.348	0.0348383	SERPINA1
0.348	0.0349767	CRIP1
-0.347	0.0353646	AKT1
0.343	0.0376467	PSMC1
0.341	0.0386759	BTBD7
0.334	0.0430875	C14ORF80
0.329	0.0466885	SERPINA1
0.328	0.0478342	IFI27L2
-0.327	0.0481088	SNHG10
-0.325	0.0495456	SNORD113-1

miRNA	Correlation	p-value	Gene Name
hsa-miR-495	0.562	0.0002929	SNORD113-8
	0.546	0.0004708	SNORD113-9
	0.539	0.0005777	SNORD113-3
	0.497	0.0017543	SNORD114-13
	0.488	0.0021939	C14ORF79
	0.474	0.0030203	MEG3
	0.44	0.00649	SNORD113-2
	0.427	0.0083825	DICER1
	0.424	0.0089313	INF2
	0.418	0.0101369	SNORD112
	0.411	0.0114544	SERPINA3
	0.408	0.0122652	EML1
	-0.406	0.0127722	ASPG
	-0.402	0.013539	LGMN
	0.402	0.0136631	CCNK
	0.381	0.0199184	SCARNA13
	-0.381	0.0201075	SNHG10
	0.377	0.0215124	BTBD7
	-0.369	0.0245998	PRIMA1
	0.364	0.0268863	AKT1
	0.363	0.0273872	EVL
	0.36	0.0287214	JAG2
	0.34	0.0394014	ANKRD9
	0.338	0.0405357	TDP1
	-0.337	0.0411147	C14ORF177
	-0.337	0.0415408	AK7
	-0.334	0.0430172	MIR342

miRNA	Correlation	p-value	Gene Name
hsa-miR-539	0.669	6.10E-06	SNORD114-13
	0.621	4.17E-05	SNORD113-8
	0.533	0.0006876	SNORD113-3
	0.508	0.0013401	SNORD113-9
	0.493	0.0019512	MEG3
	0.488	0.0021964	SNORD114-1
	0.486	0.0023157	BTBD7
	0.434	0.0072619	C14ORF79
	0.42	0.0096672	SCARNA13
	0.418	0.0101196	CCNK
	0.414	0.0108214	DICER1
	0.38	0.0203812	BAG5
	0.379	0.0205879	TRAF3
	-0.363	0.0272598	ASPG
	0.36	0.0283913	TDP1
	-0.342	0.0382094	PRIMA1
	-0.339	0.0399925	UBR7
	0.338	0.041025	EML1
	0.335	0.0426591	CRIP2
	0.331	0.0455958	SERPINA3
	-0.327	0.0481072	C14ORF177
	-0.326	0.0489475	MIR342
	0.325	0.0498515	SNORD113-2

miRNA	Correlation	p-value	Gene Name
hsa-miR-655	0.615	5.13E-05	SNORD114-13
	0.555	0.0003632	SNORD113-8
	0.484	0.00238	SNORD113-3
	0.45	0.0052048	MEG3
	-0.435	0.0071371	SNHG10
	0.428	0.0081995	C14ORF79
	0.427	0.0084141	SNORD113-9
	-0.405	0.0129053	LGMN
	0.367	0.0254946	DICER1
	0.366	0.0260105	SNORD114-1
	0.362	0.0277783	SNORD113-2
	0.357	0.0299656	EML1
	0.356	0.0304341	CCNK
	0.356	0.0305881	SCARNA13
	-0.333	0.0443046	ASPG
	-0.332	0.0450306	PRIMA1

miRNA	Correlation	p-value	Gene Name
hsa-miR-656	0.606	7.17E-05	SNORD114-13
	0.537	0.00061	MEG3
	0.506	0.0013971	SNORD113-3
	0.486	0.0022884	SNORD113-8
	0.455	0.0046914	SNORD113-2
	0.432	0.0076107	C14ORF79
	0.405	0.012972	SNORD113-9
	0.373	0.0231257	SNORD112
	0.371	0.0237479	SNORD114-1
	0.343	0.0379339	EML1
	0.332	0.044658	INF2

miRNA	Correlation	p-value	Gene Name
hsa-miR-889	0.482	0.0024987	SNORD114-13
	0.477	0.002848	SNORD113-9
	0.438	0.006722	SNORD113-2
	0.437	0.006876	SNORD113-8
	0.425	0.0088066	C14ORF79
	0.419	0.0097632	AKT1
	0.386	0.0183409	INF2
	-0.38	0.0203329	ASPG
	-0.376	0.0219733	MIR541
	0.375	0.0221473	SNORD113-3
	-0.368	0.0251128	MIR342
	-0.363	0.0273933	CCDC88C
	-0.362	0.0275965	AK7
	0.362	0.027852	EVL
	0.361	0.0282498	CDCA4
	-0.358	0.0296487	JAG2
	-0.352	0.0324125	SLC25A29
	-0.351	0.0330537	AMN
	0.347	0.0354015	PAPOLA
	-0.345	0.0367474	OTUB2
	-0.343	0.0374524	MIR376A2
	-0.342	0.0381421	LGMN
	0.342	0.0385832	WDR20
	-0.341	0.0389143	PRIMA1
	-0.337	0.0411654	C14ORF177
	-0.337	0.0416175	TDP1
	0.335	0.0425453	SERPINA1
	0.332	0.0444696	DICER1
	0.331	0.0456143	HSP90AA1
	0.329	0.0468032	SERPINA3
	0.328	0.0474426	PSMC1
	-0.325	0.0493697	CINP
