# Supplementary material for: An imprinted non-coding genomic cluster at 14q32 defines clinically relevant molecular subtypes in osteosarcoma across multiple independent datasets
Source: J Hematol Oncol. 2017 May 15;10:107. doi: 10.1186/s13045-017-0465-4 (PMC5433149; doi:10.1186/s13045-017-0465-4)
Supplement: Supplementary file 10 — Network edge differences between the two subtypes. (PDF 557 kb) [file 13045_2017_465_MOESM10_ESM.pdf]

Association between prognostic 14q32 miRNAs and cell line aggressiveness (binary analysis)

Parametric p-value	Fold-change	Symbol
0.0149805	2.13	hsa-miR-889
0.0243932	5.95	hsa-miR-411
0.0266654	5.22	hsa-miR-432
0.0300115	5.43	hsa-miR-495
0.03085	9.07	hsa-miR-495
0.0392378	3.55	hsa-miR-432
0.0508013	4.78	hsa-miR-337-5p
0.0513636	6.29	hsa-miR-337-5p
0.0521939	2.93	hsa-miR-323-3p
0.0565571	1.47	hsa-miR-379*
0.0578192	3.54	hsa-miR-329
0.0600058	2.73	hsa-miR-485-3p
0.0682584	3.36	hsa-miR-337-3p
0.0725069	1.82	hsa-miR-656
0.0929138	3.8	hsa-miR-299-5p
0.0969597	3.9	hsa-miR-410

Proliferation

Parametric p-value	Fold-change	Symbol
0.0149805	2.13	hsa-miR-889
0.0243932	5.95	hsa-miR-411
0.0266654	5.22	hsa-miR-432
0.0300115	5.43	hsa-miR-495
0.03085	9.07	hsa-miR-495
0.0392378	3.55	hsa-miR-432
0.0508013	4.78	hsa-miR-337-5p
0.0513636	6.29	hsa-miR-337-5p
0.0521939	2.93	hsa-miR-323-3p
0.0565571	1.47	hsa-miR-379*
0.0578192	3.54	hsa-miR-329
0.0600058	2.73	hsa-miR-485-3p
0.0682584	3.36	hsa-miR-337-3p
0.0725069	1.82	hsa-miR-656
0.0929138	3.8	hsa-miR-299-5p
0.0969597	3.9	hsa-miR-410

Proliferation

Parametric p-value	Fold-change	Symbol
0.0265439	4.22	hsa-miR-337-3p
0.0437058	3.8	hsa-miR-329
0.0632638	4.11	hsa-miR-432
0.0652466	4.33	hsa-miR-495
0.0657481	1.81	hsa-miR-889
0.0675384	6.71	hsa-miR-495
0.0692734	2.66	hsa-miR-337-3p
0.0734589	0.47	hsa-miR-411*
0.0811039	4.15	hsa-miR-411
0.0846585	4.05	hsa-miR-337-5p
0.0869193	1.49	hsa-miR-134
0.0988631	2.82	hsa-miR-432

Invasion

Parametric p-value	Fold-change	Symbol
0.0290295	0.4	hsa-miR-411*
0.0459386	2.1	hsa-miR-369-3p
0.076625	0.68	hsa-miR-493
0.0873662	1.52	hsa-miR-539
0.0920616	2.98	hsa-miR-432

Migration

Parametric p-value	Fold-change	Symbol
0.009654	1.53	hsa-miR-487b
0.0125473	1.62	hsa-miR-379*
0.0625324	1.37	hsa-miR-487b

Colony-Forming

Parametric p-value	Fold-change	Symbol
0.0318003	1.79	hsa-miR-487a
0.0467052	1.47	hsa-miR-487a
0.0769531	1.36	hsa-miR-487b

Tumorigenicity

Parametric p-value	Fold-change	Symbol
0.0290295	0.4	hsa-miR-411*
0.0459386	2.1	hsa-miR-369-3p
0.076625	0.68	hsa-miR-493
0.0873662	1.52	hsa-miR-539
0.0920616	2.98	hsa-miR-432

Migration

Parametric p-value	Fold-change	Symbol
0.009654	1.53	hsa-miR-487b
0.0125473	1.62	hsa-miR-379*
0.0625324	1.37	hsa-miR-487b

Colony-Forming

Parametric p-value	Fold-change	Symbol
0.0318003	1.79	hsa-miR-487a
0.0467052	1.47	hsa-miR-487a
0.0769531	1.36	hsa-miR-487b

Tumorigenicity
