# Supplementary material for: An imprinted non-coding genomic cluster at 14q32 defines clinically relevant molecular subtypes in osteosarcoma across multiple independent datasets
Source: J Hematol Oncol. 2017 May 15;10:107. doi: 10.1186/s13045-017-0465-4 (PMC5433149; doi:10.1186/s13045-017-0465-4)
Supplement: Supplementary file 11 — Significant network GO categories. (PDF 412 kb) [file 13045_2017_465_MOESM11_ESM.pdf]

Association between prognostic 14q32 miRNAs and cell line aggressiveness (continuous variable analysis)

Correlation coefficient	Parametric p-value	Symbol
0.653	0.0031178	hsa-miR-379*
0.618	0.0058023	hsa-miR-487b
0.544	0.0176169	hsa-miR-487b
-0.388	0.1018892	hsa-miR-379*
0.304	0.2059872	hsa-miR-487a
-0.297	0.2170791	hsa-miR-539
0.285	0.234456	hsa-miR-337-5p
0.276	0.24962	hsa-miR-409-3p
0.268	0.2654093	hsa-miR-409-3p
0.266	0.2686424	hsa-miR-323-3p
-0.26	0.2818264	hsa-miR-493
0.246	0.3094012	hsa-miR-889
0.241	0.316546	hsa-miR-342-3p
0.237	0.3274511	hsa-miR-342-3p
0.226	0.3499342	hsa-miR-299-5p
-0.223	0.357627	hsa-miR-485-3p
0.216	0.3733075	hsa-miR-493
0.213	0.3772889	hsa-miR-134
-0.212	0.3812945	hsa-miR-539
0.211	0.3853245	hsa-miR-889
0.208	0.3893786	hsa-miR-495
-0.204	0.3975592	hsa-miR-493*
0.202	0.4058358	hsa-miR-495
0.198	0.4142076	hsa-miR-656
0.184	0.4486335	hsa-miR-382
-0.177	0.4663982	hsa-miR-889
-0.171	0.4799573	hsa-miR-493*
0.17	0.4845211	hsa-miR-656
0.16	0.5123594	hsa-miR-485-3p

Spearman coefficients

Colony-forming

0.159	0.5170736	hsa-miR-411*
0.155	0.5265647	hsa-miR-493
-0.153	0.5313412	hsa-miR-369-3p
0.142	0.560424	hsa-miR-411*
0.141	0.560424	hsa-miR-487a
0.133	0.5851965	hsa-miR-337-3p
0.131	0.5902074	hsa-miR-410
0.118	0.6309412	hsa-miR-411
0.118	0.6309412	hsa-miR-134
0.114	0.6412971	hsa-miR-411
-0.106	0.6674706	hsa-miR-376a*
-0.104	0.6727522	hsa-miR-299-5p
0.087	0.7209422	hsa-miR-432
-0.088	0.7209422	hsa-miR-493
0.081	0.7427106	hsa-miR-329
-0.079	0.7481837	hsa-miR-323-3p
0.076	0.7536687	hsa-miR-432
0.048	0.8484919	hsa-miR-154*
0.045	0.8598111	hsa-miR-377
-0.042	0.8654809	hsa-miR-889
-0.035	0.8882225	hsa-miR-329
0.03	0.9053363	hsa-miR-337-5p
0.027	0.916769	hsa-miR-539
0.014	0.9568957	hsa-miR-376a*
0.008	0.9741299	hsa-miR-154*
-0.005	0.9856261	hsa-miR-377
-0.005	0.9856261	hsa-miR-337-3p

Correlation coefficient	Parametric p-value	Symbol
-0.54	0.0184736	hsa-miR-493
-0.418	0.0765833	hsa-miR-411*
0.405	0.0863514	hsa-miR-134
-0.344	0.1496485	hsa-miR-379*
0.331	0.1654807	hsa-miR-369-3p
0.308	0.1979246	hsa-miR-656
0.298	0.2142694	hsa-miR-410
-0.281	0.2434796	hsa-miR-299-5p
0.274	0.2558605	hsa-miR-376a*
0.265	0.2719007	hsa-miR-337-3p
-0.265	0.2719007	hsa-miR-485-3p
0.26	0.2818264	hsa-miR-154*
0.241	0.316546	hsa-miR-134
-0.233	0.334846	hsa-miR-409-3p
0.223	0.357627	hsa-miR-337-5p
0.218	0.3654182	hsa-miR-487a
0.213	0.3772889	hsa-miR-342-3p
-0.209	0.3893786	hsa-miR-409-3p
-0.201	0.4058358	hsa-miR-323-3p
0.198	0.4142076	hsa-miR-495
-0.196	0.4184291	hsa-miR-411*
0.196	0.4184291	hsa-miR-495
-0.191	0.4312344	hsa-miR-493
0.184	0.4486335	hsa-miR-889
0.179	0.4619231	hsa-miR-539
0.172	0.4799573	hsa-miR-379*
-0.172	0.4799573	hsa-miR-411
0.17	0.4845211	hsa-miR-410
0.165	0.4983436	hsa-miR-376a*

Spearman coefficients
Invasion

0.16	0.5123594	hsa-miR-493
0.151	0.5361381	hsa-miR-493*
0.146	0.5457928	hsa-miR-539
0.147	0.5457928	hsa-miR-889
-0.133	0.5851965	hsa-miR-323-3p
0.13	0.5952367	hsa-miR-329
0.127	0.6053498	hsa-miR-539
0.124	0.6104332	hsa-miR-432
0.121	0.6206528	hsa-miR-337-3p
0.122	0.6206528	hsa-miR-493
-0.12	0.6257885	hsa-miR-382
-0.103	0.6727522	hsa-miR-487b
0.097	0.6886872	hsa-miR-487a
0.092	0.7047526	hsa-miR-342-3p
0.09	0.7155323	hsa-miR-889
0.08	0.7427106	hsa-miR-377
-0.079	0.7481837	hsa-miR-377
0.069	0.7757227	hsa-miR-299-5p
0.045	0.854148	hsa-miR-487b
0.044	0.8598111	hsa-miR-432
0.039	0.8768398	hsa-miR-411
-0.036	0.8825283	hsa-miR-485-3p
-0.037	0.8825283	hsa-miR-656
0.034	0.893922	hsa-miR-337-5p
0.025	0.9224915	hsa-miR-154*
-0.022	0.9282177	hsa-miR-329
0.014	0.9568957	hsa-miR-493*
0.005	0.9856261	hsa-miR-382

Correlation coefficient	Parametric p-value	Symbol
-0.453	0.0532552	hsa-miR-493
-0.39	0.1002485	hsa-miR-411*
0.332	0.1654807	hsa-miR-889
0.302	0.2087235	hsa-miR-369-3p
-0.301	0.2087235	hsa-miR-379*
0.288	0.2314979	hsa-miR-134
0.284	0.237439	hsa-miR-337-3p
0.263	0.2751841	hsa-miR-487a
0.255	0.2919784	hsa-miR-495
0.251	0.2988721	hsa-miR-337-5p
0.24	0.320156	hsa-miR-379*
0.238	0.3237911	hsa-miR-495
0.236	0.3274511	hsa-miR-376a*
0.235	0.3311361	hsa-miR-493*
0.225	0.3537683	hsa-miR-410
0.219	0.3654182	hsa-miR-493
0.219	0.3654182	hsa-miR-656
0.219	0.3654182	hsa-miR-154*
-0.212	0.3812945	hsa-miR-299-5p
-0.188	0.4398878	hsa-miR-409-3p
-0.184	0.4486335	hsa-miR-409-3p
0.184	0.4486335	hsa-miR-410
0.174	0.4754154	hsa-miR-889
-0.169	0.4891069	hsa-miR-493
0.151	0.5361381	hsa-miR-432
0.144	0.5555273	hsa-miR-329
0.14	0.5653403	hsa-miR-134
-0.139	0.5702758	hsa-miR-485-3p
0.137	0.5752305	hsa-miR-432

Spearman coefficients
Migration

0.134	0.5851965	hsa-miR-539
0.128	0.6002843	hsa-miR-411
0.128	0.6002843	hsa-miR-493
-0.123	0.6155343	hsa-miR-889
0.119	0.6257885	hsa-miR-337-3p
0.118	0.6309412	hsa-miR-487b
-0.107	0.6622043	hsa-miR-323-3p
0.108	0.6622043	hsa-miR-376a*
0.101	0.678049	hsa-miR-337-5p
-0.1	0.6833608	hsa-miR-382
0.096	0.6940282	hsa-miR-342-3p
0.094	0.7047526	hsa-miR-377
0.077	0.7536687	hsa-miR-539
-0.075	0.7591653	hsa-miR-377
0.071	0.7701926	hsa-miR-539
0.068	0.7812635	hsa-miR-493*
0.062	0.7979473	hsa-miR-889
0.06	0.8091186	hsa-miR-299-5p
0.058	0.814718	hsa-miR-487a
-0.054	0.8259433	hsa-miR-656
-0.05	0.8428432	hsa-miR-411
-0.043	0.8598111	hsa-miR-323-3p
0.04	0.8711572	hsa-miR-329
-0.035	0.8882225	hsa-miR-411*
0.03	0.9053363	hsa-miR-154*
0.023	0.9282177	hsa-miR-485-3p
-0.02	0.9396804	hsa-miR-382
0.019	0.9396804	hsa-miR-487b
-0.012	0.9626387	hsa-miR-342-3p

Correlation coefficient	Parametric p-value	Symbol
0.646	0.0035506	hsa-miR-379*
0.604	0.0073026	hsa-miR-379*
-0.582	0.0101341	hsa-miR-345
0.568	0.0124753	hsa-miR-889
-0.53	0.0212465	hsa-miR-377*
0.526	0.0222416	hsa-miR-337-5p
0.473	0.0421715	hsa-miR-411
0.462	0.0474618	hsa-miR-654-3p
0.453	0.0532552	hsa-miR-487b
0.451	0.0542717	hsa-miR-485-5p
0.449	0.0553032	hsa-miR-376c
0.45	0.0553032	hsa-miR-379
0.426	0.0701356	hsa-miR-380*
0.421	0.0739525	hsa-miR-380
0.412	0.0806616	hsa-miR-136*
0.412	0.0806616	hsa-miR-495
0.41	0.0820568	hsa-miR-136*
0.41	0.0820568	hsa-miR-495
-0.403	0.0878198	hsa-miR-432*
0.4	0.0908126	hsa-miR-411*
0.396	0.0954438	hsa-miR-337-5p
0.394	0.0954438	hsa-miR-376c
0.389	0.1002485	hsa-miR-376a
0.389	0.1002485	hsa-miR-376b
0.39	0.1002485	hsa-miR-485-3p
0.384	0.1052301	hsa-miR-758
0.382	0.1069306	hsa-miR-380
0.38	0.1086512	hsa-miR-337-3p
0.378	0.1103921	hsa-miR-299-5p

Spearman coefficients
Proliferation

0.379	0.1103921	hsa-miR-656
0.375	0.1139352	hsa-miR-381
-0.375	0.1139352	hsa-miR-665
0.369	0.1212701	hsa-miR-493
0.367	0.1231563	hsa-miR-758
0.351	0.1411006	hsa-miR-136
0.331	0.1654807	hsa-miR-376a
0.323	0.1774847	hsa-miR-323-3p
0.322	0.1774847	hsa-miR-329
0.316	0.1875131	hsa-miR-377
0.316	0.1875131	hsa-miR-485-5p
0.312	0.1926707	hsa-miR-409-3p
0.31	0.1952856	hsa-miR-654-5p
0.309	0.1979246	hsa-miR-758
0.305	0.2032754	hsa-miR-127-3p
0.305	0.2032754	hsa-miR-487b
-0.303	0.2059872	hsa-miR-300
0.304	0.2059872	hsa-miR-323-3p
0.302	0.2087235	hsa-miR-433
0.302	0.2087235	hsa-miR-485-5p
0.302	0.2087235	hsa-miR-543
-0.301	0.2087235	hsa-miR-544
0.3	0.2114842	hsa-miR-376a*
0.299	0.2114842	hsa-miR-411
0.3	0.2114842	hsa-miR-758
0.3	0.2114842	hsa-miR-889
0.296	0.2170791	hsa-miR-379*
-0.296	0.2170791	hsa-miR-431*
0.29	0.2285647	hsa-miR-342-5p
0.288	0.2314979	hsa-miR-409-3p
0.287	0.234456	hsa-miR-410

0.284	0.237439	hsa-miR-432
0.282	0.2404468	hsa-miR-337-3p
0.283	0.2404468	hsa-miR-889
0.279	0.2465373	hsa-miR-431
0.279	0.2465373	hsa-miR-432
-0.279	0.2465373	hsa-miR-541*
0.276	0.24962	hsa-miR-154*
0.278	0.24962	hsa-miR-154*
0.278	0.24962	hsa-miR-376b
0.275	0.2527277	hsa-miR-493*
-0.274	0.2558605	hsa-miR-431*
0.268	0.2654093	hsa-miR-136
0.269	0.2654093	hsa-miR-409-5p
0.258	0.2851852	hsa-miR-377*
0.25	0.2988721	hsa-miR-379
0.249	0.3023567	hsa-miR-485-3p
0.244	0.3129611	hsa-miR-382
0.24	0.320156	hsa-miR-539
0.239	0.3237911	hsa-miR-370
0.238	0.3237911	hsa-miR-411*
0.235	0.3311361	hsa-miR-134
0.233	0.334846	hsa-miR-377
0.23	0.3423405	hsa-miR-431
0.228	0.346125	hsa-miR-134
0.228	0.346125	hsa-miR-154
0.228	0.346125	hsa-miR-369-3p
0.228	0.346125	hsa-miR-377*
-0.226	0.3499342	hsa-miR-494
-0.224	0.3537683	hsa-miR-127-5p
0.225	0.3537683	hsa-miR-381
0.223	0.357627	hsa-miR-487a

-0.221	0.3615103	hsa-miR-431*
-0.221	0.3615103	hsa-miR-494
0.221	0.3615103	hsa-miR-543
0.218	0.3693506	hsa-miR-411*
-0.218	0.3693506	hsa-miR-431*
-0.214	0.3772889	hsa-miR-665
0.213	0.3812945	hsa-miR-369-5p
0.211	0.3853245	hsa-miR-329
0.205	0.3975592	hsa-miR-127-3p
-0.202	0.4058358	hsa-miR-345
-0.202	0.4058358	hsa-miR-412
0.197	0.4142076	hsa-miR-127-5p
0.196	0.4184291	hsa-miR-410
-0.188	0.4398878	hsa-miR-432*
-0.186	0.4442492	hsa-miR-541*
0.184	0.4486335	hsa-miR-300
0.184	0.4486335	hsa-miR-323-5p
0.18	0.4574706	hsa-miR-323-5p
-0.18	0.4574706	hsa-miR-541
-0.181	0.4574706	hsa-miR-656
0.177	0.4663982	hsa-miR-433
-0.172	0.4799573	hsa-miR-379*
0.172	0.4799573	hsa-miR-544
-0.169	0.4845211	hsa-miR-342-5p
-0.169	0.4891069	hsa-miR-412
0.168	0.4891069	hsa-miR-541
0.167	0.4937144	hsa-miR-299-5p
0.164	0.4983436	hsa-miR-299-3p
0.157	0.5170736	hsa-miR-376a*
0.148	0.5457928	hsa-miR-453
0.146	0.5506501	hsa-miR-654-3p

-0.143	0.5555273	hsa-miR-300
0.144	0.5555273	hsa-miR-487a
0.138	0.5702758	hsa-miR-889
-0.138	0.5702758	hsa-miR-203
0.138	0.5702758	hsa-miR-323-5p
0.133	0.5851965	hsa-miR-299-3p
-0.131	0.5902074	hsa-miR-453
0.124	0.6155343	hsa-miR-154
0.123	0.6155343	hsa-miR-412
0.111	0.6517186	hsa-miR-654-5p
0.111	0.6517186	hsa-miR-668
0.102	0.678049	hsa-miR-382
0.1	0.6833608	hsa-miR-453
-0.098	0.6886872	hsa-miR-770-5p
0.095	0.6993834	hsa-miR-493
-0.09	0.7155323	hsa-miR-345
-0.09	0.7155323	hsa-miR-493
0.086	0.7263653	hsa-miR-380*
0.086	0.7263653	hsa-miR-409-5p
0.081	0.7427106	hsa-miR-654-5p
0.077	0.7536687	hsa-miR-539
0.07	0.7757227	hsa-miR-300
-0.068	0.7812635	hsa-miR-370
0.066	0.7868147	hsa-miR-544
0.065	0.7923761	hsa-miR-432*
0.062	0.8035283	hsa-miR-127-5p
-0.061	0.8035283	hsa-miR-412
-0.061	0.8035283	hsa-miR-668
0.06	0.8091186	hsa-miR-409-5p
0.059	0.8091186	hsa-miR-541
0.052	0.8372019	hsa-miR-493

-0.048	0.8428432	hsa-miR-409-5p
-0.047	0.8484919	hsa-miR-377*
-0.044	0.8598111	hsa-miR-345
-0.037	0.8825283	hsa-miR-342-3p
-0.035	0.8882225	hsa-miR-369-5p
0.032	0.8996268	hsa-miR-665
-0.032	0.8996268	hsa-miR-770-5p
-0.03	0.9053363	hsa-miR-342-3p
0.03	0.9053363	hsa-miR-654-5p
0.026	0.916769	hsa-miR-493*
0.026	0.916769	hsa-miR-496
0.026	0.916769	hsa-miR-665
-0.022	0.9339475	hsa-miR-380*
-0.016	0.9511548	hsa-miR-770-5p
0.01	0.9683836	hsa-miR-770-5p
-0.009	0.9741299	hsa-miR-411*
-0.01	0.9741299	hsa-miR-432*
0.007	0.9798775	hsa-miR-541*
0.007	0.9798775	hsa-miR-541*
0.004	0.9856261	hsa-miR-323-5p
0.006	0.9856261	hsa-miR-539
-0.005	0.9856261	hsa-miR-668
0.004	0.9913754	hsa-miR-541
-0.003	0.9913754	hsa-miR-668
-0.002	0.9971251	hsa-miR-203

Spearman coefficients
Colony-forming

Correlation coefficient	Parametric p-value	Symbol
0.653	0.0031178	hsa-miR-379*
0.618	0.0058023	hsa-miR-487b
0.544	0.0176169	hsa-miR-487b
-0.388	0.1018892	hsa-miR-379*
0.304	0.2059872	hsa-miR-487a
-0.297	0.2170791	hsa-miR-539
0.285	0.234456	hsa-miR-337-5p
0.276	0.24962	hsa-miR-409-3p
0.268	0.2654093	hsa-miR-409-3p
0.266	0.2686424	hsa-miR-323-3p
-0.26	0.2818264	hsa-miR-493
0.246	0.3094012	hsa-miR-889
0.241	0.316546	hsa-miR-342-3p
0.237	0.3274511	hsa-miR-342-3p
0.226	0.3499342	hsa-miR-299-5p
-0.223	0.357627	hsa-miR-485-3p
0.216	0.3733075	hsa-miR-493
0.213	0.3772889	hsa-miR-134
-0.212	0.3812945	hsa-miR-539
0.211	0.3853245	hsa-miR-889
0.208	0.3893786	hsa-miR-495
-0.204	0.3975592	hsa-miR-493*
0.202	0.4058358	hsa-miR-495
0.198	0.4142076	hsa-miR-656
0.184	0.4486335	hsa-miR-382
-0.177	0.4663982	hsa-miR-889
-0.171	0.4799573	hsa-miR-493*
0.17	0.4845211	hsa-miR-656
0.16	0.5123594	hsa-miR-485-3p

0.159	0.5170736	hsa-miR-411*
0.155	0.5265647	hsa-miR-493
-0.153	0.5313412	hsa-miR-369-3p
0.142	0.560424	hsa-miR-411*
0.141	0.560424	hsa-miR-487a
0.133	0.5851965	hsa-miR-337-3p
0.131	0.5902074	hsa-miR-410
0.118	0.6309412	hsa-miR-411
0.118	0.6309412	hsa-miR-134
0.114	0.6412971	hsa-miR-411
-0.106	0.6674706	hsa-miR-376a*
-0.104	0.6727522	hsa-miR-299-5p
0.087	0.7209422	hsa-miR-432
-0.088	0.7209422	hsa-miR-493
0.081	0.7427106	hsa-miR-329
-0.079	0.7481837	hsa-miR-323-3p
0.076	0.7536687	hsa-miR-432
0.048	0.8484919	hsa-miR-154*
0.045	0.8598111	hsa-miR-377
-0.042	0.8654809	hsa-miR-889
-0.035	0.8882225	hsa-miR-329
0.03	0.9053363	hsa-miR-337-5p
0.027	0.916769	hsa-miR-539
0.014	0.9568957	hsa-miR-376a*
0.008	0.9741299	hsa-miR-154*
-0.005	0.9856261	hsa-miR-377
-0.005	0.9856261	hsa-miR-337-3p

Correlation coefficient	Parametric p-value	Symbol
-0.54	0.0184736	hsa-miR-493
-0.418	0.0765833	hsa-miR-411*
0.405	0.0863514	hsa-miR-134
-0.344	0.1496485	hsa-miR-379*
0.331	0.1654807	hsa-miR-369-3p
0.308	0.1979246	hsa-miR-656
0.298	0.2142694	hsa-miR-410
-0.281	0.2434796	hsa-miR-299-5p
0.274	0.2558605	hsa-miR-376a*
0.265	0.2719007	hsa-miR-337-3p
-0.265	0.2719007	hsa-miR-485-3p
0.26	0.2818264	hsa-miR-154*
0.241	0.316546	hsa-miR-134
-0.233	0.334846	hsa-miR-409-3p
0.223	0.357627	hsa-miR-337-5p
0.218	0.3654182	hsa-miR-487a
0.213	0.3772889	hsa-miR-342-3p
-0.209	0.3893786	hsa-miR-409-3p
-0.201	0.4058358	hsa-miR-323-3p
0.198	0.4142076	hsa-miR-495
-0.196	0.4184291	hsa-miR-411*
0.196	0.4184291	hsa-miR-495
-0.191	0.4312344	hsa-miR-493
0.184	0.4486335	hsa-miR-889
0.179	0.4619231	hsa-miR-539
0.172	0.4799573	hsa-miR-379*
-0.172	0.4799573	hsa-miR-411
0.17	0.4845211	hsa-miR-410
0.165	0.4983436	hsa-miR-376a*

Spearman coefficients
Invasion

0.16	0.5123594	hsa-miR-493
0.151	0.5361381	hsa-miR-493*
0.146	0.5457928	hsa-miR-539
0.147	0.5457928	hsa-miR-889
-0.133	0.5851965	hsa-miR-323-3p
0.13	0.5952367	hsa-miR-329
0.127	0.6053498	hsa-miR-539
0.124	0.6104332	hsa-miR-432
0.121	0.6206528	hsa-miR-337-3p
0.122	0.6206528	hsa-miR-493
-0.12	0.6257885	hsa-miR-382
-0.103	0.6727522	hsa-miR-487b
0.097	0.6886872	hsa-miR-487a
0.092	0.7047526	hsa-miR-342-3p
0.09	0.7155323	hsa-miR-889
0.08	0.7427106	hsa-miR-377
-0.079	0.7481837	hsa-miR-377
0.069	0.7757227	hsa-miR-299-5p
0.045	0.854148	hsa-miR-487b
0.044	0.8598111	hsa-miR-432
0.039	0.8768398	hsa-miR-411
-0.036	0.8825283	hsa-miR-485-3p
-0.037	0.8825283	hsa-miR-656
0.034	0.893922	hsa-miR-337-5p
0.025	0.9224915	hsa-miR-154*
-0.022	0.9282177	hsa-miR-329
0.014	0.9568957	hsa-miR-493*
0.005	0.9856261	hsa-miR-382

Correlation coefficient	Parametric p-value	Symbol
-0.453	0.0532552	hsa-miR-493
-0.39	0.1002485	hsa-miR-411*
0.332	0.1654807	hsa-miR-889
0.302	0.2087235	hsa-miR-369-3p
-0.301	0.2087235	hsa-miR-379*
0.288	0.2314979	hsa-miR-134
0.284	0.237439	hsa-miR-337-3p
0.263	0.2751841	hsa-miR-487a
0.255	0.2919784	hsa-miR-495
0.251	0.2988721	hsa-miR-337-5p
0.24	0.320156	hsa-miR-379*
0.238	0.3237911	hsa-miR-495
0.236	0.3274511	hsa-miR-376a*
0.235	0.3311361	hsa-miR-493*
0.225	0.3537683	hsa-miR-410
0.219	0.3654182	hsa-miR-493
0.219	0.3654182	hsa-miR-656
0.219	0.3654182	hsa-miR-154*
-0.212	0.3812945	hsa-miR-299-5p
-0.188	0.4398878	hsa-miR-409-3p
-0.184	0.4486335	hsa-miR-409-3p
0.184	0.4486335	hsa-miR-410
0.174	0.4754154	hsa-miR-889
-0.169	0.4891069	hsa-miR-493
0.151	0.5361381	hsa-miR-432
0.144	0.5555273	hsa-miR-329
0.14	0.5653403	hsa-miR-134
-0.139	0.5702758	hsa-miR-485-3p
0.137	0.5752305	hsa-miR-432

Spearman coefficients
Migration

0.134	0.5851965	hsa-miR-539
0.128	0.6002843	hsa-miR-411
0.128	0.6002843	hsa-miR-493
-0.123	0.6155343	hsa-miR-889
0.119	0.6257885	hsa-miR-337-3p
0.118	0.6309412	hsa-miR-487b
-0.107	0.6622043	hsa-miR-323-3p
0.108	0.6622043	hsa-miR-376a*
0.101	0.678049	hsa-miR-337-5p
-0.1	0.6833608	hsa-miR-382
0.096	0.6940282	hsa-miR-342-3p
0.094	0.7047526	hsa-miR-377
0.077	0.7536687	hsa-miR-539
-0.075	0.7591653	hsa-miR-377
0.071	0.7701926	hsa-miR-539
0.068	0.7812635	hsa-miR-493*
0.062	0.7979473	hsa-miR-889
0.06	0.8091186	hsa-miR-299-5p
0.058	0.814718	hsa-miR-487a
-0.054	0.8259433	hsa-miR-656
-0.05	0.8428432	hsa-miR-411
-0.043	0.8598111	hsa-miR-323-3p
0.04	0.8711572	hsa-miR-329
-0.035	0.8882225	hsa-miR-411*
0.03	0.9053363	hsa-miR-154*
0.023	0.9282177	hsa-miR-485-3p
-0.02	0.9396804	hsa-miR-382
0.019	0.9396804	hsa-miR-487b
-0.012	0.9626387	hsa-miR-342-3p

Correlation coefficient	Parametric p-value	Symbol
0.646	0.0035506	hsa-miR-379*
0.604	0.0073026	hsa-miR-379*
-0.582	0.0101341	hsa-miR-345
0.568	0.0124753	hsa-miR-889
-0.53	0.0212465	hsa-miR-377*
0.526	0.0222416	hsa-miR-337-5p
0.473	0.0421715	hsa-miR-411
0.462	0.0474618	hsa-miR-654-3p
0.453	0.0532552	hsa-miR-487b
0.451	0.0542717	hsa-miR-485-5p
0.449	0.0553032	hsa-miR-376c
0.45	0.0553032	hsa-miR-379
0.426	0.0701356	hsa-miR-380*
0.421	0.0739525	hsa-miR-380
0.412	0.0806616	hsa-miR-136*
0.412	0.0806616	hsa-miR-495
0.41	0.0820568	hsa-miR-136*
0.41	0.0820568	hsa-miR-495
-0.403	0.0878198	hsa-miR-432*
0.4	0.0908126	hsa-miR-411*
0.396	0.0954438	hsa-miR-337-5p
0.394	0.0954438	hsa-miR-376c
0.389	0.1002485	hsa-miR-376a
0.389	0.1002485	hsa-miR-376b
0.39	0.1002485	hsa-miR-485-3p
0.384	0.1052301	hsa-miR-758
0.382	0.1069306	hsa-miR-380
0.38	0.1086512	hsa-miR-337-3p
0.378	0.1103921	hsa-miR-299-5p

Spearman coefficients
Proliferation

0.379	0.1103921	hsa-miR-656
0.375	0.1139352	hsa-miR-381
-0.375	0.1139352	hsa-miR-665
0.369	0.1212701	hsa-miR-493
0.367	0.1231563	hsa-miR-758
0.351	0.1411006	hsa-miR-136
0.331	0.1654807	hsa-miR-376a
0.323	0.1774847	hsa-miR-323-3p
0.322	0.1774847	hsa-miR-329
0.316	0.1875131	hsa-miR-377
0.316	0.1875131	hsa-miR-485-5p
0.312	0.1926707	hsa-miR-409-3p
0.31	0.1952856	hsa-miR-654-5p
0.309	0.1979246	hsa-miR-758
0.305	0.2032754	hsa-miR-127-3p
0.305	0.2032754	hsa-miR-487b
-0.303	0.2059872	hsa-miR-300
0.304	0.2059872	hsa-miR-323-3p
0.302	0.2087235	hsa-miR-433
0.302	0.2087235	hsa-miR-485-5p
0.302	0.2087235	hsa-miR-543
-0.301	0.2087235	hsa-miR-544
0.3	0.2114842	hsa-miR-376a*
0.299	0.2114842	hsa-miR-411
0.3	0.2114842	hsa-miR-758
0.3	0.2114842	hsa-miR-889
0.296	0.2170791	hsa-miR-379*
-0.296	0.2170791	hsa-miR-431*
0.29	0.2285647	hsa-miR-342-5p
0.288	0.2314979	hsa-miR-409-3p
0.287	0.234456	hsa-miR-410

0.284	0.237439	hsa-miR-432
0.282	0.2404468	hsa-miR-337-3p
0.283	0.2404468	hsa-miR-889
0.279	0.2465373	hsa-miR-431
0.279	0.2465373	hsa-miR-432
-0.279	0.2465373	hsa-miR-541*
0.276	0.24962	hsa-miR-154*
0.278	0.24962	hsa-miR-154*
0.278	0.24962	hsa-miR-376b
0.275	0.2527277	hsa-miR-493*
-0.274	0.2558605	hsa-miR-431*
0.268	0.2654093	hsa-miR-136
0.269	0.2654093	hsa-miR-409-5p
0.258	0.2851852	hsa-miR-377*
0.25	0.2988721	hsa-miR-379
0.249	0.3023567	hsa-miR-485-3p
0.244	0.3129611	hsa-miR-382
0.24	0.320156	hsa-miR-539
0.239	0.3237911	hsa-miR-370
0.238	0.3237911	hsa-miR-411*
0.235	0.3311361	hsa-miR-134
0.233	0.334846	hsa-miR-377
0.23	0.3423405	hsa-miR-431
0.228	0.346125	hsa-miR-134
0.228	0.346125	hsa-miR-154
0.228	0.346125	hsa-miR-369-3p
0.228	0.346125	hsa-miR-377*
-0.226	0.3499342	hsa-miR-494
-0.224	0.3537683	hsa-miR-127-5p
0.225	0.3537683	hsa-miR-381
0.223	0.357627	hsa-miR-487a

-0.221	0.3615103	hsa-miR-431*
-0.221	0.3615103	hsa-miR-494
0.221	0.3615103	hsa-miR-543
0.218	0.3693506	hsa-miR-411*
-0.218	0.3693506	hsa-miR-431*
-0.214	0.3772889	hsa-miR-665
0.213	0.3812945	hsa-miR-369-5p
0.211	0.3853245	hsa-miR-329
0.205	0.3975592	hsa-miR-127-3p
-0.202	0.4058358	hsa-miR-345
-0.202	0.4058358	hsa-miR-412
0.197	0.4142076	hsa-miR-127-5p
0.196	0.4184291	hsa-miR-410
-0.188	0.4398878	hsa-miR-432*
-0.186	0.4442492	hsa-miR-541*
0.184	0.4486335	hsa-miR-300
0.184	0.4486335	hsa-miR-323-5p
0.18	0.4574706	hsa-miR-323-5p
-0.18	0.4574706	hsa-miR-541
-0.181	0.4574706	hsa-miR-656
0.177	0.4663982	hsa-miR-433
-0.172	0.4799573	hsa-miR-379*
0.172	0.4799573	hsa-miR-544
-0.169	0.4845211	hsa-miR-342-5p
-0.169	0.4891069	hsa-miR-412
0.168	0.4891069	hsa-miR-541
0.167	0.4937144	hsa-miR-299-5p
0.164	0.4983436	hsa-miR-299-3p
0.157	0.5170736	hsa-miR-376a*
0.148	0.5457928	hsa-miR-453
0.146	0.5506501	hsa-miR-654-3p

-0.143	0.5555273	hsa-miR-300
0.144	0.5555273	hsa-miR-487a
0.138	0.5702758	hsa-miR-889
-0.138	0.5702758	hsa-miR-203
0.138	0.5702758	hsa-miR-323-5p
0.133	0.5851965	hsa-miR-299-3p
-0.131	0.5902074	hsa-miR-453
0.124	0.6155343	hsa-miR-154
0.123	0.6155343	hsa-miR-412
0.111	0.6517186	hsa-miR-654-5p
0.111	0.6517186	hsa-miR-668
0.102	0.678049	hsa-miR-382
0.1	0.6833608	hsa-miR-453
-0.098	0.6886872	hsa-miR-770-5p
0.095	0.6993834	hsa-miR-493
-0.09	0.7155323	hsa-miR-345
-0.09	0.7155323	hsa-miR-493
0.086	0.7263653	hsa-miR-380*
0.086	0.7263653	hsa-miR-409-5p
0.081	0.7427106	hsa-miR-654-5p
0.077	0.7536687	hsa-miR-539
0.07	0.7757227	hsa-miR-300
-0.068	0.7812635	hsa-miR-370
0.066	0.7868147	hsa-miR-544
0.065	0.7923761	hsa-miR-432*
0.062	0.8035283	hsa-miR-127-5p
-0.061	0.8035283	hsa-miR-412
-0.061	0.8035283	hsa-miR-668
0.06	0.8091186	hsa-miR-409-5p
0.059	0.8091186	hsa-miR-541
0.052	0.8372019	hsa-miR-493

-0.048	0.8428432	hsa-miR-409-5p
-0.047	0.8484919	hsa-miR-377*
-0.044	0.8598111	hsa-miR-345
-0.037	0.8825283	hsa-miR-342-3p
-0.035	0.8882225	hsa-miR-369-5p
0.032	0.8996268	hsa-miR-665
-0.032	0.8996268	hsa-miR-770-5p
-0.03	0.9053363	hsa-miR-342-3p
0.03	0.9053363	hsa-miR-654-5p
0.026	0.916769	hsa-miR-493*
0.026	0.916769	hsa-miR-496
0.026	0.916769	hsa-miR-665
-0.022	0.9339475	hsa-miR-380*
-0.016	0.9511548	hsa-miR-770-5p
0.01	0.9683836	hsa-miR-770-5p
-0.009	0.9741299	hsa-miR-411*
-0.01	0.9741299	hsa-miR-432*
0.007	0.9798775	hsa-miR-541*
0.007	0.9798775	hsa-miR-541*
0.004	0.9856261	hsa-miR-323-5p
0.006	0.9856261	hsa-miR-539
-0.005	0.9856261	hsa-miR-668
0.004	0.9913754	hsa-miR-541
-0.003	0.9913754	hsa-miR-668
-0.002	0.9971251	hsa-miR-203

Correlation coefficient	Parametric p-value	Symbol
0.653	0.0031178	hsa-miR-379*
0.618	0.0058023	hsa-miR-487b
0.544	0.0176169	hsa-miR-487b
-0.388	0.1018892	hsa-miR-379*
0.304	0.2059872	hsa-miR-487a
-0.297	0.2170791	hsa-miR-539
0.285	0.234456	hsa-miR-337-5p
0.276	0.24962	hsa-miR-409-3p
0.268	0.2654093	hsa-miR-409-3p
0.266	0.2686424	hsa-miR-323-3p
-0.26	0.2818264	hsa-miR-493
0.246	0.3094012	hsa-miR-889
0.241	0.316546	hsa-miR-342-3p
0.237	0.3274511	hsa-miR-342-3p
0.226	0.3499342	hsa-miR-299-5p
-0.223	0.357627	hsa-miR-485-3p
0.216	0.3733075	hsa-miR-493
0.213	0.3772889	hsa-miR-134
-0.212	0.3812945	hsa-miR-539
0.211	0.3853245	hsa-miR-889
0.208	0.3893786	hsa-miR-495
-0.204	0.3975592	hsa-miR-493*
0.202	0.4058358	hsa-miR-495
0.198	0.4142076	hsa-miR-656
0.184	0.4486335	hsa-miR-382
-0.177	0.4663982	hsa-miR-889
-0.171	0.4799573	hsa-miR-493*
0.17	0.4845211	hsa-miR-656
0.16	0.5123594	hsa-miR-485-3p

Spearman coefficients
Colony-forming

0.159	0.5170736	hsa-miR-411*
0.155	0.5265647	hsa-miR-493
-0.153	0.5313412	hsa-miR-369-3p
0.142	0.560424	hsa-miR-411*
0.141	0.560424	hsa-miR-487a
0.133	0.5851965	hsa-miR-337-3p
0.131	0.5902074	hsa-miR-410
0.118	0.6309412	hsa-miR-411
0.118	0.6309412	hsa-miR-134
0.114	0.6412971	hsa-miR-411
-0.106	0.6674706	hsa-miR-376a*
-0.104	0.6727522	hsa-miR-299-5p
0.087	0.7209422	hsa-miR-432
-0.088	0.7209422	hsa-miR-493
0.081	0.7427106	hsa-miR-329
-0.079	0.7481837	hsa-miR-323-3p
0.076	0.7536687	hsa-miR-432
0.048	0.8484919	hsa-miR-154*
0.045	0.8598111	hsa-miR-377
-0.042	0.8654809	hsa-miR-889
-0.035	0.8882225	hsa-miR-329
0.03	0.9053363	hsa-miR-337-5p
0.027	0.916769	hsa-miR-539
0.014	0.9568957	hsa-miR-376a*
0.008	0.9741299	hsa-miR-154*
-0.005	0.9856261	hsa-miR-377
-0.005	0.9856261	hsa-miR-337-3p

Correlation coefficient	Parametric p-value	Symbol
-0.54	0.0184736	hsa-miR-493
-0.418	0.0765833	hsa-miR-411*
0.405	0.0863514	hsa-miR-134
-0.344	0.1496485	hsa-miR-379*
0.331	0.1654807	hsa-miR-369-3p
0.308	0.1979246	hsa-miR-656
0.298	0.2142694	hsa-miR-410
-0.281	0.2434796	hsa-miR-299-5p
0.274	0.2558605	hsa-miR-376a*
0.265	0.2719007	hsa-miR-337-3p
-0.265	0.2719007	hsa-miR-485-3p
0.26	0.2818264	hsa-miR-154*
0.241	0.316546	hsa-miR-134
-0.233	0.334846	hsa-miR-409-3p
0.223	0.357627	hsa-miR-337-5p
0.218	0.3654182	hsa-miR-487a
0.213	0.3772889	hsa-miR-342-3p
-0.209	0.3893786	hsa-miR-409-3p
-0.201	0.4058358	hsa-miR-323-3p
0.198	0.4142076	hsa-miR-495
-0.196	0.4184291	hsa-miR-411*
0.196	0.4184291	hsa-miR-495
-0.191	0.4312344	hsa-miR-493
0.184	0.4486335	hsa-miR-889
0.179	0.4619231	hsa-miR-539
0.172	0.4799573	hsa-miR-379*
-0.172	0.4799573	hsa-miR-411
0.17	0.4845211	hsa-miR-410
0.165	0.4983436	hsa-miR-376a*

Spearman coefficients
Invasion

0.16	0.5123594	hsa-miR-493
0.151	0.5361381	hsa-miR-493*
0.146	0.5457928	hsa-miR-539
0.147	0.5457928	hsa-miR-889
-0.133	0.5851965	hsa-miR-323-3p
0.13	0.5952367	hsa-miR-329
0.127	0.6053498	hsa-miR-539
0.124	0.6104332	hsa-miR-432
0.121	0.6206528	hsa-miR-337-3p
0.122	0.6206528	hsa-miR-493
-0.12	0.6257885	hsa-miR-382
-0.103	0.6727522	hsa-miR-487b
0.097	0.6886872	hsa-miR-487a
0.092	0.7047526	hsa-miR-342-3p
0.09	0.7155323	hsa-miR-889
0.08	0.7427106	hsa-miR-377
-0.079	0.7481837	hsa-miR-377
0.069	0.7757227	hsa-miR-299-5p
0.045	0.854148	hsa-miR-487b
0.044	0.8598111	hsa-miR-432
0.039	0.8768398	hsa-miR-411
-0.036	0.8825283	hsa-miR-485-3p
-0.037	0.8825283	hsa-miR-656
0.034	0.893922	hsa-miR-337-5p
0.025	0.9224915	hsa-miR-154*
-0.022	0.9282177	hsa-miR-329
0.014	0.9568957	hsa-miR-493*
0.005	0.9856261	hsa-miR-382

Correlation coefficient	Parametric p-value	Symbol
-0.453	0.0532552	hsa-miR-493
-0.39	0.1002485	hsa-miR-411*
0.332	0.1654807	hsa-miR-889
0.302	0.2087235	hsa-miR-369-3p
-0.301	0.2087235	hsa-miR-379*
0.288	0.2314979	hsa-miR-134
0.284	0.237439	hsa-miR-337-3p
0.263	0.2751841	hsa-miR-487a
0.255	0.2919784	hsa-miR-495
0.251	0.2988721	hsa-miR-337-5p
0.24	0.320156	hsa-miR-379*
0.238	0.3237911	hsa-miR-495
0.236	0.3274511	hsa-miR-376a*
0.235	0.3311361	hsa-miR-493*
0.225	0.3537683	hsa-miR-410
0.219	0.3654182	hsa-miR-493
0.219	0.3654182	hsa-miR-656
0.219	0.3654182	hsa-miR-154*
-0.212	0.3812945	hsa-miR-299-5p
-0.188	0.4398878	hsa-miR-409-3p
-0.184	0.4486335	hsa-miR-409-3p
0.184	0.4486335	hsa-miR-410
0.174	0.4754154	hsa-miR-889
-0.169	0.4891069	hsa-miR-493
0.151	0.5361381	hsa-miR-432
0.144	0.5555273	hsa-miR-329
0.14	0.5653403	hsa-miR-134
-0.139	0.5702758	hsa-miR-485-3p
0.137	0.5752305	hsa-miR-432

Spearman coefficients
Migration

0.134	0.5851965	hsa-miR-539
0.128	0.6002843	hsa-miR-411
0.128	0.6002843	hsa-miR-493
-0.123	0.6155343	hsa-miR-889
0.119	0.6257885	hsa-miR-337-3p
0.118	0.6309412	hsa-miR-487b
-0.107	0.6622043	hsa-miR-323-3p
0.108	0.6622043	hsa-miR-376a*
0.101	0.678049	hsa-miR-337-5p
-0.1	0.6833608	hsa-miR-382
0.096	0.6940282	hsa-miR-342-3p
0.094	0.7047526	hsa-miR-377
0.077	0.7536687	hsa-miR-539
-0.075	0.7591653	hsa-miR-377
0.071	0.7701926	hsa-miR-539
0.068	0.7812635	hsa-miR-493*
0.062	0.7979473	hsa-miR-889
0.06	0.8091186	hsa-miR-299-5p
0.058	0.814718	hsa-miR-487a
-0.054	0.8259433	hsa-miR-656
-0.05	0.8428432	hsa-miR-411
-0.043	0.8598111	hsa-miR-323-3p
0.04	0.8711572	hsa-miR-329
-0.035	0.8882225	hsa-miR-411*
0.03	0.9053363	hsa-miR-154*
0.023	0.9282177	hsa-miR-485-3p
-0.02	0.9396804	hsa-miR-382
0.019	0.9396804	hsa-miR-487b
-0.012	0.9626387	hsa-miR-342-3p

Correlation coefficient	Parametric p-value	Symbol
0.646	0.0035506	hsa-miR-379*
0.604	0.0073026	hsa-miR-379*
-0.582	0.0101341	hsa-miR-345
0.568	0.0124753	hsa-miR-889
-0.53	0.0212465	hsa-miR-377*
0.526	0.0222416	hsa-miR-337-5p
0.473	0.0421715	hsa-miR-411
0.462	0.0474618	hsa-miR-654-3p
0.453	0.0532552	hsa-miR-487b
0.451	0.0542717	hsa-miR-485-5p
0.449	0.0553032	hsa-miR-376c
0.45	0.0553032	hsa-miR-379
0.426	0.0701356	hsa-miR-380*
0.421	0.0739525	hsa-miR-380
0.412	0.0806616	hsa-miR-136*
0.412	0.0806616	hsa-miR-495
0.41	0.0820568	hsa-miR-136*
0.41	0.0820568	hsa-miR-495
-0.403	0.0878198	hsa-miR-432*
0.4	0.0908126	hsa-miR-411*
0.396	0.0954438	hsa-miR-337-5p
0.394	0.0954438	hsa-miR-376c
0.389	0.1002485	hsa-miR-376a
0.389	0.1002485	hsa-miR-376b
0.39	0.1002485	hsa-miR-485-3p
0.384	0.1052301	hsa-miR-758
0.382	0.1069306	hsa-miR-380
0.38	0.1086512	hsa-miR-337-3p
0.378	0.1103921	hsa-miR-299-5p

Spearman coefficients
Proliferation

0.379	0.1103921	hsa-miR-656
0.375	0.1139352	hsa-miR-381
-0.375	0.1139352	hsa-miR-665
0.369	0.1212701	hsa-miR-493
0.367	0.1231563	hsa-miR-758
0.351	0.1411006	hsa-miR-136
0.331	0.1654807	hsa-miR-376a
0.323	0.1774847	hsa-miR-323-3p
0.322	0.1774847	hsa-miR-329
0.316	0.1875131	hsa-miR-377
0.316	0.1875131	hsa-miR-485-5p
0.312	0.1926707	hsa-miR-409-3p
0.31	0.1952856	hsa-miR-654-5p
0.309	0.1979246	hsa-miR-758
0.305	0.2032754	hsa-miR-127-3p
0.305	0.2032754	hsa-miR-487b
-0.303	0.2059872	hsa-miR-300
0.304	0.2059872	hsa-miR-323-3p
0.302	0.2087235	hsa-miR-433
0.302	0.2087235	hsa-miR-485-5p
0.302	0.2087235	hsa-miR-543
-0.301	0.2087235	hsa-miR-544
0.3	0.2114842	hsa-miR-376a*
0.299	0.2114842	hsa-miR-411
0.3	0.2114842	hsa-miR-758
0.3	0.2114842	hsa-miR-889
0.296	0.2170791	hsa-miR-379*
-0.296	0.2170791	hsa-miR-431*
0.29	0.2285647	hsa-miR-342-5p
0.288	0.2314979	hsa-miR-409-3p
0.287	0.234456	hsa-miR-410

0.284	0.237439	hsa-miR-432
0.282	0.2404468	hsa-miR-337-3p
0.283	0.2404468	hsa-miR-889
0.279	0.2465373	hsa-miR-431
0.279	0.2465373	hsa-miR-432
-0.279	0.2465373	hsa-miR-541*
0.276	0.24962	hsa-miR-154*
0.278	0.24962	hsa-miR-154*
0.278	0.24962	hsa-miR-376b
0.275	0.2527277	hsa-miR-493*
-0.274	0.2558605	hsa-miR-431*
0.268	0.2654093	hsa-miR-136
0.269	0.2654093	hsa-miR-409-5p
0.258	0.2851852	hsa-miR-377*
0.25	0.2988721	hsa-miR-379
0.249	0.3023567	hsa-miR-485-3p
0.244	0.3129611	hsa-miR-382
0.24	0.320156	hsa-miR-539
0.239	0.3237911	hsa-miR-370
0.238	0.3237911	hsa-miR-411*
0.235	0.3311361	hsa-miR-134
0.233	0.334846	hsa-miR-377
0.23	0.3423405	hsa-miR-431
0.228	0.346125	hsa-miR-134
0.228	0.346125	hsa-miR-154
0.228	0.346125	hsa-miR-369-3p
0.228	0.346125	hsa-miR-377*
-0.226	0.3499342	hsa-miR-494
-0.224	0.3537683	hsa-miR-127-5p
0.225	0.3537683	hsa-miR-381
0.223	0.357627	hsa-miR-487a

-0.221	0.3615103	hsa-miR-431*
-0.221	0.3615103	hsa-miR-494
0.221	0.3615103	hsa-miR-543
0.218	0.3693506	hsa-miR-411*
-0.218	0.3693506	hsa-miR-431*
-0.214	0.3772889	hsa-miR-665
0.213	0.3812945	hsa-miR-369-5p
0.211	0.3853245	hsa-miR-329
0.205	0.3975592	hsa-miR-127-3p
-0.202	0.4058358	hsa-miR-345
-0.202	0.4058358	hsa-miR-412
0.197	0.4142076	hsa-miR-127-5p
0.196	0.4184291	hsa-miR-410
-0.188	0.4398878	hsa-miR-432*
-0.186	0.4442492	hsa-miR-541*
0.184	0.4486335	hsa-miR-300
0.184	0.4486335	hsa-miR-323-5p
0.18	0.4574706	hsa-miR-323-5p
-0.18	0.4574706	hsa-miR-541
-0.181	0.4574706	hsa-miR-656
0.177	0.4663982	hsa-miR-433
-0.172	0.4799573	hsa-miR-379*
0.172	0.4799573	hsa-miR-544
-0.169	0.4845211	hsa-miR-342-5p
-0.169	0.4891069	hsa-miR-412
0.168	0.4891069	hsa-miR-541
0.167	0.4937144	hsa-miR-299-5p
0.164	0.4983436	hsa-miR-299-3p
0.157	0.5170736	hsa-miR-376a*
0.148	0.5457928	hsa-miR-453
0.146	0.5506501	hsa-miR-654-3p

-0.143	0.5555273	hsa-miR-300
0.144	0.5555273	hsa-miR-487a
0.138	0.5702758	hsa-miR-889
-0.138	0.5702758	hsa-miR-203
0.138	0.5702758	hsa-miR-323-5p
0.133	0.5851965	hsa-miR-299-3p
-0.131	0.5902074	hsa-miR-453
0.124	0.6155343	hsa-miR-154
0.123	0.6155343	hsa-miR-412
0.111	0.6517186	hsa-miR-654-5p
0.111	0.6517186	hsa-miR-668
0.102	0.678049	hsa-miR-382
0.1	0.6833608	hsa-miR-453
-0.098	0.6886872	hsa-miR-770-5p
0.095	0.6993834	hsa-miR-493
-0.09	0.7155323	hsa-miR-345
-0.09	0.7155323	hsa-miR-493
0.086	0.7263653	hsa-miR-380*
0.086	0.7263653	hsa-miR-409-5p
0.081	0.7427106	hsa-miR-654-5p
0.077	0.7536687	hsa-miR-539
0.07	0.7757227	hsa-miR-300
-0.068	0.7812635	hsa-miR-370
0.066	0.7868147	hsa-miR-544
0.065	0.7923761	hsa-miR-432*
0.062	0.8035283	hsa-miR-127-5p
-0.061	0.8035283	hsa-miR-412
-0.061	0.8035283	hsa-miR-668
0.06	0.8091186	hsa-miR-409-5p
0.059	0.8091186	hsa-miR-541
0.052	0.8372019	hsa-miR-493

-0.048	0.8428432	hsa-miR-409-5p
-0.047	0.8484919	hsa-miR-377*
-0.044	0.8598111	hsa-miR-345
-0.037	0.8825283	hsa-miR-342-3p
-0.035	0.8882225	hsa-miR-369-5p
0.032	0.8996268	hsa-miR-665
-0.032	0.8996268	hsa-miR-770-5p
-0.03	0.9053363	hsa-miR-342-3p
0.03	0.9053363	hsa-miR-654-5p
0.026	0.916769	hsa-miR-493*
0.026	0.916769	hsa-miR-496
0.026	0.916769	hsa-miR-665
-0.022	0.9339475	hsa-miR-380*
-0.016	0.9511548	hsa-miR-770-5p
0.01	0.9683836	hsa-miR-770-5p
-0.009	0.9741299	hsa-miR-411*
-0.01	0.9741299	hsa-miR-432*
0.007	0.9798775	hsa-miR-541*
0.007	0.9798775	hsa-miR-541*
0.004	0.9856261	hsa-miR-323-5p
0.006	0.9856261	hsa-miR-539
-0.005	0.9856261	hsa-miR-668
0.004	0.9913754	hsa-miR-541
-0.003	0.9913754	hsa-miR-668
-0.002	0.9971251	hsa-miR-203

Correlation coefficient	Parametric p-value	Symbol
0.653	0.0031178	hsa-miR-379*
0.618	0.0058023	hsa-miR-487b
0.544	0.0176169	hsa-miR-487b
-0.388	0.1018892	hsa-miR-379*
0.304	0.2059872	hsa-miR-487a
-0.297	0.2170791	hsa-miR-539
0.285	0.234456	hsa-miR-337-5p
0.276	0.24962	hsa-miR-409-3p
0.268	0.2654093	hsa-miR-409-3p
0.266	0.2686424	hsa-miR-323-3p
-0.26	0.2818264	hsa-miR-493
0.246	0.3094012	hsa-miR-889
0.241	0.316546	hsa-miR-342-3p
0.237	0.3274511	hsa-miR-342-3p
0.226	0.3499342	hsa-miR-299-5p
-0.223	0.357627	hsa-miR-485-3p
0.216	0.3733075	hsa-miR-493
0.213	0.3772889	hsa-miR-134
-0.212	0.3812945	hsa-miR-539
0.211	0.3853245	hsa-miR-889
0.208	0.3893786	hsa-miR-495
-0.204	0.3975592	hsa-miR-493*
0.202	0.4058358	hsa-miR-495
0.198	0.4142076	hsa-miR-656
0.184	0.4486335	hsa-miR-382
-0.177	0.4663982	hsa-miR-889
-0.171	0.4799573	hsa-miR-493*
0.17	0.4845211	hsa-miR-656
0.16	0.5123594	hsa-miR-485-3p

Spearman coefficients
Colony-forming

0.159	0.5170736	hsa-miR-411*
0.155	0.5265647	hsa-miR-493
-0.153	0.5313412	hsa-miR-369-3p
0.142	0.560424	hsa-miR-411*
0.141	0.560424	hsa-miR-487a
0.133	0.5851965	hsa-miR-337-3p
0.131	0.5902074	hsa-miR-410
0.118	0.6309412	hsa-miR-411
0.118	0.6309412	hsa-miR-134
0.114	0.6412971	hsa-miR-411
-0.106	0.6674706	hsa-miR-376a*
-0.104	0.6727522	hsa-miR-299-5p
0.087	0.7209422	hsa-miR-432
-0.088	0.7209422	hsa-miR-493
0.081	0.7427106	hsa-miR-329
-0.079	0.7481837	hsa-miR-323-3p
0.076	0.7536687	hsa-miR-432
0.048	0.8484919	hsa-miR-154*
0.045	0.8598111	hsa-miR-377
-0.042	0.8654809	hsa-miR-889
-0.035	0.8882225	hsa-miR-329
0.03	0.9053363	hsa-miR-337-5p
0.027	0.916769	hsa-miR-539
0.014	0.9568957	hsa-miR-376a*
0.008	0.9741299	hsa-miR-154*
-0.005	0.9856261	hsa-miR-377
-0.005	0.9856261	hsa-miR-337-3p

Spearman coefficients

Invasion

Correlation coefficient	Parametric p-value	Symbol
-0.54	0.0184736	hsa-miR-493
-0.418	0.0765833	hsa-miR-411*
0.405	0.0863514	hsa-miR-134
-0.344	0.1496485	hsa-miR-379*
0.331	0.1654807	hsa-miR-369-3p
0.308	0.1979246	hsa-miR-656
0.298	0.2142694	hsa-miR-410
-0.281	0.2434796	hsa-miR-299-5p
0.274	0.2558605	hsa-miR-376a*
0.265	0.2719007	hsa-miR-337-3p
-0.265	0.2719007	hsa-miR-485-3p
0.26	0.2818264	hsa-miR-154*
0.241	0.316546	hsa-miR-134
-0.233	0.334846	hsa-miR-409-3p
0.223	0.357627	hsa-miR-337-5p
0.218	0.3654182	hsa-miR-487a
0.213	0.3772889	hsa-miR-342-3p
-0.209	0.3893786	hsa-miR-409-3p
-0.201	0.4058358	hsa-miR-323-3p
0.198	0.4142076	hsa-miR-495
-0.196	0.4184291	hsa-miR-411*
0.196	0.4184291	hsa-miR-495
-0.191	0.4312344	hsa-miR-493
0.184	0.4486335	hsa-miR-889
0.179	0.4619231	hsa-miR-539
0.172	0.4799573	hsa-miR-379*
-0.172	0.4799573	hsa-miR-411
0.17	0.4845211	hsa-miR-410
0.165	0.4983436	hsa-miR-376a*

0.16	0.5123594	hsa-miR-493
0.151	0.5361381	hsa-miR-493*
0.146	0.5457928	hsa-miR-539
0.147	0.5457928	hsa-miR-889
-0.133	0.5851965	hsa-miR-323-3p
0.13	0.5952367	hsa-miR-329
0.127	0.6053498	hsa-miR-539
0.124	0.6104332	hsa-miR-432
0.121	0.6206528	hsa-miR-337-3p
0.122	0.6206528	hsa-miR-493
-0.12	0.6257885	hsa-miR-382
-0.103	0.6727522	hsa-miR-487b
0.097	0.6886872	hsa-miR-487a
0.092	0.7047526	hsa-miR-342-3p
0.09	0.7155323	hsa-miR-889
0.08	0.7427106	hsa-miR-377
-0.079	0.7481837	hsa-miR-377
0.069	0.7757227	hsa-miR-299-5p
0.045	0.854148	hsa-miR-487b
0.044	0.8598111	hsa-miR-432
0.039	0.8768398	hsa-miR-411
-0.036	0.8825283	hsa-miR-485-3p
-0.037	0.8825283	hsa-miR-656
0.034	0.893922	hsa-miR-337-5p
0.025	0.9224915	hsa-miR-154*
-0.022	0.9282177	hsa-miR-329
0.014	0.9568957	hsa-miR-493*
0.005	0.9856261	hsa-miR-382

Correlation coefficient	Parametric p-value	Symbol
-0.453	0.0532552	hsa-miR-493
-0.39	0.1002485	hsa-miR-411*
0.332	0.1654807	hsa-miR-889
0.302	0.2087235	hsa-miR-369-3p
-0.301	0.2087235	hsa-miR-379*
0.288	0.2314979	hsa-miR-134
0.284	0.237439	hsa-miR-337-3p
0.263	0.2751841	hsa-miR-487a
0.255	0.2919784	hsa-miR-495
0.251	0.2988721	hsa-miR-337-5p
0.24	0.320156	hsa-miR-379*
0.238	0.3237911	hsa-miR-495
0.236	0.3274511	hsa-miR-376a*
0.235	0.3311361	hsa-miR-493*
0.225	0.3537683	hsa-miR-410
0.219	0.3654182	hsa-miR-493
0.219	0.3654182	hsa-miR-656
0.219	0.3654182	hsa-miR-154*
-0.212	0.3812945	hsa-miR-299-5p
-0.188	0.4398878	hsa-miR-409-3p
-0.184	0.4486335	hsa-miR-409-3p
0.184	0.4486335	hsa-miR-410
0.174	0.4754154	hsa-miR-889
-0.169	0.4891069	hsa-miR-493
0.151	0.5361381	hsa-miR-432
0.144	0.5555273	hsa-miR-329
0.14	0.5653403	hsa-miR-134
-0.139	0.5702758	hsa-miR-485-3p
0.137	0.5752305	hsa-miR-432

Spearman coefficients
Migration

0.134	0.5851965	hsa-miR-539
0.128	0.6002843	hsa-miR-411
0.128	0.6002843	hsa-miR-493
-0.123	0.6155343	hsa-miR-889
0.119	0.6257885	hsa-miR-337-3p
0.118	0.6309412	hsa-miR-487b
-0.107	0.6622043	hsa-miR-323-3p
0.108	0.6622043	hsa-miR-376a*
0.101	0.678049	hsa-miR-337-5p
-0.1	0.6833608	hsa-miR-382
0.096	0.6940282	hsa-miR-342-3p
0.094	0.7047526	hsa-miR-377
0.077	0.7536687	hsa-miR-539
-0.075	0.7591653	hsa-miR-377
0.071	0.7701926	hsa-miR-539
0.068	0.7812635	hsa-miR-493*
0.062	0.7979473	hsa-miR-889
0.06	0.8091186	hsa-miR-299-5p
0.058	0.814718	hsa-miR-487a
-0.054	0.8259433	hsa-miR-656
-0.05	0.8428432	hsa-miR-411
-0.043	0.8598111	hsa-miR-323-3p
0.04	0.8711572	hsa-miR-329
-0.035	0.8882225	hsa-miR-411*
0.03	0.9053363	hsa-miR-154*
0.023	0.9282177	hsa-miR-485-3p
-0.02	0.9396804	hsa-miR-382
0.019	0.9396804	hsa-miR-487b
-0.012	0.9626387	hsa-miR-342-3p

Spearman coefficients
Proliferation

Correlation coefficient	Parametric p-value	Symbol
0.646	0.0035506	hsa-miR-379*
0.604	0.0073026	hsa-miR-379*
-0.582	0.0101341	hsa-miR-345
0.568	0.0124753	hsa-miR-889
-0.53	0.0212465	hsa-miR-377*
0.526	0.0222416	hsa-miR-337-5p
0.473	0.0421715	hsa-miR-411
0.462	0.0474618	hsa-miR-654-3p
0.453	0.0532552	hsa-miR-487b
0.451	0.0542717	hsa-miR-485-5p
0.449	0.0553032	hsa-miR-376c
0.45	0.0553032	hsa-miR-379
0.426	0.0701356	hsa-miR-380*
0.421	0.0739525	hsa-miR-380
0.412	0.0806616	hsa-miR-136*
0.412	0.0806616	hsa-miR-495
0.41	0.0820568	hsa-miR-136*
0.41	0.0820568	hsa-miR-495
-0.403	0.0878198	hsa-miR-432*
0.4	0.0908126	hsa-miR-411*
0.396	0.0954438	hsa-miR-337-5p
0.394	0.0954438	hsa-miR-376c
0.389	0.1002485	hsa-miR-376a
0.389	0.1002485	hsa-miR-376b
0.39	0.1002485	hsa-miR-485-3p
0.384	0.1052301	hsa-miR-758
0.382	0.1069306	hsa-miR-380
0.38	0.1086512	hsa-miR-337-3p
0.378	0.1103921	hsa-miR-299-5p

0.379	0.1103921	hsa-miR-656
0.375	0.1139352	hsa-miR-381
-0.375	0.1139352	hsa-miR-665
0.369	0.1212701	hsa-miR-493
0.367	0.1231563	hsa-miR-758
0.351	0.1411006	hsa-miR-136
0.331	0.1654807	hsa-miR-376a
0.323	0.1774847	hsa-miR-323-3p
0.322	0.1774847	hsa-miR-329
0.316	0.1875131	hsa-miR-377
0.316	0.1875131	hsa-miR-485-5p
0.312	0.1926707	hsa-miR-409-3p
0.31	0.1952856	hsa-miR-654-5p
0.309	0.1979246	hsa-miR-758
0.305	0.2032754	hsa-miR-127-3p
0.305	0.2032754	hsa-miR-487b
-0.303	0.2059872	hsa-miR-300
0.304	0.2059872	hsa-miR-323-3p
0.302	0.2087235	hsa-miR-433
0.302	0.2087235	hsa-miR-485-5p
0.302	0.2087235	hsa-miR-543
-0.301	0.2087235	hsa-miR-544
0.3	0.2114842	hsa-miR-376a*
0.299	0.2114842	hsa-miR-411
0.3	0.2114842	hsa-miR-758
0.3	0.2114842	hsa-miR-889
0.296	0.2170791	hsa-miR-379*
-0.296	0.2170791	hsa-miR-431*
0.29	0.2285647	hsa-miR-342-5p
0.288	0.2314979	hsa-miR-409-3p
0.287	0.234456	hsa-miR-410

0.284	0.237439	hsa-miR-432
0.282	0.2404468	hsa-miR-337-3p
0.283	0.2404468	hsa-miR-889
0.279	0.2465373	hsa-miR-431
0.279	0.2465373	hsa-miR-432
-0.279	0.2465373	hsa-miR-541*
0.276	0.24962	hsa-miR-154*
0.278	0.24962	hsa-miR-154*
0.278	0.24962	hsa-miR-376b
0.275	0.2527277	hsa-miR-493*
-0.274	0.2558605	hsa-miR-431*
0.268	0.2654093	hsa-miR-136
0.269	0.2654093	hsa-miR-409-5p
0.258	0.2851852	hsa-miR-377*
0.25	0.2988721	hsa-miR-379
0.249	0.3023567	hsa-miR-485-3p
0.244	0.3129611	hsa-miR-382
0.24	0.320156	hsa-miR-539
0.239	0.3237911	hsa-miR-370
0.238	0.3237911	hsa-miR-411*
0.235	0.3311361	hsa-miR-134
0.233	0.334846	hsa-miR-377
0.23	0.3423405	hsa-miR-431
0.228	0.346125	hsa-miR-134
0.228	0.346125	hsa-miR-154
0.228	0.346125	hsa-miR-369-3p
0.228	0.346125	hsa-miR-377*
-0.226	0.3499342	hsa-miR-494
-0.224	0.3537683	hsa-miR-127-5p
0.225	0.3537683	hsa-miR-381
0.223	0.357627	hsa-miR-487a

-0.221	0.3615103	hsa-miR-431*
-0.221	0.3615103	hsa-miR-494
0.221	0.3615103	hsa-miR-543
0.218	0.3693506	hsa-miR-411*
-0.218	0.3693506	hsa-miR-431*
-0.214	0.3772889	hsa-miR-665
0.213	0.3812945	hsa-miR-369-5p
0.211	0.3853245	hsa-miR-329
0.205	0.3975592	hsa-miR-127-3p
-0.202	0.4058358	hsa-miR-345
-0.202	0.4058358	hsa-miR-412
0.197	0.4142076	hsa-miR-127-5p
0.196	0.4184291	hsa-miR-410
-0.188	0.4398878	hsa-miR-432*
-0.186	0.4442492	hsa-miR-541*
0.184	0.4486335	hsa-miR-300
0.184	0.4486335	hsa-miR-323-5p
0.18	0.4574706	hsa-miR-323-5p
-0.18	0.4574706	hsa-miR-541
-0.181	0.4574706	hsa-miR-656
0.177	0.4663982	hsa-miR-433
-0.172	0.4799573	hsa-miR-379*
0.172	0.4799573	hsa-miR-544
-0.169	0.4845211	hsa-miR-342-5p
-0.169	0.4891069	hsa-miR-412
0.168	0.4891069	hsa-miR-541
0.167	0.4937144	hsa-miR-299-5p
0.164	0.4983436	hsa-miR-299-3p
0.157	0.5170736	hsa-miR-376a*
0.148	0.5457928	hsa-miR-453
0.146	0.5506501	hsa-miR-654-3p

-0.143	0.5555273	hsa-miR-300
0.144	0.5555273	hsa-miR-487a
0.138	0.5702758	hsa-miR-889
-0.138	0.5702758	hsa-miR-203
0.138	0.5702758	hsa-miR-323-5p
0.133	0.5851965	hsa-miR-299-3p
-0.131	0.5902074	hsa-miR-453
0.124	0.6155343	hsa-miR-154
0.123	0.6155343	hsa-miR-412
0.111	0.6517186	hsa-miR-654-5p
0.111	0.6517186	hsa-miR-668
0.102	0.678049	hsa-miR-382
0.1	0.6833608	hsa-miR-453
-0.098	0.6886872	hsa-miR-770-5p
0.095	0.6993834	hsa-miR-493
-0.09	0.7155323	hsa-miR-345
-0.09	0.7155323	hsa-miR-493
0.086	0.7263653	hsa-miR-380*
0.086	0.7263653	hsa-miR-409-5p
0.081	0.7427106	hsa-miR-654-5p
0.077	0.7536687	hsa-miR-539
0.07	0.7757227	hsa-miR-300
-0.068	0.7812635	hsa-miR-370
0.066	0.7868147	hsa-miR-544
0.065	0.7923761	hsa-miR-432*
0.062	0.8035283	hsa-miR-127-5p
-0.061	0.8035283	hsa-miR-412
-0.061	0.8035283	hsa-miR-668
0.06	0.8091186	hsa-miR-409-5p
0.059	0.8091186	hsa-miR-541
0.052	0.8372019	hsa-miR-493

-0.048	0.8428432	hsa-miR-409-5p
-0.047	0.8484919	hsa-miR-377*
-0.044	0.8598111	hsa-miR-345
-0.037	0.8825283	hsa-miR-342-3p
-0.035	0.8882225	hsa-miR-369-5p
0.032	0.8996268	hsa-miR-665
-0.032	0.8996268	hsa-miR-770-5p
-0.03	0.9053363	hsa-miR-342-3p
0.03	0.9053363	hsa-miR-654-5p
0.026	0.916769	hsa-miR-493*
0.026	0.916769	hsa-miR-496
0.026	0.916769	hsa-miR-665
-0.022	0.9339475	hsa-miR-380*
-0.016	0.9511548	hsa-miR-770-5p
0.01	0.9683836	hsa-miR-770-5p
-0.009	0.9741299	hsa-miR-411*
-0.01	0.9741299	hsa-miR-432*
0.007	0.9798775	hsa-miR-541*
0.007	0.9798775	hsa-miR-541*
0.004	0.9856261	hsa-miR-323-5p
0.006	0.9856261	hsa-miR-539
-0.005	0.9856261	hsa-miR-668
0.004	0.9913754	hsa-miR-541
-0.003	0.9913754	hsa-miR-668
-0.002	0.9971251	hsa-miR-203
