# Supplementary material for: An imprinted non-coding genomic cluster at 14q32 defines clinically relevant molecular subtypes in osteosarcoma across multiple independent datasets
Source: J Hematol Oncol. 2017 May 15;10:107. doi: 10.1186/s13045-017-0465-4 (PMC5433149; doi:10.1186/s13045-017-0465-4)
Supplement: Supplementary file 12 — Network gene-drug screen interactions. This includes a detailed list of possible gene-drug interactions as a result from the screen applied between the significant edges from the 5-miRNA network and the Drug Gene Interaction Database. (PDF 488 kb) [file 13045_2017_465_MOESM12_ESM.pdf]

Association between methylation and cell line aggressiveness

Parametric p-value	Fold-change	Symbol
0.0051552	5.85	DLK1
0.0134129	9.77	DLK1
0.0290076	4.24	MEG3
0.0324276	3.34	MEG3
0.0504055	3.37	DLK1
0.0738048	2.31	MEG3
0.0817289	2.77	DLK1

Colony-Forming

Parametric p-value	Fold-change	Symbol
0.0005137	16.34	DLK1
0.0144994	4.03	DLK1
0.0364518	3.12	DLK1
0.0452589	3.54	DLK1

Invasion

Parametric p-value	Fold-change	Symbol
0.0005137	16.34	DLK1
0.0144994	4.03	DLK1
0.0364518	3.12	DLK1
0.0452589	3.54	DLK1

Migration

Parametric p-value	Fold-change	Symbol
0.0202816	7.75	DLK1
0.0408887	3.63	DLK1
0.069721	2.68	MEG3
0.0802155	3.08	MEG3

Tumorigenicity
