# Supplementary material for: An imprinted non-coding genomic cluster at 14q32 defines clinically relevant molecular subtypes in osteosarcoma across multiple independent datasets
Source: J Hematol Oncol. 2017 May 15;10:107. doi: 10.1186/s13045-017-0465-4 (PMC5433149; doi:10.1186/s13045-017-0465-4)
Supplement: Supplementary file 13 — Supplementary methods. Additional details are provided on certain aspects of our analytical procedures. (PDF 461 kb) [file 13045_2017_465_MOESM13_ESM.pdf]

## Network edge differences between the two subtypes

| regulator | target gene | edge z score difference | absolute edge z score difference | edge qvalue |
|---|---|---|---|---|
| hsa-miR-495 | MKRN3 | 0.886582 | 0.886582 | 7.50511E-13 |
| hsa-miR-495 | FAM21C | 0.877871 | 0.877871 | 7.50511E-13 |
| hsa-miR-495 | GAS1 | 0.826982 | 0.826982 | 1.49925E-12 |
| hsa-miR-495 | GPR88 | -0.823815 | 0.823815 | 7.50511E-13 |
| hsa-miR-495 | HUWE1 | 0.802832 | 0.802832 | 1.49925E-12 |
| hsa-miR-495 | GPR155 | -0.786583 | 0.786583 | 3.73812E-12 |
| hsa-miR-495 | SEC22B | 0.781657 | 0.781657 | 7.50511E-13 |
| hsa-miR-495 | TFAP2C | 0.76405 | 0.76405 | 7.50511E-13 |
| hsa-miR-495 | MTTP | -0.758492 | 0.758492 | 7.50511E-13 |
| hsa-miR-495 | WIPI2 | 0.740641 | 0.740641 | 7.50511E-13 |
| hsa-miR-495 | ARMC1 | -0.740203 | 0.740203 | 7.50511E-13 |
| hsa-miR-495 | ANKRD46 | 0.736635 | 0.736635 | 0.000740656 |
| hsa-miR-495 | SMC5 | 0.730702 | 0.730702 | 5.96569E-05 |
| hsa-miR-495 | GPX8 | -0.725542 | 0.725542 | 7.50511E-13 |
| hsa-miR-495 | TMED9 | 0.725021 | 0.725021 | 7.50511E-13 |
| hsa-miR-495 | CD9 | 0.715041 | 0.715041 | 1.25173E-09 |
| hsa-miR-495 | ATG12 | -0.701358 | 0.701358 | 7.50511E-13 |
| hsa-miR-495 | RAVER2 | 0.688694 | 0.688694 | 7.50511E-13 |
| hsa-miR-495 | RNASEH2C | -0.684982 | 0.684982 | 0.000725865 |
| hsa-miR-495 | TANK | 0.68042 | 0.68042 | 7.50511E-13 |
| hsa-miR-495 | CRNKL1 | 0.677079 | 0.677079 | 7.50511E-13 |
| hsa-miR-495 | KHSRP | -0.66216 | 0.66216 | 1.0447E-06 |
| hsa-miR-495 | PHF7 | 0.659883 | 0.659883 | 0.002876218 |
| hsa-miR-495 | DNAJC3 | -0.650673 | 0.650673 | 7.50511E-13 |
| hsa-miR-495 | RBM27 | 0.645674 | 0.645674 | 7.50511E-13 |
| hsa-miR-495 | HJURP | 0.644625 | 0.644625 | 7.50511E-13 |
| hsa-miR-495 | PPP1R12A | 0.638437 | 0.638437 | 7.50511E-13 |
| hsa-miR-495 | CCL2 | -0.626556 | 0.626556 | 0.004822733 |
| hsa-miR-495 | ZNF599 | 0.624977 | 0.624977 | 2.50167E-07 |
| hsa-miR-495 | PTPN13 | 0.623682 | 0.623682 | 7.50511E-13 |
| hsa-miR-495 | HGF | 0.611959 | 0.611959 | 7.50511E-13 |
| hsa-miR-495 | DDX46 | 0.61064 | 0.61064 | 7.50511E-13 |
| hsa-miR-495 | TFAP4 | 0.609763 | 0.609763 | 0.003968717 |
| hsa-miR-495 | FMO5 | -0.609464 | 0.609464 | 6.1638E-07 |
| hsa-miR-495 | ABCC4 | 0.608055 | 0.608055 | 5.42237E-10 |
| hsa-miR-495 | DYRK1A | -0.607727 | 0.607727 | 2.38494E-09 |
| hsa-miR-495 | PGAP1 | 0.601463 | 0.601463 | 7.50511E-13 |
| hsa-miR-495 | RYK | 0.600678 | 0.600678 | 7.50511E-13 |
| hsa-miR-495 | ITM2A | -0.597491 | 0.597491 | 7.50511E-13 |
| hsa-miR-495 | NIF3L1 | 0.593883 | 0.593883 | 0.000183131 |
| hsa-miR-495 | MYT1L | 0.593407 | 0.593407 | 2.99516E-07 |
| hsa-miR-495 | DNAH5 | -0.591474 | 0.591474 | 1.97261E-06 |
| hsa-miR-495 | WRB | 0.590625 | 0.590625 | 7.64109E-07 |
| hsa-miR-495 | SLC6A15 | 0.589695 | 0.589695 | 2.58186E-08 |
| hsa-miR-495 | ABT1 | -0.588294 | 0.588294 | 7.50511E-13 |
| hsa-miR-495 | FANCL | -0.587168 | 0.587168 | 4.31925E-05 |
| hsa-miR-495 | PPID | 0.58546 | 0.58546 | 7.50511E-13 |
| hsa-miR-495 | MXI1 | -0.583682 | 0.583682 | 0.001777316 |
| hsa-miR-495 | KCNJ2 | 0.583285 | 0.583285 | 6.28504E-08 |
| hsa-miR-495 | KIAA0494 | 0.580634 | 0.580634 | 1.39772E-08 |
| hsa-miR-495 | RUNDC3B | -0.578876 | 0.578876 | 1.63842E-11 |
| hsa-miR-495 | ELL2 | 0.578013 | 0.578013 | 3.37322E-09 |

| | | | | |
|---|---|---|---|---|
| hsa-miR-495 | LMBRD2 | 0.577533 | 0.577533 | 0.007572514 |
| hsa-miR-495 | ACRC | -0.576326 | 0.576326 | 4.29606E-07 |
| hsa-miR-495 | PAPLN | 0.575797 | 0.575797 | 5.99482E-08 |
| hsa-miR-495 | WBP4 | 0.574133 | 0.574133 | 7.50511E-13 |
| hsa-miR-495 | NFYC | 0.571867 | 0.571867 | 6.71463E-12 |
| hsa-miR-495 | DUSP6 | -0.567071 | 0.567071 | 0.072080147 |
| hsa-miR-495 | MPP3 | 0.565279 | 0.565279 | 7.50511E-13 |
| hsa-miR-495 | DUSP9 | 0.563977 | 0.563977 | 0.045791127 |
| hsa-miR-495 | FAM21A | 0.563232 | 0.563232 | 7.50511E-13 |
| hsa-miR-495 | MALT1 | 0.55887 | 0.55887 | 1.09827E-10 |
| hsa-miR-495 | DBR1 | 0.558511 | 0.558511 | 2.2442E-12 |
| hsa-miR-495 | DAPP1 | -0.557671 | 0.557671 | 1.37489E-05 |
| hsa-miR-495 | TPD52L3 | -0.557293 | 0.557293 | 1.34093E-11 |
| hsa-miR-495 | NSUN3 | -0.555549 | 0.555549 | 7.50511E-13 |
| hsa-miR-495 | MRPL35 | 0.555057 | 0.555057 | 2.82663E-11 |
| hsa-miR-495 | PPP6C | 0.554463 | 0.554463 | 1.92824E-10 |
| hsa-miR-495 | MRPL20 | 0.554426 | 0.554426 | 7.50511E-13 |
| hsa-miR-495 | BDH2 | 0.553939 | 0.553939 | 0.000403756 |
| hsa-miR-495 | SLK | 0.553898 | 0.553898 | 7.50511E-13 |
| hsa-miR-495 | FAM150B | -0.549074 | 0.549074 | 1.18203E-08 |
| hsa-miR-495 | CBX3 | -0.545887 | 0.545887 | 0.078084156 |
| hsa-miR-329 | PRUNE2 | 0.543323 | 0.543323 | 7.50511E-13 |
| hsa-miR-495 | COX7A2L | 0.542799 | 0.542799 | 2.17467E-05 |
| hsa-miR-495 | PLA2G15 | 0.54241 | 0.54241 | 0.011283923 |
| hsa-miR-495 | TJP2 | 0.541525 | 0.541525 | 1.22863E-08 |
| hsa-miR-329 | ST3GAL1 | -0.540912 | 0.540912 | 7.50511E-13 |
| hsa-miR-495 | HOXC6 | -0.537588 | 0.537588 | 0.001341923 |
| hsa-miR-495 | CDH2 | -0.536371 | 0.536371 | 5.54628E-05 |
| hsa-miR-495 | MED12L | -0.536116 | 0.536116 | 7.50511E-13 |
| hsa-miR-495 | IL20RB | -0.535823 | 0.535823 | 5.31606E-06 |
| hsa-miR-329 | MAP4K5 | 0.535823 | 0.535823 | 1.32251E-05 |
| hsa-miR-495 | POLR3G | 0.532231 | 0.532231 | 7.50511E-13 |
| hsa-miR-495 | ANAPC1 | 0.531578 | 0.531578 | 0.00431661 |
| hsa-miR-495 | BUB1 | 0.527158 | 0.527158 | 7.50511E-13 |
| hsa-miR-495 | NCOA1 | -0.526619 | 0.526619 | 3.73308E-08 |
| hsa-miR-495 | SLC35F1 | -0.524707 | 0.524707 | 0.00226449 |
| hsa-miR-495 | RLF | -0.524633 | 0.524633 | 2.2442E-12 |
| hsa-miR-495 | RAD18 | 0.524303 | 0.524303 | 7.50511E-13 |
| hsa-miR-495 | MAP4K5 | 0.524024 | 0.524024 | 7.50511E-13 |
| hsa-miR-495 | MBTD1 | -0.523757 | 0.523757 | 0.005888537 |
| hsa-miR-495 | ZNF765 | -0.522438 | 0.522438 | 7.50511E-13 |
| hsa-miR-495 | NDUFS2 | 0.522178 | 0.522178 | 7.23951E-07 |
| hsa-miR-495 | COQ10B | -0.52148 | 0.52148 | 5.22871E-12 |
| hsa-miR-495 | CDCA7L | 0.519399 | 0.519399 | 2.9899E-10 |
| hsa-miR-495 | PCDH20 | -0.518949 | 0.518949 | 1.7999E-07 |
| hsa-miR-495 | CCR1 | 0.518382 | 0.518382 | 2.32902E-09 |
| hsa-miR-495 | HUS1 | -0.517906 | 0.517906 | 0.000586343 |
| hsa-miR-656 | MKRN3 | 0.515118 | 0.515118 | 0.073020007 |
| hsa-miR-495 | VPS24 | -0.514811 | 0.514811 | 0.000189672 |
| hsa-miR-495 | MRE11A | -0.514461 | 0.514461 | 6.18665E-05 |
| hsa-miR-495 | PCDHB9 | -0.513794 | 0.513794 | 0.00039179 |
| hsa-miR-495 | WDR69 | 0.513423 | 0.513423 | 7.50511E-13 |
| hsa-miR-495 | ARHGAP19 | 0.511885 | 0.511885 | 4.3699E-10 |

| | | | | |
|---|---|---|---|---|
| hsa-miR-495 | ASAH1 | -0.511057 | 0.511057 | 6.61962E-05 |
| hsa-miR-495 | CMPK2 | 0.511026 | 0.511026 | 0.005623791 |
| hsa-miR-495 | TNNI1 | -0.510938 | 0.510938 | 0.000732145 |
| hsa-miR-495 | VDAC2 | 0.510758 | 0.510758 | 0.000345233 |
| hsa-miR-329 | LLPH | -0.510625 | 0.510625 | 0.000858542 |
| hsa-miR-495 | GAD2 | 0.510419 | 0.510419 | 2.82663E-11 |
| hsa-miR-495 | SNRPE | 0.510331 | 0.510331 | 6.76696E-06 |
| hsa-miR-495 | TFB2M | 0.510005 | 0.510005 | 9.6527E-10 |
| hsa-miR-495 | DNM3 | -0.50979 | 0.50979 | 0.000598628 |
| hsa-miR-495 | EEF1E1 | 0.509555 | 0.509555 | 7.50511E-13 |
| hsa-miR-495 | ZFAND3 | -0.509299 | 0.509299 | 1.09547E-05 |
| hsa-miR-495 | WIF1 | -0.508421 | 0.508421 | 1.58014E-10 |
| hsa-miR-495 | KLF5 | 0.508354 | 0.508354 | 7.50511E-13 |
| hsa-miR-495 | SLITRK2 | -0.50723 | 0.50723 | 7.50511E-13 |
| hsa-miR-495 | CISD2 | 0.507116 | 0.507116 | 6.50848E-08 |
| hsa-miR-495 | FKTN | -0.506437 | 0.506437 | 0.00093599 |
| hsa-miR-495 | WDR52 | -0.506392 | 0.506392 | 7.50511E-13 |
| hsa-miR-495 | RBX1 | 0.506369 | 0.506369 | 0.160387484 |
| hsa-miR-495 | SNRPB2 | 0.505395 | 0.505395 | 9.46789E-05 |
| hsa-miR-495 | THNSL1 | 0.505077 | 0.505077 | 6.69478E-08 |
| hsa-miR-495 | TYW3 | 0.50394 | 0.50394 | 3.30726E-06 |
| hsa-miR-495 | JUB | -0.503854 | 0.503854 | 1.448E-09 |
| hsa-miR-495 | SKIL | -0.503481 | 0.503481 | 1.43959E-05 |
| hsa-miR-495 | GAL3ST4 | -0.502331 | 0.502331 | 6.71463E-12 |
| hsa-miR-495 | ARPC1A | 0.501904 | 0.501904 | 7.14434E-08 |
| hsa-miR-495 | SPINLW1 | -0.501313 | 0.501313 | 0.003512139 |
| hsa-miR-495 | MDH1B | -0.501194 | 0.501194 | 0.001990376 |
| hsa-miR-495 | SELS | 0.501157 | 0.501157 | 8.18552E-05 |
| hsa-miR-495 | DDX3Y | 0.500966 | 0.500966 | 7.50511E-13 |
| hsa-miR-495 | CILP | -0.499509 | 0.499509 | 7.50511E-13 |
| hsa-miR-495 | HDAC4 | -0.499367 | 0.499367 | 0.001791792 |
| hsa-miR-495 | STXBP5 | 0.498309 | 0.498309 | 6.91007E-05 |
| hsa-miR-495 | FBXO11 | -0.497573 | 0.497573 | 8.00718E-09 |
| hsa-miR-495 | SNX31 | -0.497257 | 0.497257 | 7.50511E-13 |
| hsa-miR-495 | PARP14 | -0.495526 | 0.495526 | 0.01353825 |
| hsa-miR-495 | LHX2 | 0.494688 | 0.494688 | 7.50511E-13 |
| hsa-miR-495 | ATXN7 | -0.493694 | 0.493694 | 7.50511E-13 |
| hsa-miR-495 | SLTM | 0.493241 | 0.493241 | 7.50511E-13 |
| hsa-miR-495 | SLFN13 | -0.49284 | 0.49284 | 5.22871E-12 |
| hsa-miR-495 | NFIA | 0.49096 | 0.49096 | 7.50511E-13 |
| hsa-miR-495 | ODZ1 | -0.490796 | 0.490796 | 1.05897E-07 |
| hsa-miR-495 | FOXK1 | 0.48743 | 0.48743 | 7.50511E-13 |
| hsa-miR-495 | MYCL1 | -0.486754 | 0.486754 | 2.70345E-08 |
| hsa-miR-495 | NRXN1 | -0.48622 | 0.48622 | 0.007826831 |
| hsa-miR-495 | STK17A | 0.484989 | 0.484989 | 2.10676E-05 |
| hsa-miR-495 | NMD3 | 0.48498 | 0.48498 | 0.002214988 |
| hsa-miR-495 | CENPH | 0.482587 | 0.482587 | 1.57501E-05 |
| hsa-miR-495 | MFAP3 | 0.482451 | 0.482451 | 7.50511E-13 |
| hsa-miR-495 | PRKAA2 | 0.482169 | 0.482169 | 5.97034E-12 |
| hsa-miR-495 | SCNN1A | -0.482129 | 0.482129 | 6.6084E-11 |
| hsa-miR-495 | PDE4D | 0.481202 | 0.481202 | 2.41593E-07 |
| hsa-miR-495 | PPP4C | 0.480809 | 0.480809 | 0.000761359 |
| hsa-miR-495 | PTPRZ1 | -0.48067 | 0.48067 | 1.98035E-06 |

| | | | | |
|---|---|---|---|---|
| hsa-miR-495 | KIAA0101 | 0.479577 | 0.479577 | 0.001965831 |
| hsa-miR-495 | CDK6 | 0.479574 | 0.479574 | 3.78867E-09 |
| hsa-miR-495 | HLA-DPB1 | 0.479281 | 0.479281 | 1.11578E-07 |
| hsa-miR-495 | FBXO32 | 0.479255 | 0.479255 | 8.55783E-06 |
| hsa-miR-495 | RNF150 | -0.478937 | 0.478937 | 7.50511E-13 |
| hsa-miR-495 | SCEL | -0.478162 | 0.478162 | 1.34571E-06 |
| hsa-miR-495 | FGF19 | -0.478148 | 0.478148 | 8.93295E-06 |
| hsa-miR-495 | BCL6B | -0.477842 | 0.477842 | 1.19683E-09 |
| hsa-miR-495 | HEATR1 | 0.477793 | 0.477793 | 2.31557E-06 |
| hsa-miR-495 | HESX1 | -0.477299 | 0.477299 | 1.35606E-06 |
| hsa-miR-495 | LRP12 | -0.476963 | 0.476963 | 0.000428074 |
| hsa-miR-495 | RAD17 | 0.475305 | 0.475305 | 0.026436339 |
| hsa-miR-329 | ARFGAP2 | 0.473934 | 0.473934 | 6.71463E-12 |
| hsa-miR-495 | RHEB | -0.47378 | 0.47378 | 7.50511E-13 |
| hsa-miR-495 | VHL | 0.473752 | 0.473752 | 0.025681388 |
| hsa-miR-495 | RAB18 | 0.471948 | 0.471948 | 7.79174E-10 |
| hsa-miR-495 | GAP43 | -0.471493 | 0.471493 | 0.002180021 |
| hsa-miR-487b | TMEM53 | -0.469706 | 0.469706 | 0.000799041 |
| hsa-miR-495 | EXOSC8 | -0.466188 | 0.466188 | 4.27454E-09 |
| hsa-miR-495 | UIMC1 | 0.465934 | 0.465934 | 2.79539E-06 |
| hsa-miR-495 | MDFIC | -0.465745 | 0.465745 | 7.50511E-13 |
| hsa-miR-495 | ZNF830 | -0.465371 | 0.465371 | 0.143840452 |
| hsa-miR-495 | IFIT5 | -0.465325 | 0.465325 | 6.81424E-07 |
| hsa-miR-495 | RUNX3 | -0.465316 | 0.465316 | 0.000325902 |
| hsa-miR-495 | NIPA2 | 0.464616 | 0.464616 | 2.45074E-06 |
| hsa-miR-495 | CTSS | -0.464215 | 0.464215 | 0.101672533 |
| hsa-miR-495 | KLK10 | -0.463956 | 0.463956 | 1.49047E-05 |
| hsa-miR-495 | GTDC1 | -0.463743 | 0.463743 | 0.000147969 |
| hsa-miR-495 | ATP8A2 | -0.463724 | 0.463724 | 6.94739E-10 |
| hsa-miR-495 | RAN | 0.463123 | 0.463123 | 2.8952E-05 |
| hsa-miR-495 | KIAA1033 | 0.462438 | 0.462438 | 4.7782E-08 |
| hsa-miR-495 | UBE4A | 0.462253 | 0.462253 | 1.25111E-05 |
| hsa-miR-495 | CSPP1 | -0.460902 | 0.460902 | 0.000126661 |
| hsa-miR-495 | RORB | 0.459484 | 0.459484 | 0.000874057 |
| hsa-miR-495 | CTAG2 | -0.45711 | 0.45711 | 4.09153E-09 |
| hsa-miR-495 | PELI1 | 0.455805 | 0.455805 | 0.00037374 |
| hsa-miR-495 | HSPA4 | 0.455551 | 0.455551 | 1.34931E-07 |
| hsa-miR-495 | RG9MTD2 | -0.454708 | 0.454708 | 4.11974E-10 |
| hsa-miR-495 | FAM122B | 0.453672 | 0.453672 | 8.34262E-10 |
| hsa-miR-329 | ADAM28 | 0.453466 | 0.453466 | 1.49925E-12 |
| hsa-miR-495 | RBM41 | -0.452745 | 0.452745 | 0.002157669 |
| hsa-miR-495 | SFPQ | -0.452641 | 0.452641 | 6.18516E-08 |
| hsa-miR-329 | TMTC2 | 0.451607 | 0.451607 | 3.48333E-06 |
| hsa-miR-495 | RASSF9 | 0.451395 | 0.451395 | 7.50511E-13 |
| hsa-miR-495 | MSL1 | 0.451082 | 0.451082 | 0.000219173 |
| hsa-miR-495 | TBL1XR1 | 0.447821 | 0.447821 | 2.68291E-08 |
| hsa-miR-495 | NELL1 | 0.447293 | 0.447293 | 7.10767E-10 |
| hsa-miR-329 | ERC2 | 0.447077 | 0.447077 | 0.00170085 |
| hsa-miR-495 | CAMK2G | 0.447062 | 0.447062 | 9.70836E-08 |
| hsa-miR-495 | ELK1 | 0.446802 | 0.446802 | 7.50511E-13 |
| hsa-miR-495 | TIMM10 | 0.446069 | 0.446069 | 1.86073E-11 |
| hsa-miR-495 | PSAPL1 | -0.445885 | 0.445885 | 1.85049E-08 |
| hsa-miR-495 | CALU | 0.444849 | 0.444849 | 2.29698E-10 |

| | | | | |
|---|---|---|---|---|
| hsa-miR-329 | MAD2L1 | 0.444406 | 0.444406 | 0.014940994 |
| hsa-miR-329 | AKAP8 | -0.444289 | 0.444289 | 8.06266E-09 |
| hsa-miR-495 | TESC | -0.443883 | 0.443883 | 0.000447498 |
| hsa-miR-495 | RTN4 | 0.443428 | 0.443428 | 0.000257928 |
| hsa-miR-487b | ZNF664 | -0.443271 | 0.443271 | 1.63842E-11 |
| hsa-miR-495 | UAP1 | 0.442499 | 0.442499 | 4.57502E-06 |
| hsa-miR-495 | TLK1 | -0.442063 | 0.442063 | 2.78435E-10 |
| hsa-miR-495 | WDR25 | 0.441322 | 0.441322 | 0.002279716 |
| hsa-miR-495 | SRRM1 | 0.44114 | 0.44114 | 2.4838E-05 |
| hsa-miR-495 | CLEC6A | -0.439449 | 0.439449 | 0.023115441 |
| hsa-miR-495 | RICTOR | -0.437883 | 0.437883 | 0.090254952 |
| hsa-miR-329 | FBN1 | -0.436574 | 0.436574 | 2.45627E-09 |
| hsa-miR-495 | MAT1A | -0.436545 | 0.436545 | 7.50511E-13 |
| hsa-miR-495 | SOHLH2 | -0.436484 | 0.436484 | 0.003829403 |
| hsa-miR-495 | FAM9C | -0.43593 | 0.43593 | 0.000110445 |
| hsa-miR-329 | PTP4A2 | -0.43556 | 0.43556 | 0.000962745 |
| hsa-miR-495 | FOXC1 | -0.434751 | 0.434751 | 0.009214637 |
| hsa-miR-495 | ARFGEF2 | 0.434661 | 0.434661 | 6.89145E-05 |
| hsa-miR-495 | RASSF5 | -0.434337 | 0.434337 | 7.07216E-06 |
| hsa-miR-495 | EPC2 | -0.433611 | 0.433611 | 7.50511E-13 |
| hsa-miR-495 | ZNF280C | -0.433521 | 0.433521 | 7.50511E-13 |
| hsa-miR-495 | CLCN6 | -0.432745 | 0.432745 | 0.012868218 |
| hsa-miR-329 | TANK | 0.432467 | 0.432467 | 1.8647E-05 |
| hsa-miR-495 | SLC16A10 | 0.431912 | 0.431912 | 2.76E-05 |
| hsa-miR-495 | EFHC1 | 0.430966 | 0.430966 | 0.00140021 |
| hsa-miR-495 | PAAF1 | 0.430764 | 0.430764 | 2.37778E-10 |
| hsa-miR-495 | ZNF319 | 0.430672 | 0.430672 | 8.59777E-07 |
| hsa-miR-495 | STAMBP | -0.430123 | 0.430123 | 4.26384E-07 |
| hsa-miR-495 | CCDC108 | -0.429755 | 0.429755 | 2.81313E-10 |
| hsa-miR-495 | PRKG1 | -0.429748 | 0.429748 | 2.19579E-09 |
| hsa-miR-329 | MON2 | -0.428684 | 0.428684 | 0.004317122 |
| hsa-miR-495 | NUP62CL | 0.427294 | 0.427294 | 1.47033E-05 |
| hsa-miR-495 | JUNB | 0.427052 | 0.427052 | 0.006830587 |
| hsa-miR-495 | TMCO1 | 0.426947 | 0.426947 | 0.035604229 |
| hsa-miR-495 | UBN2 | 0.426507 | 0.426507 | 2.7034E-06 |
| hsa-miR-495 | MINK1 | -0.426092 | 0.426092 | 0.000377255 |
| hsa-miR-495 | FGFR1OP | -0.425862 | 0.425862 | 5.71951E-06 |
| hsa-miR-495 | IRS1 | 0.425457 | 0.425457 | 0.000126233 |
| hsa-miR-495 | RMI1 | -0.424056 | 0.424056 | 0.000876408 |
| hsa-miR-495 | KIAA1324 | 0.424009 | 0.424009 | 2.6833E-06 |
| hsa-miR-495 | EDN1 | -0.423484 | 0.423484 | 0.00849161 |
| hsa-miR-495 | GABRA1 | -0.422899 | 0.422899 | 0.061328621 |
| hsa-miR-495 | SLC26A2 | -0.422865 | 0.422865 | 6.01156E-10 |
| hsa-miR-495 | SPCS3 | 0.422089 | 0.422089 | 0.057008742 |
| hsa-miR-495 | PRKCB | -0.421988 | 0.421988 | 2.88304E-06 |
| hsa-miR-656 | PNPT1 | -0.421955 | 0.421955 | 0.002520652 |
| hsa-miR-495 | SRFBP1 | 0.421837 | 0.421837 | 9.24462E-07 |
| hsa-miR-495 | FRMD7 | 0.421256 | 0.421256 | 8.94484E-12 |
| hsa-miR-495 | TMEFF1 | -0.421132 | 0.421132 | 7.8053E-05 |
| hsa-miR-495 | TRUB1 | 0.420747 | 0.420747 | 0.003037909 |
| hsa-miR-495 | DCK | 0.419458 | 0.419458 | 0.000194428 |
| hsa-miR-656 | CPEB4 | -0.419224 | 0.419224 | 0.059027895 |
| hsa-miR-495 | CD46 | 0.418519 | 0.418519 | 1.74227E-08 |

| | | | | |
|---|---|---|---|---|
| hsa-miR-495 | RASD2 | -0.417816 | 0.417816 | 8.35953E-06 |
| hsa-miR-495 | EFCAB4B | -0.417132 | 0.417132 | 3.73812E-12 |
| hsa-miR-656 | GABRP | -0.416215 | 0.416215 | 4.19709E-05 |
| hsa-miR-495 | MRPS5 | 0.416069 | 0.416069 | 2.74504E-06 |
| hsa-miR-495 | TMEM144 | 0.415763 | 0.415763 | 0.005861601 |
| hsa-miR-495 | HMGXB4 | -0.415472 | 0.415472 | 0.000763391 |
| hsa-miR-495 | ALG10B | 0.415407 | 0.415407 | 0.000125832 |
| hsa-miR-495 | DCLRE1B | 0.414392 | 0.414392 | 0.005319588 |
| hsa-miR-495 | DSC2 | 0.414337 | 0.414337 | 0.028926372 |
| hsa-miR-495 | PRDM9 | -0.414035 | 0.414035 | 1.57826E-08 |
| hsa-miR-495 | RFK | -0.413015 | 0.413015 | 0.174197709 |
| hsa-miR-495 | FASN | -0.412767 | 0.412767 | 0.0985861 |
| hsa-miR-495 | IL25 | -0.412174 | 0.412174 | 9.68736E-12 |
| hsa-miR-495 | VMAC | -0.411057 | 0.411057 | 0.000118243 |
| hsa-miR-495 | HCCS | 0.410889 | 0.410889 | 2.44261E-08 |
| hsa-miR-495 | CACNA1B | -0.410443 | 0.410443 | 0.061868756 |
| hsa-miR-495 | CCDC91 | 0.410016 | 0.410016 | 0.03586476 |
| hsa-miR-495 | LEKR1 | -0.408493 | 0.408493 | 2.00921E-10 |
| hsa-miR-495 | NCF2 | -0.40827 | 0.40827 | 9.94467E-10 |
| hsa-miR-329 | SLK | 0.407925 | 0.407925 | 7.50511E-13 |
| hsa-miR-495 | RAB21 | -0.407468 | 0.407468 | 2.71944E-05 |
| hsa-miR-495 | PTGR2 | -0.407426 | 0.407426 | 0.036304825 |
| hsa-miR-495 | ZC3HAV1L | -0.407372 | 0.407372 | 0.000707752 |
| hsa-miR-495 | PRPS2 | 0.407165 | 0.407165 | 9.09001E-08 |
| hsa-miR-495 | PPM1A | -0.405716 | 0.405716 | 3.85638E-06 |
| hsa-miR-495 | SH2D4B | -0.405581 | 0.405581 | 0.041927124 |
| hsa-miR-495 | AQP4 | -0.405133 | 0.405133 | 0.037849406 |
| hsa-miR-495 | ZNF615 | 0.404799 | 0.404799 | 0.001493479 |
| hsa-miR-495 | P4HA1 | -0.404058 | 0.404058 | 2.00899E-11 |
| hsa-miR-495 | TIPIN | 0.403133 | 0.403133 | 0.001545512 |
| hsa-miR-495 | NCAPG | 0.403099 | 0.403099 | 0.000105699 |
| hsa-miR-329 | CLDN10 | -0.402552 | 0.402552 | 7.50511E-13 |
| hsa-miR-495 | MLL3 | 0.40254 | 0.40254 | 2.40491E-05 |
| hsa-miR-495 | OPRM1 | -0.401336 | 0.401336 | 0.013411776 |
| hsa-miR-495 | ZC3H12C | 0.401073 | 0.401073 | 1.5814E-06 |
| hsa-miR-495 | EIF2A | 0.400289 | 0.400289 | 0.000555181 |
| hsa-miR-495 | MAD2L1 | 0.399468 | 0.399468 | 0.001441528 |
| hsa-miR-495 | SPOCK1 | 0.399434 | 0.399434 | 5.21761E-09 |
| hsa-miR-495 | RAP1GDS1 | 0.399219 | 0.399219 | 5.21709E-10 |
| hsa-miR-495 | ZFHX4 | 0.398895 | 0.398895 | 0.000100295 |
| hsa-miR-495 | CAV2 | -0.398839 | 0.398839 | 7.50511E-13 |
| hsa-miR-495 | POU3F1 | 0.397586 | 0.397586 | 4.98907E-06 |
| hsa-miR-656 | FAM60A | 0.396026 | 0.396026 | 0.205486382 |
| hsa-miR-495 | FLJ44635 | -0.395926 | 0.395926 | 0.010700802 |
| hsa-miR-329 | TNFSF10 | -0.395481 | 0.395481 | 1.0056E-08 |
| hsa-miR-329 | EDARADD | 0.395328 | 0.395328 | 0.0029371 |
| hsa-miR-495 | TJP1 | 0.395159 | 0.395159 | 0.000182749 |
| hsa-miR-495 | OPCML | 0.394797 | 0.394797 | 0.006045998 |
| hsa-miR-495 | LRMP | -0.394558 | 0.394558 | 3.79137E-11 |
| hsa-miR-495 | NDUFB6 | 0.393825 | 0.393825 | 0.000354357 |
| hsa-miR-495 | GPR22 | -0.393056 | 0.393056 | 0.007286774 |
| hsa-miR-329 | PRPF4B | 0.392707 | 0.392707 | 3.17506E-09 |
| hsa-miR-495 | CASS4 | 0.392515 | 0.392515 | 5.22871E-12 |

| | | | | |
|---|---|---|---|---|
| hsa-miR-495 | NUP62 | 0.392494 | 0.392494 | 0.004628624 |
| hsa-miR-495 | PMP22 | 0.392461 | 0.392461 | 0.023442482 |
| hsa-miR-495 | CT47A6 | -0.392204 | 0.392204 | 7.50511E-13 |
| hsa-miR-329 | RIMBP2 | -0.39217 | 0.39217 | 4.53597E-09 |
| hsa-miR-656 | DDX4 | -0.391917 | 0.391917 | 5.57054E-11 |
| hsa-miR-495 | TMEM206 | 0.3918 | 0.3918 | 0.000181529 |
| hsa-miR-329 | ZC3H10 | 0.391425 | 0.391425 | 1.05211E-07 |
| hsa-miR-495 | BCDIN3D | 0.391367 | 0.391367 | 1.5921E-05 |
| hsa-miR-495 | GLIPR1L1 | -0.391089 | 0.391089 | 1.02017E-07 |
| hsa-miR-656 | UGT2A2 | -0.391012 | 0.391012 | 1.07646E-05 |
| hsa-miR-495 | IBTK | 0.390252 | 0.390252 | 0.000121138 |
| hsa-miR-495 | CXCL2 | -0.390229 | 0.390229 | 0.000299928 |
| hsa-miR-495 | ITSN2 | -0.389761 | 0.389761 | 0.064676634 |
| hsa-miR-495 | UNC5C | -0.388951 | 0.388951 | 6.18846E-07 |
| hsa-miR-495 | TMEM19 | 0.388939 | 0.388939 | 7.78125E-07 |
| hsa-miR-495 | PLS3 | 0.388814 | 0.388814 | 0.000677117 |
| hsa-miR-495 | BMP6 | 0.388637 | 0.388637 | 2.69828E-06 |
| hsa-miR-495 | LRRN1 | -0.388486 | 0.388486 | 1.49925E-12 |
| hsa-miR-329 | C1QTNF7 | 0.388468 | 0.388468 | 0.001828224 |
| hsa-miR-495 | NMNAT1 | -0.388036 | 0.388036 | 0.007036728 |
| hsa-miR-495 | CREB5 | -0.388032 | 0.388032 | 3.73812E-12 |
| hsa-miR-495 | ABCA2 | 0.387897 | 0.387897 | 0.000251457 |
| hsa-miR-329 | TMEM144 | 0.387845 | 0.387845 | 1.35168E-05 |
| hsa-miR-329 | PITPNM3 | -0.386521 | 0.386521 | 4.16488E-06 |
| hsa-miR-495 | FBXL7 | -0.386197 | 0.386197 | 0.015935902 |
| hsa-miR-329 | FBXO32 | 0.385444 | 0.385444 | 0.085331459 |
| hsa-miR-495 | LEP | -0.385086 | 0.385086 | 7.12603E-11 |
| hsa-miR-410 | PDCD7 | -0.384987 | 0.384987 | 0.001589904 |
| hsa-miR-495 | LRP11 | 0.38484 | 0.38484 | 0.014999972 |
| hsa-miR-495 | DLC1 | -0.384176 | 0.384176 | 0.022976562 |
| hsa-miR-495 | KLHL15 | 0.384063 | 0.384063 | 0.000280658 |
| hsa-miR-495 | SUPT16H | 0.382766 | 0.382766 | 0.241186014 |
| hsa-miR-495 | THUMPD1 | 0.382367 | 0.382367 | 7.44642E-06 |
| hsa-miR-329 | NPTX1 | -0.382264 | 0.382264 | 1.6045E-06 |
| hsa-miR-495 | TRIP11 | -0.382237 | 0.382237 | 0.017221658 |
| hsa-miR-495 | ARHGAP20 | -0.382094 | 0.382094 | 0.022488731 |
| hsa-miR-329 | G3BP1 | 0.381291 | 0.381291 | 7.50511E-13 |
| hsa-miR-495 | NET1 | -0.381232 | 0.381232 | 0.02514289 |
| hsa-miR-495 | MBTPS1 | -0.381188 | 0.381188 | 0.137229375 |
| hsa-miR-495 | HSPA2 | -0.380794 | 0.380794 | 0.066657069 |
| hsa-miR-495 | CYP20A1 | -0.380698 | 0.380698 | 0.000198156 |
| hsa-miR-495 | SNX5 | 0.379602 | 0.379602 | 0.002687764 |
| hsa-miR-495 | CAPZA2 | 0.379345 | 0.379345 | 0.009519804 |
| hsa-miR-495 | TTC8 | -0.379104 | 0.379104 | 0.030485095 |
| hsa-miR-495 | MSRA | 0.378838 | 0.378838 | 3.66855E-05 |
| hsa-miR-495 | DAPK1 | -0.378372 | 0.378372 | 2.76537E-05 |
| hsa-miR-495 | RDH10 | 0.377892 | 0.377892 | 0.057612209 |
| hsa-miR-495 | ATP5S | 0.377719 | 0.377719 | 2.80953E-05 |
| hsa-miR-495 | HTR1E | -0.377167 | 0.377167 | 0.000261488 |
| hsa-miR-329 | DZIP3 | 0.377165 | 0.377165 | 0.002578955 |
| hsa-miR-495 | RPS29 | 0.376509 | 0.376509 | 6.50853E-05 |
| hsa-miR-495 | ZFR | 0.375557 | 0.375557 | 3.83244E-10 |
| hsa-miR-495 | TM4SF1 | 0.374934 | 0.374934 | 2.35703E-05 |

| | | | | |
|---|---|---|---|---|
| hsa-miR-495 | HNRNPF | 0.374918 | 0.374918 | 2.04822E-07 |
| hsa-miR-495 | CDH5 | 0.374814 | 0.374814 | 0.001893178 |
| hsa-miR-495 | CCDC76 | 0.37421 | 0.37421 | 0.000125832 |
| hsa-miR-487b | PAK7 | -0.373463 | 0.373463 | 9.86345E-09 |
| hsa-miR-495 | THAP11 | 0.371648 | 0.371648 | 0.000921568 |
| hsa-miR-495 | CTAG1A | -0.371486 | 0.371486 | 0.000174595 |
| hsa-miR-495 | SPON1 | -0.370659 | 0.370659 | 1.17058E-05 |
| hsa-miR-495 | MID2 | 0.370527 | 0.370527 | 7.50511E-13 |
| hsa-miR-495 | HEY1 | 0.369974 | 0.369974 | 7.50511E-13 |
| hsa-miR-495 | PREP | 0.369075 | 0.369075 | 0.051645121 |
| hsa-miR-495 | L3MBTL4 | -0.368447 | 0.368447 | 2.01738E-06 |
| hsa-miR-329 | GLUD2 | -0.368322 | 0.368322 | 3.91363E-07 |
| hsa-miR-495 | MKS1 | -0.367736 | 0.367736 | 3.45367E-07 |
| hsa-miR-329 | PTDSS2 | -0.367679 | 0.367679 | 0.202774974 |
| hsa-miR-656 | WNT7A | -0.367156 | 0.367156 | 0.00011474 |
| hsa-miR-656 | FAM100B | 0.366904 | 0.366904 | 1.04272E-06 |
| hsa-miR-495 | PHF6 | 0.366471 | 0.366471 | 0.076889158 |
| hsa-miR-656 | HNRNPA0 | 0.366069 | 0.366069 | 9.00883E-08 |
| hsa-miR-329 | MTAP | 0.365758 | 0.365758 | 0.033939516 |
| hsa-miR-495 | URB1 | -0.36533 | 0.36533 | 0.240777032 |
| hsa-miR-495 | PARP11 | -0.365231 | 0.365231 | 0.060557798 |
| hsa-miR-495 | TMEM101 | 0.364723 | 0.364723 | 0.000139659 |
| hsa-miR-495 | LPPR5 | -0.364714 | 0.364714 | 1.61397E-09 |
| hsa-miR-495 | CT47A7 | -0.364669 | 0.364669 | 0.0147739 |
| hsa-miR-495 | PRRG4 | -0.364618 | 0.364618 | 0.00228899 |
| hsa-miR-329 | CLCN5 | 0.364083 | 0.364083 | 2.9501E-08 |
| hsa-miR-495 | PTK2 | 0.363842 | 0.363842 | 0.141876893 |
| hsa-miR-495 | C2CD4C | -0.363019 | 0.363019 | 0.000166396 |
| hsa-miR-495 | GCSH | 0.361788 | 0.361788 | 5.19807E-07 |
| hsa-miR-495 | CAND1 | 0.36155 | 0.36155 | 6.57611E-06 |
| hsa-miR-495 | PHTF2 | 0.360867 | 0.360867 | 0.003145766 |
| hsa-miR-495 | ITPK1 | 0.360551 | 0.360551 | 1.49925E-12 |
| hsa-miR-495 | ABI1 | -0.360216 | 0.360216 | 0.007024235 |
| hsa-miR-495 | MYEF2 | -0.36012 | 0.36012 | 1.49925E-12 |
| hsa-miR-495 | VTA1 | 0.359955 | 0.359955 | 0.124571587 |
| hsa-miR-495 | TLK2 | -0.358932 | 0.358932 | 0.015980785 |
| hsa-miR-495 | SPSB1 | 0.358803 | 0.358803 | 4.7618E-07 |
| hsa-miR-495 | TAF1 | 0.358395 | 0.358395 | 1.36889E-08 |
| hsa-miR-329 | ZMAT2 | 0.358072 | 0.358072 | 0.014662126 |
| hsa-miR-410 | BTBD1 | 0.357716 | 0.357716 | 3.86455E-11 |
| hsa-miR-495 | UNG | 0.357058 | 0.357058 | 0.006337458 |
| hsa-miR-329 | FEV | -0.355761 | 0.355761 | 0.000234172 |
| hsa-miR-495 | PIGY | -0.355704 | 0.355704 | 0.010348962 |
| hsa-miR-495 | HSPA1B | 0.355534 | 0.355534 | 0.000488203 |
| hsa-miR-495 | ARCN1 | 0.354998 | 0.354998 | 0.000212987 |
| hsa-miR-495 | ATL2 | 0.354483 | 0.354483 | 0.0022144 |
| hsa-miR-329 | CHD7 | -0.353796 | 0.353796 | 1.11306E-08 |
| hsa-miR-495 | ULK2 | -0.352566 | 0.352566 | 0.162183617 |
| hsa-miR-495 | ZFHX3 | 0.352502 | 0.352502 | 0.004932982 |
| hsa-miR-495 | PHLDB2 | 0.35191 | 0.35191 | 2.52724E-06 |
| hsa-miR-495 | FAM105A | -0.351452 | 0.351452 | 0.124942152 |
| hsa-miR-329 | GABRG1 | 0.350577 | 0.350577 | 7.58693E-08 |
| hsa-miR-329 | TMED1 | -0.350046 | 0.350046 | 0.000551027 |

| | | | | |
|---|---|---|---|---|
| hsa-miR-495 | KCMF1 | -0.348701 | 0.348701 | 5.11517E-10 |
| hsa-miR-495 | PRKAG2 | -0.348676 | 0.348676 | 0.203271717 |
| hsa-miR-495 | SPEF2 | -0.347538 | 0.347538 | 2.94671E-07 |
| hsa-miR-495 | ACSL4 | -0.347444 | 0.347444 | 0.017914351 |
| hsa-miR-329 | DAGLB | -0.34703 | 0.34703 | 0.000526043 |
| hsa-miR-329 | LHX9 | -0.346934 | 0.346934 | 7.50511E-13 |
| hsa-miR-410 | PUS10 | 0.345758 | 0.345758 | 0.000292083 |
| hsa-miR-495 | MTA3 | -0.344656 | 0.344656 | 0.0087329 |
| hsa-miR-656 | MYOT | 0.344636 | 0.344636 | 1.15239E-08 |
| hsa-miR-329 | LY6G5C | -0.344555 | 0.344555 | 0.000141687 |
| hsa-miR-495 | IER2 | -0.344039 | 0.344039 | 0.048357528 |
| hsa-miR-656 | A1CF | -0.343946 | 0.343946 | 1.43745E-05 |
| hsa-miR-495 | MDM2 | -0.343071 | 0.343071 | 0.00628333 |
| hsa-miR-329 | PSMA8 | -0.342778 | 0.342778 | 0.223977719 |
| hsa-miR-495 | HIGD1A | 0.342219 | 0.342219 | 7.50511E-13 |
| hsa-miR-656 | DMXL1 | 0.342214 | 0.342214 | 0.018523549 |
| hsa-miR-495 | OPA1 | 0.341924 | 0.341924 | 0.241186014 |
| hsa-miR-495 | PRUNE2 | 0.340812 | 0.340812 | 0.209253116 |
| hsa-miR-495 | LIPF | -0.33963 | 0.33963 | 1.26306E-08 |
| hsa-miR-495 | POU6F1 | -0.339602 | 0.339602 | 0.090717825 |
| hsa-miR-329 | NTRK3 | -0.339049 | 0.339049 | 3.91068E-06 |
| hsa-miR-495 | FNBP4 | 0.338554 | 0.338554 | 0.125816686 |
| hsa-miR-329 | RASSF5 | -0.338052 | 0.338052 | 0.19244193 |
| hsa-miR-329 | SFXN3 | -0.337338 | 0.337338 | 0.000344085 |
| hsa-miR-495 | ARMC8 | 0.336917 | 0.336917 | 0.008827803 |
| hsa-miR-495 | ZRANB2 | 0.33688 | 0.33688 | 0.000112797 |
| hsa-miR-495 | RAI2 | -0.33668 | 0.33668 | 0.241991791 |
| hsa-miR-495 | ALK | -0.335953 | 0.335953 | 0.00070041 |
| hsa-miR-495 | SLC7A11 | 0.335699 | 0.335699 | 1.49925E-12 |
| hsa-miR-495 | OVOL2 | -0.335062 | 0.335062 | 6.35626E-05 |
| hsa-miR-495 | CDR2L | 0.335013 | 0.335013 | 0.000910766 |
| hsa-miR-495 | PHLPP2 | 0.33449 | 0.33449 | 0.01699106 |
| hsa-miR-656 | MKI67IP | 0.33236 | 0.33236 | 0.004582004 |
| hsa-miR-656 | PALLD | 0.332321 | 0.332321 | 0.001166751 |
| hsa-miR-495 | FNBP1L | 0.332277 | 0.332277 | 0.018050001 |
| hsa-miR-495 | RARB | -0.331599 | 0.331599 | 0.083239728 |
| hsa-miR-495 | UBE2Q1 | 0.329963 | 0.329963 | 0.021681022 |
| hsa-miR-656 | NLK | 0.32959 | 0.32959 | 0.008110771 |
| hsa-miR-495 | USP38 | -0.32922 | 0.32922 | 5.95787E-05 |
| hsa-miR-329 | BRF2 | -0.328875 | 0.328875 | 0.005006332 |
| hsa-miR-495 | RAB41 | -0.328726 | 0.328726 | 0.002674786 |
| hsa-miR-495 | PBOV1 | -0.32861 | 0.32861 | 0.150415159 |
| hsa-miR-495 | GCLC | -0.327753 | 0.327753 | 0.000565303 |
| hsa-miR-329 | PAPPA | -0.326668 | 0.326668 | 0.013928865 |
| hsa-miR-495 | PTEN | 0.326173 | 0.326173 | 1.97342E-09 |
| hsa-miR-495 | HPGDS | 0.326105 | 0.326105 | 0.011412023 |
| hsa-miR-495 | TAGLN2 | 0.325818 | 0.325818 | 0.018201021 |
| hsa-miR-495 | BCL10 | 0.325087 | 0.325087 | 0.000192446 |
| hsa-miR-495 | TTC23 | 0.324912 | 0.324912 | 0.087267453 |
| hsa-miR-495 | GTF3C1 | 0.324697 | 0.324697 | 0.002540204 |
| hsa-miR-495 | SLC44A5 | 0.323789 | 0.323789 | 2.03212E-05 |
| hsa-miR-495 | FNDC3A | 0.323629 | 0.323629 | 0.005201123 |
| hsa-miR-495 | BRWD3 | 0.323347 | 0.323347 | 0.220096281 |

| | | | | |
|---|---|---|---|---|
| hsa-miR-495 | KRTAP6-3 | -0.322714 | 0.322714 | 7.82735E-05 |
| hsa-miR-329 | RAD51C | 0.322279 | 0.322279 | 1.19988E-05 |
| hsa-miR-495 | GRIA3 | -0.321624 | 0.321624 | 4.81546E-05 |
| hsa-miR-495 | CLK1 | -0.32113 | 0.32113 | 0.001024692 |
| hsa-miR-329 | RASSF2 | -0.320745 | 0.320745 | 1.22047E-06 |
| hsa-miR-329 | FAH | 0.319629 | 0.319629 | 0.004619089 |
| hsa-miR-495 | ZNF697 | 0.319536 | 0.319536 | 0.000714869 |
| hsa-miR-495 | MAGOHB | -0.319278 | 0.319278 | 0.073483786 |
| hsa-miR-495 | MGA | 0.319128 | 0.319128 | 1.48911E-05 |
| hsa-miR-495 | COG5 | 0.318329 | 0.318329 | 2.02343E-10 |
| hsa-miR-495 | RCHY1 | -0.3175 | 0.3175 | 0.000208349 |
| hsa-miR-410 | OPRM1 | -0.317384 | 0.317384 | 0.027200452 |
| hsa-miR-495 | ST8SIA2 | -0.31735 | 0.31735 | 1.3866E-08 |
| hsa-miR-495 | RAP2A | 0.317245 | 0.317245 | 0.000243404 |
| hsa-miR-329 | ADRB1 | 0.317015 | 0.317015 | 0.18174368 |
| hsa-miR-495 | KIAA0430 | 0.316955 | 0.316955 | 0.007085642 |
| hsa-miR-656 | RCOR3 | 0.316906 | 0.316906 | 7.50511E-13 |
| hsa-miR-495 | PHF21A | 0.316776 | 0.316776 | 0.002614882 |
| hsa-miR-329 | KIAA1274 | -0.315818 | 0.315818 | 0.040967401 |
| hsa-miR-329 | PPP1R3B | 0.315396 | 0.315396 | 2.47666E-08 |
| hsa-miR-329 | PRKACB | -0.315168 | 0.315168 | 7.87617E-06 |
| hsa-miR-656 | OR2L13 | -0.314863 | 0.314863 | 0.000256574 |
| hsa-miR-495 | YTHDC1 | 0.314842 | 0.314842 | 0.001034991 |
| hsa-miR-656 | SLCO1B3 | 0.313547 | 0.313547 | 0.033963521 |
| hsa-miR-495 | PTPRE | 0.312545 | 0.312545 | 0.058568993 |
| hsa-miR-495 | COL4A3 | 0.312393 | 0.312393 | 0.050701019 |
| hsa-miR-329 | FAM105B | 0.310154 | 0.310154 | 0.103629834 |
| hsa-miR-495 | ANKRD7 | -0.309228 | 0.309228 | 1.57196E-05 |
| hsa-miR-410 | BNIP3L | 0.308214 | 0.308214 | 0.029519398 |
| hsa-miR-410 | TMEFF2 | -0.307891 | 0.307891 | 0.092069062 |
| hsa-miR-495 | HDDC2 | 0.307853 | 0.307853 | 0.042051032 |
| hsa-miR-495 | BPGM | 0.307732 | 0.307732 | 0.004972225 |
| hsa-miR-495 | CDKAL1 | 0.307423 | 0.307423 | 5.14702E-06 |
| hsa-miR-495 | CDC42SE2 | 0.306041 | 0.306041 | 0.026983813 |
| hsa-miR-329 | CFH | 0.305671 | 0.305671 | 0.018456303 |
| hsa-miR-410 | SYNPR | -0.304844 | 0.304844 | 0.034561561 |
| hsa-miR-329 | METTL6 | 0.304517 | 0.304517 | 0.00325793 |
| hsa-miR-495 | MAPK10 | -0.304196 | 0.304196 | 3.88182E-06 |
| hsa-miR-329 | NEIL2 | -0.303413 | 0.303413 | 0.00537643 |
| hsa-miR-495 | OTUD4 | 0.303101 | 0.303101 | 0.046216405 |
| hsa-miR-495 | KAT2B | 0.302961 | 0.302961 | 0.03429505 |
| hsa-miR-495 | MLEC | 0.302645 | 0.302645 | 0.098284259 |
| hsa-miR-487b | RPL39L | -0.300964 | 0.300964 | 0.000408778 |
| hsa-miR-495 | UBA52 | 0.300569 | 0.300569 | 0.024404561 |
| hsa-miR-329 | SMARCA5 | -0.299947 | 0.299947 | 0.048884217 |
| hsa-miR-495 | CLCC1 | -0.29991 | 0.29991 | 0.222682256 |
| hsa-miR-329 | GADL1 | -0.299838 | 0.299838 | 0.176672297 |
| hsa-miR-410 | RUFY2 | -0.29879 | 0.29879 | 0.113613201 |
| hsa-miR-656 | CREBZF | 0.298485 | 0.298485 | 0.006169896 |
| hsa-miR-410 | SLC2A13 | 0.297864 | 0.297864 | 0.01819993 |
| hsa-miR-656 | ZFY | -0.296122 | 0.296122 | 0.05574367 |
| hsa-miR-329 | TRIM55 | -0.295998 | 0.295998 | 6.86304E-05 |
| hsa-miR-410 | GTF2B | 0.295503 | 0.295503 | 0.000569018 |

| | | | | |
|---|---|---|---|---|
| hsa-miR-329 | AQP3 | 0.294988 | 0.294988 | 0.027317392 |
| hsa-miR-495 | PTPN2 | 0.293233 | 0.293233 | 0.001271666 |
| hsa-miR-495 | KLHL5 | 0.293142 | 0.293142 | 0.002339685 |
| hsa-miR-495 | EPHA5 | -0.293 | 0.293 | 7.50511E-13 |
| hsa-miR-495 | SLC35E2 | -0.291317 | 0.291317 | 0.000718642 |
| hsa-miR-329 | KCNN3 | 0.291315 | 0.291315 | 0.00310527 |
| hsa-miR-329 | MID1 | 0.29109 | 0.29109 | 7.50511E-13 |
| hsa-miR-329 | EN2 | -0.288614 | 0.288614 | 0.106780104 |
| hsa-miR-495 | METTL4 | -0.288235 | 0.288235 | 3.05282E-05 |
| hsa-miR-495 | ICA1L | 0.287678 | 0.287678 | 0.021803381 |
| hsa-miR-495 | CCND1 | -0.287087 | 0.287087 | 0.001209179 |
| hsa-miR-329 | EGFLAM | -0.286625 | 0.286625 | 0.020943224 |
| hsa-miR-656 | CHCHD4 | 0.285143 | 0.285143 | 0.017444567 |
| hsa-miR-656 | AQP4 | -0.284013 | 0.284013 | 0.101082177 |
| hsa-miR-495 | MYLK | 0.283527 | 0.283527 | 0.230698198 |
| hsa-miR-329 | ESM1 | 0.282819 | 0.282819 | 0.000134692 |
| hsa-miR-495 | RADIL | 0.281879 | 0.281879 | 0.001589782 |
| hsa-miR-410 | TMEM108 | -0.280964 | 0.280964 | 0.032506439 |
| hsa-miR-487b | SCNM1 | 0.280961 | 0.280961 | 0.000329467 |
| hsa-miR-656 | UGT2B17 | -0.280807 | 0.280807 | 0.165540014 |
| hsa-miR-495 | POGZ | 0.280755 | 0.280755 | 4.02668E-05 |
| hsa-miR-329 | RANBP17 | 0.279654 | 0.279654 | 0.16226893 |
| hsa-miR-329 | PARS2 | -0.27947 | 0.27947 | 0.007286774 |
| hsa-miR-495 | SMNDC1 | 0.27831 | 0.27831 | 0.014246543 |
| hsa-miR-656 | STEAP2 | -0.275926 | 0.275926 | 0.178353342 |
| hsa-miR-495 | R3HDM2 | 0.275906 | 0.275906 | 0.071703609 |
| hsa-miR-410 | ATP6V1H | -0.275584 | 0.275584 | 0.008767693 |
| hsa-miR-495 | P4HA2 | 0.273573 | 0.273573 | 0.036975143 |
| hsa-miR-495 | TNFRSF21 | 0.272946 | 0.272946 | 0.000507888 |
| hsa-miR-329 | DLAT | 0.272801 | 0.272801 | 0.182569525 |
| hsa-miR-495 | ADAMTS19 | -0.271174 | 0.271174 | 0.001130851 |
| hsa-miR-410 | RERE | 0.270268 | 0.270268 | 0.000524914 |
| hsa-miR-495 | VGLL4 | 0.269229 | 0.269229 | 9.52938E-09 |
| hsa-miR-487b | PRR20C | -0.267811 | 0.267811 | 0.066512205 |
| hsa-miR-495 | ZFYVE16 | 0.267302 | 0.267302 | 0.047178274 |
| hsa-miR-410 | MIA3 | 0.266735 | 0.266735 | 0.009152261 |
| hsa-miR-495 | SERPINB9 | -0.266706 | 0.266706 | 0.000407338 |
| hsa-miR-495 | SHPRH | 0.265841 | 0.265841 | 0.175415927 |
| hsa-miR-329 | SIK2 | 0.265368 | 0.265368 | 7.78316E-06 |
| hsa-miR-495 | PLAG1 | -0.264727 | 0.264727 | 6.36236E-09 |
| hsa-miR-495 | NR2F2 | 0.264663 | 0.264663 | 4.68066E-11 |
| hsa-miR-329 | PHTF2 | 0.264557 | 0.264557 | 0.002564711 |
| hsa-miR-487b | SFTPA2 | -0.264511 | 0.264511 | 0.029936231 |
| hsa-miR-495 | IRGQ | 0.264073 | 0.264073 | 0.042128107 |
| hsa-miR-495 | IRF2 | 0.263502 | 0.263502 | 0.00444561 |
| hsa-miR-495 | KIFAP3 | 0.262596 | 0.262596 | 0.000256574 |
| hsa-miR-495 | JAZF1 | 0.261143 | 0.261143 | 0.000521463 |
| hsa-miR-495 | ARNT | -0.26086 | 0.26086 | 0.176297922 |
| hsa-miR-329 | AP3B1 | 0.260432 | 0.260432 | 0.002169991 |
| hsa-miR-495 | PREX1 | 0.260103 | 0.260103 | 4.92239E-06 |
| hsa-miR-495 | CDON | -0.260047 | 0.260047 | 0.044475837 |
| hsa-miR-495 | PCDHB7 | 0.259856 | 0.259856 | 0.011736567 |
| hsa-miR-656 | PCDHB9 | -0.259241 | 0.259241 | 1.24262E-06 |

| | | | | |
|---|---|---|---|---|
| hsa-miR-410 | GRHL3 | -0.259095 | 0.259095 | 0.025128634 |
| hsa-miR-329 | ATF3 | 0.257433 | 0.257433 | 0.22942999 |
| hsa-miR-495 | ROBO2 | 0.257075 | 0.257075 | 0.023564941 |
| hsa-miR-329 | KBTBD2 | 0.256927 | 0.256927 | 0.00119646 |
| hsa-miR-329 | OFD1 | 0.256055 | 0.256055 | 0.004963446 |
| hsa-miR-495 | NAB2 | -0.255153 | 0.255153 | 0.027314087 |
| hsa-miR-656 | OCIAD1 | 0.253213 | 0.253213 | 0.007449765 |
| hsa-miR-495 | FUBP1 | 0.25096 | 0.25096 | 0.061726928 |
| hsa-miR-329 | ACOT1 | -0.250473 | 0.250473 | 0.025093417 |
| hsa-miR-410 | LNX2 | 0.24937 | 0.24937 | 0.017228813 |
| hsa-miR-656 | MERTK | -0.248205 | 0.248205 | 0.000212675 |
| hsa-miR-495 | PI4KB | 0.246769 | 0.246769 | 0.020544382 |
| hsa-miR-410 | TANC1 | 0.246493 | 0.246493 | 0.016017863 |
| hsa-miR-495 | HOXD13 | 0.246252 | 0.246252 | 0.097229344 |
| hsa-miR-329 | TSPAN2 | 0.24603 | 0.24603 | 0.007195962 |
| hsa-miR-410 | BCAR3 | 0.245761 | 0.245761 | 0.083800572 |
| hsa-miR-656 | SCAP | -0.245583 | 0.245583 | 0.002919798 |
| hsa-miR-329 | SLC9A3R2 | 0.245445 | 0.245445 | 0.028240895 |
| hsa-miR-495 | IMPAD1 | 0.244194 | 0.244194 | 0.037849406 |
| hsa-miR-495 | CST9L | -0.243936 | 0.243936 | 0.15624117 |
| hsa-miR-329 | CCNJL | -0.243662 | 0.243662 | 0.023880397 |
| hsa-miR-656 | CENPK | 0.242191 | 0.242191 | 0.063313032 |
| hsa-miR-495 | CANX | 0.241455 | 0.241455 | 0.031504985 |
| hsa-miR-329 | WASF2 | -0.239929 | 0.239929 | 0.203791047 |
| hsa-miR-410 | B3GALTL | 0.238805 | 0.238805 | 0.152568946 |
| hsa-miR-495 | ASB5 | -0.237708 | 0.237708 | 0.090025951 |
| hsa-miR-656 | BBS10 | -0.237 | 0.237 | 0.020010747 |
| hsa-miR-656 | ARHGEF11 | 0.235852 | 0.235852 | 0.073938265 |
| hsa-miR-329 | PROK2 | -0.234603 | 0.234603 | 0.14317784 |
| hsa-miR-495 | TBP | 0.23303 | 0.23303 | 0.000123201 |
| hsa-miR-329 | CNOT6L | 0.232785 | 0.232785 | 0.147710575 |
| hsa-miR-495 | CRTAM | -0.227644 | 0.227644 | 0.180044629 |
| hsa-miR-495 | CEP63 | 0.227355 | 0.227355 | 0.001304789 |
| hsa-miR-487b | WWC2 | 0.226027 | 0.226027 | 0.039680005 |
| hsa-miR-495 | CORIN | -0.225963 | 0.225963 | 0.077670991 |
| hsa-miR-495 | MAPKAPK5 | 0.225207 | 0.225207 | 0.021307333 |
| hsa-miR-329 | AFF1 | -0.224236 | 0.224236 | 0.009971239 |
| hsa-miR-329 | DIDO1 | 0.223946 | 0.223946 | 0.069435984 |
| hsa-miR-656 | NRXN1 | -0.22273 | 0.22273 | 0.052266847 |
| hsa-miR-495 | FBN2 | 0.222555 | 0.222555 | 0.067718785 |
| hsa-miR-329 | VAPA | 0.222466 | 0.222466 | 0.190734896 |
| hsa-miR-487b | SPO11 | -0.220834 | 0.220834 | 0.039897711 |
| hsa-miR-329 | KCNS2 | -0.219584 | 0.219584 | 0.00022068 |
| hsa-miR-329 | JPH1 | 0.218408 | 0.218408 | 0.002991701 |
| hsa-miR-656 | ZNF607 | 0.218391 | 0.218391 | 0.159094734 |
| hsa-miR-495 | RABGEF1 | 0.21474 | 0.21474 | 0.097585293 |
| hsa-miR-495 | KCTD9 | 0.214403 | 0.214403 | 0.026253053 |
| hsa-miR-329 | WDR17 | -0.214163 | 0.214163 | 0.054989887 |
| hsa-miR-495 | PTGES3 | 0.213565 | 0.213565 | 0.001176289 |
| hsa-miR-329 | SLC36A1 | 0.212702 | 0.212702 | 0.00061252 |
| hsa-miR-656 | ZNF193 | 0.210798 | 0.210798 | 0.209996758 |
| hsa-miR-329 | KIF5C | 0.210312 | 0.210312 | 8.49755E-05 |
| hsa-miR-487b | ARPP19 | -0.208483 | 0.208483 | 0.198557885 |

| | | | | |
|---|---|---|---|---|
| hsa-miR-329 | MAP3K15 | -0.208463 | 0.208463 | 0.16040544 |
| hsa-miR-495 | MDC1 | 0.206261 | 0.206261 | 0.059453984 |
| hsa-miR-656 | ZNF449 | 0.203453 | 0.203453 | 0.09988726 |
| hsa-miR-329 | SLC34A1 | -0.203354 | 0.203354 | 0.032692316 |
| hsa-miR-329 | MTF2 | 0.203093 | 0.203093 | 0.2317073 |
| hsa-miR-410 | LPCAT2 | -0.19907 | 0.19907 | 0.146435682 |
| hsa-miR-495 | OGFRL1 | 0.19508 | 0.19508 | 0.00095628 |
| hsa-miR-410 | LPPR5 | -0.194447 | 0.194447 | 0.229947958 |
| hsa-miR-656 | PPP2R5A | 0.193667 | 0.193667 | 0.027752777 |
| hsa-miR-487b | PTPN18 | -0.185482 | 0.185482 | 0.03881097 |
| hsa-miR-487b | LRP6 | -0.1779 | 0.1779 | 0.071710752 |
| hsa-miR-410 | PGAM4 | 0.173735 | 0.173735 | 0.026451762 |
| hsa-miR-410 | PRKD1 | -0.171355 | 0.171355 | 0.176651692 |
| hsa-miR-329 | GTPBP10 | -0.159318 | 0.159318 | 0.03074439 |
| hsa-miR-495 | DCDC2 | -0.156511 | 0.156511 | 0.103951652 |
| hsa-miR-495 | HBEGF | -0.138592 | 0.138592 | 0.116378177 |
| hsa-miR-495 | DDX20 | 0.126859 | 0.126859 | 0.249814562 |

| edge -log q value |
|---|
| 27.9180224 |
| 27.9180224 |
| 27.22605935 |
| 27.9180224 |
| 27.22605935 |
| 26.31243806 |
| 27.9180224 |
| 27.9180224 |
| 27.9180224 |
| 27.9180224 |
| 27.9180224 |
| 7.207974525 |
| 9.726901351 |
| 27.9180224 |
| 27.9180224 |
| 20.4987408 |
| 27.9180224 |
| 27.9180224 |
| 7.228147193 |
| 27.9180224 |
| 27.9180224 |
| 13.77177945 |
| 5.851279035 |
| 27.9180224 |
| 27.9180224 |
| 27.9180224 |
| 27.9180224 |
| 5.334414536 |
| 15.20113649 |
| 27.9180224 |
| 27.9180224 |
| 27.9180224 |
| 5.529312287 |
| 14.2994024 |
| 21.33531808 |
| 19.85409177 |
| 27.9180224 |
| 27.9180224 |
| 27.9180224 |
| 8.605310133 |
| 15.02109813 |
| 13.13615323 |
| 14.08455509 |
| 17.4721711 |
| 27.9180224 |
| 10.04984261 |
| 27.9180224 |
| 6.332651095 |
| 16.58250884 |
| 18.08584067 |
| 24.83470199 |
| 19.50739894 |

| |
|---|
| 4.883230204 |
| 14.6603975 |
| 16.62978519 |
| 27.9180224 |
| 25.72673256 |
| 2.629976622 |
| 27.9180224 |
| 3.083664947 |
| 27.9180224 |
| 22.93211641 |
| 26.82266986 |
| 11.19455256 |
| 25.03507458 |
| 27.9180224 |
| 24.2893516 |
| 22.36924567 |
| 27.9180224 |
| 7.814698637 |
| 27.9180224 |
| 18.25344517 |
| 2.549968112 |
| 27.9180224 |
| 10.73605064 |
| 4.484376278 |
| 18.21478037 |
| 27.9180224 |
| 6.613651871 |
| 9.799798308 |
| 27.9180224 |
| 12.14477745 |
| 11.23339538 |
| 27.9180224 |
| 5.445284845 |
| 27.9180224 |
| 17.10344721 |
| 6.090405626 |
| 26.82266986 |
| 27.9180224 |
| 27.9180224 |
| 5.134747654 |
| 27.9180224 |
| 14.13854238 |
| 25.97685722 |
| 21.93061043 |
| 15.5303669 |
| 19.87781975 |
| 7.441606021 |
| 2.617021805 |
| 8.57021214 |
| 9.690531579 |
| 7.844785747 |
| 27.9180224 |
| 21.55110979 |

| |
|---|
| 9.622887675 |
| 5.180749361 |
| 7.219532642 |
| 7.971290711 |
| 7.060274865 |
| 24.2893516 |
| 11.90345807 |
| 20.7586134 |
| 7.420870325 |
| 27.9180224 |
| 11.42174205 |
| 22.56833578 |
| 27.9180224 |
| 27.9180224 |
| 16.54757516 |
| 6.973905345 |
| 27.9180224 |
| 1.830162619 |
| 9.265019238 |
| 16.51935313 |
| 12.61939111 |
| 20.35308105 |
| 11.14856893 |
| 25.72673256 |
| 16.45436095 |
| 5.651529994 |
| 6.219431911 |
| 9.410558156 |
| 27.9180224 |
| 27.9180224 |
| 6.324539182 |
| 9.579945694 |
| 18.64292694 |
| 27.9180224 |
| 4.302236288 |
| 27.9180224 |
| 27.9180224 |
| 27.9180224 |
| 25.97685722 |
| 27.9180224 |
| 16.060799 |
| 27.9180224 |
| 17.42615373 |
| 4.850197634 |
| 10.76777307 |
| 6.112508119 |
| 11.05866234 |
| 27.9180224 |
| 25.84421802 |
| 23.44009405 |
| 15.2360104 |
| 7.180405032 |
| 13.13223743 |

| |
|---|
| 6.231840027 |
| 19.39125015 |
| 16.00854104 |
| 11.66866338 |
| 27.9180224 |
| 13.51858947 |
| 11.62576405 |
| 20.54358706 |
| 12.97585281 |
| 13.51093057 |
| 7.756215416 |
| 3.633015752 |
| 25.72673256 |
| 27.9180224 |
| 3.661988766 |
| 20.97278666 |
| 6.128420598 |
| 7.132098007 |
| 19.27058824 |
| 12.78753721 |
| 27.9180224 |
| 1.939050565 |
| 14.1990806 |
| 8.028913023 |
| 12.91912149 |
| 2.285998095 |
| 11.11383099 |
| 8.81850665 |
| 21.0874853 |
| 10.44987253 |
| 16.85661714 |
| 11.28889724 |
| 8.973993819 |
| 7.042364903 |
| 19.31434734 |
| 7.891949821 |
| 15.8185023 |
| 21.61006031 |
| 20.90447362 |
| 27.22605935 |
| 6.138726656 |
| 16.59852791 |
| 12.56752277 |
| 27.9180224 |
| 8.425651197 |
| 17.43377866 |
| 21.06467582 |
| 6.376627417 |
| 16.14769379 |
| 27.9180224 |
| 24.7074651 |
| 17.80523103 |
| 22.19425361 |

| |
|---|
| 4.203646559 |
| 18.63602211 |
| 7.711837457 |
| 8.262829463 |
| 24.83470199 |
| 12.29490043 |
| 22.00183629 |
| 6.083704411 |
| 10.60313684 |
| 3.767254434 |
| 2.405116813 |
| 19.82462241 |
| 27.9180224 |
| 5.565046248 |
| 9.110991008 |
| 6.945722273 |
| 4.686962078 |
| 9.582644252 |
| 11.85934527 |
| 27.9180224 |
| 27.9180224 |
| 4.352994766 |
| 10.88982375 |
| 10.49769565 |
| 6.57113296 |
| 22.1596845 |
| 13.96659236 |
| 14.66792463 |
| 21.99155407 |
| 19.93672223 |
| 5.445166361 |
| 11.12744055 |
| 4.986344664 |
| 3.335290862 |
| 12.82099918 |
| 7.88259014 |
| 12.07162678 |
| 8.977377277 |
| 7.039678299 |
| 12.82846443 |
| 4.768676695 |
| 2.791508652 |
| 21.23216736 |
| 2.864550646 |
| 12.75666557 |
| 5.983237723 |
| 13.89405436 |
| 25.43994374 |
| 9.458122285 |
| 5.796585735 |
| 8.54544876 |
| 2.829745146 |
| 17.86549231 |

| |
|---|
| 11.69210865 |
| 26.31243806 |
| 10.07853348 |
| 12.80571648 |
| 5.139332533 |
| 7.177740187 |
| 8.980560469 |
| 5.236359361 |
| 3.543001578 |
| 17.96435682 |
| 1.747564365 |
| 2.316825003 |
| 25.36019896 |
| 9.042771544 |
| 17.52761318 |
| 2.782739972 |
| 3.328000083 |
| 22.32810793 |
| 20.72881447 |
| 27.9180224 |
| 10.51249893 |
| 3.315804626 |
| 7.253417148 |
| 16.21350429 |
| 12.46578172 |
| 3.171822317 |
| 3.274139996 |
| 6.506646765 |
| 24.63080244 |
| 6.472399866 |
| 9.154910629 |
| 27.9180224 |
| 10.63541269 |
| 4.311622162 |
| 13.35719867 |
| 7.496217189 |
| 6.542051889 |
| 19.07122548 |
| 21.37391135 |
| 9.207397307 |
| 27.9180224 |
| 12.20826106 |
| 1.582375513 |
| 4.53743663 |
| 18.41509773 |
| 5.830332602 |
| 8.607396628 |
| 5.108358705 |
| 23.99570932 |
| 7.945205866 |
| 4.921694399 |
| 19.56793924 |
| 25.97685722 |

| |
|---|
| 5.375495598 |
| 3.753205425 |
| 27.9180224 |
| 19.21122629 |
| 23.6109433 |
| 8.61409436 |
| 16.06729561 |
| 11.04787017 |
| 16.09812391 |
| 11.4392522 |
| 9.018580492 |
| 8.111969638 |
| 2.738355289 |
| 14.29540951 |
| 14.06637904 |
| 7.297666575 |
| 12.82289461 |
| 27.22605935 |
| 6.304410034 |
| 4.956612048 |
| 26.31243806 |
| 8.288240476 |
| 11.21157786 |
| 12.38882236 |
| 4.139180724 |
| 2.461212091 |
| 23.36468131 |
| 6.444081624 |
| 4.199706934 |
| 3.773280631 |
| 8.178372713 |
| 1.422186799 |
| 11.80777661 |
| 13.34269686 |
| 4.061587486 |
| 3.794740924 |
| 27.9180224 |
| 3.683180113 |
| 1.986101484 |
| 2.708194182 |
| 8.526457165 |
| 5.919045618 |
| 4.654381067 |
| 3.4905174 |
| 10.21313029 |
| 10.4957499 |
| 2.854020772 |
| 10.47990861 |
| 8.24912327 |
| 5.960371029 |
| 9.639811453 |
| 21.68235014 |
| 10.65552217 |

| |
|---|
| 15.40112251 |
| 6.269498601 |
| 8.980560469 |
| 18.43442971 |
| 6.989433753 |
| 8.653043754 |
| 11.35542501 |
| 27.9180224 |
| 27.9180224 |
| 2.963359556 |
| 13.11371215 |
| 14.75363093 |
| 14.87865906 |
| 1.595658418 |
| 9.072839446 |
| 13.77367643 |
| 2.565390404 |
| 16.22247524 |
| 3.383175265 |
| 1.423883954 |
| 2.80415703 |
| 8.876309933 |
| 20.24456922 |
| 4.214893183 |
| 6.079644754 |
| 17.33884076 |
| 1.952795548 |
| 8.701138928 |
| 14.46980895 |
| 11.9320678 |
| 5.761697724 |
| 27.22605935 |
| 4.958389032 |
| 27.22605935 |
| 2.082874736 |
| 4.13636824 |
| 14.55746994 |
| 18.10668122 |
| 4.222487551 |
| 23.97658997 |
| 5.061277562 |
| 8.359455721 |
| 4.570869006 |
| 7.624779382 |
| 8.454280043 |
| 6.112773718 |
| 18.31357132 |
| 1.819026148 |
| 5.31181159 |
| 12.88838221 |
| 2.079904435 |
| 16.39425315 |
| 7.50372709 |

| |
|---|
| 21.3936402 |
| 1.593211686 |
| 15.03740569 |
| 4.022153177 |
| 7.550128129 |
| 27.9180224 |
| 8.13847389 |
| 4.740657812 |
| 18.27884685 |
| 8.861892826 |
| 3.029133381 |
| 11.15005591 |
| 5.069855196 |
| 1.496208701 |
| 27.9180224 |
| 3.988712422 |
| 1.422186799 |
| 1.564210679 |
| 18.18714135 |
| 2.40000141 |
| 12.45179964 |
| 2.072929303 |
| 1.647960835 |
| 7.974622581 |
| 4.729849151 |
| 9.089917559 |
| 1.418851474 |
| 7.263844782 |
| 27.22605935 |
| 9.66348499 |
| 7.001224348 |
| 4.07506798 |
| 5.385618848 |
| 6.753532139 |
| 4.014609563 |
| 2.486030545 |
| 3.831317948 |
| 4.814562401 |
| 9.728212864 |
| 5.297051756 |
| 5.92388579 |
| 1.89435608 |
| 7.47814883 |
| 4.273791951 |
| 20.04349535 |
| 4.473087808 |
| 4.006277582 |
| 8.555697506 |
| 2.438777701 |
| 5.975510745 |
| 10.80384826 |
| 5.258880631 |
| 1.513690187 |

| |
|---|
| 9.45530194 |
| 11.33070282 |
| 9.9410943 |
| 6.883363295 |
| 13.61627142 |
| 5.377557846 |
| 7.243411669 |
| 2.61069049 |
| 11.11474869 |
| 22.32105449 |
| 8.476294462 |
| 3.604521704 |
| 18.09382904 |
| 8.320789387 |
| 1.705157936 |
| 4.949684801 |
| 27.9180224 |
| 5.946536286 |
| 3.194978637 |
| 17.51376951 |
| 11.75166916 |
| 8.268092821 |
| 6.873362979 |
| 3.382468241 |
| 2.837549845 |
| 2.981809279 |
| 2.266930019 |
| 11.06060342 |
| 3.522707663 |
| 2.385216312 |
| 3.168871347 |
| 5.303887938 |
| 12.1770924 |
| 3.612518096 |
| 3.99234936 |
| 3.36501318 |
| 5.726663399 |
| 12.45920685 |
| 5.225730685 |
| 3.074420459 |
| 3.372754237 |
| 2.3198914 |
| 7.802338211 |
| 3.712985253 |
| 3.018300694 |
| 1.502009385 |
| 1.73345869 |
| 2.17495557 |
| 5.088073376 |
| 4.006337505 |
| 2.886991426 |
| 9.586774736 |
| 7.471599252 |

| |
|---|
| 3.600231716 |
| 6.667427753 |
| 6.057739091 |
| 27.9180224 |
| 7.238147765 |
| 5.774654547 |
| 27.9180224 |
| 2.23698366 |
| 10.39686131 |
| 3.825690214 |
| 6.71781371 |
| 3.865940103 |
| 4.048727022 |
| 2.291821462 |
| 1.466644926 |
| 8.912520024 |
| 6.44415817 |
| 3.426317082 |
| 8.018034767 |
| 1.798542339 |
| 10.11998239 |
| 1.818500261 |
| 4.921694399 |
| 4.251240965 |
| 1.723988628 |
| 2.635214196 |
| 4.736681617 |
| 3.29750939 |
| 7.585249701 |
| 1.700624221 |
| 6.784784695 |
| 7.552275933 |
| 18.46888646 |
| 2.710369811 |
| 3.053821797 |
| 4.693754297 |
| 7.805866175 |
| 1.740595397 |
| 11.76354756 |
| 18.87286636 |
| 23.78499778 |
| 5.965909505 |
| 3.508685794 |
| 3.167040143 |
| 5.415838218 |
| 8.268092821 |
| 7.558872272 |
| 1.735579975 |
| 6.133032062 |
| 12.22171654 |
| 3.112809224 |
| 4.445045959 |
| 13.59828483 |

| |
|---|
| 3.683747296 |
| 1.472157349 |
| 3.747995238 |
| 6.728388022 |
| 5.305655086 |
| 3.600352711 |
| 4.899572828 |
| 2.785035005 |
| 3.685149754 |
| 4.061172136 |
| 8.455746609 |
| 3.885167737 |
| 4.134050765 |
| 2.330682715 |
| 4.934235258 |
| 2.479315441 |
| 5.836240888 |
| 3.566984189 |
| 3.274139996 |
| 1.856354506 |
| 3.734697373 |
| 2.759664102 |
| 3.457609507 |
| 1.590660087 |
| 1.880138682 |
| 2.407657306 |
| 3.911485811 |
| 2.604524785 |
| 1.943667788 |
| 9.001690295 |
| 1.912500495 |
| 1.714550522 |
| 6.641713628 |
| 3.226907862 |
| 2.555273444 |
| 3.848703991 |
| 4.608050419 |
| 2.667350044 |
| 2.951393004 |
| 2.692391657 |
| 1.656870795 |
| 3.221436329 |
| 8.418797952 |
| 5.811913074 |
| 1.838255444 |
| 2.327028482 |
| 3.639972991 |
| 2.900605978 |
| 6.745390571 |
| 7.397928968 |
| 1.560663187 |
| 9.37314722 |
| 1.616674608 |

| |
|---|
| 1.830050671 |
| 2.822552639 |
| 2.303713127 |
| 3.420615221 |
| 1.462280343 |
| 1.921168978 |
| 6.952460058 |
| 1.469902264 |
| 3.584419389 |
| 3.249052345 |
| 2.635114585 |
| 3.632432492 |
| 1.733575327 |
| 3.482047747 |
| 2.263829377 |
| 2.150910248 |
| 1.387036389 |
