# Supplementary material for: An imprinted non-coding genomic cluster at 14q32 defines clinically relevant molecular subtypes in osteosarcoma across multiple independent datasets
Source: J Hematol Oncol. 2017 May 15;10:107. doi: 10.1186/s13045-017-0465-4 (PMC5433149; doi:10.1186/s13045-017-0465-4)
Supplement: Supplementary file 14 — Supplementary PUMA methods. This file is the detailed protocol followed for the implementation of PUMA in order to reconstruct networks for high- and low-risk osteosarcoma subtypes. (PDF 998 kb) [file 13045_2017_465_MOESM14_ESM.pdf]

# Significant network GO categories

| GO.ID | Term | Annotated | Significant | Expected | Classic_p_value |
|---|---|---|---|---|---|
| GO:0031573 | intra-S DNA damage checkpoint | 11 | 4 | 0.42 | 0.00055 |
| GO:0006470 | protein dephosphorylation | 172 | 16 | 6.55 | 0.00089 |
| GO:0045667 | regulation of osteoblast differentiation | 103 | 11 | 3.92 | 0.00185 |
| GO:0032026 | response to magnesium ion | 15 | 4 | 0.57 | 0.00203 |
| GO:0007141 | male meiosis I | 16 | 4 | 0.61 | 0.00262 |
| GO:0021537 | telencephalon development | 191 | 16 | 7.27 | 0.00264 |
| GO:0035581 | sequestering of extracellular ligand fro… | 8 | 3 | 0.3 | 0.00266 |
| GO:0030900 | forebrain development | 321 | 23 | 12.22 | 0.0028 |
| GO:0016311 | dephosphorylation | 360 | 25 | 13.7 | 0.00285 |
| GO:0071383 | cellular response to steroid hormone sti... | 126 | 12 | 4.8 | 0.0031 |
| GO:0046777 | protein autophosphorylation | 213 | 17 | 8.11 | 0.00326 |
| GO:0045736 | negative regulation of cyclin-dependent ... | 28 | 5 | 1.07 | 0.00373 |
| GO:1990542 | mitochondrial transmembrane transport | 40 | 6 | 1.52 | 0.00378 |
| GO:0007548 | sex differentiation | 253 | 19 | 9.63 | 0.00381 |
| GO:0035583 | sequestering of TGFbeta in extracellular... | 3 | 2 | 0.11 | 0.00423 |
| GO:0038007 | netrin-activated signaling pathway | 3 | 2 | 0.11 | 0.00423 |
| GO:0097091 | synaptic vesicle clustering | 3 | 2 | 0.11 | 0.00423 |
| GO:0030278 | regulation of ossification | 167 | 14 | 6.36 | 0.00472 |
| GO:0046626 | regulation of insulin receptor signaling... | 42 | 6 | 1.6 | 0.00484 |
| GO:1901654 | response to ketone | 117 | 11 | 4.45 | 0.005 |
| GO:0048545 | response to steroid hormone | 358 | 24 | 13.63 | 0.00531 |
| GO:0048012 | hepatocyte growth factor receptor signal... | 10 | 3 | 0.38 | 0.00539 |
| GO:0007568 | aging | 226 | 17 | 8.6 | 0.00591 |
| GO:0008380 | RNA splicing | 305 | 21 | 11.61 | 0.00657 |
| GO:0007062 | sister chromatid cohesion | 32 | 5 | 1.22 | 0.00675 |
| GO:0048010 | vascular endothelial growth factor recep... | 122 | 11 | 4.64 | 0.00683 |
| GO:0042063 | gliogenesis | 193 | 15 | 7.35 | 0.00704 |
| GO:0000076 | DNA replication checkpoint | 11 | 3 | 0.42 | 0.0072 |
| GO:0010586 | miRNA metabolic process | 11 | 3 | 0.42 | 0.0072 |
| GO:0014067 | negative regulation of phosphatidylinosi... | 11 | 3 | 0.42 | 0.0072 |
| GO:0032365 | intracellular lipid transport | 21 | 4 | 0.8 | 0.00742 |
| GO:0071548 | response to dexamethasone | 21 | 4 | 0.8 | 0.00742 |
| GO:0001503 | ossification | 349 | 23 | 13.28 | 0.00766 |
| GO:0000055 | ribosomal large subunit export from nucl... | 4 | 2 | 0.15 | 0.00825 |
| GO:0051409 | response to nitrosative stress | 4 | 2 | 0.15 | 0.00825 |
| GO:0051964 | negative regulation of synapse assembly | 4 | 2 | 0.15 | 0.00825 |
| GO:0071880 | adenylate cyclase-activating adrenergic ... | 4 | 2 | 0.15 | 0.00825 |
| GO:1902202 | regulation of hepatocyte growth factor r... | 4 | 2 | 0.15 | 0.00825 |
| GO:0045930 | negative regulation of mitotic cell cycl... | 216 | 16 | 8.22 | 0.00858 |
| GO:0043470 | regulation of carbohydrate catabolic pro... | 34 | 5 | 1.29 | 0.00876 |
| GO:0043471 | regulation of cellular carbohydrate cata... | 34 | 5 | 1.29 | 0.00876 |
| GO:0000724 | double-strand break repair via homologou. | 62 | 7 | 2.36 | 0.00896 |
| GO:0000725 | recombinational repair | 62 | 7 | 2.36 | 0.00896 |
| GO:0097305 | response to alcohol | 275 | 19 | 10.47 | 0.00912 |
| GO:0033235 | positive regulation of protein sumoylati... | 12 | 3 | 0.46 | 0.00933 |
| GO:0002768 | immune response-regulating cell surface .. | 316 | 21 | 12.03 | 0.00964 |
| GO:0051384 | response to glucocorticoid | 128 | 11 | 4.87 | 0.00968 |
| GO:0048015 | phosphatidylinositol-mediated signaling | 182 | 14 | 6.93 | 0.00985 |
| GO:0048017 | inositol lipid-mediated signaling | 182 | 14 | 6.93 | 0.00985 |
| GO:0000077 | DNA damage checkpoint | 146 | 12 | 5.56 | 0.00993 |
| GO:0002068 | glandular epithelial cell development | 23 | 4 | 0.88 | 0.01034 |
| GO:0032434 | regulation of proteasomal ubiquitin-depe... | 130 | 11 | 4.95 | 0.01081 |

| | | | | | |
|---|---|---|---|---|---|
| GO:0035335 | peptidyl-tyrosine dephosphorylation | 96 | 9 | 3.65 | 0.01082 |
| GO:1903322 | positive regulation of protein modificat... | 166 | 13 | 6.32 | 0.01097 |
| GO:0007029 | endoplasmic reticulum organization | 36 | 5 | 1.37 | 0.01115 |
| GO:1900076 | regulation of cellular response to insul... | 50 | 6 | 1.9 | 0.01136 |
| GO:0007093 | mitotic cell cycle checkpoint | 167 | 13 | 6.36 | 0.01149 |
| GO:0045722 | positive regulation of gluconeogenesis | 13 | 3 | 0.49 | 0.01179 |
| GO:0045778 | positive regulation of ossification | 81 | 8 | 3.08 | 0.0118 |
| GO:0031641 | regulation of myelination | 24 | 4 | 0.91 | 0.01204 |
| GO:1903959 | regulation of anion transmembrane transp. | 24 | 4 | 0.91 | 0.01204 |
| GO:0042552 | myelination | 98 | 9 | 3.73 | 0.01229 |
| GO:0043123 | positive regulation of I-kappaB kinase/N... | 169 | 13 | 6.43 | 0.01261 |
| GO:0044773 | mitotic DNA damage checkpoint | 99 | 9 | 3.77 | 0.01308 |
| GO:0006397 | mRNA processing | 387 | 24 | 14.73 | 0.01322 |
| GO:0001996 | positive regulation of heart rate by epi... | 5 | 2 | 0.19 | 0.0134 |
| GO:0018401 | peptidyl-proline hydroxylation to 4-hydr... | 5 | 2 | 0.19 | 0.0134 |
| GO:0034392 | negative regulation of smooth muscle cel.. | 5 | 2 | 0.19 | 0.0134 |
| GO:0035524 | proline transmembrane transport | 5 | 2 | 0.19 | 0.0134 |
| GO:0070141 | response to UV-A | 5 | 2 | 0.19 | 0.0134 |
| GO:0070295 | renal water absorption | 5 | 2 | 0.19 | 0.0134 |
| GO:0070572 | positive regulation of neuron projection... | 5 | 2 | 0.19 | 0.0134 |
| GO:0071692 | protein localization to extracellular re... | 5 | 2 | 0.19 | 0.0134 |
| GO:0071694 | maintenance of protein location in extra... | 5 | 2 | 0.19 | 0.0134 |
| GO:0033673 | negative regulation of kinase activity | 209 | 15 | 7.96 | 0.01401 |
| GO:0010675 | regulation of cellular carbohydrate meta... | 135 | 11 | 5.14 | 0.01407 |
| GO:0008286 | insulin receptor signaling pathway | 172 | 13 | 6.55 | 0.01443 |
| GO:0038093 | Fc receptor signaling pathway | 210 | 15 | 7.99 | 0.01458 |
| GO:0015791 | polyol transport | 14 | 3 | 0.53 | 0.01459 |
| GO:0071157 | negative regulation of cell cycle arrest | 14 | 3 | 0.53 | 0.01459 |
| GO:1900115 | extracellular regulation of signal trans... | 14 | 3 | 0.53 | 0.01459 |
| GO:1900116 | extracellular negative regulation of sig... | 14 | 3 | 0.53 | 0.01459 |
| GO:0031570 | DNA integrity checkpoint | 154 | 12 | 5.86 | 0.01472 |
| GO:0007272 | ensheathment of neurons | 101 | 9 | 3.84 | 0.01477 |
| GO:0008366 | axon ensheathment | 101 | 9 | 3.84 | 0.01477 |
| GO:0071385 | cellular response to glucocorticoid stim... | 39 | 5 | 1.48 | 0.01552 |
| GO:0070306 | lens fiber cell differentiation | 26 | 4 | 0.99 | 0.01596 |
| GO:0090075 | relaxation of muscle | 26 | 4 | 0.99 | 0.01596 |
| GO:0000075 | cell cycle checkpoint | 232 | 16 | 8.83 | 0.01614 |
| GO:0031960 | response to corticosteroid | 138 | 11 | 5.25 | 0.01635 |
| GO:0043467 | regulation of generation of precursor me... | 70 | 7 | 2.66 | 0.01687 |
| GO:0071384 | cellular response to corticosteroid stim... | 40 | 5 | 1.52 | 0.0172 |
| GO:1903320 | regulation of protein modification by sm... | 234 | 16 | 8.91 | 0.01736 |
| GO:0044774 | mitotic DNA integrity checkpoint | 104 | 9 | 3.96 | 0.0176 |
| GO:0014020 | primary neural tube formation | 87 | 8 | 3.31 | 0.01761 |
| GO:0010001 | glial cell differentiation | 158 | 12 | 6.01 | 0.0177 |
| GO:2000310 | regulation of N-methyl-D-aspartate selec... | 15 | 3 | 0.57 | 0.01773 |
| GO:0021543 | pallium development | 122 | 10 | 4.64 | 0.01803 |
| GO:0043010 | camera-type eye development | 275 | 18 | 10.47 | 0.01816 |
| GO:0006110 | regulation of glycolytic process | 27 | 4 | 1.03 | 0.01818 |
| GO:0061028 | establishment of endothelial barrier | 27 | 4 | 1.03 | 0.01818 |
| GO:0000375 | RNA splicing | via transest | 216 | 15 | 8.22 |
| GO:0006469 | negative regulation of protein kinase ac... | 197 | 14 | 7.5 | 0.01862 |
| GO:0001933 | negative regulation of protein phosphory... | 276 | 18 | 10.51 | 0.01877 |
| GO:0001654 | eye development | 317 | 20 | 12.07 | 0.019 |

| | | | | | |
|---|---|---|---|---|---|
| GO:0010676 | positive regulation of cellular carbohyd... | 56 | 6 | 2.13 | 0.0192 |
| GO:0019935 | cyclic-nucleotide-mediated signaling | 56 | 6 | 2.13 | 0.0192 |
| GO:0000054 | ribosomal subunit export from nucleus | 6 | 2 | 0.23 | 0.01959 |
| GO:0014870 | response to muscle inactivity | 6 | 2 | 0.23 | 0.01959 |
| GO:0014877 | response to muscle inactivity involved i... | 6 | 2 | 0.23 | 0.01959 |
| GO:0014894 | response to denervation involved in regu... | 6 | 2 | 0.23 | 0.01959 |
| GO:0016198 | axon choice point recognition | 6 | 2 | 0.23 | 0.01959 |
| GO:0033750 | ribosome localization | 6 | 2 | 0.23 | 0.01959 |
| GO:0033753 | establishment of ribosome localization | 6 | 2 | 0.23 | 0.01959 |
| GO:0060024 | rhythmic synaptic transmission | 6 | 2 | 0.23 | 0.01959 |
| GO:0060136 | embryonic process involved in female pre. | 6 | 2 | 0.23 | 0.01959 |
| GO:0060137 | maternal process involved in parturition | 6 | 2 | 0.23 | 0.01959 |
| GO:0060179 | male mating behavior | 6 | 2 | 0.23 | 0.01959 |
| GO:0071426 | ribonucleoprotein complex export from nu. | 6 | 2 | 0.23 | 0.01959 |
| GO:0071428 | rRNA-containing ribonucleoprotein comple | 6 | 2 | 0.23 | 0.01959 |
| GO:0090557 | establishment of endothelial intestinal ... | 6 | 2 | 0.23 | 0.01959 |
| GO:1901897 | regulation of relaxation of cardiac musc... | 6 | 2 | 0.23 | 0.01959 |
| GO:2000628 | regulation of miRNA metabolic process | 6 | 2 | 0.23 | 0.01959 |
| GO:0014065 | phosphatidylinositol 3-kinase signaling | 89 | 8 | 3.39 | 0.01995 |
| GO:0006109 | regulation of carbohydrate metabolic pro... | 143 | 11 | 5.44 | 0.02075 |
| GO:0032436 | positive regulation of proteasomal ubiqu... | 57 | 6 | 2.17 | 0.02079 |
| GO:0045669 | positive regulation of osteoblast differ... | 57 | 6 | 2.17 | 0.02079 |
| GO:0006302 | double-strand break repair | 125 | 10 | 4.76 | 0.021 |
| GO:0071901 | negative regulation of protein serine/th... | 125 | 10 | 4.76 | 0.021 |
| GO:0042326 | negative regulation of phosphorylation | 341 | 21 | 12.98 | 0.021 |
| GO:0043584 | nose development | 16 | 3 | 0.61 | 0.02122 |
| GO:0055119 | relaxation of cardiac muscle | 16 | 3 | 0.61 | 0.02122 |
| GO:0033273 | response to vitamin | 74 | 7 | 2.82 | 0.02227 |
| GO:0006281 | DNA repair | 385 | 23 | 14.66 | 0.02231 |
| GO:1901992 | positive regulation of mitotic cell cycl... | 43 | 5 | 1.64 | 0.02294 |
| GO:0001825 | blastocyst formation | 29 | 4 | 1.1 | 0.02319 |
| GO:0016339 | calcium-dependent cell-cell adhesion via... | 29 | 4 | 1.1 | 0.02319 |
| GO:0046627 | negative regulation of insulin receptor ... | 29 | 4 | 1.1 | 0.02319 |
| GO:0051385 | response to mineralocorticoid | 29 | 4 | 1.1 | 0.02319 |
| GO:0061418 | regulation of transcription from RNA pol... | 29 | 4 | 1.1 | 0.02319 |
| GO:1900449 | regulation of glutamate receptor signali... | 29 | 4 | 1.1 | 0.02319 |
| GO:0007050 | cell cycle arrest | 223 | 15 | 8.49 | 0.02372 |
| GO:0002429 | immune response-activating cell surface .. | 204 | 14 | 7.77 | 0.02432 |
| GO:1901653 | cellular response to peptide | 326 | 20 | 12.41 | 0.02477 |
| GO:0021575 | hindbrain morphogenesis | 44 | 5 | 1.67 | 0.0251 |
| GO:0043502 | regulation of muscle adaptation | 44 | 5 | 1.67 | 0.0251 |
| GO:0045446 | endothelial cell differentiation | 76 | 7 | 2.89 | 0.02538 |
| GO:0032869 | cellular response to insulin stimulus | 225 | 15 | 8.56 | 0.02544 |
| GO:0035116 | embryonic hindlimb morphogenesis | 30 | 4 | 1.14 | 0.02597 |
| GO:1900077 | negative regulation of cellular response... | 30 | 4 | 1.14 | 0.02597 |
| GO:0071407 | cellular response to organic cyclic comp... | 307 | 19 | 11.69 | 0.02604 |
| GO:0014033 | neural crest cell differentiation | 60 | 6 | 2.28 | 0.0261 |
| GO:0001672 | regulation of chromatin assembly or disa... | 7 | 2 | 0.27 | 0.02675 |
| GO:0003097 | renal water transport | 7 | 2 | 0.27 | 0.02675 |
| GO:0003321 | positive regulation of blood pressure by... | 7 | 2 | 0.27 | 0.02675 |
| GO:0006108 | malate metabolic process | 7 | 2 | 0.27 | 0.02675 |
| GO:0006538 | glutamate catabolic process | 7 | 2 | 0.27 | 0.02675 |
| GO:0031642 | negative regulation of myelination | 7 | 2 | 0.27 | 0.02675 |

| | | | | | |
|---|---|---|---|---|---|
| GO:0032933 | SREBP signaling pathway | 7 | 2 | 0.27 | 0.02675 |
| GO:0035372 | protein localization to microtubule | 7 | 2 | 0.27 | 0.02675 |
| GO:0036005 | response to macrophage colony-stimulatin | 7 | 2 | 0.27 | 0.02675 |
| GO:0036006 | cellular response to macrophage colony-s. | 7 | 2 | 0.27 | 0.02675 |
| GO:0043569 | negative regulation of insulin-like grow... | 7 | 2 | 0.27 | 0.02675 |
| GO:0071501 | cellular response to sterol depletion | 7 | 2 | 0.27 | 0.02675 |
| GO:0071346 | cellular response to interferon-gamma | 112 | 9 | 4.26 | 0.02704 |
| GO:0019933 | cAMP-mediated signaling | 45 | 5 | 1.71 | 0.02739 |
| GO:0034508 | centromere complex assembly | 45 | 5 | 1.71 | 0.02739 |
| GO:0090398 | cellular senescence | 45 | 5 | 1.71 | 0.02739 |
| GO:1901989 | positive regulation of cell cycle phase ... | 45 | 5 | 1.71 | 0.02739 |
| GO:0045913 | positive regulation of carbohydrate meta... | 61 | 6 | 2.32 | 0.02805 |
| GO:0007569 | cell aging | 78 | 7 | 2.97 | 0.02876 |
| GO:0006692 | prostanoid metabolic process | 31 | 4 | 1.18 | 0.02895 |
| GO:0006693 | prostaglandin metabolic process | 31 | 4 | 1.18 | 0.02895 |
| GO:0060674 | placenta blood vessel development | 31 | 4 | 1.18 | 0.02895 |
| GO:0002021 | response to dietary excess | 18 | 3 | 0.69 | 0.02924 |
| GO:0003323 | type B pancreatic cell development | 18 | 3 | 0.69 | 0.02924 |
| GO:0071242 | cellular response to ammonium ion | 18 | 3 | 0.69 | 0.02924 |
| GO:0090201 | negative regulation of release of cytoch... | 18 | 3 | 0.69 | 0.02924 |
| GO:0006096 | glycolytic process | 62 | 6 | 2.36 | 0.03009 |
| GO:1901800 | positive regulation of proteasomal prote... | 62 | 6 | 2.36 | 0.03009 |
| GO:0048589 | developmental growth | 397 | 23 | 15.11 | 0.03036 |
| GO:0001649 | osteoblast differentiation | 191 | 13 | 7.27 | 0.03104 |
| GO:0032868 | response to insulin | 272 | 17 | 10.35 | 0.03137 |
| GO:0010907 | positive regulation of glucose metabolic... | 32 | 4 | 1.22 | 0.03212 |
| GO:0030501 | positive regulation of bone mineralizati... | 32 | 4 | 1.22 | 0.03212 |
| GO:0048806 | genitalia development | 47 | 5 | 1.79 | 0.03234 |
| GO:0060042 | retina morphogenesis in camera-type eye | 47 | 5 | 1.79 | 0.03234 |
| GO:0071375 | cellular response to peptide hormone sti... | 315 | 19 | 11.99 | 0.03268 |
| GO:0000377 | RNA splicing | via transest | 213 | 14 | 8.11 |
| GO:0000398 | mRNA splicing | via spliceos | 213 | 14 | 8.11 |
| GO:0002757 | immune response-activating signal transd. | 337 | 20 | 12.83 | 0.03352 |
| GO:0033233 | regulation of protein sumoylation | 19 | 3 | 0.72 | 0.03376 |
| GO:0071549 | cellular response to dexamethasone stimu | 19 | 3 | 0.72 | 0.03376 |
| GO:0001938 | positive regulation of endothelial cell ... | 64 | 6 | 2.44 | 0.03447 |
| GO:0044275 | cellular carbohydrate catabolic process | 64 | 6 | 2.44 | 0.03447 |
| GO:0000819 | sister chromatid segregation | 117 | 9 | 4.45 | 0.0345 |
| GO:0006991 | response to sterol depletion | 8 | 2 | 0.3 | 0.03478 |
| GO:0014820 | tonic smooth muscle contraction | 8 | 2 | 0.3 | 0.03478 |
| GO:0014854 | response to inactivity | 8 | 2 | 0.3 | 0.03478 |
| GO:0035864 | response to potassium ion | 8 | 2 | 0.3 | 0.03478 |
| GO:0045842 | positive regulation of mitotic metaphase... | 8 | 2 | 0.3 | 0.03478 |
| GO:0046886 | positive regulation of hormone biosynthe... | 8 | 2 | 0.3 | 0.03478 |
| GO:0060059 | embryonic retina morphogenesis in camera | 8 | 2 | 0.3 | 0.03478 |
| GO:0071166 | ribonucleoprotein complex localization | 8 | 2 | 0.3 | 0.03478 |
| GO:1901077 | regulation of relaxation of muscle | 8 | 2 | 0.3 | 0.03478 |
| GO:1901970 | positive regulation of mitotic sister ch... | 8 | 2 | 0.3 | 0.03478 |
| GO:1902101 | positive regulation of metaphase/anaphas. | 8 | 2 | 0.3 | 0.03478 |
| GO:0001841 | neural tube formation | 99 | 8 | 3.77 | 0.03494 |
| GO:0031644 | regulation of neurological system proces... | 48 | 5 | 1.83 | 0.03501 |
| GO:0051348 | negative regulation of transferase activ... | 297 | 18 | 11.31 | 0.03548 |
| GO:0003401 | axis elongation | 33 | 4 | 1.26 | 0.03549 |

| | | | | | |
|---|---|---|---|---|---|
| GO:0006111 | regulation of gluconeogenesis | 33 | 4 | 1.26 | 0.03549 |
| GO:0050854 | regulation of antigen receptor-mediated ... | 33 | 4 | 1.26 | 0.03549 |
| GO:0008406 | gonad development | 195 | 13 | 7.42 | 0.03582 |
| GO:0001843 | neural tube closure | 82 | 7 | 3.12 | 0.03643 |
| GO:0002088 | lens development in camera-type eye | 65 | 6 | 2.47 | 0.03681 |
| GO:0010565 | regulation of cellular ketone metabolic ... | 176 | 12 | 6.7 | 0.03688 |
| GO:0031396 | regulation of protein ubiquitination | 216 | 14 | 8.22 | 0.03698 |
| GO:0007517 | muscle organ development | 320 | 19 | 12.18 | 0.03743 |
| GO:0000086 | G2/M transition of mitotic cell cycle | 157 | 11 | 5.98 | 0.03767 |
| GO:0038095 | Fc-epsilon receptor signaling pathway | 157 | 11 | 5.98 | 0.03767 |
| GO:2001236 | regulation of extrinsic apoptotic signal... | 157 | 11 | 5.98 | 0.03767 |
| GO:0000409 | regulation of transcription by galactose | 1 | 1 | 0.04 | 0.03807 |
| GO:0000411 | positive regulation of transcription by ... | 1 | 1 | 0.04 | 0.03807 |
| GO:0000431 | regulation of transcription from RNA pol... | 1 | 1 | 0.04 | 0.03807 |
| GO:0000435 | positive regulation of transcription fro... | 1 | 1 | 0.04 | 0.03807 |
| GO:0000964 | mitochondrial RNA 5'-end processing | 1 | 1 | 0.04 | 0.03807 |
| GO:0002355 | detection of tumor cell | 1 | 1 | 0.04 | 0.03807 |
| GO:0003061 | positive regulation of the force of hear... | 1 | 1 | 0.04 | 0.03807 |
| GO:0009088 | threonine biosynthetic process | 1 | 1 | 0.04 | 0.03807 |
| GO:0009231 | riboflavin biosynthetic process | 1 | 1 | 0.04 | 0.03807 |
| GO:0009237 | siderophore metabolic process | 1 | 1 | 0.04 | 0.03807 |
| GO:0009398 | FMN biosynthetic process | 1 | 1 | 0.04 | 0.03807 |
| GO:0009956 | radial pattern formation | 1 | 1 | 0.04 | 0.03807 |
| GO:0014861 | regulation of skeletal muscle contractio... | 1 | 1 | 0.04 | 0.03807 |
| GO:0019056 | modulation by virus of host transcriptio... | 1 | 1 | 0.04 | 0.03807 |
| GO:0019290 | siderophore biosynthetic process | 1 | 1 | 0.04 | 0.03807 |
| GO:0019464 | glycine decarboxylation via glycine clea... | 1 | 1 | 0.04 | 0.03807 |
| GO:0021691 | cerebellar Purkinje cell layer maturatio... | 1 | 1 | 0.04 | 0.03807 |
| GO:0031583 | phospholipase D-activating G-protein cou... | 1 | 1 | 0.04 | 0.03807 |
| GO:0031658 | negative regulation of cyclin-dependent ... | 1 | 1 | 0.04 | 0.03807 |
| GO:0031660 | regulation of cyclin-dependent protein s... | 1 | 1 | 0.04 | 0.03807 |
| GO:0031662 | positive regulation of cyclin-dependent ... | 1 | 1 | 0.04 | 0.03807 |
| GO:0031860 | telomeric 3' overhang formation | 1 | 1 | 0.04 | 0.03807 |
| GO:0032307 | negative regulation of prostaglandin sec... | 1 | 1 | 0.04 | 0.03807 |
| GO:0033552 | response to vitamin B3 | 1 | 1 | 0.04 | 0.03807 |
| GO:0033686 | positive regulation of luteinizing hormo... | 1 | 1 | 0.04 | 0.03807 |
| GO:0035262 | gonad morphogenesis | 1 | 1 | 0.04 | 0.03807 |
| GO:0035600 | tRNA methylthiolation | 1 | 1 | 0.04 | 0.03807 |
| GO:0035659 | Wnt signaling pathway involved in wound . | 1 | 1 | 0.04 | 0.03807 |
| GO:0035684 | helper T cell extravasation | 1 | 1 | 0.04 | 0.03807 |
| GO:0038167 | epidermal growth factor receptor signali... | 1 | 1 | 0.04 | 0.03807 |
| GO:0038168 | epidermal growth factor receptor signali... | 1 | 1 | 0.04 | 0.03807 |
| GO:0042313 | protein kinase C deactivation | 1 | 1 | 0.04 | 0.03807 |
| GO:0042431 | indole metabolic process | 1 | 1 | 0.04 | 0.03807 |
| GO:0042512 | negative regulation of tyrosine phosphor... | 1 | 1 | 0.04 | 0.03807 |
| GO:0042527 | negative regulation of tyrosine phosphor... | 1 | 1 | 0.04 | 0.03807 |
| GO:0043696 | dedifferentiation | 1 | 1 | 0.04 | 0.03807 |
| GO:0043697 | cell dedifferentiation | 1 | 1 | 0.04 | 0.03807 |
| GO:0045041 | protein import into mitochondrial interm... | 1 | 1 | 0.04 | 0.03807 |
| GO:0046444 | FMN metabolic process | 1 | 1 | 0.04 | 0.03807 |
| GO:0046587 | positive regulation of calcium-dependent... | 1 | 1 | 0.04 | 0.03807 |
| GO:0048687 | positive regulation of sprouting of inju... | 1 | 1 | 0.04 | 0.03807 |
| GO:0048691 | positive regulation of axon extension in... | 1 | 1 | 0.04 | 0.03807 |

| | | | | | |
|---|---|---|---|---|---|
| GO:0051543 | regulation of elastin biosynthetic proce... | 1 | 1 | 0.04 | 0.03807 |
| GO:0051545 | negative regulation of elastin biosynthe... | 1 | 1 | 0.04 | 0.03807 |
| GO:0052026 | modulation by symbiont of host transcrip... | 1 | 1 | 0.04 | 0.03807 |
| GO:0061310 | canonical Wnt signaling pathway involved. | 1 | 1 | 0.04 | 0.03807 |
| GO:0061355 | Wnt protein secretion | 1 | 1 | 0.04 | 0.03807 |
| GO:0061356 | regulation of Wnt protein secretion | 1 | 1 | 0.04 | 0.03807 |
| GO:0061358 | negative regulation of Wnt protein secre... | 1 | 1 | 0.04 | 0.03807 |
| GO:0070194 | synaptonemal complex disassembly | 1 | 1 | 0.04 | 0.03807 |
| GO:0071042 | nuclear polyadenylation-dependent mRNA | 1 | 1 | 0.04 | 0.03807 |
| GO:0071047 | polyadenylation-dependent mRNA catabol | 1 | 1 | 0.04 | 0.03807 |
| GO:0071298 | cellular response to L-ascorbic acid | 1 | 1 | 0.04 | 0.03807 |
| GO:0071301 | cellular response to vitamin B1 | 1 | 1 | 0.04 | 0.03807 |
| GO:0071389 | cellular response to mineralocorticoid s... | 1 | 1 | 0.04 | 0.03807 |
| GO:0071403 | cellular response to high density lipopr... | 1 | 1 | 0.04 | 0.03807 |
| GO:0071494 | cellular response to UV-C | 1 | 1 | 0.04 | 0.03807 |
| GO:0072733 | response to staurosporine | 1 | 1 | 0.04 | 0.03807 |
| GO:0072734 | cellular response to staurosporine | 1 | 1 | 0.04 | 0.03807 |
| GO:0086024 | adrenergic receptor signaling pathway in... | 1 | 1 | 0.04 | 0.03807 |
| GO:0090126 | protein complex assembly involved in syn. | 1 | 1 | 0.04 | 0.03807 |
| GO:0090615 | mitochondrial mRNA processing | 1 | 1 | 0.04 | 0.03807 |
| GO:0090616 | mitochondrial mRNA 3'-end processing | 1 | 1 | 0.04 | 0.03807 |
| GO:0097112 | gamma-aminobutyric acid receptor cluster. | 1 | 1 | 0.04 | 0.03807 |
| GO:0097222 | mitochondrial mRNA polyadenylation | 1 | 1 | 0.04 | 0.03807 |
| GO:0097338 | response to clozapine | 1 | 1 | 0.04 | 0.03807 |
| GO:1900234 | regulation of Kit signaling pathway | 1 | 1 | 0.04 | 0.03807 |
| GO:1900235 | negative regulation of Kit signaling pat... | 1 | 1 | 0.04 | 0.03807 |
| GO:1901128 | gentamycin metabolic process | 1 | 1 | 0.04 | 0.03807 |
| GO:1901350 | cell-cell signaling involved in cell-cel... | 1 | 1 | 0.04 | 0.03807 |
| GO:1901684 | arsenate ion transmembrane transport | 1 | 1 | 0.04 | 0.03807 |
| GO:1902038 | positive regulation of hematopoietic ste... | 1 | 1 | 0.04 | 0.03807 |
| GO:1902074 | response to salt | 1 | 1 | 0.04 | 0.03807 |
| GO:1902204 | positive regulation of hepatocyte growth... | 1 | 1 | 0.04 | 0.03807 |
| GO:1902205 | regulation of interleukin-2-mediated sig... | 1 | 1 | 0.04 | 0.03807 |
| GO:1902206 | negative regulation of interleukin-2-med... | 1 | 1 | 0.04 | 0.03807 |
| GO:1902211 | regulation of prolactin signaling pathwa... | 1 | 1 | 0.04 | 0.03807 |
| GO:1902212 | negative regulation of prolactin signali... | 1 | 1 | 0.04 | 0.03807 |
| GO:1902214 | regulation of interleukin-4-mediated sig... | 1 | 1 | 0.04 | 0.03807 |
| GO:1902215 | negative regulation of interleukin-4-med... | 1 | 1 | 0.04 | 0.03807 |
| GO:1902226 | regulation of macrophage colony-stimulat.. | 1 | 1 | 0.04 | 0.03807 |
| GO:1902227 | negative regulation of macrophage colony. | 1 | 1 | 0.04 | 0.03807 |
| GO:1902232 | regulation of positive thymic T cell sel... | 1 | 1 | 0.04 | 0.03807 |
| GO:1902233 | negative regulation of positive thymic T... | 1 | 1 | 0.04 | 0.03807 |
| GO:1902570 | protein localization to nucleolus | 1 | 1 | 0.04 | 0.03807 |
| GO:1903798 | regulation of production of miRNAs invol... | 1 | 1 | 0.04 | 0.03807 |
| GO:1903799 | negative regulation of production of miR... | 1 | 1 | 0.04 | 0.03807 |
| GO:1903969 | regulation of response to macrophage col. | 1 | 1 | 0.04 | 0.03807 |
| GO:1903970 | negative regulation of response to macro.. | 1 | 1 | 0.04 | 0.03807 |
| GO:1903972 | regulation of cellular response to macro... | 1 | 1 | 0.04 | 0.03807 |
| GO:1903973 | negative regulation of cellular response... | 1 | 1 | 0.04 | 0.03807 |
| GO:1990145 | maintenance of translational fidelity | 1 | 1 | 0.04 | 0.03807 |
| GO:2000055 | positive regulation of Wnt signaling pat... | 1 | 1 | 0.04 | 0.03807 |
| GO:2000120 | positive regulation of sodium-dependent ... | 1 | 1 | 0.04 | 0.03807 |
| GO:2000148 | regulation of planar cell polarity pathw... | 1 | 1 | 0.04 | 0.03807 |

| | | | | | |
|---|---|---|---|---|---|
| GO:2000149 | negative regulation of planar cell polar... | 1 | 1 | 0.04 | 0.03807 |
| GO:2000150 | regulation of planar cell polarity pathw... | 1 | 1 | 0.04 | 0.03807 |
| GO:2000151 | negative regulation of planar cell polar... | 1 | 1 | 0.04 | 0.03807 |
| GO:2000159 | regulation of planar cell polarity pathw... | 1 | 1 | 0.04 | 0.03807 |
| GO:2000160 | negative regulation of planar cell polar... | 1 | 1 | 0.04 | 0.03807 |
| GO:2000161 | regulation of planar cell polarity pathw... | 1 | 1 | 0.04 | 0.03807 |
| GO:2000162 | negative regulation of planar cell polar... | 1 | 1 | 0.04 | 0.03807 |
| GO:2000163 | regulation of planar cell polarity pathw... | 1 | 1 | 0.04 | 0.03807 |
| GO:2000164 | negative regulation of planar cell polar... | 1 | 1 | 0.04 | 0.03807 |
| GO:2000165 | regulation of planar cell polarity pathw... | 1 | 1 | 0.04 | 0.03807 |
| GO:2000166 | negative regulation of planar cell polar... | 1 | 1 | 0.04 | 0.03807 |
| GO:2000168 | negative regulation of planar cell polar... | 1 | 1 | 0.04 | 0.03807 |
| GO:2000185 | regulation of phosphate transmembrane tr. | 1 | 1 | 0.04 | 0.03807 |
| GO:2000187 | positive regulation of phosphate transme... | 1 | 1 | 0.04 | 0.03807 |
| GO:2000485 | regulation of glutamine transport | 1 | 1 | 0.04 | 0.03807 |
| GO:2000486 | negative regulation of glutamine transpo... | 1 | 1 | 0.04 | 0.03807 |
| GO:2000625 | regulation of miRNA catabolic process | 1 | 1 | 0.04 | 0.03807 |
| GO:2000627 | positive regulation of miRNA catabolic p... | 1 | 1 | 0.04 | 0.03807 |
| GO:2000631 | regulation of pre-miRNA processing | 1 | 1 | 0.04 | 0.03807 |
| GO:2000632 | negative regulation of pre-miRNA process. | 1 | 1 | 0.04 | 0.03807 |
| GO:2000807 | regulation of synaptic vesicle clusterin... | 1 | 1 | 0.04 | 0.03807 |
| GO:2000808 | negative regulation of synaptic vesicle ... | 1 | 1 | 0.04 | 0.03807 |
| GO:0002253 | activation of immune response | 385 | 22 | 14.66 | 0.03828 |
| GO:0060606 | tube closure | 83 | 7 | 3.16 | 0.03854 |
| GO:0010460 | positive regulation of heart rate | 20 | 3 | 0.76 | 0.03863 |
| GO:0060716 | labyrinthine layer blood vessel developm... | 20 | 3 | 0.76 | 0.03863 |
| GO:0071709 | membrane assembly | 20 | 3 | 0.76 | 0.03863 |
| GO:1902532 | negative regulation of intracellular sig... | 364 | 21 | 13.86 | 0.03877 |
| GO:0000245 | spliceosomal complex assembly | 34 | 4 | 1.29 | 0.03905 |
| GO:0007131 | reciprocal meiotic recombination | 34 | 4 | 1.29 | 0.03905 |
| GO:0008038 | neuron recognition | 34 | 4 | 1.29 | 0.03905 |
| GO:0010823 | negative regulation of mitochondrion org... | 34 | 4 | 1.29 | 0.03905 |
| GO:0032570 | response to progesterone | 34 | 4 | 1.29 | 0.03905 |
| GO:0035825 | reciprocal DNA recombination | 34 | 4 | 1.29 | 0.03905 |
| GO:0031344 | regulation of cell projection organizati... | 365 | 21 | 13.89 | 0.03974 |
| GO:0044839 | cell cycle G2/M phase transition | 159 | 11 | 6.05 | 0.0407 |
| GO:0050810 | regulation of steroid biosynthetic proce... | 50 | 5 | 1.9 | 0.04076 |
| GO:0007215 | glutamate receptor signaling pathway | 67 | 6 | 2.55 | 0.04177 |
| GO:0018107 | peptidyl-threonine phosphorylation | 67 | 6 | 2.55 | 0.04177 |
| GO:0043410 | positive regulation of MAPK cascade | 389 | 22 | 14.81 | 0.04209 |
| GO:0045137 | development of primary sexual characteri.. | 200 | 13 | 7.61 | 0.04252 |
| GO:0006352 | DNA-templated transcription | initiation | 241 | 15 | 9.17 |
| GO:0021772 | olfactory bulb development | 35 | 4 | 1.33 | 0.04281 |
| GO:0070169 | positive regulation of biomineral tissue... | 35 | 4 | 1.33 | 0.04281 |
| GO:0098656 | anion transmembrane transport | 221 | 14 | 8.41 | 0.04345 |
| GO:0001993 | regulation of systemic arterial blood pr... | 9 | 2 | 0.34 | 0.0436 |
| GO:0006853 | carnitine shuttle | 9 | 2 | 0.34 | 0.0436 |
| GO:0015824 | proline transport | 9 | 2 | 0.34 | 0.0436 |
| GO:0019511 | peptidyl-proline hydroxylation | 9 | 2 | 0.34 | 0.0436 |
| GO:0032060 | bleb assembly | 9 | 2 | 0.34 | 0.0436 |
| GO:0060049 | regulation of protein glycosylation | 9 | 2 | 0.34 | 0.0436 |
| GO:0090151 | establishment of protein localization to... | 9 | 2 | 0.34 | 0.0436 |
| GO:0097105 | presynaptic membrane assembly | 9 | 2 | 0.34 | 0.0436 |

| | | | | | |
|---|---|---|---|---|---|
| GO:1901534 | positive regulation of hematopoietic pro... | 9 | 2 | 0.34 | 0.0436 |
| GO:0001783 | B cell apoptotic process | 21 | 3 | 0.8 | 0.04383 |
| GO:0007617 | mating behavior | 21 | 3 | 0.8 | 0.04383 |
| GO:0060602 | branch elongation of an epithelium | 21 | 3 | 0.8 | 0.04383 |
| GO:0002066 | columnar/cuboidal epithelial cell develo... | 51 | 5 | 1.94 | 0.04383 |
| GO:0031667 | response to nutrient levels | 391 | 22 | 14.88 | 0.0441 |
| GO:0014066 | regulation of phosphatidylinositol 3-kin... | 68 | 6 | 2.59 | 0.04441 |
| GO:0009953 | dorsal/ventral pattern formation | 104 | 8 | 3.96 | 0.04473 |
| GO:1903052 | positive regulation of proteolysis invol... | 142 | 10 | 5.41 | 0.04481 |
| GO:0007140 | male meiosis | 36 | 4 | 1.37 | 0.04677 |
| GO:0034080 | CENP-A containing nucleosome assembly | 36 | 4 | 1.37 | 0.04677 |
| GO:0061641 | CENP-A containing chromatin organizatior | 36 | 4 | 1.37 | 0.04677 |
| GO:0032526 | response to retinoic acid | 105 | 8 | 4 | 0.04688 |
| GO:0006383 | transcription from RNA polymerase III pr... | 52 | 5 | 1.98 | 0.04704 |
| GO:0014032 | neural crest cell development | 52 | 5 | 1.98 | 0.04704 |
| GO:0007127 | meiosis I | 69 | 6 | 2.63 | 0.04714 |
| GO:0030512 | negative regulation of transforming grow... | 69 | 6 | 2.63 | 0.04714 |
| GO:1903845 | negative regulation of cellular response... | 69 | 6 | 2.63 | 0.04714 |
| GO:0098661 | inorganic anion transmembrane transport | 124 | 9 | 4.72 | 0.04716 |
| GO:0001936 | regulation of endothelial cell prolifera... | 87 | 7 | 3.31 | 0.04776 |
| GO:0006090 | pyruvate metabolic process | 87 | 7 | 3.31 | 0.04776 |
| GO:0007416 | synapse assembly | 87 | 7 | 3.31 | 0.04776 |
| GO:0021987 | cerebral cortex development | 87 | 7 | 3.31 | 0.04776 |
| GO:0003309 | type B pancreatic cell differentiation | 22 | 3 | 0.84 | 0.04936 |
| GO:0046685 | response to arsenic-containing substance | 22 | 3 | 0.84 | 0.04936 |
| GO:0048640 | negative regulation of developmental gro... | 70 | 6 | 2.66 | 0.04998 |
| GO:1903046 | meiotic cell cycle process | 88 | 7 | 3.35 | 0.05026 |
| GO:0030071 | regulation of mitotic metaphase/anaphase. | 53 | 5 | 2.02 | 0.05039 |
| GO:0043618 | regulation of transcription from RNA pol... | 53 | 5 | 2.02 | 0.05039 |
| GO:0021988 | olfactory lobe development | 37 | 4 | 1.41 | 0.05092 |
| GO:0051963 | regulation of synapse assembly | 37 | 4 | 1.41 | 0.05092 |
| GO:1901655 | cellular response to ketone | 37 | 4 | 1.41 | 0.05092 |
| GO:0030326 | embryonic limb morphogenesis | 126 | 9 | 4.8 | 0.05128 |
| GO:0035113 | embryonic appendage morphogenesis | 126 | 9 | 4.8 | 0.05128 |
| GO:0050852 | T cell receptor signaling pathway | 107 | 8 | 4.07 | 0.05139 |
| GO:1903364 | positive regulation of cellular protein ... | 146 | 10 | 5.56 | 0.05234 |
| GO:0000079 | regulation of cyclin-dependent protein s... | 89 | 7 | 3.39 | 0.05285 |
| GO:0006939 | smooth muscle contraction | 89 | 7 | 3.39 | 0.05285 |
| GO:0018210 | peptidyl-threonine modification | 71 | 6 | 2.7 | 0.05292 |
| GO:0010172 | embryonic body morphogenesis | 10 | 2 | 0.38 | 0.05316 |
| GO:0014874 | response to stimulus involved in regulat... | 10 | 2 | 0.38 | 0.05316 |
| GO:0021684 | cerebellar granular layer formation | 10 | 2 | 0.38 | 0.05316 |
| GO:0021707 | cerebellar granule cell differentiation | 10 | 2 | 0.38 | 0.05316 |
| GO:0032366 | intracellular sterol transport | 10 | 2 | 0.38 | 0.05316 |
| GO:0032367 | intracellular cholesterol transport | 10 | 2 | 0.38 | 0.05316 |
| GO:0051382 | kinetochore assembly | 10 | 2 | 0.38 | 0.05316 |
| GO:0051497 | negative regulation of stress fiber asse... | 10 | 2 | 0.38 | 0.05316 |
| GO:0060261 | positive regulation of transcription ini... | 10 | 2 | 0.38 | 0.05316 |
| GO:0072673 | lamellipodium morphogenesis | 10 | 2 | 0.38 | 0.05316 |
| GO:0090084 | negative regulation of inclusion body as... | 10 | 2 | 0.38 | 0.05316 |
| GO:0051403 | stress-activated MAPK cascade | 228 | 14 | 8.68 | 0.05378 |
| GO:1902099 | regulation of metaphase/anaphase transit. | 54 | 5 | 2.06 | 0.05387 |
| GO:0061136 | regulation of proteasomal protein catabo... | 187 | 12 | 7.12 | 0.05409 |

| | | | | | |
|---|---|---|---|---|---|
| GO:0003002 | regionalization | 335 | 19 | 12.75 | 0.05469 |
| GO:0042026 | protein refolding | 23 | 3 | 0.88 | 0.05522 |
| GO:0043949 | regulation of cAMP-mediated signaling | 23 | 3 | 0.88 | 0.05522 |
| GO:0046621 | negative regulation of organ growth | 23 | 3 | 0.88 | 0.05522 |
| GO:0031055 | chromatin remodeling at centromere | 38 | 4 | 1.45 | 0.05527 |
| GO:0035137 | hindlimb morphogenesis | 38 | 4 | 1.45 | 0.05527 |
| GO:0003158 | endothelium development | 90 | 7 | 3.43 | 0.05552 |
| GO:0046620 | regulation of organ growth | 72 | 6 | 2.74 | 0.05596 |
| GO:0000070 | mitotic sister chromatid segregation | 109 | 8 | 4.15 | 0.05617 |
| GO:0010810 | regulation of cell-substrate adhesion | 148 | 10 | 5.63 | 0.05641 |
| GO:0046661 | male sex differentiation | 148 | 10 | 5.63 | 0.05641 |
| GO:0022613 | ribonucleoprotein complex biogenesis | 272 | 16 | 10.35 | 0.05672 |
| GO:0031331 | positive regulation of cellular cataboli... | 251 | 15 | 9.55 | 0.05701 |
| GO:0007091 | metaphase/anaphase transition of mitotic.. | 55 | 5 | 2.09 | 0.05749 |
| GO:1903050 | regulation of proteolysis involved in ce... | 252 | 15 | 9.59 | 0.0586 |
| GO:0031098 | stress-activated protein kinase signalin... | 231 | 14 | 8.79 | 0.05869 |
| GO:0043255 | regulation of carbohydrate biosynthetic ... | 73 | 6 | 2.78 | 0.05911 |
| GO:0009620 | response to fungus | 39 | 4 | 1.48 | 0.05981 |
| GO:0046580 | negative regulation of Ras protein signa... | 39 | 4 | 1.48 | 0.05981 |
| GO:0010965 | regulation of mitotic sister chromatid s... | 56 | 5 | 2.13 | 0.06124 |
| GO:0044784 | metaphase/anaphase transition of cell cy... | 56 | 5 | 2.13 | 0.06124 |
| GO:0010039 | response to iron ion | 24 | 3 | 0.91 | 0.06138 |
| GO:0035883 | enteroendocrine cell differentiation | 24 | 3 | 0.91 | 0.06138 |
| GO:0050856 | regulation of T cell receptor signaling ... | 24 | 3 | 0.91 | 0.06138 |
| GO:0051412 | response to corticosterone | 24 | 3 | 0.91 | 0.06138 |
| GO:1903573 | negative regulation of response to endop.. | 24 | 3 | 0.91 | 0.06138 |
| GO:0018209 | peptidyl-serine modification | 212 | 13 | 8.07 | 0.06204 |
| GO:0060041 | retina development in camera-type eye | 131 | 9 | 4.99 | 0.06258 |
| GO:0007063 | regulation of sister chromatid cohesion | 11 | 2 | 0.42 | 0.06337 |
| GO:0015793 | glycerol transport | 11 | 2 | 0.42 | 0.06337 |
| GO:0019471 | 4-hydroxyproline metabolic process | 11 | 2 | 0.42 | 0.06337 |
| GO:0019755 | one-carbon compound transport | 11 | 2 | 0.42 | 0.06337 |
| GO:0021670 | lateral ventricle development | 11 | 2 | 0.42 | 0.06337 |
| GO:0032352 | positive regulation of hormone metabolic... | 11 | 2 | 0.42 | 0.06337 |
| GO:0034390 | smooth muscle cell apoptotic process | 11 | 2 | 0.42 | 0.06337 |
| GO:0034391 | regulation of smooth muscle cell apoptot... | 11 | 2 | 0.42 | 0.06337 |
| GO:0035313 | wound healing | spreading c | 11 | 2 | 0.42 |
| GO:0043518 | negative regulation of DNA damage respoı | 11 | 2 | 0.42 | 0.06337 |
| GO:0046628 | positive regulation of insulin receptor ... | 11 | 2 | 0.42 | 0.06337 |
| GO:0051123 | RNA polymerase II transcriptional preini... | 11 | 2 | 0.42 | 0.06337 |
| GO:0051895 | negative regulation of focal adhesion as... | 11 | 2 | 0.42 | 0.06337 |
| GO:0060736 | prostate gland growth | 11 | 2 | 0.42 | 0.06337 |
| GO:0070570 | regulation of neuron projection regenera... | 11 | 2 | 0.42 | 0.06337 |
| GO:0071786 | endoplasmic reticulum tubular network or.. | 11 | 2 | 0.42 | 0.06337 |
| GO:1903392 | negative regulation of adherens junction... | 11 | 2 | 0.42 | 0.06337 |
| GO:0036294 | cellular response to decreased oxygen le.. | 112 | 8 | 4.26 | 0.06385 |
| GO:0051058 | negative regulation of small GTPase medi. | 40 | 4 | 1.52 | 0.06455 |
| GO:0010975 | regulation of neuron projection developm.. | 277 | 16 | 10.54 | 0.06459 |
| GO:0000187 | activation of MAPK activity | 132 | 9 | 5.02 | 0.06502 |
| GO:0034341 | response to interferon-gamma | 132 | 9 | 5.02 | 0.06502 |
| GO:0033045 | regulation of sister chromatid segregati... | 57 | 5 | 2.17 | 0.06513 |
| GO:0033047 | regulation of mitotic sister chromatid s... | 57 | 5 | 2.17 | 0.06513 |
| GO:0043620 | regulation of DNA-templated transcriptio... | 57 | 5 | 2.17 | 0.06513 |

| | | | | | |
|---|---|---|---|---|---|
| GO:0051306 | mitotic sister chromatid separation | 57 | 5 | 2.17 | 0.06513 |
| GO:0031398 | positive regulation of protein ubiquitin... | 152 | 10 | 5.79 | 0.06514 |
| GO:0034767 | positive regulation of ion transmembrane... | 75 | 6 | 2.85 | 0.06571 |
| GO:0008637 | apoptotic mitochondrial changes | 113 | 8 | 4.3 | 0.06655 |
| GO:0050678 | regulation of epithelial cell proliferat... | 257 | 15 | 9.78 | 0.06702 |
| GO:0001516 | prostaglandin biosynthetic process | 25 | 3 | 0.95 | 0.06786 |
| GO:0009065 | glutamine family amino acid catabolic pr... | 25 | 3 | 0.95 | 0.06786 |
| GO:0044091 | membrane biogenesis | 25 | 3 | 0.95 | 0.06786 |
| GO:0046457 | prostanoid biosynthetic process | 25 | 3 | 0.95 | 0.06786 |
| GO:0006094 | gluconeogenesis | 76 | 6 | 2.89 | 0.06917 |
| GO:0044070 | regulation of anion transport | 114 | 8 | 4.34 | 0.06931 |
| GO:0000096 | sulfur amino acid metabolic process | 41 | 4 | 1.56 | 0.06947 |
| GO:0021587 | cerebellum morphogenesis | 41 | 4 | 1.56 | 0.06947 |
| GO:0038061 | NIK/NF-kappaB signaling | 41 | 4 | 1.56 | 0.06947 |
| GO:0043122 | regulation of I-kappaB kinase/NF-kappaB ... | 216 | 13 | 8.22 | 0.06969 |
| GO:2001237 | negative regulation of extrinsic apoptot... | 95 | 7 | 3.62 | 0.07011 |
| GO:0007249 | I-kappaB kinase/NF-kappaB signaling | 238 | 14 | 9.06 | 0.07131 |
| GO:0034166 | toll-like receptor 10 signaling pathway | 59 | 5 | 2.25 | 0.07331 |
| GO:0042733 | embryonic digit morphogenesis | 59 | 5 | 2.25 | 0.07331 |
| GO:0071300 | cellular response to retinoic acid | 59 | 5 | 2.25 | 0.07331 |
| GO:0007007 | inner mitochondrial membrane organizatio | 12 | 2 | 0.46 | 0.07418 |
| GO:0007064 | mitotic sister chromatid cohesion | 12 | 2 | 0.46 | 0.07418 |
| GO:0007076 | mitotic chromosome condensation | 12 | 2 | 0.46 | 0.07418 |
| GO:0009219 | pyrimidine deoxyribonucleotide metabolic... | 12 | 2 | 0.46 | 0.07418 |
| GO:0010870 | positive regulation of receptor biosynth... | 12 | 2 | 0.46 | 0.07418 |
| GO:0018126 | protein hydroxylation | 12 | 2 | 0.46 | 0.07418 |
| GO:0021683 | cerebellar granular layer morphogenesis | 12 | 2 | 0.46 | 0.07418 |
| GO:0032306 | regulation of prostaglandin secretion | 12 | 2 | 0.46 | 0.07418 |
| GO:0032310 | prostaglandin secretion | 12 | 2 | 0.46 | 0.07418 |
| GO:0033197 | response to vitamin E | 12 | 2 | 0.46 | 0.07418 |
| GO:0035964 | COPI-coated vesicle budding | 12 | 2 | 0.46 | 0.07418 |
| GO:0036066 | protein O-linked fucosylation | 12 | 2 | 0.46 | 0.07418 |
| GO:0048200 | Golgi transport vesicle coating | 12 | 2 | 0.46 | 0.07418 |
| GO:0048205 | COPI coating of Golgi vesicle | 12 | 2 | 0.46 | 0.07418 |
| GO:0051383 | kinetochore organization | 12 | 2 | 0.46 | 0.07418 |
| GO:0060134 | prepulse inhibition | 12 | 2 | 0.46 | 0.07418 |
| GO:0090026 | positive regulation of monocyte chemotax. | 12 | 2 | 0.46 | 0.07418 |
| GO:0097090 | presynaptic membrane organization | 12 | 2 | 0.46 | 0.07418 |
| GO:1901889 | negative regulation of cell junction ass... | 12 | 2 | 0.46 | 0.07418 |
| GO:1902603 | carnitine transmembrane transport | 12 | 2 | 0.46 | 0.07418 |
| GO:1903362 | regulation of cellular protein catabolic... | 261 | 15 | 9.94 | 0.0743 |
| GO:0014823 | response to activity | 42 | 4 | 1.6 | 0.07459 |
| GO:0086004 | regulation of cardiac muscle cell contra... | 42 | 4 | 1.6 | 0.07459 |
| GO:0006536 | glutamate metabolic process | 26 | 3 | 0.99 | 0.07463 |
| GO:0010259 | multicellular organismal aging | 26 | 3 | 0.99 | 0.07463 |
| GO:0010559 | regulation of glycoprotein biosynthetic ... | 26 | 3 | 0.99 | 0.07463 |
| GO:0042149 | cellular response to glucose starvation | 26 | 3 | 0.99 | 0.07463 |
| GO:0042755 | eating behavior | 26 | 3 | 0.99 | 0.07463 |
| GO:0043516 | regulation of DNA damage response | signa... | 26 | 3 | 0.99 |
| GO:0045838 | positive regulation of membrane potentia... | 26 | 3 | 0.99 | 0.07463 |
| GO:0060343 | trabecula formation | 26 | 3 | 0.99 | 0.07463 |
| GO:0060571 | morphogenesis of an epithelial fold | 26 | 3 | 0.99 | 0.07463 |
| GO:0000958 | mitochondrial mRNA catabolic process | 2 | 1 | 0.08 | 0.07469 |

| | | | | | |
|---|---|---|---|---|---|
| GO:0000962 | positive regulation of mitochondrial RNA... | 2 | 1 | 0.08 | 0.07469 |
| GO:0001808 | negative regulation of type IV hypersens... | 2 | 1 | 0.08 | 0.07469 |
| GO:0002434 | immune complex clearance | 2 | 1 | 0.08 | 0.07469 |
| GO:0002436 | immune complex clearance by monocytes | 2 | 1 | 0.08 | 0.07469 |
| GO:0002884 | negative regulation of hypersensitivity | 2 | 1 | 0.08 | 0.07469 |
| GO:0003050 | regulation of systemic arterial blood pr... | 2 | 1 | 0.08 | 0.07469 |
| GO:0003275 | apoptotic process involved in outflow tr... | 2 | 1 | 0.08 | 0.07469 |
| GO:0006233 | dTDP biosynthetic process | 2 | 1 | 0.08 | 0.07469 |
| GO:0006433 | prolyl-tRNA aminoacylation | 2 | 1 | 0.08 | 0.07469 |
| GO:0006540 | glutamate decarboxylation to succinate | 2 | 1 | 0.08 | 0.07469 |
| GO:0007066 | female meiosis sister chromatid cohesion | 2 | 1 | 0.08 | 0.07469 |
| GO:0007092 | activation of APC-Cdc20 complex activity | 2 | 1 | 0.08 | 0.07469 |
| GO:0010265 | SCF complex assembly | 2 | 1 | 0.08 | 0.07469 |
| GO:0010266 | response to vitamin B1 | 2 | 1 | 0.08 | 0.07469 |
| GO:0010835 | regulation of protein ADP-ribosylation | 2 | 1 | 0.08 | 0.07469 |
| GO:0010845 | positive regulation of reciprocal meioti... | 2 | 1 | 0.08 | 0.07469 |
| GO:0014004 | microglia differentiation | 2 | 1 | 0.08 | 0.07469 |
| GO:0014005 | microglia development | 2 | 1 | 0.08 | 0.07469 |
| GO:0015798 | myo-inositol transport | 2 | 1 | 0.08 | 0.07469 |
| GO:0016554 | cytidine to uridine editing | 2 | 1 | 0.08 | 0.07469 |
| GO:0021699 | cerebellar cortex maturation | 2 | 1 | 0.08 | 0.07469 |
| GO:0021874 | Wnt signaling pathway involved in forebr... | 2 | 1 | 0.08 | 0.07469 |
| GO:0031439 | positive regulation of mRNA cleavage | 2 | 1 | 0.08 | 0.07469 |
| GO:0031635 | adenylate cyclase-inhibiting opioid rece... | 2 | 1 | 0.08 | 0.07469 |
| GO:0032346 | positive regulation of aldosterone metab... | 2 | 1 | 0.08 | 0.07469 |
| GO:0032349 | positive regulation of aldosterone biosy... | 2 | 1 | 0.08 | 0.07469 |
| GO:0032377 | regulation of intracellular lipid transp... | 2 | 1 | 0.08 | 0.07469 |
| GO:0032380 | regulation of intracellular sterol trans... | 2 | 1 | 0.08 | 0.07469 |
| GO:0032383 | regulation of intracellular cholesterol ... | 2 | 1 | 0.08 | 0.07469 |
| GO:0032765 | positive regulation of mast cell cytokin... | 2 | 1 | 0.08 | 0.07469 |
| GO:0034091 | regulation of maintenance of sister chro... | 2 | 1 | 0.08 | 0.07469 |
| GO:0034093 | positive regulation of maintenance of si... | 2 | 1 | 0.08 | 0.07469 |
| GO:0034182 | regulation of maintenance of mitotic sis... | 2 | 1 | 0.08 | 0.07469 |
| GO:0034184 | positive regulation of maintenance of mi... | 2 | 1 | 0.08 | 0.07469 |
| GO:0034439 | lipoprotein lipid oxidation | 2 | 1 | 0.08 | 0.07469 |
| GO:0034696 | response to prostaglandin F | 2 | 1 | 0.08 | 0.07469 |
| GO:0034769 | basement membrane disassembly | 2 | 1 | 0.08 | 0.07469 |
| GO:0035261 | external genitalia morphogenesis | 2 | 1 | 0.08 | 0.07469 |
| GO:0035408 | histone H3-T6 phosphorylation | 2 | 1 | 0.08 | 0.07469 |
| GO:0035998 | | 7 8-dihydrone | 2 | 1 | 0.08 |
| GO:0036304 | umbilical cord morphogenesis | 2 | 1 | 0.08 | 0.07469 |
| GO:0042505 | tyrosine phosphorylation of Stat6 protei... | 2 | 1 | 0.08 | 0.07469 |
| GO:0042525 | regulation of tyrosine phosphorylation o... | 2 | 1 | 0.08 | 0.07469 |
| GO:0042727 | flavin-containing compound biosynthetic ... | 2 | 1 | 0.08 | 0.07469 |
| GO:0043179 | rhythmic excitation | 2 | 1 | 0.08 | 0.07469 |
| GO:0044090 | positive regulation of vacuole organizat... | 2 | 1 | 0.08 | 0.07469 |
| GO:0044335 | canonical Wnt signaling pathway involved. | 2 | 1 | 0.08 | 0.07469 |
| GO:0045876 | positive regulation of sister chromatid ... | 2 | 1 | 0.08 | 0.07469 |
| GO:0046072 | dTDP metabolic process | 2 | 1 | 0.08 | 0.07469 |
| GO:0046586 | regulation of calcium-dependent cell-cel... | 2 | 1 | 0.08 | 0.07469 |
| GO:0048560 | establishment of anatomical structure or... | 2 | 1 | 0.08 | 0.07469 |
| GO:0048686 | regulation of sprouting of injured axon | 2 | 1 | 0.08 | 0.07469 |
| GO:0048690 | regulation of axon extension involved in... | 2 | 1 | 0.08 | 0.07469 |

| | | | | | |
|---|---|---|---|---|---|
| GO:0050925 | negative regulation of negative chemotax.. | 2 | 1 | 0.08 | 0.07469 |
| GO:0051542 | elastin biosynthetic process | 2 | 1 | 0.08 | 0.07469 |
| GO:0060587 | regulation of lipoprotein lipid oxidatio... | 2 | 1 | 0.08 | 0.07469 |
| GO:0060598 | dichotomous subdivision of terminal unit... | 2 | 1 | 0.08 | 0.07469 |
| GO:0060838 | lymphatic endothelial cell fate commitme... | 2 | 1 | 0.08 | 0.07469 |
| GO:0060849 | regulation of transcription involved in ... | 2 | 1 | 0.08 | 0.07469 |
| GO:0061027 | umbilical cord development | 2 | 1 | 0.08 | 0.07469 |
| GO:0061051 | positive regulation of cell growth invol... | 2 | 1 | 0.08 | 0.07469 |
| GO:0061091 | regulation of phospholipid translocation | 2 | 1 | 0.08 | 0.07469 |
| GO:0061092 | positive regulation of phospholipid tran... | 2 | 1 | 0.08 | 0.07469 |
| GO:0061324 | canonical Wnt signaling pathway involved. | 2 | 1 | 0.08 | 0.07469 |
| GO:0061347 | planar cell polarity pathway involved in... | 2 | 1 | 0.08 | 0.07469 |
| GO:0061348 | planar cell polarity pathway involved in... | 2 | 1 | 0.08 | 0.07469 |
| GO:0061349 | planar cell polarity pathway involved in... | 2 | 1 | 0.08 | 0.07469 |
| GO:0061350 | planar cell polarity pathway involved in... | 2 | 1 | 0.08 | 0.07469 |
| GO:0061354 | planar cell polarity pathway involved in... | 2 | 1 | 0.08 | 0.07469 |
| GO:0061394 | regulation of transcription from RNA pol... | 2 | 1 | 0.08 | 0.07469 |
| GO:0070104 | negative regulation of interleukin-6-med... | 2 | 1 | 0.08 | 0.07469 |
| GO:0070920 | regulation of production of small RNA in... | 2 | 1 | 0.08 | 0.07469 |
| GO:0071492 | cellular response to UV-A | 2 | 1 | 0.08 | 0.07469 |
| GO:0071816 | tail-anchored membrane protein insertion.. | 2 | 1 | 0.08 | 0.07469 |
| GO:0090076 | relaxation of skeletal muscle | 2 | 1 | 0.08 | 0.07469 |
| GO:0090118 | receptor-mediated endocytosis of low-den. | 2 | 1 | 0.08 | 0.07469 |
| GO:0090264 | regulation of immune complex clearance b | 2 | 1 | 0.08 | 0.07469 |
| GO:0090265 | positive regulation of immune complex cl... | 2 | 1 | 0.08 | 0.07469 |
| GO:0097106 | postsynaptic density organization | 2 | 1 | 0.08 | 0.07469 |
| GO:0097107 | postsynaptic density assembly | 2 | 1 | 0.08 | 0.07469 |
| GO:0097117 | guanylate kinase-associated protein clus... | 2 | 1 | 0.08 | 0.07469 |
| GO:0097118 | neuroligin clustering | 2 | 1 | 0.08 | 0.07469 |
| GO:1901078 | negative regulation of relaxation of mus... | 2 | 1 | 0.08 | 0.07469 |
| GO:1901219 | regulation of cardiac chamber morphogene | 2 | 1 | 0.08 | 0.07469 |
| GO:1901220 | negative regulation of cardiac chamber m.. | 2 | 1 | 0.08 | 0.07469 |
| GO:1901491 | negative regulation of lymphangiogenesis | 2 | 1 | 0.08 | 0.07469 |
| GO:1901630 | negative regulation of presynaptic membr.. | 2 | 1 | 0.08 | 0.07469 |
| GO:1901898 | negative regulation of relaxation of car... | 2 | 1 | 0.08 | 0.07469 |
| GO:1901963 | regulation of cell proliferation involve... | 2 | 1 | 0.08 | 0.07469 |
| GO:1902093 | positive regulation of sperm motility | 2 | 1 | 0.08 | 0.07469 |
| GO:1902256 | regulation of apoptotic process involved... | 2 | 1 | 0.08 | 0.07469 |
| GO:1902257 | negative regulation of apoptotic process... | 2 | 1 | 0.08 | 0.07469 |
| GO:1902380 | positive regulation of endoribonuclease ... | 2 | 1 | 0.08 | 0.07469 |
| GO:1903797 | positive regulation of inorganic anion t... | 2 | 1 | 0.08 | 0.07469 |
| GO:2000167 | regulation of planar cell polarity pathw... | 2 | 1 | 0.08 | 0.07469 |
| GO:2000314 | negative regulation of fibroblast growth... | 2 | 1 | 0.08 | 0.07469 |
| GO:2000489 | regulation of hepatic stellate cell acti... | 2 | 1 | 0.08 | 0.07469 |
| GO:2000491 | positive regulation of hepatic stellate ... | 2 | 1 | 0.08 | 0.07469 |
| GO:2000502 | negative regulation of natural killer ce... | 2 | 1 | 0.08 | 0.07469 |
| GO:2000586 | regulation of platelet-derived growth fa... | 2 | 1 | 0.08 | 0.07469 |
| GO:2000587 | negative regulation of platelet-derived ... | 2 | 1 | 0.08 | 0.07469 |
| GO:2000598 | regulation of cyclin catabolic process | 2 | 1 | 0.08 | 0.07469 |
| GO:2000599 | negative regulation of cyclin catabolic ... | 2 | 1 | 0.08 | 0.07469 |
| GO:2000663 | negative regulation of interleukin-5 sec... | 2 | 1 | 0.08 | 0.07469 |
| GO:2000666 | negative regulation of interleukin-13 se... | 2 | 1 | 0.08 | 0.07469 |
| GO:2000786 | positive regulation of autophagic vacuol... | 2 | 1 | 0.08 | 0.07469 |

| | | | | | |
|---|---|---|---|---|---|
| GO:2000813 | negative regulation of barbed-end actin ... | 2 | 1 | 0.08 | 0.07469 |
| GO:2000820 | negative regulation of transcription fro... | 2 | 1 | 0.08 | 0.07469 |
| GO:2000855 | regulation of mineralocorticoid secretio... | 2 | 1 | 0.08 | 0.07469 |
| GO:2000857 | positive regulation of mineralocorticoid... | 2 | 1 | 0.08 | 0.07469 |
| GO:2000858 | regulation of aldosterone secretion | 2 | 1 | 0.08 | 0.07469 |
| GO:2000860 | positive regulation of aldosterone secre... | 2 | 1 | 0.08 | 0.07469 |
| GO:2001038 | regulation of cellular response to drug | 2 | 1 | 0.08 | 0.07469 |
| GO:0007219 | Notch signaling pathway | 156 | 10 | 5.94 | 0.07469 |
| GO:0007254 | JNK cascade | 177 | 11 | 6.74 | 0.07573 |
| GO:0034146 | toll-like receptor 5 signaling pathway | 60 | 5 | 2.28 | 0.07761 |
| GO:0090257 | regulation of muscle system process | 178 | 11 | 6.78 | 0.0781 |
| GO:0048511 | rhythmic process | 285 | 16 | 10.85 | 0.07866 |
| GO:0007584 | response to nutrient | 158 | 10 | 6.01 | 0.07978 |
| GO:0090288 | negative regulation of cellular response... | 98 | 7 | 3.73 | 0.07987 |
| GO:0001885 | endothelial cell development | 43 | 4 | 1.64 | 0.07988 |
| GO:0006284 | base-excision repair | 43 | 4 | 1.64 | 0.07988 |
| GO:0008156 | negative regulation of DNA replication | 43 | 4 | 1.64 | 0.07988 |
| GO:0086003 | cardiac muscle cell contraction | 43 | 4 | 1.64 | 0.07988 |
| GO:0090199 | regulation of release of cytochrome c fr... | 43 | 4 | 1.64 | 0.07988 |
| GO:0050851 | antigen receptor-mediated signaling path.. | 138 | 9 | 5.25 | 0.08088 |
| GO:0006884 | cell volume homeostasis | 27 | 3 | 1.03 | 0.08168 |
| GO:0045823 | positive regulation of heart contraction | 27 | 3 | 1.03 | 0.08168 |
| GO:1902117 | positive regulation of organelle assembl... | 27 | 3 | 1.03 | 0.08168 |
| GO:0071396 | cellular response to lipid | 331 | 18 | 12.6 | 0.08238 |
| GO:0090305 | nucleic acid phosphodiester bond hydroly.. | 201 | 12 | 7.65 | 0.08289 |
| GO:2001233 | regulation of apoptotic signaling pathwa... | 354 | 19 | 13.48 | 0.08374 |
| GO:0019319 | hexose biosynthetic process | 80 | 6 | 3.05 | 0.08403 |
| GO:0034138 | toll-like receptor 3 signaling pathway | 80 | 6 | 3.05 | 0.08403 |
| GO:0034764 | positive regulation of transmembrane tra... | 80 | 6 | 3.05 | 0.08403 |
| GO:0001838 | embryonic epithelial tube formation | 119 | 8 | 4.53 | 0.0842 |
| GO:0007059 | chromosome segregation | 223 | 13 | 8.49 | 0.08453 |
| GO:0018105 | peptidyl-serine phosphorylation | 202 | 12 | 7.69 | 0.08526 |
| GO:0006622 | protein targeting to lysosome | 13 | 2 | 0.49 | 0.08552 |
| GO:0009992 | cellular water homeostasis | 13 | 2 | 0.49 | 0.08552 |
| GO:0015693 | magnesium ion transport | 13 | 2 | 0.49 | 0.08552 |
| GO:0015838 | amino-acid betaine transport | 13 | 2 | 0.49 | 0.08552 |
| GO:0015879 | carnitine transport | 13 | 2 | 0.49 | 0.08552 |
| GO:0019371 | cyclooxygenase pathway | 13 | 2 | 0.49 | 0.08552 |
| GO:0031645 | negative regulation of neurological syst... | 13 | 2 | 0.49 | 0.08552 |
| GO:0032232 | negative regulation of actin filament bu... | 13 | 2 | 0.49 | 0.08552 |
| GO:0034349 | glial cell apoptotic process | 13 | 2 | 0.49 | 0.08552 |
| GO:0035082 | axoneme assembly | 13 | 2 | 0.49 | 0.08552 |
| GO:0035855 | megakaryocyte development | 13 | 2 | 0.49 | 0.08552 |
| GO:0043950 | positive regulation of cAMP-mediated sig.. | 13 | 2 | 0.49 | 0.08552 |
| GO:0044321 | response to leptin | 13 | 2 | 0.49 | 0.08552 |
| GO:0051984 | positive regulation of chromosome segreg. | 13 | 2 | 0.49 | 0.08552 |
| GO:0070897 | DNA-templated transcriptional preinitiat... | 13 | 2 | 0.49 | 0.08552 |
| GO:0044262 | cellular carbohydrate metabolic process | 267 | 15 | 10.16 | 0.08618 |
| GO:0032435 | negative regulation of proteasomal ubiqu.. | 62 | 5 | 2.36 | 0.08659 |
| GO:0014031 | mesenchymal cell development | 140 | 9 | 5.33 | 0.08665 |
| GO:0072175 | epithelial tube formation | 120 | 8 | 4.57 | 0.08738 |
| GO:0031669 | cellular response to nutrient levels | 182 | 11 | 6.93 | 0.08806 |
| GO:0001958 | endochondral ossification | 28 | 3 | 1.07 | 0.08901 |

| | | | | | |
|---|---|---|---|---|---|
| GO:0033574 | response to testosterone | 28 | 3 | 1.07 | 0.08901 |
| GO:0036075 | replacement ossification | 28 | 3 | 1.07 | 0.08901 |
| GO:0044705 | multi-organism reproductive behavior | 28 | 3 | 1.07 | 0.08901 |
| GO:0044743 | intracellular protein transmembrane impo.. | 28 | 3 | 1.07 | 0.08901 |
| GO:0046939 | nucleotide phosphorylation | 28 | 3 | 1.07 | 0.08901 |
| GO:0050922 | negative regulation of chemotaxis | 28 | 3 | 1.07 | 0.08901 |
| GO:0071391 | cellular response to estrogen stimulus | 28 | 3 | 1.07 | 0.08901 |
| GO:0050679 | positive regulation of epithelial cell p... | 141 | 9 | 5.37 | 0.08962 |
| GO:0044344 | cellular response to fibroblast growth f... | 204 | 12 | 7.77 | 0.09011 |
| GO:0008584 | male gonad development | 121 | 8 | 4.61 | 0.09064 |
| GO:0046546 | development of primary male sexual chara | 121 | 8 | 4.61 | 0.09064 |
| GO:0071453 | cellular response to oxygen levels | 121 | 8 | 4.61 | 0.09064 |
| GO:0002067 | glandular epithelial cell differentiatio... | 45 | 4 | 1.71 | 0.09102 |
| GO:0032006 | regulation of TOR signaling | 45 | 4 | 1.71 | 0.09102 |
| GO:0043486 | histone exchange | 45 | 4 | 1.71 | 0.09102 |
| GO:0046847 | filopodium assembly | 45 | 4 | 1.71 | 0.09102 |
| GO:0071479 | cellular response to ionizing radiation | 45 | 4 | 1.71 | 0.09102 |
| GO:1903115 | regulation of actin filament-based movem.. | 45 | 4 | 1.71 | 0.09102 |
| GO:0030500 | regulation of bone mineralization | 63 | 5 | 2.4 | 0.09127 |
| GO:0060415 | muscle tissue morphogenesis | 63 | 5 | 2.4 | 0.09127 |
| GO:0007173 | epidermal growth factor receptor signali... | 226 | 13 | 8.6 | 0.09145 |
| GO:0009451 | RNA modification | 82 | 6 | 3.12 | 0.09208 |
| GO:0051961 | negative regulation of nervous system de.. | 205 | 12 | 7.8 | 0.09261 |
| GO:0008543 | fibroblast growth factor receptor signal... | 184 | 11 | 7 | 0.09332 |
| GO:0048736 | appendage development | 163 | 10 | 6.2 | 0.09342 |
| GO:0060173 | limb development | 163 | 10 | 6.2 | 0.09342 |
| GO:0050770 | regulation of axonogenesis | 122 | 8 | 4.64 | 0.09396 |
| GO:0001935 | endothelial cell proliferation | 102 | 7 | 3.88 | 0.09404 |
| GO:0016197 | endosomal transport | 206 | 12 | 7.84 | 0.09514 |
| GO:0097306 | cellular response to alcohol | 83 | 6 | 3.16 | 0.09625 |
| GO:0042044 | fluid transport | 46 | 4 | 1.75 | 0.09685 |
| GO:0002438 | acute inflammatory response to antigenic.. | 14 | 2 | 0.53 | 0.09734 |
| GO:0010971 | positive regulation of G2/M transition o... | 14 | 2 | 0.53 | 0.09734 |
| GO:0014002 | astrocyte development | 14 | 2 | 0.53 | 0.09734 |
| GO:0015732 | prostaglandin transport | 14 | 2 | 0.53 | 0.09734 |
| GO:0021542 | dentate gyrus development | 14 | 2 | 0.53 | 0.09734 |
| GO:0030033 | microvillus assembly | 14 | 2 | 0.53 | 0.09734 |
| GO:0032634 | interleukin-5 production | 14 | 2 | 0.53 | 0.09734 |
| GO:0032674 | regulation of interleukin-5 production | 14 | 2 | 0.53 | 0.09734 |
| GO:0032793 | positive regulation of CREB transcriptio... | 14 | 2 | 0.53 | 0.09734 |
| GO:0034389 | lipid particle organization | 14 | 2 | 0.53 | 0.09734 |
| GO:0035058 | nonmotile primary cilium assembly | 14 | 2 | 0.53 | 0.09734 |
| GO:0043101 | purine-containing compound salvage | 14 | 2 | 0.53 | 0.09734 |
| GO:0043174 | nucleoside salvage | 14 | 2 | 0.53 | 0.09734 |
| GO:0044068 | modulation by symbiont of host cellular ... | 14 | 2 | 0.53 | 0.09734 |
| GO:0045821 | positive regulation of glycolytic proces... | 14 | 2 | 0.53 | 0.09734 |
| GO:0045986 | negative regulation of smooth muscle con. | 14 | 2 | 0.53 | 0.09734 |
| GO:0051016 | barbed-end actin filament capping | 14 | 2 | 0.53 | 0.09734 |
| GO:0061462 | protein localization to lysosome | 14 | 2 | 0.53 | 0.09734 |
| GO:0071480 | cellular response to gamma radiation | 14 | 2 | 0.53 | 0.09734 |
| GO:0072698 | protein localization to microtubule cyto... | 14 | 2 | 0.53 | 0.09734 |
| GO:0097066 | response to thyroid hormone | 14 | 2 | 0.53 | 0.09734 |
| GO:1902751 | positive regulation of cell cycle G2/M p... | 14 | 2 | 0.53 | 0.09734 |

| | | | | | |
|---|---|---|---|---|---|
| GO:0071774 | response to fibroblast growth factor | 207 | 12 | 7.88 | 0.09771 |
| GO:0031345 | negative regulation of cell projection o... | 103 | 7 | 3.92 | 0.09779 |
| GO:0034660 | ncRNA metabolic process | 362 | 19 | 13.78 | 0.09854 |
| GO:0042451 | purine nucleoside biosynthetic process | 84 | 6 | 3.2 | 0.10052 |
| GO:0046129 | purine ribonucleoside biosynthetic proce... | 84 | 6 | 3.2 | 0.10052 |
| GO:0044724 | single-organism carbohydrate catabolic p.. | 124 | 8 | 4.72 | 0.10081 |
| GO:0001824 | blastocyst development | 65 | 5 | 2.47 | 0.10103 |
| GO:0034446 | substrate adhesion-dependent cell spread. | 65 | 5 | 2.47 | 0.10103 |
| GO:0038123 | toll-like receptor TLR1:TLR2 signaling p... | 65 | 5 | 2.47 | 0.10103 |
| GO:0038124 | toll-like receptor TLR6:TLR2 signaling p... | 65 | 5 | 2.47 | 0.10103 |
| GO:0043500 | muscle adaptation | 65 | 5 | 2.47 | 0.10103 |
| GO:0007346 | regulation of mitotic cell cycle | 386 | 20 | 14.69 | 0.10117 |
| GO:0030048 | actin filament-based movement | 104 | 7 | 3.96 | 0.10161 |
| GO:0006937 | regulation of muscle contraction | 145 | 9 | 5.52 | 0.10211 |
| GO:0016331 | morphogenesis of embryonic epithelium | 145 | 9 | 5.52 | 0.10211 |
| GO:0006336 | DNA replication-independent nucleosome | 47 | 4 | 1.79 | 0.10285 |
| GO:0034724 | DNA replication-independent nucleosome | 47 | 4 | 1.79 | 0.10285 |
| GO:0060711 | labyrinthine layer development | 47 | 4 | 1.79 | 0.10285 |
| GO:0044057 | regulation of system process | 387 | 20 | 14.73 | 0.1031 |
| GO:0035265 | organ growth | 125 | 8 | 4.76 | 0.10434 |
| GO:0019098 | reproductive behavior | 30 | 3 | 1.14 | 0.10446 |
| GO:0032094 | response to food | 30 | 3 | 1.14 | 0.10446 |
| GO:0035329 | hippo signaling | 30 | 3 | 1.14 | 0.10446 |
| GO:0042304 | regulation of fatty acid biosynthetic pr... | 30 | 3 | 1.14 | 0.10446 |
| GO:0046326 | positive regulation of glucose import | 30 | 3 | 1.14 | 0.10446 |
| GO:0051489 | regulation of filopodium assembly | 30 | 3 | 1.14 | 0.10446 |
| GO:0071312 | cellular response to alkaloid | 30 | 3 | 1.14 | 0.10446 |
| GO:0090342 | regulation of cell aging | 30 | 3 | 1.14 | 0.10446 |
| GO:0006942 | regulation of striated muscle contractio... | 85 | 6 | 3.24 | 0.10489 |
| GO:0009743 | response to carbohydrate | 167 | 10 | 6.36 | 0.10527 |
| GO:0035107 | appendage morphogenesis | 146 | 9 | 5.56 | 0.10538 |
| GO:0035108 | limb morphogenesis | 146 | 9 | 5.56 | 0.10538 |
| GO:0045931 | positive regulation of mitotic cell cycl... | 105 | 7 | 4 | 0.10552 |
| GO:1901799 | negative regulation of proteasomal prote... | 66 | 5 | 2.51 | 0.10609 |
| GO:0038127 | ERBB signaling pathway | 232 | 13 | 8.83 | 0.10632 |
| GO:1901991 | negative regulation of mitotic cell cycl... | 147 | 9 | 5.6 | 0.1087 |
| GO:0003044 | regulation of systemic arterial blood pr... | 48 | 4 | 1.83 | 0.10902 |
| GO:0048844 | artery morphogenesis | 48 | 4 | 1.83 | 0.10902 |
| GO:0050771 | negative regulation of axonogenesis | 48 | 4 | 1.83 | 0.10902 |
| GO:0070098 | chemokine-mediated signaling pathway | 48 | 4 | 1.83 | 0.10902 |
| GO:0010827 | regulation of glucose transport | 86 | 6 | 3.27 | 0.10936 |
| GO:0032368 | regulation of lipid transport | 86 | 6 | 3.27 | 0.10936 |
| GO:0046364 | monosaccharide biosynthetic process | 86 | 6 | 3.27 | 0.10936 |
| GO:0007567 | parturition | 15 | 2 | 0.57 | 0.10958 |
| GO:0010613 | positive regulation of cardiac muscle hy... | 15 | 2 | 0.57 | 0.10958 |
| GO:0010996 | response to auditory stimulus | 15 | 2 | 0.57 | 0.10958 |
| GO:0014742 | positive regulation of muscle hypertroph... | 15 | 2 | 0.57 | 0.10958 |
| GO:0019184 | nonribosomal peptide biosynthetic proces. | 15 | 2 | 0.57 | 0.10958 |
| GO:0021681 | cerebellar granular layer development | 15 | 2 | 0.57 | 0.10958 |
| GO:0031365 | N-terminal protein amino acid modificati... | 15 | 2 | 0.57 | 0.10958 |
| GO:0043649 | dicarboxylic acid catabolic process | 15 | 2 | 0.57 | 0.10958 |
| GO:0048194 | Golgi vesicle budding | 15 | 2 | 0.57 | 0.10958 |
| GO:0051131 | chaperone-mediated protein complex asse | 15 | 2 | 0.57 | 0.10958 |

| | | | | | |
|---|---|---|---|---|---|
| GO:0060074 | synapse maturation | 15 | 2 | 0.57 | 0.10958 |
| GO:0060218 | hematopoietic stem cell differentiation | 15 | 2 | 0.57 | 0.10958 |
| GO:0060219 | camera-type eye photoreceptor cell diffe... | 15 | 2 | 0.57 | 0.10958 |
| GO:0060260 | regulation of transcription initiation f... | 15 | 2 | 0.57 | 0.10958 |
| GO:0060347 | heart trabecula formation | 15 | 2 | 0.57 | 0.10958 |
| GO:0060438 | trachea development | 15 | 2 | 0.57 | 0.10958 |
| GO:0090179 | planar cell polarity pathway involved in... | 15 | 2 | 0.57 | 0.10958 |
| GO:0090344 | negative regulation of cell aging | 15 | 2 | 0.57 | 0.10958 |
| GO:1900078 | positive regulation of cellular response... | 15 | 2 | 0.57 | 0.10958 |
| GO:2000104 | negative regulation of DNA-dependent DN | 15 | 2 | 0.57 | 0.10958 |
| GO:0000056 | ribosomal small subunit export from nucl... | 3 | 1 | 0.11 | 0.10991 |
| GO:0000320 | re-entry into mitotic cell cycle | 3 | 1 | 0.11 | 0.10991 |
| GO:0000957 | mitochondrial RNA catabolic process | 3 | 1 | 0.11 | 0.10991 |
| GO:0000960 | regulation of mitochondrial RNA cataboli... | 3 | 1 | 0.11 | 0.10991 |
| GO:0000965 | mitochondrial RNA 3'-end processing | 3 | 1 | 0.11 | 0.10991 |
| GO:0001807 | regulation of type IV hypersensitivity | 3 | 1 | 0.11 | 0.10991 |
| GO:0001923 | B-1 B cell differentiation | 3 | 1 | 0.11 | 0.10991 |
| GO:0001997 | positive regulation of the force of hear... | 3 | 1 | 0.11 | 0.10991 |
| GO:0002023 | reduction of food intake in response to ... | 3 | 1 | 0.11 | 0.10991 |
| GO:0002025 | vasodilation by norepinephrine-epinephri... | 3 | 1 | 0.11 | 0.10991 |
| GO:0002865 | negative regulation of acute inflammator... | 3 | 1 | 0.11 | 0.10991 |
| GO:0003011 | involuntary skeletal muscle contraction | 3 | 1 | 0.11 | 0.10991 |
| GO:0003213 | cardiac right atrium morphogenesis | 3 | 1 | 0.11 | 0.10991 |
| GO:0006114 | glycerol biosynthetic process | 3 | 1 | 0.11 | 0.10991 |
| GO:0006227 | dUDP biosynthetic process | 3 | 1 | 0.11 | 0.10991 |
| GO:0006391 | transcription initiation from mitochondr... | 3 | 1 | 0.11 | 0.10991 |
| GO:0006546 | glycine catabolic process | 3 | 1 | 0.11 | 0.10991 |
| GO:0006556 | S-adenosylmethionine biosynthetic proces | 3 | 1 | 0.11 | 0.10991 |
| GO:0009139 | pyrimidine nucleoside diphosphate biosyn. | 3 | 1 | 0.11 | 0.10991 |
| GO:0009196 | pyrimidine deoxyribonucleoside diphospha | 3 | 1 | 0.11 | 0.10991 |
| GO:0009197 | pyrimidine deoxyribonucleoside diphospha | 3 | 1 | 0.11 | 0.10991 |
| GO:0010286 | heat acclimation | 3 | 1 | 0.11 | 0.10991 |
| GO:0010520 | regulation of reciprocal meiotic recombi... | 3 | 1 | 0.11 | 0.10991 |
| GO:0015820 | leucine transport | 3 | 1 | 0.11 | 0.10991 |
| GO:0016480 | negative regulation of transcription fro... | 3 | 1 | 0.11 | 0.10991 |
| GO:0019065 | receptor-mediated endocytosis of virus b... | 3 | 1 | 0.11 | 0.10991 |
| GO:0019401 | alditol biosynthetic process | 3 | 1 | 0.11 | 0.10991 |
| GO:0021553 | olfactory nerve development | 3 | 1 | 0.11 | 0.10991 |
| GO:0021590 | cerebellum maturation | 3 | 1 | 0.11 | 0.10991 |
| GO:0030050 | vesicle transport along actin filament | 3 | 1 | 0.11 | 0.10991 |
| GO:0030185 | nitric oxide transport | 3 | 1 | 0.11 | 0.10991 |
| GO:0031437 | regulation of mRNA cleavage | 3 | 1 | 0.11 | 0.10991 |
| GO:0031627 | telomeric loop formation | 3 | 1 | 0.11 | 0.10991 |
| GO:0032286 | central nervous system myelin maintenanc | 3 | 1 | 0.11 | 0.10991 |
| GO:0032417 | positive regulation of sodium:proton ant... | 3 | 1 | 0.11 | 0.10991 |
| GO:0032641 | lymphotoxin A production | 3 | 1 | 0.11 | 0.10991 |
| GO:0033216 | ferric iron import | 3 | 1 | 0.11 | 0.10991 |
| GO:0033668 | negative regulation by symbiont of host ... | 3 | 1 | 0.11 | 0.10991 |
| GO:0033864 | positive regulation of NAD(P)H oxidase a.. | 3 | 1 | 0.11 | 0.10991 |
| GO:0034141 | positive regulation of toll-like recepto... | 3 | 1 | 0.11 | 0.10991 |
| GO:0035507 | regulation of myosin-light-chain-phospha... | 3 | 1 | 0.11 | 0.10991 |
| GO:0035582 | sequestering of BMP in extracellular mat... | 3 | 1 | 0.11 | 0.10991 |
| GO:0035694 | mitochondrial protein catabolic process | 3 | 1 | 0.11 | 0.10991 |

| | | | | | |
|---|---|---|---|---|---|
| GO:0035733 | hepatic stellate cell activation | 3 | 1 | 0.11 | 0.10991 |
| GO:0035927 | RNA import into mitochondrion | 3 | 1 | 0.11 | 0.10991 |
| GO:0035928 | rRNA import into mitochondrion | 3 | 1 | 0.11 | 0.10991 |
| GO:0036215 | response to stem cell factor | 3 | 1 | 0.11 | 0.10991 |
| GO:0036216 | cellular response to stem cell factor st... | 3 | 1 | 0.11 | 0.10991 |
| GO:0036438 | maintenance of lens transparency | 3 | 1 | 0.11 | 0.10991 |
| GO:0038109 | Kit signaling pathway | 3 | 1 | 0.11 | 0.10991 |
| GO:0038145 | macrophage colony-stimulating factor sig.. | 3 | 1 | 0.11 | 0.10991 |
| GO:0039656 | modulation by virus of host gene express.. | 3 | 1 | 0.11 | 0.10991 |
| GO:0042109 | lymphotoxin A biosynthetic process | 3 | 1 | 0.11 | 0.10991 |
| GO:0042524 | negative regulation of tyrosine phosphor... | 3 | 1 | 0.11 | 0.10991 |
| GO:0044778 | meiotic DNA integrity checkpoint | 3 | 1 | 0.11 | 0.10991 |
| GO:0044789 | modulation by host of viral release from... | 3 | 1 | 0.11 | 0.10991 |
| GO:0044791 | positive regulation by host of viral rel... | 3 | 1 | 0.11 | 0.10991 |
| GO:0045048 | protein insertion into ER membrane | 3 | 1 | 0.11 | 0.10991 |
| GO:0045541 | negative regulation of cholesterol biosy... | 3 | 1 | 0.11 | 0.10991 |
| GO:0045899 | positive regulation of RNA polymerase II... | 3 | 1 | 0.11 | 0.10991 |
| GO:0045906 | negative regulation of vasoconstriction | 3 | 1 | 0.11 | 0.10991 |
| GO:0045925 | positive regulation of female receptivit... | 3 | 1 | 0.11 | 0.10991 |
| GO:0046077 | dUDP metabolic process | 3 | 1 | 0.11 | 0.10991 |
| GO:0048677 | axon extension involved in regeneration | 3 | 1 | 0.11 | 0.10991 |
| GO:0048682 | sprouting of injured axon | 3 | 1 | 0.11 | 0.10991 |
| GO:0050893 | sensory processing | 3 | 1 | 0.11 | 0.10991 |
| GO:0051066 | dihydrobiopterin metabolic process | 3 | 1 | 0.11 | 0.10991 |
| GO:0051490 | negative regulation of filopodium assemb.. | 3 | 1 | 0.11 | 0.10991 |
| GO:0051771 | negative regulation of nitric-oxide synt... | 3 | 1 | 0.11 | 0.10991 |
| GO:0052041 | negative regulation by symbiont of host ... | 3 | 1 | 0.11 | 0.10991 |
| GO:0052490 | negative regulation by organism of progr... | 3 | 1 | 0.11 | 0.10991 |
| GO:0055099 | response to high density lipoprotein par... | 3 | 1 | 0.11 | 0.10991 |
| GO:0060066 | oviduct development | 3 | 1 | 0.11 | 0.10991 |
| GO:0060161 | positive regulation of dopamine receptor... | 3 | 1 | 0.11 | 0.10991 |
| GO:0060414 | aorta smooth muscle tissue morphogenesi | 3 | 1 | 0.11 | 0.10991 |
| GO:0060584 | regulation of prostaglandin-endoperoxide.. | 3 | 1 | 0.11 | 0.10991 |
| GO:0060585 | positive regulation of prostaglandin-end... | 3 | 1 | 0.11 | 0.10991 |
| GO:0060825 | fibroblast growth factor receptor signal... | 3 | 1 | 0.11 | 0.10991 |
| GO:0061002 | negative regulation of dendritic spine m... | 3 | 1 | 0.11 | 0.10991 |
| GO:0061152 | trachea submucosa development | 3 | 1 | 0.11 | 0.10991 |
| GO:0061153 | trachea gland development | 3 | 1 | 0.11 | 0.10991 |
| GO:0061341 | non-canonical Wnt signaling pathway invo. | 3 | 1 | 0.11 | 0.10991 |
| GO:0061346 | planar cell polarity pathway involved in... | 3 | 1 | 0.11 | 0.10991 |
| GO:0061364 | apoptotic process involved in luteolysis | 3 | 1 | 0.11 | 0.10991 |
| GO:0070093 | negative regulation of glucagon secretio... | 3 | 1 | 0.11 | 0.10991 |
| GO:0070101 | positive regulation of chemokine-mediate.. | 3 | 1 | 0.11 | 0.10991 |
| GO:0070103 | regulation of interleukin-6-mediated sig... | 3 | 1 | 0.11 | 0.10991 |
| GO:0070370 | cellular heat acclimation | 3 | 1 | 0.11 | 0.10991 |
| GO:0070537 | histone H2A K63-linked deubiquitination | 3 | 1 | 0.11 | 0.10991 |
| GO:0070858 | negative regulation of bile acid biosynt... | 3 | 1 | 0.11 | 0.10991 |
| GO:0071000 | response to magnetism | 3 | 1 | 0.11 | 0.10991 |
| GO:0071315 | cellular response to morphine | 3 | 1 | 0.11 | 0.10991 |
| GO:0071394 | cellular response to testosterone stimul... | 3 | 1 | 0.11 | 0.10991 |
| GO:0075509 | endocytosis involved in viral entry into... | 3 | 1 | 0.11 | 0.10991 |
| GO:0089700 | protein kinase D signaling | 3 | 1 | 0.11 | 0.10991 |
| GO:0090069 | regulation of ribosome biogenesis | 3 | 1 | 0.11 | 0.10991 |

| | | | | | |
|---|---|---|---|---|---|
| GO:0090071 | negative regulation of ribosome biogenes.. | 3 | 1 | 0.11 | 0.10991 |
| GO:0090206 | negative regulation of cholesterol metab... | 3 | 1 | 0.11 | 0.10991 |
| GO:0090219 | negative regulation of lipid kinase acti... | 3 | 1 | 0.11 | 0.10991 |
| GO:0097116 | gephyrin clustering | 3 | 1 | 0.11 | 0.10991 |
| GO:0097187 | dentinogenesis | 3 | 1 | 0.11 | 0.10991 |
| GO:0097252 | oligodendrocyte apoptotic process | 3 | 1 | 0.11 | 0.10991 |
| GO:0097461 | ferric iron import into cell | 3 | 1 | 0.11 | 0.10991 |
| GO:1900135 | positive regulation of renin secretion i... | 3 | 1 | 0.11 | 0.10991 |
| GO:1901317 | regulation of sperm motility | 3 | 1 | 0.11 | 0.10991 |
| GO:1901624 | negative regulation of lymphocyte chemot. | 3 | 1 | 0.11 | 0.10991 |
| GO:1901727 | positive regulation of histone deacetyla... | 3 | 1 | 0.11 | 0.10991 |
| GO:1902036 | regulation of hematopoietic stem cell di... | 3 | 1 | 0.11 | 0.10991 |
| GO:1903795 | regulation of inorganic anion transmembr.. | 3 | 1 | 0.11 | 0.10991 |
| GO:1903961 | positive regulation of anion transmembra.. | 3 | 1 | 0.11 | 0.10991 |
| GO:2000053 | regulation of Wnt signaling pathway invo... | 3 | 1 | 0.11 | 0.10991 |
| GO:2000118 | regulation of sodium-dependent phosphate | 3 | 1 | 0.11 | 0.10991 |
| GO:2000172 | regulation of branching morphogenesis of. | 3 | 1 | 0.11 | 0.10991 |
| GO:2000313 | regulation of fibroblast growth factor r... | 3 | 1 | 0.11 | 0.10991 |
| GO:2000364 | regulation of STAT protein import into n... | 3 | 1 | 0.11 | 0.10991 |
| GO:2000366 | positive regulation of STAT protein impo... | 3 | 1 | 0.11 | 0.10991 |
| GO:2000812 | regulation of barbed-end actin filament ... | 3 | 1 | 0.11 | 0.10991 |
| GO:2001023 | regulation of response to drug | 3 | 1 | 0.11 | 0.10991 |
| GO:2001180 | negative regulation of interleukin-10 se... | 3 | 1 | 0.11 | 0.10991 |
| GO:0038179 | neurotrophin signaling pathway | 278 | 15 | 10.58 | 0.11094 |
| GO:0044706 | multi-multicellular organism process | 212 | 12 | 8.07 | 0.11122 |
| GO:0006900 | membrane budding | 67 | 5 | 2.55 | 0.11128 |
| GO:0034162 | toll-like receptor 9 signaling pathway | 67 | 5 | 2.55 | 0.11128 |
| GO:0051188 | cofactor biosynthetic process | 127 | 8 | 4.83 | 0.11161 |
| GO:0006338 | chromatin remodeling | 148 | 9 | 5.63 | 0.11209 |
| GO:0009896 | positive regulation of catabolic process | 301 | 16 | 11.46 | 0.11247 |
| GO:0015804 | neutral amino acid transport | 31 | 3 | 1.18 | 0.11255 |
| GO:0031670 | cellular response to nutrient | 31 | 3 | 1.18 | 0.11255 |
| GO:1900024 | regulation of substrate adhesion-depende. | 31 | 3 | 1.18 | 0.11255 |
| GO:1903018 | regulation of glycoprotein metabolic pro... | 31 | 3 | 1.18 | 0.11255 |
| GO:0009163 | nucleoside biosynthetic process | 107 | 7 | 4.07 | 0.11358 |
| GO:1901659 | glycosyl compound biosynthetic process | 107 | 7 | 4.07 | 0.11358 |
| GO:0009798 | axis specification | 87 | 6 | 3.31 | 0.11392 |
| GO:0021549 | cerebellum development | 87 | 6 | 3.31 | 0.11392 |
| GO:0042254 | ribosome biogenesis | 170 | 10 | 6.47 | 0.11471 |
| GO:0046324 | regulation of glucose import | 49 | 4 | 1.87 | 0.11534 |
| GO:0048661 | positive regulation of smooth muscle cel... | 49 | 4 | 1.87 | 0.11534 |
| GO:0021782 | glial cell development | 68 | 5 | 2.59 | 0.11659 |
| GO:0051304 | chromosome separation | 68 | 5 | 2.59 | 0.11659 |
| GO:2000147 | positive regulation of cell motility | 303 | 16 | 11.53 | 0.11723 |
| GO:0090287 | regulation of cellular response to growt... | 171 | 10 | 6.51 | 0.11795 |
| GO:0002062 | chondrocyte differentiation | 88 | 6 | 3.35 | 0.11858 |
| GO:0010977 | negative regulation of neuron projection... | 88 | 6 | 3.35 | 0.11858 |
| GO:0071559 | response to transforming growth factor b... | 215 | 12 | 8.18 | 0.11982 |
| GO:0071560 | cellular response to transforming growth... | 215 | 12 | 8.18 | 0.11982 |
| GO:0019076 | viral release from host cell | 32 | 3 | 1.22 | 0.12087 |
| GO:0048854 | brain morphogenesis | 32 | 3 | 1.22 | 0.12087 |
| GO:0050732 | negative regulation of peptidyl-tyrosine... | 32 | 3 | 1.22 | 0.12087 |
| GO:0006940 | regulation of smooth muscle contraction | 50 | 4 | 1.9 | 0.12183 |

| | | | | | |
|---|---|---|---|---|---|
| GO:0061515 | myeloid cell development | 50 | 4 | 1.9 | 0.12183 |
| GO:0046660 | female sex differentiation | 109 | 7 | 4.15 | 0.12195 |
| GO:0006476 | protein deacetylation | 69 | 5 | 2.63 | 0.12201 |
| GO:0007588 | excretion | 69 | 5 | 2.63 | 0.12201 |
| GO:0034637 | cellular carbohydrate biosynthetic proce... | 69 | 5 | 2.63 | 0.12201 |
| GO:0048644 | muscle organ morphogenesis | 69 | 5 | 2.63 | 0.12201 |
| GO:0000959 | mitochondrial RNA metabolic process | 16 | 2 | 0.61 | 0.12221 |
| GO:0001967 | suckling behavior | 16 | 2 | 0.61 | 0.12221 |
| GO:0006555 | methionine metabolic process | 16 | 2 | 0.61 | 0.12221 |
| GO:0030949 | positive regulation of vascular endothel... | 16 | 2 | 0.61 | 0.12221 |
| GO:0032008 | positive regulation of TOR signaling | 16 | 2 | 0.61 | 0.12221 |
| GO:0032095 | regulation of response to food | 16 | 2 | 0.61 | 0.12221 |
| GO:0032528 | microvillus organization | 16 | 2 | 0.61 | 0.12221 |
| GO:0044380 | protein localization to cytoskeleton | 16 | 2 | 0.61 | 0.12221 |
| GO:0061099 | negative regulation of protein tyrosine ... | 16 | 2 | 0.61 | 0.12221 |
| GO:0070207 | protein homotrimerization | 16 | 2 | 0.61 | 0.12221 |
| GO:0070293 | renal absorption | 16 | 2 | 0.61 | 0.12221 |
| GO:0090025 | regulation of monocyte chemotaxis | 16 | 2 | 0.61 | 0.12221 |
| GO:0090083 | regulation of inclusion body assembly | 16 | 2 | 0.61 | 0.12221 |
| GO:0090178 | regulation of establishment of planar po... | 16 | 2 | 0.61 | 0.12221 |
| GO:1902001 | fatty acid transmembrane transport | 16 | 2 | 0.61 | 0.12221 |
| GO:1902236 | negative regulation of endoplasmic retic... | 16 | 2 | 0.61 | 0.12221 |
| GO:2000678 | negative regulation of transcription reg... | 16 | 2 | 0.61 | 0.12221 |
| GO:0002064 | epithelial cell development | 194 | 11 | 7.38 | 0.1224 |
| GO:0043270 | positive regulation of ion transport | 194 | 11 | 7.38 | 0.1224 |
| GO:0032412 | regulation of ion transmembrane transpor.. | 151 | 9 | 5.75 | 0.12259 |
| GO:0001892 | embryonic placenta development | 89 | 6 | 3.39 | 0.12333 |
| GO:0071322 | cellular response to carbohydrate stimul... | 89 | 6 | 3.39 | 0.12333 |
| GO:0045862 | positive regulation of proteolysis | 306 | 16 | 11.65 | 0.1246 |
| GO:0021915 | neural tube development | 152 | 9 | 5.79 | 0.12621 |
| GO:0048762 | mesenchymal cell differentiation | 152 | 9 | 5.79 | 0.12621 |
| GO:0050680 | negative regulation of epithelial cell p... | 110 | 7 | 4.19 | 0.12625 |
| GO:0071456 | cellular response to hypoxia | 110 | 7 | 4.19 | 0.12625 |
| GO:0034134 | toll-like receptor 2 signaling pathway | 70 | 5 | 2.66 | 0.12755 |
| GO:0070167 | regulation of biomineral tissue developm... | 70 | 5 | 2.66 | 0.12755 |
| GO:0010906 | regulation of glucose metabolic process | 90 | 6 | 3.43 | 0.12817 |
| GO:0000018 | regulation of DNA recombination | 51 | 4 | 1.94 | 0.12847 |
| GO:0010389 | regulation of G2/M transition of mitotic... | 33 | 3 | 1.26 | 0.12941 |
| GO:0035115 | embryonic forelimb morphogenesis | 33 | 3 | 1.26 | 0.12941 |
| GO:0045646 | regulation of erythrocyte differentiatio... | 33 | 3 | 1.26 | 0.12941 |
| GO:0065002 | intracellular protein transmembrane tran... | 33 | 3 | 1.26 | 0.12941 |
| GO:0070192 | chromosome organization involved in meic | 33 | 3 | 1.26 | 0.12941 |
| GO:0090329 | regulation of DNA-dependent DNA replicat | 33 | 3 | 1.26 | 0.12941 |
| GO:1900034 | regulation of cellular response to heat | 33 | 3 | 1.26 | 0.12941 |
| GO:0007067 | mitotic nuclear division | 354 | 18 | 13.48 | 0.13049 |
| GO:0009411 | response to UV | 111 | 7 | 4.23 | 0.13063 |
| GO:0016052 | carbohydrate catabolic process | 132 | 8 | 5.02 | 0.13093 |
| GO:0032970 | regulation of actin filament-based proce... | 286 | 15 | 10.89 | 0.13138 |
| GO:0050673 | epithelial cell proliferation | 309 | 16 | 11.76 | 0.13222 |
| GO:0034765 | regulation of ion transmembrane transpor.. | 332 | 17 | 12.64 | 0.13271 |
| GO:0032075 | positive regulation of nuclease activity | 71 | 5 | 2.7 | 0.13321 |
| GO:0051149 | positive regulation of muscle cell diffe... | 71 | 5 | 2.7 | 0.13321 |
| GO:0002224 | toll-like receptor signaling pathway | 133 | 8 | 5.06 | 0.13498 |

| | | | | | |
|---|---|---|---|---|---|
| GO:0006941 | striated muscle contraction | 133 | 8 | 5.06 | 0.13498 |
| GO:0046890 | regulation of lipid biosynthetic process | 112 | 7 | 4.26 | 0.13508 |
| GO:0001522 | pseudouridine synthesis | 17 | 2 | 0.65 | 0.13516 |
| GO:0002902 | regulation of B cell apoptotic process | 17 | 2 | 0.65 | 0.13516 |
| GO:0014829 | vascular smooth muscle contraction | 17 | 2 | 0.65 | 0.13516 |
| GO:0030540 | female genitalia development | 17 | 2 | 0.65 | 0.13516 |
| GO:0032288 | myelin assembly | 17 | 2 | 0.65 | 0.13516 |
| GO:0032891 | negative regulation of organic acid tran... | 17 | 2 | 0.65 | 0.13516 |
| GO:0035338 | long-chain fatty-acyl-CoA biosynthetic p... | 17 | 2 | 0.65 | 0.13516 |
| GO:0042136 | neurotransmitter biosynthetic process | 17 | 2 | 0.65 | 0.13516 |
| GO:0051767 | nitric-oxide synthase biosynthetic proce... | 17 | 2 | 0.65 | 0.13516 |
| GO:0051769 | regulation of nitric-oxide synthase bios... | 17 | 2 | 0.65 | 0.13516 |
| GO:0090177 | establishment of planar polarity involve... | 17 | 2 | 0.65 | 0.13516 |
| GO:0090312 | positive regulation of protein deacetyla... | 17 | 2 | 0.65 | 0.13516 |
| GO:2000144 | positive regulation of DNA-templated tra... | 17 | 2 | 0.65 | 0.13516 |
| GO:0022898 | regulation of transmembrane transporter .. | 155 | 9 | 5.9 | 0.1374 |
| GO:0010828 | positive regulation of glucose transport | 34 | 3 | 1.29 | 0.13815 |
| GO:0035384 | thioester biosynthetic process | 34 | 3 | 1.29 | 0.13815 |
| GO:0035924 | cellular response to vascular endothelia... | 34 | 3 | 1.29 | 0.13815 |
| GO:0045777 | positive regulation of blood pressure | 34 | 3 | 1.29 | 0.13815 |
| GO:0071616 | acyl-CoA biosynthetic process | 34 | 3 | 1.29 | 0.13815 |
| GO:0030178 | negative regulation of Wnt signaling pat... | 177 | 10 | 6.74 | 0.13848 |
| GO:0006367 | transcription initiation from RNA polyme... | 199 | 11 | 7.58 | 0.13863 |
| GO:0035601 | protein deacylation | 72 | 5 | 2.74 | 0.13897 |
| GO:0035666 | TRIF-dependent toll-like receptor signal... | 72 | 5 | 2.74 | 0.13897 |
| GO:0045639 | positive regulation of myeloid cell diff... | 72 | 5 | 2.74 | 0.13897 |
| GO:0035148 | tube formation | 134 | 8 | 5.1 | 0.1391 |
| GO:0046165 | alcohol biosynthetic process | 134 | 8 | 5.1 | 0.1391 |
| GO:0031145 | anaphase-promoting complex-dependent p | 113 | 7 | 4.3 | 0.13961 |
| GO:0051272 | positive regulation of cellular componen... | 312 | 16 | 11.88 | 0.14011 |
| GO:1901988 | negative regulation of cell cycle phase ... | 156 | 9 | 5.94 | 0.14123 |
| GO:0000045 | autophagic vacuole assembly | 53 | 4 | 2.02 | 0.14218 |
| GO:0031100 | organ regeneration | 53 | 4 | 2.02 | 0.14218 |
| GO:0035690 | cellular response to drug | 53 | 4 | 2.02 | 0.14218 |
| GO:0051785 | positive regulation of nuclear division | 53 | 4 | 2.02 | 0.14218 |
| GO:0055008 | cardiac muscle tissue morphogenesis | 53 | 4 | 2.02 | 0.14218 |
| GO:0006821 | chloride transport | 93 | 6 | 3.54 | 0.14324 |
| GO:0050920 | regulation of chemotaxis | 135 | 8 | 5.14 | 0.14329 |
| GO:0000019 | regulation of mitotic recombination | 4 | 1 | 0.15 | 0.1438 |
| GO:0000429 | carbon catabolite regulation of transcri... | 4 | 1 | 0.15 | 0.1438 |
| GO:0000436 | carbon catabolite activation of transcri... | 4 | 1 | 0.15 | 0.1438 |
| GO:0000963 | mitochondrial RNA processing | 4 | 1 | 0.15 | 0.1438 |
| GO:0000966 | RNA 5'-end processing | 4 | 1 | 0.15 | 0.1438 |
| GO:0001806 | type IV hypersensitivity | 4 | 1 | 0.15 | 0.1438 |
| GO:0002901 | mature B cell apoptotic process | 4 | 1 | 0.15 | 0.1438 |
| GO:0002905 | regulation of mature B cell apoptotic pr... | 4 | 1 | 0.15 | 0.1438 |
| GO:0002906 | negative regulation of mature B cell apo... | 4 | 1 | 0.15 | 0.1438 |
| GO:0003099 | positive regulation of the force of hear... | 4 | 1 | 0.15 | 0.1438 |
| GO:0003199 | endocardial cushion to mesenchymal trans | 4 | 1 | 0.15 | 0.1438 |
| GO:0006015 | 5-phosphoribose 1-diphosphate biosynthet | 4 | 1 | 0.15 | 0.1438 |
| GO:0006021 | inositol biosynthetic process | 4 | 1 | 0.15 | 0.1438 |
| GO:0006166 | purine ribonucleoside salvage | 4 | 1 | 0.15 | 0.1438 |
| GO:0006235 | dTTP biosynthetic process | 4 | 1 | 0.15 | 0.1438 |

| | | | | | |
|---|---|---|---|---|---|
| GO:0006311 | meiotic gene conversion | 4 | 1 | 0.15 | 0.1438 |
| GO:0006499 | N-terminal protein myristoylation | 4 | 1 | 0.15 | 0.1438 |
| GO:0006659 | phosphatidylserine biosynthetic process | 4 | 1 | 0.15 | 0.1438 |
| GO:0006663 | platelet activating factor biosynthetic ... | 4 | 1 | 0.15 | 0.1438 |
| GO:0006771 | riboflavin metabolic process | 4 | 1 | 0.15 | 0.1438 |
| GO:0007089 | traversing start control point of mitoti... | 4 | 1 | 0.15 | 0.1438 |
| GO:0007258 | JUN phosphorylation | 4 | 1 | 0.15 | 0.1438 |
| GO:0009138 | pyrimidine nucleoside diphosphate metabc | 4 | 1 | 0.15 | 0.1438 |
| GO:0009212 | pyrimidine deoxyribonucleoside triphosph.. | 4 | 1 | 0.15 | 0.1438 |
| GO:0010587 | miRNA catabolic process | 4 | 1 | 0.15 | 0.1438 |
| GO:0010990 | regulation of SMAD protein complex asser | 4 | 1 | 0.15 | 0.1438 |
| GO:0010991 | negative regulation of SMAD protein comp | 4 | 1 | 0.15 | 0.1438 |
| GO:0014045 | establishment of endothelial blood-brain... | 4 | 1 | 0.15 | 0.1438 |
| GO:0014826 | vein smooth muscle contraction | 4 | 1 | 0.15 | 0.1438 |
| GO:0014834 | skeletal muscle satellite cell maintenan... | 4 | 1 | 0.15 | 0.1438 |
| GO:0015670 | carbon dioxide transport | 4 | 1 | 0.15 | 0.1438 |
| GO:0015801 | aromatic amino acid transport | 4 | 1 | 0.15 | 0.1438 |
| GO:0015803 | branched-chain amino acid transport | 4 | 1 | 0.15 | 0.1438 |
| GO:0016199 | axon midline choice point recognition | 4 | 1 | 0.15 | 0.1438 |
| GO:0018076 | N-terminal peptidyl-lysine acetylation | 4 | 1 | 0.15 | 0.1438 |
| GO:0019896 | axon transport of mitochondrion | 4 | 1 | 0.15 | 0.1438 |
| GO:0021943 | formation of radial glial scaffolds | 4 | 1 | 0.15 | 0.1438 |
| GO:0022009 | central nervous system vasculogenesis | 4 | 1 | 0.15 | 0.1438 |
| GO:0030091 | protein repair | 4 | 1 | 0.15 | 0.1438 |
| GO:0030222 | eosinophil differentiation | 4 | 1 | 0.15 | 0.1438 |
| GO:0031848 | protection from non-homologous end joini. | 4 | 1 | 0.15 | 0.1438 |
| GO:0032097 | positive regulation of response to food | 4 | 1 | 0.15 | 0.1438 |
| GO:0032100 | positive regulation of appetite | 4 | 1 | 0.15 | 0.1438 |
| GO:0032278 | positive regulation of gonadotropin secr... | 4 | 1 | 0.15 | 0.1438 |
| GO:0032304 | negative regulation of icosanoid secreti... | 4 | 1 | 0.15 | 0.1438 |
| GO:0032696 | negative regulation of interleukin-13 pr... | 4 | 1 | 0.15 | 0.1438 |
| GO:0032763 | regulation of mast cell cytokine product... | 4 | 1 | 0.15 | 0.1438 |
| GO:0033136 | serine phosphorylation of STAT3 protein | 4 | 1 | 0.15 | 0.1438 |
| GO:0033314 | mitotic DNA replication checkpoint | 4 | 1 | 0.15 | 0.1438 |
| GO:0033684 | regulation of luteinizing hormone secret... | 4 | 1 | 0.15 | 0.1438 |
| GO:0034139 | regulation of toll-like receptor 3 signa... | 4 | 1 | 0.15 | 0.1438 |
| GO:0034442 | regulation of lipoprotein oxidation | 4 | 1 | 0.15 | 0.1438 |
| GO:0035822 | gene conversion | 4 | 1 | 0.15 | 0.1438 |
| GO:0035948 | positive regulation of gluconeogenesis b... | 4 | 1 | 0.15 | 0.1438 |
| GO:0036492 | eiF2alpha phosphorylation in response to.. | 4 | 1 | 0.15 | 0.1438 |
| GO:0038033 | positive regulation of endothelial cell ... | 4 | 1 | 0.15 | 0.1438 |
| GO:0038161 | prolactin signaling pathway | 4 | 1 | 0.15 | 0.1438 |
| GO:0042663 | regulation of endodermal cell fate speci... | 4 | 1 | 0.15 | 0.1438 |
| GO:0042726 | flavin-containing compound metabolic pro. | 4 | 1 | 0.15 | 0.1438 |
| GO:0043247 | telomere maintenance in response to DNA | 4 | 1 | 0.15 | 0.1438 |
| GO:0043305 | negative regulation of mast cell degranu... | 4 | 1 | 0.15 | 0.1438 |
| GO:0044531 | modulation of programmed cell death in o. | 4 | 1 | 0.15 | 0.1438 |
| GO:0044532 | modulation of apoptotic process in other... | 4 | 1 | 0.15 | 0.1438 |
| GO:0044828 | negative regulation by host of viral gen... | 4 | 1 | 0.15 | 0.1438 |
| GO:0044854 | plasma membrane raft assembly | 4 | 1 | 0.15 | 0.1438 |
| GO:0044857 | plasma membrane raft organization | 4 | 1 | 0.15 | 0.1438 |
| GO:0045008 | depyrimidination | 4 | 1 | 0.15 | 0.1438 |
| GO:0045039 | protein import into mitochondrial inner ... | 4 | 1 | 0.15 | 0.1438 |

| | | | | | |
|---|---|---|---|---|---|
| GO:0045716 | positive regulation of low-density lipop... | 4 | 1 | 0.15 | 0.1438 |
| GO:0046075 | dTTP metabolic process | 4 | 1 | 0.15 | 0.1438 |
| GO:0046391 | 5-phosphoribose 1-diphosphate metabolic | 4 | 1 | 0.15 | 0.1438 |
| GO:0046881 | positive regulation of follicle-stimulat... | 4 | 1 | 0.15 | 0.1438 |
| GO:0048478 | replication fork protection | 4 | 1 | 0.15 | 0.1438 |
| GO:0048619 | embryonic hindgut morphogenesis | 4 | 1 | 0.15 | 0.1438 |
| GO:0048680 | positive regulation of axon regeneration | 4 | 1 | 0.15 | 0.1438 |
| GO:0050923 | regulation of negative chemotaxis | 4 | 1 | 0.15 | 0.1438 |
| GO:0051541 | elastin metabolic process | 4 | 1 | 0.15 | 0.1438 |
| GO:0052040 | modulation by symbiont of host programm | 4 | 1 | 0.15 | 0.1438 |
| GO:0052150 | modulation by symbiont of host apoptotic.. | 4 | 1 | 0.15 | 0.1438 |
| GO:0052248 | modulation of programmed cell death in o. | 4 | 1 | 0.15 | 0.1438 |
| GO:0052433 | modulation by organism of apoptotic proc.. | 4 | 1 | 0.15 | 0.1438 |
| GO:0060075 | regulation of resting membrane potential | 4 | 1 | 0.15 | 0.1438 |
| GO:0060214 | endocardium formation | 4 | 1 | 0.15 | 0.1438 |
| GO:0060298 | positive regulation of sarcomere organiz... | 4 | 1 | 0.15 | 0.1438 |
| GO:0060339 | negative regulation of type I interferon... | 4 | 1 | 0.15 | 0.1438 |
| GO:0060535 | trachea cartilage morphogenesis | 4 | 1 | 0.15 | 0.1438 |
| GO:0060564 | negative regulation of APC-Cdc20 comple | 4 | 1 | 0.15 | 0.1438 |
| GO:0060596 | mammary placode formation | 4 | 1 | 0.15 | 0.1438 |
| GO:0060699 | regulation of endoribonuclease activity | 4 | 1 | 0.15 | 0.1438 |
| GO:0060903 | positive regulation of meiosis I | 4 | 1 | 0.15 | 0.1438 |
| GO:0060965 | negative regulation of gene silencing by... | 4 | 1 | 0.15 | 0.1438 |
| GO:0061038 | uterus morphogenesis | 4 | 1 | 0.15 | 0.1438 |
| GO:0061086 | negative regulation of histone H3-K27 me. | 4 | 1 | 0.15 | 0.1438 |
| GO:0061325 | cell proliferation involved in outflow t... | 4 | 1 | 0.15 | 0.1438 |
| GO:0061428 | negative regulation of transcription fro... | 4 | 1 | 0.15 | 0.1438 |
| GO:0070444 | oligodendrocyte progenitor proliferation | 4 | 1 | 0.15 | 0.1438 |
| GO:0070445 | regulation of oligodendrocyte progenitor... | 4 | 1 | 0.15 | 0.1438 |
| GO:0070459 | prolactin secretion | 4 | 1 | 0.15 | 0.1438 |
| GO:0070672 | response to interleukin-15 | 4 | 1 | 0.15 | 0.1438 |
| GO:0070836 | caveola assembly | 4 | 1 | 0.15 | 0.1438 |
| GO:0071317 | cellular response to isoquinoline alkalo... | 4 | 1 | 0.15 | 0.1438 |
| GO:0072603 | interleukin-5 secretion | 4 | 1 | 0.15 | 0.1438 |
| GO:0072683 | T cell extravasation | 4 | 1 | 0.15 | 0.1438 |
| GO:0098727 | maintenance of cell number | 4 | 1 | 0.15 | 0.1438 |
| GO:1900165 | negative regulation of interleukin-6 sec... | 4 | 1 | 0.15 | 0.1438 |
| GO:1901223 | negative regulation of NIK/NF-kappaB sig. | 4 | 1 | 0.15 | 0.1438 |
| GO:1901299 | negative regulation of hydrogen peroxide.. | 4 | 1 | 0.15 | 0.1438 |
| GO:1901490 | regulation of lymphangiogenesis | 4 | 1 | 0.15 | 0.1438 |
| GO:1901629 | regulation of presynaptic membrane organ | 4 | 1 | 0.15 | 0.1438 |
| GO:1903912 | negative regulation of endoplasmic retic... | 4 | 1 | 0.15 | 0.1438 |
| GO:2000643 | positive regulation of early endosome to... | 4 | 1 | 0.15 | 0.1438 |
| GO:2000662 | regulation of interleukin-5 secretion | 4 | 1 | 0.15 | 0.1438 |
| GO:2000821 | regulation of grooming behavior | 4 | 1 | 0.15 | 0.1438 |
| GO:0010212 | response to ionizing radiation | 114 | 7 | 4.34 | 0.14421 |
| GO:0048709 | oligodendrocyte differentiation | 73 | 5 | 2.78 | 0.14484 |
| GO:0098732 | macromolecule deacylation | 73 | 5 | 2.78 | 0.14484 |
| GO:1903051 | negative regulation of proteolysis invol... | 73 | 5 | 2.78 | 0.14484 |
| GO:0043627 | response to estrogen | 157 | 9 | 5.98 | 0.14513 |
| GO:0003091 | renal water homeostasis | 35 | 3 | 1.33 | 0.14709 |
| GO:0046794 | transport of virus | 35 | 3 | 1.33 | 0.14709 |
| GO:0098801 | regulation of renal system process | 35 | 3 | 1.33 | 0.14709 |

| | | | | | |
|---|---|---|---|---|---|
| GO:1901099 | negative regulation of signal transducti... | 35 | 3 | 1.33 | 0.14709 |
| GO:1902591 | single-organism membrane budding | 35 | 3 | 1.33 | 0.14709 |
| GO:1902749 | regulation of cell cycle G2/M phase tran... | 35 | 3 | 1.33 | 0.14709 |
| GO:2001240 | negative regulation of extrinsic apoptot... | 35 | 3 | 1.33 | 0.14709 |
| GO:0035264 | multicellular organism growth | 136 | 8 | 5.18 | 0.14753 |
| GO:0006839 | mitochondrial transport | 224 | 12 | 8.53 | 0.1478 |
| GO:0001829 | trophectodermal cell differentiation | 18 | 2 | 0.69 | 0.1484 |
| GO:0006359 | regulation of transcription from RNA pol... | 18 | 2 | 0.69 | 0.1484 |
| GO:0006995 | cellular response to nitrogen starvation | 18 | 2 | 0.69 | 0.1484 |
| GO:0009067 | aspartate family amino acid biosynthetic... | 18 | 2 | 0.69 | 0.1484 |
| GO:0015697 | quaternary ammonium group transport | 18 | 2 | 0.69 | 0.1484 |
| GO:0016578 | histone deubiquitination | 18 | 2 | 0.69 | 0.1484 |
| GO:0032303 | regulation of icosanoid secretion | 18 | 2 | 0.69 | 0.1484 |
| GO:0035809 | regulation of urine volume | 18 | 2 | 0.69 | 0.1484 |
| GO:0042249 | establishment of planar polarity of embr... | 18 | 2 | 0.69 | 0.1484 |
| GO:0043562 | cellular response to nitrogen levels | 18 | 2 | 0.69 | 0.1484 |
| GO:0044319 | wound healing | spreading c | 18 | 2 | 0.69 |
| GO:0048853 | forebrain morphogenesis | 18 | 2 | 0.69 | 0.1484 |
| GO:0090505 | epiboly involved in wound healing | 18 | 2 | 0.69 | 0.1484 |
| GO:0042476 | odontogenesis | 115 | 7 | 4.38 | 0.14888 |
| GO:2001238 | positive regulation of extrinsic apoptot... | 54 | 4 | 2.06 | 0.14925 |
| GO:0006497 | protein lipidation | 74 | 5 | 2.82 | 0.15082 |
| GO:0019218 | regulation of steroid metabolic process | 74 | 5 | 2.82 | 0.15082 |
| GO:0030902 | hindbrain development | 137 | 8 | 5.22 | 0.15184 |
| GO:0007623 | circadian rhythm | 181 | 10 | 6.89 | 0.15315 |
| GO:0032355 | response to estradiol | 95 | 6 | 3.62 | 0.15372 |
| GO:0006631 | fatty acid metabolic process | 317 | 16 | 12.07 | 0.15384 |
| GO:0048193 | Golgi vesicle transport | 226 | 12 | 8.6 | 0.15445 |
| GO:0002053 | positive regulation of mesenchymal cell ... | 36 | 3 | 1.37 | 0.15621 |
| GO:0002704 | negative regulation of leukocyte mediate... | 36 | 3 | 1.37 | 0.15621 |
| GO:0006903 | vesicle targeting | 36 | 3 | 1.37 | 0.15621 |
| GO:0030815 | negative regulation of cAMP metabolic pr... | 36 | 3 | 1.37 | 0.15621 |
| GO:0044060 | regulation of endocrine process | 36 | 3 | 1.37 | 0.15621 |
| GO:0044766 | multi-organism transport | 36 | 3 | 1.37 | 0.15621 |
| GO:0045668 | negative regulation of osteoblast differ... | 36 | 3 | 1.37 | 0.15621 |
| GO:0048009 | insulin-like growth factor receptor sign... | 36 | 3 | 1.37 | 0.15621 |
| GO:1902579 | multi-organism localization | 36 | 3 | 1.37 | 0.15621 |
| GO:0021766 | hippocampus development | 55 | 4 | 2.09 | 0.15644 |
| GO:0031929 | TOR signaling | 55 | 4 | 2.09 | 0.15644 |
| GO:0045682 | regulation of epidermis development | 55 | 4 | 2.09 | 0.15644 |
| GO:0060078 | regulation of postsynaptic membrane pote | 55 | 4 | 2.09 | 0.15644 |
| GO:0030031 | cell projection assembly | 272 | 14 | 10.35 | 0.15648 |
| GO:0002431 | Fc receptor mediated stimulatory signali... | 75 | 5 | 2.85 | 0.1569 |
| GO:0002756 | MyD88-independent toll-like receptor sig... | 75 | 5 | 2.85 | 0.1569 |
| GO:0050886 | endocrine process | 75 | 5 | 2.85 | 0.1569 |
| GO:0051983 | regulation of chromosome segregation | 75 | 5 | 2.85 | 0.1569 |
| GO:0008643 | carbohydrate transport | 160 | 9 | 6.09 | 0.15713 |
| GO:0030282 | bone mineralization | 96 | 6 | 3.65 | 0.15908 |
| GO:0071496 | cellular response to external stimulus | 273 | 14 | 10.39 | 0.15958 |
| GO:0098742 | cell-cell adhesion via plasma-membrane a | 183 | 10 | 6.97 | 0.16076 |
| GO:0000266 | mitochondrial fission | 19 | 2 | 0.72 | 0.16189 |
| GO:0002076 | osteoblast development | 19 | 2 | 0.72 | 0.16189 |
| GO:0007143 | female meiotic division | 19 | 2 | 0.72 | 0.16189 |

| | | | | | |
|---|---|---|---|---|---|
| GO:0035307 | positive regulation of protein dephospho... | 19 | 2 | 0.72 | 0.16189 |
| GO:0044818 | mitotic G2/M transition checkpoint | 19 | 2 | 0.72 | 0.16189 |
| GO:0046949 | fatty-acyl-CoA biosynthetic process | 19 | 2 | 0.72 | 0.16189 |
| GO:0071295 | cellular response to vitamin | 19 | 2 | 0.72 | 0.16189 |
| GO:0071875 | adrenergic receptor signaling pathway | 19 | 2 | 0.72 | 0.16189 |
| GO:0090504 | epiboly | 19 | 2 | 0.72 | 0.16189 |
| GO:2000311 | regulation of alpha-amino-3-hydroxy-5-me. | 19 | 2 | 0.72 | 0.16189 |
| GO:2000463 | positive regulation of excitatory postsy... | 19 | 2 | 0.72 | 0.16189 |
| GO:0040017 | positive regulation of locomotion | 320 | 16 | 12.18 | 0.16241 |
| GO:0048011 | neurotrophin TRK receptor signaling path.. | 274 | 14 | 10.43 | 0.16271 |
| GO:0005977 | glycogen metabolic process | 76 | 5 | 2.89 | 0.16308 |
| GO:0019217 | regulation of fatty acid metabolic proce... | 76 | 5 | 2.89 | 0.16308 |
| GO:0051439 | regulation of ubiquitin-protein ligase a... | 76 | 5 | 2.89 | 0.16308 |
| GO:0055117 | regulation of cardiac muscle contraction | 76 | 5 | 2.89 | 0.16308 |
| GO:0060350 | endochondral bone morphogenesis | 56 | 4 | 2.13 | 0.16377 |
| GO:0060840 | artery development | 56 | 4 | 2.13 | 0.16377 |
| GO:0017015 | regulation of transforming growth factor... | 97 | 6 | 3.69 | 0.16452 |
| GO:1903844 | regulation of cellular response to trans... | 97 | 6 | 3.69 | 0.16452 |
| GO:0007179 | transforming growth factor beta receptor... | 184 | 10 | 7 | 0.16464 |
| GO:0098609 | cell-cell adhesion | 184 | 10 | 7 | 0.16464 |
| GO:0043491 | protein kinase B signaling | 140 | 8 | 5.33 | 0.16512 |
| GO:0071229 | cellular response to acid chemical | 140 | 8 | 5.33 | 0.16512 |
| GO:0030800 | negative regulation of cyclic nucleotide... | 37 | 3 | 1.41 | 0.1655 |
| GO:0045806 | negative regulation of endocytosis | 37 | 3 | 1.41 | 0.1655 |
| GO:0072350 | tricarboxylic acid metabolic process | 37 | 3 | 1.41 | 0.1655 |
| GO:0030335 | positive regulation of cell migration | 298 | 15 | 11.34 | 0.16576 |
| GO:0043161 | proteasome-mediated ubiquitin-dependent | 345 | 17 | 13.13 | 0.16745 |
| GO:0048638 | regulation of developmental growth | 230 | 12 | 8.76 | 0.16821 |
| GO:0032874 | positive regulation of stress-activated ... | 119 | 7 | 4.53 | 0.16825 |
| GO:0016051 | carbohydrate biosynthetic process | 185 | 10 | 7.04 | 0.16856 |
| GO:0006073 | cellular glucan metabolic process | 77 | 5 | 2.93 | 0.16935 |
| GO:0044042 | glucan metabolic process | 77 | 5 | 2.93 | 0.16935 |
| GO:1903363 | negative regulation of cellular protein ... | 77 | 5 | 2.93 | 0.16935 |
| GO:0001890 | placenta development | 141 | 8 | 5.37 | 0.16966 |
| GO:0009566 | fertilization | 141 | 8 | 5.37 | 0.16966 |
| GO:0042455 | ribonucleoside biosynthetic process | 98 | 6 | 3.73 | 0.17004 |
| GO:0034762 | regulation of transmembrane transport | 346 | 17 | 13.17 | 0.1703 |
| GO:0031668 | cellular response to extracellular stimu... | 208 | 11 | 7.92 | 0.17058 |
| GO:0001836 | release of cytochrome c from mitochondri.. | 57 | 4 | 2.17 | 0.17121 |
| GO:0046323 | glucose import | 57 | 4 | 2.17 | 0.17121 |
| GO:0050891 | multicellular organismal water homeostas.. | 57 | 4 | 2.17 | 0.17121 |
| GO:0007162 | negative regulation of cell adhesion | 186 | 10 | 7.08 | 0.17252 |
| GO:0070304 | positive regulation of stress-activated ... | 120 | 7 | 4.57 | 0.17326 |
| GO:0051091 | positive regulation of sequence-specific... | 209 | 11 | 7.96 | 0.17433 |
| GO:0006220 | pyrimidine nucleotide metabolic process | 38 | 3 | 1.45 | 0.17495 |
| GO:0030199 | collagen fibril organization | 38 | 3 | 1.45 | 0.17495 |
| GO:0055010 | ventricular cardiac muscle tissue morpho.. | 38 | 3 | 1.45 | 0.17495 |
| GO:0061383 | trabecula morphogenesis | 38 | 3 | 1.45 | 0.17495 |
| GO:0001945 | lymph vessel development | 20 | 2 | 0.76 | 0.17558 |
| GO:0006278 | RNA-dependent DNA replication | 20 | 2 | 0.76 | 0.17558 |
| GO:0010869 | regulation of receptor biosynthetic proc... | 20 | 2 | 0.76 | 0.17558 |
| GO:0010894 | negative regulation of steroid biosynthe... | 20 | 2 | 0.76 | 0.17558 |
| GO:0031954 | positive regulation of protein autophosp... | 20 | 2 | 0.76 | 0.17558 |

| | | | | | |
|---|---|---|---|---|---|
| GO:0033057 | multicellular organismal reproductive be... | 20 | 2 | 0.76 | 0.17558 |
| GO:0038084 | vascular endothelial growth factor signa... | 20 | 2 | 0.76 | 0.17558 |
| GO:0042474 | middle ear morphogenesis | 20 | 2 | 0.76 | 0.17558 |
| GO:0045061 | thymic T cell selection | 20 | 2 | 0.76 | 0.17558 |
| GO:0045648 | positive regulation of erythrocyte diffe... | 20 | 2 | 0.76 | 0.17558 |
| GO:0045737 | positive regulation of cyclin-dependent ... | 20 | 2 | 0.76 | 0.17558 |
| GO:0045932 | negative regulation of muscle contractio... | 20 | 2 | 0.76 | 0.17558 |
| GO:0046885 | regulation of hormone biosynthetic proce... | 20 | 2 | 0.76 | 0.17558 |
| GO:0070932 | histone H3 deacetylation | 20 | 2 | 0.76 | 0.17558 |
| GO:0097503 | sialylation | 20 | 2 | 0.76 | 0.17558 |
| GO:0009108 | coenzyme biosynthetic process | 99 | 6 | 3.77 | 0.17564 |
| GO:0022037 | metencephalon development | 99 | 6 | 3.77 | 0.17564 |
| GO:0034142 | toll-like receptor 4 signaling pathway | 99 | 6 | 3.77 | 0.17564 |
| GO:0001952 | regulation of cell-matrix adhesion | 78 | 5 | 2.97 | 0.17572 |
| GO:0002027 | regulation of heart rate | 78 | 5 | 2.97 | 0.17572 |
| GO:0002755 | MyD88-dependent toll-like receptor signa.. | 78 | 5 | 2.97 | 0.17572 |
| GO:0032069 | regulation of nuclease activity | 78 | 5 | 2.97 | 0.17572 |
| GO:0051705 | multi-organism behavior | 78 | 5 | 2.97 | 0.17572 |
| GO:0001554 | luteolysis | 5 | 1 | 0.19 | 0.1764 |
| GO:0003100 | regulation of systemic arterial blood pr... | 5 | 1 | 0.19 | 0.1764 |
| GO:0003278 | apoptotic process involved in heart morp... | 5 | 1 | 0.19 | 0.1764 |
| GO:0003308 | negative regulation of Wnt signaling pat... | 5 | 1 | 0.19 | 0.1764 |
| GO:0003344 | pericardium morphogenesis | 5 | 1 | 0.19 | 0.1764 |
| GO:0003406 | retinal pigment epithelium development | 5 | 1 | 0.19 | 0.1764 |
| GO:0006048 | UDP-N-acetylglucosamine biosynthetic prc | 5 | 1 | 0.19 | 0.1764 |
| GO:0006474 | N-terminal protein amino acid acetylatio... | 5 | 1 | 0.19 | 0.1764 |
| GO:0006537 | glutamate biosynthetic process | 5 | 1 | 0.19 | 0.1764 |
| GO:0006566 | threonine metabolic process | 5 | 1 | 0.19 | 0.1764 |
| GO:0006572 | tyrosine catabolic process | 5 | 1 | 0.19 | 0.1764 |
| GO:0006868 | glutamine transport | 5 | 1 | 0.19 | 0.1764 |
| GO:0007008 | outer mitochondrial membrane organizatio | 5 | 1 | 0.19 | 0.1764 |
| GO:0008054 | negative regulation of cyclin-dependent ... | 5 | 1 | 0.19 | 0.1764 |
| GO:0008063 | Toll signaling pathway | 5 | 1 | 0.19 | 0.1764 |
| GO:0009189 | deoxyribonucleoside diphosphate biosynth | 5 | 1 | 0.19 | 0.1764 |
| GO:0009202 | deoxyribonucleoside triphosphate biosynt.. | 5 | 1 | 0.19 | 0.1764 |
| GO:0009211 | pyrimidine deoxyribonucleoside triphosph.. | 5 | 1 | 0.19 | 0.1764 |
| GO:0009414 | response to water deprivation | 5 | 1 | 0.19 | 0.1764 |
| GO:0010193 | response to ozone | 5 | 1 | 0.19 | 0.1764 |
| GO:0010606 | positive regulation of cytoplasmic mRNA .. | 5 | 1 | 0.19 | 0.1764 |
| GO:0010966 | regulation of phosphate transport | 5 | 1 | 0.19 | 0.1764 |
| GO:0014029 | neural crest formation | 5 | 1 | 0.19 | 0.1764 |
| GO:0014719 | skeletal muscle satellite cell activatio... | 5 | 1 | 0.19 | 0.1764 |
| GO:0016233 | telomere capping | 5 | 1 | 0.19 | 0.1764 |
| GO:0018377 | protein myristoylation | 5 | 1 | 0.19 | 0.1764 |
| GO:0021861 | forebrain radial glial cell differentiat... | 5 | 1 | 0.19 | 0.1764 |
| GO:0023041 | neuronal signal transduction | 5 | 1 | 0.19 | 0.1764 |
| GO:0030718 | germ-line stem cell maintenance | 5 | 1 | 0.19 | 0.1764 |
| GO:0031034 | myosin filament assembly | 5 | 1 | 0.19 | 0.1764 |
| GO:0031643 | positive regulation of myelination | 5 | 1 | 0.19 | 0.1764 |
| GO:0032415 | regulation of sodium:proton antiporter a... | 5 | 1 | 0.19 | 0.1764 |
| GO:0032534 | regulation of microvillus assembly | 5 | 1 | 0.19 | 0.1764 |
| GO:0032876 | negative regulation of DNA endoreduplica. | 5 | 1 | 0.19 | 0.1764 |
| GO:0033007 | negative regulation of mast cell activat... | 5 | 1 | 0.19 | 0.1764 |

| | | | | | |
|---|---|---|---|---|---|
| GO:0033313 | meiotic cell cycle checkpoint | 5 | 1 | 0.19 | 0.1764 |
| GO:0033564 | anterior/posterior axon guidance | 5 | 1 | 0.19 | 0.1764 |
| GO:0033591 | response to L-ascorbic acid | 5 | 1 | 0.19 | 0.1764 |
| GO:0033601 | positive regulation of mammary gland epi.. | 5 | 1 | 0.19 | 0.1764 |
| GO:0034086 | maintenance of sister chromatid cohesion | 5 | 1 | 0.19 | 0.1764 |
| GO:0034088 | maintenance of mitotic sister chromatid ... | 5 | 1 | 0.19 | 0.1764 |
| GO:0034219 | carbohydrate transmembrane transport | 5 | 1 | 0.19 | 0.1764 |
| GO:0035331 | negative regulation of hippo signaling | 5 | 1 | 0.19 | 0.1764 |
| GO:0035771 | interleukin-4-mediated signaling pathway | 5 | 1 | 0.19 | 0.1764 |
| GO:0035811 | negative regulation of urine volume | 5 | 1 | 0.19 | 0.1764 |
| GO:0035881 | amacrine cell differentiation | 5 | 1 | 0.19 | 0.1764 |
| GO:0035912 | dorsal aorta morphogenesis | 5 | 1 | 0.19 | 0.1764 |
| GO:0036490 | regulation of translation in response to... | 5 | 1 | 0.19 | 0.1764 |
| GO:0036491 | regulation of translation initiation in ... | 5 | 1 | 0.19 | 0.1764 |
| GO:0038089 | positive regulation of cell migration by... | 5 | 1 | 0.19 | 0.1764 |
| GO:0038110 | interleukin-2-mediated signaling pathway | 5 | 1 | 0.19 | 0.1764 |
| GO:0042045 | epithelial fluid transport | 5 | 1 | 0.19 | 0.1764 |
| GO:0042160 | lipoprotein modification | 5 | 1 | 0.19 | 0.1764 |
| GO:0042161 | lipoprotein oxidation | 5 | 1 | 0.19 | 0.1764 |
| GO:0042270 | protection from natural killer cell medi... | 5 | 1 | 0.19 | 0.1764 |
| GO:0042297 | vocal learning | 5 | 1 | 0.19 | 0.1764 |
| GO:0042518 | negative regulation of tyrosine phosphor... | 5 | 1 | 0.19 | 0.1764 |
| GO:0042795 | snRNA transcription from RNA polymerase | 5 | 1 | 0.19 | 0.1764 |
| GO:0043633 | polyadenylation-dependent RNA catabolic | 5 | 1 | 0.19 | 0.1764 |
| GO:0043970 | histone H3-K9 acetylation | 5 | 1 | 0.19 | 0.1764 |
| GO:0044340 | canonical Wnt signaling pathway involved. | 5 | 1 | 0.19 | 0.1764 |
| GO:0044829 | positive regulation by host of viral gen... | 5 | 1 | 0.19 | 0.1764 |
| GO:0045040 | protein import into mitochondrial outer ... | 5 | 1 | 0.19 | 0.1764 |
| GO:0045144 | meiotic sister chromatid segregation | 5 | 1 | 0.19 | 0.1764 |
| GO:0045472 | response to ether | 5 | 1 | 0.19 | 0.1764 |
| GO:0045991 | carbon catabolite activation of transcri... | 5 | 1 | 0.19 | 0.1764 |
| GO:0046469 | platelet activating factor metabolic pro... | 5 | 1 | 0.19 | 0.1764 |
| GO:0046878 | positive regulation of saliva secretion | 5 | 1 | 0.19 | 0.1764 |
| GO:0046880 | regulation of follicle-stimulating hormo... | 5 | 1 | 0.19 | 0.1764 |
| GO:0048341 | paraxial mesoderm formation | 5 | 1 | 0.19 | 0.1764 |
| GO:0048702 | embryonic neurocranium morphogenesis | 5 | 1 | 0.19 | 0.1764 |
| GO:0050861 | positive regulation of B cell receptor s... | 5 | 1 | 0.19 | 0.1764 |
| GO:0051029 | rRNA transport | 5 | 1 | 0.19 | 0.1764 |
| GO:0051136 | regulation of NK T cell differentiation | 5 | 1 | 0.19 | 0.1764 |
| GO:0051138 | positive regulation of NK T cell differe... | 5 | 1 | 0.19 | 0.1764 |
| GO:0051177 | meiotic sister chromatid cohesion | 5 | 1 | 0.19 | 0.1764 |
| GO:0051547 | regulation of keratinocyte migration | 5 | 1 | 0.19 | 0.1764 |
| GO:0051549 | positive regulation of keratinocyte migr... | 5 | 1 | 0.19 | 0.1764 |
| GO:0051561 | positive regulation of mitochondrial cal... | 5 | 1 | 0.19 | 0.1764 |
| GO:0060331 | negative regulation of response to inter... | 5 | 1 | 0.19 | 0.1764 |
| GO:0060336 | negative regulation of interferon-gamma-... | 5 | 1 | 0.19 | 0.1764 |
| GO:0060368 | regulation of Fc receptor mediated stimu... | 5 | 1 | 0.19 | 0.1764 |
| GO:0060385 | axonogenesis involved in innervation | 5 | 1 | 0.19 | 0.1764 |
| GO:0060431 | primary lung bud formation | 5 | 1 | 0.19 | 0.1764 |
| GO:0060665 | regulation of branching involved in sali... | 5 | 1 | 0.19 | 0.1764 |
| GO:0060729 | intestinal epithelial structure maintena... | 5 | 1 | 0.19 | 0.1764 |
| GO:0060750 | epithelial cell proliferation involved i... | 5 | 1 | 0.19 | 0.1764 |
| GO:0060842 | arterial endothelial cell differentiatio... | 5 | 1 | 0.19 | 0.1764 |

| | | | | | |
|---|---|---|---|---|---|
| GO:0061000 | negative regulation of dendritic spine d... | 5 | 1 | 0.19 | 0.1764 |
| GO:0070389 | chaperone cofactor-dependent protein ref. | 5 | 1 | 0.19 | 0.1764 |
| GO:0071352 | cellular response to interleukin-2 | 5 | 1 | 0.19 | 0.1764 |
| GO:0071374 | cellular response to parathyroid hormone.. | 5 | 1 | 0.19 | 0.1764 |
| GO:0071402 | cellular response to lipoprotein particl... | 5 | 1 | 0.19 | 0.1764 |
| GO:0072366 | regulation of cellular ketone metabolic ... | 5 | 1 | 0.19 | 0.1764 |
| GO:0072537 | fibroblast activation | 5 | 1 | 0.19 | 0.1764 |
| GO:0072611 | interleukin-13 secretion | 5 | 1 | 0.19 | 0.1764 |
| GO:0086023 | adrenergic receptor signaling pathway in... | 5 | 1 | 0.19 | 0.1764 |
| GO:0090394 | negative regulation of excitatory postsy... | 5 | 1 | 0.19 | 0.1764 |
| GO:0090500 | endocardial cushion to mesenchymal trans | 5 | 1 | 0.19 | 0.1764 |
| GO:0097114 | N-methyl-D-aspartate receptor clustering | 5 | 1 | 0.19 | 0.1764 |
| GO:0097459 | iron ion import into cell | 5 | 1 | 0.19 | 0.1764 |
| GO:0098596 | imitative learning | 5 | 1 | 0.19 | 0.1764 |
| GO:0098597 | observational learning | 5 | 1 | 0.19 | 0.1764 |
| GO:0098598 | learned vocalization behavior or vocal l... | 5 | 1 | 0.19 | 0.1764 |
| GO:1900133 | regulation of renin secretion into blood... | 5 | 1 | 0.19 | 0.1764 |
| GO:1901298 | regulation of hydrogen peroxide-mediated. | 5 | 1 | 0.19 | 0.1764 |
| GO:1901725 | regulation of histone deacetylase activi... | 5 | 1 | 0.19 | 0.1764 |
| GO:2000051 | negative regulation of non-canonical Wnt.. | 5 | 1 | 0.19 | 0.1764 |
| GO:2000402 | negative regulation of lymphocyte migrat... | 5 | 1 | 0.19 | 0.1764 |
| GO:2000630 | positive regulation of miRNA metabolic p... | 5 | 1 | 0.19 | 0.1764 |
| GO:2000665 | regulation of interleukin-13 secretion | 5 | 1 | 0.19 | 0.1764 |
| GO:2001275 | positive regulation of glucose import in... | 5 | 1 | 0.19 | 0.1764 |
| GO:0006310 | DNA recombination | 210 | 11 | 7.99 | 0.17813 |
| GO:0045732 | positive regulation of protein catabolic... | 210 | 11 | 7.99 | 0.17813 |
| GO:0060560 | developmental growth involved in morphog | 165 | 9 | 6.28 | 0.17815 |
| GO:0018108 | peptidyl-tyrosine phosphorylation | 302 | 15 | 11.5 | 0.17817 |
| GO:0042157 | lipoprotein metabolic process | 121 | 7 | 4.61 | 0.17833 |
| GO:0051092 | positive regulation of NF-kappaB transcr... | 121 | 7 | 4.61 | 0.17833 |
| GO:0050807 | regulation of synapse organization | 58 | 4 | 2.21 | 0.17877 |
| GO:0048592 | eye morphogenesis | 143 | 8 | 5.44 | 0.17891 |
| GO:1903708 | positive regulation of hemopoiesis | 143 | 8 | 5.44 | 0.17891 |
| GO:0015698 | inorganic anion transport | 211 | 11 | 8.03 | 0.18196 |
| GO:0009124 | nucleoside monophosphate biosynthetic pr | 79 | 5 | 3.01 | 0.18218 |
| GO:0016579 | protein deubiquitination | 79 | 5 | 3.01 | 0.18218 |
| GO:0042158 | lipoprotein biosynthetic process | 79 | 5 | 3.01 | 0.18218 |
| GO:0032409 | regulation of transporter activity | 166 | 9 | 6.32 | 0.1825 |
| GO:0010769 | regulation of cell morphogenesis involve... | 257 | 13 | 9.78 | 0.18257 |
| GO:0003205 | cardiac chamber development | 122 | 7 | 4.64 | 0.18347 |
| GO:0003018 | vascular process in circulatory system | 144 | 8 | 5.48 | 0.18362 |
| GO:0002437 | inflammatory response to antigenic stimu.. | 39 | 3 | 1.48 | 0.18454 |
| GO:0006891 | intra-Golgi vesicle-mediated transport | 39 | 3 | 1.48 | 0.18454 |
| GO:0010812 | negative regulation of cell-substrate ad... | 39 | 3 | 1.48 | 0.18454 |
| GO:0021795 | cerebral cortex cell migration | 39 | 3 | 1.48 | 0.18454 |
| GO:0035136 | forelimb morphogenesis | 39 | 3 | 1.48 | 0.18454 |
| GO:0051893 | regulation of focal adhesion assembly | 39 | 3 | 1.48 | 0.18454 |
| GO:0090109 | regulation of cell-substrate junction as... | 39 | 3 | 1.48 | 0.18454 |
| GO:0018212 | peptidyl-tyrosine modification | 304 | 15 | 11.57 | 0.18455 |
| GO:2001234 | negative regulation of apoptotic signali... | 189 | 10 | 7.19 | 0.18468 |
| GO:0040013 | negative regulation of locomotion | 212 | 11 | 8.07 | 0.18583 |
| GO:1901990 | regulation of mitotic cell cycle phase t... | 212 | 11 | 8.07 | 0.18583 |
| GO:0071902 | positive regulation of protein serine/th... | 258 | 13 | 9.82 | 0.18607 |

| | | | | | | |
|---|---|---|---|---|---|---|
| GO:0000737 | DNA catabolic process | endonuclec | 59 | 4 | 2.25 | |
| GO:0030593 | neutrophil chemotaxis | 59 | 4 | 2.25 | 0.18644 | |
| GO:0032104 | regulation of response to extracellular ... | 59 | 4 | 2.25 | 0.18644 | |
| GO:0032107 | regulation of response to nutrient level... | 59 | 4 | 2.25 | 0.18644 | |
| GO:0042147 | retrograde transport | endosome t | 59 | 4 | 2.25 | |
| GO:2001239 | regulation of extrinsic apoptotic signal... | 59 | 4 | 2.25 | 0.18644 | |
| GO:0006986 | response to unfolded protein | 145 | 8 | 5.52 | 0.18838 | |
| GO:0031099 | regeneration | 123 | 7 | 4.68 | 0.18867 | |
| GO:0071326 | cellular response to monosaccharide stim. | 80 | 5 | 3.05 | 0.18872 | |
| GO:0010837 | regulation of keratinocyte proliferation | 21 | 2 | 0.8 | 0.18945 | |
| GO:0031146 | SCF-dependent proteasomal ubiquitin-dep | 21 | 2 | 0.8 | 0.18945 | |
| GO:0032098 | regulation of appetite | 21 | 2 | 0.8 | 0.18945 | |
| GO:0035812 | renal sodium excretion | 21 | 2 | 0.8 | 0.18945 | |
| GO:0035813 | regulation of renal sodium excretion | 21 | 2 | 0.8 | 0.18945 | |
| GO:0035909 | aorta morphogenesis | 21 | 2 | 0.8 | 0.18945 | |
| GO:0045746 | negative regulation of Notch signaling p... | 21 | 2 | 0.8 | 0.18945 | |
| GO:0052312 | modulation of transcription in other org... | 21 | 2 | 0.8 | 0.18945 | |
| GO:0061037 | negative regulation of cartilage develop... | 21 | 2 | 0.8 | 0.18945 | |
| GO:0070841 | inclusion body assembly | 21 | 2 | 0.8 | 0.18945 | |
| GO:0021953 | central nervous system neuron differenti... | 168 | 9 | 6.4 | 0.19135 | |
| GO:2001235 | positive regulation of apoptotic signali... | 168 | 9 | 6.4 | 0.19135 | |
| GO:0045471 | response to ethanol | 102 | 6 | 3.88 | 0.19287 | |
| GO:0001101 | response to acid chemical | 260 | 13 | 9.9 | 0.19316 | |
| GO:0034284 | response to monosaccharide | 146 | 8 | 5.56 | 0.19319 | |
| GO:0046328 | regulation of JNK cascade | 146 | 8 | 5.56 | 0.19319 | |
| GO:0042180 | cellular ketone metabolic process | 237 | 12 | 9.02 | 0.19368 | |
| GO:0006633 | fatty acid biosynthetic process | 124 | 7 | 4.72 | 0.19392 | |
| GO:0015758 | glucose transport | 124 | 7 | 4.72 | 0.19392 | |
| GO:0042770 | signal transduction in response to DNA d... | 124 | 7 | 4.72 | 0.19392 | |
| GO:0001990 | regulation of systemic arterial blood pr... | 40 | 3 | 1.52 | 0.19427 | |
| GO:0002011 | morphogenesis of an epithelial sheet | 40 | 3 | 1.52 | 0.19427 | |
| GO:0007094 | mitotic spindle assembly checkpoint | 40 | 3 | 1.52 | 0.19427 | |
| GO:0021532 | neural tube patterning | 40 | 3 | 1.52 | 0.19427 | |
| GO:0032653 | regulation of interleukin-10 production | 40 | 3 | 1.52 | 0.19427 | |
| GO:1903391 | regulation of adherens junction organiza... | 40 | 3 | 1.52 | 0.19427 | |
| GO:0016236 | macroautophagy | 81 | 5 | 3.08 | 0.19534 | |
| GO:0060348 | bone development | 169 | 9 | 6.43 | 0.19584 | |
| GO:0010639 | negative regulation of organelle organiz... | 261 | 13 | 9.94 | 0.19676 | |
| GO:0042787 | protein ubiquitination involved in ubiqu... | 147 | 8 | 5.6 | 0.19805 | |
| GO:0003206 | cardiac chamber morphogenesis | 103 | 6 | 3.92 | 0.19875 | |
| GO:0015980 | energy derivation by oxidation of organi... | 332 | 16 | 12.64 | 0.19911 | |
| GO:0008645 | hexose transport | 125 | 7 | 4.76 | 0.19924 | |
| GO:0006112 | energy reserve metabolic process | 170 | 9 | 6.47 | 0.20038 | |
| GO:0032872 | regulation of stress-activated MAPK casc... | 170 | 9 | 6.47 | 0.20038 | |
| GO:0007006 | mitochondrial membrane organization | 82 | 5 | 3.12 | 0.20205 | |
| GO:0016575 | histone deacetylation | 61 | 4 | 2.32 | 0.20208 | |
| GO:0050803 | regulation of synapse structure or activ... | 61 | 4 | 2.32 | 0.20208 | |
| GO:0021697 | cerebellar cortex formation | 22 | 2 | 0.84 | 0.20346 | |
| GO:0032007 | negative regulation of TOR signaling | 22 | 2 | 0.84 | 0.20346 | |
| GO:0032106 | positive regulation of response to extra... | 22 | 2 | 0.84 | 0.20346 | |
| GO:0032109 | positive regulation of response to nutri... | 22 | 2 | 0.84 | 0.20346 | |
| GO:0035304 | regulation of protein dephosphorylation | 22 | 2 | 0.84 | 0.20346 | |
| GO:0044804 | nucleophagy | 22 | 2 | 0.84 | 0.20346 | |

| | | | | | |
|---|---|---|---|---|---|
| GO:0045939 | negative regulation of steroid metabolic... | 22 | 2 | 0.84 | 0.20346 |
| GO:0048103 | somatic stem cell division | 22 | 2 | 0.84 | 0.20346 |
| GO:0060317 | cardiac epithelial to mesenchymal transi... | 22 | 2 | 0.84 | 0.20346 |
| GO:0071354 | cellular response to interleukin-6 | 22 | 2 | 0.84 | 0.20346 |
| GO:0006833 | water transport | 41 | 3 | 1.56 | 0.20412 |
| GO:0045840 | positive regulation of mitotic nuclear d... | 41 | 3 | 1.56 | 0.20412 |
| GO:0048521 | negative regulation of behavior | 41 | 3 | 1.56 | 0.20412 |
| GO:0048701 | embryonic cranial skeleton morphogenesis | 41 | 3 | 1.56 | 0.20412 |
| GO:0060416 | response to growth hormone | 41 | 3 | 1.56 | 0.20412 |
| GO:0071173 | spindle assembly checkpoint | 41 | 3 | 1.56 | 0.20412 |
| GO:0050921 | positive regulation of chemotaxis | 104 | 6 | 3.96 | 0.20469 |
| GO:0007565 | female pregnancy | 171 | 9 | 6.51 | 0.20495 |
| GO:0051321 | meiotic cell cycle | 171 | 9 | 6.51 | 0.20495 |
| GO:0070302 | regulation of stress-activated protein k... | 171 | 9 | 6.51 | 0.20495 |
| GO:0060537 | muscle tissue development | 311 | 15 | 11.84 | 0.20772 |
| GO:0001842 | neural fold formation | 6 | 1 | 0.23 | 0.20776 |
| GO:0002001 | renin secretion into blood stream | 6 | 1 | 0.23 | 0.20776 |
| GO:0002420 | natural killer cell mediated cytotoxicit... | 6 | 1 | 0.23 | 0.20776 |
| GO:0002423 | natural killer cell mediated immune resp... | 6 | 1 | 0.23 | 0.20776 |
| GO:0002693 | positive regulation of cellular extravas... | 6 | 1 | 0.23 | 0.20776 |
| GO:0002765 | immune response-inhibiting signal transd.. | 6 | 1 | 0.23 | 0.20776 |
| GO:0002855 | regulation of natural killer cell mediat... | 6 | 1 | 0.23 | 0.20776 |
| GO:0002857 | positive regulation of natural killer ce... | 6 | 1 | 0.23 | 0.20776 |
| GO:0002858 | regulation of natural killer cell mediat... | 6 | 1 | 0.23 | 0.20776 |
| GO:0002860 | positive regulation of natural killer ce... | 6 | 1 | 0.23 | 0.20776 |
| GO:0003160 | endocardium morphogenesis | 6 | 1 | 0.23 | 0.20776 |
| GO:0006167 | AMP biosynthetic process | 6 | 1 | 0.23 | 0.20776 |
| GO:0006390 | transcription from mitochondrial promote... | 6 | 1 | 0.23 | 0.20776 |
| GO:0006498 | N-terminal protein lipidation | 6 | 1 | 0.23 | 0.20776 |
| GO:0007135 | meiosis II | 6 | 1 | 0.23 | 0.20776 |
| GO:0009157 | deoxyribonucleoside monophosphate bios | 6 | 1 | 0.23 | 0.20776 |
| GO:0009221 | pyrimidine deoxyribonucleotide biosynthe.. | 6 | 1 | 0.23 | 0.20776 |
| GO:0009624 | response to nematode | 6 | 1 | 0.23 | 0.20776 |
| GO:0014733 | regulation of skeletal muscle adaptation | 6 | 1 | 0.23 | 0.20776 |
| GO:0014816 | skeletal muscle satellite cell different... | 6 | 1 | 0.23 | 0.20776 |
| GO:0019509 | L-methionine biosynthetic process from m. | 6 | 1 | 0.23 | 0.20776 |
| GO:0019800 | peptide cross-linking via chondroitin 4-... | 6 | 1 | 0.23 | 0.20776 |
| GO:0021578 | hindbrain maturation | 6 | 1 | 0.23 | 0.20776 |
| GO:0021626 | central nervous system maturation | 6 | 1 | 0.23 | 0.20776 |
| GO:0031054 | pre-miRNA processing | 6 | 1 | 0.23 | 0.20776 |
| GO:0031133 | regulation of axon diameter | 6 | 1 | 0.23 | 0.20776 |
| GO:0032275 | luteinizing hormone secretion | 6 | 1 | 0.23 | 0.20776 |
| GO:0032344 | regulation of aldosterone metabolic proc... | 6 | 1 | 0.23 | 0.20776 |
| GO:0032347 | regulation of aldosterone biosynthetic p... | 6 | 1 | 0.23 | 0.20776 |
| GO:0032530 | regulation of microvillus organization | 6 | 1 | 0.23 | 0.20776 |
| GO:0032714 | negative regulation of interleukin-5 pro... | 6 | 1 | 0.23 | 0.20776 |
| GO:0032762 | mast cell cytokine production | 6 | 1 | 0.23 | 0.20776 |
| GO:0032808 | lacrimal gland development | 6 | 1 | 0.23 | 0.20776 |
| GO:0033210 | leptin-mediated signaling pathway | 6 | 1 | 0.23 | 0.20776 |
| GO:0034036 | purine ribonucleoside bisphosphate biosy.. | 6 | 1 | 0.23 | 0.20776 |
| GO:0035865 | cellular response to potassium ion | 6 | 1 | 0.23 | 0.20776 |
| GO:0035907 | dorsal aorta development | 6 | 1 | 0.23 | 0.20776 |
| GO:0035931 | mineralocorticoid secretion | 6 | 1 | 0.23 | 0.20776 |

| | | | | | |
|---|---|---|---|---|---|
| GO:0035932 | aldosterone secretion | 6 | 1 | 0.23 | 0.20776 |
| GO:0035947 | regulation of gluconeogenesis by regulat... | 6 | 1 | 0.23 | 0.20776 |
| GO:0042532 | negative regulation of tyrosine phosphor... | 6 | 1 | 0.23 | 0.20776 |
| GO:0042791 | 5S class rRNA transcription from RNA pol. | 6 | 1 | 0.23 | 0.20776 |
| GO:0042797 | tRNA transcription from RNA polymerase I | 6 | 1 | 0.23 | 0.20776 |
| GO:0043382 | positive regulation of memory T cell dif... | 6 | 1 | 0.23 | 0.20776 |
| GO:0043615 | astrocyte cell migration | 6 | 1 | 0.23 | 0.20776 |
| GO:0044332 | Wnt signaling pathway involved in dorsal... | 6 | 1 | 0.23 | 0.20776 |
| GO:0045475 | locomotor rhythm | 6 | 1 | 0.23 | 0.20776 |
| GO:0045591 | positive regulation of regulatory T cell... | 6 | 1 | 0.23 | 0.20776 |
| GO:0045898 | regulation of RNA polymerase II transcri... | 6 | 1 | 0.23 | 0.20776 |
| GO:0046884 | follicle-stimulating hormone secretion | 6 | 1 | 0.23 | 0.20776 |
| GO:0050428 | 3'-phosphoadenosine 5'-phosphosulfate bi | 6 | 1 | 0.23 | 0.20776 |
| GO:0060054 | positive regulation of epithelial cell p... | 6 | 1 | 0.23 | 0.20776 |
| GO:0060149 | negative regulation of posttranscription... | 6 | 1 | 0.23 | 0.20776 |
| GO:0060159 | regulation of dopamine receptor signalin... | 6 | 1 | 0.23 | 0.20776 |
| GO:0060287 | epithelial cilium movement involved in d... | 6 | 1 | 0.23 | 0.20776 |
| GO:0060449 | bud elongation involved in lung branchin... | 6 | 1 | 0.23 | 0.20776 |
| GO:0060534 | trachea cartilage development | 6 | 1 | 0.23 | 0.20776 |
| GO:0060700 | regulation of ribonuclease activity | 6 | 1 | 0.23 | 0.20776 |
| GO:0060751 | branch elongation involved in mammary gl | 6 | 1 | 0.23 | 0.20776 |
| GO:0060836 | lymphatic endothelial cell differentiati... | 6 | 1 | 0.23 | 0.20776 |
| GO:0060839 | endothelial cell fate commitment | 6 | 1 | 0.23 | 0.20776 |
| GO:0060856 | establishment of blood-brain barrier | 6 | 1 | 0.23 | 0.20776 |
| GO:0060967 | negative regulation of gene silencing by... | 6 | 1 | 0.23 | 0.20776 |
| GO:0061050 | regulation of cell growth involved in ca... | 6 | 1 | 0.23 | 0.20776 |
| GO:0061087 | positive regulation of histone H3-K27 me... | 6 | 1 | 0.23 | 0.20776 |
| GO:0061316 | canonical Wnt signaling pathway involved. | 6 | 1 | 0.23 | 0.20776 |
| GO:0070092 | regulation of glucagon secretion | 6 | 1 | 0.23 | 0.20776 |
| GO:0071918 | urea transmembrane transport | 6 | 1 | 0.23 | 0.20776 |
| GO:0071985 | multivesicular body sorting pathway | 6 | 1 | 0.23 | 0.20776 |
| GO:0072383 | plus-end-directed vesicle transport alon... | 6 | 1 | 0.23 | 0.20776 |
| GO:0072386 | plus-end-directed organelle transport al... | 6 | 1 | 0.23 | 0.20776 |
| GO:0086042 | cardiac muscle cell-cardiac muscle cell ... | 6 | 1 | 0.23 | 0.20776 |
| GO:0086073 | bundle of His cell-Purkinje myocyte adhe... | 6 | 1 | 0.23 | 0.20776 |
| GO:0090031 | positive regulation of steroid hormone b... | 6 | 1 | 0.23 | 0.20776 |
| GO:0090045 | positive regulation of deacetylase activ... | 6 | 1 | 0.23 | 0.20776 |
| GO:0090245 | axis elongation involved in somitogenesi... | 6 | 1 | 0.23 | 0.20776 |
| GO:0090267 | positive regulation of mitotic cell cycl... | 6 | 1 | 0.23 | 0.20776 |
| GO:0097104 | postsynaptic membrane assembly | 6 | 1 | 0.23 | 0.20776 |
| GO:0097201 | negative regulation of transcription fro... | 6 | 1 | 0.23 | 0.20776 |
| GO:1901029 | negative regulation of mitochondrial out... | 6 | 1 | 0.23 | 0.20776 |
| GO:1901620 | regulation of smoothened signaling pathw. | 6 | 1 | 0.23 | 0.20776 |
| GO:1901621 | negative regulation of smoothened signal.. | 6 | 1 | 0.23 | 0.20776 |
| GO:1902177 | positive regulation of oxidative stress-... | 6 | 1 | 0.23 | 0.20776 |
| GO:1902338 | negative regulation of apoptotic process... | 6 | 1 | 0.23 | 0.20776 |
| GO:1903224 | regulation of endodermal cell differenti... | 6 | 1 | 0.23 | 0.20776 |
| GO:2000271 | positive regulation of fibroblast apopto... | 6 | 1 | 0.23 | 0.20776 |
| GO:2000427 | positive regulation of apoptotic cell cl... | 6 | 1 | 0.23 | 0.20776 |
| GO:2000501 | regulation of natural killer cell chemot... | 6 | 1 | 0.23 | 0.20776 |
| GO:2001269 | positive regulation of cysteine-type end... | 6 | 1 | 0.23 | 0.20776 |
| GO:0009267 | cellular response to starvation | 149 | 8 | 5.67 | 0.20792 |
| GO:0006368 | transcription elongation from RNA polyme. | 83 | 5 | 3.16 | 0.20883 |

| | | | | | |
|---|---|---|---|---|---|
| GO:0014902 | myotube differentiation | 83 | 5 | 3.16 | 0.20883 |
| GO:0051053 | negative regulation of DNA metabolic pro.. | 83 | 5 | 3.16 | 0.20883 |
| GO:1902476 | chloride transmembrane transport | 83 | 5 | 3.16 | 0.20883 |
| GO:0010948 | negative regulation of cell cycle proces... | 218 | 11 | 8.3 | 0.20983 |
| GO:0015749 | monosaccharide transport | 127 | 7 | 4.83 | 0.21003 |
| GO:0050657 | nucleic acid transport | 127 | 7 | 4.83 | 0.21003 |
| GO:0050658 | RNA transport | 127 | 7 | 4.83 | 0.21003 |
| GO:0050880 | regulation of blood vessel size | 127 | 7 | 4.83 | 0.21003 |
| GO:0051236 | establishment of RNA localization | 127 | 7 | 4.83 | 0.21003 |
| GO:1990266 | neutrophil migration | 62 | 4 | 2.36 | 0.21005 |
| GO:0043406 | positive regulation of MAP kinase activi... | 195 | 10 | 7.42 | 0.2101 |
| GO:0016055 | Wnt signaling pathway | 383 | 18 | 14.58 | 0.21013 |
| GO:0046330 | positive regulation of JNK cascade | 105 | 6 | 4 | 0.2107 |
| GO:0090101 | negative regulation of transmembrane rec. | 105 | 6 | 4 | 0.2107 |
| GO:0007618 | mating | 42 | 3 | 1.6 | 0.21409 |
| GO:0009066 | aspartate family amino acid metabolic pr... | 42 | 3 | 1.6 | 0.21409 |
| GO:0010332 | response to gamma radiation | 42 | 3 | 1.6 | 0.21409 |
| GO:0010464 | regulation of mesenchymal cell prolifera... | 42 | 3 | 1.6 | 0.21409 |
| GO:0016925 | protein sumoylation | 42 | 3 | 1.6 | 0.21409 |
| GO:0032613 | interleukin-10 production | 42 | 3 | 1.6 | 0.21409 |
| GO:0044272 | sulfur compound biosynthetic process | 173 | 9 | 6.59 | 0.21424 |
| GO:0001819 | positive regulation of cytokine producti... | 337 | 16 | 12.83 | 0.21548 |
| GO:0007088 | regulation of mitotic nuclear division | 128 | 7 | 4.87 | 0.21551 |
| GO:0035150 | regulation of tube size | 128 | 7 | 4.87 | 0.21551 |
| GO:0034111 | negative regulation of homotypic cell-ce... | 84 | 5 | 3.2 | 0.21569 |
| GO:2000060 | positive regulation of protein ubiquitin... | 84 | 5 | 3.2 | 0.21569 |
| GO:0003014 | renal system process | 106 | 6 | 4.03 | 0.21678 |
| GO:0030968 | endoplasmic reticulum unfolded protein r... | 106 | 6 | 4.03 | 0.21678 |
| GO:0048593 | camera-type eye morphogenesis | 106 | 6 | 4.03 | 0.21678 |
| GO:0001964 | startle response | 23 | 2 | 0.88 | 0.21758 |
| GO:0002063 | chondrocyte development | 23 | 2 | 0.88 | 0.21758 |
| GO:0006984 | ER-nucleus signaling pathway | 23 | 2 | 0.88 | 0.21758 |
| GO:0010656 | negative regulation of muscle cell apopt... | 23 | 2 | 0.88 | 0.21758 |
| GO:0030539 | male genitalia development | 23 | 2 | 0.88 | 0.21758 |
| GO:0032897 | negative regulation of viral transcripti... | 23 | 2 | 0.88 | 0.21758 |
| GO:0033687 | osteoblast proliferation | 23 | 2 | 0.88 | 0.21758 |
| GO:0035336 | long-chain fatty-acyl-CoA metabolic proc... | 23 | 2 | 0.88 | 0.21758 |
| GO:0043043 | peptide biosynthetic process | 23 | 2 | 0.88 | 0.21758 |
| GO:0043567 | regulation of insulin-like growth factor... | 23 | 2 | 0.88 | 0.21758 |
| GO:0045987 | positive regulation of smooth muscle con... | 23 | 2 | 0.88 | 0.21758 |
| GO:0051965 | positive regulation of synapse assembly | 23 | 2 | 0.88 | 0.21758 |
| GO:0061384 | heart trabecula morphogenesis | 23 | 2 | 0.88 | 0.21758 |
| GO:0072488 | ammonium transmembrane transport | 23 | 2 | 0.88 | 0.21758 |
| GO:2000142 | regulation of DNA-templated transcriptio... | 23 | 2 | 0.88 | 0.21758 |
| GO:2000772 | regulation of cellular senescence | 23 | 2 | 0.88 | 0.21758 |
| GO:0030279 | negative regulation of ossification | 63 | 4 | 2.4 | 0.2181 |
| GO:0009612 | response to mechanical stimulus | 174 | 9 | 6.62 | 0.21894 |
| GO:0033500 | carbohydrate homeostasis | 174 | 9 | 6.62 | 0.21894 |
| GO:0042593 | glucose homeostasis | 174 | 9 | 6.62 | 0.21894 |
| GO:0070482 | response to oxygen levels | 267 | 13 | 10.16 | 0.21895 |
| GO:0097191 | extrinsic apoptotic signaling pathway | 221 | 11 | 8.41 | 0.22231 |
| GO:0010771 | negative regulation of cell morphogenesi... | 85 | 5 | 3.24 | 0.22261 |
| GO:0002237 | response to molecule of bacterial origin | 268 | 13 | 10.2 | 0.22275 |

| | | | | | |
|---|---|---|---|---|---|
| GO:0051028 | mRNA transport | 107 | 6 | 4.07 | 0.22291 |
| GO:0051783 | regulation of nuclear division | 152 | 8 | 5.79 | 0.22308 |
| GO:0003281 | ventricular septum development | 43 | 3 | 1.64 | 0.22415 |
| GO:0003300 | cardiac muscle hypertrophy | 43 | 3 | 1.64 | 0.22415 |
| GO:0008206 | bile acid metabolic process | 43 | 3 | 1.64 | 0.22415 |
| GO:0030219 | megakaryocyte differentiation | 43 | 3 | 1.64 | 0.22415 |
| GO:0033046 | negative regulation of sister chromatid ... | 43 | 3 | 1.64 | 0.22415 |
| GO:0033048 | negative regulation of mitotic sister ch... | 43 | 3 | 1.64 | 0.22415 |
| GO:0042059 | negative regulation of epidermal growth ... | 43 | 3 | 1.64 | 0.22415 |
| GO:0043392 | negative regulation of DNA binding | 43 | 3 | 1.64 | 0.22415 |
| GO:0045841 | negative regulation of mitotic metaphase... | 43 | 3 | 1.64 | 0.22415 |
| GO:0046677 | response to antibiotic | 43 | 3 | 1.64 | 0.22415 |
| GO:0051668 | localization within membrane | 43 | 3 | 1.64 | 0.22415 |
| GO:0055078 | sodium ion homeostasis | 43 | 3 | 1.64 | 0.22415 |
| GO:0071174 | mitotic spindle checkpoint | 43 | 3 | 1.64 | 0.22415 |
| GO:0071806 | protein transmembrane transport | 43 | 3 | 1.64 | 0.22415 |
| GO:1900543 | negative regulation of purine nucleotide... | 43 | 3 | 1.64 | 0.22415 |
| GO:2000816 | negative regulation of mitotic sister ch... | 43 | 3 | 1.64 | 0.22415 |
| GO:0090596 | sensory organ morphogenesis | 245 | 12 | 9.33 | 0.2248 |
| GO:0009064 | glutamine family amino acid metabolic pr... | 64 | 4 | 2.44 | 0.22624 |
| GO:0090068 | positive regulation of cell cycle proces... | 222 | 11 | 8.45 | 0.22653 |
| GO:0016053 | organic acid biosynthetic process | 269 | 13 | 10.24 | 0.22658 |
| GO:0046394 | carboxylic acid biosynthetic process | 269 | 13 | 10.24 | 0.22658 |
| GO:0007605 | sensory perception of sound | 130 | 7 | 4.95 | 0.22662 |
| GO:0010498 | proteasomal protein catabolic process | 365 | 17 | 13.89 | 0.22911 |
| GO:1903825 | organic acid transmembrane transport | 86 | 5 | 3.27 | 0.2296 |
| GO:0048864 | stem cell development | 270 | 13 | 10.28 | 0.23044 |
| GO:0001953 | negative regulation of cell-matrix adhes... | 24 | 2 | 0.91 | 0.23178 |
| GO:0002686 | negative regulation of leukocyte migrati... | 24 | 2 | 0.91 | 0.23178 |
| GO:0007214 | gamma-aminobutyric acid signaling pathw | 24 | 2 | 0.91 | 0.23178 |
| GO:0021871 | forebrain regionalization | 24 | 2 | 0.91 | 0.23178 |
| GO:0032733 | positive regulation of interleukin-10 pr... | 24 | 2 | 0.91 | 0.23178 |
| GO:0032800 | receptor biosynthetic process | 24 | 2 | 0.91 | 0.23178 |
| GO:0035235 | ionotropic glutamate receptor signaling ... | 24 | 2 | 0.91 | 0.23178 |
| GO:0035904 | aorta development | 24 | 2 | 0.91 | 0.23178 |
| GO:0036065 | fucosylation | 24 | 2 | 0.91 | 0.23178 |
| GO:0042554 | superoxide anion generation | 24 | 2 | 0.91 | 0.23178 |
| GO:0044062 | regulation of excretion | 24 | 2 | 0.91 | 0.23178 |
| GO:0046320 | regulation of fatty acid oxidation | 24 | 2 | 0.91 | 0.23178 |
| GO:0050892 | intestinal absorption | 24 | 2 | 0.91 | 0.23178 |
| GO:0061311 | cell surface receptor signaling pathway ... | 24 | 2 | 0.91 | 0.23178 |
| GO:1901797 | negative regulation of signal transducti... | 24 | 2 | 0.91 | 0.23178 |
| GO:1902235 | regulation of endoplasmic reticulum stre... | 24 | 2 | 0.91 | 0.23178 |
| GO:0051216 | cartilage development | 177 | 9 | 6.74 | 0.23329 |
| GO:0008016 | regulation of heart contraction | 154 | 8 | 5.86 | 0.23341 |
| GO:0035966 | response to topologically incorrect prot... | 154 | 8 | 5.86 | 0.23341 |
| GO:0065004 | protein-DNA complex assembly | 154 | 8 | 5.86 | 0.23341 |
| GO:0014897 | striated muscle hypertrophy | 44 | 3 | 1.67 | 0.23431 |
| GO:0032890 | regulation of organic acid transport | 44 | 3 | 1.67 | 0.23431 |
| GO:0043550 | regulation of lipid kinase activity | 44 | 3 | 1.67 | 0.23431 |
| GO:0044786 | cell cycle DNA replication | 44 | 3 | 1.67 | 0.23431 |
| GO:1901185 | negative regulation of ERBB signaling pa.. | 44 | 3 | 1.67 | 0.23431 |
| GO:1902041 | regulation of extrinsic apoptotic signal... | 44 | 3 | 1.67 | 0.23431 |

| | | | | | |
|---|---|---|---|---|---|
| GO:1902100 | negative regulation of metaphase/anaphas | 44 | 3 | 1.67 | 0.23431 |
| GO:0043044 | ATP-dependent chromatin remodeling | 65 | 4 | 2.47 | 0.23445 |
| GO:0070227 | lymphocyte apoptotic process | 65 | 4 | 2.47 | 0.23445 |
| GO:0006936 | muscle contraction | 295 | 14 | 11.23 | 0.23523 |
| GO:0030330 | DNA damage response | signal trans | 109 | 6 | 4.15 |
| GO:0034620 | cellular response to unfolded protein | 109 | 6 | 4.15 | 0.23533 |
| GO:0001525 | angiogenesis | 391 | 18 | 14.88 | 0.23542 |
| GO:0090066 | regulation of anatomical structure size | 367 | 17 | 13.97 | 0.23576 |
| GO:0002687 | positive regulation of leukocyte migrati... | 87 | 5 | 3.31 | 0.23666 |
| GO:0010469 | regulation of receptor activity | 87 | 5 | 3.31 | 0.23666 |
| GO:0042698 | ovulation cycle | 87 | 5 | 3.31 | 0.23666 |
| GO:0060333 | interferon-gamma-mediated signaling path | 87 | 5 | 3.31 | 0.23666 |
| GO:0022618 | ribonucleoprotein complex assembly | 132 | 7 | 5.02 | 0.23792 |
| GO:0051899 | membrane depolarization | 132 | 7 | 5.02 | 0.23792 |
| GO:0001574 | ganglioside biosynthetic process | 7 | 1 | 0.27 | 0.23793 |
| GO:0001865 | NK T cell differentiation | 7 | 1 | 0.27 | 0.23793 |
| GO:0001999 | renal response to blood flow involved in... | 7 | 1 | 0.27 | 0.23793 |
| GO:0002024 | diet induced thermogenesis | 7 | 1 | 0.27 | 0.23793 |
| GO:0002138 | retinoic acid biosynthetic process | 7 | 1 | 0.27 | 0.23793 |
| GO:0002883 | regulation of hypersensitivity | 7 | 1 | 0.27 | 0.23793 |
| GO:0002887 | negative regulation of myeloid leukocyte... | 7 | 1 | 0.27 | 0.23793 |
| GO:0003057 | regulation of the force of heart contrac... | 7 | 1 | 0.27 | 0.23793 |
| GO:0003062 | regulation of heart rate by chemical sig... | 7 | 1 | 0.27 | 0.23793 |
| GO:0003190 | atrioventricular valve formation | 7 | 1 | 0.27 | 0.23793 |
| GO:0003307 | regulation of Wnt signaling pathway invo... | 7 | 1 | 0.27 | 0.23793 |
| GO:0005981 | regulation of glycogen catabolic process | 7 | 1 | 0.27 | 0.23793 |
| GO:0006285 | base-excision repair | AP site forn | 7 | 1 | 0.27 |
| GO:0006384 | transcription initiation from RNA polyme... | 7 | 1 | 0.27 | 0.23793 |
| GO:0007185 | transmembrane receptor protein tyrosine .. | 7 | 1 | 0.27 | 0.23793 |
| GO:0009223 | pyrimidine deoxyribonucleotide catabolic... | 7 | 1 | 0.27 | 0.23793 |
| GO:0009304 | tRNA transcription | 7 | 1 | 0.27 | 0.23793 |
| GO:0010421 | hydrogen peroxide-mediated programmed | 7 | 1 | 0.27 | 0.23793 |
| GO:0010566 | regulation of ketone biosynthetic proces... | 7 | 1 | 0.27 | 0.23793 |
| GO:0010603 | regulation of cytoplasmic mRNA processin | 7 | 1 | 0.27 | 0.23793 |
| GO:0010759 | positive regulation of macrophage chemot. | 7 | 1 | 0.27 | 0.23793 |
| GO:0014819 | regulation of skeletal muscle contractio... | 7 | 1 | 0.27 | 0.23793 |
| GO:0014824 | artery smooth muscle contraction | 7 | 1 | 0.27 | 0.23793 |
| GO:0015677 | copper ion import | 7 | 1 | 0.27 | 0.23793 |
| GO:0015808 | L-alanine transport | 7 | 1 | 0.27 | 0.23793 |
| GO:0015840 | urea transport | 7 | 1 | 0.27 | 0.23793 |
| GO:0016102 | diterpenoid biosynthetic process | 7 | 1 | 0.27 | 0.23793 |
| GO:0016255 | attachment of GPI anchor to protein | 7 | 1 | 0.27 | 0.23793 |
| GO:0016553 | base conversion or substitution editing | 7 | 1 | 0.27 | 0.23793 |
| GO:0021999 | neural plate anterior/posterior regional... | 7 | 1 | 0.27 | 0.23793 |
| GO:0030300 | regulation of intestinal cholesterol abs... | 7 | 1 | 0.27 | 0.23793 |
| GO:0030913 | paranodal junction assembly | 7 | 1 | 0.27 | 0.23793 |
| GO:0031033 | myosin filament organization | 7 | 1 | 0.27 | 0.23793 |
| GO:0031167 | rRNA methylation | 7 | 1 | 0.27 | 0.23793 |
| GO:0032754 | positive regulation of interleukin-5 pro... | 7 | 1 | 0.27 | 0.23793 |
| GO:0033004 | negative regulation of mast cell activat... | 7 | 1 | 0.27 | 0.23793 |
| GO:0033689 | negative regulation of osteoblast prolif... | 7 | 1 | 0.27 | 0.23793 |
| GO:0033860 | regulation of NAD(P)H oxidase activity | 7 | 1 | 0.27 | 0.23793 |
| GO:0034145 | positive regulation of toll-like recepto... | 7 | 1 | 0.27 | 0.23793 |

| | | | | | |
|---|---|---|---|---|---|
| GO:0034350 | regulation of glial cell apoptotic proce... | 7 | 1 | 0.27 | 0.23793 |
| GO:0034351 | negative regulation of glial cell apopto... | 7 | 1 | 0.27 | 0.23793 |
| GO:0034497 | protein localization to pre-autophagosom... | 7 | 1 | 0.27 | 0.23793 |
| GO:0034975 | protein folding in endoplasmic reticulum | 7 | 1 | 0.27 | 0.23793 |
| GO:0034983 | peptidyl-lysine deacetylation | 7 | 1 | 0.27 | 0.23793 |
| GO:0035405 | histone-threonine phosphorylation | 7 | 1 | 0.27 | 0.23793 |
| GO:0035791 | platelet-derived growth factor receptor-... | 7 | 1 | 0.27 | 0.23793 |
| GO:0042048 | olfactory behavior | 7 | 1 | 0.27 | 0.23793 |
| GO:0042473 | outer ear morphogenesis | 7 | 1 | 0.27 | 0.23793 |
| GO:0043301 | negative regulation of leukocyte degranu... | 7 | 1 | 0.27 | 0.23793 |
| GO:0043379 | memory T cell differentiation | 7 | 1 | 0.27 | 0.23793 |
| GO:0043380 | regulation of memory T cell differentiat... | 7 | 1 | 0.27 | 0.23793 |
| GO:0044341 | sodium-dependent phosphate transport | 7 | 1 | 0.27 | 0.23793 |
| GO:0044793 | negative regulation by host of viral pro... | 7 | 1 | 0.27 | 0.23793 |
| GO:0044794 | positive regulation by host of viral pro... | 7 | 1 | 0.27 | 0.23793 |
| GO:0044827 | modulation by host of viral genome repli... | 7 | 1 | 0.27 | 0.23793 |
| GO:0045617 | negative regulation of keratinocyte diff... | 7 | 1 | 0.27 | 0.23793 |
| GO:0045654 | positive regulation of megakaryocyte dif... | 7 | 1 | 0.27 | 0.23793 |
| GO:0045820 | negative regulation of glycolytic proces... | 7 | 1 | 0.27 | 0.23793 |
| GO:0045990 | carbon catabolite regulation of transcri... | 7 | 1 | 0.27 | 0.23793 |
| GO:0046349 | amino sugar biosynthetic process | 7 | 1 | 0.27 | 0.23793 |
| GO:0046877 | regulation of saliva secretion | 7 | 1 | 0.27 | 0.23793 |
| GO:0048003 | antigen processing and presentation of l... | 7 | 1 | 0.27 | 0.23793 |
| GO:0048007 | antigen processing and presentation exo... | | 7 | 1 | 0.27 |
| GO:0048671 | negative regulation of collateral sprout... | 7 | 1 | 0.27 | 0.23793 |
| GO:0048733 | sebaceous gland development | 7 | 1 | 0.27 | 0.23793 |
| GO:0048755 | branching morphogenesis of a nerve | 7 | 1 | 0.27 | 0.23793 |
| GO:0051488 | activation of anaphase-promoting complex | 7 | 1 | 0.27 | 0.23793 |
| GO:0060019 | radial glial cell differentiation | 7 | 1 | 0.27 | 0.23793 |
| GO:0060087 | relaxation of vascular smooth muscle | 7 | 1 | 0.27 | 0.23793 |
| GO:0060297 | regulation of sarcomere organization | 7 | 1 | 0.27 | 0.23793 |
| GO:0060526 | prostate glandular acinus morphogenesis | 7 | 1 | 0.27 | 0.23793 |
| GO:0060527 | prostate epithelial cord arborization in... | 7 | 1 | 0.27 | 0.23793 |
| GO:0060631 | regulation of meiosis I | 7 | 1 | 0.27 | 0.23793 |
| GO:0060687 | regulation of branching involved in pros... | 7 | 1 | 0.27 | 0.23793 |
| GO:0060837 | blood vessel endothelial cell differenti... | 7 | 1 | 0.27 | 0.23793 |
| GO:0061157 | mRNA destabilization | 7 | 1 | 0.27 | 0.23793 |
| GO:0061307 | cardiac neural crest cell differentiatio... | 7 | 1 | 0.27 | 0.23793 |
| GO:0070091 | glucagon secretion | 7 | 1 | 0.27 | 0.23793 |
| GO:0070857 | regulation of bile acid biosynthetic pro... | 7 | 1 | 0.27 | 0.23793 |
| GO:0071420 | cellular response to histamine | 7 | 1 | 0.27 | 0.23793 |
| GO:0071476 | cellular hypotonic response | 7 | 1 | 0.27 | 0.23793 |
| GO:0090232 | positive regulation of spindle checkpoin... | 7 | 1 | 0.27 | 0.23793 |
| GO:0090244 | Wnt signaling pathway involved in somito.. | 7 | 1 | 0.27 | 0.23793 |
| GO:0090331 | negative regulation of platelet aggregat... | 7 | 1 | 0.27 | 0.23793 |
| GO:0097119 | postsynaptic density protein 95 clusteri... | 7 | 1 | 0.27 | 0.23793 |
| GO:0097468 | programmed cell death in response to rea. | 7 | 1 | 0.27 | 0.23793 |
| GO:1900119 | positive regulation of execution phase o... | 7 | 1 | 0.27 | 0.23793 |
| GO:1902231 | positive regulation of intrinsic apoptot... | 7 | 1 | 0.27 | 0.23793 |
| GO:1903306 | negative regulation of regulated secreto... | 7 | 1 | 0.27 | 0.23793 |
| GO:2000848 | positive regulation of corticosteroid ho... | 7 | 1 | 0.27 | 0.23793 |
| GO:2001138 | regulation of phospholipid transport | 7 | 1 | 0.27 | 0.23793 |
| GO:2001140 | positive regulation of phospholipid tran... | 7 | 1 | 0.27 | 0.23793 |

| | | | | | |
|---|---|---|---|---|---|
| GO:0043405 | regulation of MAP kinase activity | 272 | 13 | 10.35 | 0.23822 |
| GO:0014706 | striated muscle tissue development | 296 | 14 | 11.27 | 0.23897 |
| GO:0015850 | organic hydroxy compound transport | 202 | 10 | 7.69 | 0.24146 |
| GO:0002690 | positive regulation of leukocyte chemota... | 66 | 4 | 2.51 | 0.24273 |
| GO:0003279 | cardiac septum development | 66 | 4 | 2.51 | 0.24273 |
| GO:0060359 | response to ammonium ion | 66 | 4 | 2.51 | 0.24273 |
| GO:0003012 | muscle system process | 345 | 16 | 13.13 | 0.24285 |
| GO:0071824 | protein-DNA complex subunit organization | 179 | 9 | 6.81 | 0.24304 |
| GO:0007156 | homophilic cell adhesion via plasma memb | 133 | 7 | 5.06 | 0.24364 |
| GO:1901987 | regulation of cell cycle phase transitio... | 226 | 11 | 8.6 | 0.24373 |
| GO:0003229 | ventricular cardiac muscle tissue develo... | 45 | 3 | 1.71 | 0.24454 |
| GO:0003407 | neural retina development | 45 | 3 | 1.71 | 0.24454 |
| GO:0009948 | anterior/posterior axis specification | 45 | 3 | 1.71 | 0.24454 |
| GO:0016126 | sterol biosynthetic process | 45 | 3 | 1.71 | 0.24454 |
| GO:0018208 | peptidyl-proline modification | 45 | 3 | 1.71 | 0.24454 |
| GO:0035567 | non-canonical Wnt signaling pathway | 45 | 3 | 1.71 | 0.24454 |
| GO:0061077 | chaperone-mediated protein folding | 45 | 3 | 1.71 | 0.24454 |
| GO:0071695 | anatomical structure maturation | 45 | 3 | 1.71 | 0.24454 |
| GO:0008015 | blood circulation | 394 | 18 | 15 | 0.24523 |
| GO:0006623 | protein targeting to vacuole | 25 | 2 | 0.95 | 0.24604 |
| GO:0021533 | cell differentiation in hindbrain | 25 | 2 | 0.95 | 0.24604 |
| GO:0021591 | ventricular system development | 25 | 2 | 0.95 | 0.24604 |
| GO:0021904 | dorsal/ventral neural tube patterning | 25 | 2 | 0.95 | 0.24604 |
| GO:0033198 | response to ATP | 25 | 2 | 0.95 | 0.24604 |
| GO:0043278 | response to morphine | 25 | 2 | 0.95 | 0.24604 |
| GO:0045070 | positive regulation of viral genome repl... | 25 | 2 | 0.95 | 0.24604 |
| GO:0051930 | regulation of sensory perception of pain | 25 | 2 | 0.95 | 0.24604 |
| GO:0051931 | regulation of sensory perception | 25 | 2 | 0.95 | 0.24604 |
| GO:0072583 | clathrin-mediated endocytosis | 25 | 2 | 0.95 | 0.24604 |
| GO:0086091 | regulation of heart rate by cardiac cond... | 25 | 2 | 0.95 | 0.24604 |
| GO:1900026 | positive regulation of substrate adhesio... | 25 | 2 | 0.95 | 0.24604 |
| GO:1902042 | negative regulation of extrinsic apoptot... | 25 | 2 | 0.95 | 0.24604 |
| GO:2000191 | regulation of fatty acid transport | 25 | 2 | 0.95 | 0.24604 |
| GO:0006006 | glucose metabolic process | 203 | 10 | 7.73 | 0.24607 |
| GO:0007626 | locomotory behavior | 180 | 9 | 6.85 | 0.24797 |
| GO:0003013 | circulatory system process | 395 | 18 | 15.04 | 0.24853 |
| GO:0006643 | membrane lipid metabolic process | 157 | 8 | 5.98 | 0.2492 |
| GO:0036293 | response to decreased oxygen levels | 251 | 12 | 9.55 | 0.24938 |
| GO:0007126 | meiotic nuclear division | 134 | 7 | 5.1 | 0.2494 |
| GO:0043648 | dicarboxylic acid metabolic process | 89 | 5 | 3.39 | 0.25094 |
| GO:0070252 | actin-mediated cell contraction | 89 | 5 | 3.39 | 0.25094 |
| GO:1903902 | positive regulation of viral life cycle | 89 | 5 | 3.39 | 0.25094 |
| GO:0006488 | dolichol-linked oligosaccharide biosynth... | 67 | 4 | 2.55 | 0.25107 |
| GO:0006987 | activation of signaling protein activity... | 67 | 4 | 2.55 | 0.25107 |
| GO:0050890 | cognition | 228 | 11 | 8.68 | 0.25251 |
| GO:0050795 | regulation of behavior | 181 | 9 | 6.89 | 0.25293 |
| GO:0031397 | negative regulation of protein ubiquitin... | 112 | 6 | 4.26 | 0.25436 |
| GO:0006801 | superoxide metabolic process | 46 | 3 | 1.75 | 0.25484 |
| GO:0014896 | muscle hypertrophy | 46 | 3 | 1.75 | 0.25484 |
| GO:0042130 | negative regulation of T cell proliferat... | 46 | 3 | 1.75 | 0.25484 |
| GO:0045132 | meiotic chromosome segregation | 46 | 3 | 1.75 | 0.25484 |
| GO:0045980 | negative regulation of nucleotide metabo... | 46 | 3 | 1.75 | 0.25484 |
| GO:0051985 | negative regulation of chromosome segreg | 46 | 3 | 1.75 | 0.25484 |

| | | | | | |
|---|---|---|---|---|---|
| GO:0003007 | heart morphogenesis | 205 | 10 | 7.8 | 0.25539 |
| GO:0009952 | anterior/posterior pattern specification | 205 | 10 | 7.8 | 0.25539 |
| GO:0072330 | monocarboxylic acid biosynthetic process | 182 | 9 | 6.93 | 0.25793 |
| GO:0046467 | membrane lipid biosynthetic process | 90 | 5 | 3.43 | 0.25816 |
| GO:0071887 | leukocyte apoptotic process | 90 | 5 | 3.43 | 0.25816 |
| GO:0006637 | acyl-CoA metabolic process | 68 | 4 | 2.59 | 0.25948 |
| GO:0014013 | regulation of gliogenesis | 68 | 4 | 2.59 | 0.25948 |
| GO:0021954 | central nervous system neuron developme | 68 | 4 | 2.59 | 0.25948 |
| GO:0035383 | thioester metabolic process | 68 | 4 | 2.59 | 0.25948 |
| GO:0051817 | modification of morphology or physiology... | 68 | 4 | 2.59 | 0.25948 |
| GO:0045089 | positive regulation of innate immune res... | 206 | 10 | 7.84 | 0.2601 |
| GO:0001893 | maternal placenta development | 26 | 2 | 0.99 | 0.26032 |
| GO:0005979 | regulation of glycogen biosynthetic proc... | 26 | 2 | 0.99 | 0.26032 |
| GO:0005980 | glycogen catabolic process | 26 | 2 | 0.99 | 0.26032 |
| GO:0010611 | regulation of cardiac muscle hypertrophy | 26 | 2 | 0.99 | 0.26032 |
| GO:0010962 | regulation of glucan biosynthetic proces... | 26 | 2 | 0.99 | 0.26032 |
| GO:0014072 | response to isoquinoline alkaloid | 26 | 2 | 0.99 | 0.26032 |
| GO:0042133 | neurotransmitter metabolic process | 26 | 2 | 0.99 | 0.26032 |
| GO:0042269 | regulation of natural killer cell mediat... | 26 | 2 | 0.99 | 0.26032 |
| GO:0044058 | regulation of digestive system process | 26 | 2 | 0.99 | 0.26032 |
| GO:0045616 | regulation of keratinocyte differentiati... | 26 | 2 | 0.99 | 0.26032 |
| GO:0045992 | negative regulation of embryonic develop.. | 26 | 2 | 0.99 | 0.26032 |
| GO:0048199 | vesicle targeting to | from or withi | 26 | 2 | |
| GO:0051604 | protein maturation | 26 | 2 | 0.99 | 0.26032 |
| GO:0060444 | branching involved in mammary gland duc | 26 | 2 | 0.99 | 0.26032 |
| GO:0060612 | adipose tissue development | 26 | 2 | 0.99 | 0.26032 |
| GO:0070741 | response to interleukin-6 | 26 | 2 | 0.99 | 0.26032 |
| GO:0071353 | cellular response to interleukin-4 | 26 | 2 | 0.99 | 0.26032 |
| GO:0072666 | establishment of protein localization to... | 26 | 2 | 0.99 | 0.26032 |
| GO:0098751 | bone cell development | 26 | 2 | 0.99 | 0.26032 |
| GO:1901685 | glutathione derivative metabolic process | 26 | 2 | 0.99 | 0.26032 |
| GO:1901687 | glutathione derivative biosynthetic proc... | 26 | 2 | 0.99 | 0.26032 |
| GO:2000677 | regulation of transcription regulatory r... | 26 | 2 | 0.99 | 0.26032 |
| GO:0070613 | regulation of protein processing | 350 | 16 | 13.32 | 0.26063 |
| GO:0008037 | cell recognition | 113 | 6 | 4.3 | 0.26079 |
| GO:0006403 | RNA localization | 136 | 7 | 5.18 | 0.26105 |
| GO:0009749 | response to glucose | 136 | 7 | 5.18 | 0.26105 |
| GO:0030111 | regulation of Wnt signaling pathway | 278 | 13 | 10.58 | 0.26218 |
| GO:0005978 | glycogen biosynthetic process | 47 | 3 | 1.79 | 0.26521 |
| GO:0006513 | protein monoubiquitination | 47 | 3 | 1.79 | 0.26521 |
| GO:0009247 | glycolipid biosynthetic process | 47 | 3 | 1.79 | 0.26521 |
| GO:0009250 | glucan biosynthetic process | 47 | 3 | 1.79 | 0.26521 |
| GO:0010171 | body morphogenesis | 47 | 3 | 1.79 | 0.26521 |
| GO:0032663 | regulation of interleukin-2 production | 47 | 3 | 1.79 | 0.26521 |
| GO:1901796 | regulation of signal transduction by p53... | 47 | 3 | 1.79 | 0.26521 |
| GO:1903793 | positive regulation of anion transport | 47 | 3 | 1.79 | 0.26521 |
| GO:0002221 | pattern recognition receptor signaling p... | 160 | 8 | 6.09 | 0.26533 |
| GO:0008585 | female gonad development | 91 | 5 | 3.46 | 0.26543 |
| GO:0048524 | positive regulation of viral process | 91 | 5 | 3.46 | 0.26543 |
| GO:0060349 | bone morphogenesis | 91 | 5 | 3.46 | 0.26543 |
| GO:0050866 | negative regulation of cell activation | 137 | 7 | 5.22 | 0.26693 |
| GO:0001765 | membrane raft assembly | 8 | 1 | 0.3 | 0.26696 |
| GO:0001955 | blood vessel maturation | 8 | 1 | 0.3 | 0.26696 |

| | | | | | |
|---|---|---|---|---|---|
| GO:0002524 | hypersensitivity | 8 | 1 | 0.3 | 0.26696 |
| GO:0002689 | negative regulation of leukocyte chemota.. | 8 | 1 | 0.3 | 0.26696 |
| GO:0002716 | negative regulation of natural killer ce... | 8 | 1 | 0.3 | 0.26696 |
| GO:0002834 | regulation of response to tumor cell | 8 | 1 | 0.3 | 0.26696 |
| GO:0002836 | positive regulation of response to tumor... | 8 | 1 | 0.3 | 0.26696 |
| GO:0002837 | regulation of immune response to tumor c. | 8 | 1 | 0.3 | 0.26696 |
| GO:0002839 | positive regulation of immune response t... | 8 | 1 | 0.3 | 0.26696 |
| GO:0003334 | keratinocyte development | 8 | 1 | 0.3 | 0.26696 |
| GO:0006244 | pyrimidine nucleotide catabolic process | 8 | 1 | 0.3 | 0.26696 |
| GO:0006465 | signal peptide processing | 8 | 1 | 0.3 | 0.26696 |
| GO:0006534 | cysteine metabolic process | 8 | 1 | 0.3 | 0.26696 |
| GO:0006971 | hypotonic response | 8 | 1 | 0.3 | 0.26696 |
| GO:0007172 | signal complex assembly | 8 | 1 | 0.3 | 0.26696 |
| GO:0007262 | STAT protein import into nucleus | 8 | 1 | 0.3 | 0.26696 |
| GO:0007442 | hindgut morphogenesis | 8 | 1 | 0.3 | 0.26696 |
| GO:0009162 | deoxyribonucleoside monophosphate meta | 8 | 1 | 0.3 | 0.26696 |
| GO:0009186 | deoxyribonucleoside diphosphate metabol | 8 | 1 | 0.3 | 0.26696 |
| GO:0009301 | snRNA transcription | 8 | 1 | 0.3 | 0.26696 |
| GO:0016114 | terpenoid biosynthetic process | 8 | 1 | 0.3 | 0.26696 |
| GO:0016584 | nucleosome positioning | 8 | 1 | 0.3 | 0.26696 |
| GO:0021891 | olfactory bulb interneuron development | 8 | 1 | 0.3 | 0.26696 |
| GO:0021932 | hindbrain radial glia guided cell migrat... | 8 | 1 | 0.3 | 0.26696 |
| GO:0030854 | positive regulation of granulocyte diffe... | 8 | 1 | 0.3 | 0.26696 |
| GO:0030917 | midbrain-hindbrain boundary development | 8 | 1 | 0.3 | 0.26696 |
| GO:0031223 | auditory behavior | 8 | 1 | 0.3 | 0.26696 |
| GO:0031657 | regulation of cyclin-dependent protein s... | 8 | 1 | 0.3 | 0.26696 |
| GO:0032276 | regulation of gonadotropin secretion | 8 | 1 | 0.3 | 0.26696 |
| GO:0032471 | negative regulation of endoplasmic retic... | 8 | 1 | 0.3 | 0.26696 |
| GO:0032536 | regulation of cell projection size | 8 | 1 | 0.3 | 0.26696 |
| GO:0032875 | regulation of DNA endoreduplication | 8 | 1 | 0.3 | 0.26696 |
| GO:0033085 | negative regulation of T cell differenti... | 8 | 1 | 0.3 | 0.26696 |
| GO:0035330 | regulation of hippo signaling | 8 | 1 | 0.3 | 0.26696 |
| GO:0035630 | bone mineralization involved in bone mat.. | 8 | 1 | 0.3 | 0.26696 |
| GO:0035728 | response to hepatocyte growth factor | 8 | 1 | 0.3 | 0.26696 |
| GO:0035729 | cellular response to hepatocyte growth f... | 8 | 1 | 0.3 | 0.26696 |
| GO:0035747 | natural killer cell chemotaxis | 8 | 1 | 0.3 | 0.26696 |
| GO:0035970 | peptidyl-threonine dephosphorylation | 8 | 1 | 0.3 | 0.26696 |
| GO:0042023 | DNA endoreduplication | 8 | 1 | 0.3 | 0.26696 |
| GO:0042364 | water-soluble vitamin biosynthetic proce... | 8 | 1 | 0.3 | 0.26696 |
| GO:0042538 | hyperosmotic salinity response | 8 | 1 | 0.3 | 0.26696 |
| GO:0043102 | amino acid salvage | 8 | 1 | 0.3 | 0.26696 |
| GO:0043497 | regulation of protein heterodimerization... | 8 | 1 | 0.3 | 0.26696 |
| GO:0043653 | mitochondrial fragmentation involved in ... | 8 | 1 | 0.3 | 0.26696 |
| GO:0043951 | negative regulation of cAMP-mediated sig. | 8 | 1 | 0.3 | 0.26696 |
| GO:0044557 | relaxation of smooth muscle | 8 | 1 | 0.3 | 0.26696 |
| GO:0045116 | protein neddylation | 8 | 1 | 0.3 | 0.26696 |
| GO:0045416 | positive regulation of interleukin-8 bio... | 8 | 1 | 0.3 | 0.26696 |
| GO:0045650 | negative regulation of macrophage differ... | 8 | 1 | 0.3 | 0.26696 |
| GO:0045714 | regulation of low-density lipoprotein pa... | 8 | 1 | 0.3 | 0.26696 |
| GO:0045792 | negative regulation of cell size | 8 | 1 | 0.3 | 0.26696 |
| GO:0045924 | regulation of female receptivity | 8 | 1 | 0.3 | 0.26696 |
| GO:0045953 | negative regulation of natural killer ce... | 8 | 1 | 0.3 | 0.26696 |
| GO:0048149 | behavioral response to ethanol | 8 | 1 | 0.3 | 0.26696 |

| | | | | | |
|---|---|---|---|---|---|
| GO:0048752 | semicircular canal morphogenesis | 8 | 1 | 0.3 | 0.26696 |
| GO:0051451 | myoblast migration | 8 | 1 | 0.3 | 0.26696 |
| GO:0051481 | negative regulation of cytosolic calcium... | 8 | 1 | 0.3 | 0.26696 |
| GO:0060180 | female mating behavior | 8 | 1 | 0.3 | 0.26696 |
| GO:0060252 | positive regulation of glial cell prolif... | 8 | 1 | 0.3 | 0.26696 |
| GO:0060439 | trachea morphogenesis | 8 | 1 | 0.3 | 0.26696 |
| GO:0060897 | neural plate regionalization | 8 | 1 | 0.3 | 0.26696 |
| GO:0060964 | regulation of gene silencing by miRNA | 8 | 1 | 0.3 | 0.26696 |
| GO:0070099 | regulation of chemokine-mediated signali.. | 8 | 1 | 0.3 | 0.26696 |
| GO:0070669 | response to interleukin-2 | 8 | 1 | 0.3 | 0.26696 |
| GO:0071107 | response to parathyroid hormone | 8 | 1 | 0.3 | 0.26696 |
| GO:0071265 | L-methionine biosynthetic process | 8 | 1 | 0.3 | 0.26696 |
| GO:0071267 | L-methionine salvage | 8 | 1 | 0.3 | 0.26696 |
| GO:0072321 | chaperone-mediated protein transport | 8 | 1 | 0.3 | 0.26696 |
| GO:0072364 | regulation of cellular ketone metabolic ... | 8 | 1 | 0.3 | 0.26696 |
| GO:0072578 | neurotransmitter-gated ion channel clust... | 8 | 1 | 0.3 | 0.26696 |
| GO:0086013 | membrane repolarization during cardiac m | 8 | 1 | 0.3 | 0.26696 |
| GO:0086103 | G-protein coupled receptor signaling pat... | 8 | 1 | 0.3 | 0.26696 |
| GO:0090129 | positive regulation of synapse maturatio... | 8 | 1 | 0.3 | 0.26696 |
| GO:0090400 | stress-induced premature senescence | 8 | 1 | 0.3 | 0.26696 |
| GO:0097062 | dendritic spine maintenance | 8 | 1 | 0.3 | 0.26696 |
| GO:0097286 | iron ion import | 8 | 1 | 0.3 | 0.26696 |
| GO:0098911 | regulation of ventricular cardiac muscle... | 8 | 1 | 0.3 | 0.26696 |
| GO:1901978 | positive regulation of cell cycle checkp... | 8 | 1 | 0.3 | 0.26696 |
| GO:1903209 | positive regulation of oxidative stress-... | 8 | 1 | 0.3 | 0.26696 |
| GO:1903896 | positive regulation of IRE1-mediated unf... | 8 | 1 | 0.3 | 0.26696 |
| GO:2000074 | regulation of type B pancreatic cell dev... | 8 | 1 | 0.3 | 0.26696 |
| GO:2000192 | negative regulation of fatty acid transp... | 8 | 1 | 0.3 | 0.26696 |
| GO:0042471 | ear morphogenesis | 114 | 6 | 4.34 | 0.26727 |
| GO:0006308 | DNA catabolic process | 69 | 4 | 2.63 | 0.26794 |
| GO:0007422 | peripheral nervous system development | 69 | 4 | 2.63 | 0.26794 |
| GO:0030104 | water homeostasis | 69 | 4 | 2.63 | 0.26794 |
| GO:0048705 | skeletal system morphogenesis | 208 | 10 | 7.92 | 0.26959 |
| GO:0005996 | monosaccharide metabolic process | 280 | 13 | 10.66 | 0.27035 |
| GO:0006333 | chromatin assembly or disassembly | 161 | 8 | 6.13 | 0.27077 |
| GO:0022407 | regulation of cell-cell adhesion | 329 | 15 | 12.52 | 0.27272 |
| GO:0001837 | epithelial to mesenchymal transition | 92 | 5 | 3.5 | 0.27275 |
| GO:0003231 | cardiac ventricle development | 92 | 5 | 3.5 | 0.27275 |
| GO:0007292 | female gamete generation | 92 | 5 | 3.5 | 0.27275 |
| GO:0044264 | cellular polysaccharide metabolic proces... | 92 | 5 | 3.5 | 0.27275 |
| GO:0055072 | iron ion homeostasis | 92 | 5 | 3.5 | 0.27275 |
| GO:0060491 | regulation of cell projection assembly | 92 | 5 | 3.5 | 0.27275 |
| GO:0045787 | positive regulation of cell cycle | 281 | 13 | 10.7 | 0.27447 |
| GO:0002707 | negative regulation of lymphocyte mediat.. | 27 | 2 | 1.03 | 0.27461 |
| GO:0002715 | regulation of natural killer cell mediat... | 27 | 2 | 1.03 | 0.27461 |
| GO:0009251 | glucan catabolic process | 27 | 2 | 1.03 | 0.27461 |
| GO:0009394 | 2'-deoxyribonucleotide metabolic process | 27 | 2 | 1.03 | 0.27461 |
| GO:0014743 | regulation of muscle hypertrophy | 27 | 2 | 1.03 | 0.27461 |
| GO:0030449 | regulation of complement activation | 27 | 2 | 1.03 | 0.27461 |
| GO:0030947 | regulation of vascular endothelial growt... | 27 | 2 | 1.03 | 0.27461 |
| GO:0033598 | mammary gland epithelial cell proliferat... | 27 | 2 | 1.03 | 0.27461 |
| GO:0043552 | positive regulation of phosphatidylinosi... | 27 | 2 | 1.03 | 0.27461 |
| GO:0045880 | positive regulation of smoothened signal... | 27 | 2 | 1.03 | 0.27461 |

| | | | | | |
|---|---|---|---|---|---|
| GO:0060314 | regulation of ryanodine-sensitive calciu... | 27 | 2 | 1.03 | 0.27461 |
| GO:0072665 | protein localization to vacuole | 27 | 2 | 1.03 | 0.27461 |
| GO:1903523 | negative regulation of blood circulation | 27 | 2 | 1.03 | 0.27461 |
| GO:2000826 | regulation of heart morphogenesis | 27 | 2 | 1.03 | 0.27461 |
| GO:1901605 | alpha-amino acid metabolic process | 233 | 11 | 8.87 | 0.27491 |
| GO:0007041 | lysosomal transport | 48 | 3 | 1.83 | 0.27562 |
| GO:0046456 | icosanoid biosynthetic process | 48 | 3 | 1.83 | 0.27562 |
| GO:1901570 | fatty acid derivative biosynthetic proce... | 48 | 3 | 1.83 | 0.27562 |
| GO:0002758 | innate immune response-activating signal. | 162 | 8 | 6.17 | 0.27624 |
| GO:0007187 | G-protein coupled receptor signaling pat... | 162 | 8 | 6.17 | 0.27624 |
| GO:0002433 | immune response-regulating cell surface .. | 70 | 4 | 2.66 | 0.27645 |
| GO:0038096 | Fc-gamma receptor signaling pathway invo | 70 | 4 | 2.66 | 0.27645 |
| GO:0051437 | positive regulation of ubiquitin-protein... | 70 | 4 | 2.66 | 0.27645 |
| GO:0007519 | skeletal muscle tissue development | 139 | 7 | 5.29 | 0.2788 |
| GO:0032147 | activation of protein kinase activity | 258 | 12 | 9.82 | 0.27923 |
| GO:0050821 | protein stabilization | 93 | 5 | 3.54 | 0.28011 |
| GO:2000058 | regulation of protein ubiquitination inv... | 93 | 5 | 3.54 | 0.28011 |
| GO:0022408 | negative regulation of cell-cell adhesio... | 116 | 6 | 4.42 | 0.28034 |
| GO:0035967 | cellular response to topologically incor... | 116 | 6 | 4.42 | 0.28034 |
| GO:0043280 | positive regulation of cysteine-type end... | 116 | 6 | 4.42 | 0.28034 |
| GO:0038094 | Fc-gamma receptor signaling pathway | 71 | 4 | 2.7 | 0.285 |
| GO:1901607 | alpha-amino acid biosynthetic process | 71 | 4 | 2.7 | 0.285 |
| GO:0001910 | regulation of leukocyte mediated cytotox... | 49 | 3 | 1.87 | 0.28607 |
| GO:0021695 | cerebellar cortex development | 49 | 3 | 1.87 | 0.28607 |
| GO:0032370 | positive regulation of lipid transport | 49 | 3 | 1.87 | 0.28607 |
| GO:0032414 | positive regulation of ion transmembrane... | 49 | 3 | 1.87 | 0.28607 |
| GO:0001676 | long-chain fatty acid metabolic process | 94 | 5 | 3.58 | 0.2875 |
| GO:0070646 | protein modification by small protein re... | 94 | 5 | 3.58 | 0.2875 |
| GO:0000380 | alternative mRNA splicing | via spliceos | 28 | 2 | 1.07 |
| GO:0001937 | negative regulation of endothelial cell ... | 28 | 2 | 1.07 | 0.28889 |
| GO:0002720 | positive regulation of cytokine producti... | 28 | 2 | 1.07 | 0.28889 |
| GO:0006084 | acetyl-CoA metabolic process | 28 | 2 | 1.07 | 0.28889 |
| GO:0007129 | synapsis | 28 | 2 | 1.07 | 0.28889 |
| GO:0008347 | glial cell migration | 28 | 2 | 1.07 | 0.28889 |
| GO:0030261 | chromosome condensation | 28 | 2 | 1.07 | 0.28889 |
| GO:0030851 | granulocyte differentiation | 28 | 2 | 1.07 | 0.28889 |
| GO:0032350 | regulation of hormone metabolic process | 28 | 2 | 1.07 | 0.28889 |
| GO:0035337 | fatty-acyl-CoA metabolic process | 28 | 2 | 1.07 | 0.28889 |
| GO:0044003 | modification by symbiont of host morphol.. | 28 | 2 | 1.07 | 0.28889 |
| GO:0044247 | cellular polysaccharide catabolic proces... | 28 | 2 | 1.07 | 0.28889 |
| GO:0044253 | positive regulation of multicellular org... | 28 | 2 | 1.07 | 0.28889 |
| GO:0048741 | skeletal muscle fiber development | 28 | 2 | 1.07 | 0.28889 |
| GO:1903792 | negative regulation of anion transport | 28 | 2 | 1.07 | 0.28889 |
| GO:0009746 | response to hexose | 141 | 7 | 5.37 | 0.29079 |
| GO:0071826 | ribonucleoprotein complex subunit organi.. | 141 | 7 | 5.37 | 0.29079 |
| GO:0019318 | hexose metabolic process | 237 | 11 | 9.02 | 0.29327 |
| GO:0002685 | regulation of leukocyte migration | 118 | 6 | 4.49 | 0.29355 |
| GO:0001570 | vasculogenesis | 72 | 4 | 2.74 | 0.29359 |
| GO:0009062 | fatty acid catabolic process | 72 | 4 | 2.74 | 0.29359 |
| GO:0009156 | ribonucleoside monophosphate biosynthet | 72 | 4 | 2.74 | 0.29359 |
| GO:0015908 | fatty acid transport | 72 | 4 | 2.74 | 0.29359 |
| GO:2000106 | regulation of leukocyte apoptotic proces... | 72 | 4 | 2.74 | 0.29359 |
| GO:2001257 | regulation of cation channel activity | 72 | 4 | 2.74 | 0.29359 |

| | | | | | |
|---|---|---|---|---|---|
| GO:0000244 | spliceosomal tri-snRNP complex assembly | 9 | 1 | 0.34 | 0.29488 |
| GO:0001714 | endodermal cell fate specification | 9 | 1 | 0.34 | 0.29488 |
| GO:0002480 | antigen processing and presentation of e... | 9 | 1 | 0.34 | 0.29488 |
| GO:0002864 | regulation of acute inflammatory respons... | 9 | 1 | 0.34 | 0.29488 |
| GO:0003177 | pulmonary valve development | 9 | 1 | 0.34 | 0.29488 |
| GO:0003184 | pulmonary valve morphogenesis | 9 | 1 | 0.34 | 0.29488 |
| GO:0003198 | epithelial to mesenchymal transition inv... | 9 | 1 | 0.34 | 0.29488 |
| GO:0003222 | ventricular trabecula myocardium morphog | 9 | 1 | 0.34 | 0.29488 |
| GO:0006020 | inositol metabolic process | 9 | 1 | 0.34 | 0.29488 |
| GO:0006054 | N-acetylneuraminate metabolic process | 9 | 1 | 0.34 | 0.29488 |
| GO:0006527 | arginine catabolic process | 9 | 1 | 0.34 | 0.29488 |
| GO:0006559 | L-phenylalanine catabolic process | 9 | 1 | 0.34 | 0.29488 |
| GO:0006570 | tyrosine metabolic process | 9 | 1 | 0.34 | 0.29488 |
| GO:0009133 | nucleoside diphosphate biosynthetic proc... | 9 | 1 | 0.34 | 0.29488 |
| GO:0009265 | 2'-deoxyribonucleotide biosynthetic proc... | 9 | 1 | 0.34 | 0.29488 |
| GO:0010944 | negative regulation of transcription by ... | 9 | 1 | 0.34 | 0.29488 |
| GO:0010972 | negative regulation of G2/M transition o... | 9 | 1 | 0.34 | 0.29488 |
| GO:0015816 | glycine transport | 9 | 1 | 0.34 | 0.29488 |
| GO:0019985 | translesion synthesis | 9 | 1 | 0.34 | 0.29488 |
| GO:0021873 | forebrain neuroblast division | 9 | 1 | 0.34 | 0.29488 |
| GO:0022417 | protein maturation by protein folding | 9 | 1 | 0.34 | 0.29488 |
| GO:0030007 | cellular potassium ion homeostasis | 9 | 1 | 0.34 | 0.29488 |
| GO:0030647 | aminoglycoside antibiotic metabolic proc... | 9 | 1 | 0.34 | 0.29488 |
| GO:0032000 | positive regulation of fatty acid beta-o... | 9 | 1 | 0.34 | 0.29488 |
| GO:0032328 | alanine transport | 9 | 1 | 0.34 | 0.29488 |
| GO:0032341 | aldosterone metabolic process | 9 | 1 | 0.34 | 0.29488 |
| GO:0032342 | aldosterone biosynthetic process | 9 | 1 | 0.34 | 0.29488 |
| GO:0032957 | inositol trisphosphate metabolic process | 9 | 1 | 0.34 | 0.29488 |
| GO:0035246 | peptidyl-arginine N-methylation | 9 | 1 | 0.34 | 0.29488 |
| GO:0036120 | cellular response to platelet-derived gr... | 9 | 1 | 0.34 | 0.29488 |
| GO:0042670 | retinal cone cell differentiation | 9 | 1 | 0.34 | 0.29488 |
| GO:0043116 | negative regulation of vascular permeabi... | 9 | 1 | 0.34 | 0.29488 |
| GO:0044381 | glucose import in response to insulin st... | 9 | 1 | 0.34 | 0.29488 |
| GO:0045589 | regulation of regulatory T cell differen... | 9 | 1 | 0.34 | 0.29488 |
| GO:0045713 | low-density lipoprotein particle recepto... | 9 | 1 | 0.34 | 0.29488 |
| GO:0045793 | positive regulation of cell size | 9 | 1 | 0.34 | 0.29488 |
| GO:0046385 | deoxyribose phosphate biosynthetic proce | 9 | 1 | 0.34 | 0.29488 |
| GO:0046548 | retinal rod cell development | 9 | 1 | 0.34 | 0.29488 |
| GO:0046549 | retinal cone cell development | 9 | 1 | 0.34 | 0.29488 |
| GO:0048340 | paraxial mesoderm morphogenesis | 9 | 1 | 0.34 | 0.29488 |
| GO:0048703 | embryonic viscerocranium morphogenesis | 9 | 1 | 0.34 | 0.29488 |
| GO:0050746 | regulation of lipoprotein metabolic proc... | 9 | 1 | 0.34 | 0.29488 |
| GO:0050774 | negative regulation of dendrite morphoge... | 9 | 1 | 0.34 | 0.29488 |
| GO:0050779 | RNA destabilization | 9 | 1 | 0.34 | 0.29488 |
| GO:0051450 | myoblast proliferation | 9 | 1 | 0.34 | 0.29488 |
| GO:0051560 | mitochondrial calcium ion homeostasis | 9 | 1 | 0.34 | 0.29488 |
| GO:0051956 | negative regulation of amino acid transp... | 9 | 1 | 0.34 | 0.29488 |
| GO:0060052 | neurofilament cytoskeleton organization | 9 | 1 | 0.34 | 0.29488 |
| GO:0060123 | regulation of growth hormone secretion | 9 | 1 | 0.34 | 0.29488 |
| GO:0060456 | positive regulation of digestive system ... | 9 | 1 | 0.34 | 0.29488 |
| GO:0060592 | mammary gland formation | 9 | 1 | 0.34 | 0.29488 |
| GO:0060638 | mesenchymal-epithelial cell signaling | 9 | 1 | 0.34 | 0.29488 |
| GO:0061085 | regulation of histone H3-K27 methylation | 9 | 1 | 0.34 | 0.29488 |

| | | | | | |
|---|---|---|---|---|---|
| GO:0061525 | hindgut development | 9 | 1 | 0.34 | 0.29488 |
| GO:0097120 | receptor localization to synapse | 9 | 1 | 0.34 | 0.29488 |
| GO:1901844 | regulation of cell communication by elec... | 9 | 1 | 0.34 | 0.29488 |
| GO:1902222 | erythrose 4-phosphate/phosphoenolpyruva | 9 | 1 | 0.34 | 0.29488 |
| GO:2000425 | regulation of apoptotic cell clearance | 9 | 1 | 0.34 | 0.29488 |
| GO:2001028 | positive regulation of endothelial cell ... | 9 | 1 | 0.34 | 0.29488 |
| GO:2001273 | regulation of glucose import in response... | 9 | 1 | 0.34 | 0.29488 |
| GO:0006690 | icosanoid metabolic process | 95 | 5 | 3.62 | 0.29494 |
| GO:0007631 | feeding behavior | 95 | 5 | 3.62 | 0.29494 |
| GO:0046545 | development of primary female sexual cha | 95 | 5 | 3.62 | 0.29494 |
| GO:1901568 | fatty acid derivative metabolic process | 95 | 5 | 3.62 | 0.29494 |
| GO:0008593 | regulation of Notch signaling pathway | 50 | 3 | 1.9 | 0.29656 |
| GO:0010463 | mesenchymal cell proliferation | 50 | 3 | 1.9 | 0.29656 |
| GO:0022029 | telencephalon cell migration | 50 | 3 | 1.9 | 0.29656 |
| GO:0042398 | cellular modified amino acid biosyntheti... | 50 | 3 | 1.9 | 0.29656 |
| GO:0060079 | regulation of excitatory postsynaptic me... | 50 | 3 | 1.9 | 0.29656 |
| GO:0060135 | maternal process involved in female preg... | 50 | 3 | 1.9 | 0.29656 |
| GO:0060411 | cardiac septum morphogenesis | 50 | 3 | 1.9 | 0.29656 |
| GO:0070228 | regulation of lymphocyte apoptotic proce... | 50 | 3 | 1.9 | 0.29656 |
| GO:0006694 | steroid biosynthetic process | 142 | 7 | 5.41 | 0.29683 |
| GO:0046486 | glycerolipid metabolic process | 311 | 14 | 11.84 | 0.29778 |
| GO:0032956 | regulation of actin cytoskeleton organiz... | 238 | 11 | 9.06 | 0.29791 |
| GO:0045444 | fat cell differentiation | 166 | 8 | 6.32 | 0.29843 |
| GO:0045637 | regulation of myeloid cell differentiati... | 166 | 8 | 6.32 | 0.29843 |
| GO:0006665 | sphingolipid metabolic process | 119 | 6 | 4.53 | 0.30021 |
| GO:1903321 | negative regulation of protein modificat... | 119 | 6 | 4.53 | 0.30021 |
| GO:0051962 | positive regulation of nervous system de... | 312 | 14 | 11.88 | 0.30185 |
| GO:0031532 | actin cytoskeleton reorganization | 73 | 4 | 2.78 | 0.30221 |
| GO:0046782 | regulation of viral transcription | 73 | 4 | 2.78 | 0.30221 |
| GO:0009755 | hormone-mediated signaling pathway | 96 | 5 | 3.65 | 0.3024 |
| GO:0051260 | protein homooligomerization | 239 | 11 | 9.1 | 0.30258 |
| GO:0051701 | interaction with host | 143 | 7 | 5.44 | 0.3029 |
| GO:0000188 | inactivation of MAPK activity | 29 | 2 | 1.1 | 0.30313 |
| GO:0006099 | tricarboxylic acid cycle | 29 | 2 | 1.1 | 0.30313 |
| GO:0006506 | GPI anchor biosynthetic process | 29 | 2 | 1.1 | 0.30313 |
| GO:0007212 | dopamine receptor signaling pathway | 29 | 2 | 1.1 | 0.30313 |
| GO:0010574 | regulation of vascular endothelial growt... | 29 | 2 | 1.1 | 0.30313 |
| GO:0019692 | deoxyribose phosphate metabolic process | 29 | 2 | 1.1 | 0.30313 |
| GO:0021955 | central nervous system neuron axonogene | 29 | 2 | 1.1 | 0.30313 |
| GO:0033003 | regulation of mast cell activation | 29 | 2 | 1.1 | 0.30313 |
| GO:0040036 | regulation of fibroblast growth factor r... | 29 | 2 | 1.1 | 0.30313 |
| GO:0045879 | negative regulation of smoothened signal... | 29 | 2 | 1.1 | 0.30313 |
| GO:0060412 | ventricular septum morphogenesis | 29 | 2 | 1.1 | 0.30313 |
| GO:0090218 | positive regulation of lipid kinase acti... | 29 | 2 | 1.1 | 0.30313 |
| GO:0090503 | RNA phosphodiester bond hydrolysis | exon... | 29 | 2 | 1.1 |
| GO:1901222 | regulation of NIK/NF-kappaB signaling | 29 | 2 | 1.1 | 0.30313 |
| GO:1902186 | regulation of viral release from host ce... | 29 | 2 | 1.1 | 0.30313 |
| GO:2000257 | regulation of protein activation cascade | 29 | 2 | 1.1 | 0.30313 |
| GO:0034110 | regulation of homotypic cell-cell adhesi... | 264 | 12 | 10.05 | 0.30565 |
| GO:0006354 | DNA-templated transcription | elongation | 120 | 6 | 4.57 |
| GO:0042384 | cilium assembly | 120 | 6 | 4.57 | 0.30689 |
| GO:0048520 | positive regulation of behavior | 120 | 6 | 4.57 | 0.30689 |
| GO:0030850 | prostate gland development | 51 | 3 | 1.94 | 0.30706 |

| | | | | | |
|---|---|---|---|---|---|
| GO:0031577 | spindle checkpoint | 51 | 3 | 1.94 | 0.30706 |
| GO:0048708 | astrocyte differentiation | 51 | 3 | 1.94 | 0.30706 |
| GO:2001244 | positive regulation of intrinsic apoptot... | 51 | 3 | 1.94 | 0.30706 |
| GO:0045926 | negative regulation of growth | 216 | 10 | 8.22 | 0.30856 |
| GO:0050954 | sensory perception of mechanical stimulu... | 144 | 7 | 5.48 | 0.309 |
| GO:0002218 | activation of innate immune response | 168 | 8 | 6.4 | 0.30967 |
| GO:0030218 | erythrocyte differentiation | 97 | 5 | 3.69 | 0.30989 |
| GO:0042493 | response to drug | 388 | 17 | 14.77 | 0.30999 |
| GO:0001558 | regulation of cell growth | 314 | 14 | 11.95 | 0.31003 |
| GO:0031589 | cell-substrate adhesion | 265 | 12 | 10.09 | 0.31012 |
| GO:0022600 | digestive system process | 74 | 4 | 2.82 | 0.31086 |
| GO:0031571 | mitotic G1 DNA damage checkpoint | 74 | 4 | 2.82 | 0.31086 |
| GO:0071621 | granulocyte chemotaxis | 74 | 4 | 2.82 | 0.31086 |
| GO:0032102 | negative regulation of response to exter... | 217 | 10 | 8.26 | 0.31352 |
| GO:2001020 | regulation of response to DNA damage sti... | 121 | 6 | 4.61 | 0.3136 |
| GO:0042176 | regulation of protein catabolic process | 365 | 16 | 13.89 | 0.3166 |
| GO:0000272 | polysaccharide catabolic process | 30 | 2 | 1.14 | 0.31732 |
| GO:0001510 | RNA methylation | 30 | 2 | 1.14 | 0.31732 |
| GO:0003009 | skeletal muscle contraction | 30 | 2 | 1.14 | 0.31732 |
| GO:0009069 | serine family amino acid metabolic proce... | 30 | 2 | 1.14 | 0.31732 |
| GO:0010573 | vascular endothelial growth factor produ... | 30 | 2 | 1.14 | 0.31732 |
| GO:0014904 | myotube cell development | 30 | 2 | 1.14 | 0.31732 |
| GO:0016572 | histone phosphorylation | 30 | 2 | 1.14 | 0.31732 |
| GO:0040018 | positive regulation of multicellular org... | 30 | 2 | 1.14 | 0.31732 |
| GO:0045824 | negative regulation of innate immune res... | 30 | 2 | 1.14 | 0.31732 |
| GO:0048665 | neuron fate specification | 30 | 2 | 1.14 | 0.31732 |
| GO:0050710 | negative regulation of cytokine secretio... | 30 | 2 | 1.14 | 0.31732 |
| GO:0060071 | Wnt signaling pathway | planar cell p | 30 | 2 | 1.14 |
| GO:0071622 | regulation of granulocyte chemotaxis | 30 | 2 | 1.14 | 0.31732 |
| GO:0072132 | mesenchyme morphogenesis | 30 | 2 | 1.14 | 0.31732 |
| GO:0090311 | regulation of protein deacetylation | 30 | 2 | 1.14 | 0.31732 |
| GO:1902742 | apoptotic process involved in developmen... | 30 | 2 | 1.14 | 0.31732 |
| GO:2001259 | positive regulation of cation channel ac... | 30 | 2 | 1.14 | 0.31732 |
| GO:0010594 | regulation of endothelial cell migration | 98 | 5 | 3.73 | 0.31741 |
| GO:2000134 | negative regulation of G1/S transition o... | 98 | 5 | 3.73 | 0.31741 |
| GO:0050434 | positive regulation of viral transcripti... | 52 | 3 | 1.98 | 0.31758 |
| GO:0070301 | cellular response to hydrogen peroxide | 52 | 3 | 1.98 | 0.31758 |
| GO:0010955 | negative regulation of protein processin... | 316 | 14 | 12.03 | 0.31828 |
| GO:0044783 | G1 DNA damage checkpoint | 75 | 4 | 2.85 | 0.31954 |
| GO:0044819 | mitotic G1/S transition checkpoint | 75 | 4 | 2.85 | 0.31954 |
| GO:0050868 | negative regulation of T cell activation | 75 | 4 | 2.85 | 0.31954 |
| GO:0018279 | protein N-linked glycosylation via aspar... | 122 | 6 | 4.64 | 0.32033 |
| GO:0010720 | positive regulation of cell development | 366 | 16 | 13.93 | 0.32045 |
| GO:0001542 | ovulation from ovarian follicle | 10 | 1 | 0.38 | 0.32173 |
| GO:0002418 | immune response to tumor cell | 10 | 1 | 0.38 | 0.32173 |
| GO:0002726 | positive regulation of T cell cytokine p... | 10 | 1 | 0.38 | 0.32173 |
| GO:0002862 | negative regulation of inflammatory resp... | 10 | 1 | 0.38 | 0.32173 |
| GO:0006047 | UDP-N-acetylglucosamine metabolic proce | 10 | 1 | 0.38 | 0.32173 |
| GO:0006491 | N-glycan processing | 10 | 1 | 0.38 | 0.32173 |
| GO:0006558 | L-phenylalanine metabolic process | 10 | 1 | 0.38 | 0.32173 |
| GO:0008343 | adult feeding behavior | 10 | 1 | 0.38 | 0.32173 |
| GO:0008655 | pyrimidine-containing compound salvage | 10 | 1 | 0.38 | 0.32173 |
| GO:0009435 | NAD biosynthetic process | 10 | 1 | 0.38 | 0.32173 |

| | | | | | |
|---|---|---|---|---|---|
| GO:0010561 | negative regulation of glycoprotein bios... | 10 | 1 | 0.38 | 0.32173 |
| GO:0010592 | positive regulation of lamellipodium ass... | 10 | 1 | 0.38 | 0.32173 |
| GO:0010642 | negative regulation of platelet-derived ... | 10 | 1 | 0.38 | 0.32173 |
| GO:0010804 | negative regulation of tumor necrosis fa... | 10 | 1 | 0.38 | 0.32173 |
| GO:0010998 | regulation of translational initiation b... | 10 | 1 | 0.38 | 0.32173 |
| GO:0015074 | DNA integration | 10 | 1 | 0.38 | 0.32173 |
| GO:0016446 | somatic hypermutation of immunoglobulin | 10 | 1 | 0.38 | 0.32173 |
| GO:0018216 | peptidyl-arginine methylation | 10 | 1 | 0.38 | 0.32173 |
| GO:0030916 | otic vesicle formation | 10 | 1 | 0.38 | 0.32173 |
| GO:0031646 | positive regulation of neurological syst... | 10 | 1 | 0.38 | 0.32173 |
| GO:0032096 | negative regulation of response to food | 10 | 1 | 0.38 | 0.32173 |
| GO:0032099 | negative regulation of appetite | 10 | 1 | 0.38 | 0.32173 |
| GO:0032274 | gonadotropin secretion | 10 | 1 | 0.38 | 0.32173 |
| GO:0032836 | glomerular basement membrane developm | 10 | 1 | 0.38 | 0.32173 |
| GO:0034776 | response to histamine | 10 | 1 | 0.38 | 0.32173 |
| GO:0035994 | response to muscle stretch | 10 | 1 | 0.38 | 0.32173 |
| GO:0036119 | response to platelet-derived growth fact... | 10 | 1 | 0.38 | 0.32173 |
| GO:0038003 | opioid receptor signaling pathway | 10 | 1 | 0.38 | 0.32173 |
| GO:0042482 | positive regulation of odontogenesis | 10 | 1 | 0.38 | 0.32173 |
| GO:0043097 | pyrimidine nucleoside salvage | 10 | 1 | 0.38 | 0.32173 |
| GO:0043619 | regulation of transcription from RNA pol... | 10 | 1 | 0.38 | 0.32173 |
| GO:0044320 | cellular response to leptin stimulus | 10 | 1 | 0.38 | 0.32173 |
| GO:0045059 | positive thymic T cell selection | 10 | 1 | 0.38 | 0.32173 |
| GO:0045292 | mRNA cis splicing | via spliceos | 10 | 1 | 0.38 |
| GO:0045916 | negative regulation of complement activa.. | 10 | 1 | 0.38 | 0.32173 |
| GO:0046855 | inositol phosphate dephosphorylation | 10 | 1 | 0.38 | 0.32173 |
| GO:0048679 | regulation of axon regeneration | 10 | 1 | 0.38 | 0.32173 |
| GO:0050765 | negative regulation of phagocytosis | 10 | 1 | 0.38 | 0.32173 |
| GO:0051546 | keratinocyte migration | 10 | 1 | 0.38 | 0.32173 |
| GO:0051593 | response to folic acid | 10 | 1 | 0.38 | 0.32173 |
| GO:0055062 | phosphate ion homeostasis | 10 | 1 | 0.38 | 0.32173 |
| GO:0060346 | bone trabecula formation | 10 | 1 | 0.38 | 0.32173 |
| GO:0060872 | semicircular canal development | 10 | 1 | 0.38 | 0.32173 |
| GO:0060896 | neural plate pattern specification | 10 | 1 | 0.38 | 0.32173 |
| GO:0061029 | eyelid development in camera-type eye | 10 | 1 | 0.38 | 0.32173 |
| GO:0061314 | Notch signaling involved in heart develo... | 10 | 1 | 0.38 | 0.32173 |
| GO:0070102 | interleukin-6-mediated signaling pathway | 10 | 1 | 0.38 | 0.32173 |
| GO:0070935 | 3'-UTR-mediated mRNA stabilization | 10 | 1 | 0.38 | 0.32173 |
| GO:0071318 | cellular response to ATP | 10 | 1 | 0.38 | 0.32173 |
| GO:0072506 | trivalent inorganic anion homeostasis | 10 | 1 | 0.38 | 0.32173 |
| GO:0090009 | primitive streak formation | 10 | 1 | 0.38 | 0.32173 |
| GO:0090128 | regulation of synapse maturation | 10 | 1 | 0.38 | 0.32173 |
| GO:0090266 | regulation of mitotic cell cycle spindle... | 10 | 1 | 0.38 | 0.32173 |
| GO:0097067 | cellular response to thyroid hormone sti... | 10 | 1 | 0.38 | 0.32173 |
| GO:0098659 | inorganic cation import into cell | 10 | 1 | 0.38 | 0.32173 |
| GO:0099587 | inorganic ion import into cell | 10 | 1 | 0.38 | 0.32173 |
| GO:1900409 | positive regulation of cellular response... | 10 | 1 | 0.38 | 0.32173 |
| GO:1902221 | erythrose 4-phosphate/phosphoenolpyruva | 10 | 1 | 0.38 | 0.32173 |
| GO:1902337 | regulation of apoptotic process involved... | 10 | 1 | 0.38 | 0.32173 |
| GO:1902750 | negative regulation of cell cycle G2/M p... | 10 | 1 | 0.38 | 0.32173 |
| GO:1902884 | positive regulation of response to oxida... | 10 | 1 | 0.38 | 0.32173 |
| GO:1903504 | regulation of mitotic spindle checkpoint | 10 | 1 | 0.38 | 0.32173 |
| GO:1903894 | regulation of IRE1-mediated unfolded pro.. | 10 | 1 | 0.38 | 0.32173 |

| | | | | | |
|---|---|---|---|---|---|
| GO:2000651 | positive regulation of sodium ion transm... | 10 | 1 | 0.38 | 0.32173 |
| GO:2000727 | positive regulation of cardiac muscle ce... | 10 | 1 | 0.38 | 0.32173 |
| GO:2000773 | negative regulation of cellular senescen... | 10 | 1 | 0.38 | 0.32173 |
| GO:2001179 | regulation of interleukin-10 secretion | 10 | 1 | 0.38 | 0.32173 |
| GO:2001267 | regulation of cysteine-type endopeptidas... | 10 | 1 | 0.38 | 0.32173 |
| GO:0045088 | regulation of innate immune response | 268 | 12 | 10.2 | 0.32363 |
| GO:1902807 | negative regulation of cell cycle G1/S p... | 99 | 5 | 3.77 | 0.32495 |
| GO:0051648 | vesicle localization | 195 | 9 | 7.42 | 0.32532 |
| GO:0032535 | regulation of cellular component size | 244 | 11 | 9.29 | 0.32616 |
| GO:0030336 | negative regulation of cell migration | 171 | 8 | 6.51 | 0.3267 |
| GO:0002695 | negative regulation of leukocyte activat... | 123 | 6 | 4.68 | 0.32708 |
| GO:0018196 | peptidyl-asparagine modification | 123 | 6 | 4.68 | 0.32708 |
| GO:0021885 | forebrain cell migration | 53 | 3 | 2.02 | 0.32811 |
| GO:0032623 | interleukin-2 production | 53 | 3 | 2.02 | 0.32811 |
| GO:0045839 | negative regulation of mitotic nuclear d... | 53 | 3 | 2.02 | 0.32811 |
| GO:0060688 | regulation of morphogenesis of a branchi.. | 53 | 3 | 2.02 | 0.32811 |
| GO:0051897 | positive regulation of protein kinase B ... | 76 | 4 | 2.89 | 0.32823 |
| GO:0033044 | regulation of chromosome organization | 196 | 9 | 7.46 | 0.33066 |
| GO:0043281 | regulation of cysteine-type endopeptidas... | 196 | 9 | 7.46 | 0.33066 |
| GO:0030099 | myeloid cell differentiation | 319 | 14 | 12.14 | 0.33076 |
| GO:0001960 | negative regulation of cytokine-mediated... | 31 | 2 | 1.18 | 0.33144 |
| GO:0002548 | monocyte chemotaxis | 31 | 2 | 1.18 | 0.33144 |
| GO:0003341 | cilium movement | 31 | 2 | 1.18 | 0.33144 |
| GO:0006505 | GPI anchor metabolic process | 31 | 2 | 1.18 | 0.33144 |
| GO:0014037 | Schwann cell differentiation | 31 | 2 | 1.18 | 0.33144 |
| GO:0014888 | striated muscle adaptation | 31 | 2 | 1.18 | 0.33144 |
| GO:0032743 | positive regulation of interleukin-2 pro... | 31 | 2 | 1.18 | 0.33144 |
| GO:0043094 | cellular metabolic compound salvage | 31 | 2 | 1.18 | 0.33144 |
| GO:0045143 | homologous chromosome segregation | 31 | 2 | 1.18 | 0.33144 |
| GO:0060396 | growth hormone receptor signaling pathwa | 31 | 2 | 1.18 | 0.33144 |
| GO:0070670 | response to interleukin-4 | 31 | 2 | 1.18 | 0.33144 |
| GO:0086002 | cardiac muscle cell action potential inv... | 31 | 2 | 1.18 | 0.33144 |
| GO:0090175 | regulation of establishment of planar po... | 31 | 2 | 1.18 | 0.33144 |
| GO:0098781 | ncRNA transcription | 31 | 2 | 1.18 | 0.33144 |
| GO:0016049 | cell growth | 394 | 17 | 15 | 0.33238 |
| GO:0008361 | regulation of cell size | 100 | 5 | 3.81 | 0.33252 |
| GO:0060048 | cardiac muscle contraction | 100 | 5 | 3.81 | 0.33252 |
| GO:0061448 | connective tissue development | 221 | 10 | 8.41 | 0.33356 |
| GO:0060538 | skeletal muscle organ development | 148 | 7 | 5.63 | 0.3336 |
| GO:0090090 | negative regulation of canonical Wnt sig... | 148 | 7 | 5.63 | 0.3336 |
| GO:0019216 | regulation of lipid metabolic process | 246 | 11 | 9.36 | 0.3357 |
| GO:0022604 | regulation of cell morphogenesis | 395 | 17 | 15.04 | 0.33615 |
| GO:0071333 | cellular response to glucose stimulus | 77 | 4 | 2.93 | 0.33693 |
| GO:0045666 | positive regulation of neuron differenti... | 222 | 10 | 8.45 | 0.33861 |
| GO:0006360 | transcription from RNA polymerase I prom. | 54 | 3 | 2.06 | 0.33863 |
| GO:0050764 | regulation of phagocytosis | 54 | 3 | 2.06 | 0.33863 |
| GO:0050772 | positive regulation of axonogenesis | 54 | 3 | 2.06 | 0.33863 |
| GO:0060419 | heart growth | 54 | 3 | 2.06 | 0.33863 |
| GO:2001022 | positive regulation of response to DNA d... | 54 | 3 | 2.06 | 0.33863 |
| GO:0001666 | response to hypoxia | 247 | 11 | 9.4 | 0.34049 |
| GO:0031214 | biomineral tissue development | 125 | 6 | 4.76 | 0.34064 |
| GO:2001056 | positive regulation of cysteine-type end... | 125 | 6 | 4.76 | 0.34064 |
| GO:0006869 | lipid transport | 272 | 12 | 10.35 | 0.34183 |

| | | | | | |
|---|---|---|---|---|---|
| GO:0019884 | antigen processing and presentation of e... | 174 | 8 | 6.62 | 0.34389 |
| GO:0001578 | microtubule bundle formation | 32 | 2 | 1.22 | 0.34548 |
| GO:0006501 | C-terminal protein lipidation | 32 | 2 | 1.22 | 0.34548 |
| GO:0007157 | heterophilic cell-cell adhesion via plas... | 32 | 2 | 1.22 | 0.34548 |
| GO:0009262 | deoxyribonucleotide metabolic process | 32 | 2 | 1.22 | 0.34548 |
| GO:0014003 | oligodendrocyte development | 32 | 2 | 1.22 | 0.34548 |
| GO:0021696 | cerebellar cortex morphogenesis | 32 | 2 | 1.22 | 0.34548 |
| GO:0030490 | maturation of SSU-rRNA | 32 | 2 | 1.22 | 0.34548 |
| GO:0030818 | negative regulation of cAMP biosynthetic... | 32 | 2 | 1.22 | 0.34548 |
| GO:0031952 | regulation of protein autophosphorylatio... | 32 | 2 | 1.22 | 0.34548 |
| GO:0032309 | icosanoid secretion | 32 | 2 | 1.22 | 0.34548 |
| GO:0032885 | regulation of polysaccharide biosyntheti... | 32 | 2 | 1.22 | 0.34548 |
| GO:0043277 | apoptotic cell clearance | 32 | 2 | 1.22 | 0.34548 |
| GO:0043616 | keratinocyte proliferation | 32 | 2 | 1.22 | 0.34548 |
| GO:0045933 | positive regulation of muscle contractio... | 32 | 2 | 1.22 | 0.34548 |
| GO:0046854 | phosphatidylinositol phosphorylation | 32 | 2 | 1.22 | 0.34548 |
| GO:0048048 | embryonic eye morphogenesis | 32 | 2 | 1.22 | 0.34548 |
| GO:0051693 | actin filament capping | 32 | 2 | 1.22 | 0.34548 |
| GO:0070873 | regulation of glycogen metabolic process | 32 | 2 | 1.22 | 0.34548 |
| GO:0071378 | cellular response to growth hormone stim... | 32 | 2 | 1.22 | 0.34548 |
| GO:0075733 | intracellular transport of virus | 32 | 2 | 1.22 | 0.34548 |
| GO:1902583 | multi-organism intracellular transport | 32 | 2 | 1.22 | 0.34548 |
| GO:0002688 | regulation of leukocyte chemotaxis | 78 | 4 | 2.97 | 0.34565 |
| GO:0021761 | limbic system development | 78 | 4 | 2.97 | 0.34565 |
| GO:0042590 | antigen processing and presentation of e... | 78 | 4 | 2.97 | 0.34565 |
| GO:0043200 | response to amino acid | 78 | 4 | 2.97 | 0.34565 |
| GO:0050709 | negative regulation of protein secretion | 78 | 4 | 2.97 | 0.34565 |
| GO:0032259 | methylation | 273 | 12 | 10.39 | 0.34642 |
| GO:0051147 | regulation of muscle cell differentiatio... | 126 | 6 | 4.8 | 0.34744 |
| GO:0000154 | rRNA modification | 11 | 1 | 0.42 | 0.34757 |
| GO:0000160 | phosphorelay signal transduction system | 11 | 1 | 0.42 | 0.34757 |
| GO:0001911 | negative regulation of leukocyte mediate... | 11 | 1 | 0.42 | 0.34757 |
| GO:0002475 | antigen processing and presentation via ... | 11 | 1 | 0.42 | 0.34757 |
| GO:0002566 | somatic diversification of immune recept... | 11 | 1 | 0.42 | 0.34757 |
| GO:0002674 | negative regulation of acute inflammator... | 11 | 1 | 0.42 | 0.34757 |
| GO:0002710 | negative regulation of T cell mediated i... | 11 | 1 | 0.42 | 0.34757 |
| GO:0002903 | negative regulation of B cell apoptotic ... | 11 | 1 | 0.42 | 0.34757 |
| GO:0003084 | positive regulation of systemic arterial... | 11 | 1 | 0.42 | 0.34757 |
| GO:0003157 | endocardium development | 11 | 1 | 0.42 | 0.34757 |
| GO:0006086 | acetyl-CoA biosynthetic process from pyr... | 11 | 1 | 0.42 | 0.34757 |
| GO:0006105 | succinate metabolic process | 11 | 1 | 0.42 | 0.34757 |
| GO:0007191 | adenylate cyclase-activating dopamine re... | 11 | 1 | 0.42 | 0.34757 |
| GO:0007342 | fusion of sperm to egg plasma membrane | 11 | 1 | 0.42 | 0.34757 |
| GO:0007638 | mechanosensory behavior | 11 | 1 | 0.42 | 0.34757 |
| GO:0008354 | germ cell migration | 11 | 1 | 0.42 | 0.34757 |
| GO:0009071 | serine family amino acid catabolic proce... | 11 | 1 | 0.42 | 0.34757 |
| GO:0010225 | response to UV-C | 11 | 1 | 0.42 | 0.34757 |
| GO:0010447 | response to acidic pH | 11 | 1 | 0.42 | 0.34757 |
| GO:0010510 | regulation of acetyl-CoA biosynthetic pr... | 11 | 1 | 0.42 | 0.34757 |
| GO:0010758 | regulation of macrophage chemotaxis | 11 | 1 | 0.42 | 0.34757 |
| GO:0010832 | negative regulation of myotube different... | 11 | 1 | 0.42 | 0.34757 |
| GO:0016556 | mRNA modification | 11 | 1 | 0.42 | 0.34757 |
| GO:0019054 | modulation by virus of host process | 11 | 1 | 0.42 | 0.34757 |

| | | | | | |
|---|---|---|---|---|---|
| GO:0019852 | L-ascorbic acid metabolic process | 11 | 1 | 0.42 | 0.34757 |
| GO:0022038 | corpus callosum development | 11 | 1 | 0.42 | 0.34757 |
| GO:0031061 | negative regulation of histone methylati... | 11 | 1 | 0.42 | 0.34757 |
| GO:0032308 | positive regulation of prostaglandin sec... | 11 | 1 | 0.42 | 0.34757 |
| GO:0033262 | regulation of nuclear cell cycle DNA rep... | 11 | 1 | 0.42 | 0.34757 |
| GO:0034143 | regulation of toll-like receptor 4 signa... | 11 | 1 | 0.42 | 0.34757 |
| GO:0035112 | genitalia morphogenesis | 11 | 1 | 0.42 | 0.34757 |
| GO:0035269 | protein O-linked mannosylation | 11 | 1 | 0.42 | 0.34757 |
| GO:0035404 | histone-serine phosphorylation | 11 | 1 | 0.42 | 0.34757 |
| GO:0040023 | establishment of nucleus localization | 11 | 1 | 0.42 | 0.34757 |
| GO:0043217 | myelin maintenance | 11 | 1 | 0.42 | 0.34757 |
| GO:0043383 | negative T cell selection | 11 | 1 | 0.42 | 0.34757 |
| GO:0043650 | dicarboxylic acid biosynthetic process | 11 | 1 | 0.42 | 0.34757 |
| GO:0043922 | negative regulation by host of viral tra... | 11 | 1 | 0.42 | 0.34757 |
| GO:0044331 | cell-cell adhesion mediated by cadherin | 11 | 1 | 0.42 | 0.34757 |
| GO:0045019 | negative regulation of nitric oxide bios... | 11 | 1 | 0.42 | 0.34757 |
| GO:0045060 | negative thymic T cell selection | 11 | 1 | 0.42 | 0.34757 |
| GO:0045540 | regulation of cholesterol biosynthetic p... | 11 | 1 | 0.42 | 0.34757 |
| GO:0045603 | positive regulation of endothelial cell ... | 11 | 1 | 0.42 | 0.34757 |
| GO:0045605 | negative regulation of epidermal cell di... | 11 | 1 | 0.42 | 0.34757 |
| GO:0046033 | AMP metabolic process | 11 | 1 | 0.42 | 0.34757 |
| GO:0046838 | phosphorylated carbohydrate dephosphory | 11 | 1 | 0.42 | 0.34757 |
| GO:0048742 | regulation of skeletal muscle fiber deve... | 11 | 1 | 0.42 | 0.34757 |
| GO:0050855 | regulation of B cell receptor signaling ... | 11 | 1 | 0.42 | 0.34757 |
| GO:0055094 | response to lipoprotein particle | 11 | 1 | 0.42 | 0.34757 |
| GO:0060068 | vagina development | 11 | 1 | 0.42 | 0.34757 |
| GO:0060080 | regulation of inhibitory postsynaptic me... | 11 | 1 | 0.42 | 0.34757 |
| GO:0060147 | regulation of posttranscriptional gene s... | 11 | 1 | 0.42 | 0.34757 |
| GO:0060576 | intestinal epithelial cell development | 11 | 1 | 0.42 | 0.34757 |
| GO:0060600 | dichotomous subdivision of an epithelial... | 11 | 1 | 0.42 | 0.34757 |
| GO:0060693 | regulation of branching involved in sali... | 11 | 1 | 0.42 | 0.34757 |
| GO:0060831 | smoothened signaling pathway involved in | 11 | 1 | 0.42 | 0.34757 |
| GO:0060966 | regulation of gene silencing by RNA | 11 | 1 | 0.42 | 0.34757 |
| GO:0061081 | positive regulation of myeloid leukocyte... | 11 | 1 | 0.42 | 0.34757 |
| GO:0070208 | protein heterotrimerization | 11 | 1 | 0.42 | 0.34757 |
| GO:0071243 | cellular response to arsenic-containing ... | 11 | 1 | 0.42 | 0.34757 |
| GO:0071545 | inositol phosphate catabolic process | 11 | 1 | 0.42 | 0.34757 |
| GO:0071711 | basement membrane organization | 11 | 1 | 0.42 | 0.34757 |
| GO:0071872 | cellular response to epinephrine stimulu... | 11 | 1 | 0.42 | 0.34757 |
| GO:0072505 | divalent inorganic anion homeostasis | 11 | 1 | 0.42 | 0.34757 |
| GO:0072608 | interleukin-10 secretion | 11 | 1 | 0.42 | 0.34757 |
| GO:0086069 | bundle of His cell to Purkinje myocyte c... | 11 | 1 | 0.42 | 0.34757 |
| GO:0090330 | regulation of platelet aggregation | 11 | 1 | 0.42 | 0.34757 |
| GO:1900103 | positive regulation of endoplasmic retic... | 11 | 1 | 0.42 | 0.34757 |
| GO:1903206 | negative regulation of hydrogen peroxide.. | 11 | 1 | 0.42 | 0.34757 |
| GO:1990126 | retrograde transport | endosome | 11 | 1 | 0.42 |
| GO:2000258 | negative regulation of protein activatio... | 11 | 1 | 0.42 | 0.34757 |
| GO:2000505 | regulation of energy homeostasis | 11 | 1 | 0.42 | 0.34757 |
| GO:2000641 | regulation of early endosome to late end... | 11 | 1 | 0.42 | 0.34757 |
| GO:2000833 | positive regulation of steroid hormone s... | 11 | 1 | 0.42 | 0.34757 |
| GO:2000846 | regulation of corticosteroid hormone sec... | 11 | 1 | 0.42 | 0.34757 |
| GO:2001044 | regulation of integrin-mediated signalin... | 11 | 1 | 0.42 | 0.34757 |
| GO:0009408 | response to heat | 102 | 5 | 3.88 | 0.34768 |

| | | | | | |
|---|---|---|---|---|---|
| GO:0071156 | regulation of cell cycle arrest | 102 | 5 | 3.88 | 0.34768 |
| GO:0006635 | fatty acid beta-oxidation | 55 | 3 | 2.09 | 0.34914 |
| GO:0031018 | endocrine pancreas development | 55 | 3 | 2.09 | 0.34914 |
| GO:0032272 | negative regulation of protein polymeriz... | 55 | 3 | 2.09 | 0.34914 |
| GO:0032411 | positive regulation of transporter activ... | 55 | 3 | 2.09 | 0.34914 |
| GO:0060485 | mesenchyme development | 200 | 9 | 7.61 | 0.35215 |
| GO:0022412 | cellular process involved in reproductio... | 225 | 10 | 8.56 | 0.35384 |
| GO:0001764 | neuron migration | 127 | 6 | 4.83 | 0.35425 |
| GO:0001708 | cell fate specification | 79 | 4 | 3.01 | 0.35437 |
| GO:0003073 | regulation of systemic arterial blood pr... | 79 | 4 | 3.01 | 0.35437 |
| GO:0006353 | DNA-templated transcription | termination | 79 | 4 | 3.01 |
| GO:0019395 | fatty acid oxidation | 79 | 4 | 3.01 | 0.35437 |
| GO:0051101 | regulation of DNA binding | 79 | 4 | 3.01 | 0.35437 |
| GO:0071331 | cellular response to hexose stimulus | 79 | 4 | 3.01 | 0.35437 |
| GO:0007033 | vacuole organization | 103 | 5 | 3.92 | 0.35528 |
| GO:0051438 | regulation of ubiquitin-protein transfer... | 103 | 5 | 3.92 | 0.35528 |
| GO:0034599 | cellular response to oxidative stress | 176 | 8 | 6.7 | 0.35542 |
| GO:0009416 | response to light stimulus | 325 | 14 | 12.37 | 0.35602 |
| GO:0051090 | regulation of sequence-specific DNA bind.. | 325 | 14 | 12.37 | 0.35602 |
| GO:0006101 | citrate metabolic process | 33 | 2 | 1.26 | 0.35942 |
| GO:0006361 | transcription initiation from RNA polyme... | 33 | 2 | 1.26 | 0.35942 |
| GO:0007520 | myoblast fusion | 33 | 2 | 1.26 | 0.35942 |
| GO:0030803 | negative regulation of cyclic nucleotide... | 33 | 2 | 1.26 | 0.35942 |
| GO:0030857 | negative regulation of epithelial cell d... | 33 | 2 | 1.26 | 0.35942 |
| GO:0031572 | G2 DNA damage checkpoint | 33 | 2 | 1.26 | 0.35942 |
| GO:0046173 | polyol biosynthetic process | 33 | 2 | 1.26 | 0.35942 |
| GO:0031341 | regulation of cell killing | 56 | 3 | 2.13 | 0.35963 |
| GO:0032945 | negative regulation of mononuclear cell ... | 56 | 3 | 2.13 | 0.35963 |
| GO:0050672 | negative regulation of lymphocyte prolif... | 56 | 3 | 2.13 | 0.35963 |
| GO:0061035 | regulation of cartilage development | 56 | 3 | 2.13 | 0.35963 |
| GO:0061097 | regulation of protein tyrosine kinase ac... | 56 | 3 | 2.13 | 0.35963 |
| GO:1901880 | negative regulation of protein depolymer... | 56 | 3 | 2.13 | 0.35963 |
| GO:1901888 | regulation of cell junction assembly | 56 | 3 | 2.13 | 0.35963 |
| GO:0050863 | regulation of T cell activation | 251 | 11 | 9.55 | 0.35978 |
| GO:2000146 | negative regulation of cell motility | 177 | 8 | 6.74 | 0.3612 |
| GO:0005976 | polysaccharide metabolic process | 104 | 5 | 3.96 | 0.36288 |
| GO:0034101 | erythrocyte homeostasis | 104 | 5 | 3.96 | 0.36288 |
| GO:0050777 | negative regulation of immune response | 104 | 5 | 3.96 | 0.36288 |
| GO:0010927 | cellular component assembly involved in .. | 202 | 9 | 7.69 | 0.36296 |
| GO:0048872 | homeostasis of number of cells | 202 | 9 | 7.69 | 0.36296 |
| GO:0022602 | ovulation cycle process | 80 | 4 | 3.05 | 0.36308 |
| GO:0048675 | axon extension | 80 | 4 | 3.05 | 0.36308 |
| GO:0097530 | granulocyte migration | 80 | 4 | 3.05 | 0.36308 |
| GO:1903038 | negative regulation of leukocyte cell-ce... | 80 | 4 | 3.05 | 0.36308 |
| GO:2001252 | positive regulation of chromosome organi.. | 80 | 4 | 3.05 | 0.36308 |
| GO:0006790 | sulfur compound metabolic process | 327 | 14 | 12.45 | 0.36452 |
| GO:0008217 | regulation of blood pressure | 153 | 7 | 5.82 | 0.3647 |
| GO:0016358 | dendrite development | 153 | 7 | 5.82 | 0.3647 |
| GO:0010876 | lipid localization | 302 | 13 | 11.5 | 0.36479 |
| GO:0030072 | peptide hormone secretion | 228 | 10 | 8.68 | 0.36916 |
| GO:0003333 | amino acid transmembrane transport | 57 | 3 | 2.17 | 0.3701 |
| GO:0006636 | unsaturated fatty acid biosynthetic proc... | 57 | 3 | 2.17 | 0.3701 |
| GO:0009142 | nucleoside triphosphate biosynthetic pro... | 57 | 3 | 2.17 | 0.3701 |

| | | | | | |
|---|---|---|---|---|---|
| GO:0010595 | positive regulation of endothelial cell ... | 57 | 3 | 2.17 | 0.3701 |
| GO:0043401 | steroid hormone mediated signaling pathw | 57 | 3 | 2.17 | 0.3701 |
| GO:0061337 | cardiac conduction | 57 | 3 | 2.17 | 0.3701 |
| GO:0051048 | negative regulation of secretion | 154 | 7 | 5.86 | 0.37096 |
| GO:0000723 | telomere maintenance | 81 | 4 | 3.08 | 0.3718 |
| GO:0006493 | protein O-linked glycosylation | 81 | 4 | 3.08 | 0.3718 |
| GO:0032200 | telomere organization | 81 | 4 | 3.08 | 0.3718 |
| GO:0034440 | lipid oxidation | 81 | 4 | 3.08 | 0.3718 |
| GO:0048660 | regulation of smooth muscle cell prolife... | 81 | 4 | 3.08 | 0.3718 |
| GO:0016570 | histone modification | 354 | 15 | 13.48 | 0.3723 |
| GO:0001821 | histamine secretion | 12 | 1 | 0.46 | 0.37242 |
| GO:0002347 | response to tumor cell | 12 | 1 | 0.46 | 0.37242 |
| GO:0002523 | leukocyte migration involved in inflamma... | 12 | 1 | 0.46 | 0.37242 |
| GO:0002921 | negative regulation of humoral immune re. | 12 | 1 | 0.46 | 0.37242 |
| GO:0003188 | heart valve formation | 12 | 1 | 0.46 | 0.37242 |
| GO:0003272 | endocardial cushion formation | 12 | 1 | 0.46 | 0.37242 |
| GO:0003298 | physiological muscle hypertrophy | 12 | 1 | 0.46 | 0.37242 |
| GO:0003301 | physiological cardiac muscle hypertrophy | 12 | 1 | 0.46 | 0.37242 |
| GO:0003306 | Wnt signaling pathway involved in heart ... | 12 | 1 | 0.46 | 0.37242 |
| GO:0006705 | mineralocorticoid biosynthetic process | 12 | 1 | 0.46 | 0.37242 |
| GO:0007183 | SMAD protein complex assembly | 12 | 1 | 0.46 | 0.37242 |
| GO:0007635 | chemosensory behavior | 12 | 1 | 0.46 | 0.37242 |
| GO:0008212 | mineralocorticoid metabolic process | 12 | 1 | 0.46 | 0.37242 |
| GO:0009263 | deoxyribonucleotide biosynthetic process | 12 | 1 | 0.46 | 0.37242 |
| GO:0009415 | response to water | 12 | 1 | 0.46 | 0.37242 |
| GO:0010288 | response to lead ion | 12 | 1 | 0.46 | 0.37242 |
| GO:0015939 | pantothenate metabolic process | 12 | 1 | 0.46 | 0.37242 |
| GO:0019082 | viral protein processing | 12 | 1 | 0.46 | 0.37242 |
| GO:0019359 | nicotinamide nucleotide biosynthetic pro... | 12 | 1 | 0.46 | 0.37242 |
| GO:0019363 | pyridine nucleotide biosynthetic process | 12 | 1 | 0.46 | 0.37242 |
| GO:0019934 | cGMP-mediated signaling | 12 | 1 | 0.46 | 0.37242 |
| GO:0021535 | cell migration in hindbrain | 12 | 1 | 0.46 | 0.37242 |
| GO:0021903 | rostrocaudal neural tube patterning | 12 | 1 | 0.46 | 0.37242 |
| GO:0021978 | telencephalon regionalization | 12 | 1 | 0.46 | 0.37242 |
| GO:0033327 | Leydig cell differentiation | 12 | 1 | 0.46 | 0.37242 |
| GO:0035196 | production of miRNAs involved in gene si.. | 12 | 1 | 0.46 | 0.37242 |
| GO:0035458 | cellular response to interferon-beta | 12 | 1 | 0.46 | 0.37242 |
| GO:0036445 | neuronal stem cell division | 12 | 1 | 0.46 | 0.37242 |
| GO:0036498 | IRE1-mediated unfolded protein response | 12 | 1 | 0.46 | 0.37242 |
| GO:0042574 | retinal metabolic process | 12 | 1 | 0.46 | 0.37242 |
| GO:0042711 | maternal behavior | 12 | 1 | 0.46 | 0.37242 |
| GO:0042754 | negative regulation of circadian rhythm | 12 | 1 | 0.46 | 0.37242 |
| GO:0042953 | lipoprotein transport | 12 | 1 | 0.46 | 0.37242 |
| GO:0043457 | regulation of cellular respiration | 12 | 1 | 0.46 | 0.37242 |
| GO:0043923 | positive regulation by host of viral tra... | 12 | 1 | 0.46 | 0.37242 |
| GO:0043984 | histone H4-K16 acetylation | 12 | 1 | 0.46 | 0.37242 |
| GO:0044788 | modulation by host of viral process | 12 | 1 | 0.46 | 0.37242 |
| GO:0044872 | lipoprotein localization | 12 | 1 | 0.46 | 0.37242 |
| GO:0045414 | regulation of interleukin-8 biosynthetic... | 12 | 1 | 0.46 | 0.37242 |
| GO:0045837 | negative regulation of membrane potentia. | 12 | 1 | 0.46 | 0.37242 |
| GO:0045943 | positive regulation of transcription fro... | 12 | 1 | 0.46 | 0.37242 |
| GO:0046500 | S-adenosylmethionine metabolic process | 12 | 1 | 0.46 | 0.37242 |
| GO:0048490 | anterograde synaptic vesicle transport | 12 | 1 | 0.46 | 0.37242 |

| | | | | | |
|---|---|---|---|---|---|
| GO:0048712 | negative regulation of astrocyte differe... | 12 | 1 | 0.46 | 0.37242 |
| GO:0050812 | regulation of acyl-CoA biosynthetic proc... | 12 | 1 | 0.46 | 0.37242 |
| GO:0050857 | positive regulation of antigen receptor-... | 12 | 1 | 0.46 | 0.37242 |
| GO:0055057 | neuroblast division | 12 | 1 | 0.46 | 0.37242 |
| GO:0055089 | fatty acid homeostasis | 12 | 1 | 0.46 | 0.37242 |
| GO:0060525 | prostate glandular acinus development | 12 | 1 | 0.46 | 0.37242 |
| GO:0060536 | cartilage morphogenesis | 12 | 1 | 0.46 | 0.37242 |
| GO:0060628 | regulation of ER to Golgi vesicle-mediat... | 12 | 1 | 0.46 | 0.37242 |
| GO:0060746 | parental behavior | 12 | 1 | 0.46 | 0.37242 |
| GO:0061049 | cell growth involved in cardiac muscle c... | 12 | 1 | 0.46 | 0.37242 |
| GO:0071600 | otic vesicle morphogenesis | 12 | 1 | 0.46 | 0.37242 |
| GO:0071675 | regulation of mononuclear cell migration | 12 | 1 | 0.46 | 0.37242 |
| GO:0071850 | mitotic cell cycle arrest | 12 | 1 | 0.46 | 0.37242 |
| GO:0071871 | response to epinephrine | 12 | 1 | 0.46 | 0.37242 |
| GO:0072604 | interleukin-6 secretion | 12 | 1 | 0.46 | 0.37242 |
| GO:0086011 | membrane repolarization during action po. | 12 | 1 | 0.46 | 0.37242 |
| GO:0098909 | regulation of cardiac muscle cell action... | 12 | 1 | 0.46 | 0.37242 |
| GO:1901032 | negative regulation of response to react... | 12 | 1 | 0.46 | 0.37242 |
| GO:1902188 | positive regulation of viral release fro... | 12 | 1 | 0.46 | 0.37242 |
| GO:1902358 | sulfate transmembrane transport | 12 | 1 | 0.46 | 0.37242 |
| GO:1903205 | regulation of hydrogen peroxide-induced .. | 12 | 1 | 0.46 | 0.37242 |
| GO:2000105 | positive regulation of DNA-dependent DNA | 12 | 1 | 0.46 | 0.37242 |
| GO:2000251 | positive regulation of actin cytoskeleto... | 12 | 1 | 0.46 | 0.37242 |
| GO:0060047 | heart contraction | 179 | 8 | 6.81 | 0.37279 |
| GO:0050708 | regulation of protein secretion | 329 | 14 | 12.52 | 0.37305 |
| GO:0006890 | retrograde vesicle-mediated transport G... | | 34 | 2 | 1.29 |
| GO:0007032 | endosome organization | 34 | 2 | 1.29 | 0.37325 |
| GO:0007257 | activation of JUN kinase activity | 34 | 2 | 1.29 | 0.37325 |
| GO:0010660 | regulation of muscle cell apoptotic proc... | 34 | 2 | 1.29 | 0.37325 |
| GO:0019068 | virion assembly | 34 | 2 | 1.29 | 0.37325 |
| GO:0031102 | neuron projection regeneration | 34 | 2 | 1.29 | 0.37325 |
| GO:0070206 | protein trimerization | 34 | 2 | 1.29 | 0.37325 |
| GO:0071715 | icosanoid transport | 34 | 2 | 1.29 | 0.37325 |
| GO:1901571 | fatty acid derivative transport | 34 | 2 | 1.29 | 0.37325 |
| GO:0006487 | protein N-linked glycosylation | 130 | 6 | 4.95 | 0.37474 |
| GO:0071478 | cellular response to radiation | 130 | 6 | 4.95 | 0.37474 |
| GO:0019751 | polyol metabolic process | 106 | 5 | 4.03 | 0.3781 |
| GO:0033559 | unsaturated fatty acid metabolic process | 106 | 5 | 4.03 | 0.3781 |
| GO:0051494 | negative regulation of cytoskeleton orga... | 106 | 5 | 4.03 | 0.3781 |
| GO:0051924 | regulation of calcium ion transport | 180 | 8 | 6.85 | 0.37859 |
| GO:1901617 | organic hydroxy compound biosynthetic pr | 205 | 9 | 7.8 | 0.37925 |
| GO:0034470 | ncRNA processing | 230 | 10 | 8.76 | 0.37942 |
| GO:0008652 | cellular amino acid biosynthetic process | 82 | 4 | 3.12 | 0.3805 |
| GO:0042475 | odontogenesis of dentin-containing tooth | 82 | 4 | 3.12 | 0.3805 |
| GO:0007045 | cell-substrate adherens junction assembl.. | 58 | 3 | 2.21 | 0.38053 |
| GO:0009127 | purine nucleoside monophosphate biosynt | 58 | 3 | 2.21 | 0.38053 |
| GO:0009168 | purine ribonucleoside monophosphate bio: | 58 | 3 | 2.21 | 0.38053 |
| GO:0042308 | negative regulation of protein import in... | 58 | 3 | 2.21 | 0.38053 |
| GO:0048041 | focal adhesion assembly | 58 | 3 | 2.21 | 0.38053 |
| GO:0006399 | tRNA metabolic process | 131 | 6 | 4.99 | 0.38158 |
| GO:0006979 | response to oxidative stress | 331 | 14 | 12.6 | 0.38161 |
| GO:0003015 | heart process | 181 | 8 | 6.89 | 0.38441 |
| GO:0042594 | response to starvation | 181 | 8 | 6.89 | 0.38441 |

| | | | | | |
|---|---|---|---|---|---|
| GO:2000116 | regulation of cysteine-type endopeptidas... | 206 | 9 | 7.84 | 0.3847 |
| GO:0002474 | antigen processing and presentation of p... | 107 | 5 | 4.07 | 0.38571 |
| GO:0034332 | adherens junction organization | 107 | 5 | 4.07 | 0.38571 |
| GO:0051250 | negative regulation of lymphocyte activa... | 107 | 5 | 4.07 | 0.38571 |
| GO:0048863 | stem cell differentiation | 332 | 14 | 12.64 | 0.3859 |
| GO:0000387 | spliceosomal snRNP assembly | 35 | 2 | 1.33 | 0.38696 |
| GO:0001755 | neural crest cell migration | 35 | 2 | 1.33 | 0.38696 |
| GO:0006754 | ATP biosynthetic process | 35 | 2 | 1.33 | 0.38696 |
| GO:0008088 | axon cargo transport | 35 | 2 | 1.33 | 0.38696 |
| GO:0017145 | stem cell division | 35 | 2 | 1.33 | 0.38696 |
| GO:0030225 | macrophage differentiation | 35 | 2 | 1.33 | 0.38696 |
| GO:0030809 | negative regulation of nucleotide biosyn... | 35 | 2 | 1.33 | 0.38696 |
| GO:0030835 | negative regulation of actin filament de... | 35 | 2 | 1.33 | 0.38696 |
| GO:0032371 | regulation of sterol transport | 35 | 2 | 1.33 | 0.38696 |
| GO:0032374 | regulation of cholesterol transport | 35 | 2 | 1.33 | 0.38696 |
| GO:0042220 | response to cocaine | 35 | 2 | 1.33 | 0.38696 |
| GO:0048566 | embryonic digestive tract development | 35 | 2 | 1.33 | 0.38696 |
| GO:0060761 | negative regulation of response to cytok... | 35 | 2 | 1.33 | 0.38696 |
| GO:0072528 | pyrimidine-containing compound biosynthe | 35 | 2 | 1.33 | 0.38696 |
| GO:0090278 | negative regulation of peptide hormone s... | 35 | 2 | 1.33 | 0.38696 |
| GO:0097061 | dendritic spine organization | 35 | 2 | 1.33 | 0.38696 |
| GO:1900372 | negative regulation of purine nucleotide... | 35 | 2 | 1.33 | 0.38696 |
| GO:0046488 | phosphatidylinositol metabolic process | 132 | 6 | 5.02 | 0.38842 |
| GO:0032496 | response to lipopolysaccharide | 257 | 11 | 9.78 | 0.38898 |
| GO:1903037 | regulation of leukocyte cell-cell adhesi... | 257 | 11 | 9.78 | 0.38898 |
| GO:0019079 | viral genome replication | 83 | 4 | 3.16 | 0.38919 |
| GO:0048659 | smooth muscle cell proliferation | 83 | 4 | 3.16 | 0.38919 |
| GO:0060021 | palate development | 83 | 4 | 3.16 | 0.38919 |
| GO:0010721 | negative regulation of cell development | 232 | 10 | 8.83 | 0.3897 |
| GO:0006968 | cellular defense response | 59 | 3 | 2.25 | 0.39093 |
| GO:0030148 | sphingolipid biosynthetic process | 59 | 3 | 2.25 | 0.39093 |
| GO:0035914 | skeletal muscle cell differentiation | 59 | 3 | 2.25 | 0.39093 |
| GO:0043507 | positive regulation of JUN kinase activi... | 59 | 3 | 2.25 | 0.39093 |
| GO:0046888 | negative regulation of hormone secretion | 59 | 3 | 2.25 | 0.39093 |
| GO:0070664 | negative regulation of leukocyte prolife... | 59 | 3 | 2.25 | 0.39093 |
| GO:0032387 | negative regulation of intracellular tra... | 108 | 5 | 4.11 | 0.39331 |
| GO:0035303 | regulation of dephosphorylation | 108 | 5 | 4.11 | 0.39331 |
| GO:1990267 | response to transition metal nanoparticl... | 108 | 5 | 4.11 | 0.39331 |
| GO:0016050 | vesicle organization | 233 | 10 | 8.87 | 0.39484 |
| GO:0071214 | cellular response to abiotic stimulus | 233 | 10 | 8.87 | 0.39484 |
| GO:0002703 | regulation of leukocyte mediated immunit... | 133 | 6 | 5.06 | 0.39526 |
| GO:0044782 | cilium organization | 133 | 6 | 5.06 | 0.39526 |
| GO:2001242 | regulation of intrinsic apoptotic signal... | 133 | 6 | 5.06 | 0.39526 |
| GO:0015931 | nucleobase-containing compound transpoı | 158 | 7 | 6.01 | 0.39601 |
| GO:0030258 | lipid modification | 158 | 7 | 6.01 | 0.39601 |
| GO:0001946 | lymphangiogenesis | 13 | 1 | 0.49 | 0.39633 |
| GO:0002691 | regulation of cellular extravasation | 13 | 1 | 0.49 | 0.39633 |
| GO:0006165 | nucleoside diphosphate phosphorylation | 13 | 1 | 0.49 | 0.39633 |
| GO:0007035 | vacuolar acidification | 13 | 1 | 0.49 | 0.39633 |
| GO:0009086 | methionine biosynthetic process | 13 | 1 | 0.49 | 0.39633 |
| GO:0010155 | regulation of proton transport | 13 | 1 | 0.49 | 0.39633 |
| GO:0010569 | regulation of double-strand break repair... | 13 | 1 | 0.49 | 0.39633 |
| GO:0010829 | negative regulation of glucose transport | 13 | 1 | 0.49 | 0.39633 |

| | | | | | |
|---|---|---|---|---|---|
| GO:0021794 | thalamus development | 13 | 1 | 0.49 | 0.39633 |
| GO:0021889 | olfactory bulb interneuron differentiati... | 13 | 1 | 0.49 | 0.39633 |
| GO:0022010 | central nervous system myelination | 13 | 1 | 0.49 | 0.39633 |
| GO:0030252 | growth hormone secretion | 13 | 1 | 0.49 | 0.39633 |
| GO:0030299 | intestinal cholesterol absorption | 13 | 1 | 0.49 | 0.39633 |
| GO:0031050 | dsRNA fragmentation | 13 | 1 | 0.49 | 0.39633 |
| GO:0031065 | positive regulation of histone deacetyla... | 13 | 1 | 0.49 | 0.39633 |
| GO:0031342 | negative regulation of cell killing | 13 | 1 | 0.49 | 0.39633 |
| GO:0031998 | regulation of fatty acid beta-oxidation | 13 | 1 | 0.49 | 0.39633 |
| GO:0032291 | axon ensheathment in central nervous sys | 13 | 1 | 0.49 | 0.39633 |
| GO:0032656 | regulation of interleukin-13 production | 13 | 1 | 0.49 | 0.39633 |
| GO:0035024 | negative regulation of Rho protein signa... | 13 | 1 | 0.49 | 0.39633 |
| GO:0035435 | phosphate ion transmembrane transport | 13 | 1 | 0.49 | 0.39633 |
| GO:0035810 | positive regulation of urine volume | 13 | 1 | 0.49 | 0.39633 |
| GO:0035815 | positive regulation of renal sodium excr... | 13 | 1 | 0.49 | 0.39633 |
| GO:0040015 | negative regulation of multicellular org... | 13 | 1 | 0.49 | 0.39633 |
| GO:0042074 | cell migration involved in gastrulation | 13 | 1 | 0.49 | 0.39633 |
| GO:0042228 | interleukin-8 biosynthetic process | 13 | 1 | 0.49 | 0.39633 |
| GO:0042510 | regulation of tyrosine phosphorylation o... | 13 | 1 | 0.49 | 0.39633 |
| GO:0042789 | mRNA transcription from RNA polymerase | 13 | 1 | 0.49 | 0.39633 |
| GO:0043252 | sodium-independent organic anion transpc | 13 | 1 | 0.49 | 0.39633 |
| GO:0043517 | positive regulation of DNA damage respon | 13 | 1 | 0.49 | 0.39633 |
| GO:0045026 | plasma membrane fusion | 13 | 1 | 0.49 | 0.39633 |
| GO:0045066 | regulatory T cell differentiation | 13 | 1 | 0.49 | 0.39633 |
| GO:0045780 | positive regulation of bone resorption | 13 | 1 | 0.49 | 0.39633 |
| GO:0045836 | positive regulation of meiotic nuclear d... | 13 | 1 | 0.49 | 0.39633 |
| GO:0046321 | positive regulation of fatty acid oxidat... | 13 | 1 | 0.49 | 0.39633 |
| GO:0046541 | saliva secretion | 13 | 1 | 0.49 | 0.39633 |
| GO:0046852 | positive regulation of bone remodeling | 13 | 1 | 0.49 | 0.39633 |
| GO:0048670 | regulation of collateral sprouting | 13 | 1 | 0.49 | 0.39633 |
| GO:0048934 | peripheral nervous system neuron differe.. | 13 | 1 | 0.49 | 0.39633 |
| GO:0048935 | peripheral nervous system neuron develop | 13 | 1 | 0.49 | 0.39633 |
| GO:0050860 | negative regulation of T cell receptor s... | 13 | 1 | 0.49 | 0.39633 |
| GO:0060707 | trophoblast giant cell differentiation | 13 | 1 | 0.49 | 0.39633 |
| GO:0061430 | bone trabecula morphogenesis | 13 | 1 | 0.49 | 0.39633 |
| GO:0070234 | positive regulation of T cell apoptotic ... | 13 | 1 | 0.49 | 0.39633 |
| GO:0070918 | production of small RNA involved in gene.. | 13 | 1 | 0.49 | 0.39633 |
| GO:0070986 | left/right axis specification | 13 | 1 | 0.49 | 0.39633 |
| GO:0071236 | cellular response to antibiotic | 13 | 1 | 0.49 | 0.39633 |
| GO:0071397 | cellular response to cholesterol | 13 | 1 | 0.49 | 0.39633 |
| GO:0071542 | dopaminergic neuron differentiation | 13 | 1 | 0.49 | 0.39633 |
| GO:0071625 | vocalization behavior | 13 | 1 | 0.49 | 0.39633 |
| GO:0071636 | positive regulation of transforming grow... | 13 | 1 | 0.49 | 0.39633 |
| GO:0089711 | L-glutamate transmembrane transport | 13 | 1 | 0.49 | 0.39633 |
| GO:0090030 | regulation of steroid hormone biosynthet... | 13 | 1 | 0.49 | 0.39633 |
| GO:0097320 | membrane tubulation | 13 | 1 | 0.49 | 0.39633 |
| GO:1903019 | negative regulation of glycoprotein meta... | 13 | 1 | 0.49 | 0.39633 |
| GO:2000095 | regulation of Wnt signaling pathway | pla... | 13 | 1 | 0.49 |
| GO:2001212 | regulation of vasculogenesis | 13 | 1 | 0.49 | 0.39633 |
| GO:0016569 | covalent chromatin modification | 360 | 15 | 13.7 | 0.39711 |
| GO:0050769 | positive regulation of neurogenesis | 284 | 12 | 10.81 | 0.39741 |
| GO:0002698 | negative regulation of immune effector p... | 84 | 4 | 3.2 | 0.39787 |
| GO:0008033 | tRNA processing | 84 | 4 | 3.2 | 0.39787 |

| | | | | | |
|---|---|---|---|---|---|
| GO:1990138 | neuron projection extension | 84 | 4 | 3.2 | 0.39787 |
| GO:0060070 | canonical Wnt signaling pathway | 259 | 11 | 9.86 | 0.39876 |
| GO:0006650 | glycerophospholipid metabolic process | 234 | 10 | 8.91 | 0.39999 |
| GO:0002792 | negative regulation of peptide secretion | 36 | 2 | 1.37 | 0.40053 |
| GO:0006040 | amino sugar metabolic process | 36 | 2 | 1.37 | 0.40053 |
| GO:0006369 | termination of RNA polymerase II transcr... | 36 | 2 | 1.37 | 0.40053 |
| GO:0007205 | protein kinase C-activating G-protein co... | 36 | 2 | 1.37 | 0.40053 |
| GO:0015682 | ferric iron transport | 36 | 2 | 1.37 | 0.40053 |
| GO:0033260 | nuclear DNA replication | 36 | 2 | 1.37 | 0.40053 |
| GO:0033628 | regulation of cell adhesion mediated by ... | 36 | 2 | 1.37 | 0.40053 |
| GO:0042516 | regulation of tyrosine phosphorylation o... | 36 | 2 | 1.37 | 0.40053 |
| GO:0061178 | regulation of insulin secretion involved... | 36 | 2 | 1.37 | 0.40053 |
| GO:0061647 | histone H3-K9 modification | 36 | 2 | 1.37 | 0.40053 |
| GO:0072512 | trivalent inorganic cation transport | 36 | 2 | 1.37 | 0.40053 |
| GO:1901532 | regulation of hematopoietic progenitor c... | 36 | 2 | 1.37 | 0.40053 |
| GO:1903313 | positive regulation of mRNA metabolic pr... | 36 | 2 | 1.37 | 0.40053 |
| GO:0030307 | positive regulation of cell growth | 109 | 5 | 4.15 | 0.40091 |
| GO:0030856 | regulation of epithelial cell differenti... | 109 | 5 | 4.15 | 0.40091 |
| GO:0043279 | response to alkaloid | 109 | 5 | 4.15 | 0.40091 |
| GO:0051340 | regulation of ligase activity | 109 | 5 | 4.15 | 0.40091 |
| GO:0061041 | regulation of wound healing | 109 | 5 | 4.15 | 0.40091 |
| GO:0003151 | outflow tract morphogenesis | 60 | 3 | 2.28 | 0.40129 |
| GO:0003208 | cardiac ventricle morphogenesis | 60 | 3 | 2.28 | 0.40129 |
| GO:0006672 | ceramide metabolic process | 60 | 3 | 2.28 | 0.40129 |
| GO:0007585 | respiratory gaseous exchange | 60 | 3 | 2.28 | 0.40129 |
| GO:0019369 | arachidonic acid metabolic process | 60 | 3 | 2.28 | 0.40129 |
| GO:0033692 | cellular polysaccharide biosynthetic pro... | 60 | 3 | 2.28 | 0.40129 |
| GO:0043242 | negative regulation of protein complex d... | 60 | 3 | 2.28 | 0.40129 |
| GO:0048477 | oogenesis | 60 | 3 | 2.28 | 0.40129 |
| GO:0045927 | positive regulation of growth | 184 | 8 | 7 | 0.40186 |
| GO:0007259 | JAK-STAT cascade | 134 | 6 | 5.1 | 0.4021 |
| GO:0045333 | cellular respiration | 159 | 7 | 6.05 | 0.40228 |
| GO:0048738 | cardiac muscle tissue development | 159 | 7 | 6.05 | 0.40228 |
| GO:0002790 | peptide secretion | 235 | 10 | 8.95 | 0.40515 |
| GO:0002576 | platelet degranulation | 85 | 4 | 3.24 | 0.40652 |
| GO:0030010 | establishment of cell polarity | 85 | 4 | 3.24 | 0.40652 |
| GO:0042752 | regulation of circadian rhythm | 85 | 4 | 3.24 | 0.40652 |
| GO:0000209 | protein polyubiquitination | 185 | 8 | 7.04 | 0.40768 |
| GO:0055067 | monovalent inorganic cation homeostasis | 110 | 5 | 4.19 | 0.40849 |
| GO:0009123 | nucleoside monophosphate metabolic proc | 160 | 7 | 6.09 | 0.40855 |
| GO:0017038 | protein import | 261 | 11 | 9.94 | 0.40855 |
| GO:0007188 | adenylate cyclase-modulating G-protein c. | 135 | 6 | 5.14 | 0.40893 |
| GO:0010950 | positive regulation of endopeptidase act... | 135 | 6 | 5.14 | 0.40893 |
| GO:0070925 | organelle assembly | 389 | 16 | 14.81 | 0.41148 |
| GO:0016525 | negative regulation of angiogenesis | 61 | 3 | 2.32 | 0.41159 |
| GO:0034605 | cellular response to heat | 61 | 3 | 2.32 | 0.41159 |
| GO:0050768 | negative regulation of neurogenesis | 186 | 8 | 7.08 | 0.4135 |
| GO:0001736 | establishment of planar polarity | 37 | 2 | 1.41 | 0.41396 |
| GO:0001912 | positive regulation of leukocyte mediate... | 37 | 2 | 1.41 | 0.41396 |
| GO:0006901 | vesicle coating | 37 | 2 | 1.41 | 0.41396 |
| GO:0007164 | establishment of tissue polarity | 37 | 2 | 1.41 | 0.41396 |
| GO:0016241 | regulation of macroautophagy | 37 | 2 | 1.41 | 0.41396 |
| GO:0018410 | C-terminal protein amino acid modificati... | 37 | 2 | 1.41 | 0.41396 |

| | | | | | |
|---|---|---|---|---|---|
| GO:0030317 | sperm motility | 37 | 2 | 1.41 | 0.41396 |
| GO:0035094 | response to nicotine | 37 | 2 | 1.41 | 0.41396 |
| GO:0043551 | regulation of phosphatidylinositol 3-kin... | 37 | 2 | 1.41 | 0.41396 |
| GO:0045429 | positive regulation of nitric oxide bios... | 37 | 2 | 1.41 | 0.41396 |
| GO:0046834 | lipid phosphorylation | 37 | 2 | 1.41 | 0.41396 |
| GO:0050766 | positive regulation of phagocytosis | 37 | 2 | 1.41 | 0.41396 |
| GO:0051445 | regulation of meiotic cell cycle | 37 | 2 | 1.41 | 0.41396 |
| GO:0070373 | negative regulation of ERK1 and ERK2 ca: | 37 | 2 | 1.41 | 0.41396 |
| GO:0071277 | cellular response to calcium ion | 37 | 2 | 1.41 | 0.41396 |
| GO:0090102 | cochlea development | 37 | 2 | 1.41 | 0.41396 |
| GO:1903201 | regulation of oxidative stress-induced c... | 37 | 2 | 1.41 | 0.41396 |
| GO:2001021 | negative regulation of response to DNA d.. | 37 | 2 | 1.41 | 0.41396 |
| GO:0010634 | positive regulation of epithelial cell m... | 86 | 4 | 3.27 | 0.41516 |
| GO:0019233 | sensory perception of pain | 86 | 4 | 3.27 | 0.41516 |
| GO:0035821 | modification of morphology or physiology... | 86 | 4 | 3.27 | 0.41516 |
| GO:0038034 | signal transduction in absence of ligand | 86 | 4 | 3.27 | 0.41516 |
| GO:0097192 | extrinsic apoptotic signaling pathway in... | 86 | 4 | 3.27 | 0.41516 |
| GO:0043542 | endothelial cell migration | 136 | 6 | 5.18 | 0.41576 |
| GO:0042177 | negative regulation of protein catabolic... | 111 | 5 | 4.23 | 0.41607 |
| GO:0001573 | ganglioside metabolic process | 14 | 1 | 0.53 | 0.41933 |
| GO:0002335 | mature B cell differentiation | 14 | 1 | 0.53 | 0.41933 |
| GO:0003148 | outflow tract septum morphogenesis | 14 | 1 | 0.53 | 0.41933 |
| GO:0003299 | muscle hypertrophy in response to stress | 14 | 1 | 0.53 | 0.41933 |
| GO:0006085 | acetyl-CoA biosynthetic process | 14 | 1 | 0.53 | 0.41933 |
| GO:0006376 | mRNA splice site selection | 14 | 1 | 0.53 | 0.41933 |
| GO:0006607 | NLS-bearing protein import into nucleus | 14 | 1 | 0.53 | 0.41933 |
| GO:0006750 | glutathione biosynthetic process | 14 | 1 | 0.53 | 0.41933 |
| GO:0008272 | sulfate transport | 14 | 1 | 0.53 | 0.41933 |
| GO:0010544 | negative regulation of platelet activati... | 14 | 1 | 0.53 | 0.41933 |
| GO:0010640 | regulation of platelet-derived growth fa... | 14 | 1 | 0.53 | 0.41933 |
| GO:0010649 | regulation of cell communication by elec... | 14 | 1 | 0.53 | 0.41933 |
| GO:0010893 | positive regulation of steroid biosynthe... | 14 | 1 | 0.53 | 0.41933 |
| GO:0014898 | cardiac muscle hypertrophy in response t.. | 14 | 1 | 0.53 | 0.41933 |
| GO:0021801 | cerebral cortex radial glia guided migra... | 14 | 1 | 0.53 | 0.41933 |
| GO:0021877 | forebrain neuron fate commitment | 14 | 1 | 0.53 | 0.41933 |
| GO:0022030 | telencephalon glial cell migration | 14 | 1 | 0.53 | 0.41933 |
| GO:0030852 | regulation of granulocyte differentiatio... | 14 | 1 | 0.53 | 0.41933 |
| GO:0030889 | negative regulation of B cell proliferat... | 14 | 1 | 0.53 | 0.41933 |
| GO:0032616 | interleukin-13 production | 14 | 1 | 0.53 | 0.41933 |
| GO:0032799 | low-density lipoprotein receptor particl... | 14 | 1 | 0.53 | 0.41933 |
| GO:0033189 | response to vitamin A | 14 | 1 | 0.53 | 0.41933 |
| GO:0034123 | positive regulation of toll-like recepto... | 14 | 1 | 0.53 | 0.41933 |
| GO:0034629 | cellular protein complex localization | 14 | 1 | 0.53 | 0.41933 |
| GO:0035268 | protein mannosylation | 14 | 1 | 0.53 | 0.41933 |
| GO:0036303 | lymph vessel morphogenesis | 14 | 1 | 0.53 | 0.41933 |
| GO:0036474 | cell death in response to hydrogen perox... | 14 | 1 | 0.53 | 0.41933 |
| GO:0040037 | negative regulation of fibroblast growth... | 14 | 1 | 0.53 | 0.41933 |
| GO:0045005 | DNA-dependent DNA replication maintena | 14 | 1 | 0.53 | 0.41933 |
| GO:0045683 | negative regulation of epidermis develop... | 14 | 1 | 0.53 | 0.41933 |
| GO:0045725 | positive regulation of glycogen biosynth... | 14 | 1 | 0.53 | 0.41933 |
| GO:0045910 | negative regulation of DNA recombination | 14 | 1 | 0.53 | 0.41933 |
| GO:0046827 | positive regulation of protein export fr... | 14 | 1 | 0.53 | 0.41933 |
| GO:0051446 | positive regulation of meiotic cell cycl... | 14 | 1 | 0.53 | 0.41933 |

| | | | | | |
|---|---|---|---|---|---|
| GO:0051608 | histamine transport | 14 | 1 | 0.53 | 0.41933 |
| GO:0051770 | positive regulation of nitric-oxide synt... | 14 | 1 | 0.53 | 0.41933 |
| GO:0060039 | pericardium development | 14 | 1 | 0.53 | 0.41933 |
| GO:0070307 | lens fiber cell development | 14 | 1 | 0.53 | 0.41933 |
| GO:0071674 | mononuclear cell migration | 14 | 1 | 0.53 | 0.41933 |
| GO:0086012 | membrane depolarization during cardiac m | 14 | 1 | 0.53 | 0.41933 |
| GO:0090005 | negative regulation of establishment of ... | 14 | 1 | 0.53 | 0.41933 |
| GO:1902307 | positive regulation of sodium ion transm... | 14 | 1 | 0.53 | 0.41933 |
| GO:1902745 | positive regulation of lamellipodium org... | 14 | 1 | 0.53 | 0.41933 |
| GO:1903427 | negative regulation of reactive oxygen s... | 14 | 1 | 0.53 | 0.41933 |
| GO:0009880 | embryonic pattern specification | 62 | 3 | 2.36 | 0.42184 |
| GO:1903531 | negative regulation of secretion by cell | 137 | 6 | 5.22 | 0.42257 |
| GO:0007589 | body fluid secretion | 87 | 4 | 3.31 | 0.42376 |
| GO:0051443 | positive regulation of ubiquitin-protein... | 87 | 4 | 3.31 | 0.42376 |
| GO:0006909 | phagocytosis | 188 | 8 | 7.16 | 0.42513 |
| GO:0030168 | platelet activation | 239 | 10 | 9.1 | 0.42577 |
| GO:0006312 | mitotic recombination | 38 | 2 | 1.45 | 0.42724 |
| GO:0010657 | muscle cell apoptotic process | 38 | 2 | 1.45 | 0.42724 |
| GO:0032480 | negative regulation of type I interferon... | 38 | 2 | 1.45 | 0.42724 |
| GO:0032881 | regulation of polysaccharide metabolic p... | 38 | 2 | 1.45 | 0.42724 |
| GO:0042503 | tyrosine phosphorylation of Stat3 protei... | 38 | 2 | 1.45 | 0.42724 |
| GO:0042992 | negative regulation of transcription fac... | 38 | 2 | 1.45 | 0.42724 |
| GO:0044246 | regulation of multicellular organismal m... | 38 | 2 | 1.45 | 0.42724 |
| GO:0045058 | T cell selection | 38 | 2 | 1.45 | 0.42724 |
| GO:0045604 | regulation of epidermal cell differentia... | 38 | 2 | 1.45 | 0.42724 |
| GO:0060603 | mammary gland duct morphogenesis | 38 | 2 | 1.45 | 0.42724 |
| GO:1903727 | positive regulation of phospholipid meta... | 38 | 2 | 1.45 | 0.42724 |
| GO:0051146 | striated muscle cell differentiation | 214 | 9 | 8.15 | 0.42833 |
| GO:1903522 | regulation of blood circulation | 214 | 9 | 8.15 | 0.42833 |
| GO:0048639 | positive regulation of developmental gro... | 113 | 5 | 4.3 | 0.43116 |
| GO:0007189 | adenylate cyclase-activating G-protein c... | 63 | 3 | 2.4 | 0.43202 |
| GO:0090559 | regulation of membrane permeability | 63 | 3 | 2.4 | 0.43202 |
| GO:2000181 | negative regulation of blood vessel morp... | 63 | 3 | 2.4 | 0.43202 |
| GO:0031124 | mRNA 3'-end processing | 88 | 4 | 3.35 | 0.43233 |
| GO:0072329 | monocarboxylic acid catabolic process | 88 | 4 | 3.35 | 0.43233 |
| GO:0050808 | synapse organization | 164 | 7 | 6.24 | 0.43356 |
| GO:0002244 | hematopoietic progenitor cell differenti... | 139 | 6 | 5.29 | 0.43617 |
| GO:0002699 | positive regulation of immune effector p... | 139 | 6 | 5.29 | 0.43617 |
| GO:0019932 | second-messenger-mediated signaling | 190 | 8 | 7.23 | 0.43674 |
| GO:0051271 | negative regulation of cellular componen... | 190 | 8 | 7.23 | 0.43674 |
| GO:0001701 | in utero embryonic development | 344 | 14 | 13.09 | 0.43762 |
| GO:0006275 | regulation of DNA replication | 114 | 5 | 4.34 | 0.43868 |
| GO:0002448 | mast cell mediated immunity | 39 | 2 | 1.48 | 0.44036 |
| GO:0007077 | mitotic nuclear envelope disassembly | 39 | 2 | 1.48 | 0.44036 |
| GO:0007595 | lactation | 39 | 2 | 1.48 | 0.44036 |
| GO:0031076 | embryonic camera-type eye development | 39 | 2 | 1.48 | 0.44036 |
| GO:0035306 | positive regulation of dephosphorylation | 39 | 2 | 1.48 | 0.44036 |
| GO:0035773 | insulin secretion involved in cellular r... | 39 | 2 | 1.48 | 0.44036 |
| GO:0045123 | cellular extravasation | 39 | 2 | 1.48 | 0.44036 |
| GO:0051055 | negative regulation of lipid biosyntheti... | 39 | 2 | 1.48 | 0.44036 |
| GO:0060420 | regulation of heart growth | 39 | 2 | 1.48 | 0.44036 |
| GO:0060986 | endocrine hormone secretion | 39 | 2 | 1.48 | 0.44036 |
| GO:1902930 | regulation of alcohol biosynthetic proce... | 39 | 2 | 1.48 | 0.44036 |

| | | | | | |
|---|---|---|---|---|---|
| GO:1903428 | positive regulation of reactive oxygen s... | 39 | 2 | 1.48 | 0.44036 |
| GO:0097194 | execution phase of apoptosis | 89 | 4 | 3.39 | 0.44086 |
| GO:1901606 | alpha-amino acid catabolic process | 89 | 4 | 3.39 | 0.44086 |
| GO:0000042 | protein targeting to Golgi | 15 | 1 | 0.57 | 0.44146 |
| GO:0002724 | regulation of T cell cytokine production | 15 | 1 | 0.57 | 0.44146 |
| GO:0003085 | negative regulation of systemic arterial... | 15 | 1 | 0.57 | 0.44146 |
| GO:0003181 | atrioventricular valve morphogenesis | 15 | 1 | 0.57 | 0.44146 |
| GO:0006004 | fucose metabolic process | 15 | 1 | 0.57 | 0.44146 |
| GO:0006544 | glycine metabolic process | 15 | 1 | 0.57 | 0.44146 |
| GO:0006895 | Golgi to endosome transport | 15 | 1 | 0.57 | 0.44146 |
| GO:0006957 | complement activation | alternative | | 15 | 1 | 0.57 |
| GO:0006978 | DNA damage response | signal trans | 15 | 1 | 0.57 |
| GO:0007095 | mitotic G2 DNA damage checkpoint | 15 | 1 | 0.57 | 0.44146 |
| GO:0007158 | neuron cell-cell adhesion | 15 | 1 | 0.57 | 0.44146 |
| GO:0007512 | adult heart development | 15 | 1 | 0.57 | 0.44146 |
| GO:0014887 | cardiac muscle adaptation | 15 | 1 | 0.57 | 0.44146 |
| GO:0018195 | peptidyl-arginine modification | 15 | 1 | 0.57 | 0.44146 |
| GO:0021756 | striatum development | 15 | 1 | 0.57 | 0.44146 |
| GO:0031293 | membrane protein intracellular domain pr.. | 15 | 1 | 0.57 | 0.44146 |
| GO:0031649 | heat generation | 15 | 1 | 0.57 | 0.44146 |
| GO:0032305 | positive regulation of icosanoid secreti... | 15 | 1 | 0.57 | 0.44146 |
| GO:0032331 | negative regulation of chondrocyte diffe... | 15 | 1 | 0.57 | 0.44146 |
| GO:0032693 | negative regulation of interleukin-10 pr... | 15 | 1 | 0.57 | 0.44146 |
| GO:0032703 | negative regulation of interleukin-2 pro... | 15 | 1 | 0.57 | 0.44146 |
| GO:0033599 | regulation of mammary gland epithelial c... | 15 | 1 | 0.57 | 0.44146 |
| GO:0033962 | cytoplasmic mRNA processing body assen | 15 | 1 | 0.57 | 0.44146 |
| GO:0034661 | ncRNA catabolic process | 15 | 1 | 0.57 | 0.44146 |
| GO:0035930 | corticosteroid hormone secretion | 15 | 1 | 0.57 | 0.44146 |
| GO:0036315 | cellular response to sterol | 15 | 1 | 0.57 | 0.44146 |
| GO:0042508 | tyrosine phosphorylation of Stat1 protei... | 15 | 1 | 0.57 | 0.44146 |
| GO:0043501 | skeletal muscle adaptation | 15 | 1 | 0.57 | 0.44146 |
| GO:0043931 | ossification involved in bone maturation | 15 | 1 | 0.57 | 0.44146 |
| GO:0045601 | regulation of endothelial cell different... | 15 | 1 | 0.57 | 0.44146 |
| GO:0046689 | response to mercury ion | 15 | 1 | 0.57 | 0.44146 |
| GO:0046755 | viral budding | 15 | 1 | 0.57 | 0.44146 |
| GO:0048261 | negative regulation of receptor-mediated... | 15 | 1 | 0.57 | 0.44146 |
| GO:0050884 | neuromuscular process controlling postur.. | 15 | 1 | 0.57 | 0.44146 |
| GO:0051193 | regulation of cofactor metabolic process | 15 | 1 | 0.57 | 0.44146 |
| GO:0051196 | regulation of coenzyme metabolic process | 15 | 1 | 0.57 | 0.44146 |
| GO:0051482 | positive regulation of cytosolic calcium... | 15 | 1 | 0.57 | 0.44146 |
| GO:0060009 | Sertoli cell development | 15 | 1 | 0.57 | 0.44146 |
| GO:0060026 | convergent extension | 15 | 1 | 0.57 | 0.44146 |
| GO:0060442 | branching involved in prostate gland mor... | 15 | 1 | 0.57 | 0.44146 |
| GO:0060969 | negative regulation of gene silencing | 15 | 1 | 0.57 | 0.44146 |
| GO:0070734 | histone H3-K27 methylation | 15 | 1 | 0.57 | 0.44146 |
| GO:0090231 | regulation of spindle checkpoint | 15 | 1 | 0.57 | 0.44146 |
| GO:0090280 | positive regulation of calcium ion impor... | 15 | 1 | 0.57 | 0.44146 |
| GO:1902043 | positive regulation of extrinsic apoptot... | 15 | 1 | 0.57 | 0.44146 |
| GO:1902590 | multi-organism organelle organization | 15 | 1 | 0.57 | 0.44146 |
| GO:1902592 | multi-organism membrane budding | 15 | 1 | 0.57 | 0.44146 |
| GO:1902931 | negative regulation of alcohol biosynthe... | 15 | 1 | 0.57 | 0.44146 |
| GO:1903779 | regulation of cardiac conduction | 15 | 1 | 0.57 | 0.44146 |
| GO:2000050 | regulation of non-canonical Wnt signalin... | 15 | 1 | 0.57 | 0.44146 |

| | | | | | |
|---|---|---|---|---|---|
| GO:2000114 | regulation of establishment of cell pola... | 15 | 1 | 0.57 | 0.44146 |
| GO:2000171 | negative regulation of dendrite developm... | 15 | 1 | 0.57 | 0.44146 |
| GO:2000269 | regulation of fibroblast apoptotic proce... | 15 | 1 | 0.57 | 0.44146 |
| GO:2000785 | regulation of autophagic vacuole assembl. | 15 | 1 | 0.57 | 0.44146 |
| GO:2001026 | regulation of endothelial cell chemotaxi... | 15 | 1 | 0.57 | 0.44146 |
| GO:0002673 | regulation of acute inflammatory respons... | 64 | 3 | 2.44 | 0.44215 |
| GO:0006977 | DNA damage response | signal trans | 64 | 3 | 2.44 |
| GO:0021872 | forebrain generation of neurons | 64 | 3 | 2.44 | 0.44215 |
| GO:0043484 | regulation of RNA splicing | 64 | 3 | 2.44 | 0.44215 |
| GO:0051784 | negative regulation of nuclear division | 64 | 3 | 2.44 | 0.44215 |
| GO:1900181 | negative regulation of protein localizat... | 64 | 3 | 2.44 | 0.44215 |
| GO:0006260 | DNA replication | 268 | 11 | 10.2 | 0.44284 |
| GO:0010506 | regulation of autophagy | 217 | 9 | 8.26 | 0.44466 |
| GO:0032844 | regulation of homeostatic process | 320 | 13 | 12.18 | 0.44531 |
| GO:0007283 | spermatogenesis | 398 | 16 | 15.15 | 0.44774 |
| GO:0006767 | water-soluble vitamin metabolic process | 90 | 4 | 3.43 | 0.44936 |
| GO:0045598 | regulation of fat cell differentiation | 90 | 4 | 3.43 | 0.44936 |
| GO:1902600 | hydrogen ion transmembrane transport | 90 | 4 | 3.43 | 0.44936 |
| GO:0031647 | regulation of protein stability | 141 | 6 | 5.37 | 0.44972 |
| GO:0060271 | cilium morphogenesis | 141 | 6 | 5.37 | 0.44972 |
| GO:0043687 | post-translational protein modification | 244 | 10 | 9.29 | 0.45149 |
| GO:0048232 | male gamete generation | 399 | 16 | 15.19 | 0.45177 |
| GO:0001756 | somitogenesis | 65 | 3 | 2.47 | 0.4522 |
| GO:0072431 | signal transduction involved in mitotic ... | 65 | 3 | 2.47 | 0.4522 |
| GO:1902400 | intracellular signal transduction involv... | 65 | 3 | 2.47 | 0.4522 |
| GO:2000648 | positive regulation of stem cell prolife... | 65 | 3 | 2.47 | 0.4522 |
| GO:0002478 | antigen processing and presentation of e... | 167 | 7 | 6.36 | 0.45223 |
| GO:0051224 | negative regulation of protein transport | 167 | 7 | 6.36 | 0.45223 |
| GO:0045600 | positive regulation of fat cell differen... | 40 | 2 | 1.52 | 0.45331 |
| GO:0048641 | regulation of skeletal muscle tissue dev... | 40 | 2 | 1.52 | 0.45331 |
| GO:0050879 | multicellular organismal movement | 40 | 2 | 1.52 | 0.45331 |
| GO:0050881 | musculoskeletal movement | 40 | 2 | 1.52 | 0.45331 |
| GO:0055081 | anion homeostasis | 40 | 2 | 1.52 | 0.45331 |
| GO:0006914 | autophagy | 296 | 12 | 11.27 | 0.45352 |
| GO:0008360 | regulation of cell shape | 116 | 5 | 4.42 | 0.45364 |
| GO:0048588 | developmental cell growth | 116 | 5 | 4.42 | 0.45364 |
| GO:0051896 | regulation of protein kinase B signaling | 116 | 5 | 4.42 | 0.45364 |
| GO:0032271 | regulation of protein polymerization | 142 | 6 | 5.41 | 0.45646 |
| GO:0001667 | ameboidal-type cell migration | 271 | 11 | 10.32 | 0.4575 |
| GO:0044089 | positive regulation of cellular componen... | 271 | 11 | 10.32 | 0.4575 |
| GO:0010811 | positive regulation of cell-substrate ad... | 91 | 4 | 3.46 | 0.45782 |
| GO:0016202 | regulation of striated muscle tissue dev... | 91 | 4 | 3.46 | 0.45782 |
| GO:0019886 | antigen processing and presentation of e... | 91 | 4 | 3.46 | 0.45782 |
| GO:0019058 | viral life cycle | 375 | 15 | 14.27 | 0.45954 |
| GO:0043583 | ear development | 194 | 8 | 7.38 | 0.45987 |
| GO:0032103 | positive regulation of response to exter... | 220 | 9 | 8.37 | 0.46094 |
| GO:0006626 | protein targeting to mitochondrion | 117 | 5 | 4.45 | 0.46109 |
| GO:0006921 | cellular component disassembly involved . | 66 | 3 | 2.51 | 0.46217 |
| GO:0046470 | phosphatidylcholine metabolic process | 66 | 3 | 2.51 | 0.46217 |
| GO:0051436 | negative regulation of ubiquitin-protein... | 66 | 3 | 2.51 | 0.46217 |
| GO:0000301 | retrograde transport | vesicle recy | 16 | 1 | 0.61 |
| GO:0001711 | endodermal cell fate commitment | 16 | 1 | 0.61 | 0.46274 |
| GO:0003351 | epithelial cilium movement | 16 | 1 | 0.61 | 0.46274 |

| | | | | | |
|---|---|---|---|---|---|
| GO:0003417 | growth plate cartilage development | 16 | 1 | 0.61 | 0.46274 |
| GO:0006688 | glycosphingolipid biosynthetic process | 16 | 1 | 0.61 | 0.46274 |
| GO:0007210 | serotonin receptor signaling pathway | 16 | 1 | 0.61 | 0.46274 |
| GO:0007379 | segment specification | 16 | 1 | 0.61 | 0.46274 |
| GO:0009226 | nucleotide-sugar biosynthetic process | 16 | 1 | 0.61 | 0.46274 |
| GO:0010803 | regulation of tumor necrosis factor-medi... | 16 | 1 | 0.61 | 0.46274 |
| GO:0010888 | negative regulation of lipid storage | 16 | 1 | 0.61 | 0.46274 |
| GO:0014821 | phasic smooth muscle contraction | 16 | 1 | 0.61 | 0.46274 |
| GO:0016239 | positive regulation of macroautophagy | 16 | 1 | 0.61 | 0.46274 |
| GO:0030277 | maintenance of gastrointestinal epitheli... | 16 | 1 | 0.61 | 0.46274 |
| GO:0030502 | negative regulation of bone mineralizati... | 16 | 1 | 0.61 | 0.46274 |
| GO:0031579 | membrane raft organization | 16 | 1 | 0.61 | 0.46274 |
| GO:0034616 | response to laminar fluid shear stress | 16 | 1 | 0.61 | 0.46274 |
| GO:0035418 | protein localization to synapse | 16 | 1 | 0.61 | 0.46274 |
| GO:0042559 | pteridine-containing compound biosynthet. | 16 | 1 | 0.61 | 0.46274 |
| GO:0042762 | regulation of sulfur metabolic process | 16 | 1 | 0.61 | 0.46274 |
| GO:0042772 | DNA damage response | signal trans | 16 | 1 | 0.61 |
| GO:0045911 | positive regulation of DNA recombination | 16 | 1 | 0.61 | 0.46274 |
| GO:0045954 | positive regulation of natural killer ce... | 16 | 1 | 0.61 | 0.46274 |
| GO:0046697 | decidualization | 16 | 1 | 0.61 | 0.46274 |
| GO:0048339 | paraxial mesoderm development | 16 | 1 | 0.61 | 0.46274 |
| GO:0050908 | detection of light stimulus involved in ... | 16 | 1 | 0.61 | 0.46274 |
| GO:0050962 | detection of light stimulus involved in ... | 16 | 1 | 0.61 | 0.46274 |
| GO:0051900 | regulation of mitochondrial depolarizati... | 16 | 1 | 0.61 | 0.46274 |
| GO:0055075 | potassium ion homeostasis | 16 | 1 | 0.61 | 0.46274 |
| GO:0060251 | regulation of glial cell proliferation | 16 | 1 | 0.61 | 0.46274 |
| GO:0060575 | intestinal epithelial cell differentiati... | 16 | 1 | 0.61 | 0.46274 |
| GO:0070193 | synaptonemal complex organization | 16 | 1 | 0.61 | 0.46274 |
| GO:0070875 | positive regulation of glycogen metaboli... | 16 | 1 | 0.61 | 0.46274 |
| GO:0070933 | histone H4 deacetylation | 16 | 1 | 0.61 | 0.46274 |
| GO:0072525 | pyridine-containing compound biosyntheti. | 16 | 1 | 0.61 | 0.46274 |
| GO:1902187 | negative regulation of viral release fro... | 16 | 1 | 0.61 | 0.46274 |
| GO:2000136 | regulation of cell proliferation involve... | 16 | 1 | 0.61 | 0.46274 |
| GO:2000193 | positive regulation of fatty acid transp... | 16 | 1 | 0.61 | 0.46274 |
| GO:2000573 | positive regulation of DNA biosynthetic ... | 16 | 1 | 0.61 | 0.46274 |
| GO:2000811 | negative regulation of anoikis | 16 | 1 | 0.61 | 0.46274 |
| GO:2000831 | regulation of steroid hormone secretion | 16 | 1 | 0.61 | 0.46274 |
| GO:0010632 | regulation of epithelial cell migration | 143 | 6 | 5.44 | 0.46319 |
| GO:0045665 | negative regulation of neuron differenti... | 143 | 6 | 5.44 | 0.46319 |
| GO:0006323 | DNA packaging | 169 | 7 | 6.43 | 0.46461 |
| GO:0030100 | regulation of endocytosis | 169 | 7 | 6.43 | 0.46461 |
| GO:0000183 | chromatin silencing at rDNA | 41 | 2 | 1.56 | 0.46609 |
| GO:0006695 | cholesterol biosynthetic process | 41 | 2 | 1.56 | 0.46609 |
| GO:0030397 | membrane disassembly | 41 | 2 | 1.56 | 0.46609 |
| GO:0032330 | regulation of chondrocyte differentiatio... | 41 | 2 | 1.56 | 0.46609 |
| GO:0035176 | social behavior | 41 | 2 | 1.56 | 0.46609 |
| GO:0051081 | nuclear envelope disassembly | 41 | 2 | 1.56 | 0.46609 |
| GO:0051155 | positive regulation of striated muscle c... | 41 | 2 | 1.56 | 0.46609 |
| GO:0051703 | intraspecies interaction between organis... | 41 | 2 | 1.56 | 0.46609 |
| GO:0051851 | modification by host of symbiont morphol.. | 41 | 2 | 1.56 | 0.46609 |
| GO:0060996 | dendritic spine development | 41 | 2 | 1.56 | 0.46609 |
| GO:2000107 | negative regulation of leukocyte apoptot... | 41 | 2 | 1.56 | 0.46609 |
| GO:0007613 | memory | 92 | 4 | 3.5 | 0.46623 |

| | | | | | |
|---|---|---|---|---|---|
| GO:0051351 | positive regulation of ligase activity | 92 | 4 | 3.5 | 0.46623 |
| GO:1901861 | regulation of muscle tissue development | 92 | 4 | 3.5 | 0.46623 |
| GO:0030216 | keratinocyte differentiation | 118 | 5 | 4.49 | 0.4685 |
| GO:0097529 | myeloid leukocyte migration | 118 | 5 | 4.49 | 0.4685 |
| GO:0045017 | glycerolipid biosynthetic process | 222 | 9 | 8.45 | 0.47174 |
| GO:0008589 | regulation of smoothened signaling pathw. | 67 | 3 | 2.55 | 0.47207 |
| GO:0046823 | negative regulation of nucleocytoplasmic... | 67 | 3 | 2.55 | 0.47207 |
| GO:0071260 | cellular response to mechanical stimulus | 67 | 3 | 2.55 | 0.47207 |
| GO:0072413 | signal transduction involved in mitotic ... | 67 | 3 | 2.55 | 0.47207 |
| GO:1901343 | negative regulation of vasculature devel... | 67 | 3 | 2.55 | 0.47207 |
| GO:1902402 | signal transduction involved in mitotic ... | 67 | 3 | 2.55 | 0.47207 |
| GO:1902403 | signal transduction involved in mitotic ... | 67 | 3 | 2.55 | 0.47207 |
| GO:0001678 | cellular glucose homeostasis | 93 | 4 | 3.54 | 0.4746 |
| GO:0002495 | antigen processing and presentation of p... | 93 | 4 | 3.54 | 0.4746 |
| GO:0006661 | phosphatidylinositol biosynthetic proces... | 93 | 4 | 3.54 | 0.4746 |
| GO:0035282 | segmentation | 93 | 4 | 3.54 | 0.4746 |
| GO:0048634 | regulation of muscle organ development | 93 | 4 | 3.54 | 0.4746 |
| GO:0002262 | myeloid cell homeostasis | 119 | 5 | 4.53 | 0.47588 |
| GO:0030833 | regulation of actin filament polymerizat... | 119 | 5 | 4.53 | 0.47588 |
| GO:0009165 | nucleotide biosynthetic process | 301 | 12 | 11.46 | 0.47679 |
| GO:0034976 | response to endoplasmic reticulum stress | 171 | 7 | 6.51 | 0.47693 |
| GO:0071103 | DNA conformation change | 223 | 9 | 8.49 | 0.47713 |
| GO:0000768 | syncytium formation by plasma membrane | 42 | 2 | 1.6 | 0.47869 |
| GO:0002920 | regulation of humoral immune response | 42 | 2 | 1.6 | 0.47869 |
| GO:0006120 | mitochondrial electron transport | NADH t... | 42 | 2 | 1.6 |
| GO:0006893 | Golgi to plasma membrane transport | 42 | 2 | 1.6 | 0.47869 |
| GO:0010508 | positive regulation of autophagy | 42 | 2 | 1.6 | 0.47869 |
| GO:0031343 | positive regulation of cell killing | 42 | 2 | 1.6 | 0.47869 |
| GO:0060324 | face development | 42 | 2 | 1.6 | 0.47869 |
| GO:0086010 | membrane depolarization during action po | 42 | 2 | 1.6 | 0.47869 |
| GO:1900407 | regulation of cellular response to oxida... | 42 | 2 | 1.6 | 0.47869 |
| GO:0045861 | negative regulation of proteolysis | 302 | 12 | 11.5 | 0.48142 |
| GO:1901293 | nucleoside phosphate biosynthetic proces. | 302 | 12 | 11.5 | 0.48142 |
| GO:0048562 | embryonic organ morphogenesis | 276 | 11 | 10.51 | 0.4818 |
| GO:0000271 | polysaccharide biosynthetic process | 68 | 3 | 2.59 | 0.48188 |
| GO:0015918 | sterol transport | 68 | 3 | 2.59 | 0.48188 |
| GO:0030301 | cholesterol transport | 68 | 3 | 2.59 | 0.48188 |
| GO:0034333 | adherens junction assembly | 68 | 3 | 2.59 | 0.48188 |
| GO:0040014 | regulation of multicellular organism gro... | 68 | 3 | 2.59 | 0.48188 |
| GO:0048663 | neuron fate commitment | 68 | 3 | 2.59 | 0.48188 |
| GO:0051153 | regulation of striated muscle cell diffe... | 68 | 3 | 2.59 | 0.48188 |
| GO:0072522 | purine-containing compound biosynthetic . | 250 | 10 | 9.52 | 0.48216 |
| GO:0002504 | antigen processing and presentation of p... | 94 | 4 | 3.58 | 0.48291 |
| GO:0032609 | interferon-gamma production | 94 | 4 | 3.58 | 0.48291 |
| GO:0048813 | dendrite morphogenesis | 94 | 4 | 3.58 | 0.48291 |
| GO:2001251 | negative regulation of chromosome organi | 94 | 4 | 3.58 | 0.48291 |
| GO:0003203 | endocardial cushion morphogenesis | 17 | 1 | 0.65 | 0.48321 |
| GO:0006337 | nucleosome disassembly | 17 | 1 | 0.65 | 0.48321 |
| GO:0006525 | arginine metabolic process | 17 | 1 | 0.65 | 0.48321 |
| GO:0006907 | pinocytosis | 17 | 1 | 0.65 | 0.48321 |
| GO:0007004 | telomere maintenance via telomerase | 17 | 1 | 0.65 | 0.48321 |
| GO:0009148 | pyrimidine nucleoside triphosphate biosy... | 17 | 1 | 0.65 | 0.48321 |
| GO:0009200 | deoxyribonucleoside triphosphate metabol | 17 | 1 | 0.65 | 0.48321 |

| | | | | | |
|---|---|---|---|---|---|
| GO:0009264 | deoxyribonucleotide catabolic process | 17 | 1 | 0.65 | 0.48321 |
| GO:0010738 | regulation of protein kinase A signaling | 17 | 1 | 0.65 | 0.48321 |
| GO:0016137 | glycoside metabolic process | 17 | 1 | 0.65 | 0.48321 |
| GO:0032753 | positive regulation of interleukin-4 pro... | 17 | 1 | 0.65 | 0.48321 |
| GO:0032986 | protein-DNA complex disassembly | 17 | 1 | 0.65 | 0.48321 |
| GO:0033866 | nucleoside bisphosphate biosynthetic pro.. | 17 | 1 | 0.65 | 0.48321 |
| GO:0034030 | ribonucleoside bisphosphate biosynthetic.. | 17 | 1 | 0.65 | 0.48321 |
| GO:0034033 | purine nucleoside bisphosphate biosynthe. | 17 | 1 | 0.65 | 0.48321 |
| GO:0034199 | activation of protein kinase A activity | 17 | 1 | 0.65 | 0.48321 |
| GO:0034383 | low-density lipoprotein particle clearan... | 17 | 1 | 0.65 | 0.48321 |
| GO:0042226 | interleukin-6 biosynthetic process | 17 | 1 | 0.65 | 0.48321 |
| GO:0042776 | mitochondrial ATP synthesis coupled prot.. | 17 | 1 | 0.65 | 0.48321 |
| GO:0043558 | regulation of translational initiation i... | 17 | 1 | 0.65 | 0.48321 |
| GO:0044346 | fibroblast apoptotic process | 17 | 1 | 0.65 | 0.48321 |
| GO:0045663 | positive regulation of myoblast differen... | 17 | 1 | 0.65 | 0.48321 |
| GO:0045745 | positive regulation of G-protein coupled... | 17 | 1 | 0.65 | 0.48321 |
| GO:0046174 | polyol catabolic process | 17 | 1 | 0.65 | 0.48321 |
| GO:0046426 | negative regulation of JAK-STAT cascade | 17 | 1 | 0.65 | 0.48321 |
| GO:0047496 | vesicle transport along microtubule | 17 | 1 | 0.65 | 0.48321 |
| GO:0048668 | collateral sprouting | 17 | 1 | 0.65 | 0.48321 |
| GO:0050858 | negative regulation of antigen receptor-... | 17 | 1 | 0.65 | 0.48321 |
| GO:0055026 | negative regulation of cardiac muscle ti... | 17 | 1 | 0.65 | 0.48321 |
| GO:0055083 | monovalent inorganic anion homeostasis | 17 | 1 | 0.65 | 0.48321 |
| GO:0060292 | long term synaptic depression | 17 | 1 | 0.65 | 0.48321 |
| GO:0060572 | morphogenesis of an epithelial bud | 17 | 1 | 0.65 | 0.48321 |
| GO:0061014 | positive regulation of mRNA catabolic pr... | 17 | 1 | 0.65 | 0.48321 |
| GO:0061323 | cell proliferation involved in heart mor... | 17 | 1 | 0.65 | 0.48321 |
| GO:0070230 | positive regulation of lymphocyte apopto... | 17 | 1 | 0.65 | 0.48321 |
| GO:0070977 | bone maturation | 17 | 1 | 0.65 | 0.48321 |
| GO:0071599 | otic vesicle development | 17 | 1 | 0.65 | 0.48321 |
| GO:0072148 | epithelial cell fate commitment | 17 | 1 | 0.65 | 0.48321 |
| GO:0072600 | establishment of protein localization to... | 17 | 1 | 0.65 | 0.48321 |
| GO:0090023 | positive regulation of neutrophil chemot... | 17 | 1 | 0.65 | 0.48321 |
| GO:0097150 | neuronal stem cell maintenance | 17 | 1 | 0.65 | 0.48321 |
| GO:0097237 | cellular response to toxic substance | 17 | 1 | 0.65 | 0.48321 |
| GO:0098657 | import into cell | 17 | 1 | 0.65 | 0.48321 |
| GO:0098901 | regulation of cardiac muscle cell action... | 17 | 1 | 0.65 | 0.48321 |
| GO:1900101 | regulation of endoplasmic reticulum unfo... | 17 | 1 | 0.65 | 0.48321 |
| GO:1901623 | regulation of lymphocyte chemotaxis | 17 | 1 | 0.65 | 0.48321 |
| GO:2000725 | regulation of cardiac muscle cell differ... | 17 | 1 | 0.65 | 0.48321 |
| GO:0010952 | positive regulation of peptidase activit... | 146 | 6 | 5.56 | 0.48323 |
| GO:0007611 | learning or memory | 199 | 8 | 7.58 | 0.48851 |
| GO:0016054 | organic acid catabolic process | 199 | 8 | 7.58 | 0.48851 |
| GO:0031346 | positive regulation of cell projection o... | 199 | 8 | 7.58 | 0.48851 |
| GO:0046395 | carboxylic acid catabolic process | 199 | 8 | 7.58 | 0.48851 |
| GO:0034728 | nucleosome organization | 147 | 6 | 5.6 | 0.48986 |
| GO:0015992 | proton transport | 121 | 5 | 4.61 | 0.49054 |
| GO:0001754 | eye photoreceptor cell differentiation | 43 | 2 | 1.64 | 0.49111 |
| GO:0009206 | purine ribonucleoside triphosphate biosy... | 43 | 2 | 1.64 | 0.49111 |
| GO:0009311 | oligosaccharide metabolic process | 43 | 2 | 1.64 | 0.49111 |
| GO:0030834 | regulation of actin filament depolymeriz... | 43 | 2 | 1.64 | 0.49111 |
| GO:0042274 | ribosomal small subunit biogenesis | 43 | 2 | 1.64 | 0.49111 |
| GO:0051353 | positive regulation of oxidoreductase ac... | 43 | 2 | 1.64 | 0.49111 |

| | | | | | |
|---|---|---|---|---|---|
| GO:0086065 | cell communication involved in cardiac c... | 43 | 2 | 1.64 | 0.49111 |
| GO:0090003 | regulation of establishment of protein l... | 43 | 2 | 1.64 | 0.49111 |
| GO:0043254 | regulation of protein complex assembly | 278 | 11 | 10.58 | 0.49146 |
| GO:0006888 | ER to Golgi vesicle-mediated transport | 69 | 3 | 2.63 | 0.4916 |
| GO:0033555 | multicellular organismal response to str... | 69 | 3 | 2.63 | 0.4916 |
| GO:0042058 | regulation of epidermal growth factor re... | 69 | 3 | 2.63 | 0.4916 |
| GO:0045833 | negative regulation of lipid metabolic p... | 69 | 3 | 2.63 | 0.4916 |
| GO:0072401 | signal transduction involved in DNA inte... | 69 | 3 | 2.63 | 0.4916 |
| GO:0072422 | signal transduction involved in DNA dama. | 69 | 3 | 2.63 | 0.4916 |
| GO:0035239 | tube morphogenesis | 357 | 14 | 13.59 | 0.49348 |
| GO:0001655 | urogenital system development | 305 | 12 | 11.61 | 0.49528 |
| GO:0018205 | peptidyl-lysine modification | 253 | 10 | 9.63 | 0.49736 |
| GO:0002065 | columnar/cuboidal epithelial cell differ... | 122 | 5 | 4.64 | 0.49781 |
| GO:0006334 | nucleosome assembly | 122 | 5 | 4.64 | 0.49781 |
| GO:0051781 | positive regulation of cell division | 122 | 5 | 4.64 | 0.49781 |
| GO:0046128 | purine ribonucleoside metabolic process | 201 | 8 | 7.65 | 0.49985 |
| GO:0060541 | respiratory system development | 201 | 8 | 7.65 | 0.49985 |
| GO:0006520 | cellular amino acid metabolic process | 385 | 15 | 14.66 | 0.50089 |
| GO:0034504 | protein localization to nucleus | 280 | 11 | 10.66 | 0.50108 |
| GO:0007034 | vacuolar transport | 70 | 3 | 2.66 | 0.50124 |
| GO:0042310 | vasoconstriction | 70 | 3 | 2.66 | 0.50124 |
| GO:0061387 | regulation of extent of cell growth | 70 | 3 | 2.66 | 0.50124 |
| GO:0072395 | signal transduction involved in cell cyc... | 70 | 3 | 2.66 | 0.50124 |
| GO:1901879 | regulation of protein depolymerization | 70 | 3 | 2.66 | 0.50124 |
| GO:0000097 | sulfur amino acid biosynthetic process | 18 | 1 | 0.69 | 0.50291 |
| GO:0001832 | blastocyst growth | 18 | 1 | 0.69 | 0.50291 |
| GO:0002407 | dendritic cell chemotaxis | 18 | 1 | 0.69 | 0.50291 |
| GO:0002717 | positive regulation of natural killer ce... | 18 | 1 | 0.69 | 0.50291 |
| GO:0003171 | atrioventricular valve development | 18 | 1 | 0.69 | 0.50291 |
| GO:0006379 | mRNA cleavage | 18 | 1 | 0.69 | 0.50291 |
| GO:0006385 | transcription elongation from RNA polyme. | 18 | 1 | 0.69 | 0.50291 |
| GO:0006386 | termination of RNA polymerase III transc... | 18 | 1 | 0.69 | 0.50291 |
| GO:0006817 | phosphate ion transport | 18 | 1 | 0.69 | 0.50291 |
| GO:0006825 | copper ion transport | 18 | 1 | 0.69 | 0.50291 |
| GO:0007099 | centriole replication | 18 | 1 | 0.69 | 0.50291 |
| GO:0009084 | glutamine family amino acid biosynthetic... | 18 | 1 | 0.69 | 0.50291 |
| GO:0009950 | dorsal/ventral axis specification | 18 | 1 | 0.69 | 0.50291 |
| GO:0015669 | gas transport | 18 | 1 | 0.69 | 0.50291 |
| GO:0032332 | positive regulation of chondrocyte diffe... | 18 | 1 | 0.69 | 0.50291 |
| GO:0032438 | melanosome organization | 18 | 1 | 0.69 | 0.50291 |
| GO:0032878 | regulation of establishment or maintenan... | 18 | 1 | 0.69 | 0.50291 |
| GO:0034035 | purine ribonucleoside bisphosphate metab | 18 | 1 | 0.69 | 0.50291 |
| GO:0035456 | response to interferon-beta | 18 | 1 | 0.69 | 0.50291 |
| GO:0042119 | neutrophil activation | 18 | 1 | 0.69 | 0.50291 |
| GO:0042659 | regulation of cell fate specification | 18 | 1 | 0.69 | 0.50291 |
| GO:0044088 | regulation of vacuole organization | 18 | 1 | 0.69 | 0.50291 |
| GO:0044241 | lipid digestion | 18 | 1 | 0.69 | 0.50291 |
| GO:0044803 | multi-organism membrane organization | 18 | 1 | 0.69 | 0.50291 |
| GO:0045332 | phospholipid translocation | 18 | 1 | 0.69 | 0.50291 |
| GO:0046386 | deoxyribose phosphate catabolic process | 18 | 1 | 0.69 | 0.50291 |
| GO:0046856 | phosphatidylinositol dephosphorylation | 18 | 1 | 0.69 | 0.50291 |
| GO:0048643 | positive regulation of skeletal muscle t... | 18 | 1 | 0.69 | 0.50291 |
| GO:0050427 | 3'-phosphoadenosine 5'-phosphosulfate m | 18 | 1 | 0.69 | 0.50291 |

| | | | | | |
|---|---|---|---|---|---|
| GO:0051447 | negative regulation of meiotic cell cycl... | 18 | 1 | 0.69 | 0.50291 |
| GO:0051968 | positive regulation of synaptic transmis... | 18 | 1 | 0.69 | 0.50291 |
| GO:0055012 | ventricular cardiac muscle cell differen... | 18 | 1 | 0.69 | 0.50291 |
| GO:0060065 | uterus development | 18 | 1 | 0.69 | 0.50291 |
| GO:0060788 | ectodermal placode formation | 18 | 1 | 0.69 | 0.50291 |
| GO:0070168 | negative regulation of biomineral tissue... | 18 | 1 | 0.69 | 0.50291 |
| GO:0070536 | protein K63-linked deubiquitination | 18 | 1 | 0.69 | 0.50291 |
| GO:0070584 | mitochondrion morphogenesis | 18 | 1 | 0.69 | 0.50291 |
| GO:0071108 | protein K48-linked deubiquitination | 18 | 1 | 0.69 | 0.50291 |
| GO:0071696 | ectodermal placode development | 18 | 1 | 0.69 | 0.50291 |
| GO:0071697 | ectodermal placode morphogenesis | 18 | 1 | 0.69 | 0.50291 |
| GO:1902624 | positive regulation of neutrophil migrat... | 18 | 1 | 0.69 | 0.50291 |
| GO:0030595 | leukocyte chemotaxis | 149 | 6 | 5.67 | 0.50303 |
| GO:0006949 | syncytium formation | 44 | 2 | 1.67 | 0.50334 |
| GO:0009145 | purine nucleoside triphosphate biosynthe.. | 44 | 2 | 1.67 | 0.50334 |
| GO:0032760 | positive regulation of tumor necrosis fa... | 44 | 2 | 1.67 | 0.50334 |
| GO:0042246 | tissue regeneration | 44 | 2 | 1.67 | 0.50334 |
| GO:0045844 | positive regulation of striated muscle t... | 44 | 2 | 1.67 | 0.50334 |
| GO:0048636 | positive regulation of muscle organ deve... | 44 | 2 | 1.67 | 0.50334 |
| GO:0051702 | interaction with symbiont | 44 | 2 | 1.67 | 0.50334 |
| GO:0086001 | cardiac muscle cell action potential | 44 | 2 | 1.67 | 0.50334 |
| GO:0090303 | positive regulation of wound healing | 44 | 2 | 1.67 | 0.50334 |
| GO:0006818 | hydrogen transport | 123 | 5 | 4.68 | 0.50505 |
| GO:0061351 | neural precursor cell proliferation | 123 | 5 | 4.68 | 0.50505 |
| GO:0006457 | protein folding | 202 | 8 | 7.69 | 0.5055 |
| GO:0046879 | hormone secretion | 281 | 11 | 10.7 | 0.50588 |
| GO:0034655 | nucleobase-containing compound cataboli | 360 | 14 | 13.7 | 0.50624 |
| GO:0006664 | glycolipid metabolic process | 97 | 4 | 3.69 | 0.50753 |
| GO:0030705 | cytoskeleton-dependent intracellular tra... | 97 | 4 | 3.69 | 0.50753 |
| GO:0042472 | inner ear morphogenesis | 97 | 4 | 3.69 | 0.50753 |
| GO:0045995 | regulation of embryonic development | 97 | 4 | 3.69 | 0.50753 |
| GO:0009161 | ribonucleoside monophosphate metabolic | 150 | 6 | 5.71 | 0.50957 |
| GO:0010976 | positive regulation of neuron projection... | 150 | 6 | 5.71 | 0.50957 |
| GO:0046849 | bone remodeling | 71 | 3 | 2.7 | 0.51077 |
| GO:0072527 | pyrimidine-containing compound metabolic | 71 | 3 | 2.7 | 0.51077 |
| GO:0042278 | purine nucleoside metabolic process | 203 | 8 | 7.73 | 0.51112 |
| GO:0016125 | sterol metabolic process | 124 | 5 | 4.72 | 0.51224 |
| GO:0033002 | muscle cell proliferation | 124 | 5 | 4.72 | 0.51224 |
| GO:0048706 | embryonic skeletal system development | 124 | 5 | 4.72 | 0.51224 |
| GO:0051168 | nuclear export | 124 | 5 | 4.72 | 0.51224 |
| GO:0001738 | morphogenesis of a polarized epithelium | 45 | 2 | 1.71 | 0.51537 |
| GO:0002763 | positive regulation of myeloid leukocyte... | 45 | 2 | 1.71 | 0.51537 |
| GO:0006418 | tRNA aminoacylation for protein translat... | 45 | 2 | 1.71 | 0.51537 |
| GO:0030195 | negative regulation of blood coagulation | 45 | 2 | 1.71 | 0.51537 |
| GO:0030837 | negative regulation of actin filament po... | 45 | 2 | 1.71 | 0.51537 |
| GO:0043967 | histone H4 acetylation | 45 | 2 | 1.71 | 0.51537 |
| GO:0048008 | platelet-derived growth factor receptor ... | 45 | 2 | 1.71 | 0.51537 |
| GO:0051148 | negative regulation of muscle cell diffe... | 45 | 2 | 1.71 | 0.51537 |
| GO:0055024 | regulation of cardiac muscle tissue deve... | 45 | 2 | 1.71 | 0.51537 |
| GO:1900047 | negative regulation of hemostasis | 45 | 2 | 1.71 | 0.51537 |
| GO:1901863 | positive regulation of muscle tissue dev... | 45 | 2 | 1.71 | 0.51537 |
| GO:1902882 | regulation of response to oxidative stre... | 45 | 2 | 1.71 | 0.51537 |
| GO:1903706 | regulation of hemopoiesis | 283 | 11 | 10.77 | 0.51543 |

| | | | | | |
|---|---|---|---|---|---|
| GO:0002761 | regulation of myeloid leukocyte differen... | 98 | 4 | 3.73 | 0.51562 |
| GO:0007286 | spermatid development | 98 | 4 | 3.73 | 0.51562 |
| GO:0042303 | molting cycle | 98 | 4 | 3.73 | 0.51562 |
| GO:0042633 | hair cycle | 98 | 4 | 3.73 | 0.51562 |
| GO:0009266 | response to temperature stimulus | 151 | 6 | 5.75 | 0.51609 |
| GO:0042886 | amide transport | 310 | 12 | 11.8 | 0.51817 |
| GO:0022612 | gland morphogenesis | 125 | 5 | 4.76 | 0.51939 |
| GO:0002456 | T cell mediated immunity | 72 | 3 | 2.74 | 0.52022 |
| GO:0061180 | mammary gland epithelium development | 72 | 3 | 2.74 | 0.52022 |
| GO:0070555 | response to interleukin-1 | 72 | 3 | 2.74 | 0.52022 |
| GO:1901184 | regulation of ERBB signaling pathway | 72 | 3 | 2.74 | 0.52022 |
| GO:0002369 | T cell cytokine production | 19 | 1 | 0.72 | 0.52185 |
| GO:0002861 | regulation of inflammatory response to a... | 19 | 1 | 0.72 | 0.52185 |
| GO:0006198 | cAMP catabolic process | 19 | 1 | 0.72 | 0.52185 |
| GO:0006471 | protein ADP-ribosylation | 19 | 1 | 0.72 | 0.52185 |
| GO:0007250 | activation of NF-kappaB-inducing kinase .. | 19 | 1 | 0.72 | 0.52185 |
| GO:0007625 | grooming behavior | 19 | 1 | 0.72 | 0.52185 |
| GO:0008053 | mitochondrial fusion | 19 | 1 | 0.72 | 0.52185 |
| GO:0009074 | aromatic amino acid family catabolic pro... | 19 | 1 | 0.72 | 0.52185 |
| GO:0009147 | pyrimidine nucleoside triphosphate metab. | 19 | 1 | 0.72 | 0.52185 |
| GO:0009299 | mRNA transcription | 19 | 1 | 0.72 | 0.52185 |
| GO:0010800 | positive regulation of peptidyl-threonin... | 19 | 1 | 0.72 | 0.52185 |
| GO:0010842 | retina layer formation | 19 | 1 | 0.72 | 0.52185 |
| GO:0014911 | positive regulation of smooth muscle cel... | 19 | 1 | 0.72 | 0.52185 |
| GO:0031498 | chromatin disassembly | 19 | 1 | 0.72 | 0.52185 |
| GO:0033137 | negative regulation of peptidyl-serine p... | 19 | 1 | 0.72 | 0.52185 |
| GO:0034204 | lipid translocation | 19 | 1 | 0.72 | 0.52185 |
| GO:0042522 | regulation of tyrosine phosphorylation o... | 19 | 1 | 0.72 | 0.52185 |
| GO:0046597 | negative regulation of viral entry into ... | 19 | 1 | 0.72 | 0.52185 |
| GO:0048753 | pigment granule organization | 19 | 1 | 0.72 | 0.52185 |
| GO:0050901 | leukocyte tethering or rolling | 19 | 1 | 0.72 | 0.52185 |
| GO:0050919 | negative chemotaxis | 19 | 1 | 0.72 | 0.52185 |
| GO:0060008 | Sertoli cell differentiation | 19 | 1 | 0.72 | 0.52185 |
| GO:0060749 | mammary gland alveolus development | 19 | 1 | 0.72 | 0.52185 |
| GO:0061377 | mammary gland lobule development | 19 | 1 | 0.72 | 0.52185 |
| GO:0090181 | regulation of cholesterol metabolic proc... | 19 | 1 | 0.72 | 0.52185 |
| GO:0097009 | energy homeostasis | 19 | 1 | 0.72 | 0.52185 |
| GO:1901381 | positive regulation of potassium ion tra... | 19 | 1 | 0.72 | 0.52185 |
| GO:1901798 | positive regulation of signal transducti... | 19 | 1 | 0.72 | 0.52185 |
| GO:1902932 | positive regulation of alcohol biosynthe... | 19 | 1 | 0.72 | 0.52185 |
| GO:1903077 | negative regulation of protein localizat... | 19 | 1 | 0.72 | 0.52185 |
| GO:0051052 | regulation of DNA metabolic process | 258 | 10 | 9.82 | 0.52242 |
| GO:0007178 | transmembrane receptor protein serine/th. | 311 | 12 | 11.84 | 0.52272 |
| GO:0031333 | negative regulation of protein complex a... | 99 | 4 | 3.77 | 0.52365 |
| GO:0050905 | neuromuscular process | 99 | 4 | 3.77 | 0.52365 |
| GO:0051592 | response to calcium ion | 99 | 4 | 3.77 | 0.52365 |
| GO:1903509 | liposaccharide metabolic process | 99 | 4 | 3.77 | 0.52365 |
| GO:0002573 | myeloid leukocyte differentiation | 179 | 7 | 6.81 | 0.52535 |
| GO:0006364 | rRNA processing | 126 | 5 | 4.8 | 0.5265 |
| GO:0006956 | complement activation | 46 | 2 | 1.75 | 0.52722 |
| GO:0030042 | actin filament depolymerization | 46 | 2 | 1.75 | 0.52722 |
| GO:0042267 | natural killer cell mediated cytotoxicit... | 46 | 2 | 1.75 | 0.52722 |
| GO:0070059 | intrinsic apoptotic signaling pathway in... | 46 | 2 | 1.75 | 0.52722 |

| | | | | | |
|---|---|---|---|---|---|
| GO:0070231 | T cell apoptotic process | 46 | 2 | 1.75 | 0.52722 |
| GO:1903557 | positive regulation of tumor necrosis fa... | 46 | 2 | 1.75 | 0.52722 |
| GO:0006913 | nucleocytoplasmic transport | 365 | 14 | 13.89 | 0.52732 |
| GO:0033077 | T cell differentiation in thymus | 73 | 3 | 2.78 | 0.52956 |
| GO:0035050 | embryonic heart tube development | 73 | 3 | 2.78 | 0.52956 |
| GO:0046718 | viral entry into host cell | 73 | 3 | 2.78 | 0.52956 |
| GO:0051352 | negative regulation of ligase activity | 73 | 3 | 2.78 | 0.52956 |
| GO:0051444 | negative regulation of ubiquitin-protein... | 73 | 3 | 2.78 | 0.52956 |
| GO:0046883 | regulation of hormone secretion | 233 | 9 | 8.87 | 0.53018 |
| GO:0007281 | germ cell development | 180 | 7 | 6.85 | 0.53129 |
| GO:0006066 | alcohol metabolic process | 366 | 14 | 13.93 | 0.53151 |
| GO:0060326 | cell chemotaxis | 207 | 8 | 7.88 | 0.53341 |
| GO:0009100 | glycoprotein metabolic process | 393 | 15 | 14.96 | 0.53347 |
| GO:0050870 | positive regulation of T cell activation | 181 | 7 | 6.89 | 0.5372 |
| GO:1901657 | glycosyl compound metabolic process | 261 | 10 | 9.94 | 0.53727 |
| GO:0002479 | antigen processing and presentation of e... | 74 | 3 | 2.82 | 0.5388 |
| GO:0007030 | Golgi organization | 74 | 3 | 2.82 | 0.5388 |
| GO:0007044 | cell-substrate junction assembly | 74 | 3 | 2.82 | 0.5388 |
| GO:0032481 | positive regulation of type I interferon... | 74 | 3 | 2.82 | 0.5388 |
| GO:0043407 | negative regulation of MAP kinase activi... | 74 | 3 | 2.82 | 0.5388 |
| GO:0043506 | regulation of JUN kinase activity | 74 | 3 | 2.82 | 0.5388 |
| GO:0009060 | aerobic respiration | 47 | 2 | 1.79 | 0.53886 |
| GO:0033627 | cell adhesion mediated by integrin | 47 | 2 | 1.79 | 0.53886 |
| GO:0043038 | amino acid activation | 47 | 2 | 1.79 | 0.53886 |
| GO:0043039 | tRNA aminoacylation | 47 | 2 | 1.79 | 0.53886 |
| GO:0045576 | mast cell activation | 47 | 2 | 1.79 | 0.53886 |
| GO:0055017 | cardiac muscle tissue growth | 47 | 2 | 1.79 | 0.53886 |
| GO:0071347 | cellular response to interleukin-1 | 47 | 2 | 1.79 | 0.53886 |
| GO:0090382 | phagosome maturation | 47 | 2 | 1.79 | 0.53886 |
| GO:0009914 | hormone transport | 288 | 11 | 10.96 | 0.53905 |
| GO:0071356 | cellular response to tumor necrosis fact... | 101 | 4 | 3.84 | 0.53951 |
| GO:0001991 | regulation of systemic arterial blood pr... | 20 | 1 | 0.76 | 0.54008 |
| GO:0002931 | response to ischemia | 20 | 1 | 0.76 | 0.54008 |
| GO:0006301 | postreplication repair | 20 | 1 | 0.76 | 0.54008 |
| GO:0006303 | double-strand break repair via nonhomolo... | 20 | 1 | 0.76 | 0.54008 |
| GO:0006595 | polyamine metabolic process | 20 | 1 | 0.76 | 0.54008 |
| GO:0007413 | axonal fasciculation | 20 | 1 | 0.76 | 0.54008 |
| GO:0008089 | anterograde axon cargo transport | 20 | 1 | 0.76 | 0.54008 |
| GO:0010591 | regulation of lamellipodium assembly | 20 | 1 | 0.76 | 0.54008 |
| GO:0010669 | epithelial structure maintenance | 20 | 1 | 0.76 | 0.54008 |
| GO:0031290 | retinal ganglion cell axon guidance | 20 | 1 | 0.76 | 0.54008 |
| GO:0032148 | activation of protein kinase B activity | 20 | 1 | 0.76 | 0.54008 |
| GO:0032469 | endoplasmic reticulum calcium ion homeos | 20 | 1 | 0.76 | 0.54008 |
| GO:0032967 | positive regulation of collagen biosynth... | 20 | 1 | 0.76 | 0.54008 |
| GO:0033688 | regulation of osteoblast proliferation | 20 | 1 | 0.76 | 0.54008 |
| GO:0036296 | response to increased oxygen levels | 20 | 1 | 0.76 | 0.54008 |
| GO:0036342 | post-anal tail morphogenesis | 20 | 1 | 0.76 | 0.54008 |
| GO:0042347 | negative regulation of NF-kappaB import ... | 20 | 1 | 0.76 | 0.54008 |
| GO:0043921 | modulation by host of viral transcriptio... | 20 | 1 | 0.76 | 0.54008 |
| GO:0044550 | secondary metabolite biosynthetic proces... | 20 | 1 | 0.76 | 0.54008 |
| GO:0045649 | regulation of macrophage differentiation | 20 | 1 | 0.76 | 0.54008 |
| GO:0045742 | positive regulation of epidermal growth ... | 20 | 1 | 0.76 | 0.54008 |
| GO:0045822 | negative regulation of heart contraction | 20 | 1 | 0.76 | 0.54008 |

| | | | | | |
|---|---|---|---|---|---|
| GO:0048557 | embryonic digestive tract morphogenesis | 20 | 1 | 0.76 | 0.54008 |
| GO:0051154 | negative regulation of striated muscle c... | 20 | 1 | 0.76 | 0.54008 |
| GO:0051491 | positive regulation of filopodium assemb... | 20 | 1 | 0.76 | 0.54008 |
| GO:0051882 | mitochondrial depolarization | 20 | 1 | 0.76 | 0.54008 |
| GO:0051955 | regulation of amino acid transport | 20 | 1 | 0.76 | 0.54008 |
| GO:0052472 | modulation by host of symbiont transcrip... | 20 | 1 | 0.76 | 0.54008 |
| GO:0055023 | positive regulation of cardiac muscle ti... | 20 | 1 | 0.76 | 0.54008 |
| GO:0055093 | response to hyperoxia | 20 | 1 | 0.76 | 0.54008 |
| GO:0060713 | labyrinthine layer morphogenesis | 20 | 1 | 0.76 | 0.54008 |
| GO:0060850 | regulation of transcription involved in ... | 20 | 1 | 0.76 | 0.54008 |
| GO:0061013 | regulation of mRNA catabolic process | 20 | 1 | 0.76 | 0.54008 |
| GO:0061082 | myeloid leukocyte cytokine production | 20 | 1 | 0.76 | 0.54008 |
| GO:0070723 | response to cholesterol | 20 | 1 | 0.76 | 0.54008 |
| GO:0071392 | cellular response to estradiol stimulus | 20 | 1 | 0.76 | 0.54008 |
| GO:0071624 | positive regulation of granulocyte chemo... | 20 | 1 | 0.76 | 0.54008 |
| GO:0072661 | protein targeting to plasma membrane | 20 | 1 | 0.76 | 0.54008 |
| GO:1900087 | positive regulation of G1/S transition o... | 20 | 1 | 0.76 | 0.54008 |
| GO:1900117 | regulation of execution phase of apoptos... | 20 | 1 | 0.76 | 0.54008 |
| GO:1901031 | regulation of response to reactive oxyge... | 20 | 1 | 0.76 | 0.54008 |
| GO:0002706 | regulation of lymphocyte mediated immuni | 102 | 4 | 3.88 | 0.54734 |
| GO:0071248 | cellular response to metal ion | 102 | 4 | 3.88 | 0.54734 |
| GO:0015718 | monocarboxylic acid transport | 129 | 5 | 4.91 | 0.54753 |
| GO:0016072 | rRNA metabolic process | 129 | 5 | 4.91 | 0.54753 |
| GO:0055076 | transition metal ion homeostasis | 129 | 5 | 4.91 | 0.54753 |
| GO:2000045 | regulation of G1/S transition of mitotic... | 129 | 5 | 4.91 | 0.54753 |
| GO:0043154 | negative regulation of cysteine-type end... | 75 | 3 | 2.85 | 0.54794 |
| GO:1903524 | positive regulation of blood circulation | 75 | 3 | 2.85 | 0.54794 |
| GO:0051169 | nuclear transport | 370 | 14 | 14.08 | 0.54814 |
| GO:0015833 | peptide transport | 290 | 11 | 11.04 | 0.5484 |
| GO:0019083 | viral transcription | 183 | 7 | 6.97 | 0.54893 |
| GO:1903039 | positive regulation of leukocyte cell-ce... | 183 | 7 | 6.97 | 0.54893 |
| GO:0042110 | T cell activation | 397 | 15 | 15.11 | 0.54953 |
| GO:0070489 | T cell aggregation | 397 | 15 | 15.11 | 0.54953 |
| GO:0008542 | visual learning | 48 | 2 | 1.83 | 0.55031 |
| GO:0009201 | ribonucleoside triphosphate biosynthetic... | 48 | 2 | 1.83 | 0.55031 |
| GO:0043488 | regulation of mRNA stability | 48 | 2 | 1.83 | 0.55031 |
| GO:0045428 | regulation of nitric oxide biosynthetic ... | 48 | 2 | 1.83 | 0.55031 |
| GO:0050819 | negative regulation of coagulation | 48 | 2 | 1.83 | 0.55031 |
| GO:0097345 | mitochondrial outer membrane permeabiliz | 48 | 2 | 1.83 | 0.55031 |
| GO:0046942 | carboxylic acid transport | 264 | 10 | 10.05 | 0.55195 |
| GO:0060562 | epithelial tube morphogenesis | 318 | 12 | 12.1 | 0.55415 |
| GO:0008154 | actin polymerization or depolymerization | 157 | 6 | 5.98 | 0.55441 |
| GO:0007270 | neuron-neuron synaptic transmission | 130 | 5 | 4.95 | 0.55444 |
| GO:0042129 | regulation of T cell proliferation | 130 | 5 | 4.95 | 0.55444 |
| GO:0034112 | positive regulation of homotypic cell-ce... | 184 | 7 | 7 | 0.55474 |
| GO:0046474 | glycerophospholipid biosynthetic process | 184 | 7 | 7 | 0.55474 |
| GO:0048515 | spermatid differentiation | 103 | 4 | 3.92 | 0.5551 |
| GO:0008625 | extrinsic apoptotic signaling pathway vi... | 76 | 3 | 2.89 | 0.55697 |
| GO:0071593 | lymphocyte aggregation | 399 | 15 | 15.19 | 0.55749 |
| GO:0000381 | regulation of alternative mRNA splicing ... | | 21 | 1 | 0.8 |
| GO:0001779 | natural killer cell differentiation | 21 | 1 | 0.8 | 0.55761 |
| GO:0006071 | glycerol metabolic process | 21 | 1 | 0.8 | 0.55761 |
| GO:0006356 | regulation of transcription from RNA pol... | 21 | 1 | 0.8 | 0.55761 |

| | | | | | |
|---|---|---|---|---|---|
| GO:0006700 | C21-steroid hormone biosynthetic process | 21 | 1 | 0.8 | 0.55761 |
| GO:0006972 | hyperosmotic response | 21 | 1 | 0.8 | 0.55761 |
| GO:0007026 | negative regulation of microtubule depol... | 21 | 1 | 0.8 | 0.55761 |
| GO:0008299 | isoprenoid biosynthetic process | 21 | 1 | 0.8 | 0.55761 |
| GO:0009214 | cyclic nucleotide catabolic process | 21 | 1 | 0.8 | 0.55761 |
| GO:0009813 | flavonoid biosynthetic process | 21 | 1 | 0.8 | 0.55761 |
| GO:0010714 | positive regulation of collagen metaboli... | 21 | 1 | 0.8 | 0.55761 |
| GO:0010719 | negative regulation of epithelial to mes... | 21 | 1 | 0.8 | 0.55761 |
| GO:0015813 | L-glutamate transport | 21 | 1 | 0.8 | 0.55761 |
| GO:0021544 | subpallium development | 21 | 1 | 0.8 | 0.55761 |
| GO:0021854 | hypothalamus development | 21 | 1 | 0.8 | 0.55761 |
| GO:0022011 | myelination in peripheral nervous system | 21 | 1 | 0.8 | 0.55761 |
| GO:0030728 | ovulation | 21 | 1 | 0.8 | 0.55761 |
| GO:0032292 | peripheral nervous system axon ensheathı | 21 | 1 | 0.8 | 0.55761 |
| GO:0033032 | regulation of myeloid cell apoptotic pro... | 21 | 1 | 0.8 | 0.55761 |
| GO:0035767 | endothelial cell chemotaxis | 21 | 1 | 0.8 | 0.55761 |
| GO:0042573 | retinoic acid metabolic process | 21 | 1 | 0.8 | 0.55761 |
| GO:0043536 | positive regulation of blood vessel endo... | 21 | 1 | 0.8 | 0.55761 |
| GO:0043555 | regulation of translation in response to... | 21 | 1 | 0.8 | 0.55761 |
| GO:0045117 | azole transport | 21 | 1 | 0.8 | 0.55761 |
| GO:0045662 | negative regulation of myoblast differen... | 21 | 1 | 0.8 | 0.55761 |
| GO:0045672 | positive regulation of osteoclast differ... | 21 | 1 | 0.8 | 0.55761 |
| GO:0045920 | negative regulation of exocytosis | 21 | 1 | 0.8 | 0.55761 |
| GO:0048745 | smooth muscle tissue development | 21 | 1 | 0.8 | 0.55761 |
| GO:0048799 | organ maturation | 21 | 1 | 0.8 | 0.55761 |
| GO:0050996 | positive regulation of lipid catabolic p... | 21 | 1 | 0.8 | 0.55761 |
| GO:0051647 | nucleus localization | 21 | 1 | 0.8 | 0.55761 |
| GO:0052696 | flavonoid glucuronidation | 21 | 1 | 0.8 | 0.55761 |
| GO:0060972 | left/right pattern formation | 21 | 1 | 0.8 | 0.55761 |
| GO:0071359 | cellular response to dsRNA | 21 | 1 | 0.8 | 0.55761 |
| GO:0090022 | regulation of neutrophil chemotaxis | 21 | 1 | 0.8 | 0.55761 |
| GO:0090114 | COPII-coated vesicle budding | 21 | 1 | 0.8 | 0.55761 |
| GO:0098534 | centriole assembly | 21 | 1 | 0.8 | 0.55761 |
| GO:1901186 | positive regulation of ERBB signaling pa... | 21 | 1 | 0.8 | 0.55761 |
| GO:1902808 | positive regulation of cell cycle G1/S p... | 21 | 1 | 0.8 | 0.55761 |
| GO:2000209 | regulation of anoikis | 21 | 1 | 0.8 | 0.55761 |
| GO:0000422 | mitochondrion degradation | 158 | 6 | 6.01 | 0.56066 |
| GO:0001763 | morphogenesis of a branching structure | 212 | 8 | 8.07 | 0.56071 |
| GO:0010770 | positive regulation of cell morphogenesi... | 131 | 5 | 4.99 | 0.5613 |
| GO:0031330 | negative regulation of cellular cataboli... | 131 | 5 | 4.99 | 0.5613 |
| GO:0002228 | natural killer cell mediated immunity | 49 | 2 | 1.87 | 0.56155 |
| GO:0002709 | regulation of T cell mediated immunity | 49 | 2 | 1.87 | 0.56155 |
| GO:0015909 | long-chain fatty acid transport | 49 | 2 | 1.87 | 0.56155 |
| GO:0015914 | phospholipid transport | 49 | 2 | 1.87 | 0.56155 |
| GO:0034644 | cellular response to UV | 49 | 2 | 1.87 | 0.56155 |
| GO:0035272 | exocrine system development | 49 | 2 | 1.87 | 0.56155 |
| GO:0070527 | platelet aggregation | 49 | 2 | 1.87 | 0.56155 |
| GO:1902110 | positive regulation of mitochondrial mem... | 49 | 2 | 1.87 | 0.56155 |
| GO:1902686 | mitochondrial outer membrane permeabiliz | 49 | 2 | 1.87 | 0.56155 |
| GO:0015849 | organic acid transport | 266 | 10 | 10.13 | 0.56164 |
| GO:0030193 | regulation of blood coagulation | 77 | 3 | 2.93 | 0.56589 |
| GO:0030260 | entry into host cell | 77 | 3 | 2.93 | 0.56589 |
| GO:0032941 | secretion by tissue | 77 | 3 | 2.93 | 0.56589 |

| | | | | | |
|---|---|---|---|---|---|
| GO:0044409 | entry into host | 77 | 3 | 2.93 | 0.56589 |
| GO:0051297 | centrosome organization | 77 | 3 | 2.93 | 0.56589 |
| GO:0051782 | negative regulation of cell division | 77 | 3 | 2.93 | 0.56589 |
| GO:0051806 | entry into cell of other organism involv... | 77 | 3 | 2.93 | 0.56589 |
| GO:0051828 | entry into other organism involved in sy... | 77 | 3 | 2.93 | 0.56589 |
| GO:0052126 | movement in host environment | 77 | 3 | 2.93 | 0.56589 |
| GO:0052192 | movement in environment of other organis | 77 | 3 | 2.93 | 0.56589 |
| GO:0090317 | negative regulation of intracellular pro... | 77 | 3 | 2.93 | 0.56589 |
| GO:1900046 | regulation of hemostasis | 77 | 3 | 2.93 | 0.56589 |
| GO:1902115 | regulation of organelle assembly | 77 | 3 | 2.93 | 0.56589 |
| GO:2000117 | negative regulation of cysteine-type end... | 77 | 3 | 2.93 | 0.56589 |
| GO:0022409 | positive regulation of cell-cell adhesio... | 213 | 8 | 8.11 | 0.56609 |
| GO:0030534 | adult behavior | 132 | 5 | 5.02 | 0.56811 |
| GO:0043409 | negative regulation of MAPK cascade | 132 | 5 | 5.02 | 0.56811 |
| GO:1902806 | regulation of cell cycle G1/S phase tran... | 132 | 5 | 5.02 | 0.56811 |
| GO:0006641 | triglyceride metabolic process | 105 | 4 | 4 | 0.57041 |
| GO:0022904 | respiratory electron transport chain | 105 | 4 | 4 | 0.57041 |
| GO:0032479 | regulation of type I interferon producti... | 105 | 4 | 4 | 0.57041 |
| GO:1903828 | negative regulation of cellular protein ... | 105 | 4 | 4 | 0.57041 |
| GO:0046822 | regulation of nucleocytoplasmic transpor... | 187 | 7 | 7.12 | 0.57199 |
| GO:0050730 | regulation of peptidyl-tyrosine phosphor... | 187 | 7 | 7.12 | 0.57199 |
| GO:0051650 | establishment of vesicle localization | 187 | 7 | 7.12 | 0.57199 |
| GO:0090276 | regulation of peptide hormone secretion | 187 | 7 | 7.12 | 0.57199 |
| GO:0002718 | regulation of cytokine production involv... | 50 | 2 | 1.9 | 0.57259 |
| GO:0014812 | muscle cell migration | 50 | 2 | 1.9 | 0.57259 |
| GO:0043487 | regulation of RNA stability | 50 | 2 | 1.9 | 0.57259 |
| GO:0045685 | regulation of glial cell differentiation | 50 | 2 | 1.9 | 0.57259 |
| GO:0072331 | signal transduction by p53 class mediato... | 160 | 6 | 6.09 | 0.57304 |
| GO:0000726 | non-recombinational repair | 22 | 1 | 0.84 | 0.57448 |
| GO:0001502 | cartilage condensation | 22 | 1 | 0.84 | 0.57448 |
| GO:0001941 | postsynaptic membrane organization | 22 | 1 | 0.84 | 0.57448 |
| GO:0003416 | endochondral bone growth | 22 | 1 | 0.84 | 0.57448 |
| GO:0007220 | Notch receptor processing | 22 | 1 | 0.84 | 0.57448 |
| GO:0007398 | ectoderm development | 22 | 1 | 0.84 | 0.57448 |
| GO:0008105 | asymmetric protein localization | 22 | 1 | 0.84 | 0.57448 |
| GO:0010737 | protein kinase A signaling | 22 | 1 | 0.84 | 0.57448 |
| GO:0015800 | acidic amino acid transport | 22 | 1 | 0.84 | 0.57448 |
| GO:0015985 | energy coupled proton transport | down el... | 22 | 1 | 0.84 |
| GO:0015986 | ATP synthesis coupled proton transport | 22 | 1 | 0.84 | 0.57448 |
| GO:0019048 | modulation by virus of host morphology o.. | 22 | 1 | 0.84 | 0.57448 |
| GO:0030517 | negative regulation of axon extension | 22 | 1 | 0.84 | 0.57448 |
| GO:0031063 | regulation of histone deacetylation | 22 | 1 | 0.84 | 0.57448 |
| GO:0032105 | negative regulation of response to extra... | 22 | 1 | 0.84 | 0.57448 |
| GO:0032108 | negative regulation of response to nutri... | 22 | 1 | 0.84 | 0.57448 |
| GO:0032786 | positive regulation of DNA-templated tra... | 22 | 1 | 0.84 | 0.57448 |
| GO:0033081 | regulation of T cell differentiation in ... | 22 | 1 | 0.84 | 0.57448 |
| GO:0035929 | steroid hormone secretion | 22 | 1 | 0.84 | 0.57448 |
| GO:0036314 | response to sterol | 22 | 1 | 0.84 | 0.57448 |
| GO:0042506 | tyrosine phosphorylation of Stat5 protei... | 22 | 1 | 0.84 | 0.57448 |
| GO:0042572 | retinol metabolic process | 22 | 1 | 0.84 | 0.57448 |
| GO:0042994 | cytoplasmic sequestering of transcriptio... | 22 | 1 | 0.84 | 0.57448 |
| GO:0043954 | cellular component maintenance | 22 | 1 | 0.84 | 0.57448 |
| GO:0060384 | innervation | 22 | 1 | 0.84 | 0.57448 |

| | | | | | |
|---|---|---|---|---|---|
| GO:0061001 | regulation of dendritic spine morphogene.. | 22 | 1 | 0.84 | 0.57448 |
| GO:0071470 | cellular response to osmotic stress | 22 | 1 | 0.84 | 0.57448 |
| GO:0071634 | regulation of transforming growth factor... | 22 | 1 | 0.84 | 0.57448 |
| GO:0086005 | ventricular cardiac muscle cell action p... | 22 | 1 | 0.84 | 0.57448 |
| GO:0086064 | cell communication by electrical couplin... | 22 | 1 | 0.84 | 0.57448 |
| GO:0090103 | cochlea morphogenesis | 22 | 1 | 0.84 | 0.57448 |
| GO:0098743 | cell aggregation | 22 | 1 | 0.84 | 0.57448 |
| GO:1901522 | positive regulation of transcription fro... | 22 | 1 | 0.84 | 0.57448 |
| GO:1902622 | regulation of neutrophil migration | 22 | 1 | 0.84 | 0.57448 |
| GO:0001909 | leukocyte mediated cytotoxicity | 78 | 3 | 2.97 | 0.5747 |
| GO:0061053 | somite development | 78 | 3 | 2.97 | 0.5747 |
| GO:1903901 | negative regulation of viral life cycle | 78 | 3 | 2.97 | 0.5747 |
| GO:0030041 | actin filament polymerization | 133 | 5 | 5.06 | 0.57486 |
| GO:0030879 | mammary gland development | 133 | 5 | 5.06 | 0.57486 |
| GO:0006401 | RNA catabolic process | 215 | 8 | 8.18 | 0.57676 |
| GO:0060828 | regulation of canonical Wnt signaling pa... | 215 | 8 | 8.18 | 0.57676 |
| GO:0030217 | T cell differentiation | 188 | 7 | 7.16 | 0.57767 |
| GO:0031123 | RNA 3'-end processing | 106 | 4 | 4.03 | 0.57795 |
| GO:0030203 | glycosaminoglycan metabolic process | 161 | 6 | 6.13 | 0.57917 |
| GO:1903900 | regulation of viral life cycle | 161 | 6 | 6.13 | 0.57917 |
| GO:0006644 | phospholipid metabolic process | 324 | 12 | 12.33 | 0.58047 |
| GO:0043902 | positive regulation of multi-organism pr... | 134 | 5 | 5.1 | 0.58155 |
| GO:0007204 | positive regulation of cytosolic calcium... | 216 | 8 | 8.22 | 0.58205 |
| GO:0006289 | nucleotide-excision repair | 79 | 3 | 3.01 | 0.58339 |
| GO:0001541 | ovarian follicle development | 51 | 2 | 1.94 | 0.58343 |
| GO:0006611 | protein export from nucleus | 51 | 2 | 1.94 | 0.58343 |
| GO:0035794 | positive regulation of mitochondrial mem... | 51 | 2 | 1.94 | 0.58343 |
| GO:0048747 | muscle fiber development | 51 | 2 | 1.94 | 0.58343 |
| GO:0051493 | regulation of cytoskeleton organization | 352 | 13 | 13.4 | 0.58473 |
| GO:0042098 | T cell proliferation | 162 | 6 | 6.17 | 0.58525 |
| GO:2000027 | regulation of organ morphogenesis | 162 | 6 | 6.17 | 0.58525 |
| GO:0022900 | electron transport chain | 107 | 4 | 4.07 | 0.58541 |
| GO:0006486 | protein glycosylation | 271 | 10 | 10.32 | 0.58548 |
| GO:0043413 | macromolecule glycosylation | 271 | 10 | 10.32 | 0.58548 |
| GO:0009116 | nucleoside metabolic process | 244 | 9 | 9.29 | 0.58621 |
| GO:0008064 | regulation of actin polymerization or de... | 135 | 5 | 5.14 | 0.58819 |
| GO:0002694 | regulation of leukocyte activation | 380 | 14 | 14.47 | 0.58878 |
| GO:0055074 | calcium ion homeostasis | 326 | 12 | 12.41 | 0.5891 |
| GO:0000038 | very long-chain fatty acid metabolic pro... | 23 | 1 | 0.88 | 0.5907 |
| GO:0001961 | positive regulation of cytokine-mediated... | 23 | 1 | 0.88 | 0.5907 |
| GO:0002089 | lens morphogenesis in camera-type eye | 23 | 1 | 0.88 | 0.5907 |
| GO:0002833 | positive regulation of response to bioti... | 23 | 1 | 0.88 | 0.5907 |
| GO:0009303 | rRNA transcription | 23 | 1 | 0.88 | 0.5907 |
| GO:0010882 | regulation of cardiac muscle contraction... | 23 | 1 | 0.88 | 0.5907 |
| GO:0014009 | glial cell proliferation | 23 | 1 | 0.88 | 0.5907 |
| GO:0015721 | bile acid and bile salt transport | 23 | 1 | 0.88 | 0.5907 |
| GO:0016338 | calcium-independent cell-cell adhesion v... | 23 | 1 | 0.88 | 0.5907 |
| GO:0019400 | alditol metabolic process | 23 | 1 | 0.88 | 0.5907 |
| GO:0031114 | regulation of microtubule depolymerizati... | 23 | 1 | 0.88 | 0.5907 |
| GO:0033006 | regulation of mast cell activation invol... | 23 | 1 | 0.88 | 0.5907 |
| GO:0036230 | granulocyte activation | 23 | 1 | 0.88 | 0.5907 |
| GO:0036336 | dendritic cell migration | 23 | 1 | 0.88 | 0.5907 |
| GO:0042501 | serine phosphorylation of STAT protein | 23 | 1 | 0.88 | 0.5907 |

| | | | | | |
|---|---|---|---|---|---|
| GO:0043304 | regulation of mast cell degranulation | 23 | 1 | 0.88 | 0.5907 |
| GO:0045686 | negative regulation of glial cell differ... | 23 | 1 | 0.88 | 0.5907 |
| GO:0046839 | phospholipid dephosphorylation | 23 | 1 | 0.88 | 0.5907 |
| GO:0048246 | macrophage chemotaxis | 23 | 1 | 0.88 | 0.5907 |
| GO:0051953 | negative regulation of amine transport | 23 | 1 | 0.88 | 0.5907 |
| GO:0060669 | embryonic placenta morphogenesis | 23 | 1 | 0.88 | 0.5907 |
| GO:0071604 | transforming growth factor beta producti... | 23 | 1 | 0.88 | 0.5907 |
| GO:0071868 | cellular response to monoamine stimulus | 23 | 1 | 0.88 | 0.5907 |
| GO:0071870 | cellular response to catecholamine stimu... | 23 | 1 | 0.88 | 0.5907 |
| GO:1901224 | positive regulation of NIK/NF-kappaB sig... | 23 | 1 | 0.88 | 0.5907 |
| GO:1902475 | L-alpha-amino acid transmembrane transp | 23 | 1 | 0.88 | 0.5907 |
| GO:0030316 | osteoclast differentiation | 80 | 3 | 3.05 | 0.59198 |
| GO:0043604 | amide biosynthetic process | 80 | 3 | 3.05 | 0.59198 |
| GO:0048525 | negative regulation of viral process | 80 | 3 | 3.05 | 0.59198 |
| GO:0090501 | RNA phosphodiester bond hydrolysis | 80 | 3 | 3.05 | 0.59198 |
| GO:0007338 | single fertilization | 108 | 4 | 4.11 | 0.5928 |
| GO:0032606 | type I interferon production | 108 | 4 | 4.11 | 0.5928 |
| GO:0007405 | neuroblast proliferation | 52 | 2 | 1.98 | 0.59406 |
| GO:0014068 | positive regulation of phosphatidylinosi... | 52 | 2 | 1.98 | 0.59406 |
| GO:0031047 | gene silencing by RNA | 52 | 2 | 1.98 | 0.59406 |
| GO:0032508 | DNA duplex unwinding | 52 | 2 | 1.98 | 0.59406 |
| GO:0042773 | ATP synthesis coupled electron transport | 52 | 2 | 1.98 | 0.59406 |
| GO:0042775 | mitochondrial ATP synthesis coupled elec. | 52 | 2 | 1.98 | 0.59406 |
| GO:0050918 | positive chemotaxis | 52 | 2 | 1.98 | 0.59406 |
| GO:0060443 | mammary gland morphogenesis | 52 | 2 | 1.98 | 0.59406 |
| GO:0070509 | calcium ion import | 52 | 2 | 1.98 | 0.59406 |
| GO:0006402 | mRNA catabolic process | 191 | 7 | 7.27 | 0.59449 |
| GO:0048002 | antigen processing and presentation of p... | 191 | 7 | 7.27 | 0.59449 |
| GO:0006865 | amino acid transport | 136 | 5 | 5.18 | 0.59477 |
| GO:0009167 | purine ribonucleoside monophosphate met | 136 | 5 | 5.18 | 0.59477 |
| GO:0030832 | regulation of actin filament length | 136 | 5 | 5.18 | 0.59477 |
| GO:0031497 | chromatin assembly | 136 | 5 | 5.18 | 0.59477 |
| GO:0008202 | steroid metabolic process | 273 | 10 | 10.39 | 0.59485 |
| GO:0010959 | regulation of metal ion transport | 273 | 10 | 10.39 | 0.59485 |
| GO:0002697 | regulation of immune effector process | 274 | 10 | 10.43 | 0.5995 |
| GO:0002791 | regulation of peptide secretion | 192 | 7 | 7.31 | 0.60002 |
| GO:0001508 | action potential | 109 | 4 | 4.15 | 0.60011 |
| GO:0009063 | cellular amino acid catabolic process | 109 | 4 | 4.15 | 0.60011 |
| GO:0033135 | regulation of peptidyl-serine phosphoryl... | 109 | 4 | 4.15 | 0.60011 |
| GO:0042990 | regulation of transcription factor impor... | 81 | 3 | 3.08 | 0.60045 |
| GO:0009126 | purine nucleoside monophosphate metabc | 137 | 5 | 5.22 | 0.6013 |
| GO:1903035 | negative regulation of response to wound.. | 137 | 5 | 5.22 | 0.6013 |
| GO:0045785 | positive regulation of cell adhesion | 329 | 12 | 12.52 | 0.6019 |
| GO:0010821 | regulation of mitochondrion organization | 275 | 10 | 10.47 | 0.60413 |
| GO:0007632 | visual behavior | 53 | 2 | 2.02 | 0.60449 |
| GO:0010923 | negative regulation of phosphatase activ... | 53 | 2 | 2.02 | 0.60449 |
| GO:0015807 | L-amino acid transport | 53 | 2 | 2.02 | 0.60449 |
| GO:0019229 | regulation of vasoconstriction | 53 | 2 | 2.02 | 0.60449 |
| GO:0032392 | DNA geometric change | 53 | 2 | 2.02 | 0.60449 |
| GO:0032729 | positive regulation of interferon-gamma ... | 53 | 2 | 2.02 | 0.60449 |
| GO:0048546 | digestive tract morphogenesis | 53 | 2 | 2.02 | 0.60449 |
| GO:0050885 | neuromuscular process controlling balanc. | 53 | 2 | 2.02 | 0.60449 |
| GO:0061045 | negative regulation of wound healing | 53 | 2 | 2.02 | 0.60449 |

| | | | | | |
|---|---|---|---|---|---|
| GO:1902108 | regulation of mitochondrial membrane per. | 53 | 2 | 2.02 | 0.60449 |
| GO:0019080 | viral gene expression | 193 | 7 | 7.35 | 0.60551 |
| GO:0045165 | cell fate commitment | 248 | 9 | 9.44 | 0.6058 |
| GO:0000002 | mitochondrial genome maintenance | 24 | 1 | 0.91 | 0.6063 |
| GO:0000462 | maturation of SSU-rRNA from tricistronic... | 24 | 1 | 0.91 | 0.6063 |
| GO:0001881 | receptor recycling | 24 | 1 | 0.91 | 0.6063 |
| GO:0008334 | histone mRNA metabolic process | 24 | 1 | 0.91 | 0.6063 |
| GO:0009110 | vitamin biosynthetic process | 24 | 1 | 0.91 | 0.6063 |
| GO:0009651 | response to salt stress | 24 | 1 | 0.91 | 0.6063 |
| GO:0014044 | Schwann cell development | 24 | 1 | 0.91 | 0.6063 |
| GO:0019674 | NAD metabolic process | 24 | 1 | 0.91 | 0.6063 |
| GO:0032728 | positive regulation of interferon-beta p... | 24 | 1 | 0.91 | 0.6063 |
| GO:0034694 | response to prostaglandin | 24 | 1 | 0.91 | 0.6063 |
| GO:0043114 | regulation of vascular permeability | 24 | 1 | 0.91 | 0.6063 |
| GO:0043368 | positive T cell selection | 24 | 1 | 0.91 | 0.6063 |
| GO:0045940 | positive regulation of steroid metabolic... | 24 | 1 | 0.91 | 0.6063 |
| GO:0048710 | regulation of astrocyte differentiation | 24 | 1 | 0.91 | 0.6063 |
| GO:0048873 | homeostasis of number of cells within a ... | 24 | 1 | 0.91 | 0.6063 |
| GO:0050927 | positive regulation of positive chemotax... | 24 | 1 | 0.91 | 0.6063 |
| GO:0060081 | membrane hyperpolarization | 24 | 1 | 0.91 | 0.6063 |
| GO:0060334 | regulation of interferon-gamma-mediated . | 24 | 1 | 0.91 | 0.6063 |
| GO:0060397 | JAK-STAT cascade involved in growth hor | 24 | 1 | 0.91 | 0.6063 |
| GO:0060421 | positive regulation of heart growth | 24 | 1 | 0.91 | 0.6063 |
| GO:0060706 | cell differentiation involved in embryon... | 24 | 1 | 0.91 | 0.6063 |
| GO:0060760 | positive regulation of response to cytok... | 24 | 1 | 0.91 | 0.6063 |
| GO:2000779 | regulation of double-strand break repair | 24 | 1 | 0.91 | 0.6063 |
| GO:0051051 | negative regulation of transport | 385 | 14 | 14.66 | 0.60852 |
| GO:0001942 | hair follicle development | 82 | 3 | 3.12 | 0.6088 |
| GO:0022404 | molting cycle process | 82 | 3 | 3.12 | 0.6088 |
| GO:0022405 | hair cycle process | 82 | 3 | 3.12 | 0.6088 |
| GO:0031016 | pancreas development | 82 | 3 | 3.12 | 0.6088 |
| GO:0031023 | microtubule organizing center organizati... | 82 | 3 | 3.12 | 0.6088 |
| GO:0042991 | transcription factor import into nucleus | 82 | 3 | 3.12 | 0.6088 |
| GO:0050818 | regulation of coagulation | 82 | 3 | 3.12 | 0.6088 |
| GO:0098773 | skin epidermis development | 82 | 3 | 3.12 | 0.6088 |
| GO:2001243 | negative regulation of intrinsic apoptot... | 82 | 3 | 3.12 | 0.6088 |
| GO:1900180 | regulation of protein localization to nu... | 166 | 6 | 6.32 | 0.60911 |
| GO:0010631 | epithelial cell migration | 194 | 7 | 7.38 | 0.61097 |
| GO:0046034 | ATP metabolic process | 111 | 4 | 4.23 | 0.61449 |
| GO:0001947 | heart looping | 54 | 2 | 2.06 | 0.61472 |
| GO:0002702 | positive regulation of production of mol... | 54 | 2 | 2.06 | 0.61472 |
| GO:0030888 | regulation of B cell proliferation | 54 | 2 | 2.06 | 0.61472 |
| GO:0045670 | regulation of osteoclast differentiation | 54 | 2 | 2.06 | 0.61472 |
| GO:0046530 | photoreceptor cell differentiation | 54 | 2 | 2.06 | 0.61472 |
| GO:0051492 | regulation of stress fiber assembly | 54 | 2 | 2.06 | 0.61472 |
| GO:0051302 | regulation of cell division | 250 | 9 | 9.52 | 0.61543 |
| GO:0032649 | regulation of interferon-gamma productio... | 83 | 3 | 3.16 | 0.61703 |
| GO:0043244 | regulation of protein complex disassembl.. | 83 | 3 | 3.16 | 0.61703 |
| GO:0070124 | mitochondrial translational initiation | 83 | 3 | 3.16 | 0.61703 |
| GO:0070125 | mitochondrial translational elongation | 83 | 3 | 3.16 | 0.61703 |
| GO:0070126 | mitochondrial translational termination | 83 | 3 | 3.16 | 0.61703 |
| GO:0071158 | positive regulation of cell cycle arrest | 83 | 3 | 3.16 | 0.61703 |
| GO:0042391 | regulation of membrane potential | 306 | 11 | 11.65 | 0.62032 |

| | | | | | |
|---|---|---|---|---|---|
| GO:0006022 | aminoglycan metabolic process | 168 | 6 | 6.4 | 0.62075 |
| GO:0050796 | regulation of insulin secretion | 168 | 6 | 6.4 | 0.62075 |
| GO:0001963 | synaptic transmission | dopaminerg | 25 | 1 | 0.95 |
| GO:0003071 | renal system process involved in regulat... | 25 | 1 | 0.95 | 0.62131 |
| GO:0003179 | heart valve morphogenesis | 25 | 1 | 0.95 | 0.62131 |
| GO:0007194 | negative regulation of adenylate cyclase... | 25 | 1 | 0.95 | 0.62131 |
| GO:0010107 | potassium ion import | 25 | 1 | 0.95 | 0.62131 |
| GO:0010575 | positive regulation vascular endothelial... | 25 | 1 | 0.95 | 0.62131 |
| GO:0010880 | regulation of release of sequestered cal... | 25 | 1 | 0.95 | 0.62131 |
| GO:0016073 | snRNA metabolic process | 25 | 1 | 0.95 | 0.62131 |
| GO:0021680 | cerebellar Purkinje cell layer developme... | 25 | 1 | 0.95 | 0.62131 |
| GO:0021799 | cerebral cortex radially oriented cell m... | 25 | 1 | 0.95 | 0.62131 |
| GO:0032892 | positive regulation of organic acid tran... | 25 | 1 | 0.95 | 0.62131 |
| GO:0032965 | regulation of collagen biosynthetic proc... | 25 | 1 | 0.95 | 0.62131 |
| GO:0033028 | myeloid cell apoptotic process | 25 | 1 | 0.95 | 0.62131 |
| GO:0034105 | positive regulation of tissue remodeling | 25 | 1 | 0.95 | 0.62131 |
| GO:0036151 | phosphatidylcholine acyl-chain remodelin.. | 25 | 1 | 0.95 | 0.62131 |
| GO:0045652 | regulation of megakaryocyte differentiat... | 25 | 1 | 0.95 | 0.62131 |
| GO:0045730 | respiratory burst | 25 | 1 | 0.95 | 0.62131 |
| GO:0046596 | regulation of viral entry into host cell | 25 | 1 | 0.95 | 0.62131 |
| GO:0048821 | erythrocyte development | 25 | 1 | 0.95 | 0.62131 |
| GO:0050926 | regulation of positive chemotaxis | 25 | 1 | 0.95 | 0.62131 |
| GO:0052695 | cellular glucuronidation | 25 | 1 | 0.95 | 0.62131 |
| GO:0055025 | positive regulation of cardiac muscle ti... | 25 | 1 | 0.95 | 0.62131 |
| GO:0060330 | regulation of response to interferon-gam... | 25 | 1 | 0.95 | 0.62131 |
| GO:0071867 | response to monoamine | 25 | 1 | 0.95 | 0.62131 |
| GO:0071869 | response to catecholamine | 25 | 1 | 0.95 | 0.62131 |
| GO:0090004 | positive regulation of establishment of ... | 25 | 1 | 0.95 | 0.62131 |
| GO:2000108 | positive regulation of leukocyte apoptot... | 25 | 1 | 0.95 | 0.62131 |
| GO:0035637 | multicellular organismal signaling | 112 | 4 | 4.26 | 0.62156 |
| GO:0090132 | epithelium migration | 196 | 7 | 7.46 | 0.62175 |
| GO:0030098 | lymphocyte differentiation | 279 | 10 | 10.62 | 0.62236 |
| GO:0070085 | glycosylation | 279 | 10 | 10.62 | 0.62236 |
| GO:0051249 | regulation of lymphocyte activation | 334 | 12 | 12.71 | 0.62281 |
| GO:0021879 | forebrain neuron differentiation | 55 | 2 | 2.09 | 0.62474 |
| GO:0036473 | cell death in response to oxidative stre... | 55 | 2 | 2.09 | 0.62474 |
| GO:0042509 | regulation of tyrosine phosphorylation o... | 55 | 2 | 2.09 | 0.62474 |
| GO:0043331 | response to dsRNA | 55 | 2 | 2.09 | 0.62474 |
| GO:0048814 | regulation of dendrite morphogenesis | 55 | 2 | 2.09 | 0.62474 |
| GO:1903076 | regulation of protein localization to pl... | 55 | 2 | 2.09 | 0.62474 |
| GO:1903426 | regulation of reactive oxygen species bi... | 55 | 2 | 2.09 | 0.62474 |
| GO:1903725 | regulation of phospholipid metabolic pro... | 55 | 2 | 2.09 | 0.62474 |
| GO:0050773 | regulation of dendrite development | 84 | 3 | 3.2 | 0.62515 |
| GO:0071706 | tumor necrosis factor superfamily cytoki... | 84 | 3 | 3.2 | 0.62515 |
| GO:1903311 | regulation of mRNA metabolic process | 84 | 3 | 3.2 | 0.62515 |
| GO:0030073 | insulin secretion | 197 | 7 | 7.5 | 0.62708 |
| GO:0009119 | ribonucleoside metabolic process | 225 | 8 | 8.56 | 0.62819 |
| GO:0006639 | acylglycerol metabolic process | 113 | 4 | 4.3 | 0.62855 |
| GO:0008203 | cholesterol metabolic process | 113 | 4 | 4.3 | 0.62855 |
| GO:1902107 | positive regulation of leukocyte differe... | 113 | 4 | 4.3 | 0.62855 |
| GO:0051656 | establishment of organelle localization | 281 | 10 | 10.7 | 0.63132 |
| GO:0050792 | regulation of viral process | 170 | 6 | 6.47 | 0.6322 |
| GO:0090130 | tissue migration | 198 | 7 | 7.54 | 0.63236 |

| | | | | | |
|---|---|---|---|---|---|
| GO:0002705 | positive regulation of leukocyte mediate... | 85 | 3 | 3.24 | 0.63315 |
| GO:0010921 | regulation of phosphatase activity | 85 | 3 | 3.24 | 0.63315 |
| GO:0050728 | negative regulation of inflammatory resp... | 85 | 3 | 3.24 | 0.63315 |
| GO:0051640 | organelle localization | 364 | 13 | 13.86 | 0.63321 |
| GO:0006826 | iron ion transport | 56 | 2 | 2.13 | 0.63456 |
| GO:0019432 | triglyceride biosynthetic process | 56 | 2 | 2.13 | 0.63456 |
| GO:0030204 | chondroitin sulfate metabolic process | 56 | 2 | 2.13 | 0.63456 |
| GO:0006638 | neutral lipid metabolic process | 114 | 4 | 4.34 | 0.63545 |
| GO:0007224 | smoothened signaling pathway | 114 | 4 | 4.34 | 0.63545 |
| GO:0050707 | regulation of cytokine secretion | 114 | 4 | 4.34 | 0.63545 |
| GO:0003209 | cardiac atrium morphogenesis | 26 | 1 | 0.99 | 0.63575 |
| GO:0006063 | uronic acid metabolic process | 26 | 1 | 0.99 | 0.63575 |
| GO:0006699 | bile acid biosynthetic process | 26 | 1 | 0.99 | 0.63575 |
| GO:0008045 | motor neuron axon guidance | 26 | 1 | 0.99 | 0.63575 |
| GO:0009595 | detection of biotic stimulus | 26 | 1 | 0.99 | 0.63575 |
| GO:0010165 | response to X-ray | 26 | 1 | 0.99 | 0.63575 |
| GO:0010543 | regulation of platelet activation | 26 | 1 | 0.99 | 0.63575 |
| GO:0019433 | triglyceride catabolic process | 26 | 1 | 0.99 | 0.63575 |
| GO:0019585 | glucuronate metabolic process | 26 | 1 | 0.99 | 0.63575 |
| GO:0031062 | positive regulation of histone methylati... | 26 | 1 | 0.99 | 0.63575 |
| GO:0031280 | negative regulation of cyclase activity | 26 | 1 | 0.99 | 0.63575 |
| GO:0031648 | protein destabilization | 26 | 1 | 0.99 | 0.63575 |
| GO:0032369 | negative regulation of lipid transport | 26 | 1 | 0.99 | 0.63575 |
| GO:0032673 | regulation of interleukin-4 production | 26 | 1 | 0.99 | 0.63575 |
| GO:0033280 | response to vitamin D | 26 | 1 | 0.99 | 0.63575 |
| GO:0042255 | ribosome assembly | 26 | 1 | 0.99 | 0.63575 |
| GO:0042430 | indole-containing compound metabolic pro | 26 | 1 | 0.99 | 0.63575 |
| GO:0042481 | regulation of odontogenesis | 26 | 1 | 0.99 | 0.63575 |
| GO:0043276 | anoikis | 26 | 1 | 0.99 | 0.63575 |
| GO:0043403 | skeletal muscle tissue regeneration | 26 | 1 | 0.99 | 0.63575 |
| GO:0050832 | defense response to fungus | 26 | 1 | 0.99 | 0.63575 |
| GO:0060306 | regulation of membrane repolarization | 26 | 1 | 0.99 | 0.63575 |
| GO:0060351 | cartilage development involved in endoch. | 26 | 1 | 0.99 | 0.63575 |
| GO:0060445 | branching involved in salivary gland mor... | 26 | 1 | 0.99 | 0.63575 |
| GO:0060561 | apoptotic process involved in morphogene | 26 | 1 | 0.99 | 0.63575 |
| GO:0061036 | positive regulation of cartilage develop... | 26 | 1 | 0.99 | 0.63575 |
| GO:0090200 | positive regulation of release of cytoch... | 26 | 1 | 0.99 | 0.63575 |
| GO:0090279 | regulation of calcium ion import | 26 | 1 | 0.99 | 0.63575 |
| GO:0090630 | activation of GTPase activity | 26 | 1 | 0.99 | 0.63575 |
| GO:0097502 | mannosylation | 26 | 1 | 0.99 | 0.63575 |
| GO:1902175 | regulation of oxidative stress-induced i... | 26 | 1 | 0.99 | 0.63575 |
| GO:1902743 | regulation of lamellipodium organization | 26 | 1 | 0.99 | 0.63575 |
| GO:2000249 | regulation of actin cytoskeleton reorgan... | 26 | 1 | 0.99 | 0.63575 |
| GO:0090092 | regulation of transmembrane receptor pro. | 199 | 7 | 7.58 | 0.63761 |
| GO:1903008 | organelle disassembly | 171 | 6 | 6.51 | 0.63784 |
| GO:0015696 | ammonium transport | 86 | 3 | 3.27 | 0.64103 |
| GO:0042439 | ethanolamine-containing compound metab | 86 | 3 | 3.27 | 0.64103 |
| GO:0071241 | cellular response to inorganic substance | 115 | 4 | 4.38 | 0.64227 |
| GO:0006732 | coenzyme metabolic process | 200 | 7 | 7.61 | 0.64281 |
| GO:0006898 | receptor-mediated endocytosis | 228 | 8 | 8.68 | 0.64294 |
| GO:0019882 | antigen processing and presentation | 228 | 8 | 8.68 | 0.64294 |
| GO:0006282 | regulation of DNA repair | 57 | 2 | 2.17 | 0.64418 |
| GO:0061371 | determination of heart left/right asymme... | 57 | 2 | 2.17 | 0.64418 |

| | | | | | |
|---|---|---|---|---|---|
| GO:1901019 | regulation of calcium ion transmembrane .. | 57 | 2 | 2.17 | 0.64418 |
| GO:1903169 | regulation of calcium ion transmembrane .. | 57 | 2 | 2.17 | 0.64418 |
| GO:0072655 | establishment of protein localization to... | 144 | 5 | 5.48 | 0.64524 |
| GO:0050900 | leukocyte migration | 312 | 11 | 11.88 | 0.64577 |
| GO:0009101 | glycoprotein biosynthetic process | 340 | 12 | 12.94 | 0.64718 |
| GO:0006606 | protein import into nucleus | 229 | 8 | 8.72 | 0.64778 |
| GO:0044744 | protein targeting to nucleus | 229 | 8 | 8.72 | 0.64778 |
| GO:1902593 | single-organism nuclear import | 229 | 8 | 8.72 | 0.64778 |
| GO:0061138 | morphogenesis of a branching epithelium | 201 | 7 | 7.65 | 0.64797 |
| GO:1901342 | regulation of vasculature development | 201 | 7 | 7.65 | 0.64797 |
| GO:0070838 | divalent metal ion transport | 368 | 13 | 14.01 | 0.64872 |
| GO:0033138 | positive regulation of peptidyl-serine p... | 87 | 3 | 3.31 | 0.64879 |
| GO:0072091 | regulation of stem cell proliferation | 87 | 3 | 3.31 | 0.64879 |
| GO:1903650 | negative regulation of cytoplasmic trans... | 87 | 3 | 3.31 | 0.64879 |
| GO:0031349 | positive regulation of defense response | 285 | 10 | 10.85 | 0.64889 |
| GO:0009895 | negative regulation of catabolic process | 173 | 6 | 6.59 | 0.64898 |
| GO:0000722 | telomere maintenance via recombination | 27 | 1 | 1.03 | 0.64964 |
| GO:0002026 | regulation of the force of heart contrac... | 27 | 1 | 1.03 | 0.64964 |
| GO:0002823 | negative regulation of adaptive immune r... | 27 | 1 | 1.03 | 0.64964 |
| GO:0003081 | regulation of systemic arterial blood pr... | 27 | 1 | 1.03 | 0.64964 |
| GO:0003170 | heart valve development | 27 | 1 | 1.03 | 0.64964 |
| GO:0003197 | endocardial cushion development | 27 | 1 | 1.03 | 0.64964 |
| GO:0006221 | pyrimidine nucleotide biosynthetic proce... | 27 | 1 | 1.03 | 0.64964 |
| GO:0006656 | phosphatidylcholine biosynthetic process | 27 | 1 | 1.03 | 0.64964 |
| GO:0006658 | phosphatidylserine metabolic process | 27 | 1 | 1.03 | 0.64964 |
| GO:0009812 | flavonoid metabolic process | 27 | 1 | 1.03 | 0.64964 |
| GO:0010507 | negative regulation of autophagy | 27 | 1 | 1.03 | 0.64964 |
| GO:0018146 | keratan sulfate biosynthetic process | 27 | 1 | 1.03 | 0.64964 |
| GO:0031103 | axon regeneration | 27 | 1 | 1.03 | 0.64964 |
| GO:0034587 | piRNA metabolic process | 27 | 1 | 1.03 | 0.64964 |
| GO:0043268 | positive regulation of potassium ion tra... | 27 | 1 | 1.03 | 0.64964 |
| GO:0045581 | negative regulation of T cell differenti... | 27 | 1 | 1.03 | 0.64964 |
| GO:0045739 | positive regulation of DNA repair | 27 | 1 | 1.03 | 0.64964 |
| GO:0048016 | inositol phosphate-mediated signaling | 27 | 1 | 1.03 | 0.64964 |
| GO:0048596 | embryonic camera-type eye morphogenes | 27 | 1 | 1.03 | 0.64964 |
| GO:0048713 | regulation of oligodendrocyte differenti... | 27 | 1 | 1.03 | 0.64964 |
| GO:0051350 | negative regulation of lyase activity | 27 | 1 | 1.03 | 0.64964 |
| GO:0051452 | intracellular pH reduction | 27 | 1 | 1.03 | 0.64964 |
| GO:0060997 | dendritic spine morphogenesis | 27 | 1 | 1.03 | 0.64964 |
| GO:0070229 | negative regulation of lymphocyte apopto.. | 27 | 1 | 1.03 | 0.64964 |
| GO:0070979 | protein K11-linked ubiquitination | 27 | 1 | 1.03 | 0.64964 |
| GO:1901976 | regulation of cell cycle checkpoint | 27 | 1 | 1.03 | 0.64964 |
| GO:1903078 | positive regulation of protein localizat... | 27 | 1 | 1.03 | 0.64964 |
| GO:0000082 | G1/S transition of mitotic cell cycle | 230 | 8 | 8.76 | 0.65258 |
| GO:0044033 | multi-organism metabolic process | 202 | 7 | 7.69 | 0.65308 |
| GO:0046460 | neutral lipid biosynthetic process | 58 | 2 | 2.21 | 0.65359 |
| GO:0046463 | acylglycerol biosynthetic process | 58 | 2 | 2.21 | 0.65359 |
| GO:0050654 | chondroitin sulfate proteoglycan metabol... | 58 | 2 | 2.21 | 0.65359 |
| GO:0097581 | lamellipodium organization | 58 | 2 | 2.21 | 0.65359 |
| GO:1901216 | positive regulation of neuron death | 58 | 2 | 2.21 | 0.65359 |
| GO:0006874 | cellular calcium ion homeostasis | 314 | 11 | 11.95 | 0.65405 |
| GO:0045185 | maintenance of protein location | 117 | 4 | 4.45 | 0.65566 |
| GO:0006919 | activation of cysteine-type endopeptidas... | 88 | 3 | 3.35 | 0.65643 |

| | | | | | |
|---|---|---|---|---|---|
| GO:0010970 | microtubule-based transport | 88 | 3 | 3.35 | 0.65643 |
| GO:0050806 | positive regulation of synaptic transmis... | 88 | 3 | 3.35 | 0.65643 |
| GO:0051261 | protein depolymerization | 88 | 3 | 3.35 | 0.65643 |
| GO:0051186 | cofactor metabolic process | 259 | 9 | 9.86 | 0.6572 |
| GO:0009141 | nucleoside triphosphate metabolic proces. | 146 | 5 | 5.56 | 0.65722 |
| GO:0043588 | skin development | 231 | 8 | 8.79 | 0.65735 |
| GO:0051098 | regulation of binding | 231 | 8 | 8.79 | 0.65735 |
| GO:0072511 | divalent inorganic cation transport | 371 | 13 | 14.12 | 0.66012 |
| GO:0051251 | positive regulation of lymphocyte activa... | 232 | 8 | 8.83 | 0.66208 |
| GO:0042692 | muscle cell differentiation | 316 | 11 | 12.03 | 0.66222 |
| GO:0007612 | learning | 118 | 4 | 4.49 | 0.66223 |
| GO:0045814 | negative regulation of gene expression ... | | 118 | 4 | 4.49 |
| GO:0097479 | synaptic vesicle localization | 118 | 4 | 4.49 | 0.66223 |
| GO:1902275 | regulation of chromatin organization | 118 | 4 | 4.49 | 0.66223 |
| GO:0006809 | nitric oxide biosynthetic process | 59 | 2 | 2.25 | 0.66281 |
| GO:0007260 | tyrosine phosphorylation of STAT protein | 59 | 2 | 2.25 | 0.66281 |
| GO:0031503 | protein complex localization | 59 | 2 | 2.25 | 0.66281 |
| GO:0035305 | negative regulation of dephosphorylation | 59 | 2 | 2.25 | 0.66281 |
| GO:0000281 | mitotic cytokinesis | 28 | 1 | 1.07 | 0.663 |
| GO:0001782 | B cell homeostasis | 28 | 1 | 1.07 | 0.663 |
| GO:0009072 | aromatic amino acid family metabolic pro.. | 28 | 1 | 1.07 | 0.663 |
| GO:0009268 | response to pH | 28 | 1 | 1.07 | 0.663 |
| GO:0010644 | cell communication by electrical couplin... | 28 | 1 | 1.07 | 0.663 |
| GO:0010799 | regulation of peptidyl-threonine phospho... | 28 | 1 | 1.07 | 0.663 |
| GO:0021884 | forebrain neuron development | 28 | 1 | 1.07 | 0.663 |
| GO:0032715 | negative regulation of interleukin-6 pro... | 28 | 1 | 1.07 | 0.663 |
| GO:0035195 | gene silencing by miRNA | 28 | 1 | 1.07 | 0.663 |
| GO:0043489 | RNA stabilization | 28 | 1 | 1.07 | 0.663 |
| GO:0046825 | regulation of protein export from nucleu... | 28 | 1 | 1.07 | 0.663 |
| GO:0048247 | lymphocyte chemotaxis | 28 | 1 | 1.07 | 0.663 |
| GO:0048255 | mRNA stabilization | 28 | 1 | 1.07 | 0.663 |
| GO:1903202 | negative regulation of oxidative stress-... | 28 | 1 | 1.07 | 0.663 |
| GO:0030308 | negative regulation of cell growth | 147 | 5 | 5.6 | 0.66312 |
| GO:0070585 | protein localization to mitochondrion | 147 | 5 | 5.6 | 0.66312 |
| GO:0045216 | cell-cell junction organization | 204 | 7 | 7.77 | 0.66318 |
| GO:0007368 | determination of left/right symmetry | 89 | 3 | 3.39 | 0.66395 |
| GO:0007018 | microtubule-based movement | 176 | 6 | 6.7 | 0.66529 |
| GO:0010038 | response to metal ion | 261 | 9 | 9.94 | 0.66612 |
| GO:0044843 | cell cycle G1/S phase transition | 233 | 8 | 8.87 | 0.66677 |
| GO:0051170 | nuclear import | 233 | 8 | 8.87 | 0.66677 |
| GO:0090087 | regulation of peptide transport | 233 | 8 | 8.87 | 0.66677 |
| GO:0006518 | peptide metabolic process | 119 | 4 | 4.53 | 0.66871 |
| GO:0048013 | ephrin receptor signaling pathway | 90 | 3 | 3.43 | 0.67135 |
| GO:0051480 | cytosolic calcium ion homeostasis | 234 | 8 | 8.91 | 0.67142 |
| GO:0002260 | lymphocyte homeostasis | 60 | 2 | 2.28 | 0.67183 |
| GO:0002367 | cytokine production involved in immune r... | 60 | 2 | 2.28 | 0.67183 |
| GO:0003143 | embryonic heart tube morphogenesis | 60 | 2 | 2.28 | 0.67183 |
| GO:0007193 | adenylate cyclase-inhibiting G-protein c... | 60 | 2 | 2.28 | 0.67183 |
| GO:0030516 | regulation of axon extension | 60 | 2 | 2.28 | 0.67183 |
| GO:0031058 | positive regulation of histone modificat... | 60 | 2 | 2.28 | 0.67183 |
| GO:0032231 | regulation of actin filament bundle asse... | 60 | 2 | 2.28 | 0.67183 |
| GO:0046902 | regulation of mitochondrial membrane per. | 60 | 2 | 2.28 | 0.67183 |
| GO:0052548 | regulation of endopeptidase activity | 347 | 12 | 13.21 | 0.67451 |

| | | | | | |
|---|---|---|---|---|---|
| GO:0042306 | regulation of protein import into nucleu... | 149 | 5 | 5.67 | 0.6747 |
| GO:0046058 | cAMP metabolic process | 149 | 5 | 5.67 | 0.6747 |
| GO:0044282 | small molecule catabolic process | 263 | 9 | 10.01 | 0.6749 |
| GO:0031348 | negative regulation of defense response | 120 | 4 | 4.57 | 0.67511 |
| GO:0051054 | positive regulation of DNA metabolic pro... | 120 | 4 | 4.57 | 0.67511 |
| GO:0001702 | gastrulation with mouth forming second | 29 | 1 | 1.1 | 0.67586 |
| GO:0002886 | regulation of myeloid leukocyte mediated.. | 29 | 1 | 1.1 | 0.67586 |
| GO:0003230 | cardiac atrium development | 29 | 1 | 1.1 | 0.67586 |
| GO:0009225 | nucleotide-sugar metabolic process | 29 | 1 | 1.1 | 0.67586 |
| GO:0010470 | regulation of gastrulation | 29 | 1 | 1.1 | 0.67586 |
| GO:0014808 | release of sequestered calcium ion into ... | 29 | 1 | 1.1 | 0.67586 |
| GO:0015988 | energy coupled proton transmembrane tra | 29 | 1 | 1.1 | 0.67586 |
| GO:0015991 | ATP hydrolysis coupled proton transport | 29 | 1 | 1.1 | 0.67586 |
| GO:0018149 | peptide cross-linking | 29 | 1 | 1.1 | 0.67586 |
| GO:0019228 | neuronal action potential | 29 | 1 | 1.1 | 0.67586 |
| GO:0031111 | negative regulation of microtubule polym... | 29 | 1 | 1.1 | 0.67586 |
| GO:0034067 | protein localization to Golgi apparatus | 29 | 1 | 1.1 | 0.67586 |
| GO:0034314 | Arp2/3 complex-mediated actin nucleation | 29 | 1 | 1.1 | 0.67586 |
| GO:0042168 | heme metabolic process | 29 | 1 | 1.1 | 0.67586 |
| GO:0043928 | exonucleolytic nuclear-transcribed mRNA . | 29 | 1 | 1.1 | 0.67586 |
| GO:0045923 | positive regulation of fatty acid metabo... | 29 | 1 | 1.1 | 0.67586 |
| GO:0046134 | pyrimidine nucleoside biosynthetic proce... | 29 | 1 | 1.1 | 0.67586 |
| GO:0046513 | ceramide biosynthetic process | 29 | 1 | 1.1 | 0.67586 |
| GO:0048488 | synaptic vesicle endocytosis | 29 | 1 | 1.1 | 0.67586 |
| GO:0050685 | positive regulation of mRNA processing | 29 | 1 | 1.1 | 0.67586 |
| GO:0050690 | regulation of defense response to virus ... | 29 | 1 | 1.1 | 0.67586 |
| GO:0070423 | nucleotide-binding oligomerization domai.. | 29 | 1 | 1.1 | 0.67586 |
| GO:1900408 | negative regulation of cellular response... | 29 | 1 | 1.1 | 0.67586 |
| GO:1902883 | negative regulation of response to oxida... | 29 | 1 | 1.1 | 0.67586 |
| GO:1903513 | endoplasmic reticulum to cytosol transpo... | 29 | 1 | 1.1 | 0.67586 |
| GO:1903514 | calcium ion transport from endoplasmic r... | 29 | 1 | 1.1 | 0.67586 |
| GO:0034329 | cell junction assembly | 207 | 7 | 7.88 | 0.67798 |
| GO:0045807 | positive regulation of endocytosis | 91 | 3 | 3.46 | 0.67863 |
| GO:0051928 | positive regulation of calcium ion trans... | 91 | 3 | 3.46 | 0.67863 |
| GO:0050867 | positive regulation of cell activation | 264 | 9 | 10.05 | 0.67924 |
| GO:0006687 | glycosphingolipid metabolic process | 61 | 2 | 2.32 | 0.68065 |
| GO:0009166 | nucleotide catabolic process | 61 | 2 | 2.32 | 0.68065 |
| GO:0030521 | androgen receptor signaling pathway | 61 | 2 | 2.32 | 0.68065 |
| GO:0045582 | positive regulation of T cell differenti... | 61 | 2 | 2.32 | 0.68065 |
| GO:1903729 | regulation of plasma membrane organizati | 61 | 2 | 2.32 | 0.68065 |
| GO:1903955 | positive regulation of protein targeting... | 61 | 2 | 2.32 | 0.68065 |
| GO:0009205 | purine ribonucleoside triphosphate metab.. | 121 | 4 | 4.61 | 0.68142 |
| GO:0046683 | response to organophosphorus | 121 | 4 | 4.61 | 0.68142 |
| GO:1903510 | mucopolysaccharide metabolic process | 121 | 4 | 4.61 | 0.68142 |
| GO:1903034 | regulation of response to wounding | 349 | 12 | 13.28 | 0.6821 |
| GO:0072507 | divalent inorganic cation homeostasis | 350 | 12 | 13.32 | 0.68585 |
| GO:0044242 | cellular lipid catabolic process | 151 | 5 | 5.75 | 0.68603 |
| GO:0016573 | histone acetylation | 122 | 4 | 4.64 | 0.68764 |
| GO:0072001 | renal system development | 266 | 9 | 10.13 | 0.68781 |
| GO:0000289 | nuclear-transcribed mRNA poly(A) tail sh... | 30 | 1 | 1.14 | 0.68822 |
| GO:0002820 | negative regulation of adaptive immune r... | 30 | 1 | 1.14 | 0.68822 |
| GO:0006362 | transcription elongation from RNA polyme. | 30 | 1 | 1.14 | 0.68822 |
| GO:0006363 | termination of RNA polymerase I transcri... | 30 | 1 | 1.14 | 0.68822 |

| | | | | | |
|---|---|---|---|---|---|
| GO:0006958 | complement activation | classical pa | 30 | 1 | 1.14 |
| GO:0008207 | C21-steroid hormone metabolic process | 30 | 1 | 1.14 | 0.68822 |
| GO:0008333 | endosome to lysosome transport | 30 | 1 | 1.14 | 0.68822 |
| GO:0009154 | purine ribonucleotide catabolic process | 30 | 1 | 1.14 | 0.68822 |
| GO:0010712 | regulation of collagen metabolic process | 30 | 1 | 1.14 | 0.68822 |
| GO:0010765 | positive regulation of sodium ion transp... | 30 | 1 | 1.14 | 0.68822 |
| GO:0021846 | cell proliferation in forebrain | 30 | 1 | 1.14 | 0.68822 |
| GO:0031057 | negative regulation of histone modificat... | 30 | 1 | 1.14 | 0.68822 |
| GO:0031069 | hair follicle morphogenesis | 30 | 1 | 1.14 | 0.68822 |
| GO:0032689 | negative regulation of interferon-gamma ... | 30 | 1 | 1.14 | 0.68822 |
| GO:0035194 | posttranscriptional gene silencing by RN... | 30 | 1 | 1.14 | 0.68822 |
| GO:0040020 | regulation of meiosis | 30 | 1 | 1.14 | 0.68822 |
| GO:0042517 | positive regulation of tyrosine phosphor... | 30 | 1 | 1.14 | 0.68822 |
| GO:0043300 | regulation of leukocyte degranulation | 30 | 1 | 1.14 | 0.68822 |
| GO:0045124 | regulation of bone resorption | 30 | 1 | 1.14 | 0.68822 |
| GO:0045494 | photoreceptor cell maintenance | 30 | 1 | 1.14 | 0.68822 |
| GO:0045851 | pH reduction | 30 | 1 | 1.14 | 0.68822 |
| GO:0046461 | neutral lipid catabolic process | 30 | 1 | 1.14 | 0.68822 |
| GO:0046464 | acylglycerol catabolic process | 30 | 1 | 1.14 | 0.68822 |
| GO:0048512 | circadian behavior | 30 | 1 | 1.14 | 0.68822 |
| GO:0060740 | prostate gland epithelium morphogenesis | 30 | 1 | 1.14 | 0.68822 |
| GO:0098900 | regulation of action potential | 30 | 1 | 1.14 | 0.68822 |
| GO:1902229 | regulation of intrinsic apoptotic signal... | 30 | 1 | 1.14 | 0.68822 |
| GO:0000288 | nuclear-transcribed mRNA catabolic proce | 62 | 2 | 2.36 | 0.68927 |
| GO:0006998 | nuclear envelope organization | 62 | 2 | 2.36 | 0.68927 |
| GO:0042311 | vasodilation | 62 | 2 | 2.36 | 0.68927 |
| GO:0042446 | hormone biosynthetic process | 62 | 2 | 2.36 | 0.68927 |
| GO:0048259 | regulation of receptor-mediated endocyto.. | 62 | 2 | 2.36 | 0.68927 |
| GO:0002683 | negative regulation of immune system pro. | 323 | 11 | 12.3 | 0.68995 |
| GO:0002526 | acute inflammatory response | 123 | 4 | 4.68 | 0.69378 |
| GO:0022617 | extracellular matrix disassembly | 123 | 4 | 4.68 | 0.69378 |
| GO:0034612 | response to tumor necrosis factor | 123 | 4 | 4.68 | 0.69378 |
| GO:0048565 | digestive tract development | 123 | 4 | 4.68 | 0.69378 |
| GO:0055002 | striated muscle cell development | 123 | 4 | 4.68 | 0.69378 |
| GO:0030004 | cellular monovalent inorganic cation hom.. | 63 | 2 | 2.4 | 0.69771 |
| GO:0043647 | inositol phosphate metabolic process | 63 | 2 | 2.4 | 0.69771 |
| GO:1903310 | positive regulation of chromatin modific... | 63 | 2 | 2.4 | 0.69771 |
| GO:0001656 | metanephros development | 94 | 3 | 3.58 | 0.69975 |
| GO:0034614 | cellular response to reactive oxygen spe... | 94 | 3 | 3.58 | 0.69975 |
| GO:0042542 | response to hydrogen peroxide | 94 | 3 | 3.58 | 0.69975 |
| GO:0048704 | embryonic skeletal system morphogenesis | 94 | 3 | 3.58 | 0.69975 |
| GO:0097202 | activation of cysteine-type endopeptidas... | 94 | 3 | 3.58 | 0.69975 |
| GO:0030814 | regulation of cAMP metabolic process | 124 | 4 | 4.72 | 0.69983 |
| GO:0000291 | nuclear-transcribed mRNA catabolic proce | 31 | 1 | 1.18 | 0.70011 |
| GO:0009132 | nucleoside diphosphate metabolic process | 31 | 1 | 1.18 | 0.70011 |
| GO:0009261 | ribonucleotide catabolic process | 31 | 1 | 1.18 | 0.70011 |
| GO:0010324 | membrane invagination | 31 | 1 | 1.18 | 0.70011 |
| GO:0010453 | regulation of cell fate commitment | 31 | 1 | 1.18 | 0.70011 |
| GO:0014014 | negative regulation of gliogenesis | 31 | 1 | 1.18 | 0.70011 |
| GO:0016441 | posttranscriptional gene silencing | 31 | 1 | 1.18 | 0.70011 |
| GO:0032964 | collagen biosynthetic process | 31 | 1 | 1.18 | 0.70011 |
| GO:0034381 | plasma lipoprotein particle clearance | 31 | 1 | 1.18 | 0.70011 |
| GO:0034405 | response to fluid shear stress | 31 | 1 | 1.18 | 0.70011 |

| | | | | | |
|---|---|---|---|---|---|
| GO:0034502 | protein localization to chromosome | 31 | 1 | 1.18 | 0.70011 |
| GO:0036465 | synaptic vesicle recycling | 31 | 1 | 1.18 | 0.70011 |
| GO:0043666 | regulation of phosphoprotein phosphatase | 31 | 1 | 1.18 | 0.70011 |
| GO:0045022 | early endosome to late endosome transpo | 31 | 1 | 1.18 | 0.70011 |
| GO:0045773 | positive regulation of axon extension | 31 | 1 | 1.18 | 0.70011 |
| GO:0046676 | negative regulation of insulin secretion | 31 | 1 | 1.18 | 0.70011 |
| GO:0050869 | negative regulation of B cell activation | 31 | 1 | 1.18 | 0.70011 |
| GO:0060325 | face morphogenesis | 31 | 1 | 1.18 | 0.70011 |
| GO:0070232 | regulation of T cell apoptotic process | 31 | 1 | 1.18 | 0.70011 |
| GO:0072348 | sulfur compound transport | 31 | 1 | 1.18 | 0.70011 |
| GO:2000378 | negative regulation of reactive oxygen s... | 31 | 1 | 1.18 | 0.70011 |
| GO:2000401 | regulation of lymphocyte migration | 31 | 1 | 1.18 | 0.70011 |
| GO:0021700 | developmental maturation | 212 | 7 | 8.07 | 0.70173 |
| GO:0016458 | gene silencing | 125 | 4 | 4.76 | 0.7058 |
| GO:0018393 | internal peptidyl-lysine acetylation | 125 | 4 | 4.76 | 0.7058 |
| GO:0090263 | positive regulation of canonical Wnt sig... | 125 | 4 | 4.76 | 0.7058 |
| GO:0045069 | regulation of viral genome replication | 64 | 2 | 2.44 | 0.70595 |
| GO:0008654 | phospholipid biosynthetic process | 213 | 7 | 8.11 | 0.70634 |
| GO:0010951 | negative regulation of endopeptidase act... | 213 | 7 | 8.11 | 0.70634 |
| GO:0006029 | proteoglycan metabolic process | 95 | 3 | 3.62 | 0.70655 |
| GO:0045580 | regulation of T cell differentiation | 95 | 3 | 3.62 | 0.70655 |
| GO:0006816 | calcium ion transport | 328 | 11 | 12.49 | 0.70889 |
| GO:0045765 | regulation of angiogenesis | 185 | 6 | 7.04 | 0.71129 |
| GO:0006730 | one-carbon metabolic process | 32 | 1 | 1.22 | 0.71155 |
| GO:0007622 | rhythmic behavior | 32 | 1 | 1.22 | 0.71155 |
| GO:0030212 | hyaluronan metabolic process | 32 | 1 | 1.22 | 0.71155 |
| GO:0032633 | interleukin-4 production | 32 | 1 | 1.22 | 0.71155 |
| GO:0034121 | regulation of toll-like receptor signali... | 32 | 1 | 1.22 | 0.71155 |
| GO:0042339 | keratan sulfate metabolic process | 32 | 1 | 1.22 | 0.71155 |
| GO:0042462 | eye photoreceptor cell development | 32 | 1 | 1.22 | 0.71155 |
| GO:0042558 | pteridine-containing compound metabolic . | 32 | 1 | 1.22 | 0.71155 |
| GO:0046329 | negative regulation of JNK cascade | 32 | 1 | 1.22 | 0.71155 |
| GO:0048265 | response to pain | 32 | 1 | 1.22 | 0.71155 |
| GO:0051898 | negative regulation of protein kinase B ... | 32 | 1 | 1.22 | 0.71155 |
| GO:0060512 | prostate gland morphogenesis | 32 | 1 | 1.22 | 0.71155 |
| GO:0060795 | cell fate commitment involved in formati... | 32 | 1 | 1.22 | 0.71155 |
| GO:0070296 | sarcoplasmic reticulum calcium ion trans... | 32 | 1 | 1.22 | 0.71155 |
| GO:0086009 | membrane repolarization | 32 | 1 | 1.22 | 0.71155 |
| GO:0097035 | regulation of membrane lipid distributio... | 32 | 1 | 1.22 | 0.71155 |
| GO:0009199 | ribonucleoside triphosphate metabolic pr... | 126 | 4 | 4.8 | 0.71168 |
| GO:0044236 | multicellular organismal metabolic proce... | 126 | 4 | 4.8 | 0.71168 |
| GO:0001959 | regulation of cytokine-mediated signalin... | 96 | 3 | 3.65 | 0.71323 |
| GO:0006885 | regulation of pH | 65 | 2 | 2.47 | 0.71401 |
| GO:0043149 | stress fiber assembly | 65 | 2 | 2.47 | 0.71401 |
| GO:0072376 | protein activation cascade | 65 | 2 | 2.47 | 0.71401 |
| GO:0009260 | ribonucleotide biosynthetic process | 244 | 8 | 9.29 | 0.71572 |
| GO:0030177 | positive regulation of Wnt signaling pat... | 157 | 5 | 5.98 | 0.71837 |
| GO:0046887 | positive regulation of hormone secretion | 97 | 3 | 3.69 | 0.7198 |
| GO:0072503 | cellular divalent inorganic cation homeo... | 331 | 11 | 12.6 | 0.71991 |
| GO:0007015 | actin filament organization | 274 | 9 | 10.43 | 0.72067 |
| GO:0002444 | myeloid leukocyte mediated immunity | 66 | 2 | 2.51 | 0.72188 |
| GO:0006119 | oxidative phosphorylation | 66 | 2 | 2.51 | 0.72188 |
| GO:0006879 | cellular iron ion homeostasis | 66 | 2 | 2.51 | 0.72188 |

| | | | | | |
|---|---|---|---|---|---|
| GO:0021675 | nerve development | 66 | 2 | 2.51 | 0.72188 |
| GO:0001659 | temperature homeostasis | 33 | 1 | 1.26 | 0.72256 |
| GO:0006206 | pyrimidine nucleobase metabolic process | 33 | 1 | 1.26 | 0.72256 |
| GO:0006400 | tRNA modification | 33 | 1 | 1.26 | 0.72256 |
| GO:0007019 | microtubule depolymerization | 33 | 1 | 1.26 | 0.72256 |
| GO:0010596 | negative regulation of endothelial cell ... | 33 | 1 | 1.26 | 0.72256 |
| GO:0016486 | peptide hormone processing | 33 | 1 | 1.26 | 0.72256 |
| GO:0042181 | ketone biosynthetic process | 33 | 1 | 1.26 | 0.72256 |
| GO:0045214 | sarcomere organization | 33 | 1 | 1.26 | 0.72256 |
| GO:0045843 | negative regulation of striated muscle t... | 33 | 1 | 1.26 | 0.72256 |
| GO:0046638 | positive regulation of alpha-beta T cell... | 33 | 1 | 1.26 | 0.72256 |
| GO:0051568 | histone H3-K4 methylation | 33 | 1 | 1.26 | 0.72256 |
| GO:0055021 | regulation of cardiac muscle tissue grow... | 33 | 1 | 1.26 | 0.72256 |
| GO:0060441 | epithelial tube branching involved in lu... | 33 | 1 | 1.26 | 0.72256 |
| GO:0061098 | positive regulation of protein tyrosine ... | 33 | 1 | 1.26 | 0.72256 |
| GO:0072529 | pyrimidine-containing compound catabolic. | 33 | 1 | 1.26 | 0.72256 |
| GO:1901998 | toxin transport | 33 | 1 | 1.26 | 0.72256 |
| GO:0043524 | negative regulation of neuron apoptotic ... | 128 | 4 | 4.87 | 0.72317 |
| GO:0009855 | determination of bilateral symmetry | 98 | 3 | 3.73 | 0.72624 |
| GO:0055088 | lipid homeostasis | 98 | 3 | 3.73 | 0.72624 |
| GO:0006475 | internal protein amino acid acetylation | 129 | 4 | 4.91 | 0.7288 |
| GO:0006576 | cellular biogenic amine metabolic proces... | 129 | 4 | 4.91 | 0.7288 |
| GO:0009144 | purine nucleoside triphosphate metabolic.. | 129 | 4 | 4.91 | 0.7288 |
| GO:0018394 | peptidyl-lysine acetylation | 129 | 4 | 4.91 | 0.7288 |
| GO:1903649 | regulation of cytoplasmic transport | 362 | 12 | 13.78 | 0.72881 |
| GO:0007200 | phospholipase C-activating G-protein cou.. | 67 | 2 | 2.55 | 0.72956 |
| GO:0008306 | associative learning | 67 | 2 | 2.55 | 0.72956 |
| GO:0043903 | regulation of symbiosis | encompass | 189 | 6 | 7.19 |
| GO:0046390 | ribose phosphate biosynthetic process | 248 | 8 | 9.44 | 0.73229 |
| GO:0009799 | specification of symmetry | 99 | 3 | 3.77 | 0.73257 |
| GO:0007190 | activation of adenylate cyclase activity | 34 | 1 | 1.29 | 0.73314 |
| GO:0022616 | DNA strand elongation | 34 | 1 | 1.29 | 0.73314 |
| GO:0033572 | transferrin transport | 34 | 1 | 1.29 | 0.73314 |
| GO:0042596 | fear response | 34 | 1 | 1.29 | 0.73314 |
| GO:0046622 | positive regulation of organ growth | 34 | 1 | 1.29 | 0.73314 |
| GO:0052646 | alditol phosphate metabolic process | 34 | 1 | 1.29 | 0.73314 |
| GO:0060968 | regulation of gene silencing | 34 | 1 | 1.29 | 0.73314 |
| GO:0060998 | regulation of dendritic spine developmen... | 34 | 1 | 1.29 | 0.73314 |
| GO:0071398 | cellular response to fatty acid | 34 | 1 | 1.29 | 0.73314 |
| GO:1901862 | negative regulation of muscle tissue dev... | 34 | 1 | 1.29 | 0.73314 |
| GO:0007586 | digestion | 130 | 4 | 4.95 | 0.73433 |
| GO:0044801 | single-organism membrane fusion | 130 | 4 | 4.95 | 0.73433 |
| GO:0006342 | chromatin silencing | 68 | 2 | 2.59 | 0.73707 |
| GO:0006970 | response to osmotic stress | 68 | 2 | 2.59 | 0.73707 |
| GO:1901292 | nucleoside phosphate catabolic process | 68 | 2 | 2.59 | 0.73707 |
| GO:1903214 | regulation of protein targeting to mitoc... | 68 | 2 | 2.59 | 0.73707 |
| GO:0001657 | ureteric bud development | 100 | 3 | 3.81 | 0.73879 |
| GO:0006892 | post-Golgi vesicle-mediated transport | 100 | 3 | 3.81 | 0.73879 |
| GO:0035051 | cardiocyte differentiation | 100 | 3 | 3.81 | 0.73879 |
| GO:0052547 | regulation of peptidase activity | 365 | 12 | 13.89 | 0.73893 |
| GO:0006766 | vitamin metabolic process | 131 | 4 | 4.99 | 0.73978 |
| GO:0050663 | cytokine secretion | 131 | 4 | 4.99 | 0.73978 |
| GO:0090150 | establishment of protein localization to... | 308 | 10 | 11.72 | 0.74034 |

| | | | | | |
|---|---|---|---|---|---|
| GO:0010466 | negative regulation of peptidase activit... | 221 | 7 | 8.41 | 0.74151 |
| GO:0006378 | mRNA polyadenylation | 35 | 1 | 1.33 | 0.74332 |
| GO:0007628 | adult walking behavior | 35 | 1 | 1.33 | 0.74332 |
| GO:0009409 | response to cold | 35 | 1 | 1.33 | 0.74332 |
| GO:0021522 | spinal cord motor neuron differentiation | 35 | 1 | 1.33 | 0.74332 |
| GO:0032467 | positive regulation of cytokinesis | 35 | 1 | 1.33 | 0.74332 |
| GO:0032648 | regulation of interferon-beta production | 35 | 1 | 1.33 | 0.74332 |
| GO:0034453 | microtubule anchoring | 35 | 1 | 1.33 | 0.74332 |
| GO:0048635 | negative regulation of muscle organ deve.. | 35 | 1 | 1.33 | 0.74332 |
| GO:0048730 | epidermis morphogenesis | 35 | 1 | 1.33 | 0.74332 |
| GO:0050873 | brown fat cell differentiation | 35 | 1 | 1.33 | 0.74332 |
| GO:0051057 | positive regulation of small GTPase medi.. | 35 | 1 | 1.33 | 0.74332 |
| GO:0051932 | synaptic transmission | GABAergic | 35 | 1 | 1.33 |
| GO:0060038 | cardiac muscle cell proliferation | 35 | 1 | 1.33 | 0.74332 |
| GO:0072384 | organelle transport along microtubule | 35 | 1 | 1.33 | 0.74332 |
| GO:1903305 | regulation of regulated secretory pathwa... | 35 | 1 | 1.33 | 0.74332 |
| GO:2000278 | regulation of DNA biosynthetic process | 35 | 1 | 1.33 | 0.74332 |
| GO:0061025 | membrane fusion | 162 | 5 | 6.17 | 0.74346 |
| GO:2000177 | regulation of neural precursor cell prol... | 69 | 2 | 2.63 | 0.7444 |
| GO:0006261 | DNA-dependent DNA replication | 101 | 3 | 3.84 | 0.74489 |
| GO:0072163 | mesonephric epithelium development | 101 | 3 | 3.84 | 0.74489 |
| GO:0072164 | mesonephric tubule development | 101 | 3 | 3.84 | 0.74489 |
| GO:0032846 | positive regulation of homeostatic proce... | 132 | 4 | 5.02 | 0.74514 |
| GO:0072089 | stem cell proliferation | 132 | 4 | 5.02 | 0.74514 |
| GO:0015711 | organic anion transport | 367 | 12 | 13.97 | 0.74554 |
| GO:0044708 | single-organism behavior | 367 | 12 | 13.97 | 0.74554 |
| GO:0060249 | anatomical structure homeostasis | 281 | 9 | 10.7 | 0.7475 |
| GO:0001822 | kidney development | 252 | 8 | 9.59 | 0.74819 |
| GO:0040029 | regulation of gene expression | epigeneti... | 223 | 7 | 8.49 |
| GO:0048771 | tissue remodeling | 133 | 4 | 5.06 | 0.75043 |
| GO:0055123 | digestive system development | 133 | 4 | 5.06 | 0.75043 |
| GO:0001906 | cell killing | 102 | 3 | 3.88 | 0.75088 |
| GO:0060759 | regulation of response to cytokine stimu... | 102 | 3 | 3.88 | 0.75088 |
| GO:0002708 | positive regulation of lymphocyte mediat... | 70 | 2 | 2.66 | 0.75155 |
| GO:0031295 | T cell costimulation | 70 | 2 | 2.66 | 0.75155 |
| GO:0000413 | protein peptidyl-prolyl isomerization | 36 | 1 | 1.37 | 0.75312 |
| GO:0000578 | embryonic axis specification | 36 | 1 | 1.37 | 0.75312 |
| GO:0002711 | positive regulation of T cell mediated i... | 36 | 1 | 1.37 | 0.75312 |
| GO:0003254 | regulation of membrane depolarization | 36 | 1 | 1.37 | 0.75312 |
| GO:0007435 | salivary gland morphogenesis | 36 | 1 | 1.37 | 0.75312 |
| GO:0014910 | regulation of smooth muscle cell migrati... | 36 | 1 | 1.37 | 0.75312 |
| GO:0033865 | nucleoside bisphosphate metabolic proces | 36 | 1 | 1.37 | 0.75312 |
| GO:0033875 | ribonucleoside bisphosphate metabolic pr.. | 36 | 1 | 1.37 | 0.75312 |
| GO:0034032 | purine nucleoside bisphosphate metabolic. | 36 | 1 | 1.37 | 0.75312 |
| GO:0046850 | regulation of bone remodeling | 36 | 1 | 1.37 | 0.75312 |
| GO:0048260 | positive regulation of receptor-mediated... | 36 | 1 | 1.37 | 0.75312 |
| GO:0051220 | cytoplasmic sequestering of protein | 36 | 1 | 1.37 | 0.75312 |
| GO:0060323 | head morphogenesis | 36 | 1 | 1.37 | 0.75312 |
| GO:0061640 | cytoskeleton-dependent cytokinesis | 36 | 1 | 1.37 | 0.75312 |
| GO:0072678 | T cell migration | 36 | 1 | 1.37 | 0.75312 |
| GO:2000649 | regulation of sodium ion transmembrane t. | 36 | 1 | 1.37 | 0.75312 |
| GO:0070972 | protein localization to endoplasmic reti... | 134 | 4 | 5.1 | 0.75562 |
| GO:0002696 | positive regulation of leukocyte activat... | 254 | 8 | 9.67 | 0.75589 |

| | | | | | |
|---|---|---|---|---|---|
| GO:0002449 | lymphocyte mediated immunity | 195 | 6 | 7.42 | 0.7571 |
| GO:0001776 | leukocyte homeostasis | 71 | 2 | 2.7 | 0.75853 |
| GO:0031294 | lymphocyte costimulation | 71 | 2 | 2.7 | 0.75853 |
| GO:0045055 | regulated secretory pathway | 71 | 2 | 2.7 | 0.75853 |
| GO:0014074 | response to purine-containing compound | 135 | 4 | 5.14 | 0.76073 |
| GO:0055001 | muscle cell development | 135 | 4 | 5.14 | 0.76073 |
| GO:0009913 | epidermal cell differentiation | 166 | 5 | 6.32 | 0.76233 |
| GO:2000021 | regulation of ion homeostasis | 166 | 5 | 6.32 | 0.76233 |
| GO:0032543 | mitochondrial translation | 104 | 3 | 3.96 | 0.76251 |
| GO:0051100 | negative regulation of binding | 104 | 3 | 3.96 | 0.76251 |
| GO:0002279 | mast cell activation involved in immune ... | 37 | 1 | 1.41 | 0.76254 |
| GO:0006144 | purine nucleobase metabolic process | 37 | 1 | 1.41 | 0.76254 |
| GO:0007339 | binding of sperm to zona pellucida | 37 | 1 | 1.41 | 0.76254 |
| GO:0010883 | regulation of lipid storage | 37 | 1 | 1.41 | 0.76254 |
| GO:0014015 | positive regulation of gliogenesis | 37 | 1 | 1.41 | 0.76254 |
| GO:0032608 | interferon-beta production | 37 | 1 | 1.41 | 0.76254 |
| GO:0032757 | positive regulation of interleukin-8 pro... | 37 | 1 | 1.41 | 0.76254 |
| GO:0032873 | negative regulation of stress-activated ... | 37 | 1 | 1.41 | 0.76254 |
| GO:0042551 | neuron maturation | 37 | 1 | 1.41 | 0.76254 |
| GO:0043303 | mast cell degranulation | 37 | 1 | 1.41 | 0.76254 |
| GO:0043631 | RNA polyadenylation | 37 | 1 | 1.41 | 0.76254 |
| GO:0045010 | actin nucleation | 37 | 1 | 1.41 | 0.76254 |
| GO:0046686 | response to cadmium ion | 37 | 1 | 1.41 | 0.76254 |
| GO:0060291 | long-term synaptic potentiation | 37 | 1 | 1.41 | 0.76254 |
| GO:0070303 | negative regulation of stress-activated ... | 37 | 1 | 1.41 | 0.76254 |
| GO:0072577 | endothelial cell apoptotic process | 37 | 1 | 1.41 | 0.76254 |
| GO:0035019 | somatic stem cell maintenance | 72 | 2 | 2.74 | 0.76534 |
| GO:0045621 | positive regulation of lymphocyte differ... | 72 | 2 | 2.74 | 0.76534 |
| GO:0007601 | visual perception | 197 | 6 | 7.5 | 0.7656 |
| GO:1903036 | positive regulation of response to wound... | 136 | 4 | 5.18 | 0.76576 |
| GO:0050714 | positive regulation of protein secretion | 167 | 5 | 6.36 | 0.76687 |
| GO:0070372 | regulation of ERK1 and ERK2 cascade | 167 | 5 | 6.36 | 0.76687 |
| GO:0002443 | leukocyte mediated immunity | 257 | 8 | 9.78 | 0.76713 |
| GO:0001823 | mesonephros development | 105 | 3 | 4 | 0.76816 |
| GO:0030183 | B cell differentiation | 105 | 3 | 4 | 0.76816 |
| GO:0045834 | positive regulation of lipid metabolic p... | 105 | 3 | 4 | 0.76816 |
| GO:0046425 | regulation of JAK-STAT cascade | 105 | 3 | 4 | 0.76816 |
| GO:0007009 | plasma membrane organization | 228 | 7 | 8.68 | 0.76976 |
| GO:0001954 | positive regulation of cell-matrix adhes... | 38 | 1 | 1.45 | 0.7716 |
| GO:0001974 | blood vessel remodeling | 38 | 1 | 1.45 | 0.7716 |
| GO:0006778 | porphyrin-containing compound metabolic | 38 | 1 | 1.45 | 0.7716 |
| GO:0016574 | histone ubiquitination | 38 | 1 | 1.45 | 0.7716 |
| GO:0030890 | positive regulation of B cell proliferat... | 38 | 1 | 1.45 | 0.7716 |
| GO:0032784 | regulation of DNA-templated transcriptio... | 38 | 1 | 1.45 | 0.7716 |
| GO:0045104 | intermediate filament cytoskeleton organ... | 38 | 1 | 1.45 | 0.7716 |
| GO:0045620 | negative regulation of lymphocyte differ... | 38 | 1 | 1.45 | 0.7716 |
| GO:0051205 | protein insertion into membrane | 38 | 1 | 1.45 | 0.7716 |
| GO:0060338 | regulation of type I interferon-mediated... | 38 | 1 | 1.45 | 0.7716 |
| GO:0071377 | cellular response to glucagon stimulus | 38 | 1 | 1.45 | 0.7716 |
| GO:2000737 | negative regulation of stem cell differe... | 38 | 1 | 1.45 | 0.7716 |
| GO:0015748 | organophosphate ester transport | 73 | 2 | 2.78 | 0.77198 |
| GO:0021536 | diencephalon development | 73 | 2 | 2.78 | 0.77198 |
| GO:0030574 | collagen catabolic process | 73 | 2 | 2.78 | 0.77198 |

| | | | | | |
|---|---|---|---|---|---|
| GO:0034968 | histone lysine methylation | 73 | 2 | 2.78 | 0.77198 |
| GO:0046209 | nitric oxide metabolic process | 73 | 2 | 2.78 | 0.77198 |
| GO:0050684 | regulation of mRNA processing | 73 | 2 | 2.78 | 0.77198 |
| GO:0043393 | regulation of protein binding | 138 | 4 | 5.25 | 0.77557 |
| GO:0050731 | positive regulation of peptidyl-tyrosine... | 138 | 4 | 5.25 | 0.77557 |
| GO:0048754 | branching morphogenesis of an epithelial.. | 169 | 5 | 6.43 | 0.77577 |
| GO:0008544 | epidermis development | 289 | 9 | 11 | 0.77593 |
| GO:0043241 | protein complex disassembly | 289 | 9 | 11 | 0.77593 |
| GO:0006887 | exocytosis | 348 | 11 | 13.25 | 0.77718 |
| GO:0006575 | cellular modified amino acid metabolic p... | 200 | 6 | 7.61 | 0.77792 |
| GO:0030838 | positive regulation of actin filament po... | 74 | 2 | 2.82 | 0.77846 |
| GO:0045761 | regulation of adenylate cyclase activity | 74 | 2 | 2.82 | 0.77846 |
| GO:2000379 | positive regulation of reactive oxygen s... | 74 | 2 | 2.82 | 0.77846 |
| GO:2001057 | reactive nitrogen species metabolic proc... | 74 | 2 | 2.82 | 0.77846 |
| GO:0006614 | SRP-dependent cotranslational protein ta.. | 107 | 3 | 4.07 | 0.77913 |
| GO:0032963 | collagen metabolic process | 107 | 3 | 4.07 | 0.77913 |
| GO:0090002 | establishment of protein localization to... | 107 | 3 | 4.07 | 0.77913 |
| GO:0000302 | response to reactive oxygen species | 170 | 5 | 6.47 | 0.78012 |
| GO:0001569 | patterning of blood vessels | 39 | 1 | 1.48 | 0.78032 |
| GO:0007431 | salivary gland development | 39 | 1 | 1.48 | 0.78032 |
| GO:0030049 | muscle filament sliding | 39 | 1 | 1.48 | 0.78032 |
| GO:0033275 | actin-myosin filament sliding | 39 | 1 | 1.48 | 0.78032 |
| GO:0038032 | termination of G-protein coupled recepto... | 39 | 1 | 1.48 | 0.78032 |
| GO:0045103 | intermediate filament-based process | 39 | 1 | 1.48 | 0.78032 |
| GO:0045599 | negative regulation of fat cell differen... | 39 | 1 | 1.48 | 0.78032 |
| GO:0045661 | regulation of myoblast differentiation | 39 | 1 | 1.48 | 0.78032 |
| GO:0046503 | glycerolipid catabolic process | 39 | 1 | 1.48 | 0.78032 |
| GO:0050853 | B cell receptor signaling pathway | 39 | 1 | 1.48 | 0.78032 |
| GO:0071230 | cellular response to amino acid stimulus | 39 | 1 | 1.48 | 0.78032 |
| GO:1903309 | negative regulation of chromatin modific... | 39 | 1 | 1.48 | 0.78032 |
| GO:0007163 | establishment or maintenance of cell pol... | 139 | 4 | 5.29 | 0.78036 |
| GO:0009152 | purine ribonucleotide biosynthetic proce... | 231 | 7 | 8.79 | 0.78116 |
| GO:1903533 | regulation of protein targeting | 231 | 7 | 8.79 | 0.78116 |
| GO:0050953 | sensory perception of light stimulus | 201 | 6 | 7.65 | 0.78192 |
| GO:0000226 | microtubule cytoskeleton organization | 321 | 10 | 12.22 | 0.78433 |
| GO:0006612 | protein targeting to membrane | 171 | 5 | 6.51 | 0.78441 |
| GO:0048839 | inner ear development | 171 | 5 | 6.51 | 0.78441 |
| GO:0018022 | peptidyl-lysine methylation | 75 | 2 | 2.85 | 0.78477 |
| GO:0050715 | positive regulation of cytokine secretio... | 75 | 2 | 2.85 | 0.78477 |
| GO:0043603 | cellular amide metabolic process | 262 | 8 | 9.97 | 0.78503 |
| GO:0034330 | cell junction organization | 233 | 7 | 8.87 | 0.78852 |
| GO:0002762 | negative regulation of myeloid leukocyte... | 40 | 1 | 1.52 | 0.7887 |
| GO:0010043 | response to zinc ion | 40 | 1 | 1.52 | 0.7887 |
| GO:0010623 | developmental programmed cell death | 40 | 1 | 1.52 | 0.7887 |
| GO:0010677 | negative regulation of cellular carbohyd... | 40 | 1 | 1.52 | 0.7887 |
| GO:0010830 | regulation of myotube differentiation | 40 | 1 | 1.52 | 0.7887 |
| GO:0021545 | cranial nerve development | 40 | 1 | 1.52 | 0.7887 |
| GO:0030514 | negative regulation of BMP signaling pat... | 40 | 1 | 1.52 | 0.7887 |
| GO:0031060 | regulation of histone methylation | 40 | 1 | 1.52 | 0.7887 |
| GO:0034308 | primary alcohol metabolic process | 40 | 1 | 1.52 | 0.7887 |
| GO:0042461 | photoreceptor cell development | 40 | 1 | 1.52 | 0.7887 |
| GO:0046637 | regulation of alpha-beta T cell differen... | 40 | 1 | 1.52 | 0.7887 |
| GO:2000179 | positive regulation of neural precursor ... | 40 | 1 | 1.52 | 0.7887 |

| | | | | | |
|---|---|---|---|---|---|
| GO:0006613 | cotranslational protein targeting to mem... | 109 | 3 | 4.15 | 0.78967 |
| GO:0030817 | regulation of cAMP biosynthetic process | 109 | 3 | 4.15 | 0.78967 |
| GO:0008277 | regulation of G-protein coupled receptor... | 141 | 4 | 5.37 | 0.78968 |
| GO:0009636 | response to toxic substance | 141 | 4 | 5.37 | 0.78968 |
| GO:0035249 | synaptic transmission | glutamaterg | 76 | 2 | 2.89 |
| GO:1903409 | reactive oxygen species biosynthetic pro... | 76 | 2 | 2.89 | 0.79093 |
| GO:0009259 | ribonucleotide metabolic process | 382 | 12 | 14.54 | 0.79156 |
| GO:0010035 | response to inorganic substance | 382 | 12 | 14.54 | 0.79156 |
| GO:0048871 | multicellular organismal homeostasis | 234 | 7 | 8.91 | 0.79214 |
| GO:0006415 | translational termination | 173 | 5 | 6.59 | 0.79279 |
| GO:0050670 | regulation of lymphocyte proliferation | 173 | 5 | 6.59 | 0.79279 |
| GO:0045766 | positive regulation of angiogenesis | 110 | 3 | 4.19 | 0.79478 |
| GO:0043624 | cellular protein complex disassembly | 265 | 8 | 10.09 | 0.79527 |
| GO:0002292 | T cell differentiation involved in immun... | 41 | 1 | 1.56 | 0.79677 |
| GO:0007040 | lysosome organization | 41 | 1 | 1.56 | 0.79677 |
| GO:0010833 | telomere maintenance via telomere length. | 41 | 1 | 1.56 | 0.79677 |
| GO:0014909 | smooth muscle cell migration | 41 | 1 | 1.56 | 0.79677 |
| GO:0042345 | regulation of NF-kappaB import into nucl... | 41 | 1 | 1.56 | 0.79677 |
| GO:0042348 | NF-kappaB import into nucleus | 41 | 1 | 1.56 | 0.79677 |
| GO:0043113 | receptor clustering | 41 | 1 | 1.56 | 0.79677 |
| GO:0045776 | negative regulation of blood pressure | 41 | 1 | 1.56 | 0.79677 |
| GO:0048024 | regulation of mRNA splicing | via spliceo. | 41 | 1 | 1.56 |
| GO:0080171 | lytic vacuole organization | 41 | 1 | 1.56 | 0.79677 |
| GO:1901028 | regulation of mitochondrial outer membra.. | 41 | 1 | 1.56 | 0.79677 |
| GO:0007160 | cell-matrix adhesion | 174 | 5 | 6.62 | 0.79688 |
| GO:0032944 | regulation of mononuclear cell prolifera... | 174 | 5 | 6.62 | 0.79688 |
| GO:0031334 | positive regulation of protein complex a... | 143 | 4 | 5.44 | 0.79868 |
| GO:0045047 | protein targeting to ER | 111 | 3 | 4.23 | 0.79979 |
| GO:0042113 | B cell activation | 206 | 6 | 7.84 | 0.8011 |
| GO:0051258 | protein polymerization | 206 | 6 | 7.84 | 0.8011 |
| GO:0051402 | neuron apoptotic process | 206 | 6 | 7.84 | 0.8011 |
| GO:0006164 | purine nucleotide biosynthetic process | 237 | 7 | 9.02 | 0.80269 |
| GO:0030901 | midbrain development | 78 | 2 | 2.97 | 0.80278 |
| GO:0032680 | regulation of tumor necrosis factor prod... | 78 | 2 | 2.97 | 0.80278 |
| GO:0060337 | type I interferon signaling pathway | 78 | 2 | 2.97 | 0.80278 |
| GO:0071357 | cellular response to type I interferon | 78 | 2 | 2.97 | 0.80278 |
| GO:0002753 | cytoplasmic pattern recognition receptor... | 42 | 1 | 1.6 | 0.80453 |
| GO:0007566 | embryo implantation | 42 | 1 | 1.6 | 0.80453 |
| GO:0023021 | termination of signal transduction | 42 | 1 | 1.6 | 0.80453 |
| GO:0050994 | regulation of lipid catabolic process | 42 | 1 | 1.6 | 0.80453 |
| GO:0071320 | cellular response to cAMP | 42 | 1 | 1.6 | 0.80453 |
| GO:0006997 | nucleus organization | 112 | 3 | 4.26 | 0.8047 |
| GO:0032984 | macromolecular complex disassembly | 298 | 9 | 11.34 | 0.80508 |
| GO:0019827 | stem cell maintenance | 145 | 4 | 5.52 | 0.80737 |
| GO:0032640 | tumor necrosis factor production | 79 | 2 | 3.01 | 0.80848 |
| GO:0034340 | response to type I interferon | 79 | 2 | 3.01 | 0.80848 |
| GO:0043543 | protein acylation | 177 | 5 | 6.74 | 0.80877 |
| GO:0006479 | protein methylation | 113 | 3 | 4.3 | 0.8095 |
| GO:0008213 | protein alkylation | 113 | 3 | 4.3 | 0.8095 |
| GO:0044259 | multicellular organismal macromolecule m. | 113 | 3 | 4.3 | 0.8095 |
| GO:0046434 | organophosphate catabolic process | 113 | 3 | 4.3 | 0.8095 |
| GO:0060402 | calcium ion transport into cytosol | 113 | 3 | 4.3 | 0.8095 |
| GO:0006473 | protein acetylation | 146 | 4 | 5.56 | 0.8116 |

| | | | | | |
|---|---|---|---|---|---|
| GO:1902105 | regulation of leukocyte differentiation | 209 | 6 | 7.96 | 0.81197 |
| GO:0016447 | somatic recombination of immunoglobulin | 43 | 1 | 1.64 | 0.81199 |
| GO:0033209 | tumor necrosis factor-mediated signaling... | 43 | 1 | 1.64 | 0.81199 |
| GO:0034113 | heterotypic cell-cell adhesion | 43 | 1 | 1.64 | 0.81199 |
| GO:0038083 | peptidyl-tyrosine autophosphorylation | 43 | 1 | 1.64 | 0.81199 |
| GO:0045071 | negative regulation of viral genome repl... | 43 | 1 | 1.64 | 0.81199 |
| GO:2000738 | positive regulation of stem cell differe... | 43 | 1 | 1.64 | 0.81199 |
| GO:0001894 | tissue homeostasis | 178 | 5 | 6.78 | 0.81262 |
| GO:0042445 | hormone metabolic process | 178 | 5 | 6.78 | 0.81262 |
| GO:0044243 | multicellular organismal catabolic proce... | 80 | 2 | 3.05 | 0.81403 |
| GO:0051341 | regulation of oxidoreductase activity | 80 | 2 | 3.05 | 0.81403 |
| GO:0060401 | cytosolic calcium ion transport | 114 | 3 | 4.34 | 0.81421 |
| GO:0070371 | ERK1 and ERK2 cascade | 179 | 5 | 6.81 | 0.81639 |
| GO:0070663 | regulation of leukocyte proliferation | 179 | 5 | 6.81 | 0.81639 |
| GO:1903532 | positive regulation of secretion by cell | 272 | 8 | 10.35 | 0.81774 |
| GO:0031638 | zymogen activation | 115 | 3 | 4.38 | 0.81881 |
| GO:0072599 | establishment of protein localization to... | 115 | 3 | 4.38 | 0.81881 |
| GO:0008631 | intrinsic apoptotic signaling pathway in... | 44 | 1 | 1.67 | 0.81917 |
| GO:0014855 | striated muscle cell proliferation | 44 | 1 | 1.67 | 0.81917 |
| GO:0031663 | lipopolysaccharide-mediated signaling pa.. | 44 | 1 | 1.67 | 0.81917 |
| GO:0032418 | lysosome localization | 44 | 1 | 1.67 | 0.81917 |
| GO:0043535 | regulation of blood vessel endothelial c... | 44 | 1 | 1.67 | 0.81917 |
| GO:0048384 | retinoic acid receptor signaling pathway | 44 | 1 | 1.67 | 0.81917 |
| GO:0051084 | 'de novo' posttranslational protein fold... | 44 | 1 | 1.67 | 0.81917 |
| GO:0070936 | protein K48-linked ubiquitination | 44 | 1 | 1.67 | 0.81917 |
| GO:0090502 | RNA phosphodiester bond hydrolysis endo... | | 44 | 1 | 1.67 |
| GO:1902305 | regulation of sodium ion transmembrane t. | 44 | 1 | 1.67 | 0.81917 |
| GO:0002700 | regulation of production of molecular me... | 81 | 2 | 3.08 | 0.81943 |
| GO:0008344 | adult locomotory behavior | 81 | 2 | 3.08 | 0.81943 |
| GO:0045638 | negative regulation of myeloid cell diff... | 81 | 2 | 3.08 | 0.81943 |
| GO:1903555 | regulation of tumor necrosis factor supe... | 81 | 2 | 3.08 | 0.81943 |
| GO:0007269 | neurotransmitter secretion | 148 | 4 | 5.63 | 0.81983 |
| GO:0030799 | regulation of cyclic nucleotide metaboli... | 148 | 4 | 5.63 | 0.81983 |
| GO:0050671 | positive regulation of lymphocyte prolif... | 116 | 3 | 4.42 | 0.82332 |
| GO:0001818 | negative regulation of cytokine producti... | 181 | 5 | 6.89 | 0.82377 |
| GO:0030198 | extracellular matrix organization | 365 | 11 | 13.89 | 0.82568 |
| GO:0002455 | humoral immune response mediated by ciı | 45 | 1 | 1.71 | 0.82607 |
| GO:0006195 | purine nucleotide catabolic process | 45 | 1 | 1.71 | 0.82607 |
| GO:0006749 | glutathione metabolic process | 45 | 1 | 1.71 | 0.82607 |
| GO:0033059 | cellular pigmentation | 45 | 1 | 1.71 | 0.82607 |
| GO:0035987 | endodermal cell differentiation | 45 | 1 | 1.71 | 0.82607 |
| GO:0051298 | centrosome duplication | 45 | 1 | 1.71 | 0.82607 |
| GO:0051453 | regulation of intracellular pH | 45 | 1 | 1.71 | 0.82607 |
| GO:0051865 | protein autoubiquitination | 45 | 1 | 1.71 | 0.82607 |
| GO:0051966 | regulation of synaptic transmission glu... | | 45 | 1 | 1.71 |
| GO:0032946 | positive regulation of mononuclear cell ... | 117 | 3 | 4.45 | 0.82773 |
| GO:0043062 | extracellular structure organization | 366 | 11 | 13.93 | 0.82826 |
| GO:0055007 | cardiac muscle cell differentiation | 83 | 2 | 3.16 | 0.82983 |
| GO:0009150 | purine ribonucleotide metabolic process | 367 | 11 | 13.97 | 0.83082 |
| GO:0019693 | ribose phosphate metabolic process | 397 | 12 | 15.11 | 0.8313 |
| GO:0048284 | organelle fusion | 118 | 3 | 4.49 | 0.83205 |
| GO:0010633 | negative regulation of epithelial cell m... | 46 | 1 | 1.75 | 0.83271 |
| GO:0010862 | positive regulation of pathway-restricte... | 46 | 1 | 1.75 | 0.83271 |

| | | | | | |
|---|---|---|---|---|---|
| GO:0031110 | regulation of microtubule polymerization... | 46 | 1 | 1.75 | 0.83271 |
| GO:0035036 | sperm-egg recognition | 46 | 1 | 1.75 | 0.83271 |
| GO:0046635 | positive regulation of alpha-beta T cell... | 46 | 1 | 1.75 | 0.83271 |
| GO:0048146 | positive regulation of fibroblast prolif... | 46 | 1 | 1.75 | 0.83271 |
| GO:0050805 | negative regulation of synaptic transmis... | 46 | 1 | 1.75 | 0.83271 |
| GO:0051145 | smooth muscle cell differentiation | 46 | 1 | 1.75 | 0.83271 |
| GO:1901379 | regulation of potassium ion transmembran | 46 | 1 | 1.75 | 0.83271 |
| GO:0043523 | regulation of neuron apoptotic process | 184 | 5 | 7 | 0.83438 |
| GO:0042100 | B cell proliferation | 84 | 2 | 3.2 | 0.83482 |
| GO:0042102 | positive regulation of T cell proliferat... | 84 | 2 | 3.2 | 0.83482 |
| GO:0071897 | DNA biosynthetic process | 84 | 2 | 3.2 | 0.83482 |
| GO:0006959 | humoral immune response | 152 | 4 | 5.79 | 0.83541 |
| GO:0051495 | positive regulation of cytoskeleton orga... | 152 | 4 | 5.79 | 0.83541 |
| GO:1901215 | negative regulation of neuron death | 152 | 4 | 5.79 | 0.83541 |
| GO:0072593 | reactive oxygen species metabolic proces. | 216 | 6 | 8.22 | 0.83552 |
| GO:0006171 | cAMP biosynthetic process | 119 | 3 | 4.53 | 0.83627 |
| GO:0070374 | positive regulation of ERK1 and ERK2 cas | 119 | 3 | 4.53 | 0.83627 |
| GO:1904018 | positive regulation of vasculature devel... | 119 | 3 | 4.53 | 0.83627 |
| GO:0006213 | pyrimidine nucleoside metabolic process | 47 | 1 | 1.79 | 0.8391 |
| GO:0016266 | O-glycan processing | 47 | 1 | 1.79 | 0.8391 |
| GO:0016445 | somatic diversification of immunoglobuli... | 47 | 1 | 1.79 | 0.8391 |
| GO:0021517 | ventral spinal cord development | 47 | 1 | 1.79 | 0.8391 |
| GO:0030641 | regulation of cellular pH | 47 | 1 | 1.79 | 0.8391 |
| GO:0035872 | nucleotide-binding domain | leucine rich | 47 | 1 | 1.79 |
| GO:0043966 | histone H3 acetylation | 47 | 1 | 1.79 | 0.8391 |
| GO:0045912 | negative regulation of carbohydrate meta.. | 47 | 1 | 1.79 | 0.8391 |
| GO:0070665 | positive regulation of leukocyte prolife... | 120 | 3 | 4.57 | 0.84041 |
| GO:0016042 | lipid catabolic process | 250 | 7 | 9.52 | 0.84374 |
| GO:0007218 | neuropeptide signaling pathway | 86 | 2 | 3.27 | 0.84441 |
| GO:0007229 | integrin-mediated signaling pathway | 86 | 2 | 3.27 | 0.84441 |
| GO:0032273 | positive regulation of protein polymeriz... | 86 | 2 | 3.27 | 0.84441 |
| GO:0032635 | interleukin-6 production | 86 | 2 | 3.27 | 0.84441 |
| GO:0010822 | positive regulation of mitochondrion org... | 121 | 3 | 4.61 | 0.84445 |
| GO:0071222 | cellular response to lipopolysaccharide | 121 | 3 | 4.61 | 0.84445 |
| GO:0001505 | regulation of neurotransmitter levels | 187 | 5 | 7.12 | 0.84446 |
| GO:0032677 | regulation of interleukin-8 production | 48 | 1 | 1.83 | 0.84525 |
| GO:0042531 | positive regulation of tyrosine phosphor... | 48 | 1 | 1.83 | 0.84525 |
| GO:0043525 | positive regulation of neuron apoptotic ... | 48 | 1 | 1.83 | 0.84525 |
| GO:0045762 | positive regulation of adenylate cyclase... | 48 | 1 | 1.83 | 0.84525 |
| GO:0048678 | response to axon injury | 48 | 1 | 1.83 | 0.84525 |
| GO:0009187 | cyclic nucleotide metabolic process | 188 | 5 | 7.16 | 0.84771 |
| GO:0045619 | regulation of lymphocyte differentiation | 122 | 3 | 4.64 | 0.8484 |
| GO:0007043 | cell-cell junction assembly | 87 | 2 | 3.31 | 0.84901 |
| GO:1903749 | positive regulation of establishment of ... | 87 | 2 | 3.31 | 0.84901 |
| GO:0033157 | regulation of intracellular protein tran... | 283 | 8 | 10.77 | 0.84917 |
| GO:0006458 | 'de novo' protein folding | 49 | 1 | 1.87 | 0.85116 |
| GO:0019362 | pyridine nucleotide metabolic process | 49 | 1 | 1.87 | 0.85116 |
| GO:0019748 | secondary metabolic process | 49 | 1 | 1.87 | 0.85116 |
| GO:0030032 | lamellipodium assembly | 49 | 1 | 1.87 | 0.85116 |
| GO:0033762 | response to glucagon | 49 | 1 | 1.87 | 0.85116 |
| GO:0045453 | bone resorption | 49 | 1 | 1.87 | 0.85116 |
| GO:0046496 | nicotinamide nucleotide metabolic proces.. | 49 | 1 | 1.87 | 0.85116 |
| GO:0051881 | regulation of mitochondrial membrane pot. | 49 | 1 | 1.87 | 0.85116 |

| | | | | | |
|---|---|---|---|---|---|
| GO:0060395 | SMAD protein signal transduction | 49 | 1 | 1.87 | 0.85116 |
| GO:0031279 | regulation of cyclase activity | 88 | 2 | 3.35 | 0.85349 |
| GO:0051209 | release of sequestered calcium ion into ... | 88 | 2 | 3.35 | 0.85349 |
| GO:0051283 | negative regulation of sequestering of c... | 88 | 2 | 3.35 | 0.85349 |
| GO:0070997 | neuron death | 254 | 7 | 9.67 | 0.85491 |
| GO:0000910 | cytokinesis | 124 | 3 | 4.72 | 0.85605 |
| GO:0043112 | receptor metabolic process | 124 | 3 | 4.72 | 0.85605 |
| GO:0043124 | negative regulation of I-kappaB kinase/N... | 50 | 1 | 1.9 | 0.85684 |
| GO:0072523 | purine-containing compound catabolic pro. | 50 | 1 | 1.9 | 0.85684 |
| GO:0006836 | neurotransmitter transport | 191 | 5 | 7.27 | 0.85712 |
| GO:0006413 | translational initiation | 255 | 7 | 9.71 | 0.85759 |
| GO:0043473 | pigmentation | 89 | 2 | 3.39 | 0.85785 |
| GO:0051282 | regulation of sequestering of calcium io... | 89 | 2 | 3.39 | 0.85785 |
| GO:0051339 | regulation of lyase activity | 89 | 2 | 3.39 | 0.85785 |
| GO:1901214 | regulation of neuron death | 224 | 6 | 8.53 | 0.85946 |
| GO:2000377 | regulation of reactive oxygen species me.. | 125 | 3 | 4.76 | 0.85974 |
| GO:0007051 | spindle organization | 90 | 2 | 3.43 | 0.86209 |
| GO:0071482 | cellular response to light stimulus | 90 | 2 | 3.43 | 0.86209 |
| GO:0006081 | cellular aldehyde metabolic process | 51 | 1 | 1.94 | 0.86231 |
| GO:0030239 | myofibril assembly | 51 | 1 | 1.94 | 0.86231 |
| GO:0055013 | cardiac muscle cell development | 51 | 1 | 1.94 | 0.86231 |
| GO:0051591 | response to cAMP | 91 | 2 | 3.46 | 0.86621 |
| GO:1900542 | regulation of purine nucleotide metaboli... | 161 | 4 | 6.13 | 0.86637 |
| GO:0030509 | BMP signaling pathway | 127 | 3 | 4.83 | 0.86688 |
| GO:0032637 | interleukin-8 production | 52 | 1 | 1.98 | 0.86757 |
| GO:0033619 | membrane protein proteolysis | 52 | 1 | 1.98 | 0.86757 |
| GO:0045454 | cell redox homeostasis | 52 | 1 | 1.98 | 0.86757 |
| GO:0046889 | positive regulation of lipid biosyntheti... | 52 | 1 | 1.98 | 0.86757 |
| GO:0009405 | pathogenesis | 259 | 7 | 9.86 | 0.86794 |
| GO:0051208 | sequestering of calcium ion | 92 | 2 | 3.5 | 0.87022 |
| GO:0030802 | regulation of cyclic nucleotide biosynth... | 128 | 3 | 4.87 | 0.87033 |
| GO:0071219 | cellular response to molecule of bacteri... | 128 | 3 | 4.87 | 0.87033 |
| GO:0070588 | calcium ion transmembrane transport | 163 | 4 | 6.2 | 0.87253 |
| GO:0030858 | positive regulation of epithelial cell d... | 53 | 1 | 2.02 | 0.87263 |
| GO:0043299 | leukocyte degranulation | 53 | 1 | 2.02 | 0.87263 |
| GO:0055006 | cardiac cell development | 53 | 1 | 2.02 | 0.87263 |
| GO:0060425 | lung morphogenesis | 53 | 1 | 2.02 | 0.87263 |
| GO:0072676 | lymphocyte migration | 53 | 1 | 2.02 | 0.87263 |
| GO:0002440 | production of molecular mediator of immu. | 129 | 3 | 4.91 | 0.8737 |
| GO:0031032 | actomyosin structure organization | 129 | 3 | 4.91 | 0.8737 |
| GO:1903146 | regulation of mitochondrion degradation | 129 | 3 | 4.91 | 0.8737 |
| GO:0006906 | vesicle fusion | 93 | 2 | 3.54 | 0.87412 |
| GO:0016571 | histone methylation | 93 | 2 | 3.54 | 0.87412 |
| GO:0046717 | acid secretion | 93 | 2 | 3.54 | 0.87412 |
| GO:0006414 | translational elongation | 197 | 5 | 7.5 | 0.87448 |
| GO:0044106 | cellular amine metabolic process | 198 | 5 | 7.54 | 0.87719 |
| GO:0002562 | somatic diversification of immune recept... | 54 | 1 | 2.06 | 0.87749 |
| GO:0010717 | regulation of epithelial to mesenchymal ... | 54 | 1 | 2.06 | 0.87749 |
| GO:0016444 | somatic cell DNA recombination | 54 | 1 | 2.06 | 0.87749 |
| GO:0034103 | regulation of tissue remodeling | 54 | 1 | 2.06 | 0.87749 |
| GO:0042490 | mechanoreceptor differentiation | 54 | 1 | 2.06 | 0.87749 |
| GO:0046164 | alcohol catabolic process | 54 | 1 | 2.06 | 0.87749 |
| GO:0097006 | regulation of plasma lipoprotein particl... | 54 | 1 | 2.06 | 0.87749 |

| | | | | | |
|---|---|---|---|---|---|
| GO:1903747 | regulation of establishment of protein l... | 94 | 2 | 3.58 | 0.87791 |
| GO:0006140 | regulation of nucleotide metabolic proce... | 165 | 4 | 6.28 | 0.87844 |
| GO:0006163 | purine nucleotide metabolic process | 389 | 11 | 14.81 | 0.87998 |
| GO:0002040 | sprouting angiogenesis | 55 | 1 | 2.09 | 0.88217 |
| GO:0007052 | mitotic spindle organization | 55 | 1 | 2.09 | 0.88217 |
| GO:0021515 | cell differentiation in spinal cord | 55 | 1 | 2.09 | 0.88217 |
| GO:0030166 | proteoglycan biosynthetic process | 55 | 1 | 2.09 | 0.88217 |
| GO:0032835 | glomerulus development | 55 | 1 | 2.09 | 0.88217 |
| GO:0051225 | spindle assembly | 55 | 1 | 2.09 | 0.88217 |
| GO:0030808 | regulation of nucleotide biosynthetic pr... | 132 | 3 | 5.02 | 0.88335 |
| GO:1900371 | regulation of purine nucleotide biosynth... | 132 | 3 | 5.02 | 0.88335 |
| GO:2000736 | regulation of stem cell differentiation | 96 | 2 | 3.65 | 0.88517 |
| GO:0048732 | gland development | 392 | 11 | 14.92 | 0.88569 |
| GO:0001706 | endoderm formation | 56 | 1 | 2.13 | 0.88668 |
| GO:0007098 | centrosome cycle | 56 | 1 | 2.13 | 0.88668 |
| GO:0019915 | lipid storage | 56 | 1 | 2.13 | 0.88668 |
| GO:0032465 | regulation of cytokinesis | 56 | 1 | 2.13 | 0.88668 |
| GO:0051289 | protein homotetramerization | 56 | 1 | 2.13 | 0.88668 |
| GO:0072524 | pyridine-containing compound metabolic p | 56 | 1 | 2.13 | 0.88668 |
| GO:0006805 | xenobiotic metabolic process | 168 | 4 | 6.4 | 0.88687 |
| GO:0016064 | immunoglobulin mediated immune respons | 97 | 2 | 3.69 | 0.88866 |
| GO:0046916 | cellular transition metal ion homeostasi... | 97 | 2 | 3.69 | 0.88866 |
| GO:0035270 | endocrine system development | 134 | 3 | 5.1 | 0.88941 |
| GO:0043901 | negative regulation of multi-organism pr... | 134 | 3 | 5.1 | 0.88941 |
| GO:0071466 | cellular response to xenobiotic stimulus | 169 | 4 | 6.43 | 0.88956 |
| GO:0001895 | retina homeostasis | 57 | 1 | 2.17 | 0.89101 |
| GO:0045740 | positive regulation of DNA replication | 57 | 1 | 2.17 | 0.89101 |
| GO:0048278 | vesicle docking | 57 | 1 | 2.17 | 0.89101 |
| GO:0060393 | regulation of pathway-restricted SMAD pr.. | 57 | 1 | 2.17 | 0.89101 |
| GO:0050864 | regulation of B cell activation | 98 | 2 | 3.73 | 0.89204 |
| GO:0051017 | actin filament bundle assembly | 98 | 2 | 3.73 | 0.89204 |
| GO:0061572 | actin filament bundle organization | 98 | 2 | 3.73 | 0.89204 |
| GO:0090174 | organelle membrane fusion | 98 | 2 | 3.73 | 0.89204 |
| GO:0002250 | adaptive immune response | 237 | 6 | 9.02 | 0.8922 |
| GO:0051047 | positive regulation of secretion | 303 | 8 | 11.53 | 0.89512 |
| GO:0000186 | activation of MAPKK activity | 58 | 1 | 2.21 | 0.89517 |
| GO:0002275 | myeloid cell activation involved in immu... | 58 | 1 | 2.21 | 0.89517 |
| GO:0006406 | mRNA export from nucleus | 58 | 1 | 2.21 | 0.89517 |
| GO:0009988 | cell-cell recognition | 58 | 1 | 2.21 | 0.89517 |
| GO:0032092 | positive regulation of protein binding | 58 | 1 | 2.21 | 0.89517 |
| GO:0050909 | sensory perception of taste | 58 | 1 | 2.21 | 0.89517 |
| GO:0034754 | cellular hormone metabolic process | 99 | 2 | 3.77 | 0.89533 |
| GO:0030522 | intracellular receptor signaling pathway | 271 | 7 | 10.32 | 0.89534 |
| GO:0051346 | negative regulation of hydrolase activit... | 335 | 9 | 12.75 | 0.89565 |
| GO:0046651 | lymphocyte proliferation | 239 | 6 | 9.1 | 0.89662 |
| GO:0009308 | amine metabolic process | 206 | 5 | 7.84 | 0.89715 |
| GO:0051607 | defense response to virus | 206 | 5 | 7.84 | 0.89715 |
| GO:0072659 | protein localization to plasma membrane | 172 | 4 | 6.55 | 0.89731 |
| GO:0032845 | negative regulation of homeostatic proce... | 137 | 3 | 5.22 | 0.89798 |
| GO:1903827 | regulation of cellular protein localizat... | 399 | 11 | 15.19 | 0.89816 |
| GO:0019724 | B cell mediated immunity | 100 | 2 | 3.81 | 0.89852 |
| GO:0031056 | regulation of histone modification | 100 | 2 | 3.81 | 0.89852 |
| GO:0043271 | negative regulation of ion transport | 100 | 2 | 3.81 | 0.89852 |

| | | | | | |
|---|---|---|---|---|---|
| GO:0002200 | somatic diversification of immune recept... | 59 | 1 | 2.25 | 0.89918 |
| GO:0033013 | tetrapyrrole metabolic process | 59 | 1 | 2.25 | 0.89918 |
| GO:0042108 | positive regulation of cytokine biosynth... | 59 | 1 | 2.25 | 0.89918 |
| GO:0048645 | organ formation | 59 | 1 | 2.25 | 0.89918 |
| GO:0070542 | response to fatty acid | 59 | 1 | 2.25 | 0.89918 |
| GO:0043414 | macromolecule methylation | 207 | 5 | 7.88 | 0.89944 |
| GO:0032943 | mononuclear cell proliferation | 241 | 6 | 9.17 | 0.90087 |
| GO:0046824 | positive regulation of nucleocytoplasmic... | 101 | 2 | 3.84 | 0.90163 |
| GO:0009410 | response to xenobiotic stimulus | 174 | 4 | 6.62 | 0.90221 |
| GO:0007202 | activation of phospholipase C activity | 60 | 1 | 2.28 | 0.90303 |
| GO:0046634 | regulation of alpha-beta T cell activati... | 60 | 1 | 2.28 | 0.90303 |
| GO:0051279 | regulation of release of sequestered cal... | 60 | 1 | 2.28 | 0.90303 |
| GO:0051952 | regulation of amine transport | 60 | 1 | 2.28 | 0.90303 |
| GO:0060389 | pathway-restricted SMAD protein phospho | 60 | 1 | 2.28 | 0.90303 |
| GO:0002274 | myeloid leukocyte activation | 139 | 3 | 5.29 | 0.90335 |
| GO:1903651 | positive regulation of cytoplasmic trans... | 209 | 5 | 7.96 | 0.90388 |
| GO:0051238 | sequestering of metal ion | 102 | 2 | 3.88 | 0.90464 |
| GO:0006521 | regulation of cellular amino acid metabo... | 61 | 1 | 2.32 | 0.90674 |
| GO:0006733 | oxidoreduction coenzyme metabolic proce | 61 | 1 | 2.32 | 0.90674 |
| GO:0019226 | transmission of nerve impulse | 61 | 1 | 2.32 | 0.90674 |
| GO:0031281 | positive regulation of cyclase activity | 61 | 1 | 2.32 | 0.90674 |
| GO:0032088 | negative regulation of NF-kappaB transcr.. | 61 | 1 | 2.32 | 0.90674 |
| GO:0051349 | positive regulation of lyase activity | 61 | 1 | 2.32 | 0.90674 |
| GO:0060627 | regulation of vesicle-mediated transport | 342 | 9 | 13.02 | 0.90809 |
| GO:0017157 | regulation of exocytosis | 141 | 3 | 5.37 | 0.90847 |
| GO:0072073 | kidney epithelium development | 141 | 3 | 5.37 | 0.90847 |
| GO:0050871 | positive regulation of B cell activation | 62 | 1 | 2.36 | 0.9103 |
| GO:0002822 | regulation of adaptive immune response b. | 104 | 2 | 3.96 | 0.91041 |
| GO:0051222 | positive regulation of protein transport | 344 | 9 | 13.09 | 0.91141 |
| GO:0009615 | response to virus | 280 | 7 | 10.66 | 0.91259 |
| GO:0050727 | regulation of inflammatory response | 247 | 6 | 9.4 | 0.91276 |
| GO:0000956 | nuclear-transcribed mRNA catabolic proce | 179 | 4 | 6.81 | 0.91355 |
| GO:0001707 | mesoderm formation | 63 | 1 | 2.4 | 0.91373 |
| GO:0043266 | regulation of potassium ion transport | 63 | 1 | 2.4 | 0.91373 |
| GO:0046427 | positive regulation of JAK-STAT cascade | 63 | 1 | 2.4 | 0.91373 |
| GO:0051235 | maintenance of location | 281 | 7 | 10.7 | 0.91435 |
| GO:0071216 | cellular response to biotic stimulus | 144 | 3 | 5.48 | 0.91569 |
| GO:0000041 | transition metal ion transport | 106 | 2 | 4.03 | 0.91586 |
| GO:0042440 | pigment metabolic process | 64 | 1 | 2.44 | 0.91703 |
| GO:0042632 | cholesterol homeostasis | 64 | 1 | 2.44 | 0.91703 |
| GO:0001889 | liver development | 107 | 2 | 4.07 | 0.91846 |
| GO:0006814 | sodium ion transport | 182 | 4 | 6.93 | 0.91979 |
| GO:0070661 | leukocyte proliferation | 251 | 6 | 9.55 | 0.91998 |
| GO:0002028 | regulation of sodium ion transport | 65 | 1 | 2.47 | 0.9202 |
| GO:0009112 | nucleobase metabolic process | 65 | 1 | 2.47 | 0.9202 |
| GO:0055092 | sterol homeostasis | 65 | 1 | 2.47 | 0.9202 |
| GO:0002831 | regulation of response to biotic stimulu... | 108 | 2 | 4.11 | 0.92099 |
| GO:0015837 | amine transport | 66 | 1 | 2.51 | 0.92325 |
| GO:0043534 | blood vessel endothelial cell migration | 66 | 1 | 2.51 | 0.92325 |
| GO:0045921 | positive regulation of exocytosis | 66 | 1 | 2.51 | 0.92325 |
| GO:0061008 | hepaticobiliary system development | 109 | 2 | 4.15 | 0.92345 |
| GO:0009584 | detection of visible light | 110 | 2 | 4.19 | 0.92583 |
| GO:0032507 | maintenance of protein location in cell | 110 | 2 | 4.19 | 0.92583 |

| | | | | | |
|---|---|---|---|---|---|
| GO:0017148 | negative regulation of translation | 67 | 1 | 2.55 | 0.92619 |
| GO:0045744 | negative regulation of G-protein coupled... | 67 | 1 | 2.55 | 0.92619 |
| GO:0048332 | mesoderm morphogenesis | 67 | 1 | 2.55 | 0.92619 |
| GO:1904019 | epithelial cell apoptotic process | 67 | 1 | 2.55 | 0.92619 |
| GO:0009190 | cyclic nucleotide biosynthetic process | 149 | 3 | 5.67 | 0.92658 |
| GO:0052652 | cyclic purine nucleotide metabolic proce... | 149 | 3 | 5.67 | 0.92658 |
| GO:0001704 | formation of primary germ layer | 111 | 2 | 4.23 | 0.92814 |
| GO:0019722 | calcium-mediated signaling | 111 | 2 | 4.23 | 0.92814 |
| GO:0035725 | sodium ion transmembrane transport | 111 | 2 | 4.23 | 0.92814 |
| GO:0010863 | positive regulation of phospholipase C a... | 68 | 1 | 2.59 | 0.92901 |
| GO:0022406 | membrane docking | 68 | 1 | 2.59 | 0.92901 |
| GO:1901616 | organic hydroxy compound catabolic proce | 68 | 1 | 2.59 | 0.92901 |
| GO:1903308 | regulation of chromatin modification | 112 | 2 | 4.26 | 0.93039 |
| GO:0090316 | positive regulation of intracellular pro... | 188 | 4 | 7.16 | 0.93106 |
| GO:0006405 | RNA export from nucleus | 69 | 1 | 2.63 | 0.93173 |
| GO:0031109 | microtubule polymerization or depolymeri... | 69 | 1 | 2.63 | 0.93173 |
| GO:0002819 | regulation of adaptive immune response | 113 | 2 | 4.3 | 0.93256 |
| GO:0030101 | natural killer cell activation | 70 | 1 | 2.66 | 0.93434 |
| GO:0045445 | myoblast differentiation | 70 | 1 | 2.66 | 0.93434 |
| GO:1900274 | regulation of phospholipase C activity | 70 | 1 | 2.66 | 0.93434 |
| GO:0000184 | nuclear-transcribed mRNA catabolic proce | 115 | 2 | 4.38 | 0.93673 |
| GO:0007498 | mesoderm development | 115 | 2 | 4.38 | 0.93673 |
| GO:0002824 | positive regulation of adaptive immune r... | 71 | 1 | 2.7 | 0.93685 |
| GO:0030510 | regulation of BMP signaling pathway | 71 | 1 | 2.7 | 0.93685 |
| GO:0048489 | synaptic vesicle transport | 116 | 2 | 4.42 | 0.93872 |
| GO:0097480 | establishment of synaptic vesicle locali... | 116 | 2 | 4.42 | 0.93872 |
| GO:0030518 | intracellular steroid hormone receptor s... | 117 | 2 | 4.45 | 0.94065 |
| GO:0071804 | cellular potassium ion transport | 157 | 3 | 5.98 | 0.94134 |
| GO:0071805 | potassium ion transmembrane transport | 157 | 3 | 5.98 | 0.94134 |
| GO:0002286 | T cell activation involved in immune res... | 73 | 1 | 2.78 | 0.94159 |
| GO:0048145 | regulation of fibroblast proliferation | 73 | 1 | 2.78 | 0.94159 |
| GO:0043433 | negative regulation of sequence-specific... | 118 | 2 | 4.49 | 0.94252 |
| GO:0002821 | positive regulation of adaptive immune r... | 74 | 1 | 2.82 | 0.94382 |
| GO:0006446 | regulation of translational initiation | 74 | 1 | 2.82 | 0.94382 |
| GO:0046632 | alpha-beta T cell differentiation | 75 | 1 | 2.85 | 0.94597 |
| GO:0051651 | maintenance of location in cell | 120 | 2 | 4.57 | 0.9461 |
| GO:1903707 | negative regulation of hemopoiesis | 121 | 2 | 4.61 | 0.94781 |
| GO:0010522 | regulation of calcium ion transport into... | 76 | 1 | 2.89 | 0.94804 |
| GO:0030819 | positive regulation of cAMP biosynthetic... | 76 | 1 | 2.89 | 0.94804 |
| GO:0048144 | fibroblast proliferation | 76 | 1 | 2.89 | 0.94804 |
| GO:1902106 | negative regulation of leukocyte differe... | 76 | 1 | 2.89 | 0.94804 |
| GO:0002460 | adaptive immune response based on soma | 199 | 4 | 7.58 | 0.94807 |
| GO:0097193 | intrinsic apoptotic signaling pathway | 272 | 6 | 10.35 | 0.94992 |
| GO:0006835 | dicarboxylic acid transport | 77 | 1 | 2.93 | 0.95003 |
| GO:0007369 | gastrulation | 163 | 3 | 6.2 | 0.95056 |
| GO:0007492 | endoderm development | 78 | 1 | 2.97 | 0.95194 |
| GO:0009583 | detection of light stimulus | 124 | 2 | 4.72 | 0.95263 |
| GO:0050688 | regulation of defense response to virus | 79 | 1 | 3.01 | 0.95378 |
| GO:0002377 | immunoglobulin production | 80 | 1 | 3.05 | 0.95555 |
| GO:0033238 | regulation of cellular amine metabolic p... | 81 | 1 | 3.08 | 0.95725 |
| GO:0097164 | ammonium ion metabolic process | 169 | 3 | 6.43 | 0.95841 |
| GO:0001523 | retinoid metabolic process | 82 | 1 | 3.12 | 0.95889 |
| GO:0010518 | positive regulation of phospholipase act... | 82 | 1 | 3.12 | 0.95889 |

| | | | | | |
|---|---|---|---|---|---|
| GO:0032675 | regulation of interleukin-6 production | 82 | 1 | 3.12 | 0.95889 |
| GO:0030816 | positive regulation of cAMP metabolic pr... | 84 | 1 | 3.2 | 0.96197 |
| GO:0006417 | regulation of translation | 250 | 5 | 9.52 | 0.96393 |
| GO:0030324 | lung development | 174 | 3 | 6.62 | 0.96405 |
| GO:0043900 | regulation of multi-organism process | 322 | 7 | 12.26 | 0.96446 |
| GO:1903829 | positive regulation of cellular protein ... | 251 | 5 | 9.55 | 0.96482 |
| GO:0042307 | positive regulation of protein import in... | 86 | 1 | 3.27 | 0.96483 |
| GO:2000241 | regulation of reproductive process | 86 | 1 | 3.27 | 0.96483 |
| GO:0016101 | diterpenoid metabolic process | 88 | 1 | 3.35 | 0.96747 |
| GO:0030323 | respiratory tube development | 178 | 3 | 6.78 | 0.96803 |
| GO:0009791 | post-embryonic development | 91 | 1 | 3.46 | 0.97107 |
| GO:0021510 | spinal cord development | 91 | 1 | 3.46 | 0.97107 |
| GO:0050729 | positive regulation of inflammatory resp... | 91 | 1 | 3.46 | 0.97107 |
| GO:0010517 | regulation of phospholipase activity | 92 | 1 | 3.5 | 0.97218 |
| GO:0042035 | regulation of cytokine biosynthetic proc... | 92 | 1 | 3.5 | 0.97218 |
| GO:0048469 | cell maturation | 141 | 2 | 5.37 | 0.97286 |
| GO:0030804 | positive regulation of cyclic nucleotide... | 93 | 1 | 3.54 | 0.97324 |
| GO:0060193 | positive regulation of lipase activity | 93 | 1 | 3.54 | 0.97324 |
| GO:0090100 | positive regulation of transmembrane rec.. | 93 | 1 | 3.54 | 0.97324 |
| GO:1900182 | positive regulation of protein localizat... | 94 | 1 | 3.58 | 0.97427 |
| GO:0007603 | phototransduction | visible light | 96 | 1 | 3.65 |
| GO:0030810 | positive regulation of nucleotide biosyn... | 96 | 1 | 3.65 | 0.9762 |
| GO:1900373 | positive regulation of purine nucleotide... | 96 | 1 | 3.65 | 0.9762 |
| GO:0006721 | terpenoid metabolic process | 97 | 1 | 3.69 | 0.97711 |
| GO:0061326 | renal tubule development | 97 | 1 | 3.69 | 0.97711 |
| GO:0046631 | alpha-beta T cell activation | 99 | 1 | 3.77 | 0.97883 |
| GO:0070507 | regulation of microtubule cytoskeleton o... | 99 | 1 | 3.77 | 0.97883 |
| GO:0006813 | potassium ion transport | 193 | 3 | 7.35 | 0.97955 |
| GO:0008630 | intrinsic apoptotic signaling pathway in... | 101 | 1 | 3.84 | 0.98043 |
| GO:0051099 | positive regulation of binding | 101 | 1 | 3.84 | 0.98043 |
| GO:0032388 | positive regulation of intracellular tra... | 276 | 5 | 10.51 | 0.9815 |
| GO:1901565 | organonitrogen compound catabolic proce | 314 | 6 | 11.95 | 0.98168 |
| GO:0030801 | positive regulation of cyclic nucleotide... | 103 | 1 | 3.92 | 0.9819 |
| GO:0042089 | cytokine biosynthetic process | 103 | 1 | 3.92 | 0.9819 |
| GO:0051291 | protein heterooligomerization | 103 | 1 | 3.92 | 0.9819 |
| GO:0050804 | regulation of synaptic transmission | 240 | 4 | 9.14 | 0.98295 |
| GO:0006304 | DNA modification | 106 | 1 | 4.03 | 0.9839 |
| GO:0006024 | glycosaminoglycan biosynthetic process | 107 | 1 | 4.07 | 0.98452 |
| GO:0042107 | cytokine metabolic process | 107 | 1 | 4.07 | 0.98452 |
| GO:0045981 | positive regulation of nucleotide metabo... | 107 | 1 | 4.07 | 0.98452 |
| GO:1900544 | positive regulation of purine nucleotide... | 107 | 1 | 4.07 | 0.98452 |
| GO:0006023 | aminoglycan biosynthetic process | 108 | 1 | 4.11 | 0.98511 |
| GO:0060191 | regulation of lipase activity | 109 | 1 | 4.15 | 0.98568 |
| GO:0007602 | phototransduction | 113 | 1 | 4.3 | 0.98776 |
| GO:1901136 | carbohydrate derivative catabolic proces... | 166 | 2 | 6.32 | 0.98827 |
| GO:0006720 | isoprenoid metabolic process | 115 | 1 | 4.38 | 0.98868 |
| GO:0051262 | protein tetramerization | 116 | 1 | 4.42 | 0.98911 |
| GO:0032886 | regulation of microtubule-based process | 117 | 1 | 4.45 | 0.98953 |
| GO:0009581 | detection of external stimulus | 172 | 2 | 6.55 | 0.99044 |
| GO:0009582 | detection of abiotic stimulus | 175 | 2 | 6.66 | 0.99137 |
| GO:0048167 | regulation of synaptic plasticity | 122 | 1 | 4.64 | 0.99139 |
| GO:0002285 | lymphocyte activation involved in immune. | 126 | 1 | 4.8 | 0.99264 |
| GO:0002366 | leukocyte activation involved in immune ... | 181 | 2 | 6.89 | 0.99297 |

| | | | | | |
|---|---|---|---|---|---|
| GO:0002263 | cell activation involved in immune respo... | 182 | 2 | 6.93 | 0.99321 |
| GO:0072006 | nephron development | 133 | 1 | 5.06 | 0.9944 |
| GO:0098542 | defense response to other organism | 383 | 6 | 14.58 | 0.997 |
| GO:0007608 | sensory perception of smell | 388 | 2 | 14.77 | 1 |
| GO:0050911 | detection of chemical stimulus involved ... | 362 | 1 | 13.78 | 1 |
| GO:0050907 | detection of chemical stimulus involved ... | 399 | 1 | 15.19 | 1 |
| GO:0000012 | single strand break repair | 7 | 0 | 0.27 | 1 |
| GO:0000022 | mitotic spindle elongation | 7 | 0 | 0.27 | 1 |
| GO:0000023 | maltose metabolic process | 1 | 0 | 0.04 | 1 |
| GO:0000027 | ribosomal large subunit assembly | 4 | 0 | 0.15 | 1 |
| GO:0000028 | ribosomal small subunit assembly | 10 | 0 | 0.38 | 1 |
| GO:0000046 | autophagic vacuole fusion | 6 | 0 | 0.23 | 1 |
| GO:0000050 | urea cycle | 12 | 0 | 0.46 | 1 |
| GO:0000052 | citrulline metabolic process | 13 | 0 | 0.49 | 1 |
| GO:0000053 | argininosuccinate metabolic process | 1 | 0 | 0.04 | 1 |
| GO:0000059 | protein import into nucleus | docking | 2 | 0 | 0.08 |
| GO:0000060 | protein import into nucleus | translocati.. | 39 | 0 | 1.48 |
| GO:0000066 | mitochondrial ornithine transport | 1 | 0 | 0.04 | 1 |
| GO:0000083 | regulation of transcription involved in ... | 22 | 0 | 0.84 | 1 |
| GO:0000098 | sulfur amino acid catabolic process | 13 | 0 | 0.49 | 1 |
| GO:0000101 | sulfur amino acid transport | 6 | 0 | 0.23 | 1 |
| GO:0000103 | sulfate assimilation | 8 | 0 | 0.3 | 1 |
| GO:0000105 | histidine biosynthetic process | 2 | 0 | 0.08 | 1 |
| GO:0000117 | regulation of transcription involved in ... | 1 | 0 | 0.04 | 1 |
| GO:0000132 | establishment of mitotic spindle orienta... | 18 | 0 | 0.69 | 1 |
| GO:0000173 | inactivation of MAPK activity involved i... | 1 | 0 | 0.04 | 1 |
| GO:0000185 | activation of MAPKKK activity | 8 | 0 | 0.3 | 1 |
| GO:0000189 | MAPK import into nucleus | 2 | 0 | 0.08 | 1 |
| GO:0000212 | meiotic spindle organization | 7 | 0 | 0.27 | 1 |
| GO:0000255 | allantoin metabolic process | 4 | 0 | 0.15 | 1 |
| GO:0000256 | allantoin catabolic process | 1 | 0 | 0.04 | 1 |
| GO:0000270 | peptidoglycan metabolic process | 7 | 0 | 0.27 | 1 |
| GO:0000290 | deadenylation-dependent decapping of nu | 7 | 0 | 0.27 | 1 |
| GO:0000296 | spermine transport | 1 | 0 | 0.04 | 1 |
| GO:0000303 | response to superoxide | 22 | 0 | 0.84 | 1 |
| GO:0000305 | response to oxygen radical | 23 | 0 | 0.88 | 1 |
| GO:0000338 | protein deneddylation | 12 | 0 | 0.46 | 1 |
| GO:0000349 | generation of catalytic spliceosome for ... | 1 | 0 | 0.04 | 1 |
| GO:0000354 | cis assembly of pre-catalytic spliceosom... | 2 | 0 | 0.08 | 1 |
| GO:0000379 | tRNA-type intron splice site recognition... | 2 | 0 | 0.08 | 1 |
| GO:0000389 | mRNA 3'-splice site recognition | 5 | 0 | 0.19 | 1 |
| GO:0000390 | spliceosomal complex disassembly | 1 | 0 | 0.04 | 1 |
| GO:0000393 | spliceosomal conformational changes to g | 1 | 0 | 0.04 | 1 |
| GO:0000394 | RNA splicing | via endonu | 9 | 0 | 0.34 |
| GO:0000395 | mRNA 5'-splice site recognition | 2 | 0 | 0.08 | 1 |
| GO:0000414 | regulation of histone H3-K36 methylation | 3 | 0 | 0.11 | 1 |
| GO:0000415 | negative regulation of histone H3-K36 me. | 1 | 0 | 0.04 | 1 |
| GO:0000416 | positive regulation of histone H3-K36 me... | 1 | 0 | 0.04 | 1 |
| GO:0000423 | macromitophagy | 1 | 0 | 0.04 | 1 |
| GO:0000430 | regulation of transcription from RNA pol... | 3 | 0 | 0.11 | 1 |
| GO:0000432 | positive regulation of transcription fro... | 3 | 0 | 0.11 | 1 |
| GO:0000433 | negative regulation of transcription fro... | 1 | 0 | 0.04 | 1 |
| GO:0000437 | carbon catabolite repression of transcri... | 1 | 0 | 0.04 | 1 |

| | | | | | | |
|---|---|---|---|---|---|---|
| GO:0000447 | endonucleolytic cleavage in ITS1 to sepa.. | | 4 | 0 | 0.15 | 1 |
| GO:0000448 | cleavage in ITS2 between 5.8S rRNA and | | 2 | 0 | 0.08 | 1 |
| GO:0000451 | rRNA 2'-O-methylation | | 1 | 0 | 0.04 | 1 |
| GO:0000453 | enzyme-directed rRNA 2'-O-methylation | | 1 | 0 | 0.04 | 1 |
| GO:0000459 | exonucleolytic trimming involved in rRNA.. | | 4 | 0 | 0.15 | 1 |
| GO:0000460 | maturation of 5.8S rRNA | | 18 | 0 | 0.69 | 1 |
| GO:0000461 | endonucleolytic cleavage to generate mat. | | 2 | 0 | 0.08 | 1 |
| GO:0000463 | maturation of LSU-rRNA from tricistronic... | | 5 | 0 | 0.19 | 1 |
| GO:0000466 | maturation of 5.8S rRNA from tricistroni... | | 12 | 0 | 0.46 | 1 |
| GO:0000467 | exonucleolytic trimming to generate matu.. | | 4 | 0 | 0.15 | 1 |
| GO:0000469 | cleavage involved in rRNA processing | | 11 | 0 | 0.42 | 1 |
| GO:0000470 | maturation of LSU-rRNA | | 6 | 0 | 0.23 | 1 |
| GO:0000472 | endonucleolytic cleavage to generate mat. | | 2 | 0 | 0.08 | 1 |
| GO:0000478 | endonucleolytic cleavage involved in rRN.. | | 7 | 0 | 0.27 | 1 |
| GO:0000479 | endonucleolytic cleavage of tricistronic... | | 7 | 0 | 0.27 | 1 |
| GO:0000480 | endonucleolytic cleavage in 5'-ETS of tr... | | 3 | 0 | 0.11 | 1 |
| GO:0000481 | maturation of 5S rRNA | | 1 | 0 | 0.04 | 1 |
| GO:0000491 | small nucleolar ribonucleoprotein comple.. | | 3 | 0 | 0.11 | 1 |
| GO:0000492 | box C/D snoRNP assembly | | 3 | 0 | 0.11 | 1 |
| GO:0000494 | box C/D snoRNA 3'-end processing | | 1 | 0 | 0.04 | 1 |
| GO:0000495 | box H/ACA snoRNA 3'-end processing | | 1 | 0 | 0.04 | 1 |
| GO:0000710 | meiotic mismatch repair | | 3 | 0 | 0.11 | 1 |
| GO:0000711 | meiotic DNA repair synthesis | | 3 | 0 | 0.11 | 1 |
| GO:0000712 | resolution of meiotic recombination inte... | | 9 | 0 | 0.34 | 1 |
| GO:0000715 | nucleotide-excision repair | DNA damag | | 2 | 0 | 0.08 |
| GO:0000717 | nucleotide-excision repair | DNA duplex | | 1 | 0 | 0.04 |
| GO:0000718 | nucleotide-excision repair | DNA damag | | 21 | 0 | 0.8 |
| GO:0000720 | pyrimidine dimer repair by nucleotide-ex... | | 3 | 0 | 0.11 | 1 |
| GO:0000727 | double-strand break repair via break-ind... | | 1 | 0 | 0.04 | 1 |
| GO:0000729 | DNA double-strand break processing | | 8 | 0 | 0.3 | 1 |
| GO:0000730 | DNA recombinase assembly | | 5 | 0 | 0.19 | 1 |
| GO:0000731 | DNA synthesis involved in DNA repair | | 12 | 0 | 0.46 | 1 |
| GO:0000733 | DNA strand renaturation | | 9 | 0 | 0.34 | 1 |
| GO:0000738 | DNA catabolic process | exonucleoly | | 13 | 0 | 0.49 |
| GO:0000820 | regulation of glutamine family amino aci... | | 3 | 0 | 0.11 | 1 |
| GO:0000821 | regulation of arginine metabolic process | | 1 | 0 | 0.04 | 1 |
| GO:0000912 | assembly of actomyosin apparatus involve | | 1 | 0 | 0.04 | 1 |
| GO:0000915 | actomyosin contractile ring assembly | | 1 | 0 | 0.04 | 1 |
| GO:0000917 | barrier septum assembly | | 2 | 0 | 0.08 | 1 |
| GO:0000920 | cell separation after cytokinesis | | 16 | 0 | 0.61 | 1 |
| GO:0000921 | septin ring assembly | | 1 | 0 | 0.04 | 1 |
| GO:0000961 | negative regulation of mitochondrial RNA.. | | 1 | 0 | 0.04 | 1 |
| GO:0000967 | rRNA 5'-end processing | | 2 | 0 | 0.08 | 1 |
| GO:0000972 | transcription-dependent tethering of RNA.. | | 1 | 0 | 0.04 | 1 |
| GO:0001079 | nitrogen catabolite regulation of transc... | | 1 | 0 | 0.04 | 1 |
| GO:0001080 | nitrogen catabolite activation of transc... | | 1 | 0 | 0.04 | 1 |
| GO:0001172 | transcription | RNA-templa | | 2 | 0 | 0.08 |
| GO:0001180 | transcription initiation from RNA polyme... | | 2 | 0 | 0.08 | 1 |
| GO:0001188 | RNA polymerase I transcriptional preinit... | | 2 | 0 | 0.08 | 1 |
| GO:0001189 | RNA polymerase I transcriptional preinit... | | 2 | 0 | 0.08 | 1 |
| GO:0001192 | maintenance of transcriptional fidelity ... | | 1 | 0 | 0.04 | 1 |
| GO:0001193 | maintenance of transcriptional fidelity ... | | 1 | 0 | 0.04 | 1 |
| GO:0001207 | histone displacement | | 1 | 0 | 0.04 | 1 |

| | | | | | |
|---|---|---|---|---|---|
| GO:0001300 | chronological cell aging | 2 | 0 | 0.08 | 1 |
| GO:0001302 | replicative cell aging | 6 | 0 | 0.23 | 1 |
| GO:0001306 | age-dependent response to oxidative stre. | 3 | 0 | 0.11 | 1 |
| GO:0001315 | age-dependent response to reactive oxyge | 1 | 0 | 0.04 | 1 |
| GO:0001504 | neurotransmitter uptake | 25 | 0 | 0.95 | 1 |
| GO:0001507 | acetylcholine catabolic process in synap... | 2 | 0 | 0.08 | 1 |
| GO:0001514 | selenocysteine incorporation | 7 | 0 | 0.27 | 1 |
| GO:0001519 | peptide amidation | 2 | 0 | 0.08 | 1 |
| GO:0001539 | cilium or flagellum-dependent cell motil... | 13 | 0 | 0.49 | 1 |
| GO:0001543 | ovarian follicle rupture | 2 | 0 | 0.08 | 1 |
| GO:0001544 | initiation of primordial ovarian follicl... | 1 | 0 | 0.04 | 1 |
| GO:0001545 | primary ovarian follicle growth | 1 | 0 | 0.04 | 1 |
| GO:0001546 | preantral ovarian follicle growth | 2 | 0 | 0.08 | 1 |
| GO:0001547 | antral ovarian follicle growth | 6 | 0 | 0.23 | 1 |
| GO:0001550 | ovarian cumulus expansion | 2 | 0 | 0.08 | 1 |
| GO:0001552 | ovarian follicle atresia | 1 | 0 | 0.04 | 1 |
| GO:0001553 | luteinization | 11 | 0 | 0.42 | 1 |
| GO:0001555 | oocyte growth | 1 | 0 | 0.04 | 1 |
| GO:0001556 | oocyte maturation | 21 | 0 | 0.8 | 1 |
| GO:0001560 | regulation of cell growth by extracellul... | 3 | 0 | 0.11 | 1 |
| GO:0001561 | fatty acid alpha-oxidation | 6 | 0 | 0.23 | 1 |
| GO:0001562 | response to protozoan | 20 | 0 | 0.76 | 1 |
| GO:0001575 | globoside metabolic process | 2 | 0 | 0.08 | 1 |
| GO:0001576 | globoside biosynthetic process | 1 | 0 | 0.04 | 1 |
| GO:0001579 | medium-chain fatty acid transport | 2 | 0 | 0.08 | 1 |
| GO:0001580 | detection of chemical stimulus involved ... | 30 | 0 | 1.14 | 1 |
| GO:0001581 | detection of chemical stimulus involved ... | 2 | 0 | 0.08 | 1 |
| GO:0001582 | detection of chemical stimulus involved ... | 2 | 0 | 0.08 | 1 |
| GO:0001658 | branching involved in ureteric bud morph... | 61 | 0 | 2.32 | 1 |
| GO:0001660 | fever generation | 10 | 0 | 0.38 | 1 |
| GO:0001661 | conditioned taste aversion | 5 | 0 | 0.19 | 1 |
| GO:0001662 | behavioral fear response | 30 | 0 | 1.14 | 1 |
| GO:0001675 | acrosome assembly | 14 | 0 | 0.53 | 1 |
| GO:0001680 | tRNA 3'-terminal CCA addition | 1 | 0 | 0.04 | 1 |
| GO:0001682 | tRNA 5'-leader removal | 1 | 0 | 0.04 | 1 |
| GO:0001692 | histamine metabolic process | 3 | 0 | 0.11 | 1 |
| GO:0001694 | histamine biosynthetic process | 2 | 0 | 0.08 | 1 |
| GO:0001696 | gastric acid secretion | 12 | 0 | 0.46 | 1 |
| GO:0001697 | histamine-induced gastric acid secretion | 1 | 0 | 0.04 | 1 |
| GO:0001698 | gastrin-induced gastric acid secretion | 1 | 0 | 0.04 | 1 |
| GO:0001705 | ectoderm formation | 3 | 0 | 0.11 | 1 |
| GO:0001709 | cell fate determination | 42 | 0 | 1.6 | 1 |
| GO:0001710 | mesodermal cell fate commitment | 19 | 0 | 0.72 | 1 |
| GO:0001712 | ectodermal cell fate commitment | 1 | 0 | 0.04 | 1 |
| GO:0001715 | ectodermal cell fate specification | 1 | 0 | 0.04 | 1 |
| GO:0001731 | formation of translation preinitiation c... | 17 | 0 | 0.65 | 1 |
| GO:0001732 | formation of cytoplasmic translation ini... | 2 | 0 | 0.08 | 1 |
| GO:0001743 | optic placode formation | 3 | 0 | 0.11 | 1 |
| GO:0001757 | somite specification | 5 | 0 | 0.19 | 1 |
| GO:0001759 | organ induction | 22 | 0 | 0.84 | 1 |
| GO:0001766 | membrane raft polarization | 4 | 0 | 0.15 | 1 |
| GO:0001767 | establishment of lymphocyte polarity | 7 | 0 | 0.27 | 1 |
| GO:0001768 | establishment of T cell polarity | 6 | 0 | 0.23 | 1 |

| | | | | | |
|---|---|---|---|---|---|
| GO:0001770 | establishment of natural killer cell pol... | 1 | 0 | 0.04 | 1 |
| GO:0001771 | immunological synapse formation | 8 | 0 | 0.3 | 1 |
| GO:0001773 | myeloid dendritic cell activation | 27 | 0 | 1.03 | 1 |
| GO:0001774 | microglial cell activation | 12 | 0 | 0.46 | 1 |
| GO:0001777 | T cell homeostatic proliferation | 3 | 0 | 0.11 | 1 |
| GO:0001778 | plasma membrane repair | 7 | 0 | 0.27 | 1 |
| GO:0001780 | neutrophil homeostasis | 5 | 0 | 0.19 | 1 |
| GO:0001781 | neutrophil apoptotic process | 3 | 0 | 0.11 | 1 |
| GO:0001787 | natural killer cell proliferation | 8 | 0 | 0.3 | 1 |
| GO:0001794 | type IIa hypersensitivity | 2 | 0 | 0.08 | 1 |
| GO:0001796 | regulation of type IIa hypersensitivity | 2 | 0 | 0.08 | 1 |
| GO:0001798 | positive regulation of type IIa hypersen... | 2 | 0 | 0.08 | 1 |
| GO:0001802 | type III hypersensitivity | 1 | 0 | 0.04 | 1 |
| GO:0001803 | regulation of type III hypersensitivity | 1 | 0 | 0.04 | 1 |
| GO:0001805 | positive regulation of type III hypersen... | 1 | 0 | 0.04 | 1 |
| GO:0001809 | positive regulation of type IV hypersens... | 1 | 0 | 0.04 | 1 |
| GO:0001810 | regulation of type I hypersensitivity | 2 | 0 | 0.08 | 1 |
| GO:0001812 | positive regulation of type I hypersensi... | 2 | 0 | 0.08 | 1 |
| GO:0001820 | serotonin secretion | 10 | 0 | 0.38 | 1 |
| GO:0001826 | inner cell mass cell differentiation | 5 | 0 | 0.19 | 1 |
| GO:0001827 | inner cell mass cell fate commitment | 1 | 0 | 0.04 | 1 |
| GO:0001828 | inner cell mass cellular morphogenesis | 1 | 0 | 0.04 | 1 |
| GO:0001831 | trophectodermal cellular morphogenesis | 3 | 0 | 0.11 | 1 |
| GO:0001833 | inner cell mass cell proliferation | 14 | 0 | 0.53 | 1 |
| GO:0001834 | trophectodermal cell proliferation | 1 | 0 | 0.04 | 1 |
| GO:0001835 | blastocyst hatching | 4 | 0 | 0.15 | 1 |
| GO:0001839 | neural plate morphogenesis | 8 | 0 | 0.3 | 1 |
| GO:0001840 | neural plate development | 11 | 0 | 0.42 | 1 |
| GO:0001844 | protein insertion into mitochondrial mem... | 28 | 0 | 1.07 | 1 |
| GO:0001845 | phagolysosome assembly | 8 | 0 | 0.3 | 1 |
| GO:0001866 | NK T cell proliferation | 5 | 0 | 0.19 | 1 |
| GO:0001867 | complement activation | lectin pathw | 9 | 0 | 0.34 |
| GO:0001868 | regulation of complement activation | lec... | 2 | 0 | 0.08 |
| GO:0001869 | negative regulation of complement activa.. | 2 | 0 | 0.08 | 1 |
| GO:0001878 | response to yeast | 5 | 0 | 0.19 | 1 |
| GO:0001880 | Mullerian duct regression | 5 | 0 | 0.19 | 1 |
| GO:0001886 | endothelial cell morphogenesis | 10 | 0 | 0.38 | 1 |
| GO:0001887 | selenium compound metabolic process | 2 | 0 | 0.08 | 1 |
| GO:0001896 | autolysis | 1 | 0 | 0.04 | 1 |
| GO:0001897 | cytolysis by symbiont of host cells | 2 | 0 | 0.08 | 1 |
| GO:0001905 | activation of membrane attack complex | 3 | 0 | 0.11 | 1 |
| GO:0001907 | killing by symbiont of host cells | 4 | 0 | 0.15 | 1 |
| GO:0001913 | T cell mediated cytotoxicity | 35 | 0 | 1.33 | 1 |
| GO:0001914 | regulation of T cell mediated cytotoxici... | 27 | 0 | 1.03 | 1 |
| GO:0001915 | negative regulation of T cell mediated c... | 4 | 0 | 0.15 | 1 |
| GO:0001916 | positive regulation of T cell mediated c... | 22 | 0 | 0.84 | 1 |
| GO:0001919 | regulation of receptor recycling | 16 | 0 | 0.61 | 1 |
| GO:0001920 | negative regulation of receptor recyclin... | 3 | 0 | 0.11 | 1 |
| GO:0001921 | positive regulation of receptor recyclin... | 10 | 0 | 0.38 | 1 |
| GO:0001922 | B-1 B cell homeostasis | 4 | 0 | 0.15 | 1 |
| GO:0001927 | exocyst assembly | 2 | 0 | 0.08 | 1 |
| GO:0001928 | regulation of exocyst assembly | 1 | 0 | 0.04 | 1 |
| GO:0001949 | sebaceous gland cell differentiation | 1 | 0 | 0.04 | 1 |

| | | | | | |
|---|---|---|---|---|---|
| GO:0001956 | positive regulation of neurotransmitter ... | 10 | 0 | 0.38 | 1 |
| GO:0001957 | intramembranous ossification | 6 | 0 | 0.23 | 1 |
| GO:0001969 | regulation of activation of membrane att... | 3 | 0 | 0.11 | 1 |
| GO:0001970 | positive regulation of activation of mem... | 2 | 0 | 0.08 | 1 |
| GO:0001971 | negative regulation of activation of mem... | 1 | 0 | 0.04 | 1 |
| GO:0001973 | adenosine receptor signaling pathway | 12 | 0 | 0.46 | 1 |
| GO:0001975 | response to amphetamine | 29 | 0 | 1.1 | 1 |
| GO:0001976 | neurological system process involved in ... | 15 | 0 | 0.57 | 1 |
| GO:0001977 | renal system process involved in regulat... | 17 | 0 | 0.65 | 1 |
| GO:0001978 | regulation of systemic arterial blood pr... | 4 | 0 | 0.15 | 1 |
| GO:0001979 | regulation of systemic arterial blood pr... | 1 | 0 | 0.04 | 1 |
| GO:0001980 | regulation of systemic arterial blood pr... | 1 | 0 | 0.04 | 1 |
| GO:0001982 | baroreceptor response to decreased syste | 1 | 0 | 0.04 | 1 |
| GO:0001983 | baroreceptor response to increased syste. | 3 | 0 | 0.11 | 1 |
| GO:0001984 | vasodilation of artery involved in baror... | 1 | 0 | 0.04 | 1 |
| GO:0001985 | negative regulation of heart rate involv... | 1 | 0 | 0.04 | 1 |
| GO:0001986 | negative regulation of the force of hear... | 1 | 0 | 0.04 | 1 |
| GO:0001987 | vasoconstriction of artery involved in b... | 1 | 0 | 0.04 | 1 |
| GO:0001992 | regulation of systemic arterial blood pr... | 5 | 0 | 0.19 | 1 |
| GO:0001994 | norepinephrine-epinephrine vasoconstrict.. | 3 | 0 | 0.11 | 1 |
| GO:0001998 | angiotensin mediated vasoconstriction in... | 1 | 0 | 0.04 | 1 |
| GO:0002002 | regulation of angiotensin levels in bloo... | 13 | 0 | 0.49 | 1 |
| GO:0002003 | angiotensin maturation | 12 | 0 | 0.46 | 1 |
| GO:0002005 | angiotensin catabolic process in blood | 3 | 0 | 0.11 | 1 |
| GO:0002007 | detection of hypoxic conditions in blood... | 1 | 0 | 0.04 | 1 |
| GO:0002016 | regulation of blood volume by renin-angi... | 10 | 0 | 0.38 | 1 |
| GO:0002017 | regulation of blood volume by renal aldo... | 2 | 0 | 0.08 | 1 |
| GO:0002018 | renin-angiotensin regulation of aldoster... | 4 | 0 | 0.15 | 1 |
| GO:0002019 | regulation of renal output by angiotensi... | 3 | 0 | 0.11 | 1 |
| GO:0002029 | desensitization of G-protein coupled rec... | 15 | 0 | 0.57 | 1 |
| GO:0002030 | inhibitory G-protein coupled receptor ph... | 1 | 0 | 0.04 | 1 |
| GO:0002031 | G-protein coupled receptor internalizati... | 10 | 0 | 0.38 | 1 |
| GO:0002032 | desensitization of G-protein coupled rec... | 2 | 0 | 0.08 | 1 |
| GO:0002033 | vasodilation by angiotensin involved in ... | 1 | 0 | 0.04 | 1 |
| GO:0002034 | regulation of blood vessel size by renin... | 4 | 0 | 0.15 | 1 |
| GO:0002035 | brain renin-angiotensin system | 5 | 0 | 0.19 | 1 |
| GO:0002036 | regulation of L-glutamate transport | 2 | 0 | 0.08 | 1 |
| GO:0002037 | negative regulation of L-glutamate trans... | 1 | 0 | 0.04 | 1 |
| GO:0002041 | intussusceptive angiogenesis | 2 | 0 | 0.08 | 1 |
| GO:0002042 | cell migration involved in sprouting ang... | 30 | 0 | 1.14 | 1 |
| GO:0002043 | blood vessel endothelial cell proliferat... | 9 | 0 | 0.34 | 1 |
| GO:0002044 | blood vessel endothelial cell migration ... | 1 | 0 | 0.04 | 1 |
| GO:0002051 | osteoblast fate commitment | 4 | 0 | 0.15 | 1 |
| GO:0002052 | positive regulation of neuroblast prolif... | 19 | 0 | 0.72 | 1 |
| GO:0002069 | columnar/cuboidal epithelial cell matura... | 7 | 0 | 0.27 | 1 |
| GO:0002070 | epithelial cell maturation | 17 | 0 | 0.65 | 1 |
| GO:0002071 | glandular epithelial cell maturation | 3 | 0 | 0.11 | 1 |
| GO:0002072 | optic cup morphogenesis involved in came | 8 | 0 | 0.3 | 1 |
| GO:0002074 | extraocular skeletal muscle development | 3 | 0 | 0.11 | 1 |
| GO:0002077 | acrosome matrix dispersal | 1 | 0 | 0.04 | 1 |
| GO:0002082 | regulation of oxidative phosphorylation | 9 | 0 | 0.34 | 1 |
| GO:0002084 | protein depalmitoylation | 3 | 0 | 0.11 | 1 |
| GO:0002085 | inhibition of neuroepithelial cell diffe... | 3 | 0 | 0.11 | 1 |

| | | | | | |
|---|---|---|---|---|---|
| GO:0002086 | diaphragm contraction | 2 | 0 | 0.08 | 1 |
| GO:0002087 | regulation of respiratory gaseous exchan... | 13 | 0 | 0.49 | 1 |
| GO:0002090 | regulation of receptor internalization | 29 | 0 | 1.1 | 1 |
| GO:0002091 | negative regulation of receptor internal... | 6 | 0 | 0.23 | 1 |
| GO:0002092 | positive regulation of receptor internal... | 19 | 0 | 0.72 | 1 |
| GO:0002093 | auditory receptor cell morphogenesis | 11 | 0 | 0.42 | 1 |
| GO:0002097 | tRNA wobble base modification | 7 | 0 | 0.27 | 1 |
| GO:0002098 | tRNA wobble uridine modification | 6 | 0 | 0.23 | 1 |
| GO:0002100 | tRNA wobble adenosine to inosine editing | 1 | 0 | 0.04 | 1 |
| GO:0002115 | store-operated calcium entry | 7 | 0 | 0.27 | 1 |
| GO:0002118 | aggressive behavior | 10 | 0 | 0.38 | 1 |
| GO:0002121 | inter-male aggressive behavior | 2 | 0 | 0.08 | 1 |
| GO:0002125 | maternal aggressive behavior | 4 | 0 | 0.15 | 1 |
| GO:0002128 | tRNA nucleoside ribose methylation | 1 | 0 | 0.04 | 1 |
| GO:0002143 | tRNA wobble position uridine thiolation | 1 | 0 | 0.04 | 1 |
| GO:0002148 | hypochlorous acid metabolic process | 1 | 0 | 0.04 | 1 |
| GO:0002149 | hypochlorous acid biosynthetic process | 1 | 0 | 0.04 | 1 |
| GO:0002152 | bile acid conjugation | 1 | 0 | 0.04 | 1 |
| GO:0002154 | thyroid hormone mediated signaling pathw | 4 | 0 | 0.15 | 1 |
| GO:0002155 | regulation of thyroid hormone mediated s... | 3 | 0 | 0.11 | 1 |
| GO:0002158 | osteoclast proliferation | 5 | 0 | 0.19 | 1 |
| GO:0002159 | desmosome assembly | 4 | 0 | 0.15 | 1 |
| GO:0002175 | protein localization to paranode region ... | 4 | 0 | 0.15 | 1 |
| GO:0002176 | male germ cell proliferation | 5 | 0 | 0.19 | 1 |
| GO:0002181 | cytoplasmic translation | 19 | 0 | 0.72 | 1 |
| GO:0002182 | cytoplasmic translational elongation | 2 | 0 | 0.08 | 1 |
| GO:0002183 | cytoplasmic translational initiation | 8 | 0 | 0.3 | 1 |
| GO:0002190 | cap-independent translational initiation | 5 | 0 | 0.19 | 1 |
| GO:0002191 | cap-dependent translational initiation | 1 | 0 | 0.04 | 1 |
| GO:0002192 | IRES-dependent translational initiation | 4 | 0 | 0.15 | 1 |
| GO:0002194 | hepatocyte cell migration | 1 | 0 | 0.04 | 1 |
| GO:0002204 | somatic recombination of immunoglobulin | 38 | 0 | 1.45 | 1 |
| GO:0002208 | somatic diversification of immunoglobuli... | 38 | 0 | 1.45 | 1 |
| GO:0002209 | behavioral defense response | 30 | 0 | 1.14 | 1 |
| GO:0002215 | defense response to nematode | 3 | 0 | 0.11 | 1 |
| GO:0002220 | innate immune response activating cell s... | 3 | 0 | 0.11 | 1 |
| GO:0002225 | positive regulation of antimicrobial pep... | 2 | 0 | 0.08 | 1 |
| GO:0002227 | innate immune response in mucosa | 22 | 0 | 0.84 | 1 |
| GO:0002230 | positive regulation of defense response ... | 17 | 0 | 0.65 | 1 |
| GO:0002232 | leukocyte chemotaxis involved in inflamm... | 2 | 0 | 0.08 | 1 |
| GO:0002238 | response to molecule of fungal origin | 3 | 0 | 0.11 | 1 |
| GO:0002246 | wound healing involved in inflammatory r... | 6 | 0 | 0.23 | 1 |
| GO:0002248 | connective tissue replacement involved i... | 4 | 0 | 0.15 | 1 |
| GO:0002249 | lymphocyte anergy | 7 | 0 | 0.27 | 1 |
| GO:0002251 | organ or tissue specific immune response | 33 | 0 | 1.26 | 1 |
| GO:0002254 | kinin cascade | 3 | 0 | 0.11 | 1 |
| GO:0002266 | follicular dendritic cell activation | 2 | 0 | 0.08 | 1 |
| GO:0002268 | follicular dendritic cell differentiatio... | 2 | 0 | 0.08 | 1 |
| GO:0002276 | basophil activation involved in immune r... | 1 | 0 | 0.04 | 1 |
| GO:0002277 | myeloid dendritic cell activation involv... | 2 | 0 | 0.08 | 1 |
| GO:0002278 | eosinophil activation involved in immune... | 6 | 0 | 0.23 | 1 |
| GO:0002281 | macrophage activation involved in immune | 8 | 0 | 0.3 | 1 |
| GO:0002282 | microglial cell activation involved in i... | 2 | 0 | 0.08 | 1 |

| | | | | | |
|---|---|---|---|---|---|
| GO:0002283 | neutrophil activation involved in immune... | 11 | 0 | 0.42 | 1 |
| GO:0002287 | alpha-beta T cell activation involved in... | 40 | 0 | 1.52 | 1 |
| GO:0002290 | gamma-delta T cell activation involved i... | 1 | 0 | 0.04 | 1 |
| GO:0002291 | T cell activation via T cell receptor co... | 5 | 0 | 0.19 | 1 |
| GO:0002293 | alpha-beta T cell differentiation involv... | 40 | 0 | 1.52 | 1 |
| GO:0002294 | CD4-positive | alpha-beta | 38 | 0 | 1.45 |
| GO:0002295 | T-helper cell lineage commitment | 10 | 0 | 0.38 | 1 |
| GO:0002296 | T-helper 1 cell lineage commitment | 2 | 0 | 0.08 | 1 |
| GO:0002302 | CD8-positive | alpha-beta | 2 | 0 | 0.08 |
| GO:0002309 | T cell proliferation involved in immune ... | 6 | 0 | 0.23 | 1 |
| GO:0002312 | B cell activation involved in immune res... | 54 | 0 | 2.06 | 1 |
| GO:0002313 | mature B cell differentiation involved i... | 10 | 0 | 0.38 | 1 |
| GO:0002314 | germinal center B cell differentiation | 2 | 0 | 0.08 | 1 |
| GO:0002315 | marginal zone B cell differentiation | 2 | 0 | 0.08 | 1 |
| GO:0002316 | follicular B cell differentiation | 1 | 0 | 0.04 | 1 |
| GO:0002317 | plasma cell differentiation | 4 | 0 | 0.15 | 1 |
| GO:0002318 | myeloid progenitor cell differentiation | 7 | 0 | 0.27 | 1 |
| GO:0002320 | lymphoid progenitor cell differentiation | 18 | 0 | 0.69 | 1 |
| GO:0002322 | B cell proliferation involved in immune ... | 5 | 0 | 0.19 | 1 |
| GO:0002323 | natural killer cell activation involved ... | 27 | 0 | 1.03 | 1 |
| GO:0002325 | natural killer cell differentiation invo... | 4 | 0 | 0.15 | 1 |
| GO:0002326 | B cell lineage commitment | 5 | 0 | 0.19 | 1 |
| GO:0002327 | immature B cell differentiation | 8 | 0 | 0.3 | 1 |
| GO:0002328 | pro-B cell differentiation | 10 | 0 | 0.38 | 1 |
| GO:0002329 | pre-B cell differentiation | 5 | 0 | 0.19 | 1 |
| GO:0002331 | pre-B cell allelic exclusion | 3 | 0 | 0.11 | 1 |
| GO:0002332 | transitional stage B cell differentiatio... | 1 | 0 | 0.04 | 1 |
| GO:0002333 | transitional one stage B cell differenti... | 1 | 0 | 0.04 | 1 |
| GO:0002337 | B-1a B cell differentiation | 2 | 0 | 0.08 | 1 |
| GO:0002339 | B cell selection | 2 | 0 | 0.08 | 1 |
| GO:0002349 | histamine production involved in inflamm... | 8 | 0 | 0.3 | 1 |
| GO:0002351 | serotonin production involved in inflamm... | 3 | 0 | 0.11 | 1 |
| GO:0002352 | B cell negative selection | 2 | 0 | 0.08 | 1 |
| GO:0002353 | plasma kallikrein-kinin cascade | 3 | 0 | 0.11 | 1 |
| GO:0002357 | defense response to tumor cell | 1 | 0 | 0.04 | 1 |
| GO:0002358 | B cell homeostatic proliferation | 2 | 0 | 0.08 | 1 |
| GO:0002360 | T cell lineage commitment | 17 | 0 | 0.65 | 1 |
| GO:0002361 | CD4-positive | CD25-posit | alpha-beta .. | 3 | 0 |
| GO:0002362 | CD4-positive | CD25-posit | alpha-beta .. | 1 | 0 |
| GO:0002363 | alpha-beta T cell lineage commitment | 12 | 0 | 0.46 | 1 |
| GO:0002368 | B cell cytokine production | 2 | 0 | 0.08 | 1 |
| GO:0002370 | natural killer cell cytokine production | 1 | 0 | 0.04 | 1 |
| GO:0002371 | dendritic cell cytokine production | 4 | 0 | 0.15 | 1 |
| GO:0002373 | plasmacytoid dendritic cell cytokine pro... | 1 | 0 | 0.04 | 1 |
| GO:0002374 | cytokine secretion involved in immune re... | 14 | 0 | 0.53 | 1 |
| GO:0002378 | immunoglobulin biosynthetic process | 2 | 0 | 0.08 | 1 |
| GO:0002380 | immunoglobulin secretion involved in imm. | 1 | 0 | 0.04 | 1 |
| GO:0002381 | immunoglobulin production involved in im.. | 48 | 0 | 1.83 | 1 |
| GO:0002384 | hepatic immune response | 2 | 0 | 0.08 | 1 |
| GO:0002385 | mucosal immune response | 31 | 0 | 1.18 | 1 |
| GO:0002386 | immune response in mucosal-associated l | 3 | 0 | 0.11 | 1 |
| GO:0002396 | MHC protein complex assembly | 6 | 0 | 0.23 | 1 |
| GO:0002397 | MHC class I protein complex assembly | 1 | 0 | 0.04 | 1 |

| | | | | | |
|---|---|---|---|---|---|
| GO:0002399 | MHC class II protein complex assembly | 5 | 0 | 0.19 | 1 |
| GO:0002404 | antigen sampling in mucosal-associated l.. | 1 | 0 | 0.04 | 1 |
| GO:0002406 | antigen sampling by M cells in mucosal-a.. | 1 | 0 | 0.04 | 1 |
| GO:0002408 | myeloid dendritic cell chemotaxis | 4 | 0 | 0.15 | 1 |
| GO:0002412 | antigen transcytosis by M cells in mucos... | 1 | 0 | 0.04 | 1 |
| GO:0002414 | immunoglobulin transcytosis in epithelia... | 4 | 0 | 0.15 | 1 |
| GO:0002415 | immunoglobulin transcytosis in epithelia... | 2 | 0 | 0.08 | 1 |
| GO:0002416 | IgG immunoglobulin transcytosis in epith... | 1 | 0 | 0.04 | 1 |
| GO:0002424 | T cell mediated immune response to tumor | 1 | 0 | 0.04 | 1 |
| GO:0002426 | immunoglobulin production in mucosal tis.. | 3 | 0 | 0.11 | 1 |
| GO:0002428 | antigen processing and presentation of p... | 4 | 0 | 0.15 | 1 |
| GO:0002430 | complement receptor mediated signaling p | 5 | 0 | 0.19 | 1 |
| GO:0002432 | granuloma formation | 1 | 0 | 0.04 | 1 |
| GO:0002439 | chronic inflammatory response to antigen.. | 5 | 0 | 0.19 | 1 |
| GO:0002441 | histamine secretion involved in inflamma... | 8 | 0 | 0.3 | 1 |
| GO:0002442 | serotonin secretion involved in inflamma... | 3 | 0 | 0.11 | 1 |
| GO:0002445 | type II hypersensitivity | 2 | 0 | 0.08 | 1 |
| GO:0002446 | neutrophil mediated immunity | 24 | 0 | 0.91 | 1 |
| GO:0002447 | eosinophil mediated immunity | 6 | 0 | 0.23 | 1 |
| GO:0002457 | T cell antigen processing and presentati... | 3 | 0 | 0.11 | 1 |
| GO:0002458 | peripheral T cell tolerance induction | 1 | 0 | 0.04 | 1 |
| GO:0002461 | tolerance induction dependent upon immu | 1 | 0 | 0.04 | 1 |
| GO:0002465 | peripheral tolerance induction | 1 | 0 | 0.04 | 1 |
| GO:0002467 | germinal center formation | 15 | 0 | 0.57 | 1 |
| GO:0002468 | dendritic cell antigen processing and pr... | 8 | 0 | 0.3 | 1 |
| GO:0002476 | antigen processing and presentation of e... | 3 | 0 | 0.11 | 1 |
| GO:0002477 | antigen processing and presentation of e... | 3 | 0 | 0.11 | 1 |
| GO:0002481 | antigen processing and presentation of e... | 3 | 0 | 0.11 | 1 |
| GO:0002483 | antigen processing and presentation of e... | 10 | 0 | 0.38 | 1 |
| GO:0002484 | antigen processing and presentation of e... | 5 | 0 | 0.19 | 1 |
| GO:0002485 | antigen processing and presentation of e... | 2 | 0 | 0.08 | 1 |
| GO:0002486 | antigen processing and presentation of e... | 3 | 0 | 0.11 | 1 |
| GO:0002488 | antigen processing and presentation of e... | 2 | 0 | 0.08 | 1 |
| GO:0002489 | antigen processing and presentation of e... | 2 | 0 | 0.08 | 1 |
| GO:0002501 | peptide antigen assembly with MHC protei | 6 | 0 | 0.23 | 1 |
| GO:0002502 | peptide antigen assembly with MHC class | 1 | 0 | 0.04 | 1 |
| GO:0002503 | peptide antigen assembly with MHC class | 5 | 0 | 0.19 | 1 |
| GO:0002505 | antigen processing and presentation of p... | 2 | 0 | 0.08 | 1 |
| GO:0002506 | polysaccharide assembly with MHC class I | 2 | 0 | 0.08 | 1 |
| GO:0002507 | tolerance induction | 18 | 0 | 0.69 | 1 |
| GO:0002508 | central tolerance induction | 1 | 0 | 0.04 | 1 |
| GO:0002510 | central B cell tolerance induction | 1 | 0 | 0.04 | 1 |
| GO:0002513 | tolerance induction to self antigen | 4 | 0 | 0.15 | 1 |
| GO:0002514 | B cell tolerance induction | 2 | 0 | 0.08 | 1 |
| GO:0002517 | T cell tolerance induction | 12 | 0 | 0.46 | 1 |
| GO:0002518 | lymphocyte chemotaxis across high endotl | 1 | 0 | 0.04 | 1 |
| GO:0002522 | leukocyte migration involved in immune r... | 2 | 0 | 0.08 | 1 |
| GO:0002525 | acute inflammatory response to non-antig. | 3 | 0 | 0.11 | 1 |
| GO:0002528 | regulation of vascular permeability invo... | 2 | 0 | 0.08 | 1 |
| GO:0002532 | production of molecular mediator involve... | 35 | 0 | 1.33 | 1 |
| GO:0002534 | cytokine production involved in inflamma... | 14 | 0 | 0.53 | 1 |
| GO:0002536 | respiratory burst involved in inflammato... | 4 | 0 | 0.15 | 1 |
| GO:0002537 | nitric oxide production involved in infl... | 2 | 0 | 0.08 | 1 |

| | | | | | |
|---|---|---|---|---|---|
| GO:0002538 | arachidonic acid metabolite production i... | 2 | 0 | 0.08 | 1 |
| GO:0002540 | leukotriene production involved in infla... | 2 | 0 | 0.08 | 1 |
| GO:0002541 | activation of plasma proteins involved i... | 3 | 0 | 0.11 | 1 |
| GO:0002542 | Factor XII activation | 2 | 0 | 0.08 | 1 |
| GO:0002543 | activation of blood coagulation via clot... | 2 | 0 | 0.08 | 1 |
| GO:0002544 | chronic inflammatory response | 22 | 0 | 0.84 | 1 |
| GO:0002545 | chronic inflammatory response to non-ant. | 1 | 0 | 0.04 | 1 |
| GO:0002551 | mast cell chemotaxis | 9 | 0 | 0.34 | 1 |
| GO:0002553 | histamine secretion by mast cell | 8 | 0 | 0.3 | 1 |
| GO:0002554 | serotonin secretion by platelet | 3 | 0 | 0.11 | 1 |
| GO:0002560 | basophil mediated immunity | 1 | 0 | 0.04 | 1 |
| GO:0002561 | basophil degranulation | 1 | 0 | 0.04 | 1 |
| GO:0002568 | somatic diversification of T cell recept... | 5 | 0 | 0.19 | 1 |
| GO:0002572 | pro-T cell differentiation | 3 | 0 | 0.11 | 1 |
| GO:0002575 | basophil chemotaxis | 1 | 0 | 0.04 | 1 |
| GO:0002577 | regulation of antigen processing and pre... | 17 | 0 | 0.65 | 1 |
| GO:0002578 | negative regulation of antigen processin... | 5 | 0 | 0.19 | 1 |
| GO:0002579 | positive regulation of antigen processin... | 11 | 0 | 0.42 | 1 |
| GO:0002580 | regulation of antigen processing and pre... | 5 | 0 | 0.19 | 1 |
| GO:0002581 | negative regulation of antigen processin... | 3 | 0 | 0.11 | 1 |
| GO:0002582 | positive regulation of antigen processin... | 2 | 0 | 0.08 | 1 |
| GO:0002583 | regulation of antigen processing and pre... | 7 | 0 | 0.27 | 1 |
| GO:0002584 | negative regulation of antigen processin... | 3 | 0 | 0.11 | 1 |
| GO:0002585 | positive regulation of antigen processin... | 4 | 0 | 0.15 | 1 |
| GO:0002586 | regulation of antigen processing and pre... | 4 | 0 | 0.15 | 1 |
| GO:0002587 | negative regulation of antigen processin... | 2 | 0 | 0.08 | 1 |
| GO:0002588 | positive regulation of antigen processin... | 2 | 0 | 0.08 | 1 |
| GO:0002589 | regulation of antigen processing and pre... | 3 | 0 | 0.11 | 1 |
| GO:0002590 | negative regulation of antigen processin... | 1 | 0 | 0.04 | 1 |
| GO:0002591 | positive regulation of antigen processin... | 2 | 0 | 0.08 | 1 |
| GO:0002604 | regulation of dendritic cell antigen pro... | 8 | 0 | 0.3 | 1 |
| GO:0002605 | negative regulation of dendritic cell an... | 1 | 0 | 0.04 | 1 |
| GO:0002606 | positive regulation of dendritic cell an... | 7 | 0 | 0.27 | 1 |
| GO:0002625 | regulation of T cell antigen processing ... | 1 | 0 | 0.04 | 1 |
| GO:0002634 | regulation of germinal center formation | 9 | 0 | 0.34 | 1 |
| GO:0002635 | negative regulation of germinal center f... | 2 | 0 | 0.08 | 1 |
| GO:0002636 | positive regulation of germinal center f... | 4 | 0 | 0.15 | 1 |
| GO:0002637 | regulation of immunoglobulin production | 40 | 0 | 1.52 | 1 |
| GO:0002638 | negative regulation of immunoglobulin pr... | 5 | 0 | 0.19 | 1 |
| GO:0002639 | positive regulation of immunoglobulin pr... | 28 | 0 | 1.07 | 1 |
| GO:0002643 | regulation of tolerance induction | 13 | 0 | 0.49 | 1 |
| GO:0002644 | negative regulation of tolerance inducti... | 1 | 0 | 0.04 | 1 |
| GO:0002645 | positive regulation of tolerance inducti... | 10 | 0 | 0.38 | 1 |
| GO:0002646 | regulation of central tolerance inductio... | 1 | 0 | 0.04 | 1 |
| GO:0002648 | positive regulation of central tolerance... | 1 | 0 | 0.04 | 1 |
| GO:0002649 | regulation of tolerance induction to sel... | 1 | 0 | 0.04 | 1 |
| GO:0002651 | positive regulation of tolerance inducti... | 1 | 0 | 0.04 | 1 |
| GO:0002652 | regulation of tolerance induction depend... | 1 | 0 | 0.04 | 1 |
| GO:0002654 | positive regulation of tolerance inducti... | 1 | 0 | 0.04 | 1 |
| GO:0002658 | regulation of peripheral tolerance induc... | 1 | 0 | 0.04 | 1 |
| GO:0002660 | positive regulation of peripheral tolera... | 1 | 0 | 0.04 | 1 |
| GO:0002661 | regulation of B cell tolerance induction | 2 | 0 | 0.08 | 1 |
| GO:0002663 | positive regulation of B cell tolerance ... | 2 | 0 | 0.08 | 1 |

| | | | | | |
|---|---|---|---|---|---|
| GO:0002664 | regulation of T cell tolerance induction | 11 | 0 | 0.42 | 1 |
| GO:0002665 | negative regulation of T cell tolerance ... | 1 | 0 | 0.04 | 1 |
| GO:0002666 | positive regulation of T cell tolerance ... | 8 | 0 | 0.3 | 1 |
| GO:0002667 | regulation of T cell anergy | 7 | 0 | 0.27 | 1 |
| GO:0002668 | negative regulation of T cell anergy | 1 | 0 | 0.04 | 1 |
| GO:0002669 | positive regulation of T cell anergy | 4 | 0 | 0.15 | 1 |
| GO:0002675 | positive regulation of acute inflammator... | 25 | 0 | 0.95 | 1 |
| GO:0002676 | regulation of chronic inflammatory respo... | 9 | 0 | 0.34 | 1 |
| GO:0002677 | negative regulation of chronic inflammat... | 4 | 0 | 0.15 | 1 |
| GO:0002678 | positive regulation of chronic inflammat... | 4 | 0 | 0.15 | 1 |
| GO:0002679 | respiratory burst involved in defense re... | 9 | 0 | 0.34 | 1 |
| GO:0002681 | somatic recombination of T cell receptor... | 5 | 0 | 0.19 | 1 |
| GO:0002692 | negative regulation of cellular extravas... | 6 | 0 | 0.23 | 1 |
| GO:0002701 | negative regulation of production of mol... | 21 | 0 | 0.8 | 1 |
| GO:0002712 | regulation of B cell mediated immunity | 40 | 0 | 1.52 | 1 |
| GO:0002713 | negative regulation of B cell mediated i... | 10 | 0 | 0.38 | 1 |
| GO:0002714 | positive regulation of B cell mediated i... | 25 | 0 | 0.95 | 1 |
| GO:0002719 | negative regulation of cytokine producti... | 18 | 0 | 0.69 | 1 |
| GO:0002721 | regulation of B cell cytokine production | 1 | 0 | 0.04 | 1 |
| GO:0002725 | negative regulation of T cell cytokine p... | 4 | 0 | 0.15 | 1 |
| GO:0002727 | regulation of natural killer cell cytoki... | 1 | 0 | 0.04 | 1 |
| GO:0002729 | positive regulation of natural killer ce... | 1 | 0 | 0.04 | 1 |
| GO:0002730 | regulation of dendritic cell cytokine pr... | 3 | 0 | 0.11 | 1 |
| GO:0002731 | negative regulation of dendritic cell cy... | 2 | 0 | 0.08 | 1 |
| GO:0002732 | positive regulation of dendritic cell cy... | 1 | 0 | 0.04 | 1 |
| GO:0002736 | regulation of plasmacytoid dendritic cel... | 1 | 0 | 0.04 | 1 |
| GO:0002737 | negative regulation of plasmacytoid dend.. | 1 | 0 | 0.04 | 1 |
| GO:0002739 | regulation of cytokine secretion involve... | 11 | 0 | 0.42 | 1 |
| GO:0002740 | negative regulation of cytokine secretio... | 6 | 0 | 0.23 | 1 |
| GO:0002741 | positive regulation of cytokine secretio... | 4 | 0 | 0.15 | 1 |
| GO:0002752 | cell surface pattern recognition recepto... | 3 | 0 | 0.11 | 1 |
| GO:0002759 | regulation of antimicrobial humoral resp... | 3 | 0 | 0.11 | 1 |
| GO:0002760 | positive regulation of antimicrobial hum... | 2 | 0 | 0.08 | 1 |
| GO:0002767 | immune response-inhibiting cell surface ... | 5 | 0 | 0.19 | 1 |
| GO:0002769 | natural killer cell inhibitory signaling... | 1 | 0 | 0.04 | 1 |
| GO:0002774 | Fc receptor mediated inhibitory signalin... | 3 | 0 | 0.11 | 1 |
| GO:0002775 | antimicrobial peptide production | 3 | 0 | 0.11 | 1 |
| GO:0002776 | antimicrobial peptide secretion | 1 | 0 | 0.04 | 1 |
| GO:0002777 | antimicrobial peptide biosynthetic proce... | 3 | 0 | 0.11 | 1 |
| GO:0002778 | antibacterial peptide production | 3 | 0 | 0.11 | 1 |
| GO:0002779 | antibacterial peptide secretion | 1 | 0 | 0.04 | 1 |
| GO:0002780 | antibacterial peptide biosynthetic proce... | 3 | 0 | 0.11 | 1 |
| GO:0002784 | regulation of antimicrobial peptide prod... | 2 | 0 | 0.08 | 1 |
| GO:0002786 | regulation of antibacterial peptide prod... | 2 | 0 | 0.08 | 1 |
| GO:0002793 | positive regulation of peptide secretion | 73 | 0 | 2.78 | 1 |
| GO:0002803 | positive regulation of antibacterial pep... | 2 | 0 | 0.08 | 1 |
| GO:0002805 | regulation of antimicrobial peptide bios... | 2 | 0 | 0.08 | 1 |
| GO:0002807 | positive regulation of antimicrobial pep... | 2 | 0 | 0.08 | 1 |
| GO:0002808 | regulation of antibacterial peptide bios... | 2 | 0 | 0.08 | 1 |
| GO:0002815 | biosynthetic process of antibacterial pe... | 2 | 0 | 0.08 | 1 |
| GO:0002816 | regulation of biosynthetic process of an... | 2 | 0 | 0.08 | 1 |
| GO:0002825 | regulation of T-helper 1 type immune res... | 20 | 0 | 0.76 | 1 |
| GO:0002826 | negative regulation of T-helper 1 type i... | 6 | 0 | 0.23 | 1 |

| | | | | | |
|---|---|---|---|---|---|
| GO:0002827 | positive regulation of T-helper 1 type i... | 14 | 0 | 0.53 | 1 |
| GO:0002828 | regulation of type 2 immune response | 22 | 0 | 0.84 | 1 |
| GO:0002829 | negative regulation of type 2 immune res... | 9 | 0 | 0.34 | 1 |
| GO:0002830 | positive regulation of type 2 immune res... | 12 | 0 | 0.46 | 1 |
| GO:0002832 | negative regulation of response to bioti... | 25 | 0 | 0.95 | 1 |
| GO:0002840 | regulation of T cell mediated immune res... | 1 | 0 | 0.04 | 1 |
| GO:0002842 | positive regulation of T cell mediated i... | 1 | 0 | 0.04 | 1 |
| GO:0002849 | regulation of peripheral T cell toleranc... | 1 | 0 | 0.04 | 1 |
| GO:0002851 | positive regulation of peripheral T cell... | 1 | 0 | 0.04 | 1 |
| GO:0002863 | positive regulation of inflammatory resp... | 9 | 0 | 0.34 | 1 |
| GO:0002866 | positive regulation of acute inflammator... | 6 | 0 | 0.23 | 1 |
| GO:0002870 | T cell anergy | 7 | 0 | 0.27 | 1 |
| GO:0002874 | regulation of chronic inflammatory respo... | 3 | 0 | 0.11 | 1 |
| GO:0002875 | negative regulation of chronic inflammat... | 1 | 0 | 0.04 | 1 |
| GO:0002876 | positive regulation of chronic inflammat... | 2 | 0 | 0.08 | 1 |
| GO:0002877 | regulation of acute inflammatory respons... | 3 | 0 | 0.11 | 1 |
| GO:0002878 | negative regulation of acute inflammator... | 1 | 0 | 0.04 | 1 |
| GO:0002879 | positive regulation of acute inflammator... | 2 | 0 | 0.08 | 1 |
| GO:0002880 | regulation of chronic inflammatory respo... | 1 | 0 | 0.04 | 1 |
| GO:0002882 | positive regulation of chronic inflammat... | 1 | 0 | 0.04 | 1 |
| GO:0002885 | positive regulation of hypersensitivity | 5 | 0 | 0.19 | 1 |
| GO:0002888 | positive regulation of myeloid leukocyte... | 16 | 0 | 0.61 | 1 |
| GO:0002889 | regulation of immunoglobulin mediated im. | 39 | 0 | 1.48 | 1 |
| GO:0002890 | negative regulation of immunoglobulin me. | 10 | 0 | 0.38 | 1 |
| GO:0002891 | positive regulation of immunoglobulin me.. | 25 | 0 | 0.95 | 1 |
| GO:0002892 | regulation of type II hypersensitivity | 2 | 0 | 0.08 | 1 |
| GO:0002894 | positive regulation of type II hypersens... | 2 | 0 | 0.08 | 1 |
| GO:0002895 | regulation of central B cell tolerance i... | 1 | 0 | 0.04 | 1 |
| GO:0002897 | positive regulation of central B cell to... | 1 | 0 | 0.04 | 1 |
| GO:0002904 | positive regulation of B cell apoptotic ... | 3 | 0 | 0.11 | 1 |
| GO:0002911 | regulation of lymphocyte anergy | 7 | 0 | 0.27 | 1 |
| GO:0002912 | negative regulation of lymphocyte anergy | 1 | 0 | 0.04 | 1 |
| GO:0002913 | positive regulation of lymphocyte anergy | 4 | 0 | 0.15 | 1 |
| GO:0002922 | positive regulation of humoral immune re... | 11 | 0 | 0.42 | 1 |
| GO:0002923 | regulation of humoral immune response m | 11 | 0 | 0.42 | 1 |
| GO:0002924 | negative regulation of humoral immune re. | 6 | 0 | 0.23 | 1 |
| GO:0002925 | positive regulation of humoral immune re... | 5 | 0 | 0.19 | 1 |
| GO:0002930 | trabecular meshwork development | 2 | 0 | 0.08 | 1 |
| GO:0002932 | tendon sheath development | 1 | 0 | 0.04 | 1 |
| GO:0002934 | desmosome organization | 9 | 0 | 0.34 | 1 |
| GO:0002943 | tRNA dihydrouridine synthesis | 3 | 0 | 0.11 | 1 |
| GO:0003010 | voluntary skeletal muscle contraction | 3 | 0 | 0.11 | 1 |
| GO:0003016 | respiratory system process | 26 | 0 | 0.99 | 1 |
| GO:0003025 | regulation of systemic arterial blood pr... | 5 | 0 | 0.19 | 1 |
| GO:0003027 | regulation of systemic arterial blood pr... | 1 | 0 | 0.04 | 1 |
| GO:0003029 | detection of hypoxic conditions in blood... | 1 | 0 | 0.04 | 1 |
| GO:0003032 | detection of oxygen | 5 | 0 | 0.19 | 1 |
| GO:0003051 | angiotensin-mediated drinking behavior | 3 | 0 | 0.11 | 1 |
| GO:0003056 | regulation of vascular smooth muscle con. | 4 | 0 | 0.15 | 1 |
| GO:0003058 | hormonal regulation of the force of hear... | 1 | 0 | 0.04 | 1 |
| GO:0003064 | regulation of heart rate by hormone | 2 | 0 | 0.08 | 1 |
| GO:0003065 | positive regulation of heart rate by epi... | 1 | 0 | 0.04 | 1 |
| GO:0003068 | regulation of systemic arterial blood pr... | 1 | 0 | 0.04 | 1 |

| | | | | | |
|---|---|---|---|---|---|
| GO:0003069 | vasodilation by acetylcholine involved i... | 1 | 0 | 0.04 | 1 |
| GO:0003070 | regulation of systemic arterial blood pr... | 1 | 0 | 0.04 | 1 |
| GO:0003072 | renal control of peripheral vascular res... | 4 | 0 | 0.15 | 1 |
| GO:0003083 | negative regulation of renal output by a... | 1 | 0 | 0.04 | 1 |
| GO:0003093 | regulation of glomerular filtration | 12 | 0 | 0.46 | 1 |
| GO:0003094 | glomerular filtration | 21 | 0 | 0.8 | 1 |
| GO:0003095 | pressure natriuresis | 3 | 0 | 0.11 | 1 |
| GO:0003096 | renal sodium ion transport | 5 | 0 | 0.19 | 1 |
| GO:0003104 | positive regulation of glomerular filtra... | 4 | 0 | 0.15 | 1 |
| GO:0003105 | negative regulation of glomerular filtra... | 6 | 0 | 0.23 | 1 |
| GO:0003106 | negative regulation of glomerular filtra... | 1 | 0 | 0.04 | 1 |
| GO:0003108 | negative regulation of the force of hear... | 1 | 0 | 0.04 | 1 |
| GO:0003116 | regulation of vasoconstriction by norepi... | 1 | 0 | 0.04 | 1 |
| GO:0003117 | regulation of vasoconstriction by circul... | 1 | 0 | 0.04 | 1 |
| GO:0003127 | detection of nodal flow | 2 | 0 | 0.08 | 1 |
| GO:0003128 | heart field specification | 12 | 0 | 0.46 | 1 |
| GO:0003129 | heart induction | 6 | 0 | 0.23 | 1 |
| GO:0003130 | BMP signaling pathway involved in heart .. | 3 | 0 | 0.11 | 1 |
| GO:0003131 | mesodermal-endodermal cell signaling | 1 | 0 | 0.04 | 1 |
| GO:0003133 | endodermal-mesodermal cell signaling | 3 | 0 | 0.11 | 1 |
| GO:0003134 | endodermal-mesodermal cell signaling inv. | 3 | 0 | 0.11 | 1 |
| GO:0003136 | negative regulation of heart induction b... | 1 | 0 | 0.04 | 1 |
| GO:0003137 | Notch signaling pathway involved in hear... | 1 | 0 | 0.04 | 1 |
| GO:0003138 | primary heart field specification | 1 | 0 | 0.04 | 1 |
| GO:0003139 | secondary heart field specification | 8 | 0 | 0.3 | 1 |
| GO:0003140 | determination of left/right asymmetry in... | 7 | 0 | 0.27 | 1 |
| GO:0003142 | cardiogenic plate morphogenesis | 1 | 0 | 0.04 | 1 |
| GO:0003144 | embryonic heart tube formation | 1 | 0 | 0.04 | 1 |
| GO:0003149 | membranous septum morphogenesis | 6 | 0 | 0.23 | 1 |
| GO:0003150 | muscular septum morphogenesis | 4 | 0 | 0.15 | 1 |
| GO:0003156 | regulation of organ formation | 35 | 0 | 1.33 | 1 |
| GO:0003159 | morphogenesis of an endothelium | 11 | 0 | 0.42 | 1 |
| GO:0003161 | cardiac conduction system development | 14 | 0 | 0.53 | 1 |
| GO:0003162 | atrioventricular node development | 3 | 0 | 0.11 | 1 |
| GO:0003163 | sinoatrial node development | 5 | 0 | 0.19 | 1 |
| GO:0003164 | His-Purkinje system development | 5 | 0 | 0.19 | 1 |
| GO:0003165 | Purkinje myocyte development | 2 | 0 | 0.08 | 1 |
| GO:0003166 | bundle of His development | 4 | 0 | 0.15 | 1 |
| GO:0003167 | atrioventricular bundle cell differentia... | 1 | 0 | 0.04 | 1 |
| GO:0003168 | Purkinje myocyte differentiation | 1 | 0 | 0.04 | 1 |
| GO:0003169 | coronary vein morphogenesis | 2 | 0 | 0.08 | 1 |
| GO:0003172 | sinoatrial valve development | 1 | 0 | 0.04 | 1 |
| GO:0003174 | mitral valve development | 7 | 0 | 0.27 | 1 |
| GO:0003175 | tricuspid valve development | 3 | 0 | 0.11 | 1 |
| GO:0003176 | aortic valve development | 5 | 0 | 0.19 | 1 |
| GO:0003180 | aortic valve morphogenesis | 5 | 0 | 0.19 | 1 |
| GO:0003183 | mitral valve morphogenesis | 6 | 0 | 0.23 | 1 |
| GO:0003185 | sinoatrial valve morphogenesis | 1 | 0 | 0.04 | 1 |
| GO:0003186 | tricuspid valve morphogenesis | 2 | 0 | 0.08 | 1 |
| GO:0003192 | mitral valve formation | 2 | 0 | 0.08 | 1 |
| GO:0003193 | pulmonary valve formation | 2 | 0 | 0.08 | 1 |
| GO:0003195 | tricuspid valve formation | 2 | 0 | 0.08 | 1 |
| GO:0003207 | cardiac chamber formation | 12 | 0 | 0.46 | 1 |

| | | | | | |
|---|---|---|---|---|---|
| GO:0003210 | cardiac atrium formation | 1 | 0 | 0.04 | 1 |
| GO:0003211 | cardiac ventricle formation | 10 | 0 | 0.38 | 1 |
| GO:0003214 | cardiac left ventricle morphogenesis | 12 | 0 | 0.46 | 1 |
| GO:0003215 | cardiac right ventricle morphogenesis | 16 | 0 | 0.61 | 1 |
| GO:0003218 | cardiac left ventricle formation | 2 | 0 | 0.08 | 1 |
| GO:0003219 | cardiac right ventricle formation | 4 | 0 | 0.15 | 1 |
| GO:0003220 | left ventricular cardiac muscle tissue m... | 1 | 0 | 0.04 | 1 |
| GO:0003221 | right ventricular cardiac muscle tissue ... | 2 | 0 | 0.08 | 1 |
| GO:0003223 | ventricular compact myocardium morphog | 2 | 0 | 0.08 | 1 |
| GO:0003228 | atrial cardiac muscle tissue development | 6 | 0 | 0.23 | 1 |
| GO:0003235 | sinus venosus development | 1 | 0 | 0.04 | 1 |
| GO:0003236 | sinus venosus morphogenesis | 1 | 0 | 0.04 | 1 |
| GO:0003241 | growth involved in heart morphogenesis | 3 | 0 | 0.11 | 1 |
| GO:0003245 | cardiac muscle tissue growth involved in... | 1 | 0 | 0.04 | 1 |
| GO:0003249 | cell proliferation involved in heart val... | 1 | 0 | 0.04 | 1 |
| GO:0003250 | regulation of cell proliferation involve... | 1 | 0 | 0.04 | 1 |
| GO:0003251 | positive regulation of cell proliferatio... | 1 | 0 | 0.04 | 1 |
| GO:0003253 | cardiac neural crest cell migration invo... | 3 | 0 | 0.11 | 1 |
| GO:0003256 | regulation of transcription from RNA pol... | 7 | 0 | 0.27 | 1 |
| GO:0003257 | positive regulation of transcription fro... | 4 | 0 | 0.15 | 1 |
| GO:0003259 | cardioblast anterior-lateral migration | 1 | 0 | 0.04 | 1 |
| GO:0003260 | cardioblast migration | 1 | 0 | 0.04 | 1 |
| GO:0003263 | cardioblast proliferation | 9 | 0 | 0.34 | 1 |
| GO:0003264 | regulation of cardioblast proliferation | 9 | 0 | 0.34 | 1 |
| GO:0003266 | regulation of secondary heart field card... | 9 | 0 | 0.34 | 1 |
| GO:0003270 | Notch signaling pathway involved in regu.. | 1 | 0 | 0.04 | 1 |
| GO:0003273 | cell migration involved in endocardial c... | 3 | 0 | 0.11 | 1 |
| GO:0003283 | atrial septum development | 17 | 0 | 0.65 | 1 |
| GO:0003284 | septum primum development | 5 | 0 | 0.19 | 1 |
| GO:0003285 | septum secundum development | 3 | 0 | 0.11 | 1 |
| GO:0003289 | atrial septum primum morphogenesis | 4 | 0 | 0.15 | 1 |
| GO:0003290 | atrial septum secundum morphogenesis | 2 | 0 | 0.08 | 1 |
| GO:0003294 | atrial ventricular junction remodeling | 2 | 0 | 0.08 | 1 |
| GO:0003310 | pancreatic A cell differentiation | 7 | 0 | 0.27 | 1 |
| GO:0003311 | pancreatic D cell differentiation | 1 | 0 | 0.04 | 1 |
| GO:0003312 | pancreatic PP cell differentiation | 2 | 0 | 0.08 | 1 |
| GO:0003318 | cell migration to the midline involved i... | 1 | 0 | 0.04 | 1 |
| GO:0003322 | pancreatic A cell development | 1 | 0 | 0.04 | 1 |
| GO:0003326 | pancreatic A cell fate commitment | 2 | 0 | 0.08 | 1 |
| GO:0003327 | type B pancreatic cell fate commitment | 1 | 0 | 0.04 | 1 |
| GO:0003329 | pancreatic PP cell fate commitment | 2 | 0 | 0.08 | 1 |
| GO:0003330 | regulation of extracellular matrix const... | 2 | 0 | 0.08 | 1 |
| GO:0003331 | positive regulation of extracellular mat... | 2 | 0 | 0.08 | 1 |
| GO:0003335 | corneocyte development | 1 | 0 | 0.04 | 1 |
| GO:0003337 | mesenchymal to epithelial transition inv... | 14 | 0 | 0.53 | 1 |
| GO:0003338 | metanephros morphogenesis | 33 | 0 | 1.26 | 1 |
| GO:0003339 | regulation of mesenchymal to epithelial ... | 8 | 0 | 0.3 | 1 |
| GO:0003340 | negative regulation of mesenchymal to ep. | 3 | 0 | 0.11 | 1 |
| GO:0003342 | proepicardium development | 2 | 0 | 0.08 | 1 |
| GO:0003343 | septum transversum development | 2 | 0 | 0.08 | 1 |
| GO:0003348 | cardiac endothelial cell differentiation | 6 | 0 | 0.23 | 1 |
| GO:0003350 | pulmonary myocardium development | 3 | 0 | 0.11 | 1 |
| GO:0003352 | regulation of cilium movement | 5 | 0 | 0.19 | 1 |

| | | | | | |
|---|---|---|---|---|---|
| GO:0003353 | positive regulation of cilium movement | 1 | 0 | 0.04 | 1 |
| GO:0003357 | noradrenergic neuron differentiation | 7 | 0 | 0.27 | 1 |
| GO:0003358 | noradrenergic neuron development | 3 | 0 | 0.11 | 1 |
| GO:0003359 | noradrenergic neuron fate commitment | 1 | 0 | 0.04 | 1 |
| GO:0003360 | brainstem development | 7 | 0 | 0.27 | 1 |
| GO:0003363 | lamellipodium assembly involved in amebo | 1 | 0 | 0.04 | 1 |
| GO:0003365 | establishment of cell polarity involved ... | 1 | 0 | 0.04 | 1 |
| GO:0003366 | cell-matrix adhesion involved in ameboid... | 2 | 0 | 0.08 | 1 |
| GO:0003373 | dynamin polymerization involved in membr | 1 | 0 | 0.04 | 1 |
| GO:0003374 | dynamin polymerization involved in mitoc... | 1 | 0 | 0.04 | 1 |
| GO:0003376 | sphingosine-1-phosphate signaling pathwa | 9 | 0 | 0.34 | 1 |
| GO:0003382 | epithelial cell morphogenesis | 49 | 0 | 1.87 | 1 |
| GO:0003383 | apical constriction | 4 | 0 | 0.15 | 1 |
| GO:0003402 | planar cell polarity pathway involved in... | 5 | 0 | 0.19 | 1 |
| GO:0003404 | optic vesicle morphogenesis | 2 | 0 | 0.08 | 1 |
| GO:0003408 | optic cup formation involved in camera-t... | 3 | 0 | 0.11 | 1 |
| GO:0003409 | optic cup structural organization | 1 | 0 | 0.04 | 1 |
| GO:0003413 | chondrocyte differentiation involved in ... | 8 | 0 | 0.3 | 1 |
| GO:0003414 | chondrocyte morphogenesis involved in er | 1 | 0 | 0.04 | 1 |
| GO:0003415 | chondrocyte hypertrophy | 4 | 0 | 0.15 | 1 |
| GO:0003418 | growth plate cartilage chondrocyte diffe... | 5 | 0 | 0.19 | 1 |
| GO:0003419 | growth plate cartilage chondrocyte proli... | 4 | 0 | 0.15 | 1 |
| GO:0003420 | regulation of growth plate cartilage cho... | 2 | 0 | 0.08 | 1 |
| GO:0003421 | growth plate cartilage axis specificatio... | 1 | 0 | 0.04 | 1 |
| GO:0003422 | growth plate cartilage morphogenesis | 2 | 0 | 0.08 | 1 |
| GO:0003429 | growth plate cartilage chondrocyte morph.. | 1 | 0 | 0.04 | 1 |
| GO:0003430 | growth plate cartilage chondrocyte growt... | 2 | 0 | 0.08 | 1 |
| GO:0003431 | growth plate cartilage chondrocyte devel... | 3 | 0 | 0.11 | 1 |
| GO:0003433 | chondrocyte development involved in endo | 5 | 0 | 0.19 | 1 |
| GO:0005513 | detection of calcium ion | 14 | 0 | 0.53 | 1 |
| GO:0005982 | starch metabolic process | 1 | 0 | 0.04 | 1 |
| GO:0005983 | starch catabolic process | 1 | 0 | 0.04 | 1 |
| GO:0005984 | disaccharide metabolic process | 5 | 0 | 0.19 | 1 |
| GO:0005985 | sucrose metabolic process | 1 | 0 | 0.04 | 1 |
| GO:0005988 | lactose metabolic process | 2 | 0 | 0.08 | 1 |
| GO:0005989 | lactose biosynthetic process | 2 | 0 | 0.08 | 1 |
| GO:0005991 | trehalose metabolic process | 1 | 0 | 0.04 | 1 |
| GO:0005993 | trehalose catabolic process | 1 | 0 | 0.04 | 1 |
| GO:0005997 | xylulose metabolic process | 4 | 0 | 0.15 | 1 |
| GO:0005998 | xylulose catabolic process | 1 | 0 | 0.04 | 1 |
| GO:0005999 | xylulose biosynthetic process | 2 | 0 | 0.08 | 1 |
| GO:0006000 | fructose metabolic process | 11 | 0 | 0.42 | 1 |
| GO:0006001 | fructose catabolic process | 2 | 0 | 0.08 | 1 |
| GO:0006002 | fructose 6-phosphate metabolic process | 6 | 0 | 0.23 | 1 |
| GO:0006003 | fructose 2 | 6-bisphospl | 5 | 0 | 0.19 |
| GO:0006007 | glucose catabolic process | 4 | 0 | 0.15 | 1 |
| GO:0006011 | UDP-glucose metabolic process | 4 | 0 | 0.15 | 1 |
| GO:0006012 | galactose metabolic process | 12 | 0 | 0.46 | 1 |
| GO:0006013 | mannose metabolic process | 7 | 0 | 0.27 | 1 |
| GO:0006014 | D-ribose metabolic process | 2 | 0 | 0.08 | 1 |
| GO:0006026 | aminoglycan catabolic process | 63 | 0 | 2.4 | 1 |
| GO:0006027 | glycosaminoglycan catabolic process | 58 | 0 | 2.21 | 1 |
| GO:0006030 | chitin metabolic process | 5 | 0 | 0.19 | 1 |

| | | | | | |
|---|---|---|---|---|---|
| GO:0006032 | chitin catabolic process | 5 | 0 | 0.19 | 1 |
| GO:0006037 | cell wall chitin metabolic process | 1 | 0 | 0.04 | 1 |
| GO:0006041 | glucosamine metabolic process | 4 | 0 | 0.15 | 1 |
| GO:0006042 | glucosamine biosynthetic process | 1 | 0 | 0.04 | 1 |
| GO:0006043 | glucosamine catabolic process | 1 | 0 | 0.04 | 1 |
| GO:0006044 | N-acetylglucosamine metabolic process | 16 | 0 | 0.61 | 1 |
| GO:0006045 | N-acetylglucosamine biosynthetic process | 2 | 0 | 0.08 | 1 |
| GO:0006049 | UDP-N-acetylglucosamine catabolic proce | 1 | 0 | 0.04 | 1 |
| GO:0006050 | mannosamine metabolic process | 2 | 0 | 0.08 | 1 |
| GO:0006051 | N-acetylmannosamine metabolic process | 2 | 0 | 0.08 | 1 |
| GO:0006059 | hexitol metabolic process | 2 | 0 | 0.08 | 1 |
| GO:0006060 | sorbitol metabolic process | 1 | 0 | 0.04 | 1 |
| GO:0006062 | sorbitol catabolic process | 1 | 0 | 0.04 | 1 |
| GO:0006064 | glucuronate catabolic process | 1 | 0 | 0.04 | 1 |
| GO:0006065 | UDP-glucuronate biosynthetic process | 3 | 0 | 0.11 | 1 |
| GO:0006067 | ethanol metabolic process | 14 | 0 | 0.53 | 1 |
| GO:0006068 | ethanol catabolic process | 6 | 0 | 0.23 | 1 |
| GO:0006069 | ethanol oxidation | 11 | 0 | 0.42 | 1 |
| GO:0006072 | glycerol-3-phosphate metabolic process | 6 | 0 | 0.23 | 1 |
| GO:0006083 | acetate metabolic process | 3 | 0 | 0.11 | 1 |
| GO:0006089 | lactate metabolic process | 7 | 0 | 0.27 | 1 |
| GO:0006097 | glyoxylate cycle | 2 | 0 | 0.08 | 1 |
| GO:0006098 | pentose-phosphate shunt | 12 | 0 | 0.46 | 1 |
| GO:0006102 | isocitrate metabolic process | 5 | 0 | 0.19 | 1 |
| GO:0006103 | 2-oxoglutarate metabolic process | 16 | 0 | 0.61 | 1 |
| GO:0006104 | succinyl-CoA metabolic process | 5 | 0 | 0.19 | 1 |
| GO:0006106 | fumarate metabolic process | 3 | 0 | 0.11 | 1 |
| GO:0006107 | oxaloacetate metabolic process | 11 | 0 | 0.42 | 1 |
| GO:0006113 | fermentation | 1 | 0 | 0.04 | 1 |
| GO:0006116 | NADH oxidation | 3 | 0 | 0.11 | 1 |
| GO:0006117 | acetaldehyde metabolic process | 1 | 0 | 0.04 | 1 |
| GO:0006121 | mitochondrial electron transport | succin... | 1 | 0 | 0.04 |
| GO:0006122 | mitochondrial electron transport | ubiqui... | 5 | 0 | 0.19 |
| GO:0006123 | mitochondrial electron transport | cytoch... | 3 | 0 | 0.11 |
| GO:0006127 | glycerophosphate shuttle | 1 | 0 | 0.04 | 1 |
| GO:0006145 | purine nucleobase catabolic process | 3 | 0 | 0.11 | 1 |
| GO:0006147 | guanine catabolic process | 1 | 0 | 0.04 | 1 |
| GO:0006152 | purine nucleoside catabolic process | 12 | 0 | 0.46 | 1 |
| GO:0006154 | adenosine catabolic process | 3 | 0 | 0.11 | 1 |
| GO:0006157 | deoxyadenosine catabolic process | 1 | 0 | 0.04 | 1 |
| GO:0006168 | adenine salvage | 2 | 0 | 0.08 | 1 |
| GO:0006172 | ADP biosynthetic process | 4 | 0 | 0.15 | 1 |
| GO:0006173 | dADP biosynthetic process | 1 | 0 | 0.04 | 1 |
| GO:0006175 | dATP biosynthetic process | 1 | 0 | 0.04 | 1 |
| GO:0006177 | GMP biosynthetic process | 4 | 0 | 0.15 | 1 |
| GO:0006178 | guanine salvage | 1 | 0 | 0.04 | 1 |
| GO:0006182 | cGMP biosynthetic process | 30 | 0 | 1.14 | 1 |
| GO:0006183 | GTP biosynthetic process | 8 | 0 | 0.3 | 1 |
| GO:0006185 | dGDP biosynthetic process | 1 | 0 | 0.04 | 1 |
| GO:0006188 | IMP biosynthetic process | 10 | 0 | 0.38 | 1 |
| GO:0006189 | 'de novo' IMP biosynthetic process | 6 | 0 | 0.23 | 1 |
| GO:0006193 | ITP catabolic process | 1 | 0 | 0.04 | 1 |
| GO:0006196 | AMP catabolic process | 2 | 0 | 0.08 | 1 |

| | | | | | | |
|---|---|---|---|---|---|---|
| GO:0006203 | dGTP catabolic process | | 3 | 0 | 0.11 | 1 |
| GO:0006207 | 'de novo' pyrimidine nucleobase biosynth.. | | 4 | 0 | 0.15 | 1 |
| GO:0006208 | pyrimidine nucleobase catabolic process | | 8 | 0 | 0.3 | 1 |
| GO:0006210 | thymine catabolic process | | 3 | 0 | 0.11 | 1 |
| GO:0006211 | 5-methylcytosine catabolic process | | 1 | 0 | 0.04 | 1 |
| GO:0006212 | uracil catabolic process | | 2 | 0 | 0.08 | 1 |
| GO:0006214 | thymidine catabolic process | | 1 | 0 | 0.04 | 1 |
| GO:0006216 | cytidine catabolic process | | 9 | 0 | 0.34 | 1 |
| GO:0006222 | UMP biosynthetic process | | 11 | 0 | 0.42 | 1 |
| GO:0006225 | UDP biosynthetic process | | 1 | 0 | 0.04 | 1 |
| GO:0006226 | dUMP biosynthetic process | | 1 | 0 | 0.04 | 1 |
| GO:0006228 | UTP biosynthetic process | | 9 | 0 | 0.34 | 1 |
| GO:0006231 | dTMP biosynthetic process | | 1 | 0 | 0.04 | 1 |
| GO:0006238 | CMP salvage | | 1 | 0 | 0.04 | 1 |
| GO:0006240 | dCDP biosynthetic process | | 1 | 0 | 0.04 | 1 |
| GO:0006241 | CTP biosynthetic process | | 12 | 0 | 0.46 | 1 |
| GO:0006256 | UDP catabolic process | | 1 | 0 | 0.04 | 1 |
| GO:0006258 | UDP-glucose catabolic process | | 2 | 0 | 0.08 | 1 |
| GO:0006264 | mitochondrial DNA replication | | 12 | 0 | 0.46 | 1 |
| GO:0006265 | DNA topological change | | 9 | 0 | 0.34 | 1 |
| GO:0006266 | DNA ligation | | 13 | 0 | 0.49 | 1 |
| GO:0006268 | DNA unwinding involved in DNA replicatio. | | 9 | 0 | 0.34 | 1 |
| GO:0006269 | DNA replication | synthesis o | 4 | 0 | 0.15 | |
| GO:0006270 | DNA replication initiation | | 23 | 0 | 0.88 | 1 |
| GO:0006271 | DNA strand elongation involved in DNA re. | | 31 | 0 | 1.18 | 1 |
| GO:0006272 | leading strand elongation | | 2 | 0 | 0.08 | 1 |
| GO:0006273 | lagging strand elongation | | 4 | 0 | 0.15 | 1 |
| GO:0006283 | transcription-coupled nucleotide-excisio... | | 47 | 0 | 1.79 | 1 |
| GO:0006287 | base-excision repair | gap-filling | 4 | 0 | 0.15 | |
| GO:0006288 | base-excision repair | DNA ligatio | 3 | 0 | 0.11 | |
| GO:0006290 | pyrimidine dimer repair | | 7 | 0 | 0.27 | 1 |
| GO:0006294 | nucleotide-excision repair | preincision | 1 | 0 | 0.04 | |
| GO:0006295 | nucleotide-excision repair | DNA incisio | 3 | 0 | 0.11 | |
| GO:0006296 | nucleotide-excision repair | DNA incisio | 3 | 0 | 0.11 | |
| GO:0006297 | nucleotide-excision repair | DNA gap fil | 19 | 0 | 0.72 | |
| GO:0006298 | mismatch repair | | 26 | 0 | 0.99 | 1 |
| GO:0006305 | DNA alkylation | | 78 | 0 | 2.97 | 1 |
| GO:0006306 | DNA methylation | | 78 | 0 | 2.97 | 1 |
| GO:0006307 | DNA dealkylation involved in DNA repair | | 7 | 0 | 0.27 | 1 |
| GO:0006309 | apoptotic DNA fragmentation | | 20 | 0 | 0.76 | 1 |
| GO:0006313 | transposition | DNA-media | 2 | 0 | 0.08 | |
| GO:0006335 | DNA replication-dependent nucleosome as | | 31 | 0 | 1.18 | 1 |
| GO:0006343 | establishment of chromatin silencing | | 1 | 0 | 0.04 | 1 |
| GO:0006344 | maintenance of chromatin silencing | | 3 | 0 | 0.11 | 1 |
| GO:0006346 | methylation-dependent chromatin silencin. | | 10 | 0 | 0.38 | 1 |
| GO:0006348 | chromatin silencing at telomere | | 2 | 0 | 0.08 | 1 |
| GO:0006349 | regulation of gene expression by genetic... | | 17 | 0 | 0.65 | 1 |
| GO:0006370 | 7-methylguanosine mRNA capping | | 29 | 0 | 1.1 | 1 |
| GO:0006382 | adenosine to inosine editing | | 3 | 0 | 0.11 | 1 |
| GO:0006388 | tRNA splicing | via endonu | 8 | 0 | 0.3 | |
| GO:0006398 | histone mRNA 3'-end processing | | 5 | 0 | 0.19 | 1 |
| GO:0006404 | RNA import into nucleus | | 2 | 0 | 0.08 | 1 |
| GO:0006407 | rRNA export from nucleus | | 2 | 0 | 0.08 | 1 |

| | | | | | |
|---|---|---|---|---|---|
| GO:0006408 | snRNA export from nucleus | 2 | 0 | 0.08 | 1 |
| GO:0006409 | tRNA export from nucleus | 2 | 0 | 0.08 | 1 |
| GO:0006419 | alanyl-tRNA aminoacylation | 3 | 0 | 0.11 | 1 |
| GO:0006420 | arginyl-tRNA aminoacylation | 3 | 0 | 0.11 | 1 |
| GO:0006421 | asparaginyl-tRNA aminoacylation | 3 | 0 | 0.11 | 1 |
| GO:0006422 | aspartyl-tRNA aminoacylation | 1 | 0 | 0.04 | 1 |
| GO:0006423 | cysteinyl-tRNA aminoacylation | 2 | 0 | 0.08 | 1 |
| GO:0006424 | glutamyl-tRNA aminoacylation | 2 | 0 | 0.08 | 1 |
| GO:0006425 | glutaminyl-tRNA aminoacylation | 1 | 0 | 0.04 | 1 |
| GO:0006426 | glycyl-tRNA aminoacylation | 1 | 0 | 0.04 | 1 |
| GO:0006427 | histidyl-tRNA aminoacylation | 2 | 0 | 0.08 | 1 |
| GO:0006428 | isoleucyl-tRNA aminoacylation | 2 | 0 | 0.08 | 1 |
| GO:0006429 | leucyl-tRNA aminoacylation | 2 | 0 | 0.08 | 1 |
| GO:0006430 | lysyl-tRNA aminoacylation | 1 | 0 | 0.04 | 1 |
| GO:0006431 | methionyl-tRNA aminoacylation | 2 | 0 | 0.08 | 1 |
| GO:0006432 | phenylalanyl-tRNA aminoacylation | 4 | 0 | 0.15 | 1 |
| GO:0006434 | seryl-tRNA aminoacylation | 2 | 0 | 0.08 | 1 |
| GO:0006435 | threonyl-tRNA aminoacylation | 3 | 0 | 0.11 | 1 |
| GO:0006436 | tryptophanyl-tRNA aminoacylation | 2 | 0 | 0.08 | 1 |
| GO:0006437 | tyrosyl-tRNA aminoacylation | 2 | 0 | 0.08 | 1 |
| GO:0006438 | valyl-tRNA aminoacylation | 2 | 0 | 0.08 | 1 |
| GO:0006447 | regulation of translational initiation b... | 3 | 0 | 0.11 | 1 |
| GO:0006448 | regulation of translational elongation | 18 | 0 | 0.69 | 1 |
| GO:0006449 | regulation of translational termination | 7 | 0 | 0.27 | 1 |
| GO:0006450 | regulation of translational fidelity | 12 | 0 | 0.46 | 1 |
| GO:0006451 | translational readthrough | 7 | 0 | 0.27 | 1 |
| GO:0006452 | translational frameshifting | 2 | 0 | 0.08 | 1 |
| GO:0006463 | steroid hormone receptor complex assemb | 1 | 0 | 0.04 | 1 |
| GO:0006477 | protein sulfation | 6 | 0 | 0.23 | 1 |
| GO:0006478 | peptidyl-tyrosine sulfation | 2 | 0 | 0.08 | 1 |
| GO:0006480 | N-terminal protein amino acid methylatio... | 1 | 0 | 0.04 | 1 |
| GO:0006481 | C-terminal protein methylation | 2 | 0 | 0.08 | 1 |
| GO:0006482 | protein demethylation | 26 | 0 | 0.99 | 1 |
| GO:0006489 | dolichyl diphosphate biosynthetic proces... | 5 | 0 | 0.19 | 1 |
| GO:0006490 | oligosaccharide-lipid intermediate biosy... | 2 | 0 | 0.08 | 1 |
| GO:0006500 | N-terminal protein palmitoylation | 2 | 0 | 0.08 | 1 |
| GO:0006507 | GPI anchor release | 1 | 0 | 0.04 | 1 |
| GO:0006509 | membrane protein ectodomain proteolysis | 39 | 0 | 1.48 | 1 |
| GO:0006515 | misfolded or incompletely synthesized pr... | 12 | 0 | 0.46 | 1 |
| GO:0006516 | glycoprotein catabolic process | 13 | 0 | 0.49 | 1 |
| GO:0006517 | protein deglycosylation | 4 | 0 | 0.15 | 1 |
| GO:0006522 | alanine metabolic process | 5 | 0 | 0.19 | 1 |
| GO:0006524 | alanine catabolic process | 5 | 0 | 0.19 | 1 |
| GO:0006526 | arginine biosynthetic process | 6 | 0 | 0.23 | 1 |
| GO:0006528 | asparagine metabolic process | 5 | 0 | 0.19 | 1 |
| GO:0006529 | asparagine biosynthetic process | 2 | 0 | 0.08 | 1 |
| GO:0006530 | asparagine catabolic process | 1 | 0 | 0.04 | 1 |
| GO:0006531 | aspartate metabolic process | 7 | 0 | 0.27 | 1 |
| GO:0006532 | aspartate biosynthetic process | 2 | 0 | 0.08 | 1 |
| GO:0006533 | aspartate catabolic process | 4 | 0 | 0.15 | 1 |
| GO:0006535 | cysteine biosynthetic process from serin... | 1 | 0 | 0.04 | 1 |
| GO:0006541 | glutamine metabolic process | 23 | 0 | 0.88 | 1 |
| GO:0006542 | glutamine biosynthetic process | 2 | 0 | 0.08 | 1 |

| | | | | | |
|---|---|---|---|---|---|
| GO:0006543 | glutamine catabolic process | 4 | 0 | 0.15 | 1 |
| GO:0006545 | glycine biosynthetic process | 6 | 0 | 0.23 | 1 |
| GO:0006547 | histidine metabolic process | 7 | 0 | 0.27 | 1 |
| GO:0006548 | histidine catabolic process | 5 | 0 | 0.19 | 1 |
| GO:0006549 | isoleucine metabolic process | 4 | 0 | 0.15 | 1 |
| GO:0006550 | isoleucine catabolic process | 1 | 0 | 0.04 | 1 |
| GO:0006551 | leucine metabolic process | 7 | 0 | 0.27 | 1 |
| GO:0006552 | leucine catabolic process | 5 | 0 | 0.19 | 1 |
| GO:0006553 | lysine metabolic process | 9 | 0 | 0.34 | 1 |
| GO:0006554 | lysine catabolic process | 9 | 0 | 0.34 | 1 |
| GO:0006557 | S-adenosylmethioninamine biosynthetic pr | 1 | 0 | 0.04 | 1 |
| GO:0006560 | proline metabolic process | 9 | 0 | 0.34 | 1 |
| GO:0006561 | proline biosynthetic process | 6 | 0 | 0.23 | 1 |
| GO:0006562 | proline catabolic process | 4 | 0 | 0.15 | 1 |
| GO:0006563 | L-serine metabolic process | 9 | 0 | 0.34 | 1 |
| GO:0006564 | L-serine biosynthetic process | 4 | 0 | 0.15 | 1 |
| GO:0006565 | L-serine catabolic process | 4 | 0 | 0.15 | 1 |
| GO:0006567 | threonine catabolic process | 3 | 0 | 0.11 | 1 |
| GO:0006568 | tryptophan metabolic process | 13 | 0 | 0.49 | 1 |
| GO:0006569 | tryptophan catabolic process | 11 | 0 | 0.42 | 1 |
| GO:0006573 | valine metabolic process | 8 | 0 | 0.3 | 1 |
| GO:0006574 | valine catabolic process | 4 | 0 | 0.15 | 1 |
| GO:0006577 | amino-acid betaine metabolic process | 16 | 0 | 0.61 | 1 |
| GO:0006578 | amino-acid betaine biosynthetic process | 7 | 0 | 0.27 | 1 |
| GO:0006579 | amino-acid betaine catabolic process | 3 | 0 | 0.11 | 1 |
| GO:0006581 | acetylcholine catabolic process | 2 | 0 | 0.08 | 1 |
| GO:0006582 | melanin metabolic process | 17 | 0 | 0.65 | 1 |
| GO:0006583 | melanin biosynthetic process from tyrosi... | 2 | 0 | 0.08 | 1 |
| GO:0006584 | catecholamine metabolic process | 48 | 0 | 1.83 | 1 |
| GO:0006585 | dopamine biosynthetic process from tyros. | 1 | 0 | 0.04 | 1 |
| GO:0006586 | indolalkylamine metabolic process | 18 | 0 | 0.69 | 1 |
| GO:0006590 | thyroid hormone generation | 15 | 0 | 0.57 | 1 |
| GO:0006591 | ornithine metabolic process | 5 | 0 | 0.19 | 1 |
| GO:0006592 | ornithine biosynthetic process | 1 | 0 | 0.04 | 1 |
| GO:0006593 | ornithine catabolic process | 1 | 0 | 0.04 | 1 |
| GO:0006596 | polyamine biosynthetic process | 12 | 0 | 0.46 | 1 |
| GO:0006597 | spermine biosynthetic process | 2 | 0 | 0.08 | 1 |
| GO:0006598 | polyamine catabolic process | 5 | 0 | 0.19 | 1 |
| GO:0006599 | phosphagen metabolic process | 1 | 0 | 0.04 | 1 |
| GO:0006600 | creatine metabolic process | 11 | 0 | 0.42 | 1 |
| GO:0006601 | creatine biosynthetic process | 2 | 0 | 0.08 | 1 |
| GO:0006603 | phosphocreatine metabolic process | 1 | 0 | 0.04 | 1 |
| GO:0006608 | snRNP protein import into nucleus | 1 | 0 | 0.04 | 1 |
| GO:0006610 | ribosomal protein import into nucleus | 4 | 0 | 0.15 | 1 |
| GO:0006616 | SRP-dependent cotranslational protein ta.. | 2 | 0 | 0.08 | 1 |
| GO:0006617 | SRP-dependent cotranslational protein ta.. | 1 | 0 | 0.04 | 1 |
| GO:0006620 | posttranslational protein targeting to m... | 6 | 0 | 0.23 | 1 |
| GO:0006621 | protein retention in ER lumen | 9 | 0 | 0.34 | 1 |
| GO:0006625 | protein targeting to peroxisome | 16 | 0 | 0.61 | 1 |
| GO:0006627 | protein processing involved in protein t... | 4 | 0 | 0.15 | 1 |
| GO:0006642 | triglyceride mobilization | 5 | 0 | 0.19 | 1 |
| GO:0006646 | phosphatidylethanolamine biosynthetic pr.. | 13 | 0 | 0.49 | 1 |
| GO:0006649 | phospholipid transfer to membrane | 2 | 0 | 0.08 | 1 |

| | | | | | |
|---|---|---|---|---|---|
| GO:0006651 | diacylglycerol biosynthetic process | 4 | 0 | 0.15 | 1 |
| GO:0006654 | phosphatidic acid biosynthetic process | 30 | 0 | 1.14 | 1 |
| GO:0006655 | phosphatidylglycerol biosynthetic proces... | 12 | 0 | 0.46 | 1 |
| GO:0006657 | CDP-choline pathway | 7 | 0 | 0.27 | 1 |
| GO:0006660 | phosphatidylserine catabolic process | 1 | 0 | 0.04 | 1 |
| GO:0006662 | glycerol ether metabolic process | 12 | 0 | 0.46 | 1 |
| GO:0006666 | 3-keto-sphinganine metabolic process | 1 | 0 | 0.04 | 1 |
| GO:0006667 | sphinganine metabolic process | 3 | 0 | 0.11 | 1 |
| GO:0006668 | sphinganine-1-phosphate metabolic proce: | 2 | 0 | 0.08 | 1 |
| GO:0006669 | sphinganine-1-phosphate biosynthetic pro. | 1 | 0 | 0.04 | 1 |
| GO:0006670 | sphingosine metabolic process | 10 | 0 | 0.38 | 1 |
| GO:0006671 | phytosphingosine metabolic process | 1 | 0 | 0.04 | 1 |
| GO:0006677 | glycosylceramide metabolic process | 15 | 0 | 0.57 | 1 |
| GO:0006678 | glucosylceramide metabolic process | 7 | 0 | 0.27 | 1 |
| GO:0006679 | glucosylceramide biosynthetic process | 2 | 0 | 0.08 | 1 |
| GO:0006680 | glucosylceramide catabolic process | 3 | 0 | 0.11 | 1 |
| GO:0006681 | galactosylceramide metabolic process | 5 | 0 | 0.19 | 1 |
| GO:0006682 | galactosylceramide biosynthetic process | 2 | 0 | 0.08 | 1 |
| GO:0006683 | galactosylceramide catabolic process | 1 | 0 | 0.04 | 1 |
| GO:0006684 | sphingomyelin metabolic process | 14 | 0 | 0.53 | 1 |
| GO:0006685 | sphingomyelin catabolic process | 7 | 0 | 0.27 | 1 |
| GO:0006686 | sphingomyelin biosynthetic process | 5 | 0 | 0.19 | 1 |
| GO:0006689 | ganglioside catabolic process | 5 | 0 | 0.19 | 1 |
| GO:0006691 | leukotriene metabolic process | 31 | 0 | 1.18 | 1 |
| GO:0006701 | progesterone biosynthetic process | 7 | 0 | 0.27 | 1 |
| GO:0006702 | androgen biosynthetic process | 15 | 0 | 0.57 | 1 |
| GO:0006703 | estrogen biosynthetic process | 8 | 0 | 0.3 | 1 |
| GO:0006704 | glucocorticoid biosynthetic process | 17 | 0 | 0.65 | 1 |
| GO:0006706 | steroid catabolic process | 24 | 0 | 0.91 | 1 |
| GO:0006707 | cholesterol catabolic process | 11 | 0 | 0.42 | 1 |
| GO:0006710 | androgen catabolic process | 2 | 0 | 0.08 | 1 |
| GO:0006711 | estrogen catabolic process | 1 | 0 | 0.04 | 1 |
| GO:0006713 | glucocorticoid catabolic process | 1 | 0 | 0.04 | 1 |
| GO:0006714 | sesquiterpenoid metabolic process | 3 | 0 | 0.11 | 1 |
| GO:0006722 | triterpenoid metabolic process | 1 | 0 | 0.04 | 1 |
| GO:0006726 | eye pigment biosynthetic process | 4 | 0 | 0.15 | 1 |
| GO:0006729 | tetrahydrobiopterin biosynthetic process | 6 | 0 | 0.23 | 1 |
| GO:0006734 | NADH metabolic process | 9 | 0 | 0.34 | 1 |
| GO:0006739 | NADP metabolic process | 27 | 0 | 1.03 | 1 |
| GO:0006740 | NADPH regeneration | 2 | 0 | 0.08 | 1 |
| GO:0006741 | NADP biosynthetic process | 2 | 0 | 0.08 | 1 |
| GO:0006742 | NADP catabolic process | 1 | 0 | 0.04 | 1 |
| GO:0006743 | ubiquinone metabolic process | 12 | 0 | 0.46 | 1 |
| GO:0006744 | ubiquinone biosynthetic process | 11 | 0 | 0.42 | 1 |
| GO:0006747 | FAD biosynthetic process | 1 | 0 | 0.04 | 1 |
| GO:0006751 | glutathione catabolic process | 1 | 0 | 0.04 | 1 |
| GO:0006760 | folic acid-containing compound metabolic.. | 25 | 0 | 0.95 | 1 |
| GO:0006768 | biotin metabolic process | 11 | 0 | 0.42 | 1 |
| GO:0006769 | nicotinamide metabolic process | 3 | 0 | 0.11 | 1 |
| GO:0006772 | thiamine metabolic process | 4 | 0 | 0.15 | 1 |
| GO:0006775 | fat-soluble vitamin metabolic process | 40 | 0 | 1.52 | 1 |
| GO:0006776 | vitamin A metabolic process | 8 | 0 | 0.3 | 1 |
| GO:0006777 | Mo-molybdopterin cofactor biosynthetic p.. | 7 | 0 | 0.27 | 1 |

| | | | | | |
|---|---|---|---|---|---|
| GO:0006779 | porphyrin-containing compound biosynthet | 26 | 0 | 0.99 | 1 |
| GO:0006780 | uroporphyrinogen III biosynthetic proces... | 2 | 0 | 0.08 | 1 |
| GO:0006781 | succinyl-CoA pathway | 1 | 0 | 0.04 | 1 |
| GO:0006782 | protoporphyrinogen IX biosynthetic proce.. | 9 | 0 | 0.34 | 1 |
| GO:0006783 | heme biosynthetic process | 20 | 0 | 0.76 | 1 |
| GO:0006784 | heme a biosynthetic process | 2 | 0 | 0.08 | 1 |
| GO:0006787 | porphyrin-containing compound catabolic . | 7 | 0 | 0.27 | 1 |
| GO:0006788 | heme oxidation | 2 | 0 | 0.08 | 1 |
| GO:0006789 | bilirubin conjugation | 1 | 0 | 0.04 | 1 |
| GO:0006797 | polyphosphate metabolic process | 1 | 0 | 0.04 | 1 |
| GO:0006808 | regulation of nitrogen utilization | 3 | 0 | 0.11 | 1 |
| GO:0006824 | cobalt ion transport | 5 | 0 | 0.19 | 1 |
| GO:0006828 | manganese ion transport | 12 | 0 | 0.46 | 1 |
| GO:0006829 | zinc II ion transport | 27 | 0 | 1.03 | 1 |
| GO:0006837 | serotonin transport | 15 | 0 | 0.57 | 1 |
| GO:0006842 | tricarboxylic acid transport | 3 | 0 | 0.11 | 1 |
| GO:0006844 | acyl carnitine transport | 1 | 0 | 0.04 | 1 |
| GO:0006848 | pyruvate transport | 1 | 0 | 0.04 | 1 |
| GO:0006851 | mitochondrial calcium ion transport | 3 | 0 | 0.11 | 1 |
| GO:0006855 | drug transmembrane transport | 17 | 0 | 0.65 | 1 |
| GO:0006857 | oligopeptide transport | 8 | 0 | 0.3 | 1 |
| GO:0006858 | extracellular transport | 6 | 0 | 0.23 | 1 |
| GO:0006860 | extracellular amino acid transport | 1 | 0 | 0.04 | 1 |
| GO:0006862 | nucleotide transport | 21 | 0 | 0.8 | 1 |
| GO:0006863 | purine nucleobase transport | 5 | 0 | 0.19 | 1 |
| GO:0006864 | pyrimidine nucleotide transport | 2 | 0 | 0.08 | 1 |
| GO:0006867 | asparagine transport | 2 | 0 | 0.08 | 1 |
| GO:0006876 | cellular cadmium ion homeostasis | 1 | 0 | 0.04 | 1 |
| GO:0006878 | cellular copper ion homeostasis | 14 | 0 | 0.53 | 1 |
| GO:0006880 | intracellular sequestering of iron ion | 2 | 0 | 0.08 | 1 |
| GO:0006882 | cellular zinc ion homeostasis | 18 | 0 | 0.69 | 1 |
| GO:0006883 | cellular sodium ion homeostasis | 13 | 0 | 0.49 | 1 |
| GO:0006896 | Golgi to vacuole transport | 6 | 0 | 0.23 | 1 |
| GO:0006904 | vesicle docking involved in exocytosis | 41 | 0 | 1.56 | 1 |
| GO:0006910 | phagocytosis | recognition | 11 | 0 | 0.42 |
| GO:0006911 | phagocytosis | engulfment | 23 | 0 | 0.88 |
| GO:0006924 | activation-induced cell death of T cells | 11 | 0 | 0.42 | 1 |
| GO:0006925 | inflammatory cell apoptotic process | 13 | 0 | 0.49 | 1 |
| GO:0006927 | transformed cell apoptotic process | 6 | 0 | 0.23 | 1 |
| GO:0006929 | substrate-dependent cell migration | 26 | 0 | 0.99 | 1 |
| GO:0006930 | substrate-dependent cell migration | cell... | 7 | 0 | 0.27 |
| GO:0006931 | substrate-dependent cell migration | cell... | 3 | 0 | 0.11 |
| GO:0006933 | negative regulation of cell adhesion inv... | 4 | 0 | 0.15 | 1 |
| GO:0006948 | induction by virus of host cell-cell fus... | 1 | 0 | 0.04 | 1 |
| GO:0006953 | acute-phase response | 46 | 0 | 1.75 | 1 |
| GO:0006963 | positive regulation of antibacterial pep... | 2 | 0 | 0.08 | 1 |
| GO:0006965 | positive regulation of biosynthetic proc... | 2 | 0 | 0.08 | 1 |
| GO:0006975 | DNA damage induced protein phosphoryla | 8 | 0 | 0.3 | 1 |
| GO:0006982 | response to lipid hydroperoxide | 4 | 0 | 0.15 | 1 |
| GO:0006983 | ER overload response | 9 | 0 | 0.34 | 1 |
| GO:0006990 | positive regulation of transcription fro... | 4 | 0 | 0.15 | 1 |
| GO:0006999 | nuclear pore organization | 10 | 0 | 0.38 | 1 |
| GO:0007000 | nucleolus organization | 5 | 0 | 0.19 | 1 |

| | | | | | |
|---|---|---|---|---|---|
| GO:0007016 | cytoskeletal anchoring at plasma membrar | 12 | 0 | 0.46 | 1 |
| GO:0007020 | microtubule nucleation | 17 | 0 | 0.65 | 1 |
| GO:0007021 | tubulin complex assembly | 4 | 0 | 0.15 | 1 |
| GO:0007023 | post-chaperonin tubulin folding pathway | 4 | 0 | 0.15 | 1 |
| GO:0007028 | cytoplasm organization | 8 | 0 | 0.3 | 1 |
| GO:0007031 | peroxisome organization | 32 | 0 | 1.22 | 1 |
| GO:0007039 | protein catabolic process in the vacuole | 1 | 0 | 0.04 | 1 |
| GO:0007042 | lysosomal lumen acidification | 4 | 0 | 0.15 | 1 |
| GO:0007056 | spindle assembly involved in female meio. | 2 | 0 | 0.08 | 1 |
| GO:0007057 | spindle assembly involved in female meio. | 2 | 0 | 0.08 | 1 |
| GO:0007060 | male meiosis chromosome segregation | 2 | 0 | 0.08 | 1 |
| GO:0007068 | negative regulation of transcription dur... | 2 | 0 | 0.08 | 1 |
| GO:0007070 | negative regulation of transcription fro... | 2 | 0 | 0.08 | 1 |
| GO:0007079 | mitotic chromosome movement towards sp | 3 | 0 | 0.11 | 1 |
| GO:0007080 | mitotic metaphase plate congression | 30 | 0 | 1.14 | 1 |
| GO:0007084 | mitotic nuclear envelope reassembly | 10 | 0 | 0.38 | 1 |
| GO:0007096 | regulation of exit from mitosis | 12 | 0 | 0.46 | 1 |
| GO:0007097 | nuclear migration | 7 | 0 | 0.27 | 1 |
| GO:0007100 | mitotic centrosome separation | 7 | 0 | 0.27 | 1 |
| GO:0007113 | endomitotic cell cycle | 3 | 0 | 0.11 | 1 |
| GO:0007130 | synaptonemal complex assembly | 14 | 0 | 0.53 | 1 |
| GO:0007144 | female meiosis I | 5 | 0 | 0.19 | 1 |
| GO:0007147 | female meiosis II | 1 | 0 | 0.04 | 1 |
| GO:0007161 | calcium-independent cell-matrix adhesion | 4 | 0 | 0.15 | 1 |
| GO:0007168 | receptor guanylyl cyclase signaling path... | 11 | 0 | 0.42 | 1 |
| GO:0007171 | activation of transmembrane receptor pro.. | 11 | 0 | 0.42 | 1 |
| GO:0007174 | epidermal growth factor catabolic proces... | 1 | 0 | 0.04 | 1 |
| GO:0007175 | negative regulation of epidermal growth ... | 8 | 0 | 0.3 | 1 |
| GO:0007176 | regulation of epidermal growth factor-ac... | 23 | 0 | 0.88 | 1 |
| GO:0007181 | transforming growth factor beta receptor... | 3 | 0 | 0.11 | 1 |
| GO:0007182 | common-partner SMAD protein phosphory | 6 | 0 | 0.23 | 1 |
| GO:0007184 | SMAD protein import into nucleus | 20 | 0 | 0.76 | 1 |
| GO:0007195 | adenylate cyclase-inhibiting dopamine re.. | 5 | 0 | 0.19 | 1 |
| GO:0007196 | adenylate cyclase-inhibiting G-protein c... | 7 | 0 | 0.27 | 1 |
| GO:0007197 | adenylate cyclase-inhibiting G-protein c... | 5 | 0 | 0.19 | 1 |
| GO:0007198 | adenylate cyclase-inhibiting serotonin r... | 1 | 0 | 0.04 | 1 |
| GO:0007199 | G-protein coupled receptor signaling pat... | 3 | 0 | 0.11 | 1 |
| GO:0007206 | phospholipase C-activating G-protein cou.. | 2 | 0 | 0.08 | 1 |
| GO:0007207 | phospholipase C-activating G-protein cou.. | 6 | 0 | 0.23 | 1 |
| GO:0007208 | phospholipase C-activating serotonin rec... | 2 | 0 | 0.08 | 1 |
| GO:0007213 | G-protein coupled acetylcholine receptor... | 12 | 0 | 0.46 | 1 |
| GO:0007216 | G-protein coupled glutamate receptor sig.. | 13 | 0 | 0.49 | 1 |
| GO:0007217 | tachykinin receptor signaling pathway | 8 | 0 | 0.3 | 1 |
| GO:0007221 | positive regulation of transcription of ... | 5 | 0 | 0.19 | 1 |
| GO:0007223 | Wnt signaling pathway | calcium mo | 3 | 0 | 0.11 |
| GO:0007225 | patched ligand maturation | 1 | 0 | 0.04 | 1 |
| GO:0007227 | signal transduction downstream of smooth | 4 | 0 | 0.15 | 1 |
| GO:0007228 | positive regulation of hh target transcr... | 3 | 0 | 0.11 | 1 |
| GO:0007231 | osmosensory signaling pathway | 1 | 0 | 0.04 | 1 |
| GO:0007252 | I-kappaB phosphorylation | 13 | 0 | 0.49 | 1 |
| GO:0007253 | cytoplasmic sequestering of NF-kappaB | 11 | 0 | 0.42 | 1 |
| GO:0007256 | activation of JNKK activity | 6 | 0 | 0.23 | 1 |
| GO:0007263 | nitric oxide mediated signal transductio... | 23 | 0 | 0.88 | 1 |

| | | | | | |
|---|---|---|---|---|---|
| GO:0007271 | synaptic transmission | cholinergic | 29 | 0 | 1.1 |
| GO:0007274 | neuromuscular synaptic transmission | 21 | 0 | 0.8 | 1 |
| GO:0007285 | primary spermatocyte growth | 1 | 0 | 0.04 | 1 |
| GO:0007288 | sperm axoneme assembly | 5 | 0 | 0.19 | 1 |
| GO:0007289 | spermatid nucleus differentiation | 19 | 0 | 0.72 | 1 |
| GO:0007290 | spermatid nucleus elongation | 4 | 0 | 0.15 | 1 |
| GO:0007291 | sperm individualization | 1 | 0 | 0.04 | 1 |
| GO:0007296 | vitellogenesis | 2 | 0 | 0.08 | 1 |
| GO:0007308 | oocyte construction | 2 | 0 | 0.08 | 1 |
| GO:0007309 | oocyte axis specification | 2 | 0 | 0.08 | 1 |
| GO:0007314 | oocyte anterior/posterior axis specifica... | 2 | 0 | 0.08 | 1 |
| GO:0007315 | pole plasm assembly | 2 | 0 | 0.08 | 1 |
| GO:0007320 | insemination | 13 | 0 | 0.49 | 1 |
| GO:0007340 | acrosome reaction | 22 | 0 | 0.84 | 1 |
| GO:0007341 | penetration of zona pellucida | 5 | 0 | 0.19 | 1 |
| GO:0007343 | egg activation | 7 | 0 | 0.27 | 1 |
| GO:0007349 | cellularization | 1 | 0 | 0.04 | 1 |
| GO:0007350 | blastoderm segmentation | 17 | 0 | 0.65 | 1 |
| GO:0007351 | tripartite regional subdivision | 14 | 0 | 0.53 | 1 |
| GO:0007352 | zygotic specification of dorsal/ventral ... | 3 | 0 | 0.11 | 1 |
| GO:0007354 | zygotic determination of anterior/poster... | 4 | 0 | 0.15 | 1 |
| GO:0007356 | thorax and anterior abdomen determinatio | 2 | 0 | 0.08 | 1 |
| GO:0007371 | ventral midline determination | 1 | 0 | 0.04 | 1 |
| GO:0007386 | compartment pattern specification | 5 | 0 | 0.19 | 1 |
| GO:0007387 | anterior compartment pattern formation | 1 | 0 | 0.04 | 1 |
| GO:0007388 | posterior compartment specification | 1 | 0 | 0.04 | 1 |
| GO:0007400 | neuroblast fate determination | 1 | 0 | 0.04 | 1 |
| GO:0007402 | ganglion mother cell fate determination | 2 | 0 | 0.08 | 1 |
| GO:0007403 | glial cell fate determination | 3 | 0 | 0.11 | 1 |
| GO:0007406 | negative regulation of neuroblast prolif... | 7 | 0 | 0.27 | 1 |
| GO:0007412 | axon target recognition | 4 | 0 | 0.15 | 1 |
| GO:0007418 | ventral midline development | 4 | 0 | 0.15 | 1 |
| GO:0007424 | open tracheal system development | 1 | 0 | 0.04 | 1 |
| GO:0007439 | ectodermal digestive tract development | 2 | 0 | 0.08 | 1 |
| GO:0007440 | foregut morphogenesis | 12 | 0 | 0.46 | 1 |
| GO:0007468 | regulation of rhodopsin gene expression | 3 | 0 | 0.11 | 1 |
| GO:0007493 | endodermal cell fate determination | 2 | 0 | 0.08 | 1 |
| GO:0007494 | midgut development | 12 | 0 | 0.46 | 1 |
| GO:0007495 | visceral mesoderm-endoderm interaction i | 1 | 0 | 0.04 | 1 |
| GO:0007497 | posterior midgut development | 2 | 0 | 0.08 | 1 |
| GO:0007499 | ectoderm and mesoderm interaction | 1 | 0 | 0.04 | 1 |
| GO:0007500 | mesodermal cell fate determination | 3 | 0 | 0.11 | 1 |
| GO:0007501 | mesodermal cell fate specification | 13 | 0 | 0.49 | 1 |
| GO:0007506 | gonadal mesoderm development | 5 | 0 | 0.19 | 1 |
| GO:0007509 | mesoderm migration involved in gastrulat.. | 2 | 0 | 0.08 | 1 |
| GO:0007518 | myoblast fate determination | 2 | 0 | 0.08 | 1 |
| GO:0007521 | muscle cell fate determination | 2 | 0 | 0.08 | 1 |
| GO:0007522 | visceral muscle development | 1 | 0 | 0.04 | 1 |
| GO:0007525 | somatic muscle development | 4 | 0 | 0.15 | 1 |
| GO:0007527 | adult somatic muscle development | 1 | 0 | 0.04 | 1 |
| GO:0007528 | neuromuscular junction development | 44 | 0 | 1.67 | 1 |
| GO:0007529 | establishment of synaptic specificity at... | 3 | 0 | 0.11 | 1 |
| GO:0007530 | sex determination | 22 | 0 | 0.84 | 1 |

| | | | | | |
|---|---|---|---|---|---|
| GO:0007538 | primary sex determination | 3 | 0 | 0.11 | 1 |
| GO:0007542 | primary sex determination | germ-line | 2 | 0 | 0.08 |
| GO:0007549 | dosage compensation | 6 | 0 | 0.23 | 1 |
| GO:0007571 | age-dependent general metabolic decline | 3 | 0 | 0.11 | 1 |
| GO:0007597 | blood coagulation | intrinsic pat | 17 | 0 | 0.65 |
| GO:0007598 | blood coagulation | extrinsic pa | 5 | 0 | 0.19 |
| GO:0007614 | short-term memory | 8 | 0 | 0.3 | 1 |
| GO:0007616 | long-term memory | 28 | 0 | 1.07 | 1 |
| GO:0007619 | courtship behavior | 3 | 0 | 0.11 | 1 |
| GO:0007620 | copulation | 23 | 0 | 0.88 | 1 |
| GO:0007621 | negative regulation of female receptivit... | 4 | 0 | 0.15 | 1 |
| GO:0007624 | ultradian rhythm | 1 | 0 | 0.04 | 1 |
| GO:0007634 | optokinetic behavior | 1 | 0 | 0.04 | 1 |
| GO:0008039 | synaptic target recognition | 1 | 0 | 0.04 | 1 |
| GO:0008049 | male courtship behavior | 1 | 0 | 0.04 | 1 |
| GO:0008050 | female courtship behavior | 2 | 0 | 0.08 | 1 |
| GO:0008052 | sensory organ boundary specification | 1 | 0 | 0.04 | 1 |
| GO:0008057 | eye pigment granule organization | 1 | 0 | 0.04 | 1 |
| GO:0008065 | establishment of blood-nerve barrier | 3 | 0 | 0.11 | 1 |
| GO:0008078 | mesodermal cell migration | 5 | 0 | 0.19 | 1 |
| GO:0008090 | retrograde axon cargo transport | 5 | 0 | 0.19 | 1 |
| GO:0008209 | androgen metabolic process | 31 | 0 | 1.18 | 1 |
| GO:0008210 | estrogen metabolic process | 20 | 0 | 0.76 | 1 |
| GO:0008211 | glucocorticoid metabolic process | 24 | 0 | 0.91 | 1 |
| GO:0008214 | protein dealkylation | 26 | 0 | 0.99 | 1 |
| GO:0008215 | spermine metabolic process | 5 | 0 | 0.19 | 1 |
| GO:0008216 | spermidine metabolic process | 7 | 0 | 0.27 | 1 |
| GO:0008228 | opsonization | 9 | 0 | 0.34 | 1 |
| GO:0008291 | acetylcholine metabolic process | 4 | 0 | 0.15 | 1 |
| GO:0008292 | acetylcholine biosynthetic process | 2 | 0 | 0.08 | 1 |
| GO:0008295 | spermidine biosynthetic process | 4 | 0 | 0.15 | 1 |
| GO:0008298 | intracellular mRNA localization | 8 | 0 | 0.3 | 1 |
| GO:0008300 | isoprenoid catabolic process | 7 | 0 | 0.27 | 1 |
| GO:0008340 | determination of adult lifespan | 11 | 0 | 0.42 | 1 |
| GO:0008355 | olfactory learning | 2 | 0 | 0.08 | 1 |
| GO:0008356 | asymmetric cell division | 9 | 0 | 0.34 | 1 |
| GO:0008358 | maternal determination of anterior/poste... | 2 | 0 | 0.08 | 1 |
| GO:0008535 | respiratory chain complex IV assembly | 9 | 0 | 0.34 | 1 |
| GO:0008582 | regulation of synaptic growth at neuromu... | 4 | 0 | 0.15 | 1 |
| GO:0008588 | release of cytoplasmic sequestered NF-ka | 5 | 0 | 0.19 | 1 |
| GO:0008592 | regulation of Toll signaling pathway | 2 | 0 | 0.08 | 1 |
| GO:0008594 | photoreceptor cell morphogenesis | 2 | 0 | 0.08 | 1 |
| GO:0008595 | anterior/posterior axis specification | e... | 14 | 0 | 0.53 |
| GO:0008608 | attachment of spindle microtubules to ki... | 21 | 0 | 0.8 | 1 |
| GO:0008611 | ether lipid biosynthetic process | 5 | 0 | 0.19 | 1 |
| GO:0008612 | peptidyl-lysine modification to peptidyl... | 4 | 0 | 0.15 | 1 |
| GO:0008614 | pyridoxine metabolic process | 2 | 0 | 0.08 | 1 |
| GO:0008615 | pyridoxine biosynthetic process | 2 | 0 | 0.08 | 1 |
| GO:0008616 | queuosine biosynthetic process | 2 | 0 | 0.08 | 1 |
| GO:0008617 | guanosine metabolic process | 1 | 0 | 0.04 | 1 |
| GO:0008626 | granzyme-mediated apoptotic signaling pa | 5 | 0 | 0.19 | 1 |
| GO:0008627 | intrinsic apoptotic signaling pathway in... | 4 | 0 | 0.15 | 1 |
| GO:0008628 | hormone-mediated apoptotic signaling pat | 5 | 0 | 0.19 | 1 |

| | | | | | | |
|---|---|---|---|---|---|---|
| GO:0008635 | activation of cysteine-type endopeptidas... | | 8 | 0 | 0.3 | 1 |
| GO:0008653 | lipopolysaccharide metabolic process | | 2 | 0 | 0.08 | 1 |
| GO:0009048 | dosage compensation by inactivation of X. | | 4 | 0 | 0.15 | 1 |
| GO:0009051 | pentose-phosphate shunt | oxidative br | 3 | 0 | 0.11 | |
| GO:0009052 | pentose-phosphate shunt | non-oxidati | 4 | 0 | 0.15 | |
| GO:0009068 | aspartate family amino acid catabolic pr... | | 17 | 0 | 0.65 | 1 |
| GO:0009070 | serine family amino acid biosynthetic pr... | | 14 | 0 | 0.53 | 1 |
| GO:0009078 | pyruvate family amino acid metabolic pro.. | | 5 | 0 | 0.19 | 1 |
| GO:0009080 | pyruvate family amino acid catabolic pro... | | 5 | 0 | 0.19 | 1 |
| GO:0009081 | branched-chain amino acid metabolic proc | | 23 | 0 | 0.88 | 1 |
| GO:0009082 | branched-chain amino acid biosynthetic p. | | 2 | 0 | 0.08 | 1 |
| GO:0009083 | branched-chain amino acid catabolic proc. | | 19 | 0 | 0.72 | 1 |
| GO:0009092 | homoserine metabolic process | | 2 | 0 | 0.08 | 1 |
| GO:0009093 | cysteine catabolic process | | 3 | 0 | 0.11 | 1 |
| GO:0009103 | lipopolysaccharide biosynthetic process | | 2 | 0 | 0.08 | 1 |
| GO:0009106 | lipoate metabolic process | | 1 | 0 | 0.04 | 1 |
| GO:0009107 | lipoate biosynthetic process | | 1 | 0 | 0.04 | 1 |
| GO:0009109 | coenzyme catabolic process | | 9 | 0 | 0.34 | 1 |
| GO:0009111 | vitamin catabolic process | | 8 | 0 | 0.3 | 1 |
| GO:0009113 | purine nucleobase biosynthetic process | | 10 | 0 | 0.38 | 1 |
| GO:0009115 | xanthine catabolic process | | 1 | 0 | 0.04 | 1 |
| GO:0009118 | regulation of nucleoside metabolic proce... | | 13 | 0 | 0.49 | 1 |
| GO:0009120 | deoxyribonucleoside metabolic process | | 7 | 0 | 0.27 | 1 |
| GO:0009125 | nucleoside monophosphate catabolic proc | | 6 | 0 | 0.23 | 1 |
| GO:0009128 | purine nucleoside monophosphate catabol | | 5 | 0 | 0.19 | 1 |
| GO:0009129 | pyrimidine nucleoside monophosphate me | | 14 | 0 | 0.53 | 1 |
| GO:0009130 | pyrimidine nucleoside monophosphate bio | | 13 | 0 | 0.49 | 1 |
| GO:0009131 | pyrimidine nucleoside monophosphate cat | | 1 | 0 | 0.04 | 1 |
| GO:0009134 | nucleoside diphosphate catabolic process | | 8 | 0 | 0.3 | 1 |
| GO:0009135 | purine nucleoside diphosphate metabolic . | | 12 | 0 | 0.46 | 1 |
| GO:0009136 | purine nucleoside diphosphate biosynthet. | | 5 | 0 | 0.19 | 1 |
| GO:0009137 | purine nucleoside diphosphate catabolic ... | | 5 | 0 | 0.19 | 1 |
| GO:0009140 | pyrimidine nucleoside diphosphate catabo. | | 1 | 0 | 0.04 | 1 |
| GO:0009143 | nucleoside triphosphate catabolic proces... | | 11 | 0 | 0.42 | 1 |
| GO:0009146 | purine nucleoside triphosphate catabolic... | | 6 | 0 | 0.23 | 1 |
| GO:0009149 | pyrimidine nucleoside triphosphate catab.. | | 1 | 0 | 0.04 | 1 |
| GO:0009151 | purine deoxyribonucleotide metabolic pro.. | | 15 | 0 | 0.57 | 1 |
| GO:0009153 | purine deoxyribonucleotide biosynthetic ... | | 3 | 0 | 0.11 | 1 |
| GO:0009155 | purine deoxyribonucleotide catabolic pro... | | 9 | 0 | 0.34 | 1 |
| GO:0009158 | ribonucleoside monophosphate catabolic p | | 4 | 0 | 0.15 | 1 |
| GO:0009159 | deoxyribonucleoside monophosphate cata | | 1 | 0 | 0.04 | 1 |
| GO:0009164 | nucleoside catabolic process | | 33 | 0 | 1.26 | 1 |
| GO:0009169 | purine ribonucleoside monophosphate cat | | 4 | 0 | 0.15 | 1 |
| GO:0009170 | purine deoxyribonucleoside monophospha | | 1 | 0 | 0.04 | 1 |
| GO:0009173 | pyrimidine ribonucleoside monophosphate | | 11 | 0 | 0.42 | 1 |
| GO:0009174 | pyrimidine ribonucleoside monophosphate | | 11 | 0 | 0.42 | 1 |
| GO:0009176 | pyrimidine deoxyribonucleoside monophos | | 3 | 0 | 0.11 | 1 |
| GO:0009177 | pyrimidine deoxyribonucleoside monophos | | 2 | 0 | 0.08 | 1 |
| GO:0009178 | pyrimidine deoxyribonucleoside monophos | | 1 | 0 | 0.04 | 1 |
| GO:0009179 | purine ribonucleoside diphosphate metabo | | 12 | 0 | 0.46 | 1 |
| GO:0009180 | purine ribonucleoside diphosphate biosyn. | | 5 | 0 | 0.19 | 1 |
| GO:0009181 | purine ribonucleoside diphosphate catabo. | | 5 | 0 | 0.19 | 1 |
| GO:0009182 | purine deoxyribonucleoside diphosphate m | | 3 | 0 | 0.11 | 1 |

| | | | | | |
|---|---|---|---|---|---|
| GO:0009183 | purine deoxyribonucleoside diphosphate b | 2 | 0 | 0.08 | 1 |
| GO:0009184 | purine deoxyribonucleoside diphosphate c | 1 | 0 | 0.04 | 1 |
| GO:0009185 | ribonucleoside diphosphate metabolic pro. | 15 | 0 | 0.57 | 1 |
| GO:0009188 | ribonucleoside diphosphate biosynthetic ... | 6 | 0 | 0.23 | 1 |
| GO:0009191 | ribonucleoside diphosphate catabolic pro.. | 7 | 0 | 0.27 | 1 |
| GO:0009192 | deoxyribonucleoside diphosphate cataboli. | 1 | 0 | 0.04 | 1 |
| GO:0009193 | pyrimidine ribonucleoside diphosphate me | 2 | 0 | 0.08 | 1 |
| GO:0009194 | pyrimidine ribonucleoside diphosphate bi.. | 1 | 0 | 0.04 | 1 |
| GO:0009195 | pyrimidine ribonucleoside diphosphate ca.. | 1 | 0 | 0.04 | 1 |
| GO:0009203 | ribonucleoside triphosphate catabolic pr... | 1 | 0 | 0.04 | 1 |
| GO:0009204 | deoxyribonucleoside triphosphate catabol. | 7 | 0 | 0.27 | 1 |
| GO:0009207 | purine ribonucleoside triphosphate catab... | 1 | 0 | 0.04 | 1 |
| GO:0009208 | pyrimidine ribonucleoside triphosphate m.. | 14 | 0 | 0.53 | 1 |
| GO:0009209 | pyrimidine ribonucleoside triphosphate b... | 13 | 0 | 0.49 | 1 |
| GO:0009213 | pyrimidine deoxyribonucleoside triphosph.. | 1 | 0 | 0.04 | 1 |
| GO:0009215 | purine deoxyribonucleoside triphosphate .. | 10 | 0 | 0.38 | 1 |
| GO:0009216 | purine deoxyribonucleoside triphosphate .. | 1 | 0 | 0.04 | 1 |
| GO:0009217 | purine deoxyribonucleoside triphosphate .. | 5 | 0 | 0.19 | 1 |
| GO:0009218 | pyrimidine ribonucleotide metabolic proc... | 23 | 0 | 0.88 | 1 |
| GO:0009220 | pyrimidine ribonucleotide biosynthetic p... | 21 | 0 | 0.8 | 1 |
| GO:0009222 | pyrimidine ribonucleotide catabolic proc... | 1 | 0 | 0.04 | 1 |
| GO:0009224 | CMP biosynthetic process | 1 | 0 | 0.04 | 1 |
| GO:0009227 | nucleotide-sugar catabolic process | 3 | 0 | 0.11 | 1 |
| GO:0009229 | thiamine diphosphate biosynthetic proces.. | 1 | 0 | 0.04 | 1 |
| GO:0009233 | menaquinone metabolic process | 3 | 0 | 0.11 | 1 |
| GO:0009234 | menaquinone biosynthetic process | 1 | 0 | 0.04 | 1 |
| GO:0009235 | cobalamin metabolic process | 20 | 0 | 0.76 | 1 |
| GO:0009236 | cobalamin biosynthetic process | 3 | 0 | 0.11 | 1 |
| GO:0009240 | isopentenyl diphosphate biosynthetic pro... | 3 | 0 | 0.11 | 1 |
| GO:0009249 | protein lipoylation | 4 | 0 | 0.15 | 1 |
| GO:0009253 | peptidoglycan catabolic process | 7 | 0 | 0.27 | 1 |
| GO:0009256 | 10-formyltetrahydrofolate metabolic proc... | 2 | 0 | 0.08 | 1 |
| GO:0009258 | 10-formyltetrahydrofolate catabolic proc... | 2 | 0 | 0.08 | 1 |
| GO:0009298 | GDP-mannose biosynthetic process | 5 | 0 | 0.19 | 1 |
| GO:0009305 | protein biotinylation | 1 | 0 | 0.04 | 1 |
| GO:0009309 | amine biosynthetic process | 20 | 0 | 0.76 | 1 |
| GO:0009310 | amine catabolic process | 17 | 0 | 0.65 | 1 |
| GO:0009312 | oligosaccharide biosynthetic process | 13 | 0 | 0.49 | 1 |
| GO:0009313 | oligosaccharide catabolic process | 8 | 0 | 0.3 | 1 |
| GO:0009372 | quorum sensing | 1 | 0 | 0.04 | 1 |
| GO:0009386 | translational attenuation | 2 | 0 | 0.08 | 1 |
| GO:0009395 | phospholipid catabolic process | 30 | 0 | 1.14 | 1 |
| GO:0009396 | folic acid-containing compound biosynthe.. | 10 | 0 | 0.38 | 1 |
| GO:0009397 | folic acid-containing compound catabolic... | 2 | 0 | 0.08 | 1 |
| GO:0009399 | nitrogen fixation | 1 | 0 | 0.04 | 1 |
| GO:0009403 | toxin biosynthetic process | 1 | 0 | 0.04 | 1 |
| GO:0009404 | toxin metabolic process | 12 | 0 | 0.46 | 1 |
| GO:0009436 | glyoxylate catabolic process | 2 | 0 | 0.08 | 1 |
| GO:0009437 | carnitine metabolic process | 12 | 0 | 0.46 | 1 |
| GO:0009438 | methylglyoxal metabolic process | 3 | 0 | 0.11 | 1 |
| GO:0009439 | cyanate metabolic process | 2 | 0 | 0.08 | 1 |
| GO:0009440 | cyanate catabolic process | 2 | 0 | 0.08 | 1 |
| GO:0009441 | glycolate metabolic process | 3 | 0 | 0.11 | 1 |

| | | | | | |
|---|---|---|---|---|---|
| GO:0009443 | pyridoxal 5'-phosphate salvage | 1 | 0 | 0.04 | 1 |
| GO:0009445 | putrescine metabolic process | 5 | 0 | 0.19 | 1 |
| GO:0009446 | putrescine biosynthetic process | 3 | 0 | 0.11 | 1 |
| GO:0009447 | putrescine catabolic process | 3 | 0 | 0.11 | 1 |
| GO:0009448 | gamma-aminobutyric acid metabolic proce | 5 | 0 | 0.19 | 1 |
| GO:0009449 | gamma-aminobutyric acid biosynthetic pro | 2 | 0 | 0.08 | 1 |
| GO:0009450 | gamma-aminobutyric acid catabolic proces | 2 | 0 | 0.08 | 1 |
| GO:0009452 | 7-methylguanosine RNA capping | 32 | 0 | 1.22 | 1 |
| GO:0009585 | red | far-red light | 1 | 0 | 0.04 |
| GO:0009589 | detection of UV | 1 | 0 | 0.04 | 1 |
| GO:0009590 | detection of gravity | 2 | 0 | 0.08 | 1 |
| GO:0009597 | detection of virus | 3 | 0 | 0.11 | 1 |
| GO:0009608 | response to symbiont | 2 | 0 | 0.08 | 1 |
| GO:0009609 | response to symbiotic bacterium | 2 | 0 | 0.08 | 1 |
| GO:0009629 | response to gravity | 9 | 0 | 0.34 | 1 |
| GO:0009631 | cold acclimation | 1 | 0 | 0.04 | 1 |
| GO:0009635 | response to herbicide | 8 | 0 | 0.3 | 1 |
| GO:0009637 | response to blue light | 3 | 0 | 0.11 | 1 |
| GO:0009639 | response to red or far red light | 1 | 0 | 0.04 | 1 |
| GO:0009642 | response to light intensity | 9 | 0 | 0.34 | 1 |
| GO:0009644 | response to high light intensity | 1 | 0 | 0.04 | 1 |
| GO:0009645 | response to low light intensity stimulus | 2 | 0 | 0.08 | 1 |
| GO:0009648 | photoperiodism | 22 | 0 | 0.84 | 1 |
| GO:0009649 | entrainment of circadian clock | 22 | 0 | 0.84 | 1 |
| GO:0009650 | UV protection | 12 | 0 | 0.46 | 1 |
| GO:0009698 | phenylpropanoid metabolic process | 9 | 0 | 0.34 | 1 |
| GO:0009712 | catechol-containing compound metabolic p | 48 | 0 | 1.83 | 1 |
| GO:0009713 | catechol-containing compound biosyntheti | 19 | 0 | 0.72 | 1 |
| GO:0009720 | detection of hormone stimulus | 1 | 0 | 0.04 | 1 |
| GO:0009726 | detection of endogenous stimulus | 3 | 0 | 0.11 | 1 |
| GO:0009730 | detection of carbohydrate stimulus | 4 | 0 | 0.15 | 1 |
| GO:0009732 | detection of hexose stimulus | 4 | 0 | 0.15 | 1 |
| GO:0009737 | response to abscisic acid | 1 | 0 | 0.04 | 1 |
| GO:0009738 | abscisic acid-activated signaling pathwa... | 1 | 0 | 0.04 | 1 |
| GO:0009744 | response to sucrose | 6 | 0 | 0.23 | 1 |
| GO:0009750 | response to fructose | 6 | 0 | 0.23 | 1 |
| GO:0009753 | response to jasmonic acid | 4 | 0 | 0.15 | 1 |
| GO:0009756 | carbohydrate mediated signaling | 3 | 0 | 0.11 | 1 |
| GO:0009757 | hexose mediated signaling | 1 | 0 | 0.04 | 1 |
| GO:0009785 | blue light signaling pathway | 2 | 0 | 0.08 | 1 |
| GO:0009786 | regulation of asymmetric cell division | 4 | 0 | 0.15 | 1 |
| GO:0009787 | regulation of abscisic acid-activated si... | 1 | 0 | 0.04 | 1 |
| GO:0009789 | positive regulation of abscisic acid-act... | 1 | 0 | 0.04 | 1 |
| GO:0009794 | regulation of mitotic cell cycle | embryo... | 5 | 0 | 0.19 |
| GO:0009804 | coumarin metabolic process | 8 | 0 | 0.3 | 1 |
| GO:0009810 | stilbene metabolic process | 1 | 0 | 0.04 | 1 |
| GO:0009814 | defense response | incompatibl | 1 | 0 | 0.04 |
| GO:0009817 | defense response to fungus | incompatibl | 1 | 0 | 0.04 |
| GO:0009820 | alkaloid metabolic process | 10 | 0 | 0.38 | 1 |
| GO:0009822 | alkaloid catabolic process | 3 | 0 | 0.11 | 1 |
| GO:0009826 | unidimensional cell growth | 3 | 0 | 0.11 | 1 |
| GO:0009838 | abscission | 5 | 0 | 0.19 | 1 |
| GO:0009886 | post-embryonic morphogenesis | 10 | 0 | 0.38 | 1 |

| | | | | | |
|---|---|---|---|---|---|
| GO:0009912 | auditory receptor cell fate commitment | 6 | 0 | 0.23 | 1 |
| GO:0009946 | proximal/distal axis specification | 3 | 0 | 0.11 | 1 |
| GO:0009949 | polarity specification of anterior/poste... | 1 | 0 | 0.04 | 1 |
| GO:0009954 | proximal/distal pattern formation | 33 | 0 | 1.26 | 1 |
| GO:0009957 | epidermal cell fate specification | 1 | 0 | 0.04 | 1 |
| GO:0009972 | cytidine deamination | 9 | 0 | 0.34 | 1 |
| GO:0009994 | oocyte differentiation | 37 | 0 | 1.41 | 1 |
| GO:0009996 | negative regulation of cell fate specifi... | 7 | 0 | 0.27 | 1 |
| GO:0009997 | negative regulation of cardioblast cell ... | 1 | 0 | 0.04 | 1 |
| GO:0010002 | cardioblast differentiation | 19 | 0 | 0.72 | 1 |
| GO:0010015 | root morphogenesis | 1 | 0 | 0.04 | 1 |
| GO:0010017 | red or far-red light signaling pathway | 1 | 0 | 0.04 | 1 |
| GO:0010021 | amylopectin biosynthetic process | 1 | 0 | 0.04 | 1 |
| GO:0010025 | wax biosynthetic process | 2 | 0 | 0.08 | 1 |
| GO:0010032 | meiotic chromosome condensation | 3 | 0 | 0.11 | 1 |
| GO:0010034 | response to acetate | 1 | 0 | 0.04 | 1 |
| GO:0010040 | response to iron(II) ion | 4 | 0 | 0.15 | 1 |
| GO:0010041 | response to iron(III) ion | 3 | 0 | 0.11 | 1 |
| GO:0010042 | response to manganese ion | 12 | 0 | 0.46 | 1 |
| GO:0010044 | response to aluminum ion | 4 | 0 | 0.15 | 1 |
| GO:0010045 | response to nickel cation | 1 | 0 | 0.04 | 1 |
| GO:0010046 | response to mycotoxin | 2 | 0 | 0.08 | 1 |
| GO:0010053 | root epidermal cell differentiation | 1 | 0 | 0.04 | 1 |
| GO:0010054 | trichoblast differentiation | 1 | 0 | 0.04 | 1 |
| GO:0010070 | zygote asymmetric cell division | 2 | 0 | 0.08 | 1 |
| GO:0010085 | polarity specification of proximal/dista... | 1 | 0 | 0.04 | 1 |
| GO:0010092 | specification of organ identity | 34 | 0 | 1.29 | 1 |
| GO:0010106 | cellular response to iron ion starvation | 1 | 0 | 0.04 | 1 |
| GO:0010124 | phenylacetate catabolic process | 1 | 0 | 0.04 | 1 |
| GO:0010133 | proline catabolic process to glutamate | 3 | 0 | 0.11 | 1 |
| GO:0010138 | pyrimidine ribonucleotide salvage | 5 | 0 | 0.19 | 1 |
| GO:0010157 | response to chlorate | 2 | 0 | 0.08 | 1 |
| GO:0010159 | specification of organ position | 3 | 0 | 0.11 | 1 |
| GO:0010160 | formation of organ boundary | 1 | 0 | 0.04 | 1 |
| GO:0010166 | wax metabolic process | 2 | 0 | 0.08 | 1 |
| GO:0010182 | sugar mediated signaling pathway | 1 | 0 | 0.04 | 1 |
| GO:0010189 | vitamin E biosynthetic process | 1 | 0 | 0.04 | 1 |
| GO:0010216 | maintenance of DNA methylation | 5 | 0 | 0.19 | 1 |
| GO:0010224 | response to UV-B | 17 | 0 | 0.65 | 1 |
| GO:0010226 | response to lithium ion | 26 | 0 | 0.99 | 1 |
| GO:0010232 | vascular transport | 1 | 0 | 0.04 | 1 |
| GO:0010248 | establishment or maintenance of transmem | 3 | 0 | 0.11 | 1 |
| GO:0010255 | glucose mediated signaling pathway | 1 | 0 | 0.04 | 1 |
| GO:0010257 | NADH dehydrogenase complex assembly | 11 | 0 | 0.42 | 1 |
| GO:0010260 | organ senescence | 2 | 0 | 0.08 | 1 |
| GO:0010269 | response to selenium ion | 6 | 0 | 0.23 | 1 |
| GO:0010273 | detoxification of copper ion | 3 | 0 | 0.11 | 1 |
| GO:0010310 | regulation of hydrogen peroxide metaboli.. | 11 | 0 | 0.42 | 1 |
| GO:0010359 | regulation of anion channel activity | 2 | 0 | 0.08 | 1 |
| GO:0010360 | negative regulation of anion channel act... | 1 | 0 | 0.04 | 1 |
| GO:0010383 | cell wall polysaccharide metabolic proce... | 1 | 0 | 0.04 | 1 |
| GO:0010387 | COP9 signalosome assembly | 1 | 0 | 0.04 | 1 |
| GO:0010388 | cullin deneddylation | 9 | 0 | 0.34 | 1 |

| | | | | | |
|---|---|---|---|---|---|
| GO:0010390 | histone monoubiquitination | 23 | 0 | 0.88 | 1 |
| GO:0010412 | mannan metabolic process | 1 | 0 | 0.04 | 1 |
| GO:0010424 | DNA methylation on cytosine within a CG . | 3 | 0 | 0.11 | 1 |
| GO:0010430 | fatty acid omega-oxidation | 2 | 0 | 0.08 | 1 |
| GO:0010446 | response to alkaline pH | 3 | 0 | 0.11 | 1 |
| GO:0010452 | histone H3-K36 methylation | 9 | 0 | 0.34 | 1 |
| GO:0010454 | negative regulation of cell fate commitm... | 11 | 0 | 0.42 | 1 |
| GO:0010455 | positive regulation of cell fate commitm... | 13 | 0 | 0.49 | 1 |
| GO:0010457 | centriole-centriole cohesion | 8 | 0 | 0.3 | 1 |
| GO:0010458 | exit from mitosis | 17 | 0 | 0.65 | 1 |
| GO:0010459 | negative regulation of heart rate | 9 | 0 | 0.34 | 1 |
| GO:0010477 | response to sulfur dioxide | 1 | 0 | 0.04 | 1 |
| GO:0010481 | epidermal cell division | 2 | 0 | 0.08 | 1 |
| GO:0010482 | regulation of epidermal cell division | 2 | 0 | 0.08 | 1 |
| GO:0010499 | proteasomal ubiquitin-independent protei.. | 4 | 0 | 0.15 | 1 |
| GO:0010501 | RNA secondary structure unwinding | 2 | 0 | 0.08 | 1 |
| GO:0010509 | polyamine homeostasis | 1 | 0 | 0.04 | 1 |
| GO:0010511 | regulation of phosphatidylinositol biosy... | 6 | 0 | 0.23 | 1 |
| GO:0010512 | negative regulation of phosphatidylinosi... | 2 | 0 | 0.08 | 1 |
| GO:0010513 | positive regulation of phosphatidylinosi... | 4 | 0 | 0.15 | 1 |
| GO:0010519 | negative regulation of phospholipase act... | 7 | 0 | 0.27 | 1 |
| GO:0010523 | negative regulation of calcium ion trans... | 7 | 0 | 0.27 | 1 |
| GO:0010524 | positive regulation of calcium ion trans... | 37 | 0 | 1.41 | 1 |
| GO:0010528 | regulation of transposition | 9 | 0 | 0.34 | 1 |
| GO:0010529 | negative regulation of transposition | 9 | 0 | 0.34 | 1 |
| GO:0010533 | regulation of activation of Janus kinase... | 8 | 0 | 0.3 | 1 |
| GO:0010534 | regulation of activation of JAK2 kinase ... | 7 | 0 | 0.27 | 1 |
| GO:0010535 | positive regulation of activation of JAK... | 6 | 0 | 0.23 | 1 |
| GO:0010536 | positive regulation of activation of Jan... | 7 | 0 | 0.27 | 1 |
| GO:0010560 | positive regulation of glycoprotein bios... | 10 | 0 | 0.38 | 1 |
| GO:0010571 | positive regulation of nuclear cell cycl... | 5 | 0 | 0.19 | 1 |
| GO:0010572 | positive regulation of platelet activati... | 5 | 0 | 0.19 | 1 |
| GO:0010578 | regulation of adenylate cyclase activity... | 16 | 0 | 0.61 | 1 |
| GO:0010579 | positive regulation of adenylate cyclase... | 16 | 0 | 0.61 | 1 |
| GO:0010593 | negative regulation of lamellipodium ass... | 3 | 0 | 0.11 | 1 |
| GO:0010609 | mRNA localization resulting in posttrans... | 2 | 0 | 0.08 | 1 |
| GO:0010610 | regulation of mRNA stability involved in... | 1 | 0 | 0.04 | 1 |
| GO:0010612 | regulation of cardiac muscle adaptation | 5 | 0 | 0.19 | 1 |
| GO:0010614 | negative regulation of cardiac muscle hy... | 9 | 0 | 0.34 | 1 |
| GO:0010615 | positive regulation of cardiac muscle ad... | 2 | 0 | 0.08 | 1 |
| GO:0010616 | negative regulation of cardiac muscle ad... | 2 | 0 | 0.08 | 1 |
| GO:0010621 | negative regulation of transcription by ... | 3 | 0 | 0.11 | 1 |
| GO:0010624 | regulation of Schwann cell proliferation | 5 | 0 | 0.19 | 1 |
| GO:0010625 | positive regulation of Schwann cell prol... | 1 | 0 | 0.04 | 1 |
| GO:0010626 | negative regulation of Schwann cell prol... | 4 | 0 | 0.15 | 1 |
| GO:0010635 | regulation of mitochondrial fusion | 6 | 0 | 0.23 | 1 |
| GO:0010636 | positive regulation of mitochondrial fus... | 1 | 0 | 0.04 | 1 |
| GO:0010637 | negative regulation of mitochondrial fus... | 5 | 0 | 0.19 | 1 |
| GO:0010641 | positive regulation of platelet-derived ... | 3 | 0 | 0.11 | 1 |
| GO:0010643 | cell communication by chemical coupling | 3 | 0 | 0.11 | 1 |
| GO:0010645 | regulation of cell communication by chem.. | 2 | 0 | 0.08 | 1 |
| GO:0010650 | positive regulation of cell communicatio... | 2 | 0 | 0.08 | 1 |
| GO:0010651 | negative regulation of cell communicatio... | 1 | 0 | 0.04 | 1 |

| | | | | | |
|---|---|---|---|---|---|
| GO:0010652 | positive regulation of cell communicatio... | 2 | 0 | 0.08 | 1 |
| GO:0010658 | striated muscle cell apoptotic process | 19 | 0 | 0.72 | 1 |
| GO:0010659 | cardiac muscle cell apoptotic process | 16 | 0 | 0.61 | 1 |
| GO:0010661 | positive regulation of muscle cell apopt... | 9 | 0 | 0.34 | 1 |
| GO:0010662 | regulation of striated muscle cell apopt... | 16 | 0 | 0.61 | 1 |
| GO:0010663 | positive regulation of striated muscle c... | 5 | 0 | 0.19 | 1 |
| GO:0010664 | negative regulation of striated muscle c... | 12 | 0 | 0.46 | 1 |
| GO:0010665 | regulation of cardiac muscle cell apopto... | 13 | 0 | 0.49 | 1 |
| GO:0010666 | positive regulation of cardiac muscle ce... | 5 | 0 | 0.19 | 1 |
| GO:0010667 | negative regulation of cardiac muscle ce... | 8 | 0 | 0.3 | 1 |
| GO:0010668 | ectodermal cell differentiation | 6 | 0 | 0.23 | 1 |
| GO:0010692 | regulation of alkaline phosphatase activ... | 10 | 0 | 0.38 | 1 |
| GO:0010693 | negative regulation of alkaline phosphat... | 4 | 0 | 0.15 | 1 |
| GO:0010694 | positive regulation of alkaline phosphat... | 6 | 0 | 0.23 | 1 |
| GO:0010700 | negative regulation of norepinephrine se... | 8 | 0 | 0.3 | 1 |
| GO:0010701 | positive regulation of norepinephrine se... | 2 | 0 | 0.08 | 1 |
| GO:0010710 | regulation of collagen catabolic process | 3 | 0 | 0.11 | 1 |
| GO:0010711 | negative regulation of collagen cataboli... | 1 | 0 | 0.04 | 1 |
| GO:0010713 | negative regulation of collagen metaboli... | 6 | 0 | 0.23 | 1 |
| GO:0010715 | regulation of extracellular matrix disas... | 9 | 0 | 0.34 | 1 |
| GO:0010716 | negative regulation of extracellular mat... | 4 | 0 | 0.15 | 1 |
| GO:0010718 | positive regulation of epithelial to mes... | 28 | 0 | 1.07 | 1 |
| GO:0010722 | regulation of ferrochelatase activity | 1 | 0 | 0.04 | 1 |
| GO:0010724 | regulation of definitive erythrocyte dif... | 3 | 0 | 0.11 | 1 |
| GO:0010726 | positive regulation of hydrogen peroxide... | 2 | 0 | 0.08 | 1 |
| GO:0010727 | negative regulation of hydrogen peroxide.. | 4 | 0 | 0.15 | 1 |
| GO:0010728 | regulation of hydrogen peroxide biosynth... | 2 | 0 | 0.08 | 1 |
| GO:0010729 | positive regulation of hydrogen peroxide... | 1 | 0 | 0.04 | 1 |
| GO:0010730 | negative regulation of hydrogen peroxide.. | 1 | 0 | 0.04 | 1 |
| GO:0010731 | protein glutathionylation | 1 | 0 | 0.04 | 1 |
| GO:0010732 | regulation of protein glutathionylation | 1 | 0 | 0.04 | 1 |
| GO:0010734 | negative regulation of protein glutathio... | 1 | 0 | 0.04 | 1 |
| GO:0010735 | positive regulation of transcription via... | 4 | 0 | 0.15 | 1 |
| GO:0010739 | positive regulation of protein kinase A ... | 5 | 0 | 0.19 | 1 |
| GO:0010742 | macrophage derived foam cell differentia... | 31 | 0 | 1.18 | 1 |
| GO:0010743 | regulation of macrophage derived foam ce | 28 | 0 | 1.07 | 1 |
| GO:0010744 | positive regulation of macrophage derive... | 15 | 0 | 0.57 | 1 |
| GO:0010745 | negative regulation of macrophage derive. | 13 | 0 | 0.49 | 1 |
| GO:0010746 | regulation of plasma membrane long-chair | 5 | 0 | 0.19 | 1 |
| GO:0010747 | positive regulation of plasma membrane l.. | 1 | 0 | 0.04 | 1 |
| GO:0010748 | negative regulation of plasma membrane l | 4 | 0 | 0.15 | 1 |
| GO:0010749 | regulation of nitric oxide mediated sign... | 4 | 0 | 0.15 | 1 |
| GO:0010750 | positive regulation of nitric oxide medi... | 1 | 0 | 0.04 | 1 |
| GO:0010751 | negative regulation of nitric oxide medi... | 3 | 0 | 0.11 | 1 |
| GO:0010752 | regulation of cGMP-mediated signaling | 2 | 0 | 0.08 | 1 |
| GO:0010754 | negative regulation of cGMP-mediated sig | 1 | 0 | 0.04 | 1 |
| GO:0010755 | regulation of plasminogen activation | 9 | 0 | 0.34 | 1 |
| GO:0010756 | positive regulation of plasminogen activ... | 4 | 0 | 0.15 | 1 |
| GO:0010757 | negative regulation of plasminogen activ... | 5 | 0 | 0.19 | 1 |
| GO:0010760 | negative regulation of macrophage chemo | 2 | 0 | 0.08 | 1 |
| GO:0010761 | fibroblast migration | 24 | 0 | 0.91 | 1 |
| GO:0010762 | regulation of fibroblast migration | 16 | 0 | 0.61 | 1 |
| GO:0010763 | positive regulation of fibroblast migrat... | 7 | 0 | 0.27 | 1 |

| | | | | | |
|---|---|---|---|---|---|
| GO:0010764 | negative regulation of fibroblast migrat... | 6 | 0 | 0.23 | 1 |
| GO:0010766 | negative regulation of sodium ion transp... | 10 | 0 | 0.38 | 1 |
| GO:0010767 | regulation of transcription from RNA pol... | 2 | 0 | 0.08 | 1 |
| GO:0010768 | negative regulation of transcription fro... | 1 | 0 | 0.04 | 1 |
| GO:0010792 | DNA double-strand break processing invol | 2 | 0 | 0.08 | 1 |
| GO:0010793 | regulation of mRNA export from nucleus | 5 | 0 | 0.19 | 1 |
| GO:0010796 | regulation of multivesicular body size | 2 | 0 | 0.08 | 1 |
| GO:0010797 | regulation of multivesicular body size i... | 1 | 0 | 0.04 | 1 |
| GO:0010801 | negative regulation of peptidyl-threonin... | 12 | 0 | 0.46 | 1 |
| GO:0010807 | regulation of synaptic vesicle priming | 5 | 0 | 0.19 | 1 |
| GO:0010808 | positive regulation of synaptic vesicle ... | 1 | 0 | 0.04 | 1 |
| GO:0010813 | neuropeptide catabolic process | 2 | 0 | 0.08 | 1 |
| GO:0010814 | substance P catabolic process | 1 | 0 | 0.04 | 1 |
| GO:0010815 | bradykinin catabolic process | 5 | 0 | 0.19 | 1 |
| GO:0010816 | calcitonin catabolic process | 1 | 0 | 0.04 | 1 |
| GO:0010818 | T cell chemotaxis | 18 | 0 | 0.69 | 1 |
| GO:0010819 | regulation of T cell chemotaxis | 11 | 0 | 0.42 | 1 |
| GO:0010820 | positive regulation of T cell chemotaxis | 10 | 0 | 0.38 | 1 |
| GO:0010824 | regulation of centrosome duplication | 28 | 0 | 1.07 | 1 |
| GO:0010825 | positive regulation of centrosome duplic... | 3 | 0 | 0.11 | 1 |
| GO:0010826 | negative regulation of centrosome duplic... | 9 | 0 | 0.34 | 1 |
| GO:0010831 | positive regulation of myotube different... | 22 | 0 | 0.84 | 1 |
| GO:0010836 | negative regulation of protein ADP-ribos... | 1 | 0 | 0.04 | 1 |
| GO:0010838 | positive regulation of keratinocyte prol... | 6 | 0 | 0.23 | 1 |
| GO:0010839 | negative regulation of keratinocyte prol... | 11 | 0 | 0.42 | 1 |
| GO:0010840 | regulation of circadian sleep/wake cycle... | 4 | 0 | 0.15 | 1 |
| GO:0010841 | positive regulation of circadian sleep/w... | 4 | 0 | 0.15 | 1 |
| GO:0010847 | regulation of chromatin assembly | 2 | 0 | 0.08 | 1 |
| GO:0010848 | regulation of chromatin disassembly | 2 | 0 | 0.08 | 1 |
| GO:0010849 | regulation of proton-transporting ATPase... | 1 | 0 | 0.04 | 1 |
| GO:0010866 | regulation of triglyceride biosynthetic ... | 14 | 0 | 0.53 | 1 |
| GO:0010867 | positive regulation of triglyceride bios... | 9 | 0 | 0.34 | 1 |
| GO:0010868 | negative regulation of triglyceride bios... | 2 | 0 | 0.08 | 1 |
| GO:0010871 | negative regulation of receptor biosynth... | 6 | 0 | 0.23 | 1 |
| GO:0010872 | regulation of cholesterol esterification | 9 | 0 | 0.34 | 1 |
| GO:0010873 | positive regulation of cholesterol ester... | 8 | 0 | 0.3 | 1 |
| GO:0010874 | regulation of cholesterol efflux | 19 | 0 | 0.72 | 1 |
| GO:0010875 | positive regulation of cholesterol efflu... | 14 | 0 | 0.53 | 1 |
| GO:0010878 | cholesterol storage | 14 | 0 | 0.53 | 1 |
| GO:0010881 | regulation of cardiac muscle contraction... | 19 | 0 | 0.72 | 1 |
| GO:0010884 | positive regulation of lipid storage | 18 | 0 | 0.69 | 1 |
| GO:0010885 | regulation of cholesterol storage | 13 | 0 | 0.49 | 1 |
| GO:0010886 | positive regulation of cholesterol stora... | 7 | 0 | 0.27 | 1 |
| GO:0010887 | negative regulation of cholesterol stora... | 6 | 0 | 0.23 | 1 |
| GO:0010889 | regulation of sequestering of triglyceri... | 11 | 0 | 0.42 | 1 |
| GO:0010890 | positive regulation of sequestering of t... | 6 | 0 | 0.23 | 1 |
| GO:0010891 | negative regulation of sequestering of t... | 5 | 0 | 0.19 | 1 |
| GO:0010896 | regulation of triglyceride catabolic pro... | 11 | 0 | 0.42 | 1 |
| GO:0010897 | negative regulation of triglyceride cata... | 4 | 0 | 0.15 | 1 |
| GO:0010898 | positive regulation of triglyceride cata... | 7 | 0 | 0.27 | 1 |
| GO:0010899 | regulation of phosphatidylcholine catabo... | 3 | 0 | 0.11 | 1 |
| GO:0010900 | negative regulation of phosphatidylcholi... | 1 | 0 | 0.04 | 1 |
| GO:0010901 | regulation of very-low-density lipoprote... | 5 | 0 | 0.19 | 1 |

| | | | | | |
|---|---|---|---|---|---|
| GO:0010902 | positive regulation of very-low-density ... | 2 | 0 | 0.08 | 1 |
| GO:0010903 | negative regulation of very-low-density ... | 3 | 0 | 0.11 | 1 |
| GO:0010904 | regulation of UDP-glucose catabolic proc.. | 1 | 0 | 0.04 | 1 |
| GO:0010905 | negative regulation of UDP-glucose catab. | 1 | 0 | 0.04 | 1 |
| GO:0010908 | regulation of heparan sulfate proteoglyc... | 2 | 0 | 0.08 | 1 |
| GO:0010909 | positive regulation of heparan sulfate p... | 2 | 0 | 0.08 | 1 |
| GO:0010911 | regulation of isomerase activity | 4 | 0 | 0.15 | 1 |
| GO:0010912 | positive regulation of isomerase activit... | 4 | 0 | 0.15 | 1 |
| GO:0010915 | regulation of very-low-density lipoprote... | 4 | 0 | 0.15 | 1 |
| GO:0010916 | negative regulation of very-low-density ... | 4 | 0 | 0.15 | 1 |
| GO:0010917 | negative regulation of mitochondrial mem.. | 5 | 0 | 0.19 | 1 |
| GO:0010918 | positive regulation of mitochondrial mem... | 2 | 0 | 0.08 | 1 |
| GO:0010919 | regulation of inositol phosphate biosynt... | 10 | 0 | 0.38 | 1 |
| GO:0010920 | negative regulation of inositol phosphat... | 1 | 0 | 0.04 | 1 |
| GO:0010922 | positive regulation of phosphatase activ... | 24 | 0 | 0.91 | 1 |
| GO:0010924 | regulation of inositol-polyphosphate 5-p... | 1 | 0 | 0.04 | 1 |
| GO:0010925 | positive regulation of inositol-polyphos... | 1 | 0 | 0.04 | 1 |
| GO:0010931 | macrophage tolerance induction | 1 | 0 | 0.04 | 1 |
| GO:0010932 | regulation of macrophage tolerance induc. | 1 | 0 | 0.04 | 1 |
| GO:0010933 | positive regulation of macrophage tolera... | 1 | 0 | 0.04 | 1 |
| GO:0010934 | macrophage cytokine production | 13 | 0 | 0.49 | 1 |
| GO:0010935 | regulation of macrophage cytokine produc. | 12 | 0 | 0.46 | 1 |
| GO:0010936 | negative regulation of macrophage cytoki.. | 4 | 0 | 0.15 | 1 |
| GO:0010938 | cytoplasmic microtubule depolymerization | 1 | 0 | 0.04 | 1 |
| GO:0010939 | regulation of necrotic cell death | 16 | 0 | 0.61 | 1 |
| GO:0010940 | positive regulation of necrotic cell dea... | 5 | 0 | 0.19 | 1 |
| GO:0010949 | negative regulation of intestinal phytos... | 2 | 0 | 0.08 | 1 |
| GO:0010954 | positive regulation of protein processin... | 15 | 0 | 0.57 | 1 |
| GO:0010956 | negative regulation of calcidiol 1-monoo... | 3 | 0 | 0.11 | 1 |
| GO:0010957 | negative regulation of vitamin D biosynt... | 5 | 0 | 0.19 | 1 |
| GO:0010958 | regulation of amino acid import | 2 | 0 | 0.08 | 1 |
| GO:0010960 | magnesium ion homeostasis | 10 | 0 | 0.38 | 1 |
| GO:0010961 | cellular magnesium ion homeostasis | 4 | 0 | 0.15 | 1 |
| GO:0010963 | regulation of L-arginine import | 1 | 0 | 0.04 | 1 |
| GO:0010967 | regulation of polyamine biosynthetic pro... | 1 | 0 | 0.04 | 1 |
| GO:0010968 | regulation of microtubule nucleation | 1 | 0 | 0.04 | 1 |
| GO:0010979 | regulation of vitamin D 24-hydroxylase a... | 3 | 0 | 0.11 | 1 |
| GO:0010980 | positive regulation of vitamin D 24-hydr... | 3 | 0 | 0.11 | 1 |
| GO:0010982 | regulation of high-density lipoprotein p... | 3 | 0 | 0.11 | 1 |
| GO:0010983 | positive regulation of high-density lipo... | 2 | 0 | 0.08 | 1 |
| GO:0010984 | regulation of lipoprotein particle clear... | 13 | 0 | 0.49 | 1 |
| GO:0010985 | negative regulation of lipoprotein parti... | 7 | 0 | 0.27 | 1 |
| GO:0010986 | positive regulation of lipoprotein parti... | 3 | 0 | 0.11 | 1 |
| GO:0010987 | negative regulation of high-density lipo... | 1 | 0 | 0.04 | 1 |
| GO:0010988 | regulation of low-density lipoprotein pa... | 7 | 0 | 0.27 | 1 |
| GO:0010989 | negative regulation of low-density lipop... | 4 | 0 | 0.15 | 1 |
| GO:0010992 | ubiquitin homeostasis | 3 | 0 | 0.11 | 1 |
| GO:0010993 | regulation of ubiquitin homeostasis | 2 | 0 | 0.08 | 1 |
| GO:0010994 | free ubiquitin chain polymerization | 2 | 0 | 0.08 | 1 |
| GO:0010999 | regulation of eIF2 alpha phosphorylation... | 2 | 0 | 0.08 | 1 |
| GO:0012502 | induction of programmed cell death | 2 | 0 | 0.08 | 1 |
| GO:0014010 | Schwann cell proliferation | 7 | 0 | 0.27 | 1 |
| GO:0014012 | peripheral nervous system axon regenerat | 6 | 0 | 0.23 | 1 |

| | | | | | |
|---|---|---|---|---|---|
| GO:0014016 | neuroblast differentiation | 3 | 0 | 0.11 | 1 |
| GO:0014017 | neuroblast fate commitment | 1 | 0 | 0.04 | 1 |
| GO:0014022 | neural plate elongation | 3 | 0 | 0.11 | 1 |
| GO:0014028 | notochord formation | 4 | 0 | 0.15 | 1 |
| GO:0014034 | neural crest cell fate commitment | 3 | 0 | 0.11 | 1 |
| GO:0014036 | neural crest cell fate specification | 1 | 0 | 0.04 | 1 |
| GO:0014038 | regulation of Schwann cell differentiati... | 2 | 0 | 0.08 | 1 |
| GO:0014040 | positive regulation of Schwann cell diff... | 2 | 0 | 0.08 | 1 |
| GO:0014041 | regulation of neuron maturation | 7 | 0 | 0.27 | 1 |
| GO:0014042 | positive regulation of neuron maturation | 3 | 0 | 0.11 | 1 |
| GO:0014043 | negative regulation of neuron maturation | 3 | 0 | 0.11 | 1 |
| GO:0014046 | dopamine secretion | 18 | 0 | 0.69 | 1 |
| GO:0014047 | glutamate secretion | 37 | 0 | 1.41 | 1 |
| GO:0014048 | regulation of glutamate secretion | 13 | 0 | 0.49 | 1 |
| GO:0014049 | positive regulation of glutamate secreti... | 5 | 0 | 0.19 | 1 |
| GO:0014050 | negative regulation of glutamate secreti... | 7 | 0 | 0.27 | 1 |
| GO:0014051 | gamma-aminobutyric acid secretion | 10 | 0 | 0.38 | 1 |
| GO:0014052 | regulation of gamma-aminobutyric acid se. | 6 | 0 | 0.23 | 1 |
| GO:0014053 | negative regulation of gamma-aminobutyri | 2 | 0 | 0.08 | 1 |
| GO:0014054 | positive regulation of gamma-aminobutyri.. | 5 | 0 | 0.19 | 1 |
| GO:0014055 | acetylcholine secretion | neurotransr | 4 | 0 | 0.15 |
| GO:0014056 | regulation of acetylcholine secretion | n... | 4 | 0 | 0.15 |
| GO:0014057 | positive regulation of acetylcholine sec... | 1 | 0 | 0.04 | 1 |
| GO:0014058 | negative regulation of acetylcholine sec... | 1 | 0 | 0.04 | 1 |
| GO:0014059 | regulation of dopamine secretion | 18 | 0 | 0.69 | 1 |
| GO:0014060 | regulation of epinephrine secretion | 7 | 0 | 0.27 | 1 |
| GO:0014061 | regulation of norepinephrine secretion | 13 | 0 | 0.49 | 1 |
| GO:0014062 | regulation of serotonin secretion | 5 | 0 | 0.19 | 1 |
| GO:0014063 | negative regulation of serotonin secreti... | 3 | 0 | 0.11 | 1 |
| GO:0014075 | response to amine | 39 | 0 | 1.48 | 1 |
| GO:0014076 | response to fluoxetine | 1 | 0 | 0.04 | 1 |
| GO:0014707 | branchiomeric skeletal muscle developmei | 3 | 0 | 0.11 | 1 |
| GO:0014717 | regulation of satellite cell activation ... | 3 | 0 | 0.11 | 1 |
| GO:0014718 | positive regulation of satellite cell ac... | 2 | 0 | 0.08 | 1 |
| GO:0014721 | twitch skeletal muscle contraction | 3 | 0 | 0.11 | 1 |
| GO:0014722 | regulation of skeletal muscle contractio... | 4 | 0 | 0.15 | 1 |
| GO:0014724 | regulation of twitch skeletal muscle con... | 1 | 0 | 0.04 | 1 |
| GO:0014732 | skeletal muscle atrophy | 5 | 0 | 0.19 | 1 |
| GO:0014734 | skeletal muscle hypertrophy | 1 | 0 | 0.04 | 1 |
| GO:0014735 | regulation of muscle atrophy | 1 | 0 | 0.04 | 1 |
| GO:0014737 | positive regulation of muscle atrophy | 1 | 0 | 0.04 | 1 |
| GO:0014738 | regulation of muscle hyperplasia | 3 | 0 | 0.11 | 1 |
| GO:0014739 | positive regulation of muscle hyperplasi... | 1 | 0 | 0.04 | 1 |
| GO:0014740 | negative regulation of muscle hyperplasi... | 2 | 0 | 0.08 | 1 |
| GO:0014741 | negative regulation of muscle hypertroph.. | 10 | 0 | 0.38 | 1 |
| GO:0014744 | positive regulation of muscle adaptation | 4 | 0 | 0.15 | 1 |
| GO:0014745 | negative regulation of muscle adaptation | 4 | 0 | 0.15 | 1 |
| GO:0014805 | smooth muscle adaptation | 4 | 0 | 0.15 | 1 |
| GO:0014806 | smooth muscle hyperplasia | 2 | 0 | 0.08 | 1 |
| GO:0014807 | regulation of somitogenesis | 8 | 0 | 0.3 | 1 |
| GO:0014809 | regulation of skeletal muscle contractio... | 4 | 0 | 0.15 | 1 |
| GO:0014810 | positive regulation of skeletal muscle c... | 1 | 0 | 0.04 | 1 |
| GO:0014813 | skeletal muscle satellite cell commitmen... | 1 | 0 | 0.04 | 1 |

| | | | | | |
|---|---|---|---|---|---|
| GO:0014822 | detection of wounding | 1 | 0 | 0.04 | 1 |
| GO:0014827 | intestine smooth muscle contraction | 3 | 0 | 0.11 | 1 |
| GO:0014831 | gastro-intestinal system smooth muscle c.. | 5 | 0 | 0.19 | 1 |
| GO:0014832 | urinary bladder smooth muscle contractio.. | 7 | 0 | 0.27 | 1 |
| GO:0014835 | myoblast differentiation involved in ske... | 3 | 0 | 0.11 | 1 |
| GO:0014839 | myoblast migration involved in skeletal ... | 1 | 0 | 0.04 | 1 |
| GO:0014841 | skeletal muscle satellite cell prolifera... | 8 | 0 | 0.3 | 1 |
| GO:0014842 | regulation of satellite cell proliferati... | 7 | 0 | 0.27 | 1 |
| GO:0014843 | growth factor dependent regulation of sk... | 1 | 0 | 0.04 | 1 |
| GO:0014846 | esophagus smooth muscle contraction | 2 | 0 | 0.08 | 1 |
| GO:0014848 | urinary tract smooth muscle contraction | 8 | 0 | 0.3 | 1 |
| GO:0014849 | ureter smooth muscle contraction | 1 | 0 | 0.04 | 1 |
| GO:0014850 | response to muscle activity | 11 | 0 | 0.42 | 1 |
| GO:0014856 | skeletal muscle cell proliferation | 10 | 0 | 0.38 | 1 |
| GO:0014857 | regulation of skeletal muscle cell proli... | 8 | 0 | 0.3 | 1 |
| GO:0014858 | positive regulation of skeletal muscle c... | 1 | 0 | 0.04 | 1 |
| GO:0014859 | negative regulation of skeletal muscle c... | 2 | 0 | 0.08 | 1 |
| GO:0014866 | skeletal myofibril assembly | 7 | 0 | 0.27 | 1 |
| GO:0014873 | response to muscle activity involved in ... | 2 | 0 | 0.08 | 1 |
| GO:0014876 | response to injury involved in regulatio... | 1 | 0 | 0.04 | 1 |
| GO:0014878 | response to electrical stimulus involved... | 3 | 0 | 0.11 | 1 |
| GO:0014883 | transition between fast and slow fiber | 1 | 0 | 0.04 | 1 |
| GO:0014886 | transition between slow and fast fiber | 2 | 0 | 0.08 | 1 |
| GO:0014889 | muscle atrophy | 8 | 0 | 0.3 | 1 |
| GO:0014891 | striated muscle atrophy | 7 | 0 | 0.27 | 1 |
| GO:0014895 | smooth muscle hypertrophy | 1 | 0 | 0.04 | 1 |
| GO:0014900 | muscle hyperplasia | 3 | 0 | 0.11 | 1 |
| GO:0014901 | satellite cell activation involved in sk... | 3 | 0 | 0.11 | 1 |
| GO:0014908 | myotube differentiation involved in skel... | 2 | 0 | 0.08 | 1 |
| GO:0014912 | negative regulation of smooth muscle cel.. | 13 | 0 | 0.49 | 1 |
| GO:0014916 | regulation of lung blood pressure | 2 | 0 | 0.08 | 1 |
| GO:0015012 | heparan sulfate proteoglycan biosyntheti... | 23 | 0 | 0.88 | 1 |
| GO:0015014 | heparan sulfate proteoglycan biosyntheti... | 3 | 0 | 0.11 | 1 |
| GO:0015015 | heparan sulfate proteoglycan biosyntheti... | 5 | 0 | 0.19 | 1 |
| GO:0015671 | oxygen transport | 14 | 0 | 0.53 | 1 |
| GO:0015675 | nickel cation transport | 1 | 0 | 0.04 | 1 |
| GO:0015676 | vanadium ion transport | 1 | 0 | 0.04 | 1 |
| GO:0015679 | plasma membrane copper ion transport | 1 | 0 | 0.04 | 1 |
| GO:0015680 | intracellular copper ion transport | 3 | 0 | 0.11 | 1 |
| GO:0015684 | ferrous iron transport | 2 | 0 | 0.08 | 1 |
| GO:0015688 | iron chelate transport | 2 | 0 | 0.08 | 1 |
| GO:0015689 | molybdate ion transport | 1 | 0 | 0.04 | 1 |
| GO:0015691 | cadmium ion transport | 4 | 0 | 0.15 | 1 |
| GO:0015692 | lead ion transport | 1 | 0 | 0.04 | 1 |
| GO:0015695 | organic cation transport | 14 | 0 | 0.53 | 1 |
| GO:0015701 | bicarbonate transport | 33 | 0 | 1.26 | 1 |
| GO:0015705 | iodide transport | 4 | 0 | 0.15 | 1 |
| GO:0015706 | nitrate transport | 1 | 0 | 0.04 | 1 |
| GO:0015707 | nitrite transport | 1 | 0 | 0.04 | 1 |
| GO:0015712 | hexose phosphate transport | 4 | 0 | 0.15 | 1 |
| GO:0015722 | canalicular bile acid transport | 4 | 0 | 0.15 | 1 |
| GO:0015724 | formate transport | 1 | 0 | 0.04 | 1 |
| GO:0015727 | lactate transport | 7 | 0 | 0.27 | 1 |

| | | | | | |
|---|---|---|---|---|---|
| GO:0015728 | mevalonate transport | 1 | 0 | 0.04 | 1 |
| GO:0015734 | taurine transport | 1 | 0 | 0.04 | 1 |
| GO:0015739 | sialic acid transport | 1 | 0 | 0.04 | 1 |
| GO:0015740 | C4-dicarboxylate transport | 8 | 0 | 0.3 | 1 |
| GO:0015742 | alpha-ketoglutarate transport | 2 | 0 | 0.08 | 1 |
| GO:0015744 | succinate transport | 1 | 0 | 0.04 | 1 |
| GO:0015746 | citrate transport | 3 | 0 | 0.11 | 1 |
| GO:0015747 | urate transport | 3 | 0 | 0.11 | 1 |
| GO:0015755 | fructose transport | 1 | 0 | 0.04 | 1 |
| GO:0015760 | glucose-6-phosphate transport | 4 | 0 | 0.15 | 1 |
| GO:0015761 | mannose transport | 1 | 0 | 0.04 | 1 |
| GO:0015780 | nucleotide-sugar transport | 7 | 0 | 0.27 | 1 |
| GO:0015781 | pyrimidine nucleotide-sugar transport | 6 | 0 | 0.23 | 1 |
| GO:0015782 | CMP-N-acetylneuraminate transport | 1 | 0 | 0.04 | 1 |
| GO:0015785 | UDP-galactose transport | 2 | 0 | 0.08 | 1 |
| GO:0015787 | UDP-glucuronic acid transport | 1 | 0 | 0.04 | 1 |
| GO:0015788 | UDP-N-acetylglucosamine transport | 2 | 0 | 0.08 | 1 |
| GO:0015790 | UDP-xylose transport | 1 | 0 | 0.04 | 1 |
| GO:0015797 | mannitol transport | 1 | 0 | 0.04 | 1 |
| GO:0015802 | basic amino acid transport | 12 | 0 | 0.46 | 1 |
| GO:0015805 | S-adenosyl-L-methionine transport | 1 | 0 | 0.04 | 1 |
| GO:0015809 | arginine transport | 7 | 0 | 0.27 | 1 |
| GO:0015810 | aspartate transport | 7 | 0 | 0.27 | 1 |
| GO:0015811 | L-cystine transport | 4 | 0 | 0.15 | 1 |
| GO:0015812 | gamma-aminobutyric acid transport | 14 | 0 | 0.53 | 1 |
| GO:0015817 | histidine transport | 3 | 0 | 0.11 | 1 |
| GO:0015819 | lysine transport | 3 | 0 | 0.11 | 1 |
| GO:0015821 | methionine transport | 1 | 0 | 0.04 | 1 |
| GO:0015822 | ornithine transport | 2 | 0 | 0.08 | 1 |
| GO:0015825 | L-serine transport | 8 | 0 | 0.3 | 1 |
| GO:0015826 | threonine transport | 1 | 0 | 0.04 | 1 |
| GO:0015827 | tryptophan transport | 2 | 0 | 0.08 | 1 |
| GO:0015828 | tyrosine transport | 1 | 0 | 0.04 | 1 |
| GO:0015842 | synaptic vesicle amine transport | 2 | 0 | 0.08 | 1 |
| GO:0015844 | monoamine transport | 65 | 0 | 2.47 | 1 |
| GO:0015846 | polyamine transport | 5 | 0 | 0.19 | 1 |
| GO:0015847 | putrescine transport | 1 | 0 | 0.04 | 1 |
| GO:0015851 | nucleobase transport | 7 | 0 | 0.27 | 1 |
| GO:0015853 | adenine transport | 3 | 0 | 0.11 | 1 |
| GO:0015855 | pyrimidine nucleobase transport | 2 | 0 | 0.08 | 1 |
| GO:0015858 | nucleoside transport | 17 | 0 | 0.65 | 1 |
| GO:0015860 | purine nucleoside transmembrane transpo | 6 | 0 | 0.23 | 1 |
| GO:0015862 | uridine transport | 1 | 0 | 0.04 | 1 |
| GO:0015864 | pyrimidine nucleoside transport | 2 | 0 | 0.08 | 1 |
| GO:0015865 | purine nucleotide transport | 9 | 0 | 0.34 | 1 |
| GO:0015866 | ADP transport | 3 | 0 | 0.11 | 1 |
| GO:0015867 | ATP transport | 6 | 0 | 0.23 | 1 |
| GO:0015868 | purine ribonucleotide transport | 7 | 0 | 0.27 | 1 |
| GO:0015870 | acetylcholine transport | 6 | 0 | 0.23 | 1 |
| GO:0015871 | choline transport | 5 | 0 | 0.19 | 1 |
| GO:0015872 | dopamine transport | 29 | 0 | 1.1 | 1 |
| GO:0015874 | norepinephrine transport | 17 | 0 | 0.65 | 1 |
| GO:0015876 | acetyl-CoA transport | 1 | 0 | 0.04 | 1 |

| | | | | | |
|---|---|---|---|---|---|
| GO:0015878 | biotin transport | 1 | 0 | 0.04 | 1 |
| GO:0015880 | coenzyme A transport | 2 | 0 | 0.08 | 1 |
| GO:0015881 | creatine transport | 2 | 0 | 0.08 | 1 |
| GO:0015882 | L-ascorbic acid transport | 2 | 0 | 0.08 | 1 |
| GO:0015883 | FAD transport | 1 | 0 | 0.04 | 1 |
| GO:0015884 | folic acid transport | 7 | 0 | 0.27 | 1 |
| GO:0015886 | heme transport | 9 | 0 | 0.34 | 1 |
| GO:0015887 | pantothenate transmembrane transport | 1 | 0 | 0.04 | 1 |
| GO:0015888 | thiamine transport | 4 | 0 | 0.15 | 1 |
| GO:0015889 | cobalamin transport | 5 | 0 | 0.19 | 1 |
| GO:0015891 | siderophore transport | 2 | 0 | 0.08 | 1 |
| GO:0015893 | drug transport | 24 | 0 | 0.91 | 1 |
| GO:0015904 | tetracycline transport | 1 | 0 | 0.04 | 1 |
| GO:0015910 | peroxisomal long-chain fatty acid import | 1 | 0 | 0.04 | 1 |
| GO:0015911 | plasma membrane long-chain fatty acid tr.. | 7 | 0 | 0.27 | 1 |
| GO:0015912 | short-chain fatty acid transport | 1 | 0 | 0.04 | 1 |
| GO:0015913 | short-chain fatty acid import | 1 | 0 | 0.04 | 1 |
| GO:0015916 | fatty-acyl-CoA transport | 1 | 0 | 0.04 | 1 |
| GO:0015917 | aminophospholipid transport | 1 | 0 | 0.04 | 1 |
| GO:0015919 | peroxisomal membrane transport | 7 | 0 | 0.27 | 1 |
| GO:0015920 | lipopolysaccharide transport | 2 | 0 | 0.08 | 1 |
| GO:0015936 | coenzyme A metabolic process | 18 | 0 | 0.69 | 1 |
| GO:0015937 | coenzyme A biosynthetic process | 11 | 0 | 0.42 | 1 |
| GO:0015938 | coenzyme A catabolic process | 2 | 0 | 0.08 | 1 |
| GO:0015942 | formate metabolic process | 6 | 0 | 0.23 | 1 |
| GO:0015949 | nucleobase-containing small molecule int.. | 17 | 0 | 0.65 | 1 |
| GO:0015959 | diadenosine polyphosphate metabolic proc | 3 | 0 | 0.11 | 1 |
| GO:0015960 | diadenosine polyphosphate biosynthetic p. | 2 | 0 | 0.08 | 1 |
| GO:0015961 | diadenosine polyphosphate catabolic proc. | 1 | 0 | 0.04 | 1 |
| GO:0015965 | diadenosine tetraphosphate metabolic pro. | 2 | 0 | 0.08 | 1 |
| GO:0015966 | diadenosine tetraphosphate biosynthetic .. | 2 | 0 | 0.08 | 1 |
| GO:0015993 | molecular hydrogen transport | 2 | 0 | 0.08 | 1 |
| GO:0016024 | CDP-diacylglycerol biosynthetic process | 12 | 0 | 0.46 | 1 |
| GO:0016036 | cellular response to phosphate starvatio... | 1 | 0 | 0.04 | 1 |
| GO:0016045 | detection of bacterium | 18 | 0 | 0.69 | 1 |
| GO:0016046 | detection of fungus | 1 | 0 | 0.04 | 1 |
| GO:0016048 | detection of temperature stimulus | 13 | 0 | 0.49 | 1 |
| GO:0016056 | rhodopsin mediated signaling pathway | 37 | 0 | 1.41 | 1 |
| GO:0016062 | adaptation of rhodopsin mediated signali... | 2 | 0 | 0.08 | 1 |
| GO:0016068 | type I hypersensitivity | 2 | 0 | 0.08 | 1 |
| GO:0016074 | snoRNA metabolic process | 5 | 0 | 0.19 | 1 |
| GO:0016075 | rRNA catabolic process | 10 | 0 | 0.38 | 1 |
| GO:0016077 | snoRNA catabolic process | 1 | 0 | 0.04 | 1 |
| GO:0016078 | tRNA catabolic process | 3 | 0 | 0.11 | 1 |
| GO:0016079 | synaptic vesicle exocytosis | 78 | 0 | 2.97 | 1 |
| GO:0016080 | synaptic vesicle targeting | 3 | 0 | 0.11 | 1 |
| GO:0016081 | synaptic vesicle docking | 7 | 0 | 0.27 | 1 |
| GO:0016082 | synaptic vesicle priming | 12 | 0 | 0.46 | 1 |
| GO:0016093 | polyprenol metabolic process | 4 | 0 | 0.15 | 1 |
| GO:0016094 | polyprenol biosynthetic process | 1 | 0 | 0.04 | 1 |
| GO:0016095 | polyprenol catabolic process | 1 | 0 | 0.04 | 1 |
| GO:0016098 | monoterpenoid metabolic process | 6 | 0 | 0.23 | 1 |
| GO:0016103 | diterpenoid catabolic process | 3 | 0 | 0.11 | 1 |

| | | | | | |
|---|---|---|---|---|---|
| GO:0016107 | sesquiterpenoid catabolic process | 2 | 0 | 0.08 | 1 |
| GO:0016108 | tetraterpenoid metabolic process | 1 | 0 | 0.04 | 1 |
| GO:0016115 | terpenoid catabolic process | 5 | 0 | 0.19 | 1 |
| GO:0016116 | carotenoid metabolic process | 1 | 0 | 0.04 | 1 |
| GO:0016119 | carotene metabolic process | 1 | 0 | 0.04 | 1 |
| GO:0016121 | carotene catabolic process | 1 | 0 | 0.04 | 1 |
| GO:0016127 | sterol catabolic process | 11 | 0 | 0.42 | 1 |
| GO:0016139 | glycoside catabolic process | 7 | 0 | 0.27 | 1 |
| GO:0016180 | snRNA processing | 15 | 0 | 0.57 | 1 |
| GO:0016188 | synaptic vesicle maturation | 8 | 0 | 0.3 | 1 |
| GO:0016189 | synaptic vesicle to endosome fusion | 1 | 0 | 0.04 | 1 |
| GO:0016191 | synaptic vesicle uncoating | 1 | 0 | 0.04 | 1 |
| GO:0016226 | iron-sulfur cluster assembly | 17 | 0 | 0.65 | 1 |
| GO:0016237 | microautophagy | 2 | 0 | 0.08 | 1 |
| GO:0016242 | negative regulation of macroautophagy | 10 | 0 | 0.38 | 1 |
| GO:0016246 | RNA interference | 6 | 0 | 0.23 | 1 |
| GO:0016254 | preassembly of GPI anchor in ER membra | 16 | 0 | 0.61 | 1 |
| GO:0016256 | N-glycan processing to lysosome | 1 | 0 | 0.04 | 1 |
| GO:0016259 | selenocysteine metabolic process | 1 | 0 | 0.04 | 1 |
| GO:0016260 | selenocysteine biosynthetic process | 1 | 0 | 0.04 | 1 |
| GO:0016264 | gap junction assembly | 12 | 0 | 0.46 | 1 |
| GO:0016269 | O-glycan processing | core 3 | 1 | 0 | 0.04 |
| GO:0016320 | endoplasmic reticulum membrane fusion | 2 | 0 | 0.08 | 1 |
| GO:0016322 | neuron remodeling | 9 | 0 | 0.34 | 1 |
| GO:0016332 | establishment or maintenance of polarity... | 1 | 0 | 0.04 | 1 |
| GO:0016340 | calcium-dependent cell-matrix adhesion | 2 | 0 | 0.08 | 1 |
| GO:0016344 | meiotic chromosome movement towards s | 3 | 0 | 0.11 | 1 |
| GO:0016479 | negative regulation of transcription fro... | 4 | 0 | 0.15 | 1 |
| GO:0016487 | farnesol metabolic process | 2 | 0 | 0.08 | 1 |
| GO:0016488 | farnesol catabolic process | 2 | 0 | 0.08 | 1 |
| GO:0016539 | intein-mediated protein splicing | 2 | 0 | 0.08 | 1 |
| GO:0016540 | protein autoprocessing | 10 | 0 | 0.38 | 1 |
| GO:0016557 | peroxisome membrane biogenesis | 4 | 0 | 0.15 | 1 |
| GO:0016558 | protein import into peroxisome matrix | 12 | 0 | 0.46 | 1 |
| GO:0016559 | peroxisome fission | 10 | 0 | 0.38 | 1 |
| GO:0016560 | protein import into peroxisome matrix | d... | 3 | 0 | 0.11 |
| GO:0016561 | protein import into peroxisome matrix | t... | 3 | 0 | 0.11 |
| GO:0016576 | histone dephosphorylation | 4 | 0 | 0.15 | 1 |
| GO:0016577 | histone demethylation | 24 | 0 | 0.91 | 1 |
| GO:0016598 | protein arginylation | 1 | 0 | 0.04 | 1 |
| GO:0016926 | protein desumoylation | 4 | 0 | 0.15 | 1 |
| GO:0016973 | poly(A)+ mRNA export from nucleus | 10 | 0 | 0.38 | 1 |
| GO:0016998 | cell wall macromolecule catabolic proces... | 4 | 0 | 0.15 | 1 |
| GO:0016999 | antibiotic metabolic process | 1 | 0 | 0.04 | 1 |
| GO:0017004 | cytochrome complex assembly | 12 | 0 | 0.46 | 1 |
| GO:0017014 | protein nitrosylation | 9 | 0 | 0.34 | 1 |
| GO:0017055 | negative regulation of RNA polymerase II... | 2 | 0 | 0.08 | 1 |
| GO:0017062 | respiratory chain complex III assembly | 3 | 0 | 0.11 | 1 |
| GO:0017085 | response to insecticide | 12 | 0 | 0.46 | 1 |
| GO:0017121 | phospholipid scrambling | 11 | 0 | 0.42 | 1 |
| GO:0017126 | nucleologenesis | 1 | 0 | 0.04 | 1 |
| GO:0017143 | insecticide metabolic process | 2 | 0 | 0.08 | 1 |
| GO:0017144 | drug metabolic process | 43 | 0 | 1.64 | 1 |

| | | | | | |
|---|---|---|---|---|---|
| GO:0017156 | calcium ion-dependent exocytosis | 68 | 0 | 2.59 | 1 |
| GO:0017158 | regulation of calcium ion-dependent exoc.. | 45 | 0 | 1.71 | 1 |
| GO:0017182 | peptidyl-diphthamide metabolic process | 6 | 0 | 0.23 | 1 |
| GO:0017183 | peptidyl-diphthamide biosynthetic proces... | 6 | 0 | 0.23 | 1 |
| GO:0017185 | peptidyl-lysine hydroxylation | 1 | 0 | 0.04 | 1 |
| GO:0017186 | peptidyl-pyroglutamic acid biosynthetic ... | 2 | 0 | 0.08 | 1 |
| GO:0017187 | peptidyl-glutamic acid carboxylation | 12 | 0 | 0.46 | 1 |
| GO:0018003 | peptidyl-lysine N6-acetylation | 2 | 0 | 0.08 | 1 |
| GO:0018008 | N-terminal peptidyl-glycine N-myristoyla... | 2 | 0 | 0.08 | 1 |
| GO:0018009 | N-terminal peptidyl-L-cysteine N-palmito... | 1 | 0 | 0.04 | 1 |
| GO:0018023 | peptidyl-lysine trimethylation | 17 | 0 | 0.65 | 1 |
| GO:0018026 | peptidyl-lysine monomethylation | 6 | 0 | 0.23 | 1 |
| GO:0018027 | peptidyl-lysine dimethylation | 9 | 0 | 0.34 | 1 |
| GO:0018065 | protein-cofactor linkage | 8 | 0 | 0.3 | 1 |
| GO:0018094 | protein polyglycylation | 1 | 0 | 0.04 | 1 |
| GO:0018095 | protein polyglutamylation | 5 | 0 | 0.19 | 1 |
| GO:0018101 | protein citrullination | 5 | 0 | 0.19 | 1 |
| GO:0018103 | protein C-linked glycosylation | 4 | 0 | 0.15 | 1 |
| GO:0018106 | peptidyl-histidine phosphorylation | 1 | 0 | 0.04 | 1 |
| GO:0018117 | protein adenylylation | 2 | 0 | 0.08 | 1 |
| GO:0018119 | peptidyl-cysteine S-nitrosylation | 9 | 0 | 0.34 | 1 |
| GO:0018125 | peptidyl-cysteine methylation | 3 | 0 | 0.11 | 1 |
| GO:0018153 | isopeptide cross-linking via N6-(L-isogl... | 2 | 0 | 0.08 | 1 |
| GO:0018158 | protein oxidation | 5 | 0 | 0.19 | 1 |
| GO:0018160 | peptidyl-pyrromethane cofactor linkage | 1 | 0 | 0.04 | 1 |
| GO:0018171 | peptidyl-cysteine oxidation | 2 | 0 | 0.08 | 1 |
| GO:0018175 | protein nucleotidylation | 2 | 0 | 0.08 | 1 |
| GO:0018190 | protein octanoylation | 1 | 0 | 0.04 | 1 |
| GO:0018191 | peptidyl-serine octanoylation | 1 | 0 | 0.04 | 1 |
| GO:0018192 | enzyme active site formation via L-cyste... | 1 | 0 | 0.04 | 1 |
| GO:0018197 | peptidyl-aspartic acid modification | 2 | 0 | 0.08 | 1 |
| GO:0018198 | peptidyl-cysteine modification | 22 | 0 | 0.84 | 1 |
| GO:0018199 | peptidyl-glutamine modification | 4 | 0 | 0.15 | 1 |
| GO:0018200 | peptidyl-glutamic acid modification | 22 | 0 | 0.84 | 1 |
| GO:0018201 | peptidyl-glycine modification | 2 | 0 | 0.08 | 1 |
| GO:0018202 | peptidyl-histidine modification | 8 | 0 | 0.3 | 1 |
| GO:0018206 | peptidyl-methionine modification | 5 | 0 | 0.19 | 1 |
| GO:0018211 | peptidyl-tryptophan modification | 4 | 0 | 0.15 | 1 |
| GO:0018214 | protein carboxylation | 12 | 0 | 0.46 | 1 |
| GO:0018230 | peptidyl-L-cysteine S-palmitoylation | 5 | 0 | 0.19 | 1 |
| GO:0018231 | peptidyl-S-diacylglycerol-L-cysteine bio... | 5 | 0 | 0.19 | 1 |
| GO:0018242 | protein O-linked glycosylation via serin... | 3 | 0 | 0.11 | 1 |
| GO:0018243 | protein O-linked glycosylation via threo... | 4 | 0 | 0.15 | 1 |
| GO:0018262 | isopeptide cross-linking | 2 | 0 | 0.08 | 1 |
| GO:0018272 | protein-pyridoxal-5-phosphate linkage vi... | 1 | 0 | 0.04 | 1 |
| GO:0018277 | protein deamination | 2 | 0 | 0.08 | 1 |
| GO:0018282 | metal incorporation into metallo-sulfur ... | 2 | 0 | 0.08 | 1 |
| GO:0018283 | iron incorporation into metallo-sulfur c... | 2 | 0 | 0.08 | 1 |
| GO:0018293 | protein-FAD linkage | 1 | 0 | 0.04 | 1 |
| GO:0018298 | protein-chromophore linkage | 13 | 0 | 0.49 | 1 |
| GO:0018307 | enzyme active site formation | 2 | 0 | 0.08 | 1 |
| GO:0018315 | molybdenum incorporation into molybdenu | 1 | 0 | 0.04 | 1 |
| GO:0018317 | protein C-linked glycosylation via trypt... | 4 | 0 | 0.15 | 1 |

| | | | | | |
|---|---|---|---|---|---|
| GO:0018323 | enzyme active site formation via L-cyste... | 1 | 0 | 0.04 | 1 |
| GO:0018342 | protein prenylation | 10 | 0 | 0.38 | 1 |
| GO:0018343 | protein farnesylation | 3 | 0 | 0.11 | 1 |
| GO:0018344 | protein geranylgeranylation | 7 | 0 | 0.27 | 1 |
| GO:0018345 | protein palmitoylation | 24 | 0 | 0.91 | 1 |
| GO:0018350 | protein esterification | 1 | 0 | 0.04 | 1 |
| GO:0018352 | protein-pyridoxal-5-phosphate linkage | 2 | 0 | 0.08 | 1 |
| GO:0018364 | peptidyl-glutamine methylation | 2 | 0 | 0.08 | 1 |
| GO:0018395 | peptidyl-lysine hydroxylation to 5-hydro... | 1 | 0 | 0.04 | 1 |
| GO:0018400 | peptidyl-proline hydroxylation to 3-hydr... | 1 | 0 | 0.04 | 1 |
| GO:0018406 | protein C-linked glycosylation via 2'-al... | 4 | 0 | 0.15 | 1 |
| GO:0018872 | arsonoacetate metabolic process | 1 | 0 | 0.04 | 1 |
| GO:0018874 | benzoate metabolic process | 1 | 0 | 0.04 | 1 |
| GO:0018879 | biphenyl metabolic process | 6 | 0 | 0.23 | 1 |
| GO:0018894 | dibenzo-p-dioxin metabolic process | 6 | 0 | 0.23 | 1 |
| GO:0018904 | ether metabolic process | 13 | 0 | 0.49 | 1 |
| GO:0018916 | nitrobenzene metabolic process | 4 | 0 | 0.15 | 1 |
| GO:0018917 | fluorene metabolic process | 1 | 0 | 0.04 | 1 |
| GO:0018924 | mandelate metabolic process | 1 | 0 | 0.04 | 1 |
| GO:0018931 | naphthalene metabolic process | 1 | 0 | 0.04 | 1 |
| GO:0018958 | phenol-containing compound metabolic pro | 90 | 0 | 3.43 | 1 |
| GO:0018963 | phthalate metabolic process | 6 | 0 | 0.23 | 1 |
| GO:0018964 | propylene metabolic process | 1 | 0 | 0.04 | 1 |
| GO:0018969 | thiocyanate metabolic process | 1 | 0 | 0.04 | 1 |
| GO:0018979 | trichloroethylene metabolic process | 1 | 0 | 0.04 | 1 |
| GO:0018992 | germ-line sex determination | 2 | 0 | 0.08 | 1 |
| GO:0018993 | somatic sex determination | 1 | 0 | 0.04 | 1 |
| GO:0019042 | viral latency | 11 | 0 | 0.42 | 1 |
| GO:0019043 | establishment of viral latency | 10 | 0 | 0.38 | 1 |
| GO:0019046 | release from viral latency | 2 | 0 | 0.08 | 1 |
| GO:0019049 | evasion or tolerance of host defenses by... | 3 | 0 | 0.11 | 1 |
| GO:0019050 | suppression by virus of host apoptotic p... | 2 | 0 | 0.08 | 1 |
| GO:0019060 | intracellular transport of viral protein... | 5 | 0 | 0.19 | 1 |
| GO:0019061 | uncoating of virus | 1 | 0 | 0.04 | 1 |
| GO:0019062 | virion attachment to host cell | 9 | 0 | 0.34 | 1 |
| GO:0019064 | fusion of virus membrane with host plasm. | 3 | 0 | 0.11 | 1 |
| GO:0019072 | viral genome packaging | 2 | 0 | 0.08 | 1 |
| GO:0019074 | viral RNA genome packaging | 2 | 0 | 0.08 | 1 |
| GO:0019086 | late viral transcription | 2 | 0 | 0.08 | 1 |
| GO:0019087 | transformation of host cell by virus | 3 | 0 | 0.11 | 1 |
| GO:0019088 | immortalization of host cell by virus | 1 | 0 | 0.04 | 1 |
| GO:0019089 | transmission of virus | 1 | 0 | 0.04 | 1 |
| GO:0019100 | male germ-line sex determination | 2 | 0 | 0.08 | 1 |
| GO:0019101 | female somatic sex determination | 1 | 0 | 0.04 | 1 |
| GO:0019227 | neuronal action potential propagation | 8 | 0 | 0.3 | 1 |
| GO:0019230 | proprioception | 4 | 0 | 0.15 | 1 |
| GO:0019236 | response to pheromone | 5 | 0 | 0.19 | 1 |
| GO:0019240 | citrulline biosynthetic process | 9 | 0 | 0.34 | 1 |
| GO:0019242 | methylglyoxal biosynthetic process | 1 | 0 | 0.04 | 1 |
| GO:0019243 | methylglyoxal catabolic process to D-lac... | 1 | 0 | 0.04 | 1 |
| GO:0019244 | lactate biosynthetic process from pyruva... | 2 | 0 | 0.08 | 1 |
| GO:0019249 | lactate biosynthetic process | 4 | 0 | 0.15 | 1 |
| GO:0019254 | carnitine metabolic process | CoA-linked | 3 | 0 | 0.11 |

| | | | | | | |
|---|---|---|---|---|---|---|
| GO:0019255 | glucose 1-phosphate metabolic process | | 2 | 0 | 0.08 | 1 |
| GO:0019262 | N-acetylneuraminate catabolic process | | 4 | 0 | 0.15 | 1 |
| GO:0019264 | glycine biosynthetic process from serine | | 2 | 0 | 0.08 | 1 |
| GO:0019265 | glycine biosynthetic process | by transam | 2 | 0 | 0.08 | |
| GO:0019276 | UDP-N-acetylgalactosamine metabolic pro | | 3 | 0 | 0.11 | 1 |
| GO:0019284 | L-methionine biosynthetic process from S.. | | 2 | 0 | 0.08 | 1 |
| GO:0019285 | glycine betaine biosynthetic process fro... | | 2 | 0 | 0.08 | 1 |
| GO:0019287 | isopentenyl diphosphate biosynthetic pro... | | 3 | 0 | 0.11 | 1 |
| GO:0019303 | D-ribose catabolic process | | 2 | 0 | 0.08 | 1 |
| GO:0019307 | mannose biosynthetic process | | 2 | 0 | 0.08 | 1 |
| GO:0019310 | inositol catabolic process | | 1 | 0 | 0.04 | 1 |
| GO:0019317 | fucose catabolic process | | 9 | 0 | 0.34 | 1 |
| GO:0019320 | hexose catabolic process | | 21 | 0 | 0.8 | 1 |
| GO:0019321 | pentose metabolic process | | 10 | 0 | 0.38 | 1 |
| GO:0019322 | pentose biosynthetic process | | 4 | 0 | 0.15 | 1 |
| GO:0019323 | pentose catabolic process | | 5 | 0 | 0.19 | 1 |
| GO:0019336 | phenol-containing compound catabolic pro | | 7 | 0 | 0.27 | 1 |
| GO:0019341 | dibenzo-p-dioxin catabolic process | | 1 | 0 | 0.04 | 1 |
| GO:0019343 | cysteine biosynthetic process via cystat... | | 1 | 0 | 0.04 | 1 |
| GO:0019344 | cysteine biosynthetic process | | 3 | 0 | 0.11 | 1 |
| GO:0019346 | transsulfuration | | 2 | 0 | 0.08 | 1 |
| GO:0019348 | dolichol metabolic process | | 4 | 0 | 0.15 | 1 |
| GO:0019364 | pyridine nucleotide catabolic process | | 1 | 0 | 0.04 | 1 |
| GO:0019367 | fatty acid elongation | saturated fa | 5 | 0 | 0.19 | |
| GO:0019368 | fatty acid elongation | unsaturatec | 5 | 0 | 0.19 | |
| GO:0019370 | leukotriene biosynthetic process | | 19 | 0 | 0.72 | 1 |
| GO:0019372 | lipoxygenase pathway | | 13 | 0 | 0.49 | 1 |
| GO:0019373 | epoxygenase P450 pathway | | 19 | 0 | 0.72 | 1 |
| GO:0019374 | galactolipid metabolic process | | 6 | 0 | 0.23 | 1 |
| GO:0019375 | galactolipid biosynthetic process | | 2 | 0 | 0.08 | 1 |
| GO:0019376 | galactolipid catabolic process | | 2 | 0 | 0.08 | 1 |
| GO:0019377 | glycolipid catabolic process | | 13 | 0 | 0.49 | 1 |
| GO:0019388 | galactose catabolic process | | 6 | 0 | 0.23 | 1 |
| GO:0019389 | glucuronoside metabolic process | | 1 | 0 | 0.04 | 1 |
| GO:0019391 | glucuronoside catabolic process | | 1 | 0 | 0.04 | 1 |
| GO:0019402 | galactitol metabolic process | | 1 | 0 | 0.04 | 1 |
| GO:0019405 | alditol catabolic process | | 5 | 0 | 0.19 | 1 |
| GO:0019407 | hexitol catabolic process | | 1 | 0 | 0.04 | 1 |
| GO:0019408 | dolichol biosynthetic process | | 1 | 0 | 0.04 | 1 |
| GO:0019413 | acetate biosynthetic process | | 2 | 0 | 0.08 | 1 |
| GO:0019417 | sulfur oxidation | | 2 | 0 | 0.08 | 1 |
| GO:0019418 | sulfide oxidation | | 5 | 0 | 0.19 | 1 |
| GO:0019427 | acetyl-CoA biosynthetic process from ace.. | | 2 | 0 | 0.08 | 1 |
| GO:0019430 | removal of superoxide radicals | | 17 | 0 | 0.65 | 1 |
| GO:0019441 | tryptophan catabolic process to kynureni... | | 7 | 0 | 0.27 | 1 |
| GO:0019442 | tryptophan catabolic process to acetyl-C... | | 2 | 0 | 0.08 | 1 |
| GO:0019448 | L-cysteine catabolic process | | 3 | 0 | 0.11 | 1 |
| GO:0019470 | 4-hydroxyproline catabolic process | | 3 | 0 | 0.11 | 1 |
| GO:0019474 | L-lysine catabolic process to acetyl-CoA | | 4 | 0 | 0.15 | 1 |
| GO:0019477 | L-lysine catabolic process | | 4 | 0 | 0.15 | 1 |
| GO:0019478 | D-amino acid catabolic process | | 3 | 0 | 0.11 | 1 |
| GO:0019481 | L-alanine catabolic process | by transami | 1 | 0 | 0.04 | |
| GO:0019482 | beta-alanine metabolic process | | 3 | 0 | 0.11 | 1 |

| | | | | | |
|---|---|---|---|---|---|
| GO:0019483 | beta-alanine biosynthetic process | 2 | 0 | 0.08 | 1 |
| GO:0019510 | S-adenosylhomocysteine catabolic proces | 1 | 0 | 0.04 | 1 |
| GO:0019516 | lactate oxidation | 1 | 0 | 0.04 | 1 |
| GO:0019518 | L-threonine catabolic process to glycine | 1 | 0 | 0.04 | 1 |
| GO:0019519 | pentitol metabolic process | 1 | 0 | 0.04 | 1 |
| GO:0019520 | aldonic acid metabolic process | 1 | 0 | 0.04 | 1 |
| GO:0019521 | D-gluconate metabolic process | 1 | 0 | 0.04 | 1 |
| GO:0019527 | pentitol catabolic process | 1 | 0 | 0.04 | 1 |
| GO:0019530 | taurine metabolic process | 7 | 0 | 0.27 | 1 |
| GO:0019532 | oxalate transport | 5 | 0 | 0.19 | 1 |
| GO:0019541 | propionate metabolic process | 2 | 0 | 0.08 | 1 |
| GO:0019542 | propionate biosynthetic process | 2 | 0 | 0.08 | 1 |
| GO:0019550 | glutamate catabolic process to aspartate | 2 | 0 | 0.08 | 1 |
| GO:0019551 | glutamate catabolic process to 2-oxoglut... | 2 | 0 | 0.08 | 1 |
| GO:0019556 | histidine catabolic process to glutamate... | 4 | 0 | 0.15 | 1 |
| GO:0019557 | histidine catabolic process to glutamate... | 4 | 0 | 0.15 | 1 |
| GO:0019563 | glycerol catabolic process | 4 | 0 | 0.15 | 1 |
| GO:0019605 | butyrate metabolic process | 2 | 0 | 0.08 | 1 |
| GO:0019614 | catechol-containing compound catabolic p | 6 | 0 | 0.23 | 1 |
| GO:0019626 | short-chain fatty acid catabolic process | 6 | 0 | 0.23 | 1 |
| GO:0019627 | urea metabolic process | 14 | 0 | 0.53 | 1 |
| GO:0019673 | GDP-mannose metabolic process | 9 | 0 | 0.34 | 1 |
| GO:0019676 | ammonia assimilation cycle | 1 | 0 | 0.04 | 1 |
| GO:0019677 | NAD catabolic process | 1 | 0 | 0.04 | 1 |
| GO:0019682 | glyceraldehyde-3-phosphate metabolic prc | 12 | 0 | 0.46 | 1 |
| GO:0019694 | alkanesulfonate metabolic process | 1 | 0 | 0.04 | 1 |
| GO:0019695 | choline metabolic process | 6 | 0 | 0.23 | 1 |
| GO:0019720 | Mo-molybdopterin cofactor metabolic proc | 7 | 0 | 0.27 | 1 |
| GO:0019730 | antimicrobial humoral response | 34 | 0 | 1.29 | 1 |
| GO:0019731 | antibacterial humoral response | 32 | 0 | 1.22 | 1 |
| GO:0019732 | antifungal humoral response | 1 | 0 | 0.04 | 1 |
| GO:0019740 | nitrogen utilization | 3 | 0 | 0.11 | 1 |
| GO:0019742 | pentacyclic triterpenoid metabolic proce... | 1 | 0 | 0.04 | 1 |
| GO:0019747 | regulation of isoprenoid metabolic proce... | 3 | 0 | 0.11 | 1 |
| GO:0019805 | quinolinate biosynthetic process | 4 | 0 | 0.15 | 1 |
| GO:0019835 | cytolysis | 30 | 0 | 1.14 | 1 |
| GO:0019836 | hemolysis by symbiont of host erythrocyt... | 2 | 0 | 0.08 | 1 |
| GO:0019853 | L-ascorbic acid biosynthetic process | 2 | 0 | 0.08 | 1 |
| GO:0019856 | pyrimidine nucleobase biosynthetic proce.. | 4 | 0 | 0.15 | 1 |
| GO:0019857 | 5-methylcytosine metabolic process | 1 | 0 | 0.04 | 1 |
| GO:0019858 | cytosine metabolic process | 3 | 0 | 0.11 | 1 |
| GO:0019859 | thymine metabolic process | 3 | 0 | 0.11 | 1 |
| GO:0019860 | uracil metabolic process | 3 | 0 | 0.11 | 1 |
| GO:0019883 | antigen processing and presentation of e.. | 12 | 0 | 0.46 | 1 |
| GO:0019885 | antigen processing and presentation of e.. | 9 | 0 | 0.34 | 1 |
| GO:0019889 | pteridine metabolic process | 1 | 0 | 0.04 | 1 |
| GO:0019918 | peptidyl-arginine methylation | to symmet.. | 2 | 0 | 0.08 |
| GO:0019919 | peptidyl-arginine methylation | to asymme. | 7 | 0 | 0.27 |
| GO:0019988 | charged-tRNA amino acid modification | 1 | 0 | 0.04 | 1 |
| GO:0020012 | evasion or tolerance of host immune resp.. | 1 | 0 | 0.04 | 1 |
| GO:0020021 | immortalization of host cell | 1 | 0 | 0.04 | 1 |
| GO:0020027 | hemoglobin metabolic process | 16 | 0 | 0.61 | 1 |
| GO:0021501 | prechordal plate formation | 1 | 0 | 0.04 | 1 |

| | | | | | |
|---|---|---|---|---|---|
| GO:0021502 | neural fold elevation formation | 1 | 0 | 0.04 | 1 |
| GO:0021503 | neural fold bending | 1 | 0 | 0.04 | 1 |
| GO:0021506 | anterior neuropore closure | 2 | 0 | 0.08 | 1 |
| GO:0021508 | floor plate formation | 1 | 0 | 0.04 | 1 |
| GO:0021509 | roof plate formation | 1 | 0 | 0.04 | 1 |
| GO:0021511 | spinal cord patterning | 27 | 0 | 1.03 | 1 |
| GO:0021512 | spinal cord anterior/posterior patternin... | 1 | 0 | 0.04 | 1 |
| GO:0021513 | spinal cord dorsal/ventral patterning | 25 | 0 | 0.95 | 1 |
| GO:0021514 | ventral spinal cord interneuron differen... | 17 | 0 | 0.65 | 1 |
| GO:0021516 | dorsal spinal cord development | 21 | 0 | 0.8 | 1 |
| GO:0021519 | spinal cord association neuron specifica... | 1 | 0 | 0.04 | 1 |
| GO:0021520 | spinal cord motor neuron cell fate speci... | 13 | 0 | 0.49 | 1 |
| GO:0021521 | ventral spinal cord interneuron specific... | 11 | 0 | 0.42 | 1 |
| GO:0021523 | somatic motor neuron differentiation | 6 | 0 | 0.23 | 1 |
| GO:0021524 | visceral motor neuron differentiation | 3 | 0 | 0.11 | 1 |
| GO:0021526 | medial motor column neuron differentiati... | 2 | 0 | 0.08 | 1 |
| GO:0021527 | spinal cord association neuron different... | 15 | 0 | 0.57 | 1 |
| GO:0021529 | spinal cord oligodendrocyte cell differe... | 3 | 0 | 0.11 | 1 |
| GO:0021530 | spinal cord oligodendrocyte cell fate sp... | 3 | 0 | 0.11 | 1 |
| GO:0021534 | cell proliferation in hindbrain | 15 | 0 | 0.57 | 1 |
| GO:0021538 | epithalamus development | 2 | 0 | 0.08 | 1 |
| GO:0021539 | subthalamus development | 2 | 0 | 0.08 | 1 |
| GO:0021540 | corpus callosum morphogenesis | 4 | 0 | 0.15 | 1 |
| GO:0021541 | ammon gyrus development | 1 | 0 | 0.04 | 1 |
| GO:0021546 | rhombomere development | 9 | 0 | 0.34 | 1 |
| GO:0021547 | midbrain-hindbrain boundary initiation | 1 | 0 | 0.04 | 1 |
| GO:0021548 | pons development | 13 | 0 | 0.49 | 1 |
| GO:0021550 | medulla oblongata development | 3 | 0 | 0.11 | 1 |
| GO:0021551 | central nervous system morphogenesis | 2 | 0 | 0.08 | 1 |
| GO:0021554 | optic nerve development | 11 | 0 | 0.42 | 1 |
| GO:0021555 | midbrain-hindbrain boundary morphogenes | 3 | 0 | 0.11 | 1 |
| GO:0021557 | oculomotor nerve development | 3 | 0 | 0.11 | 1 |
| GO:0021558 | trochlear nerve development | 2 | 0 | 0.08 | 1 |
| GO:0021559 | trigeminal nerve development | 9 | 0 | 0.34 | 1 |
| GO:0021560 | abducens nerve development | 1 | 0 | 0.04 | 1 |
| GO:0021561 | facial nerve development | 10 | 0 | 0.38 | 1 |
| GO:0021562 | vestibulocochlear nerve development | 6 | 0 | 0.23 | 1 |
| GO:0021563 | glossopharyngeal nerve development | 4 | 0 | 0.15 | 1 |
| GO:0021564 | vagus nerve development | 3 | 0 | 0.11 | 1 |
| GO:0021568 | rhombomere 2 development | 2 | 0 | 0.08 | 1 |
| GO:0021569 | rhombomere 3 development | 4 | 0 | 0.15 | 1 |
| GO:0021570 | rhombomere 4 development | 3 | 0 | 0.11 | 1 |
| GO:0021571 | rhombomere 5 development | 4 | 0 | 0.15 | 1 |
| GO:0021572 | rhombomere 6 development | 1 | 0 | 0.04 | 1 |
| GO:0021576 | hindbrain formation | 1 | 0 | 0.04 | 1 |
| GO:0021577 | hindbrain structural organization | 2 | 0 | 0.08 | 1 |
| GO:0021586 | pons maturation | 3 | 0 | 0.11 | 1 |
| GO:0021588 | cerebellum formation | 1 | 0 | 0.04 | 1 |
| GO:0021589 | cerebellum structural organization | 2 | 0 | 0.08 | 1 |
| GO:0021592 | fourth ventricle development | 2 | 0 | 0.08 | 1 |
| GO:0021593 | rhombomere morphogenesis | 3 | 0 | 0.11 | 1 |
| GO:0021594 | rhombomere formation | 1 | 0 | 0.04 | 1 |
| GO:0021598 | abducens nerve morphogenesis | 1 | 0 | 0.04 | 1 |

| | | | | | |
|---|---|---|---|---|---|
| GO:0021599 | abducens nerve formation | 1 | 0 | 0.04 | 1 |
| GO:0021602 | cranial nerve morphogenesis | 22 | 0 | 0.84 | 1 |
| GO:0021603 | cranial nerve formation | 5 | 0 | 0.19 | 1 |
| GO:0021604 | cranial nerve structural organization | 11 | 0 | 0.42 | 1 |
| GO:0021610 | facial nerve morphogenesis | 10 | 0 | 0.38 | 1 |
| GO:0021612 | facial nerve structural organization | 9 | 0 | 0.34 | 1 |
| GO:0021615 | glossopharyngeal nerve morphogenesis | 3 | 0 | 0.11 | 1 |
| GO:0021622 | oculomotor nerve morphogenesis | 2 | 0 | 0.08 | 1 |
| GO:0021623 | oculomotor nerve formation | 2 | 0 | 0.08 | 1 |
| GO:0021631 | optic nerve morphogenesis | 6 | 0 | 0.23 | 1 |
| GO:0021633 | optic nerve structural organization | 2 | 0 | 0.08 | 1 |
| GO:0021634 | optic nerve formation | 1 | 0 | 0.04 | 1 |
| GO:0021636 | trigeminal nerve morphogenesis | 5 | 0 | 0.19 | 1 |
| GO:0021637 | trigeminal nerve structural organization | 5 | 0 | 0.19 | 1 |
| GO:0021639 | trochlear nerve morphogenesis | 1 | 0 | 0.04 | 1 |
| GO:0021642 | trochlear nerve formation | 1 | 0 | 0.04 | 1 |
| GO:0021644 | vagus nerve morphogenesis | 2 | 0 | 0.08 | 1 |
| GO:0021648 | vestibulocochlear nerve morphogenesis | 3 | 0 | 0.11 | 1 |
| GO:0021649 | vestibulocochlear nerve structural organ... | 1 | 0 | 0.04 | 1 |
| GO:0021650 | vestibulocochlear nerve formation | 2 | 0 | 0.08 | 1 |
| GO:0021658 | rhombomere 3 morphogenesis | 2 | 0 | 0.08 | 1 |
| GO:0021660 | rhombomere 3 formation | 1 | 0 | 0.04 | 1 |
| GO:0021664 | rhombomere 5 morphogenesis | 1 | 0 | 0.04 | 1 |
| GO:0021666 | rhombomere 5 formation | 1 | 0 | 0.04 | 1 |
| GO:0021678 | third ventricle development | 2 | 0 | 0.08 | 1 |
| GO:0021679 | cerebellar molecular layer development | 1 | 0 | 0.04 | 1 |
| GO:0021682 | nerve maturation | 1 | 0 | 0.04 | 1 |
| GO:0021685 | cerebellar granular layer structural org... | 1 | 0 | 0.04 | 1 |
| GO:0021686 | cerebellar granular layer maturation | 1 | 0 | 0.04 | 1 |
| GO:0021692 | cerebellar Purkinje cell layer morphogen... | 14 | 0 | 0.53 | 1 |
| GO:0021693 | cerebellar Purkinje cell layer structura... | 1 | 0 | 0.04 | 1 |
| GO:0021694 | cerebellar Purkinje cell layer formation | 12 | 0 | 0.46 | 1 |
| GO:0021698 | cerebellar cortex structural organizatio... | 2 | 0 | 0.08 | 1 |
| GO:0021702 | cerebellar Purkinje cell differentiation | 12 | 0 | 0.46 | 1 |
| GO:0021703 | locus ceruleus development | 1 | 0 | 0.04 | 1 |
| GO:0021718 | superior olivary nucleus development | 2 | 0 | 0.08 | 1 |
| GO:0021722 | superior olivary nucleus maturation | 2 | 0 | 0.08 | 1 |
| GO:0021723 | medullary reticular formation developmen... | 1 | 0 | 0.04 | 1 |
| GO:0021730 | trigeminal sensory nucleus development | 2 | 0 | 0.08 | 1 |
| GO:0021732 | midbrain-hindbrain boundary maturation | 1 | 0 | 0.04 | 1 |
| GO:0021740 | principal sensory nucleus of trigeminal ... | 2 | 0 | 0.08 | 1 |
| GO:0021747 | cochlear nucleus development | 4 | 0 | 0.15 | 1 |
| GO:0021750 | vestibular nucleus development | 2 | 0 | 0.08 | 1 |
| GO:0021754 | facial nucleus development | 2 | 0 | 0.08 | 1 |
| GO:0021757 | caudate nucleus development | 1 | 0 | 0.04 | 1 |
| GO:0021758 | putamen development | 1 | 0 | 0.04 | 1 |
| GO:0021759 | globus pallidus development | 2 | 0 | 0.08 | 1 |
| GO:0021762 | substantia nigra development | 45 | 0 | 1.71 | 1 |
| GO:0021763 | subthalamic nucleus development | 1 | 0 | 0.04 | 1 |
| GO:0021764 | amygdala development | 1 | 0 | 0.04 | 1 |
| GO:0021767 | mammillary body development | 1 | 0 | 0.04 | 1 |
| GO:0021768 | nucleus accumbens development | 1 | 0 | 0.04 | 1 |
| GO:0021769 | orbitofrontal cortex development | 3 | 0 | 0.11 | 1 |

| | | | | | |
|---|---|---|---|---|---|
| GO:0021771 | lateral geniculate nucleus development | 1 | 0 | 0.04 | 1 |
| GO:0021773 | striatal medium spiny neuron differentia... | 2 | 0 | 0.08 | 1 |
| GO:0021775 | smoothened signaling pathway involved in | 3 | 0 | 0.11 | 1 |
| GO:0021776 | smoothened signaling pathway involved in | 3 | 0 | 0.11 | 1 |
| GO:0021778 | oligodendrocyte cell fate specification | 5 | 0 | 0.19 | 1 |
| GO:0021779 | oligodendrocyte cell fate commitment | 5 | 0 | 0.19 | 1 |
| GO:0021780 | glial cell fate specification | 5 | 0 | 0.19 | 1 |
| GO:0021781 | glial cell fate commitment | 15 | 0 | 0.57 | 1 |
| GO:0021783 | preganglionic parasympathetic fiber deve.. | 15 | 0 | 0.57 | 1 |
| GO:0021784 | postganglionic parasympathetic fiber dev... | 2 | 0 | 0.08 | 1 |
| GO:0021785 | branchiomotor neuron axon guidance | 6 | 0 | 0.23 | 1 |
| GO:0021793 | chemorepulsion of branchiomotor axon | 1 | 0 | 0.04 | 1 |
| GO:0021796 | cerebral cortex regionalization | 6 | 0 | 0.23 | 1 |
| GO:0021797 | forebrain anterior/posterior pattern spe... | 6 | 0 | 0.23 | 1 |
| GO:0021798 | forebrain dorsal/ventral pattern formati... | 7 | 0 | 0.27 | 1 |
| GO:0021800 | cerebral cortex tangential migration | 8 | 0 | 0.3 | 1 |
| GO:0021812 | neuronal-glial interaction involved in c... | 1 | 0 | 0.04 | 1 |
| GO:0021813 | cell-cell adhesion involved in neuronal-... | 1 | 0 | 0.04 | 1 |
| GO:0021814 | cell motility involved in cerebral corte... | 2 | 0 | 0.08 | 1 |
| GO:0021815 | modulation of microtubule cytoskeleton i... | 1 | 0 | 0.04 | 1 |
| GO:0021816 | extension of a leading process involved ... | 1 | 0 | 0.04 | 1 |
| GO:0021819 | layer formation in cerebral cortex | 10 | 0 | 0.38 | 1 |
| GO:0021823 | cerebral cortex tangential migration usi... | 2 | 0 | 0.08 | 1 |
| GO:0021824 | cerebral cortex tangential migration usi... | 2 | 0 | 0.08 | 1 |
| GO:0021825 | substrate-dependent cerebral cortex tang.. | 4 | 0 | 0.15 | 1 |
| GO:0021826 | substrate-independent telencephalic tang.. | 8 | 0 | 0.3 | 1 |
| GO:0021827 | postnatal olfactory bulb interneuron mig... | 2 | 0 | 0.08 | 1 |
| GO:0021828 | gonadotrophin-releasing hormone neurona | 2 | 0 | 0.08 | 1 |
| GO:0021830 | interneuron migration from the subpalliu... | 7 | 0 | 0.27 | 1 |
| GO:0021831 | embryonic olfactory bulb interneuron pre... | 2 | 0 | 0.08 | 1 |
| GO:0021836 | chemorepulsion involved in postnatal olf... | 2 | 0 | 0.08 | 1 |
| GO:0021843 | substrate-independent telencephalic tang.. | 8 | 0 | 0.3 | 1 |
| GO:0021847 | ventricular zone neuroblast division | 3 | 0 | 0.11 | 1 |
| GO:0021849 | neuroblast division in subventricular zo... | 2 | 0 | 0.08 | 1 |
| GO:0021853 | cerebral cortex GABAergic interneuron mi. | 6 | 0 | 0.23 | 1 |
| GO:0021855 | hypothalamus cell migration | 4 | 0 | 0.15 | 1 |
| GO:0021856 | hypothalamic tangential migration using ... | 2 | 0 | 0.08 | 1 |
| GO:0021858 | GABAergic neuron differentiation in basa.. | 1 | 0 | 0.04 | 1 |
| GO:0021859 | pyramidal neuron differentiation | 7 | 0 | 0.27 | 1 |
| GO:0021860 | pyramidal neuron development | 6 | 0 | 0.23 | 1 |
| GO:0021869 | forebrain ventricular zone progenitor ce... | 6 | 0 | 0.23 | 1 |
| GO:0021870 | Cajal-Retzius cell differentiation | 1 | 0 | 0.04 | 1 |
| GO:0021881 | Wnt-activated signaling pathway involved.. | 1 | 0 | 0.04 | 1 |
| GO:0021882 | regulation of transcription from RNA pol... | 3 | 0 | 0.11 | 1 |
| GO:0021886 | hypothalamus gonadotrophin-releasing ho | 2 | 0 | 0.08 | 1 |
| GO:0021888 | hypothalamus gonadotrophin-releasing ho | 2 | 0 | 0.08 | 1 |
| GO:0021892 | cerebral cortex GABAergic interneuron di.. | 10 | 0 | 0.38 | 1 |
| GO:0021893 | cerebral cortex GABAergic interneuron fa.. | 2 | 0 | 0.08 | 1 |
| GO:0021894 | cerebral cortex GABAergic interneuron de. | 6 | 0 | 0.23 | 1 |
| GO:0021895 | cerebral cortex neuron differentiation | 20 | 0 | 0.76 | 1 |
| GO:0021896 | forebrain astrocyte differentiation | 1 | 0 | 0.04 | 1 |
| GO:0021897 | forebrain astrocyte development | 1 | 0 | 0.04 | 1 |
| GO:0021898 | commitment of multipotent stem cells to ... | 4 | 0 | 0.15 | 1 |

| | | | | | |
|---|---|---|---|---|---|
| GO:0021902 | commitment of neuronal cell to specific ... | 7 | 0 | 0.27 | 1 |
| GO:0021905 | forebrain-midbrain boundary formation | 1 | 0 | 0.04 | 1 |
| GO:0021910 | smoothened signaling pathway involved in | 8 | 0 | 0.3 | 1 |
| GO:0021912 | regulation of transcription from RNA pol... | 3 | 0 | 0.11 | 1 |
| GO:0021913 | regulation of transcription from RNA pol... | 4 | 0 | 0.15 | 1 |
| GO:0021914 | negative regulation of smoothened signal.. | 4 | 0 | 0.15 | 1 |
| GO:0021917 | somatic motor neuron fate commitment | 1 | 0 | 0.04 | 1 |
| GO:0021918 | regulation of transcription from RNA pol... | 1 | 0 | 0.04 | 1 |
| GO:0021919 | BMP signaling pathway involved in spinal.. | 1 | 0 | 0.04 | 1 |
| GO:0021920 | regulation of transcription from RNA pol... | 1 | 0 | 0.04 | 1 |
| GO:0021924 | cell proliferation in external granule l... | 14 | 0 | 0.53 | 1 |
| GO:0021930 | cerebellar granule cell precursor prolif... | 14 | 0 | 0.53 | 1 |
| GO:0021933 | radial glia guided migration of cerebell... | 2 | 0 | 0.08 | 1 |
| GO:0021934 | hindbrain tangential cell migration | 2 | 0 | 0.08 | 1 |
| GO:0021935 | cerebellar granule cell precursor tangen... | 1 | 0 | 0.04 | 1 |
| GO:0021936 | regulation of cerebellar granule cell pr... | 11 | 0 | 0.42 | 1 |
| GO:0021937 | cerebellar Purkinje cell-granule cell pr... | 6 | 0 | 0.23 | 1 |
| GO:0021938 | smoothened signaling pathway involved in | 4 | 0 | 0.15 | 1 |
| GO:0021940 | positive regulation of cerebellar granul... | 9 | 0 | 0.34 | 1 |
| GO:0021941 | negative regulation of cerebellar granul... | 2 | 0 | 0.08 | 1 |
| GO:0021942 | radial glia guided migration of Purkinje... | 2 | 0 | 0.08 | 1 |
| GO:0021952 | central nervous system projection neuron.. | 23 | 0 | 0.88 | 1 |
| GO:0021956 | central nervous system interneuron axono. | 1 | 0 | 0.04 | 1 |
| GO:0021957 | corticospinal tract morphogenesis | 7 | 0 | 0.27 | 1 |
| GO:0021960 | anterior commissure morphogenesis | 6 | 0 | 0.23 | 1 |
| GO:0021965 | spinal cord ventral commissure morphoge | 2 | 0 | 0.08 | 1 |
| GO:0021966 | corticospinal neuron axon guidance | 2 | 0 | 0.08 | 1 |
| GO:0021972 | corticospinal neuron axon guidance throu.. | 1 | 0 | 0.04 | 1 |
| GO:0021979 | hypothalamus cell differentiation | 6 | 0 | 0.23 | 1 |
| GO:0021983 | pituitary gland development | 42 | 0 | 1.6 | 1 |
| GO:0021984 | adenohypophysis development | 14 | 0 | 0.53 | 1 |
| GO:0021985 | neurohypophysis development | 2 | 0 | 0.08 | 1 |
| GO:0021986 | habenula development | 2 | 0 | 0.08 | 1 |
| GO:0021990 | neural plate formation | 1 | 0 | 0.04 | 1 |
| GO:0021993 | initiation of neural tube closure | 1 | 0 | 0.04 | 1 |
| GO:0021995 | neuropore closure | 2 | 0 | 0.08 | 1 |
| GO:0021997 | neural plate axis specification | 2 | 0 | 0.08 | 1 |
| GO:0021998 | neural plate mediolateral regionalizatio... | 1 | 0 | 0.04 | 1 |
| GO:0022004 | midbrain-hindbrain boundary maturation d. | 1 | 0 | 0.04 | 1 |
| GO:0022007 | convergent extension involved in neural ... | 3 | 0 | 0.11 | 1 |
| GO:0022012 | subpallium cell proliferation in forebra... | 1 | 0 | 0.04 | 1 |
| GO:0022018 | lateral ganglionic eminence cell prolife... | 1 | 0 | 0.04 | 1 |
| GO:0022027 | interkinetic nuclear migration | 9 | 0 | 0.34 | 1 |
| GO:0022028 | tangential migration from the subventric... | 7 | 0 | 0.27 | 1 |
| GO:0022400 | regulation of rhodopsin mediated signali... | 32 | 0 | 1.22 | 1 |
| GO:0022401 | negative adaptation of signaling pathway | 15 | 0 | 0.57 | 1 |
| GO:0022410 | circadian sleep/wake cycle process | 20 | 0 | 0.76 | 1 |
| GO:0022601 | menstrual cycle phase | 2 | 0 | 0.08 | 1 |
| GO:0022605 | oogenesis stage | 5 | 0 | 0.19 | 1 |
| GO:0022614 | membrane to membrane docking | 5 | 0 | 0.19 | 1 |
| GO:0022615 | protein to membrane docking | 7 | 0 | 0.27 | 1 |
| GO:0022622 | root system development | 1 | 0 | 0.04 | 1 |
| GO:0023019 | signal transduction involved in regulati... | 21 | 0 | 0.8 | 1 |

| | | | | | |
|---|---|---|---|---|---|
| GO:0023035 | CD40 signaling pathway | 5 | 0 | 0.19 | 1 |
| GO:0023058 | adaptation of signaling pathway | 17 | 0 | 0.65 | 1 |
| GO:0030002 | cellular anion homeostasis | 12 | 0 | 0.46 | 1 |
| GO:0030011 | maintenance of cell polarity | 12 | 0 | 0.46 | 1 |
| GO:0030026 | cellular manganese ion homeostasis | 1 | 0 | 0.04 | 1 |
| GO:0030035 | microspike assembly | 4 | 0 | 0.15 | 1 |
| GO:0030037 | actin filament reorganization involved i... | 1 | 0 | 0.04 | 1 |
| GO:0030038 | contractile actin filament bundle assemb... | 1 | 0 | 0.04 | 1 |
| GO:0030046 | parallel actin filament bundle assembly | 2 | 0 | 0.08 | 1 |
| GO:0030047 | actin modification | 2 | 0 | 0.08 | 1 |
| GO:0030070 | insulin processing | 3 | 0 | 0.11 | 1 |
| GO:0030103 | vasopressin secretion | 2 | 0 | 0.08 | 1 |
| GO:0030149 | sphingolipid catabolic process | 22 | 0 | 0.84 | 1 |
| GO:0030150 | protein import into mitochondrial matrix | 3 | 0 | 0.11 | 1 |
| GO:0030157 | pancreatic juice secretion | 10 | 0 | 0.38 | 1 |
| GO:0030167 | proteoglycan catabolic process | 3 | 0 | 0.11 | 1 |
| GO:0030174 | regulation of DNA-dependent DNA replicat | 4 | 0 | 0.15 | 1 |
| GO:0030186 | melatonin metabolic process | 2 | 0 | 0.08 | 1 |
| GO:0030187 | melatonin biosynthetic process | 2 | 0 | 0.08 | 1 |
| GO:0030194 | positive regulation of blood coagulation | 24 | 0 | 0.91 | 1 |
| GO:0030200 | heparan sulfate proteoglycan catabolic p... | 2 | 0 | 0.08 | 1 |
| GO:0030201 | heparan sulfate proteoglycan metabolic p.. | 28 | 0 | 1.07 | 1 |
| GO:0030202 | heparin metabolic process | 8 | 0 | 0.3 | 1 |
| GO:0030205 | dermatan sulfate metabolic process | 13 | 0 | 0.49 | 1 |
| GO:0030206 | chondroitin sulfate biosynthetic process | 25 | 0 | 0.95 | 1 |
| GO:0030207 | chondroitin sulfate catabolic process | 14 | 0 | 0.53 | 1 |
| GO:0030208 | dermatan sulfate biosynthetic process | 12 | 0 | 0.46 | 1 |
| GO:0030209 | dermatan sulfate catabolic process | 1 | 0 | 0.04 | 1 |
| GO:0030210 | heparin biosynthetic process | 8 | 0 | 0.3 | 1 |
| GO:0030213 | hyaluronan biosynthetic process | 12 | 0 | 0.46 | 1 |
| GO:0030214 | hyaluronan catabolic process | 13 | 0 | 0.49 | 1 |
| GO:0030220 | platelet formation | 15 | 0 | 0.57 | 1 |
| GO:0030221 | basophil differentiation | 2 | 0 | 0.08 | 1 |
| GO:0030223 | neutrophil differentiation | 4 | 0 | 0.15 | 1 |
| GO:0030224 | monocyte differentiation | 27 | 0 | 1.03 | 1 |
| GO:0030237 | female sex determination | 2 | 0 | 0.08 | 1 |
| GO:0030238 | male sex determination | 13 | 0 | 0.49 | 1 |
| GO:0030240 | skeletal muscle thin filament assembly | 5 | 0 | 0.19 | 1 |
| GO:0030241 | skeletal muscle myosin thick filament as... | 3 | 0 | 0.11 | 1 |
| GO:0030242 | peroxisome degradation | 2 | 0 | 0.08 | 1 |
| GO:0030259 | lipid glycosylation | 4 | 0 | 0.15 | 1 |
| GO:0030262 | apoptotic nuclear changes | 29 | 0 | 1.1 | 1 |
| GO:0030263 | apoptotic chromosome condensation | 4 | 0 | 0.15 | 1 |
| GO:0030264 | nuclear fragmentation involved in apopto... | 1 | 0 | 0.04 | 1 |
| GO:0030302 | deoxynucleotide transport | 1 | 0 | 0.04 | 1 |
| GO:0030309 | poly-N-acetyllactosamine metabolic proce. | 5 | 0 | 0.19 | 1 |
| GO:0030311 | poly-N-acetyllactosamine biosynthetic pr... | 4 | 0 | 0.15 | 1 |
| GO:0030318 | melanocyte differentiation | 25 | 0 | 0.95 | 1 |
| GO:0030320 | cellular monovalent inorganic anion home. | 12 | 0 | 0.46 | 1 |
| GO:0030321 | transepithelial chloride transport | 6 | 0 | 0.23 | 1 |
| GO:0030322 | stabilization of membrane potential | 16 | 0 | 0.61 | 1 |
| GO:0030325 | adrenal gland development | 26 | 0 | 0.99 | 1 |
| GO:0030327 | prenylated protein catabolic process | 3 | 0 | 0.11 | 1 |

| | | | | | |
|---|---|---|---|---|---|
| GO:0030328 | prenylcysteine catabolic process | 2 | 0 | 0.08 | 1 |
| GO:0030329 | prenylcysteine metabolic process | 2 | 0 | 0.08 | 1 |
| GO:0030382 | sperm mitochondrion organization | 1 | 0 | 0.04 | 1 |
| GO:0030388 | fructose 1 | 6-bisphospl | 5 | 0 | 0.19 |
| GO:0030389 | fructosamine metabolic process | 1 | 0 | 0.04 | 1 |
| GO:0030393 | fructoselysine metabolic process | 1 | 0 | 0.04 | 1 |
| GO:0030421 | defecation | 1 | 0 | 0.04 | 1 |
| GO:0030422 | production of siRNA involved in RNA inte... | 3 | 0 | 0.11 | 1 |
| GO:0030423 | targeting of mRNA for destruction involv... | 3 | 0 | 0.11 | 1 |
| GO:0030431 | sleep | 29 | 0 | 1.1 | 1 |
| GO:0030432 | peristalsis | 9 | 0 | 0.34 | 1 |
| GO:0030433 | ER-associated ubiquitin-dependent protei... | 44 | 0 | 1.67 | 1 |
| GO:0030450 | regulation of complement activation | cla... | 4 | 0 | 0.15 |
| GO:0030451 | regulation of complement activation | alt... | 2 | 0 | 0.08 |
| GO:0030472 | mitotic spindle organization in nucleus | 1 | 0 | 0.04 | 1 |
| GO:0030488 | tRNA methylation | 11 | 0 | 0.42 | 1 |
| GO:0030497 | fatty acid elongation | 8 | 0 | 0.3 | 1 |
| GO:0030505 | inorganic diphosphate transport | 2 | 0 | 0.08 | 1 |
| GO:0030511 | positive regulation of transforming grow... | 23 | 0 | 0.88 | 1 |
| GO:0030513 | positive regulation of BMP signaling pat... | 27 | 0 | 1.03 | 1 |
| GO:0030520 | intracellular estrogen receptor signalin... | 42 | 0 | 1.6 | 1 |
| GO:0030538 | embryonic genitalia morphogenesis | 4 | 0 | 0.15 | 1 |
| GO:0030573 | bile acid catabolic process | 3 | 0 | 0.11 | 1 |
| GO:0030575 | nuclear body organization | 6 | 0 | 0.23 | 1 |
| GO:0030576 | Cajal body organization | 1 | 0 | 0.04 | 1 |
| GO:0030578 | PML body organization | 4 | 0 | 0.15 | 1 |
| GO:0030579 | ubiquitin-dependent SMAD protein catabol | 6 | 0 | 0.23 | 1 |
| GO:0030581 | symbiont intracellular protein transport... | 5 | 0 | 0.19 | 1 |
| GO:0030638 | polyketide metabolic process | 8 | 0 | 0.3 | 1 |
| GO:0030643 | cellular phosphate ion homeostasis | 6 | 0 | 0.23 | 1 |
| GO:0030644 | cellular chloride ion homeostasis | 5 | 0 | 0.19 | 1 |
| GO:0030656 | regulation of vitamin metabolic process | 12 | 0 | 0.46 | 1 |
| GO:0030682 | evasion or tolerance of host defense res... | 1 | 0 | 0.04 | 1 |
| GO:0030683 | evasion or tolerance by virus of host im... | 1 | 0 | 0.04 | 1 |
| GO:0030704 | vitelline membrane formation | 1 | 0 | 0.04 | 1 |
| GO:0030719 | P granule organization | 2 | 0 | 0.08 | 1 |
| GO:0030730 | sequestering of triglyceride | 14 | 0 | 0.53 | 1 |
| GO:0030805 | regulation of cyclic nucleotide cataboli... | 4 | 0 | 0.15 | 1 |
| GO:0030806 | negative regulation of cyclic nucleotide... | 2 | 0 | 0.08 | 1 |
| GO:0030807 | positive regulation of cyclic nucleotide... | 2 | 0 | 0.08 | 1 |
| GO:0030811 | regulation of nucleotide catabolic proce... | 5 | 0 | 0.19 | 1 |
| GO:0030812 | negative regulation of nucleotide catabo... | 3 | 0 | 0.11 | 1 |
| GO:0030813 | positive regulation of nucleotide catabo... | 2 | 0 | 0.08 | 1 |
| GO:0030820 | regulation of cAMP catabolic process | 4 | 0 | 0.15 | 1 |
| GO:0030821 | negative regulation of cAMP catabolic pr... | 2 | 0 | 0.08 | 1 |
| GO:0030822 | positive regulation of cAMP catabolic pr... | 2 | 0 | 0.08 | 1 |
| GO:0030823 | regulation of cGMP metabolic process | 27 | 0 | 1.03 | 1 |
| GO:0030824 | negative regulation of cGMP metabolic pr... | 1 | 0 | 0.04 | 1 |
| GO:0030825 | positive regulation of cGMP metabolic pr... | 20 | 0 | 0.76 | 1 |
| GO:0030826 | regulation of cGMP biosynthetic process | 20 | 0 | 0.76 | 1 |
| GO:0030827 | negative regulation of cGMP biosynthetic... | 1 | 0 | 0.04 | 1 |
| GO:0030828 | positive regulation of cGMP biosynthetic... | 18 | 0 | 0.69 | 1 |
| GO:0030836 | positive regulation of actin filament de... | 8 | 0 | 0.3 | 1 |

| | | | | | |
|---|---|---|---|---|---|
| GO:0030842 | regulation of intermediate filament depo... | 1 | 0 | 0.04 | 1 |
| GO:0030844 | positive regulation of intermediate fila... | 1 | 0 | 0.04 | 1 |
| GO:0030845 | phospholipase C-inhibiting G-protein cou... | 1 | 0 | 0.04 | 1 |
| GO:0030846 | termination of RNA polymerase II transcr... | 1 | 0 | 0.04 | 1 |
| GO:0030853 | negative regulation of granulocyte diffe... | 7 | 0 | 0.27 | 1 |
| GO:0030859 | polarized epithelial cell differentiatio... | 9 | 0 | 0.34 | 1 |
| GO:0030860 | regulation of polarized epithelial cell ... | 2 | 0 | 0.08 | 1 |
| GO:0030862 | positive regulation of polarized epithel... | 2 | 0 | 0.08 | 1 |
| GO:0030865 | cortical cytoskeleton organization | 22 | 0 | 0.84 | 1 |
| GO:0030866 | cortical actin cytoskeleton organization | 19 | 0 | 0.72 | 1 |
| GO:0030878 | thyroid gland development | 18 | 0 | 0.69 | 1 |
| GO:0030885 | regulation of myeloid dendritic cell act... | 5 | 0 | 0.19 | 1 |
| GO:0030886 | negative regulation of myeloid dendritic... | 3 | 0 | 0.11 | 1 |
| GO:0030887 | positive regulation of myeloid dendritic... | 1 | 0 | 0.04 | 1 |
| GO:0030903 | notochord development | 16 | 0 | 0.61 | 1 |
| GO:0030908 | protein splicing | 2 | 0 | 0.08 | 1 |
| GO:0030910 | olfactory placode formation | 6 | 0 | 0.23 | 1 |
| GO:0030948 | negative regulation of vascular endothel... | 7 | 0 | 0.27 | 1 |
| GO:0030950 | establishment or maintenance of actin cy... | 2 | 0 | 0.08 | 1 |
| GO:0030951 | establishment or maintenance of microtub... | 3 | 0 | 0.11 | 1 |
| GO:0030952 | establishment or maintenance of cytoskel... | 5 | 0 | 0.19 | 1 |
| GO:0030953 | astral microtubule organization | 4 | 0 | 0.15 | 1 |
| GO:0030969 | UFP-specific transcription factor mRNA p... | 1 | 0 | 0.04 | 1 |
| GO:0030970 | retrograde protein transport | ER to cyto... | 4 | 0 | 0.15 |
| GO:0030997 | regulation of centriole-centriole cohesi... | 3 | 0 | 0.11 | 1 |
| GO:0030999 | linear element assembly | 1 | 0 | 0.04 | 1 |
| GO:0031000 | response to caffeine | 15 | 0 | 0.57 | 1 |
| GO:0031017 | exocrine pancreas development | 7 | 0 | 0.27 | 1 |
| GO:0031022 | nuclear migration along microfilament | 1 | 0 | 0.04 | 1 |
| GO:0031038 | myosin II filament organization | 2 | 0 | 0.08 | 1 |
| GO:0031049 | programmed DNA elimination | 1 | 0 | 0.04 | 1 |
| GO:0031052 | chromosome breakage | 1 | 0 | 0.04 | 1 |
| GO:0031053 | primary miRNA processing | 4 | 0 | 0.15 | 1 |
| GO:0031064 | negative regulation of histone deacetyla... | 4 | 0 | 0.15 | 1 |
| GO:0031077 | post-embryonic camera-type eye developm | 7 | 0 | 0.27 | 1 |
| GO:0031086 | nuclear-transcribed mRNA catabolic proce | 2 | 0 | 0.08 | 1 |
| GO:0031087 | deadenylation-independent decapping of r | 2 | 0 | 0.08 | 1 |
| GO:0031104 | dendrite regeneration | 1 | 0 | 0.04 | 1 |
| GO:0031106 | septin ring organization | 1 | 0 | 0.04 | 1 |
| GO:0031112 | positive regulation of microtubule polym... | 15 | 0 | 0.57 | 1 |
| GO:0031113 | regulation of microtubule polymerization | 21 | 0 | 0.8 | 1 |
| GO:0031115 | negative regulation of microtubule polym... | 9 | 0 | 0.34 | 1 |
| GO:0031116 | positive regulation of microtubule polym... | 11 | 0 | 0.42 | 1 |
| GO:0031117 | positive regulation of microtubule depol... | 4 | 0 | 0.15 | 1 |
| GO:0031118 | rRNA pseudouridine synthesis | 2 | 0 | 0.08 | 1 |
| GO:0031119 | tRNA pseudouridine synthesis | 2 | 0 | 0.08 | 1 |
| GO:0031120 | snRNA pseudouridine synthesis | 1 | 0 | 0.04 | 1 |
| GO:0031122 | cytoplasmic microtubule organization | 29 | 0 | 1.1 | 1 |
| GO:0031125 | rRNA 3'-end processing | 6 | 0 | 0.23 | 1 |
| GO:0031126 | snoRNA 3'-end processing | 4 | 0 | 0.15 | 1 |
| GO:0031128 | developmental induction | 36 | 0 | 1.37 | 1 |
| GO:0031129 | inductive cell-cell signaling | 1 | 0 | 0.04 | 1 |
| GO:0031134 | sister chromatid biorientation | 1 | 0 | 0.04 | 1 |

| | | | | | |
|---|---|---|---|---|---|
| GO:0031161 | phosphatidylinositol catabolic process | 1 | 0 | 0.04 | 1 |
| GO:0031163 | metallo-sulfur cluster assembly | 17 | 0 | 0.65 | 1 |
| GO:0031179 | peptide modification | 2 | 0 | 0.08 | 1 |
| GO:0031204 | posttranslational protein targeting to m... | 1 | 0 | 0.04 | 1 |
| GO:0031247 | actin rod assembly | 2 | 0 | 0.08 | 1 |
| GO:0031268 | pseudopodium organization | 14 | 0 | 0.53 | 1 |
| GO:0031269 | pseudopodium assembly | 13 | 0 | 0.49 | 1 |
| GO:0031272 | regulation of pseudopodium assembly | 12 | 0 | 0.46 | 1 |
| GO:0031274 | positive regulation of pseudopodium asse. | 12 | 0 | 0.46 | 1 |
| GO:0031282 | regulation of guanylate cyclase activity | 12 | 0 | 0.46 | 1 |
| GO:0031283 | negative regulation of guanylate cyclase... | 1 | 0 | 0.04 | 1 |
| GO:0031284 | positive regulation of guanylate cyclase... | 11 | 0 | 0.42 | 1 |
| GO:0031289 | actin phosphorylation | 1 | 0 | 0.04 | 1 |
| GO:0031296 | B cell costimulation | 3 | 0 | 0.11 | 1 |
| GO:0031297 | replication fork processing | 10 | 0 | 0.38 | 1 |
| GO:0031335 | regulation of sulfur amino acid metaboli... | 2 | 0 | 0.08 | 1 |
| GO:0031337 | positive regulation of sulfur amino acid... | 1 | 0 | 0.04 | 1 |
| GO:0031338 | regulation of vesicle fusion | 20 | 0 | 0.76 | 1 |
| GO:0031339 | negative regulation of vesicle fusion | 5 | 0 | 0.19 | 1 |
| GO:0031340 | positive regulation of vesicle fusion | 7 | 0 | 0.27 | 1 |
| GO:0031392 | regulation of prostaglandin biosynthetic... | 6 | 0 | 0.23 | 1 |
| GO:0031393 | negative regulation of prostaglandin bio... | 1 | 0 | 0.04 | 1 |
| GO:0031394 | positive regulation of prostaglandin bio... | 5 | 0 | 0.19 | 1 |
| GO:0031407 | oxylipin metabolic process | 1 | 0 | 0.04 | 1 |
| GO:0031424 | keratinization | 48 | 0 | 1.83 | 1 |
| GO:0031427 | response to methotrexate | 5 | 0 | 0.19 | 1 |
| GO:0031438 | negative regulation of mRNA cleavage | 1 | 0 | 0.04 | 1 |
| GO:0031440 | regulation of mRNA 3'-end processing | 24 | 0 | 0.91 | 1 |
| GO:0031441 | negative regulation of mRNA 3'-end proce. | 9 | 0 | 0.34 | 1 |
| GO:0031442 | positive regulation of mRNA 3'-end proce.. | 15 | 0 | 0.57 | 1 |
| GO:0031443 | fast-twitch skeletal muscle fiber contra... | 1 | 0 | 0.04 | 1 |
| GO:0031444 | slow-twitch skeletal muscle fiber contra... | 2 | 0 | 0.08 | 1 |
| GO:0031445 | regulation of heterochromatin assembly | 1 | 0 | 0.04 | 1 |
| GO:0031446 | regulation of fast-twitch skeletal muscl... | 1 | 0 | 0.04 | 1 |
| GO:0031448 | positive regulation of fast-twitch skele... | 1 | 0 | 0.04 | 1 |
| GO:0031453 | positive regulation of heterochromatin a... | 1 | 0 | 0.04 | 1 |
| GO:0031455 | glycine betaine metabolic process | 2 | 0 | 0.08 | 1 |
| GO:0031456 | glycine betaine biosynthetic process | 2 | 0 | 0.08 | 1 |
| GO:0031468 | nuclear envelope reassembly | 13 | 0 | 0.49 | 1 |
| GO:0031507 | heterochromatin assembly | 8 | 0 | 0.3 | 1 |
| GO:0031508 | pericentric heterochromatin assembly | 2 | 0 | 0.08 | 1 |
| GO:0031529 | ruffle organization | 35 | 0 | 1.33 | 1 |
| GO:0031536 | positive regulation of exit from mitosis | 4 | 0 | 0.15 | 1 |
| GO:0031547 | brain-derived neurotrophic factor recept... | 3 | 0 | 0.11 | 1 |
| GO:0031554 | regulation of DNA-templated transcriptio... | 2 | 0 | 0.08 | 1 |
| GO:0031580 | membrane raft distribution | 4 | 0 | 0.15 | 1 |
| GO:0031581 | hemidesmosome assembly | 11 | 0 | 0.42 | 1 |
| GO:0031584 | activation of phospholipase D activity | 5 | 0 | 0.19 | 1 |
| GO:0031585 | regulation of inositol 1 | 4 | 5-trisphosph | 5 | 0 |
| GO:0031587 | positive regulation of inositol 1 | 4 | 5-tr... | 3 | 0 |
| GO:0031620 | regulation of fever generation | 8 | 0 | 0.3 | 1 |
| GO:0031622 | positive regulation of fever generation | 7 | 0 | 0.27 | 1 |
| GO:0031623 | receptor internalization | 65 | 0 | 2.47 | 1 |

| | | | | | |
|---|---|---|---|---|---|
| GO:0031629 | synaptic vesicle fusion to presynaptic m... | 15 | 0 | 0.57 | 1 |
| GO:0031630 | regulation of synaptic vesicle fusion to... | 3 | 0 | 0.11 | 1 |
| GO:0031639 | plasminogen activation | 16 | 0 | 0.61 | 1 |
| GO:0031640 | killing of cells of other organism | 29 | 0 | 1.1 | 1 |
| GO:0031650 | regulation of heat generation | 10 | 0 | 0.38 | 1 |
| GO:0031651 | negative regulation of heat generation | 1 | 0 | 0.04 | 1 |
| GO:0031652 | positive regulation of heat generation | 8 | 0 | 0.3 | 1 |
| GO:0031659 | positive regulation of cyclin-dependent ... | 7 | 0 | 0.27 | 1 |
| GO:0031664 | regulation of lipopolysaccharide-mediate... | 17 | 0 | 0.65 | 1 |
| GO:0031665 | negative regulation of lipopolysaccharid... | 7 | 0 | 0.27 | 1 |
| GO:0031666 | positive regulation of lipopolysaccharid... | 7 | 0 | 0.27 | 1 |
| GO:0031914 | negative regulation of synaptic plastici... | 3 | 0 | 0.11 | 1 |
| GO:0031915 | positive regulation of synaptic plastici... | 5 | 0 | 0.19 | 1 |
| GO:0031930 | mitochondria-nucleus signaling pathway | 1 | 0 | 0.04 | 1 |
| GO:0031935 | regulation of chromatin silencing | 13 | 0 | 0.49 | 1 |
| GO:0031936 | negative regulation of chromatin silenci... | 9 | 0 | 0.34 | 1 |
| GO:0031937 | positive regulation of chromatin silenci... | 3 | 0 | 0.11 | 1 |
| GO:0031943 | regulation of glucocorticoid metabolic p... | 9 | 0 | 0.34 | 1 |
| GO:0031944 | negative regulation of glucocorticoid me... | 5 | 0 | 0.19 | 1 |
| GO:0031945 | positive regulation of glucocorticoid me... | 1 | 0 | 0.04 | 1 |
| GO:0031946 | regulation of glucocorticoid biosyntheti... | 7 | 0 | 0.27 | 1 |
| GO:0031947 | negative regulation of glucocorticoid bi... | 5 | 0 | 0.19 | 1 |
| GO:0031948 | positive regulation of glucocorticoid bi... | 1 | 0 | 0.04 | 1 |
| GO:0031953 | negative regulation of protein autophosp... | 9 | 0 | 0.34 | 1 |
| GO:0031958 | corticosteroid receptor signaling pathwa... | 15 | 0 | 0.57 | 1 |
| GO:0031959 | mineralocorticoid receptor signaling pat... | 1 | 0 | 0.04 | 1 |
| GO:0031987 | locomotion involved in locomotory behavi.. | 9 | 0 | 0.34 | 1 |
| GO:0031989 | bombesin receptor signaling pathway | 3 | 0 | 0.11 | 1 |
| GO:0031990 | mRNA export from nucleus in response to | 2 | 0 | 0.08 | 1 |
| GO:0031999 | negative regulation of fatty acid beta-o... | 3 | 0 | 0.11 | 1 |
| GO:0032020 | ISG15-protein conjugation | 6 | 0 | 0.23 | 1 |
| GO:0032023 | trypsinogen activation | 1 | 0 | 0.04 | 1 |
| GO:0032024 | positive regulation of insulin secretion | 55 | 0 | 2.09 | 1 |
| GO:0032025 | response to cobalt ion | 6 | 0 | 0.23 | 1 |
| GO:0032042 | mitochondrial DNA metabolic process | 15 | 0 | 0.57 | 1 |
| GO:0032048 | cardiolipin metabolic process | 13 | 0 | 0.49 | 1 |
| GO:0032049 | cardiolipin biosynthetic process | 6 | 0 | 0.23 | 1 |
| GO:0032055 | negative regulation of translation in re... | 7 | 0 | 0.27 | 1 |
| GO:0032056 | positive regulation of translation in re... | 2 | 0 | 0.08 | 1 |
| GO:0032057 | negative regulation of translational ini... | 5 | 0 | 0.19 | 1 |
| GO:0032058 | positive regulation of translational ini... | 1 | 0 | 0.04 | 1 |
| GO:0032066 | nucleolus to nucleoplasm transport | 3 | 0 | 0.11 | 1 |
| GO:0032070 | regulation of deoxyribonuclease activity | 4 | 0 | 0.15 | 1 |
| GO:0032071 | regulation of endodeoxyribonuclease acti.. | 3 | 0 | 0.11 | 1 |
| GO:0032074 | negative regulation of nuclease activity | 3 | 0 | 0.11 | 1 |
| GO:0032076 | negative regulation of deoxyribonuclease.. | 1 | 0 | 0.04 | 1 |
| GO:0032077 | positive regulation of deoxyribonuclease... | 1 | 0 | 0.04 | 1 |
| GO:0032078 | negative regulation of endodeoxyribonucl.. | 1 | 0 | 0.04 | 1 |
| GO:0032091 | negative regulation of protein binding | 57 | 0 | 2.17 | 1 |
| GO:0032113 | regulation of carbohydrate phosphatase a. | 1 | 0 | 0.04 | 1 |
| GO:0032119 | sequestering of zinc ion | 8 | 0 | 0.3 | 1 |
| GO:0032185 | septin cytoskeleton organization | 1 | 0 | 0.04 | 1 |
| GO:0032196 | transposition | 11 | 0 | 0.42 | 1 |

| | | | | | | |
|---|---|---|---|---|---|---|
| GO:0032201 | telomere maintenance via semi-conservati | | 25 | 0 | 0.95 | 1 |
| GO:0032202 | telomere assembly | | 3 | 0 | 0.11 | 1 |
| GO:0032204 | regulation of telomere maintenance | | 16 | 0 | 0.61 | 1 |
| GO:0032205 | negative regulation of telomere maintena.. | | 10 | 0 | 0.38 | 1 |
| GO:0032206 | positive regulation of telomere maintena... | | 7 | 0 | 0.27 | 1 |
| GO:0032210 | regulation of telomere maintenance via t... | | 8 | 0 | 0.3 | 1 |
| GO:0032211 | negative regulation of telomere maintena.. | | 6 | 0 | 0.23 | 1 |
| GO:0032212 | positive regulation of telomere maintena... | | 3 | 0 | 0.11 | 1 |
| GO:0032213 | regulation of telomere maintenance via s... | | 2 | 0 | 0.08 | 1 |
| GO:0032214 | negative regulation of telomere maintena.. | | 2 | 0 | 0.08 | 1 |
| GO:0032222 | regulation of synaptic transmission | cho... | | 11 | 0 | 0.42 |
| GO:0032223 | negative regulation of synaptic transmis... | | 2 | 0 | 0.08 | 1 |
| GO:0032224 | positive regulation of synaptic transmis... | | 6 | 0 | 0.23 | 1 |
| GO:0032225 | regulation of synaptic transmission | dop... | | 16 | 0 | 0.61 |
| GO:0032226 | positive regulation of synaptic transmis... | | 7 | 0 | 0.27 | 1 |
| GO:0032227 | negative regulation of synaptic transmis... | | 3 | 0 | 0.11 | 1 |
| GO:0032228 | regulation of synaptic transmission | GAB... | | 31 | 0 | 1.18 |
| GO:0032229 | negative regulation of synaptic transmis... | | 6 | 0 | 0.23 | 1 |
| GO:0032230 | positive regulation of synaptic transmis... | | 13 | 0 | 0.49 | 1 |
| GO:0032233 | positive regulation of actin filament bu... | | 41 | 0 | 1.56 | 1 |
| GO:0032237 | activation of store-operated calcium cha... | | 4 | 0 | 0.15 | 1 |
| GO:0032239 | regulation of nucleobase-containing comp. | | 12 | 0 | 0.46 | 1 |
| GO:0032240 | negative regulation of nucleobase-contai... | | 2 | 0 | 0.08 | 1 |
| GO:0032241 | positive regulation of nucleobase-contai... | | 3 | 0 | 0.11 | 1 |
| GO:0032242 | regulation of nucleoside transport | | 2 | 0 | 0.08 | 1 |
| GO:0032244 | positive regulation of nucleoside transp... | | 1 | 0 | 0.04 | 1 |
| GO:0032252 | secretory granule localization | | 4 | 0 | 0.15 | 1 |
| GO:0032258 | CVT pathway | | 1 | 0 | 0.04 | 1 |
| GO:0032261 | purine nucleotide salvage | | 7 | 0 | 0.27 | 1 |
| GO:0032262 | pyrimidine nucleotide salvage | | 5 | 0 | 0.19 | 1 |
| GO:0032263 | GMP salvage | | 1 | 0 | 0.04 | 1 |
| GO:0032264 | IMP salvage | | 4 | 0 | 0.15 | 1 |
| GO:0032277 | negative regulation of gonadotropin secr... | | 7 | 0 | 0.27 | 1 |
| GO:0032287 | peripheral nervous system myelin mainten | | 7 | 0 | 0.27 | 1 |
| GO:0032289 | central nervous system myelin formation | | 2 | 0 | 0.08 | 1 |
| GO:0032290 | peripheral nervous system myelin formati.. | | 1 | 0 | 0.04 | 1 |
| GO:0032298 | positive regulation of DNA-dependent DNA | | 1 | 0 | 0.04 | 1 |
| GO:0032324 | molybdopterin cofactor biosynthetic proc... | | 7 | 0 | 0.27 | 1 |
| GO:0032329 | serine transport | | 9 | 0 | 0.34 | 1 |
| GO:0032345 | negative regulation of aldosterone metab.. | | 4 | 0 | 0.15 | 1 |
| GO:0032348 | negative regulation of aldosterone biosy... | | 4 | 0 | 0.15 | 1 |
| GO:0032351 | negative regulation of hormone metabolic.. | | 9 | 0 | 0.34 | 1 |
| GO:0032353 | negative regulation of hormone biosynthe.. | | 8 | 0 | 0.3 | 1 |
| GO:0032354 | response to follicle-stimulating hormone | | 11 | 0 | 0.42 | 1 |
| GO:0032361 | pyridoxal phosphate catabolic process | | 1 | 0 | 0.04 | 1 |
| GO:0032364 | oxygen homeostasis | | 6 | 0 | 0.23 | 1 |
| GO:0032372 | negative regulation of sterol transport | | 12 | 0 | 0.46 | 1 |
| GO:0032373 | positive regulation of sterol transport | | 16 | 0 | 0.61 | 1 |
| GO:0032375 | negative regulation of cholesterol trans... | | 12 | 0 | 0.46 | 1 |
| GO:0032376 | positive regulation of cholesterol trans... | | 16 | 0 | 0.61 | 1 |
| GO:0032379 | positive regulation of intracellular lip... | | 1 | 0 | 0.04 | 1 |
| GO:0032382 | positive regulation of intracellular ste... | | 1 | 0 | 0.04 | 1 |
| GO:0032385 | positive regulation of intracellular cho... | | 1 | 0 | 0.04 | 1 |

| | | | | | |
|---|---|---|---|---|---|
| GO:0032400 | melanosome localization | 26 | 0 | 0.99 | 1 |
| GO:0032401 | establishment of melanosome localization | 23 | 0 | 0.88 | 1 |
| GO:0032402 | melanosome transport | 22 | 0 | 0.84 | 1 |
| GO:0032410 | negative regulation of transporter activ... | 47 | 0 | 1.79 | 1 |
| GO:0032413 | negative regulation of ion transmembrane. | 39 | 0 | 1.48 | 1 |
| GO:0032416 | negative regulation of sodium:proton ant... | 2 | 0 | 0.08 | 1 |
| GO:0032423 | regulation of mismatch repair | 2 | 0 | 0.08 | 1 |
| GO:0032425 | positive regulation of mismatch repair | 1 | 0 | 0.04 | 1 |
| GO:0032429 | regulation of phospholipase A2 activity | 10 | 0 | 0.38 | 1 |
| GO:0032430 | positive regulation of phospholipase A2 ... | 5 | 0 | 0.19 | 1 |
| GO:0032431 | activation of phospholipase A2 activity | 4 | 0 | 0.15 | 1 |
| GO:0032439 | endosome localization | 2 | 0 | 0.08 | 1 |
| GO:0032447 | protein urmylation | 3 | 0 | 0.11 | 1 |
| GO:0032455 | nerve growth factor processing | 4 | 0 | 0.15 | 1 |
| GO:0032456 | endocytic recycling | 19 | 0 | 0.72 | 1 |
| GO:0032458 | slow endocytic recycling | 1 | 0 | 0.04 | 1 |
| GO:0032459 | regulation of protein oligomerization | 31 | 0 | 1.18 | 1 |
| GO:0032460 | negative regulation of protein oligomeri... | 11 | 0 | 0.42 | 1 |
| GO:0032461 | positive regulation of protein oligomeri... | 19 | 0 | 0.72 | 1 |
| GO:0032462 | regulation of protein homooligomerizatio... | 14 | 0 | 0.53 | 1 |
| GO:0032463 | negative regulation of protein homooligo... | 6 | 0 | 0.23 | 1 |
| GO:0032464 | positive regulation of protein homooligo... | 8 | 0 | 0.3 | 1 |
| GO:0032466 | negative regulation of cytokinesis | 7 | 0 | 0.27 | 1 |
| GO:0032468 | Golgi calcium ion homeostasis | 1 | 0 | 0.04 | 1 |
| GO:0032470 | positive regulation of endoplasmic retic... | 2 | 0 | 0.08 | 1 |
| GO:0032472 | Golgi calcium ion transport | 2 | 0 | 0.08 | 1 |
| GO:0032474 | otolith morphogenesis | 2 | 0 | 0.08 | 1 |
| GO:0032490 | detection of molecule of bacterial origi... | 9 | 0 | 0.34 | 1 |
| GO:0032493 | response to bacterial lipoprotein | 5 | 0 | 0.19 | 1 |
| GO:0032494 | response to peptidoglycan | 11 | 0 | 0.42 | 1 |
| GO:0032495 | response to muramyl dipeptide | 12 | 0 | 0.46 | 1 |
| GO:0032497 | detection of lipopolysaccharide | 4 | 0 | 0.15 | 1 |
| GO:0032498 | detection of muramyl dipeptide | 1 | 0 | 0.04 | 1 |
| GO:0032499 | detection of peptidoglycan | 2 | 0 | 0.08 | 1 |
| GO:0032506 | cytokinetic process | 13 | 0 | 0.49 | 1 |
| GO:0032509 | endosome transport via multivesicular bo.. | 6 | 0 | 0.23 | 1 |
| GO:0032510 | endosome to lysosome transport via multi. | 2 | 0 | 0.08 | 1 |
| GO:0032511 | late endosome to vacuole transport via m.. | 3 | 0 | 0.11 | 1 |
| GO:0032512 | regulation of protein phosphatase type 2... | 4 | 0 | 0.15 | 1 |
| GO:0032513 | negative regulation of protein phosphata... | 3 | 0 | 0.11 | 1 |
| GO:0032514 | positive regulation of protein phosphata... | 1 | 0 | 0.04 | 1 |
| GO:0032515 | negative regulation of phosphoprotein ph.. | 10 | 0 | 0.38 | 1 |
| GO:0032516 | positive regulation of phosphoprotein ph... | 13 | 0 | 0.49 | 1 |
| GO:0032525 | somite rostral/caudal axis specification | 10 | 0 | 0.38 | 1 |
| GO:0032527 | protein exit from endoplasmic reticulum | 6 | 0 | 0.23 | 1 |
| GO:0032532 | regulation of microvillus length | 1 | 0 | 0.04 | 1 |
| GO:0032571 | response to vitamin K | 3 | 0 | 0.11 | 1 |
| GO:0032581 | ER-dependent peroxisome organization | 1 | 0 | 0.04 | 1 |
| GO:0032594 | protein transport within lipid bilayer | 7 | 0 | 0.27 | 1 |
| GO:0032595 | B cell receptor transport within lipid b... | 1 | 0 | 0.04 | 1 |
| GO:0032596 | protein transport into membrane raft | 6 | 0 | 0.23 | 1 |
| GO:0032597 | B cell receptor transport into membrane ... | 1 | 0 | 0.04 | 1 |
| GO:0032599 | protein transport out of membrane raft | 1 | 0 | 0.04 | 1 |

| | | | | | |
|---|---|---|---|---|---|
| GO:0032600 | chemokine receptor transport out of memb | 1 | 0 | 0.04 | 1 |
| GO:0032601 | connective tissue growth factor producti... | 2 | 0 | 0.08 | 1 |
| GO:0032602 | chemokine production | 61 | 0 | 2.32 | 1 |
| GO:0032603 | fractalkine production | 1 | 0 | 0.04 | 1 |
| GO:0032604 | granulocyte macrophage colony-stimulatin | 13 | 0 | 0.49 | 1 |
| GO:0032605 | hepatocyte growth factor production | 1 | 0 | 0.04 | 1 |
| GO:0032607 | interferon-alpha production | 16 | 0 | 0.61 | 1 |
| GO:0032610 | interleukin-1 alpha production | 7 | 0 | 0.27 | 1 |
| GO:0032611 | interleukin-1 beta production | 45 | 0 | 1.71 | 1 |
| GO:0032612 | interleukin-1 production | 51 | 0 | 1.94 | 1 |
| GO:0032615 | interleukin-12 production | 47 | 0 | 1.79 | 1 |
| GO:0032618 | interleukin-15 production | 2 | 0 | 0.08 | 1 |
| GO:0032620 | interleukin-17 production | 22 | 0 | 0.84 | 1 |
| GO:0032621 | interleukin-18 production | 6 | 0 | 0.23 | 1 |
| GO:0032625 | interleukin-21 production | 1 | 0 | 0.04 | 1 |
| GO:0032627 | interleukin-23 production | 7 | 0 | 0.27 | 1 |
| GO:0032632 | interleukin-3 production | 3 | 0 | 0.11 | 1 |
| GO:0032639 | TRAIL production | 2 | 0 | 0.08 | 1 |
| GO:0032642 | regulation of chemokine production | 53 | 0 | 2.02 | 1 |
| GO:0032643 | regulation of connective tissue growth f... | 1 | 0 | 0.04 | 1 |
| GO:0032644 | regulation of fractalkine production | 1 | 0 | 0.04 | 1 |
| GO:0032645 | regulation of granulocyte macrophage col... | 13 | 0 | 0.49 | 1 |
| GO:0032646 | regulation of hepatocyte growth factor p... | 1 | 0 | 0.04 | 1 |
| GO:0032647 | regulation of interferon-alpha productio... | 15 | 0 | 0.57 | 1 |
| GO:0032650 | regulation of interleukin-1 alpha produc... | 6 | 0 | 0.23 | 1 |
| GO:0032651 | regulation of interleukin-1 beta product... | 38 | 0 | 1.45 | 1 |
| GO:0032652 | regulation of interleukin-1 production | 43 | 0 | 1.64 | 1 |
| GO:0032655 | regulation of interleukin-12 production | 46 | 0 | 1.75 | 1 |
| GO:0032658 | regulation of interleukin-15 production | 1 | 0 | 0.04 | 1 |
| GO:0032660 | regulation of interleukin-17 production | 20 | 0 | 0.76 | 1 |
| GO:0032661 | regulation of interleukin-18 production | 5 | 0 | 0.19 | 1 |
| GO:0032667 | regulation of interleukin-23 production | 7 | 0 | 0.27 | 1 |
| GO:0032672 | regulation of interleukin-3 production | 2 | 0 | 0.08 | 1 |
| GO:0032679 | regulation of TRAIL production | 2 | 0 | 0.08 | 1 |
| GO:0032681 | regulation of lymphotoxin A production | 1 | 0 | 0.04 | 1 |
| GO:0032682 | negative regulation of chemokine product... | 12 | 0 | 0.46 | 1 |
| GO:0032686 | negative regulation of hepatocyte growth... | 1 | 0 | 0.04 | 1 |
| GO:0032687 | negative regulation of interferon-alpha ... | 1 | 0 | 0.04 | 1 |
| GO:0032688 | negative regulation of interferon-beta p... | 8 | 0 | 0.3 | 1 |
| GO:0032691 | negative regulation of interleukin-1 bet... | 9 | 0 | 0.34 | 1 |
| GO:0032692 | negative regulation of interleukin-1 pro... | 11 | 0 | 0.42 | 1 |
| GO:0032695 | negative regulation of interleukin-12 pr... | 15 | 0 | 0.57 | 1 |
| GO:0032700 | negative regulation of interleukin-17 pr... | 10 | 0 | 0.38 | 1 |
| GO:0032701 | negative regulation of interleukin-18 pr... | 1 | 0 | 0.04 | 1 |
| GO:0032707 | negative regulation of interleukin-23 pr... | 2 | 0 | 0.08 | 1 |
| GO:0032713 | negative regulation of interleukin-4 pro... | 6 | 0 | 0.23 | 1 |
| GO:0032717 | negative regulation of interleukin-8 pro... | 11 | 0 | 0.42 | 1 |
| GO:0032720 | negative regulation of tumor necrosis fa... | 32 | 0 | 1.22 | 1 |
| GO:0032722 | positive regulation of chemokine product... | 40 | 0 | 1.52 | 1 |
| GO:0032723 | positive regulation of connective tissue... | 1 | 0 | 0.04 | 1 |
| GO:0032724 | positive regulation of fractalkine produ... | 1 | 0 | 0.04 | 1 |
| GO:0032725 | positive regulation of granulocyte macro... | 12 | 0 | 0.46 | 1 |
| GO:0032727 | positive regulation of interferon-alpha ... | 14 | 0 | 0.53 | 1 |

| | | | | | |
|---|---|---|---|---|---|
| GO:0032730 | positive regulation of interleukin-1 alp... | 6 | 0 | 0.23 | 1 |
| GO:0032731 | positive regulation of interleukin-1 bet... | 25 | 0 | 0.95 | 1 |
| GO:0032732 | positive regulation of interleukin-1 pro... | 29 | 0 | 1.1 | 1 |
| GO:0032735 | positive regulation of interleukin-12 pr... | 30 | 0 | 1.14 | 1 |
| GO:0032736 | positive regulation of interleukin-13 pr... | 9 | 0 | 0.34 | 1 |
| GO:0032738 | positive regulation of interleukin-15 pr... | 1 | 0 | 0.04 | 1 |
| GO:0032740 | positive regulation of interleukin-17 pr... | 11 | 0 | 0.42 | 1 |
| GO:0032741 | positive regulation of interleukin-18 pr... | 3 | 0 | 0.11 | 1 |
| GO:0032747 | positive regulation of interleukin-23 pr... | 5 | 0 | 0.19 | 1 |
| GO:0032752 | positive regulation of interleukin-3 pro... | 2 | 0 | 0.08 | 1 |
| GO:0032755 | positive regulation of interleukin-6 pro... | 52 | 0 | 1.98 | 1 |
| GO:0032759 | positive regulation of TRAIL production | 2 | 0 | 0.08 | 1 |
| GO:0032761 | positive regulation of lymphotoxin A pro... | 1 | 0 | 0.04 | 1 |
| GO:0032764 | negative regulation of mast cell cytokin... | 2 | 0 | 0.08 | 1 |
| GO:0032768 | regulation of monooxygenase activity | 53 | 0 | 2.02 | 1 |
| GO:0032769 | negative regulation of monooxygenase act | 12 | 0 | 0.46 | 1 |
| GO:0032770 | positive regulation of monooxygenase act. | 26 | 0 | 0.99 | 1 |
| GO:0032771 | regulation of monophenol monooxygenase | 1 | 0 | 0.04 | 1 |
| GO:0032773 | positive regulation of monophenol monoox | 1 | 0 | 0.04 | 1 |
| GO:0032776 | DNA methylation on cytosine | 35 | 0 | 1.33 | 1 |
| GO:0032780 | negative regulation of ATPase activity | 10 | 0 | 0.38 | 1 |
| GO:0032781 | positive regulation of ATPase activity | 29 | 0 | 1.1 | 1 |
| GO:0032782 | bile acid secretion | 6 | 0 | 0.23 | 1 |
| GO:0032785 | negative regulation of DNA-templated tra.. | 9 | 0 | 0.34 | 1 |
| GO:0032788 | saturated monocarboxylic acid metabolic .. | 1 | 0 | 0.04 | 1 |
| GO:0032789 | unsaturated monocarboxylic acid metaboli | 1 | 0 | 0.04 | 1 |
| GO:0032790 | ribosome disassembly | 6 | 0 | 0.23 | 1 |
| GO:0032792 | negative regulation of CREB transcriptio... | 4 | 0 | 0.15 | 1 |
| GO:0032796 | uropod organization | 2 | 0 | 0.08 | 1 |
| GO:0032801 | receptor catabolic process | 17 | 0 | 0.65 | 1 |
| GO:0032802 | low-density lipoprotein particle recepto... | 5 | 0 | 0.19 | 1 |
| GO:0032803 | regulation of low-density lipoprotein pa... | 5 | 0 | 0.19 | 1 |
| GO:0032804 | negative regulation of low-density lipop... | 2 | 0 | 0.08 | 1 |
| GO:0032805 | positive regulation of low-density lipop... | 2 | 0 | 0.08 | 1 |
| GO:0032811 | negative regulation of epinephrine secre... | 6 | 0 | 0.23 | 1 |
| GO:0032812 | positive regulation of epinephrine secre... | 1 | 0 | 0.04 | 1 |
| GO:0032814 | regulation of natural killer cell activa... | 26 | 0 | 0.99 | 1 |
| GO:0032815 | negative regulation of natural killer ce... | 6 | 0 | 0.23 | 1 |
| GO:0032816 | positive regulation of natural killer ce... | 19 | 0 | 0.72 | 1 |
| GO:0032817 | regulation of natural killer cell prolif... | 7 | 0 | 0.27 | 1 |
| GO:0032819 | positive regulation of natural killer ce... | 6 | 0 | 0.23 | 1 |
| GO:0032823 | regulation of natural killer cell differ... | 12 | 0 | 0.46 | 1 |
| GO:0032824 | negative regulation of natural killer ce... | 4 | 0 | 0.15 | 1 |
| GO:0032825 | positive regulation of natural killer ce... | 8 | 0 | 0.3 | 1 |
| GO:0032826 | regulation of natural killer cell differ... | 4 | 0 | 0.15 | 1 |
| GO:0032827 | negative regulation of natural killer ce... | 4 | 0 | 0.15 | 1 |
| GO:0032829 | regulation of CD4-positive | CD25-posit | 1 | 0 | 0.04 |
| GO:0032831 | positive regulation of CD4-positive | CD2... | 1 | 0 | 0.04 |
| GO:0032847 | regulation of cellular pH reduction | 5 | 0 | 0.19 | 1 |
| GO:0032848 | negative regulation of cellular pH reduc... | 1 | 0 | 0.04 | 1 |
| GO:0032849 | positive regulation of cellular pH reduc... | 4 | 0 | 0.15 | 1 |
| GO:0032877 | positive regulation of DNA endoreduplica... | 2 | 0 | 0.08 | 1 |
| GO:0032889 | regulation of vacuole fusion | non-autoph | 1 | 0 | 0.04 |

| | | | | | |
|---|---|---|---|---|---|
| GO:0032898 | neurotrophin production | 4 | 0 | 0.15 | 1 |
| GO:0032899 | regulation of neurotrophin production | 2 | 0 | 0.08 | 1 |
| GO:0032900 | negative regulation of neurotrophin prod... | 1 | 0 | 0.04 | 1 |
| GO:0032901 | positive regulation of neurotrophin prod... | 1 | 0 | 0.04 | 1 |
| GO:0032902 | nerve growth factor production | 2 | 0 | 0.08 | 1 |
| GO:0032905 | transforming growth factor beta1 product... | 8 | 0 | 0.3 | 1 |
| GO:0032906 | transforming growth factor beta2 product... | 8 | 0 | 0.3 | 1 |
| GO:0032907 | transforming growth factor beta3 product... | 2 | 0 | 0.08 | 1 |
| GO:0032908 | regulation of transforming growth factor... | 8 | 0 | 0.3 | 1 |
| GO:0032909 | regulation of transforming growth factor... | 8 | 0 | 0.3 | 1 |
| GO:0032910 | regulation of transforming growth factor... | 2 | 0 | 0.08 | 1 |
| GO:0032911 | negative regulation of transforming grow... | 3 | 0 | 0.11 | 1 |
| GO:0032912 | negative regulation of transforming grow... | 2 | 0 | 0.08 | 1 |
| GO:0032913 | negative regulation of transforming grow... | 1 | 0 | 0.04 | 1 |
| GO:0032914 | positive regulation of transforming grow... | 5 | 0 | 0.19 | 1 |
| GO:0032915 | positive regulation of transforming grow... | 2 | 0 | 0.08 | 1 |
| GO:0032916 | positive regulation of transforming grow... | 1 | 0 | 0.04 | 1 |
| GO:0032917 | polyamine acetylation | 2 | 0 | 0.08 | 1 |
| GO:0032918 | spermidine acetylation | 2 | 0 | 0.08 | 1 |
| GO:0032919 | spermine acetylation | 1 | 0 | 0.04 | 1 |
| GO:0032920 | putrescine acetylation | 1 | 0 | 0.04 | 1 |
| GO:0032922 | circadian regulation of gene expression | 54 | 0 | 2.06 | 1 |
| GO:0032924 | activin receptor signaling pathway | 39 | 0 | 1.48 | 1 |
| GO:0032925 | regulation of activin receptor signaling... | 23 | 0 | 0.88 | 1 |
| GO:0032926 | negative regulation of activin receptor ... | 10 | 0 | 0.38 | 1 |
| GO:0032927 | positive regulation of activin receptor ... | 8 | 0 | 0.3 | 1 |
| GO:0032928 | regulation of superoxide anion generatio... | 11 | 0 | 0.42 | 1 |
| GO:0032929 | negative regulation of superoxide anion ... | 2 | 0 | 0.08 | 1 |
| GO:0032930 | positive regulation of superoxide anion ... | 8 | 0 | 0.3 | 1 |
| GO:0032938 | negative regulation of translation in re... | 1 | 0 | 0.04 | 1 |
| GO:0032958 | inositol phosphate biosynthetic process | 16 | 0 | 0.61 | 1 |
| GO:0032959 | inositol trisphosphate biosynthetic proc... | 8 | 0 | 0.3 | 1 |
| GO:0032960 | regulation of inositol trisphosphate bio... | 5 | 0 | 0.19 | 1 |
| GO:0032962 | positive regulation of inositol trisphos... | 4 | 0 | 0.15 | 1 |
| GO:0032966 | negative regulation of collagen biosynth... | 5 | 0 | 0.19 | 1 |
| GO:0032968 | positive regulation of transcription elo... | 14 | 0 | 0.53 | 1 |
| GO:0032971 | regulation of muscle filament sliding | 3 | 0 | 0.11 | 1 |
| GO:0032972 | regulation of muscle filament sliding sp... | 1 | 0 | 0.04 | 1 |
| GO:0032976 | release of matrix enzymes from mitochond | 1 | 0 | 0.04 | 1 |
| GO:0032980 | keratinocyte activation | 1 | 0 | 0.04 | 1 |
| GO:0032981 | mitochondrial respiratory chain complex ... | 11 | 0 | 0.42 | 1 |
| GO:0032988 | ribonucleoprotein complex disassembly | 9 | 0 | 0.34 | 1 |
| GO:0033005 | positive regulation of mast cell activat... | 15 | 0 | 0.57 | 1 |
| GO:0033008 | positive regulation of mast cell activat... | 13 | 0 | 0.49 | 1 |
| GO:0033014 | tetrapyrrole biosynthetic process | 29 | 0 | 1.1 | 1 |
| GO:0033015 | tetrapyrrole catabolic process | 7 | 0 | 0.27 | 1 |
| GO:0033023 | mast cell homeostasis | 4 | 0 | 0.15 | 1 |
| GO:0033024 | mast cell apoptotic process | 4 | 0 | 0.15 | 1 |
| GO:0033025 | regulation of mast cell apoptotic proces... | 4 | 0 | 0.15 | 1 |
| GO:0033026 | negative regulation of mast cell apoptot... | 3 | 0 | 0.11 | 1 |
| GO:0033029 | regulation of neutrophil apoptotic proce... | 1 | 0 | 0.04 | 1 |
| GO:0033031 | positive regulation of neutrophil apopto... | 1 | 0 | 0.04 | 1 |
| GO:0033033 | negative regulation of myeloid cell apop... | 12 | 0 | 0.46 | 1 |

| | | | | | |
|---|---|---|---|---|---|
| GO:0033034 | positive regulation of myeloid cell apop... | 5 | 0 | 0.19 | 1 |
| GO:0033037 | polysaccharide localization | 1 | 0 | 0.04 | 1 |
| GO:0033058 | directional locomotion | 2 | 0 | 0.08 | 1 |
| GO:0033076 | isoquinoline alkaloid metabolic process | 3 | 0 | 0.11 | 1 |
| GO:0033078 | extrathymic T cell differentiation | 1 | 0 | 0.04 | 1 |
| GO:0033079 | immature T cell proliferation | 9 | 0 | 0.34 | 1 |
| GO:0033080 | immature T cell proliferation in thymus | 8 | 0 | 0.3 | 1 |
| GO:0033083 | regulation of immature T cell proliferat... | 8 | 0 | 0.3 | 1 |
| GO:0033084 | regulation of immature T cell proliferat... | 7 | 0 | 0.27 | 1 |
| GO:0033087 | negative regulation of immature T cell p... | 4 | 0 | 0.15 | 1 |
| GO:0033088 | negative regulation of immature T cell p... | 4 | 0 | 0.15 | 1 |
| GO:0033089 | positive regulation of T cell differenti... | 10 | 0 | 0.38 | 1 |
| GO:0033091 | positive regulation of immature T cell p... | 4 | 0 | 0.15 | 1 |
| GO:0033092 | positive regulation of immature T cell p... | 3 | 0 | 0.11 | 1 |
| GO:0033108 | mitochondrial respiratory chain complex ... | 17 | 0 | 0.65 | 1 |
| GO:0033119 | negative regulation of RNA splicing | 12 | 0 | 0.46 | 1 |
| GO:0033120 | positive regulation of RNA splicing | 18 | 0 | 0.69 | 1 |
| GO:0033121 | regulation of purine nucleotide cataboli... | 4 | 0 | 0.15 | 1 |
| GO:0033122 | negative regulation of purine nucleotide... | 2 | 0 | 0.08 | 1 |
| GO:0033123 | positive regulation of purine nucleotide... | 2 | 0 | 0.08 | 1 |
| GO:0033127 | regulation of histone phosphorylation | 9 | 0 | 0.34 | 1 |
| GO:0033128 | negative regulation of histone phosphory... | 3 | 0 | 0.11 | 1 |
| GO:0033129 | positive regulation of histone phosphory... | 6 | 0 | 0.23 | 1 |
| GO:0033131 | regulation of glucokinase activity | 10 | 0 | 0.38 | 1 |
| GO:0033132 | negative regulation of glucokinase activ... | 6 | 0 | 0.23 | 1 |
| GO:0033133 | positive regulation of glucokinase activ... | 5 | 0 | 0.19 | 1 |
| GO:0033139 | regulation of peptidyl-serine phosphoryl... | 18 | 0 | 0.69 | 1 |
| GO:0033141 | positive regulation of peptidyl-serine p... | 18 | 0 | 0.69 | 1 |
| GO:0033143 | regulation of intracellular steroid horm... | 55 | 0 | 2.09 | 1 |
| GO:0033144 | negative regulation of intracellular ste... | 31 | 0 | 1.18 | 1 |
| GO:0033145 | positive regulation of intracellular ste... | 9 | 0 | 0.34 | 1 |
| GO:0033146 | regulation of intracellular estrogen rec... | 24 | 0 | 0.91 | 1 |
| GO:0033147 | negative regulation of intracellular est... | 11 | 0 | 0.42 | 1 |
| GO:0033148 | positive regulation of intracellular est... | 6 | 0 | 0.23 | 1 |
| GO:0033151 | V(D)J recombination | 15 | 0 | 0.57 | 1 |
| GO:0033152 | immunoglobulin V(D)J recombination | 4 | 0 | 0.15 | 1 |
| GO:0033153 | T cell receptor V(D)J recombination | 5 | 0 | 0.19 | 1 |
| GO:0033158 | regulation of protein import into nucleu... | 15 | 0 | 0.57 | 1 |
| GO:0033159 | negative regulation of protein import in... | 3 | 0 | 0.11 | 1 |
| GO:0033160 | positive regulation of protein import in... | 8 | 0 | 0.3 | 1 |
| GO:0033168 | conversion of ds siRNA to ss siRNA invol.. | 1 | 0 | 0.04 | 1 |
| GO:0033169 | histone H3-K9 demethylation | 4 | 0 | 0.15 | 1 |
| GO:0033173 | calcineurin-NFAT signaling cascade | 17 | 0 | 0.65 | 1 |
| GO:0033182 | regulation of histone ubiquitination | 8 | 0 | 0.3 | 1 |
| GO:0033183 | negative regulation of histone ubiquitin... | 3 | 0 | 0.11 | 1 |
| GO:0033184 | positive regulation of histone ubiquitin... | 4 | 0 | 0.15 | 1 |
| GO:0033194 | response to hydroperoxide | 14 | 0 | 0.53 | 1 |
| GO:0033206 | meiotic cytokinesis | 6 | 0 | 0.23 | 1 |
| GO:0033211 | adiponectin-activated signaling pathway | 6 | 0 | 0.23 | 1 |
| GO:0033212 | iron assimilation | 1 | 0 | 0.04 | 1 |
| GO:0033214 | iron assimilation by chelation and trans... | 1 | 0 | 0.04 | 1 |
| GO:0033227 | dsRNA transport | 4 | 0 | 0.15 | 1 |
| GO:0033234 | negative regulation of protein sumoylati... | 6 | 0 | 0.23 | 1 |

| | | | | | |
|---|---|---|---|---|---|
| GO:0033239 | negative regulation of cellular amine me... | 6 | 0 | 0.23 | 1 |
| GO:0033240 | positive regulation of cellular amine me... | 12 | 0 | 0.46 | 1 |
| GO:0033241 | regulation of cellular amine catabolic p... | 1 | 0 | 0.04 | 1 |
| GO:0033242 | negative regulation of cellular amine ca... | 1 | 0 | 0.04 | 1 |
| GO:0033274 | response to vitamin B2 | 2 | 0 | 0.08 | 1 |
| GO:0033277 | abortive mitotic cell cycle | 2 | 0 | 0.08 | 1 |
| GO:0033278 | cell proliferation in midbrain | 2 | 0 | 0.08 | 1 |
| GO:0033292 | T-tubule organization | 2 | 0 | 0.08 | 1 |
| GO:0033299 | secretion of lysosomal enzymes | 5 | 0 | 0.19 | 1 |
| GO:0033301 | cell cycle comprising mitosis without cy... | 1 | 0 | 0.04 | 1 |
| GO:0033306 | phytol metabolic process | 2 | 0 | 0.08 | 1 |
| GO:0033315 | meiotic DNA replication checkpoint | 1 | 0 | 0.04 | 1 |
| GO:0033319 | UDP-D-xylose metabolic process | 1 | 0 | 0.04 | 1 |
| GO:0033320 | UDP-D-xylose biosynthetic process | 1 | 0 | 0.04 | 1 |
| GO:0033326 | cerebrospinal fluid secretion | 4 | 0 | 0.15 | 1 |
| GO:0033341 | regulation of collagen binding | 2 | 0 | 0.08 | 1 |
| GO:0033342 | negative regulation of collagen binding | 1 | 0 | 0.04 | 1 |
| GO:0033343 | positive regulation of collagen binding | 1 | 0 | 0.04 | 1 |
| GO:0033344 | cholesterol efflux | 41 | 0 | 1.56 | 1 |
| GO:0033345 | asparagine catabolic process via L-aspar.. | 1 | 0 | 0.04 | 1 |
| GO:0033353 | S-adenosylmethionine cycle | 5 | 0 | 0.19 | 1 |
| GO:0033363 | secretory granule organization | 29 | 0 | 1.1 | 1 |
| GO:0033364 | mast cell secretory granule organization | 2 | 0 | 0.08 | 1 |
| GO:0033366 | protein localization to secretory granul... | 1 | 0 | 0.04 | 1 |
| GO:0033367 | protein localization to mast cell secret... | 1 | 0 | 0.04 | 1 |
| GO:0033368 | protease localization to mast cell secre... | 1 | 0 | 0.04 | 1 |
| GO:0033370 | maintenance of protein location in mast ... | 1 | 0 | 0.04 | 1 |
| GO:0033371 | T cell secretory granule organization | 1 | 0 | 0.04 | 1 |
| GO:0033373 | maintenance of protease location in mast.. | 1 | 0 | 0.04 | 1 |
| GO:0033374 | protein localization to T cell secretory... | 1 | 0 | 0.04 | 1 |
| GO:0033375 | protease localization to T cell secretor... | 1 | 0 | 0.04 | 1 |
| GO:0033377 | maintenance of protein location in T cel... | 1 | 0 | 0.04 | 1 |
| GO:0033379 | maintenance of protease location in T ce... | 1 | 0 | 0.04 | 1 |
| GO:0033380 | granzyme B localization to T cell secret... | 1 | 0 | 0.04 | 1 |
| GO:0033382 | maintenance of granzyme B location in T . | 1 | 0 | 0.04 | 1 |
| GO:0033383 | geranyl diphosphate metabolic process | 1 | 0 | 0.04 | 1 |
| GO:0033384 | geranyl diphosphate biosynthetic process | 1 | 0 | 0.04 | 1 |
| GO:0033385 | geranylgeranyl diphosphate metabolic pro. | 1 | 0 | 0.04 | 1 |
| GO:0033386 | geranylgeranyl diphosphate biosynthetic .. | 1 | 0 | 0.04 | 1 |
| GO:0033387 | putrescine biosynthetic process from orn... | 1 | 0 | 0.04 | 1 |
| GO:0033388 | putrescine biosynthetic process from arg... | 1 | 0 | 0.04 | 1 |
| GO:0033477 | S-methylmethionine metabolic process | 2 | 0 | 0.08 | 1 |
| GO:0033483 | gas homeostasis | 8 | 0 | 0.3 | 1 |
| GO:0033484 | nitric oxide homeostasis | 2 | 0 | 0.08 | 1 |
| GO:0033488 | cholesterol biosynthetic process via 24 ... | | 1 | 0 | 0.04 |
| GO:0033504 | floor plate development | 7 | 0 | 0.27 | 1 |
| GO:0033505 | floor plate morphogenesis | 2 | 0 | 0.08 | 1 |
| GO:0033512 | L-lysine catabolic process to acetyl-CoA... | 3 | 0 | 0.11 | 1 |
| GO:0033514 | L-lysine catabolic process to acetyl-CoA... | 1 | 0 | 0.04 | 1 |
| GO:0033522 | histone H2A ubiquitination | 19 | 0 | 0.72 | 1 |
| GO:0033523 | histone H2B ubiquitination | 10 | 0 | 0.38 | 1 |
| GO:0033528 | S-methylmethionine cycle | 2 | 0 | 0.08 | 1 |
| GO:0033539 | fatty acid beta-oxidation using acyl-CoA... | 6 | 0 | 0.23 | 1 |

| | | | | | |
|---|---|---|---|---|---|
| GO:0033540 | fatty acid beta-oxidation using acyl-CoA... | 11 | 0 | 0.42 | 1 |
| GO:0033561 | regulation of water loss via skin | 19 | 0 | 0.72 | 1 |
| GO:0033563 | dorsal/ventral axon guidance | 4 | 0 | 0.15 | 1 |
| GO:0033566 | gamma-tubulin complex localization | 1 | 0 | 0.04 | 1 |
| GO:0033567 | DNA replication | Okazaki fra | 3 | 0 | 0.11 |
| GO:0033577 | protein glycosylation in endoplasmic ret... | 1 | 0 | 0.04 | 1 |
| GO:0033578 | protein glycosylation in Golgi | 5 | 0 | 0.19 | 1 |
| GO:0033590 | response to cobalamin | 2 | 0 | 0.08 | 1 |
| GO:0033594 | response to hydroxyisoflavone | 4 | 0 | 0.15 | 1 |
| GO:0033595 | response to genistein | 2 | 0 | 0.08 | 1 |
| GO:0033600 | negative regulation of mammary gland epi. | 5 | 0 | 0.19 | 1 |
| GO:0033602 | negative regulation of dopamine secretio... | 3 | 0 | 0.11 | 1 |
| GO:0033603 | positive regulation of dopamine secretio... | 6 | 0 | 0.23 | 1 |
| GO:0033604 | negative regulation of catecholamine sec.. | 13 | 0 | 0.49 | 1 |
| GO:0033605 | positive regulation of catecholamine sec... | 10 | 0 | 0.38 | 1 |
| GO:0033606 | chemokine receptor transport within lipi... | 1 | 0 | 0.04 | 1 |
| GO:0033615 | mitochondrial proton-transporting ATP sy... | 2 | 0 | 0.08 | 1 |
| GO:0033617 | mitochondrial respiratory chain complex ... | 2 | 0 | 0.08 | 1 |
| GO:0033622 | integrin activation | 13 | 0 | 0.49 | 1 |
| GO:0033623 | regulation of integrin activation | 8 | 0 | 0.3 | 1 |
| GO:0033624 | negative regulation of integrin activati... | 2 | 0 | 0.08 | 1 |
| GO:0033625 | positive regulation of integrin activati... | 3 | 0 | 0.11 | 1 |
| GO:0033629 | negative regulation of cell adhesion med... | 8 | 0 | 0.3 | 1 |
| GO:0033630 | positive regulation of cell adhesion med... | 15 | 0 | 0.57 | 1 |
| GO:0033631 | cell-cell adhesion mediated by integrin | 11 | 0 | 0.42 | 1 |
| GO:0033632 | regulation of cell-cell adhesion mediate... | 6 | 0 | 0.23 | 1 |
| GO:0033634 | positive regulation of cell-cell adhesio... | 3 | 0 | 0.11 | 1 |
| GO:0033683 | nucleotide-excision repair | DNA incisio | 7 | 0 | 0.27 |
| GO:0033685 | negative regulation of luteinizing hormo... | 3 | 0 | 0.11 | 1 |
| GO:0033690 | positive regulation of osteoblast prolif... | 10 | 0 | 0.38 | 1 |
| GO:0033693 | neurofilament bundle assembly | 3 | 0 | 0.11 | 1 |
| GO:0033700 | phospholipid efflux | 14 | 0 | 0.53 | 1 |
| GO:0033861 | negative regulation of NAD(P)H oxidase a. | 3 | 0 | 0.11 | 1 |
| GO:0033869 | nucleoside bisphosphate catabolic proces. | 2 | 0 | 0.08 | 1 |
| GO:0033967 | box C/D snoRNA metabolic process | 1 | 0 | 0.04 | 1 |
| GO:0033979 | box H/ACA snoRNA metabolic process | 1 | 0 | 0.04 | 1 |
| GO:0033986 | response to methanol | 1 | 0 | 0.04 | 1 |
| GO:0034014 | response to triglyceride | 2 | 0 | 0.08 | 1 |
| GO:0034021 | response to silicon dioxide | 2 | 0 | 0.08 | 1 |
| GO:0034031 | ribonucleoside bisphosphate catabolic pr... | 2 | 0 | 0.08 | 1 |
| GO:0034034 | purine nucleoside bisphosphate catabolic.. | 2 | 0 | 0.08 | 1 |
| GO:0034047 | regulation of protein phosphatase type 2... | 3 | 0 | 0.11 | 1 |
| GO:0034048 | negative regulation of protein phosphata... | 2 | 0 | 0.08 | 1 |
| GO:0034050 | host programmed cell death induced by sy | 1 | 0 | 0.04 | 1 |
| GO:0034058 | endosomal vesicle fusion | 3 | 0 | 0.11 | 1 |
| GO:0034059 | response to anoxia | 2 | 0 | 0.08 | 1 |
| GO:0034063 | stress granule assembly | 9 | 0 | 0.34 | 1 |
| GO:0034104 | negative regulation of tissue remodeling | 12 | 0 | 0.46 | 1 |
| GO:0034114 | regulation of heterotypic cell-cell adhe... | 16 | 0 | 0.61 | 1 |
| GO:0034115 | negative regulation of heterotypic cell-... | 7 | 0 | 0.27 | 1 |
| GO:0034116 | positive regulation of heterotypic cell-... | 9 | 0 | 0.34 | 1 |
| GO:0034117 | erythrocyte aggregation | 1 | 0 | 0.04 | 1 |
| GO:0034118 | regulation of erythrocyte aggregation | 1 | 0 | 0.04 | 1 |

| | | | | | |
|---|---|---|---|---|---|
| GO:0034120 | positive regulation of erythrocyte aggre... | 1 | 0 | 0.04 | 1 |
| GO:0034122 | negative regulation of toll-like recepto... | 17 | 0 | 0.65 | 1 |
| GO:0034124 | regulation of MyD88-dependent toll-like ... | 3 | 0 | 0.11 | 1 |
| GO:0034126 | positive regulation of MyD88-dependent t.. | 1 | 0 | 0.04 | 1 |
| GO:0034127 | regulation of MyD88-independent toll-lik... | 1 | 0 | 0.04 | 1 |
| GO:0034130 | toll-like receptor 1 signaling pathway | 1 | 0 | 0.04 | 1 |
| GO:0034135 | regulation of toll-like receptor 2 signa... | 5 | 0 | 0.19 | 1 |
| GO:0034136 | negative regulation of toll-like recepto... | 3 | 0 | 0.11 | 1 |
| GO:0034137 | positive regulation of toll-like recepto... | 2 | 0 | 0.08 | 1 |
| GO:0034140 | negative regulation of toll-like recepto... | 2 | 0 | 0.08 | 1 |
| GO:0034144 | negative regulation of toll-like recepto... | 5 | 0 | 0.19 | 1 |
| GO:0034147 | regulation of toll-like receptor 5 signa... | 1 | 0 | 0.04 | 1 |
| GO:0034148 | negative regulation of toll-like recepto... | 1 | 0 | 0.04 | 1 |
| GO:0034150 | toll-like receptor 6 signaling pathway | 1 | 0 | 0.04 | 1 |
| GO:0034154 | toll-like receptor 7 signaling pathway | 4 | 0 | 0.15 | 1 |
| GO:0034155 | regulation of toll-like receptor 7 signa... | 1 | 0 | 0.04 | 1 |
| GO:0034157 | positive regulation of toll-like recepto... | 1 | 0 | 0.04 | 1 |
| GO:0034158 | toll-like receptor 8 signaling pathway | 1 | 0 | 0.04 | 1 |
| GO:0034163 | regulation of toll-like receptor 9 signa... | 1 | 0 | 0.04 | 1 |
| GO:0034165 | positive regulation of toll-like recepto... | 1 | 0 | 0.04 | 1 |
| GO:0034196 | acylglycerol transport | 3 | 0 | 0.11 | 1 |
| GO:0034197 | triglyceride transport | 3 | 0 | 0.11 | 1 |
| GO:0034198 | cellular response to amino acid starvati... | 2 | 0 | 0.08 | 1 |
| GO:0034201 | response to oleic acid | 6 | 0 | 0.23 | 1 |
| GO:0034203 | glycolipid translocation | 1 | 0 | 0.04 | 1 |
| GO:0034205 | beta-amyloid formation | 10 | 0 | 0.38 | 1 |
| GO:0034213 | quinolinate catabolic process | 1 | 0 | 0.04 | 1 |
| GO:0034214 | protein hexamerization | 6 | 0 | 0.23 | 1 |
| GO:0034224 | cellular response to zinc ion starvation | 2 | 0 | 0.08 | 1 |
| GO:0034226 | lysine import | 1 | 0 | 0.04 | 1 |
| GO:0034227 | tRNA thio-modification | 3 | 0 | 0.11 | 1 |
| GO:0034230 | enkephalin processing | 1 | 0 | 0.04 | 1 |
| GO:0034231 | islet amyloid polypeptide processing | 1 | 0 | 0.04 | 1 |
| GO:0034238 | macrophage fusion | 2 | 0 | 0.08 | 1 |
| GO:0034239 | regulation of macrophage fusion | 2 | 0 | 0.08 | 1 |
| GO:0034240 | negative regulation of macrophage fusion | 1 | 0 | 0.04 | 1 |
| GO:0034241 | positive regulation of macrophage fusion | 1 | 0 | 0.04 | 1 |
| GO:0034242 | negative regulation of syncytium formati... | 5 | 0 | 0.19 | 1 |
| GO:0034243 | regulation of transcription elongation f... | 22 | 0 | 0.84 | 1 |
| GO:0034244 | negative regulation of transcription elo... | 8 | 0 | 0.3 | 1 |
| GO:0034248 | regulation of cellular amide metabolic p... | 18 | 0 | 0.69 | 1 |
| GO:0034249 | negative regulation of cellular amide me... | 7 | 0 | 0.27 | 1 |
| GO:0034250 | positive regulation of cellular amide me... | 9 | 0 | 0.34 | 1 |
| GO:0034255 | regulation of urea metabolic process | 1 | 0 | 0.04 | 1 |
| GO:0034260 | negative regulation of GTPase activity | 20 | 0 | 0.76 | 1 |
| GO:0034263 | autophagy in response to ER overload | 1 | 0 | 0.04 | 1 |
| GO:0034275 | kynurenic acid metabolic process | 1 | 0 | 0.04 | 1 |
| GO:0034276 | kynurenic acid biosynthetic process | 1 | 0 | 0.04 | 1 |
| GO:0034285 | response to disaccharide | 6 | 0 | 0.23 | 1 |
| GO:0034287 | detection of monosaccharide stimulus | 4 | 0 | 0.15 | 1 |
| GO:0034309 | primary alcohol biosynthetic process | 1 | 0 | 0.04 | 1 |
| GO:0034310 | primary alcohol catabolic process | 9 | 0 | 0.34 | 1 |
| GO:0034311 | diol metabolic process | 11 | 0 | 0.42 | 1 |

| | | | | | |
|---|---|---|---|---|---|
| GO:0034312 | diol biosynthetic process | 6 | 0 | 0.23 | 1 |
| GO:0034315 | regulation of Arp2/3 complex-mediated ac. | 13 | 0 | 0.49 | 1 |
| GO:0034316 | negative regulation of Arp2/3 complex-me. | 4 | 0 | 0.15 | 1 |
| GO:0034331 | cell junction maintenance | 9 | 0 | 0.34 | 1 |
| GO:0034334 | adherens junction maintenance | 2 | 0 | 0.08 | 1 |
| GO:0034343 | type III interferon production | 3 | 0 | 0.11 | 1 |
| GO:0034344 | regulation of type III interferon produc... | 3 | 0 | 0.11 | 1 |
| GO:0034346 | positive regulation of type III interfer... | 1 | 0 | 0.04 | 1 |
| GO:0034354 | 'de novo' NAD biosynthetic process from .. | 4 | 0 | 0.15 | 1 |
| GO:0034367 | macromolecular complex remodeling | 24 | 0 | 0.91 | 1 |
| GO:0034368 | protein-lipid complex remodeling | 24 | 0 | 0.91 | 1 |
| GO:0034369 | plasma lipoprotein particle remodeling | 24 | 0 | 0.91 | 1 |
| GO:0034370 | triglyceride-rich lipoprotein particle r... | 11 | 0 | 0.42 | 1 |
| GO:0034371 | chylomicron remodeling | 3 | 0 | 0.11 | 1 |
| GO:0034372 | very-low-density lipoprotein particle re... | 11 | 0 | 0.42 | 1 |
| GO:0034373 | intermediate-density lipoprotein particl... | 1 | 0 | 0.04 | 1 |
| GO:0034374 | low-density lipoprotein particle remodel... | 11 | 0 | 0.42 | 1 |
| GO:0034375 | high-density lipoprotein particle remode... | 15 | 0 | 0.57 | 1 |
| GO:0034377 | plasma lipoprotein particle assembly | 19 | 0 | 0.72 | 1 |
| GO:0034378 | chylomicron assembly | 2 | 0 | 0.08 | 1 |
| GO:0034379 | very-low-density lipoprotein particle as... | 8 | 0 | 0.3 | 1 |
| GO:0034380 | high-density lipoprotein particle assemb... | 10 | 0 | 0.38 | 1 |
| GO:0034382 | chylomicron remnant clearance | 7 | 0 | 0.27 | 1 |
| GO:0034384 | high-density lipoprotein particle cleara... | 9 | 0 | 0.34 | 1 |
| GO:0034393 | positive regulation of smooth muscle cel... | 4 | 0 | 0.15 | 1 |
| GO:0034394 | protein localization to cell surface | 43 | 0 | 1.64 | 1 |
| GO:0034395 | regulation of transcription from RNA pol... | 3 | 0 | 0.11 | 1 |
| GO:0034401 | chromatin organization involved in regul... | 2 | 0 | 0.08 | 1 |
| GO:0034402 | recruitment of 3'-end processing factors... | 1 | 0 | 0.04 | 1 |
| GO:0034414 | tRNA 3'-trailer cleavage | endonuclec | 1 | 0 | 0.04 |
| GO:0034418 | urate biosynthetic process | 1 | 0 | 0.04 | 1 |
| GO:0034421 | post-translational protein acetylation | 3 | 0 | 0.11 | 1 |
| GO:0034427 | nuclear-transcribed mRNA catabolic proce | 3 | 0 | 0.11 | 1 |
| GO:0034433 | steroid esterification | 12 | 0 | 0.46 | 1 |
| GO:0034434 | sterol esterification | 12 | 0 | 0.46 | 1 |
| GO:0034435 | cholesterol esterification | 12 | 0 | 0.46 | 1 |
| GO:0034436 | glycoprotein transport | 3 | 0 | 0.11 | 1 |
| GO:0034441 | plasma lipoprotein particle oxidation | 3 | 0 | 0.11 | 1 |
| GO:0034443 | negative regulation of lipoprotein oxida... | 3 | 0 | 0.11 | 1 |
| GO:0034444 | regulation of plasma lipoprotein particl... | 2 | 0 | 0.08 | 1 |
| GO:0034445 | negative regulation of plasma lipoprotei... | 2 | 0 | 0.08 | 1 |
| GO:0034447 | very-low-density lipoprotein particle cl... | 6 | 0 | 0.23 | 1 |
| GO:0034454 | microtubule anchoring at centrosome | 4 | 0 | 0.15 | 1 |
| GO:0034463 | 90S preribosome assembly | 1 | 0 | 0.04 | 1 |
| GO:0034465 | response to carbon monoxide | 4 | 0 | 0.15 | 1 |
| GO:0034471 | ncRNA 5'-end processing | 2 | 0 | 0.08 | 1 |
| GO:0034472 | snRNA 3'-end processing | 3 | 0 | 0.11 | 1 |
| GO:0034474 | U2 snRNA 3'-end processing | 1 | 0 | 0.04 | 1 |
| GO:0034475 | U4 snRNA 3'-end processing | 2 | 0 | 0.08 | 1 |
| GO:0034498 | early endosome to Golgi transport | 6 | 0 | 0.23 | 1 |
| GO:0034499 | late endosome to Golgi transport | 2 | 0 | 0.08 | 1 |
| GO:0034501 | protein localization to kinetochore | 7 | 0 | 0.27 | 1 |
| GO:0034505 | tooth mineralization | 15 | 0 | 0.57 | 1 |

| | | | | | |
|---|---|---|---|---|---|
| GO:0034516 | response to vitamin B6 | 1 | 0 | 0.04 | 1 |
| GO:0034551 | mitochondrial respiratory chain complex ... | 3 | 0 | 0.11 | 1 |
| GO:0034552 | respiratory chain complex II assembly | 1 | 0 | 0.04 | 1 |
| GO:0034553 | mitochondrial respiratory chain complex ... | 1 | 0 | 0.04 | 1 |
| GO:0034589 | hydroxyproline transport | 1 | 0 | 0.04 | 1 |
| GO:0034625 | fatty acid elongation | monounsatu | 3 | 0 | 0.11 |
| GO:0034626 | fatty acid elongation | polyunsatur | 4 | 0 | 0.15 |
| GO:0034627 | 'de novo' NAD biosynthetic process | 4 | 0 | 0.15 | 1 |
| GO:0034633 | retinol transport | 2 | 0 | 0.08 | 1 |
| GO:0034635 | glutathione transport | 2 | 0 | 0.08 | 1 |
| GO:0034638 | phosphatidylcholine catabolic process | 6 | 0 | 0.23 | 1 |
| GO:0034643 | establishment of mitochondrion localizat... | 9 | 0 | 0.34 | 1 |
| GO:0034650 | cortisol metabolic process | 8 | 0 | 0.3 | 1 |
| GO:0034651 | cortisol biosynthetic process | 8 | 0 | 0.3 | 1 |
| GO:0034653 | retinoic acid catabolic process | 3 | 0 | 0.11 | 1 |
| GO:0034656 | nucleobase-containing small molecule cat. | 6 | 0 | 0.23 | 1 |
| GO:0034695 | response to prostaglandin E | 16 | 0 | 0.61 | 1 |
| GO:0034698 | response to gonadotropin | 25 | 0 | 0.95 | 1 |
| GO:0034699 | response to luteinizing hormone | 2 | 0 | 0.08 | 1 |
| GO:0034720 | histone H3-K4 demethylation | 2 | 0 | 0.08 | 1 |
| GO:0034721 | histone H3-K4 demethylation | trimethyl-H. | 1 | 0 | 0.04 |
| GO:0034723 | DNA replication-dependent nucleosome or | 31 | 0 | 1.18 | 1 |
| GO:0034727 | piecemeal microautophagy of nucleus | 2 | 0 | 0.08 | 1 |
| GO:0034729 | histone H3-K79 methylation | 3 | 0 | 0.11 | 1 |
| GO:0034755 | iron ion transmembrane transport | 5 | 0 | 0.19 | 1 |
| GO:0034763 | negative regulation of transmembrane tra.. | 61 | 0 | 2.32 | 1 |
| GO:0034766 | negative regulation of ion transmembrane. | 53 | 0 | 2.02 | 1 |
| GO:0034770 | histone H4-K20 methylation | 8 | 0 | 0.3 | 1 |
| GO:0034773 | histone H4-K20 trimethylation | 4 | 0 | 0.15 | 1 |
| GO:0034959 | endothelin maturation | 1 | 0 | 0.04 | 1 |
| GO:0034963 | box C/D snoRNA processing | 1 | 0 | 0.04 | 1 |
| GO:0034964 | box H/ACA snoRNA processing | 1 | 0 | 0.04 | 1 |
| GO:0034969 | histone arginine methylation | 9 | 0 | 0.34 | 1 |
| GO:0034970 | histone H3-R2 methylation | 2 | 0 | 0.08 | 1 |
| GO:0034971 | histone H3-R17 methylation | 1 | 0 | 0.04 | 1 |
| GO:0034972 | histone H3-R26 methylation | 1 | 0 | 0.04 | 1 |
| GO:0034982 | mitochondrial protein processing | 7 | 0 | 0.27 | 1 |
| GO:0035021 | negative regulation of Rac protein signa... | 4 | 0 | 0.15 | 1 |
| GO:0035022 | positive regulation of Rac protein signa... | 5 | 0 | 0.19 | 1 |
| GO:0035025 | positive regulation of Rho protein signa... | 10 | 0 | 0.38 | 1 |
| GO:0035026 | leading edge cell differentiation | 2 | 0 | 0.08 | 1 |
| GO:0035037 | sperm entry | 1 | 0 | 0.04 | 1 |
| GO:0035038 | female pronucleus assembly | 1 | 0 | 0.04 | 1 |
| GO:0035039 | male pronucleus assembly | 2 | 0 | 0.08 | 1 |
| GO:0035041 | sperm chromatin decondensation | 1 | 0 | 0.04 | 1 |
| GO:0035042 | fertilization | exchange c | 1 | 0 | 0.04 |
| GO:0035048 | splicing factor protein import into nucl... | 1 | 0 | 0.04 | 1 |
| GO:0035054 | embryonic heart tube anterior/posterior ... | 3 | 0 | 0.11 | 1 |
| GO:0035063 | nuclear speck organization | 1 | 0 | 0.04 | 1 |
| GO:0035065 | regulation of histone acetylation | 39 | 0 | 1.48 | 1 |
| GO:0035066 | positive regulation of histone acetylati... | 19 | 0 | 0.72 | 1 |
| GO:0035067 | negative regulation of histone acetylati... | 14 | 0 | 0.53 | 1 |
| GO:0035087 | siRNA loading onto RISC involved in RNA | 1 | 0 | 0.04 | 1 |

| | | | | | |
|---|---|---|---|---|---|
| GO:0035088 | establishment or maintenance of apical/b.. | 27 | 0 | 1.03 | 1 |
| GO:0035089 | establishment of apical/basal cell polar... | 7 | 0 | 0.27 | 1 |
| GO:0035090 | maintenance of apical/basal cell polarit... | 7 | 0 | 0.27 | 1 |
| GO:0035092 | sperm chromatin condensation | 8 | 0 | 0.3 | 1 |
| GO:0035093 | spermatogenesis | exchange c | 4 | 0 | 0.15 |
| GO:0035095 | behavioral response to nicotine | 7 | 0 | 0.27 | 1 |
| GO:0035105 | sterol regulatory element binding protei... | 1 | 0 | 0.04 | 1 |
| GO:0035106 | operant conditioning | 4 | 0 | 0.15 | 1 |
| GO:0035162 | embryonic hemopoiesis | 23 | 0 | 0.88 | 1 |
| GO:0035166 | post-embryonic hemopoiesis | 3 | 0 | 0.11 | 1 |
| GO:0035188 | hatching | 4 | 0 | 0.15 | 1 |
| GO:0035212 | cell competition in a multicellular orga... | 1 | 0 | 0.04 | 1 |
| GO:0035227 | regulation of glutamate-cysteine ligase ... | 1 | 0 | 0.04 | 1 |
| GO:0035229 | positive regulation of glutamate-cystein... | 1 | 0 | 0.04 | 1 |
| GO:0035234 | ectopic germ cell programmed cell death | 8 | 0 | 0.3 | 1 |
| GO:0035247 | peptidyl-arginine omega-N-methylation | 8 | 0 | 0.3 | 1 |
| GO:0035278 | negative regulation of translation invol... | 5 | 0 | 0.19 | 1 |
| GO:0035279 | mRNA cleavage involved in gene silencing | 1 | 0 | 0.04 | 1 |
| GO:0035280 | miRNA loading onto RISC involved in gene | 3 | 0 | 0.11 | 1 |
| GO:0035281 | pre-miRNA export from nucleus | 1 | 0 | 0.04 | 1 |
| GO:0035283 | central nervous system segmentation | 3 | 0 | 0.11 | 1 |
| GO:0035284 | brain segmentation | 3 | 0 | 0.11 | 1 |
| GO:0035290 | trunk segmentation | 3 | 0 | 0.11 | 1 |
| GO:0035308 | negative regulation of protein dephospho.. | 6 | 0 | 0.23 | 1 |
| GO:0035315 | hair cell differentiation | 36 | 0 | 1.37 | 1 |
| GO:0035332 | positive regulation of hippo signaling | 1 | 0 | 0.04 | 1 |
| GO:0035349 | coenzyme A transmembrane transport | 2 | 0 | 0.08 | 1 |
| GO:0035350 | FAD transmembrane transport | 1 | 0 | 0.04 | 1 |
| GO:0035356 | cellular triglyceride homeostasis | 6 | 0 | 0.23 | 1 |
| GO:0035357 | peroxisome proliferator activated recept... | 17 | 0 | 0.65 | 1 |
| GO:0035358 | regulation of peroxisome proliferator ac... | 11 | 0 | 0.42 | 1 |
| GO:0035359 | negative regulation of peroxisome prolif... | 3 | 0 | 0.11 | 1 |
| GO:0035360 | positive regulation of peroxisome prolif... | 6 | 0 | 0.23 | 1 |
| GO:0035376 | sterol import | 5 | 0 | 0.19 | 1 |
| GO:0035377 | transepithelial water transport | 1 | 0 | 0.04 | 1 |
| GO:0035378 | carbon dioxide transmembrane transport | 1 | 0 | 0.04 | 1 |
| GO:0035382 | sterol transmembrane transport | 5 | 0 | 0.19 | 1 |
| GO:0035385 | Roundabout signaling pathway | 3 | 0 | 0.11 | 1 |
| GO:0035397 | helper T cell enhancement of adaptive im.. | 1 | 0 | 0.04 | 1 |
| GO:0035406 | histone-tyrosine phosphorylation | 1 | 0 | 0.04 | 1 |
| GO:0035407 | histone H3-T11 phosphorylation | 2 | 0 | 0.08 | 1 |
| GO:0035409 | histone H3-Y41 phosphorylation | 1 | 0 | 0.04 | 1 |
| GO:0035411 | catenin import into nucleus | 25 | 0 | 0.95 | 1 |
| GO:0035412 | regulation of catenin import into nucleu... | 23 | 0 | 0.88 | 1 |
| GO:0035413 | positive regulation of catenin import in... | 11 | 0 | 0.42 | 1 |
| GO:0035414 | negative regulation of catenin import in... | 9 | 0 | 0.34 | 1 |
| GO:0035419 | activation of MAPK activity involved in ... | 2 | 0 | 0.08 | 1 |
| GO:0035425 | autocrine signaling | 2 | 0 | 0.08 | 1 |
| GO:0035426 | extracellular matrix-cell signaling | 5 | 0 | 0.19 | 1 |
| GO:0035428 | hexose transmembrane transport | 1 | 0 | 0.04 | 1 |
| GO:0035434 | copper ion transmembrane transport | 7 | 0 | 0.27 | 1 |
| GO:0035437 | maintenance of protein localization in e... | 10 | 0 | 0.38 | 1 |
| GO:0035441 | cell migration involved in vasculogenesi... | 3 | 0 | 0.11 | 1 |

| | | | | | |
|---|---|---|---|---|---|
| GO:0035442 | dipeptide transmembrane transport | 2 | 0 | 0.08 | 1 |
| GO:0035444 | nickel cation transmembrane transport | 1 | 0 | 0.04 | 1 |
| GO:0035445 | borate transmembrane transport | 1 | 0 | 0.04 | 1 |
| GO:0035455 | response to interferon-alpha | 17 | 0 | 0.65 | 1 |
| GO:0035457 | cellular response to interferon-alpha | 8 | 0 | 0.3 | 1 |
| GO:0035459 | cargo loading into vesicle | 9 | 0 | 0.34 | 1 |
| GO:0035461 | vitamin transmembrane transport | 8 | 0 | 0.3 | 1 |
| GO:0035469 | determination of pancreatic left/right a... | 3 | 0 | 0.11 | 1 |
| GO:0035470 | positive regulation of vascular wound he... | 2 | 0 | 0.08 | 1 |
| GO:0035480 | regulation of Notch signaling pathway in... | 1 | 0 | 0.04 | 1 |
| GO:0035481 | positive regulation of Notch signaling p... | 1 | 0 | 0.04 | 1 |
| GO:0035482 | gastric motility | 2 | 0 | 0.08 | 1 |
| GO:0035483 | gastric emptying | 2 | 0 | 0.08 | 1 |
| GO:0035490 | regulation of leukotriene production inv... | 1 | 0 | 0.04 | 1 |
| GO:0035491 | positive regulation of leukotriene produ... | 1 | 0 | 0.04 | 1 |
| GO:0035493 | SNARE complex assembly | 15 | 0 | 0.57 | 1 |
| GO:0035494 | SNARE complex disassembly | 2 | 0 | 0.08 | 1 |
| GO:0035502 | metanephric part of ureteric bud develop... | 6 | 0 | 0.23 | 1 |
| GO:0035504 | regulation of myosin light chain kinase ... | 1 | 0 | 0.04 | 1 |
| GO:0035505 | positive regulation of myosin light chai... | 1 | 0 | 0.04 | 1 |
| GO:0035509 | negative regulation of myosin-light-chai... | 1 | 0 | 0.04 | 1 |
| GO:0035510 | DNA dealkylation | 18 | 0 | 0.69 | 1 |
| GO:0035511 | oxidative DNA demethylation | 5 | 0 | 0.19 | 1 |
| GO:0035513 | oxidative RNA demethylation | 2 | 0 | 0.08 | 1 |
| GO:0035518 | histone H2A monoubiquitination | 12 | 0 | 0.46 | 1 |
| GO:0035519 | protein K29-linked ubiquitination | 5 | 0 | 0.19 | 1 |
| GO:0035520 | monoubiquitinated protein deubiquitinati... | 7 | 0 | 0.27 | 1 |
| GO:0035521 | monoubiquitinated histone deubiquitinati... | 3 | 0 | 0.11 | 1 |
| GO:0035522 | monoubiquitinated histone H2A deubiquiti. | 3 | 0 | 0.11 | 1 |
| GO:0035523 | protein K29-linked deubiquitination | 3 | 0 | 0.11 | 1 |
| GO:0035526 | retrograde transport | plasma mei | 2 | 0 | 0.08 |
| GO:0035542 | regulation of SNARE complex assembly | 8 | 0 | 0.3 | 1 |
| GO:0035543 | positive regulation of SNARE complex ass | 1 | 0 | 0.04 | 1 |
| GO:0035544 | negative regulation of SNARE complex as: | 2 | 0 | 0.08 | 1 |
| GO:0035545 | determination of left/right asymmetry in... | 1 | 0 | 0.04 | 1 |
| GO:0035546 | interferon-beta secretion | 1 | 0 | 0.04 | 1 |
| GO:0035547 | regulation of interferon-beta secretion | 1 | 0 | 0.04 | 1 |
| GO:0035548 | negative regulation of interferon-beta s... | 1 | 0 | 0.04 | 1 |
| GO:0035552 | oxidative single-stranded DNA demethylat. | 2 | 0 | 0.08 | 1 |
| GO:0035553 | oxidative single-stranded RNA demethylat. | 2 | 0 | 0.08 | 1 |
| GO:0035561 | regulation of chromatin binding | 8 | 0 | 0.3 | 1 |
| GO:0035562 | negative regulation of chromatin binding | 4 | 0 | 0.15 | 1 |
| GO:0035563 | positive regulation of chromatin binding | 4 | 0 | 0.15 | 1 |
| GO:0035564 | regulation of kidney size | 4 | 0 | 0.15 | 1 |
| GO:0035565 | regulation of pronephros size | 1 | 0 | 0.04 | 1 |
| GO:0035566 | regulation of metanephros size | 2 | 0 | 0.08 | 1 |
| GO:0035574 | histone H4-K20 demethylation | 14 | 0 | 0.53 | 1 |
| GO:0035584 | calcium-mediated signaling using intrace... | 10 | 0 | 0.38 | 1 |
| GO:0035585 | calcium-mediated signaling using extrace.. | 1 | 0 | 0.04 | 1 |
| GO:0035587 | purinergic receptor signaling pathway | 30 | 0 | 1.14 | 1 |
| GO:0035588 | G-protein coupled purinergic receptor si... | 23 | 0 | 0.88 | 1 |
| GO:0035589 | G-protein coupled purinergic nucleotide ... | 14 | 0 | 0.53 | 1 |
| GO:0035590 | purinergic nucleotide receptor signaling... | 21 | 0 | 0.8 | 1 |

| | | | | | |
|---|---|---|---|---|---|
| GO:0035602 | fibroblast growth factor receptor signal... | 1 | 0 | 0.04 | 1 |
| GO:0035603 | fibroblast growth factor receptor signal... | 1 | 0 | 0.04 | 1 |
| GO:0035604 | fibroblast growth factor receptor signal... | 1 | 0 | 0.04 | 1 |
| GO:0035606 | peptidyl-cysteine S-trans-nitrosylation | 1 | 0 | 0.04 | 1 |
| GO:0035607 | fibroblast growth factor receptor signal... | 2 | 0 | 0.08 | 1 |
| GO:0035608 | protein deglutamylation | 5 | 0 | 0.19 | 1 |
| GO:0035609 | C-terminal protein deglutamylation | 3 | 0 | 0.11 | 1 |
| GO:0035610 | protein side chain deglutamylation | 4 | 0 | 0.15 | 1 |
| GO:0035611 | protein branching point deglutamylation | 1 | 0 | 0.04 | 1 |
| GO:0035616 | histone H2B conserved C-terminal lysine .. | 2 | 0 | 0.08 | 1 |
| GO:0035617 | stress granule disassembly | 2 | 0 | 0.08 | 1 |
| GO:0035621 | ER to Golgi ceramide transport | 2 | 0 | 0.08 | 1 |
| GO:0035622 | intrahepatic bile duct development | 2 | 0 | 0.08 | 1 |
| GO:0035623 | renal glucose absorption | 1 | 0 | 0.04 | 1 |
| GO:0035624 | receptor transactivation | 5 | 0 | 0.19 | 1 |
| GO:0035625 | epidermal growth factor-activated recept... | 4 | 0 | 0.15 | 1 |
| GO:0035627 | ceramide transport | 3 | 0 | 0.11 | 1 |
| GO:0035633 | maintenance of blood-brain barrier | 1 | 0 | 0.04 | 1 |
| GO:0035634 | response to stilbenoid | 7 | 0 | 0.27 | 1 |
| GO:0035635 | entry of bacterium into host cell | 2 | 0 | 0.08 | 1 |
| GO:0035638 | signal maturation | 1 | 0 | 0.04 | 1 |
| GO:0035640 | exploration behavior | 18 | 0 | 0.69 | 1 |
| GO:0035641 | locomotory exploration behavior | 8 | 0 | 0.3 | 1 |
| GO:0035644 | phosphoanandamide dephosphorylation | 1 | 0 | 0.04 | 1 |
| GO:0035645 | enteric smooth muscle cell differentiati... | 1 | 0 | 0.04 | 1 |
| GO:0035646 | endosome to melanosome transport | 7 | 0 | 0.27 | 1 |
| GO:0035655 | interleukin-18-mediated signaling pathwa.. | 2 | 0 | 0.08 | 1 |
| GO:0035664 | TIRAP-dependent toll-like receptor signa... | 1 | 0 | 0.04 | 1 |
| GO:0035665 | TIRAP-dependent toll-like receptor 4 sig... | 1 | 0 | 0.04 | 1 |
| GO:0035668 | TRAM-dependent toll-like receptor signal.. | 2 | 0 | 0.08 | 1 |
| GO:0035669 | TRAM-dependent toll-like receptor 4 sign.. | 2 | 0 | 0.08 | 1 |
| GO:0035672 | oligopeptide transmembrane transport | 4 | 0 | 0.15 | 1 |
| GO:0035674 | tricarboxylic acid transmembrane transpo.. | 1 | 0 | 0.04 | 1 |
| GO:0035691 | macrophage migration inhibitory factor s... | 2 | 0 | 0.08 | 1 |
| GO:0035695 | mitochondrion degradation by induced vac | 1 | 0 | 0.04 | 1 |
| GO:0035696 | monocyte extravasation | 3 | 0 | 0.11 | 1 |
| GO:0035697 | CD8-positive | alpha-beta | 3 | 0 | 0.11 |
| GO:0035698 | CD8-positive | alpha-beta | 2 | 0 | 0.08 |
| GO:0035700 | astrocyte chemotaxis | 1 | 0 | 0.04 | 1 |
| GO:0035701 | hematopoietic stem cell migration | 4 | 0 | 0.15 | 1 |
| GO:0035704 | helper T cell chemotaxis | 1 | 0 | 0.04 | 1 |
| GO:0035705 | T-helper 17 cell chemotaxis | 1 | 0 | 0.04 | 1 |
| GO:0035708 | interleukin-4-dependent isotype switchin... | 1 | 0 | 0.04 | 1 |
| GO:0035709 | memory T cell activation | 2 | 0 | 0.08 | 1 |
| GO:0035710 | CD4-positive | alpha-beta | 52 | 0 | 1.98 |
| GO:0035711 | T-helper 1 cell activation | 1 | 0 | 0.04 | 1 |
| GO:0035712 | T-helper 2 cell activation | 2 | 0 | 0.08 | 1 |
| GO:0035713 | response to nitrogen dioxide | 1 | 0 | 0.04 | 1 |
| GO:0035714 | cellular response to nitrogen dioxide | 1 | 0 | 0.04 | 1 |
| GO:0035720 | intraciliary anterograde transport | 1 | 0 | 0.04 | 1 |
| GO:0035721 | intraciliary retrograde transport | 6 | 0 | 0.23 | 1 |
| GO:0035722 | interleukin-12-mediated signaling pathwa.. | 3 | 0 | 0.11 | 1 |
| GO:0035723 | interleukin-15-mediated signaling pathwa.. | 1 | 0 | 0.04 | 1 |

| | | | | | |
|---|---|---|---|---|---|
| GO:0035724 | CD24 biosynthetic process | 1 | 0 | 0.04 | 1 |
| GO:0035726 | common myeloid progenitor cell prolifera... | 4 | 0 | 0.15 | 1 |
| GO:0035732 | nitric oxide storage | 1 | 0 | 0.04 | 1 |
| GO:0035735 | intraciliary transport involved in ciliu... | 5 | 0 | 0.19 | 1 |
| GO:0035739 | CD4-positive | alpha-beta | 2 | 0 | 0.08 |
| GO:0035740 | CD8-positive | alpha-beta | 4 | 0 | 0.15 |
| GO:0035743 | CD4-positive | alpha-beta | 7 | 0 | 0.27 |
| GO:0035744 | T-helper 1 cell cytokine production | 1 | 0 | 0.04 | 1 |
| GO:0035745 | T-helper 2 cell cytokine production | 6 | 0 | 0.23 | 1 |
| GO:0035746 | granzyme A production | 1 | 0 | 0.04 | 1 |
| GO:0035750 | protein localization to myelin sheath ab... | 1 | 0 | 0.04 | 1 |
| GO:0035751 | regulation of lysosomal lumen pH | 5 | 0 | 0.19 | 1 |
| GO:0035752 | lysosomal lumen pH elevation | 1 | 0 | 0.04 | 1 |
| GO:0035754 | B cell chemotaxis | 5 | 0 | 0.19 | 1 |
| GO:0035759 | mesangial cell-matrix adhesion | 2 | 0 | 0.08 | 1 |
| GO:0035766 | cell chemotaxis to fibroblast growth fac... | 6 | 0 | 0.23 | 1 |
| GO:0035768 | endothelial cell chemotaxis to fibroblas... | 6 | 0 | 0.23 | 1 |
| GO:0035769 | B cell chemotaxis across high endothelia... | 1 | 0 | 0.04 | 1 |
| GO:0035772 | interleukin-13-mediated signaling pathwa... | 1 | 0 | 0.04 | 1 |
| GO:0035774 | positive regulation of insulin secretion... | 20 | 0 | 0.76 | 1 |
| GO:0035782 | mature natural killer cell chemotaxis | 1 | 0 | 0.04 | 1 |
| GO:0035783 | CD4-positive | alpha-beta | 1 | 0 | 0.04 |
| GO:0035787 | cell migration involved in kidney develo... | 5 | 0 | 0.19 | 1 |
| GO:0035788 | cell migration involved in metanephros d... | 4 | 0 | 0.15 | 1 |
| GO:0035789 | metanephric mesenchymal cell migration | 4 | 0 | 0.15 | 1 |
| GO:0035790 | platelet-derived growth factor receptor-... | 3 | 0 | 0.11 | 1 |
| GO:0035793 | positive regulation of metanephric mesen... | 3 | 0 | 0.11 | 1 |
| GO:0035795 | negative regulation of mitochondrial mem... | 1 | 0 | 0.04 | 1 |
| GO:0035799 | ureter maturation | 4 | 0 | 0.15 | 1 |
| GO:0035801 | adrenal cortex development | 2 | 0 | 0.08 | 1 |
| GO:0035802 | adrenal cortex formation | 2 | 0 | 0.08 | 1 |
| GO:0035803 | egg coat formation | 2 | 0 | 0.08 | 1 |
| GO:0035814 | negative regulation of renal sodium excr... | 5 | 0 | 0.19 | 1 |
| GO:0035826 | rubidium ion transport | 6 | 0 | 0.23 | 1 |
| GO:0035844 | cloaca development | 4 | 0 | 0.15 | 1 |
| GO:0035845 | photoreceptor cell outer segment organiz... | 6 | 0 | 0.23 | 1 |
| GO:0035846 | oviduct epithelium development | 1 | 0 | 0.04 | 1 |
| GO:0035847 | uterine epithelium development | 1 | 0 | 0.04 | 1 |
| GO:0035849 | nephric duct elongation | 1 | 0 | 0.04 | 1 |
| GO:0035850 | epithelial cell differentiation involved... | 36 | 0 | 1.37 | 1 |
| GO:0035852 | horizontal cell localization | 1 | 0 | 0.04 | 1 |
| GO:0035853 | chromosome passenger complex localizati | 1 | 0 | 0.04 | 1 |
| GO:0035854 | eosinophil fate commitment | 2 | 0 | 0.08 | 1 |
| GO:0035860 | glial cell-derived neurotrophic factor r... | 6 | 0 | 0.23 | 1 |
| GO:0035862 | dITP metabolic process | 1 | 0 | 0.04 | 1 |
| GO:0035863 | dITP catabolic process | 1 | 0 | 0.04 | 1 |
| GO:0035871 | protein K11-linked deubiquitination | 9 | 0 | 0.34 | 1 |
| GO:0035873 | lactate transmembrane transport | 7 | 0 | 0.27 | 1 |
| GO:0035878 | nail development | 8 | 0 | 0.3 | 1 |
| GO:0035879 | plasma membrane lactate transport | 7 | 0 | 0.27 | 1 |
| GO:0035880 | embryonic nail plate morphogenesis | 3 | 0 | 0.11 | 1 |
| GO:0035886 | vascular smooth muscle cell differentiat... | 14 | 0 | 0.53 | 1 |
| GO:0035887 | aortic smooth muscle cell differentiatio... | 2 | 0 | 0.08 | 1 |

| | | | | | |
|---|---|---|---|---|---|
| GO:0035898 | parathyroid hormone secretion | 1 | 0 | 0.04 | 1 |
| GO:0035900 | response to isolation stress | 1 | 0 | 0.04 | 1 |
| GO:0035902 | response to immobilization stress | 17 | 0 | 0.65 | 1 |
| GO:0035905 | ascending aorta development | 3 | 0 | 0.11 | 1 |
| GO:0035910 | ascending aorta morphogenesis | 3 | 0 | 0.11 | 1 |
| GO:0035921 | desmosome disassembly | 1 | 0 | 0.04 | 1 |
| GO:0035922 | foramen ovale closure | 2 | 0 | 0.08 | 1 |
| GO:0035926 | chemokine (C-C motif) ligand 2 secretion | 2 | 0 | 0.08 | 1 |
| GO:0035933 | glucocorticoid secretion | 9 | 0 | 0.34 | 1 |
| GO:0035934 | corticosterone secretion | 4 | 0 | 0.15 | 1 |
| GO:0035935 | androgen secretion | 2 | 0 | 0.08 | 1 |
| GO:0035936 | testosterone secretion | 1 | 0 | 0.04 | 1 |
| GO:0035945 | mitochondrial ncRNA surveillance | 1 | 0 | 0.04 | 1 |
| GO:0035946 | mitochondrial mRNA surveillance | 1 | 0 | 0.04 | 1 |
| GO:0035962 | response to interleukin-13 | 3 | 0 | 0.11 | 1 |
| GO:0035963 | cellular response to interleukin-13 | 3 | 0 | 0.11 | 1 |
| GO:0035965 | cardiolipin acyl-chain remodeling | 7 | 0 | 0.27 | 1 |
| GO:0035971 | peptidyl-histidine dephosphorylation | 1 | 0 | 0.04 | 1 |
| GO:0035973 | aggrephagy | 1 | 0 | 0.04 | 1 |
| GO:0035978 | histone H2A-S139 phosphorylation | 1 | 0 | 0.04 | 1 |
| GO:0035981 | tongue muscle cell differentiation | 2 | 0 | 0.08 | 1 |
| GO:0035983 | response to trichostatin A | 1 | 0 | 0.04 | 1 |
| GO:0035984 | cellular response to trichostatin A | 1 | 0 | 0.04 | 1 |
| GO:0035986 | senescence-associated heterochromatin fo | 3 | 0 | 0.11 | 1 |
| GO:0035988 | chondrocyte proliferation | 12 | 0 | 0.46 | 1 |
| GO:0035989 | tendon development | 6 | 0 | 0.23 | 1 |
| GO:0035990 | tendon cell differentiation | 4 | 0 | 0.15 | 1 |
| GO:0035992 | tendon formation | 4 | 0 | 0.15 | 1 |
| GO:0035993 | deltoid tuberosity development | 2 | 0 | 0.08 | 1 |
| GO:0035995 | detection of muscle stretch | 3 | 0 | 0.11 | 1 |
| GO:0035999 | tetrahydrofolate interconversion | 7 | 0 | 0.27 | 1 |
| GO:0036003 | positive regulation of transcription fro... | 19 | 0 | 0.72 | 1 |
| GO:0036010 | protein localization to endosome | 10 | 0 | 0.38 | 1 |
| GO:0036015 | response to interleukin-3 | 5 | 0 | 0.19 | 1 |
| GO:0036016 | cellular response to interleukin-3 | 5 | 0 | 0.19 | 1 |
| GO:0036017 | response to erythropoietin | 4 | 0 | 0.15 | 1 |
| GO:0036018 | cellular response to erythropoietin | 4 | 0 | 0.15 | 1 |
| GO:0036022 | limb joint morphogenesis | 3 | 0 | 0.11 | 1 |
| GO:0036023 | embryonic skeletal limb joint morphogene. | 3 | 0 | 0.11 | 1 |
| GO:0036031 | recruitment of mRNA capping enzyme to F | 1 | 0 | 0.04 | 1 |
| GO:0036034 | mediator complex assembly | 1 | 0 | 0.04 | 1 |
| GO:0036035 | osteoclast development | 13 | 0 | 0.49 | 1 |
| GO:0036037 | CD8-positive | alpha-beta | 12 | 0 | 0.46 |
| GO:0036046 | protein demalonylation | 1 | 0 | 0.04 | 1 |
| GO:0036047 | peptidyl-lysine demalonylation | 1 | 0 | 0.04 | 1 |
| GO:0036048 | protein desuccinylation | 1 | 0 | 0.04 | 1 |
| GO:0036049 | peptidyl-lysine desuccinylation | 1 | 0 | 0.04 | 1 |
| GO:0036058 | filtration diaphragm assembly | 1 | 0 | 0.04 | 1 |
| GO:0036060 | slit diaphragm assembly | 1 | 0 | 0.04 | 1 |
| GO:0036071 | N-glycan fucosylation | 1 | 0 | 0.04 | 1 |
| GO:0036072 | direct ossification | 6 | 0 | 0.23 | 1 |
| GO:0036088 | D-serine catabolic process | 1 | 0 | 0.04 | 1 |
| GO:0036089 | cleavage furrow formation | 7 | 0 | 0.27 | 1 |

| | | | | | |
|---|---|---|---|---|---|
| GO:0036090 | cleavage furrow ingression | 1 | 0 | 0.04 | 1 |
| GO:0036091 | positive regulation of transcription fro... | 4 | 0 | 0.15 | 1 |
| GO:0036092 | phosphatidylinositol-3-phosphate biosynt... | 15 | 0 | 0.57 | 1 |
| GO:0036093 | germ cell proliferation | 5 | 0 | 0.19 | 1 |
| GO:0036100 | leukotriene catabolic process | 4 | 0 | 0.15 | 1 |
| GO:0036101 | leukotriene B4 catabolic process | 4 | 0 | 0.15 | 1 |
| GO:0036102 | leukotriene B4 metabolic process | 4 | 0 | 0.15 | 1 |
| GO:0036109 | alpha-linolenic acid metabolic process | 11 | 0 | 0.42 | 1 |
| GO:0036111 | very long-chain fatty-acyl-CoA metabolic... | 1 | 0 | 0.04 | 1 |
| GO:0036112 | medium-chain fatty-acyl-CoA metabolic pr. | 2 | 0 | 0.08 | 1 |
| GO:0036114 | medium-chain fatty-acyl-CoA catabolic pr.. | 1 | 0 | 0.04 | 1 |
| GO:0036115 | fatty-acyl-CoA catabolic process | 2 | 0 | 0.08 | 1 |
| GO:0036116 | long-chain fatty-acyl-CoA catabolic proc... | 1 | 0 | 0.04 | 1 |
| GO:0036118 | hyaluranon cable assembly | 3 | 0 | 0.11 | 1 |
| GO:0036123 | histone H3-K9 dimethylation | 3 | 0 | 0.11 | 1 |
| GO:0036124 | histone H3-K9 trimethylation | 6 | 0 | 0.23 | 1 |
| GO:0036138 | peptidyl-histidine hydroxylation | 1 | 0 | 0.04 | 1 |
| GO:0036148 | phosphatidylglycerol acyl-chain remodeli... | 17 | 0 | 0.65 | 1 |
| GO:0036149 | phosphatidylinositol acyl-chain remodeli... | 15 | 0 | 0.57 | 1 |
| GO:0036150 | phosphatidylserine acyl-chain remodeling | 17 | 0 | 0.65 | 1 |
| GO:0036152 | phosphatidylethanolamine acyl-chain remc | 22 | 0 | 0.84 | 1 |
| GO:0036153 | triglyceride acyl-chain remodeling | 1 | 0 | 0.04 | 1 |
| GO:0036155 | acylglycerol acyl-chain remodeling | 5 | 0 | 0.19 | 1 |
| GO:0036158 | outer dynein arm assembly | 6 | 0 | 0.23 | 1 |
| GO:0036159 | inner dynein arm assembly | 5 | 0 | 0.19 | 1 |
| GO:0036179 | osteoclast maturation | 1 | 0 | 0.04 | 1 |
| GO:0036233 | glycine import | 1 | 0 | 0.04 | 1 |
| GO:0036245 | cellular response to menadione | 2 | 0 | 0.08 | 1 |
| GO:0036250 | peroxisome transport along microtubule | 1 | 0 | 0.04 | 1 |
| GO:0036257 | multivesicular body organization | 2 | 0 | 0.08 | 1 |
| GO:0036258 | multivesicular body assembly | 1 | 0 | 0.04 | 1 |
| GO:0036260 | RNA capping | 32 | 0 | 1.22 | 1 |
| GO:0036261 | 7-methylguanosine cap hypermethylation | 1 | 0 | 0.04 | 1 |
| GO:0036265 | RNA (guanine-N7)-methylation | 3 | 0 | 0.11 | 1 |
| GO:0036269 | swimming behavior | 1 | 0 | 0.04 | 1 |
| GO:0036270 | response to diuretic | 1 | 0 | 0.04 | 1 |
| GO:0036273 | response to statin | 2 | 0 | 0.08 | 1 |
| GO:0036289 | peptidyl-serine autophosphorylation | 5 | 0 | 0.19 | 1 |
| GO:0036290 | protein trans-autophosphorylation | 1 | 0 | 0.04 | 1 |
| GO:0036292 | DNA rewinding | 2 | 0 | 0.08 | 1 |
| GO:0036295 | cellular response to increased oxygen le... | 7 | 0 | 0.27 | 1 |
| GO:0036297 | interstrand cross-link repair | 5 | 0 | 0.19 | 1 |
| GO:0036301 | macrophage colony-stimulating factor pro.. | 3 | 0 | 0.11 | 1 |
| GO:0036302 | atrioventricular canal development | 7 | 0 | 0.27 | 1 |
| GO:0036306 | embryonic heart tube elongation | 1 | 0 | 0.04 | 1 |
| GO:0036309 | protein localization to M-band | 2 | 0 | 0.08 | 1 |
| GO:0036323 | vascular endothelial growth factor recep... | 1 | 0 | 0.04 | 1 |
| GO:0036324 | vascular endothelial growth factor recep... | 2 | 0 | 0.08 | 1 |
| GO:0036334 | epidermal stem cell homeostasis | 1 | 0 | 0.04 | 1 |
| GO:0036337 | Fas signaling pathway | 3 | 0 | 0.11 | 1 |
| GO:0036344 | platelet morphogenesis | 16 | 0 | 0.61 | 1 |
| GO:0036351 | histone H2A-K13 ubiquitination | 1 | 0 | 0.04 | 1 |
| GO:0036352 | histone H2A-K15 ubiquitination | 1 | 0 | 0.04 | 1 |

| | | | | | |
|---|---|---|---|---|---|
| GO:0036353 | histone H2A-K119 monoubiquitination | 2 | 0 | 0.08 | 1 |
| GO:0036363 | transforming growth factor beta activati... | 1 | 0 | 0.04 | 1 |
| GO:0036367 | light adaption | 2 | 0 | 0.08 | 1 |
| GO:0036371 | protein localization to T-tubule | 1 | 0 | 0.04 | 1 |
| GO:0036372 | opsin transport | 1 | 0 | 0.04 | 1 |
| GO:0036376 | sodium ion export from cell | 8 | 0 | 0.3 | 1 |
| GO:0036378 | calcitriol biosynthetic process from cal... | 4 | 0 | 0.15 | 1 |
| GO:0036399 | TCR signalosome assembly | 1 | 0 | 0.04 | 1 |
| GO:0036404 | conversion of ds siRNA to ss siRNA | 1 | 0 | 0.04 | 1 |
| GO:0036413 | histone H3-R26 citrullination | 2 | 0 | 0.08 | 1 |
| GO:0036414 | histone citrullination | 2 | 0 | 0.08 | 1 |
| GO:0036462 | TRAIL-activated apoptotic signaling path... | 4 | 0 | 0.15 | 1 |
| GO:0036466 | synaptic vesicle recycling via endosome | 1 | 0 | 0.04 | 1 |
| GO:0036471 | cellular response to glyoxal | 1 | 0 | 0.04 | 1 |
| GO:0036475 | neuron death in response to oxidative st... | 9 | 0 | 0.34 | 1 |
| GO:0036476 | neuron death in response to hydrogen per. | 3 | 0 | 0.11 | 1 |
| GO:0036480 | neuron intrinsic apoptotic signaling pat... | 7 | 0 | 0.27 | 1 |
| GO:0036481 | intrinsic apoptotic signaling pathway in... | 4 | 0 | 0.15 | 1 |
| GO:0036482 | neuron intrinsic apoptotic signaling pat... | 2 | 0 | 0.08 | 1 |
| GO:0036483 | neuron intrinsic apoptotic signaling pat... | 1 | 0 | 0.04 | 1 |
| GO:0036484 | trunk neural crest cell migration | 3 | 0 | 0.11 | 1 |
| GO:0036486 | ventral trunk neural crest cell migratio... | 3 | 0 | 0.11 | 1 |
| GO:0036493 | positive regulation of translation in re... | 1 | 0 | 0.04 | 1 |
| GO:0036496 | regulation of translational initiation b... | 2 | 0 | 0.08 | 1 |
| GO:0036497 | eIF2alpha dephosphorylation in response . | 1 | 0 | 0.04 | 1 |
| GO:0036499 | PERK-mediated unfolded protein response | 7 | 0 | 0.27 | 1 |
| GO:0036500 | ATF6-mediated unfolded protein response | 2 | 0 | 0.08 | 1 |
| GO:0038001 | paracrine signaling | 3 | 0 | 0.11 | 1 |
| GO:0038009 | regulation of signal transduction by rec... | 1 | 0 | 0.04 | 1 |
| GO:0038016 | insulin receptor internalization | 1 | 0 | 0.04 | 1 |
| GO:0038018 | Wnt receptor catabolic process | 1 | 0 | 0.04 | 1 |
| GO:0038026 | reelin-mediated signaling pathway | 4 | 0 | 0.15 | 1 |
| GO:0038027 | apolipoprotein A-I-mediated signaling pa... | 5 | 0 | 0.19 | 1 |
| GO:0038028 | insulin receptor signaling pathway via p... | 3 | 0 | 0.11 | 1 |
| GO:0038030 | non-canonical Wnt signaling pathway via .. | 7 | 0 | 0.27 | 1 |
| GO:0038031 | non-canonical Wnt signaling pathway via .. | 6 | 0 | 0.23 | 1 |
| GO:0038042 | dimeric G-protein coupled receptor signa... | 1 | 0 | 0.04 | 1 |
| GO:0038043 | interleukin-5-mediated signaling pathway | 2 | 0 | 0.08 | 1 |
| GO:0038044 | transforming growth factor-beta secretio... | 1 | 0 | 0.04 | 1 |
| GO:0038060 | nitric oxide-cGMP-mediated signaling pat... | 1 | 0 | 0.04 | 1 |
| GO:0038063 | collagen-activated tyrosine kinase recep... | 2 | 0 | 0.08 | 1 |
| GO:0038065 | collagen-activated signaling pathway | 4 | 0 | 0.15 | 1 |
| GO:0038066 | p38MAPK cascade | 14 | 0 | 0.53 | 1 |
| GO:0038086 | VEGF-activated platelet-derived growth f... | 3 | 0 | 0.11 | 1 |
| GO:0038091 | positive regulation of cell proliferatio... | 3 | 0 | 0.11 | 1 |
| GO:0038092 | nodal signaling pathway | 14 | 0 | 0.53 | 1 |
| GO:0038098 | sequestering of BMP from receptor via BM | 2 | 0 | 0.08 | 1 |
| GO:0038101 | sequestering of nodal from receptor via ... | 1 | 0 | 0.04 | 1 |
| GO:0038107 | nodal signaling pathway involved in dete... | 5 | 0 | 0.19 | 1 |
| GO:0038108 | negative regulation of appetite by lepti... | 3 | 0 | 0.11 | 1 |
| GO:0038111 | interleukin-7-mediated signaling pathway | 2 | 0 | 0.08 | 1 |
| GO:0038112 | interleukin-8-mediated signaling pathway | 1 | 0 | 0.04 | 1 |
| GO:0038113 | interleukin-9-mediated signaling pathway | 1 | 0 | 0.04 | 1 |

| | | | | | |
|---|---|---|---|---|---|
| GO:0038114 | interleukin-21-mediated signaling pathwa.. | 1 | 0 | 0.04 | 1 |
| GO:0038115 | chemokine (C-C motif) ligand 19 signalin... | 1 | 0 | 0.04 | 1 |
| GO:0038116 | chemokine (C-C motif) ligand 21 signalin... | 1 | 0 | 0.04 | 1 |
| GO:0038118 | C-C chemokine receptor CCR7 signaling p | 1 | 0 | 0.04 | 1 |
| GO:0038128 | ERBB2 signaling pathway | 2 | 0 | 0.08 | 1 |
| GO:0038129 | ERBB3 signaling pathway | 1 | 0 | 0.04 | 1 |
| GO:0038133 | ERBB2-ERBB3 signaling pathway | 1 | 0 | 0.04 | 1 |
| GO:0038154 | interleukin-11-mediated signaling pathwa.. | 1 | 0 | 0.04 | 1 |
| GO:0038155 | interleukin-23-mediated signaling pathwa.. | 2 | 0 | 0.08 | 1 |
| GO:0038156 | interleukin-3-mediated signaling pathway | 2 | 0 | 0.08 | 1 |
| GO:0038158 | granulocyte colony-stimulating factor si... | 1 | 0 | 0.04 | 1 |
| GO:0038162 | erythropoietin-mediated signaling pathwa.. | 2 | 0 | 0.08 | 1 |
| GO:0038163 | thrombopoietin-mediated signaling pathwa | 2 | 0 | 0.08 | 1 |
| GO:0038165 | oncostatin-M-mediated signaling pathway | 4 | 0 | 0.15 | 1 |
| GO:0038166 | angiotensin-activated signaling pathway | 9 | 0 | 0.34 | 1 |
| GO:0038169 | somatostatin receptor signaling pathway | 5 | 0 | 0.19 | 1 |
| GO:0038170 | somatostatin signaling pathway | 5 | 0 | 0.19 | 1 |
| GO:0038171 | cannabinoid signaling pathway | 6 | 0 | 0.23 | 1 |
| GO:0038172 | interleukin-33-mediated signaling pathwa.. | 1 | 0 | 0.04 | 1 |
| GO:0038178 | complement component C5a signaling pat | 1 | 0 | 0.04 | 1 |
| GO:0038180 | nerve growth factor signaling pathway | 9 | 0 | 0.34 | 1 |
| GO:0038183 | bile acid signaling pathway | 3 | 0 | 0.11 | 1 |
| GO:0038184 | cell surface bile acid receptor signalin... | 1 | 0 | 0.04 | 1 |
| GO:0038185 | intracellular bile acid receptor signali... | 1 | 0 | 0.04 | 1 |
| GO:0038188 | cholecystokinin signaling pathway | 2 | 0 | 0.08 | 1 |
| GO:0038189 | neuropilin signaling pathway | 2 | 0 | 0.08 | 1 |
| GO:0038190 | VEGF-activated neuropilin signaling path.. | 2 | 0 | 0.08 | 1 |
| GO:0038192 | gastric inhibitory peptide signaling pat... | 1 | 0 | 0.04 | 1 |
| GO:0038193 | thromboxane A2 signaling pathway | 1 | 0 | 0.04 | 1 |
| GO:0038194 | thyroid-stimulating hormone signaling pa... | 2 | 0 | 0.08 | 1 |
| GO:0038195 | urokinase plasminogen activator signalin... | 1 | 0 | 0.04 | 1 |
| GO:0038203 | TORC2 signaling | 1 | 0 | 0.04 | 1 |
| GO:0039003 | pronephric field specification | 2 | 0 | 0.08 | 1 |
| GO:0039007 | pronephric nephron morphogenesis | 1 | 0 | 0.04 | 1 |
| GO:0039008 | pronephric nephron tubule morphogenesis | 1 | 0 | 0.04 | 1 |
| GO:0039017 | pattern specification involved in pronep... | 2 | 0 | 0.08 | 1 |
| GO:0039019 | pronephric nephron development | 2 | 0 | 0.08 | 1 |
| GO:0039020 | pronephric nephron tubule development | 2 | 0 | 0.08 | 1 |
| GO:0039022 | pronephric duct development | 1 | 0 | 0.04 | 1 |
| GO:0039023 | pronephric duct morphogenesis | 1 | 0 | 0.04 | 1 |
| GO:0039519 | modulation by virus of host autophagy | 1 | 0 | 0.04 | 1 |
| GO:0039521 | suppression by virus of host autophagy | 1 | 0 | 0.04 | 1 |
| GO:0039526 | modulation by virus of host apoptotic pr... | 2 | 0 | 0.08 | 1 |
| GO:0039528 | cytoplasmic pattern recognition receptor... | 15 | 0 | 0.57 | 1 |
| GO:0039529 | RIG-I signaling pathway | 11 | 0 | 0.42 | 1 |
| GO:0039530 | MDA-5 signaling pathway | 6 | 0 | 0.23 | 1 |
| GO:0039531 | regulation of viral-induced cytoplasmic ... | 10 | 0 | 0.38 | 1 |
| GO:0039532 | negative regulation of viral-induced cyt... | 4 | 0 | 0.15 | 1 |
| GO:0039533 | regulation of MDA-5 signaling pathway | 4 | 0 | 0.15 | 1 |
| GO:0039534 | negative regulation of MDA-5 signaling p... | 2 | 0 | 0.08 | 1 |
| GO:0039535 | regulation of RIG-I signaling pathway | 9 | 0 | 0.34 | 1 |
| GO:0039536 | negative regulation of RIG-I signaling p... | 3 | 0 | 0.11 | 1 |
| GO:0039663 | membrane fusion involved in viral entry ... | 3 | 0 | 0.11 | 1 |

| | | | | | |
|---|---|---|---|---|---|
| GO:0039689 | negative stranded viral RNA replication | 4 | 0 | 0.15 | 1 |
| GO:0039692 | single stranded viral RNA replication vi... | 9 | 0 | 0.34 | 1 |
| GO:0039694 | viral RNA genome replication | 13 | 0 | 0.49 | 1 |
| GO:0039702 | viral budding via host ESCRT complex | 12 | 0 | 0.46 | 1 |
| GO:0039703 | RNA replication | 13 | 0 | 0.49 | 1 |
| GO:0040001 | establishment of mitotic spindle localiz... | 20 | 0 | 0.76 | 1 |
| GO:0040009 | regulation of growth rate | 3 | 0 | 0.11 | 1 |
| GO:0040016 | embryonic cleavage | 7 | 0 | 0.27 | 1 |
| GO:0040019 | positive regulation of embryonic develop... | 24 | 0 | 0.91 | 1 |
| GO:0040030 | regulation of molecular function | epigen... | 2 | 0 | 0.08 |
| GO:0040031 | snRNA modification | 2 | 0 | 0.08 | 1 |
| GO:0040032 | post-embryonic body morphogenesis | 1 | 0 | 0.04 | 1 |
| GO:0040033 | negative regulation of translation | ncRN... | 5 | 0 | 0.19 |
| GO:0040034 | regulation of development | heterochror | 12 | 0 | 0.46 |
| GO:0040038 | polar body extrusion after meiotic divis... | 4 | 0 | 0.15 | 1 |
| GO:0040040 | thermosensory behavior | 1 | 0 | 0.04 | 1 |
| GO:0042000 | translocation of peptides or proteins in... | 1 | 0 | 0.04 | 1 |
| GO:0042033 | chemokine biosynthetic process | 13 | 0 | 0.49 | 1 |
| GO:0042036 | negative regulation of cytokine biosynth... | 27 | 0 | 1.03 | 1 |
| GO:0042040 | metal incorporation into metallo-molybdo... | 1 | 0 | 0.04 | 1 |
| GO:0042053 | regulation of dopamine metabolic process | 15 | 0 | 0.57 | 1 |
| GO:0042069 | regulation of catecholamine metabolic pr... | 15 | 0 | 0.57 | 1 |
| GO:0042073 | intraciliary transport | 24 | 0 | 0.91 | 1 |
| GO:0042078 | germ-line stem cell division | 3 | 0 | 0.11 | 1 |
| GO:0042088 | T-helper 1 type immune response | 37 | 0 | 1.41 | 1 |
| GO:0042090 | interleukin-12 biosynthetic process | 9 | 0 | 0.34 | 1 |
| GO:0042091 | interleukin-10 biosynthetic process | 3 | 0 | 0.11 | 1 |
| GO:0042092 | type 2 immune response | 28 | 0 | 1.07 | 1 |
| GO:0042093 | T-helper cell differentiation | 38 | 0 | 1.45 | 1 |
| GO:0042094 | interleukin-2 biosynthetic process | 22 | 0 | 0.84 | 1 |
| GO:0042095 | interferon-gamma biosynthetic process | 17 | 0 | 0.65 | 1 |
| GO:0042097 | interleukin-4 biosynthetic process | 3 | 0 | 0.11 | 1 |
| GO:0042104 | positive regulation of activated T cell ... | 23 | 0 | 0.88 | 1 |
| GO:0042116 | macrophage activation | 46 | 0 | 1.75 | 1 |
| GO:0042117 | monocyte activation | 8 | 0 | 0.3 | 1 |
| GO:0042118 | endothelial cell activation | 9 | 0 | 0.34 | 1 |
| GO:0042126 | nitrate metabolic process | 1 | 0 | 0.04 | 1 |
| GO:0042135 | neurotransmitter catabolic process | 9 | 0 | 0.34 | 1 |
| GO:0042137 | sequestering of neurotransmitter | 2 | 0 | 0.08 | 1 |
| GO:0042138 | meiotic DNA double-strand break formatio. | 2 | 0 | 0.08 | 1 |
| GO:0042144 | vacuole fusion | non-autoph | 2 | 0 | 0.08 |
| GO:0042148 | strand invasion | 1 | 0 | 0.04 | 1 |
| GO:0042159 | lipoprotein catabolic process | 7 | 0 | 0.27 | 1 |
| GO:0042167 | heme catabolic process | 7 | 0 | 0.27 | 1 |
| GO:0042178 | xenobiotic catabolic process | 10 | 0 | 0.38 | 1 |
| GO:0042182 | ketone catabolic process | 7 | 0 | 0.27 | 1 |
| GO:0042196 | chlorinated hydrocarbon metabolic proces. | 1 | 0 | 0.04 | 1 |
| GO:0042197 | halogenated hydrocarbon metabolic proce | 1 | 0 | 0.04 | 1 |
| GO:0042214 | terpene metabolic process | 2 | 0 | 0.08 | 1 |
| GO:0042219 | cellular modified amino acid catabolic p... | 15 | 0 | 0.57 | 1 |
| GO:0042222 | interleukin-1 biosynthetic process | 3 | 0 | 0.11 | 1 |
| GO:0042223 | interleukin-3 biosynthetic process | 2 | 0 | 0.08 | 1 |
| GO:0042225 | interleukin-5 biosynthetic process | 1 | 0 | 0.04 | 1 |

| | | | | | |
|---|---|---|---|---|---|
| GO:0042231 | interleukin-13 biosynthetic process | 2 | 0 | 0.08 | 1 |
| GO:0042241 | interleukin-18 biosynthetic process | 1 | 0 | 0.04 | 1 |
| GO:0042245 | RNA repair | 2 | 0 | 0.08 | 1 |
| GO:0042253 | granulocyte macrophage colony-stimulatin | 5 | 0 | 0.19 | 1 |
| GO:0042256 | mature ribosome assembly | 6 | 0 | 0.23 | 1 |
| GO:0042262 | DNA protection | 2 | 0 | 0.08 | 1 |
| GO:0042264 | peptidyl-aspartic acid hydroxylation | 2 | 0 | 0.08 | 1 |
| GO:0042265 | peptidyl-asparagine hydroxylation | 1 | 0 | 0.04 | 1 |
| GO:0042268 | regulation of cytolysis | 8 | 0 | 0.3 | 1 |
| GO:0042271 | susceptibility to natural killer cell me... | 3 | 0 | 0.11 | 1 |
| GO:0042273 | ribosomal large subunit biogenesis | 20 | 0 | 0.76 | 1 |
| GO:0042275 | error-free postreplication DNA repair | 1 | 0 | 0.04 | 1 |
| GO:0042276 | error-prone translesion synthesis | 4 | 0 | 0.15 | 1 |
| GO:0042309 | homoiothermy | 2 | 0 | 0.08 | 1 |
| GO:0042312 | regulation of vasodilation | 39 | 0 | 1.48 | 1 |
| GO:0042320 | regulation of circadian sleep/wake cycle... | 6 | 0 | 0.23 | 1 |
| GO:0042321 | negative regulation of circadian sleep/w... | 5 | 0 | 0.19 | 1 |
| GO:0042322 | negative regulation of circadian sleep/w... | 2 | 0 | 0.08 | 1 |
| GO:0042323 | negative regulation of circadian sleep/w... | 2 | 0 | 0.08 | 1 |
| GO:0042335 | cuticle development | 3 | 0 | 0.11 | 1 |
| GO:0042340 | keratan sulfate catabolic process | 12 | 0 | 0.46 | 1 |
| GO:0042346 | positive regulation of NF-kappaB import ... | 24 | 0 | 0.91 | 1 |
| GO:0042350 | GDP-L-fucose biosynthetic process | 2 | 0 | 0.08 | 1 |
| GO:0042351 | 'de novo' GDP-L-fucose biosynthetic proc... | 2 | 0 | 0.08 | 1 |
| GO:0042354 | L-fucose metabolic process | 9 | 0 | 0.34 | 1 |
| GO:0042355 | L-fucose catabolic process | 9 | 0 | 0.34 | 1 |
| GO:0042357 | thiamine diphosphate metabolic process | 2 | 0 | 0.08 | 1 |
| GO:0042359 | vitamin D metabolic process | 20 | 0 | 0.76 | 1 |
| GO:0042360 | vitamin E metabolic process | 4 | 0 | 0.15 | 1 |
| GO:0042361 | menaquinone catabolic process | 2 | 0 | 0.08 | 1 |
| GO:0042362 | fat-soluble vitamin biosynthetic process | 16 | 0 | 0.61 | 1 |
| GO:0042363 | fat-soluble vitamin catabolic process | 8 | 0 | 0.3 | 1 |
| GO:0042368 | vitamin D biosynthetic process | 12 | 0 | 0.46 | 1 |
| GO:0042369 | vitamin D catabolic process | 3 | 0 | 0.11 | 1 |
| GO:0042371 | vitamin K biosynthetic process | 3 | 0 | 0.11 | 1 |
| GO:0042373 | vitamin K metabolic process | 7 | 0 | 0.27 | 1 |
| GO:0042374 | phylloquinone metabolic process | 3 | 0 | 0.11 | 1 |
| GO:0042376 | phylloquinone catabolic process | 3 | 0 | 0.11 | 1 |
| GO:0042377 | vitamin K catabolic process | 2 | 0 | 0.08 | 1 |
| GO:0042396 | phosphagen biosynthetic process | 1 | 0 | 0.04 | 1 |
| GO:0042401 | cellular biogenic amine biosynthetic pro... | 19 | 0 | 0.72 | 1 |
| GO:0042402 | cellular biogenic amine catabolic proces... | 16 | 0 | 0.61 | 1 |
| GO:0042403 | thyroid hormone metabolic process | 19 | 0 | 0.72 | 1 |
| GO:0042404 | thyroid hormone catabolic process | 1 | 0 | 0.04 | 1 |
| GO:0042407 | cristae formation | 6 | 0 | 0.23 | 1 |
| GO:0042412 | taurine biosynthetic process | 2 | 0 | 0.08 | 1 |
| GO:0042413 | carnitine catabolic process | 1 | 0 | 0.04 | 1 |
| GO:0042414 | epinephrine metabolic process | 3 | 0 | 0.11 | 1 |
| GO:0042415 | norepinephrine metabolic process | 11 | 0 | 0.42 | 1 |
| GO:0042416 | dopamine biosynthetic process | 11 | 0 | 0.42 | 1 |
| GO:0042417 | dopamine metabolic process | 32 | 0 | 1.22 | 1 |
| GO:0042418 | epinephrine biosynthetic process | 2 | 0 | 0.08 | 1 |
| GO:0042420 | dopamine catabolic process | 5 | 0 | 0.19 | 1 |

| | | | | | |
|---|---|---|---|---|---|
| GO:0042421 | norepinephrine biosynthetic process | 6 | 0 | 0.23 | 1 |
| GO:0042423 | catecholamine biosynthetic process | 19 | 0 | 0.72 | 1 |
| GO:0042424 | catecholamine catabolic process | 6 | 0 | 0.23 | 1 |
| GO:0042427 | serotonin biosynthetic process | 3 | 0 | 0.11 | 1 |
| GO:0042428 | serotonin metabolic process | 11 | 0 | 0.42 | 1 |
| GO:0042435 | indole-containing compound biosynthetic .. | 5 | 0 | 0.19 | 1 |
| GO:0042436 | indole-containing compound catabolic pro. | 11 | 0 | 0.42 | 1 |
| GO:0042438 | melanin biosynthetic process | 16 | 0 | 0.61 | 1 |
| GO:0042441 | eye pigment metabolic process | 4 | 0 | 0.15 | 1 |
| GO:0042447 | hormone catabolic process | 7 | 0 | 0.27 | 1 |
| GO:0042448 | progesterone metabolic process | 14 | 0 | 0.53 | 1 |
| GO:0042450 | arginine biosynthetic process via ornith... | 2 | 0 | 0.08 | 1 |
| GO:0042454 | ribonucleoside catabolic process | 22 | 0 | 0.84 | 1 |
| GO:0042483 | negative regulation of odontogenesis | 4 | 0 | 0.15 | 1 |
| GO:0042487 | regulation of odontogenesis of dentin-co... | 12 | 0 | 0.46 | 1 |
| GO:0042488 | positive regulation of odontogenesis of ... | 2 | 0 | 0.08 | 1 |
| GO:0042489 | negative regulation of odontogenesis of ... | 1 | 0 | 0.04 | 1 |
| GO:0042491 | auditory receptor cell differentiation | 30 | 0 | 1.14 | 1 |
| GO:0042492 | gamma-delta T cell differentiation | 9 | 0 | 0.34 | 1 |
| GO:0042494 | detection of bacterial lipoprotein | 3 | 0 | 0.11 | 1 |
| GO:0042495 | detection of triacyl bacterial lipopepti... | 2 | 0 | 0.08 | 1 |
| GO:0042496 | detection of diacyl bacterial lipopeptid... | 2 | 0 | 0.08 | 1 |
| GO:0042504 | tyrosine phosphorylation of Stat4 protei... | 4 | 0 | 0.15 | 1 |
| GO:0042511 | positive regulation of tyrosine phosphor... | 9 | 0 | 0.34 | 1 |
| GO:0042519 | regulation of tyrosine phosphorylation o... | 4 | 0 | 0.15 | 1 |
| GO:0042520 | positive regulation of tyrosine phosphor... | 4 | 0 | 0.15 | 1 |
| GO:0042523 | positive regulation of tyrosine phosphor... | 16 | 0 | 0.61 | 1 |
| GO:0042526 | positive regulation of tyrosine phosphor... | 1 | 0 | 0.04 | 1 |
| GO:0042533 | tumor necrosis factor biosynthetic proce... | 16 | 0 | 0.61 | 1 |
| GO:0042534 | regulation of tumor necrosis factor bios... | 16 | 0 | 0.61 | 1 |
| GO:0042535 | positive regulation of tumor necrosis fa... | 10 | 0 | 0.38 | 1 |
| GO:0042536 | negative regulation of tumor necrosis fa... | 5 | 0 | 0.19 | 1 |
| GO:0042537 | benzene-containing compound metabolic p | 26 | 0 | 0.99 | 1 |
| GO:0042539 | hypotonic salinity response | 1 | 0 | 0.04 | 1 |
| GO:0042541 | hemoglobin biosynthetic process | 10 | 0 | 0.38 | 1 |
| GO:0042560 | pteridine-containing compound catabolic .. | 2 | 0 | 0.08 | 1 |
| GO:0042595 | behavioral response to starvation | 2 | 0 | 0.08 | 1 |
| GO:0042628 | mating plug formation | 2 | 0 | 0.08 | 1 |
| GO:0042631 | cellular response to water deprivation | 3 | 0 | 0.11 | 1 |
| GO:0042634 | regulation of hair cycle | 19 | 0 | 0.72 | 1 |
| GO:0042635 | positive regulation of hair cycle | 9 | 0 | 0.34 | 1 |
| GO:0042636 | negative regulation of hair cycle | 4 | 0 | 0.15 | 1 |
| GO:0042637 | catagen | 4 | 0 | 0.15 | 1 |
| GO:0042640 | anagen | 2 | 0 | 0.08 | 1 |
| GO:0042660 | positive regulation of cell fate specifi... | 4 | 0 | 0.15 | 1 |
| GO:0042661 | regulation of mesodermal cell fate speci... | 7 | 0 | 0.27 | 1 |
| GO:0042662 | negative regulation of mesodermal cell f... | 4 | 0 | 0.15 | 1 |
| GO:0042664 | negative regulation of endodermal cell f... | 1 | 0 | 0.04 | 1 |
| GO:0042665 | regulation of ectodermal cell fate speci... | 1 | 0 | 0.04 | 1 |
| GO:0042666 | negative regulation of ectodermal cell f... | 1 | 0 | 0.04 | 1 |
| GO:0042667 | auditory receptor cell fate specificatio... | 1 | 0 | 0.04 | 1 |
| GO:0042668 | auditory receptor cell fate determinatio... | 3 | 0 | 0.11 | 1 |
| GO:0042684 | cardioblast cell fate commitment | 1 | 0 | 0.04 | 1 |

| | | | | | |
|---|---|---|---|---|---|
| GO:0042685 | cardioblast cell fate specification | 1 | 0 | 0.04 | 1 |
| GO:0042686 | regulation of cardioblast cell fate spec... | 1 | 0 | 0.04 | 1 |
| GO:0042693 | muscle cell fate commitment | 17 | 0 | 0.65 | 1 |
| GO:0042694 | muscle cell fate specification | 1 | 0 | 0.04 | 1 |
| GO:0042695 | thelarche | 6 | 0 | 0.23 | 1 |
| GO:0042699 | follicle-stimulating hormone signaling p... | 3 | 0 | 0.11 | 1 |
| GO:0042700 | luteinizing hormone signaling pathway | 2 | 0 | 0.08 | 1 |
| GO:0042701 | progesterone secretion | 5 | 0 | 0.19 | 1 |
| GO:0042703 | menstruation | 1 | 0 | 0.04 | 1 |
| GO:0042704 | uterine wall breakdown | 2 | 0 | 0.08 | 1 |
| GO:0042706 | eye photoreceptor cell fate commitment | 2 | 0 | 0.08 | 1 |
| GO:0042710 | biofilm formation | 1 | 0 | 0.04 | 1 |
| GO:0042713 | sperm ejaculation | 6 | 0 | 0.23 | 1 |
| GO:0042723 | thiamine-containing compound metabolic p | 6 | 0 | 0.23 | 1 |
| GO:0042730 | fibrinolysis | 24 | 0 | 0.91 | 1 |
| GO:0042732 | D-xylose metabolic process | 3 | 0 | 0.11 | 1 |
| GO:0042737 | drug catabolic process | 23 | 0 | 0.88 | 1 |
| GO:0042738 | exogenous drug catabolic process | 21 | 0 | 0.8 | 1 |
| GO:0042742 | defense response to bacterium | 188 | 0 | 7.16 | 1 |
| GO:0042743 | hydrogen peroxide metabolic process | 45 | 0 | 1.71 | 1 |
| GO:0042744 | hydrogen peroxide catabolic process | 23 | 0 | 0.88 | 1 |
| GO:0042745 | circadian sleep/wake cycle | 23 | 0 | 0.88 | 1 |
| GO:0042746 | circadian sleep/wake cycle | wakefulnes | 4 | 0 | 0.15 |
| GO:0042747 | circadian sleep/wake cycle | REM sleep | 7 | 0 | 0.27 |
| GO:0042748 | circadian sleep/wake cycle | non-REM s | 8 | 0 | 0.3 |
| GO:0042749 | regulation of circadian sleep/wake cycle | 18 | 0 | 0.69 | 1 |
| GO:0042753 | positive regulation of circadian rhythm | 16 | 0 | 0.61 | 1 |
| GO:0042756 | drinking behavior | 7 | 0 | 0.27 | 1 |
| GO:0042758 | long-chain fatty acid catabolic process | 7 | 0 | 0.27 | 1 |
| GO:0042759 | long-chain fatty acid biosynthetic proce... | 10 | 0 | 0.38 | 1 |
| GO:0042760 | very long-chain fatty acid catabolic pro... | 5 | 0 | 0.19 | 1 |
| GO:0042761 | very long-chain fatty acid biosynthetic ... | 7 | 0 | 0.27 | 1 |
| GO:0042766 | nucleosome mobilization | 1 | 0 | 0.04 | 1 |
| GO:0042769 | DNA damage response | detection of | 9 | 0 | 0.34 |
| GO:0042771 | intrinsic apoptotic signaling pathway in... | 39 | 0 | 1.48 | 1 |
| GO:0042779 | tRNA 3'-trailer cleavage | 2 | 0 | 0.08 | 1 |
| GO:0042780 | tRNA 3'-end processing | 4 | 0 | 0.15 | 1 |
| GO:0042790 | transcription of nuclear large rRNA tran... | 8 | 0 | 0.3 | 1 |
| GO:0042796 | snRNA transcription from RNA polymerase | 3 | 0 | 0.11 | 1 |
| GO:0042816 | vitamin B6 metabolic process | 4 | 0 | 0.15 | 1 |
| GO:0042819 | vitamin B6 biosynthetic process | 2 | 0 | 0.08 | 1 |
| GO:0042822 | pyridoxal phosphate metabolic process | 3 | 0 | 0.11 | 1 |
| GO:0042823 | pyridoxal phosphate biosynthetic process | 2 | 0 | 0.08 | 1 |
| GO:0042832 | defense response to protozoan | 17 | 0 | 0.65 | 1 |
| GO:0042839 | D-glucuronate metabolic process | 1 | 0 | 0.04 | 1 |
| GO:0042840 | D-glucuronate catabolic process | 1 | 0 | 0.04 | 1 |
| GO:0042843 | D-xylose catabolic process | 1 | 0 | 0.04 | 1 |
| GO:0042851 | L-alanine metabolic process | 4 | 0 | 0.15 | 1 |
| GO:0042853 | L-alanine catabolic process | 4 | 0 | 0.15 | 1 |
| GO:0042866 | pyruvate biosynthetic process | 4 | 0 | 0.15 | 1 |
| GO:0042883 | cysteine transport | 1 | 0 | 0.04 | 1 |
| GO:0042891 | antibiotic transport | 4 | 0 | 0.15 | 1 |
| GO:0042892 | chloramphenicol transport | 1 | 0 | 0.04 | 1 |

| | | | | | |
|---|---|---|---|---|---|
| GO:0042904 | 9-cis-retinoic acid biosynthetic process | 4 | 0 | 0.15 | 1 |
| GO:0042905 | 9-cis-retinoic acid metabolic process | 4 | 0 | 0.15 | 1 |
| GO:0042908 | xenobiotic transport | 5 | 0 | 0.19 | 1 |
| GO:0042921 | glucocorticoid receptor signaling pathwa... | 14 | 0 | 0.53 | 1 |
| GO:0042938 | dipeptide transport | 3 | 0 | 0.11 | 1 |
| GO:0042939 | tripeptide transport | 2 | 0 | 0.08 | 1 |
| GO:0042940 | D-amino acid transport | 5 | 0 | 0.19 | 1 |
| GO:0042941 | D-alanine transport | 1 | 0 | 0.04 | 1 |
| GO:0042942 | D-serine transport | 2 | 0 | 0.08 | 1 |
| GO:0042976 | activation of Janus kinase activity | 12 | 0 | 0.46 | 1 |
| GO:0042977 | activation of JAK2 kinase activity | 10 | 0 | 0.38 | 1 |
| GO:0042982 | amyloid precursor protein metabolic proc... | 29 | 0 | 1.1 | 1 |
| GO:0042983 | amyloid precursor protein biosynthetic p... | 7 | 0 | 0.27 | 1 |
| GO:0042984 | regulation of amyloid precursor protein ... | 7 | 0 | 0.27 | 1 |
| GO:0042985 | negative regulation of amyloid precursor... | 4 | 0 | 0.15 | 1 |
| GO:0042986 | positive regulation of amyloid precursor... | 2 | 0 | 0.08 | 1 |
| GO:0042987 | amyloid precursor protein catabolic proc... | 19 | 0 | 0.72 | 1 |
| GO:0042989 | sequestering of actin monomers | 8 | 0 | 0.3 | 1 |
| GO:0042993 | positive regulation of transcription fac... | 44 | 0 | 1.67 | 1 |
| GO:0042996 | regulation of Golgi to plasma membrane p. | 8 | 0 | 0.3 | 1 |
| GO:0042997 | negative regulation of Golgi to plasma m... | 6 | 0 | 0.23 | 1 |
| GO:0042998 | positive regulation of Golgi to plasma m... | 2 | 0 | 0.08 | 1 |
| GO:0042999 | regulation of Golgi to plasma membrane C | 1 | 0 | 0.04 | 1 |
| GO:0043000 | Golgi to plasma membrane CFTR protein t | 2 | 0 | 0.08 | 1 |
| GO:0043001 | Golgi to plasma membrane protein transpc | 32 | 0 | 1.22 | 1 |
| GO:0043002 | negative regulation of Golgi to plasma m... | 1 | 0 | 0.04 | 1 |
| GO:0043004 | cytoplasmic sequestering of CFTR protein | 1 | 0 | 0.04 | 1 |
| GO:0043006 | activation of phospholipase A2 activity ... | 2 | 0 | 0.08 | 1 |
| GO:0043011 | myeloid dendritic cell differentiation | 19 | 0 | 0.72 | 1 |
| GO:0043012 | regulation of fusion of sperm to egg pla... | 1 | 0 | 0.04 | 1 |
| GO:0043016 | regulation of lymphotoxin A biosynthetic... | 1 | 0 | 0.04 | 1 |
| GO:0043017 | positive regulation of lymphotoxin A bio... | 1 | 0 | 0.04 | 1 |
| GO:0043029 | T cell homeostasis | 37 | 0 | 1.41 | 1 |
| GO:0043030 | regulation of macrophage activation | 23 | 0 | 0.88 | 1 |
| GO:0043031 | negative regulation of macrophage activa... | 6 | 0 | 0.23 | 1 |
| GO:0043032 | positive regulation of macrophage activa... | 10 | 0 | 0.38 | 1 |
| GO:0043045 | DNA methylation involved in embryo devel | 4 | 0 | 0.15 | 1 |
| GO:0043046 | DNA methylation involved in gamete gene | 16 | 0 | 0.61 | 1 |
| GO:0043048 | dolichyl monophosphate biosynthetic proc. | 1 | 0 | 0.04 | 1 |
| GO:0043049 | otic placode formation | 2 | 0 | 0.08 | 1 |
| GO:0043060 | meiotic metaphase I plate congression | 1 | 0 | 0.04 | 1 |
| GO:0043063 | intercellular bridge organization | 1 | 0 | 0.04 | 1 |
| GO:0043084 | penile erection | 12 | 0 | 0.46 | 1 |
| GO:0043090 | amino acid import | 14 | 0 | 0.53 | 1 |
| GO:0043091 | L-arginine import | 3 | 0 | 0.11 | 1 |
| GO:0043092 | L-amino acid import | 12 | 0 | 0.46 | 1 |
| GO:0043095 | regulation of GTP cyclohydrolase I activ... | 1 | 0 | 0.04 | 1 |
| GO:0043096 | purine nucleobase salvage | 4 | 0 | 0.15 | 1 |
| GO:0043103 | hypoxanthine salvage | 3 | 0 | 0.11 | 1 |
| GO:0043105 | negative regulation of GTP cyclohydrolas... | 1 | 0 | 0.04 | 1 |
| GO:0043111 | replication fork arrest | 1 | 0 | 0.04 | 1 |
| GO:0043117 | positive regulation of vascular permeabi... | 7 | 0 | 0.27 | 1 |
| GO:0043126 | regulation of 1-phosphatidylinositol 4-k... | 1 | 0 | 0.04 | 1 |

| | | | | | |
|---|---|---|---|---|---|
| GO:0043128 | positive regulation of 1-phosphatidylino... | 1 | 0 | 0.04 | 1 |
| GO:0043129 | surfactant homeostasis | 9 | 0 | 0.34 | 1 |
| GO:0043132 | NAD transport | 2 | 0 | 0.08 | 1 |
| GO:0043133 | hindgut contraction | 2 | 0 | 0.08 | 1 |
| GO:0043134 | regulation of hindgut contraction | 2 | 0 | 0.08 | 1 |
| GO:0043137 | DNA replication | removal of | 3 | 0 | 0.11 |
| GO:0043143 | regulation of translation by machinery l... | 1 | 0 | 0.04 | 1 |
| GO:0043144 | snoRNA processing | 4 | 0 | 0.15 | 1 |
| GO:0043146 | spindle stabilization | 4 | 0 | 0.15 | 1 |
| GO:0043148 | mitotic spindle stabilization | 2 | 0 | 0.08 | 1 |
| GO:0043152 | induction of bacterial agglutination | 4 | 0 | 0.15 | 1 |
| GO:0043153 | entrainment of circadian clock by photop... | 18 | 0 | 0.69 | 1 |
| GO:0043162 | ubiquitin-dependent protein catabolic pr... | 16 | 0 | 0.61 | 1 |
| GO:0043163 | cell envelope organization | 3 | 0 | 0.11 | 1 |
| GO:0043171 | peptide catabolic process | 12 | 0 | 0.46 | 1 |
| GO:0043173 | nucleotide salvage | 12 | 0 | 0.46 | 1 |
| GO:0043181 | vacuolar sequestering | 1 | 0 | 0.04 | 1 |
| GO:0043201 | response to leucine | 6 | 0 | 0.23 | 1 |
| GO:0043206 | extracellular fibril organization | 11 | 0 | 0.42 | 1 |
| GO:0043243 | positive regulation of protein complex d... | 21 | 0 | 0.8 | 1 |
| GO:0043248 | proteasome assembly | 8 | 0 | 0.3 | 1 |
| GO:0043249 | erythrocyte maturation | 11 | 0 | 0.42 | 1 |
| GO:0043251 | sodium-dependent organic anion transport | 1 | 0 | 0.04 | 1 |
| GO:0043267 | negative regulation of potassium ion tra... | 22 | 0 | 0.84 | 1 |
| GO:0043297 | apical junction assembly | 56 | 0 | 2.13 | 1 |
| GO:0043302 | positive regulation of leukocyte degranu... | 17 | 0 | 0.65 | 1 |
| GO:0043306 | positive regulation of mast cell degranu... | 13 | 0 | 0.49 | 1 |
| GO:0043307 | eosinophil activation | 7 | 0 | 0.27 | 1 |
| GO:0043308 | eosinophil degranulation | 6 | 0 | 0.23 | 1 |
| GO:0043309 | regulation of eosinophil degranulation | 3 | 0 | 0.11 | 1 |
| GO:0043310 | negative regulation of eosinophil degran... | 1 | 0 | 0.04 | 1 |
| GO:0043311 | positive regulation of eosinophil degran... | 2 | 0 | 0.08 | 1 |
| GO:0043312 | neutrophil degranulation | 9 | 0 | 0.34 | 1 |
| GO:0043313 | regulation of neutrophil degranulation | 3 | 0 | 0.11 | 1 |
| GO:0043314 | negative regulation of neutrophil degran... | 2 | 0 | 0.08 | 1 |
| GO:0043316 | cytotoxic T cell degranulation | 2 | 0 | 0.08 | 1 |
| GO:0043320 | natural killer cell degranulation | 6 | 0 | 0.23 | 1 |
| GO:0043321 | regulation of natural killer cell degran... | 2 | 0 | 0.08 | 1 |
| GO:0043323 | positive regulation of natural killer ce... | 2 | 0 | 0.08 | 1 |
| GO:0043324 | pigment metabolic process involved in de.. | 4 | 0 | 0.15 | 1 |
| GO:0043328 | protein targeting to vacuole involved in... | 3 | 0 | 0.11 | 1 |
| GO:0043330 | response to exogenous dsRNA | 39 | 0 | 1.48 | 1 |
| GO:0043335 | protein unfolding | 1 | 0 | 0.04 | 1 |
| GO:0043353 | enucleate erythrocyte differentiation | 8 | 0 | 0.3 | 1 |
| GO:0043366 | beta selection | 2 | 0 | 0.08 | 1 |
| GO:0043367 | CD4-positive | alpha-beta | 48 | 0 | 1.83 |
| GO:0043369 | CD4-positive or CD8-positive | alpha-beta. | 13 | 0 | 0.49 |
| GO:0043370 | regulation of CD4-positive | alpha-beta | 27 | 0 | 1.03 |
| GO:0043371 | negative regulation of CD4-positive | alp... | 9 | 0 | 0.34 |
| GO:0043372 | positive regulation of CD4-positive | alp... | 20 | 0 | 0.76 |
| GO:0043373 | CD4-positive | alpha-beta | 11 | 0 | 0.42 |
| GO:0043374 | CD8-positive | alpha-beta | 8 | 0 | 0.3 |
| GO:0043375 | CD8-positive | alpha-beta | 1 | 0 | 0.04 |

| | | | | | |
|---|---|---|---|---|---|
| GO:0043376 | regulation of CD8-positive | alpha-beta | 1 | 0 | 0.04 |
| GO:0043378 | positive regulation of CD8-positive | alp... | 1 | 0 | 0.04 |
| GO:0043385 | mycotoxin metabolic process | 1 | 0 | 0.04 | 1 |
| GO:0043388 | positive regulation of DNA binding | 35 | 0 | 1.33 | 1 |
| GO:0043390 | aflatoxin B1 metabolic process | 1 | 0 | 0.04 | 1 |
| GO:0043396 | corticotropin-releasing hormone secretio... | 2 | 0 | 0.08 | 1 |
| GO:0043397 | regulation of corticotropin-releasing ho... | 2 | 0 | 0.08 | 1 |
| GO:0043400 | cortisol secretion | 5 | 0 | 0.19 | 1 |
| GO:0043402 | glucocorticoid mediated signaling pathwa... | 2 | 0 | 0.08 | 1 |
| GO:0043415 | positive regulation of skeletal muscle t... | 5 | 0 | 0.19 | 1 |
| GO:0043416 | regulation of skeletal muscle tissue reg... | 6 | 0 | 0.23 | 1 |
| GO:0043418 | homocysteine catabolic process | 2 | 0 | 0.08 | 1 |
| GO:0043420 | anthranilate metabolic process | 3 | 0 | 0.11 | 1 |
| GO:0043435 | response to corticotropin-releasing horm... | 2 | 0 | 0.08 | 1 |
| GO:0043438 | acetoacetic acid metabolic process | 1 | 0 | 0.04 | 1 |
| GO:0043449 | cellular alkene metabolic process | 1 | 0 | 0.04 | 1 |
| GO:0043455 | regulation of secondary metabolic proces... | 6 | 0 | 0.23 | 1 |
| GO:0043456 | regulation of pentose-phosphate shunt | 1 | 0 | 0.04 | 1 |
| GO:0043461 | proton-transporting ATP synthase complex | 3 | 0 | 0.11 | 1 |
| GO:0043462 | regulation of ATPase activity | 43 | 0 | 1.64 | 1 |
| GO:0043465 | regulation of fermentation | 1 | 0 | 0.04 | 1 |
| GO:0043474 | pigment metabolic process involved in pi... | 4 | 0 | 0.15 | 1 |
| GO:0043476 | pigment accumulation | 9 | 0 | 0.34 | 1 |
| GO:0043482 | cellular pigment accumulation | 9 | 0 | 0.34 | 1 |
| GO:0043485 | endosome to pigment granule transport | 7 | 0 | 0.27 | 1 |
| GO:0043490 | malate-aspartate shuttle | 2 | 0 | 0.08 | 1 |
| GO:0043496 | regulation of protein homodimerization a... | 18 | 0 | 0.69 | 1 |
| GO:0043503 | skeletal muscle fiber adaptation | 2 | 0 | 0.08 | 1 |
| GO:0043504 | mitochondrial DNA repair | 3 | 0 | 0.11 | 1 |
| GO:0043508 | negative regulation of JUN kinase activi... | 14 | 0 | 0.53 | 1 |
| GO:0043519 | regulation of myosin II filament organiz... | 2 | 0 | 0.08 | 1 |
| GO:0043537 | negative regulation of blood vessel endo... | 21 | 0 | 0.8 | 1 |
| GO:0043538 | regulation of actin phosphorylation | 1 | 0 | 0.04 | 1 |
| GO:0043545 | molybdopterin cofactor metabolic process | 7 | 0 | 0.27 | 1 |
| GO:0043553 | negative regulation of phosphatidylinosi... | 2 | 0 | 0.08 | 1 |
| GO:0043556 | regulation of translation in response to... | 1 | 0 | 0.04 | 1 |
| GO:0043568 | positive regulation of insulin-like grow... | 13 | 0 | 0.49 | 1 |
| GO:0043570 | maintenance of DNA repeat elements | 4 | 0 | 0.15 | 1 |
| GO:0043574 | peroxisomal transport | 17 | 0 | 0.65 | 1 |
| GO:0043576 | regulation of respiratory gaseous exchan... | 20 | 0 | 0.76 | 1 |
| GO:0043578 | nuclear matrix organization | 1 | 0 | 0.04 | 1 |
| GO:0043585 | nose morphogenesis | 3 | 0 | 0.11 | 1 |
| GO:0043586 | tongue development | 21 | 0 | 0.8 | 1 |
| GO:0043587 | tongue morphogenesis | 9 | 0 | 0.34 | 1 |
| GO:0043589 | skin morphogenesis | 9 | 0 | 0.34 | 1 |
| GO:0043602 | nitrate catabolic process | 1 | 0 | 0.04 | 1 |
| GO:0043605 | cellular amide catabolic process | 2 | 0 | 0.08 | 1 |
| GO:0043606 | formamide metabolic process | 4 | 0 | 0.15 | 1 |
| GO:0043622 | cortical microtubule organization | 1 | 0 | 0.04 | 1 |
| GO:0043628 | ncRNA 3'-end processing | 13 | 0 | 0.49 | 1 |
| GO:0043634 | polyadenylation-dependent ncRNA catabo | 4 | 0 | 0.15 | 1 |
| GO:0043651 | linoleic acid metabolic process | 12 | 0 | 0.46 | 1 |
| GO:0043652 | engulfment of apoptotic cell | 5 | 0 | 0.19 | 1 |

| | | | | | |
|---|---|---|---|---|---|
| GO:0043654 | recognition of apoptotic cell | 7 | 0 | 0.27 | 1 |
| GO:0043686 | co-translational protein modification | 3 | 0 | 0.11 | 1 |
| GO:0043691 | reverse cholesterol transport | 17 | 0 | 0.65 | 1 |
| GO:0043932 | ossification involved in bone remodeling | 3 | 0 | 0.11 | 1 |
| GO:0043968 | histone H2A acetylation | 11 | 0 | 0.42 | 1 |
| GO:0043969 | histone H2B acetylation | 1 | 0 | 0.04 | 1 |
| GO:0043973 | histone H3-K4 acetylation | 1 | 0 | 0.04 | 1 |
| GO:0043974 | histone H3-K27 acetylation | 2 | 0 | 0.08 | 1 |
| GO:0043981 | histone H4-K5 acetylation | 6 | 0 | 0.23 | 1 |
| GO:0043982 | histone H4-K8 acetylation | 6 | 0 | 0.23 | 1 |
| GO:0043983 | histone H4-K12 acetylation | 3 | 0 | 0.11 | 1 |
| GO:0043985 | histone H4-R3 methylation | 5 | 0 | 0.19 | 1 |
| GO:0043987 | histone H3-S10 phosphorylation | 4 | 0 | 0.15 | 1 |
| GO:0043988 | histone H3-S28 phosphorylation | 3 | 0 | 0.11 | 1 |
| GO:0043990 | histone H2A-S1 phosphorylation | 1 | 0 | 0.04 | 1 |
| GO:0044004 | disruption by symbiont of host cell | 4 | 0 | 0.15 | 1 |
| GO:0044007 | dissemination or transmission of symbion.. | 1 | 0 | 0.04 | 1 |
| GO:0044010 | single-species biofilm formation | 1 | 0 | 0.04 | 1 |
| GO:0044026 | DNA hypermethylation | 4 | 0 | 0.15 | 1 |
| GO:0044027 | hypermethylation of CpG island | 4 | 0 | 0.15 | 1 |
| GO:0044028 | DNA hypomethylation | 1 | 0 | 0.04 | 1 |
| GO:0044029 | hypomethylation of CpG island | 1 | 0 | 0.04 | 1 |
| GO:0044030 | regulation of DNA methylation | 13 | 0 | 0.49 | 1 |
| GO:0044034 | multi-organism biosynthetic process | 4 | 0 | 0.15 | 1 |
| GO:0044036 | cell wall macromolecule metabolic proces. | 5 | 0 | 0.19 | 1 |
| GO:0044053 | translocation of peptides or proteins in... | 1 | 0 | 0.04 | 1 |
| GO:0044065 | regulation of respiratory system process | 15 | 0 | 0.57 | 1 |
| GO:0044107 | cellular alcohol metabolic process | 4 | 0 | 0.15 | 1 |
| GO:0044108 | cellular alcohol biosynthetic process | 4 | 0 | 0.15 | 1 |
| GO:0044110 | growth involved in symbiotic interaction | 20 | 0 | 0.76 | 1 |
| GO:0044111 | development involved in symbiotic intera... | 1 | 0 | 0.04 | 1 |
| GO:0044116 | growth of symbiont involved in interacti... | 20 | 0 | 0.76 | 1 |
| GO:0044117 | growth of symbiont in host | 20 | 0 | 0.76 | 1 |
| GO:0044126 | regulation of growth of symbiont in host | 16 | 0 | 0.61 | 1 |
| GO:0044130 | negative regulation of growth of symbion... | 16 | 0 | 0.61 | 1 |
| GO:0044133 | growth of symbiont on or near host | 1 | 0 | 0.04 | 1 |
| GO:0044139 | modulation of growth of symbiont on or n... | 1 | 0 | 0.04 | 1 |
| GO:0044140 | negative regulation of growth of symbion... | 1 | 0 | 0.04 | 1 |
| GO:0044144 | modulation of growth of symbiont involve... | 16 | 0 | 0.61 | 1 |
| GO:0044146 | negative regulation of growth of symbion... | 16 | 0 | 0.61 | 1 |
| GO:0044154 | histone H3-K14 acetylation | 5 | 0 | 0.19 | 1 |
| GO:0044179 | hemolysis in other organism | 2 | 0 | 0.08 | 1 |
| GO:0044205 | 'de novo' UMP biosynthetic process | 3 | 0 | 0.11 | 1 |
| GO:0044206 | UMP salvage | 5 | 0 | 0.19 | 1 |
| GO:0044208 | 'de novo' AMP biosynthetic process | 3 | 0 | 0.11 | 1 |
| GO:0044209 | AMP salvage | 2 | 0 | 0.08 | 1 |
| GO:0044210 | 'de novo' CTP biosynthetic process | 1 | 0 | 0.04 | 1 |
| GO:0044211 | CTP salvage | 3 | 0 | 0.11 | 1 |
| GO:0044240 | multicellular organismal lipid catabolic... | 3 | 0 | 0.11 | 1 |
| GO:0044245 | polysaccharide digestion | 5 | 0 | 0.19 | 1 |
| GO:0044252 | negative regulation of multicellular org... | 7 | 0 | 0.27 | 1 |
| GO:0044254 | multicellular organismal protein catabol... | 4 | 0 | 0.15 | 1 |
| GO:0044256 | protein digestion | 4 | 0 | 0.15 | 1 |

| | | | | | |
|---|---|---|---|---|---|
| GO:0044258 | intestinal lipid catabolic process | 1 | 0 | 0.04 | 1 |
| GO:0044266 | multicellular organismal macromolecule c.. | 4 | 0 | 0.15 | 1 |
| GO:0044268 | multicellular organismal protein metabol... | 5 | 0 | 0.19 | 1 |
| GO:0044273 | sulfur compound catabolic process | 44 | 0 | 1.67 | 1 |
| GO:0044313 | protein K6-linked deubiquitination | 1 | 0 | 0.04 | 1 |
| GO:0044314 | protein K27-linked ubiquitination | 5 | 0 | 0.19 | 1 |
| GO:0044324 | regulation of transcription involved in ... | 2 | 0 | 0.08 | 1 |
| GO:0044328 | canonical Wnt signaling pathway involved. | 1 | 0 | 0.04 | 1 |
| GO:0044329 | canonical Wnt signaling pathway involved. | 1 | 0 | 0.04 | 1 |
| GO:0044330 | canonical Wnt signaling pathway involved. | 1 | 0 | 0.04 | 1 |
| GO:0044333 | Wnt signaling pathway involved in digest... | 1 | 0 | 0.04 | 1 |
| GO:0044334 | canonical Wnt signaling pathway involved. | 2 | 0 | 0.08 | 1 |
| GO:0044336 | canonical Wnt signaling pathway involved. | 5 | 0 | 0.19 | 1 |
| GO:0044337 | canonical Wnt signaling pathway involved. | 2 | 0 | 0.08 | 1 |
| GO:0044338 | canonical Wnt signaling pathway involved. | 2 | 0 | 0.08 | 1 |
| GO:0044339 | canonical Wnt signaling pathway involved. | 2 | 0 | 0.08 | 1 |
| GO:0044342 | type B pancreatic cell proliferation | 10 | 0 | 0.38 | 1 |
| GO:0044343 | canonical Wnt signaling pathway involved. | 2 | 0 | 0.08 | 1 |
| GO:0044345 | stromal-epithelial cell signaling involv... | 1 | 0 | 0.04 | 1 |
| GO:0044349 | DNA excision | 21 | 0 | 0.8 | 1 |
| GO:0044351 | macropinocytosis | 5 | 0 | 0.19 | 1 |
| GO:0044355 | clearance of foreign intracellular DNA | 1 | 0 | 0.04 | 1 |
| GO:0044356 | clearance of foreign intracellular DNA b... | 1 | 0 | 0.04 | 1 |
| GO:0044359 | modulation of molecular function in othe... | 2 | 0 | 0.08 | 1 |
| GO:0044362 | negative regulation of molecular functio... | 2 | 0 | 0.08 | 1 |
| GO:0044364 | disruption of cells of other organism | 29 | 0 | 1.1 | 1 |
| GO:0044375 | regulation of peroxisome size | 3 | 0 | 0.11 | 1 |
| GO:0044387 | negative regulation of protein kinase ac... | 6 | 0 | 0.23 | 1 |
| GO:0044395 | protein targeting to vacuolar membrane | 1 | 0 | 0.04 | 1 |
| GO:0044406 | adhesion of symbiont to host | 12 | 0 | 0.46 | 1 |
| GO:0044407 | single-species biofilm formation in or o... | 1 | 0 | 0.04 | 1 |
| GO:0044413 | avoidance of host defenses | 3 | 0 | 0.11 | 1 |
| GO:0044415 | evasion or tolerance of host defenses | 3 | 0 | 0.11 | 1 |
| GO:0044416 | induction by symbiont of host defense re... | 2 | 0 | 0.08 | 1 |
| GO:0044417 | translocation of molecules into host | 1 | 0 | 0.04 | 1 |
| GO:0044458 | motile cilium assembly | 5 | 0 | 0.19 | 1 |
| GO:0044467 | glial cell line-derived neurotrophic fac... | 2 | 0 | 0.08 | 1 |
| GO:0044501 | modulation of signal transduction in oth... | 1 | 0 | 0.04 | 1 |
| GO:0044524 | protein sulfhydration | 1 | 0 | 0.04 | 1 |
| GO:0044533 | positive regulation of apoptotic process... | 2 | 0 | 0.08 | 1 |
| GO:0044539 | long-chain fatty acid import | 6 | 0 | 0.23 | 1 |
| GO:0044546 | NLRP3 inflammasome complex assembly | 3 | 0 | 0.11 | 1 |
| GO:0044565 | dendritic cell proliferation | 2 | 0 | 0.08 | 1 |
| GO:0044597 | daunorubicin metabolic process | 8 | 0 | 0.3 | 1 |
| GO:0044598 | doxorubicin metabolic process | 8 | 0 | 0.3 | 1 |
| GO:0044650 | adhesion of symbiont to host cell | 9 | 0 | 0.34 | 1 |
| GO:0044691 | tooth eruption | 2 | 0 | 0.08 | 1 |
| GO:0044704 | single-organism reproductive behavior | 2 | 0 | 0.08 | 1 |
| GO:0044721 | protein import into peroxisome matrix s... | | 1 | 0 | 0.04 |
| GO:0044722 | renal phosphate excretion | 1 | 0 | 0.04 | 1 |
| GO:0044725 | chromatin reprogramming in the zygote | 1 | 0 | 0.04 | 1 |
| GO:0044726 | protection of DNA demethylation of femal.. | 1 | 0 | 0.04 | 1 |
| GO:0044728 | DNA methylation or demethylation | 90 | 0 | 3.43 | 1 |

| | | | | | |
|---|---|---|---|---|---|
| GO:0044752 | response to human chorionic gonadotropir | 1 | 0 | 0.04 | 1 |
| GO:0044771 | meiotic cell cycle phase transition | 3 | 0 | 0.11 | 1 |
| GO:0044785 | metaphase/anaphase transition of meiotic. | 1 | 0 | 0.04 | 1 |
| GO:0044790 | negative regulation by host of viral rel... | 1 | 0 | 0.04 | 1 |
| GO:0044795 | trans-Golgi network to recycling endosom. | 1 | 0 | 0.04 | 1 |
| GO:0044800 | multi-organism membrane fusion | 3 | 0 | 0.11 | 1 |
| GO:0044805 | late nucleophagy | 2 | 0 | 0.08 | 1 |
| GO:0044837 | actomyosin contractile ring organization | 1 | 0 | 0.04 | 1 |
| GO:0044848 | biological phase | 8 | 0 | 0.3 | 1 |
| GO:0044851 | hair cycle phase | 6 | 0 | 0.23 | 1 |
| GO:0044855 | plasma membrane raft distribution | 1 | 0 | 0.04 | 1 |
| GO:0044856 | plasma membrane raft localization | 1 | 0 | 0.04 | 1 |
| GO:0044858 | plasma membrane raft polarization | 1 | 0 | 0.04 | 1 |
| GO:0044860 | protein localization to plasma membrane .. | 4 | 0 | 0.15 | 1 |
| GO:0044861 | protein transport into plasma membrane r.. | 3 | 0 | 0.11 | 1 |
| GO:0044866 | modulation by host of viral exo-alpha-si... | 2 | 0 | 0.08 | 1 |
| GO:0044867 | modulation by host of viral catalytic ac... | 2 | 0 | 0.08 | 1 |
| GO:0044868 | modulation by host of viral molecular fu... | 2 | 0 | 0.08 | 1 |
| GO:0044869 | negative regulation by host of viral exo... | 2 | 0 | 0.08 | 1 |
| GO:0044870 | modulation by host of viral glycoprotein... | 2 | 0 | 0.08 | 1 |
| GO:0044871 | negative regulation by host of viral gly... | 2 | 0 | 0.08 | 1 |
| GO:0045002 | double-strand break repair via single-st... | 2 | 0 | 0.08 | 1 |
| GO:0045003 | double-strand break repair via synthesis... | 5 | 0 | 0.19 | 1 |
| GO:0045004 | DNA replication proofreading | 1 | 0 | 0.04 | 1 |
| GO:0045006 | DNA deamination | 9 | 0 | 0.34 | 1 |
| GO:0045007 | depurination | 3 | 0 | 0.11 | 1 |
| GO:0045013 | carbon catabolite repression of transcri... | 2 | 0 | 0.08 | 1 |
| GO:0045014 | negative regulation of transcription by ... | 2 | 0 | 0.08 | 1 |
| GO:0045023 | G0 to G1 transition | 9 | 0 | 0.34 | 1 |
| GO:0045046 | protein import into peroxisome membrane | 6 | 0 | 0.23 | 1 |
| GO:0045053 | protein retention in Golgi apparatus | 5 | 0 | 0.19 | 1 |
| GO:0045054 | constitutive secretory pathway | 4 | 0 | 0.15 | 1 |
| GO:0045056 | transcytosis | 9 | 0 | 0.34 | 1 |
| GO:0045062 | extrathymic T cell selection | 1 | 0 | 0.04 | 1 |
| GO:0045063 | T-helper 1 cell differentiation | 15 | 0 | 0.57 | 1 |
| GO:0045064 | T-helper 2 cell differentiation | 13 | 0 | 0.49 | 1 |
| GO:0045065 | cytotoxic T cell differentiation | 3 | 0 | 0.11 | 1 |
| GO:0045072 | regulation of interferon-gamma biosynthe.. | 16 | 0 | 0.61 | 1 |
| GO:0045073 | regulation of chemokine biosynthetic pro... | 12 | 0 | 0.46 | 1 |
| GO:0045074 | regulation of interleukin-10 biosyntheti... | 3 | 0 | 0.11 | 1 |
| GO:0045075 | regulation of interleukin-12 biosyntheti... | 9 | 0 | 0.34 | 1 |
| GO:0045076 | regulation of interleukin-2 biosynthetic... | 19 | 0 | 0.72 | 1 |
| GO:0045077 | negative regulation of interferon-gamma ... | 5 | 0 | 0.19 | 1 |
| GO:0045078 | positive regulation of interferon-gamma ... | 12 | 0 | 0.46 | 1 |
| GO:0045079 | negative regulation of chemokine biosynt.. | 3 | 0 | 0.11 | 1 |
| GO:0045080 | positive regulation of chemokine biosynt... | 9 | 0 | 0.34 | 1 |
| GO:0045081 | negative regulation of interleukin-10 bi... | 1 | 0 | 0.04 | 1 |
| GO:0045082 | positive regulation of interleukin-10 bi... | 2 | 0 | 0.08 | 1 |
| GO:0045083 | negative regulation of interleukin-12 bi... | 1 | 0 | 0.04 | 1 |
| GO:0045084 | positive regulation of interleukin-12 bi... | 7 | 0 | 0.27 | 1 |
| GO:0045085 | negative regulation of interleukin-2 bio... | 4 | 0 | 0.15 | 1 |
| GO:0045086 | positive regulation of interleukin-2 bio... | 13 | 0 | 0.49 | 1 |
| GO:0045091 | regulation of single stranded viral RNA ... | 7 | 0 | 0.27 | 1 |

| | | | | | |
|---|---|---|---|---|---|
| GO:0045105 | intermediate filament polymerization or ... | 1 | 0 | 0.04 | 1 |
| GO:0045106 | intermediate filament depolymerization | 1 | 0 | 0.04 | 1 |
| GO:0045108 | regulation of intermediate filament poly... | 1 | 0 | 0.04 | 1 |
| GO:0045109 | intermediate filament organization | 18 | 0 | 0.69 | 1 |
| GO:0045110 | intermediate filament bundle assembly | 6 | 0 | 0.23 | 1 |
| GO:0045112 | integrin biosynthetic process | 5 | 0 | 0.19 | 1 |
| GO:0045113 | regulation of integrin biosynthetic proc... | 4 | 0 | 0.15 | 1 |
| GO:0045128 | negative regulation of reciprocal meioti... | 1 | 0 | 0.04 | 1 |
| GO:0045136 | development of secondary sexual characte | 10 | 0 | 0.38 | 1 |
| GO:0045161 | neuronal ion channel clustering | 12 | 0 | 0.46 | 1 |
| GO:0045162 | clustering of voltage-gated sodium chann... | 7 | 0 | 0.27 | 1 |
| GO:0045163 | clustering of voltage-gated potassium ch... | 3 | 0 | 0.11 | 1 |
| GO:0045168 | cell-cell signaling involved in cell fat... | 37 | 0 | 1.41 | 1 |
| GO:0045175 | basal protein localization | 2 | 0 | 0.08 | 1 |
| GO:0045176 | apical protein localization | 11 | 0 | 0.42 | 1 |
| GO:0045186 | zonula adherens assembly | 1 | 0 | 0.04 | 1 |
| GO:0045187 | regulation of circadian sleep/wake cycle... | 17 | 0 | 0.65 | 1 |
| GO:0045188 | regulation of circadian sleep/wake cycle... | 7 | 0 | 0.27 | 1 |
| GO:0045189 | connective tissue growth factor biosynth... | 1 | 0 | 0.04 | 1 |
| GO:0045190 | isotype switching | 38 | 0 | 1.45 | 1 |
| GO:0045191 | regulation of isotype switching | 22 | 0 | 0.84 | 1 |
| GO:0045196 | establishment or maintenance of neurobla | 3 | 0 | 0.11 | 1 |
| GO:0045197 | establishment or maintenance of epitheli... | 20 | 0 | 0.76 | 1 |
| GO:0045198 | establishment of epithelial cell apical/... | 4 | 0 | 0.15 | 1 |
| GO:0045199 | maintenance of epithelial cell apical/ba... | 6 | 0 | 0.23 | 1 |
| GO:0045200 | establishment of neuroblast polarity | 3 | 0 | 0.11 | 1 |
| GO:0045204 | MAPK export from nucleus | 3 | 0 | 0.11 | 1 |
| GO:0045208 | MAPK phosphatase export from nucleus | 1 | 0 | 0.04 | 1 |
| GO:0045209 | MAPK phosphatase export from nucleus le... | | 1 | 0 | 0.04 |
| GO:0045210 | FasL biosynthetic process | 2 | 0 | 0.08 | 1 |
| GO:0045212 | neurotransmitter receptor biosynthetic p... | 1 | 0 | 0.04 | 1 |
| GO:0045213 | neurotransmitter receptor metabolic proc... | 4 | 0 | 0.15 | 1 |
| GO:0045217 | cell-cell junction maintenance | 7 | 0 | 0.27 | 1 |
| GO:0045218 | zonula adherens maintenance | 2 | 0 | 0.08 | 1 |
| GO:0045219 | regulation of FasL biosynthetic process | 1 | 0 | 0.04 | 1 |
| GO:0045221 | negative regulation of FasL biosynthetic... | 1 | 0 | 0.04 | 1 |
| GO:0045226 | extracellular polysaccharide biosyntheti... | 3 | 0 | 0.11 | 1 |
| GO:0045229 | external encapsulating structure organiz... | 3 | 0 | 0.11 | 1 |
| GO:0045234 | protein palmitoleylation | 1 | 0 | 0.04 | 1 |
| GO:0045297 | post-mating behavior | 2 | 0 | 0.08 | 1 |
| GO:0045299 | otolith mineralization | 1 | 0 | 0.04 | 1 |
| GO:0045324 | late endosome to vacuole transport | 9 | 0 | 0.34 | 1 |
| GO:0045329 | carnitine biosynthetic process | 5 | 0 | 0.19 | 1 |
| GO:0045337 | farnesyl diphosphate biosynthetic proces... | 1 | 0 | 0.04 | 1 |
| GO:0045338 | farnesyl diphosphate metabolic process | 2 | 0 | 0.08 | 1 |
| GO:0045341 | MHC class I biosynthetic process | 21 | 0 | 0.8 | 1 |
| GO:0045342 | MHC class II biosynthetic process | 13 | 0 | 0.49 | 1 |
| GO:0045343 | regulation of MHC class I biosynthetic p... | 21 | 0 | 0.8 | 1 |
| GO:0045345 | positive regulation of MHC class I biosy... | 4 | 0 | 0.15 | 1 |
| GO:0045346 | regulation of MHC class II biosynthetic ... | 12 | 0 | 0.46 | 1 |
| GO:0045347 | negative regulation of MHC class II bios... | 5 | 0 | 0.19 | 1 |
| GO:0045348 | positive regulation of MHC class II bios... | 8 | 0 | 0.3 | 1 |
| GO:0045349 | interferon-alpha biosynthetic process | 5 | 0 | 0.19 | 1 |

| | | | | | |
|---|---|---|---|---|---|
| GO:0045350 | interferon-beta biosynthetic process | 8 | 0 | 0.3 | 1 |
| GO:0045351 | type I interferon biosynthetic process | 12 | 0 | 0.46 | 1 |
| GO:0045354 | regulation of interferon-alpha biosynthe... | 5 | 0 | 0.19 | 1 |
| GO:0045355 | negative regulation of interferon-alpha ... | 1 | 0 | 0.04 | 1 |
| GO:0045356 | positive regulation of interferon-alpha ... | 4 | 0 | 0.15 | 1 |
| GO:0045357 | regulation of interferon-beta biosynthet... | 8 | 0 | 0.3 | 1 |
| GO:0045358 | negative regulation of interferon-beta b... | 1 | 0 | 0.04 | 1 |
| GO:0045359 | positive regulation of interferon-beta b... | 7 | 0 | 0.27 | 1 |
| GO:0045360 | regulation of interleukin-1 biosynthetic... | 2 | 0 | 0.08 | 1 |
| GO:0045362 | positive regulation of interleukin-1 bio... | 2 | 0 | 0.08 | 1 |
| GO:0045366 | regulation of interleukin-13 biosyntheti... | 1 | 0 | 0.04 | 1 |
| GO:0045368 | positive regulation of interleukin-13 bi... | 1 | 0 | 0.04 | 1 |
| GO:0045381 | regulation of interleukin-18 biosyntheti... | 1 | 0 | 0.04 | 1 |
| GO:0045399 | regulation of interleukin-3 biosynthetic... | 2 | 0 | 0.08 | 1 |
| GO:0045401 | positive regulation of interleukin-3 bio... | 2 | 0 | 0.08 | 1 |
| GO:0045402 | regulation of interleukin-4 biosynthetic... | 3 | 0 | 0.11 | 1 |
| GO:0045403 | negative regulation of interleukin-4 bio... | 1 | 0 | 0.04 | 1 |
| GO:0045404 | positive regulation of interleukin-4 bio... | 2 | 0 | 0.08 | 1 |
| GO:0045405 | regulation of interleukin-5 biosynthetic... | 1 | 0 | 0.04 | 1 |
| GO:0045407 | positive regulation of interleukin-5 bio... | 1 | 0 | 0.04 | 1 |
| GO:0045408 | regulation of interleukin-6 biosynthetic... | 15 | 0 | 0.57 | 1 |
| GO:0045409 | negative regulation of interleukin-6 bio... | 5 | 0 | 0.19 | 1 |
| GO:0045410 | positive regulation of interleukin-6 bio... | 7 | 0 | 0.27 | 1 |
| GO:0045415 | negative regulation of interleukin-8 bio... | 4 | 0 | 0.15 | 1 |
| GO:0045423 | regulation of granulocyte macrophage col... | 4 | 0 | 0.15 | 1 |
| GO:0045425 | positive regulation of granulocyte macro... | 3 | 0 | 0.11 | 1 |
| GO:0045448 | mitotic cell cycle | embryonic | 5 | 0 | 0.19 |
| GO:0045542 | positive regulation of cholesterol biosy... | 5 | 0 | 0.19 | 1 |
| GO:0045553 | TRAIL biosynthetic process | 1 | 0 | 0.04 | 1 |
| GO:0045554 | regulation of TRAIL biosynthetic process | 1 | 0 | 0.04 | 1 |
| GO:0045556 | positive regulation of TRAIL biosyntheti... | 1 | 0 | 0.04 | 1 |
| GO:0045575 | basophil activation | 2 | 0 | 0.08 | 1 |
| GO:0045577 | regulation of B cell differentiation | 22 | 0 | 0.84 | 1 |
| GO:0045578 | negative regulation of B cell differenti... | 6 | 0 | 0.23 | 1 |
| GO:0045579 | positive regulation of B cell differenti... | 12 | 0 | 0.46 | 1 |
| GO:0045583 | regulation of cytotoxic T cell different... | 2 | 0 | 0.08 | 1 |
| GO:0045585 | positive regulation of cytotoxic T cell ... | 2 | 0 | 0.08 | 1 |
| GO:0045586 | regulation of gamma-delta T cell differe... | 8 | 0 | 0.3 | 1 |
| GO:0045588 | positive regulation of gamma-delta T cel... | 5 | 0 | 0.19 | 1 |
| GO:0045590 | negative regulation of regulatory T cell... | 3 | 0 | 0.11 | 1 |
| GO:0045602 | negative regulation of endothelial cell ... | 4 | 0 | 0.15 | 1 |
| GO:0045606 | positive regulation of epidermal cell di... | 16 | 0 | 0.61 | 1 |
| GO:0045607 | regulation of auditory receptor cell dif... | 4 | 0 | 0.15 | 1 |
| GO:0045608 | negative regulation of auditory receptor... | 2 | 0 | 0.08 | 1 |
| GO:0045609 | positive regulation of auditory receptor... | 1 | 0 | 0.04 | 1 |
| GO:0045618 | positive regulation of keratinocyte diff... | 12 | 0 | 0.46 | 1 |
| GO:0045622 | regulation of T-helper cell differentiat... | 23 | 0 | 0.88 | 1 |
| GO:0045623 | negative regulation of T-helper cell dif... | 9 | 0 | 0.34 | 1 |
| GO:0045624 | positive regulation of T-helper cell dif... | 16 | 0 | 0.61 | 1 |
| GO:0045625 | regulation of T-helper 1 cell differenti... | 9 | 0 | 0.34 | 1 |
| GO:0045626 | negative regulation of T-helper 1 cell d... | 3 | 0 | 0.11 | 1 |
| GO:0045627 | positive regulation of T-helper 1 cell d... | 5 | 0 | 0.19 | 1 |
| GO:0045628 | regulation of T-helper 2 cell differenti... | 9 | 0 | 0.34 | 1 |

| | | | | | |
|---|---|---|---|---|---|
| GO:0045629 | negative regulation of T-helper 2 cell d... | 3 | 0 | 0.11 | 1 |
| GO:0045630 | positive regulation of T-helper 2 cell d... | 6 | 0 | 0.23 | 1 |
| GO:0045631 | regulation of mechanoreceptor differenti... | 5 | 0 | 0.19 | 1 |
| GO:0045632 | negative regulation of mechanoreceptor d. | 3 | 0 | 0.11 | 1 |
| GO:0045633 | positive regulation of mechanoreceptor d... | 1 | 0 | 0.04 | 1 |
| GO:0045634 | regulation of melanocyte differentiation | 7 | 0 | 0.27 | 1 |
| GO:0045636 | positive regulation of melanocyte differ... | 5 | 0 | 0.19 | 1 |
| GO:0045643 | regulation of eosinophil differentiation | 1 | 0 | 0.04 | 1 |
| GO:0045645 | positive regulation of eosinophil differ... | 1 | 0 | 0.04 | 1 |
| GO:0045647 | negative regulation of erythrocyte diffe... | 8 | 0 | 0.3 | 1 |
| GO:0045651 | positive regulation of macrophage differ... | 12 | 0 | 0.46 | 1 |
| GO:0045653 | negative regulation of megakaryocyte dif... | 17 | 0 | 0.65 | 1 |
| GO:0045655 | regulation of monocyte differentiation | 12 | 0 | 0.46 | 1 |
| GO:0045656 | negative regulation of monocyte differen... | 5 | 0 | 0.19 | 1 |
| GO:0045657 | positive regulation of monocyte differen... | 5 | 0 | 0.19 | 1 |
| GO:0045658 | regulation of neutrophil differentiation | 1 | 0 | 0.04 | 1 |
| GO:0045660 | positive regulation of neutrophil differ... | 1 | 0 | 0.04 | 1 |
| GO:0045671 | negative regulation of osteoclast differ... | 21 | 0 | 0.8 | 1 |
| GO:0045684 | positive regulation of epidermis develop... | 27 | 0 | 1.03 | 1 |
| GO:0045687 | positive regulation of glial cell differ... | 27 | 0 | 1.03 | 1 |
| GO:0045715 | negative regulation of low-density lipop... | 3 | 0 | 0.11 | 1 |
| GO:0045717 | negative regulation of fatty acid biosyn... | 10 | 0 | 0.38 | 1 |
| GO:0045719 | negative regulation of glycogen biosynth... | 7 | 0 | 0.27 | 1 |
| GO:0045720 | negative regulation of integrin biosynth... | 1 | 0 | 0.04 | 1 |
| GO:0045721 | negative regulation of gluconeogenesis | 10 | 0 | 0.38 | 1 |
| GO:0045723 | positive regulation of fatty acid biosyn... | 14 | 0 | 0.53 | 1 |
| GO:0045724 | positive regulation of cilium assembly | 3 | 0 | 0.11 | 1 |
| GO:0045726 | positive regulation of integrin biosynth... | 3 | 0 | 0.11 | 1 |
| GO:0045727 | positive regulation of translation | 64 | 0 | 2.44 | 1 |
| GO:0045728 | respiratory burst after phagocytosis | 1 | 0 | 0.04 | 1 |
| GO:0045738 | negative regulation of DNA repair | 7 | 0 | 0.27 | 1 |
| GO:0045741 | positive regulation of epidermal growth ... | 10 | 0 | 0.38 | 1 |
| GO:0045743 | positive regulation of fibroblast growth... | 7 | 0 | 0.27 | 1 |
| GO:0045747 | positive regulation of Notch signaling p... | 24 | 0 | 0.91 | 1 |
| GO:0045751 | negative regulation of Toll signaling pa... | 2 | 0 | 0.08 | 1 |
| GO:0045759 | negative regulation of action potential | 4 | 0 | 0.15 | 1 |
| GO:0045760 | positive regulation of action potential | 11 | 0 | 0.42 | 1 |
| GO:0045763 | negative regulation of cellular amino ac... | 2 | 0 | 0.08 | 1 |
| GO:0045764 | positive regulation of cellular amino ac... | 6 | 0 | 0.23 | 1 |
| GO:0045769 | negative regulation of asymmetric cell d... | 1 | 0 | 0.04 | 1 |
| GO:0045779 | negative regulation of bone resorption | 8 | 0 | 0.3 | 1 |
| GO:0045794 | negative regulation of cell volume | 3 | 0 | 0.11 | 1 |
| GO:0045795 | positive regulation of cell volume | 1 | 0 | 0.04 | 1 |
| GO:0045796 | negative regulation of intestinal choles... | 2 | 0 | 0.08 | 1 |
| GO:0045799 | positive regulation of chromatin assembl... | 2 | 0 | 0.08 | 1 |
| GO:0045815 | positive regulation of gene expression ... | | 17 | 0 | 0.65 |
| GO:0045818 | negative regulation of glycogen cataboli... | 2 | 0 | 0.08 | 1 |
| GO:0045819 | positive regulation of glycogen cataboli... | 2 | 0 | 0.08 | 1 |
| GO:0045827 | negative regulation of isoprenoid metabo... | 1 | 0 | 0.04 | 1 |
| GO:0045829 | negative regulation of isotype switching | 4 | 0 | 0.15 | 1 |
| GO:0045830 | positive regulation of isotype switching | 14 | 0 | 0.53 | 1 |
| GO:0045835 | negative regulation of meiotic nuclear d... | 11 | 0 | 0.42 | 1 |
| GO:0045852 | pH elevation | 5 | 0 | 0.19 | 1 |

| | | | | | |
|---|---|---|---|---|---|
| GO:0045869 | negative regulation of single stranded v... | 6 | 0 | 0.23 | 1 |
| GO:0045870 | positive regulation of single stranded v... | 1 | 0 | 0.04 | 1 |
| GO:0045872 | positive regulation of rhodopsin gene ex... | 2 | 0 | 0.08 | 1 |
| GO:0045875 | negative regulation of sister chromatid ... | 2 | 0 | 0.08 | 1 |
| GO:0045887 | positive regulation of synaptic growth a... | 1 | 0 | 0.04 | 1 |
| GO:0045896 | regulation of transcription during mitos... | 4 | 0 | 0.15 | 1 |
| GO:0045897 | positive regulation of transcription dur... | 2 | 0 | 0.08 | 1 |
| GO:0045900 | negative regulation of translational elo... | 3 | 0 | 0.11 | 1 |
| GO:0045901 | positive regulation of translational elo... | 3 | 0 | 0.11 | 1 |
| GO:0045903 | positive regulation of translational fid... | 1 | 0 | 0.04 | 1 |
| GO:0045905 | positive regulation of translational ter... | 2 | 0 | 0.08 | 1 |
| GO:0045907 | positive regulation of vasoconstriction | 28 | 0 | 1.07 | 1 |
| GO:0045908 | negative regulation of vasodilation | 4 | 0 | 0.15 | 1 |
| GO:0045909 | positive regulation of vasodilation | 27 | 0 | 1.03 | 1 |
| GO:0045914 | negative regulation of catecholamine met.. | 2 | 0 | 0.08 | 1 |
| GO:0045915 | positive regulation of catecholamine met... | 4 | 0 | 0.15 | 1 |
| GO:0045917 | positive regulation of complement activa... | 2 | 0 | 0.08 | 1 |
| GO:0045918 | negative regulation of cytolysis | 4 | 0 | 0.15 | 1 |
| GO:0045919 | positive regulation of cytolysis | 4 | 0 | 0.15 | 1 |
| GO:0045922 | negative regulation of fatty acid metabo... | 24 | 0 | 0.91 | 1 |
| GO:0045938 | positive regulation of circadian sleep/w... | 6 | 0 | 0.23 | 1 |
| GO:0045945 | positive regulation of transcription fro... | 8 | 0 | 0.3 | 1 |
| GO:0045947 | negative regulation of translational ini... | 17 | 0 | 0.65 | 1 |
| GO:0045948 | positive regulation of translational ini... | 16 | 0 | 0.61 | 1 |
| GO:0045950 | negative regulation of mitotic recombina... | 2 | 0 | 0.08 | 1 |
| GO:0045955 | negative regulation of calcium ion-depen... | 7 | 0 | 0.27 | 1 |
| GO:0045956 | positive regulation of calcium ion-depen... | 16 | 0 | 0.61 | 1 |
| GO:0045957 | negative regulation of complement activa.. | 2 | 0 | 0.08 | 1 |
| GO:0045959 | negative regulation of complement activa.. | 4 | 0 | 0.15 | 1 |
| GO:0045963 | negative regulation of dopamine metaboli.. | 2 | 0 | 0.08 | 1 |
| GO:0045964 | positive regulation of dopamine metaboli... | 4 | 0 | 0.15 | 1 |
| GO:0045974 | regulation of translation | ncRNA-me | 5 | 0 | 0.19 |
| GO:0045976 | negative regulation of mitotic cell cycl... | 2 | 0 | 0.08 | 1 |
| GO:0045977 | positive regulation of mitotic cell cycl... | 2 | 0 | 0.08 | 1 |
| GO:0045978 | negative regulation of nucleoside metabo.. | 6 | 0 | 0.23 | 1 |
| GO:0045979 | positive regulation of nucleoside metabo... | 4 | 0 | 0.15 | 1 |
| GO:0045988 | negative regulation of striated muscle c... | 6 | 0 | 0.23 | 1 |
| GO:0045989 | positive regulation of striated muscle c... | 10 | 0 | 0.38 | 1 |
| GO:0045993 | negative regulation of translational ini... | 1 | 0 | 0.04 | 1 |
| GO:0045994 | positive regulation of translational ini... | 2 | 0 | 0.08 | 1 |
| GO:0046005 | positive regulation of circadian sleep/w... | 3 | 0 | 0.11 | 1 |
| GO:0046006 | regulation of activated T cell prolifera... | 33 | 0 | 1.26 | 1 |
| GO:0046007 | negative regulation of activated T cell ... | 8 | 0 | 0.3 | 1 |
| GO:0046010 | positive regulation of circadian sleep/w... | 3 | 0 | 0.11 | 1 |
| GO:0046013 | regulation of T cell homeostatic prolife... | 2 | 0 | 0.08 | 1 |
| GO:0046015 | regulation of transcription by glucose | 6 | 0 | 0.23 | 1 |
| GO:0046016 | positive regulation of transcription by ... | 4 | 0 | 0.15 | 1 |
| GO:0046021 | regulation of transcription from RNA pol... | 4 | 0 | 0.15 | 1 |
| GO:0046022 | positive regulation of transcription fro... | 2 | 0 | 0.08 | 1 |
| GO:0046031 | ADP metabolic process | 7 | 0 | 0.27 | 1 |
| GO:0046032 | ADP catabolic process | 1 | 0 | 0.04 | 1 |
| GO:0046035 | CMP metabolic process | 1 | 0 | 0.04 | 1 |
| GO:0046036 | CTP metabolic process | 12 | 0 | 0.46 | 1 |

| | | | | | |
|---|---|---|---|---|---|
| GO:0046037 | GMP metabolic process | 8 | 0 | 0.3 | 1 |
| GO:0046038 | GMP catabolic process | 1 | 0 | 0.04 | 1 |
| GO:0046039 | GTP metabolic process | 9 | 0 | 0.34 | 1 |
| GO:0046040 | IMP metabolic process | 13 | 0 | 0.49 | 1 |
| GO:0046041 | ITP metabolic process | 2 | 0 | 0.08 | 1 |
| GO:0046048 | UDP metabolic process | 2 | 0 | 0.08 | 1 |
| GO:0046049 | UMP metabolic process | 11 | 0 | 0.42 | 1 |
| GO:0046051 | UTP metabolic process | 10 | 0 | 0.38 | 1 |
| GO:0046054 | dGMP metabolic process | 1 | 0 | 0.04 | 1 |
| GO:0046056 | dADP metabolic process | 2 | 0 | 0.08 | 1 |
| GO:0046057 | dADP catabolic process | 1 | 0 | 0.04 | 1 |
| GO:0046060 | dATP metabolic process | 7 | 0 | 0.27 | 1 |
| GO:0046061 | dATP catabolic process | 3 | 0 | 0.11 | 1 |
| GO:0046062 | dCDP metabolic process | 1 | 0 | 0.04 | 1 |
| GO:0046066 | dGDP metabolic process | 2 | 0 | 0.08 | 1 |
| GO:0046067 | dGDP catabolic process | 1 | 0 | 0.04 | 1 |
| GO:0046068 | cGMP metabolic process | 43 | 0 | 1.64 | 1 |
| GO:0046069 | cGMP catabolic process | 6 | 0 | 0.23 | 1 |
| GO:0046070 | dGTP metabolic process | 4 | 0 | 0.15 | 1 |
| GO:0046073 | dTMP metabolic process | 1 | 0 | 0.04 | 1 |
| GO:0046078 | dUMP metabolic process | 3 | 0 | 0.11 | 1 |
| GO:0046079 | dUMP catabolic process | 1 | 0 | 0.04 | 1 |
| GO:0046080 | dUTP metabolic process | 1 | 0 | 0.04 | 1 |
| GO:0046081 | dUTP catabolic process | 1 | 0 | 0.04 | 1 |
| GO:0046083 | adenine metabolic process | 2 | 0 | 0.08 | 1 |
| GO:0046084 | adenine biosynthetic process | 2 | 0 | 0.08 | 1 |
| GO:0046085 | adenosine metabolic process | 13 | 0 | 0.49 | 1 |
| GO:0046086 | adenosine biosynthetic process | 1 | 0 | 0.04 | 1 |
| GO:0046087 | cytidine metabolic process | 9 | 0 | 0.34 | 1 |
| GO:0046090 | deoxyadenosine metabolic process | 1 | 0 | 0.04 | 1 |
| GO:0046092 | deoxycytidine metabolic process | 1 | 0 | 0.04 | 1 |
| GO:0046098 | guanine metabolic process | 2 | 0 | 0.08 | 1 |
| GO:0046099 | guanine biosynthetic process | 1 | 0 | 0.04 | 1 |
| GO:0046100 | hypoxanthine metabolic process | 4 | 0 | 0.15 | 1 |
| GO:0046101 | hypoxanthine biosynthetic process | 4 | 0 | 0.15 | 1 |
| GO:0046102 | inosine metabolic process | 3 | 0 | 0.11 | 1 |
| GO:0046103 | inosine biosynthetic process | 3 | 0 | 0.11 | 1 |
| GO:0046104 | thymidine metabolic process | 4 | 0 | 0.15 | 1 |
| GO:0046105 | thymidine biosynthetic process | 1 | 0 | 0.04 | 1 |
| GO:0046108 | uridine metabolic process | 2 | 0 | 0.08 | 1 |
| GO:0046110 | xanthine metabolic process | 2 | 0 | 0.08 | 1 |
| GO:0046111 | xanthine biosynthetic process | 1 | 0 | 0.04 | 1 |
| GO:0046112 | nucleobase biosynthetic process | 14 | 0 | 0.53 | 1 |
| GO:0046113 | nucleobase catabolic process | 10 | 0 | 0.38 | 1 |
| GO:0046116 | queuosine metabolic process | 2 | 0 | 0.08 | 1 |
| GO:0046120 | deoxyribonucleoside biosynthetic process | 1 | 0 | 0.04 | 1 |
| GO:0046121 | deoxyribonucleoside catabolic process | 3 | 0 | 0.11 | 1 |
| GO:0046122 | purine deoxyribonucleoside metabolic pro. | 2 | 0 | 0.08 | 1 |
| GO:0046124 | purine deoxyribonucleoside catabolic pro.. | 1 | 0 | 0.04 | 1 |
| GO:0046125 | pyrimidine deoxyribonucleoside metabolic. | 4 | 0 | 0.15 | 1 |
| GO:0046126 | pyrimidine deoxyribonucleoside biosynthe. | 1 | 0 | 0.04 | 1 |
| GO:0046127 | pyrimidine deoxyribonucleoside catabolic.. | 1 | 0 | 0.04 | 1 |
| GO:0046130 | purine ribonucleoside catabolic process | 12 | 0 | 0.46 | 1 |

| | | | | | |
|---|---|---|---|---|---|
| GO:0046131 | pyrimidine ribonucleoside metabolic proc... | 31 | 0 | 1.18 | 1 |
| GO:0046132 | pyrimidine ribonucleoside biosynthetic p... | 20 | 0 | 0.76 | 1 |
| GO:0046133 | pyrimidine ribonucleoside catabolic proc... | 10 | 0 | 0.38 | 1 |
| GO:0046135 | pyrimidine nucleoside catabolic process | 21 | 0 | 0.8 | 1 |
| GO:0046136 | positive regulation of vitamin metabolic... | 3 | 0 | 0.11 | 1 |
| GO:0046137 | negative regulation of vitamin metabolic... | 6 | 0 | 0.23 | 1 |
| GO:0046144 | D-alanine family amino acid metabolic pr... | 1 | 0 | 0.04 | 1 |
| GO:0046146 | tetrahydrobiopterin metabolic process | 6 | 0 | 0.23 | 1 |
| GO:0046148 | pigment biosynthetic process | 49 | 0 | 1.87 | 1 |
| GO:0046149 | pigment catabolic process | 7 | 0 | 0.27 | 1 |
| GO:0046160 | heme a metabolic process | 2 | 0 | 0.08 | 1 |
| GO:0046166 | glyceraldehyde-3-phosphate biosynthetic ... | 1 | 0 | 0.04 | 1 |
| GO:0046167 | glycerol-3-phosphate biosynthetic proces... | 1 | 0 | 0.04 | 1 |
| GO:0046168 | glycerol-3-phosphate catabolic process | 2 | 0 | 0.08 | 1 |
| GO:0046184 | aldehyde biosynthetic process | 4 | 0 | 0.15 | 1 |
| GO:0046185 | aldehyde catabolic process | 9 | 0 | 0.34 | 1 |
| GO:0046189 | phenol-containing compound biosynthetic | 37 | 0 | 1.41 | 1 |
| GO:0046203 | spermidine catabolic process | 2 | 0 | 0.08 | 1 |
| GO:0046204 | nor-spermidine metabolic process | 1 | 0 | 0.04 | 1 |
| GO:0046208 | spermine catabolic process | 2 | 0 | 0.08 | 1 |
| GO:0046210 | nitric oxide catabolic process | 1 | 0 | 0.04 | 1 |
| GO:0046218 | indolalkylamine catabolic process | 11 | 0 | 0.42 | 1 |
| GO:0046219 | indolalkylamine biosynthetic process | 5 | 0 | 0.19 | 1 |
| GO:0046222 | aflatoxin metabolic process | 1 | 0 | 0.04 | 1 |
| GO:0046226 | coumarin catabolic process | 2 | 0 | 0.08 | 1 |
| GO:0046247 | terpene catabolic process | 1 | 0 | 0.04 | 1 |
| GO:0046271 | phenylpropanoid catabolic process | 3 | 0 | 0.11 | 1 |
| GO:0046272 | stilbene catabolic process | 1 | 0 | 0.04 | 1 |
| GO:0046292 | formaldehyde metabolic process | 2 | 0 | 0.08 | 1 |
| GO:0046294 | formaldehyde catabolic process | 2 | 0 | 0.08 | 1 |
| GO:0046295 | glycolate biosynthetic process | 1 | 0 | 0.04 | 1 |
| GO:0046296 | glycolate catabolic process | 1 | 0 | 0.04 | 1 |
| GO:0046314 | phosphocreatine biosynthetic process | 1 | 0 | 0.04 | 1 |
| GO:0046317 | regulation of glucosylceramide biosynthe... | 1 | 0 | 0.04 | 1 |
| GO:0046318 | negative regulation of glucosylceramide ... | 1 | 0 | 0.04 | 1 |
| GO:0046322 | negative regulation of fatty acid oxidat... | 7 | 0 | 0.27 | 1 |
| GO:0046325 | negative regulation of glucose import | 10 | 0 | 0.38 | 1 |
| GO:0046327 | glycerol biosynthetic process from pyruv... | 1 | 0 | 0.04 | 1 |
| GO:0046331 | lateral inhibition | 1 | 0 | 0.04 | 1 |
| GO:0046337 | phosphatidylethanolamine metabolic proce | 14 | 0 | 0.53 | 1 |
| GO:0046338 | phosphatidylethanolamine catabolic proce | 1 | 0 | 0.04 | 1 |
| GO:0046339 | diacylglycerol metabolic process | 13 | 0 | 0.49 | 1 |
| GO:0046340 | diacylglycerol catabolic process | 3 | 0 | 0.11 | 1 |
| GO:0046341 | CDP-diacylglycerol metabolic process | 13 | 0 | 0.49 | 1 |
| GO:0046348 | amino sugar catabolic process | 11 | 0 | 0.42 | 1 |
| GO:0046351 | disaccharide biosynthetic process | 2 | 0 | 0.08 | 1 |
| GO:0046352 | disaccharide catabolic process | 1 | 0 | 0.04 | 1 |
| GO:0046355 | mannan catabolic process | 1 | 0 | 0.04 | 1 |
| GO:0046356 | acetyl-CoA catabolic process | 1 | 0 | 0.04 | 1 |
| GO:0046359 | butyrate catabolic process | 1 | 0 | 0.04 | 1 |
| GO:0046360 | 2-oxobutyrate biosynthetic process | 1 | 0 | 0.04 | 1 |
| GO:0046361 | 2-oxobutyrate metabolic process | 1 | 0 | 0.04 | 1 |
| GO:0046365 | monosaccharide catabolic process | 27 | 0 | 1.03 | 1 |

| | | | | | |
|---|---|---|---|---|---|
| GO:0046368 | GDP-L-fucose metabolic process | 3 | 0 | 0.11 | 1 |
| GO:0046370 | fructose biosynthetic process | 1 | 0 | 0.04 | 1 |
| GO:0046379 | extracellular polysaccharide metabolic p... | 3 | 0 | 0.11 | 1 |
| GO:0046380 | N-acetylneuraminate biosynthetic process | 1 | 0 | 0.04 | 1 |
| GO:0046398 | UDP-glucuronate metabolic process | 4 | 0 | 0.15 | 1 |
| GO:0046415 | urate metabolic process | 11 | 0 | 0.42 | 1 |
| GO:0046416 | D-amino acid metabolic process | 4 | 0 | 0.15 | 1 |
| GO:0046436 | D-alanine metabolic process | 1 | 0 | 0.04 | 1 |
| GO:0046437 | D-amino acid biosynthetic process | 1 | 0 | 0.04 | 1 |
| GO:0046439 | L-cysteine metabolic process | 3 | 0 | 0.11 | 1 |
| GO:0046440 | L-lysine metabolic process | 4 | 0 | 0.15 | 1 |
| GO:0046443 | FAD metabolic process | 1 | 0 | 0.04 | 1 |
| GO:0046448 | tropane alkaloid metabolic process | 1 | 0 | 0.04 | 1 |
| GO:0046449 | creatinine metabolic process | 4 | 0 | 0.15 | 1 |
| GO:0046452 | dihydrofolate metabolic process | 1 | 0 | 0.04 | 1 |
| GO:0046459 | short-chain fatty acid metabolic process | 14 | 0 | 0.53 | 1 |
| GO:0046465 | dolichyl diphosphate metabolic process | 5 | 0 | 0.19 | 1 |
| GO:0046466 | membrane lipid catabolic process | 24 | 0 | 0.91 | 1 |
| GO:0046471 | phosphatidylglycerol metabolic process | 34 | 0 | 1.29 | 1 |
| GO:0046473 | phosphatidic acid metabolic process | 30 | 0 | 1.14 | 1 |
| GO:0046475 | glycerophospholipid catabolic process | 11 | 0 | 0.42 | 1 |
| GO:0046476 | glycosylceramide biosynthetic process | 4 | 0 | 0.15 | 1 |
| GO:0046477 | glycosylceramide catabolic process | 7 | 0 | 0.27 | 1 |
| GO:0046479 | glycosphingolipid catabolic process | 12 | 0 | 0.46 | 1 |
| GO:0046485 | ether lipid metabolic process | 7 | 0 | 0.27 | 1 |
| GO:0046487 | glyoxylate metabolic process | 7 | 0 | 0.27 | 1 |
| GO:0046490 | isopentenyl diphosphate metabolic proces. | 4 | 0 | 0.15 | 1 |
| GO:0046491 | L-methylmalonyl-CoA metabolic process | 1 | 0 | 0.04 | 1 |
| GO:0046498 | S-adenosylhomocysteine metabolic proces | 4 | 0 | 0.15 | 1 |
| GO:0046499 | S-adenosylmethioninamine metabolic proc | 3 | 0 | 0.11 | 1 |
| GO:0046501 | protoporphyrinogen IX metabolic process | 11 | 0 | 0.42 | 1 |
| GO:0046502 | uroporphyrinogen III metabolic process | 2 | 0 | 0.08 | 1 |
| GO:0046504 | glycerol ether biosynthetic process | 5 | 0 | 0.19 | 1 |
| GO:0046511 | sphinganine biosynthetic process | 2 | 0 | 0.08 | 1 |
| GO:0046512 | sphingosine biosynthetic process | 6 | 0 | 0.23 | 1 |
| GO:0046514 | ceramide catabolic process | 13 | 0 | 0.49 | 1 |
| GO:0046519 | sphingoid metabolic process | 13 | 0 | 0.49 | 1 |
| GO:0046520 | sphingoid biosynthetic process | 7 | 0 | 0.27 | 1 |
| GO:0046521 | sphingoid catabolic process | 1 | 0 | 0.04 | 1 |
| GO:0046532 | regulation of photoreceptor cell differe... | 5 | 0 | 0.19 | 1 |
| GO:0046533 | negative regulation of photoreceptor cel... | 4 | 0 | 0.15 | 1 |
| GO:0046534 | positive regulation of photoreceptor cel... | 1 | 0 | 0.04 | 1 |
| GO:0046543 | development of secondary female sexual c | 8 | 0 | 0.3 | 1 |
| GO:0046544 | development of secondary male sexual ch | 2 | 0 | 0.08 | 1 |
| GO:0046552 | photoreceptor cell fate commitment | 2 | 0 | 0.08 | 1 |
| GO:0046579 | positive regulation of Ras protein signa... | 31 | 0 | 1.18 | 1 |
| GO:0046588 | negative regulation of calcium-dependent.. | 1 | 0 | 0.04 | 1 |
| GO:0046598 | positive regulation of viral entry into ... | 4 | 0 | 0.15 | 1 |
| GO:0046599 | regulation of centriole replication | 11 | 0 | 0.42 | 1 |
| GO:0046600 | negative regulation of centriole replica... | 5 | 0 | 0.19 | 1 |
| GO:0046601 | positive regulation of centriole replica... | 2 | 0 | 0.08 | 1 |
| GO:0046602 | regulation of mitotic centrosome separat... | 3 | 0 | 0.11 | 1 |
| GO:0046605 | regulation of centrosome cycle | 32 | 0 | 1.22 | 1 |

| | | | | | |
|---|---|---|---|---|---|
| GO:0046606 | negative regulation of centrosome cycle | 9 | 0 | 0.34 | 1 |
| GO:0046607 | positive regulation of centrosome cycle | 3 | 0 | 0.11 | 1 |
| GO:0046618 | drug export | 1 | 0 | 0.04 | 1 |
| GO:0046619 | optic placode formation involved in came... | 2 | 0 | 0.08 | 1 |
| GO:0046629 | gamma-delta T cell activation | 15 | 0 | 0.57 | 1 |
| GO:0046633 | alpha-beta T cell proliferation | 25 | 0 | 0.95 | 1 |
| GO:0046636 | negative regulation of alpha-beta T cell... | 19 | 0 | 0.72 | 1 |
| GO:0046639 | negative regulation of alpha-beta T cell... | 12 | 0 | 0.46 | 1 |
| GO:0046640 | regulation of alpha-beta T cell prolifer... | 22 | 0 | 0.84 | 1 |
| GO:0046641 | positive regulation of alpha-beta T cell... | 17 | 0 | 0.65 | 1 |
| GO:0046642 | negative regulation of alpha-beta T cell... | 5 | 0 | 0.19 | 1 |
| GO:0046643 | regulation of gamma-delta T cell activat... | 10 | 0 | 0.38 | 1 |
| GO:0046645 | positive regulation of gamma-delta T cel... | 7 | 0 | 0.27 | 1 |
| GO:0046653 | tetrahydrofolate metabolic process | 18 | 0 | 0.69 | 1 |
| GO:0046654 | tetrahydrofolate biosynthetic process | 5 | 0 | 0.19 | 1 |
| GO:0046655 | folic acid metabolic process | 10 | 0 | 0.38 | 1 |
| GO:0046666 | retinal cell programmed cell death | 6 | 0 | 0.23 | 1 |
| GO:0046668 | regulation of retinal cell programmed ce... | 3 | 0 | 0.11 | 1 |
| GO:0046671 | negative regulation of retinal cell prog... | 1 | 0 | 0.04 | 1 |
| GO:0046680 | response to DDT | 1 | 0 | 0.04 | 1 |
| GO:0046684 | response to pyrethroid | 4 | 0 | 0.15 | 1 |
| GO:0046687 | response to chromate | 2 | 0 | 0.08 | 1 |
| GO:0046688 | response to copper ion | 19 | 0 | 0.72 | 1 |
| GO:0046690 | response to tellurium ion | 1 | 0 | 0.04 | 1 |
| GO:0046704 | CDP metabolic process | 1 | 0 | 0.04 | 1 |
| GO:0046705 | CDP biosynthetic process | 1 | 0 | 0.04 | 1 |
| GO:0046707 | IDP metabolic process | 2 | 0 | 0.08 | 1 |
| GO:0046709 | IDP catabolic process | 2 | 0 | 0.08 | 1 |
| GO:0046710 | GDP metabolic process | 2 | 0 | 0.08 | 1 |
| GO:0046711 | GDP biosynthetic process | 1 | 0 | 0.04 | 1 |
| GO:0046712 | GDP catabolic process | 1 | 0 | 0.04 | 1 |
| GO:0046713 | borate transport | 1 | 0 | 0.04 | 1 |
| GO:0046716 | muscle cell cellular homeostasis | 19 | 0 | 0.72 | 1 |
| GO:0046719 | regulation by virus of viral protein lev... | 10 | 0 | 0.38 | 1 |
| GO:0046724 | oxalic acid secretion | 2 | 0 | 0.08 | 1 |
| GO:0046725 | negative regulation by virus of viral pr... | 2 | 0 | 0.08 | 1 |
| GO:0046726 | positive regulation by virus of viral pr... | 3 | 0 | 0.11 | 1 |
| GO:0046730 | induction of host immune response by vir... | 2 | 0 | 0.08 | 1 |
| GO:0046732 | active induction of host immune response... | 2 | 0 | 0.08 | 1 |
| GO:0046778 | modification by virus of host mRNA proce... | 2 | 0 | 0.08 | 1 |
| GO:0046784 | viral mRNA export from host cell nucleus | 6 | 0 | 0.23 | 1 |
| GO:0046785 | microtubule polymerization | 40 | 0 | 1.52 | 1 |
| GO:0046813 | receptor-mediated virion attachment to h... | 5 | 0 | 0.19 | 1 |
| GO:0046814 | coreceptor-mediated virion attachment to... | 1 | 0 | 0.04 | 1 |
| GO:0046826 | negative regulation of protein export fr... | 7 | 0 | 0.27 | 1 |
| GO:0046831 | regulation of RNA export from nucleus | 10 | 0 | 0.38 | 1 |
| GO:0046832 | negative regulation of RNA export from n... | 2 | 0 | 0.08 | 1 |
| GO:0046833 | positive regulation of RNA export from n... | 2 | 0 | 0.08 | 1 |
| GO:0046835 | carbohydrate phosphorylation | 23 | 0 | 0.88 | 1 |
| GO:0046836 | glycolipid transport | 5 | 0 | 0.19 | 1 |
| GO:0046851 | negative regulation of bone remodeling | 10 | 0 | 0.38 | 1 |
| GO:0046864 | isoprenoid transport | 2 | 0 | 0.08 | 1 |
| GO:0046865 | terpenoid transport | 2 | 0 | 0.08 | 1 |

| | | | | | | |
|---|---|---|---|---|---|---|
| GO:0046874 | quinolinate metabolic process | | 6 | 0 | 0.23 | 1 |
| GO:0046882 | negative regulation of follicle-stimulat... | | 4 | 0 | 0.15 | 1 |
| GO:0046898 | response to cycloheximide | | 4 | 0 | 0.15 | 1 |
| GO:0046900 | tetrahydrofolylpolyglutamate metabolic p... | | 1 | 0 | 0.04 | 1 |
| GO:0046901 | tetrahydrofolylpolyglutamate biosyntheti... | | 1 | 0 | 0.04 | 1 |
| GO:0046909 | intermembrane transport | | 2 | 0 | 0.08 | 1 |
| GO:0046928 | regulation of neurotransmitter secretion | | 45 | 0 | 1.71 | 1 |
| GO:0046929 | negative regulation of neurotransmitter ... | | 7 | 0 | 0.27 | 1 |
| GO:0046931 | pore complex assembly | | 11 | 0 | 0.42 | 1 |
| GO:0046940 | nucleoside monophosphate phosphorylatic | | 1 | 0 | 0.04 | 1 |
| GO:0046946 | hydroxylysine metabolic process | | 1 | 0 | 0.04 | 1 |
| GO:0046947 | hydroxylysine biosynthetic process | | 1 | 0 | 0.04 | 1 |
| GO:0046950 | cellular ketone body metabolic process | | 8 | 0 | 0.3 | 1 |
| GO:0046951 | ketone body biosynthetic process | | 6 | 0 | 0.23 | 1 |
| GO:0046952 | ketone body catabolic process | | 4 | 0 | 0.15 | 1 |
| GO:0046958 | nonassociative learning | | 5 | 0 | 0.19 | 1 |
| GO:0046959 | habituation | | 3 | 0 | 0.11 | 1 |
| GO:0046960 | sensitization | | 2 | 0 | 0.08 | 1 |
| GO:0046963 | 3'-phosphoadenosine 5'-phosphosulfate tr. | | 1 | 0 | 0.04 | 1 |
| GO:0046967 | cytosol to ER transport | | 2 | 0 | 0.08 | 1 |
| GO:0046968 | peptide antigen transport | | 3 | 0 | 0.11 | 1 |
| GO:0046984 | regulation of hemoglobin biosynthetic pr... | | 4 | 0 | 0.15 | 1 |
| GO:0046985 | positive regulation of hemoglobin biosyn... | | 2 | 0 | 0.08 | 1 |
| GO:0046986 | negative regulation of hemoglobin biosyn.. | | 1 | 0 | 0.04 | 1 |
| GO:0047484 | regulation of response to osmotic stress | | 7 | 0 | 0.27 | 1 |
| GO:0047497 | mitochondrion transport along microtubul.. | | 9 | 0 | 0.34 | 1 |
| GO:0048006 | antigen processing and presentation | end... | | 1 | 0 | 0.04 |
| GO:0048014 | Tie signaling pathway | | 3 | 0 | 0.11 | 1 |
| GO:0048021 | regulation of melanin biosynthetic proce... | | 5 | 0 | 0.19 | 1 |
| GO:0048022 | negative regulation of melanin biosynthe... | | 2 | 0 | 0.08 | 1 |
| GO:0048023 | positive regulation of melanin biosynthe... | | 3 | 0 | 0.11 | 1 |
| GO:0048025 | negative regulation of mRNA splicing | vi... | | 9 | 0 | 0.34 |
| GO:0048026 | positive regulation of mRNA splicing | vi... | | 12 | 0 | 0.46 |
| GO:0048033 | heme o metabolic process | | 1 | 0 | 0.04 | 1 |
| GO:0048034 | heme O biosynthetic process | | 1 | 0 | 0.04 | 1 |
| GO:0048050 | post-embryonic eye morphogenesis | | 3 | 0 | 0.11 | 1 |
| GO:0048058 | compound eye corneal lens development | | 1 | 0 | 0.04 | 1 |
| GO:0048066 | developmental pigmentation | | 45 | 0 | 1.71 | 1 |
| GO:0048069 | eye pigmentation | | 10 | 0 | 0.38 | 1 |
| GO:0048070 | regulation of developmental pigmentation | | 15 | 0 | 0.57 | 1 |
| GO:0048073 | regulation of eye pigmentation | | 3 | 0 | 0.11 | 1 |
| GO:0048074 | negative regulation of eye pigmentation | | 1 | 0 | 0.04 | 1 |
| GO:0048075 | positive regulation of eye pigmentation | | 1 | 0 | 0.04 | 1 |
| GO:0048086 | negative regulation of developmental pig... | | 1 | 0 | 0.04 | 1 |
| GO:0048087 | positive regulation of developmental pig... | | 7 | 0 | 0.27 | 1 |
| GO:0048096 | chromatin-mediated maintenance of transc | | 6 | 0 | 0.23 | 1 |
| GO:0048102 | autophagic cell death | | 5 | 0 | 0.19 | 1 |
| GO:0048104 | establishment of body hair or bristle pl... | | 2 | 0 | 0.08 | 1 |
| GO:0048105 | establishment of body hair planar orient... | | 2 | 0 | 0.08 | 1 |
| GO:0048133 | male germ-line stem cell asymmetric divi... | | 3 | 0 | 0.11 | 1 |
| GO:0048143 | astrocyte activation | | 2 | 0 | 0.08 | 1 |
| GO:0048147 | negative regulation of fibroblast prolif... | | 27 | 0 | 1.03 | 1 |
| GO:0048148 | behavioral response to cocaine | | 12 | 0 | 0.46 | 1 |

| | | | | | |
|---|---|---|---|---|---|
| GO:0048160 | primary follicle stage | 1 | 0 | 0.04 | 1 |
| GO:0048162 | multi-layer follicle stage | 2 | 0 | 0.08 | 1 |
| GO:0048165 | fused antrum stage | 2 | 0 | 0.08 | 1 |
| GO:0048168 | regulation of neuronal synaptic plastici... | 45 | 0 | 1.71 | 1 |
| GO:0048169 | regulation of long-term neuronal synapti... | 27 | 0 | 1.03 | 1 |
| GO:0048170 | positive regulation of long-term neurona... | 6 | 0 | 0.23 | 1 |
| GO:0048172 | regulation of short-term neuronal synapt... | 9 | 0 | 0.34 | 1 |
| GO:0048175 | hepatocyte growth factor biosynthetic pr... | 1 | 0 | 0.04 | 1 |
| GO:0048176 | regulation of hepatocyte growth factor b... | 1 | 0 | 0.04 | 1 |
| GO:0048178 | negative regulation of hepatocyte growth... | 1 | 0 | 0.04 | 1 |
| GO:0048203 | vesicle targeting | trans-Golgi | 1 | 0 | 0.04 |
| GO:0048207 | vesicle targeting | rough ER to | 15 | 0 | 0.57 |
| GO:0048208 | COPII vesicle coating | 15 | 0 | 0.57 | 1 |
| GO:0048210 | Golgi vesicle fusion to target membrane | 3 | 0 | 0.11 | 1 |
| GO:0048211 | Golgi vesicle docking | 2 | 0 | 0.08 | 1 |
| GO:0048213 | Golgi vesicle prefusion complex stabiliz... | 1 | 0 | 0.04 | 1 |
| GO:0048227 | plasma membrane to endosome transport | 8 | 0 | 0.3 | 1 |
| GO:0048239 | negative regulation of DNA recombination. | 1 | 0 | 0.04 | 1 |
| GO:0048240 | sperm capacitation | 12 | 0 | 0.46 | 1 |
| GO:0048241 | epinephrine transport | 9 | 0 | 0.34 | 1 |
| GO:0048242 | epinephrine secretion | 8 | 0 | 0.3 | 1 |
| GO:0048243 | norepinephrine secretion | 14 | 0 | 0.53 | 1 |
| GO:0048245 | eosinophil chemotaxis | 10 | 0 | 0.38 | 1 |
| GO:0048250 | mitochondrial iron ion transport | 1 | 0 | 0.04 | 1 |
| GO:0048251 | elastic fiber assembly | 7 | 0 | 0.27 | 1 |
| GO:0048262 | determination of dorsal/ventral asymmetr... | 9 | 0 | 0.34 | 1 |
| GO:0048263 | determination of dorsal identity | 9 | 0 | 0.34 | 1 |
| GO:0048266 | behavioral response to pain | 16 | 0 | 0.61 | 1 |
| GO:0048268 | clathrin coat assembly | 12 | 0 | 0.46 | 1 |
| GO:0048280 | vesicle fusion with Golgi apparatus | 8 | 0 | 0.3 | 1 |
| GO:0048286 | lung alveolus development | 45 | 0 | 1.71 | 1 |
| GO:0048289 | isotype switching to IgE isotypes | 7 | 0 | 0.27 | 1 |
| GO:0048290 | isotype switching to IgA isotypes | 4 | 0 | 0.15 | 1 |
| GO:0048291 | isotype switching to IgG isotypes | 10 | 0 | 0.38 | 1 |
| GO:0048293 | regulation of isotype switching to IgE i... | 7 | 0 | 0.27 | 1 |
| GO:0048294 | negative regulation of isotype switching... | 3 | 0 | 0.11 | 1 |
| GO:0048295 | positive regulation of isotype switching... | 4 | 0 | 0.15 | 1 |
| GO:0048296 | regulation of isotype switching to IgA i... | 4 | 0 | 0.15 | 1 |
| GO:0048297 | negative regulation of isotype switching... | 1 | 0 | 0.04 | 1 |
| GO:0048298 | positive regulation of isotype switching... | 3 | 0 | 0.11 | 1 |
| GO:0048302 | regulation of isotype switching to IgG i... | 9 | 0 | 0.34 | 1 |
| GO:0048304 | positive regulation of isotype switching... | 6 | 0 | 0.23 | 1 |
| GO:0048305 | immunoglobulin secretion | 19 | 0 | 0.72 | 1 |
| GO:0048308 | organelle inheritance | 11 | 0 | 0.42 | 1 |
| GO:0048311 | mitochondrion distribution | 7 | 0 | 0.27 | 1 |
| GO:0048312 | intracellular distribution of mitochondr... | 3 | 0 | 0.11 | 1 |
| GO:0048313 | Golgi inheritance | 11 | 0 | 0.42 | 1 |
| GO:0048318 | axial mesoderm development | 8 | 0 | 0.3 | 1 |
| GO:0048319 | axial mesoderm morphogenesis | 5 | 0 | 0.19 | 1 |
| GO:0048320 | axial mesoderm formation | 3 | 0 | 0.11 | 1 |
| GO:0048321 | axial mesodermal cell differentiation | 1 | 0 | 0.04 | 1 |
| GO:0048322 | axial mesodermal cell fate commitment | 1 | 0 | 0.04 | 1 |
| GO:0048327 | axial mesodermal cell fate specification | 1 | 0 | 0.04 | 1 |

| | | | | | |
|---|---|---|---|---|---|
| GO:0048333 | mesodermal cell differentiation | 31 | 0 | 1.18 | 1 |
| GO:0048337 | positive regulation of mesodermal cell f... | 1 | 0 | 0.04 | 1 |
| GO:0048338 | mesoderm structural organization | 1 | 0 | 0.04 | 1 |
| GO:0048342 | paraxial mesodermal cell differentiation | 2 | 0 | 0.08 | 1 |
| GO:0048343 | paraxial mesodermal cell fate commitment | 2 | 0 | 0.08 | 1 |
| GO:0048352 | paraxial mesoderm structural organizatio... | 1 | 0 | 0.04 | 1 |
| GO:0048364 | root development | 1 | 0 | 0.04 | 1 |
| GO:0048368 | lateral mesoderm development | 14 | 0 | 0.53 | 1 |
| GO:0048369 | lateral mesoderm morphogenesis | 4 | 0 | 0.15 | 1 |
| GO:0048370 | lateral mesoderm formation | 4 | 0 | 0.15 | 1 |
| GO:0048371 | lateral mesodermal cell differentiation | 3 | 0 | 0.11 | 1 |
| GO:0048372 | lateral mesodermal cell fate commitment | 2 | 0 | 0.08 | 1 |
| GO:0048377 | lateral mesodermal cell fate specificati... | 2 | 0 | 0.08 | 1 |
| GO:0048378 | regulation of lateral mesodermal cell fa... | 2 | 0 | 0.08 | 1 |
| GO:0048382 | mesendoderm development | 3 | 0 | 0.11 | 1 |
| GO:0048385 | regulation of retinoic acid receptor sig... | 30 | 0 | 1.14 | 1 |
| GO:0048386 | positive regulation of retinoic acid rec... | 5 | 0 | 0.19 | 1 |
| GO:0048387 | negative regulation of retinoic acid rec... | 24 | 0 | 0.91 | 1 |
| GO:0048388 | endosomal lumen acidification | 3 | 0 | 0.11 | 1 |
| GO:0048389 | intermediate mesoderm development | 2 | 0 | 0.08 | 1 |
| GO:0048390 | intermediate mesoderm morphogenesis | 1 | 0 | 0.04 | 1 |
| GO:0048391 | intermediate mesoderm formation | 1 | 0 | 0.04 | 1 |
| GO:0048392 | intermediate mesodermal cell differentia... | 1 | 0 | 0.04 | 1 |
| GO:0048483 | autonomic nervous system development | 43 | 0 | 1.64 | 1 |
| GO:0048484 | enteric nervous system development | 12 | 0 | 0.46 | 1 |
| GO:0048485 | sympathetic nervous system development | 22 | 0 | 0.84 | 1 |
| GO:0048486 | parasympathetic nervous system developm | 17 | 0 | 0.65 | 1 |
| GO:0048496 | maintenance of organ identity | 4 | 0 | 0.15 | 1 |
| GO:0048499 | synaptic vesicle membrane organization | 3 | 0 | 0.11 | 1 |
| GO:0048505 | regulation of timing of cell differentia... | 11 | 0 | 0.42 | 1 |
| GO:0048532 | anatomical structure arrangement | 18 | 0 | 0.69 | 1 |
| GO:0048535 | lymph node development | 17 | 0 | 0.65 | 1 |
| GO:0048536 | spleen development | 34 | 0 | 1.29 | 1 |
| GO:0048537 | mucosal-associated lymphoid tissue devel | 12 | 0 | 0.46 | 1 |
| GO:0048538 | thymus development | 40 | 0 | 1.52 | 1 |
| GO:0048539 | bone marrow development | 5 | 0 | 0.19 | 1 |
| GO:0048541 | Peyer's patch development | 12 | 0 | 0.46 | 1 |
| GO:0048548 | regulation of pinocytosis | 8 | 0 | 0.3 | 1 |
| GO:0048549 | positive regulation of pinocytosis | 4 | 0 | 0.15 | 1 |
| GO:0048550 | negative regulation of pinocytosis | 4 | 0 | 0.15 | 1 |
| GO:0048552 | regulation of metalloenzyme activity | 12 | 0 | 0.46 | 1 |
| GO:0048553 | negative regulation of metalloenzyme act... | 6 | 0 | 0.23 | 1 |
| GO:0048554 | positive regulation of metalloenzyme act... | 6 | 0 | 0.23 | 1 |
| GO:0048561 | establishment of organ orientation | 1 | 0 | 0.04 | 1 |
| GO:0048563 | post-embryonic organ morphogenesis | 5 | 0 | 0.19 | 1 |
| GO:0048567 | ectodermal digestive tract morphogenesis | 1 | 0 | 0.04 | 1 |
| GO:0048569 | post-embryonic organ development | 11 | 0 | 0.42 | 1 |
| GO:0048570 | notochord morphogenesis | 8 | 0 | 0.3 | 1 |
| GO:0048597 | post-embryonic camera-type eye morphog | 3 | 0 | 0.11 | 1 |
| GO:0048599 | oocyte development | 36 | 0 | 1.37 | 1 |
| GO:0048611 | embryonic ectodermal digestive tract dev... | 2 | 0 | 0.08 | 1 |
| GO:0048613 | embryonic ectodermal digestive tract mor... | 1 | 0 | 0.04 | 1 |
| GO:0048617 | embryonic foregut morphogenesis | 11 | 0 | 0.42 | 1 |

| | | | | | |
|---|---|---|---|---|---|
| GO:0048621 | post-embryonic digestive tract morphogen. | 1 | 0 | 0.04 | 1 |
| GO:0048625 | myoblast fate commitment | 5 | 0 | 0.19 | 1 |
| GO:0048627 | myoblast development | 2 | 0 | 0.08 | 1 |
| GO:0048630 | skeletal muscle tissue growth | 6 | 0 | 0.23 | 1 |
| GO:0048631 | regulation of skeletal muscle tissue gro... | 3 | 0 | 0.11 | 1 |
| GO:0048632 | negative regulation of skeletal muscle t... | 1 | 0 | 0.04 | 1 |
| GO:0048633 | positive regulation of skeletal muscle t... | 2 | 0 | 0.08 | 1 |
| GO:0048642 | negative regulation of skeletal muscle t... | 11 | 0 | 0.42 | 1 |
| GO:0048662 | negative regulation of smooth muscle cel.. | 29 | 0 | 1.1 | 1 |
| GO:0048664 | neuron fate determination | 8 | 0 | 0.3 | 1 |
| GO:0048669 | collateral sprouting in absence of injur... | 5 | 0 | 0.19 | 1 |
| GO:0048672 | positive regulation of collateral sprout... | 6 | 0 | 0.23 | 1 |
| GO:0048673 | collateral sprouting of intact axon in r... | 2 | 0 | 0.08 | 1 |
| GO:0048681 | negative regulation of axon regeneration | 4 | 0 | 0.15 | 1 |
| GO:0048683 | regulation of collateral sprouting of in... | 2 | 0 | 0.08 | 1 |
| GO:0048685 | negative regulation of collateral sprout... | 1 | 0 | 0.04 | 1 |
| GO:0048688 | negative regulation of sprouting of inju... | 1 | 0 | 0.04 | 1 |
| GO:0048692 | negative regulation of axon extension in... | 1 | 0 | 0.04 | 1 |
| GO:0048696 | regulation of collateral sprouting in ab... | 3 | 0 | 0.11 | 1 |
| GO:0048697 | positive regulation of collateral sprout... | 2 | 0 | 0.08 | 1 |
| GO:0048698 | negative regulation of collateral sprout... | 1 | 0 | 0.04 | 1 |
| GO:0048711 | positive regulation of astrocyte differe... | 10 | 0 | 0.38 | 1 |
| GO:0048714 | positive regulation of oligodendrocyte d... | 11 | 0 | 0.42 | 1 |
| GO:0048715 | negative regulation of oligodendrocyte d... | 12 | 0 | 0.46 | 1 |
| GO:0048739 | cardiac muscle fiber development | 8 | 0 | 0.3 | 1 |
| GO:0048743 | positive regulation of skeletal muscle f... | 6 | 0 | 0.23 | 1 |
| GO:0048749 | compound eye development | 2 | 0 | 0.08 | 1 |
| GO:0048757 | pigment granule maturation | 7 | 0 | 0.27 | 1 |
| GO:0048764 | trichoblast maturation | 1 | 0 | 0.04 | 1 |
| GO:0048765 | root hair cell differentiation | 1 | 0 | 0.04 | 1 |
| GO:0048767 | root hair elongation | 1 | 0 | 0.04 | 1 |
| GO:0048769 | sarcomerogenesis | 3 | 0 | 0.11 | 1 |
| GO:0048773 | erythrophore differentiation | 1 | 0 | 0.04 | 1 |
| GO:0048789 | cytoskeletal matrix organization at acti... | 1 | 0 | 0.04 | 1 |
| GO:0048790 | maintenance of presynaptic active zone s. | 1 | 0 | 0.04 | 1 |
| GO:0048791 | calcium ion-dependent exocytosis of neur. | 27 | 0 | 1.03 | 1 |
| GO:0048793 | pronephros development | 8 | 0 | 0.3 | 1 |
| GO:0048807 | female genitalia morphogenesis | 3 | 0 | 0.11 | 1 |
| GO:0048808 | male genitalia morphogenesis | 3 | 0 | 0.11 | 1 |
| GO:0048817 | negative regulation of hair follicle mat... | 1 | 0 | 0.04 | 1 |
| GO:0048818 | positive regulation of hair follicle mat... | 4 | 0 | 0.15 | 1 |
| GO:0048819 | regulation of hair follicle maturation | 5 | 0 | 0.19 | 1 |
| GO:0048820 | hair follicle maturation | 8 | 0 | 0.3 | 1 |
| GO:0048822 | enucleate erythrocyte development | 2 | 0 | 0.08 | 1 |
| GO:0048840 | otolith development | 5 | 0 | 0.19 | 1 |
| GO:0048841 | regulation of axon extension involved in... | 15 | 0 | 0.57 | 1 |
| GO:0048842 | positive regulation of axon extension in... | 7 | 0 | 0.27 | 1 |
| GO:0048843 | negative regulation of axon extension in... | 9 | 0 | 0.34 | 1 |
| GO:0048845 | venous blood vessel morphogenesis | 8 | 0 | 0.3 | 1 |
| GO:0048846 | axon extension involved in axon guidance | 18 | 0 | 0.69 | 1 |
| GO:0048850 | hypophysis morphogenesis | 3 | 0 | 0.11 | 1 |
| GO:0048852 | diencephalon morphogenesis | 6 | 0 | 0.23 | 1 |
| GO:0048855 | adenohypophysis morphogenesis | 1 | 0 | 0.04 | 1 |

| | | | | | |
|---|---|---|---|---|---|
| GO:0048857 | neural nucleus development | 66 | 0 | 2.51 | 1 |
| GO:0048859 | formation of anatomical boundary | 6 | 0 | 0.23 | 1 |
| GO:0048861 | leukemia inhibitory factor signaling pat... | 4 | 0 | 0.15 | 1 |
| GO:0048865 | stem cell fate commitment | 5 | 0 | 0.19 | 1 |
| GO:0048866 | stem cell fate specification | 2 | 0 | 0.08 | 1 |
| GO:0048867 | stem cell fate determination | 3 | 0 | 0.11 | 1 |
| GO:0048874 | homeostasis of number of cells in a free... | 1 | 0 | 0.04 | 1 |
| GO:0048875 | chemical homeostasis within a tissue | 11 | 0 | 0.42 | 1 |
| GO:0048880 | sensory system development | 4 | 0 | 0.15 | 1 |
| GO:0048892 | lateral line nerve development | 1 | 0 | 0.04 | 1 |
| GO:0048894 | efferent axon development in a lateral l... | 1 | 0 | 0.04 | 1 |
| GO:0048925 | lateral line system development | 1 | 0 | 0.04 | 1 |
| GO:0048936 | peripheral nervous system neuron axonog | 3 | 0 | 0.11 | 1 |
| GO:0050000 | chromosome localization | 44 | 0 | 1.67 | 1 |
| GO:0050432 | catecholamine secretion | 39 | 0 | 1.48 | 1 |
| GO:0050433 | regulation of catecholamine secretion | 35 | 0 | 1.33 | 1 |
| GO:0050435 | beta-amyloid metabolic process | 22 | 0 | 0.84 | 1 |
| GO:0050482 | arachidonic acid secretion | 19 | 0 | 0.72 | 1 |
| GO:0050650 | chondroitin sulfate proteoglycan biosynt... | 28 | 0 | 1.07 | 1 |
| GO:0050651 | dermatan sulfate proteoglycan biosynthet.. | 15 | 0 | 0.57 | 1 |
| GO:0050652 | dermatan sulfate proteoglycan biosynthet.. | 1 | 0 | 0.04 | 1 |
| GO:0050653 | chondroitin sulfate proteoglycan biosynt... | 2 | 0 | 0.08 | 1 |
| GO:0050655 | dermatan sulfate proteoglycan metabolic .. | 16 | 0 | 0.61 | 1 |
| GO:0050665 | hydrogen peroxide biosynthetic process | 11 | 0 | 0.42 | 1 |
| GO:0050666 | regulation of homocysteine metabolic pro.. | 2 | 0 | 0.08 | 1 |
| GO:0050667 | homocysteine metabolic process | 11 | 0 | 0.42 | 1 |
| GO:0050668 | positive regulation of homocysteine meta.. | 1 | 0 | 0.04 | 1 |
| GO:0050674 | urothelial cell proliferation | 1 | 0 | 0.04 | 1 |
| GO:0050675 | regulation of urothelial cell proliferat... | 1 | 0 | 0.04 | 1 |
| GO:0050677 | positive regulation of urothelial cell p... | 1 | 0 | 0.04 | 1 |
| GO:0050686 | negative regulation of mRNA processing | 19 | 0 | 0.72 | 1 |
| GO:0050687 | negative regulation of defense response ... | 17 | 0 | 0.65 | 1 |
| GO:0050689 | negative regulation of defense response ... | 5 | 0 | 0.19 | 1 |
| GO:0050691 | regulation of defense response to virus ... | 26 | 0 | 0.99 | 1 |
| GO:0050701 | interleukin-1 secretion | 28 | 0 | 1.07 | 1 |
| GO:0050702 | interleukin-1 beta secretion | 25 | 0 | 0.95 | 1 |
| GO:0050703 | interleukin-1 alpha secretion | 4 | 0 | 0.15 | 1 |
| GO:0050704 | regulation of interleukin-1 secretion | 24 | 0 | 0.91 | 1 |
| GO:0050705 | regulation of interleukin-1 alpha secret... | 3 | 0 | 0.11 | 1 |
| GO:0050706 | regulation of interleukin-1 beta secreti... | 22 | 0 | 0.84 | 1 |
| GO:0050711 | negative regulation of interleukin-1 sec... | 4 | 0 | 0.15 | 1 |
| GO:0050713 | negative regulation of interleukin-1 bet... | 2 | 0 | 0.08 | 1 |
| GO:0050716 | positive regulation of interleukin-1 sec... | 20 | 0 | 0.76 | 1 |
| GO:0050717 | positive regulation of interleukin-1 alp... | 3 | 0 | 0.11 | 1 |
| GO:0050718 | positive regulation of interleukin-1 bet... | 19 | 0 | 0.72 | 1 |
| GO:0050719 | interleukin-1 alpha biosynthetic process | 1 | 0 | 0.04 | 1 |
| GO:0050720 | interleukin-1 beta biosynthetic process | 2 | 0 | 0.08 | 1 |
| GO:0050721 | regulation of interleukin-1 alpha biosyn... | 1 | 0 | 0.04 | 1 |
| GO:0050722 | regulation of interleukin-1 beta biosynt... | 1 | 0 | 0.04 | 1 |
| GO:0050725 | positive regulation of interleukin-1 bet... | 1 | 0 | 0.04 | 1 |
| GO:0050726 | positive regulation of interleukin-1 alp... | 1 | 0 | 0.04 | 1 |
| GO:0050747 | positive regulation of lipoprotein metab... | 1 | 0 | 0.04 | 1 |
| GO:0050748 | negative regulation of lipoprotein metab... | 6 | 0 | 0.23 | 1 |

| | | | | | |
|---|---|---|---|---|---|
| GO:0050751 | fractalkine biosynthetic process | 1 | 0 | 0.04 | 1 |
| GO:0050752 | regulation of fractalkine biosynthetic p... | 1 | 0 | 0.04 | 1 |
| GO:0050754 | positive regulation of fractalkine biosy... | 1 | 0 | 0.04 | 1 |
| GO:0050755 | chemokine metabolic process | 16 | 0 | 0.61 | 1 |
| GO:0050756 | fractalkine metabolic process | 3 | 0 | 0.11 | 1 |
| GO:0050757 | thymidylate synthase biosynthetic proces.. | 1 | 0 | 0.04 | 1 |
| GO:0050758 | regulation of thymidylate synthase biosy... | 1 | 0 | 0.04 | 1 |
| GO:0050760 | negative regulation of thymidylate synth... | 1 | 0 | 0.04 | 1 |
| GO:0050775 | positive regulation of dendrite morphoge... | 23 | 0 | 0.88 | 1 |
| GO:0050783 | cocaine metabolic process | 1 | 0 | 0.04 | 1 |
| GO:0050787 | detoxification of mercury ion | 4 | 0 | 0.15 | 1 |
| GO:0050798 | activated T cell proliferation | 41 | 0 | 1.56 | 1 |
| GO:0050802 | circadian sleep/wake cycle | sleep | 19 | 0 | 0.72 |
| GO:0050820 | positive regulation of coagulation | 26 | 0 | 0.99 | 1 |
| GO:0050822 | peptide stabilization | 1 | 0 | 0.04 | 1 |
| GO:0050823 | peptide antigen stabilization | 1 | 0 | 0.04 | 1 |
| GO:0050826 | response to freezing | 1 | 0 | 0.04 | 1 |
| GO:0050829 | defense response to Gram-negative bacte | 33 | 0 | 1.26 | 1 |
| GO:0050830 | defense response to Gram-positive bacter. | 63 | 0 | 2.4 | 1 |
| GO:0050847 | progesterone receptor signaling pathway | 6 | 0 | 0.23 | 1 |
| GO:0050848 | regulation of calcium-mediated signaling | 41 | 0 | 1.56 | 1 |
| GO:0050849 | negative regulation of calcium-mediated ... | 6 | 0 | 0.23 | 1 |
| GO:0050850 | positive regulation of calcium-mediated ... | 25 | 0 | 0.95 | 1 |
| GO:0050859 | negative regulation of B cell receptor s... | 5 | 0 | 0.19 | 1 |
| GO:0050862 | positive regulation of T cell receptor s... | 6 | 0 | 0.23 | 1 |
| GO:0050872 | white fat cell differentiation | 12 | 0 | 0.46 | 1 |
| GO:0050882 | voluntary musculoskeletal movement | 3 | 0 | 0.11 | 1 |
| GO:0050883 | musculoskeletal movement | spinal refle› | 3 | 0 | 0.11 |
| GO:0050894 | determination of affect | 1 | 0 | 0.04 | 1 |
| GO:0050902 | leukocyte adhesive activation | 2 | 0 | 0.08 | 1 |
| GO:0050904 | diapedesis | 2 | 0 | 0.08 | 1 |
| GO:0050910 | detection of mechanical stimulus involve... | 13 | 0 | 0.49 | 1 |
| GO:0050912 | detection of chemical stimulus involved ... | 41 | 0 | 1.56 | 1 |
| GO:0050913 | sensory perception of bitter taste | 35 | 0 | 1.33 | 1 |
| GO:0050915 | sensory perception of sour taste | 2 | 0 | 0.08 | 1 |
| GO:0050916 | sensory perception of sweet taste | 5 | 0 | 0.19 | 1 |
| GO:0050917 | sensory perception of umami taste | 5 | 0 | 0.19 | 1 |
| GO:0050924 | positive regulation of negative chemotax... | 1 | 0 | 0.04 | 1 |
| GO:0050928 | negative regulation of positive chemotax... | 1 | 0 | 0.04 | 1 |
| GO:0050929 | induction of negative chemotaxis | 1 | 0 | 0.04 | 1 |
| GO:0050930 | induction of positive chemotaxis | 14 | 0 | 0.53 | 1 |
| GO:0050931 | pigment cell differentiation | 33 | 0 | 1.26 | 1 |
| GO:0050932 | regulation of pigment cell differentiati... | 7 | 0 | 0.27 | 1 |
| GO:0050942 | positive regulation of pigment cell diff... | 5 | 0 | 0.19 | 1 |
| GO:0050951 | sensory perception of temperature stimul.. | 16 | 0 | 0.61 | 1 |
| GO:0050955 | thermoception | 4 | 0 | 0.15 | 1 |
| GO:0050957 | equilibrioception | 8 | 0 | 0.3 | 1 |
| GO:0050961 | detection of temperature stimulus involv... | 9 | 0 | 0.34 | 1 |
| GO:0050965 | detection of temperature stimulus involv... | 9 | 0 | 0.34 | 1 |
| GO:0050966 | detection of mechanical stimulus involve... | 8 | 0 | 0.3 | 1 |
| GO:0050968 | detection of chemical stimulus involved ... | 1 | 0 | 0.04 | 1 |
| GO:0050973 | detection of mechanical stimulus involve... | 1 | 0 | 0.04 | 1 |
| GO:0050974 | detection of mechanical stimulus involve... | 23 | 0 | 0.88 | 1 |

| | | | | | |
|---|---|---|---|---|---|
| GO:0050975 | sensory perception of touch | 4 | 0 | 0.15 | 1 |
| GO:0050976 | detection of mechanical stimulus involve... | 2 | 0 | 0.08 | 1 |
| GO:0050982 | detection of mechanical stimulus | 34 | 0 | 1.29 | 1 |
| GO:0050983 | deoxyhypusine biosynthetic process from . | 1 | 0 | 0.04 | 1 |
| GO:0050992 | dimethylallyl diphosphate biosynthetic p... | 2 | 0 | 0.08 | 1 |
| GO:0050993 | dimethylallyl diphosphate metabolic proc... | 2 | 0 | 0.08 | 1 |
| GO:0050995 | negative regulation of lipid catabolic p... | 18 | 0 | 0.69 | 1 |
| GO:0050999 | regulation of nitric-oxide synthase acti... | 41 | 0 | 1.56 | 1 |
| GO:0051000 | positive regulation of nitric-oxide synt... | 19 | 0 | 0.72 | 1 |
| GO:0051001 | negative regulation of nitric-oxide synt... | 8 | 0 | 0.3 | 1 |
| GO:0051004 | regulation of lipoprotein lipase activit... | 15 | 0 | 0.57 | 1 |
| GO:0051005 | negative regulation of lipoprotein lipas... | 5 | 0 | 0.19 | 1 |
| GO:0051006 | positive regulation of lipoprotein lipas... | 10 | 0 | 0.38 | 1 |
| GO:0051012 | microtubule sliding | 1 | 0 | 0.04 | 1 |
| GO:0051013 | microtubule severing | 7 | 0 | 0.27 | 1 |
| GO:0051014 | actin filament severing | 8 | 0 | 0.3 | 1 |
| GO:0051023 | regulation of immunoglobulin secretion | 15 | 0 | 0.57 | 1 |
| GO:0051024 | positive regulation of immunoglobulin se... | 10 | 0 | 0.38 | 1 |
| GO:0051025 | negative regulation of immunoglobulin se.. | 1 | 0 | 0.04 | 1 |
| GO:0051026 | chiasma assembly | 7 | 0 | 0.27 | 1 |
| GO:0051030 | snRNA transport | 3 | 0 | 0.11 | 1 |
| GO:0051031 | tRNA transport | 2 | 0 | 0.08 | 1 |
| GO:0051036 | regulation of endosome size | 4 | 0 | 0.15 | 1 |
| GO:0051037 | regulation of transcription during meios... | 1 | 0 | 0.04 | 1 |
| GO:0051039 | positive regulation of transcription dur... | 1 | 0 | 0.04 | 1 |
| GO:0051040 | regulation of calcium-independent cell-c... | 2 | 0 | 0.08 | 1 |
| GO:0051041 | positive regulation of calcium-independe... | 1 | 0 | 0.04 | 1 |
| GO:0051042 | negative regulation of calcium-independe.. | 1 | 0 | 0.04 | 1 |
| GO:0051043 | regulation of membrane protein ectodomai | 21 | 0 | 0.8 | 1 |
| GO:0051044 | positive regulation of membrane protein ... | 15 | 0 | 0.57 | 1 |
| GO:0051045 | negative regulation of membrane protein .. | 6 | 0 | 0.23 | 1 |
| GO:0051083 | 'de novo' cotranslational protein foldin... | 2 | 0 | 0.08 | 1 |
| GO:0051085 | chaperone mediated protein folding requi.. | 10 | 0 | 0.38 | 1 |
| GO:0051088 | PMA-inducible membrane protein ectodom | 3 | 0 | 0.11 | 1 |
| GO:0051089 | constitutive protein ectodomain proteoly... | 1 | 0 | 0.04 | 1 |
| GO:0051095 | regulation of helicase activity | 8 | 0 | 0.3 | 1 |
| GO:0051096 | positive regulation of helicase activity | 5 | 0 | 0.19 | 1 |
| GO:0051097 | negative regulation of helicase activity | 3 | 0 | 0.11 | 1 |
| GO:0051102 | DNA ligation involved in DNA recombinati.. | 1 | 0 | 0.04 | 1 |
| GO:0051103 | DNA ligation involved in DNA repair | 5 | 0 | 0.19 | 1 |
| GO:0051105 | regulation of DNA ligation | 3 | 0 | 0.11 | 1 |
| GO:0051106 | positive regulation of DNA ligation | 3 | 0 | 0.11 | 1 |
| GO:0051121 | hepoxilin metabolic process | 5 | 0 | 0.19 | 1 |
| GO:0051122 | hepoxilin biosynthetic process | 5 | 0 | 0.19 | 1 |
| GO:0051124 | synaptic growth at neuromuscular junctio.. | 6 | 0 | 0.23 | 1 |
| GO:0051125 | regulation of actin nucleation | 18 | 0 | 0.69 | 1 |
| GO:0051126 | negative regulation of actin nucleation | 5 | 0 | 0.19 | 1 |
| GO:0051127 | positive regulation of actin nucleation | 9 | 0 | 0.34 | 1 |
| GO:0051132 | NK T cell activation | 7 | 0 | 0.27 | 1 |
| GO:0051133 | regulation of NK T cell activation | 6 | 0 | 0.23 | 1 |
| GO:0051134 | negative regulation of NK T cell activat... | 1 | 0 | 0.04 | 1 |
| GO:0051135 | positive regulation of NK T cell activat... | 5 | 0 | 0.19 | 1 |
| GO:0051140 | regulation of NK T cell proliferation | 3 | 0 | 0.11 | 1 |

| | | | | | |
|---|---|---|---|---|---|
| GO:0051142 | positive regulation of NK T cell prolife... | 3 | 0 | 0.11 | 1 |
| GO:0051150 | regulation of smooth muscle cell differe... | 15 | 0 | 0.57 | 1 |
| GO:0051151 | negative regulation of smooth muscle cel.. | 6 | 0 | 0.23 | 1 |
| GO:0051152 | positive regulation of smooth muscle cel... | 5 | 0 | 0.19 | 1 |
| GO:0051156 | glucose 6-phosphate metabolic process | 21 | 0 | 0.8 | 1 |
| GO:0051160 | L-xylitol catabolic process | 1 | 0 | 0.04 | 1 |
| GO:0051164 | L-xylitol metabolic process | 1 | 0 | 0.04 | 1 |
| GO:0051176 | positive regulation of sulfur metabolic ... | 3 | 0 | 0.11 | 1 |
| GO:0051180 | vitamin transport | 26 | 0 | 0.99 | 1 |
| GO:0051181 | cofactor transport | 23 | 0 | 0.88 | 1 |
| GO:0051182 | coenzyme transport | 5 | 0 | 0.19 | 1 |
| GO:0051187 | cofactor catabolic process | 17 | 0 | 0.65 | 1 |
| GO:0051189 | prosthetic group metabolic process | 7 | 0 | 0.27 | 1 |
| GO:0051204 | protein insertion into mitochondrial mem... | 29 | 0 | 1.1 | 1 |
| GO:0051231 | spindle elongation | 7 | 0 | 0.27 | 1 |
| GO:0051255 | spindle midzone assembly | 8 | 0 | 0.3 | 1 |
| GO:0051256 | mitotic spindle midzone assembly | 6 | 0 | 0.23 | 1 |
| GO:0051257 | meiotic spindle midzone assembly | 1 | 0 | 0.04 | 1 |
| GO:0051280 | negative regulation of release of seques... | 3 | 0 | 0.11 | 1 |
| GO:0051281 | positive regulation of release of seques... | 25 | 0 | 0.95 | 1 |
| GO:0051284 | positive regulation of sequestering of c... | 2 | 0 | 0.08 | 1 |
| GO:0051290 | protein heterotetramerization | 35 | 0 | 1.33 | 1 |
| GO:0051292 | nuclear pore complex assembly | 7 | 0 | 0.27 | 1 |
| GO:0051293 | establishment of spindle localization | 28 | 0 | 1.07 | 1 |
| GO:0051294 | establishment of spindle orientation | 21 | 0 | 0.8 | 1 |
| GO:0051295 | establishment of meiotic spindle localiz... | 5 | 0 | 0.19 | 1 |
| GO:0051296 | establishment of meiotic spindle orienta... | 1 | 0 | 0.04 | 1 |
| GO:0051299 | centrosome separation | 8 | 0 | 0.3 | 1 |
| GO:0051303 | establishment of chromosome localization | 44 | 0 | 1.67 | 1 |
| GO:0051305 | chromosome movement towards spindle p | 6 | 0 | 0.23 | 1 |
| GO:0051307 | meiotic chromosome separation | 9 | 0 | 0.34 | 1 |
| GO:0051310 | metaphase plate congression | 35 | 0 | 1.33 | 1 |
| GO:0051311 | meiotic metaphase plate congression | 1 | 0 | 0.04 | 1 |
| GO:0051315 | attachment of mitotic spindle microtubul... | 4 | 0 | 0.15 | 1 |
| GO:0051342 | regulation of cyclic-nucleotide phosphod... | 8 | 0 | 0.3 | 1 |
| GO:0051343 | positive regulation of cyclic-nucleotide... | 5 | 0 | 0.19 | 1 |
| GO:0051344 | negative regulation of cyclic-nucleotide... | 4 | 0 | 0.15 | 1 |
| GO:0051354 | negative regulation of oxidoreductase ac... | 24 | 0 | 0.91 | 1 |
| GO:0051355 | proprioception involved in equilibriocep... | 1 | 0 | 0.04 | 1 |
| GO:0051365 | cellular response to potassium ion starv... | 1 | 0 | 0.04 | 1 |
| GO:0051386 | regulation of neurotrophin TRK receptor ... | 7 | 0 | 0.27 | 1 |
| GO:0051387 | negative regulation of neurotrophin TRK ... | 4 | 0 | 0.15 | 1 |
| GO:0051388 | positive regulation of neurotrophin TRK ... | 1 | 0 | 0.04 | 1 |
| GO:0051389 | inactivation of MAPKK activity | 1 | 0 | 0.04 | 1 |
| GO:0051394 | regulation of nerve growth factor recept... | 1 | 0 | 0.04 | 1 |
| GO:0051410 | detoxification of nitrogen compound | 3 | 0 | 0.11 | 1 |
| GO:0051414 | response to cortisol | 6 | 0 | 0.23 | 1 |
| GO:0051454 | intracellular pH elevation | 5 | 0 | 0.19 | 1 |
| GO:0051457 | maintenance of protein location in nucle... | 11 | 0 | 0.42 | 1 |
| GO:0051458 | corticotropin secretion | 7 | 0 | 0.27 | 1 |
| GO:0051459 | regulation of corticotropin secretion | 5 | 0 | 0.19 | 1 |
| GO:0051460 | negative regulation of corticotropin sec... | 1 | 0 | 0.04 | 1 |
| GO:0051461 | positive regulation of corticotropin sec... | 4 | 0 | 0.15 | 1 |

| | | | | | |
|---|---|---|---|---|---|
| GO:0051462 | regulation of cortisol secretion | 5 | 0 | 0.19 | 1 |
| GO:0051463 | negative regulation of cortisol secretio... | 1 | 0 | 0.04 | 1 |
| GO:0051464 | positive regulation of cortisol secretio... | 4 | 0 | 0.15 | 1 |
| GO:0051466 | positive regulation of corticotropin-rel... | 1 | 0 | 0.04 | 1 |
| GO:0051496 | positive regulation of stress fiber asse... | 37 | 0 | 1.41 | 1 |
| GO:0051503 | adenine nucleotide transport | 8 | 0 | 0.3 | 1 |
| GO:0051531 | NFAT protein import into nucleus | 13 | 0 | 0.49 | 1 |
| GO:0051532 | regulation of NFAT protein import into n... | 12 | 0 | 0.46 | 1 |
| GO:0051533 | positive regulation of NFAT protein impo... | 8 | 0 | 0.3 | 1 |
| GO:0051534 | negative regulation of NFAT protein impo.. | 4 | 0 | 0.15 | 1 |
| GO:0051552 | flavone metabolic process | 6 | 0 | 0.23 | 1 |
| GO:0051562 | negative regulation of mitochondrial cal... | 1 | 0 | 0.04 | 1 |
| GO:0051563 | smooth endoplasmic reticulum calcium ion | 2 | 0 | 0.08 | 1 |
| GO:0051567 | histone H3-K9 methylation | 20 | 0 | 0.76 | 1 |
| GO:0051569 | regulation of histone H3-K4 methylation | 19 | 0 | 0.72 | 1 |
| GO:0051570 | regulation of histone H3-K9 methylation | 9 | 0 | 0.34 | 1 |
| GO:0051571 | positive regulation of histone H3-K4 met... | 12 | 0 | 0.46 | 1 |
| GO:0051572 | negative regulation of histone H3-K4 met.. | 3 | 0 | 0.11 | 1 |
| GO:0051573 | negative regulation of histone H3-K9 met.. | 4 | 0 | 0.15 | 1 |
| GO:0051574 | positive regulation of histone H3-K9 met... | 6 | 0 | 0.23 | 1 |
| GO:0051580 | regulation of neurotransmitter uptake | 13 | 0 | 0.49 | 1 |
| GO:0051581 | negative regulation of neurotransmitter ... | 3 | 0 | 0.11 | 1 |
| GO:0051582 | positive regulation of neurotransmitter ... | 4 | 0 | 0.15 | 1 |
| GO:0051583 | dopamine uptake involved in synaptic tra... | 11 | 0 | 0.42 | 1 |
| GO:0051584 | regulation of dopamine uptake involved i... | 9 | 0 | 0.34 | 1 |
| GO:0051585 | negative regulation of dopamine uptake i... | 1 | 0 | 0.04 | 1 |
| GO:0051586 | positive regulation of dopamine uptake i... | 4 | 0 | 0.15 | 1 |
| GO:0051588 | regulation of neurotransmitter transport | 57 | 0 | 2.17 | 1 |
| GO:0051589 | negative regulation of neurotransmitter ... | 11 | 0 | 0.42 | 1 |
| GO:0051590 | positive regulation of neurotransmitter ... | 14 | 0 | 0.53 | 1 |
| GO:0051594 | detection of glucose | 4 | 0 | 0.15 | 1 |
| GO:0051595 | response to methylglyoxal | 1 | 0 | 0.04 | 1 |
| GO:0051596 | methylglyoxal catabolic process | 1 | 0 | 0.04 | 1 |
| GO:0051597 | response to methylmercury | 8 | 0 | 0.3 | 1 |
| GO:0051598 | meiotic recombination checkpoint | 2 | 0 | 0.08 | 1 |
| GO:0051599 | response to hydrostatic pressure | 7 | 0 | 0.27 | 1 |
| GO:0051601 | exocyst localization | 6 | 0 | 0.23 | 1 |
| GO:0051602 | response to electrical stimulus | 38 | 0 | 1.45 | 1 |
| GO:0051610 | serotonin uptake | 4 | 0 | 0.15 | 1 |
| GO:0051611 | regulation of serotonin uptake | 3 | 0 | 0.11 | 1 |
| GO:0051612 | negative regulation of serotonin uptake | 3 | 0 | 0.11 | 1 |
| GO:0051615 | histamine uptake | 1 | 0 | 0.04 | 1 |
| GO:0051620 | norepinephrine uptake | 1 | 0 | 0.04 | 1 |
| GO:0051621 | regulation of norepinephrine uptake | 1 | 0 | 0.04 | 1 |
| GO:0051622 | negative regulation of norepinephrine up... | 1 | 0 | 0.04 | 1 |
| GO:0051638 | barbed-end actin filament uncapping | 1 | 0 | 0.04 | 1 |
| GO:0051639 | actin filament network formation | 2 | 0 | 0.08 | 1 |
| GO:0051642 | centrosome localization | 13 | 0 | 0.49 | 1 |
| GO:0051643 | endoplasmic reticulum localization | 1 | 0 | 0.04 | 1 |
| GO:0051645 | Golgi localization | 10 | 0 | 0.38 | 1 |
| GO:0051646 | mitochondrion localization | 24 | 0 | 0.91 | 1 |
| GO:0051653 | spindle localization | 31 | 0 | 1.18 | 1 |
| GO:0051654 | establishment of mitochondrion localizat... | 11 | 0 | 0.42 | 1 |

| | | | | | |
|---|---|---|---|---|---|
| GO:0051657 | maintenance of organelle location | 6 | 0 | 0.23 | 1 |
| GO:0051659 | maintenance of mitochondrion location | 2 | 0 | 0.08 | 1 |
| GO:0051660 | establishment of centrosome localization | 1 | 0 | 0.04 | 1 |
| GO:0051661 | maintenance of centrosome location | 2 | 0 | 0.08 | 1 |
| GO:0051665 | membrane raft localization | 5 | 0 | 0.19 | 1 |
| GO:0051673 | membrane disruption in other organism | 1 | 0 | 0.04 | 1 |
| GO:0051683 | establishment of Golgi localization | 5 | 0 | 0.19 | 1 |
| GO:0051684 | maintenance of Golgi location | 3 | 0 | 0.11 | 1 |
| GO:0051691 | cellular oligosaccharide metabolic proce... | 2 | 0 | 0.08 | 1 |
| GO:0051694 | pointed-end actin filament capping | 6 | 0 | 0.23 | 1 |
| GO:0051695 | actin filament uncapping | 1 | 0 | 0.04 | 1 |
| GO:0051697 | protein delipidation | 4 | 0 | 0.15 | 1 |
| GO:0051708 | intracellular protein transport in other... | 5 | 0 | 0.19 | 1 |
| GO:0051709 | regulation of killing of cells of other ... | 7 | 0 | 0.27 | 1 |
| GO:0051712 | positive regulation of killing of cells ... | 6 | 0 | 0.23 | 1 |
| GO:0051715 | cytolysis in other organism | 2 | 0 | 0.08 | 1 |
| GO:0051725 | protein de-ADP-ribosylation | 4 | 0 | 0.15 | 1 |
| GO:0051728 | cell cycle switching | mitotic to m | 1 | 0 | 0.04 |
| GO:0051729 | germline cell cycle switching | mitotic t... | 1 | 0 | 0.04 |
| GO:0051754 | meiotic sister chromatid cohesion | centr... | 1 | 0 | 0.04 |
| GO:0051758 | homologous chromosome movement towa | 1 | 0 | 0.04 | 1 |
| GO:0051764 | actin crosslink formation | 5 | 0 | 0.19 | 1 |
| GO:0051775 | response to redox state | 10 | 0 | 0.38 | 1 |
| GO:0051780 | behavioral response to nutrient | 1 | 0 | 0.04 | 1 |
| GO:0051788 | response to misfolded protein | 10 | 0 | 0.38 | 1 |
| GO:0051790 | short-chain fatty acid biosynthetic proc... | 4 | 0 | 0.15 | 1 |
| GO:0051791 | medium-chain fatty acid metabolic proces. | 4 | 0 | 0.15 | 1 |
| GO:0051792 | medium-chain fatty acid biosynthetic pro... | 2 | 0 | 0.08 | 1 |
| GO:0051793 | medium-chain fatty acid catabolic proces... | 1 | 0 | 0.04 | 1 |
| GO:0051794 | regulation of catagen | 4 | 0 | 0.15 | 1 |
| GO:0051795 | positive regulation of catagen | 3 | 0 | 0.11 | 1 |
| GO:0051796 | negative regulation of catagen | 1 | 0 | 0.04 | 1 |
| GO:0051797 | regulation of hair follicle development | 13 | 0 | 0.49 | 1 |
| GO:0051798 | positive regulation of hair follicle dev... | 9 | 0 | 0.34 | 1 |
| GO:0051799 | negative regulation of hair follicle dev... | 3 | 0 | 0.11 | 1 |
| GO:0051801 | cytolysis in other organism involved in ... | 2 | 0 | 0.08 | 1 |
| GO:0051805 | evasion or tolerance of immune response . | 1 | 0 | 0.04 | 1 |
| GO:0051807 | evasion or tolerance of defense response. | 1 | 0 | 0.04 | 1 |
| GO:0051808 | translocation of peptides or proteins in... | 1 | 0 | 0.04 | 1 |
| GO:0051818 | disruption of cells of other organism in... | 12 | 0 | 0.46 | 1 |
| GO:0051821 | dissemination or transmission of organis... | 1 | 0 | 0.04 | 1 |
| GO:0051823 | regulation of synapse structural plastic... | 8 | 0 | 0.3 | 1 |
| GO:0051832 | avoidance of defenses of other organism .. | 3 | 0 | 0.11 | 1 |
| GO:0051834 | evasion or tolerance of defenses of othe... | 3 | 0 | 0.11 | 1 |
| GO:0051835 | positive regulation of synapse structura... | 4 | 0 | 0.15 | 1 |
| GO:0051836 | translocation of molecules into other or... | 1 | 0 | 0.04 | 1 |
| GO:0051852 | disruption by host of symbiont cells | 8 | 0 | 0.3 | 1 |
| GO:0051866 | general adaptation syndrome | 2 | 0 | 0.08 | 1 |
| GO:0051867 | general adaptation syndrome | behavioral . | 1 | 0 | 0.04 |
| GO:0051873 | killing by host of symbiont cells | 8 | 0 | 0.3 | 1 |
| GO:0051875 | pigment granule localization | 27 | 0 | 1.03 | 1 |
| GO:0051877 | pigment granule aggregation in cell cent... | 2 | 0 | 0.08 | 1 |
| GO:0051878 | lateral element assembly | 1 | 0 | 0.04 | 1 |

| | | | | | |
|---|---|---|---|---|---|
| GO:0051883 | killing of cells in other organism invol... | 12 | 0 | 0.46 | 1 |
| GO:0051884 | regulation of anagen | 1 | 0 | 0.04 | 1 |
| GO:0051885 | positive regulation of anagen | 1 | 0 | 0.04 | 1 |
| GO:0051890 | regulation of cardioblast differentiatio... | 7 | 0 | 0.27 | 1 |
| GO:0051891 | positive regulation of cardioblast diffe... | 5 | 0 | 0.19 | 1 |
| GO:0051892 | negative regulation of cardioblast diffe... | 2 | 0 | 0.08 | 1 |
| GO:0051894 | positive regulation of focal adhesion as... | 19 | 0 | 0.72 | 1 |
| GO:0051901 | positive regulation of mitochondrial dep... | 8 | 0 | 0.3 | 1 |
| GO:0051902 | negative regulation of mitochondrial dep... | 4 | 0 | 0.15 | 1 |
| GO:0051904 | pigment granule transport | 23 | 0 | 0.88 | 1 |
| GO:0051905 | establishment of pigment granule localiz... | 24 | 0 | 0.91 | 1 |
| GO:0051917 | regulation of fibrinolysis | 14 | 0 | 0.53 | 1 |
| GO:0051918 | negative regulation of fibrinolysis | 10 | 0 | 0.38 | 1 |
| GO:0051919 | positive regulation of fibrinolysis | 4 | 0 | 0.15 | 1 |
| GO:0051923 | sulfation | 15 | 0 | 0.57 | 1 |
| GO:0051926 | negative regulation of calcium ion trans... | 41 | 0 | 1.56 | 1 |
| GO:0051933 | amino acid uptake involved in synaptic t... | 3 | 0 | 0.11 | 1 |
| GO:0051934 | catecholamine uptake involved in synapti.. | 11 | 0 | 0.42 | 1 |
| GO:0051935 | L-glutamate uptake involved in synaptic ... | 3 | 0 | 0.11 | 1 |
| GO:0051937 | catecholamine transport | 51 | 0 | 1.94 | 1 |
| GO:0051938 | L-glutamate import | 8 | 0 | 0.3 | 1 |
| GO:0051939 | gamma-aminobutyric acid import | 1 | 0 | 0.04 | 1 |
| GO:0051940 | regulation of catecholamine uptake invol... | 9 | 0 | 0.34 | 1 |
| GO:0051941 | regulation of amino acid uptake involved... | 1 | 0 | 0.04 | 1 |
| GO:0051944 | positive regulation of catecholamine upt... | 4 | 0 | 0.15 | 1 |
| GO:0051945 | negative regulation of catecholamine upt... | 1 | 0 | 0.04 | 1 |
| GO:0051946 | regulation of glutamate uptake involved ... | 1 | 0 | 0.04 | 1 |
| GO:0051954 | positive regulation of amine transport | 24 | 0 | 0.91 | 1 |
| GO:0051957 | positive regulation of amino acid transp... | 10 | 0 | 0.38 | 1 |
| GO:0051958 | methotrexate transport | 2 | 0 | 0.08 | 1 |
| GO:0051967 | negative regulation of synaptic transmis... | 9 | 0 | 0.34 | 1 |
| GO:0051969 | regulation of transmission of nerve impu... | 10 | 0 | 0.38 | 1 |
| GO:0051970 | negative regulation of transmission of n... | 4 | 0 | 0.15 | 1 |
| GO:0051971 | positive regulation of transmission of n... | 5 | 0 | 0.19 | 1 |
| GO:0051972 | regulation of telomerase activity | 12 | 0 | 0.46 | 1 |
| GO:0051973 | positive regulation of telomerase activi... | 6 | 0 | 0.23 | 1 |
| GO:0051974 | negative regulation of telomerase activi... | 7 | 0 | 0.27 | 1 |
| GO:0051977 | lysophospholipid transport | 1 | 0 | 0.04 | 1 |
| GO:0051987 | positive regulation of attachment of spi... | 4 | 0 | 0.15 | 1 |
| GO:0051988 | regulation of attachment of spindle micr... | 10 | 0 | 0.38 | 1 |
| GO:0052027 | modulation by symbiont of host signal tr... | 1 | 0 | 0.04 | 1 |
| GO:0052031 | modulation by symbiont of host defense r.. | 3 | 0 | 0.11 | 1 |
| GO:0052042 | positive regulation by symbiont of host ... | 2 | 0 | 0.08 | 1 |
| GO:0052063 | induction by symbiont of defense-related... | 1 | 0 | 0.04 | 1 |
| GO:0052066 | entry of symbiont into host cell by prom... | 1 | 0 | 0.04 | 1 |
| GO:0052097 | interspecies quorum sensing | 1 | 0 | 0.04 | 1 |
| GO:0052106 | quorum sensing involved in interaction w... | 1 | 0 | 0.04 | 1 |
| GO:0052151 | positive regulation by symbiont of host ... | 2 | 0 | 0.08 | 1 |
| GO:0052163 | modulation by symbiont of defense-relate.. | 1 | 0 | 0.04 | 1 |
| GO:0052173 | response to defenses of other organism i.. | 8 | 0 | 0.3 | 1 |
| GO:0052190 | modulation by symbiont of host phagocyto | 1 | 0 | 0.04 | 1 |
| GO:0052191 | positive regulation by symbiont of host ... | 1 | 0 | 0.04 | 1 |
| GO:0052199 | negative regulation of catalytic activit... | 2 | 0 | 0.08 | 1 |

| | | | | | |
|---|---|---|---|---|---|
| GO:0052200 | response to host defenses | 8 | 0 | 0.3 | 1 |
| GO:0052203 | modulation of catalytic activity in othe... | 2 | 0 | 0.08 | 1 |
| GO:0052204 | negative regulation of molecular functio... | 2 | 0 | 0.08 | 1 |
| GO:0052205 | modulation of molecular function in othe... | 2 | 0 | 0.08 | 1 |
| GO:0052231 | modulation of phagocytosis in other orga... | 1 | 0 | 0.04 | 1 |
| GO:0052250 | modulation of signal transduction in oth... | 1 | 0 | 0.04 | 1 |
| GO:0052251 | induction by organism of defense respons. | 3 | 0 | 0.11 | 1 |
| GO:0052255 | modulation by organism of defense respor | 3 | 0 | 0.11 | 1 |
| GO:0052263 | induction by organism of defense-related.. | 1 | 0 | 0.04 | 1 |
| GO:0052302 | modulation by organism of defense-relate. | 1 | 0 | 0.04 | 1 |
| GO:0052314 | phytoalexin metabolic process | 2 | 0 | 0.08 | 1 |
| GO:0052330 | positive regulation by organism of progr... | 2 | 0 | 0.08 | 1 |
| GO:0052331 | hemolysis in other organism involved in ... | 2 | 0 | 0.08 | 1 |
| GO:0052345 | positive regulation by organism of defen... | 1 | 0 | 0.04 | 1 |
| GO:0052347 | positive regulation by symbiont of defen... | 1 | 0 | 0.04 | 1 |
| GO:0052370 | entry of organism into cell of other org... | 1 | 0 | 0.04 | 1 |
| GO:0052403 | negative regulation by host of symbiont ... | 2 | 0 | 0.08 | 1 |
| GO:0052405 | negative regulation by host of symbiont ... | 2 | 0 | 0.08 | 1 |
| GO:0052422 | modulation by host of symbiont catalytic... | 2 | 0 | 0.08 | 1 |
| GO:0052428 | modification by host of symbiont molecul... | 2 | 0 | 0.08 | 1 |
| GO:0052501 | positive regulation by organism of apopt... | 2 | 0 | 0.08 | 1 |
| GO:0052509 | positive regulation by symbiont of host ... | 3 | 0 | 0.11 | 1 |
| GO:0052510 | positive regulation by organism of defen... | 3 | 0 | 0.11 | 1 |
| GO:0052522 | positive regulation by organism of phago... | 1 | 0 | 0.04 | 1 |
| GO:0052550 | response to defense-related reactive oxy... | 1 | 0 | 0.04 | 1 |
| GO:0052551 | response to defense-related nitric oxide... | 2 | 0 | 0.08 | 1 |
| GO:0052552 | modulation by organism of immune respor | 3 | 0 | 0.11 | 1 |
| GO:0052553 | modulation by symbiont of host immune re | 3 | 0 | 0.11 | 1 |
| GO:0052555 | positive regulation by organism of immun.. | 1 | 0 | 0.04 | 1 |
| GO:0052556 | positive regulation by symbiont of host ... | 1 | 0 | 0.04 | 1 |
| GO:0052558 | induction by organism of immune response | 1 | 0 | 0.04 | 1 |
| GO:0052559 | induction by symbiont of host immune res. | 1 | 0 | 0.04 | 1 |
| GO:0052564 | response to immune response of other org | 7 | 0 | 0.27 | 1 |
| GO:0052565 | response to defense-related host nitric ... | 2 | 0 | 0.08 | 1 |
| GO:0052567 | response to defense-related host reactiv... | 1 | 0 | 0.04 | 1 |
| GO:0052572 | response to host immune response | 7 | 0 | 0.27 | 1 |
| GO:0052697 | xenobiotic glucuronidation | 9 | 0 | 0.34 | 1 |
| GO:0052746 | inositol phosphorylation | 1 | 0 | 0.04 | 1 |
| GO:0052803 | imidazole-containing compound metabolic | 9 | 0 | 0.34 | 1 |
| GO:0052805 | imidazole-containing compound catabolic . | 5 | 0 | 0.19 | 1 |
| GO:0055003 | cardiac myofibril assembly | 15 | 0 | 0.57 | 1 |
| GO:0055005 | ventricular cardiac myofibril assembly | 3 | 0 | 0.11 | 1 |
| GO:0055009 | atrial cardiac muscle tissue morphogenes. | 6 | 0 | 0.23 | 1 |
| GO:0055011 | atrial cardiac muscle cell differentiati... | 2 | 0 | 0.08 | 1 |
| GO:0055014 | atrial cardiac muscle cell development | 2 | 0 | 0.08 | 1 |
| GO:0055015 | ventricular cardiac muscle cell developm... | 11 | 0 | 0.42 | 1 |
| GO:0055018 | regulation of cardiac muscle fiber devel... | 1 | 0 | 0.04 | 1 |
| GO:0055020 | positive regulation of cardiac muscle fi... | 1 | 0 | 0.04 | 1 |
| GO:0055022 | negative regulation of cardiac muscle ti... | 11 | 0 | 0.42 | 1 |
| GO:0055059 | asymmetric neuroblast division | 3 | 0 | 0.11 | 1 |
| GO:0055064 | chloride ion homeostasis | 6 | 0 | 0.23 | 1 |
| GO:0055069 | zinc ion homeostasis | 20 | 0 | 0.76 | 1 |
| GO:0055070 | copper ion homeostasis | 17 | 0 | 0.65 | 1 |

| | | | | | |
|---|---|---|---|---|---|
| GO:0055071 | manganese ion homeostasis | 1 | 0 | 0.04 | 1 |
| GO:0055073 | cadmium ion homeostasis | 2 | 0 | 0.08 | 1 |
| GO:0055090 | acylglycerol homeostasis | 28 | 0 | 1.07 | 1 |
| GO:0055091 | phospholipid homeostasis | 10 | 0 | 0.38 | 1 |
| GO:0055095 | lipoprotein particle mediated signaling | 2 | 0 | 0.08 | 1 |
| GO:0055096 | low-density lipoprotein particle mediate... | 2 | 0 | 0.08 | 1 |
| GO:0055098 | response to low-density lipoprotein part... | 6 | 0 | 0.23 | 1 |
| GO:0055107 | Golgi to secretory granule transport | 1 | 0 | 0.04 | 1 |
| GO:0055113 | epiboly involved in gastrulation with mo... | 1 | 0 | 0.04 | 1 |
| GO:0055118 | negative regulation of cardiac muscle co... | 3 | 0 | 0.11 | 1 |
| GO:0055129 | L-proline biosynthetic process | 5 | 0 | 0.19 | 1 |
| GO:0055130 | D-alanine catabolic process | 1 | 0 | 0.04 | 1 |
| GO:0060003 | copper ion export | 2 | 0 | 0.08 | 1 |
| GO:0060004 | reflex | 19 | 0 | 0.72 | 1 |
| GO:0060005 | vestibular reflex | 4 | 0 | 0.15 | 1 |
| GO:0060007 | linear vestibuloocular reflex | 1 | 0 | 0.04 | 1 |
| GO:0060010 | Sertoli cell fate commitment | 1 | 0 | 0.04 | 1 |
| GO:0060011 | Sertoli cell proliferation | 4 | 0 | 0.15 | 1 |
| GO:0060012 | synaptic transmission | glycinergic | 3 | 0 | 0.11 |
| GO:0060013 | righting reflex | 7 | 0 | 0.27 | 1 |
| GO:0060014 | granulosa cell differentiation | 3 | 0 | 0.11 | 1 |
| GO:0060016 | granulosa cell development | 1 | 0 | 0.04 | 1 |
| GO:0060017 | parathyroid gland development | 7 | 0 | 0.27 | 1 |
| GO:0060018 | astrocyte fate commitment | 4 | 0 | 0.15 | 1 |
| GO:0060020 | Bergmann glial cell differentiation | 6 | 0 | 0.23 | 1 |
| GO:0060022 | hard palate development | 6 | 0 | 0.23 | 1 |
| GO:0060023 | soft palate development | 5 | 0 | 0.19 | 1 |
| GO:0060025 | regulation of synaptic activity | 1 | 0 | 0.04 | 1 |
| GO:0060027 | convergent extension involved in gastrul... | 2 | 0 | 0.08 | 1 |
| GO:0060028 | convergent extension involved in axis el... | 6 | 0 | 0.23 | 1 |
| GO:0060029 | convergent extension involved in organog. | 6 | 0 | 0.23 | 1 |
| GO:0060032 | notochord regression | 2 | 0 | 0.08 | 1 |
| GO:0060033 | anatomical structure regression | 13 | 0 | 0.49 | 1 |
| GO:0060034 | notochord cell differentiation | 1 | 0 | 0.04 | 1 |
| GO:0060035 | notochord cell development | 1 | 0 | 0.04 | 1 |
| GO:0060037 | pharyngeal system development | 15 | 0 | 0.57 | 1 |
| GO:0060040 | retinal bipolar neuron differentiation | 4 | 0 | 0.15 | 1 |
| GO:0060043 | regulation of cardiac muscle cell prolif... | 27 | 0 | 1.03 | 1 |
| GO:0060044 | negative regulation of cardiac muscle ce... | 8 | 0 | 0.3 | 1 |
| GO:0060045 | positive regulation of cardiac muscle ce... | 18 | 0 | 0.69 | 1 |
| GO:0060046 | regulation of acrosome reaction | 10 | 0 | 0.38 | 1 |
| GO:0060050 | positive regulation of protein glycosyla... | 2 | 0 | 0.08 | 1 |
| GO:0060051 | negative regulation of protein glycosyla... | 4 | 0 | 0.15 | 1 |
| GO:0060055 | angiogenesis involved in wound healing | 14 | 0 | 0.53 | 1 |
| GO:0060056 | mammary gland involution | 9 | 0 | 0.34 | 1 |
| GO:0060057 | apoptotic process involved in mammary gl. | 4 | 0 | 0.15 | 1 |
| GO:0060058 | positive regulation of apoptotic process... | 4 | 0 | 0.15 | 1 |
| GO:0060060 | post-embryonic retina morphogenesis in c. | 3 | 0 | 0.11 | 1 |
| GO:0060061 | Spemann organizer formation | 3 | 0 | 0.11 | 1 |
| GO:0060064 | Spemann organizer formation at the anter. | 1 | 0 | 0.04 | 1 |
| GO:0060067 | cervix development | 2 | 0 | 0.08 | 1 |
| GO:0060073 | micturition | 7 | 0 | 0.27 | 1 |
| GO:0060083 | smooth muscle contraction involved in mi.. | 3 | 0 | 0.11 | 1 |

| | | | | | |
|---|---|---|---|---|---|
| GO:0060084 | synaptic transmission involved in mictur... | 3 | 0 | 0.11 | 1 |
| GO:0060086 | circadian temperature homeostasis | 2 | 0 | 0.08 | 1 |
| GO:0060088 | auditory receptor cell stereocilium orga... | 9 | 0 | 0.34 | 1 |
| GO:0060096 | serotonin secretion | neurotransr | 1 | 0 | 0.04 |
| GO:0060099 | regulation of phagocytosis | engulfment | 8 | 0 | 0.3 |
| GO:0060100 | positive regulation of phagocytosis | eng... | 6 | 0 | 0.23 |
| GO:0060101 | negative regulation of phagocytosis | eng... | 1 | 0 | 0.04 |
| GO:0060112 | generation of ovulation cycle rhythm | 1 | 0 | 0.04 | 1 |
| GO:0060113 | inner ear receptor cell differentiation | 48 | 0 | 1.83 | 1 |
| GO:0060117 | auditory receptor cell development | 15 | 0 | 0.57 | 1 |
| GO:0060119 | inner ear receptor cell development | 27 | 0 | 1.03 | 1 |
| GO:0060120 | inner ear receptor cell fate commitment | 6 | 0 | 0.23 | 1 |
| GO:0060122 | inner ear receptor stereocilium organiza... | 14 | 0 | 0.53 | 1 |
| GO:0060124 | positive regulation of growth hormone se... | 7 | 0 | 0.27 | 1 |
| GO:0060125 | negative regulation of growth hormone se. | 1 | 0 | 0.04 | 1 |
| GO:0060126 | somatotropin secreting cell differentiat... | 5 | 0 | 0.19 | 1 |
| GO:0060127 | prolactin secreting cell differentiation | 1 | 0 | 0.04 | 1 |
| GO:0060128 | corticotropin hormone secreting cell dif... | 2 | 0 | 0.08 | 1 |
| GO:0060129 | thyroid-stimulating hormone-secreting ce... | 3 | 0 | 0.11 | 1 |
| GO:0060133 | somatotropin secreting cell development | 2 | 0 | 0.08 | 1 |
| GO:0060138 | fetal process involved in parturition | 1 | 0 | 0.04 | 1 |
| GO:0060139 | positive regulation of apoptotic process... | 2 | 0 | 0.08 | 1 |
| GO:0060142 | regulation of syncytium formation by pla... | 22 | 0 | 0.84 | 1 |
| GO:0060143 | positive regulation of syncytium formati... | 18 | 0 | 0.69 | 1 |
| GO:0060148 | positive regulation of posttranscription... | 4 | 0 | 0.15 | 1 |
| GO:0060151 | peroxisome localization | 2 | 0 | 0.08 | 1 |
| GO:0060152 | microtubule-based peroxisome localizatio.. | 2 | 0 | 0.08 | 1 |
| GO:0060154 | cellular process regulating host cell cy... | 3 | 0 | 0.11 | 1 |
| GO:0060155 | platelet dense granule organization | 8 | 0 | 0.3 | 1 |
| GO:0060156 | milk ejection | 1 | 0 | 0.04 | 1 |
| GO:0060157 | urinary bladder development | 5 | 0 | 0.19 | 1 |
| GO:0060158 | phospholipase C-activating dopamine rece | 9 | 0 | 0.34 | 1 |
| GO:0060160 | negative regulation of dopamine receptor.. | 3 | 0 | 0.11 | 1 |
| GO:0060163 | subpallium neuron fate commitment | 2 | 0 | 0.08 | 1 |
| GO:0060164 | regulation of timing of neuron different... | 4 | 0 | 0.15 | 1 |
| GO:0060165 | regulation of timing of subpallium neuro... | 1 | 0 | 0.04 | 1 |
| GO:0060166 | olfactory pit development | 3 | 0 | 0.11 | 1 |
| GO:0060167 | regulation of adenosine receptor signali... | 3 | 0 | 0.11 | 1 |
| GO:0060168 | positive regulation of adenosine recepto... | 2 | 0 | 0.08 | 1 |
| GO:0060169 | negative regulation of adenosine recepto... | 1 | 0 | 0.04 | 1 |
| GO:0060174 | limb bud formation | 10 | 0 | 0.38 | 1 |
| GO:0060177 | regulation of angiotensin metabolic proc... | 13 | 0 | 0.49 | 1 |
| GO:0060178 | regulation of exocyst localization | 2 | 0 | 0.08 | 1 |
| GO:0060184 | cell cycle switching | 1 | 0 | 0.04 | 1 |
| GO:0060192 | negative regulation of lipase activity | 15 | 0 | 0.57 | 1 |
| GO:0060197 | cloacal septation | 3 | 0 | 0.11 | 1 |
| GO:0060211 | regulation of nuclear-transcribed mRNA p.. | 10 | 0 | 0.38 | 1 |
| GO:0060212 | negative regulation of nuclear-transcrib... | 1 | 0 | 0.04 | 1 |
| GO:0060213 | positive regulation of nuclear-transcrib... | 10 | 0 | 0.38 | 1 |
| GO:0060215 | primitive hemopoiesis | 6 | 0 | 0.23 | 1 |
| GO:0060216 | definitive hemopoiesis | 22 | 0 | 0.84 | 1 |
| GO:0060217 | hemangioblast cell differentiation | 1 | 0 | 0.04 | 1 |
| GO:0060220 | camera-type eye photoreceptor cell fate ... | 1 | 0 | 0.04 | 1 |

| | | | | | |
|---|---|---|---|---|---|
| GO:0060221 | retinal rod cell differentiation | 2 | 0 | 0.08 | 1 |
| GO:0060231 | mesenchymal to epithelial transition | 20 | 0 | 0.76 | 1 |
| GO:0060235 | lens induction in camera-type eye | 7 | 0 | 0.27 | 1 |
| GO:0060236 | regulation of mitotic spindle organizati... | 16 | 0 | 0.61 | 1 |
| GO:0060242 | contact inhibition | 8 | 0 | 0.3 | 1 |
| GO:0060244 | negative regulation of cell proliferatio... | 4 | 0 | 0.15 | 1 |
| GO:0060245 | detection of cell density | 1 | 0 | 0.04 | 1 |
| GO:0060246 | detection of cell density by contact sti... | 1 | 0 | 0.04 | 1 |
| GO:0060248 | detection of cell density by contact sti... | 1 | 0 | 0.04 | 1 |
| GO:0060253 | negative regulation of glial cell prolif... | 9 | 0 | 0.34 | 1 |
| GO:0060254 | regulation of N-terminal protein palmito... | 1 | 0 | 0.04 | 1 |
| GO:0060259 | regulation of feeding behavior | 14 | 0 | 0.53 | 1 |
| GO:0060262 | negative regulation of N-terminal protei... | 1 | 0 | 0.04 | 1 |
| GO:0060263 | regulation of respiratory burst | 11 | 0 | 0.42 | 1 |
| GO:0060264 | regulation of respiratory burst involved... | 4 | 0 | 0.15 | 1 |
| GO:0060265 | positive regulation of respiratory burst... | 2 | 0 | 0.08 | 1 |
| GO:0060266 | negative regulation of respiratory burst... | 3 | 0 | 0.11 | 1 |
| GO:0060267 | positive regulation of respiratory burst | 6 | 0 | 0.23 | 1 |
| GO:0060268 | negative regulation of respiratory burst | 3 | 0 | 0.11 | 1 |
| GO:0060272 | embryonic skeletal joint morphogenesis | 13 | 0 | 0.49 | 1 |
| GO:0060278 | regulation of ovulation | 4 | 0 | 0.15 | 1 |
| GO:0060279 | positive regulation of ovulation | 3 | 0 | 0.11 | 1 |
| GO:0060280 | negative regulation of ovulation | 1 | 0 | 0.04 | 1 |
| GO:0060281 | regulation of oocyte development | 8 | 0 | 0.3 | 1 |
| GO:0060282 | positive regulation of oocyte developmen.. | 4 | 0 | 0.15 | 1 |
| GO:0060283 | negative regulation of oocyte developmen. | 4 | 0 | 0.15 | 1 |
| GO:0060285 | cilium-dependent cell motility | 6 | 0 | 0.23 | 1 |
| GO:0060290 | transdifferentiation | 6 | 0 | 0.23 | 1 |
| GO:0060294 | cilium movement involved in cell motilit... | 4 | 0 | 0.15 | 1 |
| GO:0060295 | regulation of cilium movement involved i... | 4 | 0 | 0.15 | 1 |
| GO:0060296 | regulation of cilium beat frequency invo... | 4 | 0 | 0.15 | 1 |
| GO:0060299 | negative regulation of sarcomere organiz.. | 1 | 0 | 0.04 | 1 |
| GO:0060300 | regulation of cytokine activity | 4 | 0 | 0.15 | 1 |
| GO:0060301 | positive regulation of cytokine activity | 1 | 0 | 0.04 | 1 |
| GO:0060302 | negative regulation of cytokine activity | 2 | 0 | 0.08 | 1 |
| GO:0060304 | regulation of phosphatidylinositol depho... | 1 | 0 | 0.04 | 1 |
| GO:0060305 | regulation of cell diameter | 1 | 0 | 0.04 | 1 |
| GO:0060307 | regulation of ventricular cardiac muscle... | 14 | 0 | 0.53 | 1 |
| GO:0060309 | elastin catabolic process | 1 | 0 | 0.04 | 1 |
| GO:0060310 | regulation of elastin catabolic process | 1 | 0 | 0.04 | 1 |
| GO:0060311 | negative regulation of elastin catabolic... | 1 | 0 | 0.04 | 1 |
| GO:0060312 | regulation of blood vessel remodeling | 5 | 0 | 0.19 | 1 |
| GO:0060313 | negative regulation of blood vessel remo... | 1 | 0 | 0.04 | 1 |
| GO:0060315 | negative regulation of ryanodine-sensiti... | 11 | 0 | 0.42 | 1 |
| GO:0060316 | positive regulation of ryanodine-sensiti... | 10 | 0 | 0.38 | 1 |
| GO:0060318 | definitive erythrocyte differentiation | 6 | 0 | 0.23 | 1 |
| GO:0060319 | primitive erythrocyte differentiation | 2 | 0 | 0.08 | 1 |
| GO:0060327 | cytoplasmic actin-based contraction invo... | 1 | 0 | 0.04 | 1 |
| GO:0060332 | positive regulation of response to inter... | 4 | 0 | 0.15 | 1 |
| GO:0060335 | positive regulation of interferon-gamma-... | 4 | 0 | 0.15 | 1 |
| GO:0060340 | positive regulation of type I interferon... | 7 | 0 | 0.27 | 1 |
| GO:0060345 | spleen trabecula formation | 1 | 0 | 0.04 | 1 |
| GO:0060352 | cell adhesion molecule production | 4 | 0 | 0.15 | 1 |

| | | | | | |
|---|---|---|---|---|---|
| GO:0060353 | regulation of cell adhesion molecule pro... | 2 | 0 | 0.08 | 1 |
| GO:0060354 | negative regulation of cell adhesion mol... | 1 | 0 | 0.04 | 1 |
| GO:0060355 | positive regulation of cell adhesion mol... | 1 | 0 | 0.04 | 1 |
| GO:0060356 | leucine import | 1 | 0 | 0.04 | 1 |
| GO:0060363 | cranial suture morphogenesis | 10 | 0 | 0.38 | 1 |
| GO:0060364 | frontal suture morphogenesis | 4 | 0 | 0.15 | 1 |
| GO:0060365 | coronal suture morphogenesis | 1 | 0 | 0.04 | 1 |
| GO:0060366 | lambdoid suture morphogenesis | 1 | 0 | 0.04 | 1 |
| GO:0060367 | sagittal suture morphogenesis | 1 | 0 | 0.04 | 1 |
| GO:0060369 | positive regulation of Fc receptor media... | 2 | 0 | 0.08 | 1 |
| GO:0060370 | susceptibility to T cell mediated cytoto... | 2 | 0 | 0.08 | 1 |
| GO:0060371 | regulation of atrial cardiac muscle cell... | 8 | 0 | 0.3 | 1 |
| GO:0060372 | regulation of atrial cardiac muscle cell... | 5 | 0 | 0.19 | 1 |
| GO:0060373 | regulation of ventricular cardiac muscle... | 7 | 0 | 0.27 | 1 |
| GO:0060374 | mast cell differentiation | 6 | 0 | 0.23 | 1 |
| GO:0060375 | regulation of mast cell differentiation | 3 | 0 | 0.11 | 1 |
| GO:0060376 | positive regulation of mast cell differe... | 1 | 0 | 0.04 | 1 |
| GO:0060377 | negative regulation of mast cell differe... | 1 | 0 | 0.04 | 1 |
| GO:0060379 | cardiac muscle cell myoblast differentia... | 11 | 0 | 0.42 | 1 |
| GO:0060380 | regulation of single-stranded telomeric ... | 1 | 0 | 0.04 | 1 |
| GO:0060381 | positive regulation of single-stranded t... | 1 | 0 | 0.04 | 1 |
| GO:0060382 | regulation of DNA strand elongation | 1 | 0 | 0.04 | 1 |
| GO:0060383 | positive regulation of DNA strand elonga... | 1 | 0 | 0.04 | 1 |
| GO:0060386 | synapse assembly involved in innervation | 1 | 0 | 0.04 | 1 |
| GO:0060390 | regulation of SMAD protein import into n... | 14 | 0 | 0.53 | 1 |
| GO:0060391 | positive regulation of SMAD protein impo.. | 11 | 0 | 0.42 | 1 |
| GO:0060392 | negative regulation of SMAD protein impo. | 2 | 0 | 0.08 | 1 |
| GO:0060394 | negative regulation of pathway-restricte... | 10 | 0 | 0.38 | 1 |
| GO:0060398 | regulation of growth hormone receptor si... | 5 | 0 | 0.19 | 1 |
| GO:0060399 | positive regulation of growth hormone re... | 3 | 0 | 0.11 | 1 |
| GO:0060400 | negative regulation of growth hormone re.. | 1 | 0 | 0.04 | 1 |
| GO:0060404 | axonemal microtubule depolymerization | 1 | 0 | 0.04 | 1 |
| GO:0060405 | regulation of penile erection | 8 | 0 | 0.3 | 1 |
| GO:0060406 | positive regulation of penile erection | 6 | 0 | 0.23 | 1 |
| GO:0060407 | negative regulation of penile erection | 1 | 0 | 0.04 | 1 |
| GO:0060413 | atrial septum morphogenesis | 14 | 0 | 0.53 | 1 |
| GO:0060423 | foregut regionalization | 3 | 0 | 0.11 | 1 |
| GO:0060424 | lung field specification | 3 | 0 | 0.11 | 1 |
| GO:0060426 | lung vasculature development | 8 | 0 | 0.3 | 1 |
| GO:0060428 | lung epithelium development | 40 | 0 | 1.52 | 1 |
| GO:0060430 | lung saccule development | 8 | 0 | 0.3 | 1 |
| GO:0060432 | lung pattern specification process | 1 | 0 | 0.04 | 1 |
| GO:0060433 | bronchus development | 9 | 0 | 0.34 | 1 |
| GO:0060434 | bronchus morphogenesis | 2 | 0 | 0.08 | 1 |
| GO:0060435 | bronchiole development | 3 | 0 | 0.11 | 1 |
| GO:0060436 | bronchiole morphogenesis | 1 | 0 | 0.04 | 1 |
| GO:0060437 | lung growth | 4 | 0 | 0.15 | 1 |
| GO:0060440 | trachea formation | 3 | 0 | 0.11 | 1 |
| GO:0060446 | branching involved in open tracheal syst... | 1 | 0 | 0.04 | 1 |
| GO:0060447 | bud outgrowth involved in lung branching | 4 | 0 | 0.15 | 1 |
| GO:0060448 | dichotomous subdivision of terminal unit... | 3 | 0 | 0.11 | 1 |
| GO:0060450 | positive regulation of hindgut contracti... | 1 | 0 | 0.04 | 1 |
| GO:0060452 | positive regulation of cardiac muscle co... | 7 | 0 | 0.27 | 1 |

| | | | | | |
|---|---|---|---|---|---|
| GO:0060453 | regulation of gastric acid secretion | 5 | 0 | 0.19 | 1 |
| GO:0060455 | negative regulation of gastric acid secr... | 4 | 0 | 0.15 | 1 |
| GO:0060457 | negative regulation of digestive system ... | 8 | 0 | 0.3 | 1 |
| GO:0060458 | right lung development | 2 | 0 | 0.08 | 1 |
| GO:0060459 | left lung development | 3 | 0 | 0.11 | 1 |
| GO:0060460 | left lung morphogenesis | 2 | 0 | 0.08 | 1 |
| GO:0060461 | right lung morphogenesis | 1 | 0 | 0.04 | 1 |
| GO:0060462 | lung lobe development | 9 | 0 | 0.34 | 1 |
| GO:0060463 | lung lobe morphogenesis | 9 | 0 | 0.34 | 1 |
| GO:0060464 | lung lobe formation | 1 | 0 | 0.04 | 1 |
| GO:0060465 | pharynx development | 2 | 0 | 0.08 | 1 |
| GO:0060466 | activation of meiosis involved in egg ac... | 2 | 0 | 0.08 | 1 |
| GO:0060467 | negative regulation of fertilization | 6 | 0 | 0.23 | 1 |
| GO:0060468 | prevention of polyspermy | 3 | 0 | 0.11 | 1 |
| GO:0060478 | acrosomal vesicle exocytosis | 3 | 0 | 0.11 | 1 |
| GO:0060479 | lung cell differentiation | 29 | 0 | 1.1 | 1 |
| GO:0060480 | lung goblet cell differentiation | 5 | 0 | 0.19 | 1 |
| GO:0060481 | lobar bronchus epithelium development | 5 | 0 | 0.19 | 1 |
| GO:0060482 | lobar bronchus development | 6 | 0 | 0.23 | 1 |
| GO:0060484 | lung-associated mesenchyme developmer | 11 | 0 | 0.42 | 1 |
| GO:0060486 | Clara cell differentiation | 4 | 0 | 0.15 | 1 |
| GO:0060487 | lung epithelial cell differentiation | 28 | 0 | 1.07 | 1 |
| GO:0060488 | orthogonal dichotomous subdivision of te.. | 2 | 0 | 0.08 | 1 |
| GO:0060489 | planar dichotomous subdivision of termin.. | 2 | 0 | 0.08 | 1 |
| GO:0060490 | lateral sprouting involved in lung morph... | 2 | 0 | 0.08 | 1 |
| GO:0060492 | lung induction | 3 | 0 | 0.11 | 1 |
| GO:0060495 | cell-cell signaling involved in lung dev... | 1 | 0 | 0.04 | 1 |
| GO:0060496 | mesenchymal-epithelial cell signaling in... | 1 | 0 | 0.04 | 1 |
| GO:0060501 | positive regulation of epithelial cell p... | 4 | 0 | 0.15 | 1 |
| GO:0060502 | epithelial cell proliferation involved i... | 7 | 0 | 0.27 | 1 |
| GO:0060503 | bud dilation involved in lung branching | 1 | 0 | 0.04 | 1 |
| GO:0060509 | Type I pneumocyte differentiation | 6 | 0 | 0.23 | 1 |
| GO:0060510 | Type II pneumocyte differentiation | 6 | 0 | 0.23 | 1 |
| GO:0060513 | prostatic bud formation | 9 | 0 | 0.34 | 1 |
| GO:0060516 | primary prostatic bud elongation | 2 | 0 | 0.08 | 1 |
| GO:0060517 | epithelial cell proliferation involved i... | 1 | 0 | 0.04 | 1 |
| GO:0060523 | prostate epithelial cord elongation | 3 | 0 | 0.11 | 1 |
| GO:0060528 | secretory columnal luminar epithelial ce... | 4 | 0 | 0.15 | 1 |
| GO:0060529 | squamous basal epithelial stem cell diff... | 2 | 0 | 0.08 | 1 |
| GO:0060539 | diaphragm development | 9 | 0 | 0.34 | 1 |
| GO:0060544 | regulation of necroptotic process | 11 | 0 | 0.42 | 1 |
| GO:0060545 | positive regulation of necroptotic proce... | 2 | 0 | 0.08 | 1 |
| GO:0060546 | negative regulation of necroptotic proce... | 8 | 0 | 0.3 | 1 |
| GO:0060547 | negative regulation of necrotic cell dea... | 10 | 0 | 0.38 | 1 |
| GO:0060549 | regulation of fructose 1 | 6-bisphospl | 1 | 0 | 0.04 |
| GO:0060550 | positive regulation of fructose 1 | 6-bisp... | 1 | 0 | 0.04 |
| GO:0060551 | regulation of fructose 1 | 6-bisphospl | 1 | 0 | 0.04 |
| GO:0060552 | positive regulation of fructose 1 | 6-bisp... | 1 | 0 | 0.04 |
| GO:0060556 | regulation of vitamin D biosynthetic pro... | 9 | 0 | 0.34 | 1 |
| GO:0060557 | positive regulation of vitamin D biosynt... | 3 | 0 | 0.11 | 1 |
| GO:0060558 | regulation of calcidiol 1-monooxygenase .. | 7 | 0 | 0.27 | 1 |
| GO:0060559 | positive regulation of calcidiol 1-monoo... | 3 | 0 | 0.11 | 1 |
| GO:0060563 | neuroepithelial cell differentiation | 69 | 0 | 2.63 | 1 |

| | | | | | |
|---|---|---|---|---|---|
| GO:0060565 | inhibition of APC-Cdc20 complex activity | 1 | 0 | 0.04 | 1 |
| GO:0060566 | positive regulation of DNA-templated tra... | 1 | 0 | 0.04 | 1 |
| GO:0060567 | negative regulation of DNA-templated tra.. | 1 | 0 | 0.04 | 1 |
| GO:0060573 | cell fate specification involved in patt... | 11 | 0 | 0.42 | 1 |
| GO:0060574 | intestinal epithelial cell maturation | 4 | 0 | 0.15 | 1 |
| GO:0060577 | pulmonary vein morphogenesis | 2 | 0 | 0.08 | 1 |
| GO:0060578 | superior vena cava morphogenesis | 1 | 0 | 0.04 | 1 |
| GO:0060579 | ventral spinal cord interneuron fate com... | 15 | 0 | 0.57 | 1 |
| GO:0060580 | ventral spinal cord interneuron fate det... | 1 | 0 | 0.04 | 1 |
| GO:0060581 | cell fate commitment involved in pattern... | 15 | 0 | 0.57 | 1 |
| GO:0060582 | cell fate determination involved in patt... | 1 | 0 | 0.04 | 1 |
| GO:0060586 | multicellular organismal iron ion homeos... | 7 | 0 | 0.27 | 1 |
| GO:0060588 | negative regulation of lipoprotein lipid... | 1 | 0 | 0.04 | 1 |
| GO:0060591 | chondroblast differentiation | 4 | 0 | 0.15 | 1 |
| GO:0060594 | mammary gland specification | 4 | 0 | 0.15 | 1 |
| GO:0060595 | fibroblast growth factor receptor signal... | 2 | 0 | 0.08 | 1 |
| GO:0060599 | lateral sprouting involved in mammary gl... | 1 | 0 | 0.04 | 1 |
| GO:0060601 | lateral sprouting from an epithelium | 12 | 0 | 0.46 | 1 |
| GO:0060605 | tube lumen cavitation | 5 | 0 | 0.19 | 1 |
| GO:0060611 | mammary gland fat development | 1 | 0 | 0.04 | 1 |
| GO:0060613 | fat pad development | 3 | 0 | 0.11 | 1 |
| GO:0060615 | mammary gland bud formation | 2 | 0 | 0.08 | 1 |
| GO:0060618 | nipple development | 1 | 0 | 0.04 | 1 |
| GO:0060620 | regulation of cholesterol import | 2 | 0 | 0.08 | 1 |
| GO:0060621 | negative regulation of cholesterol impor... | 2 | 0 | 0.08 | 1 |
| GO:0060623 | regulation of chromosome condensation | 2 | 0 | 0.08 | 1 |
| GO:0060629 | regulation of homologous chromosome se | 1 | 0 | 0.04 | 1 |
| GO:0060632 | regulation of microtubule-based movemen | 11 | 0 | 0.42 | 1 |
| GO:0060633 | negative regulation of transcription ini... | 4 | 0 | 0.15 | 1 |
| GO:0060644 | mammary gland epithelial cell differenti... | 16 | 0 | 0.61 | 1 |
| GO:0060648 | mammary gland bud morphogenesis | 4 | 0 | 0.15 | 1 |
| GO:0060649 | mammary gland bud elongation | 1 | 0 | 0.04 | 1 |
| GO:0060658 | nipple morphogenesis | 1 | 0 | 0.04 | 1 |
| GO:0060659 | nipple sheath formation | 1 | 0 | 0.04 | 1 |
| GO:0060661 | submandibular salivary gland formation | 2 | 0 | 0.08 | 1 |
| GO:0060662 | salivary gland cavitation | 5 | 0 | 0.19 | 1 |
| GO:0060664 | epithelial cell proliferation involved i... | 7 | 0 | 0.27 | 1 |
| GO:0060666 | dichotomous subdivision of terminal unit... | 5 | 0 | 0.19 | 1 |
| GO:0060667 | branch elongation involved in salivary g... | 2 | 0 | 0.08 | 1 |
| GO:0060668 | regulation of branching involved in sali... | 1 | 0 | 0.04 | 1 |
| GO:0060670 | branching involved in labyrinthine layer... | 9 | 0 | 0.34 | 1 |
| GO:0060675 | ureteric bud morphogenesis | 65 | 0 | 2.47 | 1 |
| GO:0060676 | ureteric bud formation | 6 | 0 | 0.23 | 1 |
| GO:0060677 | ureteric bud elongation | 7 | 0 | 0.27 | 1 |
| GO:0060678 | dichotomous subdivision of terminal unit... | 1 | 0 | 0.04 | 1 |
| GO:0060681 | branch elongation involved in ureteric b... | 5 | 0 | 0.19 | 1 |
| GO:0060683 | regulation of branching involved in sali... | 1 | 0 | 0.04 | 1 |
| GO:0060684 | epithelial-mesenchymal cell signaling | 7 | 0 | 0.27 | 1 |
| GO:0060685 | regulation of prostatic bud formation | 5 | 0 | 0.19 | 1 |
| GO:0060686 | negative regulation of prostatic bud for... | 4 | 0 | 0.15 | 1 |
| GO:0060689 | cell differentiation involved in salivar... | 2 | 0 | 0.08 | 1 |
| GO:0060690 | epithelial cell differentiation involved... | 1 | 0 | 0.04 | 1 |
| GO:0060691 | epithelial cell maturation involved in s... | 1 | 0 | 0.04 | 1 |

| | | | | | |
|---|---|---|---|---|---|
| GO:0060694 | regulation of cholesterol transporter ac... | 2 | 0 | 0.08 | 1 |
| GO:0060695 | negative regulation of cholesterol trans... | 1 | 0 | 0.04 | 1 |
| GO:0060696 | regulation of phospholipid catabolic pro... | 5 | 0 | 0.19 | 1 |
| GO:0060697 | positive regulation of phospholipid cata... | 2 | 0 | 0.08 | 1 |
| GO:0060701 | negative regulation of ribonuclease acti... | 1 | 0 | 0.04 | 1 |
| GO:0060702 | negative regulation of endoribonuclease ... | 1 | 0 | 0.04 | 1 |
| GO:0060708 | spongiotrophoblast differentiation | 4 | 0 | 0.15 | 1 |
| GO:0060709 | glycogen cell differentiation involved i... | 1 | 0 | 0.04 | 1 |
| GO:0060710 | chorio-allantoic fusion | 7 | 0 | 0.27 | 1 |
| GO:0060712 | spongiotrophoblast layer development | 14 | 0 | 0.53 | 1 |
| GO:0060715 | syncytiotrophoblast cell differentiation... | 2 | 0 | 0.08 | 1 |
| GO:0060717 | chorion development | 5 | 0 | 0.19 | 1 |
| GO:0060718 | chorionic trophoblast cell differentiati... | 4 | 0 | 0.15 | 1 |
| GO:0060720 | spongiotrophoblast cell proliferation | 1 | 0 | 0.04 | 1 |
| GO:0060721 | regulation of spongiotrophoblast cell pr... | 1 | 0 | 0.04 | 1 |
| GO:0060722 | cell proliferation involved in embryonic... | 1 | 0 | 0.04 | 1 |
| GO:0060723 | regulation of cell proliferation involve... | 1 | 0 | 0.04 | 1 |
| GO:0060730 | regulation of intestinal epithelial stru... | 2 | 0 | 0.08 | 1 |
| GO:0060731 | positive regulation of intestinal epithe... | 1 | 0 | 0.04 | 1 |
| GO:0060732 | positive regulation of inositol phosphat... | 8 | 0 | 0.3 | 1 |
| GO:0060735 | regulation of eIF2 alpha phosphorylation... | 1 | 0 | 0.04 | 1 |
| GO:0060737 | prostate gland morphogenetic growth | 4 | 0 | 0.15 | 1 |
| GO:0060738 | epithelial-mesenchymal signaling involve... | 2 | 0 | 0.08 | 1 |
| GO:0060739 | mesenchymal-epithelial cell signaling in... | 1 | 0 | 0.04 | 1 |
| GO:0060741 | prostate gland stromal morphogenesis | 3 | 0 | 0.11 | 1 |
| GO:0060742 | epithelial cell differentiation involved... | 12 | 0 | 0.46 | 1 |
| GO:0060743 | epithelial cell maturation involved in p... | 4 | 0 | 0.15 | 1 |
| GO:0060744 | mammary gland branching involved in thel | 6 | 0 | 0.23 | 1 |
| GO:0060745 | mammary gland branching involved in pre | 5 | 0 | 0.19 | 1 |
| GO:0060748 | tertiary branching involved in mammary g.. | 2 | 0 | 0.08 | 1 |
| GO:0060752 | intestinal phytosterol absorption | 2 | 0 | 0.08 | 1 |
| GO:0060753 | regulation of mast cell chemotaxis | 4 | 0 | 0.15 | 1 |
| GO:0060754 | positive regulation of mast cell chemota... | 4 | 0 | 0.15 | 1 |
| GO:0060762 | regulation of branching involved in mamm. | 6 | 0 | 0.23 | 1 |
| GO:0060763 | mammary duct terminal end bud growth | 5 | 0 | 0.19 | 1 |
| GO:0060764 | cell-cell signaling involved in mammary ... | 2 | 0 | 0.08 | 1 |
| GO:0060765 | regulation of androgen receptor signalin... | 23 | 0 | 0.88 | 1 |
| GO:0060766 | negative regulation of androgen receptor... | 14 | 0 | 0.53 | 1 |
| GO:0060767 | epithelial cell proliferation involved i... | 11 | 0 | 0.42 | 1 |
| GO:0060768 | regulation of epithelial cell proliferat... | 10 | 0 | 0.38 | 1 |
| GO:0060769 | positive regulation of epithelial cell p... | 2 | 0 | 0.08 | 1 |
| GO:0060770 | negative regulation of epithelial cell p... | 7 | 0 | 0.27 | 1 |
| GO:0060781 | mesenchymal cell proliferation involved ... | 1 | 0 | 0.04 | 1 |
| GO:0060782 | regulation of mesenchymal cell prolifera... | 1 | 0 | 0.04 | 1 |
| GO:0060783 | mesenchymal smoothened signaling pathv | 1 | 0 | 0.04 | 1 |
| GO:0060784 | regulation of cell proliferation involve... | 1 | 0 | 0.04 | 1 |
| GO:0060785 | regulation of apoptosis involved in tiss... | 1 | 0 | 0.04 | 1 |
| GO:0060789 | hair follicle placode formation | 6 | 0 | 0.23 | 1 |
| GO:0060796 | regulation of transcription involved in ... | 2 | 0 | 0.08 | 1 |
| GO:0060800 | regulation of cell differentiation invol... | 1 | 0 | 0.04 | 1 |
| GO:0060802 | epiblast cell-extraembryonic ectoderm ce.. | 1 | 0 | 0.04 | 1 |
| GO:0060803 | BMP signaling pathway involved in mesod | 1 | 0 | 0.04 | 1 |
| GO:0060804 | positive regulation of Wnt signaling pat... | 1 | 0 | 0.04 | 1 |

| | | | | | |
|---|---|---|---|---|---|
| GO:0060806 | negative regulation of cell differentiat... | 1 | 0 | 0.04 | 1 |
| GO:0060807 | regulation of transcription from RNA pol... | 1 | 0 | 0.04 | 1 |
| GO:0060809 | mesodermal to mesenchymal transition inv | 1 | 0 | 0.04 | 1 |
| GO:0060823 | canonical Wnt signaling pathway involved. | 2 | 0 | 0.08 | 1 |
| GO:0060827 | regulation of canonical Wnt signaling pa... | 1 | 0 | 0.04 | 1 |
| GO:0060829 | negative regulation of canonical Wnt sig... | 1 | 0 | 0.04 | 1 |
| GO:0060830 | ciliary receptor clustering involved in ... | 4 | 0 | 0.15 | 1 |
| GO:0060841 | venous blood vessel development | 14 | 0 | 0.53 | 1 |
| GO:0060843 | venous endothelial cell differentiation | 1 | 0 | 0.04 | 1 |
| GO:0060844 | arterial endothelial cell fate commitmen... | 1 | 0 | 0.04 | 1 |
| GO:0060846 | blood vessel endothelial cell fate commi... | 2 | 0 | 0.08 | 1 |
| GO:0060847 | endothelial cell fate specification | 3 | 0 | 0.11 | 1 |
| GO:0060857 | establishment of glial blood-brain barri... | 1 | 0 | 0.04 | 1 |
| GO:0060873 | anterior semicircular canal development | 1 | 0 | 0.04 | 1 |
| GO:0060875 | lateral semicircular canal development | 1 | 0 | 0.04 | 1 |
| GO:0060876 | semicircular canal formation | 2 | 0 | 0.08 | 1 |
| GO:0060879 | semicircular canal fusion | 1 | 0 | 0.04 | 1 |
| GO:0060900 | embryonic camera-type eye formation | 12 | 0 | 0.46 | 1 |
| GO:0060901 | regulation of hair cycle by canonical Wn... | 1 | 0 | 0.04 | 1 |
| GO:0060904 | regulation of protein folding in endopla... | 1 | 0 | 0.04 | 1 |
| GO:0060907 | positive regulation of macrophage cytoki... | 8 | 0 | 0.3 | 1 |
| GO:0060911 | cardiac cell fate commitment | 12 | 0 | 0.46 | 1 |
| GO:0060912 | cardiac cell fate specification | 2 | 0 | 0.08 | 1 |
| GO:0060913 | cardiac cell fate determination | 4 | 0 | 0.15 | 1 |
| GO:0060914 | heart formation | 23 | 0 | 0.88 | 1 |
| GO:0060915 | mesenchymal cell differentiation involve... | 2 | 0 | 0.08 | 1 |
| GO:0060916 | mesenchymal cell proliferation involved ... | 5 | 0 | 0.19 | 1 |
| GO:0060920 | cardiac pacemaker cell differentiation | 6 | 0 | 0.23 | 1 |
| GO:0060921 | sinoatrial node cell differentiation | 4 | 0 | 0.15 | 1 |
| GO:0060922 | atrioventricular node cell differentiati... | 2 | 0 | 0.08 | 1 |
| GO:0060923 | cardiac muscle cell fate commitment | 8 | 0 | 0.3 | 1 |
| GO:0060926 | cardiac pacemaker cell development | 5 | 0 | 0.19 | 1 |
| GO:0060927 | cardiac pacemaker cell fate commitment | 1 | 0 | 0.04 | 1 |
| GO:0060928 | atrioventricular node cell development | 2 | 0 | 0.08 | 1 |
| GO:0060929 | atrioventricular node cell fate commitme... | 1 | 0 | 0.04 | 1 |
| GO:0060931 | sinoatrial node cell development | 3 | 0 | 0.11 | 1 |
| GO:0060932 | His-Purkinje system cell differentiation | 2 | 0 | 0.08 | 1 |
| GO:0060947 | cardiac vascular smooth muscle cell diff... | 7 | 0 | 0.27 | 1 |
| GO:0060948 | cardiac vascular smooth muscle cell deve. | 3 | 0 | 0.11 | 1 |
| GO:0060956 | endocardial cell differentiation | 6 | 0 | 0.23 | 1 |
| GO:0060957 | endocardial cell fate commitment | 1 | 0 | 0.04 | 1 |
| GO:0060971 | embryonic heart tube left/right pattern ... | 5 | 0 | 0.19 | 1 |
| GO:0060973 | cell migration involved in heart develop... | 9 | 0 | 0.34 | 1 |
| GO:0060974 | cell migration involved in heart formati... | 1 | 0 | 0.04 | 1 |
| GO:0060975 | cardioblast migration to the midline inv... | 1 | 0 | 0.04 | 1 |
| GO:0060976 | coronary vasculature development | 16 | 0 | 0.61 | 1 |
| GO:0060977 | coronary vasculature morphogenesis | 10 | 0 | 0.38 | 1 |
| GO:0060978 | angiogenesis involved in coronary vascul.. | 2 | 0 | 0.08 | 1 |
| GO:0060979 | vasculogenesis involved in coronary vasc.. | 2 | 0 | 0.08 | 1 |
| GO:0060980 | cell migration involved in coronary vasc... | 1 | 0 | 0.04 | 1 |
| GO:0060981 | cell migration involved in coronary angi... | 1 | 0 | 0.04 | 1 |
| GO:0060982 | coronary artery morphogenesis | 6 | 0 | 0.23 | 1 |
| GO:0060988 | lipid tube assembly | 2 | 0 | 0.08 | 1 |

| | | | | | |
|---|---|---|---|---|---|
| GO:0060992 | response to fungicide | 4 | 0 | 0.15 | 1 |
| GO:0060993 | kidney morphogenesis | 95 | 0 | 3.62 | 1 |
| GO:0060994 | regulation of transcription from RNA pol... | 1 | 0 | 0.04 | 1 |
| GO:0060995 | cell-cell signaling involved in kidney d... | 2 | 0 | 0.08 | 1 |
| GO:0060999 | positive regulation of dendritic spine d... | 19 | 0 | 0.72 | 1 |
| GO:0061003 | positive regulation of dendritic spine m... | 9 | 0 | 0.34 | 1 |
| GO:0061004 | pattern specification involved in kidney... | 9 | 0 | 0.34 | 1 |
| GO:0061005 | cell differentiation involved in kidney ... | 46 | 0 | 1.75 | 1 |
| GO:0061009 | common bile duct development | 5 | 0 | 0.19 | 1 |
| GO:0061010 | gall bladder development | 3 | 0 | 0.11 | 1 |
| GO:0061011 | hepatic duct development | 1 | 0 | 0.04 | 1 |
| GO:0061015 | snRNA import into nucleus | 1 | 0 | 0.04 | 1 |
| GO:0061017 | hepatoblast differentiation | 2 | 0 | 0.08 | 1 |
| GO:0061026 | cardiac muscle tissue regeneration | 1 | 0 | 0.04 | 1 |
| GO:0061030 | epithelial cell differentiation involved... | 4 | 0 | 0.15 | 1 |
| GO:0061031 | endodermal digestive tract morphogenesis | 4 | 0 | 0.15 | 1 |
| GO:0061032 | visceral serous pericardium development | 2 | 0 | 0.08 | 1 |
| GO:0061033 | secretion by lung epithelial cell involv... | 2 | 0 | 0.08 | 1 |
| GO:0061034 | olfactory bulb mitral cell layer develop... | 2 | 0 | 0.08 | 1 |
| GO:0061042 | vascular wound healing | 7 | 0 | 0.27 | 1 |
| GO:0061043 | regulation of vascular wound healing | 4 | 0 | 0.15 | 1 |
| GO:0061044 | negative regulation of vascular wound he.. | 1 | 0 | 0.04 | 1 |
| GO:0061046 | regulation of branching involved in lung... | 6 | 0 | 0.23 | 1 |
| GO:0061047 | positive regulation of branching involve... | 4 | 0 | 0.15 | 1 |
| GO:0061048 | negative regulation of branching involve... | 1 | 0 | 0.04 | 1 |
| GO:0061052 | negative regulation of cell growth invol... | 3 | 0 | 0.11 | 1 |
| GO:0061054 | dermatome development | 4 | 0 | 0.15 | 1 |
| GO:0061055 | myotome development | 4 | 0 | 0.15 | 1 |
| GO:0061056 | sclerotome development | 4 | 0 | 0.15 | 1 |
| GO:0061072 | iris morphogenesis | 7 | 0 | 0.27 | 1 |
| GO:0061073 | ciliary body morphogenesis | 3 | 0 | 0.11 | 1 |
| GO:0061074 | regulation of neural retina development | 3 | 0 | 0.11 | 1 |
| GO:0061078 | positive regulation of prostaglandin sec... | 1 | 0 | 0.04 | 1 |
| GO:0061083 | regulation of protein refolding | 3 | 0 | 0.11 | 1 |
| GO:0061084 | negative regulation of protein refolding | 3 | 0 | 0.11 | 1 |
| GO:0061088 | regulation of sequestering of zinc ion | 5 | 0 | 0.19 | 1 |
| GO:0061090 | positive regulation of sequestering of z... | 1 | 0 | 0.04 | 1 |
| GO:0061100 | lung neuroendocrine cell differentiation | 1 | 0 | 0.04 | 1 |
| GO:0061101 | neuroendocrine cell differentiation | 8 | 0 | 0.3 | 1 |
| GO:0061102 | stomach neuroendocrine cell differentiat... | 2 | 0 | 0.08 | 1 |
| GO:0061103 | carotid body glomus cell differentiation | 1 | 0 | 0.04 | 1 |
| GO:0061104 | adrenal chromaffin cell differentiation | 2 | 0 | 0.08 | 1 |
| GO:0061105 | regulation of stomach neuroendocrine cel.. | 1 | 0 | 0.04 | 1 |
| GO:0061106 | negative regulation of stomach neuroendo | 1 | 0 | 0.04 | 1 |
| GO:0061107 | seminal vesicle development | 1 | 0 | 0.04 | 1 |
| GO:0061108 | seminal vesicle epithelium development | 1 | 0 | 0.04 | 1 |
| GO:0061110 | dense core granule biogenesis | 3 | 0 | 0.11 | 1 |
| GO:0061113 | pancreas morphogenesis | 2 | 0 | 0.08 | 1 |
| GO:0061114 | branching involved in pancreas morphoge | 1 | 0 | 0.04 | 1 |
| GO:0061115 | lung proximal/distal axis specification | 1 | 0 | 0.04 | 1 |
| GO:0061117 | negative regulation of heart growth | 11 | 0 | 0.42 | 1 |
| GO:0061140 | lung secretory cell differentiation | 12 | 0 | 0.46 | 1 |
| GO:0061141 | lung ciliated cell differentiation | 3 | 0 | 0.11 | 1 |

| | | | | | |
|---|---|---|---|---|---|
| GO:0061143 | alveolar primary septum development | 1 | 0 | 0.04 | 1 |
| GO:0061144 | alveolar secondary septum development | 2 | 0 | 0.08 | 1 |
| GO:0061146 | Peyer's patch morphogenesis | 3 | 0 | 0.11 | 1 |
| GO:0061149 | BMP signaling pathway involved in ureter.. | 1 | 0 | 0.04 | 1 |
| GO:0061150 | renal system segmentation | 1 | 0 | 0.04 | 1 |
| GO:0061151 | BMP signaling pathway involved in renal .. | 1 | 0 | 0.04 | 1 |
| GO:0061154 | endothelial tube morphogenesis | 11 | 0 | 0.42 | 1 |
| GO:0061155 | pulmonary artery endothelial tube morpho. | 1 | 0 | 0.04 | 1 |
| GO:0061156 | pulmonary artery morphogenesis | 5 | 0 | 0.19 | 1 |
| GO:0061158 | 3'-UTR-mediated mRNA destabilization | 3 | 0 | 0.11 | 1 |
| GO:0061162 | establishment of monopolar cell polarity | 9 | 0 | 0.34 | 1 |
| GO:0061179 | negative regulation of insulin secretion... | 6 | 0 | 0.23 | 1 |
| GO:0061181 | regulation of chondrocyte development | 1 | 0 | 0.04 | 1 |
| GO:0061183 | regulation of dermatome development | 4 | 0 | 0.15 | 1 |
| GO:0061184 | positive regulation of dermatome develop.. | 3 | 0 | 0.11 | 1 |
| GO:0061185 | negative regulation of dermatome develop | 1 | 0 | 0.04 | 1 |
| GO:0061187 | regulation of chromatin silencing at rDN... | 2 | 0 | 0.08 | 1 |
| GO:0061188 | negative regulation of chromatin silenci... | 2 | 0 | 0.08 | 1 |
| GO:0061189 | positive regulation of sclerotome develo... | 1 | 0 | 0.04 | 1 |
| GO:0061190 | regulation of sclerotome development | 1 | 0 | 0.04 | 1 |
| GO:0061193 | taste bud development | 2 | 0 | 0.08 | 1 |
| GO:0061196 | fungiform papilla development | 6 | 0 | 0.23 | 1 |
| GO:0061197 | fungiform papilla morphogenesis | 5 | 0 | 0.19 | 1 |
| GO:0061198 | fungiform papilla formation | 3 | 0 | 0.11 | 1 |
| GO:0061205 | paramesonephric duct development | 4 | 0 | 0.15 | 1 |
| GO:0061206 | mesonephros morphogenesis | 1 | 0 | 0.04 | 1 |
| GO:0061209 | cell proliferation involved in mesonephr... | 4 | 0 | 0.15 | 1 |
| GO:0061213 | positive regulation of mesonephros devel.. | 22 | 0 | 0.84 | 1 |
| GO:0061215 | mesonephric nephron development | 1 | 0 | 0.04 | 1 |
| GO:0061216 | regulation of transcription from RNA pol... | 1 | 0 | 0.04 | 1 |
| GO:0061217 | regulation of mesonephros development | 26 | 0 | 0.99 | 1 |
| GO:0061218 | negative regulation of mesonephros devel. | 6 | 0 | 0.23 | 1 |
| GO:0061227 | pattern specification involved in mesone... | 2 | 0 | 0.08 | 1 |
| GO:0061228 | mesonephric nephron morphogenesis | 1 | 0 | 0.04 | 1 |
| GO:0061235 | mesenchymal stem cell maintenance invol | 1 | 0 | 0.04 | 1 |
| GO:0061245 | establishment or maintenance of bipolar ... | 27 | 0 | 1.03 | 1 |
| GO:0061289 | Wnt signaling pathway involved in kidney.. | 2 | 0 | 0.08 | 1 |
| GO:0061290 | canonical Wnt signaling pathway involved. | 2 | 0 | 0.08 | 1 |
| GO:0061295 | regulation of mesenchymal cell apoptotic.. | 1 | 0 | 0.04 | 1 |
| GO:0061296 | negative regulation of mesenchymal cell .. | 1 | 0 | 0.04 | 1 |
| GO:0061298 | retina vasculature development in camera. | 18 | 0 | 0.69 | 1 |
| GO:0061299 | retina vasculature morphogenesis in came | 11 | 0 | 0.42 | 1 |
| GO:0061300 | cerebellum vasculature development | 1 | 0 | 0.04 | 1 |
| GO:0061301 | cerebellum vasculature morphogenesis | 1 | 0 | 0.04 | 1 |
| GO:0061302 | smooth muscle cell-matrix adhesion | 5 | 0 | 0.19 | 1 |
| GO:0061303 | cornea development in camera-type eye | 9 | 0 | 0.34 | 1 |
| GO:0061304 | retinal blood vessel morphogenesis | 5 | 0 | 0.19 | 1 |
| GO:0061308 | cardiac neural crest cell development in... | 6 | 0 | 0.23 | 1 |
| GO:0061309 | cardiac neural crest cell development in... | 6 | 0 | 0.23 | 1 |
| GO:0061312 | BMP signaling pathway involved in heart .. | 5 | 0 | 0.19 | 1 |
| GO:0061317 | canonical Wnt signaling pathway involved. | 4 | 0 | 0.15 | 1 |
| GO:0061318 | renal filtration cell differentiation | 16 | 0 | 0.61 | 1 |
| GO:0061333 | renal tubule morphogenesis | 77 | 0 | 2.93 | 1 |

| | | | | | |
|---|---|---|---|---|---|
| GO:0061339 | establishment or maintenance of monopola | 9 | 0 | 0.34 | 1 |
| GO:0061360 | optic chiasma development | 1 | 0 | 0.04 | 1 |
| GO:0061365 | positive regulation of triglyceride lipa... | 11 | 0 | 0.42 | 1 |
| GO:0061369 | negative regulation of testicular blood ... | 1 | 0 | 0.04 | 1 |
| GO:0061370 | testosterone biosynthetic process | 6 | 0 | 0.23 | 1 |
| GO:0061373 | mammillary axonal complex development | 1 | 0 | 0.04 | 1 |
| GO:0061374 | mammillothalamic axonal tract developme | 1 | 0 | 0.04 | 1 |
| GO:0061378 | corpora quadrigemina development | 2 | 0 | 0.08 | 1 |
| GO:0061379 | inferior colliculus development | 2 | 0 | 0.08 | 1 |
| GO:0061381 | cell migration in diencephalon | 1 | 0 | 0.04 | 1 |
| GO:0061386 | closure of optic fissure | 2 | 0 | 0.08 | 1 |
| GO:0061395 | positive regulation of transcription fro... | 1 | 0 | 0.04 | 1 |
| GO:0061419 | positive regulation of transcription fro... | 3 | 0 | 0.11 | 1 |
| GO:0061433 | cellular response to caloric restriction | 1 | 0 | 0.04 | 1 |
| GO:0061436 | establishment of skin barrier | 16 | 0 | 0.61 | 1 |
| GO:0061437 | renal system vasculature development | 22 | 0 | 0.84 | 1 |
| GO:0061438 | renal system vasculature morphogenesis | 7 | 0 | 0.27 | 1 |
| GO:0061439 | kidney vasculature morphogenesis | 7 | 0 | 0.27 | 1 |
| GO:0061440 | kidney vasculature development | 22 | 0 | 0.84 | 1 |
| GO:0061441 | renal artery morphogenesis | 2 | 0 | 0.08 | 1 |
| GO:0061443 | endocardial cushion cell differentiation | 2 | 0 | 0.08 | 1 |
| GO:0061444 | endocardial cushion cell development | 1 | 0 | 0.04 | 1 |
| GO:0061445 | endocardial cushion cell fate commitment | 1 | 0 | 0.04 | 1 |
| GO:0061450 | trophoblast cell migration | 7 | 0 | 0.27 | 1 |
| GO:0061451 | retrotrapezoid nucleus development | 1 | 0 | 0.04 | 1 |
| GO:0061452 | retrotrapezoid nucleus neuron differenti... | 1 | 0 | 0.04 | 1 |
| GO:0061461 | L-lysine import | 1 | 0 | 0.04 | 1 |
| GO:0061467 | basolateral protein localization | 1 | 0 | 0.04 | 1 |
| GO:0061469 | regulation of type B pancreatic cell pro... | 3 | 0 | 0.11 | 1 |
| GO:0061470 | T follicular helper cell differentiation | 3 | 0 | 0.11 | 1 |
| GO:0061484 | hematopoietic stem cell homeostasis | 3 | 0 | 0.11 | 1 |
| GO:0061485 | memory T cell proliferation | 1 | 0 | 0.04 | 1 |
| GO:0061502 | early endosome to recycling endosome tra | 4 | 0 | 0.15 | 1 |
| GO:0061511 | centriole elongation | 3 | 0 | 0.11 | 1 |
| GO:0061512 | protein localization to cilium | 14 | 0 | 0.53 | 1 |
| GO:0061517 | macrophage proliferation | 1 | 0 | 0.04 | 1 |
| GO:0061518 | microglial cell proliferation | 1 | 0 | 0.04 | 1 |
| GO:0061526 | acetylcholine secretion | 5 | 0 | 0.19 | 1 |
| GO:0061535 | glutamate secretion | neurotransr | 1 | 0 | 0.04 |
| GO:0061548 | ganglion development | 13 | 0 | 0.49 | 1 |
| GO:0061549 | sympathetic ganglion development | 9 | 0 | 0.34 | 1 |
| GO:0061550 | cranial ganglion development | 4 | 0 | 0.15 | 1 |
| GO:0061551 | trigeminal ganglion development | 4 | 0 | 0.15 | 1 |
| GO:0061552 | ganglion morphogenesis | 1 | 0 | 0.04 | 1 |
| GO:0061577 | generation of L-type calcium current | 4 | 0 | 0.15 | 1 |
| GO:0061580 | colon epithelial cell migration | 1 | 0 | 0.04 | 1 |
| GO:0061582 | intestinal epithelial cell migration | 1 | 0 | 0.04 | 1 |
| GO:0061588 | calcium activated phospholipid scramblin.. | 5 | 0 | 0.19 | 1 |
| GO:0061589 | calcium activated phosphatidylserine scr... | 4 | 0 | 0.15 | 1 |
| GO:0061590 | calcium activated phosphatidylcholine sc... | 5 | 0 | 0.19 | 1 |
| GO:0061591 | calcium activated galactosylceramide scr... | 5 | 0 | 0.19 | 1 |
| GO:0061614 | pri-miRNA transcription from RNA polymer | 3 | 0 | 0.11 | 1 |
| GO:0061626 | pharyngeal arch artery morphogenesis | 1 | 0 | 0.04 | 1 |

| | | | | | |
|---|---|---|---|---|---|
| GO:0061635 | regulation of protein complex stability | 1 | 0 | 0.04 | 1 |
| GO:0061643 | chemorepulsion of axon | 1 | 0 | 0.04 | 1 |
| GO:0061646 | positive regulation of glutamate neurotr... | 1 | 0 | 0.04 | 1 |
| GO:0061668 | mitochondrial ribosome assembly | 1 | 0 | 0.04 | 1 |
| GO:0061687 | detoxification of inorganic compound | 7 | 0 | 0.27 | 1 |
| GO:0065001 | specification of axis polarity | 4 | 0 | 0.15 | 1 |
| GO:0065005 | protein-lipid complex assembly | 21 | 0 | 0.8 | 1 |
| GO:0065006 | protein-carbohydrate complex assembly | 2 | 0 | 0.08 | 1 |
| GO:0070050 | neuron cellular homeostasis | 7 | 0 | 0.27 | 1 |
| GO:0070054 | mRNA splicing | via endonu | 1 | 0 | 0.04 |
| GO:0070055 | HAC1-type intron splice site recognition... | 1 | 0 | 0.04 | 1 |
| GO:0070060 | 'de novo' actin filament nucleation | 1 | 0 | 0.04 | 1 |
| GO:0070070 | proton-transporting V-type ATPase comple | 5 | 0 | 0.19 | 1 |
| GO:0070071 | proton-transporting two-sector ATPase co. | 8 | 0 | 0.3 | 1 |
| GO:0070072 | vacuolar proton-transporting V-type ATPa.. | 5 | 0 | 0.19 | 1 |
| GO:0070075 | tear secretion | 2 | 0 | 0.08 | 1 |
| GO:0070076 | histone lysine demethylation | 23 | 0 | 0.88 | 1 |
| GO:0070077 | histone arginine demethylation | 1 | 0 | 0.04 | 1 |
| GO:0070078 | histone H3-R2 demethylation | 1 | 0 | 0.04 | 1 |
| GO:0070079 | histone H4-R3 demethylation | 1 | 0 | 0.04 | 1 |
| GO:0070084 | protein initiator methionine removal | 2 | 0 | 0.08 | 1 |
| GO:0070086 | ubiquitin-dependent endocytosis | 2 | 0 | 0.08 | 1 |
| GO:0070094 | positive regulation of glucagon secretio... | 2 | 0 | 0.08 | 1 |
| GO:0070100 | negative regulation of chemokine-mediate. | 5 | 0 | 0.19 | 1 |
| GO:0070105 | positive regulation of interleukin-6-med... | 1 | 0 | 0.04 | 1 |
| GO:0070106 | interleukin-27-mediated signaling pathwa.. | 2 | 0 | 0.08 | 1 |
| GO:0070120 | ciliary neurotrophic factor-mediated sig... | 5 | 0 | 0.19 | 1 |
| GO:0070121 | Kupffer's vesicle development | 1 | 0 | 0.04 | 1 |
| GO:0070127 | tRNA aminoacylation for mitochondrial pr... | 5 | 0 | 0.19 | 1 |
| GO:0070129 | regulation of mitochondrial translation | 5 | 0 | 0.19 | 1 |
| GO:0070131 | positive regulation of mitochondrial tra... | 3 | 0 | 0.11 | 1 |
| GO:0070143 | mitochondrial alanyl-tRNA aminoacylation | 1 | 0 | 0.04 | 1 |
| GO:0070145 | mitochondrial asparaginyl-tRNA aminoacyl | 1 | 0 | 0.04 | 1 |
| GO:0070159 | mitochondrial threonyl-tRNA aminoacylati.. | 1 | 0 | 0.04 | 1 |
| GO:0070162 | adiponectin secretion | 5 | 0 | 0.19 | 1 |
| GO:0070163 | regulation of adiponectin secretion | 5 | 0 | 0.19 | 1 |
| GO:0070164 | negative regulation of adiponectin secre... | 4 | 0 | 0.15 | 1 |
| GO:0070165 | positive regulation of adiponectin secre... | 1 | 0 | 0.04 | 1 |
| GO:0070166 | enamel mineralization | 9 | 0 | 0.34 | 1 |
| GO:0070170 | regulation of tooth mineralization | 7 | 0 | 0.27 | 1 |
| GO:0070171 | negative regulation of tooth mineralizat... | 2 | 0 | 0.08 | 1 |
| GO:0070172 | positive regulation of tooth mineralizat... | 3 | 0 | 0.11 | 1 |
| GO:0070173 | regulation of enamel mineralization | 2 | 0 | 0.08 | 1 |
| GO:0070178 | D-serine metabolic process | 2 | 0 | 0.08 | 1 |
| GO:0070179 | D-serine biosynthetic process | 1 | 0 | 0.04 | 1 |
| GO:0070184 | mitochondrial tyrosyl-tRNA aminoacylatio.. | 1 | 0 | 0.04 | 1 |
| GO:0070189 | kynurenine metabolic process | 10 | 0 | 0.38 | 1 |
| GO:0070198 | protein localization to chromosome | telo... | 6 | 0 | 0.23 |
| GO:0070199 | establishment of protein localization to... | 6 | 0 | 0.23 | 1 |
| GO:0070212 | protein poly-ADP-ribosylation | 3 | 0 | 0.11 | 1 |
| GO:0070213 | protein auto-ADP-ribosylation | 4 | 0 | 0.15 | 1 |
| GO:0070217 | transcription factor TFIIIB complex asse... | 1 | 0 | 0.04 | 1 |
| GO:0070221 | sulfide oxidation | using sulfid | 5 | 0 | 0.19 |

| | | | | | |
|---|---|---|---|---|---|
| GO:0070233 | negative regulation of T cell apoptotic ... | 17 | 0 | 0.65 | 1 |
| GO:0070235 | regulation of activation-induced cell de... | 5 | 0 | 0.19 | 1 |
| GO:0070236 | negative regulation of activation-induce... | 3 | 0 | 0.11 | 1 |
| GO:0070237 | positive regulation of activation-induce... | 1 | 0 | 0.04 | 1 |
| GO:0070242 | thymocyte apoptotic process | 19 | 0 | 0.72 | 1 |
| GO:0070243 | regulation of thymocyte apoptotic proces... | 14 | 0 | 0.53 | 1 |
| GO:0070244 | negative regulation of thymocyte apoptot... | 7 | 0 | 0.27 | 1 |
| GO:0070245 | positive regulation of thymocyte apoptot... | 6 | 0 | 0.23 | 1 |
| GO:0070246 | natural killer cell apoptotic process | 1 | 0 | 0.04 | 1 |
| GO:0070247 | regulation of natural killer cell apopto... | 1 | 0 | 0.04 | 1 |
| GO:0070253 | somatostatin secretion | 3 | 0 | 0.11 | 1 |
| GO:0070254 | mucus secretion | 13 | 0 | 0.49 | 1 |
| GO:0070255 | regulation of mucus secretion | 11 | 0 | 0.42 | 1 |
| GO:0070256 | negative regulation of mucus secretion | 2 | 0 | 0.08 | 1 |
| GO:0070257 | positive regulation of mucus secretion | 8 | 0 | 0.3 | 1 |
| GO:0070262 | peptidyl-serine dephosphorylation | 7 | 0 | 0.27 | 1 |
| GO:0070265 | necrotic cell death | 37 | 0 | 1.41 | 1 |
| GO:0070266 | necroptotic process | 24 | 0 | 0.91 | 1 |
| GO:0070267 | oncosis | 1 | 0 | 0.04 | 1 |
| GO:0070268 | cornification | 3 | 0 | 0.11 | 1 |
| GO:0070269 | pyroptosis | 4 | 0 | 0.15 | 1 |
| GO:0070272 | proton-transporting ATP synthase complex | 3 | 0 | 0.11 | 1 |
| GO:0070278 | extracellular matrix constituent secreti... | 4 | 0 | 0.15 | 1 |
| GO:0070286 | axonemal dynein complex assembly | 9 | 0 | 0.34 | 1 |
| GO:0070294 | renal sodium ion absorption | 4 | 0 | 0.15 | 1 |
| GO:0070305 | response to cGMP | 7 | 0 | 0.27 | 1 |
| GO:0070309 | lens fiber cell morphogenesis | 4 | 0 | 0.15 | 1 |
| GO:0070314 | G1 to G0 transition | 6 | 0 | 0.23 | 1 |
| GO:0070315 | G1 to G0 transition involved in cell dif... | 3 | 0 | 0.11 | 1 |
| GO:0070316 | regulation of G0 to G1 transition | 7 | 0 | 0.27 | 1 |
| GO:0070317 | negative regulation of G0 to G1 transiti... | 4 | 0 | 0.15 | 1 |
| GO:0070318 | positive regulation of G0 to G1 transiti... | 3 | 0 | 0.11 | 1 |
| GO:0070327 | thyroid hormone transport | 5 | 0 | 0.19 | 1 |
| GO:0070328 | triglyceride homeostasis | 28 | 0 | 1.07 | 1 |
| GO:0070339 | response to bacterial lipopeptide | 5 | 0 | 0.19 | 1 |
| GO:0070340 | detection of bacterial lipopeptide | 3 | 0 | 0.11 | 1 |
| GO:0070341 | fat cell proliferation | 9 | 0 | 0.34 | 1 |
| GO:0070342 | brown fat cell proliferation | 1 | 0 | 0.04 | 1 |
| GO:0070343 | white fat cell proliferation | 2 | 0 | 0.08 | 1 |
| GO:0070344 | regulation of fat cell proliferation | 9 | 0 | 0.34 | 1 |
| GO:0070345 | negative regulation of fat cell prolifer... | 5 | 0 | 0.19 | 1 |
| GO:0070346 | positive regulation of fat cell prolifer... | 3 | 0 | 0.11 | 1 |
| GO:0070347 | regulation of brown fat cell proliferati... | 1 | 0 | 0.04 | 1 |
| GO:0070349 | positive regulation of brown fat cell pr... | 1 | 0 | 0.04 | 1 |
| GO:0070350 | regulation of white fat cell proliferati... | 2 | 0 | 0.08 | 1 |
| GO:0070352 | positive regulation of white fat cell pr... | 1 | 0 | 0.04 | 1 |
| GO:0070358 | actin polymerization-dependent cell moti... | 3 | 0 | 0.11 | 1 |
| GO:0070365 | hepatocyte differentiation | 11 | 0 | 0.42 | 1 |
| GO:0070366 | regulation of hepatocyte differentiation | 2 | 0 | 0.08 | 1 |
| GO:0070367 | negative regulation of hepatocyte differ... | 1 | 0 | 0.04 | 1 |
| GO:0070368 | positive regulation of hepatocyte differ... | 1 | 0 | 0.04 | 1 |
| GO:0070375 | ERK5 cascade | 2 | 0 | 0.08 | 1 |
| GO:0070383 | DNA cytosine deamination | 5 | 0 | 0.19 | 1 |

| | | | | | |
|---|---|---|---|---|---|
| GO:0070384 | Harderian gland development | 2 | 0 | 0.08 | 1 |
| GO:0070391 | response to lipoteichoic acid | 9 | 0 | 0.34 | 1 |
| GO:0070392 | detection of lipoteichoic acid | 1 | 0 | 0.04 | 1 |
| GO:0070407 | oxidation-dependent protein catabolic pr... | 1 | 0 | 0.04 | 1 |
| GO:0070408 | carbamoyl phosphate metabolic process | 2 | 0 | 0.08 | 1 |
| GO:0070409 | carbamoyl phosphate biosynthetic process | 2 | 0 | 0.08 | 1 |
| GO:0070417 | cellular response to cold | 4 | 0 | 0.15 | 1 |
| GO:0070424 | regulation of nucleotide-binding oligome... | 6 | 0 | 0.23 | 1 |
| GO:0070425 | negative regulation of nucleotide-bindin... | 1 | 0 | 0.04 | 1 |
| GO:0070426 | positive regulation of nucleotide-bindin... | 2 | 0 | 0.08 | 1 |
| GO:0070427 | nucleotide-binding oligomerization domai... | 5 | 0 | 0.19 | 1 |
| GO:0070428 | regulation of nucleotide-binding oligome... | 3 | 0 | 0.11 | 1 |
| GO:0070429 | negative regulation of nucleotide-bindin... | 1 | 0 | 0.04 | 1 |
| GO:0070430 | positive regulation of nucleotide-bindin... | 1 | 0 | 0.04 | 1 |
| GO:0070431 | nucleotide-binding oligomerization domai... | 7 | 0 | 0.27 | 1 |
| GO:0070432 | regulation of nucleotide-binding oligome... | 3 | 0 | 0.11 | 1 |
| GO:0070433 | negative regulation of nucleotide-bindin... | 1 | 0 | 0.04 | 1 |
| GO:0070434 | positive regulation of nucleotide-bindin... | 2 | 0 | 0.08 | 1 |
| GO:0070446 | negative regulation of oligodendrocyte p... | 1 | 0 | 0.04 | 1 |
| GO:0070447 | positive regulation of oligodendrocyte p... | 1 | 0 | 0.04 | 1 |
| GO:0070458 | cellular detoxification of nitrogen comp... | 3 | 0 | 0.11 | 1 |
| GO:0070460 | thyroid-stimulating hormone secretion | 1 | 0 | 0.04 | 1 |
| GO:0070462 | plus-end specific microtubule depolymeri... | 1 | 0 | 0.04 | 1 |
| GO:0070471 | uterine smooth muscle contraction | 10 | 0 | 0.38 | 1 |
| GO:0070472 | regulation of uterine smooth muscle cont... | 9 | 0 | 0.34 | 1 |
| GO:0070473 | negative regulation of uterine smooth mu... | 2 | 0 | 0.08 | 1 |
| GO:0070474 | positive regulation of uterine smooth mu... | 7 | 0 | 0.27 | 1 |
| GO:0070481 | nuclear-transcribed mRNA catabolic proce | 1 | 0 | 0.04 | 1 |
| GO:0070483 | detection of hypoxia | 2 | 0 | 0.08 | 1 |
| GO:0070487 | monocyte aggregation | 4 | 0 | 0.15 | 1 |
| GO:0070488 | neutrophil aggregation | 2 | 0 | 0.08 | 1 |
| GO:0070493 | thrombin receptor signaling pathway | 10 | 0 | 0.38 | 1 |
| GO:0070494 | regulation of thrombin receptor signalin... | 1 | 0 | 0.04 | 1 |
| GO:0070495 | negative regulation of thrombin receptor... | 1 | 0 | 0.04 | 1 |
| GO:0070498 | interleukin-1-mediated signaling pathway | 15 | 0 | 0.57 | 1 |
| GO:0070508 | cholesterol import | 5 | 0 | 0.19 | 1 |
| GO:0070510 | regulation of histone H4-K20 methylation | 1 | 0 | 0.04 | 1 |
| GO:0070512 | positive regulation of histone H4-K20 me... | 1 | 0 | 0.04 | 1 |
| GO:0070525 | threonylcarbamoyladenosine metabolic pro | 4 | 0 | 0.15 | 1 |
| GO:0070526 | threonylcarbamoyladenosine biosynthetic | 2 | 0 | 0.08 | 1 |
| GO:0070528 | protein kinase C signaling | 25 | 0 | 0.95 | 1 |
| GO:0070534 | protein K63-linked ubiquitination | 41 | 0 | 1.56 | 1 |
| GO:0070535 | histone H2A K63-linked ubiquitination | 5 | 0 | 0.19 | 1 |
| GO:0070541 | response to platinum ion | 2 | 0 | 0.08 | 1 |
| GO:0070543 | response to linoleic acid | 1 | 0 | 0.04 | 1 |
| GO:0070544 | histone H3-K36 demethylation | 2 | 0 | 0.08 | 1 |
| GO:0070560 | protein secretion by platelet | 1 | 0 | 0.04 | 1 |
| GO:0070561 | vitamin D receptor signaling pathway | 9 | 0 | 0.34 | 1 |
| GO:0070562 | regulation of vitamin D receptor signali... | 6 | 0 | 0.23 | 1 |
| GO:0070563 | negative regulation of vitamin D recepto... | 2 | 0 | 0.08 | 1 |
| GO:0070564 | positive regulation of vitamin D recepto... | 2 | 0 | 0.08 | 1 |
| GO:0070571 | negative regulation of neuron projection... | 4 | 0 | 0.15 | 1 |
| GO:0070574 | cadmium ion transmembrane transport | 4 | 0 | 0.15 | 1 |

| | | | | | |
|---|---|---|---|---|---|
| GO:0070586 | cell-cell adhesion involved in gastrulat... | 8 | 0 | 0.3 | 1 |
| GO:0070587 | regulation of cell-cell adhesion involve... | 7 | 0 | 0.27 | 1 |
| GO:0070593 | dendrite self-avoidance | 2 | 0 | 0.08 | 1 |
| GO:0070601 | centromeric sister chromatid cohesion | 4 | 0 | 0.15 | 1 |
| GO:0070602 | regulation of centromeric sister chromat... | 3 | 0 | 0.11 | 1 |
| GO:0070625 | zymogen granule exocytosis | 1 | 0 | 0.04 | 1 |
| GO:0070627 | ferrous iron import | 1 | 0 | 0.04 | 1 |
| GO:0070633 | transepithelial transport | 13 | 0 | 0.49 | 1 |
| GO:0070634 | transepithelial ammonium transport | 3 | 0 | 0.11 | 1 |
| GO:0070640 | vitamin D3 metabolic process | 4 | 0 | 0.15 | 1 |
| GO:0070649 | formin-nucleated actin cable assembly | 3 | 0 | 0.11 | 1 |
| GO:0070662 | mast cell proliferation | 5 | 0 | 0.19 | 1 |
| GO:0070666 | regulation of mast cell proliferation | 4 | 0 | 0.15 | 1 |
| GO:0070667 | negative regulation of mast cell prolife... | 1 | 0 | 0.04 | 1 |
| GO:0070668 | positive regulation of mast cell prolife... | 4 | 0 | 0.15 | 1 |
| GO:0070671 | response to interleukin-12 | 5 | 0 | 0.19 | 1 |
| GO:0070673 | response to interleukin-18 | 4 | 0 | 0.15 | 1 |
| GO:0070676 | intralumenal vesicle formation | 1 | 0 | 0.04 | 1 |
| GO:0070681 | glutaminyl-tRNAGln biosynthesis via tran.. | 2 | 0 | 0.08 | 1 |
| GO:0070682 | proteasome regulatory particle assembly | 3 | 0 | 0.11 | 1 |
| GO:0070684 | seminal clot liquefaction | 1 | 0 | 0.04 | 1 |
| GO:0070715 | sodium-dependent organic cation transpor | 1 | 0 | 0.04 | 1 |
| GO:0070777 | D-aspartate transport | 3 | 0 | 0.11 | 1 |
| GO:0070778 | L-aspartate transport | 4 | 0 | 0.15 | 1 |
| GO:0070779 | D-aspartate import | 3 | 0 | 0.11 | 1 |
| GO:0070781 | response to biotin | 2 | 0 | 0.08 | 1 |
| GO:0070782 | phosphatidylserine exposure on apoptotic. | 1 | 0 | 0.04 | 1 |
| GO:0070813 | hydrogen sulfide metabolic process | 5 | 0 | 0.19 | 1 |
| GO:0070814 | hydrogen sulfide biosynthetic process | 3 | 0 | 0.11 | 1 |
| GO:0070816 | phosphorylation of RNA polymerase II C-t. | 3 | 0 | 0.11 | 1 |
| GO:0070827 | chromatin maintenance | 3 | 0 | 0.11 | 1 |
| GO:0070828 | heterochromatin organization | 12 | 0 | 0.46 | 1 |
| GO:0070829 | heterochromatin maintenance | 2 | 0 | 0.08 | 1 |
| GO:0070830 | tight junction assembly | 48 | 0 | 1.83 | 1 |
| GO:0070831 | basement membrane assembly | 2 | 0 | 0.08 | 1 |
| GO:0070837 | dehydroascorbic acid transport | 3 | 0 | 0.11 | 1 |
| GO:0070839 | divalent metal ion export | 1 | 0 | 0.04 | 1 |
| GO:0070842 | aggresome assembly | 5 | 0 | 0.19 | 1 |
| GO:0070843 | misfolded protein transport | 2 | 0 | 0.08 | 1 |
| GO:0070844 | polyubiquitinated protein transport | 2 | 0 | 0.08 | 1 |
| GO:0070845 | polyubiquitinated misfolded protein tran... | 2 | 0 | 0.08 | 1 |
| GO:0070846 | Hsp90 deacetylation | 1 | 0 | 0.04 | 1 |
| GO:0070849 | response to epidermal growth factor | 24 | 0 | 0.91 | 1 |
| GO:0070859 | positive regulation of bile acid biosynt... | 1 | 0 | 0.04 | 1 |
| GO:0070861 | regulation of protein exit from endoplas... | 5 | 0 | 0.19 | 1 |
| GO:0070863 | positive regulation of protein exit from... | 4 | 0 | 0.15 | 1 |
| GO:0070868 | heterochromatin organization involved in... | 1 | 0 | 0.04 | 1 |
| GO:0070869 | heterochromatin assembly involved in chr.. | 1 | 0 | 0.04 | 1 |
| GO:0070874 | negative regulation of glycogen metaboli... | 9 | 0 | 0.34 | 1 |
| GO:0070884 | regulation of calcineurin-NFAT signaling... | 13 | 0 | 0.49 | 1 |
| GO:0070885 | negative regulation of calcineurin-NFAT ... | 3 | 0 | 0.11 | 1 |
| GO:0070886 | positive regulation of calcineurin-NFAT ... | 6 | 0 | 0.23 | 1 |
| GO:0070889 | platelet alpha granule organization | 2 | 0 | 0.08 | 1 |

| | | | | | |
|---|---|---|---|---|---|
| GO:0070893 | transposon integration | 2 | 0 | 0.08 | 1 |
| GO:0070894 | regulation of transposon integration | 2 | 0 | 0.08 | 1 |
| GO:0070895 | negative regulation of transposon integr... | 2 | 0 | 0.08 | 1 |
| GO:0070899 | mitochondrial tRNA wobble uridine modifi.. | 1 | 0 | 0.04 | 1 |
| GO:0070900 | mitochondrial tRNA modification | 1 | 0 | 0.04 | 1 |
| GO:0070904 | transepithelial L-ascorbic acid transpor... | 2 | 0 | 0.08 | 1 |
| GO:0070914 | UV-damage excision repair | 9 | 0 | 0.34 | 1 |
| GO:0070922 | small RNA loading onto RISC | 4 | 0 | 0.15 | 1 |
| GO:0070926 | regulation of ATP:ADP antiporter activit... | 1 | 0 | 0.04 | 1 |
| GO:0070934 | CRD-mediated mRNA stabilization | 5 | 0 | 0.19 | 1 |
| GO:0070940 | dephosphorylation of RNA polymerase II C | 4 | 0 | 0.15 | 1 |
| GO:0070942 | neutrophil mediated cytotoxicity | 6 | 0 | 0.23 | 1 |
| GO:0070943 | neutrophil mediated killing of symbiont ... | 6 | 0 | 0.23 | 1 |
| GO:0070944 | neutrophil mediated killing of bacterium | 5 | 0 | 0.19 | 1 |
| GO:0070945 | neutrophil mediated killing of gram-nega... | 3 | 0 | 0.11 | 1 |
| GO:0070946 | neutrophil mediated killing of gram-posi... | 2 | 0 | 0.08 | 1 |
| GO:0070947 | neutrophil mediated killing of fungus | 2 | 0 | 0.08 | 1 |
| GO:0070948 | regulation of neutrophil mediated cytoto... | 1 | 0 | 0.04 | 1 |
| GO:0070949 | regulation of neutrophil mediated killin... | 1 | 0 | 0.04 | 1 |
| GO:0070950 | regulation of neutrophil mediated killin... | 1 | 0 | 0.04 | 1 |
| GO:0070951 | regulation of neutrophil mediated killin... | 1 | 0 | 0.04 | 1 |
| GO:0070960 | positive regulation of neutrophil mediat... | 1 | 0 | 0.04 | 1 |
| GO:0070961 | positive regulation of neutrophil mediat... | 1 | 0 | 0.04 | 1 |
| GO:0070962 | positive regulation of neutrophil mediat... | 1 | 0 | 0.04 | 1 |
| GO:0070963 | positive regulation of neutrophil mediat... | 1 | 0 | 0.04 | 1 |
| GO:0070966 | nuclear-transcribed mRNA catabolic proce | 3 | 0 | 0.11 | 1 |
| GO:0070970 | interleukin-2 secretion | 8 | 0 | 0.3 | 1 |
| GO:0070973 | protein localization to endoplasmic reti... | 3 | 0 | 0.11 | 1 |
| GO:0070980 | biphenyl catabolic process | 1 | 0 | 0.04 | 1 |
| GO:0070981 | L-asparagine biosynthetic process | 1 | 0 | 0.04 | 1 |
| GO:0070982 | L-asparagine metabolic process | 1 | 0 | 0.04 | 1 |
| GO:0070988 | demethylation | 51 | 0 | 1.94 | 1 |
| GO:0070989 | oxidative demethylation | 13 | 0 | 0.49 | 1 |
| GO:0070994 | detection of oxidative stress | 1 | 0 | 0.04 | 1 |
| GO:0070995 | NADPH oxidation | 4 | 0 | 0.15 | 1 |
| GO:0071025 | RNA surveillance | 8 | 0 | 0.3 | 1 |
| GO:0071026 | cytoplasmic RNA surveillance | 1 | 0 | 0.04 | 1 |
| GO:0071027 | nuclear RNA surveillance | 6 | 0 | 0.23 | 1 |
| GO:0071028 | nuclear mRNA surveillance | 6 | 0 | 0.23 | 1 |
| GO:0071029 | nuclear ncRNA surveillance | 4 | 0 | 0.15 | 1 |
| GO:0071030 | nuclear mRNA surveillance of spliceosoma | 1 | 0 | 0.04 | 1 |
| GO:0071031 | nuclear mRNA surveillance of mRNA 3'-en | 2 | 0 | 0.08 | 1 |
| GO:0071033 | nuclear retention of pre-mRNA at the sit... | 3 | 0 | 0.11 | 1 |
| GO:0071034 | CUT catabolic process | 4 | 0 | 0.15 | 1 |
| GO:0071035 | nuclear polyadenylation-dependent rRNA c | 4 | 0 | 0.15 | 1 |
| GO:0071038 | nuclear polyadenylation-dependent tRNA c | 2 | 0 | 0.08 | 1 |
| GO:0071043 | CUT metabolic process | 4 | 0 | 0.15 | 1 |
| GO:0071044 | histone mRNA catabolic process | 10 | 0 | 0.38 | 1 |
| GO:0071046 | nuclear polyadenylation-dependent ncRNA | 4 | 0 | 0.15 | 1 |
| GO:0071048 | nuclear retention of unspliced pre-mRNA .. | 1 | 0 | 0.04 | 1 |
| GO:0071049 | nuclear retention of pre-mRNA with aberr.. | 2 | 0 | 0.08 | 1 |
| GO:0071051 | polyadenylation-dependent snoRNA 3'-enc | 2 | 0 | 0.08 | 1 |
| GO:0071071 | regulation of phospholipid biosynthetic ... | 11 | 0 | 0.42 | 1 |

| | | | | | |
|---|---|---|---|---|---|
| GO:0071072 | negative regulation of phospholipid bios... | 4 | 0 | 0.15 | 1 |
| GO:0071073 | positive regulation of phospholipid bios... | 7 | 0 | 0.27 | 1 |
| GO:0071104 | response to interleukin-9 | 3 | 0 | 0.11 | 1 |
| GO:0071105 | response to interleukin-11 | 1 | 0 | 0.04 | 1 |
| GO:0071106 | adenosine 3' | 5'-bisphosp | 2 | 0 | 0.08 |
| GO:0071109 | superior temporal gyrus development | 2 | 0 | 0.08 | 1 |
| GO:0071110 | histone biotinylation | 1 | 0 | 0.04 | 1 |
| GO:0071139 | resolution of recombination intermediate... | 2 | 0 | 0.08 | 1 |
| GO:0071140 | resolution of mitotic recombination inte... | 2 | 0 | 0.08 | 1 |
| GO:0071167 | ribonucleoprotein complex import into nu... | 2 | 0 | 0.08 | 1 |
| GO:0071168 | protein localization to chromatin | 14 | 0 | 0.53 | 1 |
| GO:0071169 | establishment of protein localization to... | 6 | 0 | 0.23 | 1 |
| GO:0071205 | protein localization to juxtaparanode re... | 4 | 0 | 0.15 | 1 |
| GO:0071206 | establishment of protein localization to... | 1 | 0 | 0.04 | 1 |
| GO:0071215 | cellular response to abscisic acid stimu... | 1 | 0 | 0.04 | 1 |
| GO:0071218 | cellular response to misfolded protein | 8 | 0 | 0.3 | 1 |
| GO:0071220 | cellular response to bacterial lipoprote... | 5 | 0 | 0.19 | 1 |
| GO:0071221 | cellular response to bacterial lipopepti... | 5 | 0 | 0.19 | 1 |
| GO:0071223 | cellular response to lipoteichoic acid | 9 | 0 | 0.34 | 1 |
| GO:0071224 | cellular response to peptidoglycan | 5 | 0 | 0.19 | 1 |
| GO:0071225 | cellular response to muramyl dipeptide | 3 | 0 | 0.11 | 1 |
| GO:0071226 | cellular response to molecule of fungal ... | 2 | 0 | 0.08 | 1 |
| GO:0071228 | cellular response to tumor cell | 1 | 0 | 0.04 | 1 |
| GO:0071233 | cellular response to leucine | 2 | 0 | 0.08 | 1 |
| GO:0071247 | cellular response to chromate | 1 | 0 | 0.04 | 1 |
| GO:0071250 | cellular response to nitrite | 1 | 0 | 0.04 | 1 |
| GO:0071257 | cellular response to electrical stimulus | 10 | 0 | 0.38 | 1 |
| GO:0071259 | cellular response to magnetism | 1 | 0 | 0.04 | 1 |
| GO:0071268 | homocysteine biosynthetic process | 1 | 0 | 0.04 | 1 |
| GO:0071275 | cellular response to aluminum ion | 1 | 0 | 0.04 | 1 |
| GO:0071276 | cellular response to cadmium ion | 15 | 0 | 0.57 | 1 |
| GO:0071279 | cellular response to cobalt ion | 2 | 0 | 0.08 | 1 |
| GO:0071280 | cellular response to copper ion | 5 | 0 | 0.19 | 1 |
| GO:0071281 | cellular response to iron ion | 6 | 0 | 0.23 | 1 |
| GO:0071283 | cellular response to iron(III) ion | 1 | 0 | 0.04 | 1 |
| GO:0071285 | cellular response to lithium ion | 16 | 0 | 0.61 | 1 |
| GO:0071286 | cellular response to magnesium ion | 4 | 0 | 0.15 | 1 |
| GO:0071287 | cellular response to manganese ion | 5 | 0 | 0.19 | 1 |
| GO:0071288 | cellular response to mercury ion | 3 | 0 | 0.11 | 1 |
| GO:0071294 | cellular response to zinc ion | 13 | 0 | 0.49 | 1 |
| GO:0071305 | cellular response to vitamin D | 14 | 0 | 0.53 | 1 |
| GO:0071306 | cellular response to vitamin E | 2 | 0 | 0.08 | 1 |
| GO:0071307 | cellular response to vitamin K | 1 | 0 | 0.04 | 1 |
| GO:0071313 | cellular response to caffeine | 7 | 0 | 0.27 | 1 |
| GO:0071314 | cellular response to cocaine | 2 | 0 | 0.08 | 1 |
| GO:0071316 | cellular response to nicotine | 8 | 0 | 0.3 | 1 |
| GO:0071321 | cellular response to cGMP | 6 | 0 | 0.23 | 1 |
| GO:0071324 | cellular response to disaccharide stimul... | 1 | 0 | 0.04 | 1 |
| GO:0071329 | cellular response to sucrose stimulus | 1 | 0 | 0.04 | 1 |
| GO:0071332 | cellular response to fructose stimulus | 3 | 0 | 0.11 | 1 |
| GO:0071335 | hair follicle cell proliferation | 4 | 0 | 0.15 | 1 |
| GO:0071336 | regulation of hair follicle cell prolife... | 3 | 0 | 0.11 | 1 |
| GO:0071338 | positive regulation of hair follicle cel... | 2 | 0 | 0.08 | 1 |

| | | | | | |
|---|---|---|---|---|---|
| GO:0071340 | skeletal muscle acetylcholine-gated chan.. | 7 | 0 | 0.27 | 1 |
| GO:0071344 | diphosphate metabolic process | 2 | 0 | 0.08 | 1 |
| GO:0071349 | cellular response to interleukin-12 | 3 | 0 | 0.11 | 1 |
| GO:0071350 | cellular response to interleukin-15 | 1 | 0 | 0.04 | 1 |
| GO:0071351 | cellular response to interleukin-18 | 3 | 0 | 0.11 | 1 |
| GO:0071355 | cellular response to interleukin-9 | 1 | 0 | 0.04 | 1 |
| GO:0071360 | cellular response to exogenous dsRNA | 6 | 0 | 0.23 | 1 |
| GO:0071361 | cellular response to ethanol | 8 | 0 | 0.3 | 1 |
| GO:0071364 | cellular response to epidermal growth fa... | 20 | 0 | 0.76 | 1 |
| GO:0071371 | cellular response to gonadotropin stimul... | 17 | 0 | 0.65 | 1 |
| GO:0071372 | cellular response to follicle-stimulatin... | 8 | 0 | 0.3 | 1 |
| GO:0071373 | cellular response to luteinizing hormone... | 1 | 0 | 0.04 | 1 |
| GO:0071376 | cellular response to corticotropin-relea... | 2 | 0 | 0.08 | 1 |
| GO:0071379 | cellular response to prostaglandin stimu... | 15 | 0 | 0.57 | 1 |
| GO:0071380 | cellular response to prostaglandin E sti... | 9 | 0 | 0.34 | 1 |
| GO:0071387 | cellular response to cortisol stimulus | 1 | 0 | 0.04 | 1 |
| GO:0071393 | cellular response to progesterone stimul... | 1 | 0 | 0.04 | 1 |
| GO:0071395 | cellular response to jasmonic acid stimu... | 4 | 0 | 0.15 | 1 |
| GO:0071400 | cellular response to oleic acid | 3 | 0 | 0.11 | 1 |
| GO:0071401 | cellular response to triglyceride | 1 | 0 | 0.04 | 1 |
| GO:0071404 | cellular response to low-density lipopro... | 3 | 0 | 0.11 | 1 |
| GO:0071409 | cellular response to cycloheximide | 2 | 0 | 0.08 | 1 |
| GO:0071412 | cellular response to genistein | 1 | 0 | 0.04 | 1 |
| GO:0071413 | cellular response to hydroxyisoflavone | 1 | 0 | 0.04 | 1 |
| GO:0071415 | cellular response to purine-containing c... | 7 | 0 | 0.27 | 1 |
| GO:0071418 | cellular response to amine stimulus | 2 | 0 | 0.08 | 1 |
| GO:0071421 | manganese ion transmembrane transport | 5 | 0 | 0.19 | 1 |
| GO:0071422 | succinate transmembrane transport | 1 | 0 | 0.04 | 1 |
| GO:0071425 | hematopoietic stem cell proliferation | 16 | 0 | 0.61 | 1 |
| GO:0071435 | potassium ion export | 10 | 0 | 0.38 | 1 |
| GO:0071436 | sodium ion export | 10 | 0 | 0.38 | 1 |
| GO:0071440 | regulation of histone H3-K14 acetylation | 3 | 0 | 0.11 | 1 |
| GO:0071441 | negative regulation of histone H3-K14 ac... | 1 | 0 | 0.04 | 1 |
| GO:0071442 | positive regulation of histone H3-K14 ac... | 2 | 0 | 0.08 | 1 |
| GO:0071447 | cellular response to hydroperoxide | 5 | 0 | 0.19 | 1 |
| GO:0071449 | cellular response to lipid hydroperoxide | 1 | 0 | 0.04 | 1 |
| GO:0071450 | cellular response to oxygen radical | 19 | 0 | 0.72 | 1 |
| GO:0071451 | cellular response to superoxide | 19 | 0 | 0.72 | 1 |
| GO:0071455 | cellular response to hyperoxia | 7 | 0 | 0.27 | 1 |
| GO:0071459 | protein localization to chromosome | cent... | 9 | 0 | 0.34 |
| GO:0071460 | cellular response to cell-matrix adhesio... | 2 | 0 | 0.08 | 1 |
| GO:0071461 | cellular response to redox state | 1 | 0 | 0.04 | 1 |
| GO:0071462 | cellular response to water stimulus | 6 | 0 | 0.23 | 1 |
| GO:0071464 | cellular response to hydrostatic pressur... | 3 | 0 | 0.11 | 1 |
| GO:0071467 | cellular response to pH | 14 | 0 | 0.53 | 1 |
| GO:0071468 | cellular response to acidic pH | 6 | 0 | 0.23 | 1 |
| GO:0071469 | cellular response to alkaline pH | 2 | 0 | 0.08 | 1 |
| GO:0071472 | cellular response to salt stress | 5 | 0 | 0.19 | 1 |
| GO:0071474 | cellular hyperosmotic response | 7 | 0 | 0.27 | 1 |
| GO:0071475 | cellular hyperosmotic salinity response | 2 | 0 | 0.08 | 1 |
| GO:0071477 | cellular hypotonic salinity response | 1 | 0 | 0.04 | 1 |
| GO:0071481 | cellular response to X-ray | 7 | 0 | 0.27 | 1 |
| GO:0071483 | cellular response to blue light | 2 | 0 | 0.08 | 1 |

| | | | | | |
|---|---|---|---|---|---|
| GO:0071489 | cellular response to red or far red ligh... | 1 | 0 | 0.04 | 1 |
| GO:0071493 | cellular response to UV-B | 8 | 0 | 0.3 | 1 |
| GO:0071497 | cellular response to freezing | 1 | 0 | 0.04 | 1 |
| GO:0071498 | cellular response to fluid shear stress | 14 | 0 | 0.53 | 1 |
| GO:0071499 | cellular response to laminar fluid shear... | 8 | 0 | 0.3 | 1 |
| GO:0071502 | cellular response to temperature stimulu... | 2 | 0 | 0.08 | 1 |
| GO:0071503 | response to heparin | 5 | 0 | 0.19 | 1 |
| GO:0071504 | cellular response to heparin | 4 | 0 | 0.15 | 1 |
| GO:0071505 | response to mycophenolic acid | 2 | 0 | 0.08 | 1 |
| GO:0071506 | cellular response to mycophenolic acid | 2 | 0 | 0.08 | 1 |
| GO:0071514 | genetic imprinting | 27 | 0 | 1.03 | 1 |
| GO:0071526 | semaphorin-plexin signaling pathway | 20 | 0 | 0.76 | 1 |
| GO:0071527 | semaphorin-plexin signaling pathway invo. | 1 | 0 | 0.04 | 1 |
| GO:0071529 | cementum mineralization | 1 | 0 | 0.04 | 1 |
| GO:0071539 | protein localization to centrosome | 7 | 0 | 0.27 | 1 |
| GO:0071543 | diphosphoinositol polyphosphate metaboli. | 1 | 0 | 0.04 | 1 |
| GO:0071544 | diphosphoinositol polyphosphate cataboli.. | 1 | 0 | 0.04 | 1 |
| GO:0071550 | death-inducing signaling complex assembl | 2 | 0 | 0.08 | 1 |
| GO:0071554 | cell wall organization or biogenesis | 5 | 0 | 0.19 | 1 |
| GO:0071557 | histone H3-K27 demethylation | 3 | 0 | 0.11 | 1 |
| GO:0071569 | protein ufmylation | 5 | 0 | 0.19 | 1 |
| GO:0071577 | zinc II ion transmembrane transport | 16 | 0 | 0.61 | 1 |
| GO:0071578 | zinc II ion transmembrane import | 4 | 0 | 0.15 | 1 |
| GO:0071579 | regulation of zinc ion transport | 2 | 0 | 0.08 | 1 |
| GO:0071580 | regulation of zinc ion transmembrane tra... | 2 | 0 | 0.08 | 1 |
| GO:0071581 | regulation of zinc ion transmembrane imp.. | 1 | 0 | 0.04 | 1 |
| GO:0071582 | negative regulation of zinc ion transpor... | 2 | 0 | 0.08 | 1 |
| GO:0071583 | negative regulation of zinc ion transmem... | 2 | 0 | 0.08 | 1 |
| GO:0071584 | negative regulation of zinc ion transmem... | 1 | 0 | 0.04 | 1 |
| GO:0071585 | detoxification of cadmium ion | 1 | 0 | 0.04 | 1 |
| GO:0071586 | CAAX-box protein processing | 2 | 0 | 0.08 | 1 |
| GO:0071594 | thymocyte aggregation | 8 | 0 | 0.3 | 1 |
| GO:0071602 | phytosphingosine biosynthetic process | 1 | 0 | 0.04 | 1 |
| GO:0071603 | endothelial cell-cell adhesion | 3 | 0 | 0.11 | 1 |
| GO:0071605 | monocyte chemotactic protein-1 productio. | 6 | 0 | 0.23 | 1 |
| GO:0071608 | macrophage inflammatory protein-1 alpha | 2 | 0 | 0.08 | 1 |
| GO:0071609 | chemokine (C-C motif) ligand 5 productio.. | 5 | 0 | 0.19 | 1 |
| GO:0071611 | granulocyte colony-stimulating factor pr... | 2 | 0 | 0.08 | 1 |
| GO:0071613 | granzyme B production | 1 | 0 | 0.04 | 1 |
| GO:0071615 | oxidative deethylation | 1 | 0 | 0.04 | 1 |
| GO:0071619 | phosphorylation of RNA polymerase II C-t. | 1 | 0 | 0.04 | 1 |
| GO:0071623 | negative regulation of granulocyte chemo.. | 1 | 0 | 0.04 | 1 |
| GO:0071629 | cytoplasm-associated proteasomal ubiquit. | 1 | 0 | 0.04 | 1 |
| GO:0071635 | negative regulation of transforming grow... | 6 | 0 | 0.23 | 1 |
| GO:0071637 | regulation of monocyte chemotactic prote.. | 6 | 0 | 0.23 | 1 |
| GO:0071638 | negative regulation of monocyte chemotac | 3 | 0 | 0.11 | 1 |
| GO:0071639 | positive regulation of monocyte chemotac. | 3 | 0 | 0.11 | 1 |
| GO:0071640 | regulation of macrophage inflammatory pr. | 1 | 0 | 0.04 | 1 |
| GO:0071641 | negative regulation of macrophage inflam.. | 1 | 0 | 0.04 | 1 |
| GO:0071649 | regulation of chemokine (C-C motif) liga... | 4 | 0 | 0.15 | 1 |
| GO:0071650 | negative regulation of chemokine (C-C mo | 2 | 0 | 0.08 | 1 |
| GO:0071651 | positive regulation of chemokine (C-C mo.. | 3 | 0 | 0.11 | 1 |
| GO:0071655 | regulation of granulocyte colony-stimula... | 2 | 0 | 0.08 | 1 |

| | | | | | |
|---|---|---|---|---|---|
| GO:0071657 | positive regulation of granulocyte colon... | 2 | 0 | 0.08 | 1 |
| GO:0071661 | regulation of granzyme B production | 1 | 0 | 0.04 | 1 |
| GO:0071663 | positive regulation of granzyme B produc.. | 1 | 0 | 0.04 | 1 |
| GO:0071670 | smooth muscle cell chemotaxis | 5 | 0 | 0.19 | 1 |
| GO:0071671 | regulation of smooth muscle cell chemota. | 3 | 0 | 0.11 | 1 |
| GO:0071672 | negative regulation of smooth muscle cel.. | 2 | 0 | 0.08 | 1 |
| GO:0071673 | positive regulation of smooth muscle cel... | 2 | 0 | 0.08 | 1 |
| GO:0071676 | negative regulation of mononuclear cell ... | 2 | 0 | 0.08 | 1 |
| GO:0071677 | positive regulation of mononuclear cell ... | 3 | 0 | 0.11 | 1 |
| GO:0071678 | olfactory bulb axon guidance | 1 | 0 | 0.04 | 1 |
| GO:0071679 | commissural neuron axon guidance | 7 | 0 | 0.27 | 1 |
| GO:0071680 | response to indole-3-methanol | 5 | 0 | 0.19 | 1 |
| GO:0071681 | cellular response to indole-3-methanol | 5 | 0 | 0.19 | 1 |
| GO:0071684 | organism emergence from protective struc | 4 | 0 | 0.15 | 1 |
| GO:0071688 | striated muscle myosin thick filament as... | 3 | 0 | 0.11 | 1 |
| GO:0071691 | cardiac muscle thin filament assembly | 1 | 0 | 0.04 | 1 |
| GO:0071698 | olfactory placode development | 6 | 0 | 0.23 | 1 |
| GO:0071699 | olfactory placode morphogenesis | 6 | 0 | 0.23 | 1 |
| GO:0071701 | regulation of MAPK export from nucleus | 1 | 0 | 0.04 | 1 |
| GO:0071712 | ER-associated misfolded protein cataboli.. | 3 | 0 | 0.11 | 1 |
| GO:0071724 | response to diacyl bacterial lipopeptide | 2 | 0 | 0.08 | 1 |
| GO:0071725 | response to triacyl bacterial lipopeptid... | 2 | 0 | 0.08 | 1 |
| GO:0071726 | cellular response to diacyl bacterial li... | 2 | 0 | 0.08 | 1 |
| GO:0071727 | cellular response to triacyl bacterial l... | 2 | 0 | 0.08 | 1 |
| GO:0071731 | response to nitric oxide | 14 | 0 | 0.53 | 1 |
| GO:0071732 | cellular response to nitric oxide | 11 | 0 | 0.42 | 1 |
| GO:0071733 | transcriptional activation by promoter-e... | 3 | 0 | 0.11 | 1 |
| GO:0071763 | nuclear membrane organization | 5 | 0 | 0.19 | 1 |
| GO:0071765 | nuclear inner membrane organization | 1 | 0 | 0.04 | 1 |
| GO:0071772 | response to BMP | 21 | 0 | 0.8 | 1 |
| GO:0071773 | cellular response to BMP stimulus | 21 | 0 | 0.8 | 1 |
| GO:0071798 | response to prostaglandin D | 5 | 0 | 0.19 | 1 |
| GO:0071799 | cellular response to prostaglandin D sti... | 5 | 0 | 0.19 | 1 |
| GO:0071800 | podosome assembly | 13 | 0 | 0.49 | 1 |
| GO:0071801 | regulation of podosome assembly | 9 | 0 | 0.34 | 1 |
| GO:0071803 | positive regulation of podosome assembly | 6 | 0 | 0.23 | 1 |
| GO:0071810 | regulation of fever generation by regula... | 2 | 0 | 0.08 | 1 |
| GO:0071812 | positive regulation of fever generation ... | 2 | 0 | 0.08 | 1 |
| GO:0071823 | protein-carbohydrate complex subunit org. | 2 | 0 | 0.08 | 1 |
| GO:0071825 | protein-lipid complex subunit organizati... | 35 | 0 | 1.33 | 1 |
| GO:0071827 | plasma lipoprotein particle organization | 33 | 0 | 1.26 | 1 |
| GO:0071830 | triglyceride-rich lipoprotein particle c... | 7 | 0 | 0.27 | 1 |
| GO:0071838 | cell proliferation in bone marrow | 4 | 0 | 0.15 | 1 |
| GO:0071839 | apoptotic process in bone marrow | 3 | 0 | 0.11 | 1 |
| GO:0071847 | TNFSF11-mediated signaling pathway | 2 | 0 | 0.08 | 1 |
| GO:0071848 | positive regulation of ERK1 and ERK2 cas | 2 | 0 | 0.08 | 1 |
| GO:0071863 | regulation of cell proliferation in bone... | 3 | 0 | 0.11 | 1 |
| GO:0071864 | positive regulation of cell proliferatio... | 3 | 0 | 0.11 | 1 |
| GO:0071865 | regulation of apoptotic process in bone ... | 2 | 0 | 0.08 | 1 |
| GO:0071866 | negative regulation of apoptotic process... | 2 | 0 | 0.08 | 1 |
| GO:0071873 | response to norepinephrine | 5 | 0 | 0.19 | 1 |
| GO:0071874 | cellular response to norepinephrine stim... | 3 | 0 | 0.11 | 1 |
| GO:0071877 | regulation of adrenergic receptor signal... | 6 | 0 | 0.23 | 1 |

| | | | | | |
|---|---|---|---|---|---|
| GO:0071878 | negative regulation of adrenergic recept... | 3 | 0 | 0.11 | 1 |
| GO:0071879 | positive regulation of adrenergic recept... | 3 | 0 | 0.11 | 1 |
| GO:0071881 | adenylate cyclase-inhibiting adrenergic ... | 1 | 0 | 0.04 | 1 |
| GO:0071882 | phospholipase C-activating adrenergic re... | 1 | 0 | 0.04 | 1 |
| GO:0071883 | activation of MAPK activity by adrenergi... | 3 | 0 | 0.11 | 1 |
| GO:0071888 | macrophage apoptotic process | 8 | 0 | 0.3 | 1 |
| GO:0071893 | BMP signaling pathway involved in nephri... | 1 | 0 | 0.04 | 1 |
| GO:0071894 | histone H2B conserved C-terminal lysine ... | 1 | 0 | 0.04 | 1 |
| GO:0071895 | odontoblast differentiation | 3 | 0 | 0.11 | 1 |
| GO:0071896 | protein localization to adherens junctio... | 4 | 0 | 0.15 | 1 |
| GO:0071898 | regulation of estrogen receptor binding | 3 | 0 | 0.11 | 1 |
| GO:0071899 | negative regulation of estrogen receptor... | 3 | 0 | 0.11 | 1 |
| GO:0071907 | determination of digestive tract left/ri... | 5 | 0 | 0.19 | 1 |
| GO:0071908 | determination of intestine left/right as... | 1 | 0 | 0.04 | 1 |
| GO:0071909 | determination of stomach left/right asym... | 1 | 0 | 0.04 | 1 |
| GO:0071910 | determination of liver left/right asymme... | 4 | 0 | 0.15 | 1 |
| GO:0071921 | cohesin localization to chromatin | 4 | 0 | 0.15 | 1 |
| GO:0071922 | regulation of cohesin localization to ch... | 4 | 0 | 0.15 | 1 |
| GO:0071926 | endocannabinoid signaling pathway | 3 | 0 | 0.11 | 1 |
| GO:0071930 | negative regulation of transcription inv... | 3 | 0 | 0.11 | 1 |
| GO:0071931 | positive regulation of transcription inv... | 1 | 0 | 0.04 | 1 |
| GO:0071934 | thiamine transmembrane transport | 3 | 0 | 0.11 | 1 |
| GO:0071938 | vitamin A transport | 1 | 0 | 0.04 | 1 |
| GO:0071939 | vitamin A import | 1 | 0 | 0.04 | 1 |
| GO:0071941 | nitrogen cycle metabolic process | 15 | 0 | 0.57 | 1 |
| GO:0071947 | protein deubiquitination involved in ubi... | 1 | 0 | 0.04 | 1 |
| GO:0071951 | conversion of methionyl-tRNA to N-formyl... | 1 | 0 | 0.04 | 1 |
| GO:0071954 | chemokine (C-C motif) ligand 11 producti... | 2 | 0 | 0.08 | 1 |
| GO:0071955 | recycling endosome to Golgi transport | 2 | 0 | 0.08 | 1 |
| GO:0071963 | establishment or maintenance of cell pol... | 1 | 0 | 0.04 | 1 |
| GO:0071971 | extracellular vesicular exosome assembly | 7 | 0 | 0.27 | 1 |
| GO:0071976 | cell gliding | 1 | 0 | 0.04 | 1 |
| GO:0072003 | kidney rudiment formation | 3 | 0 | 0.11 | 1 |
| GO:0072004 | kidney field specification | 2 | 0 | 0.08 | 1 |
| GO:0072007 | mesangial cell differentiation | 6 | 0 | 0.23 | 1 |
| GO:0072008 | glomerular mesangial cell differentiatio... | 5 | 0 | 0.19 | 1 |
| GO:0072009 | nephron epithelium development | 111 | 0 | 4.23 | 1 |
| GO:0072010 | glomerular epithelium development | 20 | 0 | 0.76 | 1 |
| GO:0072011 | glomerular endothelium development | 4 | 0 | 0.15 | 1 |
| GO:0072012 | glomerulus vasculature development | 20 | 0 | 0.76 | 1 |
| GO:0072014 | proximal tubule development | 6 | 0 | 0.23 | 1 |
| GO:0072015 | glomerular visceral epithelial cell deve... | 9 | 0 | 0.34 | 1 |
| GO:0072016 | glomerular parietal epithelial cell deve... | 1 | 0 | 0.04 | 1 |
| GO:0072017 | distal tubule development | 12 | 0 | 0.46 | 1 |
| GO:0072019 | proximal convoluted tubule development | 2 | 0 | 0.08 | 1 |
| GO:0072020 | proximal straight tubule development | 1 | 0 | 0.04 | 1 |
| GO:0072021 | ascending thin limb development | 4 | 0 | 0.15 | 1 |
| GO:0072022 | descending thin limb development | 1 | 0 | 0.04 | 1 |
| GO:0072023 | thick ascending limb development | 2 | 0 | 0.08 | 1 |
| GO:0072024 | macula densa development | 1 | 0 | 0.04 | 1 |
| GO:0072025 | distal convoluted tubule development | 5 | 0 | 0.19 | 1 |
| GO:0072027 | connecting tubule development | 1 | 0 | 0.04 | 1 |
| GO:0072028 | nephron morphogenesis | 79 | 0 | 3.01 | 1 |

| | | | | | |
|---|---|---|---|---|---|
| GO:0072032 | proximal convoluted tubule segment 2 dev | 1 | 0 | 0.04 | 1 |
| GO:0072033 | renal vesicle formation | 7 | 0 | 0.27 | 1 |
| GO:0072034 | renal vesicle induction | 3 | 0 | 0.11 | 1 |
| GO:0072036 | mesenchymal to epithelial transition inv... | 1 | 0 | 0.04 | 1 |
| GO:0072038 | mesenchymal stem cell maintenance invol | 6 | 0 | 0.23 | 1 |
| GO:0072039 | regulation of mesenchymal cell apoptotic... | 4 | 0 | 0.15 | 1 |
| GO:0072040 | negative regulation of mesenchymal cell ... | 4 | 0 | 0.15 | 1 |
| GO:0072044 | collecting duct development | 13 | 0 | 0.49 | 1 |
| GO:0072046 | establishment of planar polarity involve... | 1 | 0 | 0.04 | 1 |
| GO:0072047 | proximal/distal pattern formation involv... | 5 | 0 | 0.19 | 1 |
| GO:0072048 | renal system pattern specification | 9 | 0 | 0.34 | 1 |
| GO:0072049 | comma-shaped body morphogenesis | 6 | 0 | 0.23 | 1 |
| GO:0072050 | S-shaped body morphogenesis | 8 | 0 | 0.3 | 1 |
| GO:0072051 | juxtaglomerular apparatus development | 1 | 0 | 0.04 | 1 |
| GO:0072053 | renal inner medulla development | 2 | 0 | 0.08 | 1 |
| GO:0072054 | renal outer medulla development | 2 | 0 | 0.08 | 1 |
| GO:0072055 | renal cortex development | 1 | 0 | 0.04 | 1 |
| GO:0072059 | cortical collecting duct development | 1 | 0 | 0.04 | 1 |
| GO:0072060 | outer medullary collecting duct developm... | 1 | 0 | 0.04 | 1 |
| GO:0072061 | inner medullary collecting duct developm... | 2 | 0 | 0.08 | 1 |
| GO:0072069 | DCT cell differentiation | 3 | 0 | 0.11 | 1 |
| GO:0072070 | loop of Henle development | 12 | 0 | 0.46 | 1 |
| GO:0072071 | kidney interstitial fibroblast different... | 7 | 0 | 0.27 | 1 |
| GO:0072074 | kidney mesenchyme development | 18 | 0 | 0.69 | 1 |
| GO:0072075 | metanephric mesenchyme development | 15 | 0 | 0.57 | 1 |
| GO:0072076 | nephrogenic mesenchyme development | 3 | 0 | 0.11 | 1 |
| GO:0072077 | renal vesicle morphogenesis | 19 | 0 | 0.72 | 1 |
| GO:0072078 | nephron tubule morphogenesis | 74 | 0 | 2.82 | 1 |
| GO:0072079 | nephron tubule formation | 19 | 0 | 0.72 | 1 |
| GO:0072080 | nephron tubule development | 95 | 0 | 3.62 | 1 |
| GO:0072081 | specification of nephron tubule identity | 5 | 0 | 0.19 | 1 |
| GO:0072086 | specification of loop of Henle identity | 4 | 0 | 0.15 | 1 |
| GO:0072087 | renal vesicle development | 20 | 0 | 0.76 | 1 |
| GO:0072088 | nephron epithelium morphogenesis | 77 | 0 | 2.93 | 1 |
| GO:0072092 | ureteric bud invasion | 2 | 0 | 0.08 | 1 |
| GO:0072093 | metanephric renal vesicle formation | 3 | 0 | 0.11 | 1 |
| GO:0072095 | regulation of branch elongation involved... | 4 | 0 | 0.15 | 1 |
| GO:0072096 | negative regulation of branch elongation... | 1 | 0 | 0.04 | 1 |
| GO:0072097 | negative regulation of branch elongation... | 1 | 0 | 0.04 | 1 |
| GO:0072098 | anterior/posterior pattern specification... | 2 | 0 | 0.08 | 1 |
| GO:0072099 | anterior/posterior pattern specification... | 1 | 0 | 0.04 | 1 |
| GO:0072100 | specification of ureteric bud anterior/p... | 1 | 0 | 0.04 | 1 |
| GO:0072101 | specification of ureteric bud anterior/p... | 1 | 0 | 0.04 | 1 |
| GO:0072102 | glomerulus morphogenesis | 8 | 0 | 0.3 | 1 |
| GO:0072103 | glomerulus vasculature morphogenesis | 5 | 0 | 0.19 | 1 |
| GO:0072104 | glomerular capillary formation | 5 | 0 | 0.19 | 1 |
| GO:0072105 | ureteric peristalsis | 1 | 0 | 0.04 | 1 |
| GO:0072106 | regulation of ureteric bud formation | 4 | 0 | 0.15 | 1 |
| GO:0072107 | positive regulation of ureteric bud form... | 4 | 0 | 0.15 | 1 |
| GO:0072108 | positive regulation of mesenchymal to ep... | 4 | 0 | 0.15 | 1 |
| GO:0072109 | glomerular mesangium development | 11 | 0 | 0.42 | 1 |
| GO:0072110 | glomerular mesangial cell proliferation | 7 | 0 | 0.27 | 1 |
| GO:0072111 | cell proliferation involved in kidney de... | 16 | 0 | 0.61 | 1 |

| | | | | | |
|---|---|---|---|---|---|
| GO:0072112 | glomerular visceral epithelial cell diff... | 16 | 0 | 0.61 | 1 |
| GO:0072114 | pronephros morphogenesis | 2 | 0 | 0.08 | 1 |
| GO:0072124 | regulation of glomerular mesangial cell ... | 6 | 0 | 0.23 | 1 |
| GO:0072125 | negative regulation of glomerular mesang. | 3 | 0 | 0.11 | 1 |
| GO:0072126 | positive regulation of glomerular mesang... | 3 | 0 | 0.11 | 1 |
| GO:0072127 | renal capsule development | 1 | 0 | 0.04 | 1 |
| GO:0072128 | renal capsule morphogenesis | 1 | 0 | 0.04 | 1 |
| GO:0072129 | renal capsule formation | 1 | 0 | 0.04 | 1 |
| GO:0072130 | renal capsule specification | 1 | 0 | 0.04 | 1 |
| GO:0072131 | kidney mesenchyme morphogenesis | 4 | 0 | 0.15 | 1 |
| GO:0072133 | metanephric mesenchyme morphogenesis | 4 | 0 | 0.15 | 1 |
| GO:0072134 | nephrogenic mesenchyme morphogenesis | 2 | 0 | 0.08 | 1 |
| GO:0072135 | kidney mesenchymal cell proliferation | 3 | 0 | 0.11 | 1 |
| GO:0072136 | metanephric mesenchymal cell proliferati.. | 3 | 0 | 0.11 | 1 |
| GO:0072138 | mesenchymal cell proliferation involved ... | 3 | 0 | 0.11 | 1 |
| GO:0072139 | glomerular parietal epithelial cell diff... | 3 | 0 | 0.11 | 1 |
| GO:0072141 | renal interstitial fibroblast developmen... | 6 | 0 | 0.23 | 1 |
| GO:0072143 | mesangial cell development | 5 | 0 | 0.19 | 1 |
| GO:0072144 | glomerular mesangial cell development | 4 | 0 | 0.15 | 1 |
| GO:0072156 | distal tubule morphogenesis | 3 | 0 | 0.11 | 1 |
| GO:0072160 | nephron tubule epithelial cell different... | 16 | 0 | 0.61 | 1 |
| GO:0072161 | mesenchymal cell differentiation involve... | 7 | 0 | 0.27 | 1 |
| GO:0072162 | metanephric mesenchymal cell differentia. | 5 | 0 | 0.19 | 1 |
| GO:0072165 | anterior mesonephric tubule development | 1 | 0 | 0.04 | 1 |
| GO:0072166 | posterior mesonephric tubule development | 2 | 0 | 0.08 | 1 |
| GO:0072167 | specification of mesonephric tubule iden... | 1 | 0 | 0.04 | 1 |
| GO:0072168 | specification of anterior mesonephric tu... | 1 | 0 | 0.04 | 1 |
| GO:0072169 | specification of posterior mesonephric t... | 1 | 0 | 0.04 | 1 |
| GO:0072170 | metanephric tubule development | 24 | 0 | 0.91 | 1 |
| GO:0072171 | mesonephric tubule morphogenesis | 66 | 0 | 2.51 | 1 |
| GO:0072172 | mesonephric tubule formation | 10 | 0 | 0.38 | 1 |
| GO:0072173 | metanephric tubule morphogenesis | 10 | 0 | 0.38 | 1 |
| GO:0072174 | metanephric tubule formation | 6 | 0 | 0.23 | 1 |
| GO:0072176 | nephric duct development | 12 | 0 | 0.46 | 1 |
| GO:0072177 | mesonephric duct development | 9 | 0 | 0.34 | 1 |
| GO:0072178 | nephric duct morphogenesis | 9 | 0 | 0.34 | 1 |
| GO:0072179 | nephric duct formation | 5 | 0 | 0.19 | 1 |
| GO:0072180 | mesonephric duct morphogenesis | 4 | 0 | 0.15 | 1 |
| GO:0072181 | mesonephric duct formation | 2 | 0 | 0.08 | 1 |
| GO:0072182 | regulation of nephron tubule epithelial ... | 14 | 0 | 0.53 | 1 |
| GO:0072183 | negative regulation of nephron tubule ep... | 3 | 0 | 0.11 | 1 |
| GO:0072184 | renal vesicle progenitor cell differenti... | 1 | 0 | 0.04 | 1 |
| GO:0072185 | metanephric cap development | 2 | 0 | 0.08 | 1 |
| GO:0072186 | metanephric cap morphogenesis | 2 | 0 | 0.08 | 1 |
| GO:0072189 | ureter development | 14 | 0 | 0.53 | 1 |
| GO:0072190 | ureter urothelium development | 3 | 0 | 0.11 | 1 |
| GO:0072191 | ureter smooth muscle development | 5 | 0 | 0.19 | 1 |
| GO:0072192 | ureter epithelial cell differentiation | 1 | 0 | 0.04 | 1 |
| GO:0072193 | ureter smooth muscle cell differentiatio... | 5 | 0 | 0.19 | 1 |
| GO:0072194 | kidney smooth muscle tissue development | 4 | 0 | 0.15 | 1 |
| GO:0072195 | kidney smooth muscle cell differentiatio... | 2 | 0 | 0.08 | 1 |
| GO:0072197 | ureter morphogenesis | 4 | 0 | 0.15 | 1 |
| GO:0072198 | mesenchymal cell proliferation involved ... | 4 | 0 | 0.15 | 1 |

| | | | | | |
|---|---|---|---|---|---|
| GO:0072199 | regulation of mesenchymal cell prolifera... | 4 | 0 | 0.15 | 1 |
| GO:0072200 | negative regulation of mesenchymal cell ... | 1 | 0 | 0.04 | 1 |
| GO:0072201 | negative regulation of mesenchymal cell ... | 6 | 0 | 0.23 | 1 |
| GO:0072202 | cell differentiation involved in metanep... | 18 | 0 | 0.69 | 1 |
| GO:0072203 | cell proliferation involved in metanephr... | 11 | 0 | 0.42 | 1 |
| GO:0072204 | cell-cell signaling involved in metaneph... | 2 | 0 | 0.08 | 1 |
| GO:0072205 | metanephric collecting duct development | 9 | 0 | 0.34 | 1 |
| GO:0072206 | metanephric juxtaglomerular apparatus de | 1 | 0 | 0.04 | 1 |
| GO:0072207 | metanephric epithelium development | 29 | 0 | 1.1 | 1 |
| GO:0072208 | metanephric smooth muscle tissue develo | 2 | 0 | 0.08 | 1 |
| GO:0072209 | metanephric mesangial cell differentiati... | 2 | 0 | 0.08 | 1 |
| GO:0072210 | metanephric nephron development | 43 | 0 | 1.64 | 1 |
| GO:0072213 | metanephric capsule development | 1 | 0 | 0.04 | 1 |
| GO:0072214 | metanephric cortex development | 1 | 0 | 0.04 | 1 |
| GO:0072215 | regulation of metanephros development | 23 | 0 | 0.88 | 1 |
| GO:0072216 | positive regulation of metanephros devel... | 14 | 0 | 0.53 | 1 |
| GO:0072217 | negative regulation of metanephros devel.. | 9 | 0 | 0.34 | 1 |
| GO:0072218 | metanephric ascending thin limb developm | 4 | 0 | 0.15 | 1 |
| GO:0072219 | metanephric cortical collecting duct dev... | 1 | 0 | 0.04 | 1 |
| GO:0072220 | metanephric descending thin limb develop | 1 | 0 | 0.04 | 1 |
| GO:0072221 | metanephric distal convoluted tubule dev... | 5 | 0 | 0.19 | 1 |
| GO:0072223 | metanephric glomerular mesangium devel | 5 | 0 | 0.19 | 1 |
| GO:0072224 | metanephric glomerulus development | 17 | 0 | 0.65 | 1 |
| GO:0072227 | metanephric macula densa development | 1 | 0 | 0.04 | 1 |
| GO:0072229 | metanephric proximal convoluted tubule d. | 2 | 0 | 0.08 | 1 |
| GO:0072230 | metanephric proximal straight tubule dev... | 1 | 0 | 0.04 | 1 |
| GO:0072232 | metanephric proximal convoluted tubule s. | 1 | 0 | 0.04 | 1 |
| GO:0072233 | metanephric thick ascending limb develop. | 2 | 0 | 0.08 | 1 |
| GO:0072234 | metanephric nephron tubule development | 22 | 0 | 0.84 | 1 |
| GO:0072235 | metanephric distal tubule development | 7 | 0 | 0.27 | 1 |
| GO:0072236 | metanephric loop of Henle development | 6 | 0 | 0.23 | 1 |
| GO:0072237 | metanephric proximal tubule development | 3 | 0 | 0.11 | 1 |
| GO:0072239 | metanephric glomerulus vasculature devel | 9 | 0 | 0.34 | 1 |
| GO:0072240 | metanephric DCT cell differentiation | 3 | 0 | 0.11 | 1 |
| GO:0072243 | metanephric nephron epithelium developm | 26 | 0 | 0.99 | 1 |
| GO:0072244 | metanephric glomerular epithelium develo. | 4 | 0 | 0.15 | 1 |
| GO:0072248 | metanephric glomerular visceral epitheli... | 3 | 0 | 0.11 | 1 |
| GO:0072249 | metanephric glomerular visceral epitheli... | 3 | 0 | 0.11 | 1 |
| GO:0072254 | metanephric glomerular mesangial cell di.. | 2 | 0 | 0.08 | 1 |
| GO:0072255 | metanephric glomerular mesangial cell de. | 1 | 0 | 0.04 | 1 |
| GO:0072257 | metanephric nephron tubule epithelial ce... | 8 | 0 | 0.3 | 1 |
| GO:0072258 | metanephric interstitial fibroblast diff... | 1 | 0 | 0.04 | 1 |
| GO:0072259 | metanephric interstitial fibroblast deve... | 1 | 0 | 0.04 | 1 |
| GO:0072262 | metanephric glomerular mesangial cell pr.. | 4 | 0 | 0.15 | 1 |
| GO:0072264 | metanephric glomerular endothelium deve | 1 | 0 | 0.04 | 1 |
| GO:0072265 | metanephric capsule morphogenesis | 1 | 0 | 0.04 | 1 |
| GO:0072266 | metanephric capsule formation | 1 | 0 | 0.04 | 1 |
| GO:0072267 | metanephric capsule specification | 1 | 0 | 0.04 | 1 |
| GO:0072268 | pattern specification involved in metane... | 4 | 0 | 0.15 | 1 |
| GO:0072272 | proximal/distal pattern formation involv... | 2 | 0 | 0.08 | 1 |
| GO:0072273 | metanephric nephron morphogenesis | 26 | 0 | 0.99 | 1 |
| GO:0072274 | metanephric glomerular basement membra | 1 | 0 | 0.04 | 1 |
| GO:0072275 | metanephric glomerulus morphogenesis | 3 | 0 | 0.11 | 1 |

| | | | | | |
|---|---|---|---|---|---|
| GO:0072276 | metanephric glomerulus vasculature morpl | 3 | 0 | 0.11 | 1 |
| GO:0072277 | metanephric glomerular capillary formati... | 3 | 0 | 0.11 | 1 |
| GO:0072278 | metanephric comma-shaped body morpho | 4 | 0 | 0.15 | 1 |
| GO:0072282 | metanephric nephron tubule morphogenes | 8 | 0 | 0.3 | 1 |
| GO:0072283 | metanephric renal vesicle morphogenesis | 16 | 0 | 0.61 | 1 |
| GO:0072284 | metanephric S-shaped body morphogenes | 6 | 0 | 0.23 | 1 |
| GO:0072285 | mesenchymal to epithelial transition inv... | 1 | 0 | 0.04 | 1 |
| GO:0072286 | metanephric connecting tubule developme | 1 | 0 | 0.04 | 1 |
| GO:0072287 | metanephric distal tubule morphogenesis | 1 | 0 | 0.04 | 1 |
| GO:0072289 | metanephric nephron tubule formation | 4 | 0 | 0.15 | 1 |
| GO:0072298 | regulation of metanephric glomerulus dev.. | 6 | 0 | 0.23 | 1 |
| GO:0072299 | negative regulation of metanephric glome.. | 1 | 0 | 0.04 | 1 |
| GO:0072300 | positive regulation of metanephric glome... | 5 | 0 | 0.19 | 1 |
| GO:0072301 | regulation of metanephric glomerular mes. | 2 | 0 | 0.08 | 1 |
| GO:0072302 | negative regulation of metanephric glome.. | 1 | 0 | 0.04 | 1 |
| GO:0072303 | positive regulation of glomerular metane... | 1 | 0 | 0.04 | 1 |
| GO:0072304 | regulation of mesenchymal cell apoptotic... | 2 | 0 | 0.08 | 1 |
| GO:0072305 | negative regulation of mesenchymal cell ... | 2 | 0 | 0.08 | 1 |
| GO:0072307 | regulation of metanephric nephron tubule.. | 8 | 0 | 0.3 | 1 |
| GO:0072308 | negative regulation of metanephric nephr.. | 2 | 0 | 0.08 | 1 |
| GO:0072309 | mesenchymal stem cell maintenance invol | 2 | 0 | 0.08 | 1 |
| GO:0072310 | glomerular epithelial cell development | 10 | 0 | 0.38 | 1 |
| GO:0072311 | glomerular epithelial cell differentiati... | 17 | 0 | 0.65 | 1 |
| GO:0072312 | metanephric glomerular epithelial cell d... | 3 | 0 | 0.11 | 1 |
| GO:0072313 | metanephric glomerular epithelial cell d... | 3 | 0 | 0.11 | 1 |
| GO:0072318 | clathrin coat disassembly | 3 | 0 | 0.11 | 1 |
| GO:0072319 | vesicle uncoating | 5 | 0 | 0.19 | 1 |
| GO:0072332 | intrinsic apoptotic signaling pathway by... | 62 | 0 | 2.36 | 1 |
| GO:0072334 | UDP-galactose transmembrane transport | 2 | 0 | 0.08 | 1 |
| GO:0072337 | modified amino acid transport | 22 | 0 | 0.84 | 1 |
| GO:0072338 | cellular lactam metabolic process | 5 | 0 | 0.19 | 1 |
| GO:0072340 | cellular lactam catabolic process | 1 | 0 | 0.04 | 1 |
| GO:0072343 | pancreatic stellate cell proliferation | 2 | 0 | 0.08 | 1 |
| GO:0072347 | response to anesthetic | 1 | 0 | 0.04 | 1 |
| GO:0072355 | histone H3-T3 phosphorylation | 2 | 0 | 0.08 | 1 |
| GO:0072356 | chromosome passenger complex localizati | 2 | 0 | 0.08 | 1 |
| GO:0072361 | regulation of glycolytic process by regu... | 2 | 0 | 0.08 | 1 |
| GO:0072362 | regulation of glycolytic process by nega... | 1 | 0 | 0.04 | 1 |
| GO:0072363 | regulation of glycolytic by positive reg... | 1 | 0 | 0.04 | 1 |
| GO:0072365 | regulation of cellular ketone metabolic ... | 1 | 0 | 0.04 | 1 |
| GO:0072367 | regulation of lipid transport by regulat... | 4 | 0 | 0.15 | 1 |
| GO:0072368 | regulation of lipid transport by negativ... | 2 | 0 | 0.08 | 1 |
| GO:0072369 | regulation of lipid transport by positiv... | 2 | 0 | 0.08 | 1 |
| GO:0072377 | blood coagulation | common pa | 1 | 0 | 0.04 |
| GO:0072378 | blood coagulation | fibrin clot fo | 21 | 0 | 0.8 |
| GO:0072385 | minus-end-directed organelle transport a... | 3 | 0 | 0.11 | 1 |
| GO:0072387 | flavin adenine dinucleotide metabolic pr... | 1 | 0 | 0.04 | 1 |
| GO:0072388 | flavin adenine dinucleotide biosynthetic... | 1 | 0 | 0.04 | 1 |
| GO:0072393 | microtubule anchoring at microtubule org.. | 6 | 0 | 0.23 | 1 |
| GO:0072396 | response to cell cycle checkpoint signal... | 4 | 0 | 0.15 | 1 |
| GO:0072402 | response to DNA integrity checkpoint sig... | 4 | 0 | 0.15 | 1 |
| GO:0072414 | response to mitotic cell cycle checkpoin... | 1 | 0 | 0.04 | 1 |
| GO:0072423 | response to DNA damage checkpoint sign | 4 | 0 | 0.15 | 1 |

| | | | | | |
|---|---|---|---|---|---|
| GO:0072425 | signal transduction involved in G2 DNA d.. | 2 | 0 | 0.08 | 1 |
| GO:0072428 | signal transduction involved in intra-S ... | 1 | 0 | 0.04 | 1 |
| GO:0072429 | response to intra-S DNA damage checkpo | 3 | 0 | 0.11 | 1 |
| GO:0072432 | response to G1 DNA damage checkpoint s | 1 | 0 | 0.04 | 1 |
| GO:0072434 | signal transduction involved in mitotic ... | 2 | 0 | 0.08 | 1 |
| GO:0072497 | mesenchymal stem cell differentiation | 8 | 0 | 0.3 | 1 |
| GO:0072498 | embryonic skeletal joint development | 16 | 0 | 0.61 | 1 |
| GO:0072501 | cellular divalent inorganic anion homeos... | 7 | 0 | 0.27 | 1 |
| GO:0072502 | cellular trivalent inorganic anion homeo... | 6 | 0 | 0.23 | 1 |
| GO:0072513 | positive regulation of secondary heart f... | 4 | 0 | 0.15 | 1 |
| GO:0072520 | seminiferous tubule development | 7 | 0 | 0.27 | 1 |
| GO:0072526 | pyridine-containing compound catabolic p. | 3 | 0 | 0.11 | 1 |
| GO:0072530 | purine-containing compound transmembra | 6 | 0 | 0.23 | 1 |
| GO:0072531 | pyrimidine-containing compound transmen | 11 | 0 | 0.42 | 1 |
| GO:0072535 | tumor necrosis factor (ligand) superfami... | 3 | 0 | 0.11 | 1 |
| GO:0072538 | T-helper 17 type immune response | 13 | 0 | 0.49 | 1 |
| GO:0072539 | T-helper 17 cell differentiation | 11 | 0 | 0.42 | 1 |
| GO:0072540 | T-helper 17 cell lineage commitment | 7 | 0 | 0.27 | 1 |
| GO:0072553 | terminal button organization | 4 | 0 | 0.15 | 1 |
| GO:0072554 | blood vessel lumenization | 2 | 0 | 0.08 | 1 |
| GO:0072560 | type B pancreatic cell maturation | 2 | 0 | 0.08 | 1 |
| GO:0072564 | blood microparticle formation | 1 | 0 | 0.04 | 1 |
| GO:0072566 | chemokine (C-X-C motif) ligand 1 product. | 1 | 0 | 0.04 | 1 |
| GO:0072567 | chemokine (C-X-C motif) ligand 2 product. | 8 | 0 | 0.3 | 1 |
| GO:0072573 | tolerance induction to lipopolysaccharid... | 1 | 0 | 0.04 | 1 |
| GO:0072574 | hepatocyte proliferation | 11 | 0 | 0.42 | 1 |
| GO:0072575 | epithelial cell proliferation involved i... | 11 | 0 | 0.42 | 1 |
| GO:0072576 | liver morphogenesis | 12 | 0 | 0.46 | 1 |
| GO:0072579 | glycine receptor clustering | 1 | 0 | 0.04 | 1 |
| GO:0072584 | caveolin-mediated endocytosis | 10 | 0 | 0.38 | 1 |
| GO:0072592 | oxygen metabolic process | 5 | 0 | 0.19 | 1 |
| GO:0072595 | maintenance of protein localization in o... | 21 | 0 | 0.8 | 1 |
| GO:0072602 | interleukin-4 secretion | 5 | 0 | 0.19 | 1 |
| GO:0072606 | interleukin-8 secretion | 15 | 0 | 0.57 | 1 |
| GO:0072610 | interleukin-12 secretion | 5 | 0 | 0.19 | 1 |
| GO:0072619 | interleukin-21 secretion | 1 | 0 | 0.04 | 1 |
| GO:0072641 | type I interferon secretion | 1 | 0 | 0.04 | 1 |
| GO:0072643 | interferon-gamma secretion | 12 | 0 | 0.46 | 1 |
| GO:0072658 | maintenance of protein location in membr.. | 1 | 0 | 0.04 | 1 |
| GO:0072660 | maintenance of protein location in plasm... | 1 | 0 | 0.04 | 1 |
| GO:0072662 | protein localization to peroxisome | 16 | 0 | 0.61 | 1 |
| GO:0072663 | establishment of protein localization to... | 16 | 0 | 0.61 | 1 |
| GO:0072668 | tubulin complex biogenesis | 7 | 0 | 0.27 | 1 |
| GO:0072672 | neutrophil extravasation | 5 | 0 | 0.19 | 1 |
| GO:0072674 | multinuclear osteoclast differentiation | 3 | 0 | 0.11 | 1 |
| GO:0072675 | osteoclast fusion | 3 | 0 | 0.11 | 1 |
| GO:0072677 | eosinophil migration | 14 | 0 | 0.53 | 1 |
| GO:0072679 | thymocyte migration | 2 | 0 | 0.08 | 1 |
| GO:0072680 | extracellular matrix-dependent thymocyte.. | 1 | 0 | 0.04 | 1 |
| GO:0072681 | fibronectin-dependent thymocyte migratio.. | 1 | 0 | 0.04 | 1 |
| GO:0072682 | eosinophil extravasation | 1 | 0 | 0.04 | 1 |
| GO:0072684 | mitochondrial tRNA 3'-trailer cleavage ... | | 1 | 0 | 0.04 |
| GO:0072695 | regulation of DNA recombination at telom.. | 1 | 0 | 0.04 | 1 |

| | | | | | |
|---|---|---|---|---|---|
| GO:0072697 | protein localization to cell cortex | 1 | 0 | 0.04 | 1 |
| GO:0072702 | response to methyl methanesulfonate | 1 | 0 | 0.04 | 1 |
| GO:0072703 | cellular response to methyl methanesulfo.. | 1 | 0 | 0.04 | 1 |
| GO:0072706 | response to sodium dodecyl sulfate | 1 | 0 | 0.04 | 1 |
| GO:0072707 | cellular response to sodium dodecyl sulf... | 1 | 0 | 0.04 | 1 |
| GO:0072708 | response to sorbitol | 3 | 0 | 0.11 | 1 |
| GO:0072709 | cellular response to sorbitol | 3 | 0 | 0.11 | 1 |
| GO:0072710 | response to hydroxyurea | 2 | 0 | 0.08 | 1 |
| GO:0072711 | cellular response to hydroxyurea | 2 | 0 | 0.08 | 1 |
| GO:0072718 | response to cisplatin | 1 | 0 | 0.04 | 1 |
| GO:0072719 | cellular response to cisplatin | 1 | 0 | 0.04 | 1 |
| GO:0072757 | cellular response to camptothecin | 2 | 0 | 0.08 | 1 |
| GO:0075136 | response to host | 8 | 0 | 0.3 | 1 |
| GO:0075519 | microtubule-dependent intracellular tran... | 1 | 0 | 0.04 | 1 |
| GO:0075521 | microtubule-dependent intracellular tran... | 1 | 0 | 0.04 | 1 |
| GO:0075528 | modulation by virus of host immune respo. | 2 | 0 | 0.08 | 1 |
| GO:0075606 | transport of viral material towards nucl... | 1 | 0 | 0.04 | 1 |
| GO:0075713 | establishment of integrated proviral lat... | 8 | 0 | 0.3 | 1 |
| GO:0075732 | viral penetration into host nucleus | 2 | 0 | 0.08 | 1 |
| GO:0080009 | mRNA methylation | 3 | 0 | 0.11 | 1 |
| GO:0080033 | response to nitrite | 1 | 0 | 0.04 | 1 |
| GO:0080058 | protein deglutathionylation | 1 | 0 | 0.04 | 1 |
| GO:0080111 | DNA demethylation | 15 | 0 | 0.57 | 1 |
| GO:0080120 | CAAX-box protein maturation | 2 | 0 | 0.08 | 1 |
| GO:0080121 | AMP transport | 2 | 0 | 0.08 | 1 |
| GO:0080125 | multicellular structure septum developme.. | 1 | 0 | 0.04 | 1 |
| GO:0080129 | proteasome core complex assembly | 1 | 0 | 0.04 | 1 |
| GO:0080144 | amino acid homeostasis | 1 | 0 | 0.04 | 1 |
| GO:0080147 | root hair cell development | 1 | 0 | 0.04 | 1 |
| GO:0080154 | regulation of fertilization | 3 | 0 | 0.11 | 1 |
| GO:0080163 | regulation of protein serine/threonine p... | 1 | 0 | 0.04 | 1 |
| GO:0080182 | histone H3-K4 trimethylation | 7 | 0 | 0.27 | 1 |
| GO:0080184 | response to phenylpropanoid | 11 | 0 | 0.42 | 1 |
| GO:0085018 | maintenance of symbiont-containing vacuo | 1 | 0 | 0.04 | 1 |
| GO:0085020 | protein K6-linked ubiquitination | 8 | 0 | 0.3 | 1 |
| GO:0085029 | extracellular matrix assembly | 21 | 0 | 0.8 | 1 |
| GO:0085032 | modulation by symbiont of host I-kappaB .. | 1 | 0 | 0.04 | 1 |
| GO:0086014 | atrial cardiac muscle cell action potent... | 9 | 0 | 0.34 | 1 |
| GO:0086015 | SA node cell action potential | 8 | 0 | 0.3 | 1 |
| GO:0086016 | AV node cell action potential | 5 | 0 | 0.19 | 1 |
| GO:0086017 | Purkinje myocyte action potential | 3 | 0 | 0.11 | 1 |
| GO:0086018 | SA node cell to atrial cardiac muscle ce... | 8 | 0 | 0.3 | 1 |
| GO:0086019 | cell-cell signaling involved in cardiac ... | 18 | 0 | 0.69 | 1 |
| GO:0086026 | atrial cardiac muscle cell to AV node ce... | 9 | 0 | 0.34 | 1 |
| GO:0086027 | AV node cell to bundle of His cell signa... | 5 | 0 | 0.19 | 1 |
| GO:0086028 | bundle of His cell to Purkinje myocyte s... | 4 | 0 | 0.15 | 1 |
| GO:0086029 | Purkinje myocyte to ventricular cardiac ... | 5 | 0 | 0.19 | 1 |
| GO:0086036 | regulation of cardiac muscle cell membra.. | 7 | 0 | 0.27 | 1 |
| GO:0086043 | bundle of His cell action potential | 4 | 0 | 0.15 | 1 |
| GO:0086045 | membrane depolarization during AV node ( | 1 | 0 | 0.04 | 1 |
| GO:0086046 | membrane depolarization during SA node | 3 | 0 | 0.11 | 1 |
| GO:0086047 | membrane depolarization during Purkinje . | 2 | 0 | 0.08 | 1 |
| GO:0086048 | membrane depolarization during bundle of | 1 | 0 | 0.04 | 1 |

| | | | | | |
|---|---|---|---|---|---|
| GO:0086050 | membrane repolarization during bundle of. | 1 | 0 | 0.04 | 1 |
| GO:0086052 | membrane repolarization during SA node c | 1 | 0 | 0.04 | 1 |
| GO:0086053 | AV node cell to bundle of His cell commu.. | 1 | 0 | 0.04 | 1 |
| GO:0086054 | bundle of His cell to Purkinje myocyte c... | 1 | 0 | 0.04 | 1 |
| GO:0086066 | atrial cardiac muscle cell to AV node ce... | 9 | 0 | 0.34 | 1 |
| GO:0086067 | AV node cell to bundle of His cell commu.. | 6 | 0 | 0.23 | 1 |
| GO:0086068 | Purkinje myocyte to ventricular cardiac ... | 5 | 0 | 0.19 | 1 |
| GO:0086070 | SA node cell to atrial cardiac muscle ce... | 8 | 0 | 0.3 | 1 |
| GO:0086092 | regulation of the force of heart contrac... | 2 | 0 | 0.08 | 1 |
| GO:0086094 | positive regulation of ryanodine-sensiti... | 1 | 0 | 0.04 | 1 |
| GO:0086097 | phospholipase C-activating angiotensin-a.. | 1 | 0 | 0.04 | 1 |
| GO:0086098 | angiotensin-activated signaling pathway ... | 2 | 0 | 0.08 | 1 |
| GO:0086100 | endothelin receptor signaling pathway | 2 | 0 | 0.08 | 1 |
| GO:0089709 | L-histidine transmembrane transport | 3 | 0 | 0.11 | 1 |
| GO:0089712 | L-aspartate transmembrane transport | 4 | 0 | 0.15 | 1 |
| GO:0089718 | amino acid import across plasma membrai | 1 | 0 | 0.04 | 1 |
| GO:0090010 | transforming growth factor beta receptor... | 1 | 0 | 0.04 | 1 |
| GO:0090024 | negative regulation of neutrophil chemot... | 1 | 0 | 0.04 | 1 |
| GO:0090027 | negative regulation of monocyte chemotax | 3 | 0 | 0.11 | 1 |
| GO:0090032 | negative regulation of steroid hormone b... | 6 | 0 | 0.23 | 1 |
| GO:0090034 | regulation of chaperone-mediated protein.. | 2 | 0 | 0.08 | 1 |
| GO:0090035 | positive regulation of chaperone-mediate... | 2 | 0 | 0.08 | 1 |
| GO:0090036 | regulation of protein kinase C signaling | 14 | 0 | 0.53 | 1 |
| GO:0090037 | positive regulation of protein kinase C ... | 8 | 0 | 0.3 | 1 |
| GO:0090038 | negative regulation of protein kinase C ... | 2 | 0 | 0.08 | 1 |
| GO:0090042 | tubulin deacetylation | 5 | 0 | 0.19 | 1 |
| GO:0090043 | regulation of tubulin deacetylation | 3 | 0 | 0.11 | 1 |
| GO:0090044 | positive regulation of tubulin deacetyla... | 1 | 0 | 0.04 | 1 |
| GO:0090049 | regulation of cell migration involved in... | 16 | 0 | 0.61 | 1 |
| GO:0090050 | positive regulation of cell migration in... | 7 | 0 | 0.27 | 1 |
| GO:0090051 | negative regulation of cell migration in... | 8 | 0 | 0.3 | 1 |
| GO:0090065 | regulation of production of siRNA involv... | 1 | 0 | 0.04 | 1 |
| GO:0090073 | positive regulation of protein homodimer... | 7 | 0 | 0.27 | 1 |
| GO:0090074 | negative regulation of protein homodimer.. | 4 | 0 | 0.15 | 1 |
| GO:0090077 | foam cell differentiation | 31 | 0 | 1.18 | 1 |
| GO:0090080 | positive regulation of MAPKKK cascade by | 6 | 0 | 0.23 | 1 |
| GO:0090081 | regulation of heart induction by regulat... | 3 | 0 | 0.11 | 1 |
| GO:0090082 | positive regulation of heart induction b... | 2 | 0 | 0.08 | 1 |
| GO:0090085 | regulation of protein deubiquitination | 6 | 0 | 0.23 | 1 |
| GO:0090086 | negative regulation of protein deubiquit... | 3 | 0 | 0.11 | 1 |
| GO:0090088 | regulation of oligopeptide transport | 2 | 0 | 0.08 | 1 |
| GO:0090089 | regulation of dipeptide transport | 2 | 0 | 0.08 | 1 |
| GO:0090091 | positive regulation of extracellular mat... | 2 | 0 | 0.08 | 1 |
| GO:0090094 | metanephric cap mesenchymal cell prolife. | 2 | 0 | 0.08 | 1 |
| GO:0090095 | regulation of metanephric cap mesenchym | 1 | 0 | 0.04 | 1 |
| GO:0090096 | positive regulation of metanephric cap m... | 1 | 0 | 0.04 | 1 |
| GO:0090104 | pancreatic epsilon cell differentiation | 1 | 0 | 0.04 | 1 |
| GO:0090107 | regulation of high-density lipoprotein p... | 2 | 0 | 0.08 | 1 |
| GO:0090108 | positive regulation of high-density lipo... | 1 | 0 | 0.04 | 1 |
| GO:0090110 | cargo loading into COPII-coated vesicle | 5 | 0 | 0.19 | 1 |
| GO:0090116 | C-5 methylation of cytosine | 4 | 0 | 0.15 | 1 |
| GO:0090131 | mesenchyme migration | 1 | 0 | 0.04 | 1 |
| GO:0090133 | mesendoderm migration | 1 | 0 | 0.04 | 1 |

| | | | | | |
|---|---|---|---|---|---|
| GO:0090134 | cell migration involved in mesendoderm m | 1 | 0 | 0.04 | 1 |
| GO:0090135 | actin filament branching | 3 | 0 | 0.11 | 1 |
| GO:0090136 | epithelial cell-cell adhesion | 13 | 0 | 0.49 | 1 |
| GO:0090138 | regulation of actin cytoskeleton organiz... | 1 | 0 | 0.04 | 1 |
| GO:0090140 | regulation of mitochondrial fission | 10 | 0 | 0.38 | 1 |
| GO:0090141 | positive regulation of mitochondrial fis... | 7 | 0 | 0.27 | 1 |
| GO:0090148 | membrane fission | 1 | 0 | 0.04 | 1 |
| GO:0090149 | mitochondrial membrane fission | 1 | 0 | 0.04 | 1 |
| GO:0090153 | regulation of sphingolipid biosynthetic ... | 7 | 0 | 0.27 | 1 |
| GO:0090154 | positive regulation of sphingolipid bios... | 3 | 0 | 0.11 | 1 |
| GO:0090155 | negative regulation of sphingolipid bios... | 3 | 0 | 0.11 | 1 |
| GO:0090156 | cellular sphingolipid homeostasis | 1 | 0 | 0.04 | 1 |
| GO:0090158 | endoplasmic reticulum membrane organiza | 2 | 0 | 0.08 | 1 |
| GO:0090160 | Golgi to lysosome transport | 3 | 0 | 0.11 | 1 |
| GO:0090161 | Golgi ribbon formation | 8 | 0 | 0.3 | 1 |
| GO:0090162 | establishment of epithelial cell polarit... | 13 | 0 | 0.49 | 1 |
| GO:0090164 | asymmetric Golgi ribbon formation | 2 | 0 | 0.08 | 1 |
| GO:0090166 | Golgi disassembly | 4 | 0 | 0.15 | 1 |
| GO:0090168 | Golgi reassembly | 4 | 0 | 0.15 | 1 |
| GO:0090169 | regulation of spindle assembly | 12 | 0 | 0.46 | 1 |
| GO:0090170 | regulation of Golgi inheritance | 3 | 0 | 0.11 | 1 |
| GO:0090171 | chondrocyte morphogenesis | 1 | 0 | 0.04 | 1 |
| GO:0090182 | regulation of secretion of lysosomal enz... | 2 | 0 | 0.08 | 1 |
| GO:0090183 | regulation of kidney development | 53 | 0 | 2.02 | 1 |
| GO:0090184 | positive regulation of kidney developmen... | 38 | 0 | 1.45 | 1 |
| GO:0090185 | negative regulation of kidney developmen. | 17 | 0 | 0.65 | 1 |
| GO:0090186 | regulation of pancreatic juice secretion | 3 | 0 | 0.11 | 1 |
| GO:0090187 | positive regulation of pancreatic juice ... | 1 | 0 | 0.04 | 1 |
| GO:0090188 | negative regulation of pancreatic juice ... | 2 | 0 | 0.08 | 1 |
| GO:0090189 | regulation of branching involved in uret... | 23 | 0 | 0.88 | 1 |
| GO:0090190 | positive regulation of branching involve... | 19 | 0 | 0.72 | 1 |
| GO:0090191 | negative regulation of branching involve... | 4 | 0 | 0.15 | 1 |
| GO:0090192 | regulation of glomerulus development | 11 | 0 | 0.42 | 1 |
| GO:0090193 | positive regulation of glomerulus develo... | 8 | 0 | 0.3 | 1 |
| GO:0090194 | negative regulation of glomerulus develo... | 3 | 0 | 0.11 | 1 |
| GO:0090195 | chemokine secretion | 10 | 0 | 0.38 | 1 |
| GO:0090196 | regulation of chemokine secretion | 9 | 0 | 0.34 | 1 |
| GO:0090197 | positive regulation of chemokine secreti... | 8 | 0 | 0.3 | 1 |
| GO:0090198 | negative regulation of chemokine secreti... | 1 | 0 | 0.04 | 1 |
| GO:0090202 | gene looping | 3 | 0 | 0.11 | 1 |
| GO:0090204 | protein localization to nuclear pore | 2 | 0 | 0.08 | 1 |
| GO:0090205 | positive regulation of cholesterol metab... | 6 | 0 | 0.23 | 1 |
| GO:0090207 | regulation of triglyceride metabolic pro... | 25 | 0 | 0.95 | 1 |
| GO:0090208 | positive regulation of triglyceride meta... | 16 | 0 | 0.61 | 1 |
| GO:0090209 | negative regulation of triglyceride meta... | 6 | 0 | 0.23 | 1 |
| GO:0090210 | regulation of establishment of blood-bra... | 1 | 0 | 0.04 | 1 |
| GO:0090212 | negative regulation of establishment of ... | 1 | 0 | 0.04 | 1 |
| GO:0090214 | spongiotrophoblast layer developmental g. | 2 | 0 | 0.08 | 1 |
| GO:0090220 | chromosome localization to nuclear envel. | 1 | 0 | 0.04 | 1 |
| GO:0090222 | centrosome-templated microtubule nucleat | 1 | 0 | 0.04 | 1 |
| GO:0090224 | regulation of spindle organization | 20 | 0 | 0.76 | 1 |
| GO:0090230 | regulation of centromere complex assembl | 3 | 0 | 0.11 | 1 |
| GO:0090233 | negative regulation of spindle checkpoin... | 1 | 0 | 0.04 | 1 |

| | | | | | |
|---|---|---|---|---|---|
| GO:0090234 | regulation of kinetochore assembly | 2 | 0 | 0.08 | 1 |
| GO:0090235 | regulation of metaphase plate congressio.. | 1 | 0 | 0.04 | 1 |
| GO:0090237 | regulation of arachidonic acid secretion | 5 | 0 | 0.19 | 1 |
| GO:0090238 | positive regulation of arachidonic acid ... | 4 | 0 | 0.15 | 1 |
| GO:0090239 | regulation of histone H4 acetylation | 9 | 0 | 0.34 | 1 |
| GO:0090240 | positive regulation of histone H4 acetyl... | 3 | 0 | 0.11 | 1 |
| GO:0090241 | negative regulation of histone H4 acetyl... | 5 | 0 | 0.19 | 1 |
| GO:0090246 | convergent extension involved in somitog.. | 1 | 0 | 0.04 | 1 |
| GO:0090247 | cell motility involved in somitogenic ax... | 2 | 0 | 0.08 | 1 |
| GO:0090249 | regulation of cell motility involved in ... | 2 | 0 | 0.08 | 1 |
| GO:0090251 | protein localization involved in establi... | 1 | 0 | 0.04 | 1 |
| GO:0090258 | negative regulation of mitochondrial fis... | 1 | 0 | 0.04 | 1 |
| GO:0090259 | regulation of retinal ganglion cell axon... | 4 | 0 | 0.15 | 1 |
| GO:0090260 | negative regulation of retinal ganglion ... | 2 | 0 | 0.08 | 1 |
| GO:0090261 | positive regulation of inclusion body as... | 4 | 0 | 0.15 | 1 |
| GO:0090269 | fibroblast growth factor production | 2 | 0 | 0.08 | 1 |
| GO:0090270 | regulation of fibroblast growth factor p... | 2 | 0 | 0.08 | 1 |
| GO:0090271 | positive regulation of fibroblast growth... | 1 | 0 | 0.04 | 1 |
| GO:0090272 | negative regulation of fibroblast growth... | 1 | 0 | 0.04 | 1 |
| GO:0090273 | regulation of somatostatin secretion | 1 | 0 | 0.04 | 1 |
| GO:0090274 | positive regulation of somatostatin secr... | 1 | 0 | 0.04 | 1 |
| GO:0090277 | positive regulation of peptide hormone s... | 69 | 0 | 2.63 | 1 |
| GO:0090281 | negative regulation of calcium ion impor... | 6 | 0 | 0.23 | 1 |
| GO:0090283 | regulation of protein glycosylation in G... | 3 | 0 | 0.11 | 1 |
| GO:0090284 | positive regulation of protein glycosyla... | 1 | 0 | 0.04 | 1 |
| GO:0090285 | negative regulation of protein glycosyla... | 2 | 0 | 0.08 | 1 |
| GO:0090286 | cytoskeletal anchoring at nuclear membra. | 3 | 0 | 0.11 | 1 |
| GO:0090289 | regulation of osteoclast proliferation | 2 | 0 | 0.08 | 1 |
| GO:0090291 | negative regulation of osteoclast prolif... | 2 | 0 | 0.08 | 1 |
| GO:0090292 | nuclear matrix anchoring at nuclear memb | 1 | 0 | 0.04 | 1 |
| GO:0090293 | nitrogen catabolite regulation of transc... | 1 | 0 | 0.04 | 1 |
| GO:0090294 | nitrogen catabolite activation of transc... | 1 | 0 | 0.04 | 1 |
| GO:0090296 | regulation of mitochondrial DNA replicat... | 4 | 0 | 0.15 | 1 |
| GO:0090297 | positive regulation of mitochondrial DNA... | 1 | 0 | 0.04 | 1 |
| GO:0090298 | negative regulation of mitochondrial DNA.. | 2 | 0 | 0.08 | 1 |
| GO:0090299 | regulation of neural crest formation | 1 | 0 | 0.04 | 1 |
| GO:0090301 | negative regulation of neural crest form... | 1 | 0 | 0.04 | 1 |
| GO:0090306 | spindle assembly involved in meiosis | 3 | 0 | 0.11 | 1 |
| GO:0090307 | mitotic spindle assembly | 30 | 0 | 1.14 | 1 |
| GO:0090308 | regulation of methylation-dependent chro.. | 4 | 0 | 0.15 | 1 |
| GO:0090310 | negative regulation of methylation-depen... | 3 | 0 | 0.11 | 1 |
| GO:0090313 | regulation of protein targeting to membr... | 14 | 0 | 0.53 | 1 |
| GO:0090314 | positive regulation of protein targeting... | 10 | 0 | 0.38 | 1 |
| GO:0090315 | negative regulation of protein targeting... | 4 | 0 | 0.15 | 1 |
| GO:0090320 | regulation of chylomicron remnant cleara... | 1 | 0 | 0.04 | 1 |
| GO:0090321 | positive regulation of chylomicron remna... | 1 | 0 | 0.04 | 1 |
| GO:0090322 | regulation of superoxide metabolic proce... | 16 | 0 | 0.61 | 1 |
| GO:0090323 | prostaglandin secretion involved in immu... | 1 | 0 | 0.04 | 1 |
| GO:0090324 | negative regulation of oxidative phospho... | 4 | 0 | 0.15 | 1 |
| GO:0090325 | regulation of locomotion involved in loc... | 3 | 0 | 0.11 | 1 |
| GO:0090327 | negative regulation of locomotion involv... | 1 | 0 | 0.04 | 1 |
| GO:0090335 | regulation of brown fat cell differentia... | 6 | 0 | 0.23 | 1 |
| GO:0090336 | positive regulation of brown fat cell di... | 5 | 0 | 0.19 | 1 |

| | | | | | |
|---|---|---|---|---|---|
| GO:0090340 | positive regulation of secretion of lyso... | 1 | 0 | 0.04 | 1 |
| GO:0090341 | negative regulation of secretion of lyso... | 1 | 0 | 0.04 | 1 |
| GO:0090343 | positive regulation of cell aging | 6 | 0 | 0.23 | 1 |
| GO:0090345 | cellular organohalogen metabolic process | 2 | 0 | 0.08 | 1 |
| GO:0090346 | cellular organofluorine metabolic proces... | 2 | 0 | 0.08 | 1 |
| GO:0090347 | regulation of cellular organohalogen met... | 1 | 0 | 0.04 | 1 |
| GO:0090348 | regulation of cellular organofluorine me... | 1 | 0 | 0.04 | 1 |
| GO:0090349 | negative regulation of cellular organoha... | 1 | 0 | 0.04 | 1 |
| GO:0090350 | negative regulation of cellular organofl... | 1 | 0 | 0.04 | 1 |
| GO:0090360 | platelet-derived growth factor productio... | 3 | 0 | 0.11 | 1 |
| GO:0090361 | regulation of platelet-derived growth fa... | 3 | 0 | 0.11 | 1 |
| GO:0090362 | positive regulation of platelet-derived ... | 2 | 0 | 0.08 | 1 |
| GO:0090365 | regulation of mRNA modification | 1 | 0 | 0.04 | 1 |
| GO:0090367 | negative regulation of mRNA modification | 1 | 0 | 0.04 | 1 |
| GO:0090370 | negative regulation of cholesterol efflu... | 5 | 0 | 0.19 | 1 |
| GO:0090381 | regulation of heart induction | 4 | 0 | 0.15 | 1 |
| GO:0090383 | phagosome acidification | 4 | 0 | 0.15 | 1 |
| GO:0090385 | phagosome-lysosome fusion | 3 | 0 | 0.11 | 1 |
| GO:0090386 | phagosome maturation involved in apoptot | 1 | 0 | 0.04 | 1 |
| GO:0090387 | phagolysosome assembly involved in apop | 1 | 0 | 0.04 | 1 |
| GO:0090399 | replicative senescence | 10 | 0 | 0.38 | 1 |
| GO:0090402 | oncogene-induced cell senescence | 2 | 0 | 0.08 | 1 |
| GO:0090403 | oxidative stress-induced premature senes. | 4 | 0 | 0.15 | 1 |
| GO:0090410 | malonate catabolic process | 1 | 0 | 0.04 | 1 |
| GO:0090420 | naphthalene-containing compound metabo | 1 | 0 | 0.04 | 1 |
| GO:0090425 | acinar cell differentiation | 1 | 0 | 0.04 | 1 |
| GO:0090427 | activation of meiosis | 4 | 0 | 0.15 | 1 |
| GO:0090435 | protein localization to nuclear envelope | 3 | 0 | 0.11 | 1 |
| GO:0090467 | arginine import | 3 | 0 | 0.11 | 1 |
| GO:0090481 | pyrimidine nucleotide-sugar transmembrar | 5 | 0 | 0.19 | 1 |
| GO:0090493 | catecholamine uptake | 11 | 0 | 0.42 | 1 |
| GO:0090494 | dopamine uptake | 11 | 0 | 0.42 | 1 |
| GO:0090520 | sphingolipid mediated signaling pathway | 10 | 0 | 0.38 | 1 |
| GO:0090521 | glomerular visceral epithelial cell migr... | 1 | 0 | 0.04 | 1 |
| GO:0090526 | regulation of gluconeogenesis involved i... | 3 | 0 | 0.11 | 1 |
| GO:0090527 | actin filament reorganization | 3 | 0 | 0.11 | 1 |
| GO:0090529 | cell septum assembly | 2 | 0 | 0.08 | 1 |
| GO:0090558 | plant epidermis development | 1 | 0 | 0.04 | 1 |
| GO:0090579 | dsDNA loop formation | 3 | 0 | 0.11 | 1 |
| GO:0090594 | inflammatory response to wounding | 6 | 0 | 0.23 | 1 |
| GO:0090598 | male anatomical structure morphogenesis | 3 | 0 | 0.11 | 1 |
| GO:0090611 | ubiquitin-independent protein catabolic ... | 5 | 0 | 0.19 | 1 |
| GO:0090627 | plant epidermal cell differentiation | 1 | 0 | 0.04 | 1 |
| GO:0097011 | cellular response to granulocyte macroph.. | 6 | 0 | 0.23 | 1 |
| GO:0097012 | response to granulocyte macrophage colo | 6 | 0 | 0.23 | 1 |
| GO:0097017 | renal protein absorption | 3 | 0 | 0.11 | 1 |
| GO:0097018 | renal albumin absorption | 2 | 0 | 0.08 | 1 |
| GO:0097021 | lymphocyte migration into lymphoid organ. | 3 | 0 | 0.11 | 1 |
| GO:0097022 | lymphocyte migration into lymph node | 1 | 0 | 0.04 | 1 |
| GO:0097026 | dendritic cell dendrite assembly | 3 | 0 | 0.11 | 1 |
| GO:0097028 | dendritic cell differentiation | 38 | 0 | 1.45 | 1 |
| GO:0097029 | mature conventional dendritic cell diffe... | 4 | 0 | 0.15 | 1 |
| GO:0097031 | mitochondrial respiratory chain complex ... | 11 | 0 | 0.42 | 1 |

| | | | | | |
|---|---|---|---|---|---|
| GO:0097032 | mitochondrial respiratory chain complex ... | 1 | 0 | 0.04 | 1 |
| GO:0097033 | mitochondrial respiratory chain complex ... | 3 | 0 | 0.11 | 1 |
| GO:0097034 | mitochondrial respiratory chain complex ... | 2 | 0 | 0.08 | 1 |
| GO:0097037 | heme export | 2 | 0 | 0.08 | 1 |
| GO:0097039 | protein linear polyubiquitination | 5 | 0 | 0.19 | 1 |
| GO:0097045 | phosphatidylserine exposure on blood pla. | 1 | 0 | 0.04 | 1 |
| GO:0097048 | dendritic cell apoptotic process | 6 | 0 | 0.23 | 1 |
| GO:0097049 | motor neuron apoptotic process | 3 | 0 | 0.11 | 1 |
| GO:0097050 | type B pancreatic cell apoptotic process | 10 | 0 | 0.38 | 1 |
| GO:0097051 | establishment of protein localization to... | 3 | 0 | 0.11 | 1 |
| GO:0097052 | L-kynurenine metabolic process | 5 | 0 | 0.19 | 1 |
| GO:0097053 | L-kynurenine catabolic process | 2 | 0 | 0.08 | 1 |
| GO:0097055 | agmatine biosynthetic process | 1 | 0 | 0.04 | 1 |
| GO:0097056 | selenocysteinyl-tRNA(Sec) biosynthetic p.. | 4 | 0 | 0.15 | 1 |
| GO:0097065 | anterior head development | 2 | 0 | 0.08 | 1 |
| GO:0097070 | ductus arteriosus closure | 5 | 0 | 0.19 | 1 |
| GO:0097084 | vascular smooth muscle cell development | 7 | 0 | 0.27 | 1 |
| GO:0097086 | amniotic stem cell differentiation | 1 | 0 | 0.04 | 1 |
| GO:0097089 | methyl-branched fatty acid metabolic pro... | 2 | 0 | 0.08 | 1 |
| GO:0097094 | craniofacial suture morphogenesis | 15 | 0 | 0.57 | 1 |
| GO:0097101 | blood vessel endothelial cell fate speci... | 2 | 0 | 0.08 | 1 |
| GO:0097102 | endothelial tip cell fate specification | 1 | 0 | 0.04 | 1 |
| GO:0097111 | endoplasmic reticulum-Golgi intermediate. | 2 | 0 | 0.08 | 1 |
| GO:0097113 | alpha-amino-3-hydroxy-5-methyl-4-isoxaz‹ | 5 | 0 | 0.19 | 1 |
| GO:0097115 | neurexin clustering | 1 | 0 | 0.04 | 1 |
| GO:0097151 | positive regulation of inhibitory postsy... | 4 | 0 | 0.15 | 1 |
| GO:0097152 | mesenchymal cell apoptotic process | 13 | 0 | 0.49 | 1 |
| GO:0097154 | GABAergic neuron differentiation | 12 | 0 | 0.46 | 1 |
| GO:0097155 | fasciculation of sensory neuron axon | 3 | 0 | 0.11 | 1 |
| GO:0097156 | fasciculation of motor neuron axon | 2 | 0 | 0.08 | 1 |
| GO:0097166 | lens epithelial cell proliferation | 1 | 0 | 0.04 | 1 |
| GO:0097167 | circadian regulation of translation | 4 | 0 | 0.15 | 1 |
| GO:0097168 | mesenchymal stem cell proliferation | 2 | 0 | 0.08 | 1 |
| GO:0097178 | ruffle assembly | 22 | 0 | 0.84 | 1 |
| GO:0097186 | amelogenesis | 16 | 0 | 0.61 | 1 |
| GO:0097195 | pilomotor reflex | 3 | 0 | 0.11 | 1 |
| GO:0097198 | histone H3-K36 trimethylation | 2 | 0 | 0.08 | 1 |
| GO:0097205 | renal filtration | 22 | 0 | 0.84 | 1 |
| GO:0097210 | response to gonadotropin-releasing hormc | 5 | 0 | 0.19 | 1 |
| GO:0097211 | cellular response to gonadotropin-releas... | 5 | 0 | 0.19 | 1 |
| GO:0097212 | lysosomal membrane organization | 1 | 0 | 0.04 | 1 |
| GO:0097213 | regulation of lysosomal membrane permea | 1 | 0 | 0.04 | 1 |
| GO:0097214 | positive regulation of lysosomal membran. | 1 | 0 | 0.04 | 1 |
| GO:0097241 | hematopoietic stem cell migration to bon... | 2 | 0 | 0.08 | 1 |
| GO:0097242 | beta-amyloid clearance | 7 | 0 | 0.27 | 1 |
| GO:0097254 | renal tubular secretion | 1 | 0 | 0.04 | 1 |
| GO:0097264 | self proteolysis | 2 | 0 | 0.08 | 1 |
| GO:0097267 | omega-hydroxylase P450 pathway | 9 | 0 | 0.34 | 1 |
| GO:0097272 | ammonia homeostasis | 2 | 0 | 0.08 | 1 |
| GO:0097273 | creatinine homeostasis | 1 | 0 | 0.04 | 1 |
| GO:0097274 | urea homeostasis | 2 | 0 | 0.08 | 1 |
| GO:0097275 | cellular ammonia homeostasis | 1 | 0 | 0.04 | 1 |
| GO:0097276 | cellular creatinine homeostasis | 1 | 0 | 0.04 | 1 |

| | | | | | |
|---|---|---|---|---|---|
| GO:0097277 | cellular urea homeostasis | 1 | 0 | 0.04 | 1 |
| GO:0097278 | complement-dependent cytotoxicity | 3 | 0 | 0.11 | 1 |
| GO:0097283 | keratinocyte apoptotic process | 4 | 0 | 0.15 | 1 |
| GO:0097284 | hepatocyte apoptotic process | 13 | 0 | 0.49 | 1 |
| GO:0097291 | renal phosphate ion absorption | 1 | 0 | 0.04 | 1 |
| GO:0097296 | activation of cysteine-type endopeptidas... | 3 | 0 | 0.11 | 1 |
| GO:0097298 | regulation of nucleus size | 1 | 0 | 0.04 | 1 |
| GO:0097300 | programmed necrotic cell death | 31 | 0 | 1.18 | 1 |
| GO:0097324 | melanocyte migration | 2 | 0 | 0.08 | 1 |
| GO:0097325 | melanocyte proliferation | 1 | 0 | 0.04 | 1 |
| GO:0097326 | melanocyte adhesion | 1 | 0 | 0.04 | 1 |
| GO:0097332 | response to antipsychotic drug | 1 | 0 | 0.04 | 1 |
| GO:0097340 | inhibition of cysteine-type endopeptidas... | 10 | 0 | 0.38 | 1 |
| GO:0097341 | zymogen inhibition | 10 | 0 | 0.38 | 1 |
| GO:0097343 | ripoptosome assembly | 5 | 0 | 0.19 | 1 |
| GO:0097350 | neutrophil clearance | 1 | 0 | 0.04 | 1 |
| GO:0097352 | autophagic vacuole maturation | 3 | 0 | 0.11 | 1 |
| GO:0097354 | prenylation | 10 | 0 | 0.38 | 1 |
| GO:0097360 | chorionic trophoblast cell proliferation | 3 | 0 | 0.11 | 1 |
| GO:0097368 | establishment of Sertoli cell barrier | 4 | 0 | 0.15 | 1 |
| GO:0097369 | sodium ion import | 2 | 0 | 0.08 | 1 |
| GO:0097374 | sensory neuron axon guidance | 2 | 0 | 0.08 | 1 |
| GO:0097384 | cellular lipid biosynthetic process | 5 | 0 | 0.19 | 1 |
| GO:0097393 | telomeric RNA transcription | 1 | 0 | 0.04 | 1 |
| GO:0097394 | telomeric RNA transcription from RNA pol.. | 1 | 0 | 0.04 | 1 |
| GO:0097402 | neuroblast migration | 1 | 0 | 0.04 | 1 |
| GO:0097411 | hypoxia-inducible factor-1alpha signalin... | 3 | 0 | 0.11 | 1 |
| GO:0097421 | liver regeneration | 4 | 0 | 0.15 | 1 |
| GO:0097435 | fibril organization | 18 | 0 | 0.69 | 1 |
| GO:0097475 | motor neuron migration | 4 | 0 | 0.15 | 1 |
| GO:0097476 | spinal cord motor neuron migration | 4 | 0 | 0.15 | 1 |
| GO:0097477 | lateral motor column neuron migration | 3 | 0 | 0.11 | 1 |
| GO:0097484 | dendrite extension | 3 | 0 | 0.11 | 1 |
| GO:0097490 | sympathetic neuron projection extension | 4 | 0 | 0.15 | 1 |
| GO:0097491 | sympathetic neuron projection guidance | 4 | 0 | 0.15 | 1 |
| GO:0097494 | regulation of vesicle size | 2 | 0 | 0.08 | 1 |
| GO:0097499 | protein localization to nonmotile primar... | 2 | 0 | 0.08 | 1 |
| GO:0097500 | receptor localization to nonmotile prima... | 1 | 0 | 0.04 | 1 |
| GO:0097501 | stress response to metal ion | 4 | 0 | 0.15 | 1 |
| GO:0097527 | necroptotic signaling pathway | 6 | 0 | 0.23 | 1 |
| GO:0097531 | mast cell migration | 9 | 0 | 0.34 | 1 |
| GO:0097549 | chromatin organization involved in negat... | 2 | 0 | 0.08 | 1 |
| GO:0097553 | calcium ion transmembrane import into cy. | 1 | 0 | 0.04 | 1 |
| GO:0097576 | vacuole fusion | 8 | 0 | 0.3 | 1 |
| GO:0097577 | sequestering of iron ion | 2 | 0 | 0.08 | 1 |
| GO:0097623 | potassium ion export across plasma memb | 3 | 0 | 0.11 | 1 |
| GO:0097638 | L-arginine import across plasma membran | 1 | 0 | 0.04 | 1 |
| GO:0097639 | L-lysine import across plasma membrane | 1 | 0 | 0.04 | 1 |
| GO:0097640 | L-ornithine import across plasma membrar | 1 | 0 | 0.04 | 1 |
| GO:0097676 | histone H3-K36 dimethylation | 1 | 0 | 0.04 | 1 |
| GO:0097680 | double-strand break repair via classical... | 1 | 0 | 0.04 | 1 |
| GO:0097681 | double-strand break repair via alternati... | 2 | 0 | 0.08 | 1 |
| GO:0097688 | glutamate receptor clustering | 5 | 0 | 0.19 | 1 |

| | | | | | |
|---|---|---|---|---|---|
| GO:0098501 | polynucleotide dephosphorylation | 4 | 0 | 0.15 | 1 |
| GO:0098506 | polynucleotide 3' dephosphorylation | 2 | 0 | 0.08 | 1 |
| GO:0098507 | polynucleotide 5' dephosphorylation | 2 | 0 | 0.08 | 1 |
| GO:0098528 | skeletal muscle fiber differentiation | 3 | 0 | 0.11 | 1 |
| GO:0098532 | histone H3-K27 trimethylation | 2 | 0 | 0.08 | 1 |
| GO:0098535 | de novo centriole assembly | 4 | 0 | 0.15 | 1 |
| GO:0098543 | detection of other organism | 19 | 0 | 0.72 | 1 |
| GO:0098581 | detection of external biotic stimulus | 23 | 0 | 0.88 | 1 |
| GO:0098586 | cellular response to virus | 19 | 0 | 0.72 | 1 |
| GO:0098700 | neurotransmitter loading into synaptic v... | 1 | 0 | 0.04 | 1 |
| GO:0098703 | calcium ion import across plasma membra | 1 | 0 | 0.04 | 1 |
| GO:0098719 | sodium ion import across plasma membra | 1 | 0 | 0.04 | 1 |
| GO:0098722 | asymmetric stem cell division | 6 | 0 | 0.23 | 1 |
| GO:0098728 | germline stem cell asymmetric division | 3 | 0 | 0.11 | 1 |
| GO:0098734 | macromolecule depalmitoylation | 4 | 0 | 0.15 | 1 |
| GO:0098735 | positive regulation of the force of hear... | 4 | 0 | 0.15 | 1 |
| GO:0098736 | negative regulation of the force of hear... | 1 | 0 | 0.04 | 1 |
| GO:0098739 | import across plasma membrane | 6 | 0 | 0.23 | 1 |
| GO:0098746 | fast | calcium ion | 1 | 0 | 0.04 |
| GO:0098754 | detoxification | 10 | 0 | 0.38 | 1 |
| GO:0098756 | response to interleukin-21 | 1 | 0 | 0.04 | 1 |
| GO:0098757 | cellular response to interleukin-21 | 1 | 0 | 0.04 | 1 |
| GO:0098758 | response to interleukin-8 | 1 | 0 | 0.04 | 1 |
| GO:0098759 | cellular response to interleukin-8 | 1 | 0 | 0.04 | 1 |
| GO:0098760 | response to interleukin-7 | 2 | 0 | 0.08 | 1 |
| GO:0098761 | cellular response to interleukin-7 | 2 | 0 | 0.08 | 1 |
| GO:0098779 | activation of mitophagy in response to m... | 6 | 0 | 0.23 | 1 |
| GO:0098902 | regulation of membrane depolarization du. | 5 | 0 | 0.19 | 1 |
| GO:0098903 | regulation of membrane repolarization du.. | 3 | 0 | 0.11 | 1 |
| GO:0098904 | regulation of AV node cell action potent... | 2 | 0 | 0.08 | 1 |
| GO:0098905 | regulation of bundle of His cell action ... | 1 | 0 | 0.04 | 1 |
| GO:0098906 | regulation of Purkinje myocyte action po... | 1 | 0 | 0.04 | 1 |
| GO:0098907 | regulation of SA node cell action potent... | 2 | 0 | 0.08 | 1 |
| GO:0098908 | regulation of neuronal action potential | 1 | 0 | 0.04 | 1 |
| GO:0098910 | regulation of atrial cardiac muscle cell... | 3 | 0 | 0.11 | 1 |
| GO:1900003 | regulation of serine-type endopeptidase ... | 4 | 0 | 0.15 | 1 |
| GO:1900004 | negative regulation of serine-type endop... | 4 | 0 | 0.15 | 1 |
| GO:1900005 | positive regulation of serine-type endop... | 1 | 0 | 0.04 | 1 |
| GO:1900006 | positive regulation of dendrite developm... | 41 | 0 | 1.56 | 1 |
| GO:1900010 | regulation of corticotropin-releasing ho... | 1 | 0 | 0.04 | 1 |
| GO:1900011 | negative regulation of corticotropin-rel... | 1 | 0 | 0.04 | 1 |
| GO:1900015 | regulation of cytokine production involv... | 12 | 0 | 0.46 | 1 |
| GO:1900016 | negative regulation of cytokine producti... | 2 | 0 | 0.08 | 1 |
| GO:1900017 | positive regulation of cytokine producti... | 8 | 0 | 0.3 | 1 |
| GO:1900019 | regulation of protein kinase C activity | 2 | 0 | 0.08 | 1 |
| GO:1900020 | positive regulation of protein kinase C ... | 2 | 0 | 0.08 | 1 |
| GO:1900025 | negative regulation of substrate adhesio... | 5 | 0 | 0.19 | 1 |
| GO:1900027 | regulation of ruffle assembly | 14 | 0 | 0.53 | 1 |
| GO:1900028 | negative regulation of ruffle assembly | 5 | 0 | 0.19 | 1 |
| GO:1900029 | positive regulation of ruffle assembly | 6 | 0 | 0.23 | 1 |
| GO:1900035 | negative regulation of cellular response... | 1 | 0 | 0.04 | 1 |
| GO:1900037 | regulation of cellular response to hypox... | 6 | 0 | 0.23 | 1 |
| GO:1900038 | negative regulation of cellular response... | 4 | 0 | 0.15 | 1 |

| | | | | | |
|---|---|---|---|---|---|
| GO:1900039 | positive regulation of cellular response... | 1 | 0 | 0.04 | 1 |
| GO:1900040 | regulation of interleukin-2 secretion | 7 | 0 | 0.27 | 1 |
| GO:1900041 | negative regulation of interleukin-2 sec... | 2 | 0 | 0.08 | 1 |
| GO:1900042 | positive regulation of interleukin-2 sec... | 5 | 0 | 0.19 | 1 |
| GO:1900044 | regulation of protein K63-linked ubiquit... | 6 | 0 | 0.23 | 1 |
| GO:1900045 | negative regulation of protein K63-linke... | 4 | 0 | 0.15 | 1 |
| GO:1900048 | positive regulation of hemostasis | 24 | 0 | 0.91 | 1 |
| GO:1900052 | regulation of retinoic acid biosynthetic... | 1 | 0 | 0.04 | 1 |
| GO:1900053 | negative regulation of retinoic acid bio... | 1 | 0 | 0.04 | 1 |
| GO:1900060 | negative regulation of ceramide biosynth... | 1 | 0 | 0.04 | 1 |
| GO:1900062 | regulation of replicative cell aging | 1 | 0 | 0.04 | 1 |
| GO:1900063 | regulation of peroxisome organization | 2 | 0 | 0.08 | 1 |
| GO:1900073 | regulation of neuromuscular synaptic tra... | 1 | 0 | 0.04 | 1 |
| GO:1900075 | positive regulation of neuromuscular syn... | 1 | 0 | 0.04 | 1 |
| GO:1900081 | regulation of arginine catabolic process | 1 | 0 | 0.04 | 1 |
| GO:1900082 | negative regulation of arginine cataboli... | 1 | 0 | 0.04 | 1 |
| GO:1900084 | regulation of peptidyl-tyrosine autophos... | 4 | 0 | 0.15 | 1 |
| GO:1900085 | negative regulation of peptidyl-tyrosine... | 1 | 0 | 0.04 | 1 |
| GO:1900086 | positive regulation of peptidyl-tyrosine... | 3 | 0 | 0.11 | 1 |
| GO:1900094 | regulation of transcription from RNA pol... | 5 | 0 | 0.19 | 1 |
| GO:1900098 | regulation of plasma cell differentiatio... | 1 | 0 | 0.04 | 1 |
| GO:1900100 | positive regulation of plasma cell diffe... | 1 | 0 | 0.04 | 1 |
| GO:1900102 | negative regulation of endoplasmic retic... | 8 | 0 | 0.3 | 1 |
| GO:1900104 | regulation of hyaluranon cable assembly | 3 | 0 | 0.11 | 1 |
| GO:1900106 | positive regulation of hyaluranon cable ... | 3 | 0 | 0.11 | 1 |
| GO:1900107 | regulation of nodal signaling pathway | 6 | 0 | 0.23 | 1 |
| GO:1900108 | negative regulation of nodal signaling p... | 2 | 0 | 0.08 | 1 |
| GO:1900109 | regulation of histone H3-K9 dimethylatio... | 1 | 0 | 0.04 | 1 |
| GO:1900111 | positive regulation of histone H3-K9 dim... | 1 | 0 | 0.04 | 1 |
| GO:1900112 | regulation of histone H3-K9 trimethylati... | 1 | 0 | 0.04 | 1 |
| GO:1900114 | positive regulation of histone H3-K9 tri... | 1 | 0 | 0.04 | 1 |
| GO:1900118 | negative regulation of execution phase o... | 9 | 0 | 0.34 | 1 |
| GO:1900120 | regulation of receptor binding | 15 | 0 | 0.57 | 1 |
| GO:1900121 | negative regulation of receptor binding | 10 | 0 | 0.38 | 1 |
| GO:1900122 | positive regulation of receptor binding | 4 | 0 | 0.15 | 1 |
| GO:1900125 | regulation of hyaluronan biosynthetic pr... | 6 | 0 | 0.23 | 1 |
| GO:1900126 | negative regulation of hyaluronan biosyn... | 3 | 0 | 0.11 | 1 |
| GO:1900127 | positive regulation of hyaluronan biosyn... | 3 | 0 | 0.11 | 1 |
| GO:1900128 | regulation of G-protein activated inward... | 1 | 0 | 0.04 | 1 |
| GO:1900130 | regulation of lipid binding | 1 | 0 | 0.04 | 1 |
| GO:1900131 | negative regulation of lipid binding | 1 | 0 | 0.04 | 1 |
| GO:1900134 | negative regulation of renin secretion i... | 1 | 0 | 0.04 | 1 |
| GO:1900138 | negative regulation of phospholipase A2 ... | 3 | 0 | 0.11 | 1 |
| GO:1900139 | negative regulation of arachidonic acid ... | 1 | 0 | 0.04 | 1 |
| GO:1900141 | regulation of oligodendrocyte apoptotic ... | 1 | 0 | 0.04 | 1 |
| GO:1900142 | negative regulation of oligodendrocyte a... | 1 | 0 | 0.04 | 1 |
| GO:1900145 | regulation of nodal signaling pathway in... | 3 | 0 | 0.11 | 1 |
| GO:1900151 | regulation of nuclear-transcribed mRNA c.. | 13 | 0 | 0.49 | 1 |
| GO:1900152 | negative regulation of nuclear-transcrib... | 1 | 0 | 0.04 | 1 |
| GO:1900153 | positive regulation of nuclear-transcrib... | 13 | 0 | 0.49 | 1 |
| GO:1900154 | regulation of bone trabecula formation | 2 | 0 | 0.08 | 1 |
| GO:1900155 | negative regulation of bone trabecula fo... | 2 | 0 | 0.08 | 1 |
| GO:1900157 | regulation of bone mineralization involv... | 3 | 0 | 0.11 | 1 |

| | | | | | |
|---|---|---|---|---|---|
| GO:1900158 | negative regulation of bone mineralizati... | 1 | 0 | 0.04 | 1 |
| GO:1900159 | positive regulation of bone mineralizati... | 2 | 0 | 0.08 | 1 |
| GO:1900161 | regulation of phospholipid scramblase ac... | 1 | 0 | 0.04 | 1 |
| GO:1900163 | positive regulation of phospholipid scra... | 1 | 0 | 0.04 | 1 |
| GO:1900164 | nodal signaling pathway involved in dete... | 5 | 0 | 0.19 | 1 |
| GO:1900166 | regulation of glial cell line-derived ne... | 2 | 0 | 0.08 | 1 |
| GO:1900168 | positive regulation of glial cell line-d... | 2 | 0 | 0.08 | 1 |
| GO:1900169 | regulation of glucocorticoid mediated si... | 2 | 0 | 0.08 | 1 |
| GO:1900170 | negative regulation of glucocorticoid me... | 1 | 0 | 0.04 | 1 |
| GO:1900175 | regulation of nodal signaling pathway in... | 3 | 0 | 0.11 | 1 |
| GO:1900186 | negative regulation of clathrin-mediated... | 1 | 0 | 0.04 | 1 |
| GO:1900190 | regulation of single-species biofilm for... | 1 | 0 | 0.04 | 1 |
| GO:1900191 | negative regulation of single-species bi... | 1 | 0 | 0.04 | 1 |
| GO:1900193 | regulation of oocyte maturation | 5 | 0 | 0.19 | 1 |
| GO:1900194 | negative regulation of oocyte maturation | 3 | 0 | 0.11 | 1 |
| GO:1900195 | positive regulation of oocyte maturation | 2 | 0 | 0.08 | 1 |
| GO:1900200 | mesenchymal cell apoptotic process invol... | 3 | 0 | 0.11 | 1 |
| GO:1900204 | apoptotic process involved in metanephri... | 2 | 0 | 0.08 | 1 |
| GO:1900205 | apoptotic process involved in metanephri... | 2 | 0 | 0.08 | 1 |
| GO:1900208 | regulation of cardiolipin metabolic proc... | 1 | 0 | 0.04 | 1 |
| GO:1900210 | positive regulation of cardiolipin metab... | 1 | 0 | 0.04 | 1 |
| GO:1900211 | regulation of mesenchymal cell apoptotic... | 3 | 0 | 0.11 | 1 |
| GO:1900212 | negative regulation of mesenchymal cell ... | 3 | 0 | 0.11 | 1 |
| GO:1900214 | regulation of apoptotic process involved... | 2 | 0 | 0.08 | 1 |
| GO:1900215 | negative regulation of apoptotic process... | 2 | 0 | 0.08 | 1 |
| GO:1900217 | regulation of apoptotic process involved... | 2 | 0 | 0.08 | 1 |
| GO:1900218 | negative regulation of apoptotic process... | 2 | 0 | 0.08 | 1 |
| GO:1900220 | semaphorin-plexin signaling pathway invo... | 2 | 0 | 0.08 | 1 |
| GO:1900221 | regulation of beta-amyloid clearance | 6 | 0 | 0.23 | 1 |
| GO:1900222 | negative regulation of beta-amyloid clea... | 3 | 0 | 0.11 | 1 |
| GO:1900223 | positive regulation of beta-amyloid clea... | 1 | 0 | 0.04 | 1 |
| GO:1900224 | positive regulation of nodal signaling p... | 2 | 0 | 0.08 | 1 |
| GO:1900225 | regulation of NLRP3 inflammasome compl | 2 | 0 | 0.08 | 1 |
| GO:1900226 | negative regulation of NLRP3 inflammasor | 1 | 0 | 0.04 | 1 |
| GO:1900228 | regulation of single-species biofilm for... | 1 | 0 | 0.04 | 1 |
| GO:1900229 | negative regulation of single-species bi... | 1 | 0 | 0.04 | 1 |
| GO:1900238 | regulation of metanephric mesenchymal ce | 3 | 0 | 0.11 | 1 |
| GO:1900242 | regulation of synaptic vesicle endocytos... | 4 | 0 | 0.15 | 1 |
| GO:1900244 | positive regulation of synaptic vesicle ... | 3 | 0 | 0.11 | 1 |
| GO:1900245 | positive regulation of MDA-5 signaling p... | 3 | 0 | 0.11 | 1 |
| GO:1900246 | positive regulation of RIG-I signaling p... | 5 | 0 | 0.19 | 1 |
| GO:1900247 | regulation of cytoplasmic translational ... | 2 | 0 | 0.08 | 1 |
| GO:1900248 | negative regulation of cytoplasmic trans... | 2 | 0 | 0.08 | 1 |
| GO:1900259 | regulation of RNA-directed RNA polymeras | 1 | 0 | 0.04 | 1 |
| GO:1900260 | negative regulation of RNA-directed RNA ... | 1 | 0 | 0.04 | 1 |
| GO:1900271 | regulation of long-term synaptic potenti... | 5 | 0 | 0.19 | 1 |
| GO:1900273 | positive regulation of long-term synapti... | 2 | 0 | 0.08 | 1 |
| GO:1900275 | negative regulation of phospholipase C a... | 2 | 0 | 0.08 | 1 |
| GO:1900276 | regulation of proteinase activated recep... | 1 | 0 | 0.04 | 1 |
| GO:1900279 | regulation of CD4-positive | alpha-beta | 1 | 0 | 0.04 |
| GO:1900281 | positive regulation of CD4-positive | alp... | 1 | 0 | 0.04 |
| GO:1900363 | regulation of mRNA polyadenylation | 8 | 0 | 0.3 | 1 |
| GO:1900364 | negative regulation of mRNA polyadenylat... | 7 | 0 | 0.27 | 1 |

| | | | | | |
|---|---|---|---|---|---|
| GO:1900365 | positive regulation of mRNA polyadenylat... | 1 | 0 | 0.04 | 1 |
| GO:1900368 | regulation of RNA interference | 3 | 0 | 0.11 | 1 |
| GO:1900369 | negative regulation of RNA interference | 2 | 0 | 0.08 | 1 |
| GO:1900370 | positive regulation of RNA interference | 1 | 0 | 0.04 | 1 |
| GO:1900376 | regulation of secondary metabolite biosy... | 5 | 0 | 0.19 | 1 |
| GO:1900377 | negative regulation of secondary metabol... | 2 | 0 | 0.08 | 1 |
| GO:1900378 | positive regulation of secondary metabol... | 3 | 0 | 0.11 | 1 |
| GO:1900387 | negative regulation of cell-cell adhesio... | 1 | 0 | 0.04 | 1 |
| GO:1900402 | regulation of carbohydrate metabolic pro... | 1 | 0 | 0.04 | 1 |
| GO:1900413 | positive regulation of phospholipid bios... | 1 | 0 | 0.04 | 1 |
| GO:1900424 | regulation of defense response to bacter... | 5 | 0 | 0.19 | 1 |
| GO:1900425 | negative regulation of defense response ... | 1 | 0 | 0.04 | 1 |
| GO:1900426 | positive regulation of defense response ... | 3 | 0 | 0.11 | 1 |
| GO:1900451 | positive regulation of glutamate recepto... | 3 | 0 | 0.11 | 1 |
| GO:1900452 | regulation of long term synaptic depress... | 3 | 0 | 0.11 | 1 |
| GO:1900453 | negative regulation of long term synapti... | 1 | 0 | 0.04 | 1 |
| GO:1900477 | negative regulation of G1/S transition o... | 1 | 0 | 0.04 | 1 |
| GO:1900483 | regulation of protein targeting to vacuo... | 1 | 0 | 0.04 | 1 |
| GO:1900533 | palmitic acid metabolic process | 1 | 0 | 0.04 | 1 |
| GO:1900535 | palmitic acid biosynthetic process | 1 | 0 | 0.04 | 1 |
| GO:1900619 | acetate ester metabolic process | 4 | 0 | 0.15 | 1 |
| GO:1900620 | acetate ester biosynthetic process | 2 | 0 | 0.08 | 1 |
| GO:1900623 | regulation of monocyte aggregation | 2 | 0 | 0.08 | 1 |
| GO:1900625 | positive regulation of monocyte aggregat... | 2 | 0 | 0.08 | 1 |
| GO:1900673 | olefin metabolic process | 2 | 0 | 0.08 | 1 |
| GO:1900736 | regulation of phospholipase C-activating... | 2 | 0 | 0.08 | 1 |
| GO:1900737 | negative regulation of phospholipase C-a... | 1 | 0 | 0.04 | 1 |
| GO:1900738 | positive regulation of phospholipase C-a... | 1 | 0 | 0.04 | 1 |
| GO:1900739 | regulation of protein insertion into mit... | 26 | 0 | 0.99 | 1 |
| GO:1900740 | positive regulation of protein insertion... | 26 | 0 | 0.99 | 1 |
| GO:1900744 | regulation of p38MAPK cascade | 13 | 0 | 0.49 | 1 |
| GO:1900745 | positive regulation of p38MAPK cascade | 9 | 0 | 0.34 | 1 |
| GO:1900746 | regulation of vascular endothelial growt... | 7 | 0 | 0.27 | 1 |
| GO:1900747 | negative regulation of vascular endothel... | 3 | 0 | 0.11 | 1 |
| GO:1900748 | positive regulation of vascular endothel... | 3 | 0 | 0.11 | 1 |
| GO:1900756 | protein processing in phagocytic vesicle | 2 | 0 | 0.08 | 1 |
| GO:1900757 | regulation of D-amino-acid oxidase activ... | 1 | 0 | 0.04 | 1 |
| GO:1900758 | negative regulation of D-amino-acid oxid... | 1 | 0 | 0.04 | 1 |
| GO:1900825 | regulation of membrane depolarization du... | 4 | 0 | 0.15 | 1 |
| GO:1900827 | positive regulation of membrane depolari... | 1 | 0 | 0.04 | 1 |
| GO:1900864 | mitochondrial RNA modification | 1 | 0 | 0.04 | 1 |
| GO:1900920 | regulation of L-glutamate import | 1 | 0 | 0.04 | 1 |
| GO:1901003 | negative regulation of fermentation | 1 | 0 | 0.04 | 1 |
| GO:1901016 | regulation of potassium ion transmembran | 31 | 0 | 1.18 | 1 |
| GO:1901017 | negative regulation of potassium ion tra... | 10 | 0 | 0.38 | 1 |
| GO:1901018 | positive regulation of potassium ion tra... | 13 | 0 | 0.49 | 1 |
| GO:1901020 | negative regulation of calcium ion trans... | 21 | 0 | 0.8 | 1 |
| GO:1901021 | positive regulation of calcium ion trans... | 21 | 0 | 0.8 | 1 |
| GO:1901026 | ripoptosome assembly involved in necropt... | 5 | 0 | 0.19 | 1 |
| GO:1901030 | positive regulation of mitochondrial out... | 33 | 0 | 1.26 | 1 |
| GO:1901033 | positive regulation of response to react... | 3 | 0 | 0.11 | 1 |
| GO:1901068 | guanosine-containing compound metabolic | 19 | 0 | 0.72 | 1 |
| GO:1901069 | guanosine-containing compound catabolic | 2 | 0 | 0.08 | 1 |

| | | | | | |
|---|---|---|---|---|---|
| GO:1901070 | guanosine-containing compound biosynthe | 13 | 0 | 0.49 | 1 |
| GO:1901071 | glucosamine-containing compound metabo | 23 | 0 | 0.88 | 1 |
| GO:1901072 | glucosamine-containing compound catabo | 6 | 0 | 0.23 | 1 |
| GO:1901073 | glucosamine-containing compound biosynt | 3 | 0 | 0.11 | 1 |
| GO:1901074 | regulation of engulfment of apoptotic ce... | 2 | 0 | 0.08 | 1 |
| GO:1901076 | positive regulation of engulfment of apo... | 1 | 0 | 0.04 | 1 |
| GO:1901079 | positive regulation of relaxation of mus... | 1 | 0 | 0.04 | 1 |
| GO:1901090 | regulation of protein tetramerization | 2 | 0 | 0.08 | 1 |
| GO:1901091 | negative regulation of protein tetrameri... | 2 | 0 | 0.08 | 1 |
| GO:1901093 | regulation of protein homotetramerizatio... | 2 | 0 | 0.08 | 1 |
| GO:1901094 | negative regulation of protein homotetra... | 2 | 0 | 0.08 | 1 |
| GO:1901096 | regulation of autophagic vacuole maturat... | 1 | 0 | 0.04 | 1 |
| GO:1901142 | insulin metabolic process | 7 | 0 | 0.27 | 1 |
| GO:1901143 | insulin catabolic process | 1 | 0 | 0.04 | 1 |
| GO:1901145 | mesenchymal cell apoptotic process invol. | 4 | 0 | 0.15 | 1 |
| GO:1901160 | primary amino compound metabolic proce: | 12 | 0 | 0.46 | 1 |
| GO:1901162 | primary amino compound biosynthetic pro | 4 | 0 | 0.15 | 1 |
| GO:1901163 | regulation of trophoblast cell migration | 7 | 0 | 0.27 | 1 |
| GO:1901164 | negative regulation of trophoblast cell ... | 4 | 0 | 0.15 | 1 |
| GO:1901165 | positive regulation of trophoblast cell ... | 3 | 0 | 0.11 | 1 |
| GO:1901166 | neural crest cell migration involved in ... | 4 | 0 | 0.15 | 1 |
| GO:1901187 | regulation of ephrin receptor signaling ... | 1 | 0 | 0.04 | 1 |
| GO:1901189 | positive regulation of ephrin receptor s... | 1 | 0 | 0.04 | 1 |
| GO:1901193 | regulation of formation of translation p... | 1 | 0 | 0.04 | 1 |
| GO:1901194 | negative regulation of formation of tran... | 1 | 0 | 0.04 | 1 |
| GO:1901201 | regulation of extracellular matrix assem... | 5 | 0 | 0.19 | 1 |
| GO:1901203 | positive regulation of extracellular mat... | 3 | 0 | 0.11 | 1 |
| GO:1901204 | regulation of adrenergic receptor signal... | 2 | 0 | 0.08 | 1 |
| GO:1901205 | negative regulation of adrenergic recept... | 2 | 0 | 0.08 | 1 |
| GO:1901206 | positive regulation of adrenergic recept... | 1 | 0 | 0.04 | 1 |
| GO:1901207 | regulation of heart looping | 2 | 0 | 0.08 | 1 |
| GO:1901208 | negative regulation of heart looping | 1 | 0 | 0.04 | 1 |
| GO:1901210 | regulation of cardiac chamber formation | 1 | 0 | 0.04 | 1 |
| GO:1901211 | negative regulation of cardiac chamber f... | 1 | 0 | 0.04 | 1 |
| GO:1901213 | regulation of transcription from RNA pol... | 13 | 0 | 0.49 | 1 |
| GO:1901227 | negative regulation of transcription fro... | 1 | 0 | 0.04 | 1 |
| GO:1901228 | positive regulation of transcription fro... | 5 | 0 | 0.19 | 1 |
| GO:1901229 | regulation of non-canonical Wnt signalin... | 1 | 0 | 0.04 | 1 |
| GO:1901231 | positive regulation of non-canonical Wnt... | 1 | 0 | 0.04 | 1 |
| GO:1901232 | regulation of convergent extension invol... | 1 | 0 | 0.04 | 1 |
| GO:1901233 | negative regulation of convergent extens... | 1 | 0 | 0.04 | 1 |
| GO:1901246 | regulation of lung ciliated cell differe... | 2 | 0 | 0.08 | 1 |
| GO:1901247 | negative regulation of lung ciliated cel... | 1 | 0 | 0.04 | 1 |
| GO:1901248 | positive regulation of lung ciliated cel... | 1 | 0 | 0.04 | 1 |
| GO:1901249 | regulation of lung goblet cell different... | 2 | 0 | 0.08 | 1 |
| GO:1901250 | negative regulation of lung goblet cell ... | 1 | 0 | 0.04 | 1 |
| GO:1901251 | positive regulation of lung goblet cell ... | 1 | 0 | 0.04 | 1 |
| GO:1901252 | regulation of intracellular transport of... | 2 | 0 | 0.08 | 1 |
| GO:1901253 | negative regulation of intracellular tra... | 2 | 0 | 0.08 | 1 |
| GO:1901255 | nucleotide-excision repair involved in i... | 1 | 0 | 0.04 | 1 |
| GO:1901256 | regulation of macrophage colony-stimulat.. | 3 | 0 | 0.11 | 1 |
| GO:1901258 | positive regulation of macrophage colony.. | 2 | 0 | 0.08 | 1 |
| GO:1901264 | carbohydrate derivative transport | 42 | 0 | 1.6 | 1 |

| | | | | | |
|---|---|---|---|---|---|
| GO:1901295 | regulation of canonical Wnt signaling pa... | 2 | 0 | 0.08 | 1 |
| GO:1901296 | negative regulation of canonical Wnt sig... | 1 | 0 | 0.04 | 1 |
| GO:1901297 | positive regulation of canonical Wnt sig... | 1 | 0 | 0.04 | 1 |
| GO:1901300 | positive regulation of hydrogen peroxide... | 1 | 0 | 0.04 | 1 |
| GO:1901301 | regulation of cargo loading into COPII-c... | 3 | 0 | 0.11 | 1 |
| GO:1901303 | negative regulation of cargo loading int... | 2 | 0 | 0.08 | 1 |
| GO:1901304 | regulation of spermidine biosynthetic pr... | 1 | 0 | 0.04 | 1 |
| GO:1901307 | positive regulation of spermidine biosyn... | 1 | 0 | 0.04 | 1 |
| GO:1901314 | regulation of histone H2A K63-linked ubi... | 3 | 0 | 0.11 | 1 |
| GO:1901315 | negative regulation of histone H2A K63-l... | 3 | 0 | 0.11 | 1 |
| GO:1901318 | negative regulation of sperm motility | 1 | 0 | 0.04 | 1 |
| GO:1901320 | negative regulation of heart induction | 1 | 0 | 0.04 | 1 |
| GO:1901321 | positive regulation of heart induction | 2 | 0 | 0.08 | 1 |
| GO:1901337 | thioester transport | 1 | 0 | 0.04 | 1 |
| GO:1901339 | regulation of store-operated calcium cha... | 5 | 0 | 0.19 | 1 |
| GO:1901341 | positive regulation of store-operated ca... | 4 | 0 | 0.15 | 1 |
| GO:1901373 | lipid hydroperoxide transport | 1 | 0 | 0.04 | 1 |
| GO:1901374 | acetate ester transport | 7 | 0 | 0.27 | 1 |
| GO:1901376 | organic heteropentacyclic compound meta | 1 | 0 | 0.04 | 1 |
| GO:1901380 | negative regulation of potassium ion tra... | 13 | 0 | 0.49 | 1 |
| GO:1901382 | regulation of chorionic trophoblast cell... | 3 | 0 | 0.11 | 1 |
| GO:1901383 | negative regulation of chorionic trophob... | 2 | 0 | 0.08 | 1 |
| GO:1901385 | regulation of voltage-gated calcium chan... | 20 | 0 | 0.76 | 1 |
| GO:1901386 | negative regulation of voltage-gated cal... | 5 | 0 | 0.19 | 1 |
| GO:1901387 | positive regulation of voltage-gated cal... | 2 | 0 | 0.08 | 1 |
| GO:1901407 | regulation of phosphorylation of RNA pol... | 3 | 0 | 0.11 | 1 |
| GO:1901409 | positive regulation of phosphorylation o... | 1 | 0 | 0.04 | 1 |
| GO:1901419 | regulation of response to alcohol | 8 | 0 | 0.3 | 1 |
| GO:1901420 | negative regulation of response to alcoh... | 3 | 0 | 0.11 | 1 |
| GO:1901421 | positive regulation of response to alcoh... | 3 | 0 | 0.11 | 1 |
| GO:1901475 | pyruvate transmembrane transport | 1 | 0 | 0.04 | 1 |
| GO:1901492 | positive regulation of lymphangiogenesis | 2 | 0 | 0.08 | 1 |
| GO:1901503 | ether biosynthetic process | 5 | 0 | 0.19 | 1 |
| GO:1901509 | regulation of endothelial tube morphogen.. | 3 | 0 | 0.11 | 1 |
| GO:1901523 | icosanoid catabolic process | 4 | 0 | 0.15 | 1 |
| GO:1901524 | regulation of macromitophagy | 1 | 0 | 0.04 | 1 |
| GO:1901525 | negative regulation of macromitophagy | 1 | 0 | 0.04 | 1 |
| GO:1901529 | positive regulation of anion channel act... | 1 | 0 | 0.04 | 1 |
| GO:1901533 | negative regulation of hematopoietic pro... | 22 | 0 | 0.84 | 1 |
| GO:1901535 | regulation of DNA demethylation | 2 | 0 | 0.08 | 1 |
| GO:1901536 | negative regulation of DNA demethylation | 2 | 0 | 0.08 | 1 |
| GO:1901538 | changes to DNA methylation involved in e. | 4 | 0 | 0.15 | 1 |
| GO:1901550 | regulation of endothelial cell developme... | 2 | 0 | 0.08 | 1 |
| GO:1901551 | negative regulation of endothelial cell ... | 1 | 0 | 0.04 | 1 |
| GO:1901552 | positive regulation of endothelial cell ... | 1 | 0 | 0.04 | 1 |
| GO:1901558 | response to metformin | 1 | 0 | 0.04 | 1 |
| GO:1901563 | response to camptothecin | 3 | 0 | 0.11 | 1 |
| GO:1901569 | fatty acid derivative catabolic process | 4 | 0 | 0.15 | 1 |
| GO:1901580 | regulation of telomeric RNA transcriptio... | 1 | 0 | 0.04 | 1 |
| GO:1901581 | negative regulation of telomeric RNA tra... | 1 | 0 | 0.04 | 1 |
| GO:1901585 | regulation of acid-sensing ion channel a... | 1 | 0 | 0.04 | 1 |
| GO:1901626 | regulation of postsynaptic membrane orga | 3 | 0 | 0.11 | 1 |
| GO:1901627 | negative regulation of postsynaptic memb. | 1 | 0 | 0.04 | 1 |

| | | | | | |
|---|---|---|---|---|---|
| GO:1901628 | positive regulation of postsynaptic memb... | 1 | 0 | 0.04 | 1 |
| GO:1901631 | positive regulation of presynaptic membr... | 2 | 0 | 0.08 | 1 |
| GO:1901632 | regulation of synaptic vesicle membrane .. | 1 | 0 | 0.04 | 1 |
| GO:1901639 | XDP catabolic process | 1 | 0 | 0.04 | 1 |
| GO:1901642 | nucleoside transmembrane transport | 11 | 0 | 0.42 | 1 |
| GO:1901658 | glycosyl compound catabolic process | 40 | 0 | 1.52 | 1 |
| GO:1901660 | calcium ion export | 2 | 0 | 0.08 | 1 |
| GO:1901661 | quinone metabolic process | 29 | 0 | 1.1 | 1 |
| GO:1901662 | quinone catabolic process | 3 | 0 | 0.11 | 1 |
| GO:1901663 | quinone biosynthetic process | 13 | 0 | 0.49 | 1 |
| GO:1901664 | regulation of NAD+ ADP-ribosyltransferas. | 1 | 0 | 0.04 | 1 |
| GO:1901666 | positive regulation of NAD+ ADP-ribosylt... | 1 | 0 | 0.04 | 1 |
| GO:1901668 | regulation of superoxide dismutase activ... | 1 | 0 | 0.04 | 1 |
| GO:1901671 | positive regulation of superoxide dismut... | 1 | 0 | 0.04 | 1 |
| GO:1901673 | regulation of mitotic spindle assembly | 10 | 0 | 0.38 | 1 |
| GO:1901674 | regulation of histone H3-K27 acetylation | 2 | 0 | 0.08 | 1 |
| GO:1901675 | negative regulation of histone H3-K27 ac... | 1 | 0 | 0.04 | 1 |
| GO:1901676 | positive regulation of histone H3-K27 ac... | 1 | 0 | 0.04 | 1 |
| GO:1901678 | iron coordination entity transport | 11 | 0 | 0.42 | 1 |
| GO:1901679 | nucleotide transmembrane transport | 12 | 0 | 0.46 | 1 |
| GO:1901722 | regulation of cell proliferation involve... | 9 | 0 | 0.34 | 1 |
| GO:1901723 | negative regulation of cell proliferatio... | 5 | 0 | 0.19 | 1 |
| GO:1901724 | positive regulation of cell proliferatio... | 4 | 0 | 0.15 | 1 |
| GO:1901726 | negative regulation of histone deacetyla... | 1 | 0 | 0.04 | 1 |
| GO:1901738 | regulation of vitamin A metabolic proces... | 1 | 0 | 0.04 | 1 |
| GO:1901739 | regulation of myoblast fusion | 16 | 0 | 0.61 | 1 |
| GO:1901740 | negative regulation of myoblast fusion | 2 | 0 | 0.08 | 1 |
| GO:1901741 | positive regulation of myoblast fusion | 14 | 0 | 0.53 | 1 |
| GO:1901751 | leukotriene A4 metabolic process | 1 | 0 | 0.04 | 1 |
| GO:1901836 | regulation of transcription of nuclear l... | 5 | 0 | 0.19 | 1 |
| GO:1901837 | negative regulation of transcription of ... | 3 | 0 | 0.11 | 1 |
| GO:1901838 | positive regulation of transcription of ... | 2 | 0 | 0.08 | 1 |
| GO:1901841 | regulation of high voltage-gated calcium... | 7 | 0 | 0.27 | 1 |
| GO:1901846 | positive regulation of cell communicatio... | 1 | 0 | 0.04 | 1 |
| GO:1901856 | negative regulation of cellular respirat... | 3 | 0 | 0.11 | 1 |
| GO:1901857 | positive regulation of cellular respirat... | 4 | 0 | 0.15 | 1 |
| GO:1901858 | regulation of mitochondrial DNA metaboli.. | 5 | 0 | 0.19 | 1 |
| GO:1901859 | negative regulation of mitochondrial DNA.. | 2 | 0 | 0.08 | 1 |
| GO:1901860 | positive regulation of mitochondrial DNA... | 2 | 0 | 0.08 | 1 |
| GO:1901873 | regulation of post-translational protein... | 1 | 0 | 0.04 | 1 |
| GO:1901874 | negative regulation of post-translationa... | 1 | 0 | 0.04 | 1 |
| GO:1901876 | regulation of calcium ion binding | 2 | 0 | 0.08 | 1 |
| GO:1901877 | negative regulation of calcium ion bindi... | 2 | 0 | 0.08 | 1 |
| GO:1901881 | positive regulation of protein depolymer... | 15 | 0 | 0.57 | 1 |
| GO:1901890 | positive regulation of cell junction ass... | 22 | 0 | 0.84 | 1 |
| GO:1901894 | regulation of calcium-transporting ATPas... | 4 | 0 | 0.15 | 1 |
| GO:1901895 | negative regulation of calcium-transport... | 2 | 0 | 0.08 | 1 |
| GO:1901896 | positive regulation of calcium-transport... | 1 | 0 | 0.04 | 1 |
| GO:1901899 | positive regulation of relaxation of car... | 1 | 0 | 0.04 | 1 |
| GO:1901962 | S-adenosyl-L-methionine transmembrane | 1 | 0 | 0.04 | 1 |
| GO:1901977 | negative regulation of cell cycle checkp... | 4 | 0 | 0.15 | 1 |
| GO:1901979 | regulation of inward rectifier potassium... | 3 | 0 | 0.11 | 1 |
| GO:1901983 | regulation of protein acetylation | 50 | 0 | 1.9 | 1 |

| | | | | | |
|---|---|---|---|---|---|
| GO:1901984 | negative regulation of protein acetylati... | 18 | 0 | 0.69 | 1 |
| GO:1901985 | positive regulation of protein acetylati... | 26 | 0 | 0.99 | 1 |
| GO:1901993 | regulation of meiotic cell cycle phase t... | 3 | 0 | 0.11 | 1 |
| GO:1901994 | negative regulation of meiotic cell cycl... | 3 | 0 | 0.11 | 1 |
| GO:1902003 | regulation of beta-amyloid formation | 9 | 0 | 0.34 | 1 |
| GO:1902004 | positive regulation of beta-amyloid form... | 4 | 0 | 0.15 | 1 |
| GO:1902017 | regulation of cilium assembly | 14 | 0 | 0.53 | 1 |
| GO:1902018 | negative regulation of cilium assembly | 2 | 0 | 0.08 | 1 |
| GO:1902019 | regulation of cilium-dependent cell moti... | 4 | 0 | 0.15 | 1 |
| GO:1902022 | L-lysine transport | 3 | 0 | 0.11 | 1 |
| GO:1902023 | L-arginine transport | 3 | 0 | 0.11 | 1 |
| GO:1902024 | L-histidine transport | 3 | 0 | 0.11 | 1 |
| GO:1902031 | regulation of NADP metabolic process | 3 | 0 | 0.11 | 1 |
| GO:1902033 | regulation of hematopoietic stem cell pr... | 5 | 0 | 0.19 | 1 |
| GO:1902035 | positive regulation of hematopoietic ste... | 3 | 0 | 0.11 | 1 |
| GO:1902037 | negative regulation of hematopoietic ste... | 1 | 0 | 0.04 | 1 |
| GO:1902044 | regulation of Fas signaling pathway | 2 | 0 | 0.08 | 1 |
| GO:1902045 | negative regulation of Fas signaling pat... | 1 | 0 | 0.04 | 1 |
| GO:1902047 | polyamine transmembrane transport | 4 | 0 | 0.15 | 1 |
| GO:1902065 | response to L-glutamate | 2 | 0 | 0.08 | 1 |
| GO:1902068 | regulation of sphingolipid mediated sign... | 1 | 0 | 0.04 | 1 |
| GO:1902071 | regulation of hypoxia-inducible factor-1... | 1 | 0 | 0.04 | 1 |
| GO:1902073 | positive regulation of hypoxia-inducible... | 1 | 0 | 0.04 | 1 |
| GO:1902076 | regulation of lateral motor column neuro... | 1 | 0 | 0.04 | 1 |
| GO:1902078 | positive regulation of lateral motor col... | 1 | 0 | 0.04 | 1 |
| GO:1902080 | regulation of calcium ion import into sa... | 1 | 0 | 0.04 | 1 |
| GO:1902081 | negative regulation of calcium ion impor... | 1 | 0 | 0.04 | 1 |
| GO:1902083 | negative regulation of peptidyl-cysteine... | 3 | 0 | 0.11 | 1 |
| GO:1902102 | regulation of metaphase/anaphase transit. | 1 | 0 | 0.04 | 1 |
| GO:1902103 | negative regulation of metaphase/anaphas | 1 | 0 | 0.04 | 1 |
| GO:1902109 | negative regulation of mitochondrial mem.. | 1 | 0 | 0.04 | 1 |
| GO:1902116 | negative regulation of organelle assembl... | 12 | 0 | 0.46 | 1 |
| GO:1902159 | regulation of cyclic nucleotide-gated io... | 1 | 0 | 0.04 | 1 |
| GO:1902162 | regulation of DNA damage response | signa... | 1 | 0 | 0.04 |
| GO:1902164 | positive regulation of DNA damage respon | 1 | 0 | 0.04 | 1 |
| GO:1902165 | regulation of intrinsic apoptotic signal... | 14 | 0 | 0.53 | 1 |
| GO:1902166 | negative regulation of intrinsic apoptot... | 12 | 0 | 0.46 | 1 |
| GO:1902167 | positive regulation of intrinsic apoptot... | 2 | 0 | 0.08 | 1 |
| GO:1902170 | cellular response to reactive nitrogen s... | 14 | 0 | 0.53 | 1 |
| GO:1902172 | regulation of keratinocyte apoptotic pro... | 4 | 0 | 0.15 | 1 |
| GO:1902173 | negative regulation of keratinocyte apop... | 1 | 0 | 0.04 | 1 |
| GO:1902174 | positive regulation of keratinocyte apop... | 3 | 0 | 0.11 | 1 |
| GO:1902176 | negative regulation of oxidative stress-... | 19 | 0 | 0.72 | 1 |
| GO:1902178 | fibroblast growth factor receptor apopto... | 1 | 0 | 0.04 | 1 |
| GO:1902203 | negative regulation of hepatocyte growth... | 1 | 0 | 0.04 | 1 |
| GO:1902217 | erythrocyte apoptotic process | 1 | 0 | 0.04 | 1 |
| GO:1902218 | regulation of intrinsic apoptotic signal... | 3 | 0 | 0.11 | 1 |
| GO:1902219 | negative regulation of intrinsic apoptot... | 3 | 0 | 0.11 | 1 |
| GO:1902224 | ketone body metabolic process | 9 | 0 | 0.34 | 1 |
| GO:1902230 | negative regulation of intrinsic apoptot... | 23 | 0 | 0.88 | 1 |
| GO:1902237 | positive regulation of endoplasmic retic... | 8 | 0 | 0.3 | 1 |
| GO:1902238 | regulation of intrinsic apoptotic signal... | 1 | 0 | 0.04 | 1 |
| GO:1902239 | negative regulation of intrinsic apoptot... | 1 | 0 | 0.04 | 1 |

| | | | | | |
|---|---|---|---|---|---|
| GO:1902250 | regulation of erythrocyte apoptotic proc... | 1 | 0 | 0.04 | 1 |
| GO:1902251 | negative regulation of erythrocyte apopt... | 1 | 0 | 0.04 | 1 |
| GO:1902253 | regulation of intrinsic apoptotic signal... | 20 | 0 | 0.76 | 1 |
| GO:1902254 | negative regulation of intrinsic apoptot... | 15 | 0 | 0.57 | 1 |
| GO:1902255 | positive regulation of intrinsic apoptot... | 4 | 0 | 0.15 | 1 |
| GO:1902259 | regulation of delayed rectifier potassiu... | 10 | 0 | 0.38 | 1 |
| GO:1902260 | negative regulation of delayed rectifier... | 2 | 0 | 0.08 | 1 |
| GO:1902261 | positive regulation of delayed rectifier... | 5 | 0 | 0.19 | 1 |
| GO:1902262 | apoptotic process involved in patterning... | 6 | 0 | 0.23 | 1 |
| GO:1902263 | apoptotic process involved in embryonic ... | 3 | 0 | 0.11 | 1 |
| GO:1902267 | regulation of polyamine transmembrane tr. | 4 | 0 | 0.15 | 1 |
| GO:1902268 | negative regulation of polyamine transme.. | 3 | 0 | 0.11 | 1 |
| GO:1902269 | positive regulation of polyamine transme... | 1 | 0 | 0.04 | 1 |
| GO:1902284 | neuron projection extension involved in ... | 18 | 0 | 0.69 | 1 |
| GO:1902285 | semaphorin-plexin signaling pathway invo. | 6 | 0 | 0.23 | 1 |
| GO:1902287 | semaphorin-plexin signaling pathway invo. | 5 | 0 | 0.19 | 1 |
| GO:1902302 | regulation of potassium ion export | 2 | 0 | 0.08 | 1 |
| GO:1902303 | negative regulation of potassium ion exp... | 1 | 0 | 0.04 | 1 |
| GO:1902304 | positive regulation of potassium ion exp... | 1 | 0 | 0.04 | 1 |
| GO:1902306 | negative regulation of sodium ion transm... | 8 | 0 | 0.3 | 1 |
| GO:1902308 | regulation of peptidyl-serine dephosphor... | 4 | 0 | 0.15 | 1 |
| GO:1902309 | negative regulation of peptidyl-serine d... | 2 | 0 | 0.08 | 1 |
| GO:1902310 | positive regulation of peptidyl-serine d... | 2 | 0 | 0.08 | 1 |
| GO:1902311 | regulation of copper ion transmembrane t.. | 1 | 0 | 0.04 | 1 |
| GO:1902336 | positive regulation of retinal ganglion ... | 2 | 0 | 0.08 | 1 |
| GO:1902339 | positive regulation of apoptotic process... | 4 | 0 | 0.15 | 1 |
| GO:1902359 | Notch signaling pathway involved in somi.. | 1 | 0 | 0.04 | 1 |
| GO:1902362 | melanocyte apoptotic process | 1 | 0 | 0.04 | 1 |
| GO:1902366 | regulation of Notch signaling pathway in... | 1 | 0 | 0.04 | 1 |
| GO:1902367 | negative regulation of Notch signaling p... | 1 | 0 | 0.04 | 1 |
| GO:1902369 | negative regulation of RNA catabolic pro... | 6 | 0 | 0.23 | 1 |
| GO:1902373 | negative regulation of mRNA catabolic pr... | 5 | 0 | 0.19 | 1 |
| GO:1902378 | VEGF-activated neuropilin signaling path.. | 1 | 0 | 0.04 | 1 |
| GO:1902396 | protein localization to tight junction | 1 | 0 | 0.04 | 1 |
| GO:1902410 | mitotic cytokinetic process | 3 | 0 | 0.11 | 1 |
| GO:1902414 | protein localization to cell junction | 6 | 0 | 0.23 | 1 |
| GO:1902415 | regulation of mRNA binding | 1 | 0 | 0.04 | 1 |
| GO:1902416 | positive regulation of mRNA binding | 1 | 0 | 0.04 | 1 |
| GO:1902423 | regulation of mitotic attachment of spin... | 1 | 0 | 0.04 | 1 |
| GO:1902425 | positive regulation of attachment of mit... | 1 | 0 | 0.04 | 1 |
| GO:1902430 | negative regulation of beta-amyloid form... | 6 | 0 | 0.23 | 1 |
| GO:1902442 | regulation of ripoptosome assembly invol.. | 2 | 0 | 0.08 | 1 |
| GO:1902443 | negative regulation of ripoptosome assem. | 2 | 0 | 0.08 | 1 |
| GO:1902445 | regulation of mitochondrial membrane per. | 3 | 0 | 0.11 | 1 |
| GO:1902452 | regulation of autophagic vacuole fusion | 1 | 0 | 0.04 | 1 |
| GO:1902455 | negative regulation of stem cell mainten... | 4 | 0 | 0.15 | 1 |
| GO:1902459 | positive regulation of stem cell mainten... | 2 | 0 | 0.08 | 1 |
| GO:1902460 | regulation of mesenchymal stem cell prol.. | 1 | 0 | 0.04 | 1 |
| GO:1902462 | positive regulation of mesenchymal stem .. | 1 | 0 | 0.04 | 1 |
| GO:1902463 | protein localization to cell leading edg... | 2 | 0 | 0.08 | 1 |
| GO:1902464 | regulation of histone H3-K27 trimethylat... | 1 | 0 | 0.04 | 1 |
| GO:1902466 | positive regulation of histone H3-K27 tr... | 1 | 0 | 0.04 | 1 |
| GO:1902473 | regulation of protein localization to sy... | 1 | 0 | 0.04 | 1 |

| | | | | | |
|---|---|---|---|---|---|
| GO:1902474 | positive regulation of protein localizat... | 1 | 0 | 0.04 | 1 |
| GO:1902498 | regulation of protein autoubiquitination | 3 | 0 | 0.11 | 1 |
| GO:1902499 | positive regulation of protein autoubiqu... | 2 | 0 | 0.08 | 1 |
| GO:1902504 | regulation of signal transduction involv... | 1 | 0 | 0.04 | 1 |
| GO:1902510 | regulation of apoptotic DNA fragmentatio... | 7 | 0 | 0.27 | 1 |
| GO:1902511 | negative regulation of apoptotic DNA fra... | 1 | 0 | 0.04 | 1 |
| GO:1902512 | positive regulation of apoptotic DNA fra... | 1 | 0 | 0.04 | 1 |
| GO:1902513 | regulation of organelle transport along ... | 3 | 0 | 0.11 | 1 |
| GO:1902514 | regulation of generation of L-type calci... | 4 | 0 | 0.15 | 1 |
| GO:1902523 | positive regulation of protein K63-linke... | 1 | 0 | 0.04 | 1 |
| GO:1902524 | positive regulation of protein K48-linke... | 1 | 0 | 0.04 | 1 |
| GO:1902525 | regulation of protein monoubiquitination | 2 | 0 | 0.08 | 1 |
| GO:1902527 | positive regulation of protein monoubiqu... | 1 | 0 | 0.04 | 1 |
| GO:1902528 | regulation of protein linear polyubiquit... | 2 | 0 | 0.08 | 1 |
| GO:1902530 | positive regulation of protein linear po... | 2 | 0 | 0.08 | 1 |
| GO:1902534 | single-organism membrane invagination | 2 | 0 | 0.08 | 1 |
| GO:1902544 | regulation of DNA N-glycosylase activity | 1 | 0 | 0.04 | 1 |
| GO:1902546 | positive regulation of DNA N-glycosylase... | 1 | 0 | 0.04 | 1 |
| GO:1902547 | regulation of cellular response to vascu... | 8 | 0 | 0.3 | 1 |
| GO:1902548 | negative regulation of cellular response... | 4 | 0 | 0.15 | 1 |
| GO:1902559 | 3'-phospho-5'-adenylyl sulfate transmemb. | 1 | 0 | 0.04 | 1 |
| GO:1902563 | regulation of neutrophil activation | 4 | 0 | 0.15 | 1 |
| GO:1902564 | negative regulation of neutrophil activa... | 3 | 0 | 0.11 | 1 |
| GO:1902566 | regulation of eosinophil activation | 4 | 0 | 0.15 | 1 |
| GO:1902567 | negative regulation of eosinophil activa... | 2 | 0 | 0.08 | 1 |
| GO:1902568 | positive regulation of eosinophil activa... | 2 | 0 | 0.08 | 1 |
| GO:1902569 | negative regulation of activation of JAK... | 1 | 0 | 0.04 | 1 |
| GO:1902571 | regulation of serine-type peptidase acti... | 5 | 0 | 0.19 | 1 |
| GO:1902572 | negative regulation of serine-type pepti... | 5 | 0 | 0.19 | 1 |
| GO:1902573 | positive regulation of serine-type pepti... | 1 | 0 | 0.04 | 1 |
| GO:1902576 | negative regulation of nuclear cell cycl... | 3 | 0 | 0.11 | 1 |
| GO:1902594 | multi-organism nuclear import | 2 | 0 | 0.08 | 1 |
| GO:1902598 | creatine transmembrane transport | 2 | 0 | 0.08 | 1 |
| GO:1902605 | heterotrimeric G-protein complex assembl. | 1 | 0 | 0.04 | 1 |
| GO:1902606 | regulation of large conductance calcium-... | 2 | 0 | 0.08 | 1 |
| GO:1902608 | positive regulation of large conductance... | 2 | 0 | 0.08 | 1 |
| GO:1902612 | regulation of anti-Mullerian hormone sig... | 1 | 0 | 0.04 | 1 |
| GO:1902613 | negative regulation of anti-Mullerian ho... | 1 | 0 | 0.04 | 1 |
| GO:1902616 | acyl carnitine transmembrane transport | 1 | 0 | 0.04 | 1 |
| GO:1902617 | response to fluoride | 2 | 0 | 0.08 | 1 |
| GO:1902618 | cellular response to fluoride | 1 | 0 | 0.04 | 1 |
| GO:1902623 | negative regulation of neutrophil migrat... | 1 | 0 | 0.04 | 1 |
| GO:1902630 | regulation of membrane hyperpolarization | 1 | 0 | 0.04 | 1 |
| GO:1902632 | positive regulation of membrane hyperpol. | 1 | 0 | 0.04 | 1 |
| GO:1902652 | secondary alcohol metabolic process | 4 | 0 | 0.15 | 1 |
| GO:1902656 | calcium ion import into cytosol | 1 | 0 | 0.04 | 1 |
| GO:1902667 | regulation of axon guidance | 17 | 0 | 0.65 | 1 |
| GO:1902668 | negative regulation of axon guidance | 11 | 0 | 0.42 | 1 |
| GO:1902669 | positive regulation of axon guidance | 7 | 0 | 0.27 | 1 |
| GO:1902683 | regulation of receptor localization to s... | 1 | 0 | 0.04 | 1 |
| GO:1902685 | positive regulation of receptor localiza... | 1 | 0 | 0.04 | 1 |
| GO:1902692 | regulation of neuroblast proliferation | 28 | 0 | 1.07 | 1 |
| GO:1902713 | regulation of interferon-gamma secretion | 4 | 0 | 0.15 | 1 |

| | | | | | |
|---|---|---|---|---|---|
| GO:1902715 | positive regulation of interferon-gamma ... | 4 | 0 | 0.15 | 1 |
| GO:1902723 | negative regulation of skeletal muscle s... | 2 | 0 | 0.08 | 1 |
| GO:1902725 | negative regulation of satellite cell di... | 2 | 0 | 0.08 | 1 |
| GO:1902730 | positive regulation of proteoglycan bios... | 2 | 0 | 0.08 | 1 |
| GO:1902731 | negative regulation of chondrocyte proli... | 2 | 0 | 0.08 | 1 |
| GO:1902732 | positive regulation of chondrocyte proli... | 2 | 0 | 0.08 | 1 |
| GO:1902744 | negative regulation of lamellipodium org... | 4 | 0 | 0.15 | 1 |
| GO:1902765 | L-arginine import into cell | 1 | 0 | 0.04 | 1 |
| GO:1902766 | skeletal muscle satellite cell migration | 2 | 0 | 0.08 | 1 |
| GO:1902769 | regulation of choline O-acetyltransferas... | 1 | 0 | 0.04 | 1 |
| GO:1902771 | positive regulation of choline O-acetylt... | 1 | 0 | 0.04 | 1 |
| GO:1902774 | late endosome to lysosome transport | 3 | 0 | 0.11 | 1 |
| GO:1902803 | regulation of synaptic vesicle transport | 24 | 0 | 0.91 | 1 |
| GO:1902804 | negative regulation of synaptic vesicle ... | 3 | 0 | 0.11 | 1 |
| GO:1902805 | positive regulation of synaptic vesicle ... | 4 | 0 | 0.15 | 1 |
| GO:1902809 | regulation of skeletal muscle fiber diff... | 2 | 0 | 0.08 | 1 |
| GO:1902811 | positive regulation of skeletal muscle f... | 2 | 0 | 0.08 | 1 |
| GO:1902822 | regulation of late endosome to lysosome .. | 3 | 0 | 0.11 | 1 |
| GO:1902823 | negative regulation of late endosome to ... | 2 | 0 | 0.08 | 1 |
| GO:1902824 | positive regulation of late endosome to ... | 1 | 0 | 0.04 | 1 |
| GO:1902837 | amino acid import into cell | 4 | 0 | 0.15 | 1 |
| GO:1902847 | regulation of neuronal signal transducti... | 1 | 0 | 0.04 | 1 |
| GO:1902850 | microtubule cytoskeleton organization in... | 30 | 0 | 1.14 | 1 |
| GO:1902855 | regulation of nonmotile primary cilium a... | 1 | 0 | 0.04 | 1 |
| GO:1902857 | positive regulation of nonmotile primary... | 1 | 0 | 0.04 | 1 |
| GO:1902861 | copper ion import into cell | 2 | 0 | 0.08 | 1 |
| GO:1902866 | regulation of retina development in came... | 3 | 0 | 0.11 | 1 |
| GO:1902890 | regulation of root hair elongation | 1 | 0 | 0.04 | 1 |
| GO:1902891 | negative regulation of root hair elongat... | 1 | 0 | 0.04 | 1 |
| GO:1902893 | regulation of pri-miRNA transcription fr... | 3 | 0 | 0.11 | 1 |
| GO:1902895 | positive regulation of pri-miRNA transcr... | 3 | 0 | 0.11 | 1 |
| GO:1902897 | regulation of postsynaptic density prote... | 1 | 0 | 0.04 | 1 |
| GO:1902902 | negative regulation of autophagic vacuol... | 4 | 0 | 0.15 | 1 |
| GO:1902903 | regulation of fibril organization | 3 | 0 | 0.11 | 1 |
| GO:1902904 | negative regulation of fibril organizati... | 1 | 0 | 0.04 | 1 |
| GO:1902905 | positive regulation of fibril organizati... | 1 | 0 | 0.04 | 1 |
| GO:1902908 | regulation of melanosome transport | 1 | 0 | 0.04 | 1 |
| GO:1902910 | positive regulation of melanosome transp.. | 1 | 0 | 0.04 | 1 |
| GO:1902913 | positive regulation of neuroepithelial c... | 6 | 0 | 0.23 | 1 |
| GO:1902914 | regulation of protein polyubiquitination | 8 | 0 | 0.3 | 1 |
| GO:1902915 | negative regulation of protein polyubiqu... | 4 | 0 | 0.15 | 1 |
| GO:1902916 | positive regulation of protein polyubiqu... | 3 | 0 | 0.11 | 1 |
| GO:1902938 | regulation of intracellular calcium acti... | 1 | 0 | 0.04 | 1 |
| GO:1902939 | negative regulation of intracellular cal... | 1 | 0 | 0.04 | 1 |
| GO:1902941 | regulation of voltage-gated chloride cha... | 1 | 0 | 0.04 | 1 |
| GO:1902943 | positive regulation of voltage-gated chl... | 1 | 0 | 0.04 | 1 |
| GO:1902946 | protein localization to early endosome | 3 | 0 | 0.11 | 1 |
| GO:1902947 | regulation of tau-protein kinase activit... | 3 | 0 | 0.11 | 1 |
| GO:1902948 | negative regulation of tau-protein kinas... | 1 | 0 | 0.04 | 1 |
| GO:1902949 | positive regulation of tau-protein kinas... | 1 | 0 | 0.04 | 1 |
| GO:1902950 | regulation of dendritic spine maintenanc... | 1 | 0 | 0.04 | 1 |
| GO:1902951 | negative regulation of dendritic spine m... | 1 | 0 | 0.04 | 1 |
| GO:1902952 | positive regulation of dendritic spine m... | 1 | 0 | 0.04 | 1 |

| | | | | | |
|---|---|---|---|---|---|
| GO:1902953 | positive regulation of ER to Golgi vesic... | 3 | 0 | 0.11 | 1 |
| GO:1902954 | regulation of early endosome to recyclin... | 2 | 0 | 0.08 | 1 |
| GO:1902955 | positive regulation of early endosome to... | 1 | 0 | 0.04 | 1 |
| GO:1902956 | regulation of mitochondrial electron tra... | 4 | 0 | 0.15 | 1 |
| GO:1902957 | negative regulation of mitochondrial ele... | 2 | 0 | 0.08 | 1 |
| GO:1902958 | positive regulation of mitochondrial ele... | 2 | 0 | 0.08 | 1 |
| GO:1902959 | regulation of aspartic-type endopeptidas... | 2 | 0 | 0.08 | 1 |
| GO:1902960 | negative regulation of aspartic-type end... | 1 | 0 | 0.04 | 1 |
| GO:1902961 | positive regulation of aspartic-type end... | 1 | 0 | 0.04 | 1 |
| GO:1902962 | regulation of metalloendopeptidase activ... | 2 | 0 | 0.08 | 1 |
| GO:1902963 | negative regulation of metalloendopeptid... | 2 | 0 | 0.08 | 1 |
| GO:1902965 | regulation of protein localization to ea... | 2 | 0 | 0.08 | 1 |
| GO:1902966 | positive regulation of protein localizat... | 2 | 0 | 0.08 | 1 |
| GO:1902969 | mitotic DNA replication | 2 | 0 | 0.08 | 1 |
| GO:1902988 | neurofibrillary tangle assembly | 3 | 0 | 0.11 | 1 |
| GO:1902991 | regulation of amyloid precursor protein ... | 11 | 0 | 0.42 | 1 |
| GO:1902992 | negative regulation of amyloid precursor... | 8 | 0 | 0.3 | 1 |
| GO:1902993 | positive regulation of amyloid precursor... | 4 | 0 | 0.15 | 1 |
| GO:1902994 | regulation of phospholipid efflux | 2 | 0 | 0.08 | 1 |
| GO:1902995 | positive regulation of phospholipid effl... | 2 | 0 | 0.08 | 1 |
| GO:1902996 | regulation of neurofibrillary tangle ass... | 3 | 0 | 0.11 | 1 |
| GO:1902997 | negative regulation of neurofibrillary t... | 1 | 0 | 0.04 | 1 |
| GO:1902998 | positive regulation of neurofibrillary t... | 2 | 0 | 0.08 | 1 |
| GO:1902999 | negative regulation of phospholipid effl... | 1 | 0 | 0.04 | 1 |
| GO:1903000 | regulation of lipid transport across blo... | 1 | 0 | 0.04 | 1 |
| GO:1903001 | negative regulation of lipid transport a... | 1 | 0 | 0.04 | 1 |
| GO:1903002 | positive regulation of lipid transport a... | 1 | 0 | 0.04 | 1 |
| GO:1903003 | positive regulation of protein deubiquit... | 2 | 0 | 0.08 | 1 |
| GO:1903004 | regulation of protein K63-linked deubiqu... | 1 | 0 | 0.04 | 1 |
| GO:1903006 | positive regulation of protein K63-linke... | 1 | 0 | 0.04 | 1 |
| GO:1903007 | positive regulation of Lys63-specific de... | 1 | 0 | 0.04 | 1 |
| GO:1903010 | regulation of bone development | 11 | 0 | 0.42 | 1 |
| GO:1903011 | negative regulation of bone development | 3 | 0 | 0.11 | 1 |
| GO:1903012 | positive regulation of bone development | 5 | 0 | 0.19 | 1 |
| GO:1903015 | regulation of exo-alpha-sialidase activi... | 2 | 0 | 0.08 | 1 |
| GO:1903016 | negative regulation of exo-alpha-sialida... | 2 | 0 | 0.08 | 1 |
| GO:1903020 | positive regulation of glycoprotein meta... | 12 | 0 | 0.46 | 1 |
| GO:1903025 | regulation of RNA polymerase II regulato... | 2 | 0 | 0.08 | 1 |
| GO:1903026 | negative regulation of RNA polymerase II.. | 2 | 0 | 0.08 | 1 |
| GO:1903027 | regulation of opsonization | 2 | 0 | 0.08 | 1 |
| GO:1903031 | regulation of microtubule plus-end bindi... | 2 | 0 | 0.08 | 1 |
| GO:1903033 | positive regulation of microtubule plus-... | 2 | 0 | 0.08 | 1 |
| GO:1903044 | protein localization to membrane raft | 7 | 0 | 0.27 | 1 |
| GO:1903045 | neural crest cell migration involved in ... | 1 | 0 | 0.04 | 1 |
| GO:1903053 | regulation of extracellular matrix organ... | 22 | 0 | 0.84 | 1 |
| GO:1903054 | negative regulation of extracellular mat... | 5 | 0 | 0.19 | 1 |
| GO:1903055 | positive regulation of extracellular mat... | 8 | 0 | 0.3 | 1 |
| GO:1903056 | regulation of melanosome organization | 1 | 0 | 0.04 | 1 |
| GO:1903059 | regulation of protein lipidation | 2 | 0 | 0.08 | 1 |
| GO:1903060 | negative regulation of protein lipidatio... | 1 | 0 | 0.04 | 1 |
| GO:1903061 | positive regulation of protein lipidatio... | 1 | 0 | 0.04 | 1 |
| GO:1903069 | regulation of ER-associated ubiquitin-de... | 4 | 0 | 0.15 | 1 |
| GO:1903070 | negative regulation of ER-associated ubi... | 2 | 0 | 0.08 | 1 |

| | | | | | |
|---|---|---|---|---|---|
| GO:1903071 | positive regulation of ER-associated ubi... | 2 | 0 | 0.08 | 1 |
| GO:1903072 | regulation of death-inducing signaling c... | 1 | 0 | 0.04 | 1 |
| GO:1903073 | negative regulation of death-inducing si... | 1 | 0 | 0.04 | 1 |
| GO:1903080 | regulation of C-C chemokine receptor CCF | 1 | 0 | 0.04 | 1 |
| GO:1903082 | positive regulation of C-C chemokine rec... | 1 | 0 | 0.04 | 1 |
| GO:1903093 | regulation of protein K48-linked deubiqu... | 1 | 0 | 0.04 | 1 |
| GO:1903094 | negative regulation of protein K48-linke... | 1 | 0 | 0.04 | 1 |
| GO:1903118 | urate homeostasis | 1 | 0 | 0.04 | 1 |
| GO:1903121 | regulation of TRAIL-activated apoptotic ... | 1 | 0 | 0.04 | 1 |
| GO:1903122 | negative regulation of TRAIL-activated a... | 1 | 0 | 0.04 | 1 |
| GO:1903125 | negative regulation of thioredoxin perox... | 1 | 0 | 0.04 | 1 |
| GO:1903126 | negative regulation of centriole-centrio... | 1 | 0 | 0.04 | 1 |
| GO:1903140 | regulation of establishment of endotheli... | 2 | 0 | 0.08 | 1 |
| GO:1903141 | negative regulation of establishment of ... | 1 | 0 | 0.04 | 1 |
| GO:1903142 | positive regulation of establishment of ... | 1 | 0 | 0.04 | 1 |
| GO:1903147 | negative regulation of mitochondrion deg... | 2 | 0 | 0.08 | 1 |
| GO:1903167 | regulation of pyrroline-5-carboxylate re... | 1 | 0 | 0.04 | 1 |
| GO:1903168 | positive regulation of pyrroline-5-carbo... | 1 | 0 | 0.04 | 1 |
| GO:1903170 | negative regulation of calcium ion trans... | 21 | 0 | 0.8 | 1 |
| GO:1903173 | fatty alcohol metabolic process | 2 | 0 | 0.08 | 1 |
| GO:1903176 | regulation of tyrosine 3-monooxygenase a | 1 | 0 | 0.04 | 1 |
| GO:1903178 | positive regulation of tyrosine 3-monoox... | 1 | 0 | 0.04 | 1 |
| GO:1903179 | regulation of dopamine biosynthetic proc... | 1 | 0 | 0.04 | 1 |
| GO:1903181 | positive regulation of dopamine biosynth... | 1 | 0 | 0.04 | 1 |
| GO:1903182 | regulation of SUMO transferase activity | 1 | 0 | 0.04 | 1 |
| GO:1903184 | L-dopa metabolic process | 1 | 0 | 0.04 | 1 |
| GO:1903185 | L-dopa biosynthetic process | 1 | 0 | 0.04 | 1 |
| GO:1903189 | glyoxal metabolic process | 1 | 0 | 0.04 | 1 |
| GO:1903190 | glyoxal catabolic process | 1 | 0 | 0.04 | 1 |
| GO:1903195 | regulation of L-dopa biosynthetic proces... | 1 | 0 | 0.04 | 1 |
| GO:1903197 | positive regulation of L-dopa biosynthet... | 1 | 0 | 0.04 | 1 |
| GO:1903198 | regulation of L-dopa decarboxylase activ... | 1 | 0 | 0.04 | 1 |
| GO:1903200 | positive regulation of L-dopa decarboxyl... | 1 | 0 | 0.04 | 1 |
| GO:1903203 | regulation of oxidative stress-induced n... | 9 | 0 | 0.34 | 1 |
| GO:1903204 | negative regulation of oxidative stress-... | 7 | 0 | 0.27 | 1 |
| GO:1903207 | regulation of hydrogen peroxide-induced .. | 3 | 0 | 0.11 | 1 |
| GO:1903208 | negative regulation of hydrogen peroxide.. | 3 | 0 | 0.11 | 1 |
| GO:1903215 | negative regulation of protein targeting... | 1 | 0 | 0.04 | 1 |
| GO:1903216 | regulation of protein processing involve... | 1 | 0 | 0.04 | 1 |
| GO:1903217 | negative regulation of protein processin... | 1 | 0 | 0.04 | 1 |
| GO:1903223 | positive regulation of oxidative stress-... | 3 | 0 | 0.11 | 1 |
| GO:1903225 | negative regulation of endodermal cell d... | 3 | 0 | 0.11 | 1 |
| GO:1903232 | melanosome assembly | 2 | 0 | 0.08 | 1 |
| GO:1903233 | regulation of calcium ion-dependent exoc.. | 1 | 0 | 0.04 | 1 |
| GO:1903236 | regulation of leukocyte tethering or rol... | 4 | 0 | 0.15 | 1 |
| GO:1903237 | negative regulation of leukocyte tetheri... | 4 | 0 | 0.15 | 1 |
| GO:1903242 | regulation of cardiac muscle hypertrophy... | 4 | 0 | 0.15 | 1 |
| GO:1903243 | negative regulation of cardiac muscle hy... | 1 | 0 | 0.04 | 1 |
| GO:1903244 | positive regulation of cardiac muscle hy... | 2 | 0 | 0.08 | 1 |
| GO:1903248 | regulation of citrulline biosynthetic pr... | 1 | 0 | 0.04 | 1 |
| GO:1903249 | negative regulation of citrulline biosyn... | 1 | 0 | 0.04 | 1 |
| GO:1903251 | multi-ciliated epithelial cell different... | 3 | 0 | 0.11 | 1 |
| GO:1903265 | positive regulation of tumor necrosis fa... | 1 | 0 | 0.04 | 1 |

| | | | | | |
|---|---|---|---|---|---|
| GO:1903273 | regulation of sodium ion export | 4 | 0 | 0.15 | 1 |
| GO:1903275 | positive regulation of sodium ion export | 4 | 0 | 0.15 | 1 |
| GO:1903276 | regulation of sodium ion export from cel... | 4 | 0 | 0.15 | 1 |
| GO:1903278 | positive regulation of sodium ion export... | 4 | 0 | 0.15 | 1 |
| GO:1903279 | regulation of calcium:sodium antiporter ... | 3 | 0 | 0.11 | 1 |
| GO:1903280 | negative regulation of calcium:sodium an.. | 1 | 0 | 0.04 | 1 |
| GO:1903281 | positive regulation of calcium:sodium an... | 2 | 0 | 0.08 | 1 |
| GO:1903282 | regulation of glutathione peroxidase act... | 1 | 0 | 0.04 | 1 |
| GO:1903284 | positive regulation of glutathione perox... | 1 | 0 | 0.04 | 1 |
| GO:1903285 | positive regulation of hydrogen peroxide... | 1 | 0 | 0.04 | 1 |
| GO:1903286 | regulation of potassium ion import | 3 | 0 | 0.11 | 1 |
| GO:1903288 | positive regulation of potassium ion imp... | 3 | 0 | 0.11 | 1 |
| GO:1903294 | regulation of glutamate secretion | neuro... | 1 | 0 | 0.04 |
| GO:1903296 | positive regulation of glutamate secreti... | 1 | 0 | 0.04 | 1 |
| GO:1903297 | regulation of hypoxia-induced intrinsic ... | 3 | 0 | 0.11 | 1 |
| GO:1903298 | negative regulation of hypoxia-induced i... | 3 | 0 | 0.11 | 1 |
| GO:1903299 | regulation of hexokinase activity | 10 | 0 | 0.38 | 1 |
| GO:1903300 | negative regulation of hexokinase activi... | 6 | 0 | 0.23 | 1 |
| GO:1903301 | positive regulation of hexokinase activi... | 5 | 0 | 0.19 | 1 |
| GO:1903307 | positive regulation of regulated secreto... | 22 | 0 | 0.84 | 1 |
| GO:1903312 | negative regulation of mRNA metabolic pr. | 24 | 0 | 0.91 | 1 |
| GO:1903314 | regulation of nitrogen cycle metabolic p... | 1 | 0 | 0.04 | 1 |
| GO:1903332 | regulation of protein folding | 5 | 0 | 0.19 | 1 |
| GO:1903333 | negative regulation of protein folding | 4 | 0 | 0.15 | 1 |
| GO:1903335 | regulation of vacuolar transport | 4 | 0 | 0.15 | 1 |
| GO:1903336 | negative regulation of vacuolar transpor... | 2 | 0 | 0.08 | 1 |
| GO:1903337 | positive regulation of vacuolar transpor... | 1 | 0 | 0.04 | 1 |
| GO:1903350 | response to dopamine | 3 | 0 | 0.11 | 1 |
| GO:1903351 | cellular response to dopamine | 3 | 0 | 0.11 | 1 |
| GO:1903352 | L-ornithine transmembrane transport | 2 | 0 | 0.08 | 1 |
| GO:1903358 | regulation of Golgi organization | 7 | 0 | 0.27 | 1 |
| GO:1903361 | protein localization to basolateral plas... | 5 | 0 | 0.19 | 1 |
| GO:1903365 | regulation of fear response | 4 | 0 | 0.15 | 1 |
| GO:1903367 | positive regulation of fear response | 4 | 0 | 0.15 | 1 |
| GO:1903371 | regulation of endoplasmic reticulum tubu... | 2 | 0 | 0.08 | 1 |
| GO:1903373 | positive regulation of endoplasmic retic... | 2 | 0 | 0.08 | 1 |
| GO:1903375 | facioacoustic ganglion development | 2 | 0 | 0.08 | 1 |
| GO:1903376 | regulation of oxidative stress-induced n... | 7 | 0 | 0.27 | 1 |
| GO:1903377 | negative regulation of oxidative stress-... | 5 | 0 | 0.19 | 1 |
| GO:1903378 | positive regulation of oxidative stress-... | 3 | 0 | 0.11 | 1 |
| GO:1903381 | regulation of endoplasmic reticulum stre... | 1 | 0 | 0.04 | 1 |
| GO:1903382 | negative regulation of endoplasmic retic... | 1 | 0 | 0.04 | 1 |
| GO:1903383 | regulation of hydrogen peroxide-induced .. | 2 | 0 | 0.08 | 1 |
| GO:1903384 | negative regulation of hydrogen peroxide.. | 2 | 0 | 0.08 | 1 |
| GO:1903385 | regulation of homophilic cell adhesion | 1 | 0 | 0.04 | 1 |
| GO:1903387 | positive regulation of homophilic cell a... | 1 | 0 | 0.04 | 1 |
| GO:1903388 | regulation of synaptic vesicle uncoating | 1 | 0 | 0.04 | 1 |
| GO:1903390 | positive regulation of synaptic vesicle ... | 1 | 0 | 0.04 | 1 |
| GO:1903393 | positive regulation of adherens junction... | 20 | 0 | 0.76 | 1 |
| GO:1903400 | L-arginine transmembrane transport | 1 | 0 | 0.04 | 1 |
| GO:1903401 | L-lysine transmembrane transport | 3 | 0 | 0.11 | 1 |
| GO:1903402 | regulation of renal phosphate excretion | 1 | 0 | 0.04 | 1 |
| GO:1903403 | negative regulation of renal phosphate e... | 1 | 0 | 0.04 | 1 |

| | | | | | |
|---|---|---|---|---|---|
| GO:1903410 | L-lysine import into cell | 1 | 0 | 0.04 | 1 |
| GO:1903411 | L-ornithine import into cell | 1 | 0 | 0.04 | 1 |
| GO:1903412 | response to bile acid | 1 | 0 | 0.04 | 1 |
| GO:1903413 | cellular response to bile acid | 1 | 0 | 0.04 | 1 |
| GO:1903416 | response to glycoside | 3 | 0 | 0.11 | 1 |
| GO:1903420 | protein localization to endoplasmic reti... | 1 | 0 | 0.04 | 1 |
| GO:1903421 | regulation of synaptic vesicle recycling | 6 | 0 | 0.23 | 1 |
| GO:1903423 | positive regulation of synaptic vesicle ... | 3 | 0 | 0.11 | 1 |
| GO:1903429 | regulation of cell maturation | 13 | 0 | 0.49 | 1 |
| GO:1903430 | negative regulation of cell maturation | 7 | 0 | 0.27 | 1 |
| GO:1903431 | positive regulation of cell maturation | 5 | 0 | 0.19 | 1 |
| GO:1903433 | regulation of constitutive secretory pat... | 2 | 0 | 0.08 | 1 |
| GO:1903434 | negative regulation of constitutive secr... | 1 | 0 | 0.04 | 1 |
| GO:1903435 | positive regulation of constitutive secr... | 1 | 0 | 0.04 | 1 |
| GO:1903441 | protein localization to ciliary membrane | 2 | 0 | 0.08 | 1 |
| GO:1903461 | Okazaki fragment processing involved in .. | 1 | 0 | 0.04 | 1 |
| GO:1903463 | regulation of mitotic cell cycle DNA rep... | 1 | 0 | 0.04 | 1 |
| GO:1903464 | negative regulation of mitotic cell cycl... | 1 | 0 | 0.04 | 1 |
| GO:1903469 | removal of RNA primer involved in mitoti... | 1 | 0 | 0.04 | 1 |
| GO:1903487 | regulation of lactation | 2 | 0 | 0.08 | 1 |
| GO:1903488 | negative regulation of lactation | 1 | 0 | 0.04 | 1 |
| GO:1903489 | positive regulation of lactation | 1 | 0 | 0.04 | 1 |
| GO:1903515 | calcium ion transport from cytosol to en... | 1 | 0 | 0.04 | 1 |
| GO:1903519 | regulation of mammary gland involution | 4 | 0 | 0.15 | 1 |
| GO:1903521 | positive regulation of mammary gland inv.. | 4 | 0 | 0.15 | 1 |
| GO:1903525 | regulation of membrane tubulation | 2 | 0 | 0.08 | 1 |
| GO:1903526 | negative regulation of membrane tubulati.. | 2 | 0 | 0.08 | 1 |
| GO:1903541 | regulation of exosomal secretion | 12 | 0 | 0.46 | 1 |
| GO:1903542 | negative regulation of exosomal secretio... | 1 | 0 | 0.04 | 1 |
| GO:1903543 | positive regulation of exosomal secretio... | 12 | 0 | 0.46 | 1 |
| GO:1903546 | protein localization to photoreceptor ou... | 1 | 0 | 0.04 | 1 |
| GO:1903551 | regulation of extracellular vesicular ex... | 6 | 0 | 0.23 | 1 |
| GO:1903553 | positive regulation of extracellular ves... | 4 | 0 | 0.15 | 1 |
| GO:1903556 | negative regulation of tumor necrosis fa... | 34 | 0 | 1.29 | 1 |
| GO:1903564 | regulation of protein localization to ci... | 1 | 0 | 0.04 | 1 |
| GO:1903565 | negative regulation of protein localizat... | 1 | 0 | 0.04 | 1 |
| GO:1903567 | regulation of protein localization to ci... | 1 | 0 | 0.04 | 1 |
| GO:1903568 | negative regulation of protein localizat... | 1 | 0 | 0.04 | 1 |
| GO:1903570 | regulation of protein kinase D signaling | 1 | 0 | 0.04 | 1 |
| GO:1903572 | positive regulation of protein kinase D ... | 1 | 0 | 0.04 | 1 |
| GO:1903578 | regulation of ATP metabolic process | 13 | 0 | 0.49 | 1 |
| GO:1903579 | negative regulation of ATP metabolic pro... | 6 | 0 | 0.23 | 1 |
| GO:1903580 | positive regulation of ATP metabolic pro... | 4 | 0 | 0.15 | 1 |
| GO:1903584 | regulation of histone deubiquitination | 1 | 0 | 0.04 | 1 |
| GO:1903586 | positive regulation of histone deubiquit... | 1 | 0 | 0.04 | 1 |
| GO:1903587 | regulation of blood vessel endothelial c... | 3 | 0 | 0.11 | 1 |
| GO:1903588 | negative regulation of blood vessel endo... | 2 | 0 | 0.08 | 1 |
| GO:1903589 | positive regulation of blood vessel endo... | 1 | 0 | 0.04 | 1 |
| GO:1903593 | regulation of histamine secretion by mas... | 3 | 0 | 0.11 | 1 |
| GO:1903595 | positive regulation of histamine secreti... | 3 | 0 | 0.11 | 1 |
| GO:1903596 | regulation of gap junction assembly | 4 | 0 | 0.15 | 1 |
| GO:1903597 | negative regulation of gap junction asse... | 1 | 0 | 0.04 | 1 |
| GO:1903598 | positive regulation of gap junction asse... | 3 | 0 | 0.11 | 1 |

| | | | | | |
|---|---|---|---|---|---|
| GO:1903599 | positive regulation of mitochondrion deg... | 7 | 0 | 0.27 | 1 |
| GO:1903621 | protein localization to photoreceptor co... | 1 | 0 | 0.04 | 1 |
| GO:1903624 | regulation of DNA catabolic process | 7 | 0 | 0.27 | 1 |
| GO:1903625 | negative regulation of DNA catabolic pro... | 1 | 0 | 0.04 | 1 |
| GO:1903626 | positive regulation of DNA catabolic pro... | 1 | 0 | 0.04 | 1 |
| GO:1903636 | regulation of protein import into mitoch... | 1 | 0 | 0.04 | 1 |
| GO:1903638 | positive regulation of protein import in... | 1 | 0 | 0.04 | 1 |
| GO:1903644 | regulation of chaperone-mediated protein.. | 1 | 0 | 0.04 | 1 |
| GO:1903645 | negative regulation of chaperone-mediate. | 1 | 0 | 0.04 | 1 |
| GO:1903659 | regulation of complement-dependent cytot | 2 | 0 | 0.08 | 1 |
| GO:1903660 | negative regulation of complement-depenc | 2 | 0 | 0.08 | 1 |
| GO:1903670 | regulation of sprouting angiogenesis | 18 | 0 | 0.69 | 1 |
| GO:1903671 | negative regulation of sprouting angioge... | 8 | 0 | 0.3 | 1 |
| GO:1903672 | positive regulation of sprouting angioge... | 9 | 0 | 0.34 | 1 |
| GO:1903674 | regulation of cap-dependent translationa... | 1 | 0 | 0.04 | 1 |
| GO:1903676 | positive regulation of cap-dependent tra... | 1 | 0 | 0.04 | 1 |
| GO:1903677 | regulation of cap-independent translatio... | 1 | 0 | 0.04 | 1 |
| GO:1903679 | positive regulation of cap-independent t... | 1 | 0 | 0.04 | 1 |
| GO:1903689 | regulation of wound healing | spreading c | 2 | 0 | 0.08 |
| GO:1903691 | positive regulation of wound healing | sp... | 1 | 0 | 0.04 |
| GO:1903697 | negative regulation of microvillus assem... | 1 | 0 | 0.04 | 1 |
| GO:1903704 | negative regulation of production of siR... | 1 | 0 | 0.04 | 1 |
| GO:1903712 | cysteine transmembrane transport | 1 | 0 | 0.04 | 1 |
| GO:1903719 | regulation of I-kappaB phosphorylation | 1 | 0 | 0.04 | 1 |
| GO:1903721 | positive regulation of I-kappaB phosphor... | 1 | 0 | 0.04 | 1 |
| GO:1903722 | regulation of centriole elongation | 2 | 0 | 0.08 | 1 |
| GO:1903723 | negative regulation of centriole elongat... | 1 | 0 | 0.04 | 1 |
| GO:1903724 | positive regulation of centriole elongat... | 1 | 0 | 0.04 | 1 |
| GO:1903726 | negative regulation of phospholipid meta... | 7 | 0 | 0.27 | 1 |
| GO:1903748 | negative regulation of establishment of ... | 1 | 0 | 0.04 | 1 |
| GO:1903750 | regulation of intrinsic apoptotic signal... | 2 | 0 | 0.08 | 1 |
| GO:1903751 | negative regulation of intrinsic apoptot... | 2 | 0 | 0.08 | 1 |
| GO:1903755 | positive regulation of SUMO transferase ... | 1 | 0 | 0.04 | 1 |
| GO:1903756 | regulation of transcription from RNA pol... | 1 | 0 | 0.04 | 1 |
| GO:1903758 | negative regulation of transcription fro... | 1 | 0 | 0.04 | 1 |
| GO:1903760 | regulation of voltage-gated potassium ch... | 2 | 0 | 0.08 | 1 |
| GO:1903761 | negative regulation of voltage-gated pot... | 1 | 0 | 0.04 | 1 |
| GO:1903762 | positive regulation of voltage-gated pot... | 2 | 0 | 0.08 | 1 |
| GO:1903764 | regulation of potassium ion export acros... | 1 | 0 | 0.04 | 1 |
| GO:1903766 | positive regulation of potassium ion exp... | 1 | 0 | 0.04 | 1 |
| GO:1903770 | negative regulation of beta-galactosidas... | 1 | 0 | 0.04 | 1 |
| GO:1903772 | regulation of viral budding via host ESC... | 4 | 0 | 0.15 | 1 |
| GO:1903774 | positive regulation of viral budding via... | 3 | 0 | 0.11 | 1 |
| GO:1903778 | protein localization to vacuolar membran... | 1 | 0 | 0.04 | 1 |
| GO:1903781 | positive regulation of cardiac conductio... | 1 | 0 | 0.04 | 1 |
| GO:1903796 | negative regulation of inorganic anion t... | 1 | 0 | 0.04 | 1 |
| GO:1903802 | L-glutamate(1-) import into cell | 3 | 0 | 0.11 | 1 |
| GO:1903817 | negative regulation of voltage-gated pot... | 3 | 0 | 0.11 | 1 |
| GO:1903818 | positive regulation of voltage-gated pot... | 5 | 0 | 0.19 | 1 |
| GO:1903826 | arginine transmembrane transport | 4 | 0 | 0.15 | 1 |
| GO:1903837 | regulation of mRNA 3'-UTR binding | 1 | 0 | 0.04 | 1 |
| GO:1903839 | positive regulation of mRNA 3'-UTR bindi.. | 1 | 0 | 0.04 | 1 |
| GO:1903846 | positive regulation of cellular response... | 23 | 0 | 0.88 | 1 |

| | | | | | |
|---|---|---|---|---|---|
| GO:1903850 | regulation of cristae formation | 1 | 0 | 0.04 | 1 |
| GO:1903852 | positive regulation of cristae formation | 1 | 0 | 0.04 | 1 |
| GO:1903862 | positive regulation of oxidative phospho... | 2 | 0 | 0.08 | 1 |
| GO:1903867 | extraembryonic membrane development | 5 | 0 | 0.19 | 1 |
| GO:1903888 | regulation of plant epidermal cell diffe... | 1 | 0 | 0.04 | 1 |
| GO:1903889 | negative regulation of plant epidermal c... | 1 | 0 | 0.04 | 1 |
| GO:1903895 | negative regulation of IRE1-mediated unf.. | 2 | 0 | 0.08 | 1 |
| GO:1903897 | regulation of PERK-mediated unfolded pro | 4 | 0 | 0.15 | 1 |
| GO:1903898 | negative regulation of PERK-mediated unf | 4 | 0 | 0.15 | 1 |
| GO:1903903 | regulation of establishment of T cell po... | 1 | 0 | 0.04 | 1 |
| GO:1903906 | regulation of plasma membrane raft polar.. | 1 | 0 | 0.04 | 1 |
| GO:1903909 | regulation of receptor clustering | 1 | 0 | 0.04 | 1 |
| GO:1903916 | regulation of endoplasmic reticulum stre... | 1 | 0 | 0.04 | 1 |
| GO:1903917 | positive regulation of endoplasmic retic... | 1 | 0 | 0.04 | 1 |
| GO:1903918 | regulation of actin filament severing | 1 | 0 | 0.04 | 1 |
| GO:1903919 | negative regulation of actin filament se... | 1 | 0 | 0.04 | 1 |
| GO:1903921 | regulation of protein processing in phag... | 2 | 0 | 0.08 | 1 |
| GO:1903923 | positive regulation of protein processin... | 2 | 0 | 0.08 | 1 |
| GO:1903946 | negative regulation of ventricular cardi... | 1 | 0 | 0.04 | 1 |
| GO:1903947 | positive regulation of ventricular cardi... | 2 | 0 | 0.08 | 1 |
| GO:1903960 | negative regulation of anion transmembra. | 5 | 0 | 0.19 | 1 |
| GO:1903963 | arachidonate transport | 19 | 0 | 0.72 | 1 |
| GO:1990000 | amyloid fibril formation | 4 | 0 | 0.15 | 1 |
| GO:1990001 | inhibition of cysteine-type endopeptidas... | 10 | 0 | 0.38 | 1 |
| GO:1990009 | retinal cell apoptotic process | 2 | 0 | 0.08 | 1 |
| GO:1990029 | vasomotion | 1 | 0 | 0.04 | 1 |
| GO:1990034 | calcium ion export from cell | 1 | 0 | 0.04 | 1 |
| GO:1990035 | calcium ion import into cell | 2 | 0 | 0.08 | 1 |
| GO:1990036 | calcium ion import into sarcoplasmic ret... | 2 | 0 | 0.08 | 1 |
| GO:1990046 | stress-induced mitochondrial fusion | 1 | 0 | 0.04 | 1 |
| GO:1990074 | polyuridylation-dependent mRNA catabolic | 1 | 0 | 0.04 | 1 |
| GO:1990079 | cartilage homeostasis | 1 | 0 | 0.04 | 1 |
| GO:1990086 | lens fiber cell apoptotic process | 4 | 0 | 0.15 | 1 |
| GO:1990089 | response to nerve growth factor | 18 | 0 | 0.69 | 1 |
| GO:1990090 | cellular response to nerve growth factor... | 18 | 0 | 0.69 | 1 |
| GO:1990117 | B cell receptor apoptotic signaling path... | 1 | 0 | 0.04 | 1 |
| GO:1990118 | sodium ion import into cell | 1 | 0 | 0.04 | 1 |
| GO:1990123 | L-glutamate import into cell | 3 | 0 | 0.11 | 1 |
| GO:1990127 | intrinsic apoptotic signaling pathway in... | 1 | 0 | 0.04 | 1 |
| GO:1990134 | epithelial cell apoptotic process involv... | 1 | 0 | 0.04 | 1 |
| GO:1990144 | intrinsic apoptotic signaling pathway in... | 4 | 0 | 0.15 | 1 |
| GO:1990164 | histone H2A phosphorylation | 3 | 0 | 0.11 | 1 |
| GO:1990166 | protein localization to site of double-s... | 1 | 0 | 0.04 | 1 |
| GO:1990167 | protein K27-linked deubiquitination | 2 | 0 | 0.08 | 1 |
| GO:1990168 | protein K33-linked deubiquitination | 3 | 0 | 0.11 | 1 |
| GO:1990169 | stress response to copper ion | 3 | 0 | 0.11 | 1 |
| GO:1990170 | stress response to cadmium ion | 1 | 0 | 0.04 | 1 |
| GO:1990172 | G-protein coupled receptor catabolic pro... | 1 | 0 | 0.04 | 1 |
| GO:1990180 | mitochondrial tRNA 3'-end processing | 1 | 0 | 0.04 | 1 |
| GO:1990182 | exosomal secretion | 16 | 0 | 0.61 | 1 |
| GO:1990245 | histone H2A-T120 phosphorylation | 1 | 0 | 0.04 | 1 |
| GO:1990256 | signal clustering | 1 | 0 | 0.04 | 1 |
| GO:1990258 | histone glutamine methylation | 1 | 0 | 0.04 | 1 |

| | | | | | |
|---|---|---|---|---|---|
| GO:1990262 | anti-Mullerian hormone signaling pathway | 1 | 0 | 0.04 | 1 |
| GO:1990264 | peptidyl-tyrosine dephosphorylation invo... | 1 | 0 | 0.04 | 1 |
| GO:1990268 | response to gold nanoparticle | 2 | 0 | 0.08 | 1 |
| GO:1990314 | cellular response to insulin-like growth... | 2 | 0 | 0.08 | 1 |
| GO:1990379 | lipid transport across blood brain barri... | 1 | 0 | 0.04 | 1 |
| GO:1990384 | hyaloid vascular plexus regression | 1 | 0 | 0.04 | 1 |
| GO:1990390 | protein K33-linked ubiquitination | 1 | 0 | 0.04 | 1 |
| GO:1990401 | embryonic lung development | 1 | 0 | 0.04 | 1 |
| GO:1990418 | response to insulin-like growth factor s... | 2 | 0 | 0.08 | 1 |
| GO:1990440 | positive regulation of transcription fro... | 10 | 0 | 0.38 | 1 |
| GO:1990441 | negative regulation of transcription fro... | 2 | 0 | 0.08 | 1 |
| GO:1990451 | cellular stress response to acidic pH | 1 | 0 | 0.04 | 1 |
| GO:1990481 | mRNA pseudouridine synthesis | 1 | 0 | 0.04 | 1 |
| GO:1990502 | dense core granule maturation | 2 | 0 | 0.08 | 1 |
| GO:1990519 | mitochondrial pyrimidine nucleotide impo... | 1 | 0 | 0.04 | 1 |
| GO:1990564 | protein polyufmylation | 4 | 0 | 0.15 | 1 |
| GO:1990569 | UDP-N-acetylglucosamine transmembrane | 2 | 0 | 0.08 | 1 |
| GO:1990573 | potassium ion import across plasma memb | 3 | 0 | 0.11 | 1 |
| GO:1990579 | peptidyl-serine trans-autophosphorylatio... | 1 | 0 | 0.04 | 1 |
| GO:2000001 | regulation of DNA damage checkpoint | 10 | 0 | 0.38 | 1 |
| GO:2000002 | negative regulation of DNA damage check | 3 | 0 | 0.11 | 1 |
| GO:2000004 | regulation of metanephric S-shaped body ... | 1 | 0 | 0.04 | 1 |
| GO:2000005 | negative regulation of metanephric S-sha... | 1 | 0 | 0.04 | 1 |
| GO:2000006 | regulation of metanephric comma-shaped | 1 | 0 | 0.04 | 1 |
| GO:2000007 | negative regulation of metanephric comma | 1 | 0 | 0.04 | 1 |
| GO:2000008 | regulation of protein localization to ce... | 24 | 0 | 0.91 | 1 |
| GO:2000009 | negative regulation of protein localizat... | 9 | 0 | 0.34 | 1 |
| GO:2000010 | positive regulation of protein localizat... | 8 | 0 | 0.3 | 1 |
| GO:2000015 | regulation of determination of dorsal id... | 4 | 0 | 0.15 | 1 |
| GO:2000016 | negative regulation of determination of ... | 1 | 0 | 0.04 | 1 |
| GO:2000017 | positive regulation of determination of ... | 3 | 0 | 0.11 | 1 |
| GO:2000018 | regulation of male gonad development | 10 | 0 | 0.38 | 1 |
| GO:2000019 | negative regulation of male gonad develo... | 2 | 0 | 0.08 | 1 |
| GO:2000020 | positive regulation of male gonad develo... | 8 | 0 | 0.3 | 1 |
| GO:2000035 | regulation of stem cell division | 4 | 0 | 0.15 | 1 |
| GO:2000036 | regulation of stem cell maintenance | 17 | 0 | 0.65 | 1 |
| GO:2000040 | regulation of planar cell polarity pathw... | 3 | 0 | 0.11 | 1 |
| GO:2000041 | negative regulation of planar cell polar... | 3 | 0 | 0.11 | 1 |
| GO:2000042 | negative regulation of double-strand bre... | 1 | 0 | 0.04 | 1 |
| GO:2000043 | regulation of cardiac cell fate specific... | 2 | 0 | 0.08 | 1 |
| GO:2000044 | negative regulation of cardiac cell fate... | 1 | 0 | 0.04 | 1 |
| GO:2000047 | regulation of cell-cell adhesion mediate... | 8 | 0 | 0.3 | 1 |
| GO:2000048 | negative regulation of cell-cell adhesio... | 3 | 0 | 0.11 | 1 |
| GO:2000049 | positive regulation of cell-cell adhesio... | 5 | 0 | 0.19 | 1 |
| GO:2000052 | positive regulation of non-canonical Wnt... | 9 | 0 | 0.34 | 1 |
| GO:2000054 | negative regulation of Wnt signaling pat... | 2 | 0 | 0.08 | 1 |
| GO:2000056 | regulation of Wnt signaling pathway invo... | 1 | 0 | 0.04 | 1 |
| GO:2000057 | negative regulation of Wnt signaling pat... | 1 | 0 | 0.04 | 1 |
| GO:2000059 | negative regulation of protein ubiquitin... | 8 | 0 | 0.3 | 1 |
| GO:2000061 | regulation of ureter smooth muscle cell ... | 1 | 0 | 0.04 | 1 |
| GO:2000062 | negative regulation of ureter smooth mus... | 1 | 0 | 0.04 | 1 |
| GO:2000063 | positive regulation of ureter smooth mus... | 1 | 0 | 0.04 | 1 |
| GO:2000064 | regulation of cortisol biosynthetic proc... | 5 | 0 | 0.19 | 1 |

| | | | | | |
|---|---|---|---|---|---|
| GO:2000065 | negative regulation of cortisol biosynth... | 4 | 0 | 0.15 | 1 |
| GO:2000066 | positive regulation of cortisol biosynth... | 1 | 0 | 0.04 | 1 |
| GO:2000067 | regulation of root morphogenesis | 1 | 0 | 0.04 | 1 |
| GO:2000077 | negative regulation of type B pancreatic... | 2 | 0 | 0.08 | 1 |
| GO:2000078 | positive regulation of type B pancreatic... | 3 | 0 | 0.11 | 1 |
| GO:2000079 | regulation of canonical Wnt signaling pa... | 2 | 0 | 0.08 | 1 |
| GO:2000080 | negative regulation of canonical Wnt sig... | 1 | 0 | 0.04 | 1 |
| GO:2000081 | positive regulation of canonical Wnt sig... | 1 | 0 | 0.04 | 1 |
| GO:2000096 | positive regulation of Wnt signaling pat... | 7 | 0 | 0.27 | 1 |
| GO:2000097 | regulation of smooth muscle cell-matrix ... | 3 | 0 | 0.11 | 1 |
| GO:2000098 | negative regulation of smooth muscle cel.. | 2 | 0 | 0.08 | 1 |
| GO:2000109 | regulation of macrophage apoptotic proce. | 7 | 0 | 0.27 | 1 |
| GO:2000110 | negative regulation of macrophage apopto | 3 | 0 | 0.11 | 1 |
| GO:2000111 | positive regulation of macrophage apopto.. | 3 | 0 | 0.11 | 1 |
| GO:2000119 | negative regulation of sodium-dependent . | 1 | 0 | 0.04 | 1 |
| GO:2000121 | regulation of removal of superoxide radi... | 5 | 0 | 0.19 | 1 |
| GO:2000124 | regulation of endocannabinoid signaling ... | 2 | 0 | 0.08 | 1 |
| GO:2000137 | negative regulation of cell proliferatio... | 1 | 0 | 0.04 | 1 |
| GO:2000138 | positive regulation of cell proliferatio... | 5 | 0 | 0.19 | 1 |
| GO:2000143 | negative regulation of DNA-templated tra.. | 4 | 0 | 0.15 | 1 |
| GO:2000152 | regulation of ubiquitin-specific proteas... | 2 | 0 | 0.08 | 1 |
| GO:2000156 | regulation of retrograde vesicle-mediate... | 2 | 0 | 0.08 | 1 |
| GO:2000157 | negative regulation of ubiquitin-specifi... | 1 | 0 | 0.04 | 1 |
| GO:2000158 | positive regulation of ubiquitin-specifi... | 1 | 0 | 0.04 | 1 |
| GO:2000169 | regulation of peptidyl-cysteine S-nitros... | 5 | 0 | 0.19 | 1 |
| GO:2000170 | positive regulation of peptidyl-cysteine... | 2 | 0 | 0.08 | 1 |
| GO:2000174 | regulation of pro-T cell differentiation | 1 | 0 | 0.04 | 1 |
| GO:2000176 | positive regulation of pro-T cell differ... | 1 | 0 | 0.04 | 1 |
| GO:2000178 | negative regulation of neural precursor ... | 20 | 0 | 0.76 | 1 |
| GO:2000180 | negative regulation of androgen biosynth.. | 1 | 0 | 0.04 | 1 |
| GO:2000188 | regulation of cholesterol homeostasis | 11 | 0 | 0.42 | 1 |
| GO:2000189 | positive regulation of cholesterol homeo... | 5 | 0 | 0.19 | 1 |
| GO:2000194 | regulation of female gonad development | 10 | 0 | 0.38 | 1 |
| GO:2000195 | negative regulation of female gonad deve.. | 3 | 0 | 0.11 | 1 |
| GO:2000196 | positive regulation of female gonad deve... | 3 | 0 | 0.11 | 1 |
| GO:2000210 | positive regulation of anoikis | 5 | 0 | 0.19 | 1 |
| GO:2000211 | regulation of glutamate metabolic proces... | 2 | 0 | 0.08 | 1 |
| GO:2000212 | negative regulation of glutamate metabol.. | 1 | 0 | 0.04 | 1 |
| GO:2000213 | positive regulation of glutamate metabol... | 1 | 0 | 0.04 | 1 |
| GO:2000224 | regulation of testosterone biosynthetic ... | 2 | 0 | 0.08 | 1 |
| GO:2000225 | negative regulation of testosterone bios... | 1 | 0 | 0.04 | 1 |
| GO:2000226 | regulation of pancreatic A cell differen... | 1 | 0 | 0.04 | 1 |
| GO:2000227 | negative regulation of pancreatic A cell... | 1 | 0 | 0.04 | 1 |
| GO:2000229 | regulation of pancreatic stellate cell p... | 2 | 0 | 0.08 | 1 |
| GO:2000230 | negative regulation of pancreatic stella... | 1 | 0 | 0.04 | 1 |
| GO:2000231 | positive regulation of pancreatic stella... | 1 | 0 | 0.04 | 1 |
| GO:2000232 | regulation of rRNA processing | 2 | 0 | 0.08 | 1 |
| GO:2000233 | negative regulation of rRNA processing | 2 | 0 | 0.08 | 1 |
| GO:2000242 | negative regulation of reproductive proc... | 35 | 0 | 1.33 | 1 |
| GO:2000243 | positive regulation of reproductive proc... | 36 | 0 | 1.37 | 1 |
| GO:2000252 | negative regulation of feeding behavior | 7 | 0 | 0.27 | 1 |
| GO:2000253 | positive regulation of feeding behavior | 3 | 0 | 0.11 | 1 |
| GO:2000254 | regulation of male germ cell proliferati... | 3 | 0 | 0.11 | 1 |

| | | | | | |
|---|---|---|---|---|---|
| GO:2000255 | negative regulation of male germ cell pr... | 1 | 0 | 0.04 | 1 |
| GO:2000256 | positive regulation of male germ cell pr... | 1 | 0 | 0.04 | 1 |
| GO:2000259 | positive regulation of protein activatio... | 2 | 0 | 0.08 | 1 |
| GO:2000260 | regulation of blood coagulation | common ... | 1 | 0 | 0.04 |
| GO:2000261 | negative regulation of blood coagulation... | 1 | 0 | 0.04 | 1 |
| GO:2000266 | regulation of blood coagulation | intrins... | 1 | 0 | 0.04 |
| GO:2000270 | negative regulation of fibroblast apopto... | 7 | 0 | 0.27 | 1 |
| GO:2000272 | negative regulation of receptor activity | 14 | 0 | 0.53 | 1 |
| GO:2000273 | positive regulation of receptor activity | 30 | 0 | 1.14 | 1 |
| GO:2000275 | regulation of oxidative phosphorylation ... | 3 | 0 | 0.11 | 1 |
| GO:2000276 | negative regulation of oxidative phospho... | 2 | 0 | 0.08 | 1 |
| GO:2000277 | positive regulation of oxidative phospho... | 1 | 0 | 0.04 | 1 |
| GO:2000279 | negative regulation of DNA biosynthetic ... | 17 | 0 | 0.65 | 1 |
| GO:2000280 | regulation of root development | 1 | 0 | 0.04 | 1 |
| GO:2000282 | regulation of cellular amino acid biosyn... | 2 | 0 | 0.08 | 1 |
| GO:2000283 | negative regulation of cellular amino ac... | 1 | 0 | 0.04 | 1 |
| GO:2000284 | positive regulation of cellular amino ac... | 1 | 0 | 0.04 | 1 |
| GO:2000286 | receptor internalization involved in can... | 3 | 0 | 0.11 | 1 |
| GO:2000287 | positive regulation of myotome developme | 1 | 0 | 0.04 | 1 |
| GO:2000288 | positive regulation of myoblast prolifer... | 5 | 0 | 0.19 | 1 |
| GO:2000290 | regulation of myotome development | 1 | 0 | 0.04 | 1 |
| GO:2000291 | regulation of myoblast proliferation | 6 | 0 | 0.23 | 1 |
| GO:2000295 | regulation of hydrogen peroxide cataboli... | 2 | 0 | 0.08 | 1 |
| GO:2000296 | negative regulation of hydrogen peroxide.. | 1 | 0 | 0.04 | 1 |
| GO:2000297 | negative regulation of synapse maturatio... | 2 | 0 | 0.08 | 1 |
| GO:2000298 | regulation of Rho-dependent protein seri... | 1 | 0 | 0.04 | 1 |
| GO:2000299 | negative regulation of Rho-dependent pro. | 1 | 0 | 0.04 | 1 |
| GO:2000300 | regulation of synaptic vesicle exocytosi... | 17 | 0 | 0.65 | 1 |
| GO:2000301 | negative regulation of synaptic vesicle ... | 3 | 0 | 0.11 | 1 |
| GO:2000302 | positive regulation of synaptic vesicle ... | 2 | 0 | 0.08 | 1 |
| GO:2000303 | regulation of ceramide biosynthetic proc... | 5 | 0 | 0.19 | 1 |
| GO:2000304 | positive regulation of ceramide biosynth... | 3 | 0 | 0.11 | 1 |
| GO:2000307 | regulation of tumor necrosis factor (lig... | 3 | 0 | 0.11 | 1 |
| GO:2000308 | negative regulation of tumor necrosis fa... | 1 | 0 | 0.04 | 1 |
| GO:2000309 | positive regulation of tumor necrosis fa... | 2 | 0 | 0.08 | 1 |
| GO:2000312 | regulation of kainate selective glutamat... | 1 | 0 | 0.04 | 1 |
| GO:2000316 | regulation of T-helper 17 type immune re... | 8 | 0 | 0.3 | 1 |
| GO:2000317 | negative regulation of T-helper 17 type ... | 2 | 0 | 0.08 | 1 |
| GO:2000318 | positive regulation of T-helper 17 type ... | 6 | 0 | 0.23 | 1 |
| GO:2000319 | regulation of T-helper 17 cell different... | 6 | 0 | 0.23 | 1 |
| GO:2000320 | negative regulation of T-helper 17 cell ... | 2 | 0 | 0.08 | 1 |
| GO:2000321 | positive regulation of T-helper 17 cell ... | 4 | 0 | 0.15 | 1 |
| GO:2000322 | regulation of glucocorticoid receptor si... | 7 | 0 | 0.27 | 1 |
| GO:2000323 | negative regulation of glucocorticoid re... | 6 | 0 | 0.23 | 1 |
| GO:2000324 | positive regulation of glucocorticoid re... | 1 | 0 | 0.04 | 1 |
| GO:2000325 | regulation of ligand-dependent nuclear r... | 3 | 0 | 0.11 | 1 |
| GO:2000326 | negative regulation of ligand-dependent ... | 1 | 0 | 0.04 | 1 |
| GO:2000327 | positive regulation of ligand-dependent ... | 2 | 0 | 0.08 | 1 |
| GO:2000328 | regulation of T-helper 17 cell lineage c... | 4 | 0 | 0.15 | 1 |
| GO:2000330 | positive regulation of T-helper 17 cell ... | 4 | 0 | 0.15 | 1 |
| GO:2000331 | regulation of terminal button organizati... | 1 | 0 | 0.04 | 1 |
| GO:2000332 | regulation of blood microparticle format... | 1 | 0 | 0.04 | 1 |
| GO:2000334 | positive regulation of blood micropartic... | 1 | 0 | 0.04 | 1 |

| | | | | | |
|---|---|---|---|---|---|
| GO:2000338 | regulation of chemokine (C-X-C motif) li... | 1 | 0 | 0.04 | 1 |
| GO:2000340 | positive regulation of chemokine (C-X-C ... | 1 | 0 | 0.04 | 1 |
| GO:2000341 | regulation of chemokine (C-X-C motif) li... | 6 | 0 | 0.23 | 1 |
| GO:2000342 | negative regulation of chemokine (C-X-C .. | 2 | 0 | 0.08 | 1 |
| GO:2000343 | positive regulation of chemokine (C-X-C ... | 4 | 0 | 0.15 | 1 |
| GO:2000344 | positive regulation of acrosome reaction | 6 | 0 | 0.23 | 1 |
| GO:2000345 | regulation of hepatocyte proliferation | 8 | 0 | 0.3 | 1 |
| GO:2000346 | negative regulation of hepatocyte prolif... | 3 | 0 | 0.11 | 1 |
| GO:2000347 | positive regulation of hepatocyte prolif... | 4 | 0 | 0.15 | 1 |
| GO:2000348 | regulation of CD40 signaling pathway | 3 | 0 | 0.11 | 1 |
| GO:2000349 | negative regulation of CD40 signaling pa... | 1 | 0 | 0.04 | 1 |
| GO:2000350 | positive regulation of CD40 signaling pa... | 1 | 0 | 0.04 | 1 |
| GO:2000351 | regulation of endothelial cell apoptotic... | 34 | 0 | 1.29 | 1 |
| GO:2000352 | negative regulation of endothelial cell ... | 24 | 0 | 0.91 | 1 |
| GO:2000353 | positive regulation of endothelial cell ... | 10 | 0 | 0.38 | 1 |
| GO:2000354 | regulation of ovarian follicle developme... | 3 | 0 | 0.11 | 1 |
| GO:2000356 | regulation of kidney smooth muscle cell ... | 1 | 0 | 0.04 | 1 |
| GO:2000357 | negative regulation of kidney smooth mus. | 1 | 0 | 0.04 | 1 |
| GO:2000358 | positive regulation of kidney smooth mus... | 1 | 0 | 0.04 | 1 |
| GO:2000359 | regulation of binding of sperm to zona p... | 4 | 0 | 0.15 | 1 |
| GO:2000360 | negative regulation of binding of sperm ... | 4 | 0 | 0.15 | 1 |
| GO:2000361 | regulation of prostaglandin-E synthase a... | 1 | 0 | 0.04 | 1 |
| GO:2000363 | positive regulation of prostaglandin-E s... | 1 | 0 | 0.04 | 1 |
| GO:2000367 | regulation of acrosomal vesicle exocytos... | 1 | 0 | 0.04 | 1 |
| GO:2000368 | positive regulation of acrosomal vesicle... | 1 | 0 | 0.04 | 1 |
| GO:2000369 | regulation of clathrin-mediated endocyto... | 10 | 0 | 0.38 | 1 |
| GO:2000370 | positive regulation of clathrin-mediated... | 3 | 0 | 0.11 | 1 |
| GO:2000371 | regulation of DNA topoisomerase (ATP-hy( | 3 | 0 | 0.11 | 1 |
| GO:2000373 | positive regulation of DNA topoisomerase.. | 3 | 0 | 0.11 | 1 |
| GO:2000374 | regulation of oxygen metabolic process | 2 | 0 | 0.08 | 1 |
| GO:2000376 | positive regulation of oxygen metabolic ... | 1 | 0 | 0.04 | 1 |
| GO:2000380 | regulation of mesoderm development | 15 | 0 | 0.57 | 1 |
| GO:2000381 | negative regulation of mesoderm developr | 7 | 0 | 0.27 | 1 |
| GO:2000382 | positive regulation of mesoderm developm | 5 | 0 | 0.19 | 1 |
| GO:2000383 | regulation of ectoderm development | 1 | 0 | 0.04 | 1 |
| GO:2000384 | negative regulation of ectoderm developm. | 1 | 0 | 0.04 | 1 |
| GO:2000386 | positive regulation of ovarian follicle ... | 1 | 0 | 0.04 | 1 |
| GO:2000387 | regulation of antral ovarian follicle gr... | 1 | 0 | 0.04 | 1 |
| GO:2000388 | positive regulation of antral ovarian fo... | 1 | 0 | 0.04 | 1 |
| GO:2000389 | regulation of neutrophil extravasation | 1 | 0 | 0.04 | 1 |
| GO:2000391 | positive regulation of neutrophil extrav... | 1 | 0 | 0.04 | 1 |
| GO:2000392 | regulation of lamellipodium morphogenesi. | 5 | 0 | 0.19 | 1 |
| GO:2000393 | negative regulation of lamellipodium mor... | 1 | 0 | 0.04 | 1 |
| GO:2000394 | positive regulation of lamellipodium mor... | 3 | 0 | 0.11 | 1 |
| GO:2000395 | regulation of ubiquitin-dependent endocy... | 1 | 0 | 0.04 | 1 |
| GO:2000397 | positive regulation of ubiquitin-depende... | 1 | 0 | 0.04 | 1 |
| GO:2000398 | regulation of thymocyte aggregation | 7 | 0 | 0.27 | 1 |
| GO:2000399 | negative regulation of thymocyte aggrega.. | 4 | 0 | 0.15 | 1 |
| GO:2000400 | positive regulation of thymocyte aggrega... | 3 | 0 | 0.11 | 1 |
| GO:2000403 | positive regulation of lymphocyte migrat... | 22 | 0 | 0.84 | 1 |
| GO:2000404 | regulation of T cell migration | 22 | 0 | 0.84 | 1 |
| GO:2000405 | negative regulation of T cell migration | 1 | 0 | 0.04 | 1 |
| GO:2000406 | positive regulation of T cell migration | 19 | 0 | 0.72 | 1 |

| | | | | | |
|---|---|---|---|---|---|
| GO:2000407 | regulation of T cell extravasation | 3 | 0 | 0.11 | 1 |
| GO:2000409 | positive regulation of T cell extravasat... | 2 | 0 | 0.08 | 1 |
| GO:2000410 | regulation of thymocyte migration | 2 | 0 | 0.08 | 1 |
| GO:2000412 | positive regulation of thymocyte migrati... | 2 | 0 | 0.08 | 1 |
| GO:2000413 | regulation of fibronectin-dependent thym... | 1 | 0 | 0.04 | 1 |
| GO:2000415 | positive regulation of fibronectin-depen... | 1 | 0 | 0.04 | 1 |
| GO:2000416 | regulation of eosinophil migration | 5 | 0 | 0.19 | 1 |
| GO:2000417 | negative regulation of eosinophil migrat... | 2 | 0 | 0.08 | 1 |
| GO:2000418 | positive regulation of eosinophil migrat... | 3 | 0 | 0.11 | 1 |
| GO:2000419 | regulation of eosinophil extravasation | 1 | 0 | 0.04 | 1 |
| GO:2000420 | negative regulation of eosinophil extrav... | 1 | 0 | 0.04 | 1 |
| GO:2000422 | regulation of eosinophil chemotaxis | 1 | 0 | 0.04 | 1 |
| GO:2000424 | positive regulation of eosinophil chemot... | 1 | 0 | 0.04 | 1 |
| GO:2000426 | negative regulation of apoptotic cell cl... | 1 | 0 | 0.04 | 1 |
| GO:2000437 | regulation of monocyte extravasation | 2 | 0 | 0.08 | 1 |
| GO:2000438 | negative regulation of monocyte extravas.. | 1 | 0 | 0.04 | 1 |
| GO:2000439 | positive regulation of monocyte extravas... | 1 | 0 | 0.04 | 1 |
| GO:2000446 | regulation of macrophage migration inhib.. | 1 | 0 | 0.04 | 1 |
| GO:2000449 | regulation of CD8-positive | alpha-beta | 3 | 0 | 0.11 |
| GO:2000451 | positive regulation of CD8-positive | alp... | 2 | 0 | 0.08 |
| GO:2000452 | regulation of CD8-positive | alpha-beta | 2 | 0 | 0.08 |
| GO:2000454 | positive regulation of CD8-positive | alp... | 1 | 0 | 0.04 |
| GO:2000458 | regulation of astrocyte chemotaxis | 1 | 0 | 0.04 | 1 |
| GO:2000464 | positive regulation of astrocyte chemota... | 1 | 0 | 0.04 | 1 |
| GO:2000465 | regulation of glycogen (starch) synthase... | 7 | 0 | 0.27 | 1 |
| GO:2000466 | negative regulation of glycogen (starch)... | 3 | 0 | 0.11 | 1 |
| GO:2000467 | positive regulation of glycogen (starch)... | 4 | 0 | 0.15 | 1 |
| GO:2000468 | regulation of peroxidase activity | 2 | 0 | 0.08 | 1 |
| GO:2000469 | negative regulation of peroxidase activi... | 1 | 0 | 0.04 | 1 |
| GO:2000470 | positive regulation of peroxidase activi... | 1 | 0 | 0.04 | 1 |
| GO:2000471 | regulation of hematopoietic stem cell mi... | 2 | 0 | 0.08 | 1 |
| GO:2000473 | positive regulation of hematopoietic ste... | 2 | 0 | 0.08 | 1 |
| GO:2000474 | regulation of opioid receptor signaling ... | 2 | 0 | 0.08 | 1 |
| GO:2000477 | regulation of metanephric glomerular vis... | 1 | 0 | 0.04 | 1 |
| GO:2000478 | positive regulation of metanephric glome... | 1 | 0 | 0.04 | 1 |
| GO:2000479 | regulation of cAMP-dependent protein kin. | 13 | 0 | 0.49 | 1 |
| GO:2000480 | negative regulation of cAMP-dependent pr | 8 | 0 | 0.3 | 1 |
| GO:2000481 | positive regulation of cAMP-dependent pr.. | 4 | 0 | 0.15 | 1 |
| GO:2000482 | regulation of interleukin-8 secretion | 13 | 0 | 0.49 | 1 |
| GO:2000483 | negative regulation of interleukin-8 sec... | 2 | 0 | 0.08 | 1 |
| GO:2000484 | positive regulation of interleukin-8 sec... | 10 | 0 | 0.38 | 1 |
| GO:2000492 | regulation of interleukin-18-mediated si... | 1 | 0 | 0.04 | 1 |
| GO:2000494 | positive regulation of interleukin-18-me... | 1 | 0 | 0.04 | 1 |
| GO:2000503 | positive regulation of natural killer ce... | 4 | 0 | 0.15 | 1 |
| GO:2000504 | positive regulation of blood vessel remo... | 2 | 0 | 0.08 | 1 |
| GO:2000506 | negative regulation of energy homeostasi.. | 1 | 0 | 0.04 | 1 |
| GO:2000507 | positive regulation of energy homeostasi... | 3 | 0 | 0.11 | 1 |
| GO:2000508 | regulation of dendritic cell chemotaxis | 5 | 0 | 0.19 | 1 |
| GO:2000510 | positive regulation of dendritic cell ch... | 5 | 0 | 0.19 | 1 |
| GO:2000511 | regulation of granzyme A production | 1 | 0 | 0.04 | 1 |
| GO:2000513 | positive regulation of granzyme A produc... | 1 | 0 | 0.04 | 1 |
| GO:2000514 | regulation of CD4-positive | alpha-beta | 30 | 0 | 1.14 |
| GO:2000515 | negative regulation of CD4-positive | alp... | 10 | 0 | 0.38 |

| | | | | | |
|---|---|---|---|---|---|
| GO:2000516 | positive regulation of CD4-positive | alp... | 23 | 0 | 0.88 |
| GO:2000517 | regulation of T-helper 1 cell activation | 1 | 0 | 0.04 | 1 |
| GO:2000518 | negative regulation of T-helper 1 cell a... | 1 | 0 | 0.04 | 1 |
| GO:2000520 | regulation of immunological synapse form. | 2 | 0 | 0.08 | 1 |
| GO:2000521 | negative regulation of immunological syn... | 1 | 0 | 0.04 | 1 |
| GO:2000522 | positive regulation of immunological syn... | 1 | 0 | 0.04 | 1 |
| GO:2000523 | regulation of T cell costimulation | 2 | 0 | 0.08 | 1 |
| GO:2000525 | positive regulation of T cell costimulat... | 2 | 0 | 0.08 | 1 |
| GO:2000526 | positive regulation of glycoprotein bios... | 1 | 0 | 0.04 | 1 |
| GO:2000527 | regulation of myeloid dendritic cell che... | 1 | 0 | 0.04 | 1 |
| GO:2000529 | positive regulation of myeloid dendritic... | 1 | 0 | 0.04 | 1 |
| GO:2000532 | regulation of renal albumin absorption | 2 | 0 | 0.08 | 1 |
| GO:2000533 | negative regulation of renal albumin abs... | 1 | 0 | 0.04 | 1 |
| GO:2000534 | positive regulation of renal albumin abs... | 1 | 0 | 0.04 | 1 |
| GO:2000535 | regulation of entry of bacterium into ho... | 2 | 0 | 0.08 | 1 |
| GO:2000536 | negative regulation of entry of bacteriu... | 1 | 0 | 0.04 | 1 |
| GO:2000537 | regulation of B cell chemotaxis | 2 | 0 | 0.08 | 1 |
| GO:2000538 | positive regulation of B cell chemotaxis | 2 | 0 | 0.08 | 1 |
| GO:2000539 | regulation of protein geranylgeranylatio... | 1 | 0 | 0.04 | 1 |
| GO:2000541 | positive regulation of protein geranylge... | 1 | 0 | 0.04 | 1 |
| GO:2000542 | negative regulation of gastrulation | 7 | 0 | 0.27 | 1 |
| GO:2000543 | positive regulation of gastrulation | 7 | 0 | 0.27 | 1 |
| GO:2000544 | regulation of endothelial cell chemotaxi... | 6 | 0 | 0.23 | 1 |
| GO:2000545 | negative regulation of endothelial cell ... | 1 | 0 | 0.04 | 1 |
| GO:2000546 | positive regulation of endothelial cell ... | 3 | 0 | 0.11 | 1 |
| GO:2000547 | regulation of dendritic cell dendrite as... | 3 | 0 | 0.11 | 1 |
| GO:2000548 | negative regulation of dendritic cell de... | 1 | 0 | 0.04 | 1 |
| GO:2000549 | positive regulation of dendritic cell de... | 1 | 0 | 0.04 | 1 |
| GO:2000551 | regulation of T-helper 2 cell cytokine p... | 5 | 0 | 0.19 | 1 |
| GO:2000552 | negative regulation of T-helper 2 cell c... | 2 | 0 | 0.08 | 1 |
| GO:2000553 | positive regulation of T-helper 2 cell c... | 3 | 0 | 0.11 | 1 |
| GO:2000554 | regulation of T-helper 1 cell cytokine p... | 1 | 0 | 0.04 | 1 |
| GO:2000556 | positive regulation of T-helper 1 cell c... | 1 | 0 | 0.04 | 1 |
| GO:2000557 | regulation of immunoglobulin production ... | 1 | 0 | 0.04 | 1 |
| GO:2000558 | positive regulation of immunoglobulin pr... | 1 | 0 | 0.04 | 1 |
| GO:2000559 | regulation of CD24 biosynthetic process | 1 | 0 | 0.04 | 1 |
| GO:2000560 | positive regulation of CD24 biosynthetic... | 1 | 0 | 0.04 | 1 |
| GO:2000561 | regulation of CD4-positive | alpha-beta | 2 | 0 | 0.08 |
| GO:2000562 | negative regulation of CD4-positive | alp... | 1 | 0 | 0.04 |
| GO:2000563 | positive regulation of CD4-positive | alp... | 2 | 0 | 0.08 |
| GO:2000564 | regulation of CD8-positive | alpha-beta | 4 | 0 | 0.15 |
| GO:2000566 | positive regulation of CD8-positive | alp... | 3 | 0 | 0.11 |
| GO:2000567 | regulation of memory T cell activation | 2 | 0 | 0.08 | 1 |
| GO:2000568 | positive regulation of memory T cell act... | 2 | 0 | 0.08 | 1 |
| GO:2000569 | regulation of T-helper 2 cell activation | 2 | 0 | 0.08 | 1 |
| GO:2000570 | positive regulation of T-helper 2 cell a... | 2 | 0 | 0.08 | 1 |
| GO:2000571 | regulation of interleukin-4-dependent is... | 1 | 0 | 0.04 | 1 |
| GO:2000572 | positive regulation of interleukin-4-dep... | 1 | 0 | 0.04 | 1 |
| GO:2000583 | regulation of platelet-derived growth fa... | 2 | 0 | 0.08 | 1 |
| GO:2000584 | negative regulation of platelet-derived ... | 2 | 0 | 0.08 | 1 |
| GO:2000589 | regulation of metanephric mesenchymal ce | 4 | 0 | 0.15 | 1 |
| GO:2000590 | negative regulation of metanephric mesen. | 1 | 0 | 0.04 | 1 |
| GO:2000591 | positive regulation of metanephric mesen.. | 3 | 0 | 0.11 | 1 |

| | | | | | |
|---|---|---|---|---|---|
| GO:2000592 | regulation of metanephric DCT cell diffe... | 2 | 0 | 0.08 | 1 |
| GO:2000594 | positive regulation of metanephric DCT c... | 2 | 0 | 0.08 | 1 |
| GO:2000595 | regulation of optic nerve formation | 1 | 0 | 0.04 | 1 |
| GO:2000597 | positive regulation of optic nerve forma... | 1 | 0 | 0.04 | 1 |
| GO:2000601 | positive regulation of Arp2/3 complex-me... | 6 | 0 | 0.23 | 1 |
| GO:2000606 | regulation of cell proliferation involve... | 1 | 0 | 0.04 | 1 |
| GO:2000607 | negative regulation of cell proliferatio... | 1 | 0 | 0.04 | 1 |
| GO:2000609 | regulation of thyroid hormone generation | 5 | 0 | 0.19 | 1 |
| GO:2000611 | positive regulation of thyroid hormone g... | 3 | 0 | 0.11 | 1 |
| GO:2000612 | regulation of thyroid-stimulating hormon... | 1 | 0 | 0.04 | 1 |
| GO:2000615 | regulation of histone H3-K9 acetylation | 3 | 0 | 0.11 | 1 |
| GO:2000617 | positive regulation of histone H3-K9 ace... | 2 | 0 | 0.08 | 1 |
| GO:2000618 | regulation of histone H4-K16 acetylation | 4 | 0 | 0.15 | 1 |
| GO:2000619 | negative regulation of histone H4-K16 ac... | 3 | 0 | 0.11 | 1 |
| GO:2000620 | positive regulation of histone H4-K16 ac... | 1 | 0 | 0.04 | 1 |
| GO:2000622 | regulation of nuclear-transcribed mRNA c... | 4 | 0 | 0.15 | 1 |
| GO:2000623 | negative regulation of nuclear-transcrib... | 4 | 0 | 0.15 | 1 |
| GO:2000637 | positive regulation of gene silencing by... | 3 | 0 | 0.11 | 1 |
| GO:2000638 | regulation of SREBP signaling pathway | 1 | 0 | 0.04 | 1 |
| GO:2000639 | negative regulation of SREBP signaling p... | 1 | 0 | 0.04 | 1 |
| GO:2000642 | negative regulation of early endosome to... | 2 | 0 | 0.08 | 1 |
| GO:2000644 | regulation of receptor catabolic process | 7 | 0 | 0.27 | 1 |
| GO:2000645 | negative regulation of receptor cataboli... | 3 | 0 | 0.11 | 1 |
| GO:2000646 | positive regulation of receptor cataboli... | 3 | 0 | 0.11 | 1 |
| GO:2000647 | negative regulation of stem cell prolife... | 14 | 0 | 0.53 | 1 |
| GO:2000650 | negative regulation of sodium ion transm... | 7 | 0 | 0.27 | 1 |
| GO:2000653 | regulation of genetic imprinting | 4 | 0 | 0.15 | 1 |
| GO:2000654 | regulation of cellular response to testo... | 1 | 0 | 0.04 | 1 |
| GO:2000655 | negative regulation of cellular response... | 1 | 0 | 0.04 | 1 |
| GO:2000656 | regulation of apolipoprotein binding | 1 | 0 | 0.04 | 1 |
| GO:2000657 | negative regulation of apolipoprotein bi... | 1 | 0 | 0.04 | 1 |
| GO:2000659 | regulation of interleukin-1-mediated sig... | 3 | 0 | 0.11 | 1 |
| GO:2000660 | negative regulation of interleukin-1-med... | 2 | 0 | 0.08 | 1 |
| GO:2000664 | positive regulation of interleukin-5 sec... | 2 | 0 | 0.08 | 1 |
| GO:2000667 | positive regulation of interleukin-13 se... | 3 | 0 | 0.11 | 1 |
| GO:2000668 | regulation of dendritic cell apoptotic p... | 6 | 0 | 0.23 | 1 |
| GO:2000669 | negative regulation of dendritic cell ap... | 3 | 0 | 0.11 | 1 |
| GO:2000670 | positive regulation of dendritic cell ap... | 3 | 0 | 0.11 | 1 |
| GO:2000671 | regulation of motor neuron apoptotic pro... | 1 | 0 | 0.04 | 1 |
| GO:2000672 | negative regulation of motor neuron apop... | 1 | 0 | 0.04 | 1 |
| GO:2000674 | regulation of type B pancreatic cell apo... | 9 | 0 | 0.34 | 1 |
| GO:2000675 | negative regulation of type B pancreatic... | 6 | 0 | 0.23 | 1 |
| GO:2000676 | positive regulation of type B pancreatic... | 3 | 0 | 0.11 | 1 |
| GO:2000679 | positive regulation of transcription reg... | 9 | 0 | 0.34 | 1 |
| GO:2000680 | regulation of rubidium ion transport | 5 | 0 | 0.19 | 1 |
| GO:2000681 | negative regulation of rubidium ion tran... | 3 | 0 | 0.11 | 1 |
| GO:2000682 | positive regulation of rubidium ion tran... | 1 | 0 | 0.04 | 1 |
| GO:2000683 | regulation of cellular response to X-ray | 2 | 0 | 0.08 | 1 |
| GO:2000685 | positive regulation of cellular response... | 1 | 0 | 0.04 | 1 |
| GO:2000686 | regulation of rubidium ion transmembrane... | 4 | 0 | 0.15 | 1 |
| GO:2000687 | negative regulation of rubidium ion tran... | 3 | 0 | 0.11 | 1 |
| GO:2000688 | positive regulation of rubidium ion tran... | 1 | 0 | 0.04 | 1 |
| GO:2000690 | regulation of cardiac muscle cell myobla... | 1 | 0 | 0.04 | 1 |

| | | | | | |
|---|---|---|---|---|---|
| GO:2000691 | negative regulation of cardiac muscle ce... | 1 | 0 | 0.04 | 1 |
| GO:2000696 | regulation of epithelial cell differenti... | 16 | 0 | 0.61 | 1 |
| GO:2000697 | negative regulation of epithelial cell d... | 3 | 0 | 0.11 | 1 |
| GO:2000698 | positive regulation of epithelial cell d... | 4 | 0 | 0.15 | 1 |
| GO:2000699 | fibroblast growth factor receptor signal... | 1 | 0 | 0.04 | 1 |
| GO:2000701 | glial cell-derived neurotrophic factor r... | 1 | 0 | 0.04 | 1 |
| GO:2000702 | regulation of fibroblast growth factor r... | 1 | 0 | 0.04 | 1 |
| GO:2000703 | negative regulation of fibroblast growth... | 1 | 0 | 0.04 | 1 |
| GO:2000705 | regulation of dense core granule biogene... | 3 | 0 | 0.11 | 1 |
| GO:2000706 | negative regulation of dense core granul... | 1 | 0 | 0.04 | 1 |
| GO:2000707 | positive regulation of dense core granul... | 2 | 0 | 0.08 | 1 |
| GO:2000721 | positive regulation of transcription fro... | 3 | 0 | 0.11 | 1 |
| GO:2000722 | regulation of cardiac vascular smooth mu... | 2 | 0 | 0.08 | 1 |
| GO:2000723 | negative regulation of cardiac vascular ... | 1 | 0 | 0.04 | 1 |
| GO:2000724 | positive regulation of cardiac vascular ... | 1 | 0 | 0.04 | 1 |
| GO:2000726 | negative regulation of cardiac muscle ce... | 6 | 0 | 0.23 | 1 |
| GO:2000729 | positive regulation of mesenchymal cell ... | 3 | 0 | 0.11 | 1 |
| GO:2000733 | regulation of glial cell-derived neurotr... | 1 | 0 | 0.04 | 1 |
| GO:2000734 | negative regulation of glial cell-derive... | 1 | 0 | 0.04 | 1 |
| GO:2000739 | regulation of mesenchymal stem cell diff... | 6 | 0 | 0.23 | 1 |
| GO:2000740 | negative regulation of mesenchymal stem | 1 | 0 | 0.04 | 1 |
| GO:2000741 | positive regulation of mesenchymal stem ... | 4 | 0 | 0.15 | 1 |
| GO:2000742 | regulation of anterior head development | 2 | 0 | 0.08 | 1 |
| GO:2000744 | positive regulation of anterior head dev... | 2 | 0 | 0.08 | 1 |
| GO:2000751 | histone H3-T3 phosphorylation involved i... | 1 | 0 | 0.04 | 1 |
| GO:2000752 | regulation of glucosylceramide catabolic... | 1 | 0 | 0.04 | 1 |
| GO:2000753 | positive regulation of glucosylceramide ... | 1 | 0 | 0.04 | 1 |
| GO:2000754 | regulation of sphingomyelin catabolic pr... | 1 | 0 | 0.04 | 1 |
| GO:2000755 | positive regulation of sphingomyelin cat... | 1 | 0 | 0.04 | 1 |
| GO:2000756 | regulation of peptidyl-lysine acetylatio... | 42 | 0 | 1.6 | 1 |
| GO:2000757 | negative regulation of peptidyl-lysine a... | 15 | 0 | 0.57 | 1 |
| GO:2000758 | positive regulation of peptidyl-lysine a... | 21 | 0 | 0.8 | 1 |
| GO:2000759 | regulation of N-terminal peptidyl-lysine... | 1 | 0 | 0.04 | 1 |
| GO:2000761 | positive regulation of N-terminal peptid... | 1 | 0 | 0.04 | 1 |
| GO:2000763 | positive regulation of transcription fro... | 1 | 0 | 0.04 | 1 |
| GO:2000764 | positive regulation of semaphorin-plexin... | 1 | 0 | 0.04 | 1 |
| GO:2000765 | regulation of cytoplasmic translation | 5 | 0 | 0.19 | 1 |
| GO:2000766 | negative regulation of cytoplasmic trans... | 3 | 0 | 0.11 | 1 |
| GO:2000767 | positive regulation of cytoplasmic trans... | 1 | 0 | 0.04 | 1 |
| GO:2000768 | positive regulation of nephron tubule ep... | 3 | 0 | 0.11 | 1 |
| GO:2000774 | positive regulation of cellular senescen... | 4 | 0 | 0.15 | 1 |
| GO:2000775 | histone H3-S10 phosphorylation involved ... | 1 | 0 | 0.04 | 1 |
| GO:2000777 | positive regulation of proteasomal ubiqu... | 2 | 0 | 0.08 | 1 |
| GO:2000778 | positive regulation of interleukin-6 sec... | 8 | 0 | 0.3 | 1 |
| GO:2000780 | negative regulation of double-strand bre... | 6 | 0 | 0.23 | 1 |
| GO:2000781 | positive regulation of double-strand bre... | 3 | 0 | 0.11 | 1 |
| GO:2000790 | regulation of mesenchymal cell prolifera... | 2 | 0 | 0.08 | 1 |
| GO:2000791 | negative regulation of mesenchymal cell ... | 2 | 0 | 0.08 | 1 |
| GO:2000793 | cell proliferation involved in heart val... | 2 | 0 | 0.08 | 1 |
| GO:2000794 | regulation of epithelial cell proliferat... | 6 | 0 | 0.23 | 1 |
| GO:2000795 | negative regulation of epithelial cell p... | 1 | 0 | 0.04 | 1 |
| GO:2000797 | regulation of amniotic stem cell differe... | 1 | 0 | 0.04 | 1 |
| GO:2000798 | negative regulation of amniotic stem cel... | 1 | 0 | 0.04 | 1 |

| | | | | | |
|---|---|---|---|---|---|
| GO:2000800 | regulation of endocardial cushion to mes... | 1 | 0 | 0.04 | 1 |
| GO:2000802 | positive regulation of endocardial cushi... | 1 | 0 | 0.04 | 1 |
| GO:2000804 | regulation of termination of RNA polymer... | 1 | 0 | 0.04 | 1 |
| GO:2000806 | positive regulation of termination of RN... | 1 | 0 | 0.04 | 1 |
| GO:2000810 | regulation of tight junction assembly | 9 | 0 | 0.34 | 1 |
| GO:2000818 | negative regulation of myoblast prolifer... | 1 | 0 | 0.04 | 1 |
| GO:2000819 | regulation of nucleotide-excision repair | 2 | 0 | 0.08 | 1 |
| GO:2000822 | regulation of behavioral fear response | 4 | 0 | 0.15 | 1 |
| GO:2000823 | regulation of androgen receptor activity | 3 | 0 | 0.11 | 1 |
| GO:2000824 | negative regulation of androgen receptor... | 2 | 0 | 0.08 | 1 |
| GO:2000825 | positive regulation of androgen receptor... | 1 | 0 | 0.04 | 1 |
| GO:2000827 | mitochondrial RNA surveillance | 1 | 0 | 0.04 | 1 |
| GO:2000832 | negative regulation of steroid hormone s... | 2 | 0 | 0.08 | 1 |
| GO:2000834 | regulation of androgen secretion | 2 | 0 | 0.08 | 1 |
| GO:2000836 | positive regulation of androgen secretio... | 2 | 0 | 0.08 | 1 |
| GO:2000843 | regulation of testosterone secretion | 1 | 0 | 0.04 | 1 |
| GO:2000845 | positive regulation of testosterone secr... | 1 | 0 | 0.04 | 1 |
| GO:2000847 | negative regulation of corticosteroid ho... | 2 | 0 | 0.08 | 1 |
| GO:2000849 | regulation of glucocorticoid secretion | 9 | 0 | 0.34 | 1 |
| GO:2000850 | negative regulation of glucocorticoid se... | 2 | 0 | 0.08 | 1 |
| GO:2000851 | positive regulation of glucocorticoid se... | 5 | 0 | 0.19 | 1 |
| GO:2000852 | regulation of corticosterone secretion | 4 | 0 | 0.15 | 1 |
| GO:2000854 | positive regulation of corticosterone se... | 2 | 0 | 0.08 | 1 |
| GO:2000870 | regulation of progesterone secretion | 3 | 0 | 0.11 | 1 |
| GO:2000872 | positive regulation of progesterone secr... | 2 | 0 | 0.08 | 1 |
| GO:2000878 | positive regulation of oligopeptide tran... | 2 | 0 | 0.08 | 1 |
| GO:2000880 | positive regulation of dipeptide transpo... | 2 | 0 | 0.08 | 1 |
| GO:2000896 | amylopectin metabolic process | 1 | 0 | 0.04 | 1 |
| GO:2000909 | regulation of sterol import | 2 | 0 | 0.08 | 1 |
| GO:2000910 | negative regulation of sterol import | 2 | 0 | 0.08 | 1 |
| GO:2000969 | positive regulation of alpha-amino-3-hyd... | 3 | 0 | 0.11 | 1 |
| GO:2000970 | regulation of detection of glucose | 1 | 0 | 0.04 | 1 |
| GO:2000971 | negative regulation of detection of gluc... | 1 | 0 | 0.04 | 1 |
| GO:2000973 | regulation of pro-B cell differentiation | 5 | 0 | 0.19 | 1 |
| GO:2000974 | negative regulation of pro-B cell differ... | 3 | 0 | 0.11 | 1 |
| GO:2000977 | regulation of forebrain neuron different... | 4 | 0 | 0.15 | 1 |
| GO:2000978 | negative regulation of forebrain neuron ... | 2 | 0 | 0.08 | 1 |
| GO:2000979 | positive regulation of forebrain neuron ... | 1 | 0 | 0.04 | 1 |
| GO:2000980 | regulation of inner ear receptor cell di... | 5 | 0 | 0.19 | 1 |
| GO:2000981 | negative regulation of inner ear recepto... | 3 | 0 | 0.11 | 1 |
| GO:2000982 | positive regulation of inner ear recepto... | 1 | 0 | 0.04 | 1 |
| GO:2000983 | regulation of ATP citrate synthase activ... | 3 | 0 | 0.11 | 1 |
| GO:2000984 | negative regulation of ATP citrate synth... | 3 | 0 | 0.11 | 1 |
| GO:2000987 | positive regulation of behavioral fear r... | 4 | 0 | 0.15 | 1 |
| GO:2001012 | mesenchymal cell differentiation involve... | 7 | 0 | 0.27 | 1 |
| GO:2001013 | epithelial cell proliferation involved i... | 2 | 0 | 0.08 | 1 |
| GO:2001014 | regulation of skeletal muscle cell diffe... | 13 | 0 | 0.49 | 1 |
| GO:2001015 | negative regulation of skeletal muscle c... | 4 | 0 | 0.15 | 1 |
| GO:2001016 | positive regulation of skeletal muscle c... | 5 | 0 | 0.19 | 1 |
| GO:2001027 | negative regulation of endothelial cell ... | 4 | 0 | 0.15 | 1 |
| GO:2001029 | regulation of cellular glucuronidation | 9 | 0 | 0.34 | 1 |
| GO:2001030 | negative regulation of cellular glucuron... | 8 | 0 | 0.3 | 1 |
| GO:2001031 | positive regulation of cellular glucuron... | 1 | 0 | 0.04 | 1 |

| | | | | | |
|---|---|---|---|---|---|
| GO:2001032 | regulation of double-strand break repair... | 4 | 0 | 0.15 | 1 |
| GO:2001033 | negative regulation of double-strand bre... | 1 | 0 | 0.04 | 1 |
| GO:2001034 | positive regulation of double-strand bre... | 1 | 0 | 0.04 | 1 |
| GO:2001035 | regulation of tongue muscle cell differe... | 2 | 0 | 0.08 | 1 |
| GO:2001037 | positive regulation of tongue muscle cel... | 2 | 0 | 0.08 | 1 |
| GO:2001045 | negative regulation of integrin-mediated... | 2 | 0 | 0.08 | 1 |
| GO:2001046 | positive regulation of integrin-mediated... | 6 | 0 | 0.23 | 1 |
| GO:2001049 | regulation of tendon cell differentiatio... | 1 | 0 | 0.04 | 1 |
| GO:2001051 | positive regulation of tendon cell diffe... | 1 | 0 | 0.04 | 1 |
| GO:2001053 | regulation of mesenchymal cell apoptotic... | 13 | 0 | 0.49 | 1 |
| GO:2001054 | negative regulation of mesenchymal cell ... | 10 | 0 | 0.38 | 1 |
| GO:2001055 | positive regulation of mesenchymal cell ... | 3 | 0 | 0.11 | 1 |
| GO:2001074 | regulation of metanephric ureteric bud d... | 2 | 0 | 0.08 | 1 |
| GO:2001076 | positive regulation of metanephric urete... | 2 | 0 | 0.08 | 1 |
| GO:2001106 | regulation of Rho guanyl-nucleotide exch... | 1 | 0 | 0.04 | 1 |
| GO:2001108 | positive regulation of Rho guanyl-nucleo... | 1 | 0 | 0.04 | 1 |
| GO:2001109 | regulation of lens epithelial cell proli... | 1 | 0 | 0.04 | 1 |
| GO:2001111 | positive regulation of lens epithelial c... | 1 | 0 | 0.04 | 1 |
| GO:2001112 | regulation of cellular response to hepat... | 1 | 0 | 0.04 | 1 |
| GO:2001113 | negative regulation of cellular response... | 1 | 0 | 0.04 | 1 |
| GO:2001135 | regulation of endocytic recycling | 4 | 0 | 0.15 | 1 |
| GO:2001137 | positive regulation of endocytic recycli... | 3 | 0 | 0.11 | 1 |
| GO:2001139 | negative regulation of phospholipid tran... | 1 | 0 | 0.04 | 1 |
| GO:2001142 | nicotinate transport | 1 | 0 | 0.04 | 1 |
| GO:2001144 | regulation of phosphatidylinositol-3 | 4 | 5... | 1 | 0 |
| GO:2001145 | negative regulation of phosphatidylinosi... | 1 | 0 | 0.04 | 1 |
| GO:2001148 | regulation of dipeptide transmembrane tr... | 2 | 0 | 0.08 | 1 |
| GO:2001150 | positive regulation of dipeptide transme... | 2 | 0 | 0.08 | 1 |
| GO:2001151 | regulation of renal water transport | 1 | 0 | 0.04 | 1 |
| GO:2001153 | positive regulation of renal water trans... | 1 | 0 | 0.04 | 1 |
| GO:2001160 | regulation of histone H3-K79 methylation | 2 | 0 | 0.08 | 1 |
| GO:2001162 | positive regulation of histone H3-K79 me... | 2 | 0 | 0.08 | 1 |
| GO:2001163 | regulation of phosphorylation of RNA pol... | 1 | 0 | 0.04 | 1 |
| GO:2001165 | positive regulation of phosphorylation o... | 1 | 0 | 0.04 | 1 |
| GO:2001166 | regulation of histone H2B ubiquitination | 4 | 0 | 0.15 | 1 |
| GO:2001168 | positive regulation of histone H2B ubiqu... | 4 | 0 | 0.15 | 1 |
| GO:2001169 | regulation of ATP biosynthetic process | 4 | 0 | 0.15 | 1 |
| GO:2001170 | negative regulation of ATP biosynthetic ... | 2 | 0 | 0.08 | 1 |
| GO:2001171 | positive regulation of ATP biosynthetic ... | 3 | 0 | 0.11 | 1 |
| GO:2001176 | regulation of mediator complex assembly | 1 | 0 | 0.04 | 1 |
| GO:2001178 | positive regulation of mediator complex ... | 1 | 0 | 0.04 | 1 |
| GO:2001181 | positive regulation of interleukin-10 se... | 3 | 0 | 0.11 | 1 |
| GO:2001182 | regulation of interleukin-12 secretion | 3 | 0 | 0.11 | 1 |
| GO:2001183 | negative regulation of interleukin-12 se... | 2 | 0 | 0.08 | 1 |
| GO:2001185 | regulation of CD8-positive | alpha-beta | 6 | 0 | 0.23 |
| GO:2001186 | negative regulation of CD8-positive | alp... | 1 | 0 | 0.04 |
| GO:2001187 | positive regulation of CD8-positive | alp... | 4 | 0 | 0.15 |
| GO:2001188 | regulation of T cell activation via T ce... | 3 | 0 | 0.11 | 1 |
| GO:2001189 | negative regulation of T cell activation... | 2 | 0 | 0.08 | 1 |
| GO:2001190 | positive regulation of T cell activation... | 1 | 0 | 0.04 | 1 |
| GO:2001191 | regulation of gamma-delta T cell activat... | 1 | 0 | 0.04 | 1 |
| GO:2001193 | positive regulation of gamma-delta T cel... | 1 | 0 | 0.04 | 1 |
| GO:2001198 | regulation of dendritic cell differentia... | 7 | 0 | 0.27 | 1 |

| | | | | | |
|---|---|---|---|---|---|
| GO:2001199 | negative regulation of dendritic cell di... | 5 | 0 | 0.19 | 1 |
| GO:2001200 | positive regulation of dendritic cell di... | 1 | 0 | 0.04 | 1 |
| GO:2001201 | regulation of transforming growth factor... | 1 | 0 | 0.04 | 1 |
| GO:2001202 | negative regulation of transforming grow... | 1 | 0 | 0.04 | 1 |
| GO:2001204 | regulation of osteoclast development | 7 | 0 | 0.27 | 1 |
| GO:2001205 | negative regulation of osteoclast develo... | 2 | 0 | 0.08 | 1 |
| GO:2001206 | positive regulation of osteoclast develo... | 3 | 0 | 0.11 | 1 |
| GO:2001213 | negative regulation of vasculogenesis | 1 | 0 | 0.04 | 1 |
| GO:2001214 | positive regulation of vasculogenesis | 9 | 0 | 0.34 | 1 |
| GO:2001222 | regulation of neuron migration | 21 | 0 | 0.8 | 1 |
| GO:2001223 | negative regulation of neuron migration | 6 | 0 | 0.23 | 1 |
| GO:2001224 | positive regulation of neuron migration | 5 | 0 | 0.19 | 1 |
| GO:2001225 | regulation of chloride transport | 4 | 0 | 0.15 | 1 |
| GO:2001226 | negative regulation of chloride transpor... | 1 | 0 | 0.04 | 1 |
| GO:2001241 | positive regulation of extrinsic apoptot... | 15 | 0 | 0.57 | 1 |
| GO:2001245 | regulation of phosphatidylcholine biosyn... | 2 | 0 | 0.08 | 1 |
| GO:2001246 | negative regulation of phosphatidylcholi... | 1 | 0 | 0.04 | 1 |
| GO:2001247 | positive regulation of phosphatidylcholi... | 1 | 0 | 0.04 | 1 |
| GO:2001248 | regulation of ammonia assimilation cycle | 1 | 0 | 0.04 | 1 |
| GO:2001250 | positive regulation of ammonia assimilat... | 1 | 0 | 0.04 | 1 |
| GO:2001253 | regulation of histone H3-K36 trimethylat... | 1 | 0 | 0.04 | 1 |
| GO:2001256 | regulation of store-operated calcium ent... | 5 | 0 | 0.19 | 1 |
| GO:2001258 | negative regulation of cation channel ac... | 22 | 0 | 0.84 | 1 |
| GO:2001260 | regulation of semaphorin-plexin signalin... | 1 | 0 | 0.04 | 1 |
| GO:2001262 | positive regulation of semaphorin-plexin... | 1 | 0 | 0.04 | 1 |
| GO:2001263 | regulation of C-C chemokine binding | 1 | 0 | 0.04 | 1 |
| GO:2001264 | negative regulation of C-C chemokine bin.. | 1 | 0 | 0.04 | 1 |
| GO:2001268 | negative regulation of cysteine-type end... | 4 | 0 | 0.15 | 1 |
| GO:2001270 | regulation of cysteine-type endopeptidas... | 3 | 0 | 0.11 | 1 |
| GO:2001271 | negative regulation of cysteine-type end... | 3 | 0 | 0.11 | 1 |
| GO:2001274 | negative regulation of glucose import in... | 2 | 0 | 0.08 | 1 |
| GO:2001279 | regulation of unsaturated fatty acid bio... | 6 | 0 | 0.23 | 1 |
| GO:2001280 | positive regulation of unsaturated fatty... | 5 | 0 | 0.19 | 1 |
| GO:2001286 | regulation of caveolin-mediated endocyto.. | 5 | 0 | 0.19 | 1 |
| GO:2001287 | negative regulation of caveolin-mediated... | 2 | 0 | 0.08 | 1 |
| GO:2001288 | positive regulation of caveolin-mediated... | 2 | 0 | 0.08 | 1 |
| GO:2001293 | malonyl-CoA metabolic process | 3 | 0 | 0.11 | 1 |
| GO:2001294 | malonyl-CoA catabolic process | 1 | 0 | 0.04 | 1 |
| GO:2001295 | malonyl-CoA biosynthetic process | 2 | 0 | 0.08 | 1 |
| GO:2001300 | lipoxin metabolic process | 7 | 0 | 0.27 | 1 |
| GO:2001301 | lipoxin biosynthetic process | 2 | 0 | 0.08 | 1 |
| GO:2001302 | lipoxin A4 metabolic process | 2 | 0 | 0.08 | 1 |
| GO:2001303 | lipoxin A4 biosynthetic process | 2 | 0 | 0.08 | 1 |
| GO:2001304 | lipoxin B4 metabolic process | 1 | 0 | 0.04 | 1 |
| GO:2001306 | lipoxin B4 biosynthetic process | 1 | 0 | 0.04 | 1 |
| GO:2001311 | lysobisphosphatidic acid metabolic proce.. | 1 | 0 | 0.04 | 1 |
